# A True Reverse Anomeric Effect Does Exist After All: A Hydrogen Bonding Stereocontrolling Effect in 2-Iminoaldoses

**DOI:** 10.1021/acs.joc.4c00562

**Published:** 2024-05-16

**Authors:** Esther Matamoros, Esther M. S. Pérez, Mark E. Light, Pedro Cintas, R. Fernando Martínez, Juan C. Palacios

**Affiliations:** † Departamento de Química Orgánica e Inorgánica, Facultad de Ciencias, and Instituto del Agua, Cambio Climático y Sostenibilidad (IACYS), Universidad de Extremadura, 06006 Badajoz, Spain; ‡ Department of Chemistry, Faculty of Natural and Environmental Sciences, University of Southampton, Southampton SO17 1BJ, U.K.; § Departamento de Química Orgánica, Universidad de Málaga, Campus Teatinos s/n, 29071 Málaga, Spain; ∥ Instituto de Investigación Biomédica de Málaga y Plataforma en Nanomedicina − IBIMA, Plataforma Bionand, Parque Tecnológico de Andalucía, 29590 Málaga, Spain

## Abstract

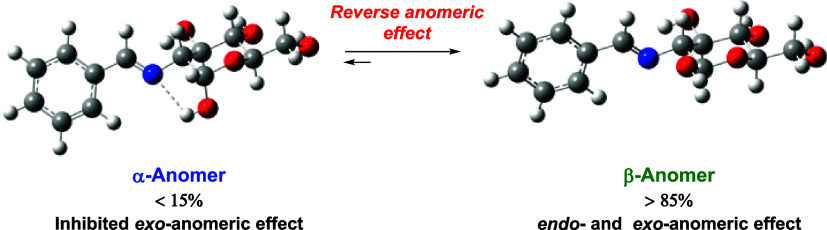

The reverse anomeric effect is usually associated with the equatorial preference of nitrogen substituents at the anomeric center. Once postulated as another anomeric effect with explanations ranging from electrostatic interactions to delocalization effects, it is now firmly considered to be essentially steric in nature. Through an extensive research on aryl imines from 2-amino-2-deoxyaldoses, spanning nearly two decades, we realized that such substances often show an anomalous anomeric behavior that cannot easily be rationalized on the basis of purely steric grounds. The apparent preference, or stabilization, of the β-anomer takes place to an extent that not only neutralizes but also overcomes the normal anomeric effect. Calculations indicate that there is no stereoelectronic effect opposing the anomeric effect, resulting from the repulsion between electron lone pairs on the imine nitrogen and the endocyclic oxygen. Such data and compelling structural evidence unravel why the exoanomeric effect is largely inhibited. We are now confident, as witnessed by 2-iminoaldoses, that elimination of the exo-anomeric effect in the α-anomer is due to the formation of an intramolecular hydrogen bond between the anomeric hydroxyl and the iminic nitrogen, thereby accounting for a true electronic effect. In addition, discrete solvation may help justify the observed preference for the β-anomer.

## Introduction

A good rule of thumb is that the effect of substituents in positions other than the anomeric one in pyranose rings (both spatial arrangement and electronic properties) is not usually taken into account to explain the origin of the anomeric effect.^1^ In context, 2-amino-2-deoxy-aldopyranoses are particularly appealing to test whether or not this assumption is ultimately correct, because functionalization close to the anomeric position can easily be achieved. Schiff bases formed by condensation with aromatic aldehydes or their synthetic equivalents belong to such target compounds.

It is known that some derivatives of **1–3** occur mainly or exclusively as the axial anomers (α-anomers), both in the solid state and in solution, such as hydrochlorides **1–3**(2,3) and their enamines **4–6** (from condensation with acetylacetone or diethyl ethoxymethylenemalonate).^4,5^
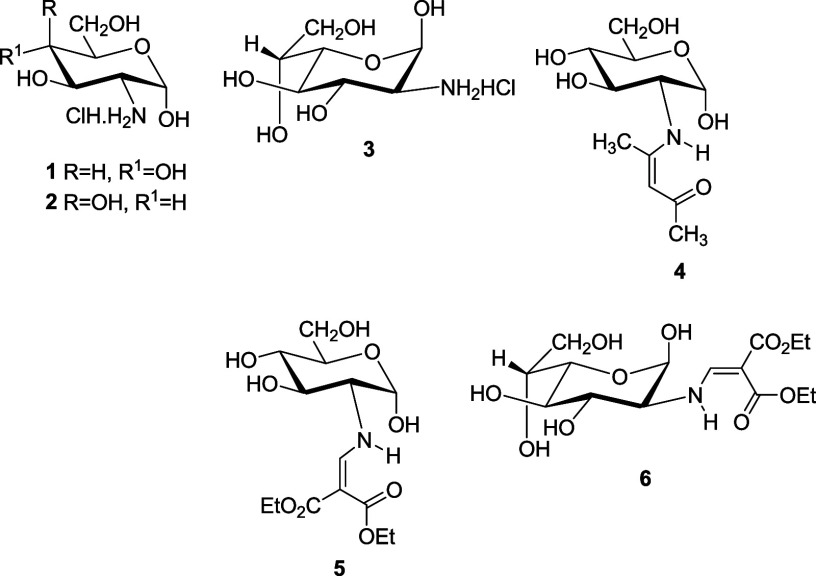


Aqueous solutions of hydrochlorides **1–3** evidence the existence of axial (α) and equatorial (β) forms in which the former predominates to a large extent. This configurational pattern should reasonably be ascribed to the anomeric effect that would favor the axial disposition of the hydroxyl group. As strange as it may be, the literature points to the prevalent β configuration (equatorial anomeric hydroxyl) for the imines derived from **1** and **3** and aromatic aldehydes (**7–10**).^5−10^ However, the opposite α-configuration for imines derived from **1** and their acetyl derivatives was postulated in previous work.^11^ From a synthetic viewpoint, such imines have been advantageously used not only to protect the amino group but also to switch the anomeric configuration of 2-amino-2-deoxyaldoses.
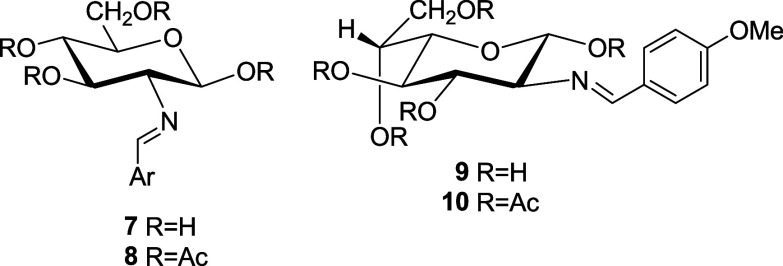


In principle, the factors governing conformational preferences and steric interactions can be inferred from those found in six-membered heterocycles.^12^ Computational data obtained for oxane (or tetrahydropyran), 1,3-dioxane^13^ and its thio analogues^14^ or piperidine or hexahydropyrimidine^15^ give valuable information on the role played by nonbinding (steric) and stereoelectronic effects, exemplified by the anomeric, gauche or Perlin effects.^16^ The hydroxylated derivatives of these heterocycles (namely, 2-hydroxytetrahydropyran, 2-hydroxypiperidine, 2-hydroxy-1,3-dioxane, and 2-hydroxyhexahydropyrimidine) have also been considered to evaluate the nature of both anomeric and exo-anomeric effects.^15a,17^ Even 2-aminotetrahydropyran and its protonated form have been studied to elucidate the existence and/or origin of the reverse anomeric effect,^17^ although little attention has been paid to the influence exerted by groups at nonanomeric positions on the anomeric center.

Several hypotheses could explain the anomalous anomeric behavior: (a) the most insoluble anomer, isolated by crystallization, is coincidental with the anomer having the most stable crystal lattice; (b) the anomer bearing the equatorial OH group is more stable than the axial anomer if the former is stabilized by an intramolecular hydrogen bond between the hydroxyl and the basic nitrogen of the imine group; (c) steric effects, caused by the bulky aryl substituent attached to the iminic carbon, force the vicinal anomeric OH to adopt an equatorial orientation; (d) interactions with surrounding solvent molecules stabilize preferentially the equatorial anomer, or (e) there should be another “hidden” effect that counterbalances efficiently the driving force provided by the anomeric effect.

To our knowledge, the origin remains unexplored. Nor can we justify these experimental observations by invoking solely steric constraints. Clearly, this represents the motto for this long reinvestigation. This manuscript attempts to shed light in a somewhat comprehensive approach, to convincingly show that all structures are actually what they are, thereby ruling out any explanation other than a new stereoelectronic bias in 2-iminoaldoses.^18^ To cope with the problem, a variety of imines derived from **1** and **3** have been synthesized and thoroughly characterized and compared with representative analogues from the previous literature. The influence of the aromatic aldehyde on the anomeric equilibrium has been determined by considering both steric and electronic effects of the substituents. Moreover, the mutarotation of the most significant imines has been disentangled as this phenomenon could stem not only from an anomeric equilibrium but also from tautomeric interconversion, variation in the sugar ring size, conformational equilibria, along with side rearrangements or formation of heterocycles through ring closures, among others. In addition, various *O*-protected derivatives, whose α or β configurations at the anomeric center are predetermined by synthesis, have been obtained as well and subjected to equilibration experiments. Since these imines cannot exhibit the intramolecular hydrogen bonding between the N atom and the anomeric OH, such experiments should clarify whether or not the aforementioned bonding is a real effect. Finally, anomerically unprotected imines, susceptible of the intramolecular bonding, will also enable their equilibration in different solvent systems.

## Results and Discussion

### Synthesis of 2-Amino-2-deoxyaldose Imines

**Scheme 1:**
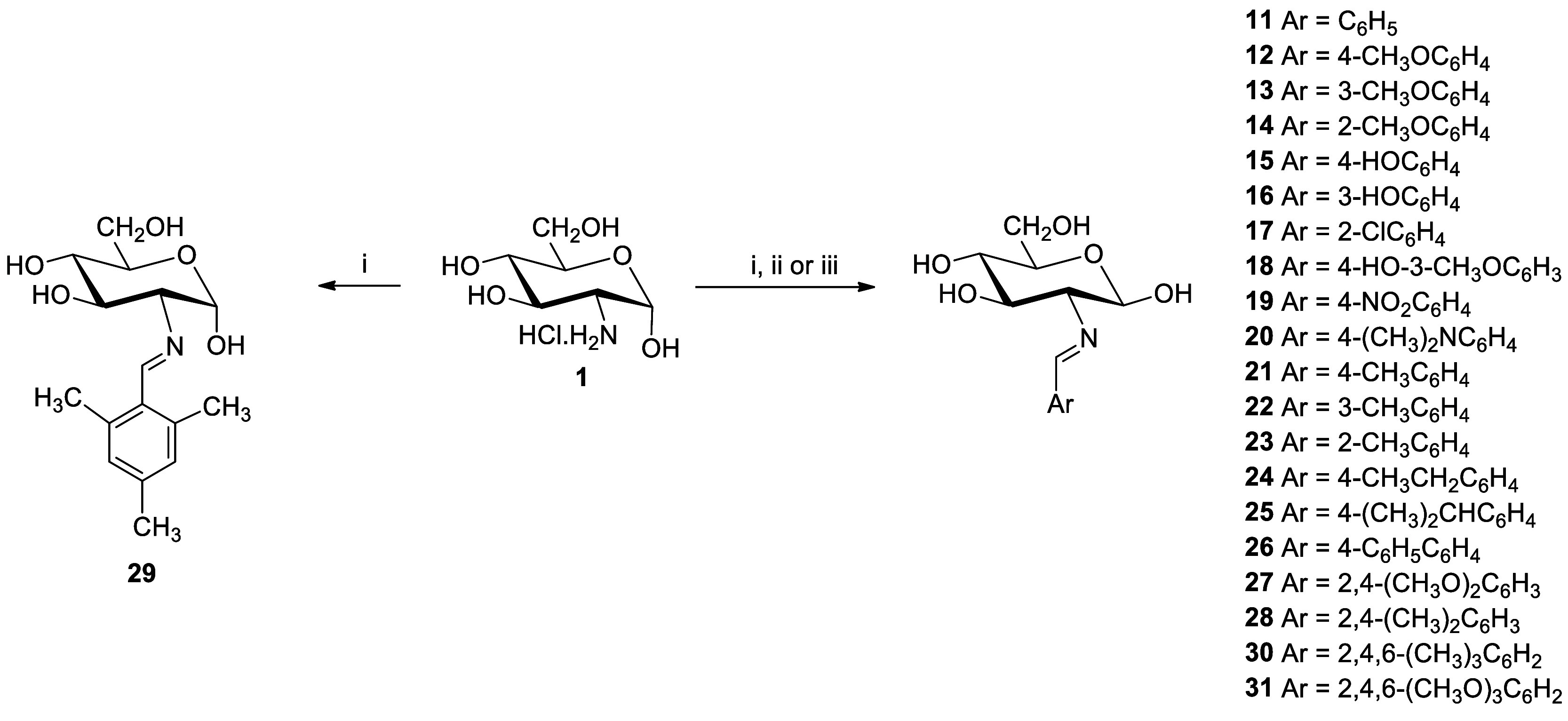
Preparation of Imines Derived from 2-Amino-2-deoxy-d-glucose Reagents: i, ArCHO, 1M NaOH; ii, ArCHO, AcONa, H_2_O/MeOH; iii, ArCHO, NaHCO_3_, H_2_O/MeOH.

The direct reaction of 2-amino-2-deoxy-α-d-glucopyranose hydrochloride with arylaldehydes in basic aqueous solution leaves usually a crystalline solid of 2-(arylmethylidene)amino-2-deoxy-β-**d**-glucopyranoses (**11–28**) from the reaction mixture (Scheme 1). Imine derivatives **11**,^19^**12**,^6^**13**,^9^**15**,^11b^**16**,^9^**18,**^20b^**19**,^20a^**21**, and **22**(9) have been described previously, while compounds **14**, **17**, **20**, and **23–30** are reported herein for the first time. In general, the resulting Schiff bases are usually pure and can be used without further purification.

The reaction of d-glucosamine with 2,4,6-trimethylbenzaldehyde leads to the almost instantaneous formation of an insoluble Schiff base. In most cases, the β-anomer is obtained (**30**), although from time to time and, in an apparently random manner, the α-anomer crystallizes (**29**). The desired anomer can however be selectively obtained by seeding. Yields are usually greater than 60%, even much higher in the cases of **14**, **23**, **27**, **28**, and **29**/**30**, despite the steric effect caused by the ortho-substituents. 2,4,6-Trimethoxybenzaldehyde did not produce the corresponding imine **31**; a fact not only attributed to steric effects but also to the lower electrophilicity of the carbonyl carbon caused by the strong electron donating effect of three methoxy groups.

The Schiff bases derived from *o*-salicylaldehyde and 2,4-dihydroxybenzaldehyde were obtained according to previous work. The first derivative crystallizes as a mixture of axial (α) and equatorial (β) anomers (**32** and **33**, ∼1:2 ratio)^21^ and the second as the axial anomer only (**34**),^22^ both with imine structure. In solution, however, the most abundant anomer shows the equatorial configuration (**33** and **35**, respectively). The hydrate of **34** and its hydrochloride were obtained as a mixture of both anomers (**36** and **37**).^22^
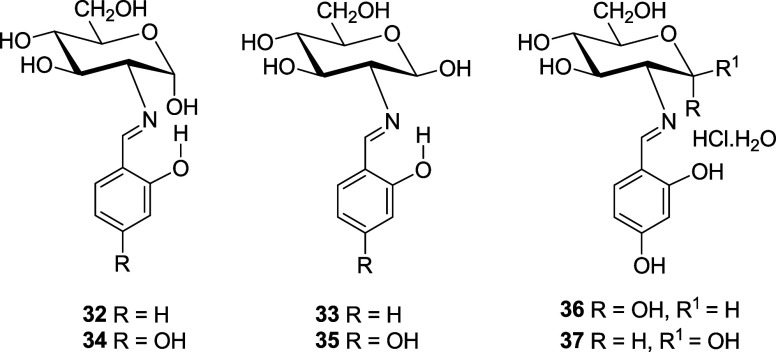


The condensation of **1** with 2,4,6-trihydroxybenzaldehyde failed to give **38**. Instead, a dark red solid was isolated, which consisted of a mixture of anomers **39**/**40** and **41**/**42,** inseparable at room temperature due to a low interconversion barrier (Δ*G*^‡^ ∼18.5 kcal/mol).^23^
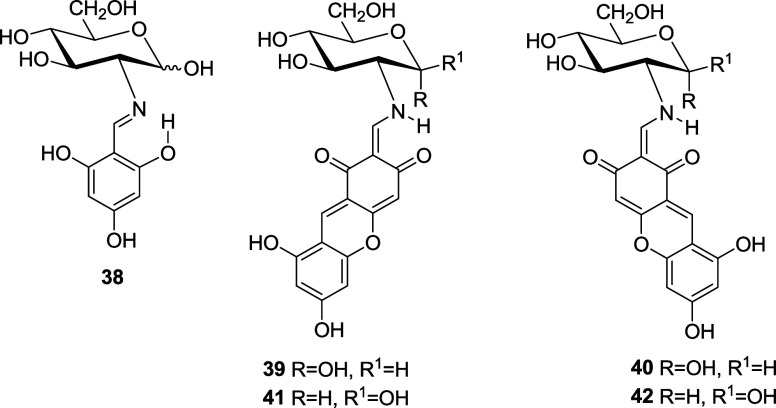


Analytical and spectroscopic data confirm the structures assigned to **11**–**42** (Tables S1–S6). The glucopyranose structure of **11**–**30** can further be confirmed by transforming some unprotected compounds into the corresponding per-*O*-acetyl derivatives (such as **43** and **44**), which were obtained in good yields, by treatment with acetic anhydride in pyridine at ambient temperature.^6,9^ The formation of α-anomers (like **46** and **47**), however, requires an indirect approach starting from 1,3,4,6-tetra-*O*-acetyl-2-amino-2-deoxy-α-d-glucopyranose hydrobromide (**45**).^4b,24^
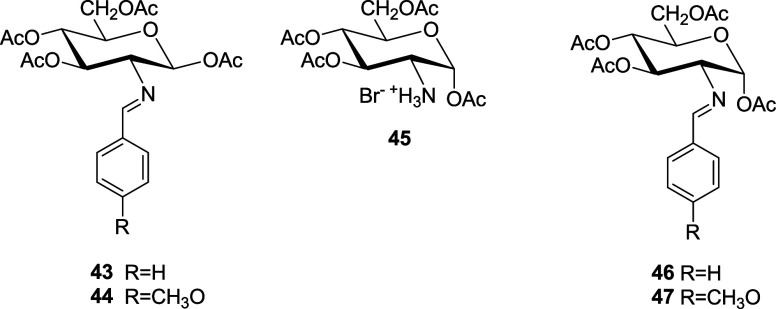


In order to disentangle the behavior of the anomeric carbon from the rest of the molecule, the synthesis of acylated imines leaving unprotected the anomeric hydroxyl was undertaken too. This type of compounds would allow studying the effects caused by substituents at C-2 and solvent’s molecules on the anomeric hydroxyl, since it is known that the extent of the anomeric effect depends on the dielectric constant of the medium.^25^ Likewise, other potential equilibria such as tautomerizations are greatly dependent on solvent effects.

**Scheme 2:**
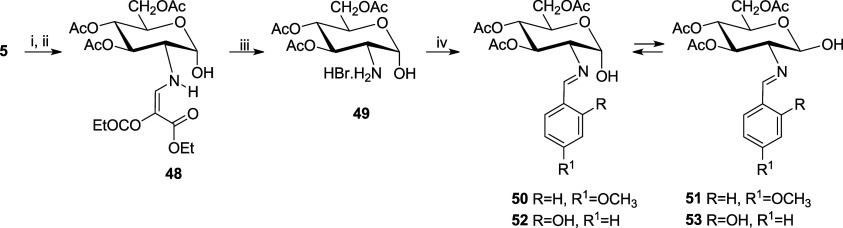
Formation of Selectively Unprotected Imine Anomers **50–53** Reagents: i, CH_3_COCl; ii, H_2_O; iii, Br_2_, H_2_O; iv, 2-R-4-R^1^C_6_H_3_CHO, pyridine.

All attempts to deacetylate the α-anomer **46** were unsuccessful, and only recovery of the starging material or decomposition products could be observed. We then turned to an alternative strategy using 3,4,6-tri-*O*-acetyl-2-amino-2-deoxy-α-d-glucopyranose hydrobromide (**49**) as a raw material, which can be obtained from d-glucosamine, through enamine **48**, according to Scheme 2.^26^ Condensation of **49** with *p*-anisaldehyde and *o*-salicylaldehyde with removal of HBr affords the corresponding imines. In the first case, the mixture of anomers **50** and **51** was impurified by anisaldehyde due to slow decomposition. However, a mixture of pure α- and β-anomers **52** and **53**, respectively, could be obtained.

### Mutarotation of Schiff Bases of 2-Amino-2-deoxyaldoses

**Scheme 3:**
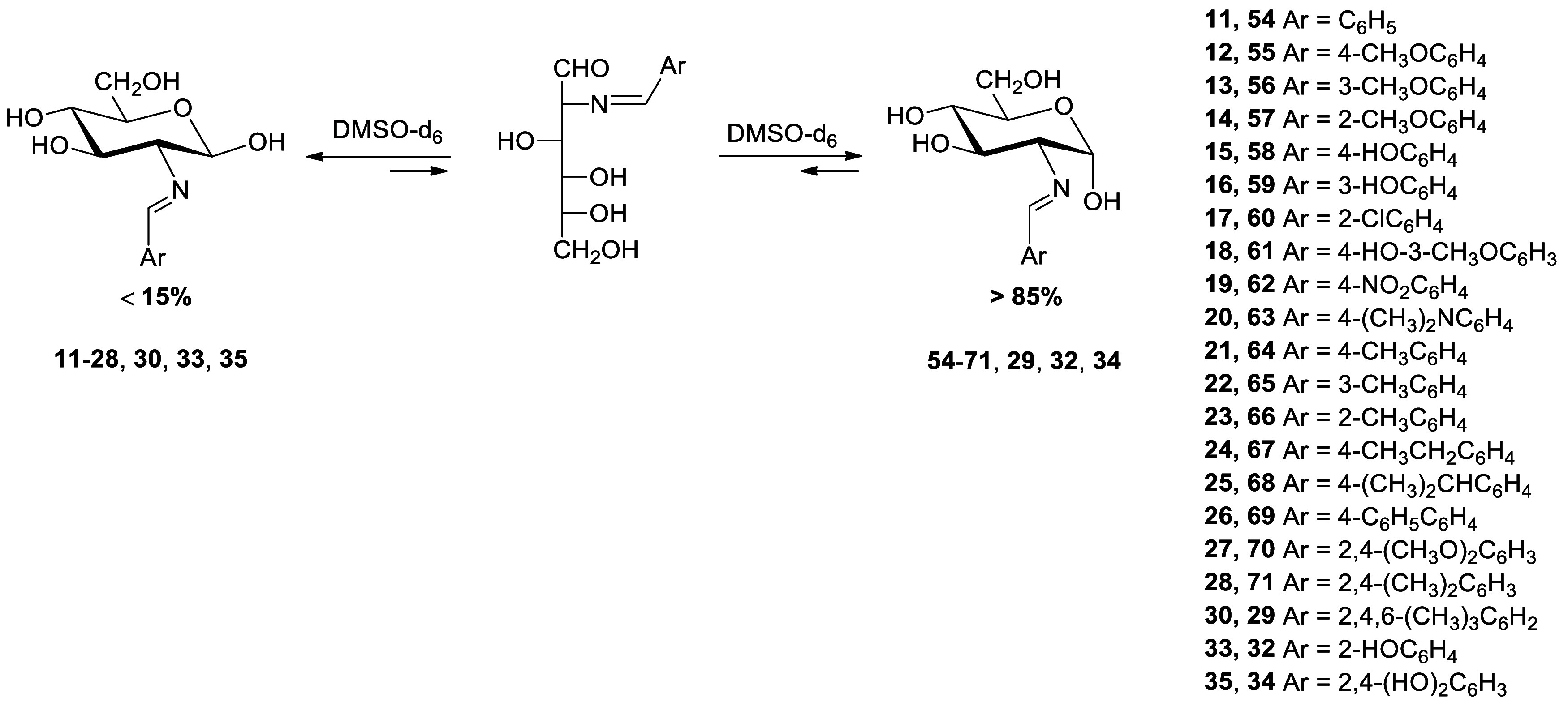
Equilibration of Aryl Imines Between α- and β-Anomers in DMSO Solutions

Like other reducing sugars, imines derived from unprotected 2-amino-2-deoxyaldoses exhibit mutarotation, that is, the optical rotation in solution varies, to a greater or lesser extent over time, until reaching a steady-state value. In order to quantify the anomeric effect, it is necessary to carry out equilibration experiments between the anomers of a given compound. The steady-state concentration of both anomers allows us to calculate the free energy change, and hence the energy imbalance associated with the anomeric effect. All imines of d-glucosamine derived from substituted benzaldehydes show β-anomeric configuration (placing the anomeric hydroxyl in equatorial disposition), with the exception of the imine from 2,4,6-trimethylbenzaldehyde (**29**). A temporal monitoring of all imines with β-anomeric configuration was performed in DMSO-*d*_6_ to determine the extent of the anomeric equilibria. As shown in Scheme 3, the resulting equilibrium solutions are largely populated by the β-anomer.

The presence of signals at ∼8–8.5 ppm and ∼162 ppm, attributable to the imine group, and the absence of typical oxazolidine signals at ∼5–6 ppm and ∼90–97 ppm^27^ rule out the possibility of a equilibrium involving such five-membered heterocycles, which result from addition of the anomeric hydroxyl to the imine bond. In any case, the amount of the axial anomer (**29** and **54**-**71**) does not exceed 15%.

**Figure 1:**
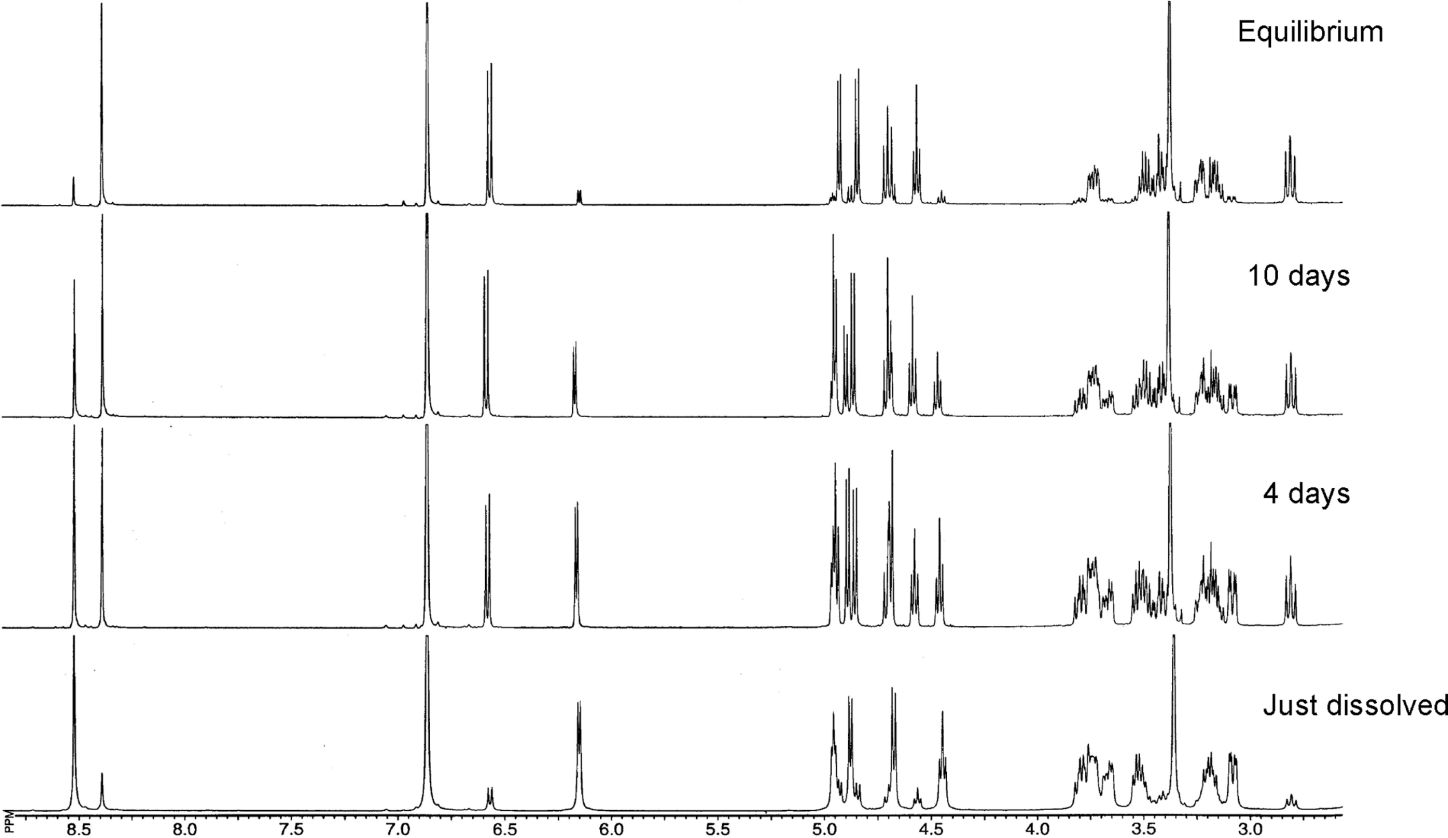
Equilibration of anomers **29** and **30** in DMSO-*d*_6_.

The α-anomer **29** is in agreement with the small value of the coupling constant *J*_1,2_ (2.9 Hz). A solution of **29** in DMSO-*d*_6_, kept at room temperature, which initially shows α-anomeric configuration, equilibrates only with another compound that becomes the predominant structure, in a proportion of ∼87%. After equilibration the new product presents a high value of *J*_1,2_ (7.3 Hz), consistent with formation of the β-anomer **30** (Figure 1).

This conclusion is confirmed not only by the value of δ_C1_ (95.90 ppm) but also by the value of ^1^*J*_C1–H1_ (158.4 Hz), measured in the coupled ^13^C{^1^H} NMR spectrum of a mixture of both anomers before equilibration, that is, a fresh solution of **29**. In contrast, the β-anomer **30** shows a higher coupling constant (^1^*J*_C1–H1_ 164.9 Hz).^28,29^

Thus, ^1^H and ^13^C NMR data evidence that only α- and β-anomers are actually involved in mutarotation equilibria and, even if we assume equilibration through an acyclic form, the latter does not reach enough concentration to be observable (Scheme 3). Other NMR data (chemical shifts and coupling constants) support likewise the α-anomeric structure assigned to **29** and the opposite β-configuration of **30** (Tables S7–S9).

For the rest of imines, the coupling constants between the proton and the anomeric carbon measured in the ^13^C{^1^H} NMR spectra confirm the assigned anomeric configuration as well. In the case of the 4-nitrobenzylidene derivative **19**, for instance, the minor product has ^1^*J*_C1,H1_^α^ = 165.0 Hz and the major product ^1^*J*_C1,H1_^β^ = 157.5 Hz. In addition, the NMR spectra of α-configured and intramolecularly H-bonded salicilydeneimine **34** show that the β-anomer is the dominant species in the equilibrium mixture. In stark contrast, enamines **39** and **40** show the prevalence of the α-anomer (α-anomers 41% + 27% = 68%). Finally, like **11**–**31**, imine **9** equilibrates in solution, where the β-anomer largely predominates.

### Conformational Analysis of Imines and Per-*O*-acetyl Imines

**Figure 2:**
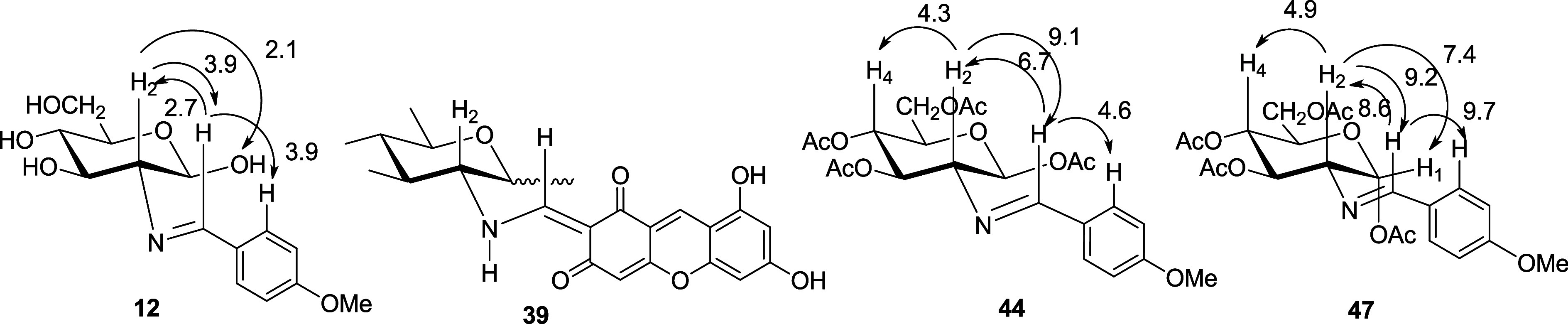
NOE enhancements and conformational arrangements of **12**, **39**, **44**, and **47**.

The large coupling constants *J*_2,3_, *J*_3,4_, and *J*_4,5_ (9.0–9.8 Hz) are fully consistent with pyranose structures of d-gluco configuration in ^4^*C*_1_ conformation for **11**-**37**, **39**, **40**, **43**, **44**, **46**, **47**, and **54**–**71** and l-gluco in conformation ^1^*C*_4_ for **9**.^30^ However, we were interested in the relative arrangement of the iminic group with respect to the sugar ring, which could a priori be determined by NOE experiments.^31^ Taking **12**, as a representative example, the appreciable NOE effects observed between H-2 and the iminic hydrogen and the latter with a hydrogen atom at the aromatic ring do indicate the close proximity of such protons adopting an approximately 1,3-diaxial arrangement. This diaxial disposition is only compatible with an E-configuration around the C=N bond. The existence of NOE effect between H-2 and the OH proton at the anomeric position, together with the absence of NOE effect between H-1 and H-2, confirms the β-configuration for these compounds. As a result, the plane containing the arylimine group should be coplanar with the aromatic ring and approximately perpendicular to the average plane of the pyranose ring (Figure 2). Likewise, the coupling constants *J*_2,NH_ (9.2 Hz) and *J*_=CH,NH_ (14.0 Hz) measured in enamine **39** (and **40**) suggest an antiperiplanar arrangement between these protons and point to a preferential conformation in solution shown in Figure 2 as well.

To ensure the validity of the above conclusions, a conformational analysis of the imine group in **11** and **54** has been carried out. To this end, we have calculated the energy of the arrangements generated around the N–C2 bond by rotating the dihedral angle θ_H2–C2–N-CH_ from 0 to 360°, with a step size of 15° each. The computational DFT study was performed using the 6-311G(d,p) basis set,^32^ with all geometries optimized in the gas phase using the B3LYP^33^ and the M06-2X^34^ hybrid density functionals without any geometrical restriction. Solvent effects were simulated using the SMD method.^35^ Probably, the most important corollary of this conformational analysis, inferred from theory and experiment, is that for all imines derived from 2-amino-2-deoxyaldopyranoses, regardless of solution or solid state structures, the lone pair of the iminic nitrogen lies in an approximately antiperiplanar disposition with respect to the C2–H2 bond, which in turn minimizes the steric effects (see the Supporting Information). We have verified that this disposition is not only adopted by unprotected imines but also by their per-*O*-acetyl derivatives. Thus, NOE experiments were conducted on the anomeric pair of **44** and **47**, derived from **1**. The resulting NOE enhancements indicate that the preferred conformation in solution is similar to that shown by **12** (Figure 2).

**Figure 3:**
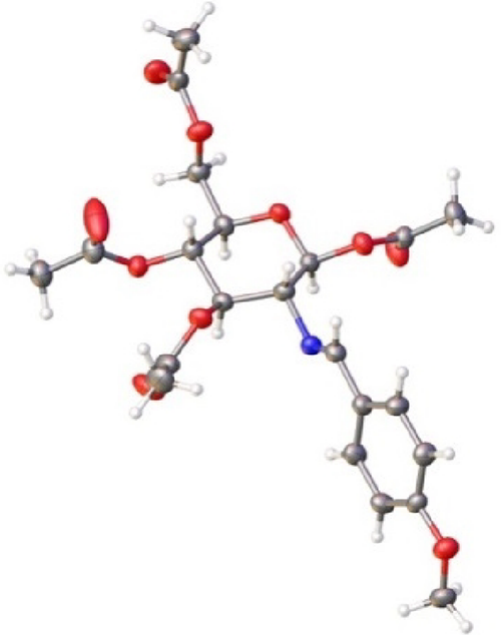
ORTEP diagram of imine **44** obtained by X-ray diffraction. Ellipsoids drawn at 50% probability.

The above conclusions were fully confirmed by solving the structure of compound **44** through single-crystal X-ray diffraction (Figure 3, see Experimental Section and Table S16). The conformation displayed by **44** in the solid state is practically the same as the one adopted in solution, as deduced from its spectroscopic data and NOE measurements.

Both experimental and calculated bond distances and dihedral angles related to the imine bond and its conformational disposition have also been included with the Supporting Information (see Table S17). Furthermore, crystallographic parameters and those calculated in the gas phase or in chloroform are practically identical.^35^ The low value of the dihedral angles θ_H2–C2–N-CH_ (<12.5°), θ_C2–N–C-H_ (<2.5°), and θ_H2–C2–CN-H_ (<11°) demonstrates that the conformational arrangement adopted by the imino group is completely general.

**Figure 4:**
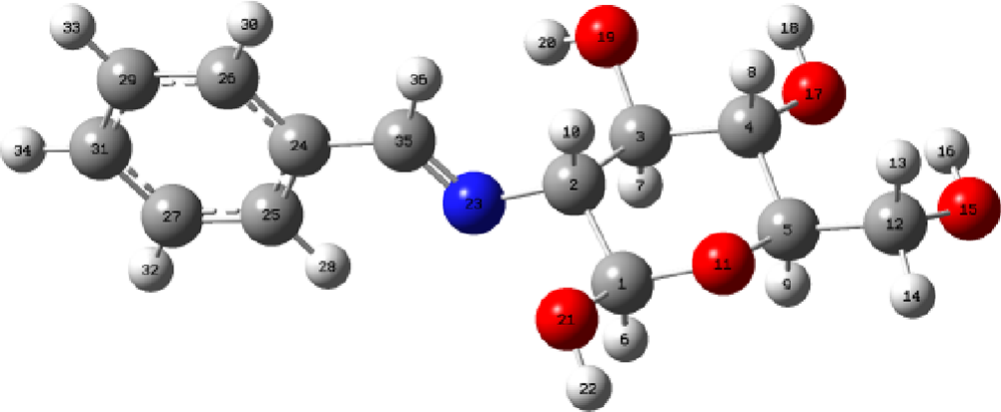
Numbering used in the NBO analysis of anomers **11** and **54**.

A natural bond orbital (NBO)^36^ analysis has been carried out for both β- and α-anomers **11** and **54**, respectively (numbering is shown in Figure 4). The most important stabilizing interactions affecting the heteroatoms attached to the anomeric carbon and C-2 are listed in Tables S18 and S19. The lone pairs on the endocyclic oxygen show delocalization effects with the antiparallel neighboring bonds C–C and C–H, with values of n_O11_ → σ*_C–C_ and σ*_C–H_ of ∼6–7 kcal/mol. In the α-anomer, the interaction responsible for the anomeric effect, n_O11_ → σ*_C1–O21_, amounts to ∼13.5 kcal/mol. Likewise, the electron pairs on the anomeric hydroxyl oxygen show similar effects, highlighting an exoanomeric effect in the β-anomer, n_O23_ → σ*_C1–O11_, of ∼15–17 kcal/mol. This effect is absent in the α-anomer due to hydrogen bonding between the anomeric hydroxyl and the imine nitrogen (see later). It is also interesting the effect caused by the lone pair of the nitrogen atom at C-2 on the protons of the two vicinal carbons. The interaction with the iminic CH, n → σ*_=CH_, takes values of ∼12–13 kcal/mol and with the proton at C-2, n → σ*_C2–H_, of ∼ 6–7 kcal/mol. Overall, these stereoelectronic effects contribute to the spatial environment of the imino group, which lies in a perpendicular disposition to the pyranose plane, in line with all data supported by NOE measurements (Figure 2).

### Theoretical Study of Imine Stability

To rationalize the experimental observations, a computational study was carried out to determine the relative stabilities of the different species involved. Initially, the basis sets 6-31G(d,p) and 6-311G(d,p) were chosen,^32^ with geometries optimized in the gas phase and using the functional hybrids B3LYP^33^ and M06-2X^34^ without any geometrical restriction. Further benchmarking with the M06-2X functional coupled with def2-TZVP valence-triple-ζ basis set^37^ has also been conducted for geometry optimizations and frequency calculations, since M06-2X/def2-TZVP has been described to provide an optimal balance between performance and precision for addressing structure and binding issues in carbohydrate derivatives.^38,39^

**Chart 1:**
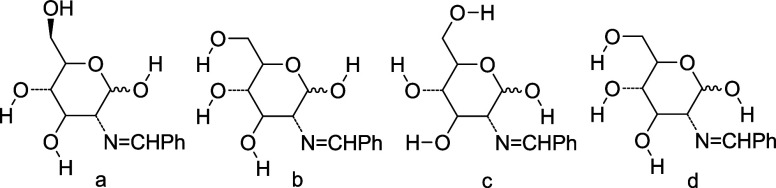
Different Orientations of OH Groups in Imines of 2-Deoxyaldoses

**Figure 5:**
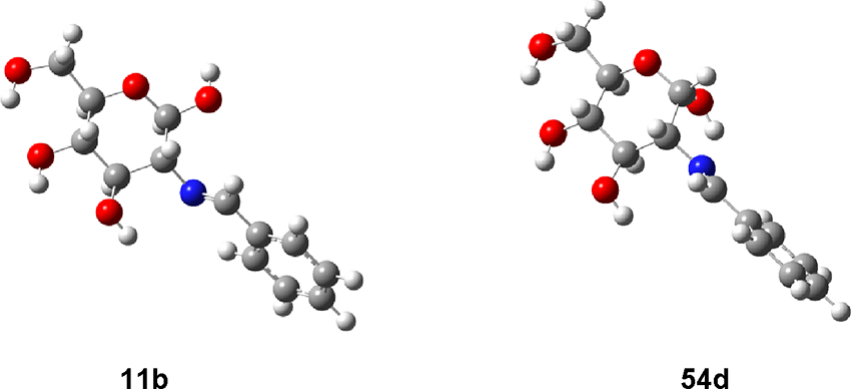
Stable arrangements of hydroxyl groups in structures **14** and **82** (B3LYP/6-31G(d,p)).

To shorten the computational cost, the simplest imine pair derived from benzaldehyde (**14**/**82**) was selected as model and only d-gluco-configured pyranose structures in ^4^*C*_1_ conformation were considered. It is pertinent to mention that the number of possible conformations is exceedingly high, that is, the three staggered conformations of three hydroxyls and the iminic substituent of the pyranose ring, together with the nine (3 × 3) conformations adopted by the hydroxymethyl group at C-5, which amount to 3^6^ = 729 conformations for each anomer. Further simplifications are possible on the hydroxyl groups, taking into account that the most stable arrangements will be those leading to intramolecular hydrogen bonding. Thus, we have considered the dispositions **a–d** for **11** and **54** (Chart 1). The most stable arrangements correspond to **11b** and **54d**, which differ by only the orientation of the anomeric hydroxyl (Table 1 and Figure 5), exhibiting a counterclockwise arrangement of the intramolecular hydrogen bond network.

**Table 1:** Relative Energies (kcal/mol) of Imines **11** and **54**^a^

imine	relative energies	**a**	**b**	**c**	**d**
**11**	Δ*E*	30.77	1.01	5.05	3.67
	Δ*G*	29.16	0.23	3.76	2.86
**54**	Δ*E*	30.72	1.19	1.18	0.00
	Δ*G*	29.55	0.77	0.91	0.00

a B3LYP/6-31G(d,p).

In the case of the α-anomer, this hydroxyl is oriented toward the electron pair of the nitrogen affording an intramolecular hydrogen bond (**54d**, Figure 5). In contrast, the β-anomer cannot form that bonding and the OH group is arranged along the direction of the endocyclic oxygen (**11b**). Accordingly, the rest of calculations began with **11b** and **54d**, thereby reducing to a significant extent the number of structures to be calculated. However, it should not be overlooked that such dispositions can be altered in protic or polar aprotic solvents (such as DMSO), capable of forming hydrogen bonds with the hydroxyl groups.

To ascertain the influence exerted by the substituents of the aromatic ring on the tautomeric equilibrium, the energies of the imine anomers derived from 4-methoxybenzaldehyde (**12**/**55**), 4-nitrobenzaldehyde (**19**/**62**), and 2,4,6-trimethylbenzaldehyde (**29**/**30**) have been calculated as well. The first two disclose the effect caused by electron-donating and electron-withdrawing groups, respectively, while the third anomeric pair reflects putative steric effects. The results are collected and compared with those of **11**/**54** in Table 2 and Figure S2. Again, the axial anomers (α) are slightly more stable than the equatorial ones (β) at both B3LYP/6-31G(d,p) and M06-2*X*/6-311G(d,p) levels, while the β-anomer becomes the most stable structure using the M06-2X/def2-TZVP method. At the latter, the energy difference (Δ*G*) found in DMSO corresponds to an equilibrium percentage for the β-anomer from 63 to 78%, calculated by eq 1 at 298 K, which agrees with the experimental data (see later).
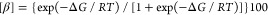
1

**Table 2:** Relative Energies (kcal/mol) for Aryl-Substituted Imines

	**gas phase**^a^		**DMSO**^a^		**gas phase**^b^		**DMSO**^b^		**gas phase**^c^		**DMSO**^c^	
**comp.**	**Δ***E*	**Δ***G*	**Δ***E*	**Δ***G*	**Δ***E*	**Δ***G*	**Δ***E*	**Δ***G*	**Δ***E*	**Δ***G*	**Δ***E*	**Δ***G*
**11**	1.01	0.22	0.87	1.20	1.22	0.21	0.99	0.36	0.58	–0.30	0.16	–0.32
**54**	0.00	0.00	0.00	0.00	0.00	0.00	0.00	0.00	0.00	0.00	0.00	0.00
**12**	1.24	0.52	1.01	1.44	1.40	0.52	1.09	0.49	0.77	0.19	0.26	–0.66
**55**	0.00	0.00	0.00	0.00	0.00	0.00	0.00	0.00	0.00	0.00	0.00	0.00
**19**	0.14	–0.50	0.62	0.07	0.37	0.12	0.81	0.39	–0.19	–1.01	0.03	–0.76
**62**	0.00	0.00	0.00	0.00	0.00	0.00	0.00	0.00	0.00	0.00	0.00	0.00
**30**	0.91	–0.10	0.86	0.34	1.71	0.68	1.24	0.80	0.84	–0.15	0.75	0.07
**29**	0.00	0.00	0.00	0.00	0.00	0.00	0.00	0.00	0.00	0.00	0.00	0.00

a B3LYP/6-31G(d,p).

b M06-2*X*/6-311G(d,p).

c M06-2X/def2-TZVP.

### Anomeric Stabilization: Origin of a Reverse Anomeric Effect in 2-Iminoaldoses

As discussed above, the origin of mutarotation in imines derived from 2-amino-2-deoxyaldoses can be traced on the one hand to anomerization equilibria and on the other to the existence of imine-enamine structures via tautomerization. Among the stereoconformational aspects controlling the molecular arrangements observed in carbohydrates, the anomeric, exoanomeric,^40^ and gauche effects^41^ are the most prominent.^42^ Not by chance, the interpretation of the anomeric effects and other associated stereoelectronic effects in six-membered saturated heterocycles has been extensively documented.^40,43−46^

Even if the first observations of “anomalous effects” date back to early studies on mutarotation,^46^ the preferential stabilization of pyranose rings when they contain an axial electronegative substituent at C1 is contrary to expectations based on considerations of steric or solvation factors.^40,47^ Three models, which are not mutually exclusive, are usually invoked to rationalize the anomeric effect: (a) dipole interaction: the model of interaction between dipoles^48^ suggests that the α-anomeric preference rather results from destabilization of the β-anomer, due to repulsion between the dipole associated with the anomeric hydroxyl and that of the endocyclic oxygen, (b) hyperconjugation or antiperiplanar lone pair hypothesis model (ALPH): the model of the molecular orbital based on the n → σ* interaction invokes the stabilizing interaction of the axial lone pair of endocyclic oxygen (n_O_, HOMO) with the empty orbital σ* of the C–OH bond of the α-anomer (LUMO),^49^ and (c) electron pair repulsion model (n → n interaction), based on the valence-shell electron-pair repulsion model (VSEPR). Its origin can be ascribed to the strong destabilization generated by the interaction between orbitals filled of two pairs of lone and spatially very close electrons.

The first two models have been discussed comprehensively in previous reviews.^43a,40d^ These two approaches cannot easily be reconciled, in part because MO theory does not support the notion of lone pairs in the manner envisioned by VSEPR theory.^50^ There is strong evidence that hyperconjugative interactions are not responsible for the anomeric effect, which is better interpreted in terms of electrostatic interactions.^51^ On the other hand, simple electronic repulsion explains much of the anomeric effect, albeit VSEPR alone cannot justify satisfactorily all the situations.^52^ In any case, the underlying physical origin(s) of this complex phenomenon remain(s) unclear.^53,54^

Although the anomeric effect is generally attributed to hyperconjugative interactions of σ-acceptors with a lone pair at oxygen (negative hyperconjugation), the recent literature reports suggested alternative explanations, which have been collected in two recent reviews.^45^ The complexity associated with the behavior of substituents at the anomeric or pseudoanomeric positions of numerous systems could be better understood by separating steric, electrostatic, and orbitalic factors (all related to the classic anomeric effect in sugars), from “anomeric interactions” dominated by hyperconjugative factors, that is, donation from oxygen lone pairs to weak acceptors, which may influence latent reactivity around the C–O bond.^45a^

**Figure 6:**
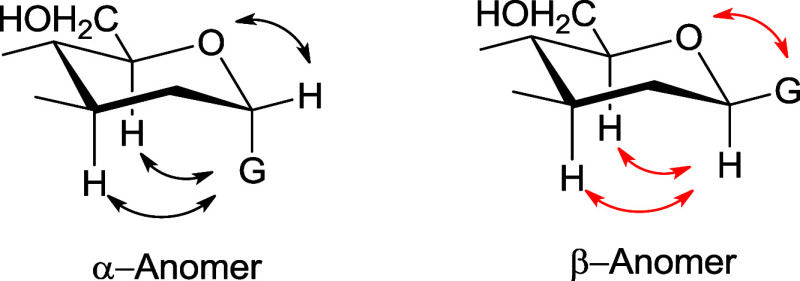
Schematic representations of favorable (black) and unfavorable (red) Coulombic interactions in axial and equatorial anomers according to ref (59).

When carbohydrates are studied in biological and chemical environments, the anomeric effect can be interpreted by a combination of steric, resonance, hyperconjugation, inductive, hydrogen bonding, electrostatic interaction, and solvation effects, leaving aside that the extent of such effects depend on the model and level of computation chosen.^55^ It is believed that both steric and electronic interactions make contributions to the conformational preferences, as any decomposition of these interactions is more or less arbitrary.^56^ Other authors concluded that the steric effect, or more specifically the electrostatic interaction, dominates the anomeric effect^57^ and found further computational evidence to disprove the hyperconjugation explanation.^51,58^ The overall corollary is that no single factor accounts for the axial preference of a substituent, while different and correlated interactions are involved.^59^ Furthermore, the hyperconjugation model involving the transfer of electrons from the ring heteroatom to an excited state of an axial bond is a minor contributor to the anomeric effect. This effect arises mainly from two separate CH···G nonbonded Coulombic attractions between a polar axial substituent (G) and the *syn*-axial hydrogen(s) in the heterocycle (Figure 6).

Conversely, the inverse (or reverse) anomeric effect was initially introduced by Lemieux and Morgan as the tendency of a positively charged aglycone to adopt an equatorial orientation in a sugar ring.^60^ However, protonation is not a prerequisite and the reverse anomeric effect has also been proposed for amino and alkylamino substituents, both neutral and charged.^61^ From a conceptual standpoint, the definition of a reverse anomeric effect simply describes a conformational preference opposite to the anomeric effect; or in other words, it denotes a contribution of additional “anomeric destabilization” to simple steric effects. In fact, the existence of the inverse/reverse anomeric effect as a distinctive stereoelectronic effect has been questioned and debated.^62,63^ The greater equatorial preference of some substituents has been attributed to an accentuation of steric effects.^64^ Most studies focused on protonated alkylamino or imidazole substituents evidence that the equatorial preference originates from favorable steric and electrostatic interactions.^63−65^

Although most of the work on the anomeric effect has been devoted to assess the influence of substituents at the anomeric center, the unequal effect exerted by the hydroxyls, and their stereochemistry, at other carbons of the pyranose ring, is well-known. Thus, the change of the equatorial hydroxyl at C-2 from d-glucose to axial hydroxyl in d-mannose causes a significant increase in the proportion of the α-anomer, which becomes predominant at equilibrium (65.5% α), which is called the Δ2 effect.^66^ As previously highlighted, the enamines formed in the condensation of β-dicarbonyl compounds with **1–3** always adopt the α-anomeric configuration placing the hydroxyl group axially, whereas the Schiff bases resulting from the condensation of such amino sugars with simple aromatic aldehydes reverse the anomeric configuration.^3a,5,6,8,9^ This switching of anomeric equilibrium has not yet received any justification and prompted us to investigate in detail the anomeric and tautomeric equilibria of these Schiff bases. To discard alternative premises, it should be emphasized again that we have verified in all cases the conformation adopted by the imine group with respect to the pyranose ring: the lone pair on the nitrogen atom is arranged approximately parallel to the axial bonds of the ring.

**Figure 7:**
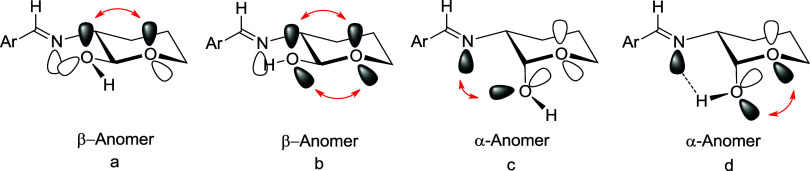
Possible stereoelectronic interactions and hydrogen bonding in the equatorial and axial anomers of iminoaldoses.

The first question is whether in this particular arrangement the lone pair of the imino group triggers a stereoelectronic effect favoring the equatorial anomer (β) or disfavoring the axial one (α) (Figure 7). Apparently, this spatial arrangement appears to have little or no influence on the different conformations adopted by the equatorial anomer. On the other hand, the conformation shown by the axial anomer can be accounted for by the VSEPR theory in aldoses, that is, a repulsive interaction between the electron pair on the nitrogen and one of the lone pairs of the anomeric hydroxyl oxygen (Figure 7c). In the absence of other factors the exo-anomeric interaction is the most dominant, even in the α-anomer.

The destabilization that these interactions cause in the axial anomer translate into a significant increase in the population of the equatorial anomer, which can now be seen as something that opposes the anomeric effect, let us say a “reverse” effect. In fact, if this hypothesis were correct, any atom directly linked to C-2 having lone pairs could exhibit similar electronic interactions. For example, the oxygen of a hydroxyl group in aldoses or the nitrogen in 2-aminoaldoses would exert a similar effect to that of the imino group, to different extents nevertheless.

Our second working hypothesis is whether a hydrogen bond can be established between the imine nitrogen at C-2 and the anomeric hydroxyl. This could alter the orientation of the lone pairs of the latter by decreasing or eliminating the exo-anomeric effect on the axial (α) anomer (Figure 7d). This bond would not occur in the equatorial (β) anomer owing to the conformational rigidity of the arylimino group (Figure 7b).

**Figure 8:**
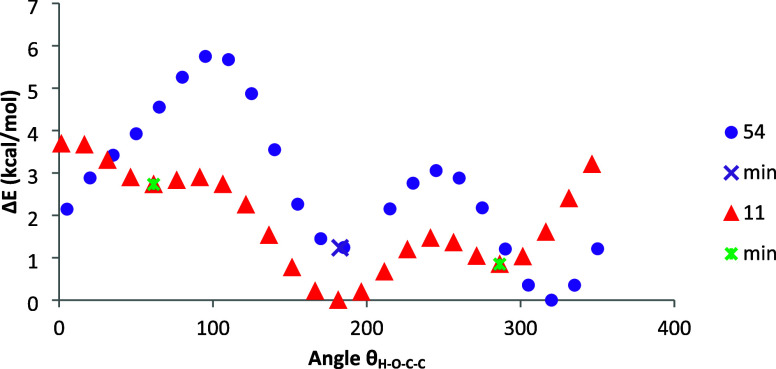
Conformational profiles of the anomeric hydroxyls of **11** (solid triangle) and **54** (circle solid circle) in the gas phase [M06-2*X*/6-311G(d,p)].

**Figure 9:**
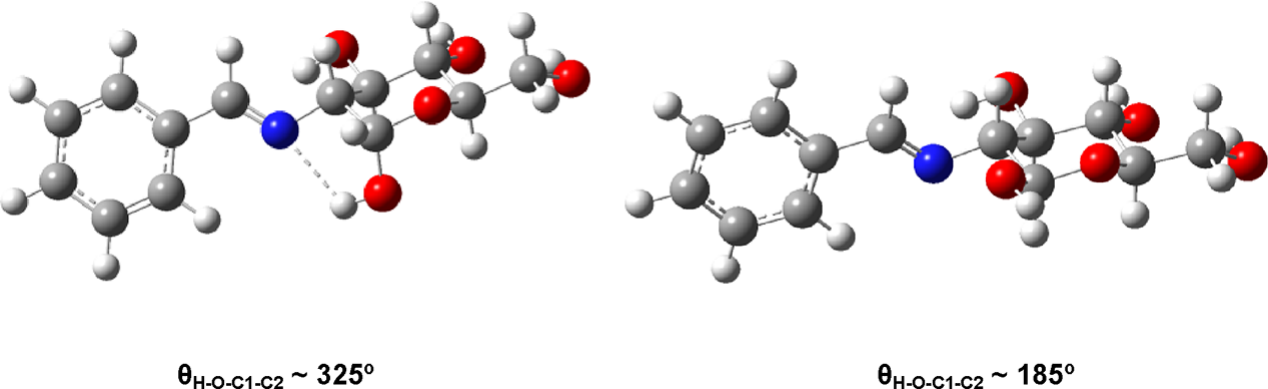
Stable conformational minima for the anomeric hydroxyls of **11** (right) and **54** (left).

In order to compare these arguments, conformational analyses of the anomeric hydroxyls of **11** and **54** were undertaken with rotational profiles shown in Figure 8. Calculations unravel the existence of three minima for β-anomer **11**, whose relative energies are shown in Table 1 (see also Figure S14). The more stable disposition appears at ∼185° in which the interactions with other groups vanish and the exo-anomeric effect is present (Figure 9). However, the two minima at angles of ∼185 and ∼300° show identical energy in DMSO.

The rotational profile for the axial anomer (**54**) shows two minima (Table 3, Figure S15). The most stable minimum occurs at an angle of θ_H–O–C1-C2_ ∼325° and corresponds to hydrogen bonding formation with the nitrogen atom, thereby fixing its conformation and inhibiting the exo-anomeric effect (Figure 9). Moreover, the NBO analysis shows the absence of this effect in the axial anomer **54**. For the equatorial anomer (**11**), however, hydrogen bonding formation is not feasible due to the “rigid” orientation of the lone pair on nitrogen. It is worth pointing out that almost coincidental results were obtained with the def2-TZVP basis set.

**Table 3:** Conformational Energies for the Anomeric Hydroxyls of **11** and **54**^a^

		**gas phase**			**DMSO**		
**derivative**	**minima**	**Δ*E***	**Δ*G***	**θ**_**H–O–C1−C2**_	**Δ*E***	**Δ*G***	**θ**_**H–O–C1-C2**_
**11**^b^	1	0.0	0.0	182.0	0.0	0.0	186.8
	2	0.9	0.8	286.4	0.0	0.0	305.4
	3	2.7	2.8	61.4	1.8	1.8	57.5
**54**^b^	1	0.0	0.0	324.5	0.0	0.0	325.1
	2	1.2	0.7	182.7	1.0	1.3	186.2
**11**^c^	1	0.0	0.0	181.4	0.0	0.0	171.7
	2	0.8	0.4	287.0	0.0	0.1	308.9
	3	2.3	2.9	64.6	1.6	1.5	58.8
**54**^c^	1	0.0	0.0	323.1	0.0	0.0	327.8
	2	1.2	1.1	184.5	0.9	1.6	187.9

a In kcal/mol.

b At M06-2*X*/6-311G(d,p).

c At M06-2X/def2-TZVP.

Remarkably, calculations for other α-anomers, namely **29**, **55**, **62**, also unveil this kind of hydrogen bonding (Figures S16–S18). Table S20 collects the calculated geometry of this intramolecular bond, whose strength can be determined by the empirical relationship (eq 2),^67^ where *d*_D···A_ is the calculated value.
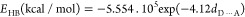
2

These values indicate that the anomeric N···HO hydrogen bond is moderately strong as it lies in the range from ∼7.8 (**55**) to 6.3 (**62**) kcal/mol in DMSO (Table S20, last column). However, the bond is weakened by ∼2 kcal mol^–1^ in salicylidene imines **32**, **34**, and **109** (with values of ∼5 kcal/mol), due to the existence of an intramolecular hydrogen bond between the nitrogen atom and the phenolic hydroxyl, which does not hamper formation of the anomeric N···OH hydrogen bond either.

### Anomeric Stabilization in Aldoses and 2-Aminoaldose Derivatives

At this stage, one wonders whether there is enough experimental evidence to support our preceding hypothesis. The extent of such interactions could be assessed in terms of the anomeric stabilization (*E*_an_) of various aldoses, 2-amino-2-deoxyaldoses, and their derivatives, as we shall delineate herein.

A central tenet of conformational analysis is that substituents on a cyclohexane ring prefer to adopt an equatorial rather than an axial arrangement for steric reasons.^40,47,68^ The parameter *A* for a substituent X is given by *A*_X_ = −Δ*G*°_steric_, where Δ*G*°_steric_ measures the variation of free energy in the axial ⇄ equatorial equilibrium for cyclohexane carrying the substituent X; that is, it measures the steric preference for the equatorial arrangement of a given substituent (eq 3)
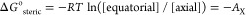
3

Thus, the *A*_OH_ value for the hydroxyl group in aqueous solution is 1.25 kca/mol and corresponds to an 89% predominance of cyclohexanol with the OH group placed in equatorial disposition (eq 4).^69^
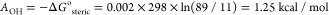
4

Similar considerations can be applied to substituted heterocycles, including the pyranose ring found in sugars. d-Glucose, the most abundant naturally occurring hexose, exists in aqueous solution at room temperature as a mixture, consisting of 64% of the β-anomer and 36% of the α-anomer.^69^ Δ*G*°_an_ is the observed free energy change for the balance between the axial and equatorial disposition, that is, α-anomer ⇄ β-anomer equilibrium (eq 5):
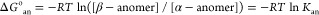
5

Anomeric stabilization (*E*_an_), defined as the nonsteric stabilization of the axial conformer, can then be quantified by correcting the axial preference of a substituent (Δ*G*^o^_an_) with the steric effects that favor an equatorial arrangement (Δ*G*^o^_steric_). The latter can be estimated on nonanomeric model compounds, with the *A*_*x*_ values of cyclohexane usually employed to this end (eq 6):
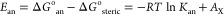
6

When one varies the substituents at nonanomeric positions, a quantitative relationship for the anomeric hydroxyl group can be expressed by eq 7:
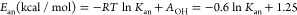
7

Table 4 shows the anomeric equilibrium data for several aldoses (**72**–**76**)^2,70^ and Table 5 those for 2-amino-2-deoxyaldoses and some derivatives (**1**, **3**, **77**–**81**).^2,3b,70,71^
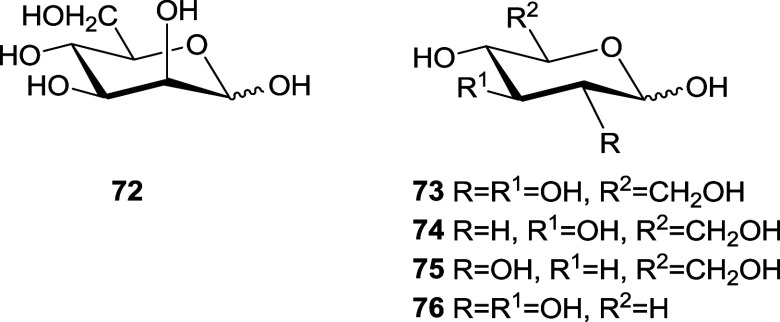


**Table 4:** Anomeric Composition (%) at Equilibrium of **72**–**76**^a^

parameter	**72**	**73**	**73**^b^	**73**^c^	**74**	**74**^d^	**75**	**76**
α-anomer	65.5	36.3	45.0	45.0	48.8	52.9	26.0	36.5
β-anomer	34.5	63.7	55.0	53.0	51.2	47.1	46.0	63.0
Δ*G*°	0.4	–0.3	–0.1	–0.1	0.0	0.1	–0.3	–0.3
*E*_an_^e^	1.6	0.9	1.1	1.2	1.2	1.3	0.9	0.9

a In D_2_O at 25 °C.

b In DMSO-*d*_6_ at 17 °C.

c In pyridine at 25 °C.

d In DMSO-*d*_6_ at 23 °C.

e Anomeric stabilization referred to cyclohexanol in kcal/mol.

**Table 5:** Anomeric Composition (%) at Equilibrium of **1**, **3**, **73**, and **77–82**^a^

parameter	**1**	**1**^b^	**3**	**73**	**77**	**78**	**79**	**80**^b^	**81**	**82**
α-anomer	63.3	87.0	66.0	36.3	39.0	23.9	25.0	80.0	83.0	90.0
β-anomer	36.7	13.0	34.0	63.7	61.0	76.1	75.0	20.0	17.0	10.0
Δ*G***°**	0.3	1.1	0.4	–0.3	–0.3	–0.7	–0.7	0.8	1.0	1.3
*E*_an_^c^	1.6	2.4	1.6	0.9	1.0	0.6	0.6	2.1	2.2	2.6
p*K*_a_(α)				12.7	12.6					
p*K*_a_(β)				12.4	12.4					

a In D_2_O.

b In DMSO-*d*_6_.

c Anomeric stabilization referred to cyclohexanol in kcal/mol.

For d-glucose (**73**), the β-anomer is the predominant species, even if the anomeric effect increases the proportion of the α-anomer three times larger than expected (*E*_an_ = 0.9 kcal/mol). The comparison of **74**–**76** shows that only the substituent at C-2 influences the anomeric balance. The *E*_an_ value that exhibits **74** (1.2 kcal/mol) should only reflect the value of the anomeric effect. The presence of equatorial OH at C-2 decreases the anomeric effect by (1.2–0.9 =) 0.3 kcal/mol, while an axial one (**72**) increases it by (1.6–1.2 =) 0.4 kcal/mol (Δ2 effect).^66^ This effect could probably be due to the elimination of the gauche interaction between the anomeric OH and that located at C-2. By switching to less polar solvents than water, the anomeric effect increases^25^ and thus for **74** in DMSO, *E*_an_ = 1.3 kcal mol (ΔΔ*G*° = 0.1 kcal/mol).
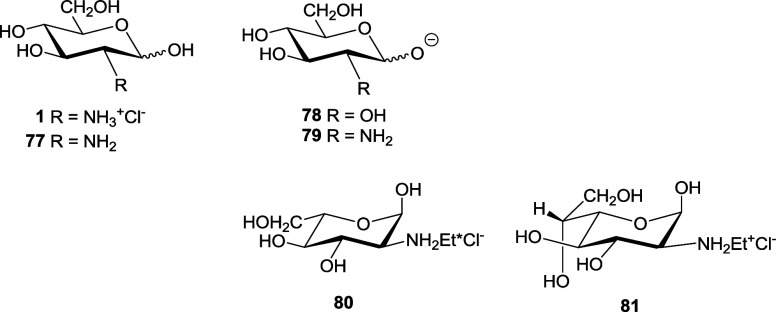


The substitution of the C-2 hydroxyl of d-glucose (**73**) by the NH_2_ group in d-glucosamine (**77**) scarcely changes the proportion of β-anomer. In contrast, the ionization of the anomeric hydroxyl (oxyanions **78** and **79**) appreciably increases the proportion of β-anomer. That the same ionized species is involved in both cases can be confirmed by the coincidence in p*K*_a_ values. The preference for the equatorial anomer may be caused by the steric effect generated by the increase in the sphere of solvation, due to the presence of the electric charge.

The anomeric equilibrium in hydrochlorides **1**, **3**, **80**,^71d^ and **81**(71e) seems to indicate that the interaction between the substituent at C-2 and the anomeric hydroxyl is of electronic and nonsteric character. The presence of longer aliphatic chains in *N*-alkyl-d-glucosamines (*n*-ethyl, *n*-propyl, *n*-pentyl, and *n*-hexyl) seldom alters the anomeric ratio (∼70% α-anomer in CD_3_OD).^71g^ In these cases, the lone pair of nitrogen is no longer available to interact with those of the anomeric hydroxyl, the destabilization disappears and the α-anomer predominates extensively, even though the steric hindrance increases significantly as there will now be a 1,3-diaxial (gauche) interaction between the hydroxyl of the α-anomer and the NH^+^. This would account for the anomeric behavior shown in Table 6, where the variation of the anomeric equilibrium of **77** and the aminodisaccharide chitobiose (**83**) is influenced by pH.
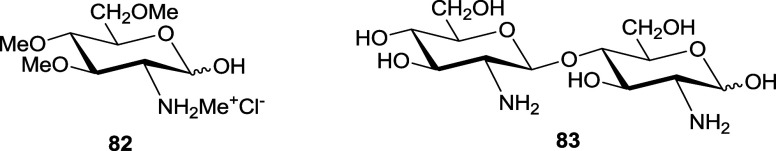


**Table 6:** Variation of the Anomeric Equilibrium of **77** and **83** with pH

	**acid pH**^c^				**basic pH**^d^			
**comp.**	**Α**	**β**	**Δ***G***°**	***E*_an_**^e^	**α**	**β**	**Δ***G***°**	***E*_an_**^e^
**77**^a^	53.3	46.7	0.1	1.3	29.3	70.7	–1.2	0.0
**77**^b^	55.0	45.0	0.1	1.4	36.7	63.3	–0.3	0.9
**83**^c^	70.0	30.0	0.5	1.8	46.5	53.5	–0.1	1.2

a Ref (70a).

b Ref (71).

c pD = 4.2–5.8.

d pD = 9.3–9.5

e Anomeric stabilization referred to cyclohexanol in kcal/mol.

The interaction of solvent molecules with the hydroxyls at C-3, C-4, and C-6 seldom affects the anomeric equilibrium of **80**. In fact, for 2-deoxy-2-methylamino-3,4,6-tri-*O*-methyl-d-glucopyranose hydrochloride (**82**), the α-anomer is the most abundant in D_2_O,^72^ despite having protected hydroxyl groups, which drastically alters the interaction with water molecules by reducing the number of possible intermolecular hydrogen bonds. Something similar happens with 2-acetamido-2-deoxyaldoses,^73^ where the lone pair on the nitrogen is not available because it is involved in a strong delocalization with the amide carbonyl, as demonstrated by the flat geometry of the amide bond and its poor basicity. Data for such derivatives are collected in Table 7.^2,71a,71f^
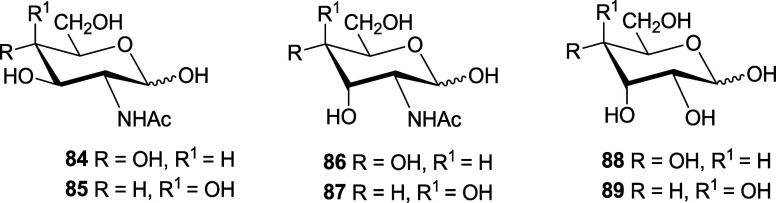


**Table 7:** Anomeric Composition (%) at Equilibrium of **1** and **84–89**^a^

**anomer**	**1**	**84**	**85**	**86**	**87**	**88**	**89**
Α	63	65.8	65	14	17	14	16
Β	37	34.2	35	72	74	77.5	78
Δ*G***°**	0.3	0.4	0.4	–1.0	–0.9	–1.0	–1.0
*E*_an_^b^	1.6	1.6	1.6	0.3	0.4	0.2	0.3
Δ*G*°_rae_	–0.3	–0.3	–0.3	1.1	1.0	1.1	1.0

a In D_2_O.

b Anomeric stabilization referred to cyclohexanol in kcal/mol.

It is evident, from the cases of **86** and **87**, that the 1,3-diaxial interactions between the hydroxyl at C-3 and the anomeric carbon destabilize the α-anomer. Similar values of *E*_an_ are found in aldoses **88** and **89** having identical configurations.

### Anomeric Equilibrium in Imines and Enamines of 2-Amino-2-deoxyaldoses

In line with the preceding section, the existence of an electronic interaction overcoming purely steric effects would justify the anomeric behavior observed in Schiff bases. Those with an imine structure will show a large preference for an equatorial arrangement of the anomeric hydroxyl. Table 8 collects data from illustrative examples of previously studied equilibria. In all cases, the *E*_an_ becomes ∼0 kcal/mol, regardless of the donor/attractor or hydrophilic/hydrophobic character of the aromatic substituents.

**Table 8:** Anomeric Stabilization (kcal/mol) at Equilibrium of Imines Derived from d-Glucosamine^a^

	**compound**									
parameter	**9**	**12**	**13**	**14**	**15**	**16**	**17**	**18**	**19**	**20**
α^b^	28.5	13.1	11.3	9.4	12.2	12.4	10.5	12.3	7.7	7.4
β^b^	81.5	86.9	88.7	90.6	87.8	87.6	89.5	87.7	92.3	92.6
Δ*G*°	–0.6	–1.1	–1.2	–1.4	–1.2	–1.2	–1.3	–1.2	–1.5	–1.5
*E*_an_^c^	0.6	0.1	0.0	–0.1	0.1	0.1	0.0	0.1	–0.2	–0.3
Δ*G*°_rae_	0.7	1.2	1.3	1.4	1.3	1.2	1.4	1.3	1.6	1.6

	compound								
parameter	**21**	**22**	**23**	**24**	**25**	**26**	**27**	**28**	**30**
α^b^	12.4	10.5	11.1	11.2	10.3	9.7	10.7	11.2	13.0
β^b^	87.6	89.5	88.9	88.8	89.7	90.3	89.3	88.8	87.0
Δ*G*°	–1.2	–1.3	–1.3	–1.2	–1.3	–1.3	–1.3	–1.2	–1.1
*E*_an_^c^	0.1	0.0	0.0	0.0	–0.1	–0.1	0.0	0.0	0.1
Δ*G*°_rae_	1.2	1.4	1.3	1.3	1.3	1.2	1.3	1.3	1.2

a In DMSO-*d*_6_.

b In %.

c Anomeric stabilization referred to cyclohexanol.

When the values of Δ*G*° (Table 8) are plotted as a function of Hammett σ constants^74^ (excluding ortho-substituted compounds), a linear relationship is obtained: Δ*G*°= −0.28σ – 1.25 (*r* = 0.819) (Figure S19). The low value of the slope indicates a low dependence on the electronic effect of the substituents. The basicity of the lone pair on nitrogen is altered through inductive effects only, since the axis of the σ orbital containing the pair lies in the nodal plane of the π system of the arylimino group.

To quantify the magnitude of the reverse anomeric effect in imines (Δ*G*°_rae_), this parameter can be determined as the difference between the stabilization due exclusively to the anomeric effect (Δ*G*°_ae_) minus the anomeric stabilization in imines (Δ*G*°_imine,_Table 8). If we take the anomeric effect as the value of *E*_an_ shown by **74** in DMSO-*d*_6_ (= 1.3 kcal/mol, Table 4), eq 8 is obtained:
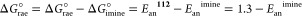
8

Results in Table 8 show that the reverse anomeric effect in imines adopts values in the range 1.6–1.2 kcal/mol, depending on the electronic character of the aromatic substituents. In enamines derived from 2-amino-2-deoxyaldoses, the electron pair on the nitrogen atom is involved in a system that is extensively delocalized, which renders it unsuitable for an electronic interaction with the axial anomeric hydroxyl (Table 9). Thus, enamines **5** and **6**, after equilibration with their β-anomers (such as **90**) in DMSO-*d*_6_, show a higher α-anomeric preference (*E*_an_ ∼ 2.3 kcal/mol). It seems clear that the absence of the destabilizing electronic interaction and the impossibility of establishing a hydrogen bond between the anomeric hydroxyl and the enamine nitrogen atom are responsible for this experimental fact; in other words, both the endo- and exo-anomeric effects now dictate the anomeric preference.

**Table 9:** Anomeric Composition (%) at Equilibrium for Imines and Enamines of **1** and **3**^a^

		**compound**									
**anomer**	**5**	**6**	**9**	**32**	**34**	**36**	**39/40**	**91**	**92**	**93**	**94**
Α	85.0	84.7	18.5	25.6	37.4	84.8	68.0^b^	21.0	22.9	70.0	74.0
Β	15.0	15.3	81.5	74.4	62.6	15.2	32.0^c^	79.0	77.1	30.0	26.0
Δ*G*°	1.0	1.0	–0.9	–0.6	–0.3	1.0	0.5	–0.8	–0.7	0.5	0.6
*E*_an_	2.3	2.3	0.4	0.6	0.9	2.3	1.7	0.5	0.5	1.8	1.9
Δ*G*°_rae_	1.0	–1.0	1.0	0.7	0.4	–1.0	–0.4	0.9	0.8	–0.4	–0.6

a In DMSO-d_6_.

b Sum of the two α-anomers.

c Sum of the two β-anomers.

Schiff bases carrying an ortho-hydroxyl substituent and having an imine structure behave similarly and, like other imines, show a predominant formation of equatorial anomer (Table 9), although the effect is less pronounced. This may be due to the participation of the lone pair on nitrogen in the formation of an intramolecular hydrogen bond with the phenolic hydroxyl. This H-bond could then reduce the electronic repulsion to some extent, without eliminating it completely and competing with the formation of hydrogen bonding with the anomeric hydroxyl. In order to verify the anomeric behavior of imines and enamines from ortho-hydroxybenzaldehydes, we prepared a series of Schiff bases derived from salicylaldehyde and its 4-hydroxy- and 4,6-dihydroxyderivatives **32**,^21a^**34**,^22^**39**/**40**,^23^**91**, and **92**,^75^ along with those derived from 2-hydroxy-1-naphthaldehyde **93**(20b) and **94**.^3b^ As pointed out earlier adducts **32** and **34** adopt an imine structure in solution, predominating the β-anomer; on the other hand, for enamines **39**/**40** and **93**, the α-anomer becomes the major isomer in solution.
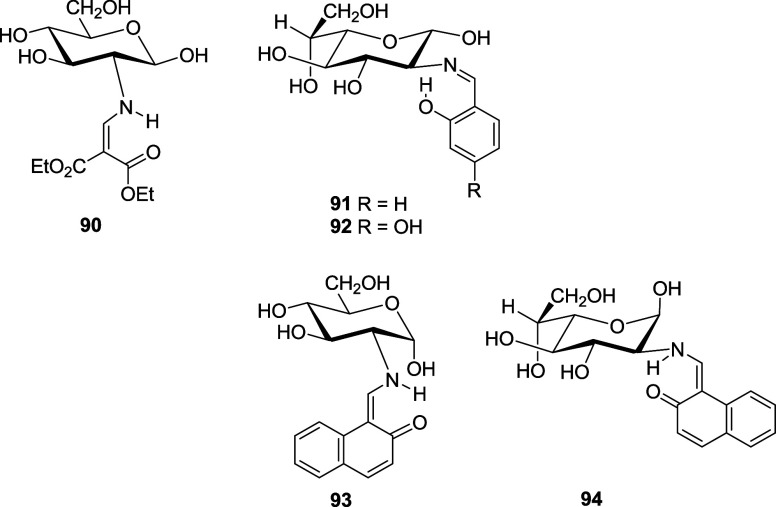


**Scheme 4:**
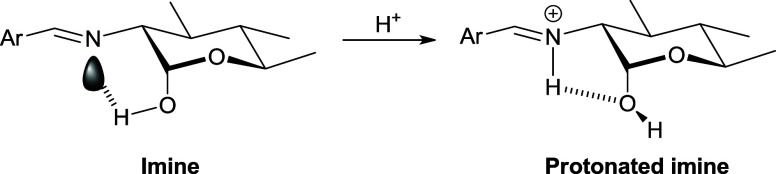
Imine Protonation-induced Restoration of the Exo-Anomeric Effect

A remarkable result is shown by hydrochloride **36**,^22^ which shows a complete reversal of the anomeric percentages when compared to **32** and an *E*_an_ coincidental with that of **5** and **6**. The free energy variation for protonated **36/37** (or deprotonated **34**/**35**) is 1.34 kcal/mol. Like the case of hydrochlorides **1**, **3**, **80**, and **81**, nitrogen protonation removes the destabilizing electronic interaction and the exoanomeric effect plays a dominant role. In addition, a strong intramolecular hydrogen bond is probably established between the NH^+^ and the axial anomeric hydroxyl (α-anomer), as described for 2-aminocyclohexanol derivatives,^76^ which restores the exoanomeric effect (Scheme 4). The conformational “rigidity” of the imine group makes it difficult the formation of this hydrogen bonding in the β anomer.

The behavior of imines and enamines derived from **3** is parallel to that adopted by the imines of **1**, which is logical since the former shows for the pyranose ring an enantiomeric relationship (l-gluco) to that of **1** (d-gluco). Therefore, in imines **9**, **91**, and **92**, the β-anomer, which has an equatorial hydroxyl at C-1, predominates significantly at equilibrium. However, when the Schiff bases derived from *o*-hydroxyaldehydes adopt the enamine structure, such as **94**, the α-anomer predominates in equilibrium, consistent with the cases of enamines **4–6**.

The calculated anomeric effect (as *E*_an_) in Tables 4–9 are approximate values. The C–O bonds in tetrahydropyrans are shorter than in cyclohexanes, and hence, the steric interactions in the axial conformation of the substituent are more intense in the former. Therefore, the values of *A*_X_ in tetrahydropyrans are greater than those obtained for cyclohexanes. The values of *A*_X_ in these rings (*A*_X_^cyclohex^) can be extrapolated approximately to the corresponding value in a tetrahydropyran ring (*A*_X_^THP^) by means of eq 9:^77^
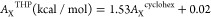
9

The *A*_OH_^THP^ value for the hydroxyl group in pyranoid molecules takes the value of 1.93, and the corresponding values of *E*_an_ would increase by 0.68 (=1.93–1.25) kcal/mol.

### Anomerization of Per-*O*-acetylimines and Per-*O*-acetyl-2-(arylmethylene)amino-d-glucopyranosyl Bromides

**Scheme 5:**
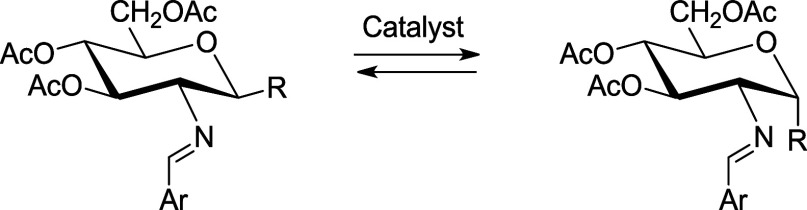
Attempted Acid-catalyzed Anomerization of *O*-Protected Imine Derivatives

To evaluate the anomeric preference of imines in the absence of hydrogen bonding between the anomeric hydroxyl and the imine nitrogen, anomerization experiments with per-*O*-acetylimines (R = OAc) and per-*O*-acetyl-2-(arylmethylene)amino-d-glucopyranosyl bromides (R = Br) were attempted using Brönsted and Lewis acid catalysts (Scheme 5). Unfortunately, equilibration between the corresponding anomers could not be detected at all (for further details, see the Supporting Information).

### Anomerization of Anomerically Unprotected Acylated Schiff Bases

To extend the mutarotational equilibrium study to less polar solvents, some anomerically unprotected derivatives such as **48**, **50**, **52**, **82**, **95**,^78^ and **96**(75) were explored.
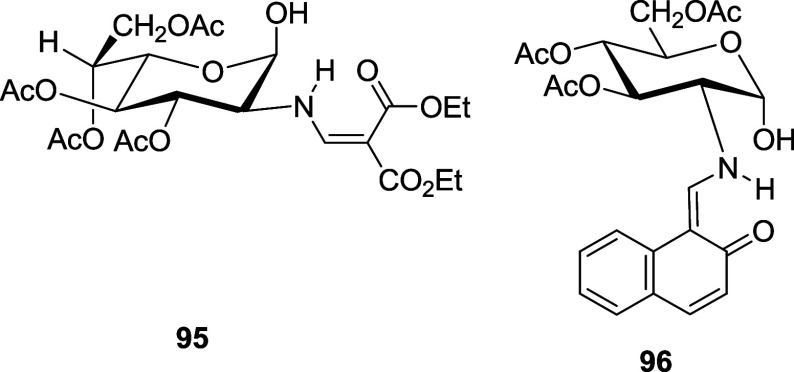


In CDCl_3_ solutions, compounds having an enamine structure (**48**, **95**, and **96**) show a higher proportion of the α-anomer at equilibrium (coincidental with those of **5** and **6**, Table 9), like the case of hydrochloride **82** (Table 10, *E*_an_ = 2.6 kcal/mol). On the other hand, the β-anomer predominates in imine **50/51** (*E*_an_ = 1.0). Again, the presence of an intramolecular hydrogen bond in **52/53** reduces the amount of β-anomer (*E*_an_ = 1.3 kcal/mol). Thus, the proportion between both anomers in equilibrium, at room temperature and in CDCl_3_ solution, is practically identical: 51% of α-anomer and 49% of β-anomer (Table 10).

**Table 10:** Anomeric Composition (%) at Equilibrium of Anomerically Unprotected Schiff Bases

	**compound**						
**anomer**	**48**^a^	**48**^b^	**50/51**^a^	**52/53**^a^	**82**^b^	**95**^b^	**96**^a^
α	86.0	91.6	41.0	51.0	90.0	84.0	84.1
β	14.0	8.4	59.0	49.0	10.0	16.0	15.9
Δ*G*°	1.1	1.4	–0.2	0.0	1.3	1.0	1.0
*E*_an_	2.3	2.7	1.0	1.3	2.6	2.2	2.3
Δ*G*°_rae_	–1.2	–1.4	0.3	0.1	–1.3	–0.9	–0.9

a In CDCl_3_

b In DMSO-*d*_6_.

Therefore, the existence of an anomeric hydroxyl is necessary for imines to have a reverse anomeric effect. From the above facts and results discussed, it seems to be a conclusive statement that an imino group is capable of inducing a reverse anomeric effect. The magnitude of this effect would be comparable to that of the anomeric effect generated by the hydroxylic group, because in most cases the former counterbalances, and even surmounts the anomeric effect. The stereoelectronic interaction and/or the hydrogen bonding prevent the exo-anomeric effect acting on the α-anomer.

### Theoretical Analysis of Anomeric Equilibrium in Imines of 2-Amino-2-deoxyaldoses

A detailed computational study has been performed in search for theoretical validation of a reverse anomeric effect. As pointed out earlier (vide supra, on the origin of anomeric stabilization), three models or their combinations could account for a genuine stereoelectronic effect, namely, (1) repulsive interactions between heteroatom lone pairs, (2) intramolecular H-bonding between the anomeric OH and the lone pair on the imino group that annihilates the exoanomeric effect, and (3) differential stabilization of the anomers induced by solvation.

To rule out the possibility that hydrogen bonds are responsible for the anomeric behavior, we first studied the acetylated derivatives **43**, **46**, and **97**–**104**. In such compounds, hydrogen bonding cannot be formed, but a repulsive stereoelectronic effect could manifest itself. Calculations in the gas phase and chloroform (ε = 4.8, SMD method) are shown in Table S21.

1,3,4,6-Tetra-*O*-acetyl-2-deoxy-d-glucopyranose (**97**) has been taken as reference compound, since the stereoelectronic effect is impossible or at least nonexistent. As expected, due to the anomeric effect, the α-anomer is more stable than the β-form, both in the gas phase and in chloroform. The presence of heteroatoms at C-2, such as fluorine (**98**), acetate (**100**), or acetamido (**102**) groups, does not produce appreciable changes, and the α-anomer is invariably more stable than its β-counterpart. In fact, the difference in stability increases among the anomers. However, with the hydroxyl (**99**) and amino (**101**) groups, even if the α-anomer remains the most stable isomer, the stability difference between anomers is significantly reduced. Also the α-anomer of imine **46** is more stable than its β-anomer (**43**).
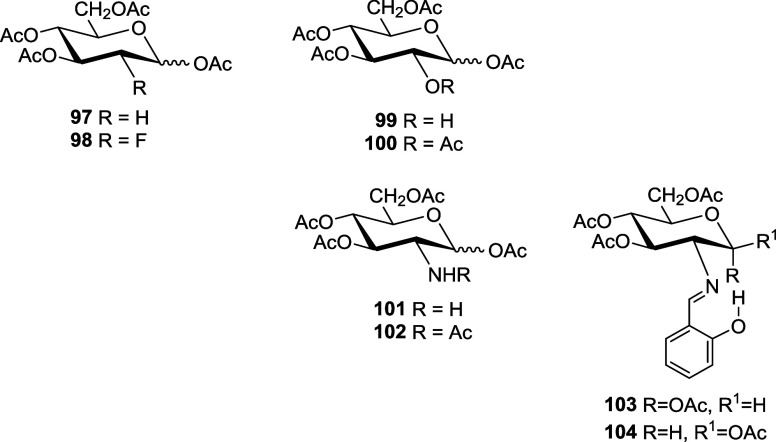


Calculations were performed on per-*O*-acetylsalicylidene derivatives **103** and **104**, trying to determine how the intramolecular hydrogen bond involving the iminic nitrogen would affect the relative stability of the anomers. In striking contrast to previous calculations, at both levels of calculation the β anomer turned out to be the most stable, irrespective of gas phase or in CHCl_3_. These results, however, are not totally conclusive to demonstrate a stereoelectronic effect caused by the repulsion of lone pairs, which, if any, should be nonexistent or very small.

Next, we moved to the unprotected imines, since the impact of hydrogen bonding on the anomeric effect is evident. The species involved in mutarotational equilibria have been evaluated in the gas phase, aqueous solution (ε = 80.1) and DMSO (ε = 46.8). We started with the simplest pair of anomers **11**/**54**, for which the α-anomer is slightly more stable than the β-form (Table S16). Experiments, however, indicate that the presence of phenylimino group in **11** causes a greater preference for the β-anomer. To ascertain the influence of substituents at the aromatic ring on the tautomeric equilibrium, the energies of imine anomers derived from 4-methoxybenzaldehyde (**12**/**55**), 4-nitrobenzaldehyde (**19**/**62**), and 2,4,6-trimethylbenzaldehyde (**29**/**30**) were estimated as well. The first two disclose the effect caused by electron-donating and electron-withdrawing groups, respectively, while the third anomeric pair reflects putative steric effects. Again, however, the α-anomers are slightly more stable than the β-ones, and neither appreciable electronic nor steric effects are detected in DMSO (Table S22, Figures S16–S18). Likewise, the influence of intramolecular H-bonding on salicylaldehydes has been determined by computing the imine and enamine structures derived from salicylaldehyde (**32**/**33, 105**/**106**) and its 4-hydroxy (**34**/**35, 107**/**108**) and 4,6-dihydroxy (**109**-**112**) derivatives (Table S23).
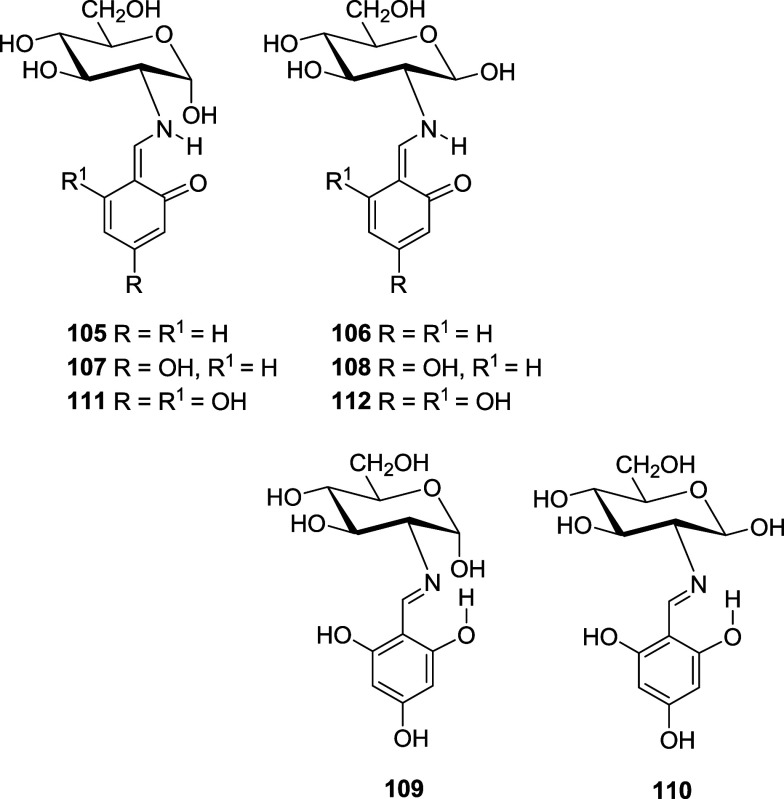


Results show that imine structures are more stable than the corresponding enamines, and the increase of phenolic hydroxyls decreases the energy difference between both tautomers. The β-configured imine is always the most stable form, although the difference in stability with respect to the α-anomer decreases as solvent’s polarity increases (DMSO). The opposite happens if the compound in question adopts an enamine structure. Both the stability differences between the tautomeric structures and between α- and β-anomers decrease as the dielectric constant of the medium increases. These results agree with the experimental observations; in solution, *o*-salicylaldehyde and 2,4-dihydroxybenzaldehyde derivatives behave as imines (**32**/**33** and **34**/**35**, respectively). It is noteworthy that the theoretical analysis unveils the greater stability of β-anomers **33** and **35**, in perfect agreement with the experimental results. The reverse anomeric effect in imines **33**, **35**, and **110** decreases as the hydrogen bond involving the anomeric hydroxyl weakens, owing to the competitive H-bonding with the phenolic hydroxyl.

**Scheme 6:**
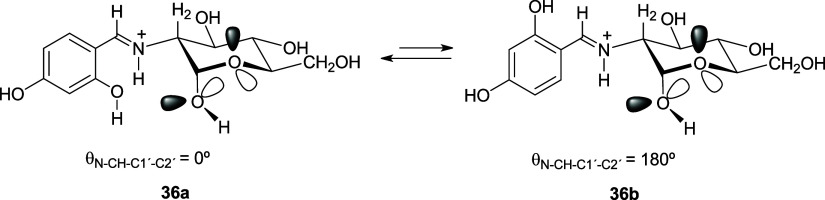
Conformational Dispositions for the Aromatic Fragment of Anomer **36**

**Figure 10:**
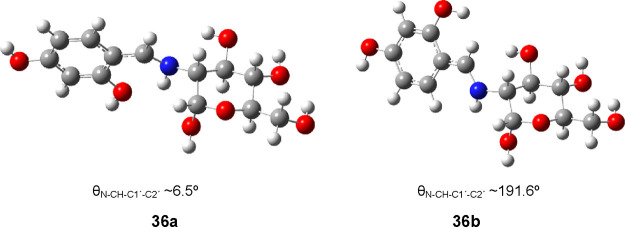
Optimized conformations for the aromatic residue of **36** in DMSO, at the M06-2*X*/6-311G(d,p) level.

As already mentioned, the surprising behavior of hydrochloride **36** in solution is noticeable, because the anomeric distribution is completely reversed when compared to its conjugate base **34** (see Table 9). In the preferred conformation based on experiments in solution, the protonated imino fragment is arranged in a similar way as the rest of the imines; that is, perpendicular to the average plane of the pyranose ring (θ_H2–C2–N-CH_ ∼0°), as evidenced by a large coupling constant *J*_H-2,NH_ (∼15 Hz). In addition, we took into account the two possible conformations of the aromatic fragment for both anomers: not only the one resulting from protonation of the more stable conformation of **34** or **35** (**36a**/**37a**, θ_N–CH-C1′-C2__′_ −0°), but also that arising from a 180° degree turn (**36b**/**37b**, θ_N–CH-C1′-C2′_ ∼180°). Scheme 6 depicts these two conformations for the α-anomer. The relative stabilities are compiled in Table S24, and Figure 10 shows the optimized structures of the two possible conformations of the aromatic moiety.

Conformers **36a** and **37a** (θ_=CH-C1′-C2′-O_ ∼0°) are more stable than **36b** and **37b**, probably because in the former a weak intramolecular hydrogen bridge forms between the NH^+^ and the phenolic OH group (Table S25). However, the α-anomer is the most stable isomer regardless of either conformer, both in gas phase and solution, which fully agrees with experimental results. This fact can be explained in terms of the hydrogen bond between the NH^+^ group and the anomeric oxygen atom,^76^ thus anchoring a conformation in which the *exo*-anomeric effect is maintained.

A more realistic view of mutarotational equilibria should involve the effect of discrete solvation, an otherwise approach employed to reproduce successfully the anomeric behavior in aldoses.^79^ To alleviate the computational cost, the anomeric pair **11**/**54** was initially chosen as model compounds and water molecules as solvent. To determine the most stable hydrate structures arising from solvation with one or more water molecules, calculations were first performed at the B3LYP/6-31G(d), followed by further reoptimization of geometries and energies at the B3LYP/6-31G(d,p) and M06-2*X*/6-311G(d,p) levels (Tables S26 and S27). In all cases, the dihedral angle θ_H2–C2–N=C_ lies in the range from −31 to +37°.

**Figure 11:**
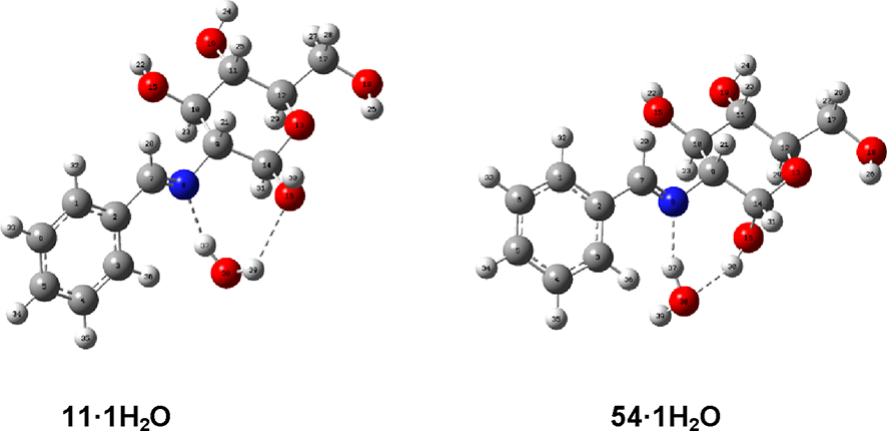
Optimized structures of anomers **11** and **54** as monohydrates.

In the absence of discrete solvent molecules, the α-anomer (**54**) is slightly more stable than its β-counterpart (**14**), with a difference in stability that increases in DMSO and then decreases as the dielectric constant rises (water). By considering the association with a single molecule of water, it seemed logical that interaction with the sugar moiety would preferentially occur with the iminic nitrogen as the most basic center. In both anomers, the water molecule establishes hydrogen bonding with the iminic nitrogen and the anomeric hydroxyl. Figure 11 shows the structure of monohydrate structures derived from **11** and **54** (see Table S28 for geometrical parameters of such H-bonds).

In general, our calculations indicate that the α-anomer is the most stable species and hydration increases the α/β relationship far from the experimental result. However, the stability difference decreases as the dielectric constant increases, being virtually identical in water at the M06-2*X*/6-311G(d,p) level, while the B3LYP/6-31G(d,p) method favors the β-anomer as the most stable isomer.

**Figure 12:**
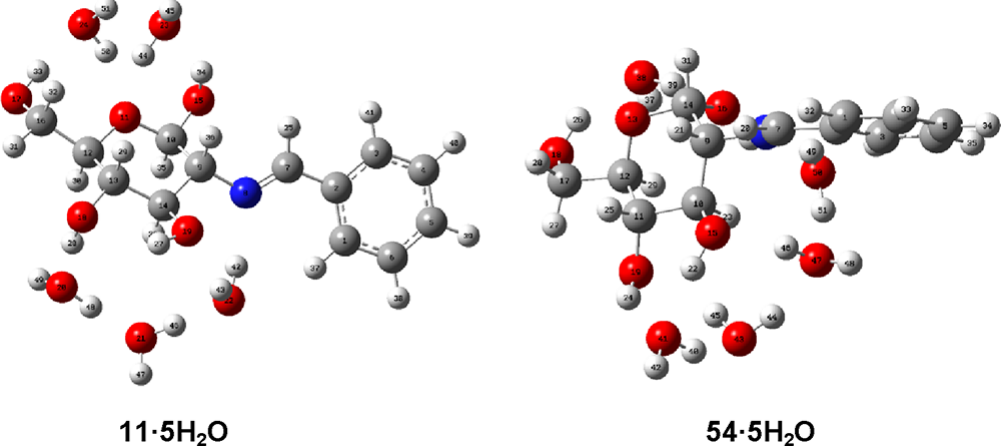
Optimized structures of pentahydrates derived from **11** and **54**.

Solvation with additional water molecules was then taken into account. Given that there are four hydroxyls, interaction with four water molecules through H-bonds results in the first solvation shell of the imine. Figure 12 shows the optimized structures of both pentahydrates, while the geometrical description of such H-bonds is gathered in Table S28. The pentahydrate form of the β-anomer becomes the most favored species by considering solvation (M06 and SMD methods), in agreement with the experimental results in solution (Table S27).

**Figure 13:**
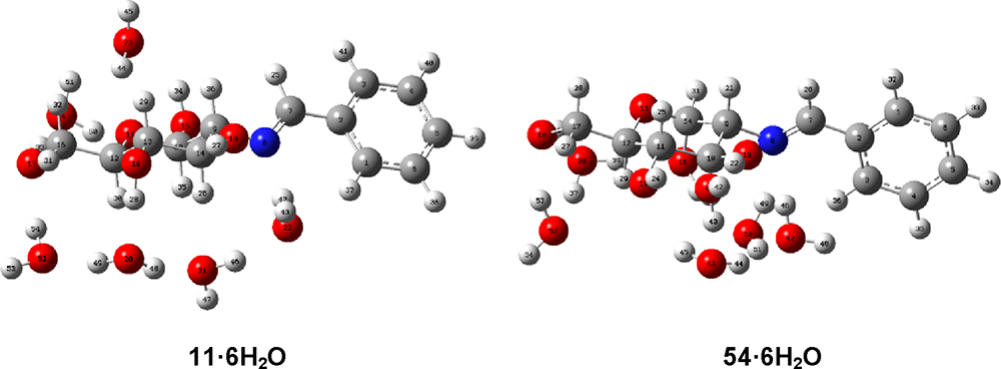
Optimized structures of hexahydrates derived from **11** and **54**.

Moreover, the introduction of a sixth water molecule increases stability still further and, therefore, the predominance of the β-anomer (Figure 13). Similar results were obtained for the corresponding hexahydrates of anomer pairs **12**/**55** and **19**/**62**, regardless of the electronic character of the substituents at the aromatic ring (Table S27). It is evident that both the progressive hydration and polarity of the medium favor the β anomer to a great extent.

For the hydrated α-anomers calculated above, a water molecule is positioned between the anomeric hydroxyl and the imine nitrogen atom, which could hamper either the partial or complete elimination of the exo-anomeric effect. An NBO analysis of mono and pentahydrate forms shows stabilizing hydration interactions (Tables S29–S33) similar to those found in the absence of water of hydration (Tables S18 and S19). The β-anomers display an appreciable exo*-*anomeric effect (∼14–17 kcal/mol) and a small anomeric effect (∼5 kca/mol) (Table S29). In contrast, for α-anomers both effects become large: the anomeric effect is ∼11–14 kcal/mol, while the exo-anomeric effect gives rise to a similar strength (∼15–16.5 kcal/mol).

However, when the water molecule between the nitrogen and the anomeric hydroxyl is removed, calculations show an even greater stability of the β-anomer (Table 11), which arise from restoring in the α-anomer the H-bonding between the anomeric hydroxyl and the nitrogen atom, along with partial cancellation of the exo-anomeric effect, as shown by the NBO analysis (∼ 2.5–7 kcal/mol, last column in Tables S31–S33). Based on calculated geometrical data, eq 2(67) shows that the hydrogen bond between the nitrogen and anomeric OH takes values of ∼7–8 kcal/mol (Table S36). In addition, we have performed a natural bond orbital analysis of steric interactions (STERIC),^36^ which shows that the possible repulsive interactions **a**–**c**, illustrated in Figure 7, are either negligible or are nonexistent (less than 0.5 kcal/mol). When the pairwise steric exchange energies associated exclusively with molecules **11** and **54** are considered, the difference between both anomers, Δd*E*(i,j) = d*E*(i,j)^α^ – d*E*(i,j)^β^, is 7.5 kcal/mol (= 593.2–585.7), favorable for the equatorial anomer (β). The same result is obtained if the five water molecules associated by hydrogen bonds to both anomers are also taken into account. In this case, the total pairwise steric exchange energies difference Δd*E*(i,j) is 7.6 kcal/mol (= 690.0–682.4). Analogous results are achieved when the effect of the solvent (DMSO) is considered, thus obtaining values of 5.8 and 7.6 kcal/mol, respectively (Tables S34 and S35).

**Table 11:** Calculated Relative Energies (kcal/mol) for Five Hydrated Anomers^a^

		**gas phase**^b^		**DMSO**^b^			**water**^b^		
anomer		Δ*E*	Δ*G*	Δ*E*	Δ*G*	[β]^d^	Δ*E*	Δ*G*	[β]^d^
**11**·5H_2_O	β	0.0	0.0	0.0	0.0	86.2	0.0	0.0	97.9
**54**·5H_2_O	α	–3.8	–2.6	–1.5	1.1		1.2	2.3	
**12**·5H_2_O	β	0.0	0.0	0.0	0.0	99.7	0.0	0.0	95.6
**55**·5H_2_O	α	3.0	2.6	2.5	3.5		3.9	1.9	
**19**·5H_**2**_O	β	0.0	0.0	0.0	0.0	89.7	0.0	0.0	98.2
**62**·5H_2_O	α	–3.0	–1.4	–0.2	1.3		1.0	2.4	

		gas phase^c^		DMSO^c^			water^c^		
**11**·5H_2_O	β	0.0	0.0	0.0	0.0	88.1	0.0	0.0	96.0
**54**·5H_2_O	α	–2.4	–0.7	0.1	1.2		1.8	1.9	
**12**·5H_2_O	β	0.0	0.0	0.0	0.0	76.3	0.0	0.0	98.9
**55**·5H_2_O	α	2.5	1.6	2.4	0.7		3.3	2.7	
**19**·5H_2_O	β	0.0	0.0	0.0	0.0	91.2	0.0	0.0	96.6
**62**·5H_2_O	α	–1.6	–0.1	1.1	1.4		1.6	2.0	

a In kcal/mol.

b At M06-2X/6-311G(d,p).

c At M06-2X/def2-TZVP.

d Calculated percentage of the β-anomer (in %) from eq 1.

Data obtained with the 6-311G(d,p) and def2-TZVP bases are similar. For the latter, the energy differences between both anomers correspond to a predominance of the equatorial anomer (β), in the mutarotational equilibrium, from 96 to 99% in water (ε = 80.1) and from 76 to 91% in DMSO (ε = 46.7) (Table 11, eq 1), which are practically coincidental with the experimental ones (Table 8). An increase in solvent polarity boosts further the calculated proportion of the equatorial anomer.

It is obvious that other arrangements involving coordination with five or six water molecules could be considered. This would imply studies with statistical methods and a higher number of penta and hexahydrate structures, which goes beyond the aims of this work.

This computational analysis show that the inverse anomeric effect presented by 2-aminoaldose imines in solution is due to the conjunction of at least two effects: (a) the increase in solvation/polarity of the medium and (b) the total or partial inhibition of the exo-anomeric effect by formation of hydrogen bonding between the anomeric hydroxyl and the imine nitrogen atom.

The anomeric preferences observed in other sugars and aminosugars may also be influenced by phenomena similar to those just described. Thus, for example, the inversion in the anomeric proportion of 2-acetamido-2-deoxyaldopyranoses having both d-allo and d-gulo configuration (**86** and **87**) with respect to those with d-gluco and d-galacto configurations (**84** and **85**) (Table 7) may be due to the inhibition of the exoanomeric effect by forming a hydrogen bond between the anomeric hydroxyl and the one located at C-3 (**113**). Similar effects could occur with aldopyranoses (**114**) or with appropriately configured 3-amino-3-deoxyaldopyranoses (**115**, **116**) and their Schiff bases (**117**, **118**).
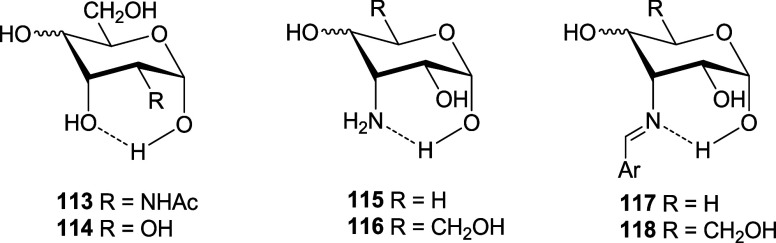


## Conclusions

All the imines obtained from 2-amino-2-deoxyaldoses (**1–3**) crystallize as equatorial (β) anomers. An exception is the imine derived from **1** and 2,4,6-trimethylbenzaldehyde, which sometimes crystallizes as the axial (α) anomer. The latter represents the first solid-state α-anomer obtained in the series of sugar imines bearing unprotected hydroxyls. In solution, however, an equilibrium between α- and β-anomers can be detected in which the latter largely predominates. At first glance, this behavior can be regarded as an apreciable “reverse” anomeric effect with values in the ranges of 1.2–1.6 or 1.9–2.3 kcal/mol. In salicylimines that the reverse anomeric effect is reduced and disappears completely when they adopt enamine structures. Also, the effect vanishes by protonation of the imino group.

The experimental findings show a preference, or stabilization, of the equatorial anomer, to an extent that not only neutralizes but also even exceeds the anomeric effect. All imines adopt a conformation in which the flat arylimine moiety is located approximately perpendicular to the midplane of the tetrahydropyran ring, with the lone pair on the imine nitrogen parallel to the axial protons. The existence of a stereoelectronic effect, resulting from interaction between the lone pair of the imine nitrogen and the anomeric oxygen and/or hydrogen bonding between the anomeric hydroxyl and the imine nitrogen, could account for this rather anomalous behavior.

Theoretical calculations show that there is no stereoelectronic effect resulting from a repulsive interaction involving the lone pairs on the imine nitrogen and the anomeric oxygen that opposes the anomeric effect. However, computation does unveil the elimination of the exoanomeric effect in the axial anomer (α). This should be ascribed to hydrogen bonding formation between the anomeric hydroxyl and iminic nitrogen, which along with solvent effects (in terms of discrete solvation) provide enough evidence supporting the preferential formation of the equatorial anomer (β), beyond a purely steric effect.

Therefore, one can reasonably conclude that 2-amino-2-deoxyaldose imines exhibit a true reverse anomeric effect, whose origin, like the anomeric effect, results from the conjunction of several interactions favoring the equatorial preference of the anomeric hydroxyl, while reducing/eliminating the stabilizing exo-anomeric effect. All these interactions may be present in other molecules carrying atoms or functional groups with unshared electrons such as hydroxyl in aldoses or amino groups in 2-amino-2-deoxyaldoses. Our results and the previous literature suggest the possibility of manipulating the properties and chemical behavior by varying substituents at the C-2 position of sugars, rather than at other positions. In the context of the present work, monosaccharides deserving further attention include d-mannose (**72**), 2-amino-2-deoxy-d-glucose (**1**), 2-amino-2-deoxy-d-galactose (**2**), 2-amino-2-deoxy-d-mannose, their 2-acetamido derivatives (**84**, **85**), and, in general, 2-heterosubstituted-2-deoxysugars.

## Experimental Section

### General Information

All characterization and spectroscopic elucidation methods and crystal acquisition data are included with the Supporting Information. All solvents and reagents were obtained from commercial suppliers and used without further purification. Compounds **3**,^3b^**5**,^4c^**6,**^5a^**9**,^5a^**10**,^5a^**11**,^19^**12**,^6^**13**,^9^**15**,^11b^**16**,^9^**18**,^20b^**19**,^20a^**21**,^9^**22**,^9^**32**,^21^**34**,^22^**36**,^22^**39**/**41**,^23^**44**,^6^**45**,^4b^**47**,^24a^**48**,^26^**49**,^26^**80**,^71d^**81**,^71e^**91**,^75^**92**,^75^**93**,^20b^**94**,^3b^**95**,^78^ and **96**(75) have been synthesized as described in the previous literature.

Crystal data and structure refinement for compound **44** were collected for a crystal with dimensions 0.60 × 0.40 × 0.10 mm^3^ using a Nonius Kappa CCD diffractometer (ϕ scans and ω scans to fill asymmetric unit sphere). All hydrogen atoms were placed in idealized positions and refined using a riding model. Crystallographic data for this compound have also been reported in an independent study by Bräse and co-workers.^80^

### Synthesis of Schiff Bases

New and reported substances were obtained according to the following general procedures. Full description of characterization data and spectra for every compound are provided with the Supporting Information. Method 1: To a solution of **1** (5.0 g, 23.2 mmol) in 1M NaOH (25 mL) is added the appropriate aromatic aldehyde (25.0 mmol) and the mixture is stirred at room temperature. A solid usually precipitates, which is collected by filtration and washed successively with cold water, cold ethanol, and ethyl ether, and dried under vacuum on silica gel. Method 2: To a solution of **1** (1.0 g, 4.7 mmol) and sodium acetate (0.63 g, 7.7 mmol) in water (10 mL) was slowly added a solution of the appropriate aromatic aldehyde (4.7 mmol) dissolved in methanol (saturated solution). The mixture was stirred at room temperature for 2 h. After crystal formation, the mixture was cooled at ∼5 °C (refrigerator). As above, the product was filtered and washed with cold water, chilled absolute ethanol, and ethyl ether, and dried under vacuum over silica gel. Method 3: Sodium hydrogen carbonate (0.50 g, 6.0 mmol) was added to a solution of **1** (1.0 g, 4.7 mmol) in water (6 mL). To the resulting mixture, a solution of the appropriate aromatic aldehyde (4.7 mmol) in methanol (saturated solution) was added dropwise. The mixture was stirred at room temperature until precipitation and then left in the refrigerator (∼5 °C) overnight. The solid was collected, washed with cold water, ethanol, and ethyl ether, and dried in vacuo.

### Synthesis of Anomerically Unprotected Compounds

To a suspension of **49** (1.16 g, 3.0 mmol) in 96% aqueous ethanol (14 mL) was added a solution of anhydrous sodium acetate (0.25 g, 3.0 mmol) in water (2 mL) and the corresponding aldehyde (3.0 mmol). The mixture was stirred for 5 min at room temperature, and then, it was poured into ice-water, and the aqueous phase was extracted with CHCl_3_ (3 × 50 mL). The resulting organic phase was washed with a saturated aqueous solution of NaHCO_3_ (50 mL) and water (50 mL), dried (MgSO_4_), and dried under vacuum (over silica gel).

### Mutarotational Equilibrium in Schiff Bases of 2-Amino-2-deoxyaldoses

An imine sample (∼15 mg) was dissolved in DMSO-*d*_6_ (0.5 mL), and its ^1^H NMR spectrum was immediately recorded, followed by temporal monitoring until the equilibrium is reached (as inferred from unaltered ^1^H and ^13^C NMR spectra over time).

### Computational Details

The computational DFT study was initially performed using the B3LYP^33^ and the M06-2X^34^ hybrid density functionals in conjunction with 6-31G(d,p) and 6-311G(d,p) basis sets^32^ as implemented in the Gaussian09 package.^81^ The M06-2X method was chosen on the basis of previous studies showing its accuracy in estimating conformational energies related to noncovalent interactions. In order to assess the influence of the level of theory on anomer stability, the def2-TZVP valence-triple-ζ basis set^37^ was also employed in combination with the M06-2X functional^34^ for geometry optimizations, as the latter has proven to be reliable enough in recent studies addressing structure and binding issues in carbohydrate derivatives.^38,39^ In all cases, frequency calculations were carried out to confirm the existence of true stationary points on the potential energy surface. All thermal corrections were calculated at the standard values of 1 atm at 298.15 K. Solvent effects were modeled through the method of density-based, self-consistent reaction field (SCRF) theory of bulk electrostatics, namely, the solvation model density (SMD) method.^35^ This solvation method includes long-range electrostatic polarization (bulk solvation) as well as short-range effects associated with cavitation, dispersion, and solvent’s structure.

We assessed mutarotational equilibria and solvent effects in 2-iminoaldose derivatives using four approaches: (a) gas-phase: the absence of solvent allows knowing the intrinsic stability of each species. (b) Continuum solvation: anomerization is studied in solution with a description of the solvent as a continuum dielectric medium, using specifically the SMD model.^35^ (c) Microsolvation: calculations are conducted in the gas phase, but one or several water molecules are added to the structures of the stationary points through the mutarotation reaction, in order to determine the stabilization induced by hydrogen bonding. In other words, mutarotation is considered to be assisted by one or more water molecules. (d) Microsolvation and continuum solvation, which represents the hybrid between (b) and (c) methods. Here, the assembly of the imine and one or several water molecules is studied in a continuum and polarizable dielectric medium.

### Natural Bond Orbital (NBO) and Steric Analysis

NBO analysis for optimized structures was performed with NBO versions 3.1^36a^ and 6.0.^36b−36d^ Both versions lead to essentially identical results. Steric interactions were estimated by NBO/NLMO steric analysis with NBO 6.0. Intramolecular interaction of the stabilization energies was performed using second order perturbation theory and listed in the Supporting Information. For each donor NBO(*i*) and acceptor NBO(*j*), the stabilization energy *E*_2_ associated with electron delocalization between donor and acceptor is estimated as
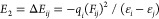
where *q*_*i*_ is the donor orbital occupancy, ε_*i*_ and ε_*j*_ are the diagonal elements (orbital energies), and *F*_*ij*_ is the off-diagonal NBO Fock matrix element. In the natural bond orbital (NBO) approach, a hydrogen bond is viewed as an interaction between an occupied nonbonded natural orbital *n*_A_ of the acceptor atom A and the unoccupied antibonding orbital of the DH bond σ_DH_*.

## Data Availability

The data underlying this study are available in the published article and its Supporting Information.

## Supporting Information Available

Full spectroscopic and analytical characterization for all compounds reported, additional discussion of structural and conformational analyses, crystal data and geometric parameters for compound **44**, FT-IR, and NMR spectra, and Cartesian coordinates and electronic energies for every stationary point optimized at a given level of theory (PDF)

## Dedication

This contribution is dedicated to the memory of María Dolores Méndez-Cordero.
