# Supplementary material for: A True Reverse Anomeric Effect Does Exist After All: A Hydrogen Bonding Stereocontrolling Effect in 2-Iminoaldoses
Source: J Org Chem. 2024 May 16;89(11):7877–98. doi: 10.1021/acs.joc.4c00562 (PMC11165589; doi:10.1021/acs.joc.4c00562)
Supplement: Supplementary file 1 — jo4c00562_si_001.pdf [file jo4c00562_si_001.pdf]

Supporting Information

A True Reverse Anomeric Effect Does Exist After All. A Hydrogen Bonding Stereocontrolling Effect in 2-Iminoaldoses

Esther Matamoros,[a,c,d]* Esther M. S. Pérez,[a] Mark E. Light,[b] Pedro Cintas,[a] R. Fernando Martínez,[a] and Juan C. Palacios[a]*

[a] *Departamento de Química Orgánica e Inorgánica, Facultad de Ciencias, and Instituto del Agua, Cambio Climático y Sostenibilidad (IACYS), Universidad de Extremadura, 06006 Badajoz, Spain*

[b]*Department of Chemistry, Faculty of Natural and Environmental Sciences, University of Southampton, Southampton SO17 1BJ, UK*

[c] *Departamento de Química Orgánica, Universidad de Málaga, Campus Teatinos s/n, 29071 Málaga, Spain.*

[d]*Instituto de Investigación Biomédica de Málaga y Plataforma en Nanomedicina – IBIMA, Plataforma Bionand, Parque Tecnológico de Andalucía, 29590 Málaga, Spain*

E-mail: esthermc@unex.es; palacios@unex.es

CONTENTS

Structural elucidation. Tables S1-S9	S2
Conformational Study of D-Glucosamine Schiff Bases. Tables S10-S15 and Figures S1-S12	S8
Single-crystal X-Ray Diffraction of **60**. Figure S13 and Tables S16-S17	S17
Tables S18-S19	S19
Figures S14-S18	S20
Table S20	S21
Figure S19	S22
Anomerization of Per-*O*-acetylimines	S22
Anomerization of Per-*O*-acetyl-2-(arylmethylene)amino-D-glucopyranosyl bromides	S23
Tables S21-S36	S25
Experimental Section	S46
IR Spectra (Figures S20-S51)	S64
NMR Spectra (Figures S52-S260)	S80
Cartesian Coordinates and Calculated Energies at the B3LYP/6-31G(d,p), M06-2X/6-311G(d,p) and M06-2X/def2-TZVP Level in Gas Phase, $CHCl_3$, DMSO and water (SMD Model)	S158

Structural Elucidation. FT-IR spectra of imines **11-30** present the typical absorptions of the hydroxyl groups between 3600 and 3000 cm^{-1} along with bands of C-O bonds between 1150 cm^{-1} and 1000 cm^{-1}. The most significant signal appears at ~1640 cm^{-1}, revealing the presence of the C=N imine bond. Signals from the aromatic moiety at ~1600 cm^{-1}, ~1580 cm^{-1} and ~1500 cm^{-1} are intense when there are electron donor groups (eg, **12**); otherwise they may be very weak (eg, **29**). NMR spectra of all imines are similar (Tables S1 and S2). In the ^{1}H NMR spectra, the most shifted downfield signal always corresponds to hydrogen of the imino group (~8 ppm); except in the cases of **15**, **16** and **18**, which show the signal of the phenolic hydroxyl at more shifted downfield resonances. The anomeric hydroxyl signal is usually more deshielded than other aliphatic hydroxyls (~6.5 ppm). The rest of such hydroxyls lie between 4.5 and 5 ppm. The anomeric proton appears as a triplet at ~4.7 ppm, whereas the H-2 signal stands out as the most shielded at ~2.8 ppm.

In the ^{13}C NMR spectra the signals of the iminic carbon appear at ~161 ppm with the most deshielded carbon of the pyranose ring being the anomeric center, bonded to two oxygen atoms (δ ~95 ppm). In contrast, the terminal hydroxymethylene carbon is the more shielded signal (δ ~62 ppm). The unambiguous assignment of all carbon resonances was carried out through HMQC, HMBC, and DEPT experiments (Table S3). The carbon carrying the imine group (C-2) appears at an unusual downfield (~78 ppm), which deviates from other 2-amino-2-deoxyaldose derivatives.[28] For example, D-glucosamine itself in D_2O shows values of 55 ppm and 58 ppm for the C-2 of the α and β anomers, respectively.[59] Likewise, 1,3,4,6-tetra-*O*-acetyl-2-acetamido-2-deoxy-α-D-glucopyranose[28a] shows the signal of C-2 at 51 ppm, and the C-2 signals of **3**.

The β-anomeric configuration assigned to the imines in solution is based on the following evidences. The coupling constant $J_{1,2}$ has always a high value (~7.5 Hz), consistent with an *anti*-periplanar arrangement between H-1 and H-2, and usual for the anomeric hydroxyl in equatorial position. Moreover, the coupling constants between the proton and the anomeric carbon (^{1}J) measured in the coupled ^{13}C NMR spectra have a value of ~160 Hz. Literature data[28-30] indicate that this constant in carbohydrate pyranoses can be used with confidence for the assignment of the anomeric configuration, because the pyranose ring with an axial anomeric proton (β-anomer) consistently shows a value ~10 Hz less than the value measured in compounds with hydrogen in equatorial position (α-anomer). This difference can be attributed to the Perlin effect,[16] generated by the axial lone pair of endocyclic oxygen on the axial β-anomeric proton. In D-glucosamine derivatives the β-anomers show values of $^{1}J_{C1\text{-}H1}$ ~160 Hz whereas this coupling is ~170 Hz for the corresponding α-anomers. Accordingly, the values shown by imines **9**, **11-28**, and **30**, do reflect their β-configuration. These data also contradict previous literature representing imines **11**, **12**, **15**, **18**, and **19** as α-anomers,[11b] when they are in fact β-anomers.

Table S1. *^{1}H NMR data (δ, ppm) of **11-28**.[a]*

Comp	ArCHN	C1OH	C6OH	H-1	H-2	H-3	H-4	H-5	H-6	H-6´
**11**	8.20 s	6.57 d	4.57 t	4.73 t	2.85 dd	3.45dt	3.16 td	3.25 m	3.74 ddd	3.50 m
**12**	8.11 s	6.52 d	4.54 t	4.74 t	2.78 dd	3.41dt	3.15 m	3.22 m	3.72 ddd	3.47 m
**13**	8.17 s	6.58 d	--	4.73 d	2.84 t	3.53 m	3.16 t	3.26 m	3.73 d	3.53 m
**14**	8.54 s	6.65 d	4.64 m	4.72 d	2.84 t	3.45 m	3.16 m	3.25 m	3.73 d	3.45 m
**15**	8.05 s	--	--	4.68 d	2.79 t	3.42 t	3.15 t	3.23 m	3.73 d	3.49 dd
**16**	8.10 s	6.55 d	4.56 t	4.70 t	2.82 t	3.44 m	3.15 td	3.25 m	3.73 dd	3.48 m
**17**	8.37 s	6.68d	4.60 t	4.79 t	2.94 t	3.51 m	3.20 td	3.28 m	3.75 ddd	3.51 m
**18**	8.01 s	6.50 d	4.58 m	4.66 t	2.74 t	3.38 m	3.13 t	3.21 m	3.65 d	3.47 dd
**19**	8.04 s	6.52 d	--	4.70 t	2.78 t	3.46 m	3.16 m	3.24 m	3.74 dd	3.46 m
**20**	8.16 s	6.57 d	--	4.72 t	2.83 dd	3.47 m	3.17 m	3.24 m	3.74 d	3.49 dd
**21**[b]	8.16 s	6.54 d	4.55 t	4.72 t	2.83 dd	3.44 dt	3.16 td	3.25 ddd	3.74 dd	3.50 m
**22**[b]	8.46 s	6.52 d	4.54 t	4.73 t	2.86 dd	3.45 dt	3.16 td	3.25 ddd	3.74 ddd	3.50 m
**23**[b]	8.17 s	6.53 d	4.55 t	4.73 t	2.83 t	3.45 dt	3.17 t	3.24 ddd	3.74 ddd	3.51 m
**24**[b]	8.26 s	6.58 d	4.56 t	4.76 t	2.88 t	3.47 m	3.19 td	3.28 ddd	3.75 ddd	3.52 m
**25**[b]	8.41 s	6.45 d	4.52 t	4.67 t	2.75 t	3.39 m	3.13 td	3.22 ddd	3.72 dd	3.48 m
**26**[b]	8.40 s	6.50 d	4.53 t	4.71 t	2.83 dd	3.43 dt	3.15 td	3.24 ddd	3.73 ddd	3.49 m
**27**	8.40 s	6.57 d	4.56 t	4.71 t	2.82 t	3.43 m	3.17 m	3.24 m	3.74 dd	3.49 dd
**28**	8.52 s	6.15 d	4.45 t	4.96 t	3.08 dd	3.77 m	3.19 m	3.53 m	3.77 m	3.67 m

[a] In DMSO-d_6 at 400 MHz; [b] In DMSO-d_6 at 500 MHz.

Table S2. *Coupling constants (Hz) of **11-28**.[a]*

Comp	$J_{1,2}$	$J_{2,3}$	$J_{3,4}$	$J_{4,5}$	$J_{5,6}$	$J_{5,6'}$	$J_{6,6'}$	$J_{H1,OH}$
**11**	7.2	9.0	9.0	8.8	1.9	6.0	11.6	6.9
**12**	7.3	8.9	8.9	--	1.8	5.8	11.6	6.7
**13**	7.5	8.5	9.1	9.1	--	--	10.7	--
**14**	7.3	8.3	8.9	8.9	--	--	11.3	--
**15**	7.2	8.8	8.8	9.4	---	6.2	11.4	--
**16**	7.2	8.5	8.9	8.9	--	--	11.4	6.8
**17**	7.2	8.5	8.7	8.7	1.5	--	11.3	6.7
**18**	7.6	8.5	9.1	9.1	--	5.8	11.6	--
**19**	7.3	8.4	9.1	9.1	--	--	11.4	--
**20**	7.2	9.0	8.8	8.8	--	5.0	11.1	6.4
**21**[b]	7.5	9.5	9.0	9.5	2.0	5.5	11.5	7.0
**22**[b]	7.5	9.0	9.0	10.0	2.0	6.0	10.0	7.0
**23**[b]	7.0	8.5	8.5	8.5	2.0	6.0	11.0	6.5
**24**[b]	7.0	9.0	9.0	9.5	2.0	5.5	11.5	6.0
**25**[b]	7.5	8.0	9.0	9.5	2.0	7.0	11.5	7.0
**26**[b]	7.0	9.0	10.0	9.0	1.5	5.5	11.5	7.0
**27**	7.3	8.4	9.2	9.2	--	--	11.0	7.0
**28**	2.9	9.2	9.2	3.7	5.3	5.6	9.9	3.6

[a] In DMSO-d_6 at 400 MHz; [b] In DMSO-d_6 at 500 MHz.

Table S3. ^{13}C NMR data (δ, ppm) of **11-28**.[a]

Comp	ArCHN	C-1	C-2	C-3	C-4	C-5	C-6
**11**	162.23	95.74	78.45	74.66	70.54	77.09	61.47
**12**	161.52	95.85	78.42	74.81	70.58	77.09	61.49
**13**	162.26	95.74	78.50	74.66	70.58	77.12	61.51
**14**	157.53	96.04	79.12	74.93	70.73	77.27	61.66
**15**	161.80	95.96	78.46	74.94	70.63	77.09	61.57
**16**	162.27	95.80	78.36	74.71	70.55	77.11	61.48
**17**	160.77	95.58	78.58	74.46	70.39	77.19	61.43
**18**	161.89	96.02	78.49	75.03	70.66	77.03	61.54
**19**	162.12	95.90	78.47	74.89	70.69	77.07	61.57
**20**	162.13	95.87	78.49	74.79	70.60	77.12	61.54
**21**[b]	162.12	95.63	78.30	74.56	70.44	76.93	61.36
**22**[b]	160.55	95.56	78.54	74.43	70.28	76.87	61.23
**23**[b]	161.68	95.49	78.13	74.43	70.28	76.76	61.19
**24**[b]	161.29	95.32	78.06	74.25	70.09	76.62	61.86
**25**[b]	156.26	95.47	78.39	74.42	70.16	76.57	61.05
**26**[b]	160.35	95.57	78.48	74.46	70.31	76.79	61.21
**27**	161.96	95.88	79.44	74.62	70.63	77.17	61.50
**28**	162.01	93.44	76.43	71.16	70.91	72.69	61.51

[a] In DMSO-d_6 at 100 MHz; [b] In DMSO-d_6 at 125 MHz.

The IR spectra of the anomeric mixture **32**/**33** and **34** are similar to those of **11**-**30**, showing absorptions between 3000 and 2500 cm^{-1}, which correspond to the aliphatic and phenolic hydroxyl groups, and the typical C=N streching vibration at ~1640 cm^{-1}. In contrast, the IR spectrum of the Schiff base **39**/**40** exhibits an intense absorption at 1643 cm^{-1} and an even more intense absorption at 1590 cm^{-1}, indicative of an enamine structure.[4] Also, ^{1}H and ^{13}C NMR spectra of imines **32**/**33** and **34** are similar to those of imines **11**-**30** (Tables S4-S6). The salicylaldehyde imine just dissolved in DMSO-d_6 shows the signals of both α- and β-anomers, (**32**/**33**), whereas the imine from 2,4-dihydroxybenzaldehyde only those indicative of the α-anomer (**34**). The α-configuration of **32** is supported by the low value of $J_{1,2}$ (2.6 Hz), the chemical shifts of H-1 ($\delta_{H\text{-}1}$ ~5.0 ppm) and C-1 ($\delta_{C\text{-}1}$ ~92 ppm), and the value for $J_{C1,H}$ (~167). On the contrary, the β-anomer **35** has a high value of $J_{1,2}$ (8.1 Hz) and signals of H-1 and C-1 ($\delta_{H\text{-}1}$ ~4.7 ppm and $\delta_{C\text{-}1}$ ~95.7 ppm), along with a $J_{C1,H}$ value (~160 Hz) in agreement with those shown by β imines **11**-**28**. In addition, the imine group is supported by the chemical shifts of the iminic proton ($\delta_{CH=N}$ ~8.2 ppm) and carbon ($\delta_{C=N}$ ~165 ppm). The protons of the *ortho*-phenolic hydroxyls show a high chemical shift (> 13 ppm), which reflects the existence of a strong intramolecular hydrogen bond with the imine nitrogen; while the resonance for the *para*-substituted hydroxyl appears above 10 ppm.

The ^{1}H NMR spectrum of the Schiff base from 2,4,6-trihydroxybenzaldehyde unequivocally shows the existence of two compounds with an enamine structure (**39** and **40**). Their formation is evidenced by the two double doublets at 11.49 ppm and 11.41 ppm, corresponding to the NH enamine groups of both α-anomers, two doublets at 8.23 ppm and 8.20 ppm arising from the =CH protons, and other two doublets at 5.17 ppm and at 5.15 ppm, assignable to the anomeric protons. The low coupling constants $J_{1,2}$ (3.0 and 3.5 Hz) support the configuration of α-anomers (Table S5). The ratio between both anomers is ~3:2. The enamine structure of **39** and **40** is

evidenced not only by the multiplicity of the signal of the NH groups, but also by the chemical shifts of two protons in the aromatic ring: at 6.18 ppm and 5.53 ppm for the first and 6.10 ppm and 5.49 ppm for the second. Such signals are significantly shielded for an aromatic ring. Nevertheless, they can be interpreted by the hexadienic structure adopted by the carbocyclic fragment in these enamines. The presence of the corresponding β-anomers is also evidenced by the peak at ~4.70 ppm (**41** and **42**). The β-configuration assigned to these minor compounds is supported by the high values of $J_{1,2}$ > 7.7 Hz. The ratio between both isomers is ~1:1.

The ^{1}H and ^{13}C NMR spectra of **34** (as monohydrate), just dissolved in DMSO-d_6, agree with those found for the α-anomer, thus pointing to the role of water molecule as water of crystallization within the crystal lattice. The chemical shifts measured for the proton and imine carbon demonstrate that this double bond remains intact. Likewise, the hydrochloride of that hydrate is actually a saline derivative, thus accounting for the basic character of the imine nitrogen. Its ^{1}H and ^{13}C NMR spectra show an equilibrium between the α- (**36**) ($J_{1,2}$ 2.1 Hz) and β- (**37**) ($J_{1,2}$ 8.0 Hz) anomers, in which the α-anomer is largely predominant (84.8%). The most interesting point to be highlighted is the appearance of the imine proton (CH=N) as a doublet at ~8.7 ppm. This splitting results from the coupling with the proton on the nitrogen ($J_{H\text{-}2,NH}$ ~15 Hz). As a consequence of this protonation, a strong deshielding of H-2 (Δδ ≥ 0.5 ppm) and, to a lesser extent for the CH=N, H-1 and H-3 signals (Δδ ~ 0.2 ppm) is observed. However, the values of coupling constants indicate that no conformational changes in the pyranose ring took place. Therefore, the structures proposed in the late 1930s for the hydrate of **34** and hydrochlorides **36/37** hydrochlorides,[22] assuming that the water molecule had been added to the imine bond, are incorrect.

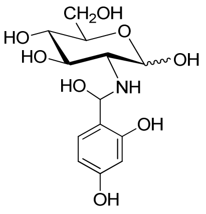

34

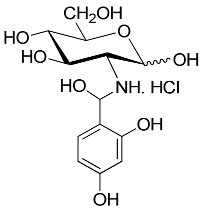

36/37

Table S4. *^{1}H NMR data (δ, ppm) of **32-37**, **39**, and **40**.*[a]

Comp	CH=N	H-1	H-2	H-3	H-4	H-5	H-6	H-6´
**32**	8.48 s	5.04 d	3.17 t	3.43 dt	3.49 m	3.49 m	3.67 dd	3.49 m
**33**	8.18 s	5.06 d	3.18 ddd	3.70 t	3.16 c	3.56 m	3.56 m	3.56 m
**34**	8.41 s	4.71 t	2.86 dd	3.43 td	3.17 t	3.28 m	3.71 dd	3.48 dd
**35**	8.22 s	4.65 d	2.80 t	3.40 t	3.52 m	3.24 m	3.63 m	3.52 m
**36**	8.69 d	5.26 d	3.68 m	3.65 m	3.24 t	--	3.54 d	3.65 m
**37**	8.60 d	4.97 d	3.64 m	3.64 m	--	3.36 t	3.48 dd	3.64 m
**39**[b]	8.23 d	5.17 d	3.39 m	3.47 m	3.66 m	3.17 t	3.66 dd	3.53 m
**40**[b]	8.20 d	5.15 d	3.39 m	3.47 m	3.66 m	3.16 t	3.65 dd	3.53 m

[a] In DMSO-d_6 at 400 MHz; [b] At 500 MHz.

Table S5. *Coupling constants (Hz) of **32-37**, **39** and **40**.*[a]

Comp	$J_{1,2}$	$J_{2,3}$	$J_{3,4}$	$J_{4,5}$	$J_{5,6}$	$J_{5,6'}$	$J_{6,6'}$
**32**	3.4	9.8	9.2	--	--	--	--
**33**	2.6	11.3	9.2	9.2	--	--	--
**34**	8.1	9.1	9.7	9.7	--	2.0	11.9
**35**	7.7	8.8	9.0	--	--	--	10.2
**36**	2.1	--	9.0	9.0	5.2	--	11.9
**37**	8.0	--	--	--	5.1	--	11.7
**39**[b]	3.0	--	--	9.0	--	--	10.0
**40**[b]	3.5	--	--	9.5	--	--	9.5

[a] in DMSO-d_6 at 400 MHz; [b] At 500 MHz.

Table S6. *13C NMR data (δ, ppm) of **32-37**, **39** and **40**.*[a]

Comp	ArCHN	C-1	C-2	C-3	C-4	C-5	C-6
	166.90	92.36	72.60	70.88	70.46	71.27	61.44
**33**	164.42	91.85	72.68	71.52	70.90	71.52	61.48
**34**	167.10	95.57	77.16	74.93	70.46	76.75	61.44
**35**	166.27	95.70	77.14	75.11	70.60	75.71	61.48
**36**	166.28	89.88	72.73	70.18	65.95	70.18	60.81
**37**	166.28	93.33	77.00	72.57	70.55	71.13	60.82
**39**[b]	159.54	90.77	72.92	71.96	64.89	70.72	61.38
**40**[b]	159.38	90.81	72.89	71.91	64.89	70.75	61.34

[a] In DMSO-d_6 at 100 MHz; [b] At 125 MHz.

The structures assigned to such *O*-acetylated derivatives **43-47** are supported by their polarimetric and spectroscopic data. The absence of IR-signals above 3100 cm^{-1} confirms that all hydroxyl groups have been acetylated. Other key absorptions correspond to the stretching vibration of the carbonyl group (~1750 cm^{-1}), the C-O-C acetate group (~1240 cm^{-1}), and the streching vibration of the C=N bond (~1650 cm^{-1}). In the ^{1}H NMR spectra the proton of the imine group, CH=N, appears at ~8.20 ppm as a singlet while the H-2 proton, the most shielded signal of the pyranosic ring resonates at ~3.50 ppm. The assigned β-anomeric configuration is in agreement with the large value of $J_{1.2}$ (~8.3 Hz). Like in unprotected Schiff bases, assignments based on ^{13}C NMR data were corroborated by DEPT and HMQC experiments. As indicated above,[28,29] the β-anomers of unprotected pyranoses exhibit a value of $^{1}J_{C1,H}$ close to 160 Hz, while the coupling constant is ~170 Hz for α-anomers. In the acetylated derivatives, however, these values are ~177 Hz and ~166 Hz for α- and β-anomers, respectively; i.e., the difference between the two species remains constant within ~10 Hz. Accordingly, the β-anomeric configuration of per-*O*-acetylimines was confirmed by the magnitued of the coupling constants $J_{C1\text{-}H}$ (~167 Hz), as measured in the coupled spectra, similar to those shown by other acetylated derivatives of **1** and **3** with identical anomeric configuration.

The IR spectra of **50-53** show a broad absorption centered at ~3500 cm^{-1}, resulting from the stretching vibration of the anomeric and phenolic hydroxyl groups, together with the typical absorption of the group C=N at ~1630 cm^{-1}. In the ^{1}H NMR spectra ($CDCl_3$) the signals of the imine proton (CH=N) at 8.33 ppm and H-4 at 5.11 ppm are coincidental in chemical shift for both

anomers, and those of H-6 and H-6' are very close. With respect to other resonances, the proton signals of **52** are more deshielded than those of **53** with significant shift differences. Thus, the most separated peaks correspond to H-5 (Δδ ~0.5 ppm), followed by H-1 (Δδ ~0.3 ppm) and by H-2 and H-3 (Δδ ~0.25 ppm). The constants $J_{2,3}$, $J_{3,4}$ and $J_{4,5}$, are identical for both anomers and their high value is in agreement with a D-*gluco* configuration in conformation $^4C_1$. The α-anomer **52** is supported by the low value of $J_{1.2}$ (3.4 Hz), while the β-anomer (**53**) exhibits a higher constant (8.1 Hz). ^{13}C NMR data evidence the formation of the most abundant α-anomer having a chemical shift at ~92 ppm, whereas the β-anomer signal appears at ~96 ppm.

Table S7. *^{1}H NMR data (δ, ppm) of **29**, **54-57**, **59**, and **62-71**.*

Comp.	CH=N	C1-OH	C6-OH	H-1	H-2
**29**[a]	8.40 s	6.57 d	4.56 t	4.71 t	2.82 dd
**54**[a]	8.33 s	6.25 d	4.49 t	4.95 m	3.15 m
**55**[a]	8.23 s	6.22 d	4.49 t	4.97 t	3.07 dd
**56**[a]	8.30 s	6.27 d	4.50 sa	4.96 m	3.13 dd
**57**[a]	8.64 s	6.20 d	4.47 t	4.92 m	3.10 dd
**59**[a]	8.22 s	6.22 d	4.45 t	4.92 m	3.10 dd
**62**[a]	8.47 s	6.38 d	4.54 sa	4.86 s.a	3.22 dd
**63**[a]	8.13 s	6.12 d	4.44 t	4.86 m	3.03 m
**64**[a]	8.28 s	6.27 sa	4.20 sa	--	3.04 dd
**65**[a]	8.28 s	6.21 d	4.47 t	4.93 m	3.11 dd
**66**[b]	8.27 s	6.21 d	4.49 t	4.72 t	3.12dd
**67**[b]	8.58 s	6.17 d	4.43 t	4.95 t	3.21 m
**68**[b]	8.27 s	6.19 d	4.47 t	4.92 t	3.10 dd
**69**[b]	8.35 s	6.31 d	4.63 t	4.97 m	3.07 m
**70**[b]	8.51 s	6.15 d	4.51 t	4.89 t	3.06 dd
**71**[b]	8.52 s	6.16 d	4.47 t	4.92 t	3.11 d

[a] In DMSO-d_6 at 400 MHz; [b] In DMSO-d_6 at 500 MHz.

Table S8. *Coupling constants (Hz) of **29**, **54-57**, **59**, and **62-71**.*

Comp.	$J_{H1,OH}$	$J_{H6,OH}$	$J_{1,2}$	$J_{2,3}$
**29**[a]	6.7	5.5	7.3	8.4
**54**[a]	4.4	5.6	--	9.4
**55**[a]	3.6	--	3.6	9.6
**56**[a]	4.2	5.0	3.2	9.8
**57**[a]	4.4	5.6	3.6	--
**59**[a]	4.4	5.8	3.3	9.9
**62**[a]	3.7	--	3.1	9.0
**63**[a]	4.4	5.6	--	--
**64**[a]	4.0	--	3.6	10.0
**65**[a]	4.0	5.0	3.0	10.0
**66**[b]	4.0	6.0	3.5	--
**67**[b]	4.0	6.0	3.5	9.5
**68**[b]	2.0	5.5	--	--
**69**[b]	3.5	5.0	3.5	10.0
**70**[b]	4.0	5.5	4.0	--

[a] In DMSO-d_6 at 400 MHz; [b] In DMSO-d_6 at 500 MHz.

Table S9. ^{13}C *NMR data (δ, ppm) of* ***29****,* ***54-57****,* ***59****, and* ***62-71****.*

Comp.	CH=N	C-1	C-2	C-3	C-4	C-5	C-6
**29**[a]	161.52	95.85	78.42	74.81	70.58	77.09	61.49
**54**[a]	162.59	93.26	75.43	71.12	71.12	72.76	61.60
**55**[a]	161.68	93.90	75.35	71.14	70.61	72.71	61.54
**56**[a]	162.44	93.20	75.37	71.18	71.06	72.76	61.57
**57**[a]	157.53	93.41	76.03	71.33	71.27	72.91	61.72
**59**[a]	162.50	93.23	75.34	71.06	71.18	72.70	61.54
**62**[a]	161.10	93.00	75.44	71.05	70.97	72.77	61.52
**63**[a]	162.26	93.27	75.44	71.22	71.10	72.77	61.58
**64**[a]	164.22	93.33	75.38	71.22	71.14	72.66	61.54
**65**[a]	161.96	93.51	75.53	71.34	71.34	72.74	61.64
**66**[b]	162.36	93.01	75.03	70.94	70.82	72.40	61.37
**67**[b]	160.66	92.95	75.31	70.86	70.71	72.35	61.31
**68**[b]	161.01	92.97	74.98	70.91	70.79	72.36	61.34
**69**[b]	162.53	93.26	79.24	72.58	71.12	75.19	61.44
**70**[b]	156.97	93.20	79.15	72.42	71.02	75.45	61.41
**71**[b]	162.13	93.09	75.34	70.95	70.85	72.39	61.38

[a] In DMSO-d_6 at 100 MHz; [b] In DMSO-d_6 at 125 MHz.

Conformational study of D-Glucosamine Schiff bases. The rotational profile of **11b** is shown in Figure S1 with calculation spotting two maxima and two minima (Figure S4), whose relative energies are shown in Table S10. However, the curve shows a point of inflection that allows us to guess another maximum at $\theta_{H2\text{-}C2\text{-}N\text{-}CH}$ ~150º and a minimum at $\theta_{H2\text{-}C2\text{-}N\text{-}CH}$ ~130º, both with an energy close to 7 kcal/mol. The conformation corresponding to the most stable minimum shows a value of the dihedral angle $\theta_{H2\text{-}C2\text{-}N\text{-}CH}$ of ~10°; i.e., the same conformation as that inferred from NOE effects.

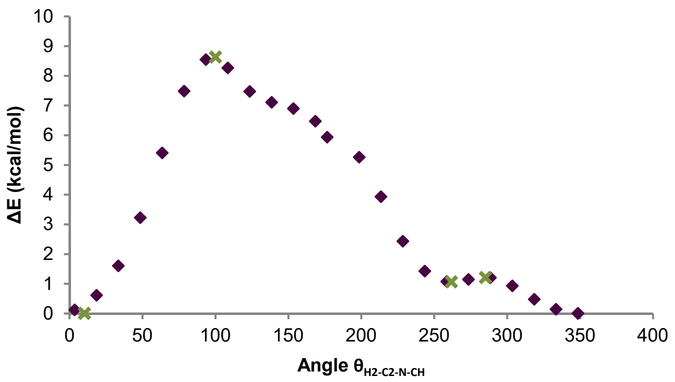

Figure S1. *Conformational profile of* ***11b*** *[B3LYP/6-31G(d,p)].*

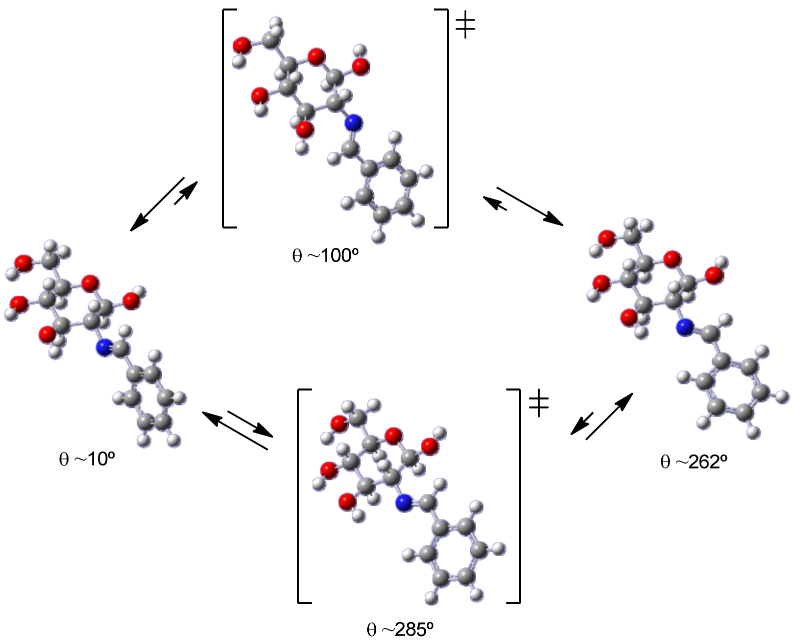

Figure S2 *Optimized structures of the stationary points for the conformational analysis of* ***11b*** *[B3LYP/6-31G(d,p)].*

Table S10. *Relative energies (kcal/mol) of minima and maxima found for* ***11b***.[a]

	$\theta_{H2-C2-N-CH}$	ΔE	ΔG	$\tilde{\nu}^{‡b}$
min 1	10.1º	0.00	0.00	0
min 2	261.6º	1.06	1.48	0
max 1	99.9º	8.63	9.10	-65.3
max 2	285.0º	1.21	2.75	-29.4

[a] B3LYP/6-31G(d,p); [b] Imaginary frequency in cm^{-1}.

The rotational profile of the α-anomer is similar (Figure S3) and shows three minima and two maxima, although again there is an inflection point at $\theta_{H2-C2-N-CH}$ ~160º (Figure S4).

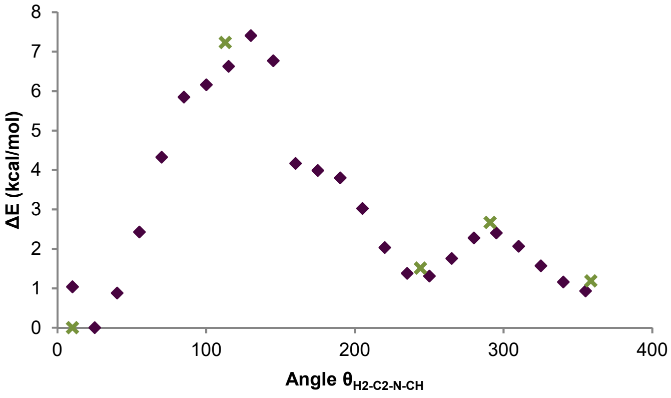

Figure S3. *Conformational profile of* ***54b*** *[B3LYP/6-31G(d,p)].*

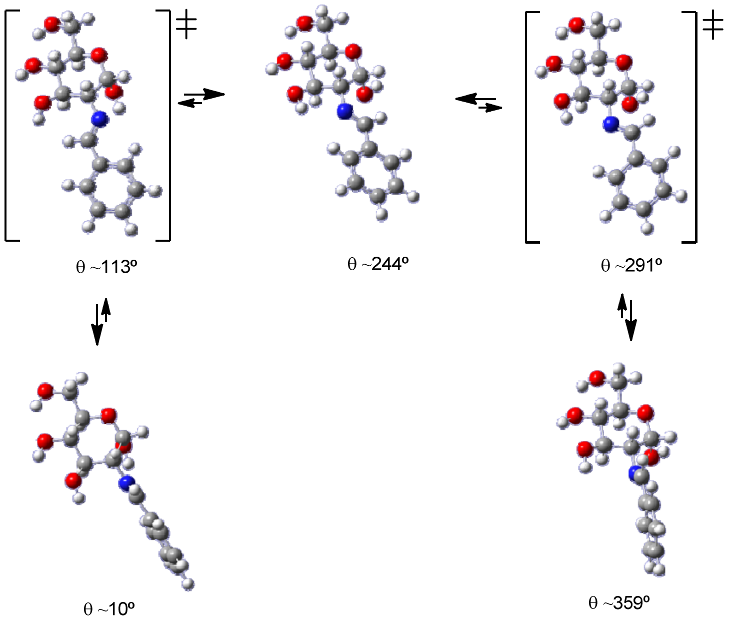

Figure S4. *Optimized structures of the stationary points for the conformational analysis of **54b** [B3LYP/6-311G(d,p)].*

The relative energies of the stationary points are listed in Table S11, at the B3LYP/6-31G(d,p). The two most stable minima show practically identical values of $\theta_{H2-C2-N-CH}$ at 10.1° and -1.3° (= 358.7°), differing only by the orientation of the anomeric hydroxyl. After the 360° turn, it should have returned to the starting point; however a re-orientation of the anomeric hydroxyl was actually found. The small energy difference between the two orientations (0.77 kcal/mol) can be caused by the formation of a weak intramolecular hydrogen bond between the anomeric OH and the lone pair on the nitrogen atom.

Table S11. *Relative energies (kcal/mol) of the maxima and minima of **54b**.*[a]

	$\theta_{H2-C2-N-CH}$	ΔE	ΔG	$\tilde{\nu}^{‡b}$
min 1	10.1°	0.00	0.00	0
min 2	244.1°	1.51	1.74	0
min 3	358.7°	1.19	0.77	0
max 1	112.9°	7.23	7.28	-94.6
max 2	290.9°	2.67	3.55	-40.9

[a] B3LYP/6-31G(d,p); [b] Imaginary frequency in cm^{-1}.

Calculations at the M06-2X/6-311G(d,p) level afford similar results, thus indicating that the computational method has little influence on the result. For the β-anomer the calculated data are collected in Figure S5 and Table S12.

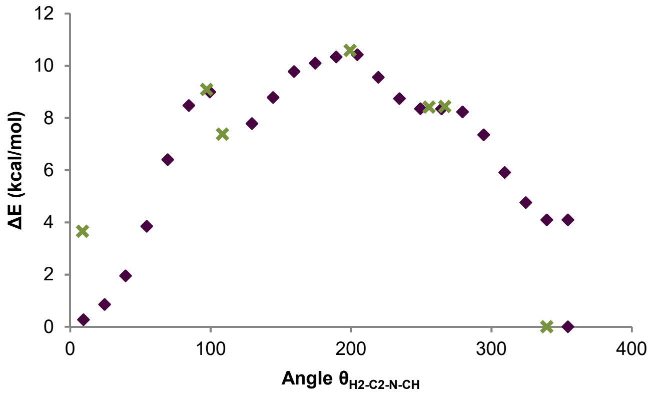

Figure S5. *Conformational profile of **11b** [M06-2X/6-311G(d,p) in gas phase].*

However, three maxima and four minima are now located (Figure S6). Again, the minima at values of $\theta_{H2\text{-}C2\text{-}N\text{-}CH}$ of ~ 9º and ~ -21º (= 339º) should be identical, but rotation around the enaminic group alters the position of the hydroxyls at C-3 and C-4, thus generating an energy difference of ~4 kcal/mol.

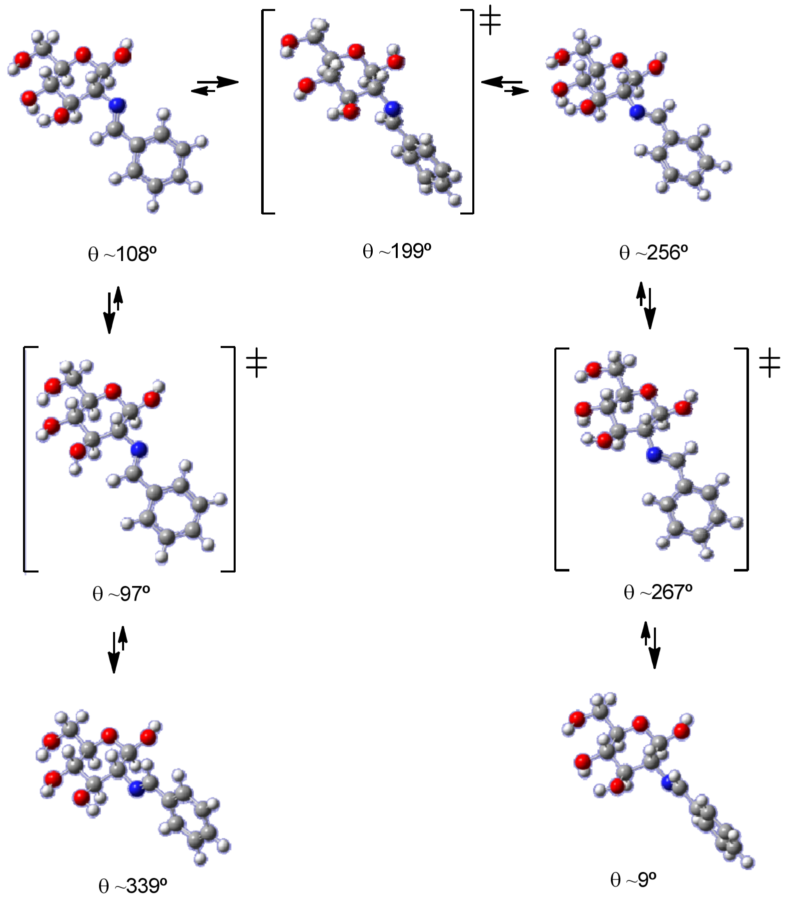

Figure S6. *Optimized structures of the stationary points for the conformational analysis of **11b** [M06-2X/6-311G(d,p) in gas phase].*

The maximum obtained for the conformational arrangement occurs with $\theta_{H2\text{-}C2\text{-}N\text{-}CH}$ at ~199° and results from a significant steric interaction between the iminic hydrogen and axial hydrogens at C-1 and C-3 (Figure S6).

Table S12. *Relative energies (kcal/mol) of the maxima and minima for* ***11b****.*[a]

	$\theta_{H2\text{-}C2\text{-}N\text{-}CH}$	ΔE	ΔG	$\tilde{v}^{\ddagger b}$
min 1	339.5°	0.00	0.00	0
min 2	108.5°	7.38	7.27	0
min 3	255.7°	8.42	8.36	0
min 4	8.8°	3.66	3.99	0
max 1	97.2°	9.09	9.62	-68.1
max 2	199.4°	10.59	11.67	-51.0
max 3	266.8°	8.44	9.64	-24.2

[a] M06-2X/6-311G(d,p); [b] Imaginary frequency in cm^{-1}.

The conformational profile for the α-anomer is almost equal to that obtained through the B3LYP/6-31G(d,p) method. Results are shown in Figures S7 and S8, and Table S13.

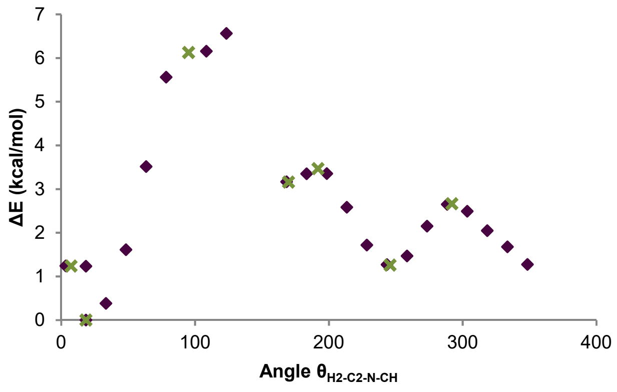

Figure S7. *Conformational profile of* ***54b*** *[M06-2X/6-311G(d,p) in gas phase].*

Like the β-anomer, four minima and three maxima are located. The minima at $\theta_{H2\text{-}C2\text{-}N\text{-}CH}$ ~7° and at ~18° differ only by the orientation of the anomeric hydroxyl. The maxima at $\theta_{H2\text{-}C2\text{-}N\text{-}CH}$ ~192° and 292° have values much lower than those presented by the β-anomer.

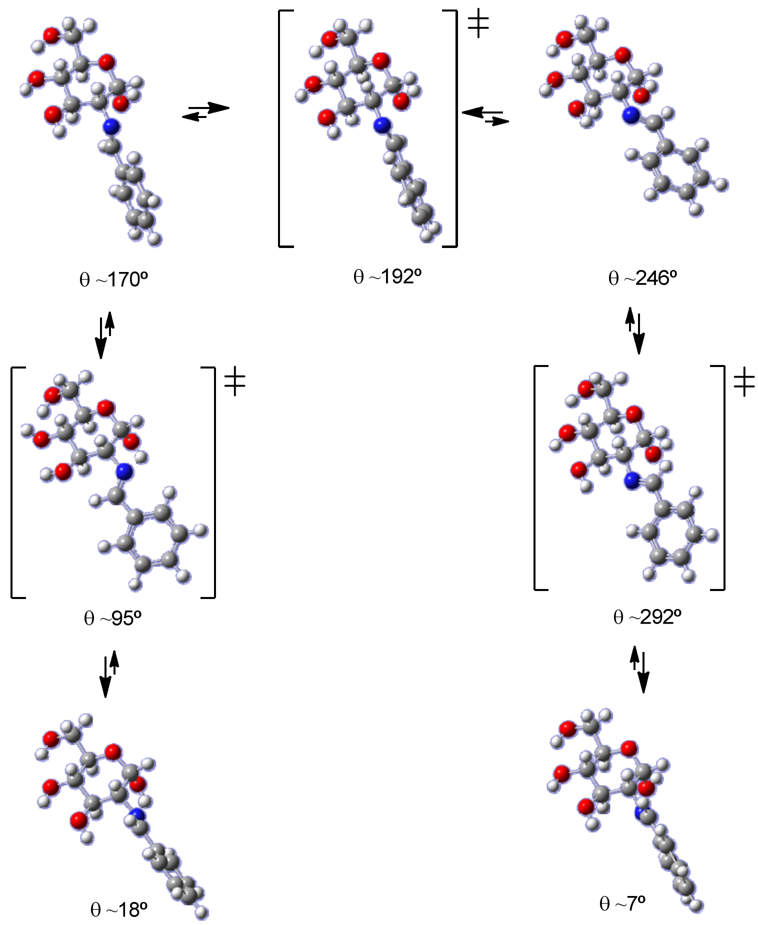

Figure S8. *Optimized structures of the stationary points for the conformational analysis of* ***54b*** *[M06-2X/6-311G(d,p) in gas phase].*

Table S13. *Relative energies (kcal/mol) of the maxima and minima for* ***82b****.*[a]

	$\theta_{H2\text{-}C2\text{-}N\text{-}CH}$	ΔE	ΔG	$\tilde{\nu}^{\ddagger b}$
min 1	18.4º	0.00	0.00	0
min 2	169.9º	3.16	3.05	0
min 3	245.8º	1.26	1.14	0
min 4	7.3º	1.23	0.69	0
max 1	95.2º	4.91	6.67	-27.9
max 2	191.8º	2.25	3.96	-53.6
max 3	291.9º	1.44	3.16	-39.9

[a] M06-2X/6-311G(d,p); [b] Imaginary frequency in cm^{-1}.

To check whether the initial disposition of the hydroxyl group influences the outcome of the conformational analysis, we carried out a similar study from **11c** in which the hydroxyl group adopts the opposite arrangement (see Figure S9). Both methods of calculation lead to practically identical profiles for each anomer, not only in shape but also in the values of the corresponding

maxima and minima. Accordingly, graphs and structures have been determined using the M06-2X/6-311G(d,p) method, although the energy values are tabulated at the two levels.

Results for the β-anomer **11** are summarized in Figure S9 and Table S14. The M06-2X/6-311G(d,p) calculation leads to three maxima and three minima, whose structures are shown in Figure S10. The symmetrical environment around the hydroxyl group translates into a quite symmetrical profile.

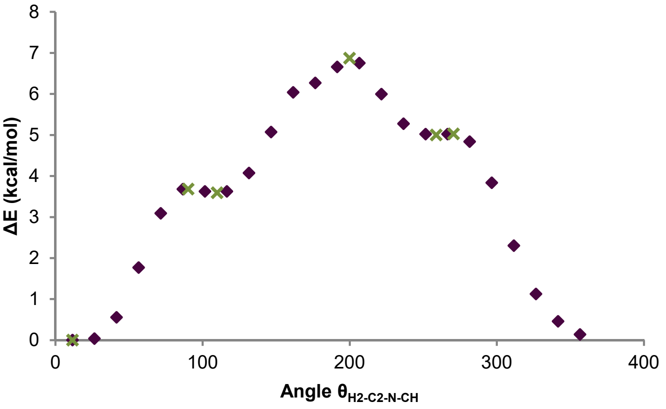

***Figure S9**. Conformational profile of **11c** [M06-2X/6-311G(d,p) in gas phase].*

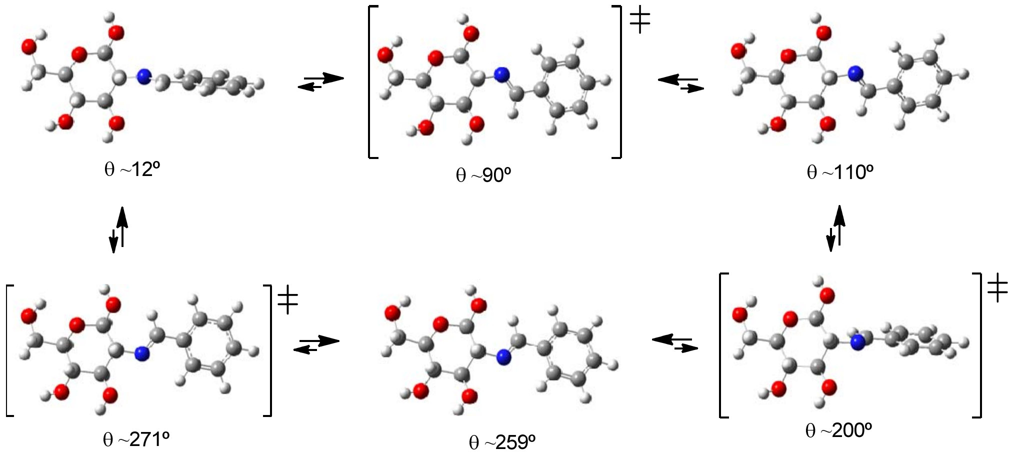

***Figure S10.** Optimized structures of the stationary points for the conformational analysis of **11c** [M06-2X/6-311G(d,p) in gas phase].*

When the calculation is done using the B3LYP/6-31G(d,p) method, only one maximum and one minimum are obtained (at values of $\theta_{H2\text{-}C2\text{-}N\text{-}CH}$ ~202º and ~7º, respectively). The other two become inflection points in the graph; nevertheless, the values of the angle $\theta_{H2\text{-}C2\text{-}N\text{-}CH}$ and the electronic energy, estimated directly from the conformational graph have also been included in Table S14. As mentioned, such data along with the structures of maxima and minima are essentially coincidental with those determined by the M06-2X/6-311G(d,p) method.

Table S14. *Relative energies (kcal/mol) of the maxima and minima found for* ***11c****.*

	$\theta_{H2\text{-}C2\text{-}N\text{-}CH}$ [a]	ΔE[a]	ΔG[a]	$\tilde{\nu}$‡[d]	$\theta_{H2\text{-}C2\text{-}N\text{-}CH}$ [b]	ΔE[b]	ΔG[b]	$\tilde{\nu}$‡[d]
min 1	6.95º	0.00	0.00	0	11.5º	0.00	0.00	0
min 2	~112º[c]	~3.9[c]			109.8º	3.59	3.91	0
min 3	~247º[c]	~5.2[c]			258.7º	5.00	5.04	0
max 1	~97º[c]	~3.8[c]			90.2º	3.68	4.92	-33.4
max 2	202.1º	6.76	7.83	-38.5	199.9º	6.87	7.82	-50.4
max 3	~262º[c]	~5.0[c]			270.5º	5.03	6.08	-28.5

[a] B3LYP/6-31G(d,p); [b] M06-2X/6-311G(d,p); [c] Estimated values; [d] Imaginary frequency in cm^{-1}.

Finally, figures S11 and S12 and Table S15 show the results found for the α-anomer **54c**.

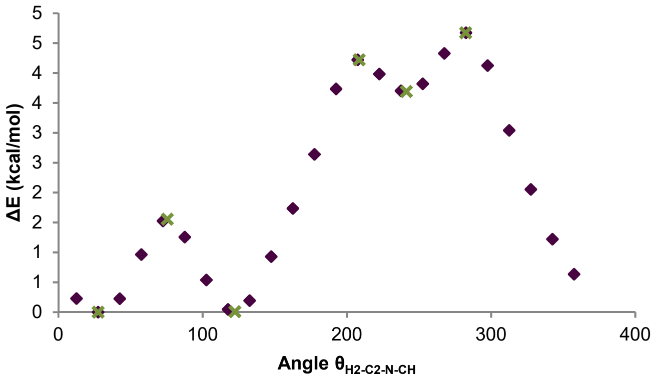

Figure S11*. Conformational profile of* ***54c*** *[M06-2X/6-311G(d,p) in gas phase].*

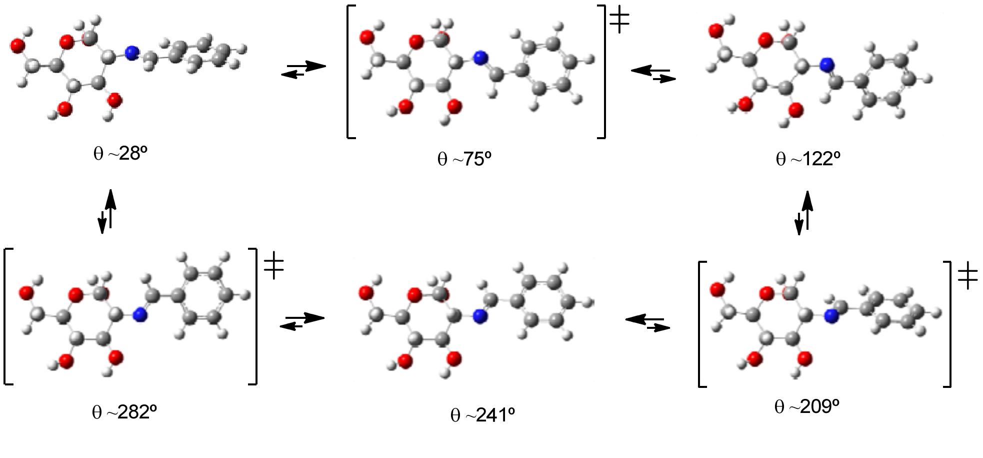

Figure S12. *Optimized structures of the stationary points for the conformational analysis of* ***54c*** *[M06-2X/6-311G(d,p) in gas phase].*

Table S15*. Relative energies (kcal/mol) of the maxima and minima for* ***54c****.*

	$\theta_{H2\text{-}C2\text{-}N\text{-}CH}$[a]	ΔE[a]	ΔG[a]	$\tilde{\nu}$‡d	$\theta_{H2\text{-}C2\text{-}N\text{-}CH}$[b]	ΔE[b]	ΔG[b]	$\tilde{\nu}$‡d
min 1	12.04	0.00	0.00	0	27.53	0.00	0.00	0
min 2	117.39	0.59	1.06	0	122.38	0.01	0.71	0
min 3	235.93	3.72	4.11	0	241.29	3.69	5.00	0
max 1	72.21	1.69	3.04	-50.8	75.46	1.56	3.80	-61.1
max 2	204.23	4.07	5.59	-34.4	208.51	4.22	5.43	-44.8
max 3	277.46	4.53	5.51	-54.3	282.31	4.67	5.69	-68.8

[a] B3LYP/6-31G(d,p); [b] M06-2X/6-311G(d,p); [c] Imaginary frequency in cm^{-1}.

Although the orientation of the hydroxyl group can modify the conformational profile, it does not reverse the stability order of the most stable conformation, which lies always in that taking a dihedral angle $\theta_{H2\text{-}C2\text{-}N\text{-}CH}$ close to 0º-20º.

Single-crystal X-ray diffraction of 60

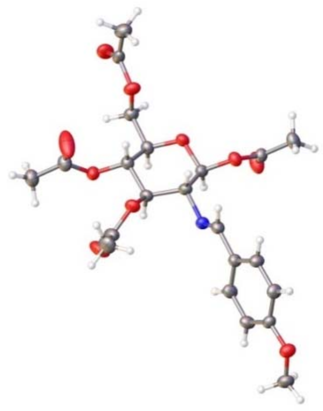

Figure S13. *ORTEP diagram of* ***44*** *obtained by X-ray diffraction.*

Table S16. *Crystal data and structure refinement of compound* ***44***

Empirical formula	$C_{22}H_{27}NO_{10}$	
Formula weight	465.45	
Temperature	120(2) K	
Wavelength	0.71069 Å	
Crystal system	Monoclinic	
Space group	$P2_1$	
Unit cell dimensions	a = 10.451(5) Å	
	b = 11.403(5) Å	β = 111.166(5)°
	c = 10.867(5) Å	
Volume	1207.7(10) Å^3	
Z	2	
Density (calculated)	1.280 Mg / m^3	
Absorption coefficient	0.102 mm^{-1}	
$F(000)$	492	
Crystal size	0.60 × 0.40 × 0.10 mm^3	
θ range for data collection	3.57 – 25.02°	
Index ranges	$-12 \leq h \leq 12$, $-13 \leq k \leq 13$, $-12 \leq l \leq 12$	
Reflections collected	10793	
Independent reflections	4259 [R_{int} = 0.0644]	
Completeness to θ = 25.02°	99.6 %	
Absorption correction	Semi–empirical from equivalents	
Max. and min. transmission	0.9899 and 0.9415	
Refinement method	Full-matrix least-squares on F^2	
Data / restraints / parameters	4259 / 1 / 304	
Goodness-of-fit on F^2	0.961	
Final R indices [$F^2 > 2\sigma(F^2)$]	$R1$ = 0.0501, $wR2$ = 0.1257	
R indices (all data)	$R1$ = 0.0615, $wR2$ = 0.1356	
Absolute structure parameter	not reliably determined	
Extinction coefficient	0.021(3)	
Largest diff. peak and hole	0.203 and –0.204 e Å^{-3}	

Table S17. *Experimental (X-ray) and Calculated Distances (Å) and Dihedral Angles (º) of **44**.*[a]

**Bond**	**X-Ray**	**Gas phase**[b]	**$CHCl_3$**[b]
C1-O_{endo}	1.42	1.40	1.40
C6-O_{endo}	1.42	1.41	1.42
C1-O_{exo}	1.41	1.40	1.40
C2-N	1.46	1.44	1.45
N-CH	1.27	1.27	1.27
**Dihedral angle**			
H1 C1 C2 H2	167.97	176.73	176.17
H2 C2 C3 H3	-165.78	-169.51	-169.93
H3 C3 C4 H4	166.71	165.84	167.22
H4 C4 C5 H5	-177.18	-176.05	-176.84
O C1 C2 C3	53.38	56.91	56.75
C1 C2 C3 C4	-49.35	-48.88	-49.39
C2 C3 C4 C5	51.80	49.65	50.70
C3 C4 C5 O	-58.14	-56.38	-57.18
C4 C5 O C1	65.32	65.39	65.59
C5 O C1 C2	-63.10	-66.86	-66.69
H2 C2 N CH	-0.52	11.60	12.35
C2 N CH H	-2.36	-1.37	-1.02
H5 C5 C6 O	-178.02	164.83	168.20
H2 C2 (=CH) H[c]	-2.58	9.54	10.53

[a] M06-2X/6-311G(d,p); [b]Structure of **44a**; [c]Dihiedral angle between separated atoms

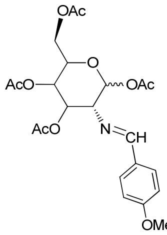

44a

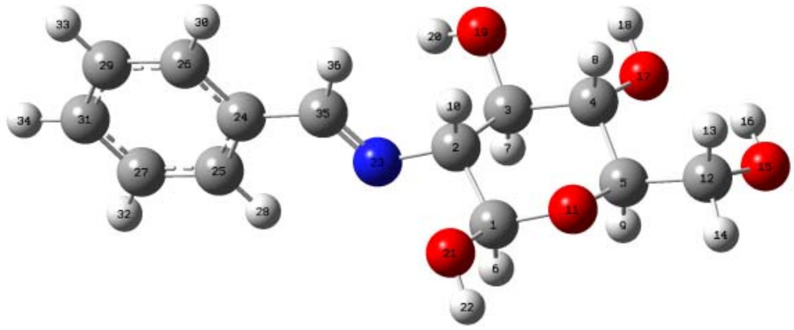

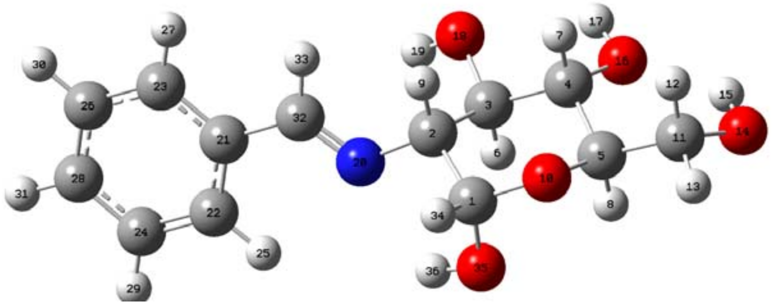

*Numbering used in the NBO analysis of **11** and **54***

Table S18. *NBO stabilizing interactions in **11**.*[a]

Donor	Acceptor	Gas phase[b]	Gas phase[c]	DMSO[b]	DMSO[c]	Water[b]
LP (1) N20	BD*(1) C2 – H10	6.28	6.53	6.95	7.18	6.41
LP (1) N20	BD*(1) C35- H36	13.11	13.92	12.55	12.44	12.24
LP (1) O35	BD*(1) C1 - C2	1.10	1.33	0.98	1.20	0.82
LP (2) O35	BD*(1) C1 - C2					
LP (2) O35	BD*(1) C1 - H6	7.21	7.09	5.82	5.71	4.55
LP (2) O35	BD*(1) C1 - O11	15.20	15.34	17.00	17.13	17.13
LP (1) O35	BD*(1) C1 - O11					
LP (1) O10	BD*(1) C1 – O21		5.31		5.01	
LP (2) O10	BD*(1) C1 – O21	4.97		4.69		4.63
LP (1) O10	BD*(1) C1 - H6		1.18		1.17	
LP (2) O10	BD*(1) C1 - C2	7.23	7.26	7.00	7.04	6.61
LP (2) O10	BD*(1) C4 - C5	7.21	7.26	7.30	7.36	7.12
LP (2) O10	BD*(1) C5 – H9	6.39	6.47	6.24	6.31	6.00

[a] M06-2X/6-311G(d,p). [b] Version 3.1. [c] Version 6.0.

*Table S19. NBO stabilizing interactions in **54**.[a]*

Donor	Acceptor	Gas phase[b]	Gas phase[c]	DMSO[b]	DMSO[c]	Water[b]
LP (1) N20	BD*(1) C2 – H10	6.38	6.66	6.08	6.39	5.62
LP (1) N20	BD*(1) C35- H36	12.95	12.79	12.06	11.92	11.90
LP (1) O35	BD*(1) C1 - C2	4.05	4.04	4.24	4.24	4.19
LP (2) O35	BD*(1) C1 - C2	2.33	2.43	1.53	1.611	1.17
LP (2) O35	BD*(1) C1 - H6	10.42	10.40	9.88	9.87	9.11
LP (2) O35	BD*(1) C1 - O11	3.32	3.39	4.57	4.67	5.09
LP (1) O35	BD*(1) C1 - O11	2.92	3.11	2.39	2.54	2.15
LP (1) O10	BD*(1) C1 – O21	1.14	1.11	0.95	0.92	0.83
LP (2) O10	BD*(1) C1 – O21	13.27	13.47	13.45	13.64	13.17
LP (1) O10	BD*(1) C1 - H6	3.86	3.80	3.74	3.68	3.59
LP (2) O10	BD*(1) C1 - C2	6.36	6.51	5.84	5.98	5.31
LP (2) O10	BD*(1) C4 - C5	5.85	6.01	5.74	5.89	5.47
LP (2) O10	BD*(1) C5 – H9	7.25	7.37	7.51	7.62	7.49

[a] M06-2X/6-311G(d,p). [b] Version 3.1. [c] Version 6.0.

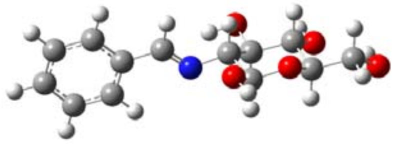

$\theta_{H\text{-}O\text{-}C1\text{-}C2}$ ~ 185º

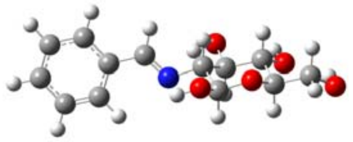

$\theta_{H\text{-}O\text{-}C1\text{-}C2}$ ~ 60º

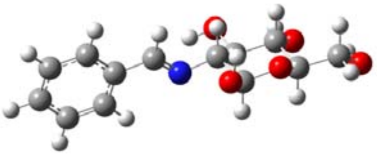

$\theta_{H\text{-}O\text{-}C1\text{-}C2}$ ~ 300º

Figure S14. *Conformational minima for the anomeric hydroxyl of **11** (gas phase).*

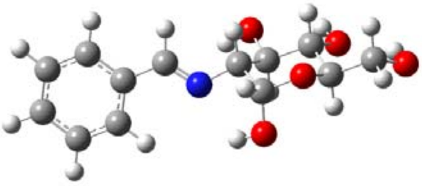

$\theta_{H\text{-}O\text{-}C1\text{-}C2}$ ~ 325º

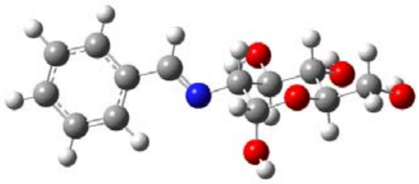

$\theta_{H\text{-}O\text{-}C1\text{-}C2}$ ~ 183º

Figure S15*. Conformational minima for the anomeric hydroxyl of **54** (gas phase).*

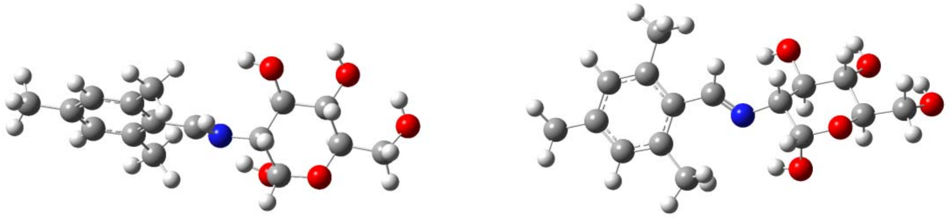

Figure S16. *Optimized structures for the α-anomer of **29** [M06-2X/6-311G(d,p) in gas phase].*

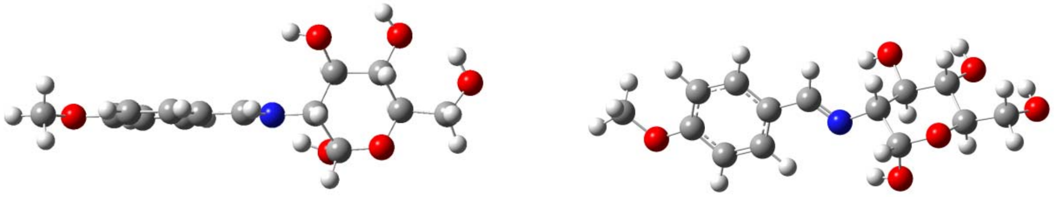

Figure S17. *Optimized structures for the α-anomer of **55** [M06-2X/6-311G(d,p) in gas phase].*

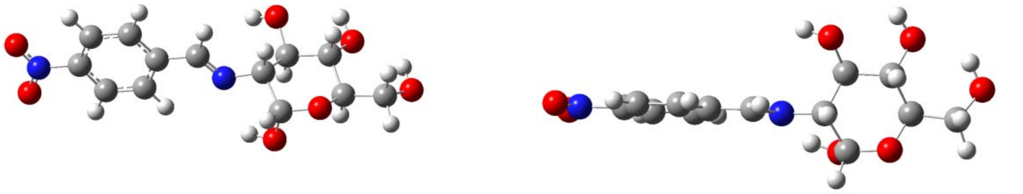

Figure S18. *Optimized structures for the α-anomer of **62** [M06-2X/6-311G(d,p) in gas phase].*

***Table S20**. Calculated geometric parameters of intramolecular hydrogen bonds for **29**, **32**, **34**, **54**, **55**, **62** and **112**.[a]*

		***D*-H···A**	***d*(*D*-H)**[b]	***d*(H···*A*)**[b]	***d*(*D*···*A*)**[b]	**<(*DHA*)**[c]	E_{HB}[d,e]
**29**	Gas phase	O-H···N	0.96544	2.17632	2.74381	116.273	6.84
	DMSO	O-H···N	0.96771	2.12493	2.7135	117.694	7.75
**32**	Gas phase	O-H···N	0.9627	2.36341	2.81727	108.243	5.05
	DMSO	O-H···N	0.96485	2.33273	2.82034	110.572	4.99
**34**	Gas phase	O-H···N	0.96278	2.34838	2.81776	109.346	5.04
	DMSO	O-H···N	0.96482	2.32196	2.81754	111.144	5.05
**54**	Gas phase	O-H···N	0.96492	2.20798	2.7599	115.148	6.40
	DMSO	O-H···N	0.96714	2.16297	2.73857	116.794	6.99
	Water	O-H···N	0.96738	2.20861	2.76816	115.638	6.19
**55**	Gas phase	O-H···N	0.96509	2.20406	2.75969	115.427	6.41
	DMSO	O-H···N	0.97449	2.10215	2.71787	119.511	7.61
**62**	Gas phase	O-H···N	0.96423	2.2268	2.76716	114.324	6.21
	DMSO	O-H···N	0.97074	2.19293	2.76333	116.275	6.31
**112**	Gas phase	O-H···N	0.96272	2.34715	2.81662	109.35	5.07
	DMSO	O-H···N	0.9647	2.3251	2.81952	111.072	5.01

[a] M06-2X/6-311G(d,p); [b] in Å; [c] in º; [d] in kcal mol^{-1}; [e] Equation 1, Reference 67.

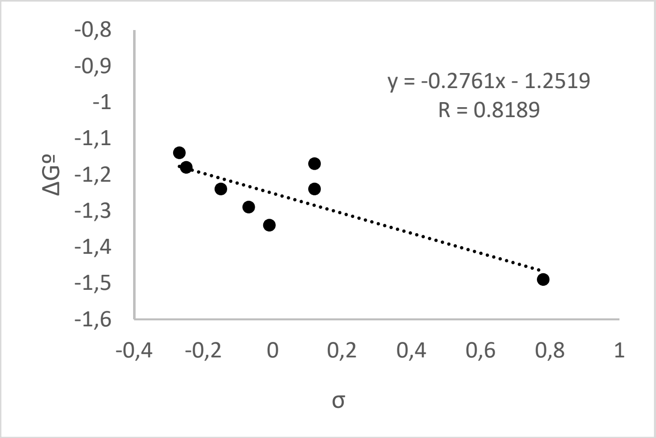

Figure S19. *Hammett plot of ΔGº for* ***12****,* ***13****,* ***16****,* ***19****,* ***21****,* ***23****,* ***25****, and* ***26****.*

Anomerization of per-*O*-acetylimines. To study the anomeric preference of imines in the absence of hydrogen bonding between the anomeric hydroxyl and the imine N, anomerization experiments with per-*O*-acetylimines were attempted using Brönsted and Lewis acid catalysts. The first anomerization experiments were conducted on α (**73**) and β (**60**) anomers derived from **1** and *p*-anisaldehyde. Acid catalysts have been used because the per-*O*-acetylated derivatives of aldoses and aminoaldoses are sensitive to bases, which promote elimination reactions (Vasella's work involving 1-deoxy-1-nitropyranose in basic medium use acetal groups for hydroxyl protection).[S1,S2]

The catalysts employed were both Brönsted and Lewis acids. Among the first ones, we chose sulfuric acid in acetic anhydride together with p-toluenesulfonic acid in glacial acetic acid and in the presence of acetic anhydride as well, to achieve anhydrous conditions and avoid the presence of water that could hydrolyze the iminic bond. Among Lewis acids, anhydrous zinc chloride in acetic anhydride, boron trifluoride in acetic anhydride, or titanium tetrachloride in chloroform were employed. Reactions were carried out at room temperature, although some experiments were performed at 80 °C. Quenching was performed by adding water, the resulting solid was collected by filtration and the aqueous phase extracted with chloroform, and washed successively with saturated sodium bicarbonate solution and water. The organic phase was dried and purified by chromatography.

The action of Brönsted (L=H^+) and Lewis acids (L) can be rationalized in terms of coordination with the anomeric acetate (**S3** and **S5**), making it possible a reversible detachment. Subsequent addition of acetate ion to the intermediate carbocation formed (**S4**) would lead to a thermodynamic equilibrium mixture of α and β anomers (**S1** and **S2**) (Scheme S1).

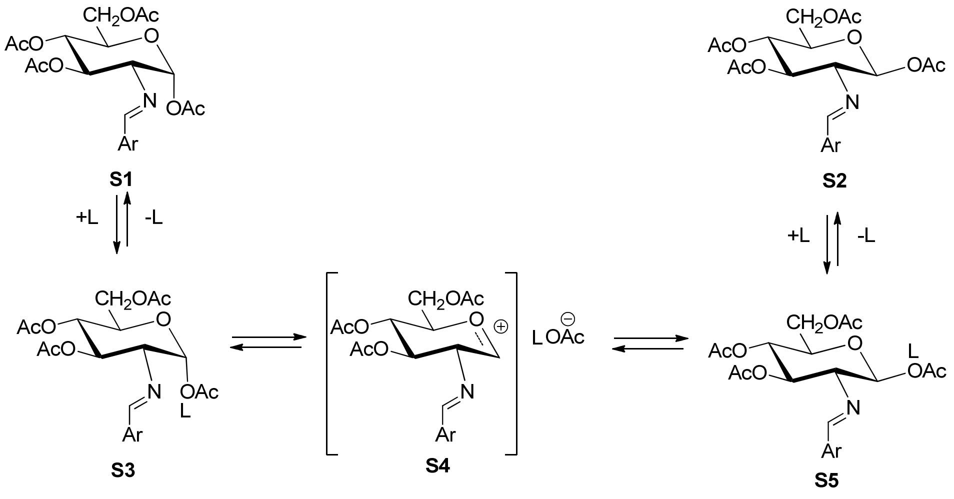

Scheme S1

All results were negative and the only products isolated with imine structure were the starting materials, which were recovered unaffected. In some experiments using compound **44**, significant amounts of 1,3,4,6-tetra-*O*-acetyl-2-acetamido-2-deoxy-β-D-glucopyranose could be isolated, which was identified by comparing its physical and spectroscopic properties with an authentic sample.

The failure of such experiments can be attributed to the basic properties shown by imines towards Brönsted acids (bear in mind the preparation of hydrochloride **36/37**) and the coordinating ability with metal-containing Lewis acids (see Scheme S1). Both types of acids coordinate with the imine nitrogen, preferentially to the oxygen of the anomeric acetate, thus inhibiting the equilibrium.

Anomerization of per-*O*-acetyl-2-(arylmethylene)amino-D-glucopyranosyl bromides. Given the failure of the aforementioned experiments, the corresponding glycosyl bromides derived from some imines were employed as starting materials for anomerization. The rationale behind this approach lies in the use of bromo derivatives in the presence of bromide ion, waiting for a thermodynamic equilibration between α and β bromoanomers (**S6** and **S7**) (Scheme S2).

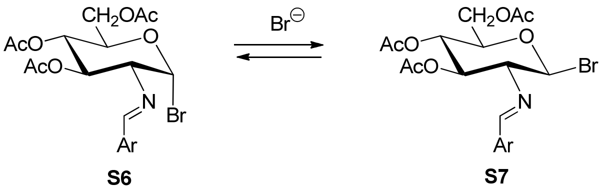

Scheme S2

This type of equilibrium has been reported previously and studied in both aldose and aminoaldose derivatives. Thus, Gervay *et al.*[S3] found that the α and β anomers of 2,3,4,6-tetra-*O*-acetyl-D-glucopyranosyl (**S9**, **S10**) and 2,3,4,6-tetra-*O*-acetyl-D-galactopyranosyl iodides (**S12**,

S13), generated by iodotrimethylsilane treatment of the corresponding per-*O*-acetylsugars (**S8**, **S11**) in the presence of iodide ion, equilibrate at low temperature and do quickly at room temperature (Scheme S3). The first product formed is the β anomer at -40 °C with a ratio **S12** (β):**S13** (α) = 30:1. Equilibration proceeds rapidly at room temperature, which favors instead the α anomer.

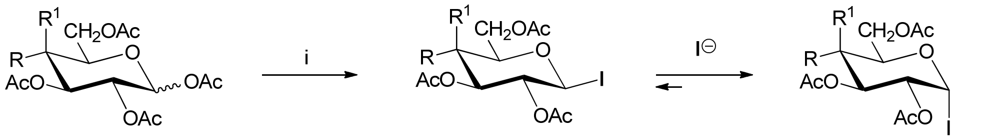

S8 R=OAc, R[1]=H
S11 R=H, R[1]=OAc

S9 R=OAc, R[1]=H
S12 R=H, R[1]=OAc

S10 R=OAc, R[1]=H
S13 R=H, R[1]=OAc

Scheme S3. *Reagents: i) Me_3SiI, CH_2Cl_2*

Portel *et al.*[S4] also evaluated the equilibration of the α and β anomers **S14** and **S15** (Scheme S4). As source of chloride ion for catalysis, benzyltriethylammonium chloride, soluble in numerous organic solvents, was employed.

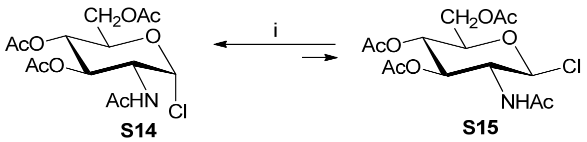

S14 **S15**

Scheme S4. *Reagents: i) $PhCH_2NEt_3Cl$, $CHCl_3$*

In following the procedure by Irving *et al.*,[S5] bromoimine **S16** was prepared as well, with equilibration conducted in the presence of tetrabutylammonium bromide as source of bromide ions (Scheme S5). A gradual decomposition was observed over time, albeit the signal for the anomeric proton of the β-anomer (**S17**) could not be detected. Additional experiments were unsuccessful as well.

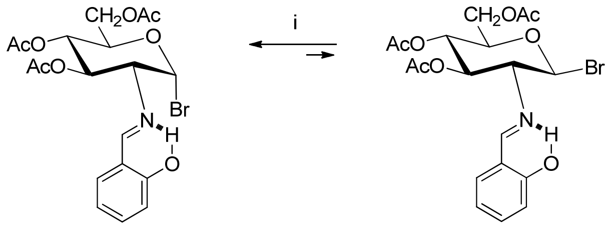

S16 **S17**

Scheme S5. *Reagents: i) Bu_4NBr, $CDCl_3$*

Since interconversion between anomers should be feasible, the failure to detect the β-anomer could be ascribed to the higher stability of the α-anomer, resulting from a strong anomeric effect that largely favors its almost exclusive formation.

References

[S1] Aebischer, B.; Hollenstein, R.; Vasella, A. Deoxy-nitrosugars. 5th Communication. The anomeric effect of the nitro group. *Helv. Chim. Acta.* **1983**, *66*, 1748-1754.

[S2] Baumberger, F.; Vasella, A.; Schauer, R. Synthesis of New Sialidase Inhibitors, 6-Amino-6-Deoxysialic Acids. *Helv. Chim. Acta*. **1988**, *71*, 429-445.

[S3] Gervay, J.; Nguyen, T. N.; Hadd, M. J. Mechanistic studies on the stereoselective formation of glycosyl iodides: first characterization of β-D-glycosyl iodides. *Carbohydr. Res*. **1997**, *300*, 119-125.

[S4] Portel, S. S.; Chirva, V. Ya.; Kadun, A. L.; Kakayan, E. S. The application of the intermediate 2-methyl-glyco-[2,1-*d*]-2-oxazolines for glycoside synthesis. *Carbohydr. Res*. **2000**, *329*, 895-899.

[S5] (a) Irvine, J. C.; Hynd, A. VIII.-Synthetical aminoglucosides derived from *d*-glucosamine**.** *J. Chem. Soc*., *Trans.* **1913**, *103*, 41-56. (b) Irvine, J. C.; Earl, J. C. CCLXXXV.-Mutarotation and pseudo-mutarotation of glucosamine and its derivatives. *J. Chem. Soc*., *Trans.* **1922**, *121*, 2370-2376.

Table S21. *Calculated Relative Energies (kcal/mol) for O-Acylated Derivatives*

	Gas phase		$CHCl_3$		Gas phase		$CHCl_3$	
	ΔE[a]	ΔG[a]	ΔE[a]	ΔG[a]	ΔE[b]	ΔG[b]	ΔE[b]	ΔG[b]
**46**	0.0	0.0	0.0	0.0	0.0	0.0	0.0	0.0
**43**	-0.1	1.6	0.2	1.7	-0.5	0.2	-0.1	1.0
**97α**	0.0	0.0	0.0	0.0	0.0	0.0	0.0	0.0
**97β**	1.5	0.9	1.34	2.0	2.5	2.4	2.4	1.8
**98α**	0.0	0.0	0.0	0.0	0.0	0.0	0.0	0.0
**98β**	0.8	2.3	1.0	0.4	0.0	2.0	0.7	1.3
**99α**	0.0	0.0	0.0	0.0	0.0	0.0	0.0	0.0
**99β**	1.0	2.4	1.0	1.8	0.3	2.2	0.8	0.3
**100α**	0.0	0.0	0.0	0.0	0.0	0.0	0.0	0.0
**100β**	1.6	1.8	1.1	2.0	2.9	3.1	2.5	2.5
**101α**	0.0	0.0	0.0	0.0	0.0	0.0	0.0	0.0
**101β**	0.1	1.3	0.1	-0.8	0.1	1.1	0.3	0.45
**102α**	0.0	0.0	0.0	0.0	0.0	0.0	0.0	0.0
**102β**	2.4	4.3	2.2	0.3	1.6	3.9	1.4	4.7
**103 α**	5.7	7.2	5.0	6.9	3.8	5.0	3.1	2.2
**103 β**	0.0	0.0	0.0	0.0	0.0	0.0	0.0	0.0

[a]At B3LYP/6-31G(d,p); [b]At M06-2X/6-311G(d,p).

Table S22. *Relative energies (kcal/mol) for Aryl-substituted Imines*

	Gas phase[a]		DMSO[a]		Gas phase[b]		DMSO[b]	
**Comp.**	**ΔE**	**ΔG**	**ΔE**	**ΔG**	**ΔE**	**ΔG**	**ΔE**	**ΔG**
**11**	1.01	0.22	0.87	1.20	1.22	0.21	0.99	0.36
**54**	0.00	0.00	0.00	0.00	0.00	0.00	0.00	0.00
**12**	1.24	0.52	1.01	1.44	1.40	0.52	1.09	0.49
**55**	0.00	0.00	0.00	0.00	0.00	0.00	0.00	0.00
**19**	0.14	-0.50	0.62	0.07	0.37	0.12	0.81	0.39
**62**	0.00	0.00	0.00	0.00	0.00	0.00	0.00	0.00
**30**	0.91	-0.10	0.86	0.34	1.71	0.68	1.24	0.80
**29**	0.00	0.00	0.00	0.00	0.00	0.00	0.00	0.00

[a]B3LYP/6-31G(d,p); [b]M06-2X/6-311G(d,p).

Table S23. *Calculated Relative Energies (kcal/mol) for Salicylaldehyde Derivatives*

		Gas phase		DMSO		Gas phase		DMSO	
	Anomer	ΔE[a]	ΔG[a]	ΔE[a]	ΔG[a]	ΔE[b]	ΔG[b]	ΔE[b]	ΔG[b]
**32**	α	1.1	1.5	0.9	0.8	0.6	0.7	0.4	0.4
**33**	β	0.0	0.0	0.0	0.0	0.0	0.0	0.0	0.0
**105**	α	4.2	4.1	1.1	1.8	6.7	6.5	3.2	3.0
**106**	β	5.7	4.8	2.6	2.5	8.7	7.9	5.3	4.8
**34**	α	0.9	1.4	0.7	0.8	0.5	0.7	0.2	0.3
**35**	β	0.0	0.0	0.0	0.0	0.0	0.0	0.0	0.0
**107**	α	3.8	3.6	0.3	1.0	6.1	6.1	2.0	2.3
**108**	β	5.1	4.3	1.7	1.9	7.9	7.2	4.1	3.7
**109**	α	0.6	1.2	0.7	0.8	0.1	0.4	0.2	0.2
**110**	β	0.0	0.0	0.0	0.0	0.0	0.0	0.0	0.0
**111**	α	2.4	2.7	-0.6	-0.1	4.1	4.4	0.8	1.4
**112**	β	4.0	2.9	1.0	0.3	6.4	5.9	3.0	2.8

[a]At B3LYP/6-31G(d,p); [b]At M06-2X/6-311G(d,p).

Table S24. *Calculated Relative Energies (kcal/mol) for Anomers and Conformers of **36**-**37***

		Gas phase[a]		DMSO[a]		Water[a]		Gas phase[b]		DMSO[b]		Water[b]	
	Anomer	ΔE	ΔG	ΔE	ΔG	ΔE	ΔG	ΔE	ΔG	ΔE	ΔG	ΔE	ΔG
**36a**	α	0.0	0.0	0.0	0.0	0.0	0.0	0.0	0.0	0.0	0.0	0.0	0.0
**36b**	α	2.5	2.5	1.5	1.1	0.4	0.8	1.5	1.7	0.9	1.1	0.7	0.5
**37a**	β	3.2	2.5	1.8	1.1	1.2	1.0	3.8	2.4	2.2	1.0	1.7	1.5
**37b**	β	5.3	4.7	2.9	2.1	2.2	1.6	4.9	3.9	2.5	1.8	2.0	1.5

[a]At B3LYP/6-31G(d,p); [b]At M06-2X/6-311G(d,p).

Table S25. *Geometric parameters of hydrogen bonding for **36a** and **36b**.*

		D-H···A	d(D-H)	d(H···A)	d(D···A)	<(DHA)	E_{HB}[c]
**36a**	Gas phase[a]	N-H···O	1.0154	2.0213	2.7610	127.64	-6.37
		N-H···O	1.0154	2.4892	2.8225	98.41	-4.95
	DMSO[a]	N-H···O	1.0147	2.0407	2.7650	126.30	-6.27
		N-H···O	1.0147	2.5277	2.8305	96.64	-4.79
	Water[a]	N-H···O	1.0152	2.0345	2.7701	127.31	-5.84
		N-H···O	1.0152	2.5463	2.8459	94.50	-4.49
	Gas phase[b]	N-H···O	1.0144	2.0065	2.7423	127.28	-6.88
		N-H···O	1.0144	2.4347	2.7795	98.95	-5.91
	DMSO[b]	N-H···O	1.0137	2.0257	2.7432	125.65	-6.86
		N-H···O	1.0137	2.4865	2.7864	96.31	-5.74
	Water[b]	N-H···O	1.0144	2.0155	2.7674	128.88	-6.21
		N-H···O	1.0144	2.4392	2.7783	98.60	-5.93
**36b**	Gas phase[a]	N-H···O	1.0147	2.4107	2.7899	101.11	-5.66
	DMSO[a]	N-H···O	1.0141	2.4922	2.8142	97.74	-5.12
	Water[a]	N-H···O	1.0148	2.4050	2.7611	99.55	-6.37
	Gas phase[b]	N-H···O	1.0142	2.3959	2.7630	100.25	-6.32
	DMSO[b]	N-H···O	1.0139	2.4939	2.7869	95.89	-5.73
	Water[b]	N-H···O	1.0145	2.4854	2.7973	97.04	-5.49

[a] B3LYP/6-31G(d); [b] M06-2X/6-311G(d,p); [c]From equation [2], Reference 67

Table S26. *Calculated Relative Energies (kcal/mol) for Hydrated Anomers*

	Gas phase[a]	Gas phase[b]		DMSO[b]		Water[b]	
	ΔE	ΔE	ΔG	ΔE	ΔG	ΔE	ΔG
**11·1H$_2$O**	4.98	4.91	2.76	2.55	1.80	0.83	-1.59
**54·1H$_2$O**	0.00	0.00	0.00	0.00	0.00	0.00	0.00
**11·5H$_2$O**	0.00	0.00	0.00	0.00	0.00	0.00	0.00
**54·5H$_2$O**	0.39	0.65	1.99	1.13	3.48	1.02	5.90
**11·6H$_2$O**		0.00	0.00	0.00	0.00	0.00	0.00
**54·6H$_2$O**		1.89	2.79	2.26	4.23	3.34	6.46
**12·6H$_2$O**		0.00	0.00	0.00	0.00	0.00	0.00
**55·6H$_2$O**		8.25	6.27	6.68	4.35	4.51	5.98
**19·6H$_2$O**		0.00	0.00	0.00	0.00	0.00	0.00
**62·6H$_2$O**		2.38	3.63	2.58	3.72	3.40	6.62

[a]At B3LYP/6-31G(d); [b]At B3LYP/6-31G(d,p)

Table S27. *Calculated Relative Energies (kcal/mol) for Hydrated Anomers.[a]*

	Gas phase		DMSO		Water	
	ΔE	ΔG	ΔE	ΔG	ΔE	ΔG
**11·1H$_2$O**	4.1	3.7	2.1	2.7	0.2	0.2
**54·1H$_2$O**	0.0	0.0	0.0	0.0	0.0	0.0
**11·5H$_2$O**	0.0	0.0	0.0	0.0	0.0	0.0
**54·5H$_2$O**	-3.9	-1.9	-3.0	0.2	-2.0	0.2
**12·5H$_2$O**	0.0	0.0	0.0	0.0	0.0	0.0
**55·5H$_2$O**	-4.0	-2.9	-3.1	0.0	-2.0	1.0
**19·5H$_2$O**	0.0	0.0	0.0	0.0	0.0	0.0
**62·5H$_2$O**	-3.1	-1.4	-2.8	0.7	-2.1	1.7
**11·6H$_2$O**	0.0	0.0	0.0	0.0	0.0	0.0
**54·6H$_2$O**	-0.2	1.1	-0.4	0.7	-0.4	3.3
**12·6H$_2$O**	0.0	0.0	0.0	0.0	0.0	0.0
**55·6H$_2$O**	5.9	4.8	3.8	3.9	3.2	3.6
**19·6H$_2$O**	0.0	0.0	0.0	0.0	0.0	0.0
**62·6H$_2$O**	0.5	0.7	-0.2	0.9	0.6	2.7

[a]At M06-2X/6-311G(d,p).

Table S28. *Geometric Parameters of H-Bonding for **11** and **54** with Different Hydration Degrees.*[a]

		θ[c]	D-H···A	d(D-H)	d(H···A)	d(D···A)	<(DHA)
**11·1H₂O**	Gas phase	30.47	O38-H37···N7	0.968	1.982	2.903	158.236
			O38-H39···O16	0.962	2.340	3.021	127.241
			O18-H26···O13	0.963	2.252	2.737	110.106
	DMSO	25.31	O38-H37···N7	0.971	1.982	2.896	155.989
			O38-H39···O16	0.964	2.210	2.961	133.872
			O18-H26···O13	0.964	2.267	2.744	109.491
	Water	25.07	O38-H37···N7	0.975	1.943	2.882	160.997
			O38-H39···O16	0.965	2.282	2.994	129.876
			O18-H26···O13	0.964	2.376	2.798	105.863
**54·1H₂O**	Gas phase	37.02	O38-H37···N8	0.981	1.848	2.792	160.279
			O16-H30···O38	0.973	1.819	2.743	157.541
			O18-H26···O13	0.963	2.241	2.740	111.152
	DMSO	0.87	O38-H37···N8	0.978	1.856	2.761	152.430
			O16-H30···O38	0.972	1.850	2.822	179.721
			O18-H26···O13	0.964	2.262	2.747	110.110
	Water	29.26	O38-H37···N8	0.981	1.882	2.832	162.447
			O16-H30···O38	0.975	1.808	2.759	164.085
			O18-H26···O13	0.964	2.398	2.804	104.812
**11·5H₂O**	Gas phase	-30.85	O22-H42···N8	0.975	1.950	2.833	149.412
			O22-H43···O19	0.969	2.028	2.846	140.839
	DMSO	-13.59	O22-H42···N8	0.972	2.001	2.857	145.703
			O22-H43···O19	0.970	1.948	2.816	147.616
	Water	-21.56	O22-H42···N8	0.981	1.878	2.850	170.100
			O22-H43···O19	0.965	2.511	3.125	121.475
**54·5H₂O**	Gas phase	18.10	O50-H49···N8	0.974	1.908	2.801	151.192
			O16-H30···O50	0.974	1.826	2.757	158.815
	DMSO	8.74	O50-H49···N8	0.978	1.883	2.792	153.350
			O16-H30···O50	0.976	1.815	2.750	159.144
	Water	17.12	O50-H49···N8	0.978	1.912	2.843	158.082
			O16-H30···O50	0.972	1.958	2.823	146.922
**11·6H₂O**	Gas phase	-24.74	O22-H42···N8	0.981	1.832	2.799	167.969
			O21-H46···O50	0.979	1.783	2.730	163.410
	DMSO	-22.03	O22-H42···N8	0.980	1.879	2.828	162.214
			O21-H46···O50	0.978	1.805	2.773	170.014
	Water	-20.74	O22-H42···N8	0.983	1.857	2.863	173.520
			O21-H46···O50	0.978	1.796	2.768	171.879
**54·6H₂O**	Gas phase	19.929	O50-H49···N8	0.974	1.902	2.803	152.615
			O16-H30···O50	0.974	1.840	2.758	155.880
	DMSO	-2.368	O50-H49···N8	0.987	1.845	2.776	155.975
			O16-H30···O50	0.985	1.817	2.770	161.678
	Water	3.895	O50-H49···N8	0.987	1.858	2.806	159.935
			O16-H30···O50	0.982	1.910	2.828	154.597

[a] M06-2X/6-311G(d,p), [b] Distances in Å and angles in º; [c] $\theta_{H2\text{-}C2\text{-}N=C}$.

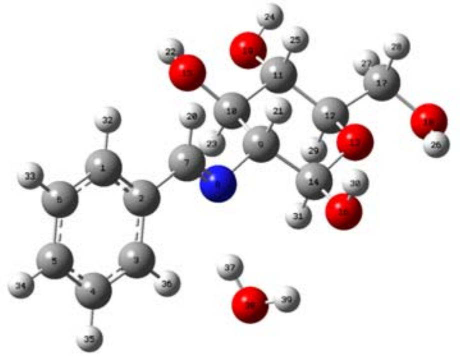

11.1H$_2$O

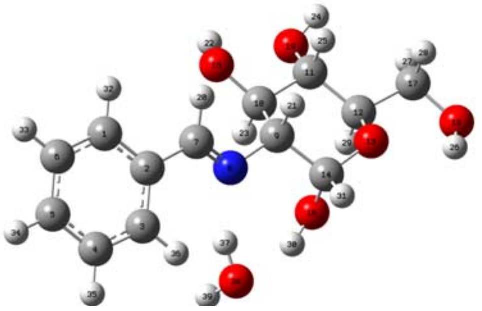

54.1H$_2$O

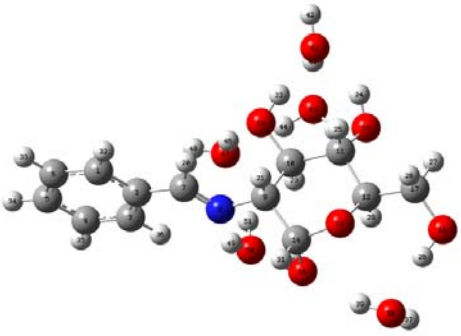

11.5H$_2$O

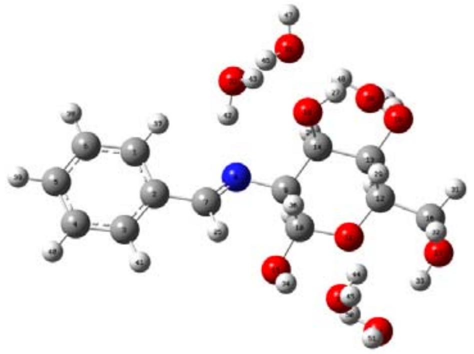

54.5H$_2$O

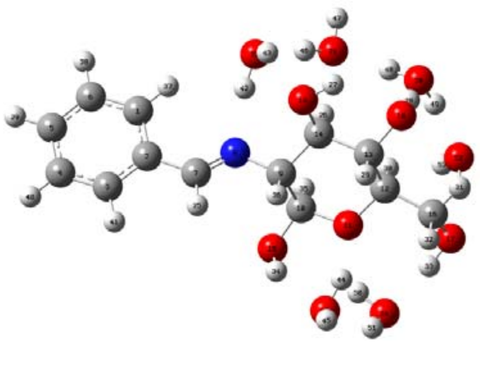

11.6H$_2$O

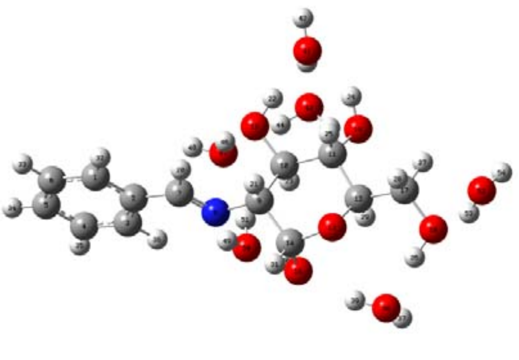

54.6H$_2$O

Table S29. *NBO Stabilizing Interactions of Hydrates Derived from* ***11****.*[a]

		**$11\cdot 1H_2O$**			**$11\cdot 5H_2O$**				
Donor	Acceptor	Gas phase[b]	DMSO[b]	Water[b]	Gas phase[b]	Gas phase[c]	DMSO[b]	DMSO[c]	Water[b]
LP (1) N23	BD*(1) C2 - H10	5.54	5.40	5.26	5.19	5.46	6.32	6.63	5.45
LP (1) N23	BD*(1) C35 - H36	12.67	11.95	11.70	12.81	12.77	12.00	11.91	11.65
LP (2) O21	BD*(1) C1 - H6	3.29	3.15	2.98	3.88	3.87	3.76	3.74	3.46
LP (2) O21	BD*(1) C1 - O11	16.59	16.90	16.93	14.06	14.14	14.09	14.15	14.11
LP (1) O21	BD*(1) C1 - C2	3.36	3.58	3.51	2.61	2.51	2.55	2.45	2.62
LP (2) O21	BD*(2) C1 - C2	4.76	4.01	3.60	7.07	7.19	6.97	7.08	6.00
LP (2) O11	BD*(1) C1 - O21	4.44	4.41	4.39	5.04	5.31	5.06	5.32	4.88
LP (2) O11	BD*(1) C1 - H6	6.04	5.97	5.79	7.24	7.36	6.78	6.86	6.00
LP (2) O11	BD*(1) C1 - C2	6.91	7.07	6.90	4.21	4.34	5.76	5.78	6.19
LP (2) O11	BD*(1) C4 - C5	7.26	7.13	7.12	3.97	4.10	5.56	5.63	6.38
LP (2) O11	BD*(1) C5 - H9	6.00	5.93	5.82	6.84	6.96	6.76	6.86	5.84

[a] M06-2X/6-311G(d,p). [b] Version 3.1. [c] Version 6.0.

Table S30. *NBO Stabilizing Interactions of Hydrates Derived from* ***54****.*[a]

		**$54\cdot 1H_2O$**			**$54\cdot 5H_2O$**				
Donor	Acceptor	Gas phase[b]	DMSO[b]	Water[b]	Gas phase[b]	Gas phase[c]	DMSO[b]	DMSO[c]	Water[b]
LP (1) N20	BD*(1) C2 - H9	3.98	6.15	4.50	6.21	6.53	6.07	6.40	5.80
LP (1) N20	BD*(1) C32- H33	12.21	11.80	11.57	12.80	12.80	11.75	12.80	11.63
LP (1) O35	BD*(1) C1 - C2		6.21		4.82	4.72	4.27	4.17	3.10
LP (2) O35	BD*(1) C1 - C2	10.60		9.56	1.58	1.53	1.67	1.60	3.26
LP (1) O35	BD*(1) C1 - H34	4.09	1.65	3.28	3.99	3.98	4.24	4.23	4.12
LP (2) O35	BD*(1) C1 - H34	2.33	8.31	2.93	3.13	3.20	2.62	2.70	1.10
LP (2) O35	BD*(1) C1 - H10	5.33	11.42	3.70	16.37	16.37	15.17	15.67	16.14
LP (1) O35	BD*(1) C1 - H10	2.76	1.24	3.13					0.58
LP (2) O10	BD*(1) C1 - O35	10.28	11.18	11.50	12.66	12.92	13.18	13.18	13.64
LP (1) O10	BD*(1) C1 - H34	3.45	3.51	3.46	3.86	3.78	3.75	3.75	3.65
LP (2) O10	BD*(1) C1 - C2	6.16	5.92	5.51	5.78	5.92	5.47	5.47	5.04
LP (2) O10	BD*(1) C4 - C5	6.21	6.33	5.67	6.77	6.98	6.46	6.46	6.08
LP (2) O10	BD*(1) C5 - H8	6.70	6.72	7.16	6.98	7.07	7.23	7.23	6.82
LP (1) O10	BD*(1) C1 - O35	1.49	1.36	1.11	1.32	1.27	1.15	1.15	1.00

[a] M06-2X/6-311G(d,p). [b] Version 3.1. [c] Version 6.0.

Table S31. *NBO Stabilizing Interactions of Hydrates Derived from **54**.*[a]

		$54\cdot5H_2O$[b]			$54\cdot5H_2O$[c*]				
**Donor**	**Acceptor**	**Gas phase**[e]	**DMSO**[e]	**Water**[d]	**Gas phase**[d]	**Gas phase**[e]	**DMSO**[d]	**DMSO**[e]	**Water**[d]
LP (1) N20	BD*(1) C2 - H9	6.53	6.40	5.80	5.69	5.92	6.18	6.48	5.86
LP (1) N20	BD*(1) C1- H34	12.80	12.80	11.63	12.50	12.35	11.71	11.62	11.80
LP (1) N20	BD*(1) O35- H36				2.80	3.12	2.61	2.90	2.01
LP (1) O35	BD*(1) C1 - C2	4.72	4.17	3.10	3.51	3.51	3.94	3.95	4.67
LP (2) O35	BD*(1) C1 - C2	1.53	1.60	3.26	2.19	2.22	1.35	1.39	
LP (1) O35	BD*(1) C1 - H34	3.98	4.23	4.12					1.29
LP (2) O35	BD*(1) C1 - H34	3.20	2.70	1.10	9.86	9.82	9.01	8.98	7.50
LP (1) O35	BD*(1) C1 - O10			0.58	2.96	3.10	2.36	2.43	1.24
LP (2) O35	BD*(1) C1 - O10	16.37	15.67	16.14	3.30	3.39	4.43	4.55	6.32

[a] M06-2X/6-311G(d,p). [b] With a water molecule between the N and the anomeric OH. [c] With direct bonding between the nitrogen and the anomeric OH. [d] Version 3.1. [e] Version 6.0.

Table S32. *NBO Stabilizing Interactions of Hydrates Derived from **55**.*[a,b]

		$55\cdot5H_2O$[c]			$55\cdot5H_2O$[d]		
**Donor**	**Acceptor**	**Gas phase**	**DMSO**	**Water**	**Gas phase**	**DMSO**	**Water**
LP (1) N20	BD*(1) C2 - H9	6.22	6.09	5.87	5.75	6.26	5.81
LP (1) N20	BD*(1) C1- H34	12.82	11.78	11.66	12.47	11.71	11.81
LP (1) N21	BD*(1) O35- H36				2.94	2.71	2.20
LP (1) O35	BD*(1) C1 - C2	5.10	4.30	3.08	2.91	4.95	4.91
LP (2) O35	BD*(1) C1 - C2	1.25	1.59	3.25	2.77		
LP (1) O35	BD*(1) C1 - H34	3.90	4.24	4.12		1.88	2.28
LP (2) O35	BD*(1) C1 - H34	3.54	2.69	1.09	9.66	6.52	6.11
LP (1) O35	BD*(1) C1 - O10			0.58	3.36	0.88	0.55
LP (2) O35	BD*(1) C1 - O10	15.72	15.01	16.11	2.46	6.04	7.33

[a] M06-2X/6-311G(d,p). [b] Version 3.1. [c] With a water molecule between the N and the anomeric OH. [d] With direct bonding between the nitrogen and the anomeric OH.

Table S33. *NBO Stabilizing Interactions of Hydrates Derived from **62**.*[a,b]

		$62\cdot5H_2O$[c]			$62\cdot5H_2O$[d]		
**Donor**	**Acceptor**	**Gas phase**	**DMSO**	**Water**	**Gas phase**	**DMSO**	**Water**
LP (1) N20	BD*(1) C2 - H9	6.19	5.95	5.67	6.08	6.00	5.51
LP (1) N20	BD*(1) C1- H34	12.84	11.72	11.60	12.74	11.86	11.75
LP (1) N21	BD*(1) O35- H36				2.36	2.65	2.11
LP (1) O35	BD*(1) C1 - C2	4.84	4.12	3.06	3.70	3.85	4.49
LP (2) O35	BD*(1) C1 - C2	1.67	1.98	3.48	2.04	1.52	0.72
LP (1) O35	BD*(1) C1 - H34	4.02	4.25	4.11			0.86
LP (2) O35	BD*(1) C1 - H34	3.07	2.33	0.97	9.89	9.13	8.07
LP (1) O35	BD*(1) C1 - O10			0.63	2.88	2.50	1.71
LP (2) O35	BD*(1) C1 - O10	16.38	15.58	16.23	3.62	4.17	5.40

[a] M06-2X/6-311G(d,p). [b] Version 3.1. [c] With a water molecule between the N and the anomeric OH. [d] With direct bonding between the nitrogen and the anomeric OH.

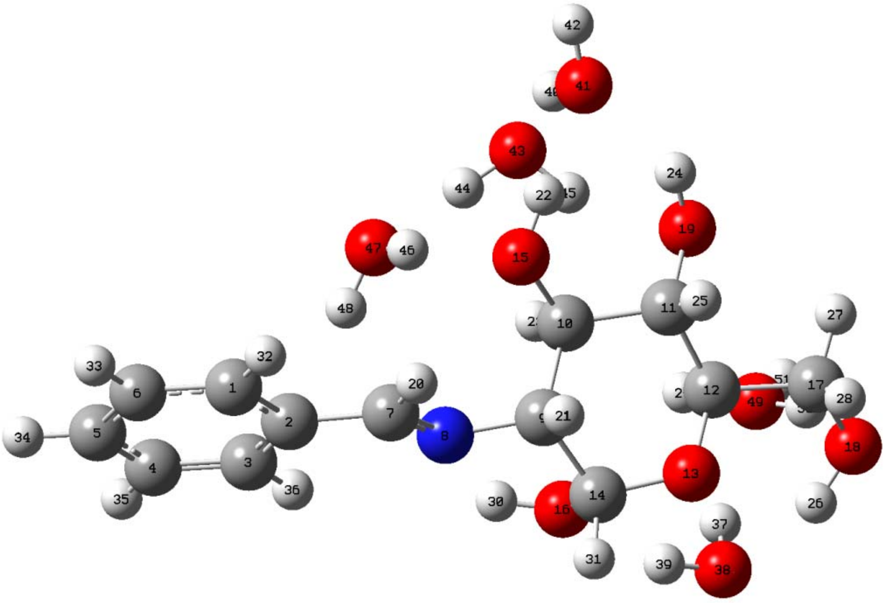

Table S34. NBO/NLMO STERIC ANALYSIS **54.5H$_2$O**:

Occupied NLMO contributions dE(i) (kcal/mol) to total steric exchange energy

NLMOs (i) in unit 1	dE(i)
1. CR (1) C 1	-0.03
2. CR (1) C 2	-0.02
3. CR (1) C 3	-0.03
4. CR (1) C 4	-0.03
5. CR (1) C 5	-0.03
6. CR (1) C 6	-0.03
7. CR (1) C 7	-0.02
8. CR (1) N 8	-0.02
9. CR (1) C 9	-0.02
10. CR (1) C 10	-0.02
11. CR (1) C 11	-0.02
12. CR (1) C 12	-0.02
13. CR (1) O 13	-0.02
14. CR (1) C 14	-0.02
15. CR (1) O 15	-0.01
16. CR (1) O 16	-0.01
17. CR (1) C 17	-0.02
18. CR (1) O 18	-0.01
19. CR (1) O 19	-0.02
25. LP (1) N 8	31.81
26. LP (1) O 13	18.40
27. LP (2) O 13	17.14
28. LP (1) O 15	11.35
29. LP (2) O 15	10.23
30. LP (1) O 16	12.16
31. LP (2) O 16	6.89
32. LP (1) O 18	8.53
33. LP (2) O 18	12.39
34. LP (1) O 19	12.71
35. LP (2) O 19	8.02
46. BD (1) C 1- C 2	81.03
47. BD (2) C 1- C 2	-17.04
48. BD (1) C 1- C 6	75.15
49. BD (1) C 1- H 32	5.42
50. BD (1) C 2- C 3	78.32
51. BD (1) C 2- C 7	65.29
52. BD (1) C 3- C 4	71.34
53. BD (2) C 3- C 4	-16.61
54. BD (1) C 3- H 36	4.79
55. BD (1) C 4- C 5	70.24
56. BD (1) C 4- H 35	2.56
57. BD (1) C 5- C 6	70.10
58. BD (2) C 5- C 6	-17.76

```
  59. BD ( 1) C  5- H 34            2.77
  60. BD ( 1) C  6- H 33            2.78
  61. BD ( 1) C  7- N  8           66.45
  62. BD ( 2) C  7- N  8           -6.61
  63. BD ( 1) C  7- H 20            5.02
  64. BD ( 1) N  8- C  9           59.03
  65. BD ( 1) C  9- C 10           75.51
  66. BD ( 1) C  9- C 14           62.80
  67. BD ( 1) C  9- H 21            6.06
  68. BD ( 1) C 10- C 11           66.14
  69. BD ( 1) C 10- O 15           40.83
  70. BD ( 1) C 10- H 23           10.58
  71. BD ( 1) C 11- C 12           67.75
  72. BD ( 1) C 11- O 19           41.41
  73. BD ( 1) C 11- H 25            3.58
  74. BD ( 1) C 12- O 13           43.89
  75. BD ( 1) C 12- C 17           60.10
  76. BD ( 1) C 12- H 29            9.33
  77. BD ( 1) O 13- C 14           51.03
  78. BD ( 1) C 14- O 16           42.00
  79. BD ( 1) C 14- H 31           -3.86
  80. BD ( 1) O 15- H 22            3.35
  81. BD ( 1) O 16- H 30            3.73
  82. BD ( 1) C 17- O 18           28.97
  83. BD ( 1) C 17- H 27            3.53
  84. BD ( 1) C 17- H 28            1.90
  85. BD ( 1) O 18- H 26            4.55
  86. BD ( 1) O 19- H 24            6.05

Steric exchange energy, unit 1:  1380.73 kcal/mol

NLMOs (i) in unit  2               dE(i)
  20. CR ( 1) O 38                  0.01
  36. LP ( 1) O 38                 -1.47
  37. LP ( 2) O 38                  2.32
  87. BD ( 1) H 37- O 38           -2.75
  88. BD ( 1) O 38- H 39           -2.28

Steric exchange energy, unit 2:    -4.17 kcal/mol

NLMOs (i) in unit  3               dE(i)
  21. CR ( 1) O 41                 -0.01
  38. LP ( 1) O 41                 -1.18
  39. LP ( 2) O 41                  7.47
  89. BD ( 1) H 40- O 41           -7.66
  90. BD ( 1) O 41- H 42           -4.60

Steric exchange energy, unit 3:    -5.98 kcal/mol

NLMOs (i) in unit  4               dE(i)
  22. CR ( 1) O 43                  0.00
  40. LP ( 1) O 43                 -3.35
  41. LP ( 2) O 43                  8.90
  91. BD ( 1) O 43- H 44           -5.87
  92. BD ( 1) O 43- H 45           -3.54

Steric exchange energy, unit 4:    -3.87 kcal/mol

NLMOs (i) in unit  5               dE(i)
  23. CR ( 1) O 47                  0.00
  42. LP ( 1) O 47                 -4.15
  43. LP ( 2) O 47                  3.08
  93. BD ( 1) H 46- O 47           -3.66
  94. BD ( 1) O 47- H 48           -3.29

Steric exchange energy, unit 5:    -8.03 kcal/mol

NLMOs (i) in unit  6               dE(i)
  24. CR ( 1) O 49                  0.00
  44. LP ( 1) O 49                 -3.96
  45. LP ( 2) O 49                 -0.19
  95. BD ( 1) O 49- H 50           -4.49
  96. BD ( 1) O 49- H 51           -2.86

Steric exchange energy, unit 6:   -11.50 kcal/mol
-------------------------------------------------
```

```
  Total steric exchange energy:  1347.19 kcal/mol
-------------------------------------------------

Pairwise steric exchange energies dE(i,j) (kcal/mol) and associated pre-NLMO
overlaps S(i,j) for disjoint (no common atoms) interactions between NLMOs i,j:

         Threshold for printing:     0.50 kcal/mol
        (Intermolecular threshold:   0.05 kcal/mol)
                                                          PNLMO    dE(i,j)
     NLMO (i)                         NLMO (j)            S(i,j)   kcal/mol
===============================================================================

within unit  1
  25. LP ( 1) N  8              50. BD ( 1) C  2- C  3        0.1205     0.53
  25. LP ( 1) N  8              51. BD ( 1) C  2- C  7        0.1264     3.84
  25. LP ( 1) N  8              54. BD ( 1) C  3- H 36        0.1158     2.12
  25. LP ( 1) N  8              63. BD ( 1) C  7- H 20       -0.1786    14.52
  25. LP ( 1) N  8              66. BD ( 1) C  9- C 14        0.1571     4.85
  25. LP ( 1) N  8              67. BD ( 1) C  9- H 21       -0.1365     8.48
  25. LP ( 1) N  8              70. BD ( 1) C 10- H 23        0.0852     0.63
  25. LP ( 1) N  8              81. BD ( 1) O 16- H 30        0.1478     4.82
  26. LP ( 1) O 13              66. BD ( 1) C  9- C 14       -0.0747     2.58
  26. LP ( 1) O 13              71. BD ( 1) C 11- C 12       -0.0566     1.74
  26. LP ( 1) O 13              75. BD ( 1) C 12- C 17        0.1486     6.54
  26. LP ( 1) O 13              76. BD ( 1) C 12- H 29       -0.0486     1.64
  26. LP ( 1) O 13              78. BD ( 1) C 14- O 16       -0.0607     1.73
  26. LP ( 1) O 13              79. BD ( 1) C 14- H 31        0.1193     5.59
  26. LP ( 1) O 13              83. BD ( 1) C 17- H 27       -0.0411     0.55
  26. LP ( 1) O 13              84. BD ( 1) C 17- H 28        0.0957     0.85
  27. LP ( 2) O 13              30. LP ( 1) O 16             -0.0876     0.64
  27. LP ( 2) O 13              64. BD ( 1) N  8- C  9       -0.0382     0.52
  27. LP ( 2) O 13              66. BD ( 1) C  9- C 14        0.1671     9.28
  27. LP ( 2) O 13              67. BD ( 1) C  9- H 21        0.0916     0.79
  27. LP ( 2) O 13              71. BD ( 1) C 11- C 12        0.1810    10.10
  27. LP ( 2) O 13              73. BD ( 1) C 11- H 25        0.0816     0.54
  27. LP ( 2) O 13              76. BD ( 1) C 12- H 29       -0.1430     9.05
  27. LP ( 2) O 13              78. BD ( 1) C 14- O 16       -0.1691     9.50
  28. LP ( 1) O 15              62. BD ( 2) C  7- N  8        0.0651     0.65
  28. LP ( 1) O 15              63. BD ( 1) C  7- H 20        0.0600     0.76
  28. LP ( 1) O 15              65. BD ( 1) C  9- C 10        0.1570     6.79
  28. LP ( 1) O 15              67. BD ( 1) C  9- H 21        0.0958     0.92
  28. LP ( 1) O 15              68. BD ( 1) C 10- C 11       -0.0369     0.81
  28. LP ( 1) O 15              70. BD ( 1) C 10- H 23       -0.0721     3.15
  29. LP ( 2) O 15              62. BD ( 2) C  7- N  8        0.0699     1.02
  29. LP ( 2) O 15              68. BD ( 1) C 10- C 11       -0.1677     9.08
  29. LP ( 2) O 15              70. BD ( 1) C 10- H 23        0.1504     9.43
  29. LP ( 2) O 15              73. BD ( 1) C 11- H 25       -0.0692     0.64
  30. LP ( 1) O 16              66. BD ( 1) C  9- C 14       -0.0972     3.81
  30. LP ( 1) O 16              76. BD ( 1) C 12- H 29        0.0574     0.73
  30. LP ( 1) O 16              77. BD ( 1) O 13- C 14        0.0869     1.35
  31. LP ( 2) O 16              65. BD ( 1) C  9- C 10        0.0981     0.65
  31. LP ( 2) O 16              66. BD ( 1) C  9- C 14        0.1271     4.60
  31. LP ( 2) O 16              70. BD ( 1) C 10- H 23        0.0643     0.75
  31. LP ( 2) O 16              76. BD ( 1) C 12- H 29        0.0960     2.02
  31. LP ( 2) O 16              77. BD ( 1) O 13- C 14        0.0955     1.96
  31. LP ( 2) O 16              79. BD ( 1) C 14- H 31       -0.1567    11.91
  32. LP ( 1) O 18              75. BD ( 1) C 12- C 17       -0.1268     6.06
  32. LP ( 1) O 18              83. BD ( 1) C 17- H 27        0.0721     1.87
  32. LP ( 1) O 18              84. BD ( 1) C 17- H 28        0.0376     0.50
  33. LP ( 2) O 18              75. BD ( 1) C 12- C 17        0.0888     2.31
  33. LP ( 2) O 18              76. BD ( 1) C 12- H 29        0.0840     0.91
  33. LP ( 2) O 18              83. BD ( 1) C 17- H 27        0.1039     4.25
  33. LP ( 2) O 18              84. BD ( 1) C 17- H 28       -0.1535    11.26
  34. LP ( 1) O 19              71. BD ( 1) C 11- C 12        0.1425     5.38
  34. LP ( 1) O 19              73. BD ( 1) C 11- H 25       -0.0638     2.80
  34. LP ( 1) O 19              83. BD ( 1) C 17- H 27        0.0645     0.72
  35. LP ( 2) O 19              68. BD ( 1) C 10- C 11       -0.2015    13.00
  35. LP ( 2) O 19              70. BD ( 1) C 10- H 23       -0.1042     1.47
  35. LP ( 2) O 19              71. BD ( 1) C 11- C 12        0.0554     1.16
  35. LP ( 2) O 19              73. BD ( 1) C 11- H 25        0.1108     5.79
  35. LP ( 2) O 19              83. BD ( 1) C 17- H 27        0.0640     1.02
  46. BD ( 1) C  1- C  2        52. BD ( 1) C  3- C  4        0.1376     3.92
  46. BD ( 1) C  1- C  2        54. BD ( 1) C  3- H 36       -0.1266     5.16
  46. BD ( 1) C  1- C  2        55. BD ( 1) C  4- C  5        0.1148     0.94
  46. BD ( 1) C  1- C  2        57. BD ( 1) C  5- C  6        0.1485     4.12
  46. BD ( 1) C  1- C  2        60. BD ( 1) C  6- H 33       -0.1253     4.92
```

46. BD (1) C 1- C 2	61. BD (1) C 7- N 8	-0.1110	2.64
46. BD (1) C 1- C 2	63. BD (1) C 7- H 20	0.1286	3.50
47. BD (2) C 1- C 2	53. BD (2) C 3- C 4	0.1976	12.25
47. BD (2) C 1- C 2	58. BD (2) C 5- C 6	-0.1678	8.43
47. BD (2) C 1- C 2	62. BD (2) C 7- N 8	-0.1747	9.15
48. BD (1) C 1- C 6	50. BD (1) C 2- C 3	0.1305	3.71
48. BD (1) C 1- C 6	51. BD (1) C 2- C 7	-0.1262	4.26
48. BD (1) C 1- C 6	52. BD (1) C 3- C 4	0.1180	0.87
48. BD (1) C 1- C 6	55. BD (1) C 4- C 5	0.1524	4.22
48. BD (1) C 1- C 6	59. BD (1) C 5- H 34	-0.1211	4.78
49. BD (1) C 1- H 32	50. BD (1) C 2- C 3	-0.1270	5.23
49. BD (1) C 1- H 32	51. BD (1) C 2- C 7	0.1214	3.58
49. BD (1) C 1- H 32	57. BD (1) C 5- C 6	-0.1245	4.99
49. BD (1) C 1- H 32	60. BD (1) C 6- H 33	0.0987	2.94
49. BD (1) C 1- H 32	63. BD (1) C 7- H 20	0.1140	1.41
50. BD (1) C 2- C 3	55. BD (1) C 4- C 5	0.1431	4.04
50. BD (1) C 2- C 3	56. BD (1) C 4- H 35	-0.1250	5.00
50. BD (1) C 2- C 3	57. BD (1) C 5- C 6	0.1098	0.88
50. BD (1) C 2- C 3	61. BD (1) C 7- N 8	0.0941	2.05
50. BD (1) C 2- C 3	63. BD (1) C 7- H 20	-0.0974	3.46
51. BD (1) C 2- C 7	52. BD (1) C 3- C 4	-0.1135	3.62
51. BD (1) C 2- C 7	54. BD (1) C 3- H 36	0.1158	3.29
51. BD (1) C 2- C 7	64. BD (1) N 8- C 9	-0.1180	4.77
52. BD (1) C 3- C 4	57. BD (1) C 5- C 6	0.1498	4.06
52. BD (1) C 3- C 4	59. BD (1) C 5- H 34	-0.1198	4.70
53. BD (2) C 3- C 4	58. BD (2) C 5- C 6	-0.1660	7.94
53. BD (2) C 3- C 4	62. BD (2) C 7- N 8	-0.0685	1.04
54. BD (1) C 3- H 36	55. BD (1) C 4- C 5	-0.1222	4.94
54. BD (1) C 3- H 36	56. BD (1) C 4- H 35	0.0998	3.01
54. BD (1) C 3- H 36	61. BD (1) C 7- N 8	0.0777	0.54
55. BD (1) C 4- C 5	60. BD (1) C 6- H 33	-0.1233	4.92
56. BD (1) C 4- H 35	57. BD (1) C 5- C 6	-0.1246	4.92
56. BD (1) C 4- H 35	59. BD (1) C 5- H 34	0.0961	2.88
58. BD (2) C 5- C 6	62. BD (2) C 7- N 8	-0.0493	1.01
59. BD (1) C 5- H 34	60. BD (1) C 6- H 33	0.0968	2.91
61. BD (1) C 7- N 8	66. BD (1) C 9- C 14	-0.0604	1.24
61. BD (1) C 7- N 8	67. BD (1) C 9- H 21	0.1254	2.72
62. BD (2) C 7- N 8	65. BD (1) C 9- C 10	0.1920	11.07
62. BD (2) C 7- N 8	66. BD (1) C 9- C 14	-0.1152	3.81
62. BD (2) C 7- N 8	67. BD (1) C 9- H 21	-0.0562	1.30
62. BD (2) C 7- N 8	69. BD (1) C 10- O 15	0.0767	0.56
62. BD (2) C 7- N 8	70. BD (1) C 10- H 23	0.0851	1.00
63. BD (1) C 7- H 20	64. BD (1) N 8- C 9	0.1429	5.57
63. BD (1) C 7- H 20	67. BD (1) C 9- H 21	0.1272	2.49
64. BD (1) N 8- C 9	68. BD (1) C 10- C 11	-0.1232	3.92
64. BD (1) N 8- C 9	69. BD (1) C 10- O 15	0.0564	0.68
64. BD (1) N 8- C 9	70. BD (1) C 10- H 23	0.1112	2.34
64. BD (1) N 8- C 9	77. BD (1) O 13- C 14	-0.1167	3.34
64. BD (1) N 8- C 9	78. BD (1) C 14- O 16	0.0740	1.18
64. BD (1) N 8- C 9	81. BD (1) O 16- H 30	0.0931	1.19
65. BD (1) C 9- C 10	71. BD (1) C 11- C 12	0.1256	3.46
65. BD (1) C 9- C 10	72. BD (1) C 11- O 19	-0.1303	4.14
65. BD (1) C 9- C 10	73. BD (1) C 11- H 25	0.0697	0.51
65. BD (1) C 9- C 10	74. BD (1) C 12- O 13	0.1105	0.99
65. BD (1) C 9- C 10	77. BD (1) O 13- C 14	0.1064	2.15
65. BD (1) C 9- C 10	78. BD (1) C 14- O 16	0.0577	0.52
65. BD (1) C 9- C 10	79. BD (1) C 14- H 31	-0.1083	4.15
65. BD (1) C 9- C 10	80. BD (1) O 15- H 22	-0.1022	4.41
66. BD (1) C 9- C 14	68. BD (1) C 10- C 11	0.1161	2.88
66. BD (1) C 9- C 14	69. BD (1) C 10- O 15	-0.1312	4.42
66. BD (1) C 9- C 14	71. BD (1) C 11- C 12	0.1138	1.39
66. BD (1) C 9- C 14	74. BD (1) C 12- O 13	0.1183	3.03
66. BD (1) C 9- C 14	81. BD (1) O 16- H 30	0.1255	5.07
67. BD (1) C 9- H 21	68. BD (1) C 10- C 11	0.0740	0.79
67. BD (1) C 9- H 21	69. BD (1) C 10- O 15	0.0949	1.72
67. BD (1) C 9- H 21	70. BD (1) C 10- H 23	-0.1165	5.40
67. BD (1) C 9- H 21	73. BD (1) C 11- H 25	0.0713	0.51
67. BD (1) C 9- H 21	78. BD (1) C 14- O 16	-0.1102	4.03
67. BD (1) C 9- H 21	79. BD (1) C 14- H 31	0.0985	2.36
68. BD (1) C 10- C 11	74. BD (1) C 12- O 13	0.0949	1.74
68. BD (1) C 10- C 11	75. BD (1) C 12- C 17	-0.1183	3.72
68. BD (1) C 10- C 11	76. BD (1) C 12- H 29	0.1067	1.93
68. BD (1) C 10- C 11	77. BD (1) O 13- C 14	0.0903	0.63
68. BD (1) C 10- C 11	80. BD (1) O 15- H 22	0.1132	3.75
68. BD (1) C 10- C 11	86. BD (1) O 19- H 24	0.0546	0.80
69. BD (1) C 10- O 15	71. BD (1) C 11- C 12	-0.1314	4.03

```
  69. BD ( 1) C 10- O 15        73. BD ( 1) C 11- H 25        0.0765     1.03
  70. BD ( 1) C 10- H 23        71. BD ( 1) C 11- C 12        0.0874     1.06
  70. BD ( 1) C 10- H 23        72. BD ( 1) C 11- O 19        0.0911     1.63
  70. BD ( 1) C 10- H 23        73. BD ( 1) C 11- H 25       -0.1084     5.05
  70. BD ( 1) C 10- H 23        76. BD ( 1) C 12- H 29        0.1022     1.13
  71. BD ( 1) C 11- C 12        77. BD ( 1) O 13- C 14        0.1044     2.30
  71. BD ( 1) C 11- C 12        82. BD ( 1) C 17- O 18       -0.1119     3.30
  71. BD ( 1) C 11- C 12        83. BD ( 1) C 17- H 27        0.1072     2.39
  71. BD ( 1) C 11- C 12        84. BD ( 1) C 17- H 28        0.0643     0.56
  71. BD ( 1) C 11- C 12        86. BD ( 1) O 19- H 24       -0.1040     4.46
  72. BD ( 1) C 11- O 19        74. BD ( 1) C 12- O 13       -0.1200     3.67
  72. BD ( 1) C 11- O 19        75. BD ( 1) C 12- C 17        0.0546     0.79
  72. BD ( 1) C 11- O 19        76. BD ( 1) C 12- H 29        0.0751     0.94
  72. BD ( 1) C 11- O 19        83. BD ( 1) C 17- H 27        0.0722     0.56
  73. BD ( 1) C 11- H 25        75. BD ( 1) C 12- C 17        0.1007     2.11
  73. BD ( 1) C 11- H 25        76. BD ( 1) C 12- H 29       -0.1166     5.58
  73. BD ( 1) C 11- H 25        84. BD ( 1) C 17- H 28        0.0723     0.51
  73. BD ( 1) C 11- H 25        86. BD ( 1) O 19- H 24        0.0956     2.88
  74. BD ( 1) C 12- O 13        78. BD ( 1) C 14- O 16        0.0714     0.97
  74. BD ( 1) C 12- O 13        79. BD ( 1) C 14- H 31       -0.0865     4.00
  74. BD ( 1) C 12- O 13        83. BD ( 1) C 17- H 27       -0.1283     4.87
  74. BD ( 1) C 12- O 13        84. BD ( 1) C 17- H 28        0.1048     1.95
  75. BD ( 1) C 12- C 17        77. BD ( 1) O 13- C 14       -0.0941     3.10
  75. BD ( 1) C 12- C 17        85. BD ( 1) O 18- H 26        0.1230     4.63
  76. BD ( 1) C 12- H 29        77. BD ( 1) O 13- C 14        0.0981     1.92
  76. BD ( 1) C 12- H 29        78. BD ( 1) C 14- O 16        0.0712     0.79
  76. BD ( 1) C 12- H 29        82. BD ( 1) C 17- O 18        0.0989     1.97
  76. BD ( 1) C 12- H 29        84. BD ( 1) C 17- H 28       -0.1086     4.82
  77. BD ( 1) O 13- C 14        81. BD ( 1) O 16- H 30       -0.1060     4.28
  83. BD ( 1) C 17- H 27        85. BD ( 1) O 18- H 26       -0.0957     4.92

                                                   sum within unit 1:    593.22

between units  1 and  2
  26. LP ( 1) O 13              37. LP ( 2) O 38              0.0345     0.17
  27. LP ( 2) O 13              37. LP ( 2) O 38             -0.0380     0.41
  27. LP ( 2) O 13              88. BD ( 1) O 38- H 39       -0.0398     0.43
  30. LP ( 1) O 16              36. LP ( 1) O 38             -0.0138     0.18
  30. LP ( 1) O 16              87. BD ( 1) H 37- O 38        0.0172    -0.07
  30. LP ( 1) O 16              88. BD ( 1) O 38- H 39        0.1267     4.70
  32. LP ( 1) O 18              37. LP ( 2) O 38              0.0098    -0.13
  33. LP ( 2) O 18              37. LP ( 2) O 38              0.0491     0.42
  33. LP ( 2) O 18              87. BD ( 1) H 37- O 38        0.0289     0.21
  36. LP ( 1) O 38              76. BD ( 1) C 12- H 29       -0.0297     0.16
  37. LP ( 2) O 38              71. BD ( 1) C 11- C 12       -0.0237     0.09
  37. LP ( 2) O 38              76. BD ( 1) C 12- H 29        0.0428     0.38
  37. LP ( 2) O 38              82. BD ( 1) C 17- O 18        0.0365    -0.31
  37. LP ( 2) O 38              85. BD ( 1) O 18- H 26        0.1654     7.76
  76. BD ( 1) C 12- H 29        87. BD ( 1) H 37- O 38        0.0253     0.21
  76. BD ( 1) C 12- H 29        88. BD ( 1) O 38- H 39        0.0330     0.29
  78. BD ( 1) C 14- O 16        88. BD ( 1) O 38- H 39        0.0203     0.06
  81. BD ( 1) O 16- H 30        88. BD ( 1) O 38- H 39       -0.0330     0.33
  85. BD ( 1) O 18- H 26        87. BD ( 1) H 37- O 38        0.0321     0.36

                                          sum between units 1 and 2:     15.94

between units  1 and  3
  28. LP ( 1) O 15              39. LP ( 2) O 41              0.0133    -0.20
  28. LP ( 1) O 15              89. BD ( 1) H 40- O 41       -0.0107     0.07
  29. LP ( 2) O 15              38. LP ( 1) O 41             -0.0185     0.09
  34. LP ( 1) O 19              38. LP ( 1) O 41              0.0196    -0.15
  35. LP ( 2) O 19              38. LP ( 1) O 41             -0.0175     0.11
  35. LP ( 2) O 19              39. LP ( 2) O 41             -0.0324     0.24
  35. LP ( 2) O 19              89. BD ( 1) H 40- O 41       -0.0289     0.22
  35. LP ( 2) O 19              90. BD ( 1) O 41- H 42        0.0121     0.07
  38. LP ( 1) O 41              72. BD ( 1) C 11- O 19        0.0250    -0.15
  38. LP ( 1) O 41              80. BD ( 1) O 15- H 22       -0.0293     0.24
  38. LP ( 1) O 41              86. BD ( 1) O 19- H 24        0.1246     4.78
  39. LP ( 2) O 41              69. BD ( 1) C 10- O 15        0.0475    -0.35
  39. LP ( 2) O 41              80. BD ( 1) O 15- H 22        0.1921    10.61
  39. LP ( 2) O 41              86. BD ( 1) O 19- H 24        0.0866     2.09
  80. BD ( 1) O 15- H 22        89. BD ( 1) H 40- O 41        0.0416     0.56
  80. BD ( 1) O 15- H 22        90. BD ( 1) O 41- H 42       -0.0287     0.20
  86. BD ( 1) O 19- H 24        89. BD ( 1) H 40- O 41        0.0382     0.53
  86. BD ( 1) O 19- H 24        90. BD ( 1) O 41- H 42       -0.0426     0.53
```

```
                                           sum between units 1 and 3:     19.71

between units  1 and  4
  34. LP ( 1) O 19            92. BD ( 1) O 43- H 45        0.0421     0.56
  35. LP ( 2) O 19            40. LP ( 1) O 43              0.0185     0.19
  35. LP ( 2) O 19            41. LP ( 2) O 43             -0.0367     0.22
  35. LP ( 2) O 19            92. BD ( 1) O 43- H 45       -0.0731     1.58
  40. LP ( 1) O 43            70. BD ( 1) C 10- H 23       -0.0356     0.30
  40. LP ( 1) O 43            80. BD ( 1) O 15- H 22       -0.0254     0.07
  41. LP ( 2) O 43            80. BD ( 1) O 15- H 22        0.0304     0.11
  41. LP ( 2) O 43            86. BD ( 1) O 19- H 24        0.0253     0.12
  65. BD ( 1) C  9- C 10      92. BD ( 1) O 43- H 45       -0.0161     0.09
  70. BD ( 1) C 10- H 23      91. BD ( 1) O 43- H 44        0.0365     0.40
  70. BD ( 1) C 10- H 23      92. BD ( 1) O 43- H 45        0.0440     0.61
  86. BD ( 1) O 19- H 24      92. BD ( 1) O 43- H 45        0.0153     0.08

                                           sum between units 1 and 4:      4.50

between units  1 and  5
  25. LP ( 1) N  8            42. LP ( 1) O 47             -0.0162     0.09
  25. LP ( 1) N  8            94. BD ( 1) O 47- H 48        0.0295     0.20
  28. LP ( 1) O 15            93. BD ( 1) H 46- O 47        0.0251     0.12
  29. LP ( 2) O 15            42. LP ( 1) O 47             -0.0103     0.12
  29. LP ( 2) O 15            43. LP ( 2) O 47              0.0318    -0.09
  29. LP ( 2) O 15            93. BD ( 1) H 46- O 47        0.1542     6.77
  29. LP ( 2) O 15            94. BD ( 1) O 47- H 48        0.0133    -0.13
  42. LP ( 1) O 47            62. BD ( 2) C  7- N  8       -0.0260     0.21
  42. LP ( 1) O 47            70. BD ( 1) C 10- H 23       -0.0470     0.46
  43. LP ( 2) O 47            47. BD ( 2) C  1- C  2        0.0206     0.09
  43. LP ( 2) O 47            70. BD ( 1) C 10- H 23        0.0559     0.88
  47. BD ( 2) C  1- C  2      93. BD ( 1) H 46- O 47       -0.0154     0.06
  47. BD ( 2) C  1- C  2      94. BD ( 1) O 47- H 48       -0.0292     0.25
  53. BD ( 2) C  3- C  4      94. BD ( 1) O 47- H 48       -0.0274     0.25
  54. BD ( 1) C  3- H 36      94. BD ( 1) O 47- H 48        0.0160     0.07
  58. BD ( 2) C  5- C  6      94. BD ( 1) O 47- H 48       -0.0131     0.07
  62. BD ( 2) C  7- N  8      93. BD ( 1) H 46- O 47        0.0597     0.92
  62. BD ( 2) C  7- N  8      94. BD ( 1) O 47- H 48        0.0447     0.59
  68. BD ( 1) C 10- C 11      93. BD ( 1) H 46- O 47       -0.0233     0.12
  69. BD ( 1) C 10- O 15      93. BD ( 1) H 46- O 47        0.0474     0.40
  70. BD ( 1) C 10- H 23      93. BD ( 1) H 46- O 47        0.0605     1.00
  70. BD ( 1) C 10- H 23      94. BD ( 1) O 47- H 48        0.0128     0.08
  80. BD ( 1) O 15- H 22      93. BD ( 1) H 46- O 47        0.0218     0.11

                                           sum between units 1 and 5:     13.12

between units  1 and  6
  32. LP ( 1) O 18            44. LP ( 1) O 49             -0.0088     0.05
  32. LP ( 1) O 18            95. BD ( 1) O 49- H 50        0.0321     0.30
  33. LP ( 2) O 18            44. LP ( 1) O 49              0.0220    -0.15
  33. LP ( 2) O 18            45. LP ( 2) O 49              0.0249    -0.09
  33. LP ( 2) O 18            95. BD ( 1) O 49- H 50        0.1790     9.24
  44. LP ( 1) O 49            76. BD ( 1) C 12- H 29        0.0563     0.90
  45. LP ( 2) O 49            76. BD ( 1) C 12- H 29        0.0111     0.05
  71. BD ( 1) C 11- C 12      95. BD ( 1) O 49- H 50       -0.0167     0.06
  75. BD ( 1) C 12- C 17      95. BD ( 1) O 49- H 50        0.0152     0.06
  76. BD ( 1) C 12- H 29      95. BD ( 1) O 49- H 50        0.0432     0.51
  76. BD ( 1) C 12- H 29      96. BD ( 1) O 49- H 51       -0.0249     0.11
  82. BD ( 1) C 17- O 18      95. BD ( 1) O 49- H 50        0.0233     0.09
  83. BD ( 1) C 17- H 27      95. BD ( 1) O 49- H 50        0.0189     0.08
  85. BD ( 1) O 18- H 26      95. BD ( 1) O 49- H 50        0.0378     0.39

                                           sum between units 1 and 6:     11.84

within unit  2

                                                   sum within unit 2:      0.00

between units  2 and  3

                                           sum between units 2 and 3:      0.00

between units  2 and  4

                                           sum between units 2 and 4:      0.00

between units  2 and  5
```

```
                                          sum between units 2 and 5:      0.00

between units  2 and  6
  36. LP ( 1) O 38            44. LP ( 1) O 49            -0.0165     0.08
  36. LP ( 1) O 38            95. BD ( 1) O 49- H 50      -0.0152     0.07
  37. LP ( 2) O 38            95. BD ( 1) O 49- H 50       0.0241     0.17
  45. LP ( 2) O 49            87. BD ( 1) H 37- O 38       0.1328     5.60
  45. LP ( 2) O 49            88. BD ( 1) O 38- H 39       0.0159    -0.13
  87. BD ( 1) H 37- O 38      95. BD ( 1) O 49- H 50       0.0409     0.64
  87. BD ( 1) H 37- O 38      96. BD ( 1) O 49- H 51      -0.0298     0.27

                                          sum between units 2 and 6:      6.86

within unit  3

                                                  sum within unit 3:      0.00

between units  3 and  4
  38. LP ( 1) O 41            41. LP ( 2) O 43             0.0143    -0.11
  38. LP ( 1) O 41            91. BD ( 1) O 43- H 44      -0.0154     0.07
  39. LP ( 2) O 41            40. LP ( 1) O 43            -0.0273     0.22
  39. LP ( 2) O 41            41. LP ( 2) O 43             0.0428    -0.34
  41. LP ( 2) O 43            89. BD ( 1) H 40- O 41       0.2279    16.32
  41. LP ( 2) O 43            90. BD ( 1) O 41- H 42       0.0098    -0.18
  89. BD ( 1) H 40- O 41      92. BD ( 1) O 43- H 45       0.0256     0.29
  90. BD ( 1) O 41- H 42      92. BD ( 1) O 43- H 45      -0.0114     0.07

                                          sum between units 3 and 4:     16.45

between units  3 and  5

                                          sum between units 3 and 5:      0.03

between units  3 and  6

                                          sum between units 3 and 6:      0.00

within unit  4

                                                  sum within unit 4:      0.00

between units  4 and  5
  40. LP ( 1) O 43            43. LP ( 2) O 47            -0.0113     0.17
  40. LP ( 1) O 43            93. BD ( 1) H 46- O 47      -0.0190     0.10
  41. LP ( 2) O 43            43. LP ( 2) O 47             0.0247    -0.10
  42. LP ( 1) O 47            91. BD ( 1) O 43- H 44       0.0129     0.09
  43. LP ( 2) O 47            91. BD ( 1) O 43- H 44       0.1599     7.67
  43. LP ( 2) O 47            92. BD ( 1) O 43- H 45       0.0213    -0.17
  91. BD ( 1) O 43- H 44      93. BD ( 1) H 46- O 47       0.0235     0.22
  91. BD ( 1) O 43- H 44      94. BD ( 1) O 47- H 48      -0.0317     0.26

                                          sum between units 4 and 5:      8.36

between units  4 and  6

                                          sum between units 4 and 6:      0.00

within unit  5

                                                  sum within unit 5:      0.00

between units  5 and  6

                                          sum between units 5 and 6:      0.00

within unit  6

                                                  sum within unit 6:      0.00

-------------------------------------------------------------------------------
      Total disjoint NLMO steric exchange energy from pairwise sum:    690.03
-------------------------------
```

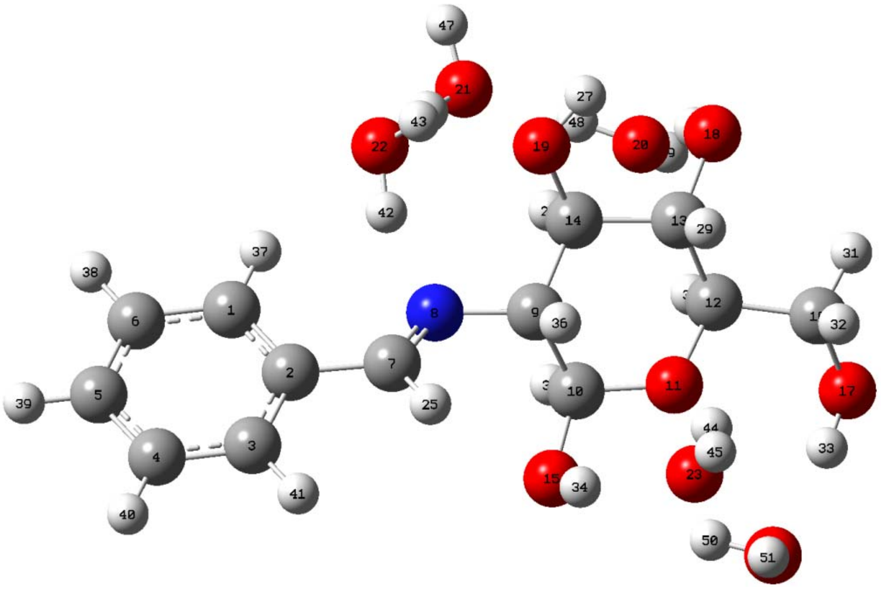

Table S35. NBO/NLMO STERIC ANALYSIS **11.5H$_2$O**:

```
Occupied NLMO contributions dE(i) (kcal/mol) to total steric exchange energy

NLMOs (i) in unit  1                  dE(i)
   1. CR ( 1) C  1                    -0.02
   2. CR ( 1) C  2                    -0.02
   3. CR ( 1) C  3                    -0.03
   4. CR ( 1) C  4                    -0.03
   5. CR ( 1) C  5                    -0.02
   6. CR ( 1) C  6                    -0.03
   7. CR ( 1) C  7                    -0.02
   8. CR ( 1) N  8                    -0.02
   9. CR ( 1) C  9                    -0.02
  10. CR ( 1) C 10                    -0.02
  11. CR ( 1) O 11                    -0.02
  12. CR ( 1) C 12                    -0.02
  13. CR ( 1) C 13                    -0.02
  14. CR ( 1) C 14                    -0.02
  15. CR ( 1) O 15                    -0.01
  16. CR ( 1) C 16                    -0.02
  17. CR ( 1) O 17                    -0.01
  18. CR ( 1) O 18                    -0.01
  19. CR ( 1) O 19                    -0.01
  25. LP ( 1) N  8                    33.92
  26. LP ( 1) O 11                    24.47
  27. LP ( 2) O 11                    22.49
  28. LP ( 1) O 15                     7.99
  29. LP ( 2) O 15                     8.22
  30. LP ( 1) O 17                     9.19
  31. LP ( 2) O 17                     5.85
  32. LP ( 1) O 18                     8.20
  33. LP ( 2) O 18                    10.09
  34. LP ( 1) O 19                    11.86
  35. LP ( 2) O 19                     7.21
  46. BD ( 1) C  1- C  2              77.66
  47. BD ( 1) C  1- C  6              71.69
  48. BD ( 2) C  1- C  6             -17.87
  49. BD ( 1) C  1- H 37               4.79
  50. BD ( 1) C  2- C  3              78.37
  51. BD ( 2) C  2- C  3             -18.42
  52. BD ( 1) C  2- C  7              62.91
  53. BD ( 1) C  3- C  4              72.04
  54. BD ( 1) C  3- H 41               4.68
  55. BD ( 1) C  4- C  5              69.44
  56. BD ( 2) C  4- C  5             -18.18
  57. BD ( 1) C  4- H 40               1.15
```

```
  58. BD ( 1) C  5- C  6           68.28
  59. BD ( 1) C  5- H 39            2.00
  60. BD ( 1) C  6- H 38            1.07
  61. BD ( 1) C  7- N  8           65.31
  62. BD ( 2) C  7- N  8           -9.78
  63. BD ( 1) C  7- H 25            6.14
  64. BD ( 1) N  8- C  9           57.06
  65. BD ( 1) C  9- C 10           62.62
  66. BD ( 1) C  9- C 14           71.23
  67. BD ( 1) C  9- H 36            9.31
  68. BD ( 1) C 10- O 11           49.42
  69. BD ( 1) C 10- O 15           42.83
  70. BD ( 1) C 10- H 35           -2.26
  71. BD ( 1) O 11- C 12           41.76
  72. BD ( 1) C 12- C 13           64.21
  73. BD ( 1) C 12- C 16           57.91
  74. BD ( 1) C 12- H 30            6.64
  75. BD ( 1) C 13- C 14           71.22
  76. BD ( 1) C 13- O 18           44.52
  77. BD ( 1) C 13- H 29            7.04
  78. BD ( 1) C 14- O 19           40.16
  79. BD ( 1) C 14- H 26            9.92
  80. BD ( 1) O 15- H 34            6.35
  81. BD ( 1) C 16- O 17           30.05
  82. BD ( 1) C 16- H 31            2.52
  83. BD ( 1) C 16- H 32            3.62
  84. BD ( 1) O 17- H 33            5.12
  85. BD ( 1) O 18- H 28            6.15
  86. BD ( 1) O 19- H 27            4.81

Steric exchange energy, unit 1:  1362.61 kcal/mol

NLMOs (i) in unit  2               dE(i)
  20. CR ( 1) O 20                 -0.01
  36. LP ( 1) O 20                 -5.38
  37. LP ( 2) O 20                  7.96
  87. BD ( 1) O 20- H 48           -7.96
  88. BD ( 1) O 20- H 49           -3.21

Steric exchange energy, unit 2:    -8.60 kcal/mol

NLMOs (i) in unit  3               dE(i)
  21. CR ( 1) O 21                  0.00
  38. LP ( 1) O 21                 -5.06
  39. LP ( 2) O 21                  7.88
  89. BD ( 1) O 21- H 46           -7.74
  90. BD ( 1) O 21- H 47           -1.89

Steric exchange energy, unit 3:    -6.82 kcal/mol

NLMOs (i) in unit  4               dE(i)
  22. CR ( 1) O 22                  0.00
  40. LP ( 1) O 22                 -1.57
  41. LP ( 2) O 22                  6.36
  91. BD ( 1) O 22- H 42           -0.89
  92. BD ( 1) O 22- H 43           -1.39

Steric exchange energy, unit 4:     2.51 kcal/mol

NLMOs (i) in unit  5               dE(i)
  23. CR ( 1) O 23                  0.00
  42. LP ( 1) O 23                 -0.76
  43. LP ( 2) O 23                 -1.47
  93. BD ( 1) O 23- H 44           -4.04
  94. BD ( 1) O 23- H 45           -4.20

Steric exchange energy, unit 5:   -10.46 kcal/mol

NLMOs (i) in unit  6               dE(i)
  24. CR ( 1) O 24                  0.01
  44. LP ( 1) O 24                 -2.26
  45. LP ( 2) O 24                  0.55
  95. BD ( 1) O 24- H 50           -3.74
  96. BD ( 1) O 24- H 51           -3.43

Steric exchange energy, unit 6:    -8.86 kcal/mol
```

```
 ------------------------------------------------
   Total steric exchange energy:  1330.38 kcal/mol
 ------------------------------------------------

 Pairwise steric exchange energies dE(i,j) (kcal/mol) and associated pre-NLMO
 overlaps S(i,j) for disjoint (no common atoms) interactions between NLMOs i,j:

          Threshold for printing:     0.50 kcal/mol
         (Intermolecular threshold:   0.05 kcal/mol)
                                                              PNLMO    dE(i,j)
      NLMO (i)                         NLMO (j)              S(i,j)   kcal/mol
 ==============================================================================

 within unit  1
   25. LP ( 1) N  8             49. BD ( 1) C  1- H 37        0.1052     1.52
   25. LP ( 1) N  8             52. BD ( 1) C  2- C  7        0.1182     3.54
   25. LP ( 1) N  8             63. BD ( 1) C  7- H 25       -0.1787    14.54
   25. LP ( 1) N  8             66. BD ( 1) C  9- C 14        0.1618     5.44
   25. LP ( 1) N  8             67. BD ( 1) C  9- H 36       -0.1247     7.31
   25. LP ( 1) N  8             79. BD ( 1) C 14- H 26        0.1184     1.04
   26. LP ( 1) O 11             29. LP ( 2) O 15             -0.1068     2.04
   26. LP ( 1) O 11             65. BD ( 1) C  9- C 10       -0.0794     2.94
   26. LP ( 1) O 11             66. BD ( 1) C  9- C 14       -0.0623     0.63
   26. LP ( 1) O 11             69. BD ( 1) C 10- O 15        0.1207     3.58
   26. LP ( 1) O 11             72. BD ( 1) C 12- C 13       -0.0948     3.92
   26. LP ( 1) O 11             73. BD ( 1) C 12- C 16        0.1345     5.92
   26. LP ( 1) O 11             82. BD ( 1) C 16- H 31       -0.0432     0.57
   26. LP ( 1) O 11             84. BD ( 1) O 17- H 33        0.0590     0.51
   27. LP ( 2) O 11             65. BD ( 1) C  9- C 10        0.1768     9.19
   27. LP ( 2) O 11             67. BD ( 1) C  9- H 36        0.1052     1.14
   27. LP ( 2) O 11             70. BD ( 1) C 10- H 35       -0.1360     9.53
   27. LP ( 2) O 11             72. BD ( 1) C 12- C 13        0.1737     8.54
   27. LP ( 2) O 11             74. BD ( 1) C 12- H 30       -0.1375     9.48
   27. LP ( 2) O 11             77. BD ( 1) C 13- H 29        0.0972     0.96
   27. LP ( 2) O 11             83. BD ( 1) C 16- H 32        0.0714     0.57
   28. LP ( 1) O 15             65. BD ( 1) C  9- C 10       -0.0763     2.46
   28. LP ( 1) O 15             68. BD ( 1) C 10- O 11       -0.0623     1.71
   28. LP ( 1) O 15             70. BD ( 1) C 10- H 35        0.1025     4.18
   29. LP ( 2) O 15             62. BD ( 2) C  7- N  8        0.0764     0.93
   29. LP ( 2) O 15             63. BD ( 1) C  7- H 25        0.0597     0.70
   29. LP ( 2) O 15             65. BD ( 1) C  9- C 10        0.1569     8.70
   29. LP ( 2) O 15             68. BD ( 1) C 10- O 11       -0.1794    10.05
   30. LP ( 1) O 17             82. BD ( 1) C 16- H 31        0.1025     4.08
   30. LP ( 1) O 17             83. BD ( 1) C 16- H 32       -0.0714     3.01
   31. LP ( 2) O 17             73. BD ( 1) C 12- C 16       -0.1974    12.36
   31. LP ( 2) O 17             74. BD ( 1) C 12- H 30       -0.0941     0.93
   31. LP ( 2) O 17             82. BD ( 1) C 16- H 31        0.0331     0.58
   31. LP ( 2) O 17             83. BD ( 1) C 16- H 32        0.1147     6.67
   32. LP ( 1) O 18             72. BD ( 1) C 12- C 13       -0.0555     1.39
   32. LP ( 1) O 18             75. BD ( 1) C 13- C 14       -0.0587     1.56
   32. LP ( 1) O 18             77. BD ( 1) C 13- H 29        0.1170     5.01
   33. LP ( 2) O 18             72. BD ( 1) C 12- C 13       -0.1752     9.98
   33. LP ( 2) O 18             75. BD ( 1) C 13- C 14        0.1888    11.87
   33. LP ( 2) O 18             82. BD ( 1) C 16- H 31       -0.0641     0.78
   33. LP ( 2) O 18             86. BD ( 1) O 19- H 27        0.1002     2.08
   34. LP ( 1) O 19             66. BD ( 1) C  9- C 14        0.1358     4.81
   34. LP ( 1) O 19             75. BD ( 1) C 13- C 14       -0.0624     1.78
   34. LP ( 1) O 19             79. BD ( 1) C 14- H 26       -0.0456     1.44
   35. LP ( 2) O 19             66. BD ( 1) C  9- C 14       -0.0552     0.85
   35. LP ( 2) O 19             75. BD ( 1) C 13- C 14       -0.1493     7.08
   35. LP ( 2) O 19             77. BD ( 1) C 13- H 29       -0.0780     0.80
   35. LP ( 2) O 19             79. BD ( 1) C 14- H 26        0.1599    11.18
   46. BD ( 1) C  1- C  2       53. BD ( 1) C  3- C  4        0.1238     3.45
   46. BD ( 1) C  1- C  2       54. BD ( 1) C  3- H 41       -0.1271     5.23
   46. BD ( 1) C  1- C  2       55. BD ( 1) C  4- C  5        0.1238     0.95
   46. BD ( 1) C  1- C  2       58. BD ( 1) C  5- C  6        0.1407     3.91
   46. BD ( 1) C  1- C  2       60. BD ( 1) C  6- H 38       -0.1240     4.95
   46. BD ( 1) C  1- C  2       61. BD ( 1) C  7- N  8        0.0861     1.69
   46. BD ( 1) C  1- C  2       63. BD ( 1) C  7- H 25       -0.0965     3.32
   47. BD ( 1) C  1- C  6       50. BD ( 1) C  2- C  3        0.1288     3.76
   47. BD ( 1) C  1- C  6       52. BD ( 1) C  2- C  7       -0.1262     4.01
   47. BD ( 1) C  1- C  6       53. BD ( 1) C  3- C  4        0.1029     0.77
   47. BD ( 1) C  1- C  6       55. BD ( 1) C  4- C  5        0.1426     3.89
   47. BD ( 1) C  1- C  6       59. BD ( 1) C  5- H 39       -0.1279     5.03
   48. BD ( 2) C  1- C  6       51. BD ( 2) C  2- C  3        0.1968    11.93
   48. BD ( 2) C  1- C  6       56. BD ( 2) C  4- C  5       -0.1661     8.05
```

48. BD (2) C 1- C 6	62. BD (2) C 7- N 8	0.0611	0.85
49. BD (1) C 1- H 37	50. BD (1) C 2- C 3	-0.1279	5.13
49. BD (1) C 1- H 37	52. BD (1) C 2- C 7	0.1147	3.23
49. BD (1) C 1- H 37	58. BD (1) C 5- C 6	-0.1202	4.78
49. BD (1) C 1- H 37	60. BD (1) C 6- H 38	0.0999	3.07
50. BD (1) C 2- C 3	55. BD (1) C 4- C 5	0.1432	3.99
50. BD (1) C 2- C 3	57. BD (1) C 4- H 40	-0.1247	4.93
50. BD (1) C 2- C 3	58. BD (1) C 5- C 6	0.1261	1.04
50. BD (1) C 2- C 3	61. BD (1) C 7- N 8	-0.1064	2.42
50. BD (1) C 2- C 3	63. BD (1) C 7- H 25	0.1283	3.53
51. BD (2) C 2- C 3	56. BD (2) C 4- C 5	-0.1685	8.65
51. BD (2) C 2- C 3	62. BD (2) C 7- N 8	0.1670	8.36
52. BD (1) C 2- C 7	53. BD (1) C 3- C 4	-0.1271	4.25
52. BD (1) C 2- C 7	54. BD (1) C 3- H 41	0.1230	3.62
52. BD (1) C 2- C 7	64. BD (1) N 8- C 9	-0.1169	4.67
53. BD (1) C 3- C 4	58. BD (1) C 5- C 6	0.1435	4.00
53. BD (1) C 3- C 4	59. BD (1) C 5- H 39	-0.1302	5.15
54. BD (1) C 3- H 41	55. BD (1) C 4- C 5	-0.1220	4.92
54. BD (1) C 3- H 41	57. BD (1) C 4- H 40	0.0967	2.88
54. BD (1) C 3- H 41	63. BD (1) C 7- H 25	0.1133	1.47
55. BD (1) C 4- C 5	60. BD (1) C 6- H 38	-0.1220	4.83
56. BD (2) C 4- C 5	62. BD (2) C 7- N 8	0.0464	0.90
57. BD (1) C 4- H 40	58. BD (1) C 5- C 6	-0.1232	4.95
57. BD (1) C 4- H 40	59. BD (1) C 5- H 39	0.0975	2.92
59. BD (1) C 5- H 39	60. BD (1) C 6- H 38	0.0978	2.93
61. BD (1) C 7- N 8	66. BD (1) C 9- C 14	-0.0617	1.28
61. BD (1) C 7- N 8	67. BD (1) C 9- H 36	0.1292	2.99
62. BD (2) C 7- N 8	65. BD (1) C 9- C 10	0.1982	11.04
62. BD (2) C 7- N 8	66. BD (1) C 9- C 14	-0.0907	2.54
62. BD (2) C 7- N 8	67. BD (1) C 9- H 36	-0.0750	2.26
63. BD (1) C 7- H 25	64. BD (1) N 8- C 9	0.1481	5.88
63. BD (1) C 7- H 25	67. BD (1) C 9- H 36	0.1343	2.81
64. BD (1) N 8- C 9	68. BD (1) C 10- O 11	-0.1192	3.46
64. BD (1) N 8- C 9	70. BD (1) C 10- H 35	0.0784	0.90
64. BD (1) N 8- C 9	75. BD (1) C 13- C 14	-0.1303	4.32
64. BD (1) N 8- C 9	78. BD (1) C 14- O 19	0.0602	0.71
64. BD (1) N 8- C 9	79. BD (1) C 14- H 26	0.0922	1.50
65. BD (1) C 9- C 10	71. BD (1) O 11- C 12	0.1025	2.24
65. BD (1) C 9- C 10	72. BD (1) C 12- C 13	0.1063	1.13
65. BD (1) C 9- C 10	75. BD (1) C 13- C 14	0.1156	2.81
65. BD (1) C 9- C 10	78. BD (1) C 14- O 19	-0.1336	4.61
65. BD (1) C 9- C 10	79. BD (1) C 14- H 26	0.0938	1.36
65. BD (1) C 9- C 10	80. BD (1) O 15- H 34	0.0828	1.79
66. BD (1) C 9- C 14	68. BD (1) C 10- O 11	0.0937	1.91
66. BD (1) C 9- C 14	69. BD (1) C 10- O 15	-0.1130	3.09
66. BD (1) C 9- C 14	70. BD (1) C 10- H 35	0.0897	1.21
66. BD (1) C 9- C 14	71. BD (1) O 11- C 12	0.1037	1.12
66. BD (1) C 9- C 14	72. BD (1) C 12- C 13	0.1020	2.19
66. BD (1) C 9- C 14	76. BD (1) C 13- O 18	-0.1251	3.95
66. BD (1) C 9- C 14	77. BD (1) C 13- H 29	0.0935	1.31
66. BD (1) C 9- C 14	86. BD (1) O 19- H 27	-0.1059	4.61
67. BD (1) C 9- H 36	68. BD (1) C 10- O 11	0.0690	0.68
67. BD (1) C 9- H 36	69. BD (1) C 10- O 15	0.0815	1.32
67. BD (1) C 9- H 36	70. BD (1) C 10- H 35	-0.1039	4.66
67. BD (1) C 9- H 36	75. BD (1) C 13- C 14	0.0941	1.32
67. BD (1) C 9- H 36	77. BD (1) C 13- H 29	0.0902	0.95
67. BD (1) C 9- H 36	78. BD (1) C 14- O 19	0.0700	0.79
67. BD (1) C 9- H 36	79. BD (1) C 14- H 26	-0.1165	5.58
67. BD (1) C 9- H 36	80. BD (1) O 15- H 34	0.0765	0.73
68. BD (1) C 10- O 11	72. BD (1) C 12- C 13	0.1032	2.36
68. BD (1) C 10- O 11	73. BD (1) C 12- C 16	-0.1019	3.49
68. BD (1) C 10- O 11	74. BD (1) C 12- H 30	0.0960	1.88
68. BD (1) C 10- O 11	75. BD (1) C 13- C 14	0.0967	0.85
68. BD (1) C 10- O 11	80. BD (1) O 15- H 34	0.0759	1.22
69. BD (1) C 10- O 15	71. BD (1) O 11- C 12	-0.0973	3.35
70. BD (1) C 10- H 35	71. BD (1) O 11- C 12	0.0910	1.55
70. BD (1) C 10- H 35	74. BD (1) C 12- H 30	0.0943	1.27
70. BD (1) C 10- H 35	79. BD (1) C 14- H 26	0.0931	0.96
70. BD (1) C 10- H 35	80. BD (1) O 15- H 34	-0.0983	5.57
71. BD (1) O 11- C 12	75. BD (1) C 13- C 14	0.1068	2.18
71. BD (1) O 11- C 12	76. BD (1) C 13- O 18	-0.1152	3.33
71. BD (1) O 11- C 12	81. BD (1) C 16- O 17	0.0549	0.55
71. BD (1) O 11- C 12	82. BD (1) C 16- H 31	-0.1316	5.02
71. BD (1) O 11- C 12	83. BD (1) C 16- H 32	0.0841	0.99
72. BD (1) C 12- C 13	78. BD (1) C 14- O 19	-0.1253	3.97
72. BD (1) C 12- C 13	79. BD (1) C 14- H 26	0.0980	1.55

```
  72. BD ( 1) C 12- C 13        81. BD ( 1) C 16- O 17       -0.1138     3.41
  72. BD ( 1) C 12- C 13        82. BD ( 1) C 16- H 31        0.0830     1.30
  72. BD ( 1) C 12- C 13        83. BD ( 1) C 16- H 32        0.0991     1.65
  72. BD ( 1) C 12- C 13        85. BD ( 1) O 18- H 28        0.0964     2.36
  73. BD ( 1) C 12- C 16        75. BD ( 1) C 13- C 14       -0.1180     3.78
  73. BD ( 1) C 12- C 16        76. BD ( 1) C 13- O 18        0.0463     0.53
  73. BD ( 1) C 12- C 16        77. BD ( 1) C 13- H 29        0.1050     2.21
  73. BD ( 1) C 12- C 16        84. BD ( 1) O 17- H 33        0.0629     1.06
  74. BD ( 1) C 12- H 30        75. BD ( 1) C 13- C 14        0.1040     1.75
  74. BD ( 1) C 12- H 30        77. BD ( 1) C 13- H 29       -0.1124     5.39
  74. BD ( 1) C 12- H 30        79. BD ( 1) C 14- H 26        0.1016     1.13
  74. BD ( 1) C 12- H 30        81. BD ( 1) C 16- O 17        0.0745     1.05
  74. BD ( 1) C 12- H 30        82. BD ( 1) C 16- H 31        0.0737     0.93
  74. BD ( 1) C 12- H 30        83. BD ( 1) C 16- H 32       -0.1125     5.18
  75. BD ( 1) C 13- C 14        85. BD ( 1) O 18- H 28        0.0778     1.42
  75. BD ( 1) C 13- C 14        86. BD ( 1) O 19- H 27        0.1355     5.92
  76. BD ( 1) C 13- O 18        78. BD ( 1) C 14- O 19        0.0542     0.64
  76. BD ( 1) C 13- O 18        79. BD ( 1) C 14- H 26        0.0762     1.07
  77. BD ( 1) C 13- H 29        78. BD ( 1) C 14- O 19        0.0692     0.85
  77. BD ( 1) C 13- H 29        79. BD ( 1) C 14- H 26       -0.1131     5.49
  77. BD ( 1) C 13- H 29        83. BD ( 1) C 16- H 32        0.0922     1.01
  77. BD ( 1) C 13- H 29        85. BD ( 1) O 18- H 28       -0.0966     5.42
  79. BD ( 1) C 14- H 26        85. BD ( 1) O 18- H 28        0.0779     0.62
  82. BD ( 1) C 16- H 31        84. BD ( 1) O 17- H 33       -0.1045     5.82
  83. BD ( 1) C 16- H 32        84. BD ( 1) O 17- H 33        0.0859     2.28

                                                   sum within unit 1:    585.68

between units  1 and  2
  32. LP ( 1) O 18              36. LP ( 1) O 20             -0.0149     0.08
  32. LP ( 1) O 18              37. LP ( 2) O 20              0.0027    -0.05
  36. LP ( 1) O 20              74. BD ( 1) C 12- H 30        0.0691     1.18
  36. LP ( 1) O 20              79. BD ( 1) C 14- H 26        0.0524     0.67
  37. LP ( 2) O 20              66. BD ( 1) C  9- C 14       -0.0202     0.08
  37. LP ( 2) O 20              71. BD ( 1) O 11- C 12       -0.0192     0.06
  37. LP ( 2) O 20              72. BD ( 1) C 12- C 13        0.0324     0.07
  37. LP ( 2) O 20              74. BD ( 1) C 12- H 30        0.0591     0.81
  37. LP ( 2) O 20              75. BD ( 1) C 13- C 14        0.0290     0.06
  37. LP ( 2) O 20              76. BD ( 1) C 13- O 18        0.0637    -0.40
  37. LP ( 2) O 20              79. BD ( 1) C 14- H 26        0.0546     0.64
  37. LP ( 2) O 20              85. BD ( 1) O 18- H 28        0.2065    12.28
  79. BD ( 1) C 14- H 26        87. BD ( 1) O 20- H 48        0.0344     0.39
  79. BD ( 1) C 14- H 26        88. BD ( 1) O 20- H 49       -0.0236     0.11
  85. BD ( 1) O 18- H 28        88. BD ( 1) O 20- H 49       -0.0290     0.19

                                             sum between units 1 and 2:     16.43

between units  1 and  3
  39. LP ( 2) O 21              79. BD ( 1) C 14- H 26        0.0307     0.17
  79. BD ( 1) C 14- H 26        89. BD ( 1) O 21- H 46        0.0209     0.13

                                             sum between units 1 and 3:      0.42

between units  1 and  4
  25. LP ( 1) N  8              91. BD ( 1) O 22- H 42        0.2040    10.44
  25. LP ( 1) N  8              92. BD ( 1) O 22- H 43        0.0570     0.09
  34. LP ( 1) O 19              92. BD ( 1) O 22- H 43        0.0492     0.66
  35. LP ( 2) O 19              40. LP ( 1) O 22             -0.0180     0.25
  35. LP ( 2) O 19              41. LP ( 2) O 22              0.0211    -0.06
  35. LP ( 2) O 19              92. BD ( 1) O 22- H 43        0.1193     4.10
  40. LP ( 1) O 22              49. BD ( 1) C  1- H 37        0.0465     0.56
  40. LP ( 1) O 22              79. BD ( 1) C 14- H 26       -0.0443     0.44
  41. LP ( 2) O 22              49. BD ( 1) C  1- H 37       -0.0314     0.28
  41. LP ( 2) O 22              62. BD ( 2) C  7- N  8        0.0129     0.07
  41. LP ( 2) O 22              79. BD ( 1) C 14- H 26        0.0499     0.62
  47. BD ( 1) C  1- C  6        91. BD ( 1) O 22- H 42       -0.0158     0.05
  49. BD ( 1) C  1- H 37        91. BD ( 1) O 22- H 42        0.0704     1.28
  62. BD ( 2) C  7- N  8        91. BD ( 1) O 22- H 42       -0.0343     0.33
  62. BD ( 2) C  7- N  8        92. BD ( 1) O 22- H 43       -0.0207     0.12
  64. BD ( 1) N  8- C  9        91. BD ( 1) O 22- H 42        0.0219     0.08
  66. BD ( 1) C  9- C 14        91. BD ( 1) O 22- H 42        0.0230     0.08
  75. BD ( 1) C 13- C 14        91. BD ( 1) O 22- H 42       -0.0281     0.20
  75. BD ( 1) C 13- C 14        92. BD ( 1) O 22- H 43       -0.0226     0.11
  78. BD ( 1) C 14- O 19        92. BD ( 1) O 22- H 43        0.0366     0.24
  79. BD ( 1) C 14- H 26        91. BD ( 1) O 22- H 42        0.0524     0.74
  79. BD ( 1) C 14- H 26        92. BD ( 1) O 22- H 43        0.0463     0.54
```

```
                                          sum between units 1 and 4:     21.42

between units  1 and  5
  26. LP ( 1) O 11             93. BD ( 1) O 23- H 44        0.1162     3.76
  26. LP ( 1) O 11             94. BD ( 1) O 23- H 45       -0.0091     0.10
  27. LP ( 2) O 11             43. LP ( 2) O 23              0.0360     0.12
  27. LP ( 2) O 11             93. BD ( 1) O 23- H 44        0.1373     5.17
  28. LP ( 1) O 15             93. BD ( 1) O 23- H 44       -0.0151     0.09
  29. LP ( 2) O 15             42. LP ( 1) O 23             -0.0183     0.10
  29. LP ( 2) O 15             93. BD ( 1) O 23- H 44       -0.0245     0.16
  42. LP ( 1) O 23             67. BD ( 1) C  9- H 36       -0.0175     0.07
  42. LP ( 1) O 23             80. BD ( 1) O 15- H 34        0.0298     0.35
  43. LP ( 2) O 23             67. BD ( 1) C  9- H 36        0.0394     0.33
  43. LP ( 2) O 23             68. BD ( 1) C 10- O 11        0.0212     0.05
  43. LP ( 2) O 23             69. BD ( 1) C 10- O 15        0.0453    -0.14
  43. LP ( 2) O 23             80. BD ( 1) O 15- H 34        0.1255     4.42
  67. BD ( 1) C  9- H 36       93. BD ( 1) O 23- H 44        0.0272     0.18
  68. BD ( 1) C 10- O 11       93. BD ( 1) O 23- H 44        0.0299     0.14
  80. BD ( 1) O 15- H 34       93. BD ( 1) O 23- H 44        0.0458     0.58
  80. BD ( 1) O 15- H 34       94. BD ( 1) O 23- H 45       -0.0341     0.33
  83. BD ( 1) C 16- H 32       93. BD ( 1) O 23- H 44        0.0233     0.13

                                          sum between units 1 and 5:     16.35

between units  1 and  6
  26. LP ( 1) O 11             44. LP ( 1) O 24             -0.0485     0.74
  26. LP ( 1) O 11             45. LP ( 2) O 24              0.0600     0.93
  26. LP ( 1) O 11             95. BD ( 1) O 24- H 50        0.0728     1.68
  26. LP ( 1) O 11             96. BD ( 1) O 24- H 51        0.0202     0.07
  27. LP ( 2) O 11             95. BD ( 1) O 24- H 50       -0.0162     0.12
  29. LP ( 2) O 15             95. BD ( 1) O 24- H 50       -0.0359     0.45
  30. LP ( 1) O 17             45. LP ( 2) O 24              0.0177    -0.24
  45. LP ( 2) O 24             81. BD ( 1) C 16- O 17        0.0380    -0.24
  45. LP ( 2) O 24             83. BD ( 1) C 16- H 32        0.0234     0.06
  45. LP ( 2) O 24             84. BD ( 1) O 17- H 33        0.1575     6.94
  84. BD ( 1) O 17- H 33       95. BD ( 1) O 24- H 50        0.0193     0.15

                                          sum between units 1 and 6:     10.96

within unit  2

                                                  sum within unit 2:      0.00

between units  2 and  3
  37. LP ( 2) O 20             38. LP ( 1) O 21             -0.0150     0.09
  37. LP ( 2) O 20             39. LP ( 2) O 21              0.0185    -0.30
  39. LP ( 2) O 21             87. BD ( 1) O 20- H 48        0.2184    14.76
  39. LP ( 2) O 21             88. BD ( 1) O 20- H 49        0.0187    -0.25

                                          sum between units 2 and 3:     14.41

between units  2 and  4

                                          sum between units 2 and 4:      0.02

between units  2 and  5

                                          sum between units 2 and 5:      0.00

between units  2 and  6

                                          sum between units 2 and 6:      0.00

within unit  3

                                                  sum within unit 3:      0.00

between units  3 and  4
  38. LP ( 1) O 21             41. LP ( 2) O 22             -0.0090     0.13
  38. LP ( 1) O 21             92. BD ( 1) O 22- H 43       -0.0130     0.07
  39. LP ( 2) O 21             40. LP ( 1) O 22             -0.0130     0.09
  39. LP ( 2) O 21             41. LP ( 2) O 22              0.0264    -0.29
  40. LP ( 1) O 22             89. BD ( 1) O 21- H 46        0.0108     0.07
  41. LP ( 2) O 22             89. BD ( 1) O 21- H 46        0.2097    13.34
  41. LP ( 2) O 22             90. BD ( 1) O 21- H 47        0.0221    -0.24
```

```
  89. BD ( 1) O 21- H 46        92. BD ( 1) O 22- H 43        0.0101     0.05
                                         sum between units 3 and 4:     13.27

between units  3 and  5
                                         sum between units 3 and 5:      0.00

between units  3 and  6
                                         sum between units 3 and 6:      0.00

within unit  4
                                                 sum within unit 4:      0.00

between units  4 and  5
                                         sum between units 4 and 5:      0.00

between units  4 and  6
                                         sum between units 4 and 6:      0.00

within unit  5
                                                 sum within unit 5:      0.00

between units  5 and  6
  42. LP ( 1) O 23              44. LP ( 1) O 24             -0.0165     0.20
  42. LP ( 1) O 23              96. BD ( 1) O 24- H 51        0.0767     2.02
  43. LP ( 2) O 23              45. LP ( 2) O 24             -0.0169     0.05
  43. LP ( 2) O 23              96. BD ( 1) O 24- H 51       -0.0306     0.27
  44. LP ( 1) O 24              93. BD ( 1) O 23- H 44       -0.0290     0.23
  93. BD ( 1) O 23- H 44        96. BD ( 1) O 24- H 51        0.0325     0.41
  94. BD ( 1) O 23- H 45        96. BD ( 1) O 24- H 51       -0.0198     0.14

                                         sum between units 5 and 6:      3.41

within unit  6
                                                 sum within unit 6:      0.00

-------------------------------------------------------------------------------
      Total disjoint NLMO steric exchange energy from pairwise sum:    682.37
```

Table S36. *Geometric Parameters[a] and Energy[b] of Anomeric Hydrogen Bonding in Five Hydrated Imines*

Comp.	Medium	D-H···A	d(D-H)	d(H···A)	d(D···A)	<(DHA)	E_{HB}[b,c]
**54·5H$_2$O***	Gas phase	O-H···N	0.966	2.143	2.711	116.164	7.83
	DMSO	O-H···N	0.968	2.154	2.718	115.756	7.61
	Water	O-H···N	0.968	2.205	2.755	114.812	6.53
**55·5H$_2$O***	Gas phase	O-H···N	0.967	2.140	2.708	116.084	7.93
	DMSO	O-H···N	0.969	2.180	2.717	115.960	7.64
	Water	O-H···N	0.968	2.191	2.751	115.529	6.64
**62·5H$_2$O***	Gas phase	O-H···N	0.966	2.165	2.724	115.423	7.42
	DMSO	O-H···N	0.969	2.153	2.716	115.590	7.67
	Water	O-H···N	0.968	2.193	2.743	114.739	6.86

[a] Calculated at M06-2X/6-311G(d,p). [b] Calculated with equation [2], reference 67. [c] In kcal/mol.

Experimental Section

(*)Citations refer to those of the bibliographic section (References) in manuscript.

General. Melting points were determined on Gallenkamp and Electrothermal IA 9000 apparatuses and are uncorrected. Optical rotations were measured on a Perkin-Elmer 241 polarimeter, with sodium light (D line, λ = 589 nm) and mercury light (λ = 578, 546, 463 nm). IR spectra were recorded in the range of 4000-600 cm^{-1} on an FT-IR Thermo spectrophotometer. Solid samples were recorded on KBr (Merck) pellets. NMR spectra were recorded on Bruker 200, 400 and 500 AC/PC instruments, in DMSO-d_6 or $CDCl_3$. Structural elucidation was facilitated through a) DEPT (Distortionless Enhancement by Polarization Transfer), b) 2D correlation spectroscopy (COSY), c) heteronuclear multiple-quantum correlation (HMQC), d) heteronuclear multiple bond correlation (HMBC), e) isotope exchange with deuterium oxide and f) nuclear Overhauser effect (NOE). All *J* values are given in hertz. Microanalyses were determined on a Leco® CHNS-932 analyser. High-resolution mass spectra (HRMS) were carried out using ESI ionization techniques with a 6520 Accurate-Mass Q-TOF LC/MS equipment from Agilent Technologies at the Servicio de Apoyo a la Investigación (SAIUEX) in the University of Extremadura.

Computational Details. The computational DFT study was initially performed using the B3LYP[33] and the M06-2X[34] hybrid density functionals in conjunction with 6-31G(d,p) and 6-311G(d,p) basis sets[32] as implemented in the Gaussian09 package.[81] The M06-2X method was chosen on the basis of previous studies showing its accuracy in estimating conformational energies related to non-covalent interactions. In order to assess the influence of the level of theory on anomer stability, def2-TZVP valence-triple-ζ basis set,[37] was also employed in combination with the M06-2X functional[34] for geometry optimizations, as the latter has proven to be reliable enough in recent studies addressing structure and binding issues in carbohydrate derivatives.[38,39] In all cases, frequency calculations were carried out to confirm the existence of true stationary points on the potential energy surface. All thermal corrections were calculated at the standard values of 1 atm at 298.15K. Solvent effects were modeled through density-based, self-consistent reaction field (SCRF) theory of bulk electrostatics, namely, the solvation model based on density (SMD) as implemented in the Gaussian09 suite of programs. This solvation method accounts for long-range electrostatic polarization (bulk solvent) as well as for short-range effects associated with cavitation, dispersion, and solvent structural effects. Intramolecular interaction of the stabilization energies were performed by means of the second order perturbation theory. *Natural Bond Orbital* analysis was performed with NBO versions 3.1[36a] and 6.0.[36b,36c] For each donor NBO(*i*) and acceptor NBO(*j*), the stabilization energy E_2 associated with electron delocalization between donor and acceptor is estimated as

$$E_2 = \Delta E_{ij} = - q_i\,(F_{ij})^2 / (\varepsilon_i - \varepsilon_j)$$

where q_i is the donor orbital occupancy, ε_i, ε_j are diagonal elements (orbital energies), and F_{ij} is the off-diagonal NBO Fock matrix element. In the natural bond orbital (NBO) approach, a hydrogen bond is viewed as an interaction between an occupied nonbonded natural orbital n_A of the acceptor atom A and the unoccupied antibonding orbital of the DH bond σ_{DH}*. Steric interactions were also estimated through NBO/NLMO steric analysis with NBO 6.0.[36b-d]

Synthetic procedures

Compounds **3**,[3b] **5**,[4c] **6**,[5a] **9**,[5a] **10**,[5a] **11**,[19] **12**,[6] **13**,[9] **15**,[11b] **16**,[9] **18**,[20b] **19**,[20a] **21**,[9] **22**,[9] **32**,[21] **34**,[22] **36**,[22] **39/41**,[23] **44**,[6] **45**,[4b] **47**,[24a] **48**,[26] **49**,[26] **80**,[71d] **81**,[71e] **91**,[75] **92**,[75] **93**,[20b] **94**,[3b] **95**,[78] and **96**[75]have been synthesized as described.

Method 1. To a solution of D-glucosamine hydrochloride (5.0 g, 23.2 mmol) in 1*M* NaOH (25 mL) was added the appropriate aromatic aldehyde (25.0 mmol) and the mixture stirred at room temperature. The resulting solid was filtered and washed with cold water, cold ethanol (-20 ºC), and ethyl ether, and dried under vacuum over silica gel.

Method 2. To a solution of D-glucosamine hydrochloride (1.0 g, 4.7 mmol) and sodium acetate (0.63 g, 7.7 mmol) in water (10 mL) was slowly added a solution of the appropriate aromatic aldehyde (4.7 mmol) dissolved in the minimal amount of methanol. The mixture was kept at room temperature under stirring for 2 h, then it was allowed to stand at that temperature until the appearance of crystals and, subsequently it was stored in the refrigerator. The filtered product was washed with cold water, cold ethanol, and ethyl ether, and dried in vacuo over silica gel.

Method 3. Sodium bicarbonate (0.50 g, 6.0 mmol) was added to a solution of D-glucosamine hydrochloride (1.0 g, 4.7 mmol) in water (6 mL). To the resulting mixture, a solution of the appropriate aromatic aldehyde (4.7 mmol) in the minimum amount of methanol was added dropwise. The mixture was stirred until a solid separated, stored in the refrigerator overnight, and then filtered, washed with cold water, cold ethanol, and ethyl ether, and dried in vacuo over silica gel.

***Method* 4**. Sodium bicarbonate (0.40 g, 4.8 mmol) was added to a solution of 2-amino-2-deoxy-D-*glycero*-L-*gluco*-heptopyranose hydrochloride (0.74 g, 3.0 mmol) in water (5 mL). Then a solution of the corresponding aromatic aldehyde (3.8 mmol) in methanol (4 or 5 mL) was added. The mixture was stirred at room temperature for one hour. After stored in the refrigerator overnight, the solid formed was filtered, washed with cold water, cold ethanol, ethyl ether, and dried under vacuum over silica gel.

***2-[(*E*)-Benzylidenamino]-2-deoxy-β-*D*-glucopyranose* (11).** Method 1 (60%). M.p: 167-168 ºC; $[\alpha]_D$ +36.0º; $[\alpha]_{578}$ +38.0º; $[\alpha]_{546}$ +42.0º; $[\alpha]_{436}$ +60.0º (*c* 0.5, pyridine); [Lit.[19] m.p. 156 ºC (decomp.) ºC]; IR (KBr) $\overline{\nu}_{máx}$ 3500-3000 (OH), 1640 (C=N), 1610, 1590 (arom), 1130, 1100, 1060, 1030, 1010 (C-O), 750 and 680 cm^{-1} (arom); ^{1}H NMR (400 MHz, DMSO-d_6) δ 8.20 (1H, s, N=CH), 7.75 (2H, m, arom), 7.44 (3H, m, arom), 6.57 (1H, d, $J_{1,OH}$ 6.9 Hz, C1-OH), 4.95 (1H, d, $J_{4,OH}$ 5.3 Hz,

C4-OH), 4.86 (1H, d, $J_{3,OH}$ 5.7 Hz, C3-OH), 4.73 (1H, t, $J_{1,OH}$= $J_{1,2}$ 7.2 Hz, H-1), 4.57 (1H, t, $J_{6,OH}$= $J_{6',OH}$ 5.8 Hz, C6-OH), 3.74 (1H, ddd, $J_{6,OH}$ 5.7 Hz, $J_{5,6}$ 1.9 Hz, $J_{6,6'}$ 11.6 Hz, H-6), 3.50 (1H, m, $J_{6',OH}$ 5.9 Hz, $J_{5,6'}$ 6.0 Hz, $J_{6,6'}$ 11.8 Hz, H-6´), 3.45 (1H, dt, $J_{3,OH}$ 5.7 Hz, $J_{2,3}$ 9.0 Hz, $J_{3,4}$ 9.0 Hz, H-3), 3.25 (1H, m, H-5), 3.16 (1H, td, $J_{C4,OH}$ 5.2 Hz, $J_{3,4}\approx J_{4,5}$ 8.8 Hz, H-4), 2.85 (1H, dd, $J_{1,2}$ 8.0 Hz, $J_{2,3}$ 9.0 Hz, H-2). ^{13}C NMR (100 MHz, DMSO-d_6): 162.35 (N=C), 130.83, 128.83 (2 C, arom), 128.31 (2 C, arom), 95.79 (C-1), 78.51 (C-2), 77.13 (C-5), 74.71 (C-3), 70.57 (C-4), 61.52 (C-6).

***2-Deoxy-2-[(*E*)-(4-methoxybenzylidene)amino]-β-D-glucopyranose* (12).** Method 1 (72%). M.p. 186-187 ºC; [α]$_D$ +27.2º; [α]$_{578}$ +28.4º; [α]$_{546}$ +33.0º (*c* 0.5, pyridine); [Lit.[6] m.p. 166 ºC]; IR (KBr) $\bar{\nu}_{máx}$ 3480, 3450-3000 (OH), 2840 (OCH_3), 1640 (C=N), 1610, 1515 (arom), 1260 (C-O-C éter), 1100, 1065, 1030 (C-O) and 830 cm^{-1} (arom, 4-sustituted). ^{1}H NMR (400 MHz, DMSO-d_6) δ 8.11 (1H, s, N=CH), 7.68 (2H, d, arom), 6.98 (2H, d, arom), 6.52 (1H, d, $J_{C1,OH}$ 6.7 Hz, C1-OH), 4.91 (1H, d, $J_{C4,OH}$ 5.3 Hz, C4-OH), 4.80 (1H, d, $J_{C3,OH}$ 5.7 Hz, C3-OH), 4.74 (1H, t, $J_{C1,OH}$= $J_{1,2}$ 7.3 Hz, H-1), 4.54 (1H, t, $J_{C6,OH}$=$J_{C6',OH}$ 5.8 Hz, C6-OH), 3.80 (3H, s, OCH_3), 3.72 (1H, ddd, $J_{C6,OH}$ 5.4 Hz, $J_{5,6}$ 1.6 Hz, $J_{6,6'}$ 11.6 Hz, H-6), 3.47 (1H, m, $J_{C6,OH}$ 5.8 Hz, $J_{6,6'}$ 11.6 Hz, H-6´), 3.41 (1H, dt, $J_{C3,OH}$ 5.6 Hz, $J_{2,3}$=$J_{3,4}$ 8.9 Hz, H-3), 3.22 (1H, m, $J_{5,6}$ 2.0 Hz, $J_{5,6'}$ 5.8, H-5), 3.15 (1H, m, $J_{C4,OH}$ 5.2 Hz, H-4), 2.78 (1H, dd, $J_{1,2}$ 8.0 Hz, $J_{2,3}$ 8.9 Hz, H-2). ^{13}C NMR (100 MHz, DMSO-d_6): 161.69 (N=C), 161.38, 129.96, 129.34, 114.20 (arom), 95.90 (C-1), 78.45 (C-2), 77.12 (C-5), 74.86 (C-3), 70.65 (C-4), 61.57 (C-6), 55.57 (OCH_3). Anal. Calcd. for $C_{14}H_{19}NO_6$: C, 56.56, H, 6.44, N, 4.71. Found: C, 56.25, H, 6.49, N, 4.85.

***2-Deoxy-2-[(*E*)-(3-methoxybenzylidene)amino]-β-D-glucopyranose* (13).[9]** Method 1 (quant. yield). M.p. 145-147 ºC; [α]$_D$ +18.4º; [α]$_{578}$ +17.6º; [α]$_{546}$ +8.0º (*c* 0.5, pyridine); IR (KBr) $\bar{\nu}_{máx}$ 3483, 3319, 2932 (OH), 1641 (C=N), 1597 (arom), 1273 (C-O-C, eter), 1067 (C-O), 860, 797 and 642 cm^{-1} (arom, *m*-substituted). ^{1}H NMR (400 MHz, DMSO-d_6) δ 8.17 (1H, s, N=CH), 7.34 (3H, m, arom), 7.02 (2H, d, arom), 6.59 (1H, sa, C1-OH), 4.94 (1H, sa, C4-OH), 4.73 (1H, d, $J_{1,2}$ 7.5 Hz, H-1), 3.78 (3H, s, OCH_3), 3.73 (1H, d, $J_{6,6'}$ 10.6 Hz, H-6), 3.53 (1H, m, H-3, H-6´), 3.26 (1H, m, $J_{5,6}$ 1.3 Hz, $J_{5,6'}$ 5.6 Hz, H-5), 3.16 (1H, t, $J_{3,4}$=$J_{4,5}$ 9.1 Hz, H-4), 2.84 (1H, t, $J_{1,2}$=$J_{2,3}$ 8.5 Hz, H-2). ^{13}C NMR (100 MHz, DMSO-d_6): 162.26 (N=C), 159.70, 137.92, 129.94, 121.22, 112.26 (arom), 95.74 (C-1), 78.50 (C-2), 77.11 (C-5), 74.66 (C-3), 70.58 (C-4), 61.51 (C-6), 55.44 (OCH_3).

***2-Deoxy-2-[(*E*)-(2-methoxybenzylidene)amino]-β-D-glucopyranose* (14).** Method 3 (34%). M.p. 126-128 ºC; [α]$_D$ +9.6º; [α]$_{578}$ +9.8º; [α]$_{546}$ +11.2º; [α]$_{436}$ +24.0º (*c* 0.5, pyridine); IR (KBr) $\bar{\nu}_{máx}$ 3530-3000 (OH), 1634 (C=N), 1601 (arom), 1088, 1028 (C-O), 729 cm^{-1} (arom); ^{1}H NMR (400 MHz, DMSO-d_6) δ 8.54 (1H, s, N=CH), 7.86 (1H, d, *J* 7.4 Hz, arom), 7.43 (1H, t, *J* 7.5 Hz, arom), 7.07 (1H, d, *J* 8.0 Hz, arom), 6.97 (1H, t, *J* 7.2 Hz, arom), 6.56 (1H, s.a., C1-OH), 5.02-4.88 (2H, m, C4-OH, C3-OH), 4.72 (1H, d, $J_{1,2}$ 7.3 Hz, H-1), 4.64 (1H, m, C6-OH), 3.83 (3H, s, OCH_3), 3.73 (1H, d, $J_{6,6'}$ 11.3 Hz, H-6), 3.45 (2H, m, H-6´, H-3), 3.25 (1H, m, H-5), 3.16 (1H, m, $J_{4,5}\approx J_{3,4}$ 8.9 Hz, H-4), 2.84 (1H, t, $J_{1,2}\approx J_{2,3}$ 8.3 Hz, H-2). ^{13}C NMR (100 MHz, DMSO-d_6): 157.77 (arom), 157.53 (N=C), 132.49 (arom), 127.23 (arom), 124.33 (arom), 120.78 (arom), 112.09 (arom), 96.04 (C-1), 79.12 (C-2), 77.27 (C-5), 74.93 (C-3), 70.73 (C-4), 61.66 (C-6), 55.98 (OCH_3). Anal. Calcd. for $C_{14}H_{19}NO_6$: C, 56.56, H, 6.44, N, 4.71. Found: C,56.32; H, 6.50;N.4.66.

***2-Deoxy-2-[(*E*)-(4-hydroxybenzylidene)amino]-β-D-glucopyranose* (15).** Method 1 (87%). M.p. 173-175 ºC; [α]$_D$ +38.8º; [α]$_{578}$ +42.8º; [α]$_{546}$ +50.8º; [α]$_{436}$ +116.0º (*c* 0.5, pyridine); [Lit.[11b] m.p.

166-167 ºC]; IR (KBr) $\bar{\nu}_{máx}$ 3500-3000 (OH), 1649 (C=N), 1597 (arom), 1147, 1033 (C-O), 831 cm^{-1} (arom); ^{1}H NMR (400 MHz, DMSO-d_6) δ 8.05 (1H, s, N=CH), 7.56 (2H, d, J 8.0 Hz, arom), 6.87 (2H, d, J 8.8 Hz, arom), 4.68 (1H, d, $J_{1,2}$ 7.2 Hz, H-1), 3.73 (1H, d, $J_{6,6'}$ 10.4 Hz, H-6), 3.49 (1H, dd, $J_{5,6}$ 6.2 Hz, $J_{6,6'}$ 11.4 Hz, H-6´), 3.42 (1H, t, $J_{2,3}$=$J_{3,4}$ 8.8 Hz, H-3), 3.23 (1H, m, H-5), 3.15 (1H, t, $J_{3,4}$=$J_{4,5}$ 9.4 Hz, H-4), 2.79 (1H, t, $J_{1,2}$≈$J_{2,3}$ 8.4 Hz, H-2). ^{13}C NMR (100 MHz, DMSO-d_6): 161.80 (N=C), 160.10 (arom), 130.11 (2C, arom), 127.74 (arom), 115.64 (2C, arom), 95.96 (C-1), 78.46 (C-2), 77.09 (C-5), 74.94 (C-3), 70.63 (C-4), 61.57 (C-6).

***2-Deoxy-2-[(*E*)-(3-hydroxybenzylidene)amino]-β-D-glucopyranose* (16).**[9] Method 1 (71%). M.p. 175-178 ºC; [α]D +21.6º; [α]578 +25.0º; [α]546 +30.6º; [α]436 +78.4º; [α]365 +224.6º (*c* 0.5, pyridine); IR (KBr) $\bar{\nu}_{máx}$ 3373, 3119, 2884 (OH), 1645 (C=N), 1585 (arom), 1028 (C-O-O), 891, 785, 685 cm^{-1} (arom).^{1}H NMR (400 MHz, DMSO-d_6) δ 9.55 (1H, s, OH-arom), 8.10 (1H, s, N=CH), 7.23 (1H, t, arom), 7.19 (1H, t, arom), 7.13 (1H, d, arom), 6.84 (1H, dd, arom), 6.55 (1H, d, $J_{1,OH}$ 6.8 Hz, C1-OH), 4.94 (1H, m, $J_{4,OH}$ 4.9 Hz, C4-OH), 4.84 (1H, d, $J_{3,OH}$ 5.6 Hz, C3-OH), 4.70 (1H, t, $J_{1,OH}$=$J_{1,2}$ 7.2 Hz, H-1), 4.56 (1H, t, $J_{6,OH}$ 5.6 Hz, C6-OH), 3.73 (1H, dd, $J_{6,6'}$ 10.8 Hz, $J_{5,6}$ 4.6 Hz, H-6), 3.48 (1H, m, $J_{6,OH}$ 5.8 Hz, H-6´), 3.44 (1H, m, H-3), 3.25 (1H, m, H-5´), 3.15 (1H, td, $J_{4,OH}$ 4.7 Hz, $J_{3,4}$=$J_{4,5}$ 8.9 Hz, H-4), 2.82 (1H, t, $J_{1,2}$=$J_{2,3}$ 8.5 Hz, H-2). ^{13}C NMR (50.3 MHz, DMSO-d_6): 162.27 (N=C), 157.72, 137.81, 129.81, 119.83, 117.96, 114.00 (C-arom), 95.80 (C-1), 78.36 (C-2), 77.11 (C-5), 74.71 (C-3), 70.55 (C-4), 61.48 (C-6). Anal. Calcd. for $C_{13}H_{17}NO_6$: C, 55.12, H, 6.05, N, 4.94. Found: C, 54.99, H, 5.91, N, 4.89.

***2-[(*E*)-(2-Chlorobenzylidene)amino]-2-deoxy-β-D-glucopyranose* (17).** Method 1 (48%). M.p. 95-97 ºC; [α]D +17.6º; [α]578 +18.9º; [α]546 +22.5º; [α]436 +51.1º (*c* 1.0, pyridine); IR (KBr) $\bar{\nu}_{máx}$ 3500-3000 (OH), 1634 (C=N), 1593 (arom), 1433, 1111, 1092, 1033 (C-O) and 749 cm^{-1} (arom); ^{1}H NMR (500 MHz, DMSO-d_6) δ 8.54 (1H, s, N=CH), 8.00 (1H, dd, J 7.5 Hz, J 1.5 Hz, arom), 7.52 (1H, td, J 7.5 Hz, J 1.5 Hz, arom), 7.48 (1H, td, J 8.0 Hz, J 2.0 Hz, arom), 7.40 (1H, td, J 7.5 Hz, J 1.5 Hz, arom), 6.60 (1H, d, $J_{C1,OH}$ 6.5 Hz, C1-OH), 4.97 (1H, d, $J_{C3,OH}$ 5.5 Hz, C3-OH), 4.89 (1H, d, $J_{C4,OH}$ 6.0 Hz, C4-OH), 4.74 (1H, t, $J_{C1,OH}$≈$J_{1,2}$ 7.5 Hz, H-1), 4.57 (1H, t, $J_{C6,OH}$ 6.0 Hz, C6-OH), 3.73 (1H, ddd, $J_{5,6}$ 2.0 Hz, $J_{6,OH}$ 5.5 Hz,$J_{6,6'}$ 11.5 Hz, H-6), 3.50 (1H, dd, $J_{6,OH}$, 5.5 Hz, $J_{6,6'}$ 11.5 Hz, H-6´), 3.44 (1H, td, $J_{C3,OH}$ 5.5 Hz, $J_{2,3}$ ≈$J_{3,4}$ 9.0 Hz, H-3), 3.26 (1H, m, H-5), 3.16 (1H, td, $J_{C4,OH}$ 5.5 Hz, $J_{3,4}$ ≈$J_{4,5}$ 9.5 Hz H-4), 2.91 (1H, dd, $J_{1,2}$ 7.5 Hz, $J_{2,3}$ 9.0 Hz, H-2). ^{13}C NMR (125 MHz, DMSO-d_6): 157.99 (N=C), 134.02 (arom), 132.65 (arom), 132.18 (arom), 129.86 (arom), 128.33 (arom), 127.39 (arom), 95.43 (C-1), 78.50 (C-2), 76.99 (C-5), 74.29 (C-3), 70.23 (C-4), 61.24 (C-6). Anal. Calcd. for $C_{13}H_{16}ClNO_5$: C, 51.74, H, 5.31, Cl, 11,61, N, 4.64. Found: C, 51.99, H, 5.21, N, 4.56. HRMS [M+H^{+}] calcd. for $C_{13}H_{16}ClNO_5$: 302.0687; Found: 302.0777.

***2-Deoxy-2-[(*E*)-(4-hydroxy-3-methoxybenzylidene)amino]-β-D-glucopyranose* (18).** Method 1 (quant. yield). M.p. 193-195 ºC; [α]D +34.0º; [α]578 +35.0º; [α]546 +42.2º (*c* 0.5, pyridine); [Lit.[20b] m.p. 184 ºC; [α]546 +64º (pyridine)]; IR (KBr) $\bar{\nu}_{máx}$ 3331 (OH), 1634 (C=N), 1587, 1526 (arom), 1298 (C-O-C, éter), 1028 (C-O) and 864 cm^{-1} (arom). ^{1}H NMR (400 MHz, DMSO-d_6) δ 8.04 (1H, s, N=CH), 7.33 (1H, s, arom), 7.92 (1H, d, arom), 6.82 (1H, d, arom), 6.54 (1H, sa, C1-OH), 4.70 (1H, d, $J_{1,2}$=$J_{1,OH}$ 7.3 Hz, H-1), 3.79 (3H, s, OCH_3), 3.73 (1H, d, $J_{6,6'}$ 11.4 Hz, H-6), 3.46 (3H, m, H-3, H-6´), 3.24 (1H, m, H-5), 3.15 (1H, m, $J_{3,4}$=$J_{4,5}$ 9.1, H-4), 2.78 (1H, t, $J_{1,2}$=$J_{2,3}$ 8.4 Hz, H-2). ^{13}C NMR (100 MHz, DMSO-d_6): 162.12 (N=C), 149.39, 148.07, 128.29, 123.15, 115.42, 110.33

(arom), 95.90 (C-1), 78.46 (C-2), 77.07 (C-5), 74.89 (C-3), 70.70 (C-4), 61.56 (C-6), 55.80 (OCH_3). Anal. Calcd. for $C_{14}H_{19}NO_7$: C, 53.67, H, 6.11, N, 4.47. Found: C, 52.53, H, 6.18, N, 4.31.

***2-Deoxy-2-[(*E*)-(4-nitrobenzylidene)amino]-β-D-glucopyranose* (19).** Method 2 (38%). M.p. 155-157 ºC; $[\alpha]_D$ +40.2º; $[\alpha]_{578}$ +41.6º; $[\alpha]_{546}$ +46.8º (*c* 0.5, pyridine); [Lit.[20a] m.p. 182-184 ºC]; IR (KBr) $\bar{\nu}_{máx}$ 3345 (OH), 1643 (C=N), 1603, 1518 (arom), 1344 ($C-NO_2$), 1094, 1018 (C-O), 864 and 748 cm^{-1} (arom); ^1H NMR (400 MHz, DMSO-d_6) δ 8.37 (1H, s, =CH), 8.31 (2H, d, arom), 8.04 (2H, d, arom), 6.68 (1H, d, $J_{1,OH}$ 6.7 Hz, C1-OH), 5.02 (1H, d, $J_{4,OH}$ 5.4 Hz, OH-4), 4.98 (1H, d, $J_{3,OH}$ 5.5 Hz, C3-OH), 4.79 (1H, t, $J_{1,2}$= $J_{1,OH}$ 7.2 Hz, H-1), 4.60 (1H, t, $J_{6,OH}$ 5.8 Hz, C6-OH), 3.75 (1H, ddd, $J_{6,OH}$ 5.5 Hz, $J_{5,6'}$ 1.5 Hz, $J_{6,6'}$ 11.3 Hz, H-6´), 3.51 (2H, m, H-3, H-6´), 3.28 (1H, m, H-5), 3.20 (1H, td, $J_{4,OH}$ 5.4 Hz, $J_{3,4}$=$J_{4,5}$ 8.7 Hz, H-4), 2.94 (1H, t, $J_{1,2}$ 8.5 Hz, H-2). ^{13}C NMR (100 MHz, DMSO-d_6): 160.77 (N=C), 148.69, 142.06, 129.30, 124.11 (arom), 95.58 (C-1), 78.58 (C-2), 77.19 (C-5), 74.46 (C-3), 70.39 (C-4), 61.43 (C-6).

***2-Deoxy-2-[(*E*)-(4-dimethylaminobenzylidene)amino]-β-D-glucopyranose* (20).** Method 3 (41%). M.p. 189-191 ºC; $[\alpha]_D$ +30.6º; $[\alpha]_{578}$ +32.4º; $[\alpha]_{546}$ +39.0º; $[\alpha]_{436}$ +92.2º (*c* 0.5, pyridine); IR (KBr) $\bar{\nu}_{máx}$ 3500-3100 (OH), 1634 (C=N), 1612 (arom), 1070, 1030 (C-O), 818 cm^{-1} (arom); ^1H NMR (400 MHz, DMSO-d_6) δ 8.01 (1H, s, N=CH), 7.54 (2H, d, *J* 8.5 Hz, arom), 6.70 (2H, d, *J* 8.6 Hz, arom), 6.50 (1H, s.a., C1-OH), 4.89-4.79 (2H, m, C4-OH, C3-OH), 4.66 (1H, d, $J_{1,2}$ 7.6 Hz, H-1), 4.58 (1H, m, C6-OH), 3.65 (1H, d, $J_{6,6'}$ 11.6 Hz, H-6), 3.47 (1H, m, $J_{5,6'}$ 5.8 Hz, $J_{6,6'}$ 11.6 Hz, H-6'), 3.38 (1H, m, H-3), 3.21 (1H, m, H-5), 3.13 (1H, t, $J_{4,5}$≈$J_{3,4}$ 9.1 Hz, H-4), 3.00 (2x3H, s, CH_3), 2.74 (1H, t, $J_{1,2}$≈$J_{2,3}$ 8.5 Hz, H-2). ^{13}C NMR (100 MHz, DMSO-d_6): 161.89 (N=C), 129.59, 124.41, 111.58 (arom), 96.02 (C-1), 78.49 (C-2), 77.03 (C-5), 75.03 (C-3), 70.66 (C-4), 61.54 (C-6). Anal. Calcd. for $C_{15}H_{22}N_2O_5$: C, 58.05, H, 7.15, N, 9.03. Found: C, 57.83; H, 7.01; N, 8.93.

***2-Deoxy-2-[(*E*)-(4-methylbenzylidene)amino]-β-D-glucopyranose* (21).**[9] Method 1 (quant. yield). M.p. 189-191 ºC; $[\alpha]_D$ +33.8º; $[\alpha]_{578}$ +35.6º; $[\alpha]_{546}$ +42.4º; $[\alpha]_{436}$ +91.4º; (*c* 0.5, pyridine); IR (KBr) $\bar{\nu}_{máx}$ 3492, 3300-3100 (OH), 1641 (C=N), 1608 (arom), 1101, 1063, 986 cm^{-1} (C-O). ^1H NMR (400 MHz, DMSO-d_6) δ 8.16 (1H, s, N=CH), 7.65 (2H, d, *J* 8.0 Hz, arom), 7.24 (2H, d, *J* 8.4 Hz, arom), 6.57 (1H, d, $J_{C1,OH}$ 6.4 Hz, C1-OH), 4.96 (1H, bs, C4-OH), 4.86 (1H, d, $J_{C3,OH}$ 4.8 Hz, C3-OH), 4.72 (1H, t, $J_{1,2}$ ≈ $J_{C1,OH}$ 7.2 Hz, H-1), 3.74 (1H, d, $J_{6,6'}$ 10.8 Hz, H-6), 3.49 (1H, dd, $J_{6,6'}$ 11.4 Hz, $J_{C6,OH}$ 5.0 Hz, H-6'), 3.47 (1H, m, H-3), 3.24 (1H, m, H-5), 3.17 (1H, m, $J_{3,4}$=$J_{4,5}$ 8.8 Hz, H-4), 2.83 (1H, dd, $J_{1,2}$ 7.8 Hz, $J_{2,3}$ 9.0 Hz, H-2), 2.40 (3H, s, CH_3). ^{13}C NMR (100 MHz, DMSO-d_6): 162.13 (N=C), 140.51, 133.90, 129.41, 128.32 (arom), 95.87 (C-1), 78.49 (C-2), 77.12 (C-5), 74.79 (C-3), 70.60 (C-4), 61.54 (C-6), 21.35 (OCH_3). Anal. Calcd. for $C_{14}H_{19}NO_5$: C, 59.79, H, 6.76, N, 4.98. Found: C, 59.99, H, 6.61, N, 4.89.

***2-Deoxy-2-[(*E*)-(3-methylbenzylidene)amino]-β-D-glucopyranose* (22).**[9] Method 1 (86%). M.p. 166-168 °C; $[\alpha]_D$ +30.5°; $[\alpha]_{578}$ +32.3°; $[\alpha]_{546}$ +38.5°; $[\alpha]_{436}$ +87.5° (*c* 0.5, pyridine); IR (KBr) $\bar{\nu}_{max}/cm^{-1}$ 3500-3000 (OH), 1645 (C=N) 1606, 1585 (arom), 1251 (C-O-C), 1119, 1104, 1068, 1036 (C-O), 887, 797, 695 (arom); ^1H NMR (500 MHz, DMSO-d_6) δ 8.16 (s, 1H, CH=N), 7.58 (s, 1H, H-arom), 7.51 (d, *J* = 7.5 Hz, 1H, H-arom), 7.33 (t, *J* = 7.5 Hz, 1H, H-arom), 7.26 (d, *J* = 7.5 Hz, 1H, H-arom), 6.54 (d, $J_{1,OH}$ = 7.0 Hz, 1H, C1-OH), 4.93 (d, $J_{4,OH}$ = 5.0 Hz, 1H, C4-OH), 4.83 (d, $J_{3,OH}$ = 5.5 Hz, 1H, C3-OH), 4.72 (t, $J_{1,OH}$≈ $J_{1,2}$ = 7.5 Hz, 1H, 1-H), 4.55 (t, $J_{6,OH}$ = 5.5 Hz, 1H, C6-OH), 3.74 (dd, $J_{5,6}$ = 3.0 Hz, $J_{6,6'}$ = 11.5 Hz, 1H, H-6), 3.50 (m, 1H, H-6'), 3.44 (dt, $J_{3,OH}$ = 5.5

Hz, $J_{2,3} \approx J_{3,4}$ = 9.0 Hz, 1H, H-3), 3.25 (ddd, $J_{5,6}$ = 2.0 Hz, $J_{5,6'}$ = 5.5 Hz, $J_{4,5}$ = 9.5 Hz, 1H, H-5), 3.16 (td, $J_{4,OH}$ = 4.5 Hz, $J_{3,4} \approx J_{4,5}$ = 9.0 Hz, 1H, H-4), 2.83 (dd, $J_{1,2}$ = 8.0 Hz, $J_{2,3}$ = 9.5 Hz, 1H, H-2), 2.35 (s, 1H, CH_3); ^{13}C NMR (125 MHz, DMSO-d_6) δ 162.12 (C=N), 137.78, 136.25, 131.20, 128.48, 128.20, 125.66 (arom), 95.63 (C-1), 78.30 (C-2), 76.93 (C-5), 74.56 (C-3), 70.44 (C-4), 61.36 (C-6), 20.93 (CH_3). Anal. Calcd. for $C_{14}H_{19}NO_5$: C, 59.79, H, 6.76, N, 4.98. Found: C, 59.87, H, 6.74, N, 4.82.

***2-Deoxy-2-[(*E*)-(2-methylbenzylidene)amino]-β-D-glucopyranose* (23).** Method 1 (67%). M.p. 165-167 °C; [α]D +12.5°; $[\alpha]_{578}$ +14.6°; $[\alpha]_{546}$ +16.1°; $[\alpha]_{436}$ +41.5° (*c* 0.5, pyridine); IR (KBr) $\bar{\nu}_{max}$/cm^{-1} 3500-3200 (OH), 1635 (C=N) 1602 (arom), 1230 (C-O-C), 1106, 1075, 1030 (C-O); ^1H NMR (500 MHz, DMSO-d_6) δ 8.46 (s, 1H, CH=N), 7.79 (d, J = 7.5 Hz, 1H, H-arom), 7.32 (dt, J = 1.0 Hz, J = 7.5 Hz, 1H, H-arom), 7.24 (m, 2H, H-arom), 6.52 (d, $J_{1,OH}$ = 7.0 Hz, 1H, C1-OH), 4.91 (d, $J_{4,OH}$ = 5 Hz, 1H, C4-OH), 4.81 (d, $J_{3,OH}$ = 6.0 Hz, 1H, C3-OH), 4.73 (t, $J_{1,OH} \approx J_{1,2}$ = 7.5 Hz, 1H, H-1), 4.54 (t, $J_{6,OH}$ = 5.5 Hz, 1H, C6-OH), 3.74 (ddd, $J_{5,6}$ = 1.5 Hz, $J_{6,OH}$ = 5.0 Hz, $J_{6,6'}$ = 11.5 Hz, 1H, H-6), 3.50 (m, 1H, H-6'), 3.45 (dt, $J_{3,OH}$ = 5.5 Hz, $J_{2,3} \approx J_{3,4}$ = 9.0 Hz, 1H, H-3), 3.25 (ddd, $J_{5,6}$ = 2.0 Hz, $J_{5,6'}$= 6.0 Hz, $J_{4,5}$ = 10.0 Hz, 1H, H-5), 3.16 (td, $J_{4,OH}$ = 5.0 Hz, $J_{3,4} \approx J_{4,5}$ = 9.0. Hz, 1H, H-4), 2.86 (dd, $J_{1,2}$ = 8.0 Hz, $J_{2,3}$ = 9.0 Hz, 1H, H-2), 2.47 (s, 1H, CH_3); ^{13}C NMR (100 MHz, DMSO-d_6) δ 160.55 (C=N), 137.25, 134.05, 130.69, 129.95, 127.31, 125.79 (arom), 95.56 (C-1), 78.54 (C-2), 76.87 (C-5), 74.43 (C-3), 70.28 (C-4), 61.23 (C-6), 18.92 (CH_3). Anal. Calcd. for $C_{14}H_{19}NO_5$: C, 59.79, H, 6.76, N, 4.98. Found:: C, 59.84, H, 6.71, N, 4.78.

***2-Deoxy-2-[(*E*)-(4-ethylbenzylidene)amino]-β-D-glucopyranose* (24).** Method 1 (83%). M.p. 163-165 °C; [α]D +24.5°; $[\alpha]_{578}$ +25.8°; $[\alpha]_{546}$ +31.4°; $[\alpha]_{436}$ +73.3° (*c* 0.5, pyridine); IR (KBr) $\bar{\nu}_{max}$/cm^{-1} 3500-3100 (OH), 1643 (C=N) 1608 (arom), 1248 (C-O-C), 1115, 1103, 1070, 1030 (C-O), 890 (arom); ^1H NMR (500 MHz, DMSO-d_6) δ 8.17 (s, 1H, CH=N), 7.67 (d, J = 8.0 Hz, 2H, H-arom), 7.28 (d, J = 8.0 Hz, 2H, H-arom), 6.53 (d, $J_{1,OH}$ = 6.5 Hz, 1H, C1-OH), 4.93 (sa, 1H, C4-OH), 4.82 (d, $J_{3,OH}$ = 5 Hz, 1H, C3-OH), 4.73 (t, $J_{1,OH} \approx J_{1,2}$ = 7 Hz, 1H, H-1), 4.55 (t, $J_{6,OH}$ = 5.0 Hz, 1H, C6-OH), 3.74 (dd, $J_{5,6}$ = 3.0 Hz, $J_{6,6'}$ = 11.0 Hz, 1H, H-6), 3.51 (m, 1H, H-6'), 3.45 (dt, $J_{3,OH}$ = 4.5 Hz, $J_{2,3} \approx J_{3,4}$ = 9.0 Hz, 1H, H-3), 3.24 (ddd, $J_{5,6}$ = 2.0 Hz, $J_{5,6'}$ = 6.0 Hz, $J_{4,5}$ = 9.5 Hz, 1H, H-5), 3.17 (t, $J_{3,4} \approx J_{4,5}$ = 8.5 Hz, 1H, H-4), 2.83 (t, $J_{1,2} \approx J_{2,3}$ = 8.5 Hz, 1H, H-2), 2.65 (c, J = 7.5 Hz, 2H, CH_2), 1.20 (t, J = 7.5 Hz, 1H, CH_3); ^{13}C NMR (125 MHz, DMSO-d_6) δ 161.68 (C=N), 146.36, 133.83, 128.00, 127.79 (arom), 95.49 (C-1), 78.13 (C-2), 76.76 (C-5), 74.43 (C-3), 70.28 (C-4), 61.19 (C-6), 27.99 (CH_2), 15.30 (CH_3). Anal. Calcd. for $C_{15}H_{21}NO_5$: C, 61.02, H, 7.12, N, 4.75. Found: C, 60.98, H, 7.23, N, 4.89.

***2-Deoxy-2-[(*E*)-(4-isopropylbenzylidene)amino]-β-D-glucopyranose* (25).** Method 1 (43%). M. p. 142-144 ºC; [α]D +26.6º; $[\alpha]_{578}$ +28.0º; $[\alpha]_{546}$ +32.8º; $[\alpha]_{436}$ +73.5º (*c* 0.5, pyridine); IR (KBr) $\bar{\nu}_{máx}$ 3469, 3288, 3174 (OH), 1646 (C=N), 1608, 1571, 1509 (arom), 1254, 1091,1080, 1034 (C-O), 991 and 835 cm^{-1} (arom); ^1H RMN (500 MHz, DMSO-d_6) δ 8.15 (s, 1H, CH=N), 7.66 (d, J = 8.0 Hz, 2H, H-arom), 7.31 (d, J = 8.0 Hz, 2H, H-arom), 6.55 (d, $J_{1,OH}$ = 6.0 Hz, 1H, C1-OH), 4.96 (sa, 1H, C4-OH), 4.81 (sa, 1H, C3-OH), 4.72 (t, $J_{1,OH} \approx J_{1,2}$ = 7.0 Hz, 1H, H-1), 4.57 (sa, 1H, C6-OH), 3.73 (d, $J_{6,6'}$ = 11.5 Hz, 1H, H-6), 3.49 (dd, $J_{5,6'}$ = 5.5 Hz, $J_{6,6'}$ = 11.5 Hz, 1H, H-6'), 3.45 (m, 1H, H-3), 3.24 (m, 1H, H-5), 3.15 (t, $J_{3,4} \approx J_{4,5}$ = 8.0 Hz, 1H, H-4), 2.92 (m, J = 7.0 Hz, 1H, CH isopropyl), 2.82 (t, $J_{1,2} \approx J_{2,3}$ = 9.0 Hz, 1H, H-2), 1.21 (d, J = 7.0 Hz, 6H, CH_3 isopropyl); ^{13}C RMN (125 MHz, DMSO-d_6) δ 161.87 (C=N), 151.15, 134.10, 128.19 (2C), 126.51 (2C) (arom), 95.62

(C-1), 78.30 (C-2), 76.90 (C-5), 74.53 (C-3), 70.36 (C-4), 61.30 (C-6), 33.44 (CH isopropyl), 15.30 (2C,CH_3). Anal. Calc. for $C_{16}H_{23}NO_5$: C, 62.14, H, 7.44, N, 4.53. Found: C, 62.01, H, 7.25, N, 4.66. HRMS [$M+H^+$] calculated for $C_{16}H_{23}NO_5$: 310.1649. Found: 310.1657.

***2-Deoxy-2-[(E)-(4-phenylbenzylidene)amino]-β-D-glucopyranose* (26).** Method 3 (84%). M.p. 177-179 °C; $[\alpha]_D$ +31.8°; $[\alpha]_{578}$ +34.6°; $[\alpha]_{546}$ +41.1°; $[\alpha]_{436}$ +96.5° (*c* 0.5, pyridine); IR (KBr) $\bar{\nu}_{max}/cm^{-1}$ 3500-3200 (OH), 1637 (C=N) 1604, 1581 (arom), 1244 (C-O-C), 1071, 999 (C-O) ; ^1H NMR (500 MHz, DMSO-*d6*) δ 8.26 (s, 1H, CH=N), 7.84 (d, *J* = 8.0 Hz, 2H, H-arom), 7.75 (d, *J* = 8.5 Hz, 2H, H-arom), 7.72 (d, *J* = 7.0 Hz, 2H, H-arom), 7.48 (t, *J* = 7.5 Hz, 2H, H-arom), 7.39 (t, *J* = 7.0 Hz, 1H, H-arom), 6.58 (d, $J_{1,OH}$ = 6.0 Hz, 1H, C1-OH), 4.94 (d, 1H, $J_{4,OH}$ = 5.0 Hz, C4-OH), 4.87 (d, $J_{3,OH}$ = 6.0 Hz, 1H, C3-OH), 4.76 (t, $J_{1,OH} \approx J_{1,2}$ = 7.0 Hz, 1H, H-1), 4.56 (t, $J_{6,OH}$ = 5.5 Hz, 1H, C6-OH), 3.75 (ddd, $J_{5,6}$ = 2.0 Hz, $J_{6,OH}$ = 5.5 Hz, $J_{6,6'}$ = 11.5 Hz, 1H, H-6), 3.52 (m, 1H, H-6'), 3.47 (m, 1H, H-3), 3.28 (ddd, $J_{5,6}$ = 2.0 Hz, $J_{5,6'}$ = 5.5 Hz, $J_{4,5}$ = 9.5 Hz, 1H, H-5), 3.19 (td, $J_{4,OH}$ = 5.5 Hz, $J_{3,4} \approx J_{4,5}$ = 9.0 Hz, 1H, H-4), 2.88 (t, $J_{1,2} \approx J_{2,3}$ = 9.0 Hz, 1H, H-2); ^{13}C NMR (125 MHz, DMSO-*d6*) δ 161.29 (C=N), 141.70, 139.12, 135.03, 128.73, 128.36, 127.59, 126.46, 126.43 (arom), 95.32 (C-1), 78.06 (C-2), 76.62 (C-5), 74.25 (C-3), 70.09 (C-4), 61.28 (C-6). Anal. Calcd. for $C_{19}H_{21}NO_5$: C, 66.47, H, 6.12, N, 4.08. Found: C, 66.32, H, 6.25, N, 4.06.

***2-Deoxy-2-[(E)-(2,4-dimethoxybenzylidene)amino]-β-D-glucopyranose* (27).** Method 1 (61%). M.p. 130-132 °C; $[\alpha]_D$ +10.3°; $[\alpha]_{578}$ +10.8°; $[\alpha]_{546}$ +13.1°; $[\alpha]_{436}$ +30.5° (*c* 0.5, pyridine); IR (KBr) $\bar{\nu}_{max}/cm^{-1}$ 3600-3200 (OH), 1634 (C=N) 1614 (arom), 1250 (C-O-C), 1084, 1026 (C-O); ^1H NMR (500 MHz, DMSO-*d6*) δ 8.41 (s, 1H, CH=N), 7.78 (d, *J* = 9.0 Hz, 1H, H-arom), 6.60 (d, *J* = 2.0 Hz, 1H, H-arom), 6.56 (dd, *J* = 2.0 Hz, *J* = 8.5 Hz, 1H, H-arom), 6.45 (d, $J_{1,OH}$ = 7.0 Hz, 1H, C1-OH), 4.88 (d, $J_{4,OH}$ = 5.0 Hz, 1H, C4-OH), 4.72 (d, $J_{3,OH}$ = 5.5 Hz, 1H, C3-OH), 4.67 (t, $J_{1,OH} \approx J_{1,2}$ = 7.5 Hz, 1H, H-1), 4.52 (t, $J_{6,OH}$ = 5.5 Hz, 1H, C6-OH), 3.84 (s, 3H, OCH_3), 3.81 (s, 3H, OCH_3), 3.72 (dd, $J_{5,6}$ = 3.0 Hz, $J_{6,6'}$ = 11.5 Hz, 1H, H-6), 3.48 (m, 1H, H-6'), 3.39 (m, 1H, H-3), 3.22 (ddd, $J_{5,6}$ = 2.0 Hz, $J_{5,6'}$ = 7.0 Hz, $J_{4,5}$ = 9.5 Hz, 1H, H-5), 3.13 (td, $J_{4,OH}$ = 4.0 Hz, $J_{3,4} \approx J_{4,5}$ = 9.0 Hz, 1H, H-4), 2.75 (t, $J_{1,2} \approx J_{2,3}$ = 8.0 Hz, 1H, H-2); ^{13}C NMR (125 MHz, DMSO- *d6*) δ 162.36, 159.46 (arom), 156.26 (C=N), 127.80, 116.97, 105.65, 97.63 (arom), 95.47 (C-1), 78.39 (C-2), 76.57 (C-5), 74.42 (C-3), 70.16 (C-4), 61.05 (C-6), 55.35, 55.11 (OCH_3). Anal. Calcd. for $C_{15}H_{21}NO_7$: C, 55.05, H, 6.42, N, 4.28. Found: C, 54.88, H, 6.27, N, 4.21.

***2-Deoxy-2-[(E)-(2,4-dimethylbenzylidene)amino]-β-D-glucopyranose* (28).** Method 1 (45%). M.p. 125-127 °C; $[\alpha]_D$ +11.2°; $[\alpha]_{578}$ +12.7°; $[\alpha]_{546}$ +15.5°; $[\alpha]_{436}$ +39.4° (c 0.5, pyridine); IR (KBr) $\bar{\nu}_{max}/cm^{-1}$ 3500-3000 (OH), 1636 (C=N) 1614 (arom), 1248 (C-O-C), 1119, 1082, 1052, 1027 (C-O), 891 (arom); ^1H NMR (500 MHz, DMSO-*d6*) δ 8.40 (s, 1H, CH=N), 7.68 (d, *J* = 8.5 Hz, 1H, H-arom), 7.04 (d, *J* = 6.5 Hz, 2H, H-arom), 6.50 (d, $J_{1,OH}$ = 7.0 Hz, 1H, C1-OH), 4.90 (d, $J_{4,OH}$ = 5.0 Hz, 1H C4-OH), 4.78 (d, $J_{3,OH}$ = 5.5 Hz, 1H, C3-OH), 4.71 (t, $J_{1,OH} \approx J_{1,2}$ = 7.0 Hz, 1H, H-1), 4.53 (t, $J_{6,OH}$ = 6.0 Hz, 1H, C6-OH), 3.73 (ddd, $J_{5,6}$ = 2.0 Hz, $J_{6,OH}$ = 5.5 Hz, $J_{6,6'}$ = 11.5 Hz, 1H, H-6), 3.49 (m, 1H, H-6'), 3.43 (dt, $J_{3,OH}$ = 5.5 Hz, $J_{2,3} \approx J_{3,4}$ = 10.0 Hz, 1H, H-3), 3.24 (ddd, $J_{5,6}$ = 1.5 Hz, $J_{5,6'}$ = 5.5 Hz, $J_{4,5}$ = 9.5 Hz, 1H, H-5), 3.15 (td, $J_{4,OH}$ = 5.0 Hz, $J_{3,4} \approx J_{4,5}$ = 9.0 Hz, 1H, H-4), 2.83 (dd, $J_{1,2}$ = 8.0 Hz, $J_{2,3}$ = 9.0 Hz, 1H, H-2), 2.43 (s, 3H, CH_3), 2.26 (s, 1H, CH_3); ^{13}C NMR (125 MHz, DMSO-*d6*) δ 160.35 (C=N), 139.40, 137.08, 131.19, 127.42, 126.42 (arom), 95.57 (C-1), 78.48 (C-2), 76.79 (C-5), 74.46 (C-3), 70.31 (C-4), 61.21 (C-6), 20.78, 18.79 (CH_3). Anal. Calcd. for $C_{15}H_{21}NO_5$: C, 61.02, H, 7.12, N, 4.75. Found: C, 59.95, H, 6.93, N, 4.89.

***2-Deoxy-2-[(*E*)-(2,4,6-trimethylbenzylidene)amino]-α-D-glucopyranose* (29).** Method 1 (90%). M.p. 201-204 ºC; [α]D -1.2º; [α]578 -0.8º; [α]546 -1.8º; [α]436 -7.4º (*c* 0.5, pyridine); IR (KBr) $\bar{\nu}_{máx}$: 3428, 3300, 3188 (OH), 1651 (C=N), 1447 (arom), 1094, 1038 (C-O-C), 849 and 737 cm^{-1} (arom); ^1H NMR (400 MHz, DMSO-d_6) δ 8.52 (1H, s, N=CH), 6.86 (2H, s, arom), 6.15 (1H, d, $J_{C1,OH}$ 4.3 Hz, C1-OH), 4.96 (1H, t, $J_{C1,OH}$≈$J_{1,2}$ 3.6 Hz, H-1), 4.86 (1H, d, $J_{C3,OH}$ 5.4 Hz, C3-OH), 4.68 (1H, d, $J_{C4,OH}$ 5.8 Hz, C4-OH), 4.45 (1H, t, $J_{C6,OH}$ 5.8 Hz, C6-OH), 3.77 (2H, m, $J_{2,3}$≈$J_{3,4}$ 9.2 Hz, H-3, H-6), 3.67 (1H, dd, $J_{5,6}$≈$J_{C6,OH}$ 5.3 Hz, $J_{6,6'}$ 9.9 Hz, H-6´), 3.53 (1H, m, $J_{5,6}$ 5.8 Hz, H-5), 3.19 (1H, m, $J_{4,5}$ 3.7 Hz, $J_{3,4}$ 9.1 Hz H-4), 3.08 (1H, dd, $J_{1,2}$ 2.9 Hz, $J_{2,3}$ 9.6 Hz, H-2), 2.35 (6H, s, CH_3), 2.23 (3H, s, CH_3). ^{13}C NMR (100 MHz, DMSO-d_6): 162.01 (N=C), 138.08 (arom), 137.65 (2 C, arom), 131.49 (2 C, arom), 129.09 (2 C, arom), 93.44 (C-1), 76.43 (C-2), 72.69 (C-5), 71.16 (C-3), 70.91 (C-4), 61.51 (C-6), 20.98 (2C, CH_3), 20.50 (1C, CH_3). Anal. Calcd. for $C_{16}H_{23}NO_5$: C, 62.14, H, 7.44, N, 4.53. Found: C, 62.21, H, 7.50, N, 4.64.

***2-Deoxy-2-[(*E*)-(2,4,6-trimethylbenzylidene)amino]-β-D-glucopyranose* (30).** Method 1 (84%). ^1H NMR (400 MHz, DMSO-d_6) δ 8.40 (1H, s, N=CH), 6.86 (2H, s, arom), 6.57 (1H, d, $J_{1,OH}$ 7.0 Hz, C1-OH), 4.92 (1H, d, $J_{4,OH}$ 5.1 Hz, C4-OH), 4.84 (1H, d, $J_{3,OH}$ 5.8 Hz, C3-OH), 4.71 (1H, t, $J_{1,2}$=J_{1-OH} 7.3 Hz, H-1), 4.56 (1H, t, $J_{6,OH}$=$J_{6',OH}$ 5.5 Hz, C6-OH), 3.74 (1H, dd, $J_{6-6'}$ 11.0 Hz, H-6), 3.49 (1H, dd, $J_{6',OH}$ 5.6 Hz, $J_{6,6'}$ 11.4 Hz, H-6´), 3.43 (1H, m, H-3), 3.24 (1H, m, $J_{4,5}$ 9.2 Hz, H-5), 3.17 (1H, m, $J_{3,4}$=$J_{4,5}$ 9.2 Hz, H-4), 2.82 (1H, t, $J_{1,2}$=$J_{2,3}$ 8.4 Hz, H-2), 2.34 (6H, s, CH_3), 2.23 (3H, s, CH_3). ^{13}C NMR (100 MHz, DMSO-d_6): 161.96 (N=C), 137.97 (arom), 137.21 (2C, arom), 131.58 (2C, arom), 129.06 (2C, arom), 95.88 (C-1), 79.44 (C-2), 77.17 (C-5), 74.62 (C-3), 70.63 (C-4), 61.50 (C-6), 20.94 (CH_3), 20.47 (2 CH_3). HRMS [M+H$^+$] calculated for $C_{16}H_{23}NO_5$: 310.1649. Found: 310.1652.

2-Deoxy-2-[(*E*)-(2-hydroxybenzylidene)amino]-α,β-D-glucopyranose* (32 and 33).** To a solution of D-glucosamine hydrochloride (5.0 g, 23.2 mmol) in water (50 mL) was added sodium bicarbonate (3.2 g, 37.5 mmol), and after 10 min, *o*-salicylaldehyde (3.2 mL). The mixture was stirred vigorously at room temperature for 4 h, albeit a crystalline solid appeared after 30 min. That solid was filtered, washed with cold water, and dried (6.5 g, 99%). M.p. 185-187 ºC; [α]D +109.2º; [α]578 +115.0º; [α]546 +136.4º (*c* 0.5, pyridine); [Lit.[21] m.p. 183.5 ºC, [α]D +11.0º; lit.[70] m.p. 182-183 ºC]; IR (KBr) $\bar{\nu}_{máx}$ 3383 (OH), 1632 (C=N), 1493 (arom), 1155, 1099, 1011 (C-O) and 767 cm^{-1} (arom). ^1H and ^{13}C NMR spectra showed the existence of α and β anomers (~1:2 ratio). ***α-Anomer (32): ^1H NMR (400 MHz, DMSO-d_6) δ 13.37 (1H, s, OH-arom), 8.48 (1H, s, N=CH), 7.42 (1H, dd, arom), 7.34 (1H, c, arom), 6.89 (2H, m, arom), 6.57 (1H, d, $J_{1,OH}$ 4.4 Hz, C1-OH), 5.04 (1H, d, $J_{1,2}$ 3.4 Hz, H-1), 5.02 (1H, m, C3-OH), 4.47 (1H, t, C6-OH), 3.67 (1H, dd, H-6), 3.51-3.47 (3H, m, H-4, 5 y 6´), 3.43 (1H, dt, $J_{2,3}$ 9.2 Hz, $J_{3,4}$ 3.8 Hz, H-3), 3.17 (1H, t, $J_{2,3}$ 9.8 Hz, H-2). ^{13}C NMR (100 MHz, DMSO-d_6): 166.90 (N=C), 162.40, 132.78, 132.10, 118.81, 118.18, 117.28 (C-arom), 92.36 (C-1), 72.60 (C-2), 71.27 (C-5), 70.88 (C-3), 70.46 (C-4), 61.44 (C-6). ***β-Anomer (33):*** ^1H NMR (400 MHz, DMSO-d_6) δ 13.37 (1H, s, OH-arom), 8.41 (1H, s, N=CH), 7.48 (1H, dd, arom), 7.43 (1H, t, arom), 6.89 (2H, m, arom), 6.77 (1H, d, $J_{1,OH}$ 6.3 Hz, C1-OH), 5.11 (1H, m, $J_{4,OH}$ 5.1 Hz, C4-OH), 5.02 (1H, m, C3-OH), 4.71 (1H, t, $J_{1,OH}$=$J_{1,2}$ 6.7 Hz, H-1), 4.58 (1H, t, C6-OH), 3.71 (1H, dd, $J_{6,6'}$ 11.9 Hz, $J_{5,6}$ 2.0 Hz, H-6), 3.48 (1H, dd, H-6´), 3.43 (1H, td, H-3), 3.28 (1H, m, H-5), 3.17 (1H, t, $J_{3,4}$=$J_{4,5}$ 9.7 Hz, H-4), 2.86 (1H, dd, $J_{1,2}$ 8.1 Hz, $J_{2,3}$ 9.1 Hz, H-2). ^{13}C

NMR (100 MHz, DMSO-d_6): 167.10 (N=C), 160.79, 132.59, 132.02, 119.16, 118.81, 116.68 (C-arom), 95.57 (C-1), 77.16 (C-2), 76.75 (C-5), 74.93 (C-3), 70.46 (C-4), 61.44 (C-6).

2-Deoxy-2-[(*E*)-(2,4-dihydroxybenzylidene)amino]-α-D-glucopyranose* (34).** To a solution of D-glucosamine hydrochloride (10.0 g, 46.4 mmol) in water (60 mL) was added sodium bicarbonate (5.0 g, 59.5 mmol) and, after 10 min, 2,4-dihydroxybenzaldehyde (6.90 g, 50.0 mmol) and methanol (10 mL), and the mixture was kept at room temperature under vigorous stirring for 2 h. A solid crystallized from the red solution, which was collected by filtration, and washed successively with cold water, cold ethanol, and ethyl ether, and dried under vacuum (13.6 g, 98%). M.p. 120-122 ºC; $[\alpha]_D$ +169.6º; $[\alpha]_{578}$ +179.0º; $[\alpha]_{546}$ +211.4º (*c* 0.5, pyridine); [Lit.[22] m.p. 116 ºC, $[\alpha]_D$ +132º (methanol)]; IR (KBr) $\bar{\nu}_{máx}$ 3243 (OH), 1628 (C=N), 1483 (arom), 1144, 1040 (C-O), 860 and 783 (arom); ^{1}H NMR (400 MHz, DMSO-d_6) δ 13.88 (1H, s, OH-arom), 8.18 (1H, s, N=CH), 7.12 (1H, d, arom), 6.77 (1H, sa, C1-OH), 6.13 (1H, d, arom), 6.03 (1H, s, arom), 5.13 (1H, bs, OH), 5.06 (1H, d, $J_{1,2}$ 2.6 Hz, H-1), 3.70 (1H, t, $J_{2,3}$=$J_{3,4}$ 11.3 Hz, H-3), 3.59-3.47 (3H, m, H-5, 6 y 6´), 3.18 (1H, ddd, H-2), 3.16 (1H, c, $J_{3,4}$=$J_{4,5}$ 9.2 Hz, H-4). ^{13}C NMR (100 MHz, DMSO-d_6): 170.93 (arom), 164.42 (N=C), 163.56, 134.67, 111.00, 106.73, 103.84 (arom), 91.85 (C-1), 72.68 (C-2), 71.52 (C-5), 70.90 (C-3), 69.36 (C-4), 61.48 (C-6). ***β-Anomer (35): ^{1}H NMR (400 MHz, DMSO-d_6) δ 13.88 (1H, s, OH-arom), 8.22 (1H, s, N=CH), 7.22 (1H, d, arom), 6.77 (1H, sa, C1-OH), 6.29 (1H, d, arom), 6.19 (1H, s, arom), 5.13 (1H, sa, OH), 4.65 (1H, d, $J_{1,OH}$=$J_{1,2}$ 7.7 Hz, H-1), 3.64-3.59 (1H, m, $J_{6,6'}$ 10.2 Hz, H-6), 3.55-3.47 (2H, m, H-4 y 6´), 3.40 (1H, t, $J_{2,3}$=$J_{3,4}$ 9.0 Hz, H-3), 3.24 (1H, m, H-5), 2.80 (1H, t, $J_{1,2}$=$J_{2,3}$ 8.6 Hz, H-2). ^{13}C NMR (100 MHz, DMSO-d_6): 166.27 (N=C), 164.96, 161.96, 133.73, 111.85, 107.28, 102.82 (C-arom), 95.70 (C-1), 77.14 (C-2), 75.71 (C-5), 75.11 (C-3), 70.60 (C-4), 61.48 (C-6).

***2-Deoxy-2-[(*E*)-(2,4-dihydroxybenzylidene)amino]-α-D-glucopyranose hydrochloride* (36).**[22] 2-Deoxy-2-(2,4-dihydroxybenzylidene)amino-α-D-glucopyranose (0.28 g, 0.93 mmol) was dissolved in hot acetone and 5*N* HCl solution (0.25 mL) was added. The white product formed was filtered and dried in vacuo over silica gel (0.22 g, 71%). IR (KBr) 3410, 3302 (OH), 1645 (C=N), 1601, 1508 (arom), 1150, 1034 (C-O), 856 and 698 (arom). ^{1}H and ^{13}C NMR spectra showed the presence of α and β anomers (~3:1 ratio). ***α-Anomer* (36)**: ^{1}H NMR (400 MHz, DMSO-d_6) δ 11.68 (1H, s, OH-arom), 10.75 (1H, s, OH), 8.69 (1H, d, $J_{CH=,2}$ 15,4 Hz, N=CH), 7.78 (1H, d, arom), 7.34 (1H, bs, NH), 6.75 (1H, d, arom), 6.56 (1H, dd, arom), 5.26 (1H, d, $J_{1,2}$ 2.1 Hz, H-1), 3.65 (3H, m, H-2, H-3, H-6´), 3.54 (1H, dd, $J_{5,6}$ 5.2 HZ $J_{6,6'}$ 11.9 Hz, H-6), 3.24 (1H, t, $J_{3,4}$=$J_{4,5}$ 9.0 Hz, H-4). ^{13}C NMR (100 MHz, DMSO-d_6): 168.66 (C-arom), 166.28 (N=C), 164.88 (C-arom), 110.69, 110.59, 106.90 (C-arom), 89.88 (C-1), 72.73 (C-2), 70.18 (C-3, C-5), 65.96 (C-4), 60.82 (C-6). ***β*-Anomer (37)**: ^{1}H NMR (400 MHz, DMSO-d_6) δ 11.68 (1H, s, OH-arom), 10.75 (1H, s, OH), 8.60 (1H, d, N=CH), 7.93 (1H, d, arom), 7.78 (1H, d, arom), 7.34 (1H, bs, NH), 6.71 (1H, d, arom), 6.52 (1H, dd, arom), 4.27 (1H, d, $J_{1,2}$ 8.0 Hz, H-1), 3.64 (3H, m, H-2, H-3, H-6´), 3.48 (1H, dd, $J_{5,6}$ 5.1 HZ $J_{6,6'}$ 11.7 Hz, H-6), 3.36 (1H, t, H-5). ^{13}C NMR (100 MHz, DMSO-d_6): 168.66 (C-arom), 166.28 (N=C), 164.88 (C-arom), 137.83 (C-arom), 110.69, 106.90, 102.79 (C-arom), 93.33 (C-1), 77.00 (C-2), 72.57 (C-3), 71.13 (C-5), 70.55 (C-4), 60.82 (C-6).

***(*E*)-2-[*N*-(2-Deoxy-α,β-D-glucopyranos-2-yl)aminomethylene]-6,8-dihydroxy-1*H*-xanthene-1,3(2*H*)-dione* (39, 41) *and (*Z*)-2-[*N*-(2-deoxy-α,β-D-glucopyranos-2-yl)aminomethylene]-6,8-dihydroxy-1*H*-xanthene-1,3(2*H*)-dione* (40, 42).**[23] Following method 3 and from 2,4,6-

trihydroxybenzaldehyde, a mixture of **39**-**42** was obtained (89%), m.p. 266-268 °C; IR (KBr) $\bar{\nu}_{max}$/cm^{-1} 3500-3000 (OH, NH), 1650 and 1590 (C=O and C=C enamine), 1254, 1207, 1074, 1028 (C-O), 824. **Spectroscopic data for 39 and 40**: ^{1}H NMR (500 MHz, DMSO-*d*$_6$) δ 11.49 (dd, $J_{2,NH}$ = 9.5 Hz, $J_{NH,=CH}$ = 14.0 Hz, 1H, NH, **39**), 11.40 (dd, $J_{2,NH}$ = 9.5 Hz, $J_{NH,=CH}$ = 14.0 Hz, 1H, NH, **40**), 8.23 (d, *J* = 14.0 Hz, 1H, CHN, **39**), 8.20 (d, *J* = 14.0 Hz, 1H, CHN, **40**), 8.19 (t, *J* = 1.0 Hz, 1H, H-arom, **39**), 8.17 (t, *J* = 0.5 Hz, 1H, H-arom, **40**), 7.09 (bs, 1H, C1-OH, **39**), 7.04 (sa, 1H, C1-OH, **40**), 6.18 (s, 2H, H-arom), 6.09 (s, 1H, H-arom), 5.53 (d, *J* = 1.5 Hz, 1H, H-arom, **39**), 5.50 (d, *J* = 1.5 Hz, 1H, H-arom, **40**), 5.31 (bs, 2H, OH), 5.17 (d, $J_{1,2}$ = 3.0 Hz, 1H, H-1, **39**), 5.15 (d, $J_{1,2}$ = 3.0 Hz, 1H, H-1, **40**), 4.55 (bs, 2H, OH), 3.66 (m, 4H, H-4, H-6, **39**/**40**), 3.53 (m, 2H, H-6', **39**/**40**), 3.47 (m, 2H, H-3, **39**/**40**), 3.39 (td, $J_{1,2}$ = 3.5 Hz, $J_{2,3}$ = $J_{2,NH}$ = 9.5 Hz, 2H, H-2, **39**), 3.36 (td, $J_{1,2}$ = 3.5 Hz, $J_{2,3}$ = $J_{2,NH}$ = 9.5 Hz, 2H, H-2, **40**), 3.17 (td, 1H, $J_{4,5}$ = $J_{5,6'}$ = 9.0 Hz, $J_{5,6}$ = 3.5 Hz, 2H, H-5, **39**/**40**), 3.16 (t, 1H, *J* = 9.0 Hz, H-5, **40**); ^{13}C NMR (125 MHz, DMSO-*d*$_6$) δ 186.32, 182.77 (CO), 180.00, 178.42, 160.68, 160.64 (C-OH), 159.54 (CN, **39**), 159.38 (CN, **40**), 155.68, 131.39, 130.47, 116.86, 107.22, 106.83, 103.17, 99.30, 99.14, 99.07 (C-arom, **39**/**40**), 90.81(C-1, **40**), 90.77 (C-1, **40**), 72.92 (C-2, **39**), 72.89 (C-2, **40**), 71.96 (C-3, **39**), 71.91 (C-3, **40**), 70.72 (C-5, **39**), 70.75 (C-5, **40**), 64.89 (C-4, **39**/**40**), 61.38 (C-6, **39**), 61.34 (C-6, **40**). **Spectroscopic data for 41 and 42**: ^{1}H NMR (500 MHz, DMSO-*d*$_6$) δ 11.47 (dd, 1H, NH), 11.42 (dd, 1H, NH), 8.15 (dd, 1H, H-arom), 8.13 (dd, 1H, H-arom), 7.09 (s, 1H, OH), 7.04 (s, 1H, OH), 6.09 (s, 2H, H-arom), 6.02 (s, 2H, H-arom), 5.56 (d, *J* = 1.5 Hz, 1H, H-arom, **41**), 5.51 (d, *J* = 1.5 Hz, 1H, H-arom, **42**), 4.74 (d, $J_{1,2}$ = 8.0 Hz, 1H, H-1, **41**), 4.70 (d, $J_{1,2}$ = 8.0 Hz, 1H, H-1, **42**), 3.22 (m, 2H, H-5, **41**/**42**), 3.05 (c, $J_{1,2}$ = $J_{2,3}$ = $J_{2,NH}$ = 9.0 Hz, 1H, H-2, **41**), 3.03 (c, $J_{1,2}$ = $J_{2,3}$ = $J_{2,NH}$ = 9.0 Hz, 1H, H-2, **42**), ^{13}C NMR (125 MHz, DMSO-*d*$_6$) δ 94.44 (C-1), 68.36 (C-2). The above assignments for **39**/**40** and **41**/**42** pairs can be interchanged. HRMS [M+H$^+$] calculated for $C_{20}H_{19}NO_{10}$: 434.1082, found: 434.1061; [M$_2$+H$^+$] calculated 867.2091, found 867.2036.

***2-Deoxy-2-[(*E*)-(4-methoxybenzylidene)amino]-β-D-*glycero-L-gluco*-heptopyranose* (9).** Method 4. M.p. 189-190 ºC (Lit.[5a] 190-192 ºC); ^{1}H NMR (400 MHz, DMSO-*d*$_6$) δ 8.11 (1H, s, CH=N), 7.68 (2H, d, *J* 8.8 Hz, H-arom), 6.98 (2H, d, *J* 8.8 Hz, H-arom), 6.50 (1H, bs, OH-1), 4.84 (1H, d, $J_{3,OH}$ 3.2 Hz, OH-3), 4.78 (1H, d, $J_{4,OH}$ 3.6 Hz, OH-4), 4.65 (1H, d, $J_{1,2}$ 7.2 Hz, H-1), 4.48 (1H, t, OH-7), 4.28 (1H, d, $J_{6,OH}$ 6.4 Hz, OH-6), 3.79 (3H, s, OCH_3), 3.76 (1H, m, H-6), 3.43 (2H, m, H-3, H-4, H-7, H-7'), 3.26 (1H, d, $J_{4,5}$ 8.8 Hz H-5), 2.78 (1H, t, $J_{1,2}$≈$J_{2,3}$ 8.8 Hz, H-2); ^{13}C NMR (100 MHz, DMSO-*d*$_6$): 161.35 (C=N), 161.22 (C-arom), 132.50 (C-arom), 129.84 (2C-arom), 114.09 (2 C-arom), 96.23 (C-1), 78.46 (C-2), 75.03 (C-3), 74.67 (C-5), 69.39 (C-4), 68.88 (C-6), 62.69 (C-7).

Schiff Bases Derived from *ortho*-Hydroxynaphthaldehydes.

***2-Deoxy-2-{[(Z)-(2-oxo-1,2-dihydro-1-naphthyliden)methyl]amino}-α-D-glucopyranose* (93).** A solution of 2-hydroxy-1-naphthaldehyde (4.2 g, 22.3 mmol) in methanol (270 mL) was added to a solution of 2-amino-2-deoxy-D-glucopyranose hydrochloride (8.4 mmol) and sodium acetate (1.5 g, 18.3 mmol) in water (18 mL). After 3 h in the dark at room temperature, the yellow solution was evaporated to dryness under reduced pressure and maintaining the temperature between

10-15 ºC. The excess aldehyde was removed by extracting the residue three times with chloroform-ethyl ether (3:1 v/v). After solvent evaporation, the residue was newly extracted with water at 0 °C (3x10 mL) to remove the salts. The resulting residue was dried and crystallized from methanol (96%); m.p. 201-205 ºC; $[\alpha]_D$ +165.6º; $[\alpha]_{578}$ +179.6º; $[\alpha]_{546}$ +231.4º (*c* 0.5, pyridine); [Lit.[20b] M.p. 202-203 ºC, $[\alpha]_{546}$ +274º (methanol)]; IR (KBr) $\overline{\nu}_{máx}$: 3244 (OH), 1636 (C=O, C=C), 1545, 1487 (arom.), 831 and 743 cm^{-1} (arom.). ^{1}H NMR (400 MHz, DMSO-d_6) δ 13.65 (1H, t, $J_{NH,CH}$=$J_{NH,H2}$ 8.9 Hz, NH), 8.92 (1H, d, $J_{CH,NH}$ 11.6 Hz, =CH), 7.97 (1H, d, arom), 7.69 (1H, d, arom), 7.60 (1H, d, arom), 7.40 (1H, t, arom), 7.16 (1H, t, arom), 7.01 (1H, d, $J_{1,OH}$ 4.8 Hz, C1-OH), 6.65 (1H, d, arom), 5.34 (1H, d, $J_{4,OH}$ 5.3 Hz, C4-OH), 5.20 (1H, t, $J_{1,2}$=$J_{1,OH}$ 3.8 Hz, H-1), 5.10 (1H, d, $J_{3,OH}$ 5.4 Hz, C3-OH), 4.54 (1H, t, $J_{6,OH}$ 5.5 Hz, C6-OH), 3.70 (1H, dd, $J_{6,6'}$ 11.1 Hz, $J_{6,OH}$ 4.3 Hz, H-6), 3.60 (1H, m, H-3), 3.56 (1H, dd, $J_{6,6'}$ 11.6 Hz, $J_{6',OH}$ 5.6 Hz, H-6´), 3.53 (1H, dd, $J_{1,2}$ 3.3 Hz, $J_{2,3}$ 9.9 Hz, H-2), 3.48 (1H, m, H-5), 3.21 (1H, m, $J_{3,4} \approx J_{4,5}$ 9.9 Hz, $J_{4,OH}$ 5.9 Hz, H-4). ^{13}C NMR (100 MHz, DMSO-d_6): 179.46 (C=O), 160.54 (C=C), 137.64, 134.85, 129.07, 128.10, 126.57, 125.75, 124.42, 122.14, 118.28, 105.42 (C-arom.), 90.99 (C-1), 72.63 (C-2), 71.64 (C-5), 70.68 (C-3), 65.52 (C-4), 61.11 (C-6). Anal. Calcd. for $C_{17}H_{19}NO_6$: C, 61.25, H, 5.75, N, 4.20; Found: C, 60.94, H, 5.63, N, 4.11.

***2-Deoxy-2-{[(Z)-(2-oxo-1,2-dihydro-1-naphthylidene)methyl]amino}-α-D-glycero-L-gluco-heptopyranose* (94).** It was obtained using the procedure described for **93** (89%); m.p. 191-192 ºC; $[\alpha]_D$ -207.8º; $[\alpha]_{578}$ -224.6º; $[\alpha]_{546}$ -292.6º (*c* 0.5, pyridine); [Lit.[3b] M.p. 202-203 ºC, $[\alpha]_{546}$ +274º (methanol)]; IR (KBr) $\overline{\nu}_{máx}$ 3100-3300 (OH), 1638 (C=O, C=C), 1539 (arom.), 1044 (C-O), 829 y 743 cm^{-1} (arom.); ^{1}H NMR (400 MHz, DMSO-d_6) δ 13.63 (1H, dd, $J_{NH,CH}$ 11.2 Hz, $J_{NH,H2}$ 8.8 Hz, NH), 8.92 (1H, d, $J_{CH,NH}$ 12.0 Hz, =CH), 7.98 (2H, d, *J* 7.6 Hz, arom), 7.69 (1H, d, *J* 9.6 Hz, arom), 7.59 (1H, d, *J* 7.6 Hz, arom), 7.40 (1H, t, *J* 7.8 Hz, arom), 7.16 (1H, t, *J* 7.4 Hz, arom), 6.87 (1H, d, $J_{1,OH}$ 4.0 Hz, C1-OH), 6.64 (1H, d, *J* 9.6 Hz, arom), 5.30 (1H, d, $J_{3,OH}$ 5.2 Hz, C3-OH), 5.19 (1H, t, $J_{1,2}$=$J_{1,OH}$ 4.0 Hz, H-1), 5.00 (1H, d, $J_{4,OH}$ 6.4 Hz, C4-OH), 4.43 (1H, t, $J_{7,OH}$ 5.6 Hz, C7-OH), 4.30 (1H, d, $J_{6,OH}$ 7.2 Hz, C6-OH), 3.83 (1H, c, $J_{6,OH} \approx J_{6,7} \approx J_{6,7'}$ 6.8 Hz, H-6), 3.74 (1H, d, $J_{4,5}$ 10.4 Hz, H-5), 3.61 (1H, dt, $J_{3,OH}$ 5.5 Hz, $J_{3,4} \approx J_{2,3}$ 9.2 Hz, H-3), 3.46 (4H, m, H-2, H-4, H-7, H-7'). ^{13}C NMR (100 MHz, DMSO-d_6): 179.55 (C=O), 158.57 (C=C), 137.68, 134.89, 129.10, 128.13, 126.65, 125.10, 122.16, 118.31 (C-arom), 105.42 (C=C), 91.05 (C-1), 71.93 (C-2), 70.56 (C-3), 69.86 (C-5), 69.02 (C-4), 65.50 (C-6), 63.10 (C-7). Anal. Calcd. for $C_{18}H_{21}NO_7$: C, 59.50, H, 5.83, N, 3.85; Found: C, 59.42; H, 5.70; N. 3.91.

Per-*O*-acetyl-2-[(*E*)-(arylmetilen)amino]-2-deoxy-β-D-glucopyranoses.

***1,3,4,6-Tetra-O-acetyl-2-[(E)-benzylidenamino]-2-deoxy-β-D-glucopyranose* (43).** Acetic anhydride (9.0 mL) was added to a suspension of 2-[(*E*)-benzylidenamino]-2-deoxy-β-D-glucopyranose (**11**) (7.1 mmol) in pyridine (9.4 mL) with stirring and external cooling, and the mixture was left at room temperature until dissolution. Then it was poured onto ice-water (*ca*. 300 mL) with stirring. The solid formed was collected by filtration and washed repeatedly with cold water and dried over silica gel (82%). Recrystallized from ethanol; m.p. 160-161 ºC; $[\alpha]_D$ +79.0º;

[α]$_{578}$ +82.2°; [α]$_{546}$ +97.0°; [α]$_{436}$ +204.2°; [α]$_{365}$ +436.0° (*c* 0.5, chloroform); IR (KBr) $\bar{\nu}_{máx}$ 2916 (OCH_3), 1751 (C=O), 1647 (C=N), 1581 (arom), 1217 (C-O-C, ester), 1031 cm^{-1} (C-O); ^{1}H NMR (400 MHz, $CDCl_3$) δ 8.24 (1H, s, N=CH), 7.70 (2H, d, H-arom), 7.40 (3H, m, H-arom), 5.97 (1H, d, $J_{1,2}$ 8.3 Hz, H-1), 5.45 (1H, t, $J_{2,3}$=$J_{3,4}$ 9.7 Hz, H-3), 5.16 (1H, t, $J_{3,4}$=$J_{4,5}$ 9.8 Hz, H-4), 4.39 (1H, dd, $J_{5,6}$ 4.5 Hz, $J_{6,6'}$ 12.4 Hz, H-6'), 4.14 (1H, dd, $J_{6,6'}$ 12.4 Hz, $J_{5,6'}$ 1.9 Hz, H-6'), 3.99 (1H, ddd, $J_{4,5}$ 10.1 Hz, $J_{5,6}$ 4.4 Hz, $J_{5,6'}$ 2.0 Hz, H-5), 3.50 (1H, dd, $J_{1,2}$ 8.4 Hz, $J_{2,3}$ 9.7 Hz, H-2), 2.10, 2.04, 2.02, 1.89 (4x3H, s, CH_3); ^{13}C NMR (100 MHz, $CDCl_3$): 170.66, 169.87, 169.49, 168.70 (C=O), 165.11 (N=C), 135.30, 131.50, 128.67, 128.56 (C-arom), 93.04 (C-1), 73.09 (C-2), 72.96 (C-5), 72.78 (C-3), 67.99 (C-4), 61.77 (C-6), 20.75, 20.66, 20.47 (CH_3). Anal. Calcd. for $C_{21}H_{25}NO_9$: C, 57.93, H, 5.79, N, 3.22; Found: C, 57.63, H, 5.73, N, 2.94.

***1,3,4,6-Tetra-*O*-acetyl-2-deoxy-2-[(*E*)-(4-methoxybenzylidene)amino]-β-D-glucopyranose* (44).** It was obtained from **12** using the procedure described for **43** (78%), the title compound crystallized from ethanol and had m.p. 181-183 °C; [α]$_D$ +82.2°; [α]$_{578}$ +86.4°; [α]$_{546}$ +103°; [α]$_{436}$ +221.8°; (*c* 0.5, chloroform); [Lit.[6] m.p. 188 °C, [α]$_D$ +98.6° (chloroform)]; IR (KBr) $\bar{\nu}_{máx}$ 2920 (OCH_3), 1750 (C=O), 1649 (C=N), 1607, 1514 (arom), 1219 (C-O-C, ester), 1165, 1082, 1034 (C-O), 897 and 833 cm^{-1} (arom); ^{1}H NMR (400 MHz, $CDCl_3$) δ 8.16 (1H, s, N=CH), 7.66 (2H, d, H-arom), 6.92 (2H, d, H-arom), 5.95 (1H, d, $J_{1,2}$ 8.3 Hz, H-1), 5.43 (1H, t, $J_{3,4}$=$J_{2,3}$ 9.6 Hz, H-3), 5.15 (1H, t, $J_{3,4}$=$J_{4,5}$ 9.8 Hz, H-4), 4.38 (1H, dd, $J_{5,6}$ 4.5 Hz, $J_{6,6'}$ 12.4 Hz, H-6), 4.13 (1H, dd, $J_{5,6'}$ 2.0 Hz, $J_{6,6'}$ 12.4 Hz, H-6´), 3.97 (1H, dc, $J_{4,5}$ 10.1 Hz, $J_{5,6}$ 4.5 Hz, $J_{5,6'}$ 1.9 Hz, H-5), 3.84 (3H, s, OCH_3), 3.45 (1H, t, $J_{1,2}$≈$J_{2,3}$ 8.6 Hz, H-2), 2.12, 2.05, 2.02, 1.88 (4x3H, s, CH_3). ^{13}C NMR (100 MHz, $CDCl_3$): 170.69, 169.90, 169.54, 168.77 (C=O), 164.29 (N=C), 162.29, 130.24, 128.26, 114.29, 114.04 (C-arom), 93.14 (C-1), 73.23 (C-2), 72.93 (C-5), 72.73 (C-3), 68.02 (C-4), 61.80 (C-6), 55.39 (OCH_3), 20.79, 20.74, 20.68, 20.50 (CH_3). Anal. Calcd. for $C_{22}H_{27}NO_{10}$: C, 56.77, H, 5.85, N, 3.01; Found: C, 56.50, H, 5.69, N, 3.16.

***1,3,4,6,7-Penta-*O*-acetyl-2-deoxy-2-[(*E*)-(4-methoxybenzylidene)amino]-β-D-*glycero-*L*-gluco-heptopyranose* (10).**[5a] It was obtained from **9** using the procedure described for **43**; m.p. 187-189 °C. ^{1}H NMR (400 MHz, $CDCl_3$) δ 8.16 (1H, s, CH=N), 7.66 (2H, d, *J* 8.8 Hz, arom), 6.91 (2H, d, *J* 8.8 Hz, arom), 5.87 (1H, d, $J_{1,2}$ 8.4 Hz, H-1), 5.42 (1H, t, $J_{3,4}$=$J_{4,5}$ 9.8 Hz, H-4), 5.35 (1H, ddd, $J_{5,6}$ 2.4 Hz, $J_{6,7}$ 5.2 Hz, $J_{6,7'}$ 7.6 Hz, H-6), 5.13 (1H, t, $J_{2,3}$=$J_{3,4}$ 10.0 Hz, H-3), 4.35 (1H, dd, $J_{6,7}$ 5.2 Hz, $J_{7,7'}$ 11.5 Hz, H-7), 4.17 (1H, dd, $J_{6,7'}$ 8.0 Hz, $J_{7,7'}$ 11.6 Hz, H-7'), 4.01 (1H, dd, $J_{4,5}$ 9.8 Hz, $J_{5,6}$ 1.8 Hz, H-5), 3.84 (3H, s, OCH_3), 3.46 (1H, t, $J_{1,2}$≈$J_{2,3}$ 8.8, H-2), 2.12, 2.06, 2.02, 2.01, 1.88 (5x3H, s, CH_3); ^{13}C NMR (100 MHz, $CDCl_3$): 170.41, 170.14, 169.89, 169.44, 168.53 (acetate), 164.25 (N=C), 162 28 (arom), 130.23 (2C, arom) 128.16, 114.0 (2C, arom), 93.53 (C-1), 73.27 (C-2), 72.91 (C-3), 72.75 (C-5), 67.14 (C-4), 66.69 (C-6), 62.05 (C-7), 55.35 (OCH_3), 20.77, 20.65, 20.59, 20.53, 20.44 (CH_3). HRMS [M+H$^+$] calculated for $C_{25}H_{31}NO_{12}$: 538.1919. Found: 538.1929.

Per-*O*-acetyl-2-[(*E*)-(arylmetilene)amino]-2-deoxy-α-D-glucopyranoses

***G*eneral Method.** To a suspension of **45** (1.4 g, 3.0 mmol) in 96% ethanol (14 mL) a solution of sodium acetate trihydrate (0.41 g, 3.0 mmol) in water (2 mL) and pyridine (0.8 mL) was added. Then, the corresponding aromatic aldehyde (0.6 mL) was added and the mixture heated for a few minutes in a boiling water bath. The solution was filtered to remove impurities and on cooling, crystalline solids were generally obtained, which were collected by filtration, washed with 50% aqueous ethanol, and dried in vacuum over silica gel.

***1,3,4,6-Tetra-*O*-acetyl-2-[(*E*)-benzylideneamino]-2-deoxy-α-D-glucopyranose* (46).** The title compound was obtained in 64% yield using the general method. M.p. 156-158 ºC; $[\alpha]_D$ +61.6º; $[\alpha]_{578}$ +64.2º; $[\alpha]_{546}$ +74.4º, $[\alpha]_{436}$ +121.2º (*c* 0.5, chloroform); IR (KBr) $\overline{\nu}_{máx}$ 1753 (C=O), 1643 (C=N), 1582 (arom), 1221 (C-O-C), 1152, 1032 (C-O), 760 and 694 cm^{-1} (arom); ^1H NMR (400 MHz, Cl_3CD) δ 8.30 (1H, s, CH=), 7.68 (2H, d, arom), 7.42 (3H, m, arom), 6.24 (1H, d, $J_{1,2}$ 3.6 Hz, H-1), 5.63 (1H, t, $J_{3,4}$=$J_{4,5}$ 9.8 Hz, H-4), 5.19 (1H, t, $J_{2,3}$=$J_{3,4}$ 9.9 Hz, H-3), 4.36 (1H, dd, $J_{5,6}$ 4.2 Hz, $J_{6,6'}$12.3 Hz, H-6), 4.27 (1H, ddd, $J_{5,4}$ 10.3 Hz, $J_{5,6}$ 4.0 Hz, $J_{5,6'}$ 2.1 Hz, H-5), 4.12 (1H, dd, $J_{5,6'}$ 2.0 Hz, $J_{6,6'}$ 12.3 Hz, H-6´), 3.71 (1H, dd, $J_{1,2}$ 3.6 Hz, $J_{2,3}$10.1 Hz, H-2), 2.22, 2.11, 2.05, 1.89 (4x3H, s, CH_3); ^{13}C NMR (100 MHz, Cl_3CD): 170.63 (C=O), 169.76 (2 C=O), 168.91 (C=O), 165.17 (CH=N), 135.30 (arom), 131.44 (arom), 128.58 (2C, arom), 128.51 (2C, arom), 91.64 (C-1), 70.98 (C-2), 70.90 (C-5), 70.05 (C-3), 68.23 (C-4), 61.83 (C-6), 20.95, 20.67, 20.62, 20.51 (CH_3). Anal. Calcd. for $C_{21}H_{25}NO_9$:; C, 57.93, H, 5.79, N, 3.22. Found: C, 58.06, H, 5.59, N, 3.01.

***1,3,4,6-Tetra-O-acetyl-2-deoxy-2-[(*E*)-(4-methoxybenzylidene)amino]-α-D-glucopyranose* (47).** It was obtained in 32% yield and had m.p. 181-183 ºC; $[\alpha]_D$ +104.2º; $[\alpha]_{578}$ +108.8º; $[\alpha]_{546}$ +126.0º, $[\alpha]_{436}$ +240.6º; $[\alpha]_{365}$ +474.2º (*c* 0.5, chloroform); [Lit.[36b] m.p. 174 ºC; $[\alpha]_D$ +123º (*c* 0.4, chloroform)]; IR (KBr) $\overline{\nu}_{máx}$ 2857 (OCH_3), 1746 (C=O), 1643 (C=N), 1617, 1514 (arom), 1250 (C-O-C), 1154, 1030 (C-O), 841 cm^{-1} (arom); ^1H NMR (400 MHz, $CDCl_3$) δ 8.21 (1H, s, CH=), 7.62 (2H, d, arom), 6.90 (2H, d, arom), 6.22 (1H, d, $J_{1,2}$ 3.6 Hz, H-1), 5.60 (1H, t, $J_{3,4}$=$J_{4,5}$ 9.8 Hz, H-4), 5.18 (1H, t, $J_{2,3}$=$J_{3,4}$ 9.8 Hz, H-3), 4.35 (1H, dd, $J_{5,6}$ 4.2 Hz, $J_{6,6'}$12.3 Hz, H-6), 4.25 (1H, ddd, $J_{4,5}$ 10.2 Hz, $J_{5,6}$ 4.0 Hz, $J_{5,6'}$ 2.1 Hz, H-5), 4.12 (1H, dd, $J_{5,6'}$ 2.1 Hz, $J_{6,6'}$ 12.3 Hz, H-6´), 3.84 (3H, s, OCH_3), 3.66 (1H, dd, $J_{1,2}$ 3.6 Hz, $J_{2,3}$10.1 Hz, H-2), 2.21, 2.10, 2.05, 1.88 (4x3H, s, CH_3);^{13}C NMR (100 MHz, Cl_3CD): 170.64 (C=O), 169.81 (2 C=O), 168.96 (C=O), 164.28 (CH=N), 162.23 (arom), 130.20 (2C, arom), 128.38 (arom), 113.97 (2C, arom), 91.80 (C-1), 71.14 (C-2), 70.87 (C-5), 70.05 (C-3), 68.30 (C-4), 61.90 (C-6), 55.34 (OCH_3), 21.00, 20.70, 20.65, 20.55 (CH_3). Anal. Calcd. for $C_{22}H_{27}NO_{10}$: C, 56.77, H, 5.85, N, 3.01. Found: C, 56.72, H, 5.76, N, 3.13.

Synthesis of Anomerically Unprotected Schiff Bases

General Method. To a suspension of **49**[26] (1.16 g, 3.0 mmol) in 96% ethanol (14 mL), anhydrous sodium acetate (0.25 g, 3.0 mmol) dissolved in water (2 mL) and the corresponding aldehyde (3.0 mmol) were added. The mixture was stirred for 5 min and then poured onto ice-water, and the aqueous phase was extracted with chloroform (3x50 mL). The organic layer was washed with saturated $NaHCO_3$ solution (50 mL) and water (50 mL), and dried with anhydrous magnesium sulfate. The organic phase was evaporated to dryness and the residue dried in vacuo over silica gel.

***3,4,6-Tri-*O*-acetyl-2-deoxy-2-[(*E*)-(4-methoxybenzylidene)amino]-α,β-D-glucopyranose* (50 and 51).** The anomeric mixture was obtained in 90% yield. IR (KBr) $\bar{\nu}_{máx}$ 1746 (C=O), 1643 (C=N), 1514 (arom), 1250 (C-O-C, ester), 1153, 1030 (C-O) y 841 cm^{-1} (arom). ^1H NMR spectrum showed the presence of α and β anomers (1.3:1 ratio). ***α-Anomer* (50):** ^1H NMR (400 MHz, Cl_3CD) δ 8.21 (1H, s, CH=), 7.66 (2H, m, arom), 6.90 (2H, m, arom), 5.54 (1H, t, $J_{2,3}$=$J_{3,4}$ 9.8 Hz, H-3), 5.23 (1H, d, $J_{1,2}$ 3.4 Hz, H-1), 5.12 (1H, t, H-4), 4.37 (1H, ddd, H-5), 4.28 (1H, dd, $J_{5,6}$ 4.8 Hz, $J_{6,6'}$ 12.1 Hz, H-6), 4.14 (1H, dd, H-6´), 3.71 (3H, s, OCH_3), 3.53 (1H, dd, H-2), 2.11, 2.04, 1.88, 1.87 (4x3H, s, CH_3). ***β-Anomer* (51):** ^1H NMR (400 MHz, Cl_3CD) δ 8.19 (1H, s, CH=), 7.66 (2H, m, arom), 6.90 (2H, m, arom), 5.40 (1H, t, $J_{2,3}$=$J_{3,4}$ 9.7 Hz, H-3), 5.14 (1H, t, H-4), 5.13 (1H, d, $J_{1,2}$ 8.1 Hz, H-1), 4.17 (1H, dd, $J_{5,6}$ 2.1 Hz, $J_{6,6'}$ 12.3 Hz, H-6), 4.14 (1H, dd, H-6´), 3.89 (1H, ddd, H-5), 3.29 (1H, dd, $J_{1,2}$ 7.8 Hz, $J_{2,3}$ 9.9 Hz, H-2), 2.12, 2.03, 1.88, 1.87 (4x3H, s, CH_3).

***3,4,6-Tri-*O*-acetyl-2-deoxy-2-[(*E*)-salicylidenamino]-α,β-D-glucopyranose* (52 and 53).** The anomeric mixture was obtained in 68% yield. IR (KBr) $\bar{\nu}_{máx}$ 3486 (OH), 1746 (C=O), 1630 (C=N), 1582, 1499 (arom), 1233 (C-O-C, ester), 1038 (C-O) and 760 cm^{-1} (arom, *o*-sustituted). ^1H NMR spectrum evidenced the presence of α and β anomers in ~1:1 ratio. ***α-Anomer* (52):** ^1H NMR (400 MHz, Cl_3CD) δ 11. 03 (OH, phenol), 8.33 (1H, s, CH=), 7.30 (1H, t, arom), 7.22 (1H, m, arom), 6.92 (1H, d, arom), 6.86 (1H, t, arom), 5.67 (1H, t, $J_{2,3}$=$J_{3,4}$ 9.8 Hz, H-3), 5.27 (1H, d, $J_{1,2}$ 3.4 Hz, H-1), 5.10 (1H, t, $J_{3,4}$=$J_{4,5}$ 9.8 Hz, H-4), 4.39 (1H, ddd, $J_{4,5}$ 10.1 Hz, H-5), 4.29 (1H, dd, $J_{5,6}$ 4.4 Hz, $J_{6,6'}$ 12.4 Hz, H-6), 4.12 (1H, dd, $J_{5,6'}$ 1.9 Hz, $J_{6,6'}$ 12.3 Hz, H-6´), 3.55 (1H, dd, $J_{1,2}$ 3.4 Hz, $J_{2,3}$ 10.3 Hz, H-2), 2.08, 2.00, 1.89, 1.88 (4x3H, s, CH_3). ***β-anomer* (53):** ^1H NMR (400 MHz, Cl_3CD) δ 11.03 (OH, phenol). 8.33 (1H, s, CH=), 7.30 (1H, t, arom), 7.22 (1H, m, arom), 6.92 (1H, d, arom), 6.86 (1H, t, arom), 5.41 (1H, t, $J_{3,4}$=$J_{2,3}$ 9.7 Hz, H-3), 5.10 (1H, t, $J_{4,5}$=$J_{3,4}$ 9.8 Hz, H-4), 4.97 (1H, d, $J_{1,2}$ 7.7 Hz, H-1), 4.25 (1H, dd, $J_{5,6}$ 4.8 Hz, $J_{6,6'}$ 12.8 Hz, H-6), 4.16 (1H, dd, $J_{5,6'}$ 2.1 Hz, $J_{6,6'}$ 12.2 Hz, H-6´), 3.86 (1H, ddd, H-5), 3.30 (1H, dd, $J_{1,2}$ 7.9 Hz, $J_{2,3}$ 9.9 Hz, H-2), 2.08, 2.00, 1.89, 1.88 (4x3H, s, CH_3).

***3,4,6-Tri-*O*-acetyl-2-deoxy-2-{[(*Z*)-(2-oxo-1,2-dihydro-1-naphthylidene)methyl]amino}-α-D-glucopyranose* (96).** It was obtained from **9** (80%), $[\alpha]_{578}$ +242.6º; $[\alpha]_{546}$ +332.2º; $[\alpha]_{436}$ +327.0º; $[\alpha]_{365}$ +320.6º (*c* 0.5, pyridine); IR (KBr) $\bar{\nu}_{máx}$ 1742 (C=O, acetate), 1632 (C=C, enamine), 1544 (arom), 1235 (C-O-C, ester), 1033 cm^{-1} (C-O); ^1H NMR (400 MHz, $CDCl_3$) δ 13. 67 (NH), 8.56 (1H, s, CH=), 7.73 (1H, d, *J* 11.6 Hz, arom), 7.67 (1H, d, *J* 8.8 Hz, arom), 7.57 (1H, d, *J* 8.0 Hz, arom), 7.41 (1H, *J* 7.4 Hz, t, arom), 7.25 (1H, m, *J* 7.6 Hz, arom), 6.81 (1H, d, *J* 9.6 Hz, arom), 5.64 (1H, t, $J_{2,3}$=$J_{3,4}$ 10.2 Hz, H-3), 5.46 (1H, d, $J_{1,2}$ 3.6 Hz, H-1), 5.14 (1H, t, $J_{3,4}$=$J_{4,5}$ 9.6 Hz, H-4), 4.39 (1H, ddd, $J_{5,6}$ 1.6 Hz, H-5), 4.45 (1H, dd, $J_{5,6}$ 4.4 Hz, $J_{6,6'}$ 12.4 Hz, H-6), 4.17 (1H, dd, $J_{5,6'}$ 1.6 Hz, $J_{6,6'}$ 12.4 Hz, H-6´), 3.71 (1H, dd, $J_{1,2}$ 3.6 Hz, $J_{2,3}$ 9.6 Hz, H-2), 2.15, 2.11, 2.06, 1.95, 1.93 (4x3H, s, CH_3). ***β-Anomer*:** ^1H NMR (400 MHz, $CDCl_3$) δ 9.05 (1H, s, CH=), 7.91 (1H, d, *J* 8.0 Hz, arom), 7.67 (1H, d, *J* 8.8 Hz, arom), 7.57 (1H, d, *J* 8.0 Hz, arom), 7.41 (1H, *J* 7.4 Hz, t, arom), 7.25 (1H, m, *J* 7.6 Hz, arom), 7.02 (1H, d, *J* 9.6 Hz, arom), 5.50 (1H, t, $J_{2,3}$=$J_{3,4}$ 9.6 Hz, H-3), 5.20 (1H, t, $J_{2,3}$=$J_{3,4}$ 9.6 Hz, H-4), 5.10 (1H, d, $J_{1,2}$ 7.2 Hz, H-1), 4.34 (1H, dd, $J_{5,6}$ 4.4 Hz, $J_{6,6'}$ 12.4 Hz,

H-6), 4.22 (1H, dd, $J_{5,6'}$ 1.6 Hz, H-6´), 3.92 (1H, ddd, $J_{5,6'}$ 2.2 Hz, $J_{5,6}$ 4.4 Hz, $J_{4,5}$ 8.0 Hz, H-5), 3.54 (1H, dd, $J_{1,2}$ 8.0 Hz, $J_{2,3}$ 10.0 Hz, H-2), 2.20, 2.10, 2.09, 2.01, 1.99 (4x3H, s, CH_3). Anal. Calcd. for $C_{23}H_{25}NO_9$: C, 61.47; H, 5.57; N, 3.12. Found: C, 61.27; H, 5.35; N, 3.03. HRMS [M+H$^+$] calculated for $C_{23}H_{25}NO_9$: 460.1612. Found: 460.1615.

Mutarrotational Equilibrium of Schiff Bases Derived from 2-Amino-2-deoxyaldoses. The corresponding imine studied (~ 15 mg) was dissolved in DMSO-*d*$_6$ (0.5 mL) and its ^{1}H NMR spectrum was immediately recorded. Further monitoring was carried out over time until reaching equilibration as spectra remained unaltered.

***2-[(*E*)-Benzylidenamino]-2-deoxy-α-D-glucopyranose* (54).** ^{1}H NMR (400 MHz, DMSO-*d*$_6$) δ 8.33 (1H, s, N=CH), 7.76 (2H, m, arom), 7.45 (3H, m, arom), 6.25 (1H, d, $J_{C1,OH}$ 4.4 Hz, C1-OH), 4.95 (2H, m, H-1, J_{OH} 5.6 Hz, OH), 4.49 (1H, t, $J_{OH,6}$ 5.6 Hz, C6-OH), (1H, t, $J_{2,3}$≈$J_{3,4}$ 9.4 Hz, H-3), 3.67 (1H, m, H-6), 3.15 (1H, m, H-2); ^{13}C NMR (100 MHz, DMSO-*d*$_6$) δ 162.59 (N=C), 136.48, 130.93, 128.93, 128.50, 128.41 (arom), 93.26 (C-1), 75.43 (C-2), 72.76 (C-5), 71.12 (C-3, C-4), 61.60 (C-6).

***2-Deoxy-2-[(*E*)-(4-methoxybenzylidene)amino]-α-D-glucopyranose* (55).** ^{1}H NMR (400 MHz, DMSO-*d*$_6$) δ 8.23 (1H, s, N=CH), 7.70 (2H, d, arom), 6.98 (2H, d, arom), 6.22 (1H, d, $J_{C1,OH}$ 4.2 Hz, C1-OH), 4.92 (2H, m, H-1, OH), 4.49 (1H, t, C6-OH), 3.86 (3H, s, OCH_3), 3.42 (1H, dd, $J_{6,6'}$ 12.2 Hz, H-6), 3.14 (1H, d, $J_{C4,OH}$ 5.0 Hz, H-4), 3.07 (1H, dd, $J_{1,2}$ 3.2 Hz, $J_{2,3}$ 9.7 Hz, H-2); ^{13}C NMR (100 MHz, DMSO-*d*$_6$) δ 161.68 (N=C), 161.38, 130.05, 129.34, 114.20 (arom), 93.30 (C-1), 75.35 (C-2), 72.70 (C-5), 71.14 (C-3, C-4), 61.54 (C-6), 55.57 (OCH_3).

***2-Deoxy-2-[(*E*)-(3-methoxybenzylidene)amino]-α-D-glucopyranose* (56).** ^{1}H NMR (400 MHz, DMSO-*d*$_6$) δ 8.30 (1H, s, N=CH), 7.34 (3H, m, arom), 7.02 (2H, d, arom), 6.27 (1H, d, $J_{C1,OH}$ 3.6 Hz, C1-OH), 4.96 (1H, d, $J_{1,2}$ 3.2 Hz, H-1), 4.50 (1H, s.a, C6-OH), 3.78 (3H, s, OCH_3), 3.13 (1H, d, $J_{1,2}$ 3.6 Hz, $J_{2,3}$ 9.6 Hz, H-2); ^{13}C NMR (100 MHz, DMSO-*d*$_6$) δ 162.44 (N=C), 160.07, 137.96, 130.71, 121.46, 117.16, 113.22 (arom), 93.20 (C-1), 75.37 (C-2), 72.76 (C-5), 71.18 (C-3), 71.06 (C-4), 61.57 (C-6), 55.47 (OCH_3).

***2-Deoxy-2-[(*E*)-(2-methoxybenzylidene)amino]-α-D-glucopyranose* (57).** ^{1}H NMR (400 MHz, DMSO-*d*$_6$) δ 8.64 (1H, s, N=CH), 7.90 (1H, dd, *J* 8.0 Hz, *J* 2.0 Hz, arom), 6.70 (2H, d, *J* 8.6 Hz, arom), 6.20 (1H, d, $J_{C1,OH}$ 4.4 Hz, C1-OH), 4.92 (2H, m, $J_{C3,OH}$ 5.2 Hz, C3-OH, H-1), 4.67 (1H, d, $J_{C4,OH}$ 5.2 Hz, C4-OH), 4.47 (1H, t, $J_{C6,OH}$ 5.6 Hz, C6-OH), 3.67 (1H, m, H-6'), 3.83 (3H, s, OCH_3), 3.55 (1H, m, H-5), 3.10 (1H, dd, $J_{1,2}$ 3.6 Hz, H-2); ^{13}C NMR (100 MHz, DMSO-*d*$_6$) δ 157.77 (arom), 157.53 (N=C), 132.49 (arom), 127.23 (arom), 124.33 (arom), 120.78 (arom), 112.09 (arom), 93.41 (C-1), 76.03 (C-2), 72.91 (C-5), 71.33 (C-3), 71.27 (C-4), 61.72 (C-6), 56.53 (OCH_3).

***2-Deoxy-2-[(*E*)-(3-hydroxybenzylidene)amino]-α-D-glucopyranose* (59).** ^{1}H NMR (400 MHz, DMSO-d_6) δ 9.55 (1H, s, OH-arom), 8.22 (1H, s, N=CH), 7.23 (1H, t, arom), 7.19 (1H, t, arom), 7.13 (1H, d, arom), 6.84 (1H, dd, arom), 6.22 (1H, d, $J_{C1,OH}$ 4.4 Hz, C1-OH), 4.92 (2H, m, $J_{C1,OH}$=$J_{1,2}$ 4.4 Hz, H-1, C4-OH), 4.45 (1H, t, $J_{C6,OH}$ 5.7 Hz, C6-OH), 3.80 (1H, dt, $J_{2,3}$ 9.2 Hz, $J_{C3,OH}$=$J_{3,4}$ 9.2 Hz, H-3), 3.67 (1H, dd, $J_{6,6'}$ 11.7 Hz, $J_{5,6}$ 5.8 Hz, H-6), 3.54 (1H, dd, $J_{C6,OH}$≈$J_{5,6}$ 5.8 Hz, H-6´), 3.14 (1H, m, $J_{C4,OH}$=$J_{4,5}$ 5.1 Hz, H-4), 3.10 (1H, dd, $J_{1,2}$ 3.3 Hz, $J_{2,3}$ 9.9 Hz, H-2); ^{13}C NMR (100 MHz, DMSO-d_6) δ 162.50 (N=C), 157.77, 130.05, 120.04, 118.04, 114.13 (arom), 93.23 (C-1), 75.34 (C-2), 72.70 (C-5), 71.06 (C-3), 71.18 (C-4), 61.54 (C-6).

***2-[(*E*)-(2-Chlorobenzylidene)amino]-2-deoxy-α-D-glucopyranose* (60)**; ^{1}H RMN (500 MHz, DMSO-d_6) δ 8.66 (1H, s, N=CH), 8.04 (1H, dd, J 8.0 Hz, J 1.5 Hz, arom), 7.53-7.46 (2H, m, arom), 7.40 (1H, m, arom), 6.27 (1H, d, $J_{C1,OH}$ 4.5 Hz, C1-OH), 4.97 (1H, d, $J_{C3,OH}$ 6.0 Hz, C3-OH), 4.93 (1H, d, $J_{C4,OH}$ 5.5 Hz, C4-OH), 4.73 (1H, m, H-1), 4.46 (1H, t, $J_{C6,OH}$ 6.0 Hz, C6-OH), 3.73 (1H, ddd, $J_{5,6}$ 2.0 Hz, $J_{6,OH}$ 5.5 Hz,$J_{6,6'}$ 11.5 Hz, H-6), 3.50 (1H, dd, $J_{6',OH}$, 5.5 Hz, $J_{6,6'}$ 11.5 Hz, H-6´), 3.44 (1H, td, $J_{C3,OH}$ 5.5 Hz, $J_{2,3}$ ≈$J_{3,4}$ 9.0 Hz, H-3), 3.26 (1H, m, H-5), 3.16 (1H, td, $J_{C4,OH}$ 5.5 Hz, $J_{3,4}$ ≈$J_{4,5}$ 9.5 Hz H-4), 2.91 (1H, dd, $J_{1,2}$ 7.5 Hz, $J_{2,3}$ 9.0 Hz, H-2). ^{13}C RMN (125 MHz, DMSO-d_6): 158.15 (N=C), 134.14 (arom), 132.85 (arom), 132.21 (arom), 129.87 (arom), 128.49 (arom), 127.41 (arom), 92.73 (C-1), 75.16 (C-2), 72.50 (C-5), 70.82 and 70.63 (C-3, C-4), 61.34 (C-6).

***2-Deoxy-2-[(*E*)-(4-hydroxy-3-methoxybenzylidene)amino]-α-D-glucopyranose* (61).** ^{1}H NMR (400 MHz, DMSO-d_6) δ 8.28 (1H, s, N=CH), 7.31 (3H, s, arom), 6.27 (1H, sa, C1-OH), 4.20 (1H, sa, OH-6), 3.81 (3H, s, OCH_3), 3.61 (1H, dd, $J_{6,6'}$ 12.1 Hz, H-6), 3.04 (1H, dd, $J_{2,3}$ 8.3 Hz, H-2); ^{13}C NMR (100 MHz, DMSO-d_6) δ 162.01 (N=C), 149.33, 149.33, 148.03, 128.28, 115.75, 110.78 (arom), 93.28 (C-1), 72.68 (C-2), 72.68 (C-5), 71.11 (C-3, C-4), 61.52 (C-6), 55.75 (OCH_3).

***2-Deoxy-2-[(*E*)-(4-nitrobenzylidene)amino]-α-D-glucopyranose* (62).** ^{1}H NMR (400 MHz, DMSO-d_6) δ 8.47 (1H, s, N=CH), 8.28 (2H, d, arom), 8.03 (2H, d, arom), 5.97 (1H, d, arom), 5.97 (1H, d, C1-OH), 4.86 (1H, sa, H-1), 4.54 (1H, sa, C6-OH), 3.85 (1H, t, $J_{2,3}$≈$J_{3,4}$ 9.0 Hz, H-3), 3.67 (1H, d, $J_{6,6'}$ 11.9 Hz, H-6), 3.22 (1H, dd, $J_{1,2}$ 3.1 Hz, H-2); ^{13}C NMR (100 MHz, DMSO-d_6) δ 161.10 (N=C), 148.78, 142.14, 129.47, 124.21 (2 C, arom), 93.00 (C-1), 75.44 (C-2), 72.77 (C-5), 71.05 (C-3), 70.97 (C-4), 61.52 (C-6).

***2-Deoxy-2-[(*E*)-(4-dimethylaminobenzylidene)amino]-α-D-glucopyranose* (63).** ^{1}H NMR (400 MHz, DMSO-d_6) δ 8.13 (1H, s, N=CH), 7.54 (2H, d, J 8.5 Hz, arom), 6.70 (2H, d, J 8.6 Hz, arom), 6.12 (1H, d, $J_{C1,OH}$ 4.4 Hz, C1-OH), 4.86 (2H, m, $J_{C3,OH}$ 5.2 Hz, C3-OH, H-1), 4.61 (1H, d, $J_{C4,OH}$ 5.2 Hz, C4-OH), 4.44 (1H, t, $J_{C6,OH}$ 5.6 Hz, C6-OH), 3.78 (2H, m, H-3, H-6), 3.65 (1H, m, H-6'), 3.53 (1H, m, H-5), 3.03 (1H, m, H-2); ^{13}C NMR (100 MHz, DMSO-d_6) δ 161.96 (N=C), 129.81, 152.01, 124.51, 111.68 (arom), 93.51 (C-1), 75.53 (C-2), 72.74 (C-5), 71.34 (C-3, C-4), 61.64 (C-6).

***2-Deoxy-2-[(*E*)-(4-methylbenzylidene)amino]-α-D-glucopyranose* (64).** ^1H NMR (400 MHz, DMSO-d_6) δ 8.28 (1H, s, N=CH), 7.80 (2H, d, arom), 7.24 (2H, d, arom), 6.21 (1H, d, $J_{C1,OH}$ 4.0 Hz, C1-OH), 4.93 (1H, m, H-1), 4.47 (1H, t, C6-OH), 3.81 (1H, m, H-6), 3.68 (1H, m, $J_{6,6'}$ 11.7 Hz, H-6'), 3.15 (1H, m, $J_{3,4} \approx J_{4,5}$ 8.8 Hz, H-4), 3.11 (1H, dd, $J_{1,2}$ 3.6 Hz, $J_{2,3}$ 10.0 Hz, H-2), 2.39 (3H, s, CH_3); ^{13}C NMR (100 MHz, DMSO-d_6) δ 162.26 (N=C), 140.55, 134.06, 134.06, 130.09, 128.48 (arom), 93.27 (C-1), 75.44 (C-2), 72.77 (C-5), 71.22 (C-3), 71.10 (C-4), 61.58 (C-6), 21.39 (CH_3).

***2-Deoxy-2-[(*E*)-(3-methylbenzylidene)amino]-α-D-glucopyranose* (65).** ^1H NMR (500 MHz, DMSO-d_6) δ 8.27 (s, 1H, CH=N), 7.60 (s, 1H, H-arom), 7.52 (m, 1H, H-arom), 7.32 (t, J = 7.5 Hz, 1H, H-arom) 7.26 (d, J = 7.5 Hz, 1H, H-arom), 6.21 (d, $J_{1,OH}$ = 4.0 Hz, 1H, C1-OH), 4.92 (m, 2H, C4-OH, C3-OH), 4.72 (t, $J_{1,OH} \approx J_{1,2}$ = 3.0 Hz, 1H, H-1), 4.49 (t, $J_{6,OH}$ = 5.0 Hz, 1H, C6-OH), 3.81 (td, $J_{6,OH}$ = 5.5 Hz, $J_{6,6'}$ = 9.5 Hz, 1H, H-6), 3.65 (m, 1H, H-6'), 3.12 (dd, $J_{1,2}$ = 3.5 Hz, $J_{2,3}$ = 10.0 Hz 1H, H-2), 2.54 (s, 1H, CH_3); ^{13}C NMR (125 MHz, DMSO-d_6) δ 162.36 (C=N), 137.76, 136.25, 129.12, 128.47, 128.34, 125.72 (arom), 93.01 (C-1), 75.03 (C-2), 72.40 (C-5), 70.94 (C-3), 70.82 (C-4), 61.37 (C-6), 20.74 (CH_3).

***2-Deoxy-2-[(*E*)-(2-methylbenzylidene)amino]-α-D-glucopyranose* (66).** ^1H NMR (500 MHz, DMSO-d_6) δ 8.58 (s, 1H, CH=N), 7.83 (d, J = 6.5 Hz, 1H, H-arom), 7.32 (m, 1H, H-arom), 7.23 (m, 2H, H-arom), 6.17 (d, $J_{1,OH}$ = 4.0 Hz, 1H, C1-OH), 4.95 (t, $J_{1,OH} \approx J_{1,2}$ = 3.5 Hz, 1H, H-1), 4.88 (d, $J_{4,OH}$ = 5.0 Hz, 1H, C4-OH), 4.67 (d, $J_{3,OH}$ = 6.0 Hz, 1H, C3-OH), 4.43 (t, $J_{6,OH}$ = 6.0 Hz, 1H, C6-OH), 3.81 (td, $J_{3,OH}$ = 6.0 Hz, $J_{2,3} \approx J_{3,4}$ = 9.5 Hz 1H, H-3), 3.67 (ddd, $J_{5,6}$ = 2.0 Hz, $J_{6,OH}$ = 5.5 Hz, $J_{6,6'}$ = 11.5 Hz, 1H, H-6), 3.53 (m, 1H, H-6'), 3.21 (m, 1H, H-2), 2.47 (s, 1H, CH3); ^{13}C NMR (125 MHz, DMSO-d_6) δ 160.66 (C=N), 137.37, 134.21, 130.62, 129.94, 125.70 (arom), 92.95 (C-1), 75.31(C-2), 72.35 (C-5), 70.86 (C-3), 70.71 (C-4), 61.31 (C-6), 18.83 (CH_3).

***2-Deoxy-2-[(*E*)-(4-ethylbenzylidene)amino]-α-D-glucopyranose* (67).** ^1H NMR (500 MHz, DMSO-d_6) δ 8.27 (s, 1H, CH=N), 7.68 (d, J = 8.0 Hz, 2H, H-arom), 7.28 (d, J = 8.0 Hz, 2H, H-arom), 6.19 (d, $J_{1,OH}$ = 4.0 Hz, 1H, C1-OH), 4.92 (t, $J_{1,OH} \approx J_{1,2}$ = 3.5 Hz, 1H, H-1), 4.90 (m, 1H, C4-OH), 4.68 (m, 1H, C3-OH), 4.47 (t, $J_{6,OH}$ = 6.0 Hz, 1H, C6-OH), 3.74 (td, $J_{5,6}$ = 4.5 Hz, $J_{6,6'}$ = 9.0 Hz, 1H, H-6), 3.66 (m, 1H, H-6'), 3.10 (dd, $J_{1,2}$ = 3.0 Hz, $J_{2,3}$ = 9.5 Hz, 1H, H-2), 2.68 (c, J = 7.5 Hz, 2H, CH_2), 1.20 (t, J = 7.5 Hz, 1H, CH_3); ^{13}C NMR (125 MHz, DMSO-d_6) δ 161.01 (C=N), 134.21, 133.97, 129.71, 128.58, (arom), 92.97 (C-1), 74.98 (C-2), 72.36 (C-5), 70.91 (C-3), 70.79 (C-4), 61.34 (C-6), 28.36 (CH_2), 15.09 (CH_3).

***2-Deoxy-2-[(*E*)-(4-isopropylbenzylidene)amino]-α-D-glucopyranose* (68)**: ^1H RMN (500 MHz, DMSO-d_6) δ 8.27 (1H, s, N=CH), 7.84 (d, J = 8.0 Hz, 2H, H-arom), 7.48 (d, J = 8.0 Hz, 2H, H-arom), 6.20 (d, $J_{1,OH}$ = 4.0 Hz, 1H, C1-OH), 4.93 (sa, 1H, C4-OH), 4.84 (sa, 1H, C3-OH), 4.72 (m, 1H, H-1), 4.48 (sa, 1H, C6-OH), 3.66 (d, $J_{6,6'}$ = 12.0 Hz, 1H, H-6), 3.50-3.40 (m, 3H, H-6', H-5, H-3), 3.25 (m, 1H, H-5), 3.10 (dd, $J_{1,2}$ 3.5 Hz, $J_{2,3}$ 10.0 Hz, 1H, H-2), 2.99 (m, J = 7.0 Hz, 1H, CH isopropyl), 1.22 (d, J = 7.0 Hz, 6H, CH_3 isopropyl); ^{13}C RMN (125 MHz, DMSO-d_6) δ 161.91

(C=N), 155.81, 134.20, 129.84 (2C), 127.23 (2C) (arom), 93.02 (C-1), 76.91 (C-2), 72.46 (C-5), 70.94 (C-3), 70.83 (C-4), 61.32 (C-6), 33.78 (CH isopropyl), 23.52 (2C,CH_3).

***2-Deoxy-2-[(*E*)-(4-phenylbenzylidene)amino]-α-D-glucopyranose* (69).** ^{1}H NMR (500 MHz, DMSO-d_6) δ 8.35 (s, 1H, CH=N), 7.98 (d, J = 8.5 Hz, 2H, H-arom), 7.88 (d, J = 8.5 Hz, 2H, H-arom), 7.48 (m, 3H, H-arom), 6.31 (d, $J_{1,OH}$ = 2.0 Hz, 1H, C1-OH), 5.03 (d, 1H, $J_{4,OH}$ = 5.5 Hz, C4-OH), 4.97 (m, 1H, H-1), 4.83 (d, $J_{3,OH}$ = 5.5 Hz, 1H, C3-OH), 4.63 (t, $J_{6,OH}$ = 5.5 Hz, 1H, C6-OH), 3.07 (m, 1H, H-2); ^{13}C NMR (125 MHz, DMSO-d_6) δ 162.53 (C=N), 142.44, 139.04, 135.49, 129.17, 128.96 (arom), 93.26 (C-1), 79.24 (C-2), 75.19 (C-5), 72.58 (C-3), 71.12 (C-4), 61.44 (C-6).

***2-Deoxy-2-[(*E*)-(2,4-dimethoxybenzylidene)amino]-α-D-glucopyranose* (70).** ^{1}H NMR (500 MHz, DMSO-d_6) δ 8.51 (s, 1H, CH=N), 7.84 (d, J = 8.5 Hz, 1H, H-arom), 6.59 (m, 2H, H-arom), 6.15 (d, $J_{1,OH}$ = 3.5 Hz, 1H, C1-OH), 4.91 (d, $J_{4,OH}$ = 6.5 Hz, 1H, C4-OH), 4.89 (t, $J_{1,OH} \approx J_{1,2}$ = 3.5 Hz, 1H, H-1), 4.57 (m, 1H, C3-OH), 4.51 (t, $J_{6,OH}$ = 5.0 Hz 1H, C6-OH), 3.83 (s, 3H, OCH_3), 3.80 (s, 3H, OCH_3), 3.06 (dd, $J_{4,OH}$ = 3.5 Hz, $J_{3,4} \approx J_{4,5}$ = 10.0 Hz, 1H, H-4), 2.76 (m, 1H, H-2); ^{13}C NMR (125 MHz, DMSO-d_6) δ 162.87, 159.95 (arom), 156.97 (C=N), 128.41, 117.32, 106.93, 98.22 (arom), 93.20 (C-1), 79.15 (C-2), 75.45 (C-5), 72.42 (C-3), 71.02 (C-4), 61.41 (C-6), 56.08, 55.91 (OCH_3).

***2-Deoxy-2-[(*E*)-(2,4-dimethylbenzylidene)amino]-α-D-glucopyranose* (71).** ^{1}H NMR (500 MHz, DMSO-d_6) δ 8.52 (s, 1H, CH=N), 7.72 (d, J = 8.0 Hz, 1H, H-arom), 7.04 (m, 2H, H-arom), 6.16 (d, $J_{1,OH}$ = 4.0 Hz, 1H, C1-OH), 4.92 (t, $J_{1,OH} \approx J_{1,2}$ = 4.0 Hz, 1H, H-1), 4.90 (d, $J_{4,OH}$ = 5.5 Hz, 1H, C4-OH), 4.67 (m, 1H, C3-OH), 4.47 (t, $J_{6,OH}$ = 5.5 Hz, 1H, C6-OH), 3.79 (td, $J_{6,OH}$ = 5.5 Hz, $J_{6,6'}$ = 9.0 Hz, 1H, H-6), 3.66 (m, 1H, H-6'), 3.11 (d, $J_{1,2}$ = 3.5 Hz, 1H, H-2), 2.42 (s, 3H, CH_3), 2.26 (s, 1H, CH_3); ^{13}C NMR (125 MHz, DMSO-d_6) δ 162.13 (C=N), 144.37, 137.40, 132.31, 131.73, 127.09, 126.52 (arom), 93.09 (C-1), 75.34 (C-2), 72.39 (C-5), 70.95 (C-3), 70.85 (C-4), 61.38 (C-6), 21.18, 18.83 (CH_3).

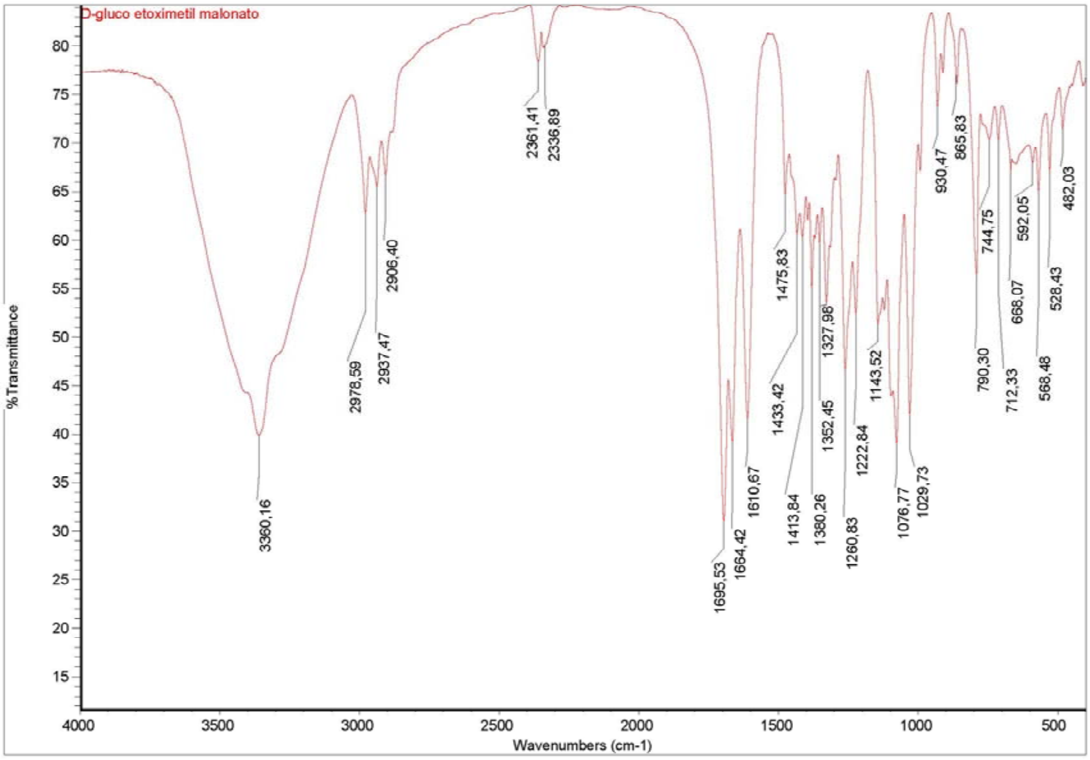

Figure S20. *IR spectrum of **5***

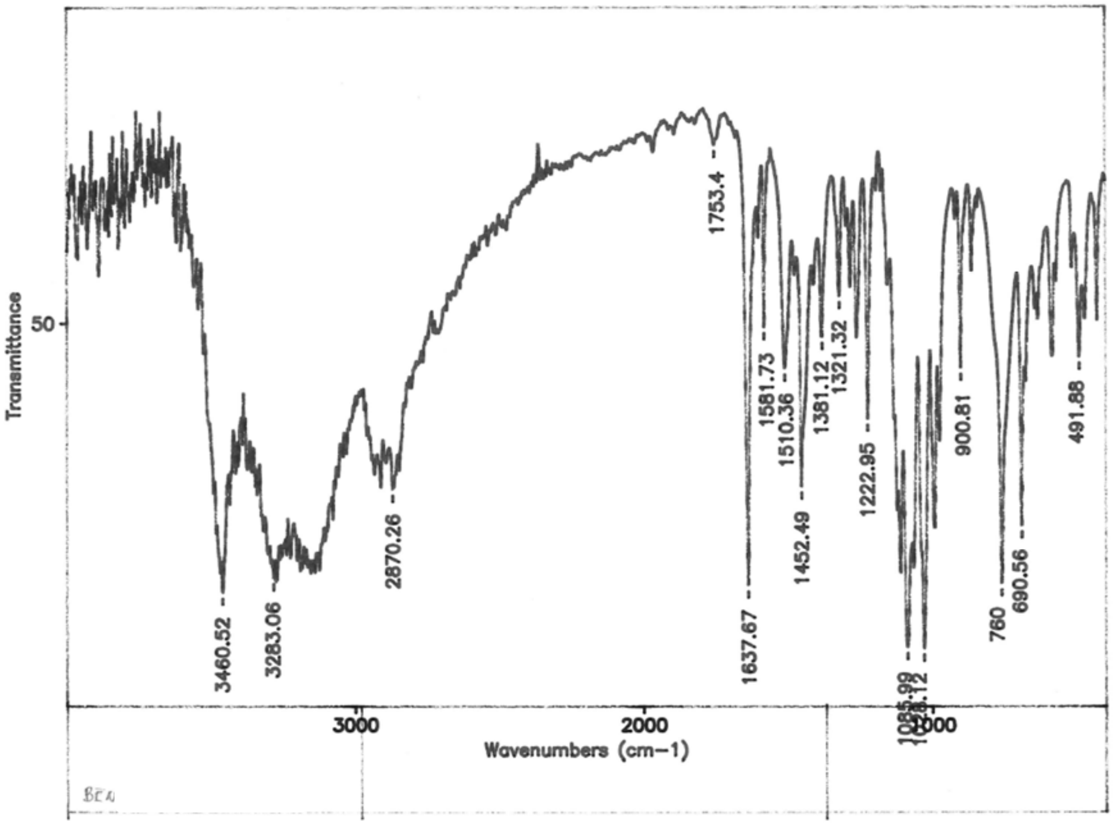

Figure S21**. IR spectrum of **11

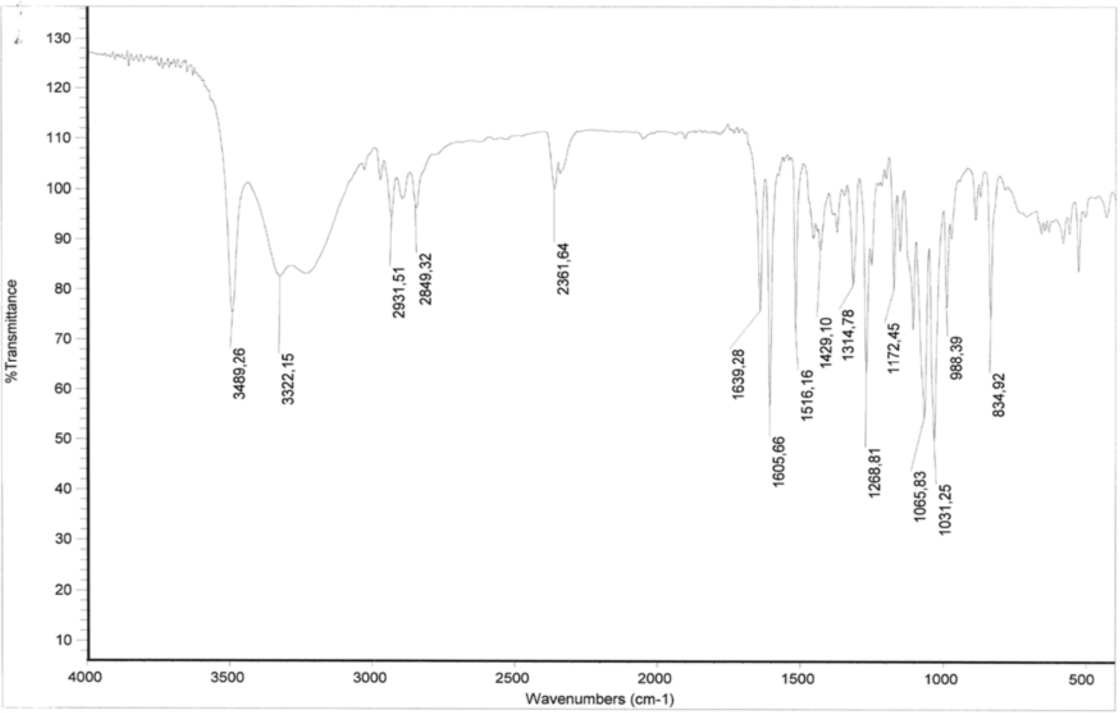

Figure S22. *IR spectrum of* ***12***

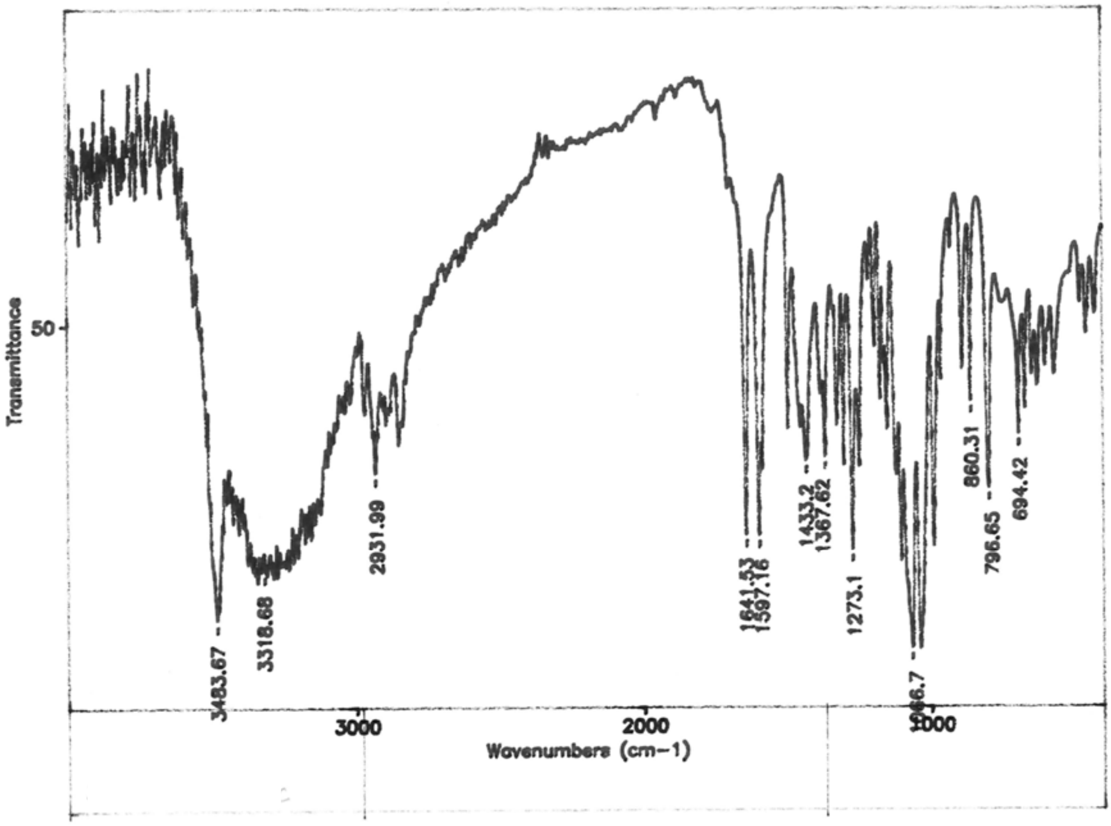

Figure S23. *IR spectrum of* ***13***

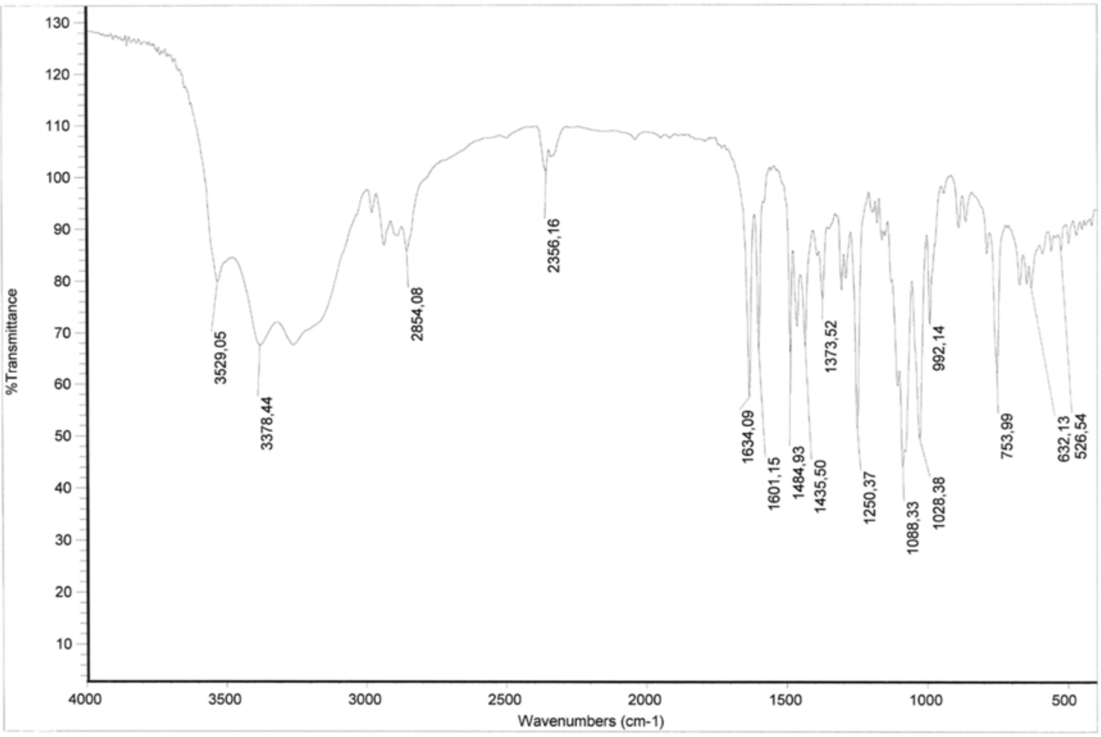

Figure S24. *IR spectrum of* ***14***

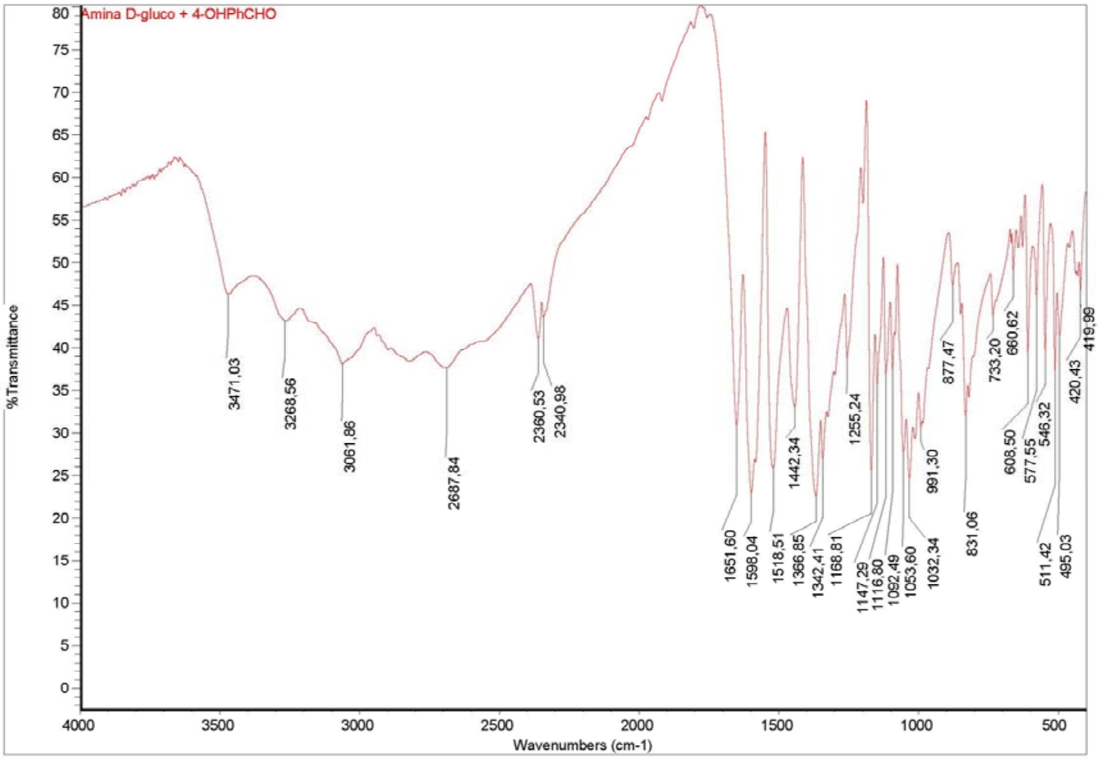

igure S25. *IR spectrum of* ***15***

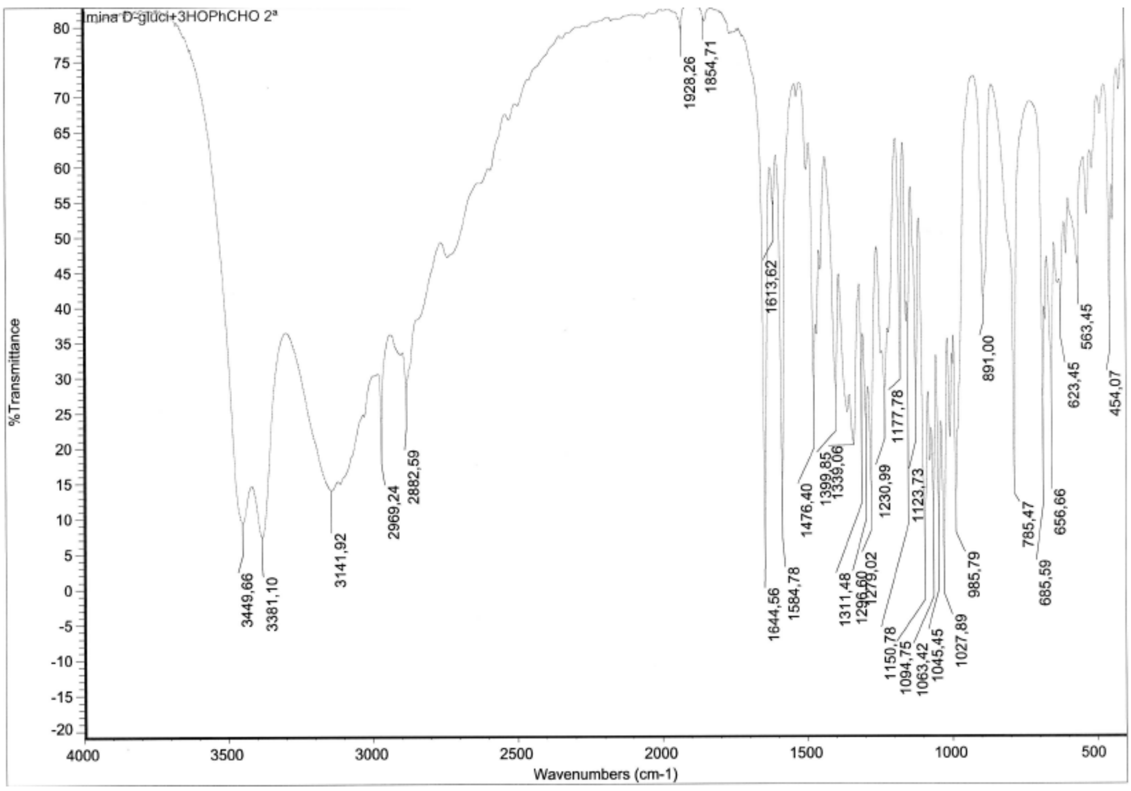

Figure S26. *IR spectrum of* ***16***

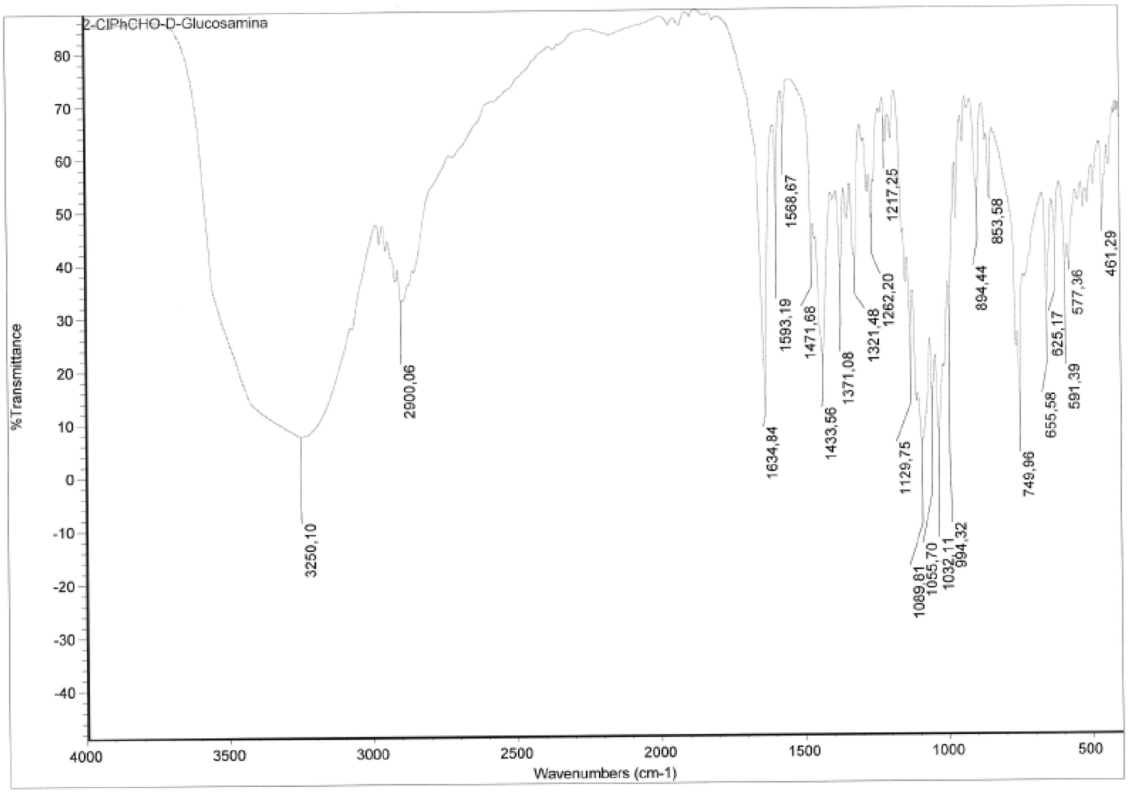

Figure S27. *IR spectrum of* ***17***

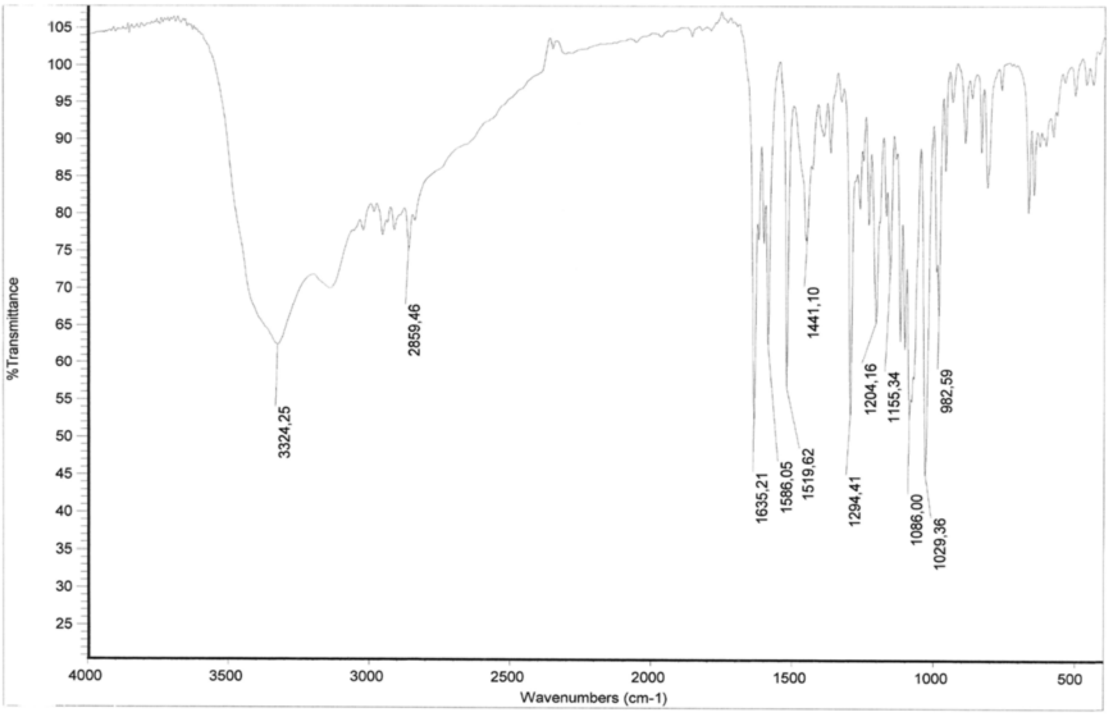

Figure S28. *IR spectrum of* ***18***

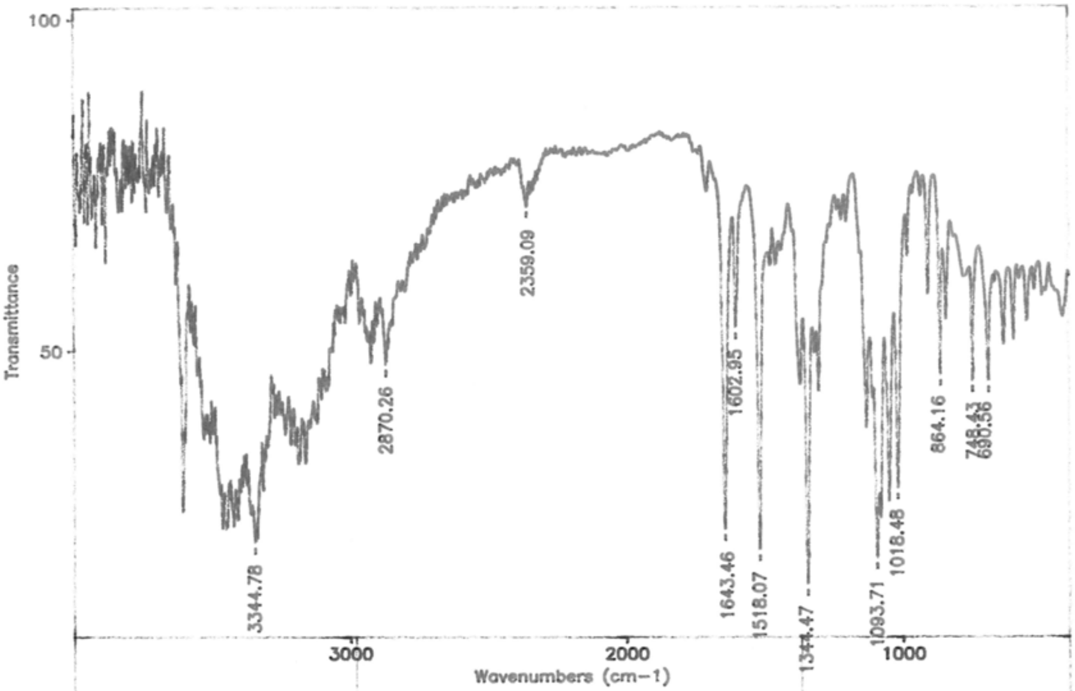

Figure S29. *IR spectrum of* ***19***

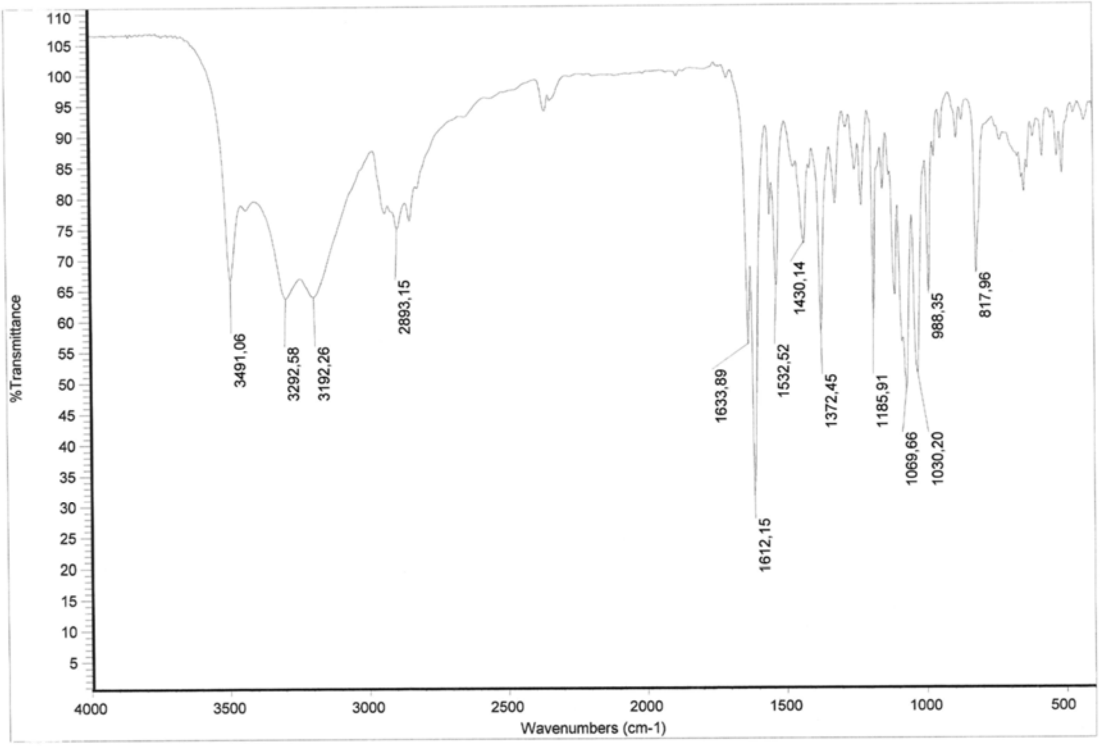

Figure S30. *IR spectrum of* ***20***

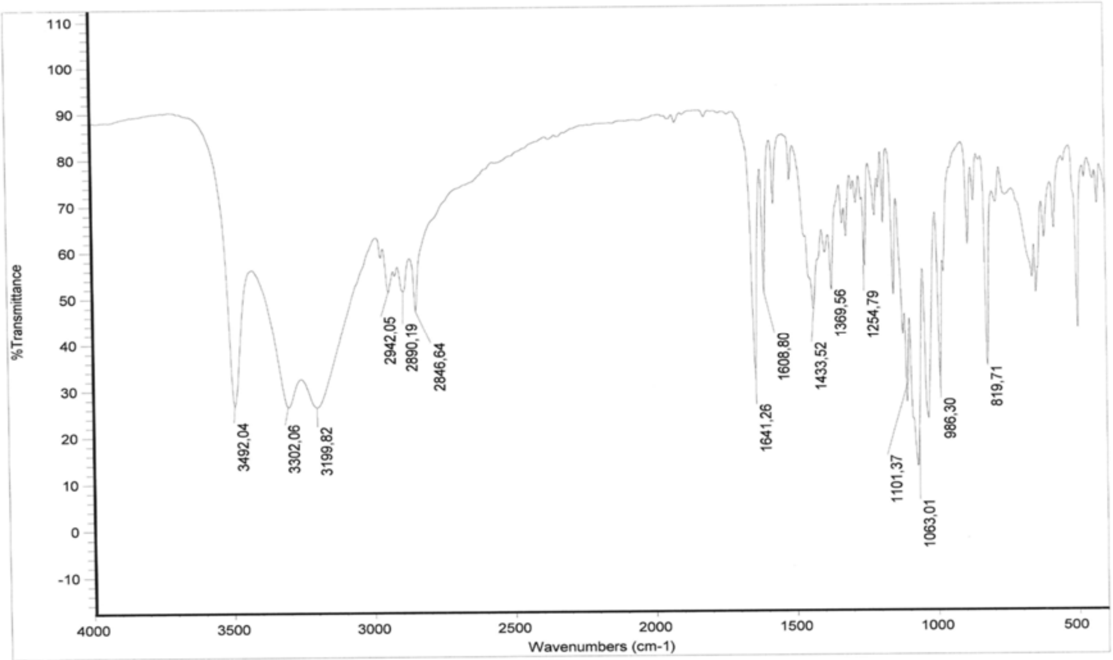

Figure S31. *IR spectrum of* ***21***

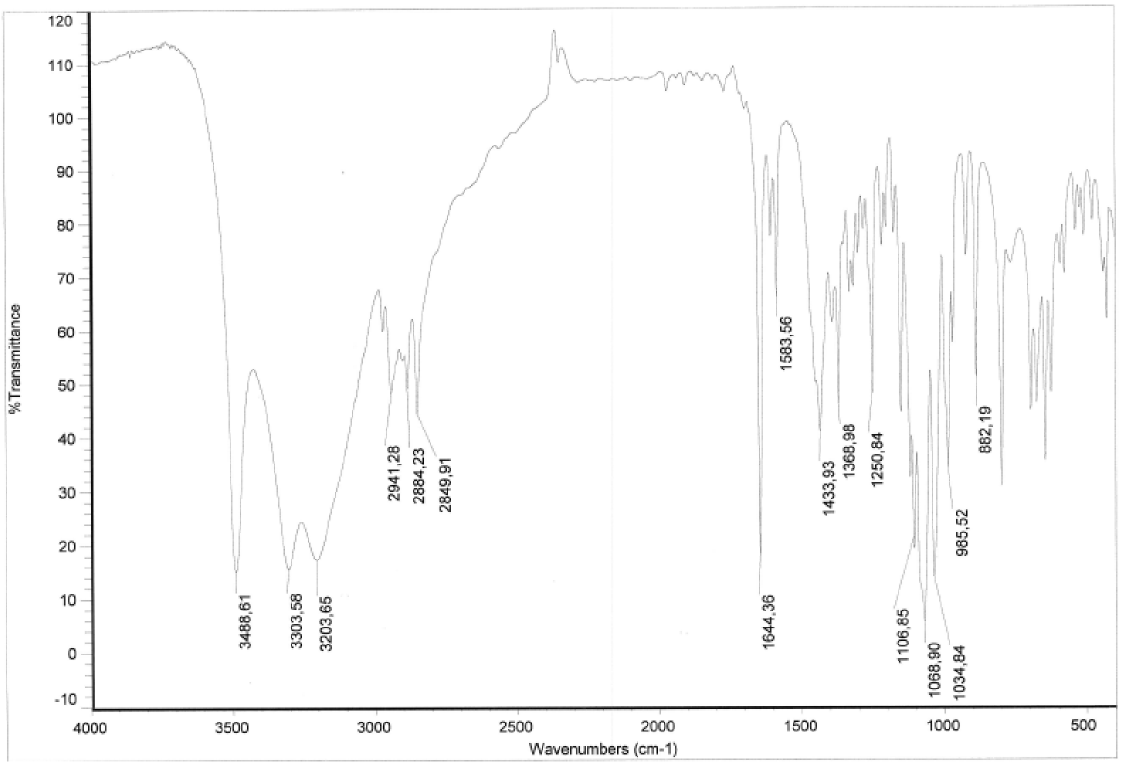

Figure S32. *IR spectrum of* ***22***

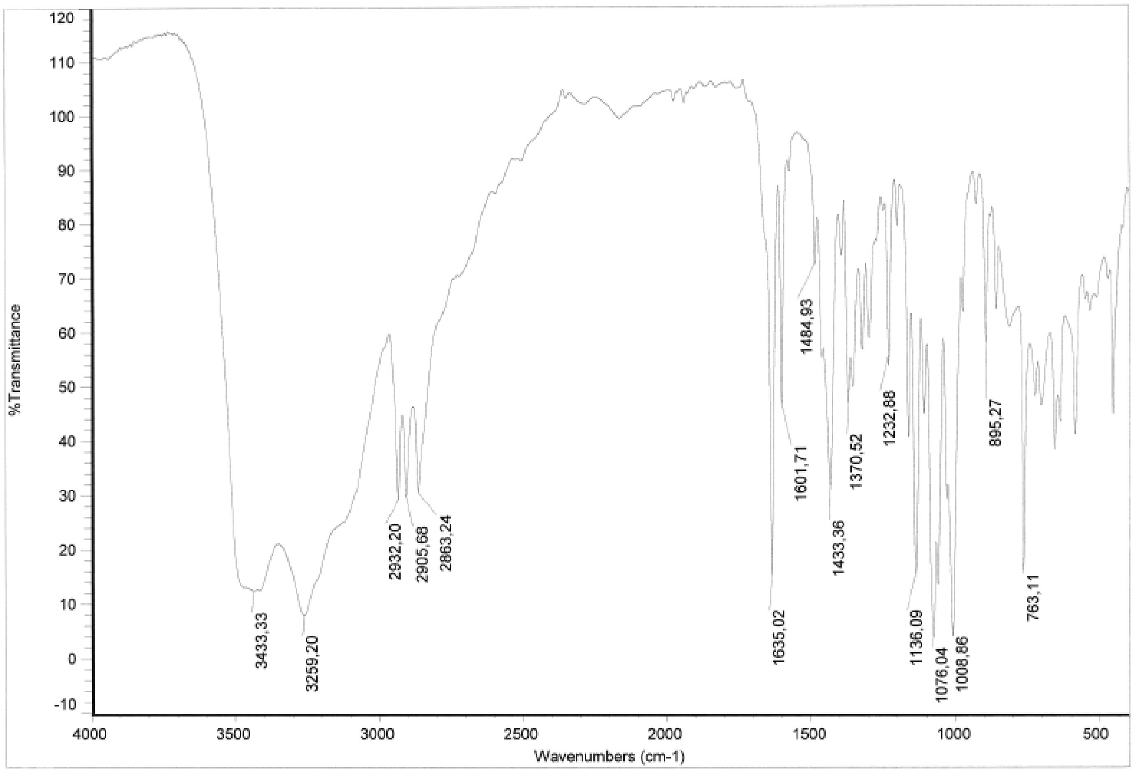

Figure S33. *IR spectrum of* ***23***

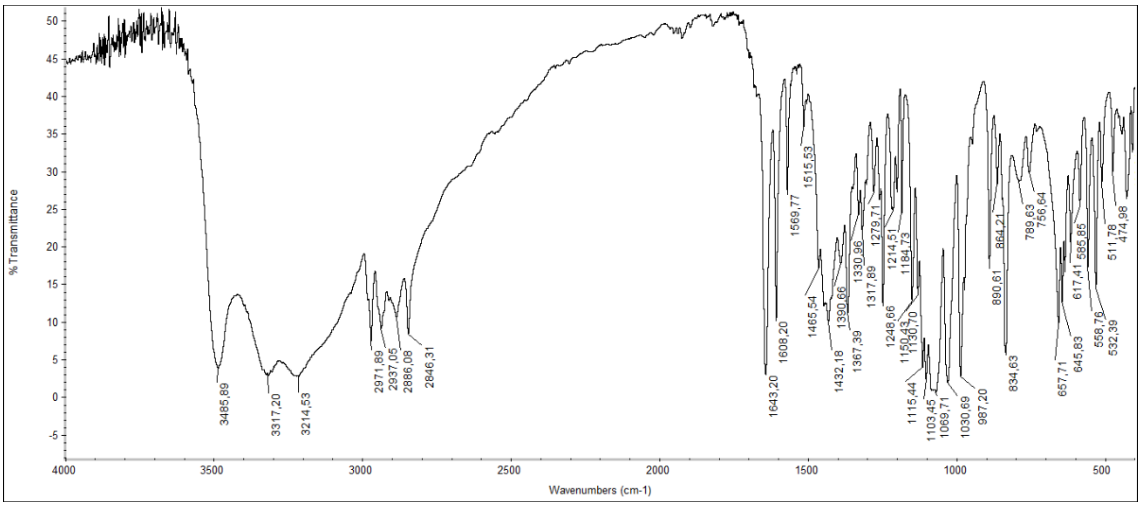

Figure S34. *IR spectrum of* ***24***

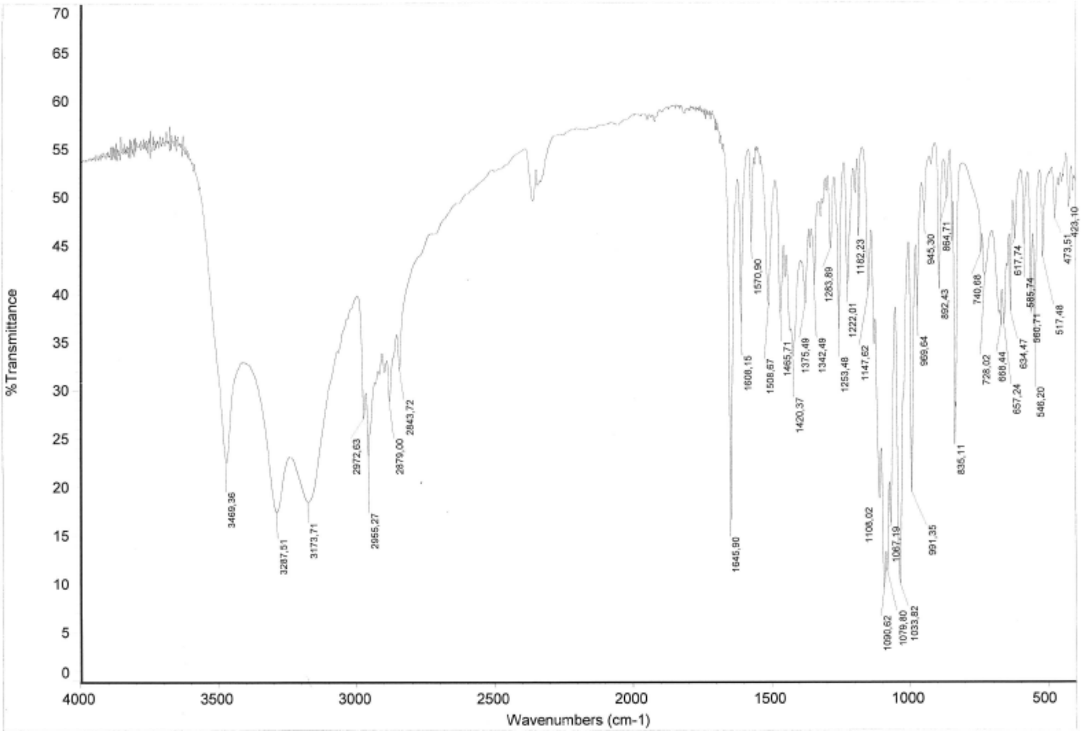

Figure S35. *IR spectrum of* ***25***

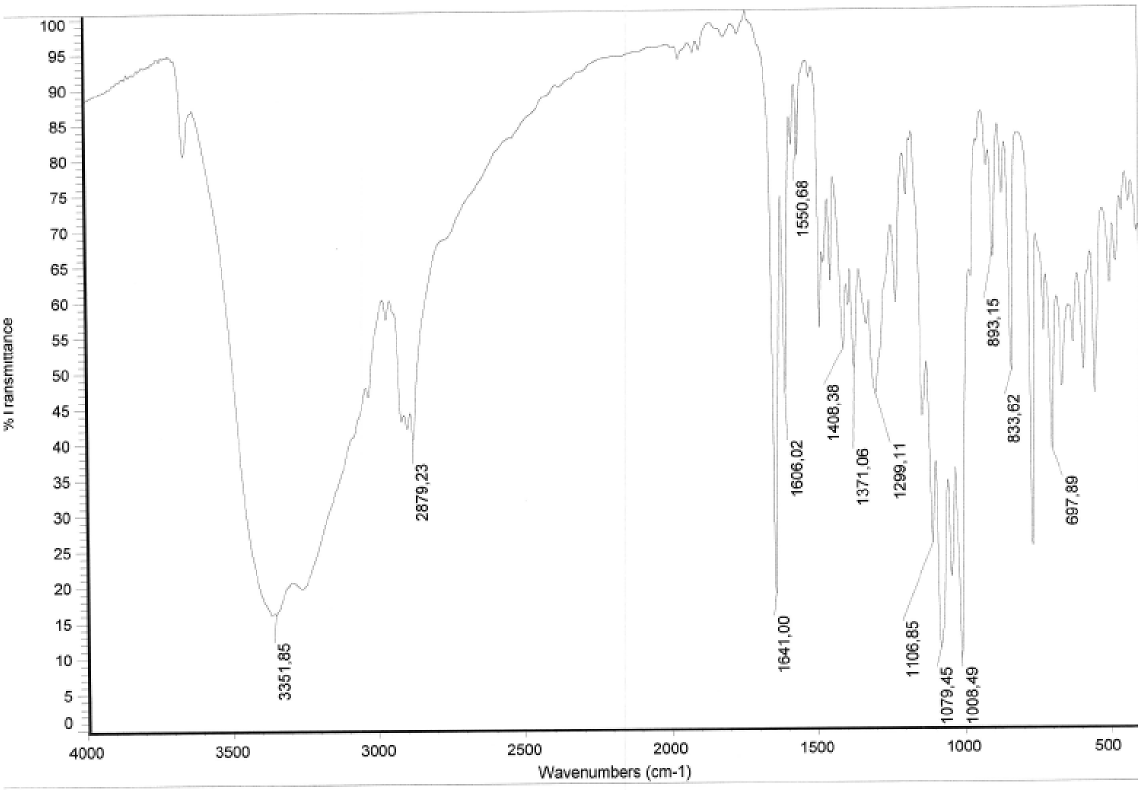

Figure S36. *IR spectrum of **26***

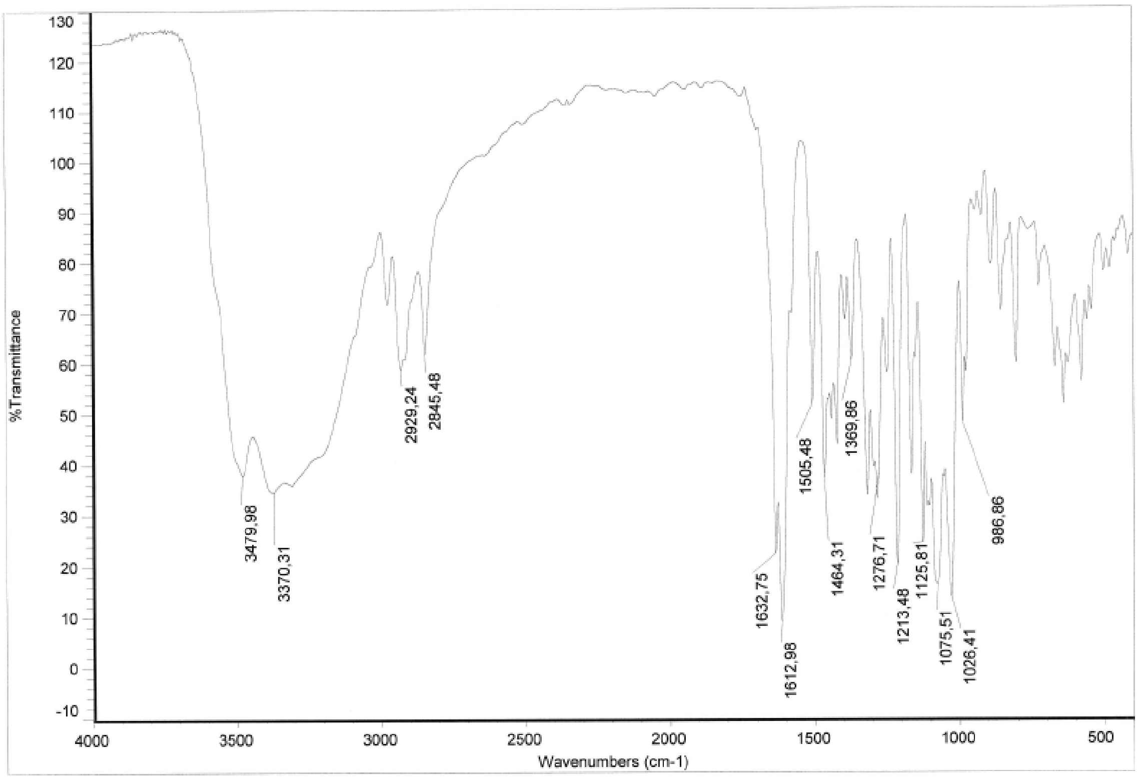

Figure S37. *IR spectrum of **27***

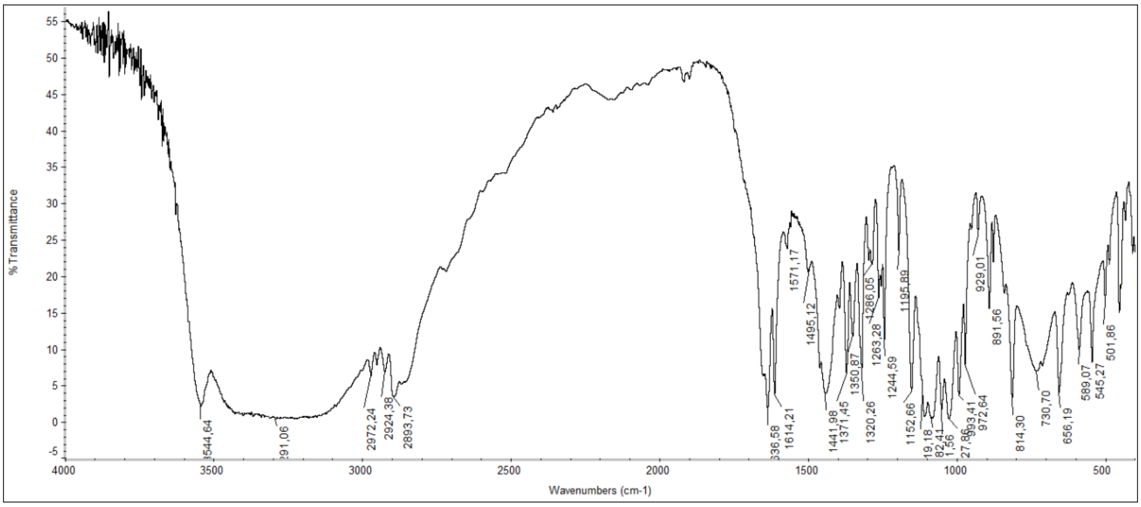

Figure S38. *IR spectrum of **28***

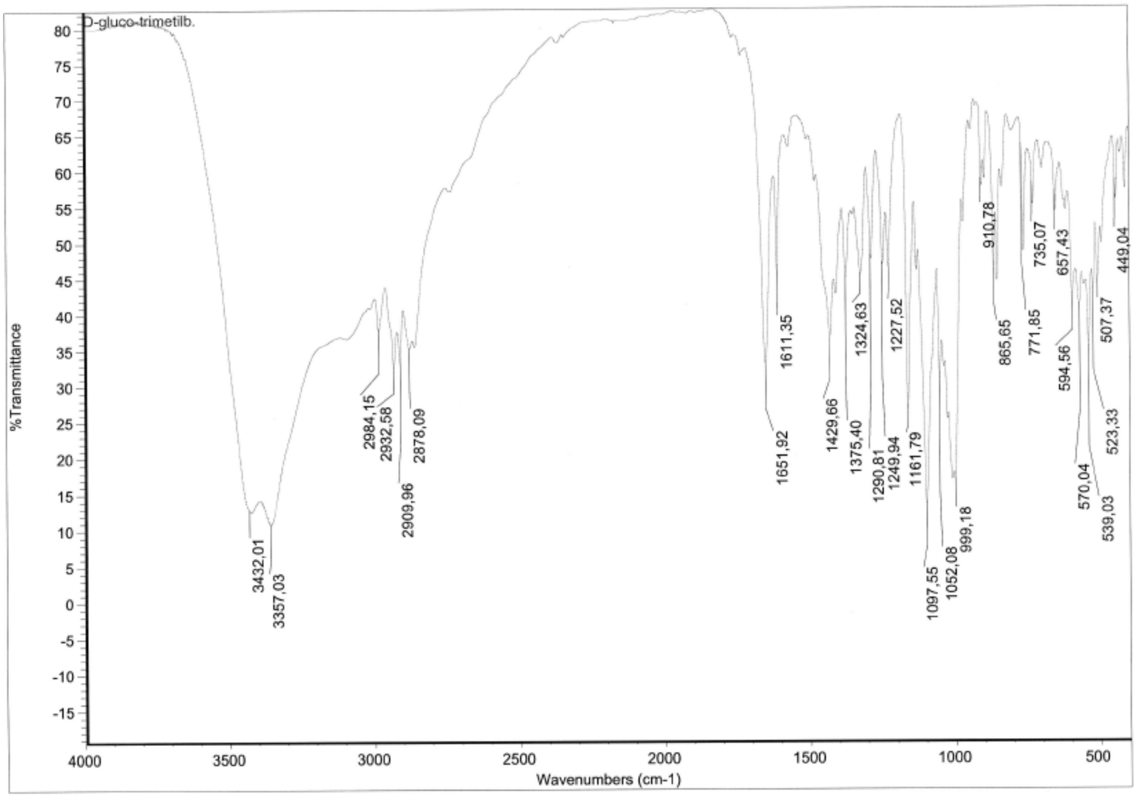

Figure S39. *IR spectrum of **29***

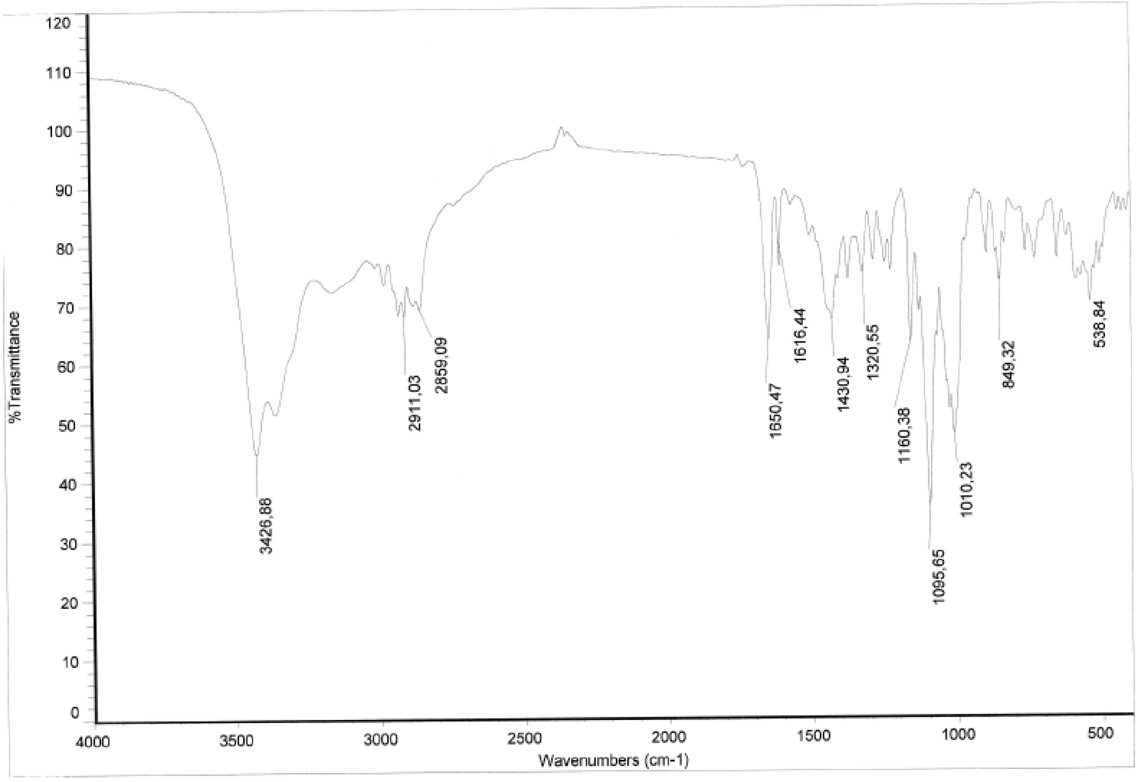

Figure S40. *IR spectrum of* ***30***

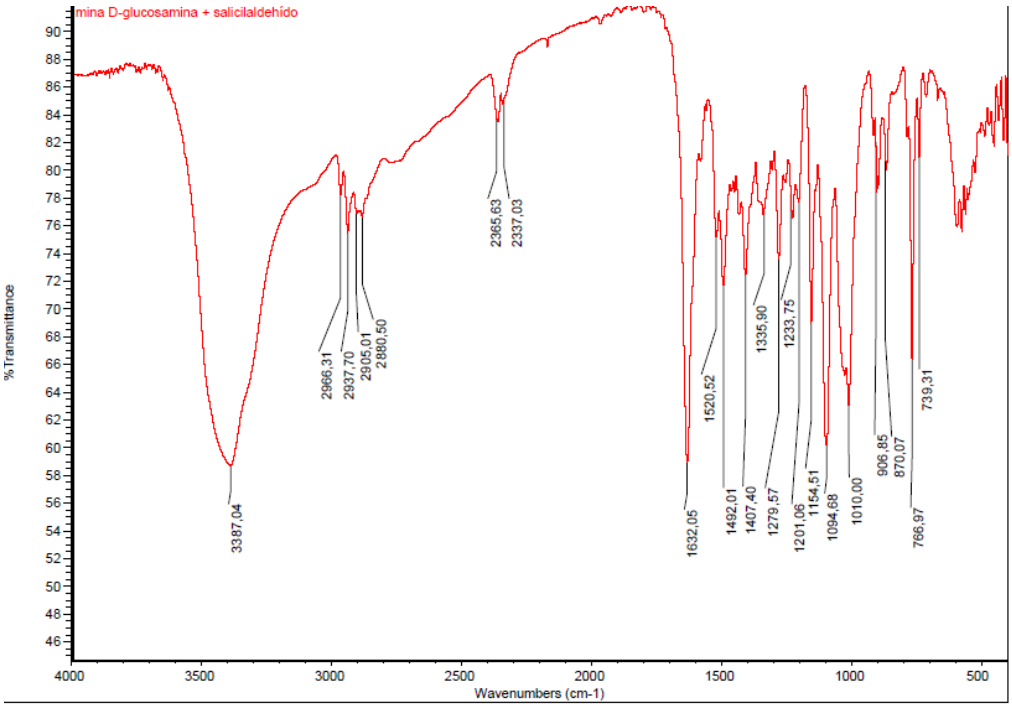

Figure S41. *IR spectrum of* ***32***

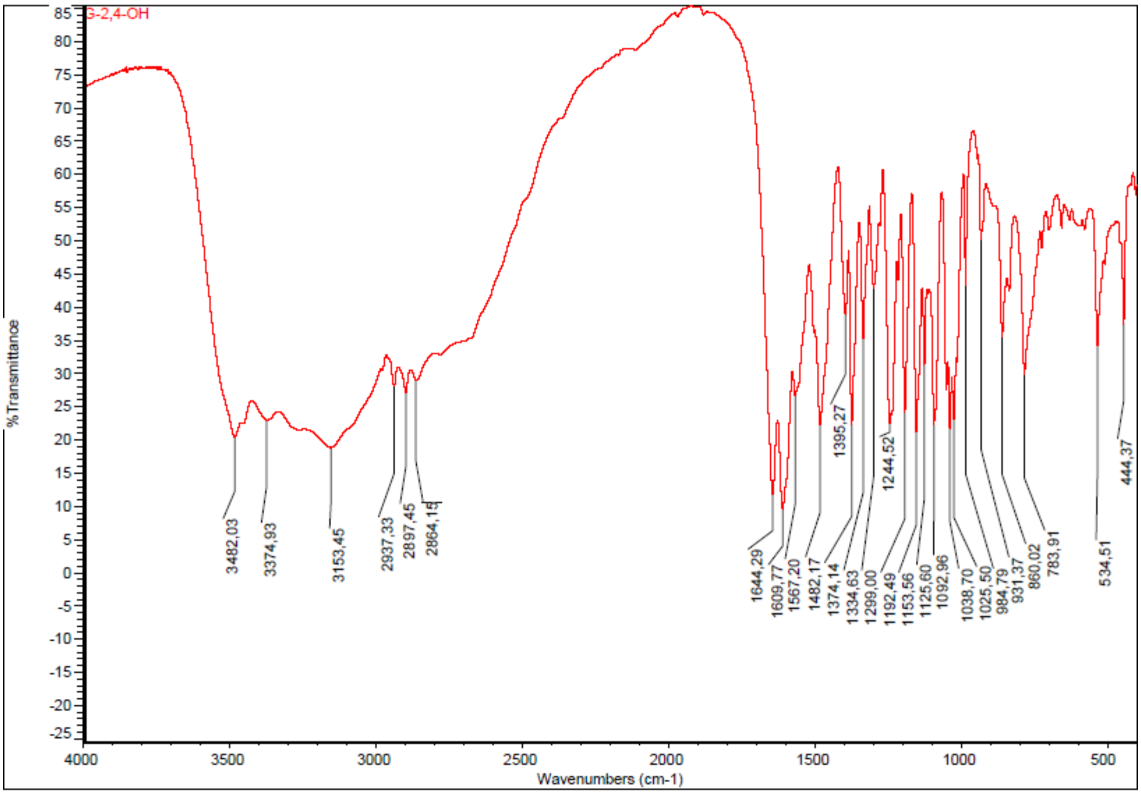

Figure S42. *IR spectrum of* ***34***

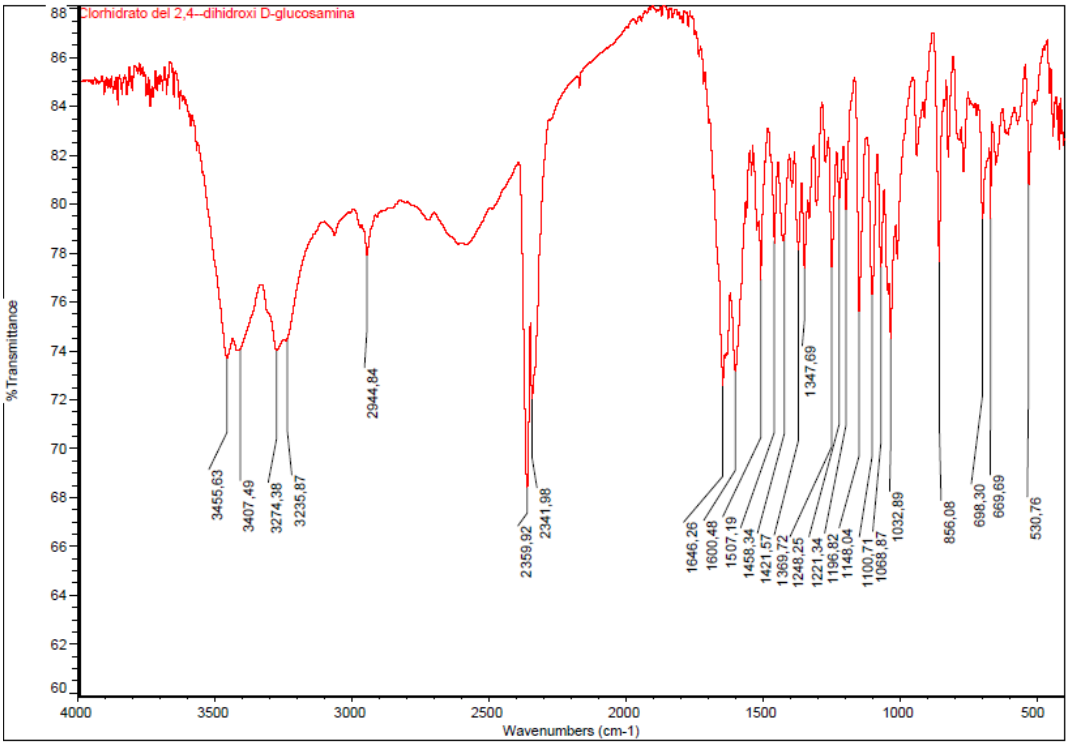

Figure S43. *IR spectrum of* ***36***

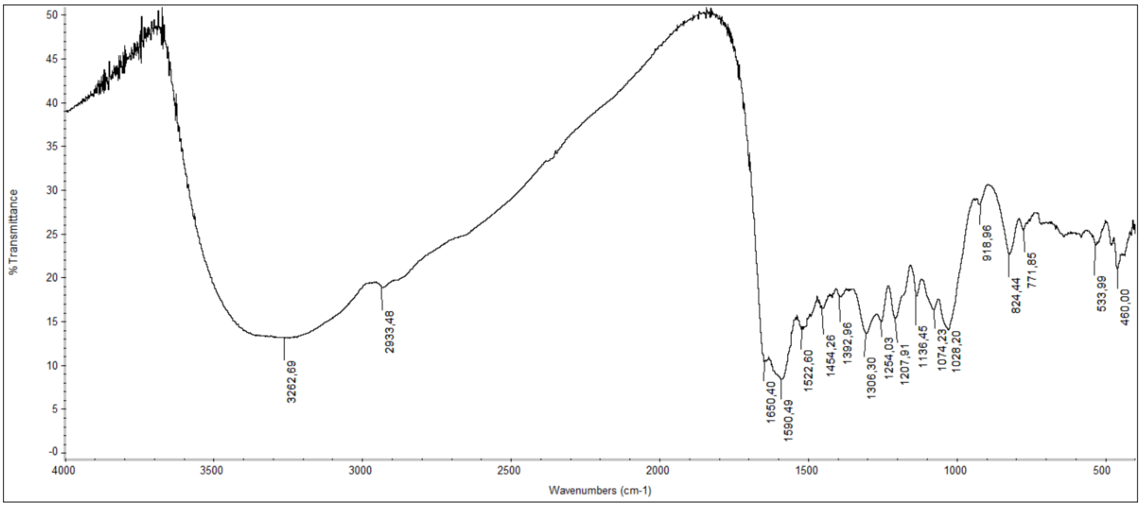

Figure S44. *IR spectrum of **39-42.***

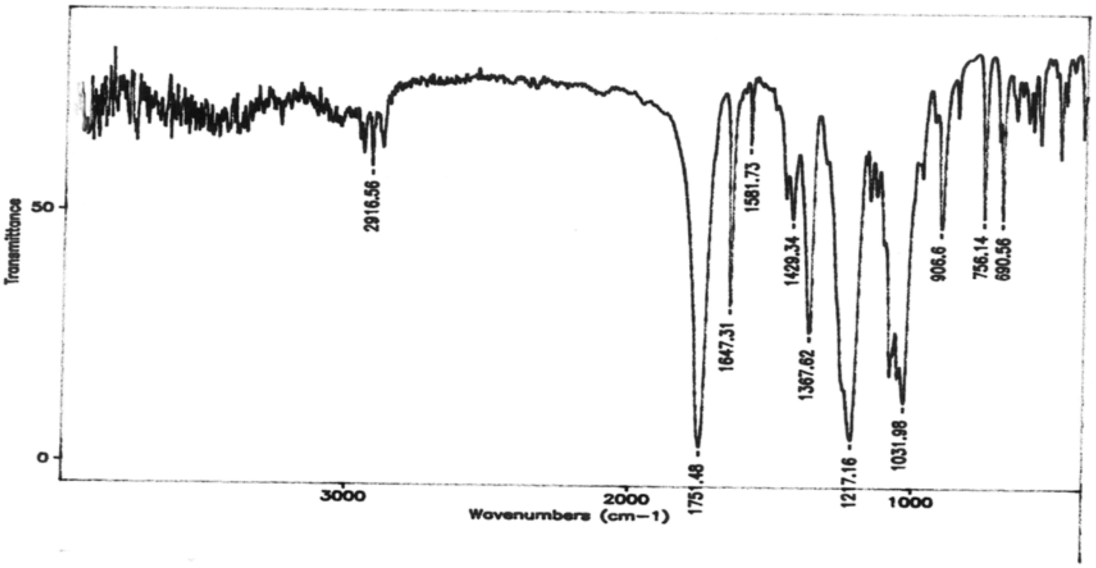

Figure S45. *IR spectrum of **43***

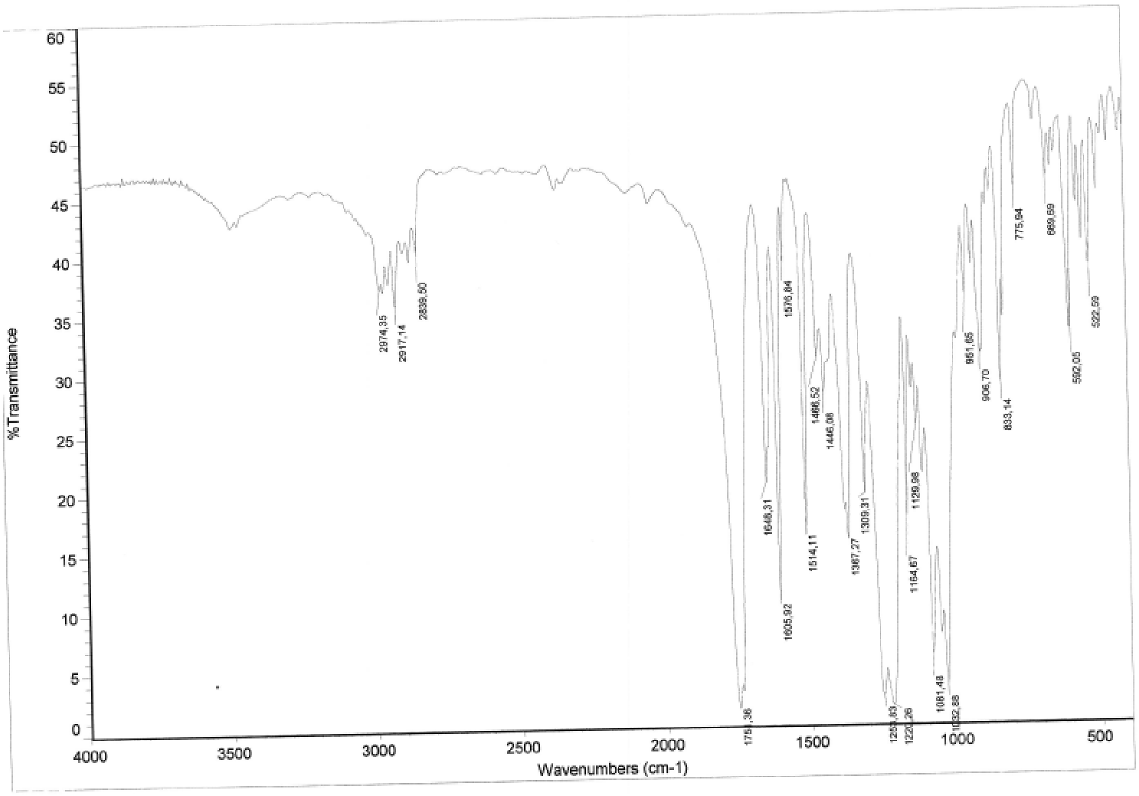

Figure S46. *IR spectrum of* ***44***

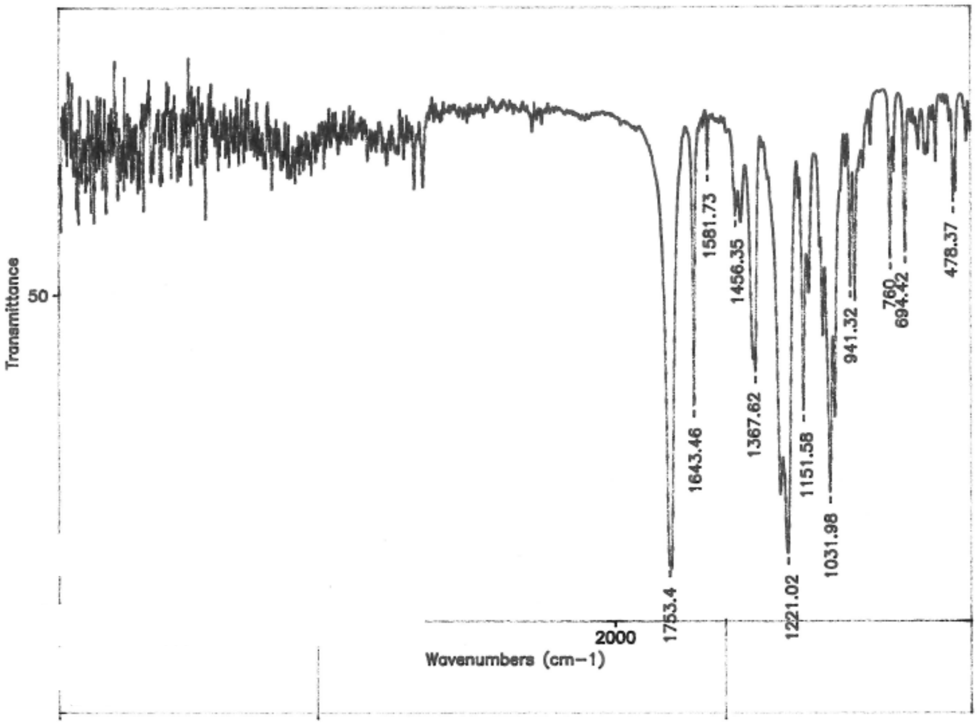

Figure S47. *IR spectrum of* ***46***

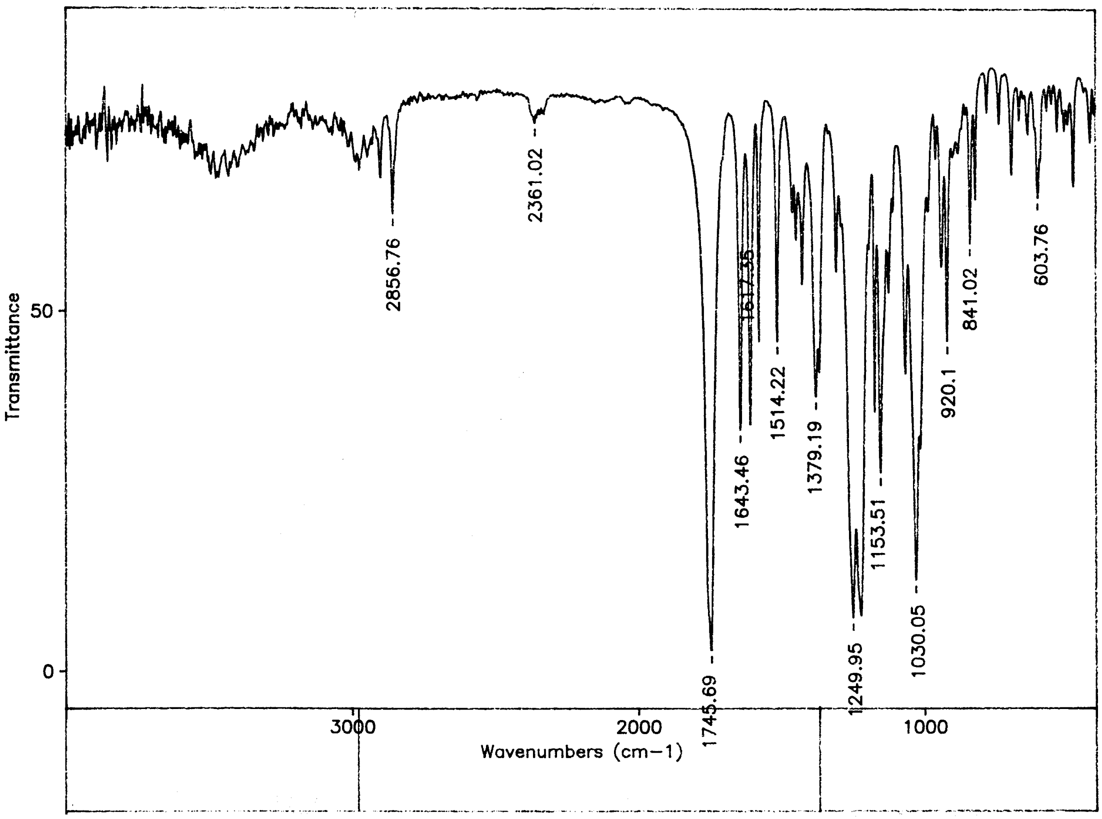

Figure S48.** IR spectrum of **47

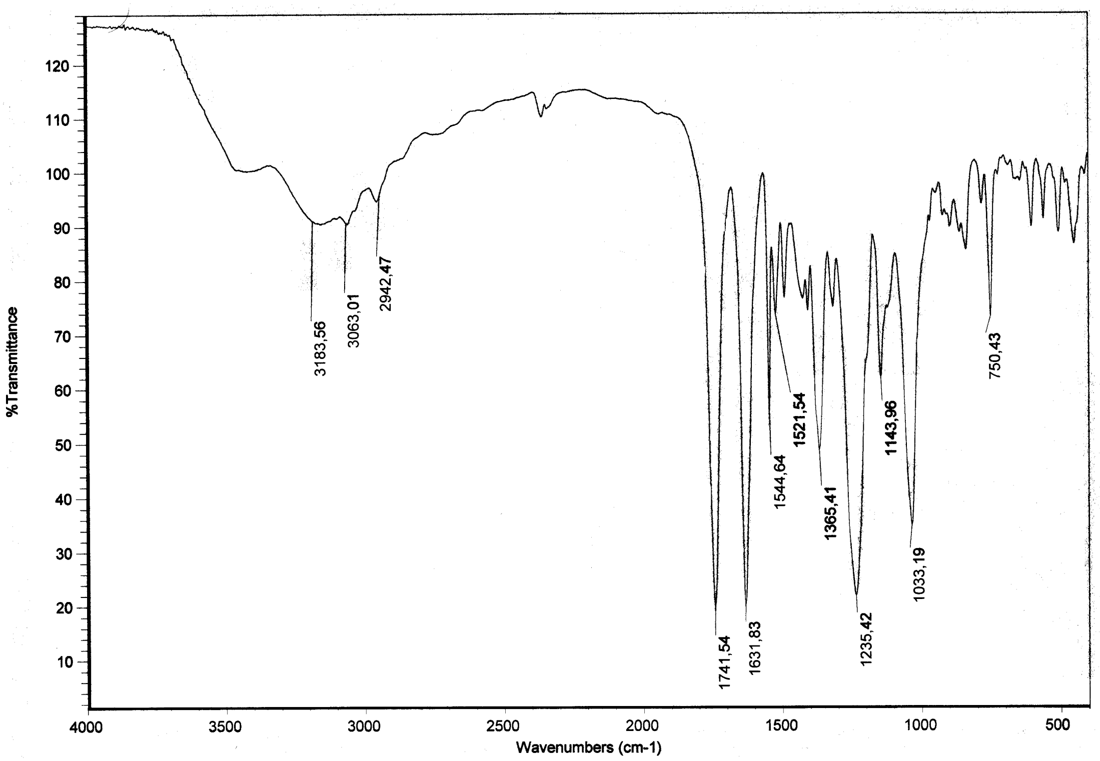

Figure 49.** IR spectrum of **50

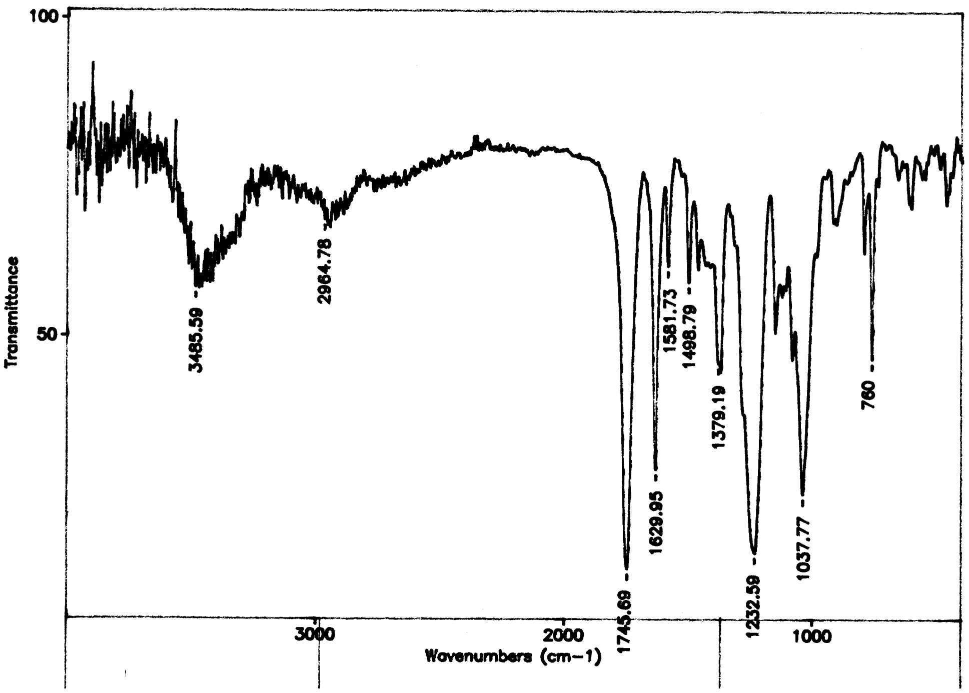

Figure S50. *IR spectrum of* ***52***

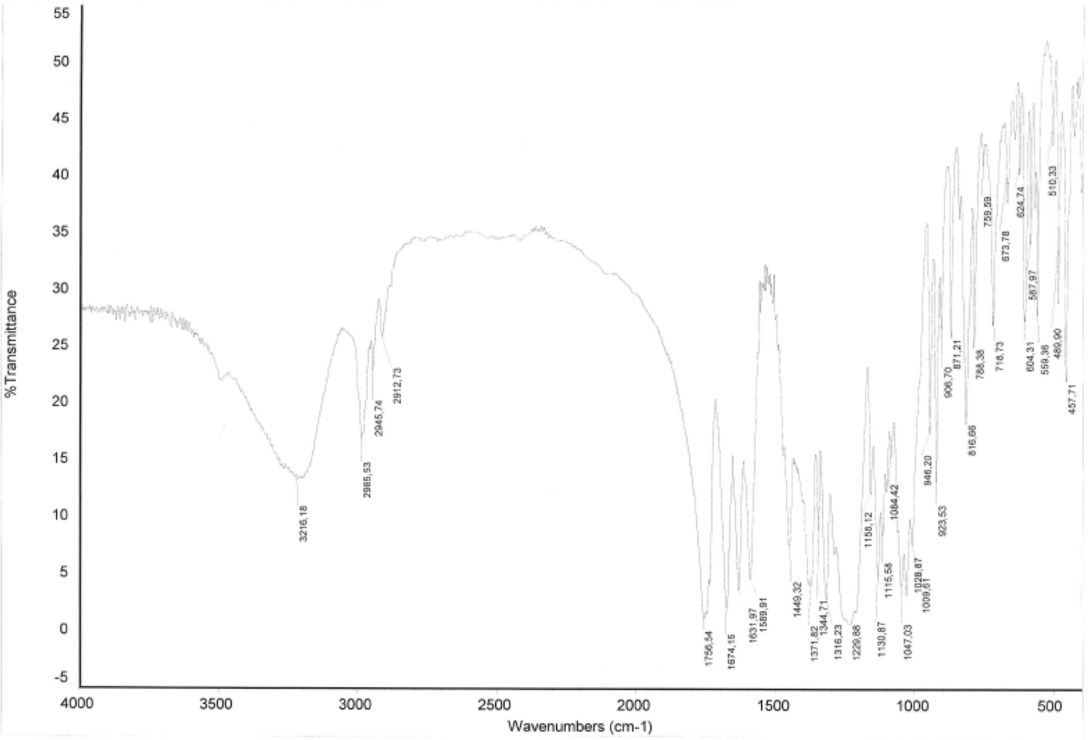

Figure S51. *IR spectrum of* ***95***

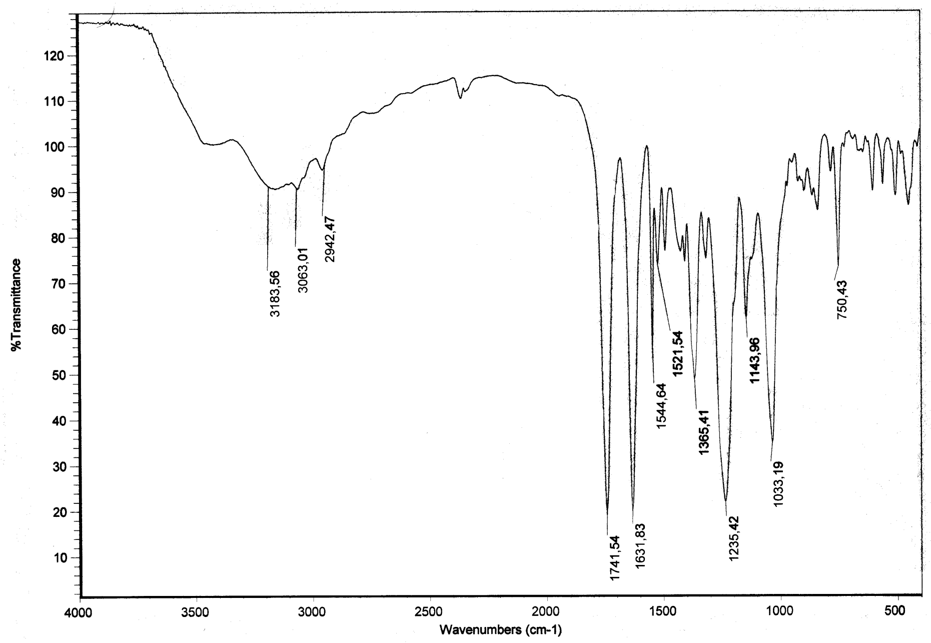

Figure S52. *IR spectrum of* ***96***

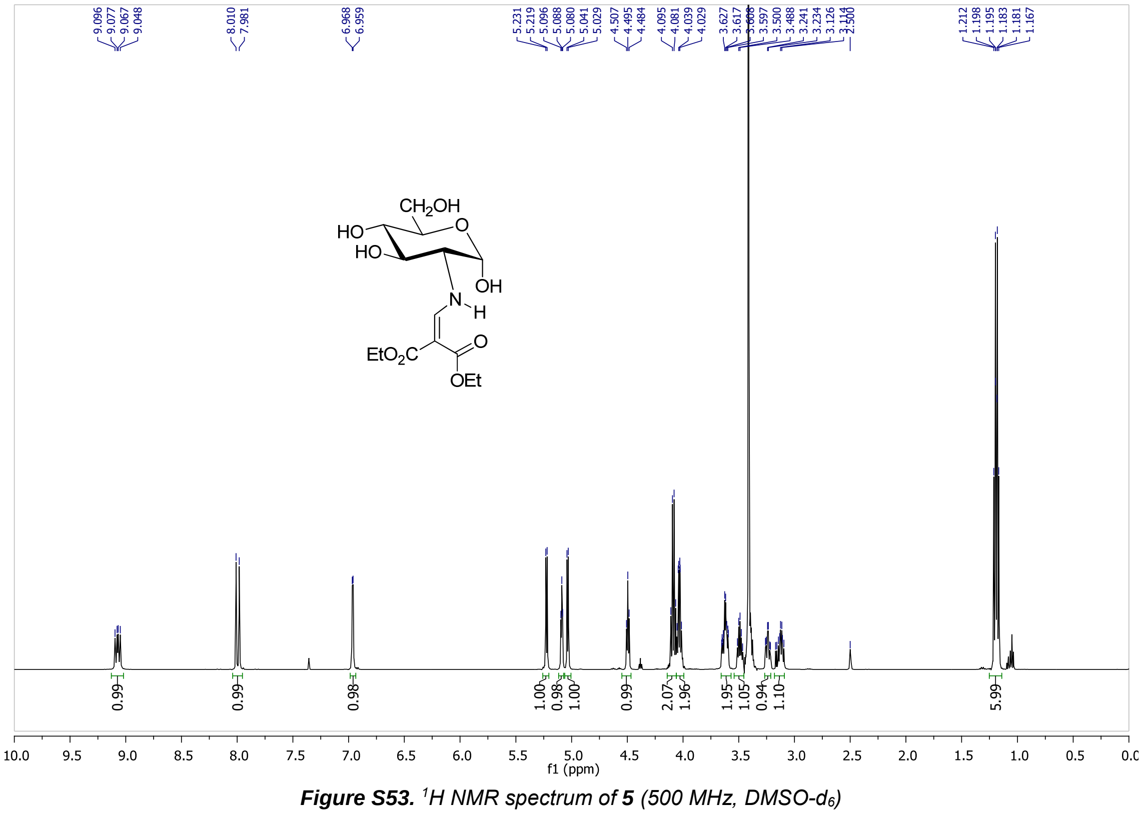

Figure S53. *^{1}H NMR spectrum of* ***5*** *(500 MHz, DMSO-d_6)*

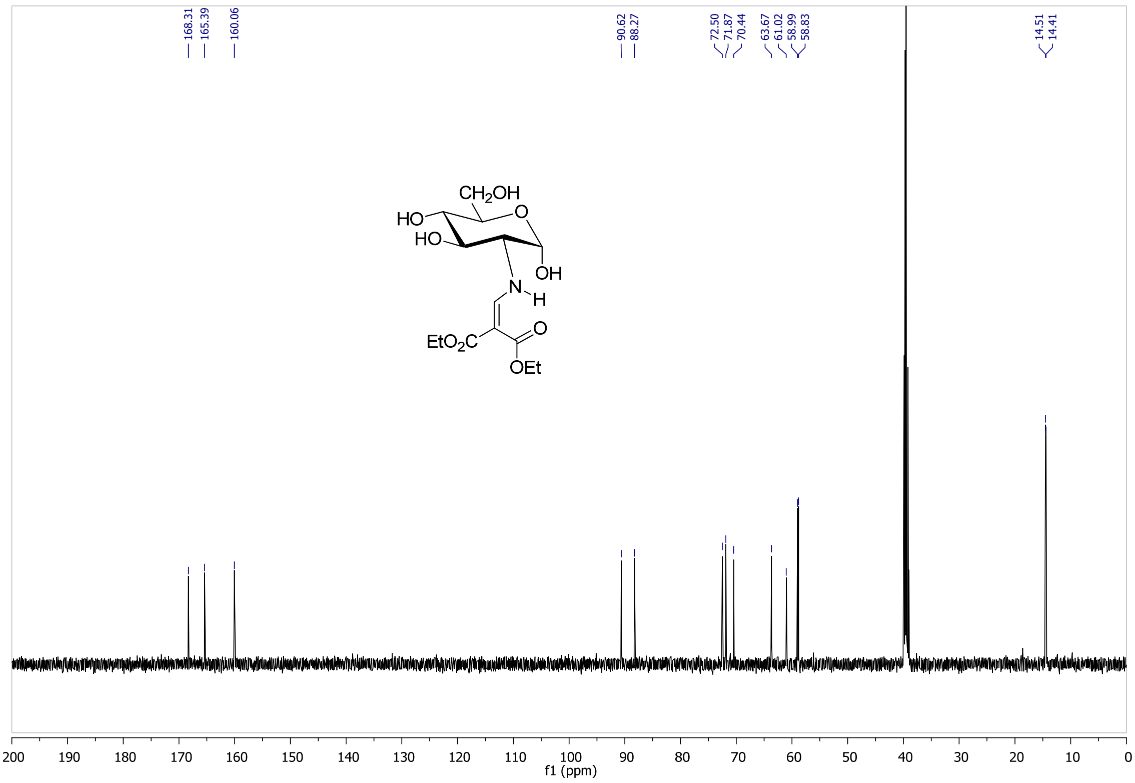

Figure S54. $^{13}C\{^{1}H\}$ *NMR spectrum of* ***5*** *(125 MHz, DMSO-d$_6$)*

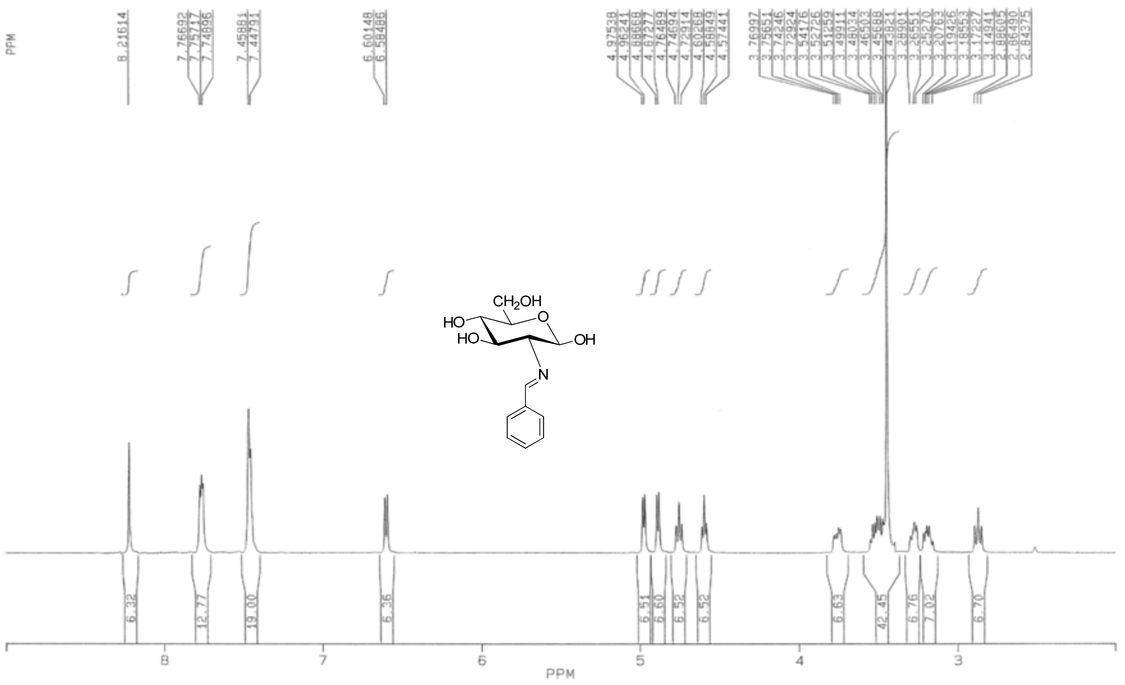

Figure S55. ^{1}H *NMR spectrum of* ***11*** *(400 MHz, DMSO-d$_6$)*

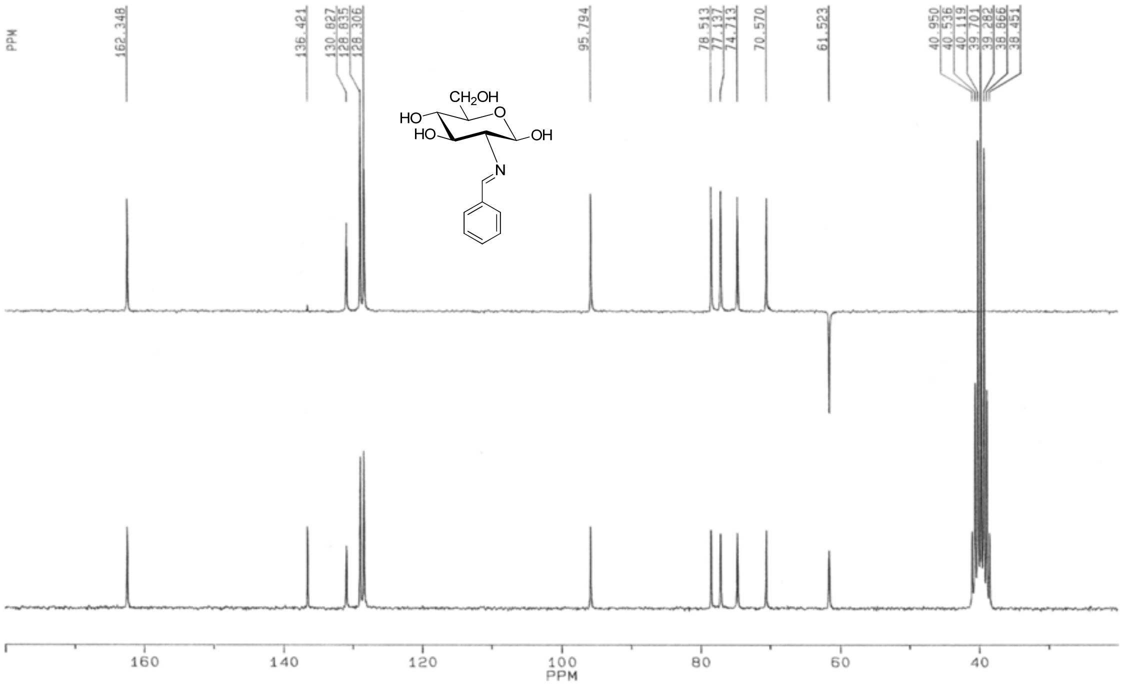

Figure S56. *$^{13}C\{^1H\}$ NMR spectra (top: DEPT) of **11** (100MHz, DMSO-d_6)*

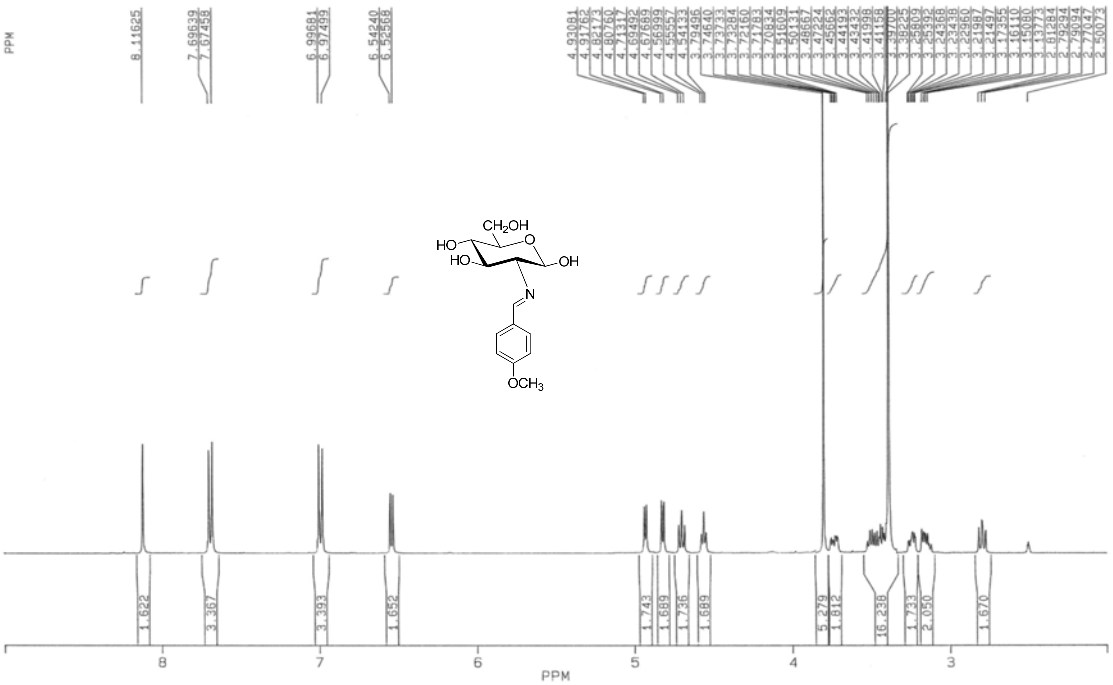

Figure S57. *^1H NMR spectrum of **12** (400 MHz, DMSO-d_6)*

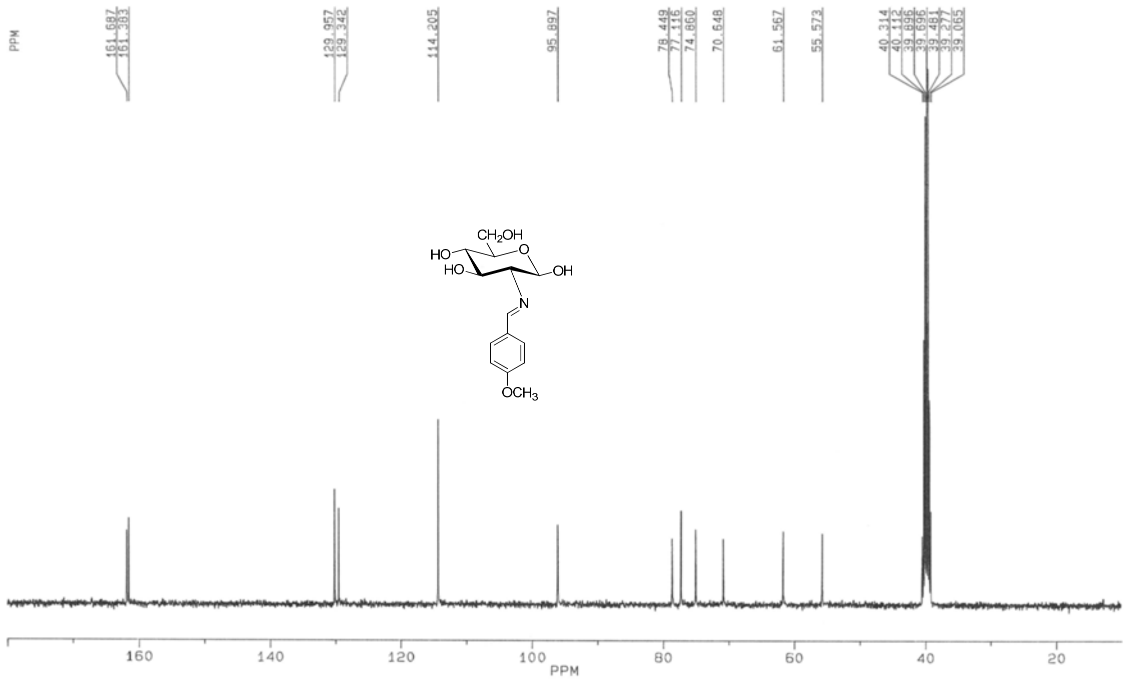

Figure S58. $^{13}C\{^{1}H\}$ NMR spectrum of **12** (100 MHz, DMSO-d_6)

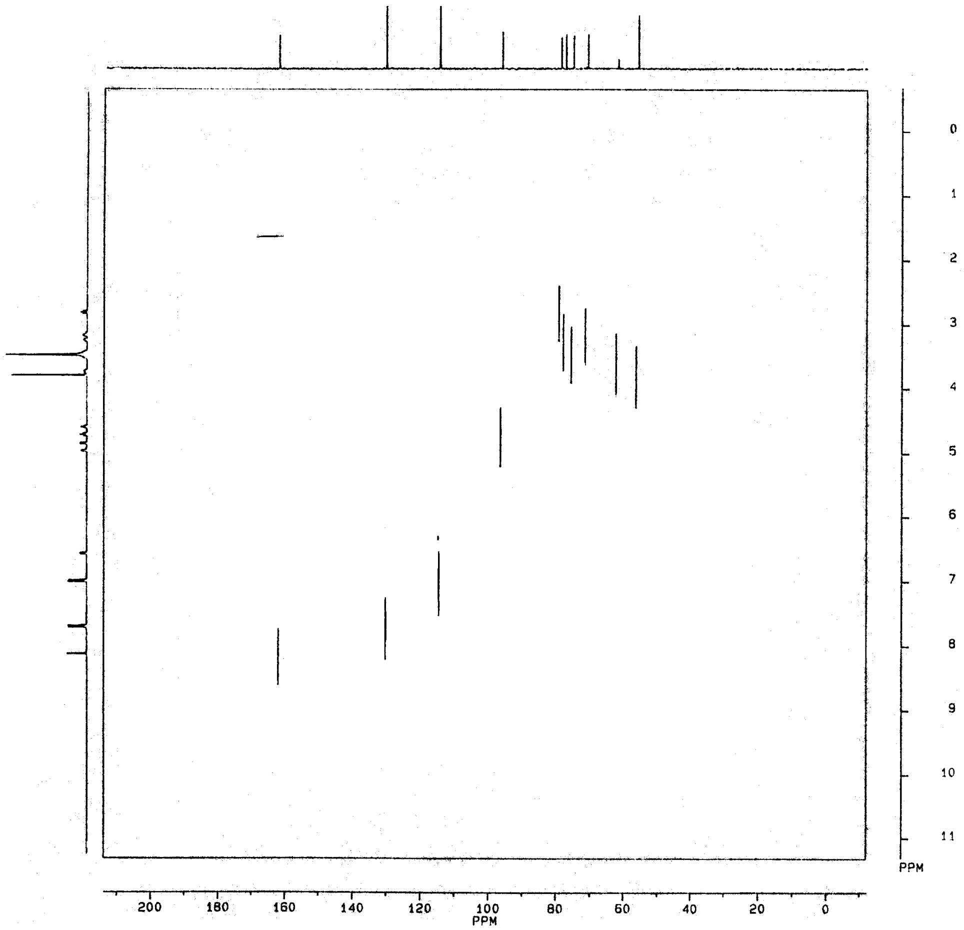

Figure S59. HMQC spectrum of **12** (DMSO-d_6)

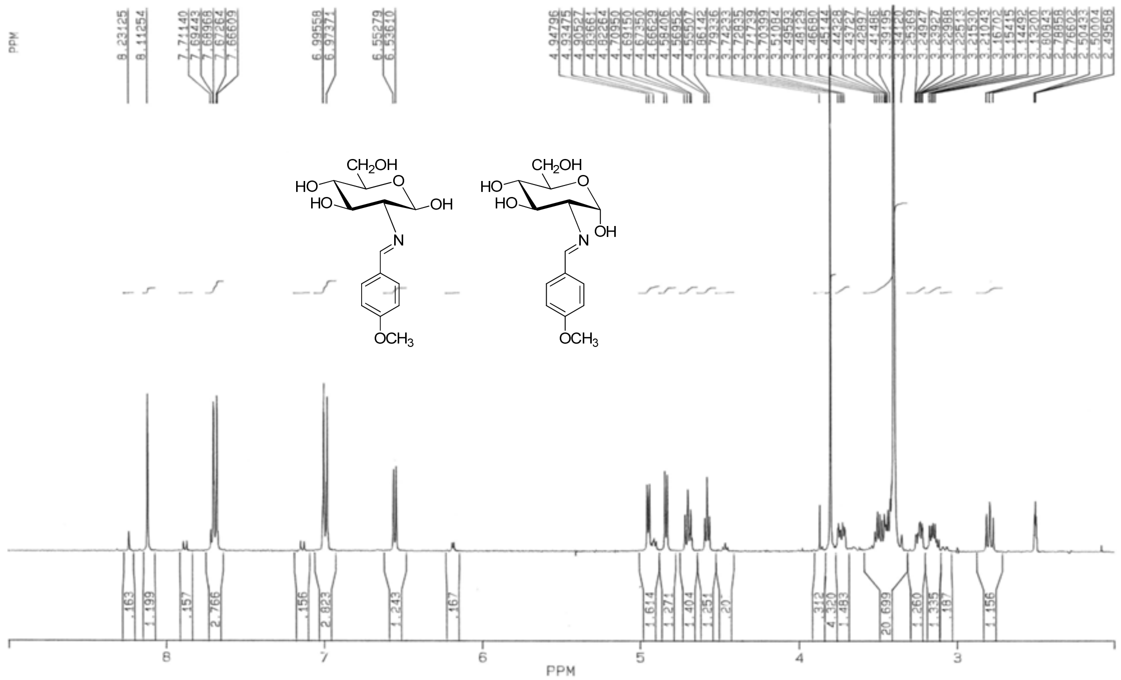

Figure S60. *^{1}H NMR spectrum of **12** and **55** (400 MHz, DMSO-d6)*

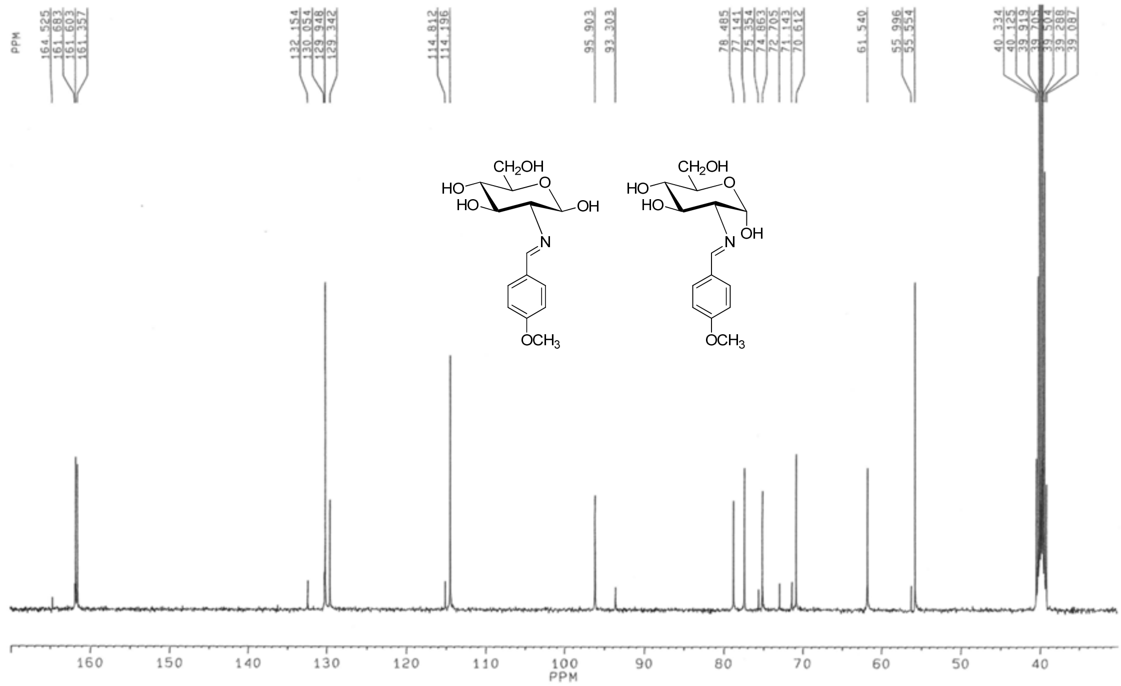

Figure 61. *^{13}C{^{1}H} NMR spectrum of **12** and **55** (100 MHz, DMSO-d6)*

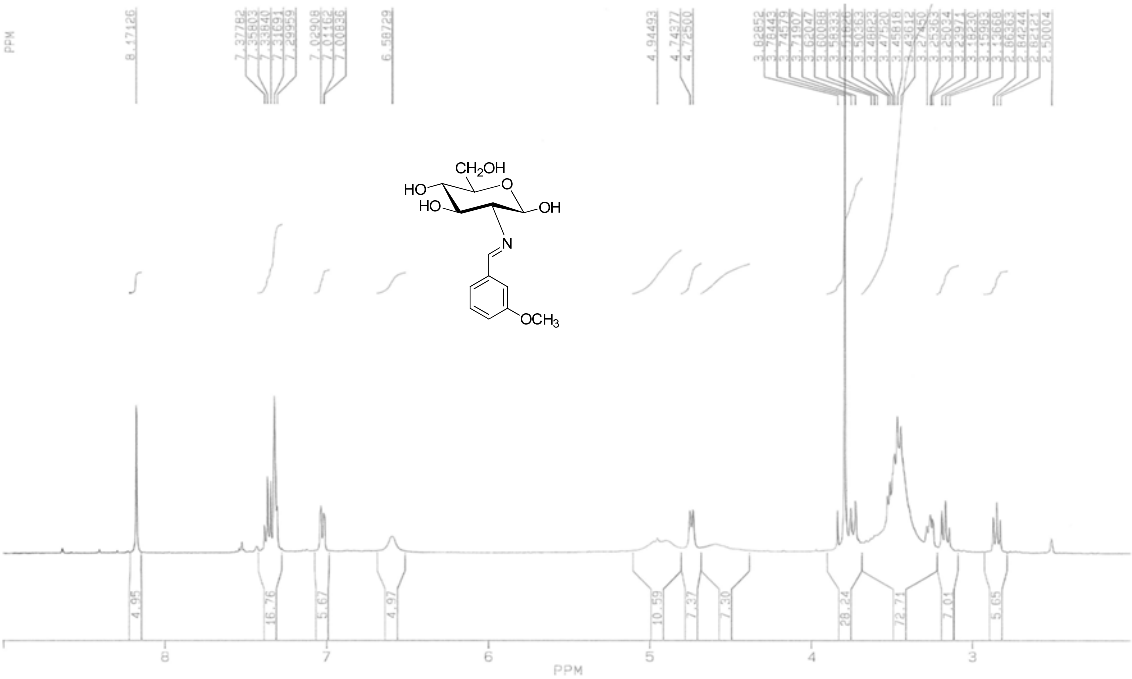

Figure S62. *^1H NMR spectrum of **13** (400 MHz, DMSO-d_6)*

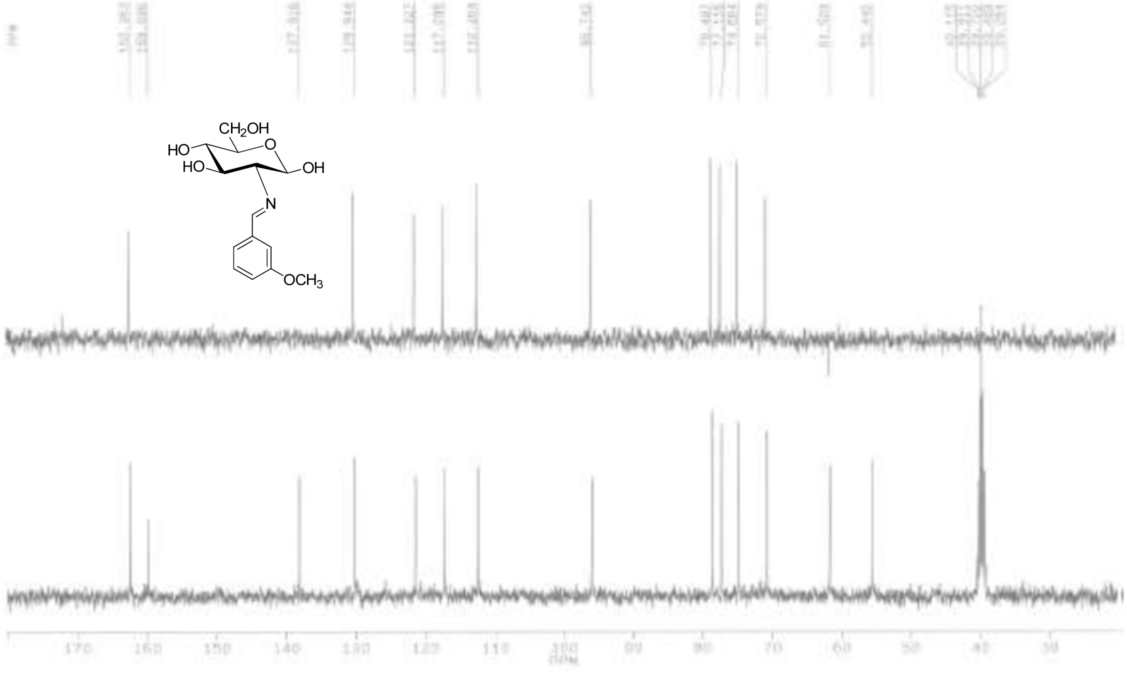

Figure S63. *$^{13}C\{^1H\}$ NMR (top: DEPT) spectra of **13** (100 MHz, DMSO-d_6)*

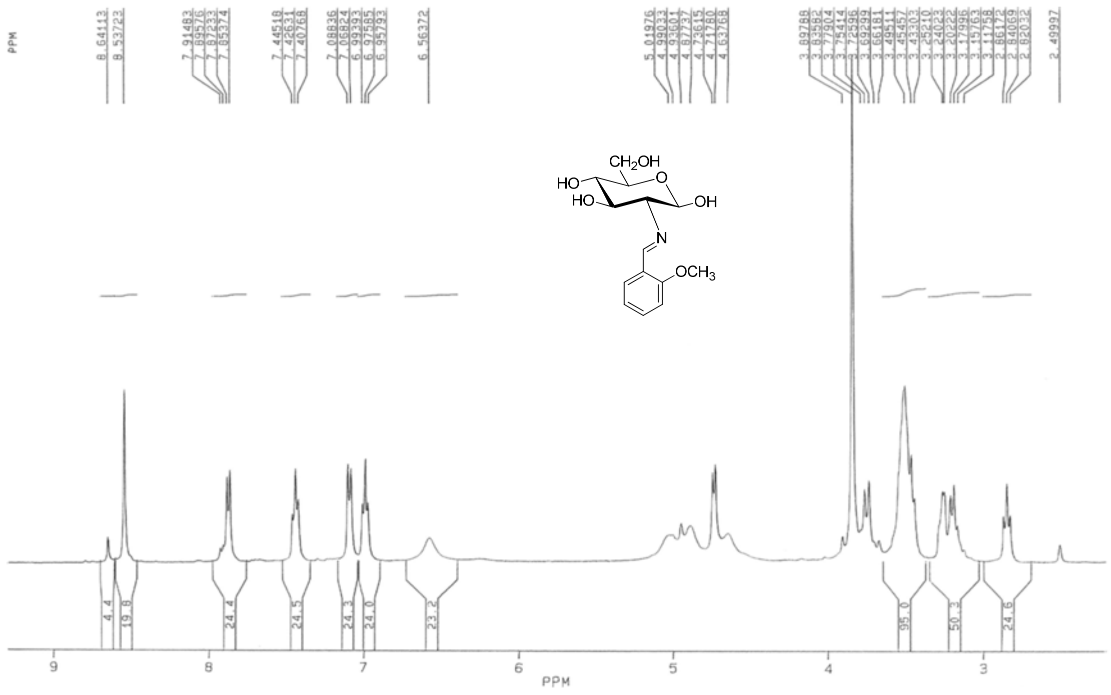

Figure S64. *^{1}H NMR spectrum of **14** (400 MHz, DMSO-d_6)*

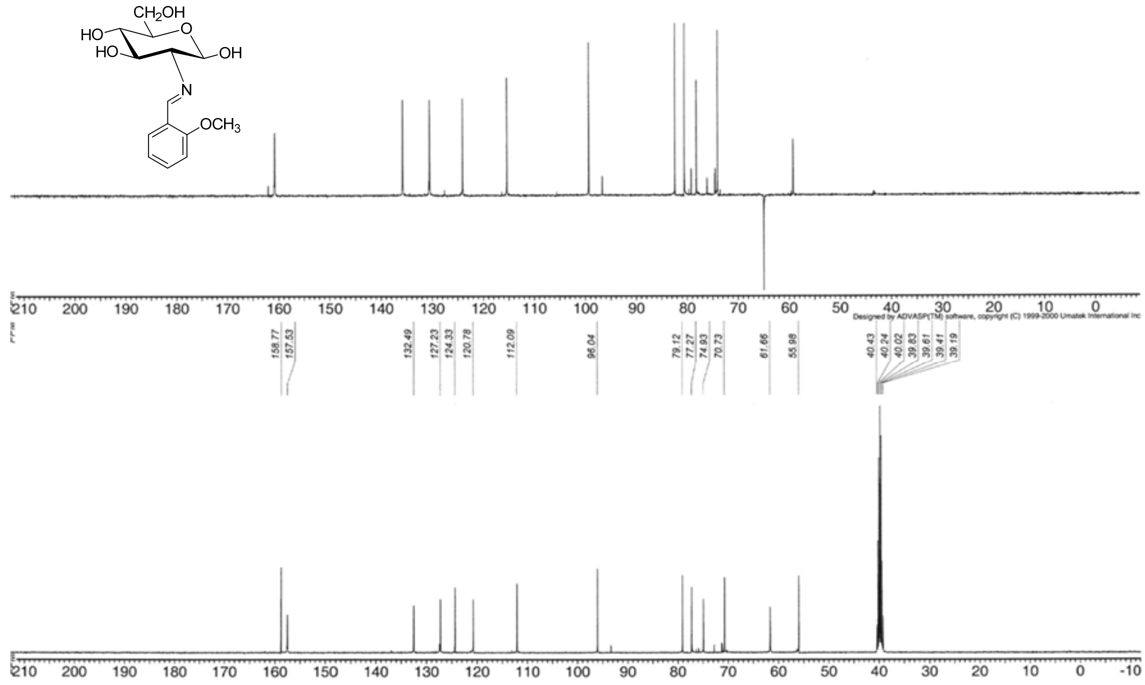

Figure S65. *^{13}C{^{1}H} NMR (top: DEPT) spectra of **14** (100 MHz, DMSO-d_6)*

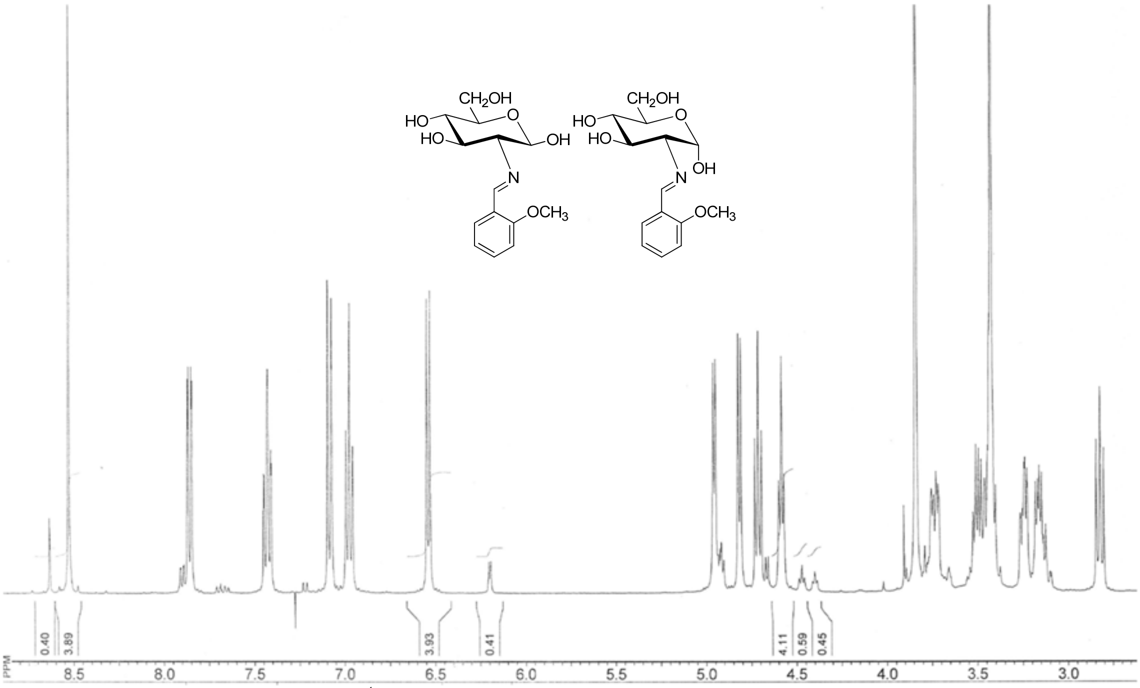

Figure S66. *^{1}H NMR spectrum of **14** and **57** (400 MHz, DMSO-d6)*

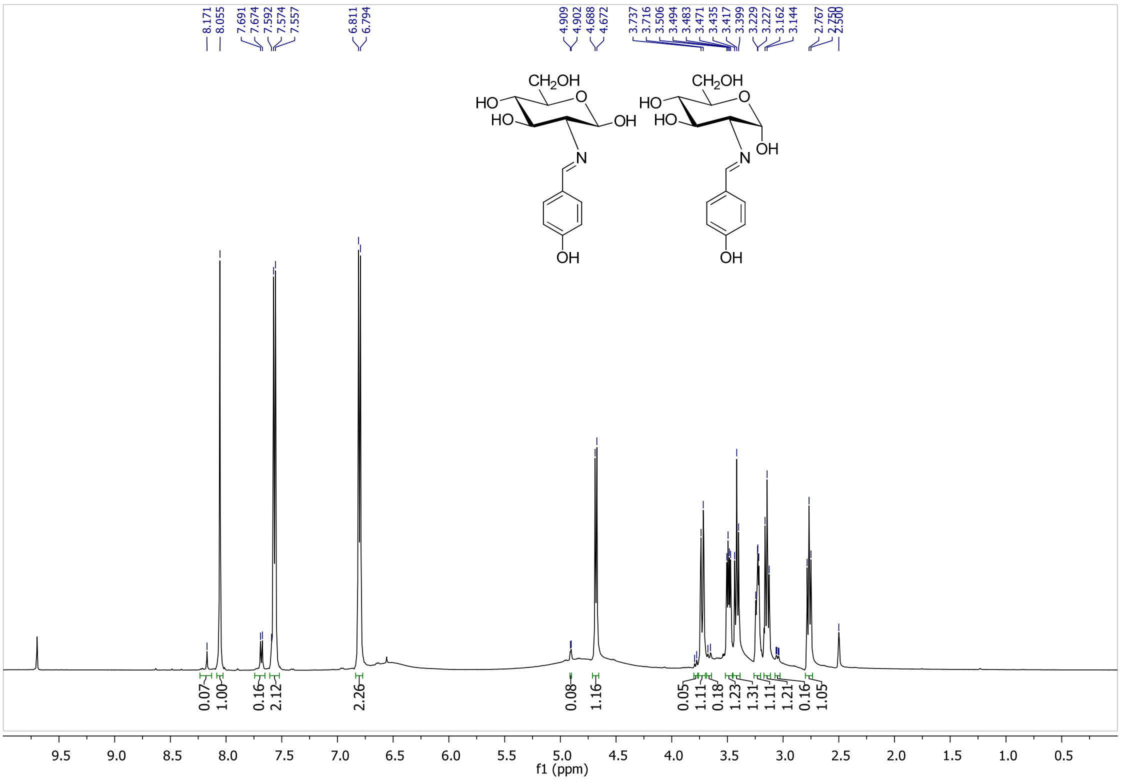

Figure S67. *^{1}H NMR spectrum of **15** and **58** (500 MHz, DMSO-d6)*

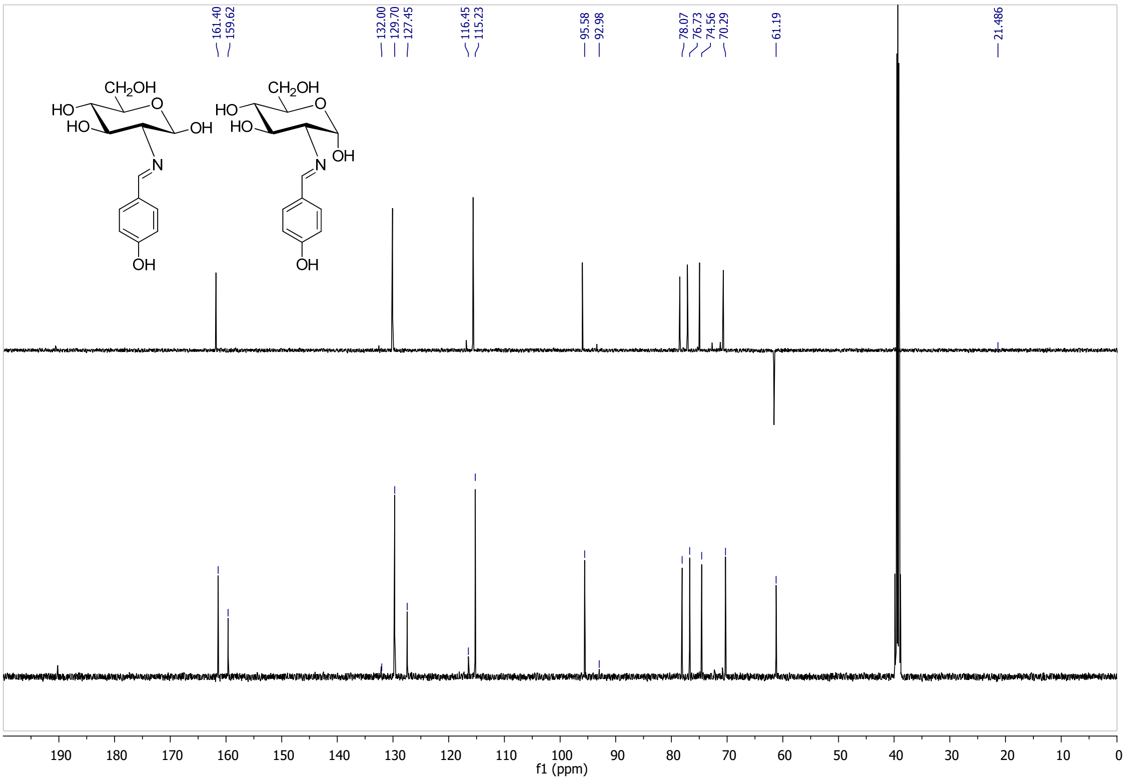

Figure S68. $^{13}C\{^1H\}$ *NMR (top: DEPT) spectra of* ***15*** *and* ***58*** *(125 MHz, DMSO-*d_6*)*

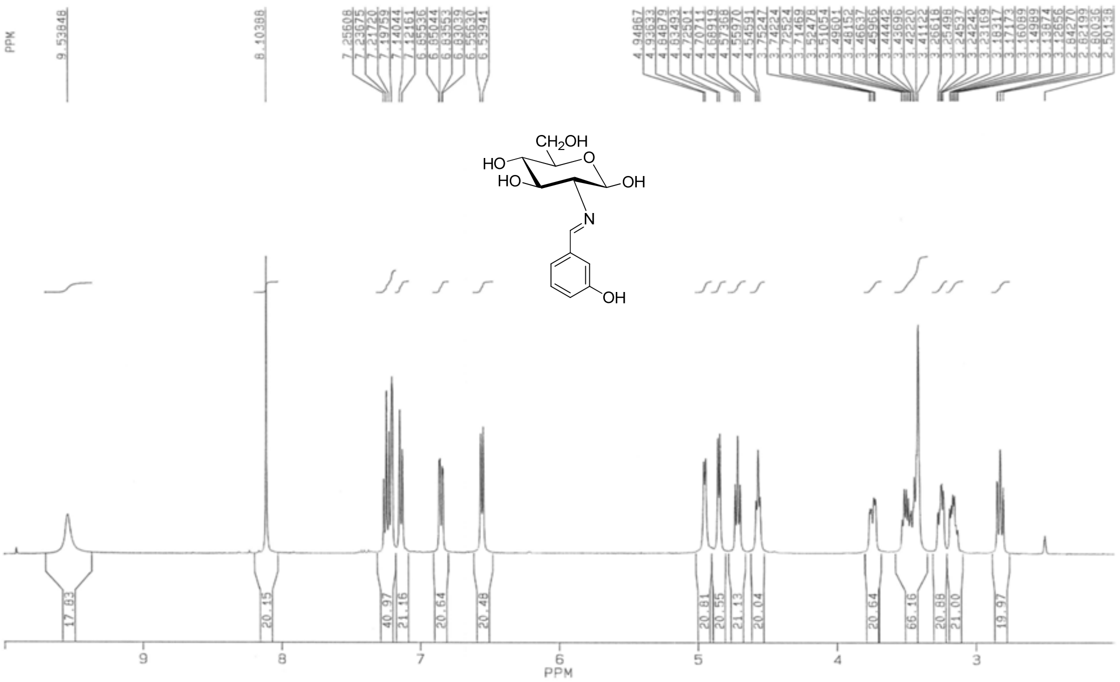

Figure S69. ^{1}H *NMR spectrum of* ***16*** *(400 MHz, DMSO-*d_6*)*

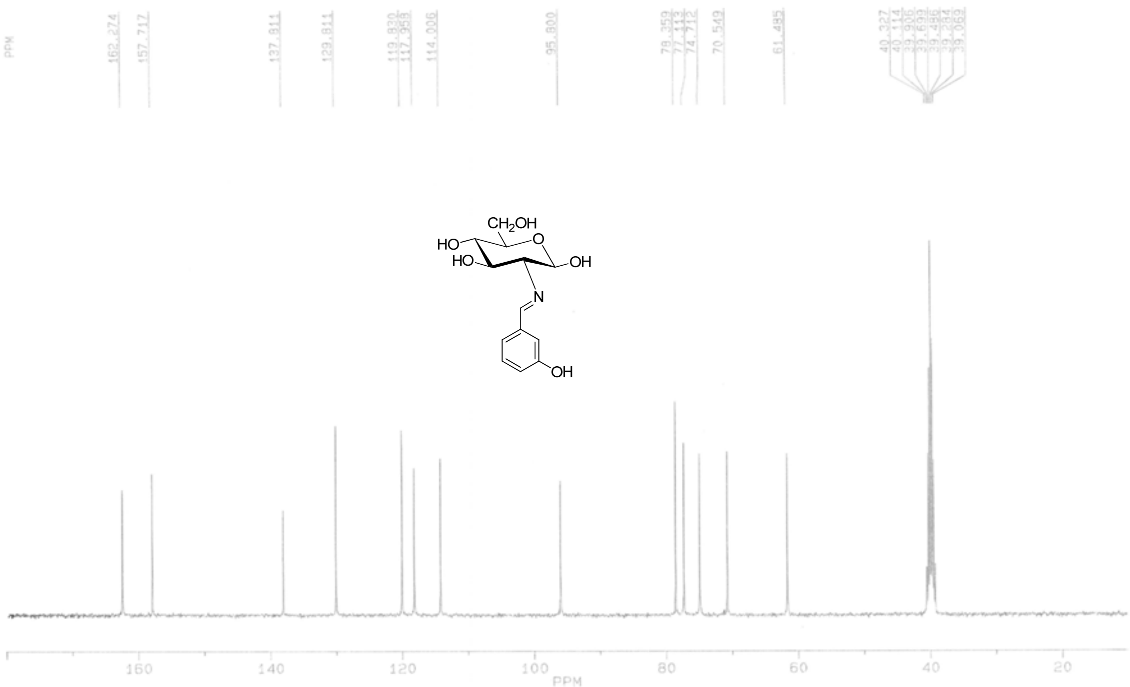

Figure S70. *$^{13}C\{^1H\}$ NMR spectrum of **16** (100 MHz, DMSO-d_6)*

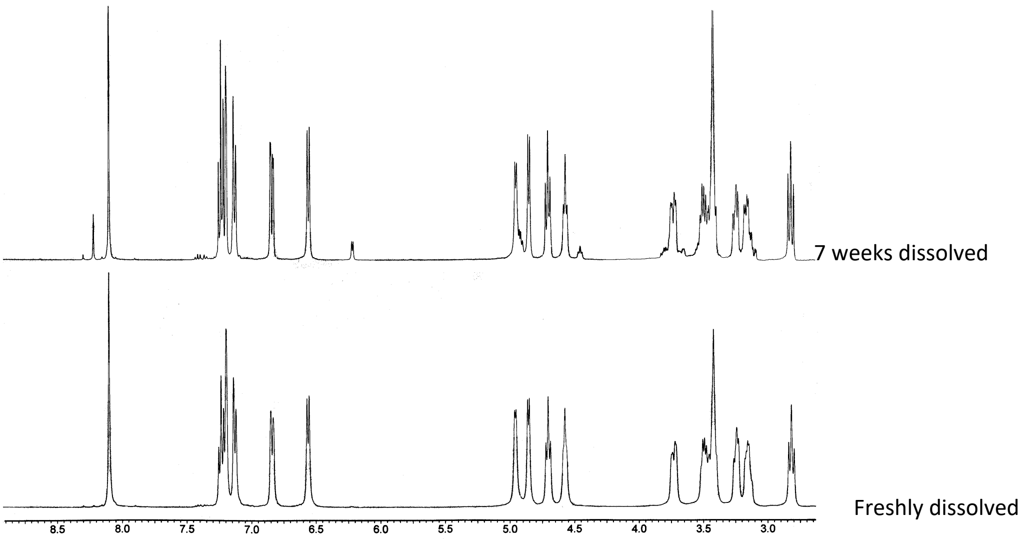

Figure S71. *^1H NMR spectra showing the temporal evolution of **16** in DMSO-d_6 solution*

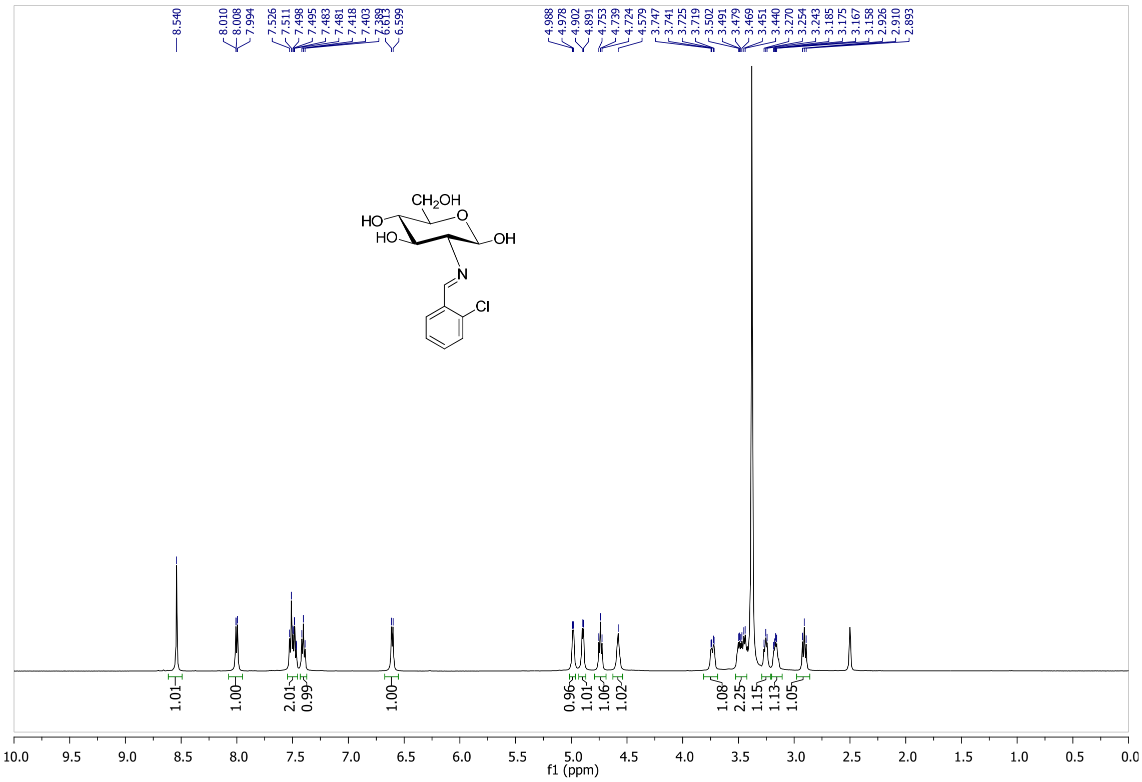

Figure S72. *^{1}H NMR spectrum of **17** (500 MHz, DMSO-d_6)*

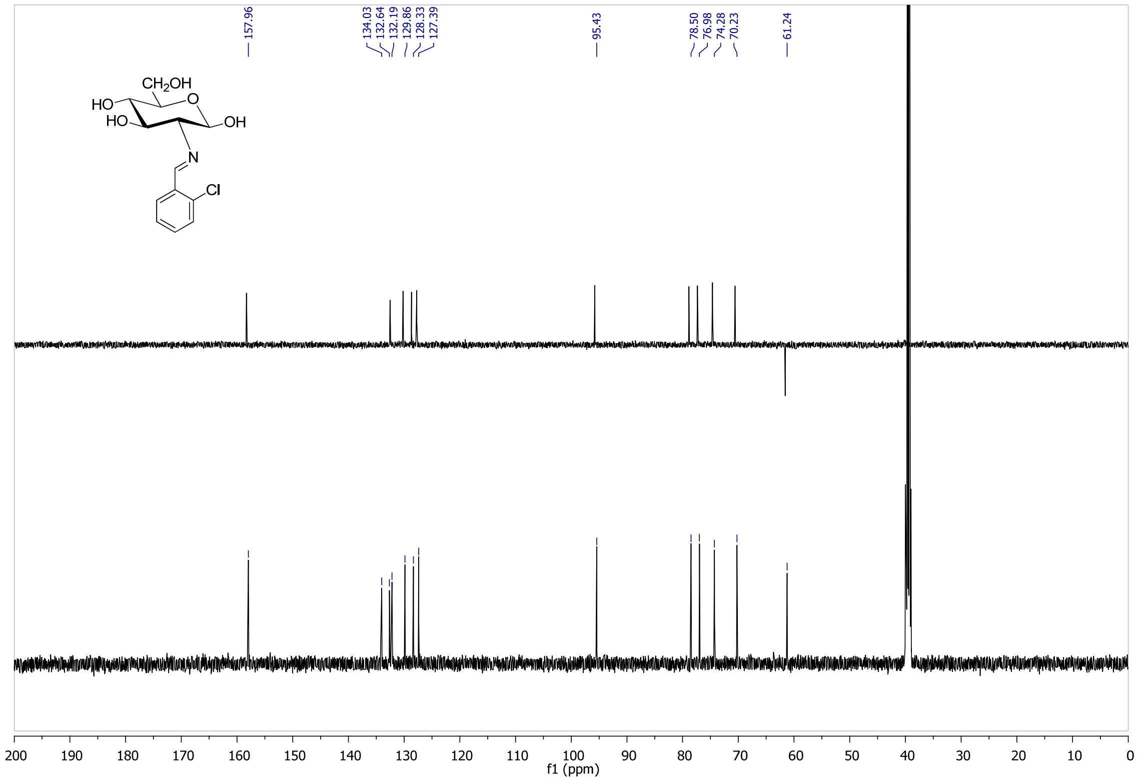

Figure S73. *^{13}C{^{1}H} NMR (top: DEPT) spectra of **17** (125 MHz, DMSO-d_6)*

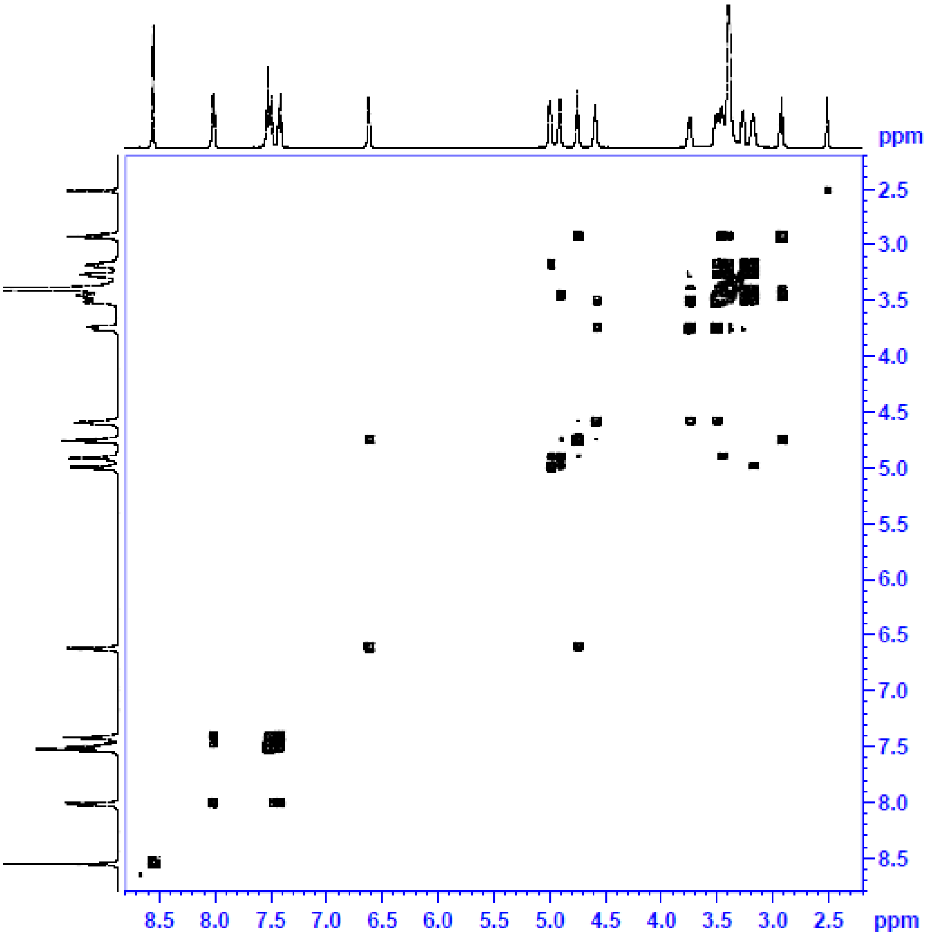

Figure S74. *COSY spectrum of **17** (DMSO-d₆)*

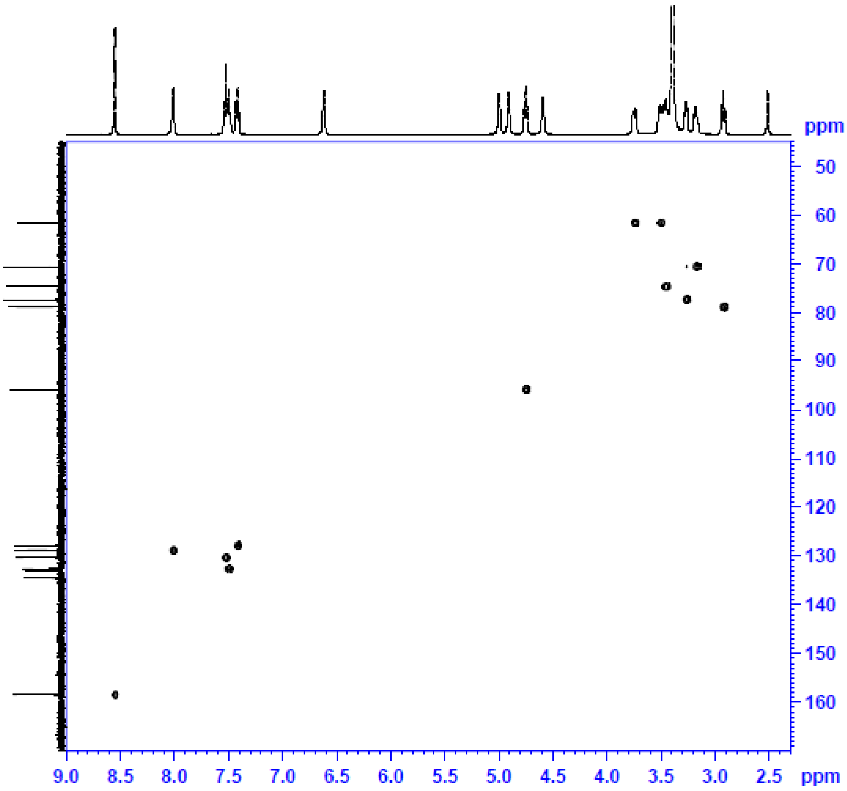

Figure S75. *HMQC spectrum of **17** (DMSO-d_6)*

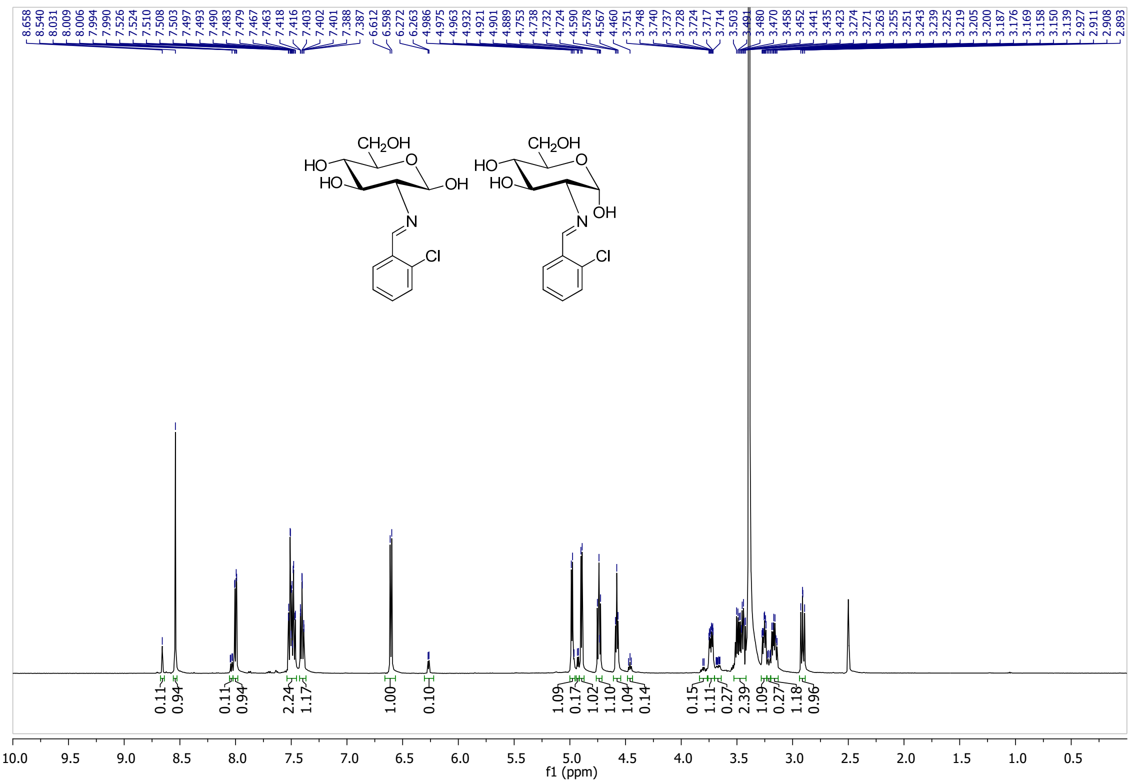

Figure S76. *^{1}H NMR spectrum of **17** and **60** (500 MHz, DMSO-d_6)*

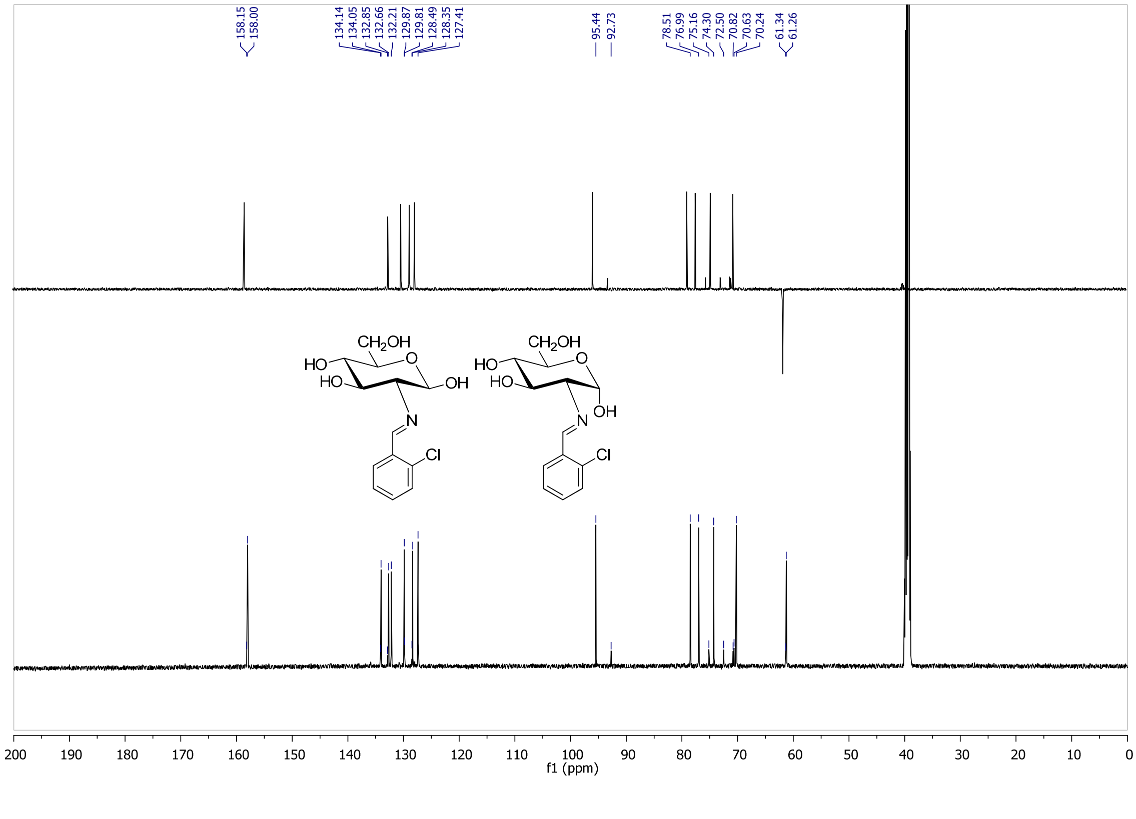

Figure S77. *$^{13}C\{^{1}H\}$ NMR (top: DEPT) spectra of **17** and **60** (125 MHz, DMSO-d_6)*

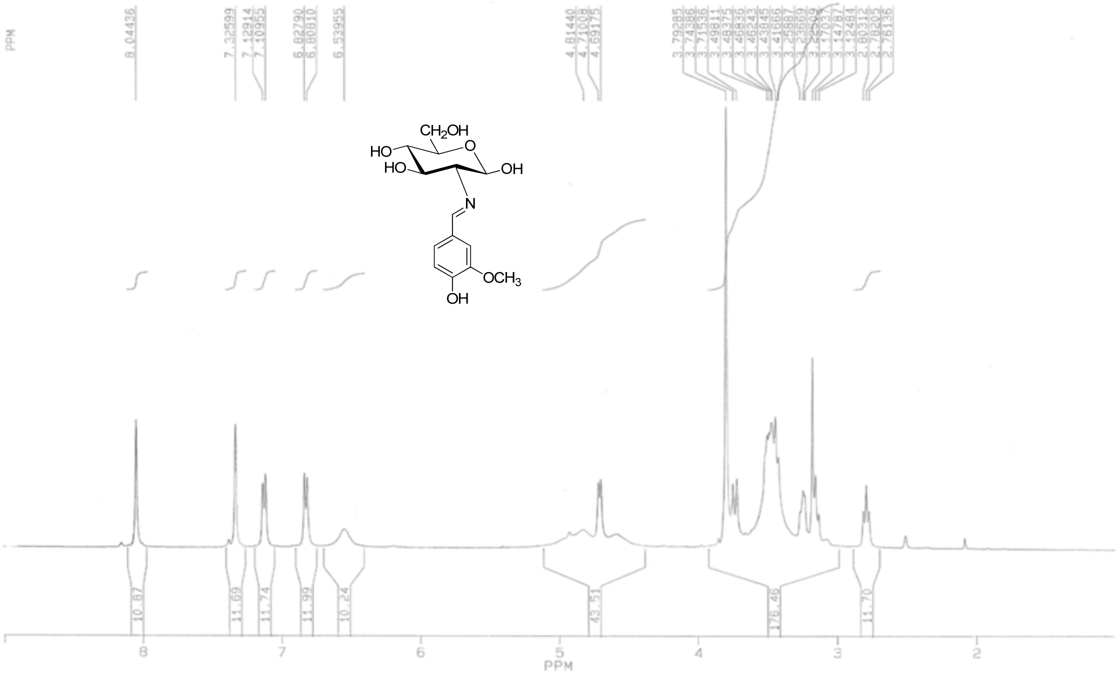

Figure S78. *^{1}H NMR spectrum of* ***18*** *(400 MHz, DMSO-d_6)*

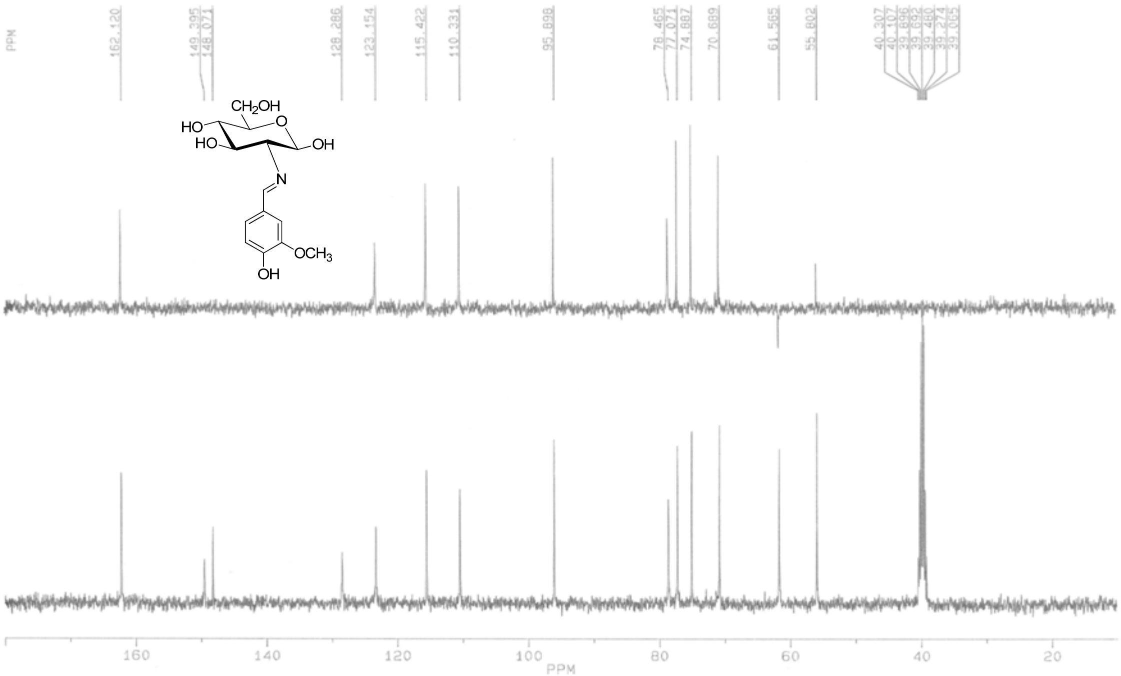

Figure S79. *$^{13}C\{^{1}H\}$ NMR (top: DEPT) spectra of* ***18*** *(100 MHz, DMSO-d_6)*

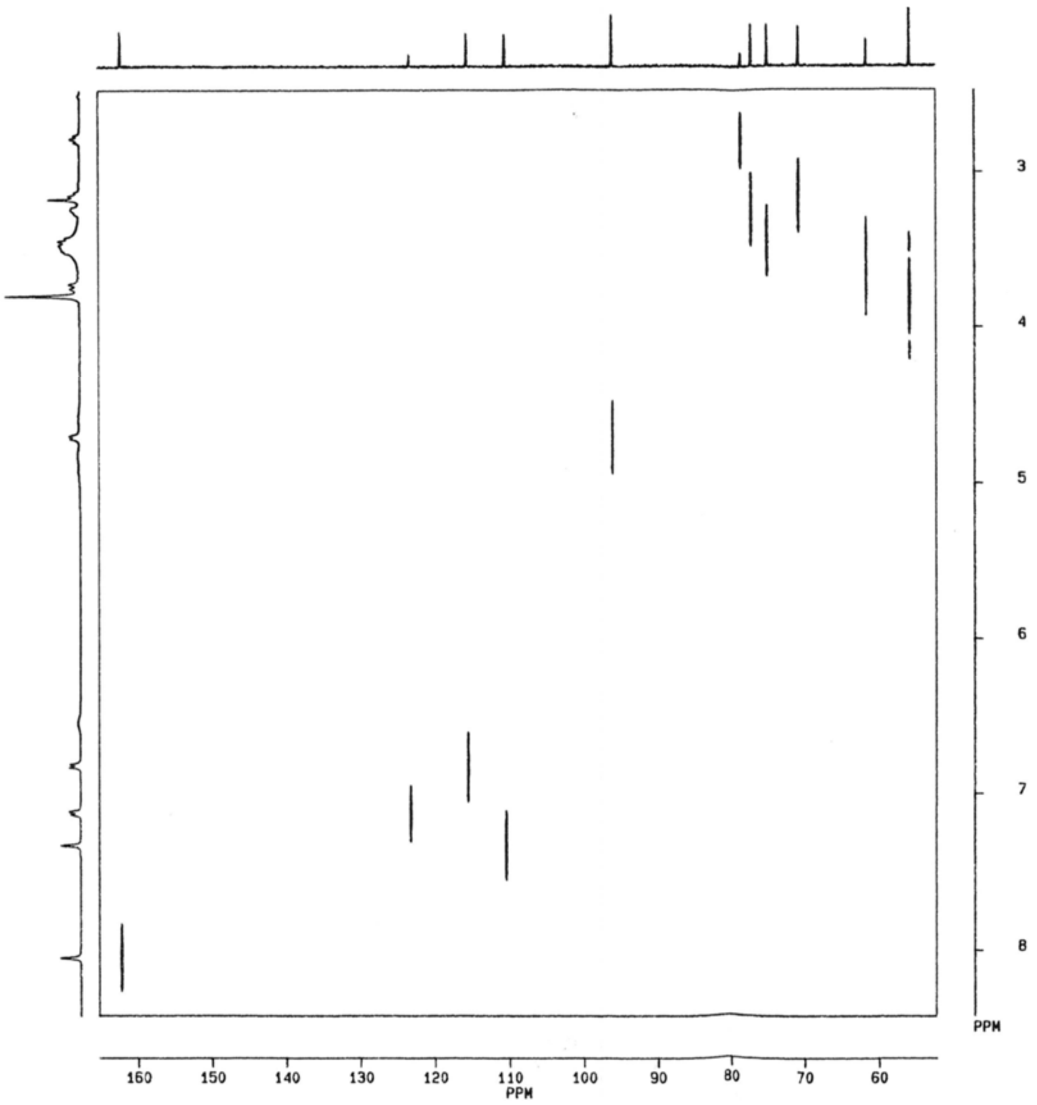

Figure S80. *HMQC spectrum of **18** (DMSO-d_6)*

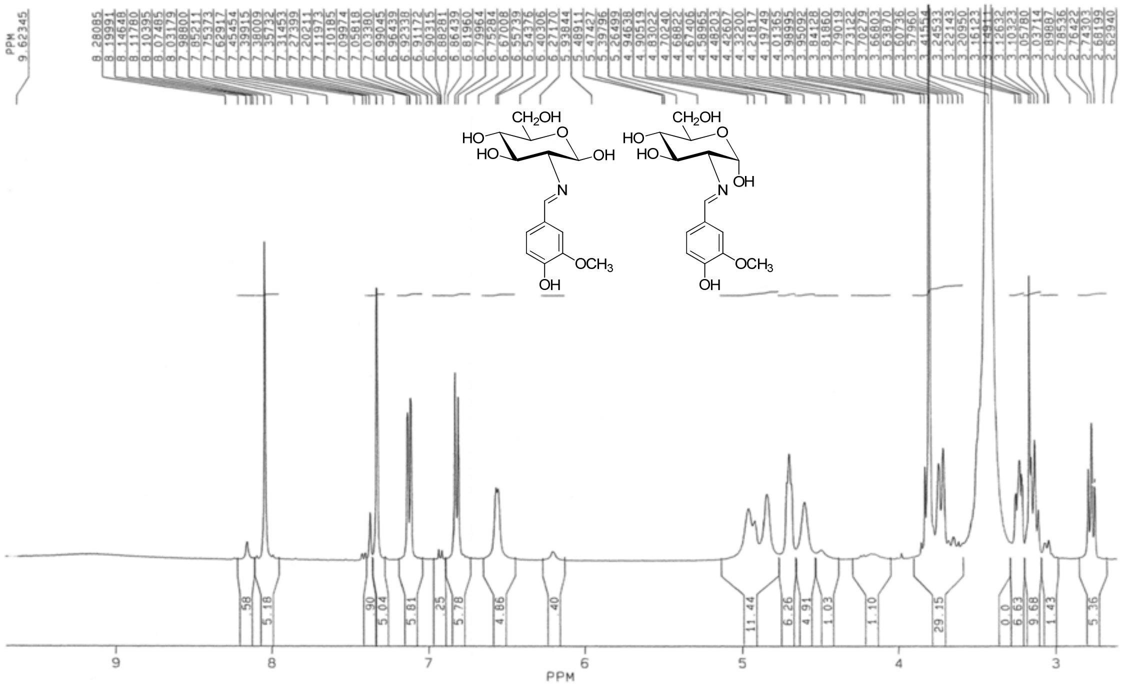

Figure S81. ^{1}H NMR spectrum of **18** and **61** (400 MHz, DMSO-d_6)

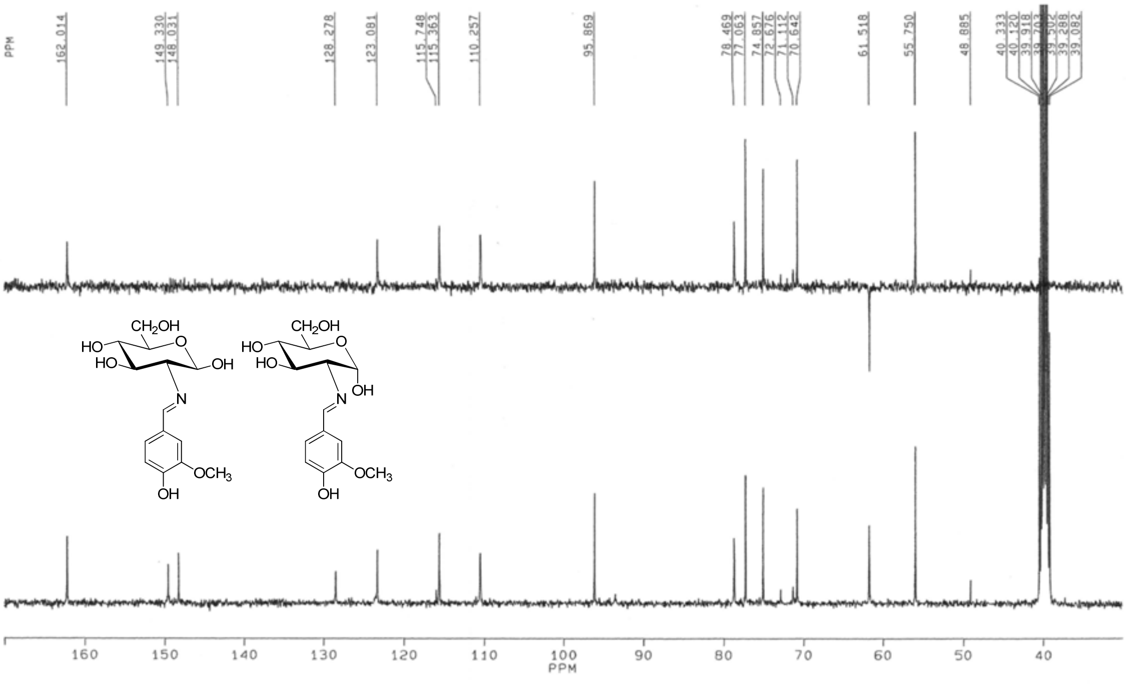

Figure S82. $^{13}C\{^{1}H\}$ NMR (top: dept) spectra of **18** and **61** (100 MHz, DMSO-d_6)

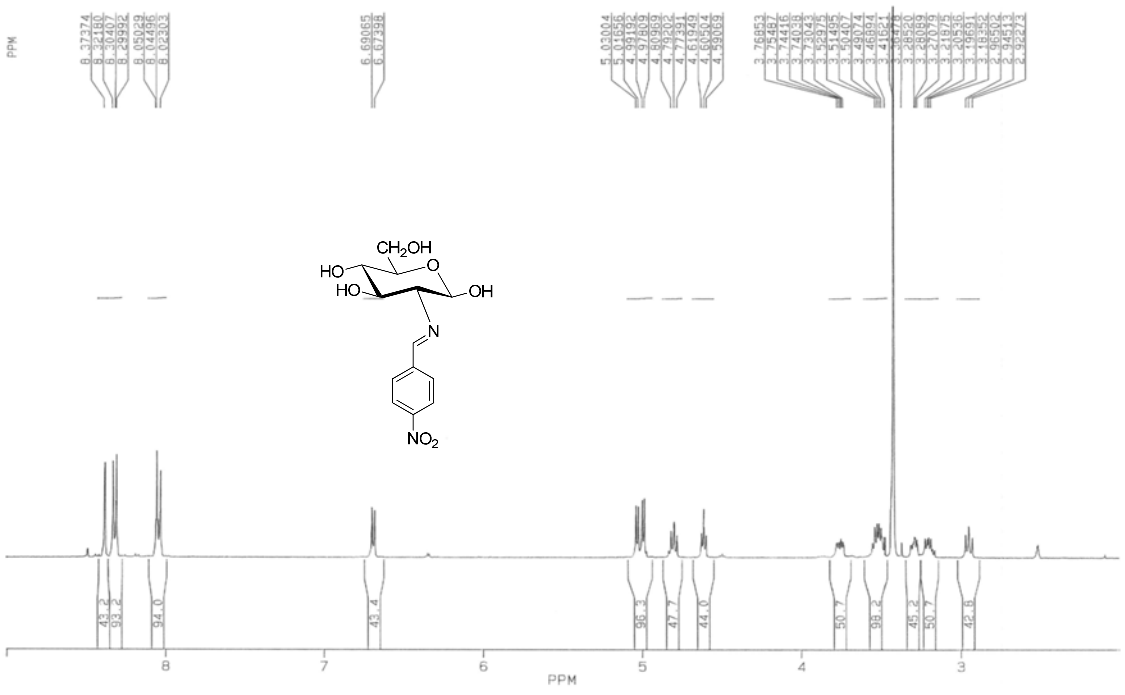

Figure S83. *^{1}H NMR spectrum of* ***19*** *(400 MHz, DMSO-d6)*

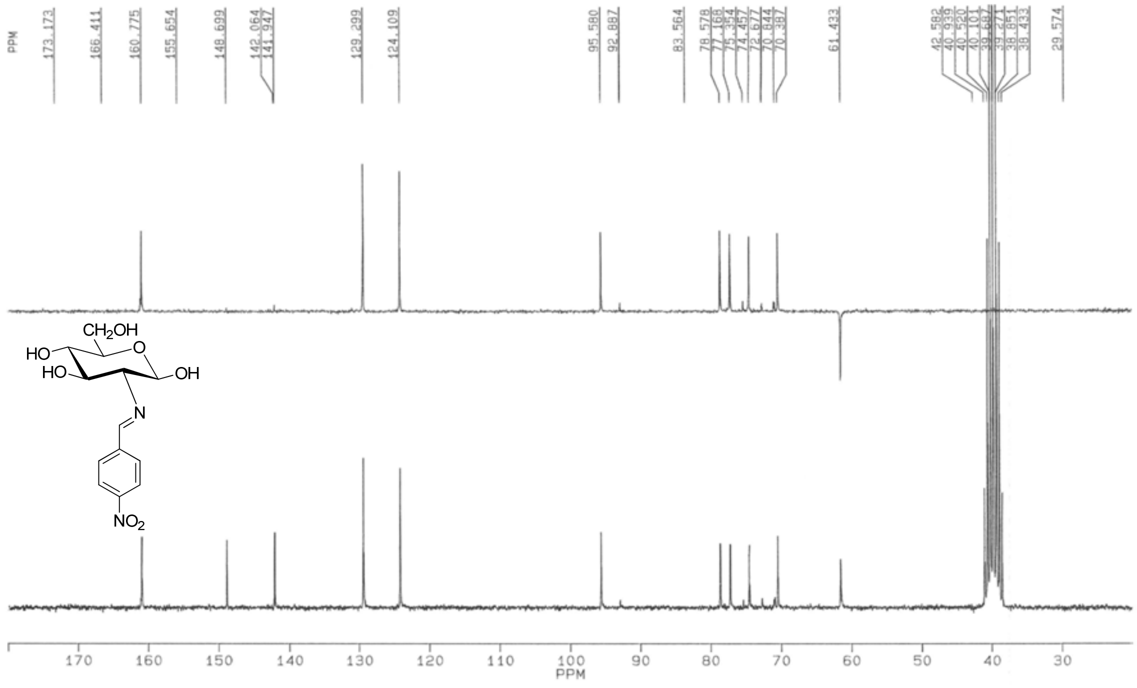

Figure S84. *^{13}C{^{1}H} NMR (top: DEPT) spectra of* **19** *(100 MHz, DMSO-d6)*

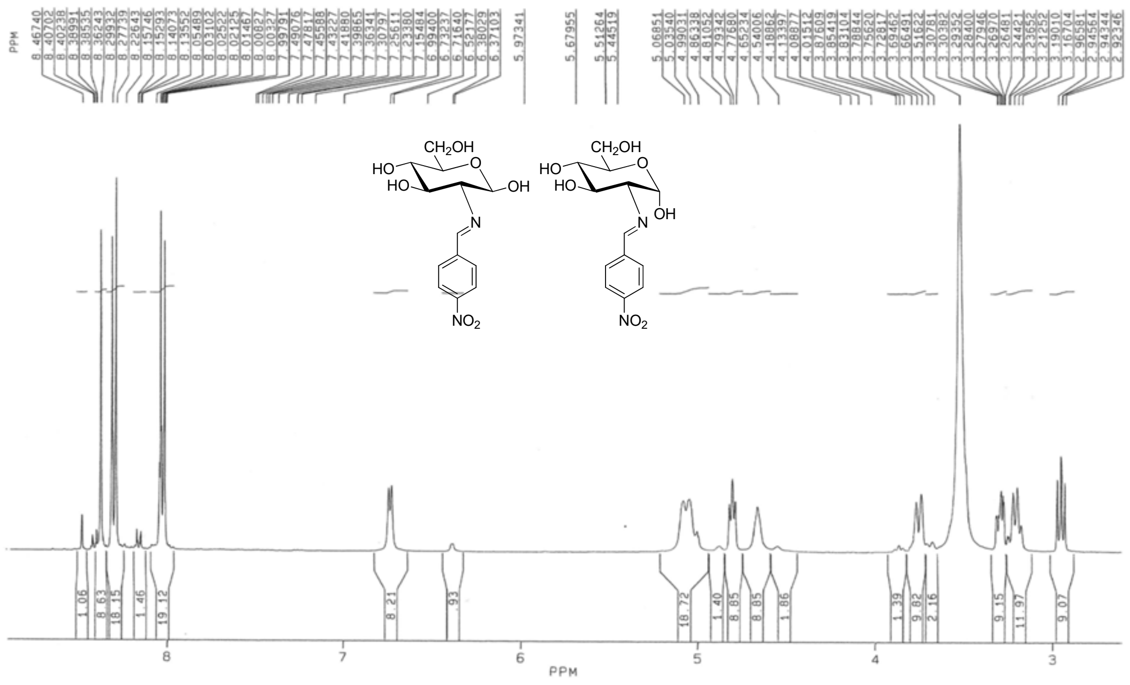

Figure S85. *^{1}H NMR spectrum of* **19** *and* **62** *(400 MHz, DMSO-d$_6$)*

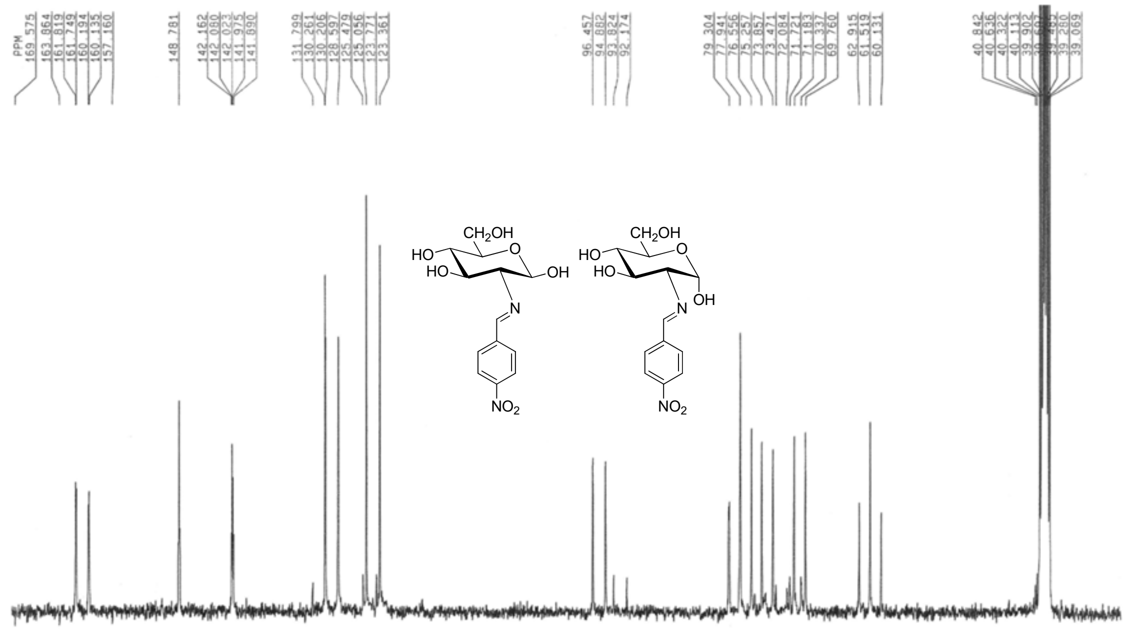

Figure S86. *Proton-coupled ^{13}C NMR spectrum of* ***19*** *and* ***62*** *(100 MHz, DMSO-d$_6$)*

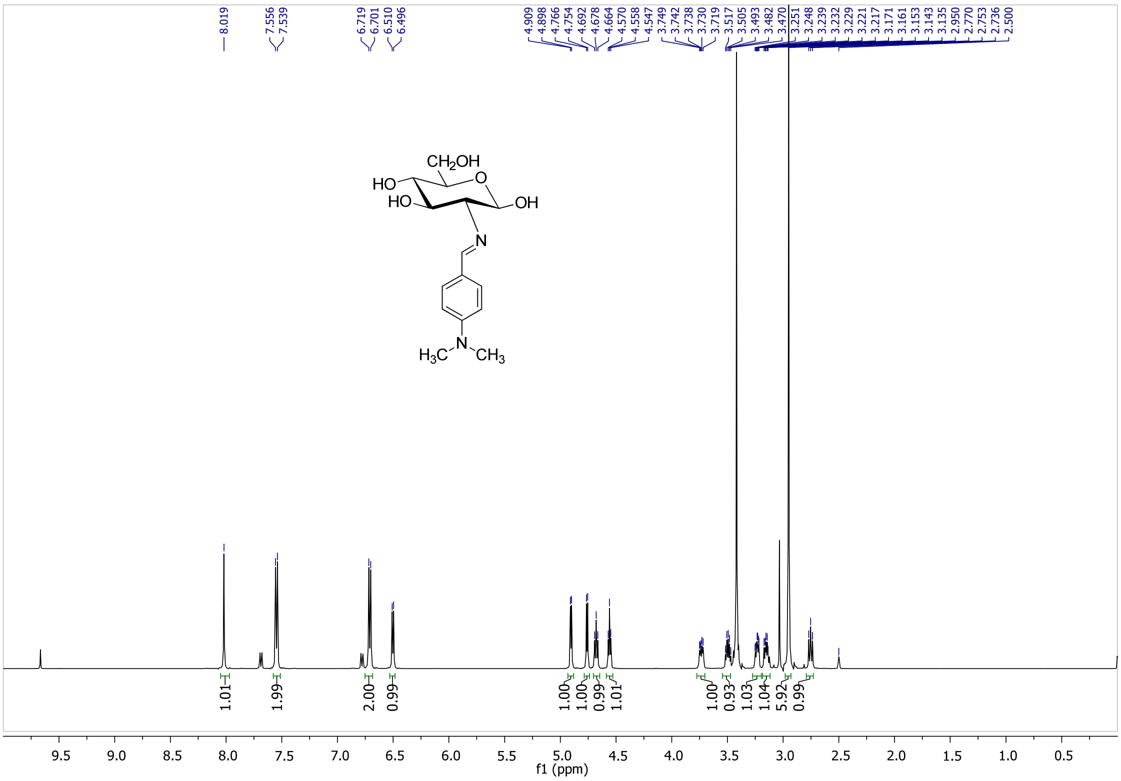

Figure S87. [1]*H NMR spectrum of **20** (500 MHz, DMSO-d6)*

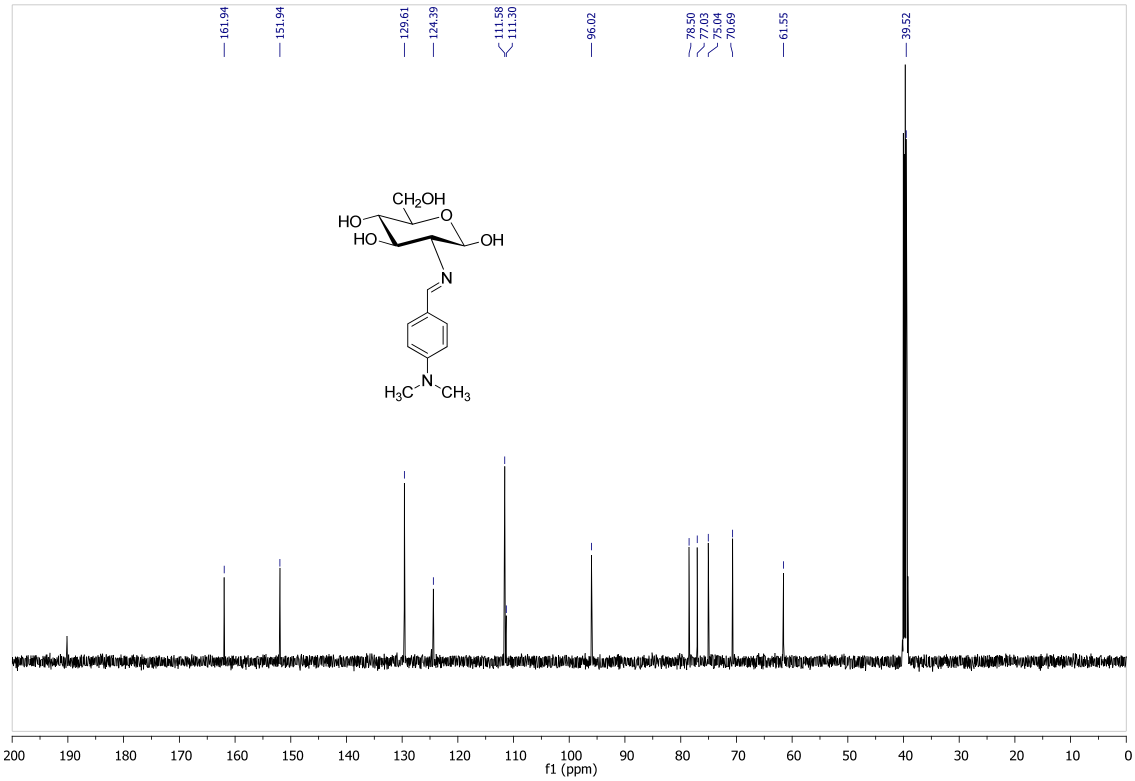

Figure S88. [13]*C{*[1]*H} NMR spectrum of **20** (125 MHz, DMSO-d6)*

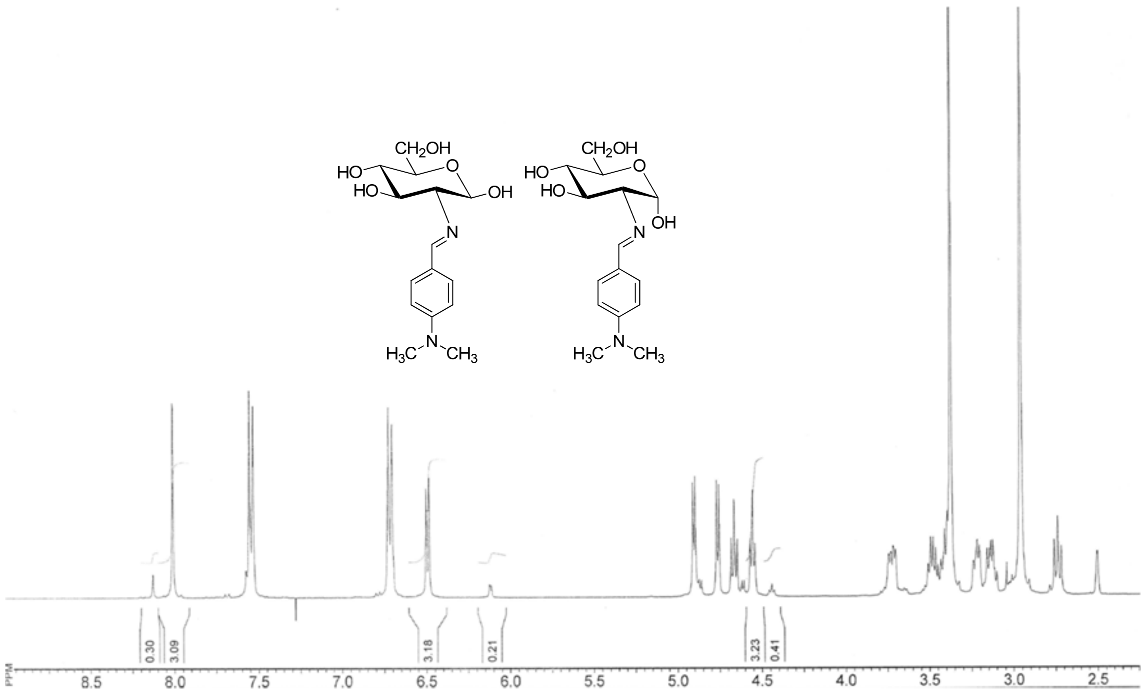

Figure S89. *^{1}H NMR spectrum of* **20** *and* **63** *(400 MHz, DMSO-d₆)*

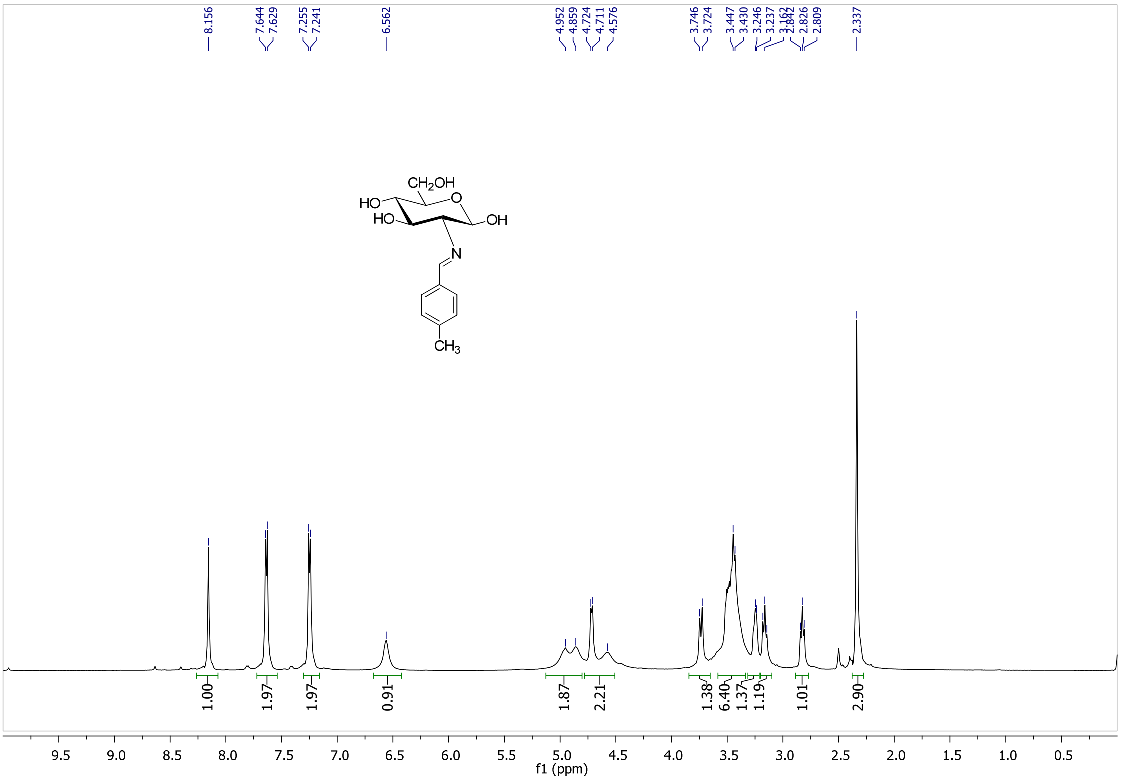

Figure S90. *^{1}H NMR spectrum of* **21** *(500 MHz, DMSO-d₆)*

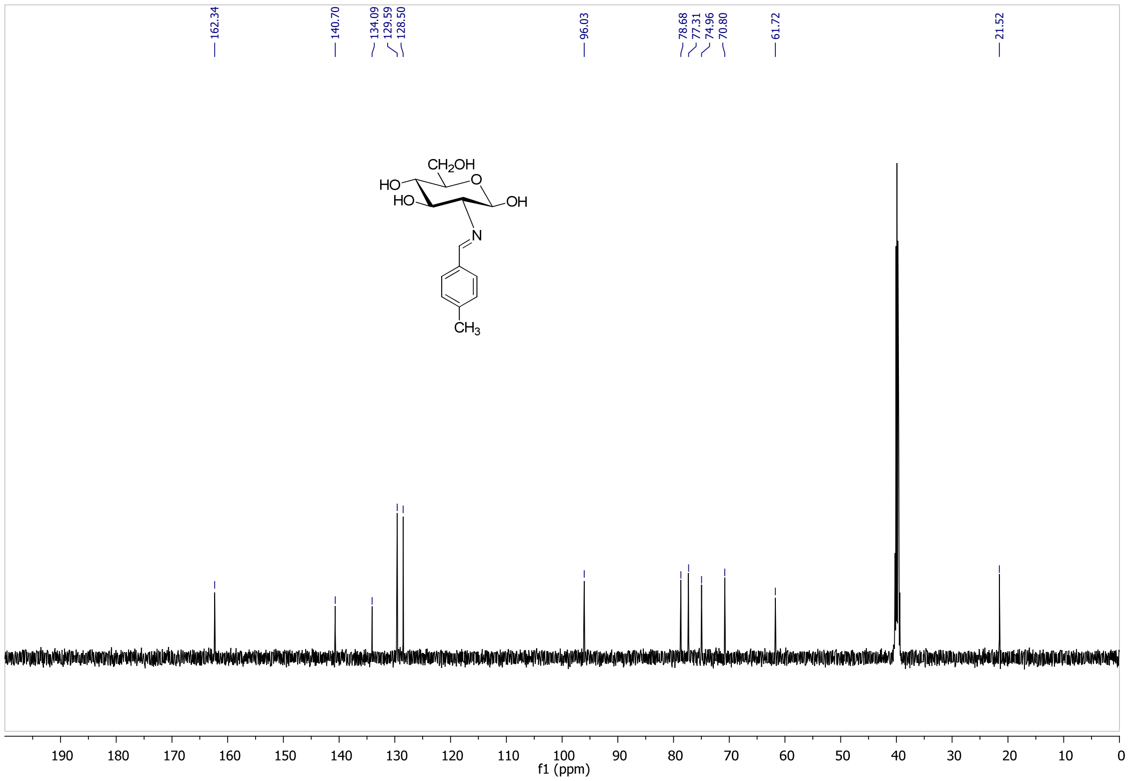

Figure S91. $^{13}C\{^{1}H\}$ *NMR spectrum of* **21** (125 *MHz, DMSO-d$_6$)*

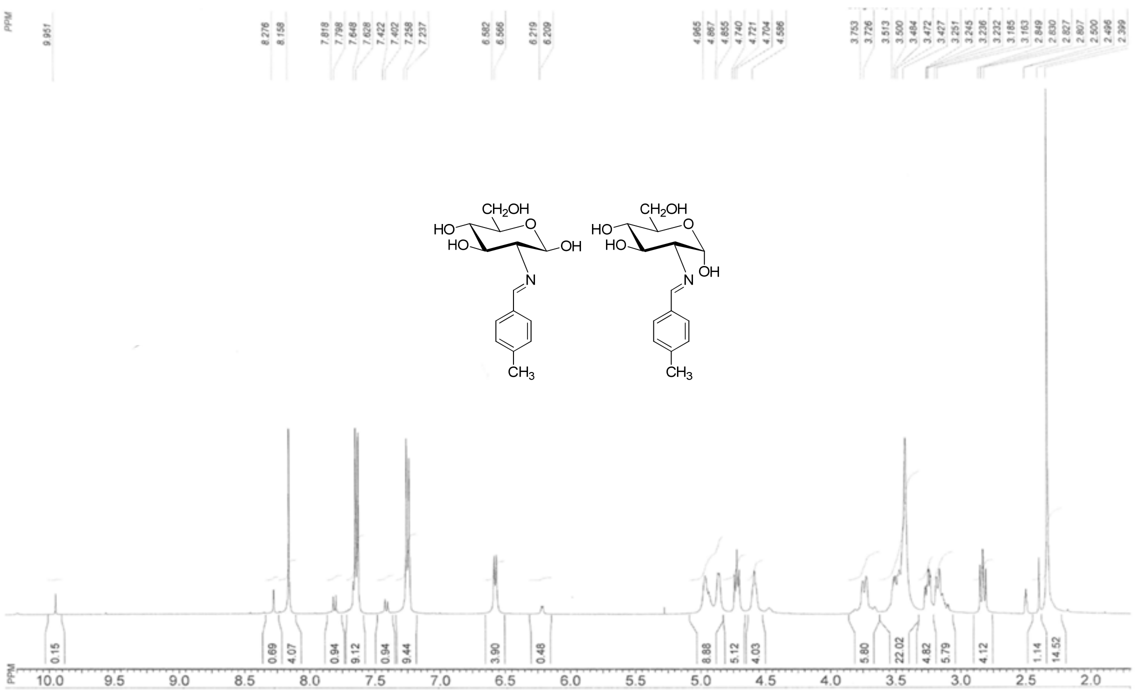

Figure S92. ^{1}H *NMR spectrum of* **21** *and* **64** *(400 MHz, DMSO-d$_6$)*

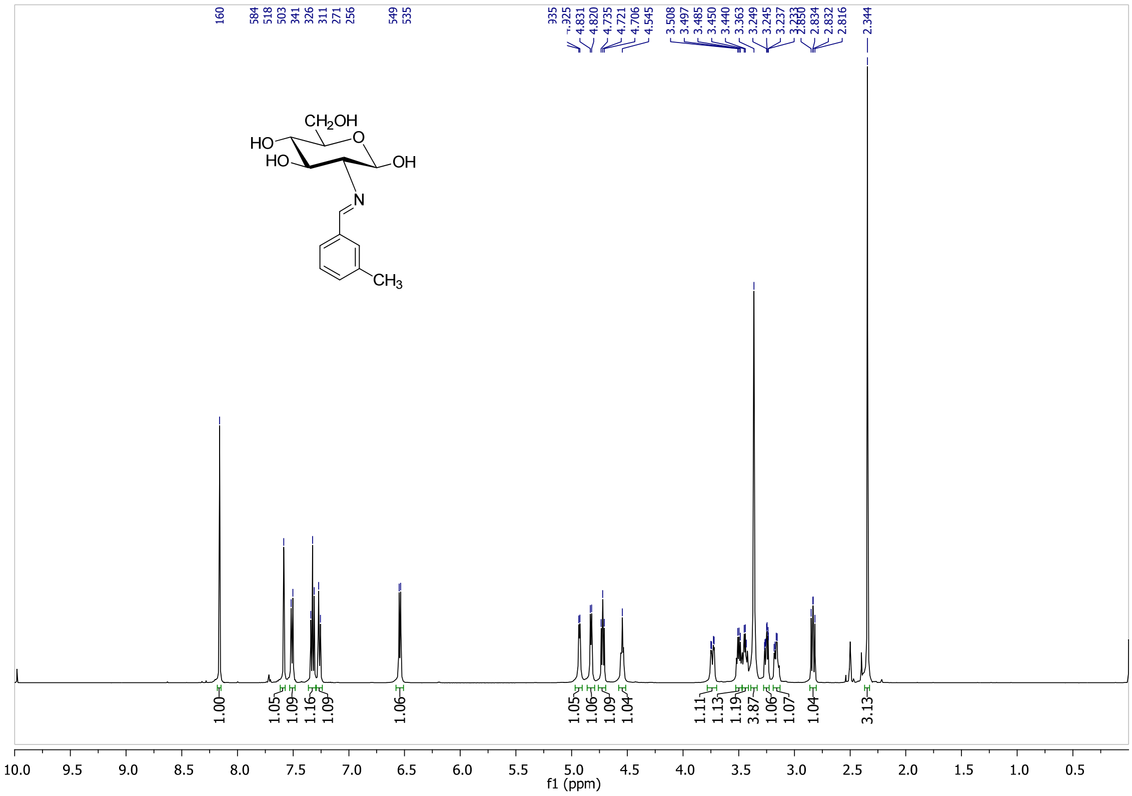

Figure S93. ^{1}H NMR spectrum of **22** (500 MHz, DMSO-d_6)

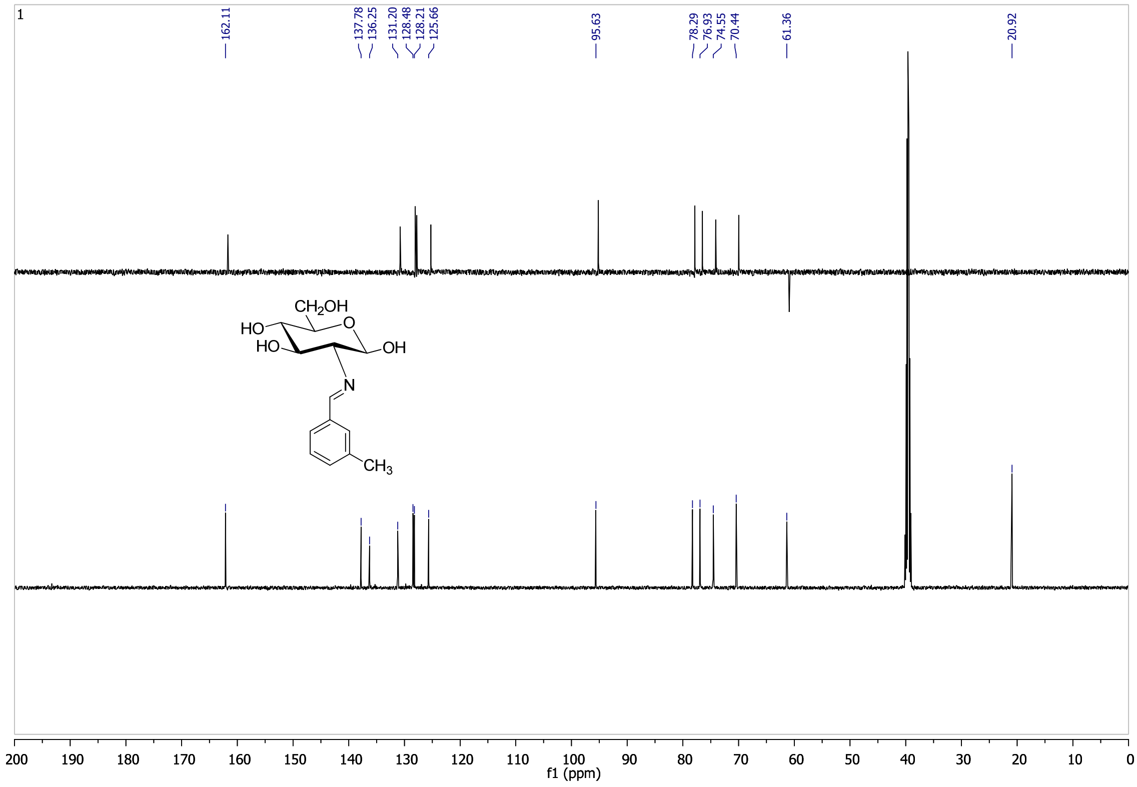

Figure S94. $^{13}C\{^{1}H\}$ NMR (top:DEPT) spectra of **22** (125 MHz, DMSO-d_6)

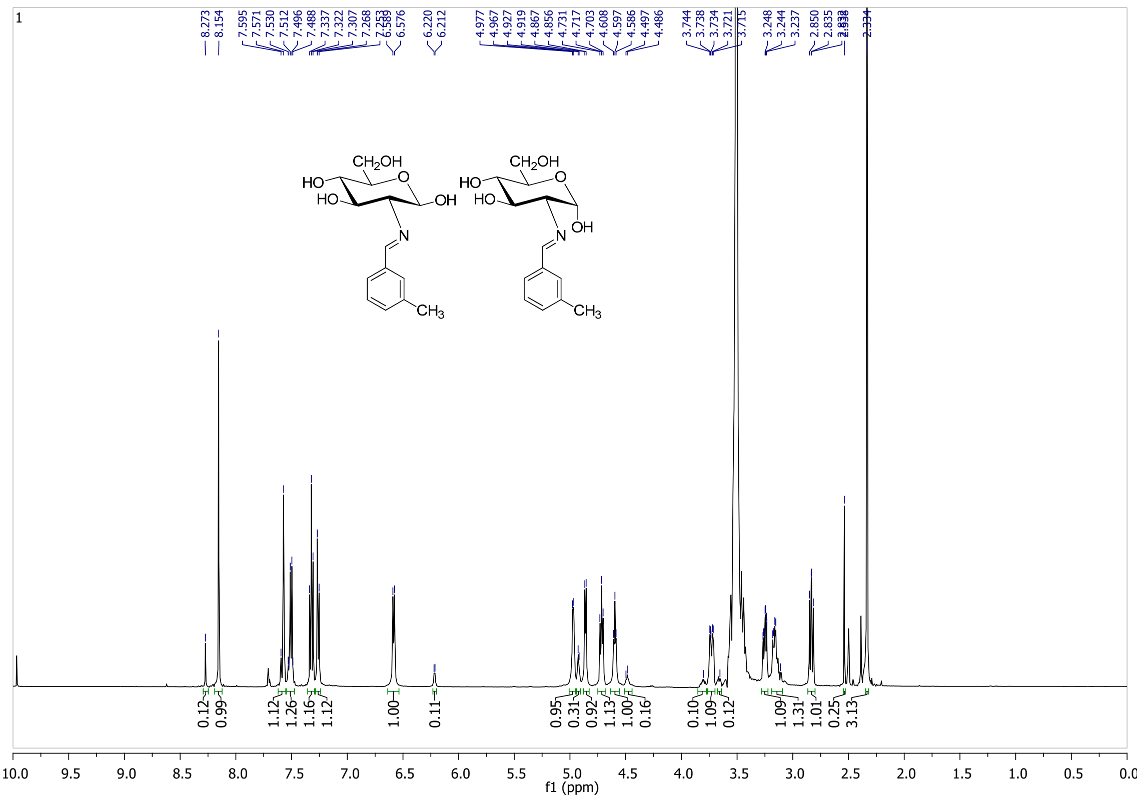

Figure S95. ^{1}H *NMR spectrum of **22** and **65** (500 MHz, DMSO-d_6)*

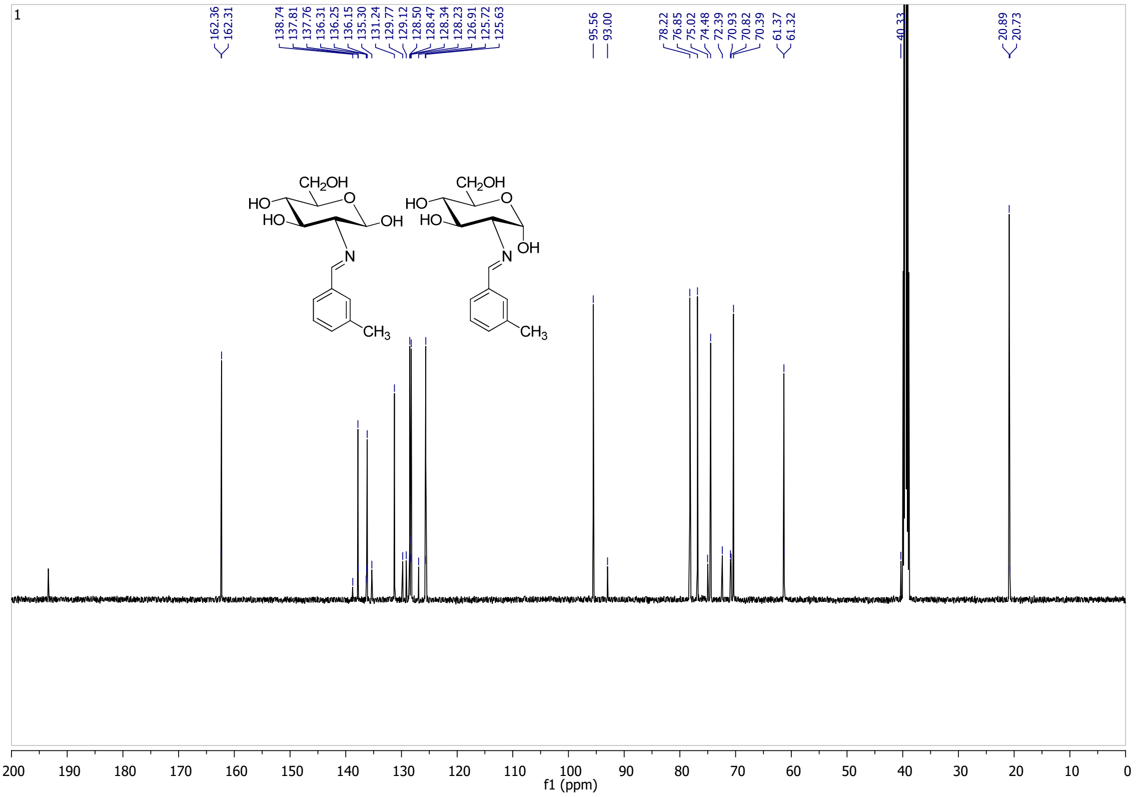

Figure S96. $^{13}C\{^{1}H\}$ *NMR spectrum of **22** and **65** (125 MHz, DMSO-d_6)*

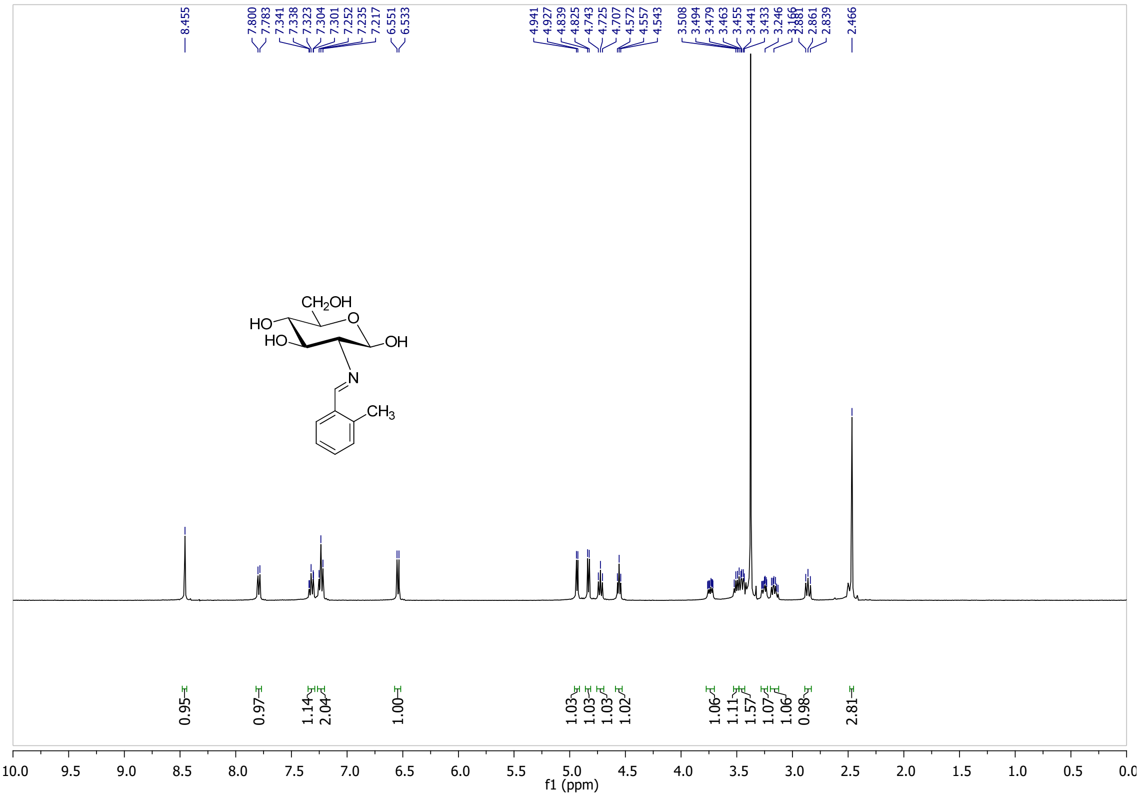

Figure S97. [1]*H NMR spectrum of **23** (500 MHz, DMSO-d$_6$)*

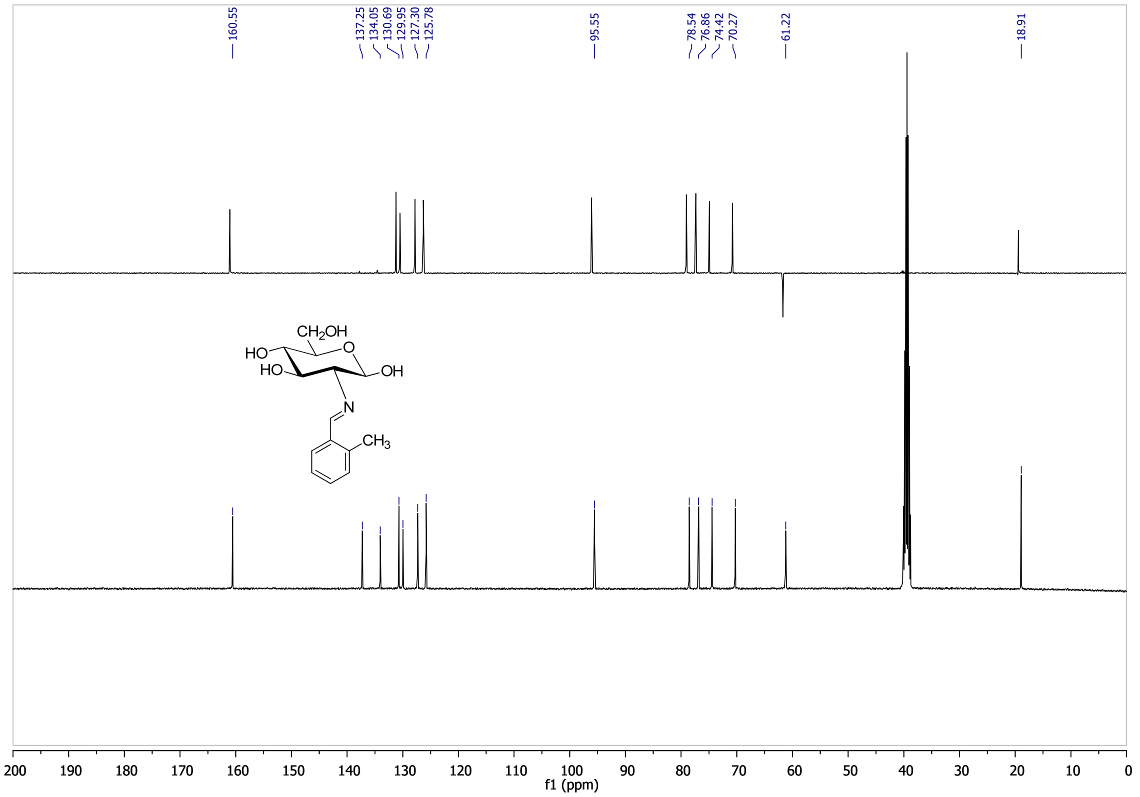

Figure S98. *$^{13}C\{^1H\}$ NMR (top: DEPT) spectra of **23** (125 MHz, DMSO-d$_6$)*

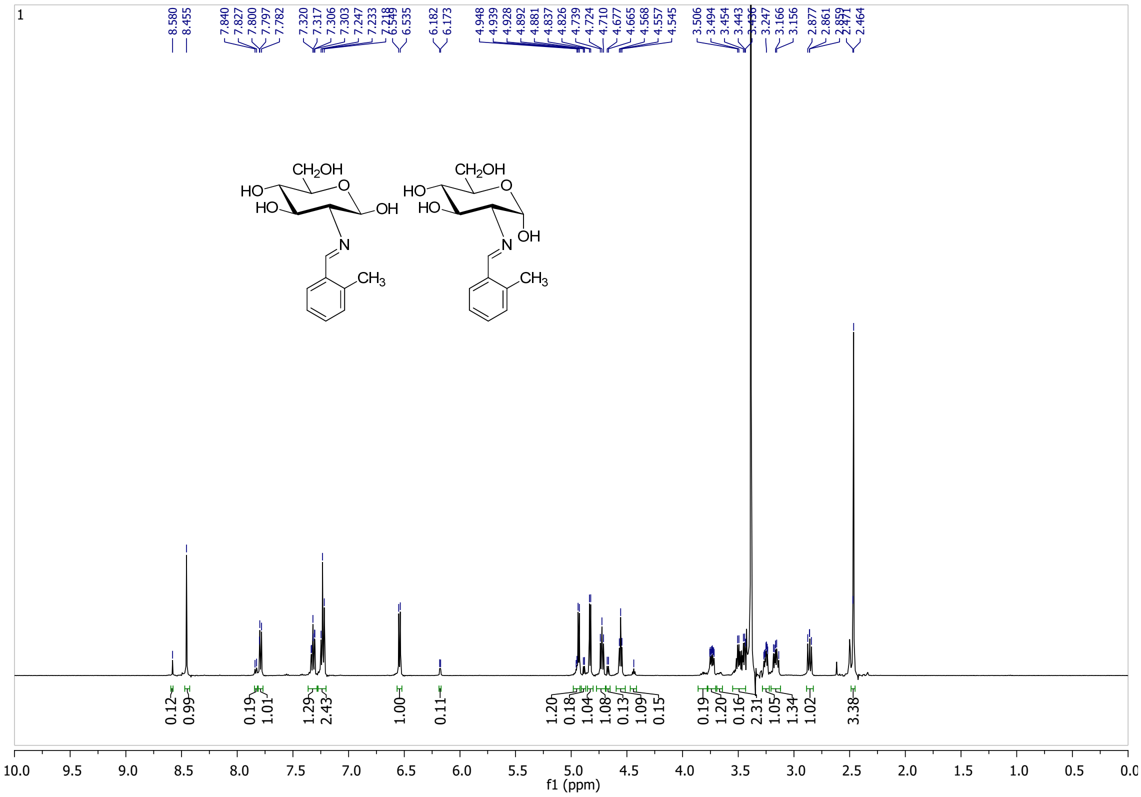

Figure S99. ^{1}H NMR spectrum of **23** and **66** (500 MHz, DMSO-d_6)

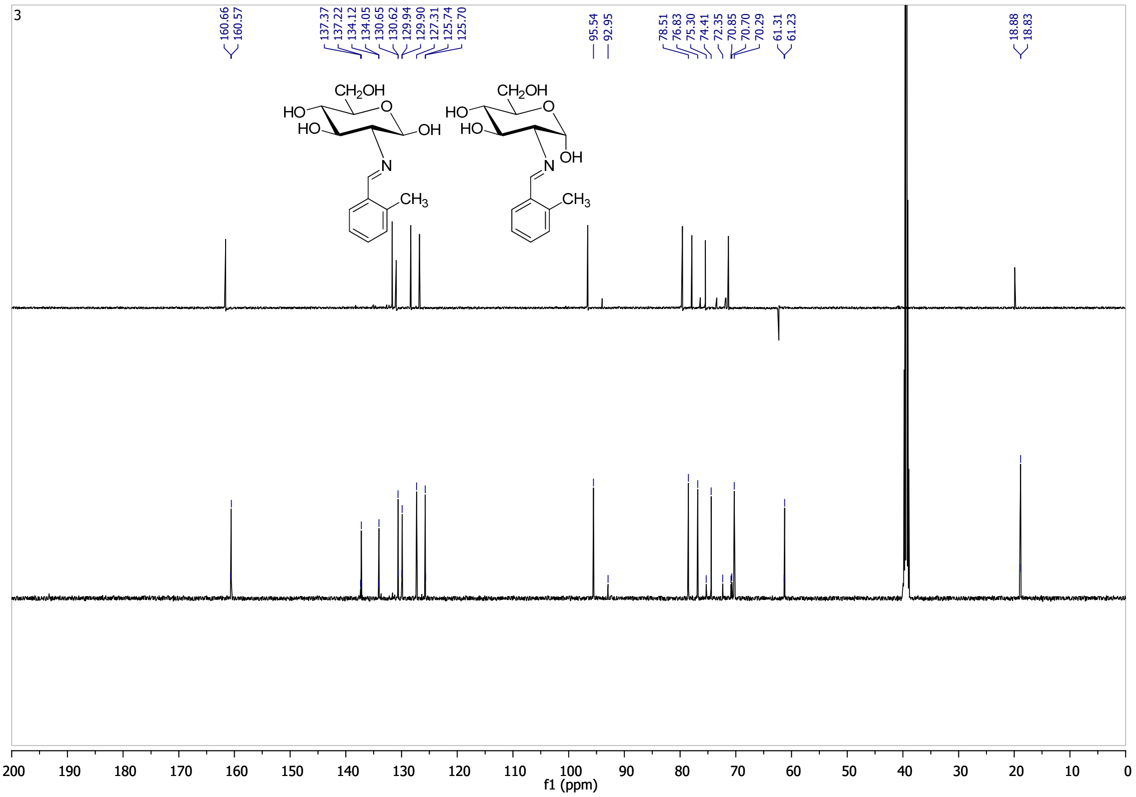

Figure S100. $^{13}C\{^{1}H\}$ NMR (top: DEPT) spectra of **23** and **66** (125MHz, DMSO-d_6)

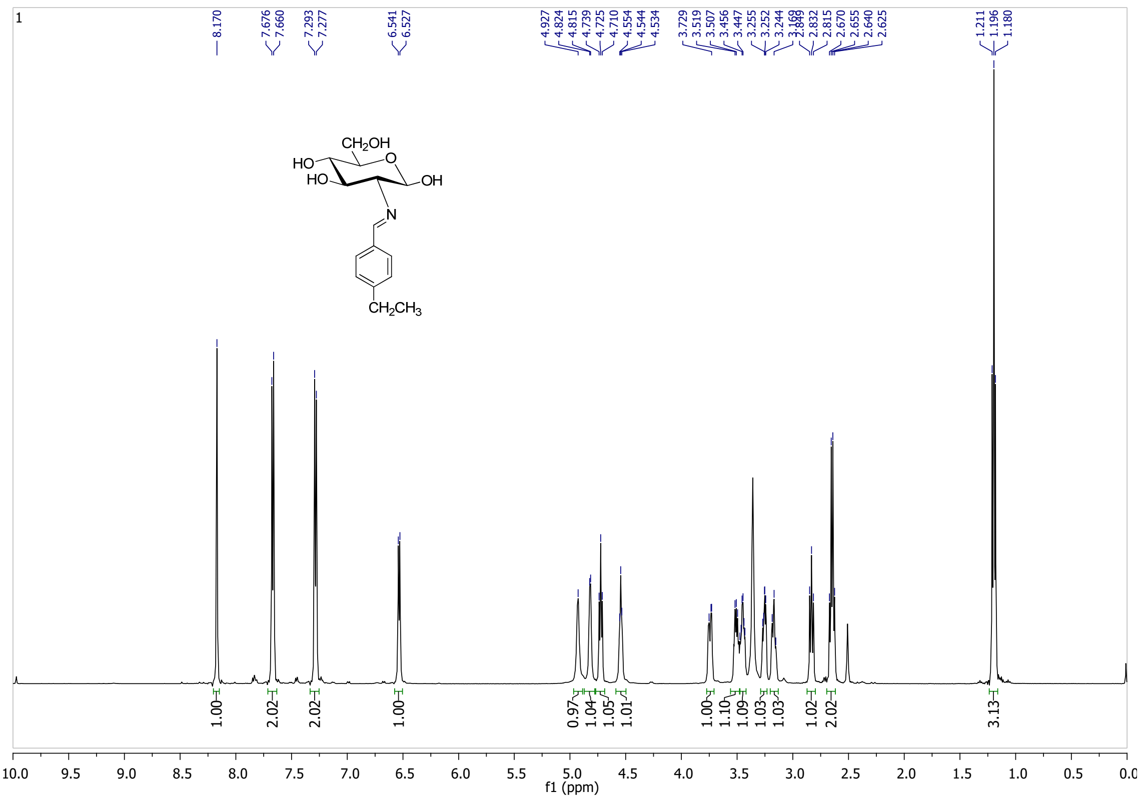

Figure S101. *^1H NMR spectrum of **24** (500 MHz, DMSO-d_6)*

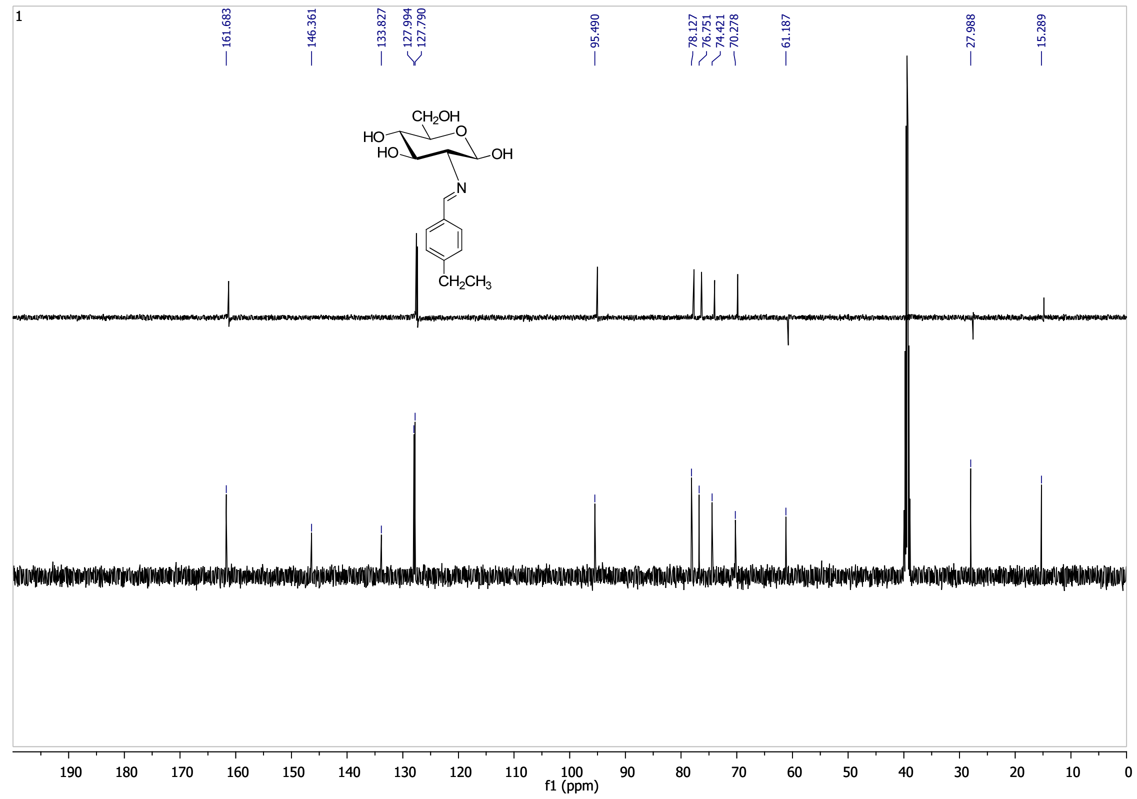

Figure S102. *$^{13}C\{^1H\}$ NMR (top: DEPT) spectra of **24** (125 MHz, DMSO-d_6)*

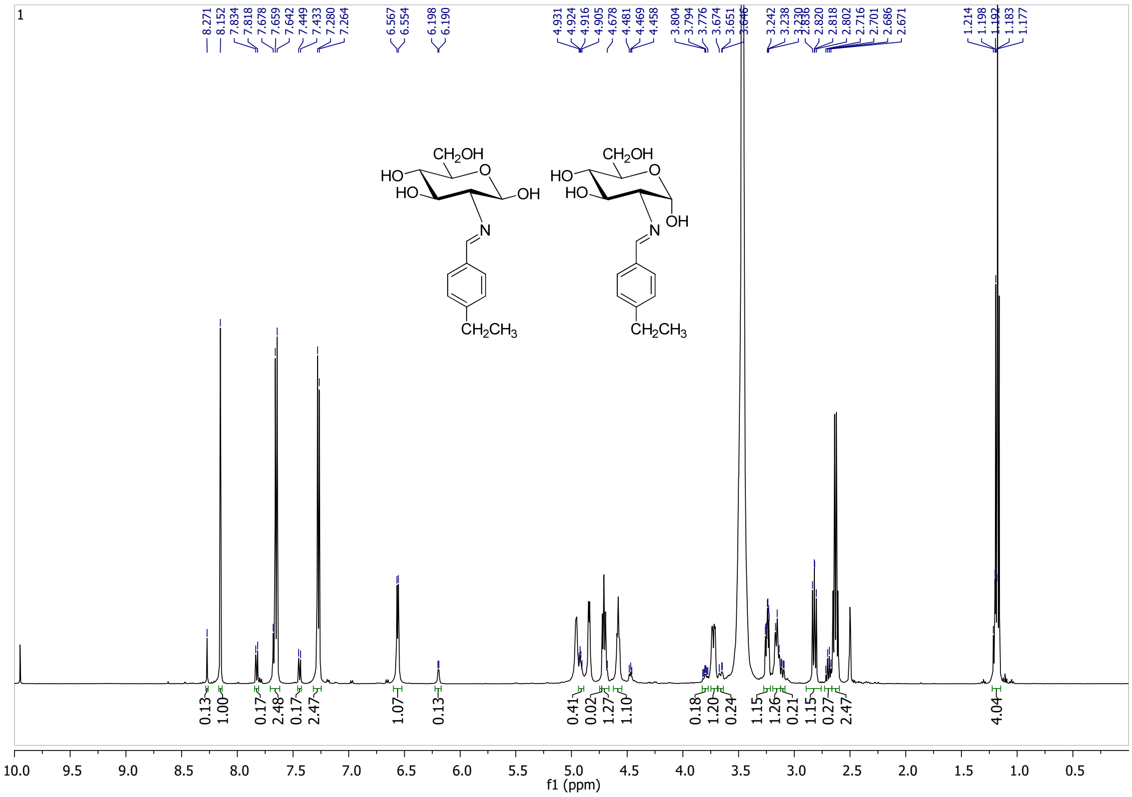

Figure S103. *^{1}H NMR spectrum of **24** and **67** (500 MHz, DMSO-d_6)*

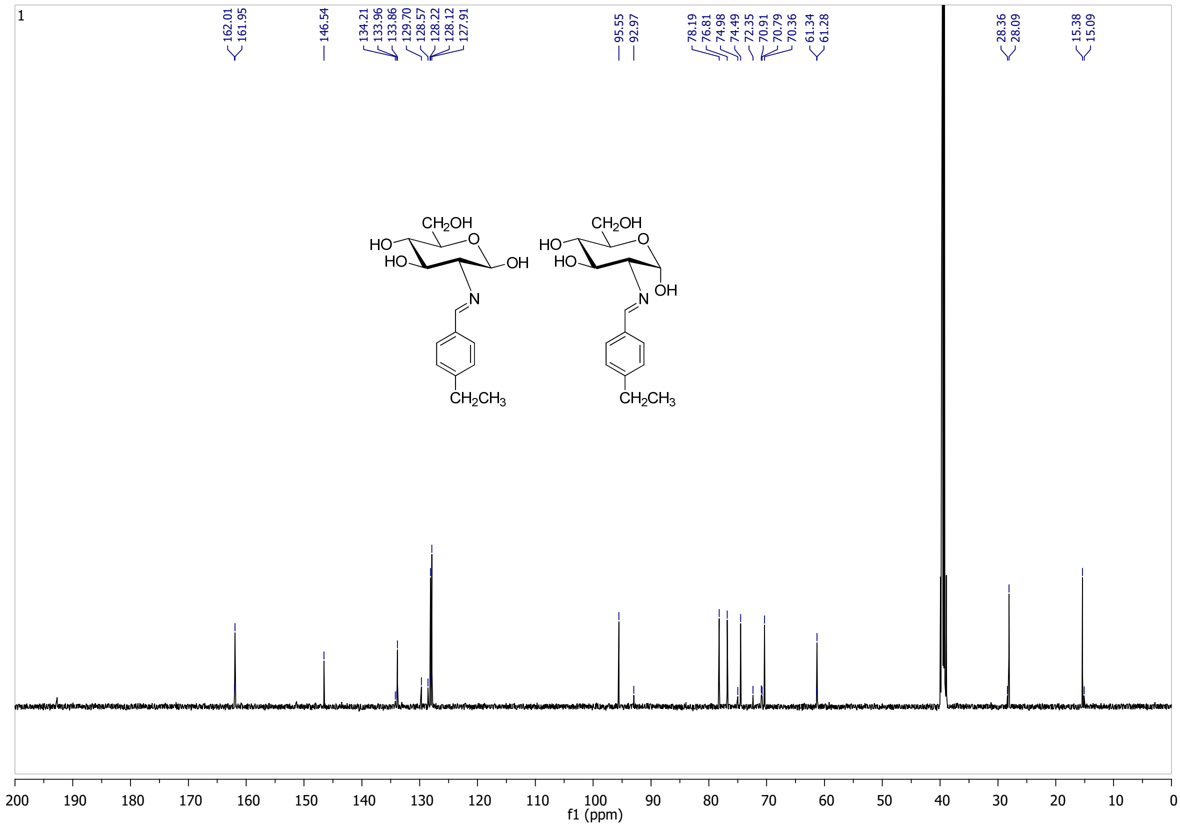

Figure S104. *$^{13}C\{^{1}H\}$ NMR spectrum of **24** and **67** (125 MHz, DMSO-d_6)*

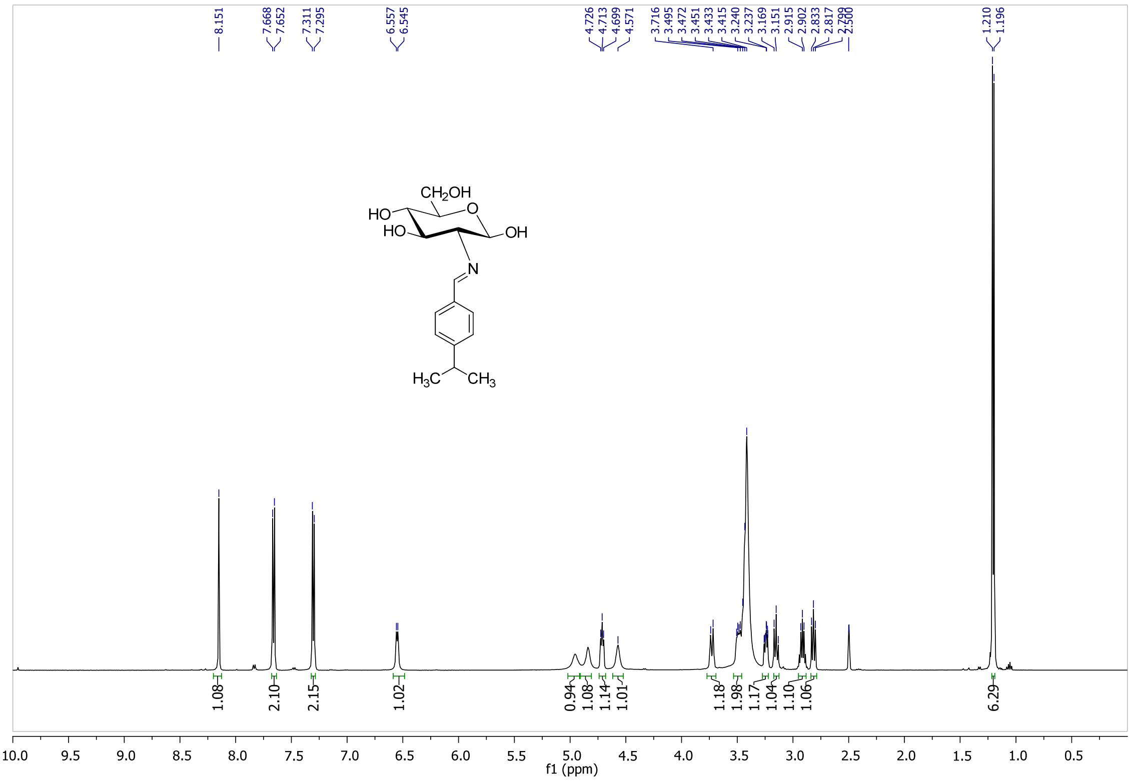

Figure S105. ^{1}H NMR spectrum of **25** (500 MHz, DMSO-d_6)

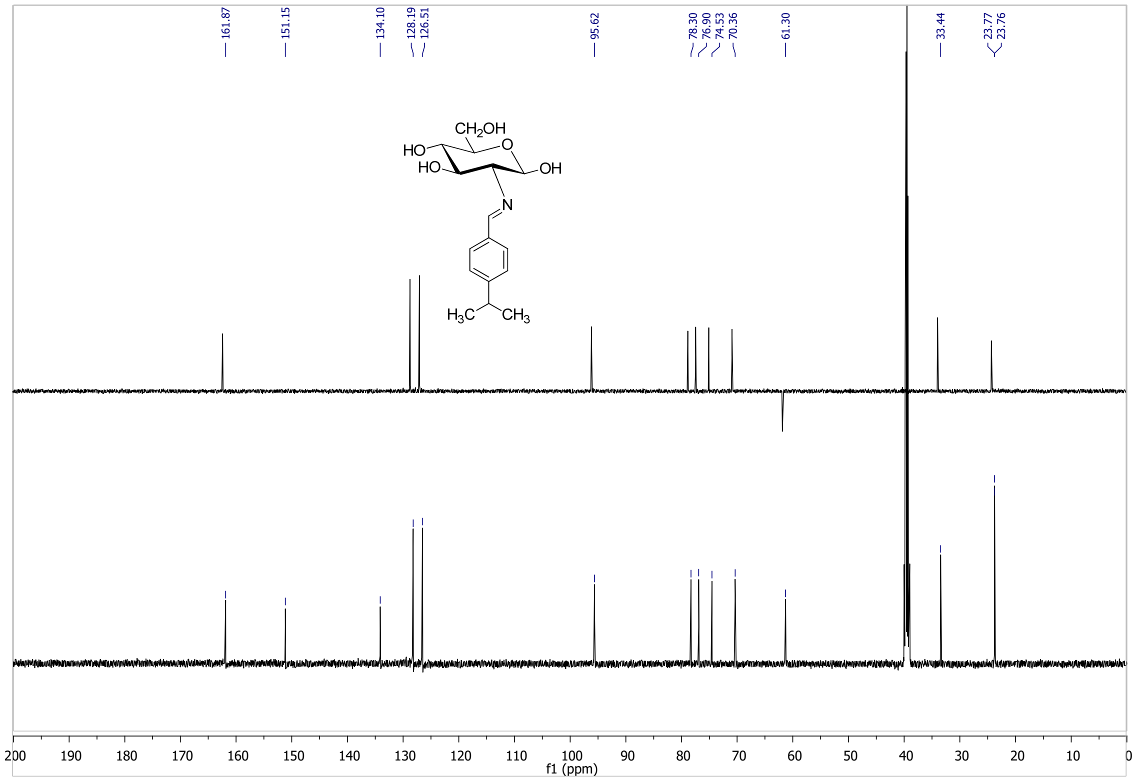

Figure S106. $^{13}C\{^{1}H\}$ NMR (top: DEPT) spectra of **25** (125 MHz, DMSO-d_6)

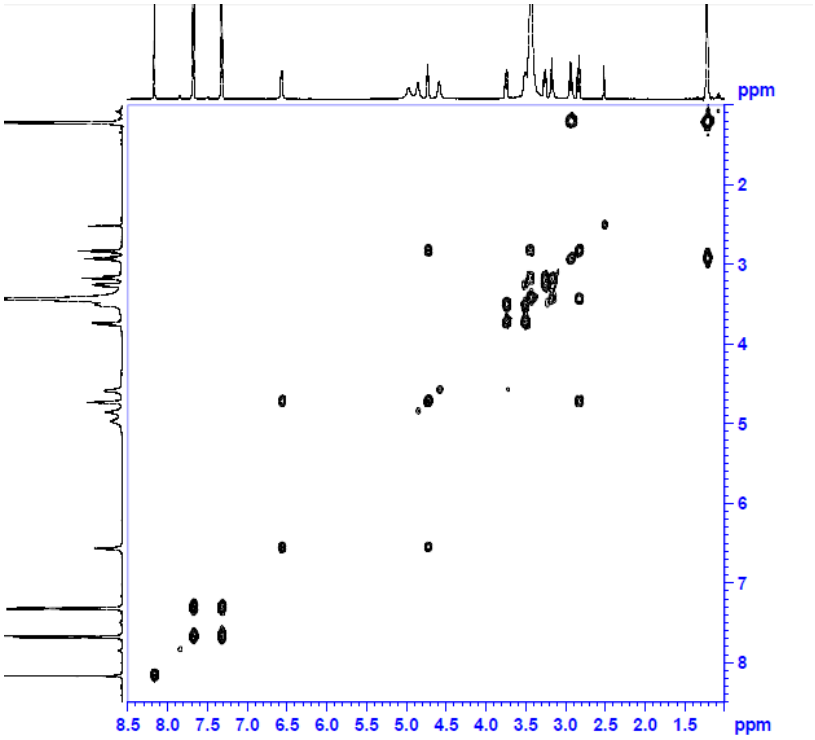

Figure S107. *COSY spectrum of* ***25*** *(DMSO-d6)*

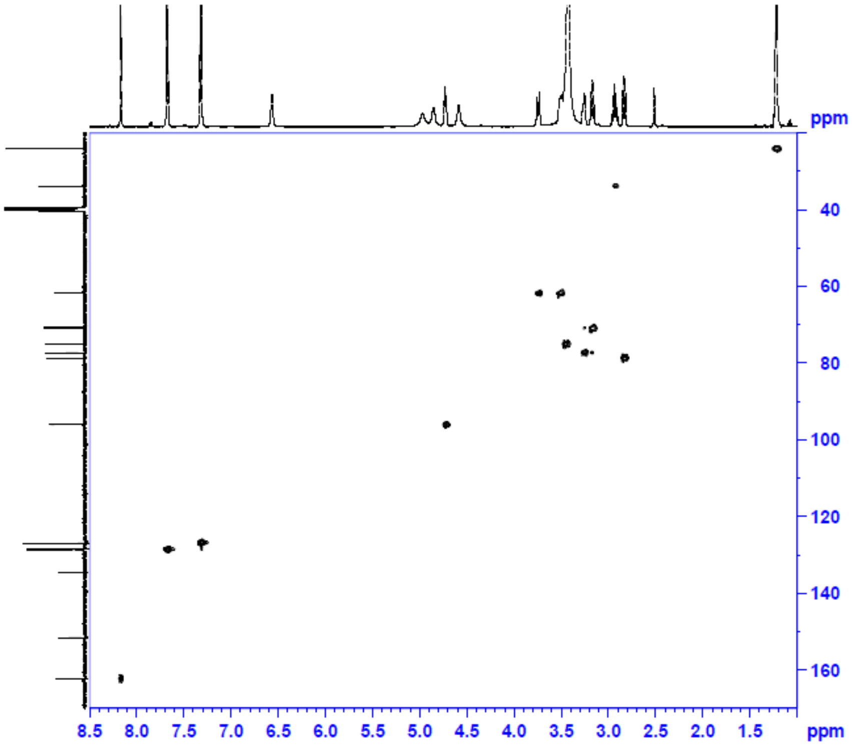

Figure S108. *HMQC spectrum of* ***25*** *(DMSO-d6)*

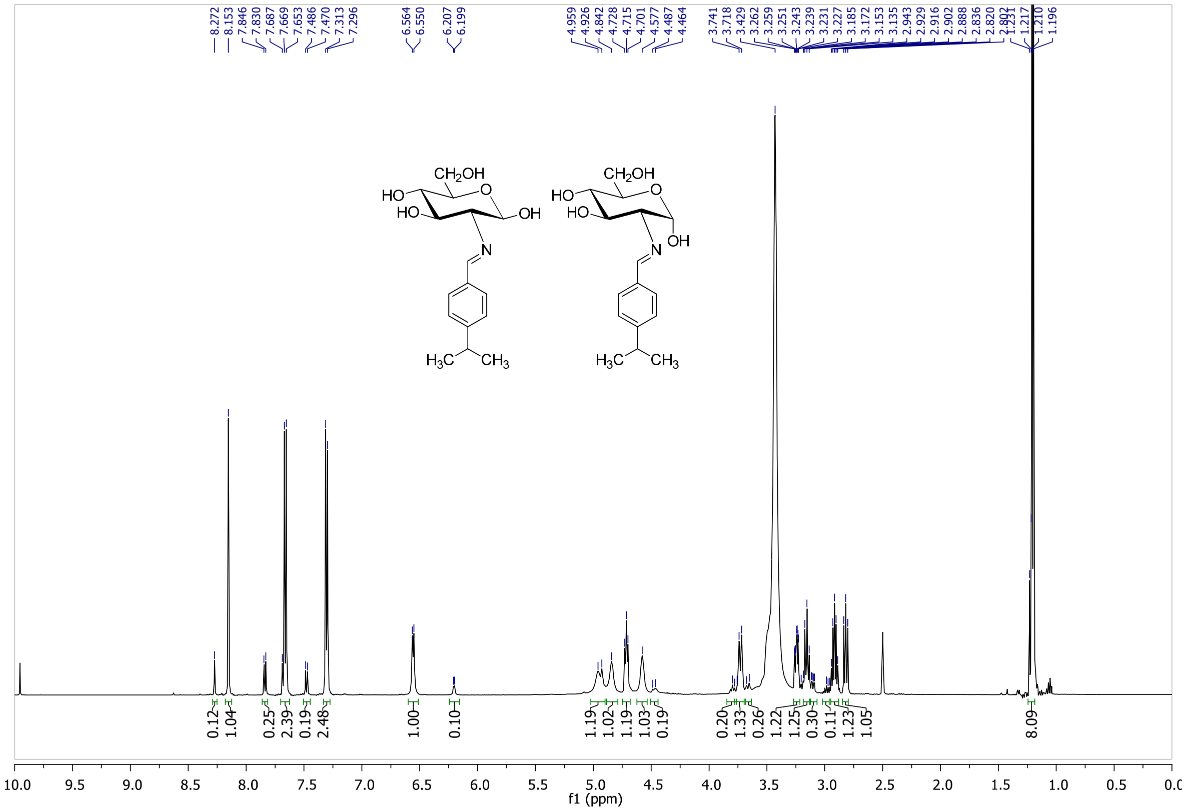

Figure S109. *¹H NMR spectrum of **25** and **68** (500 MHz, DMSO-d₆)*

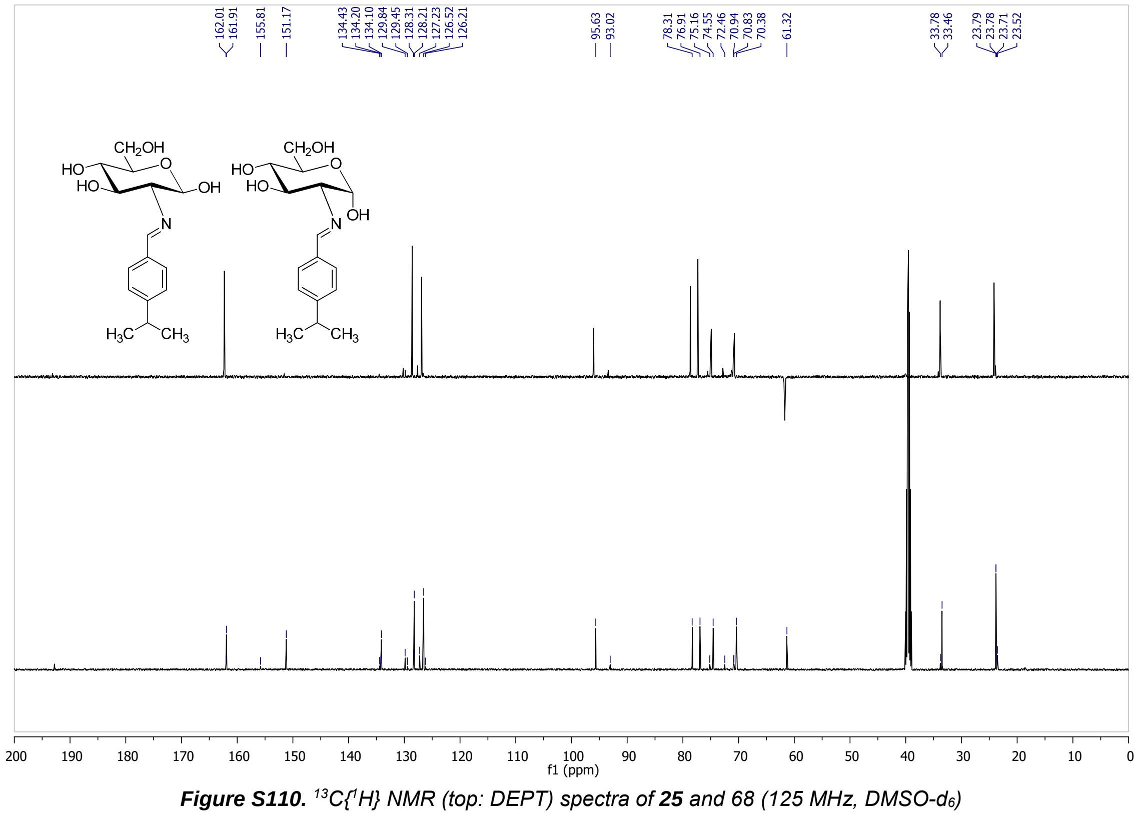

Figure S110. *$^{13}C\{^1H\}$ NMR (top: DEPT) spectra of **25** and 68 (125 MHz, DMSO-d₆)*

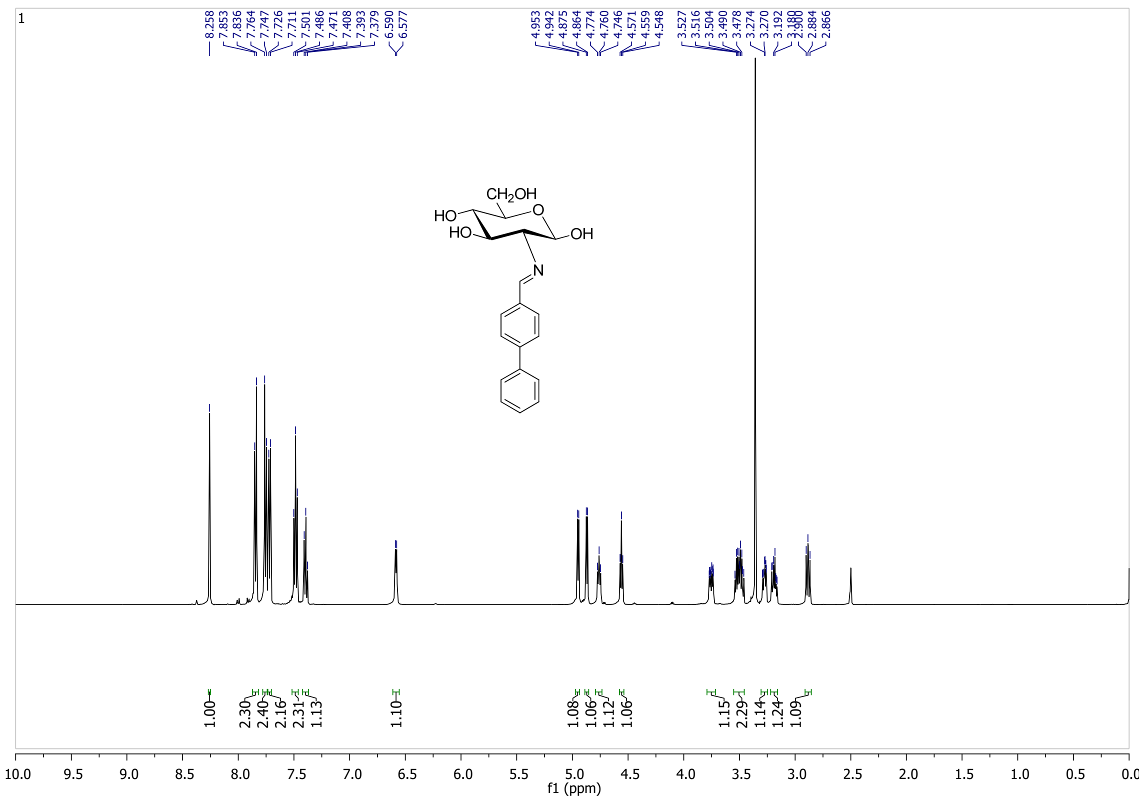

Figure S111. ^{1}H NMR spectrum of **26** (500 MHz, DMSO-d_6)

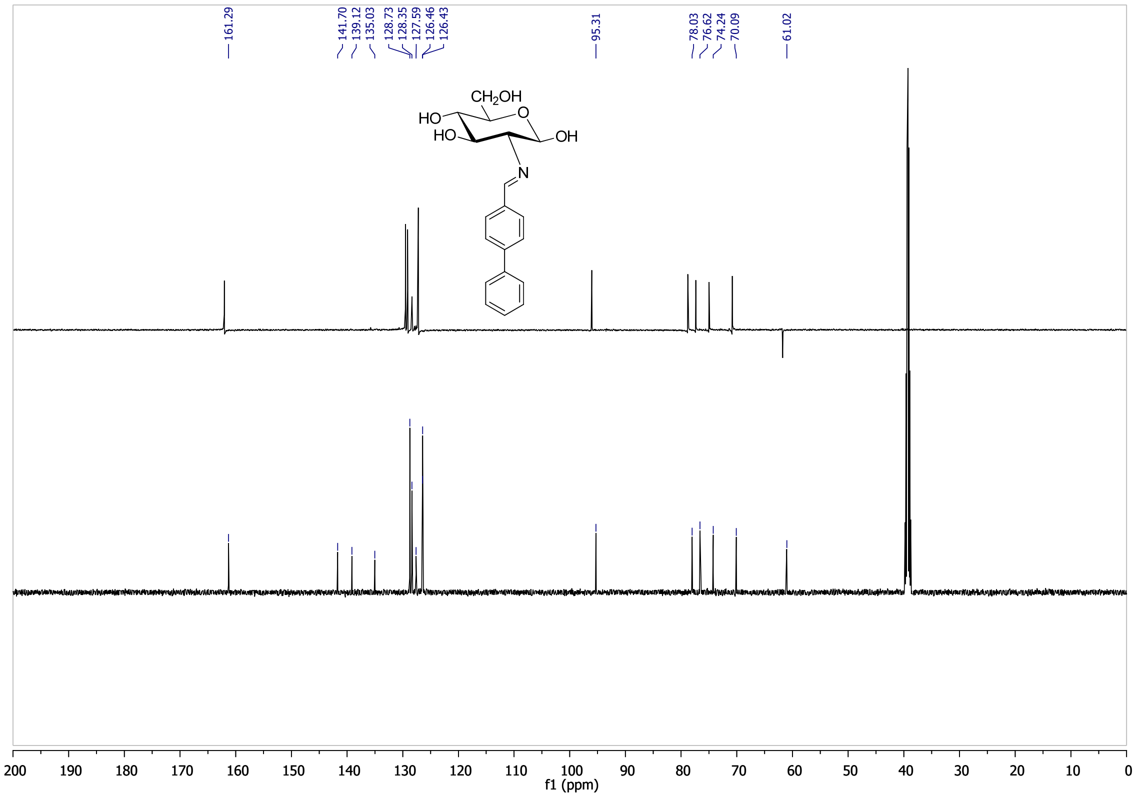

Figure S112. $^{13}C\{^{1}H\}$ NMR (top: DEPT) spectra of **26** (125 MHz, DMSO-d_6)

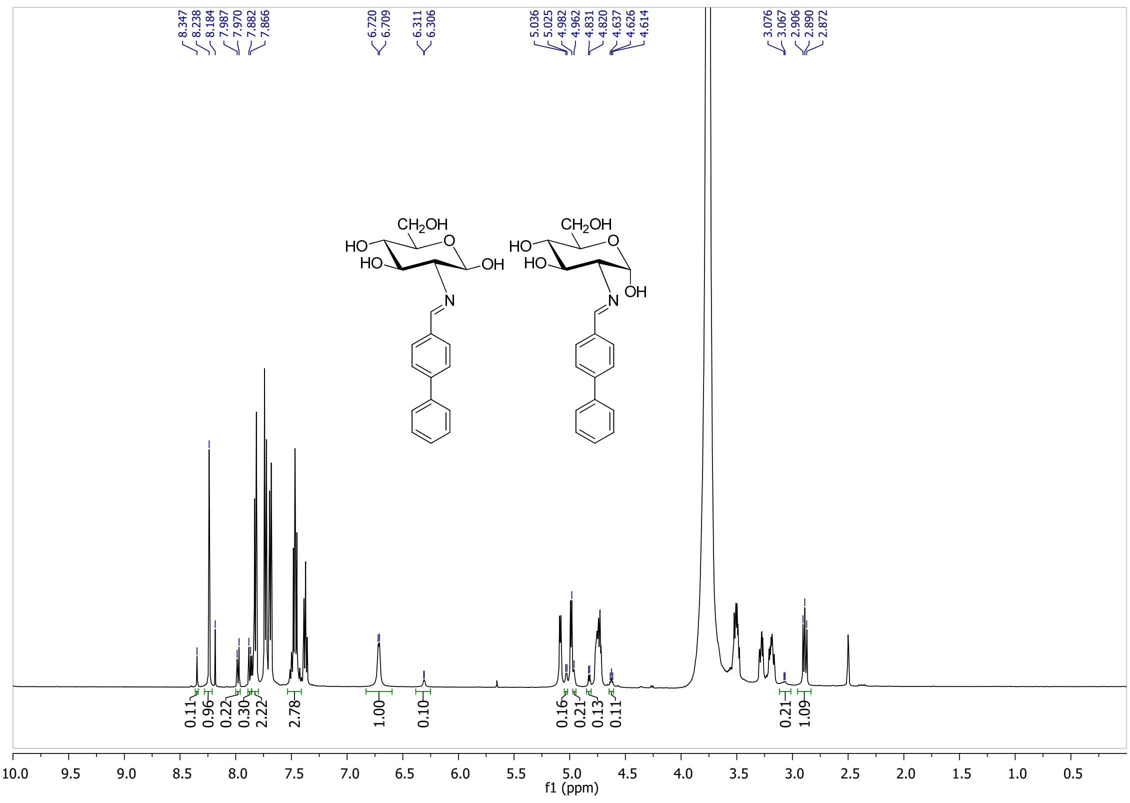

Figure S113. *^{1}H NMR spectrum of **26** and **69** (500 MHz, DMSO-d₆)*

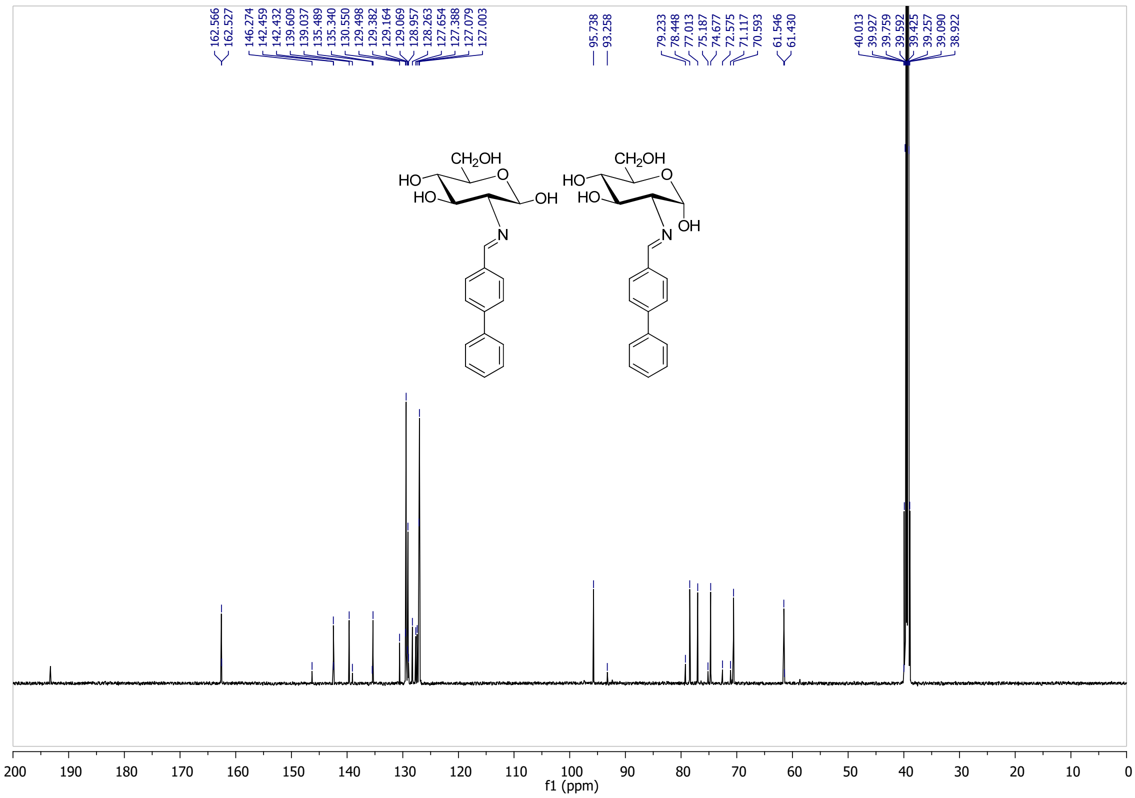

Figure S114. *^{13}C{^{1}H} NMR spectrum of **26** and **69** (125 MHz, DMSO-d₆)*

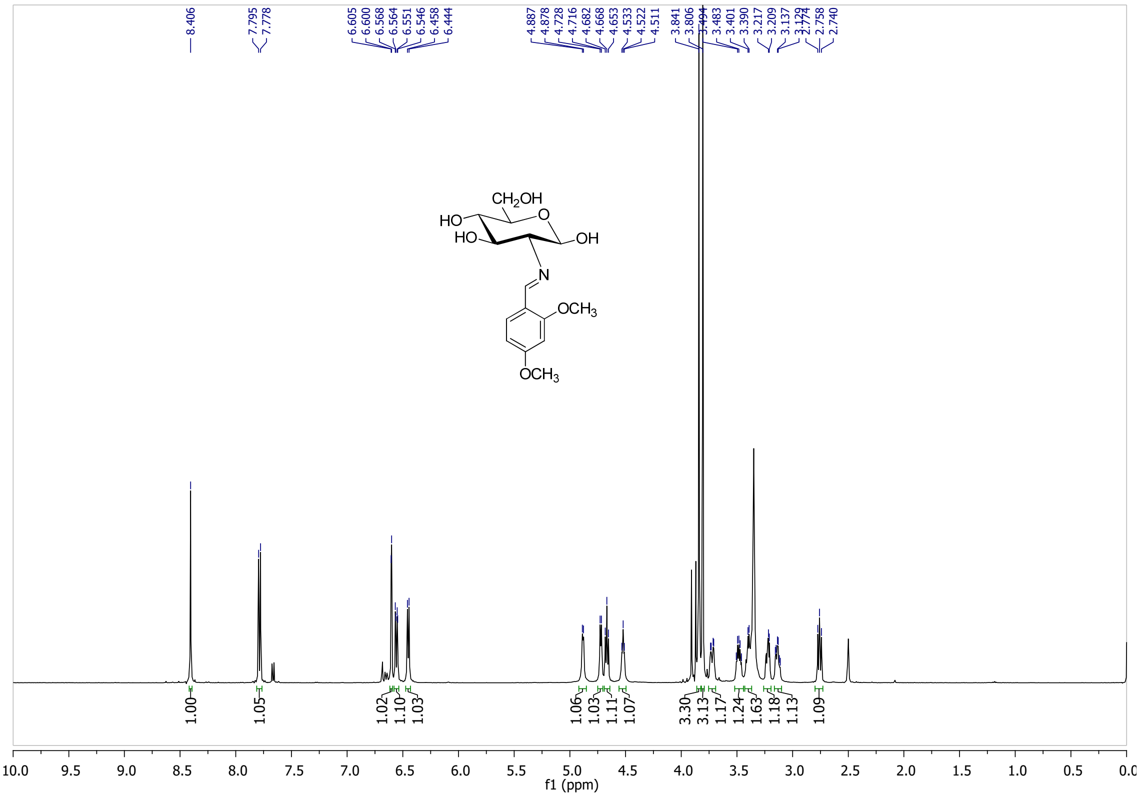

Figure S115. *^{1}H NMR spectrum of **27** (500 MHz, DMSO-d_6)*

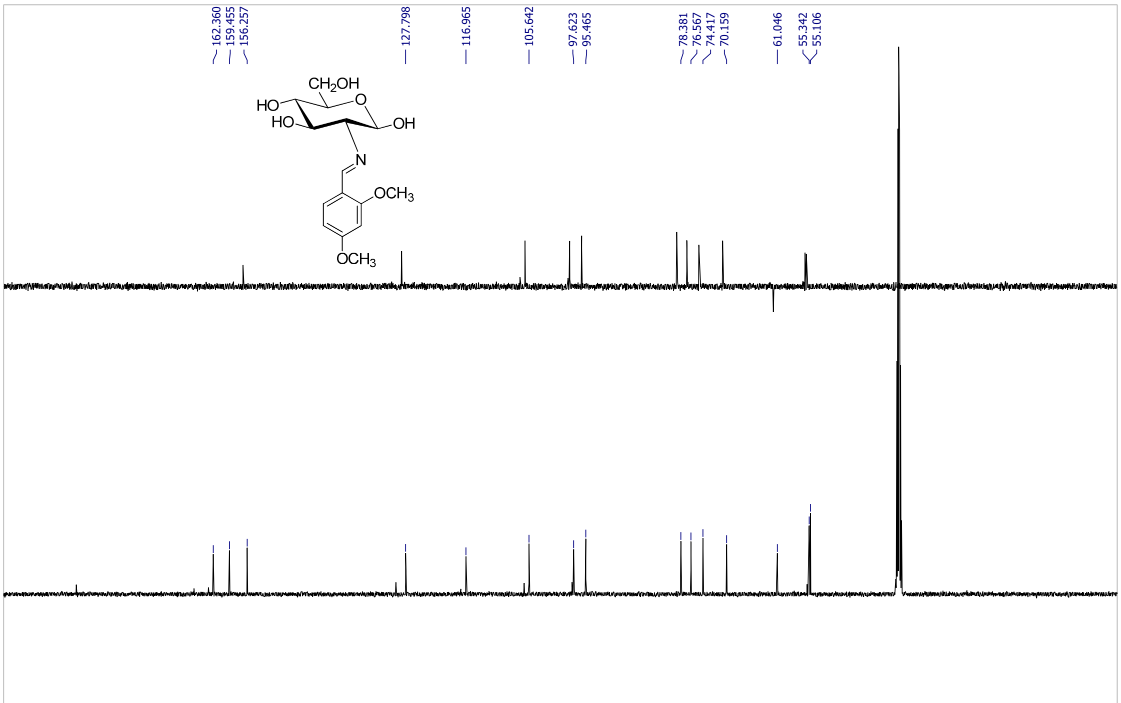

Figure S116. *^{13}C{^{1}H} NMR (top: DEPT) spectra of **27** (125 MHz, DMSO-d_6)*

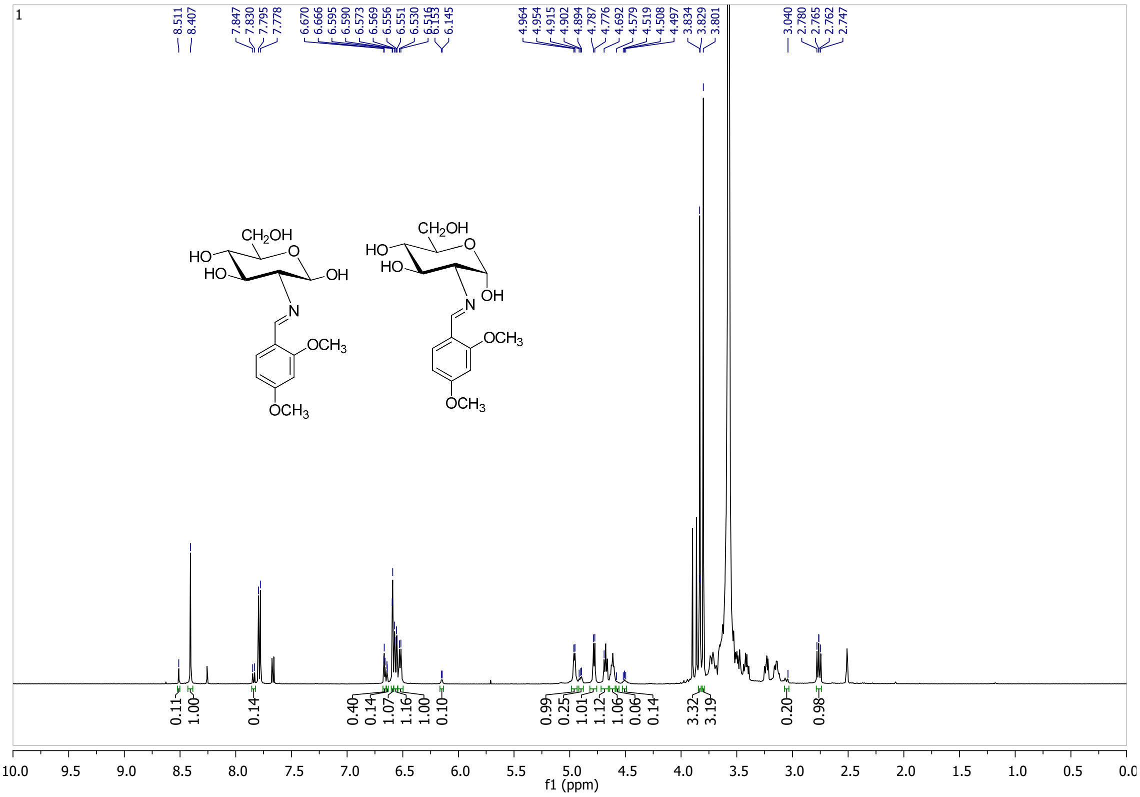

Figure S117. *^{1}H NMR spectrum of **27** and **70** (500 MHz, DMSO-d6)*

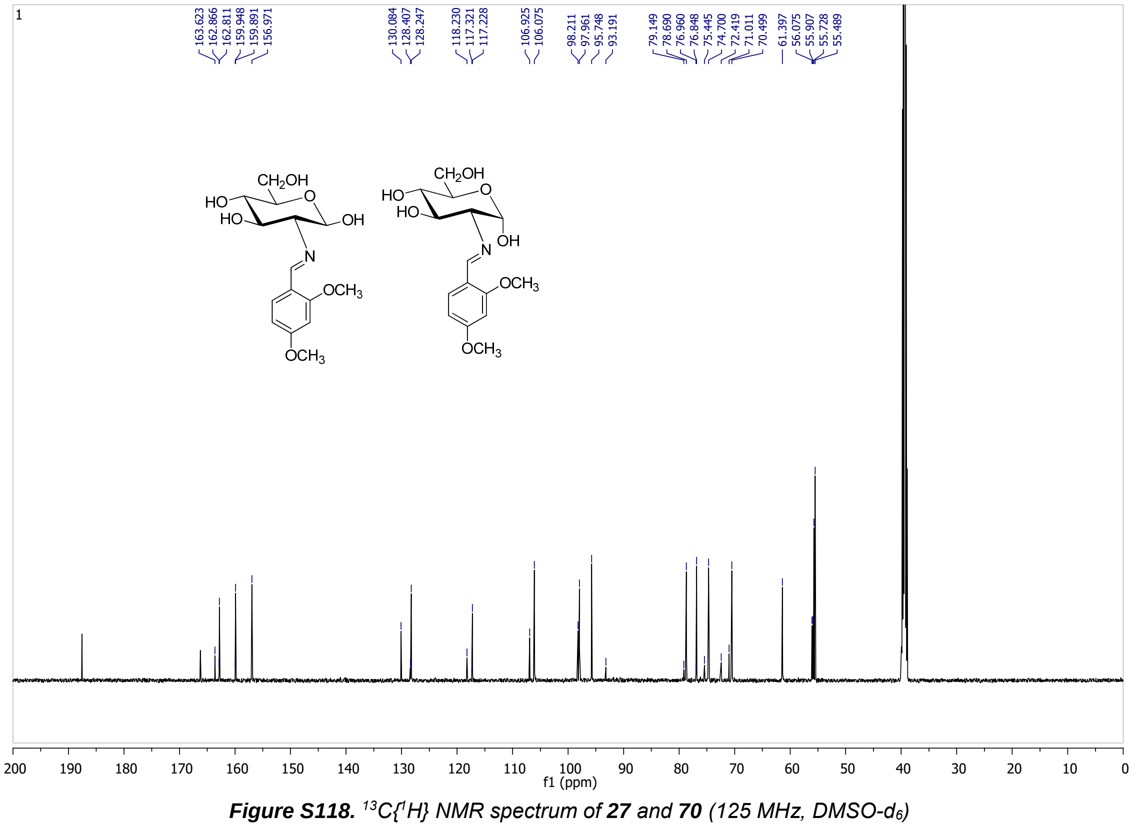

Figure S118. *^{13}C{^{1}H} NMR spectrum of **27** and **70** (125 MHz, DMSO-d6)*

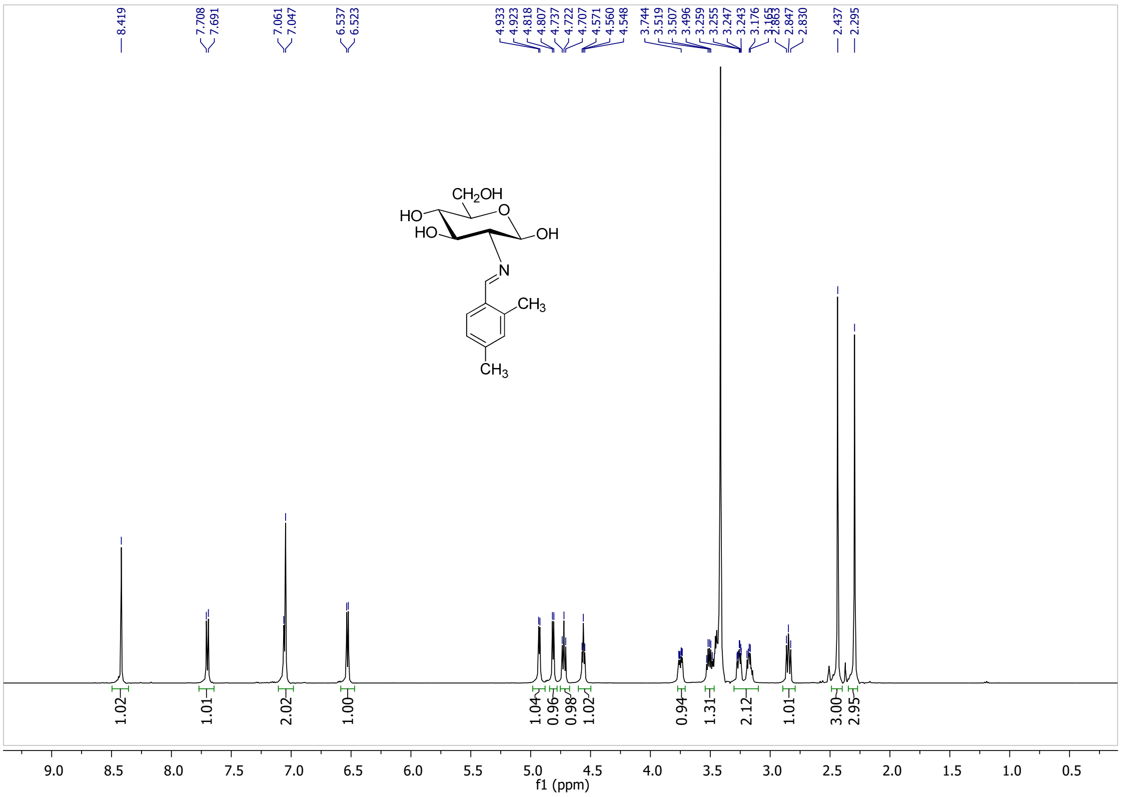

Figure S119. _$^{13}C\{^1H\}$ NMR spectrum of **28** (500 MHz, DMSO-d_6)_

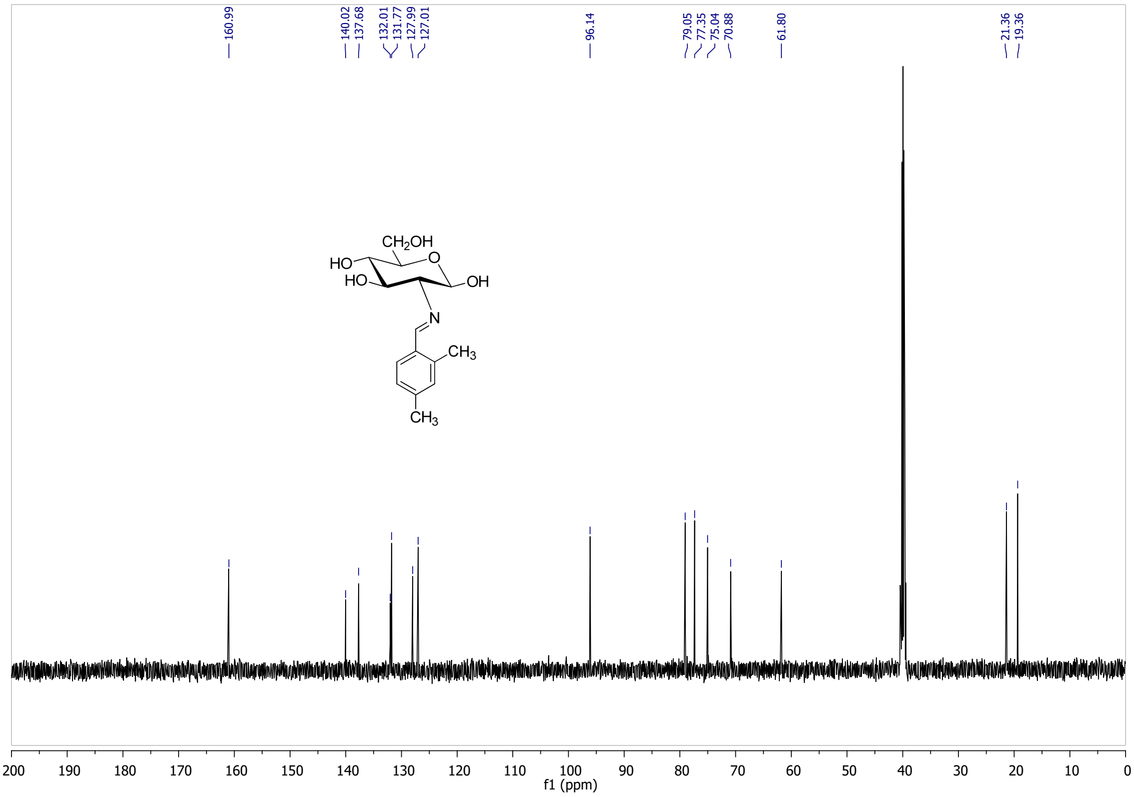

Figure S120. _$^{13}C\{^1H\}$ NMR spectrum of **28** (125 MHz, DMSO-d_6)_

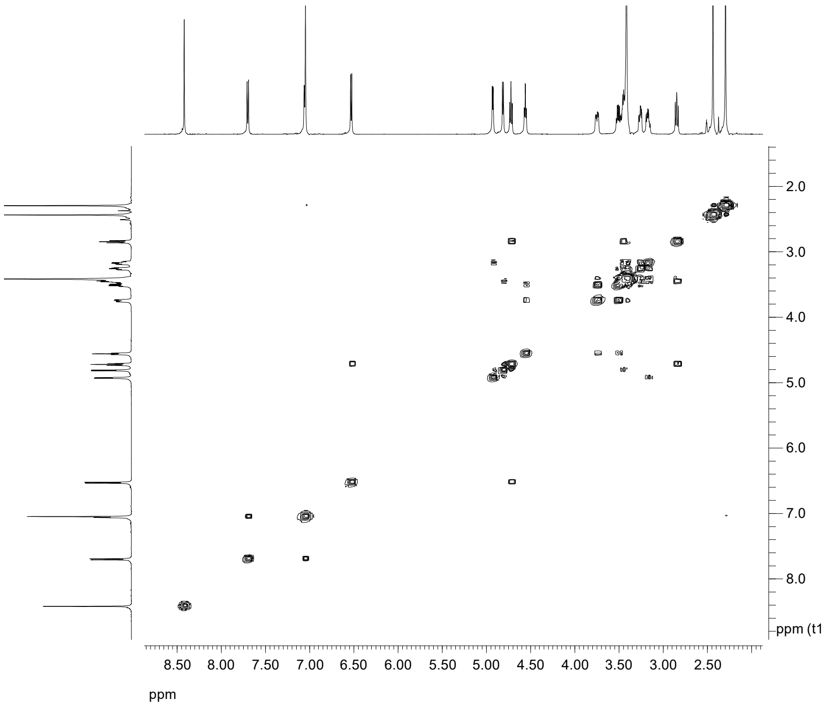

Figure S121. *COSY spectrum of **28** (DMSO-d*$_6$*)*

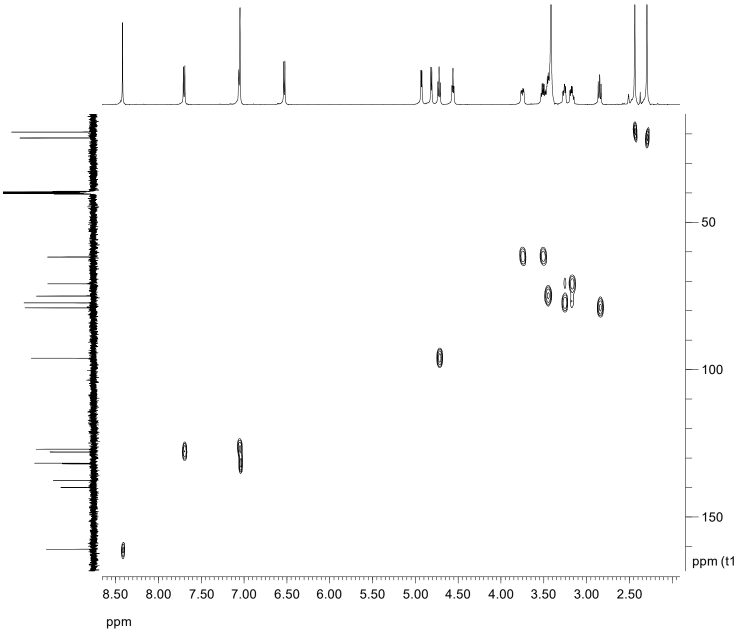

Figure S122. *HMQC spectrum of **28** (DMSO-d*$_6$*)*

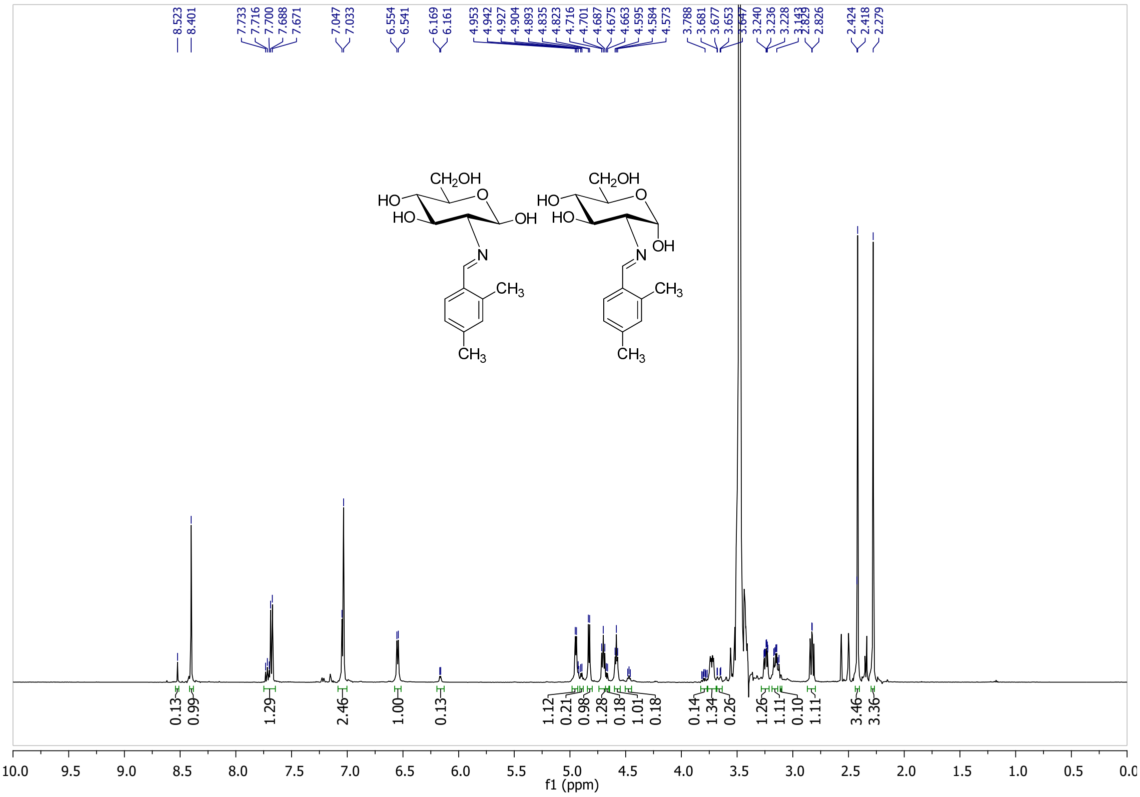

Figure S123. *^{1}H NMR spectrum of* ***28*** *and* ***71*** *(500 MHz, DMSO-d₆)*

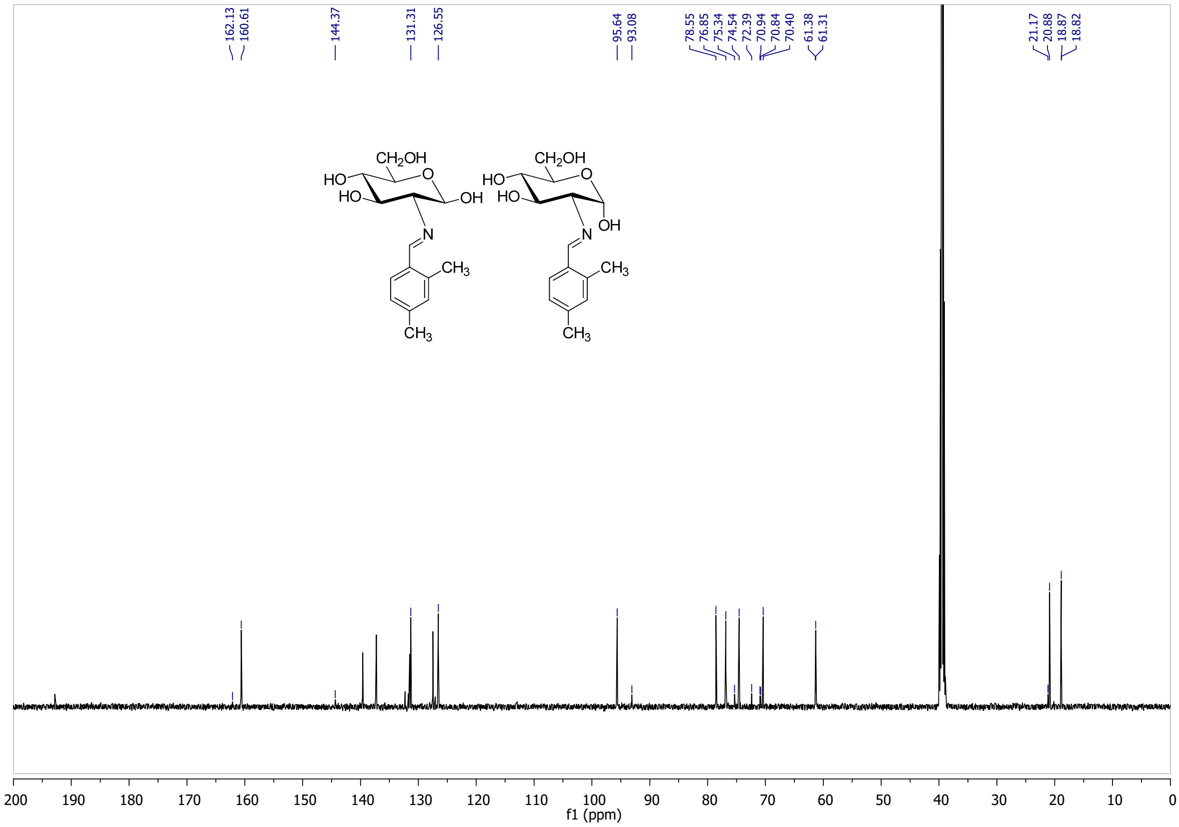

Figure S124. *^{13}C{^{1}H} NMR spectrum of* ***28*** *and* ***71*** *(125 MHz, DMSO-d₆)*

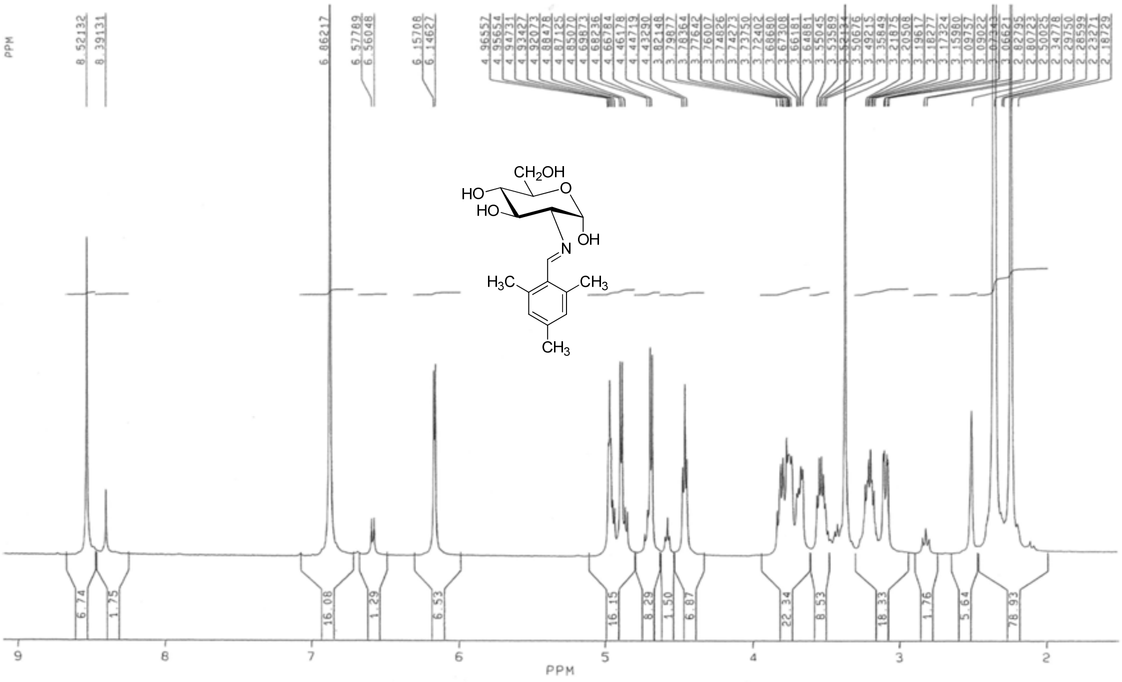

Figure S125. *¹H NMR spectrum of **29** (400 MHz, DMSO-d₆)*

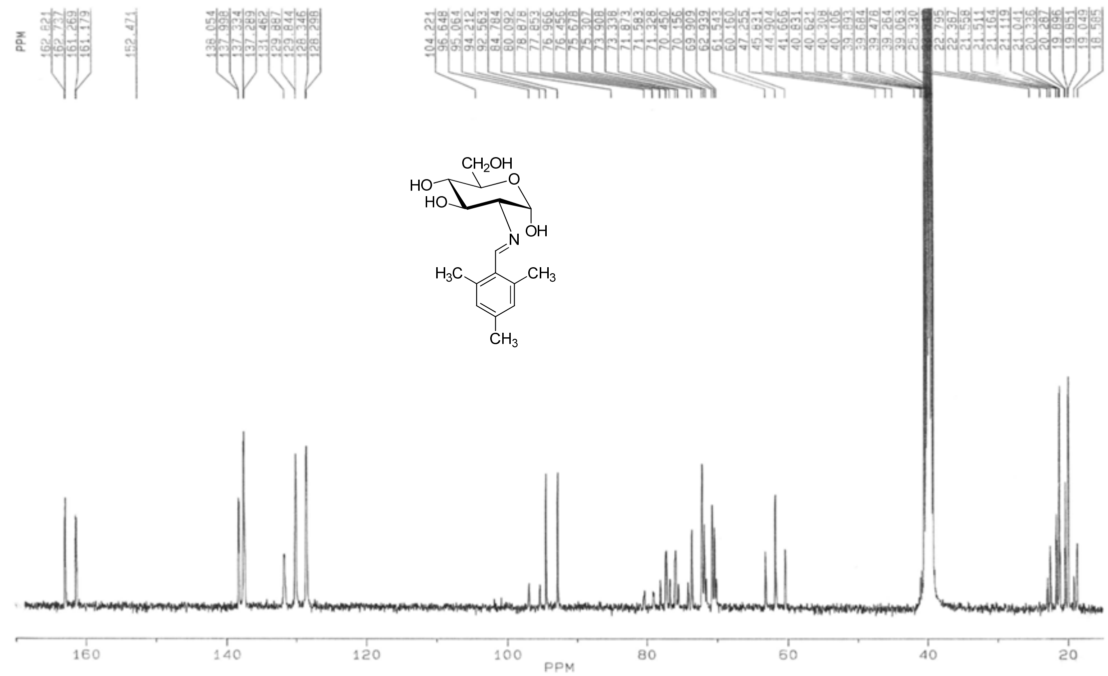

Figure S126. *Proton-coupled ¹³C NMR spectrum of **29** (100 MHz, DMSO-d₆)*

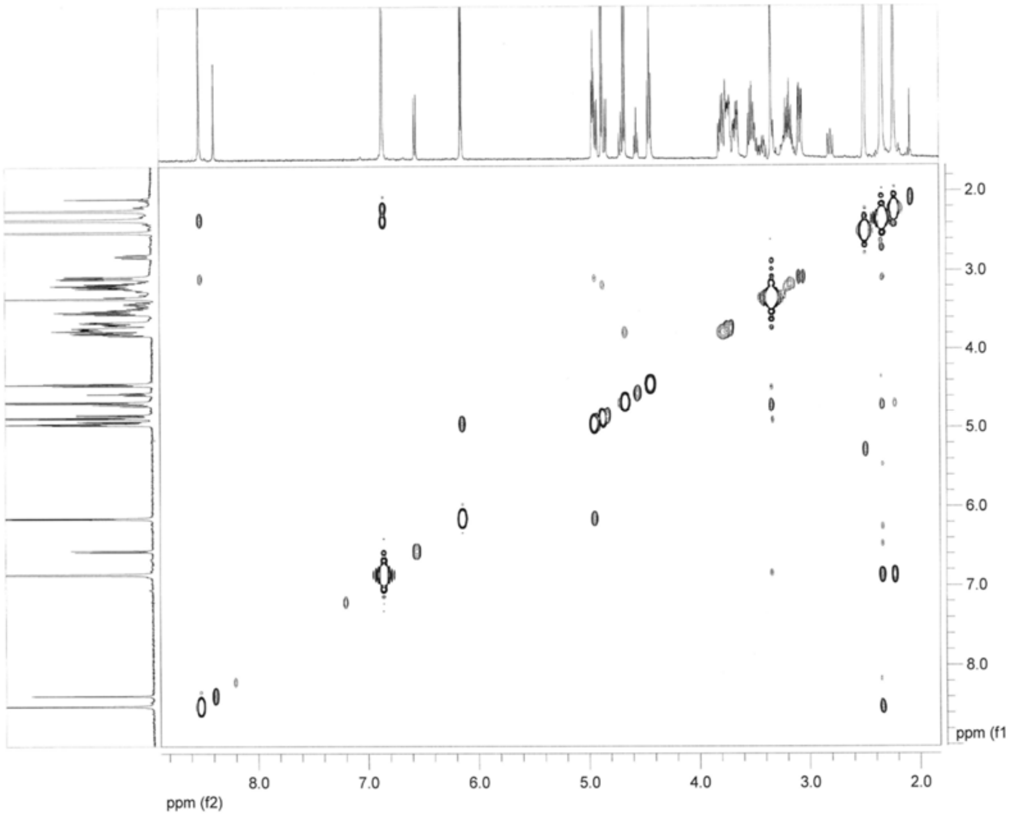

Figure S127. *COSY spectrum of **29** (DMSO-d_6)*

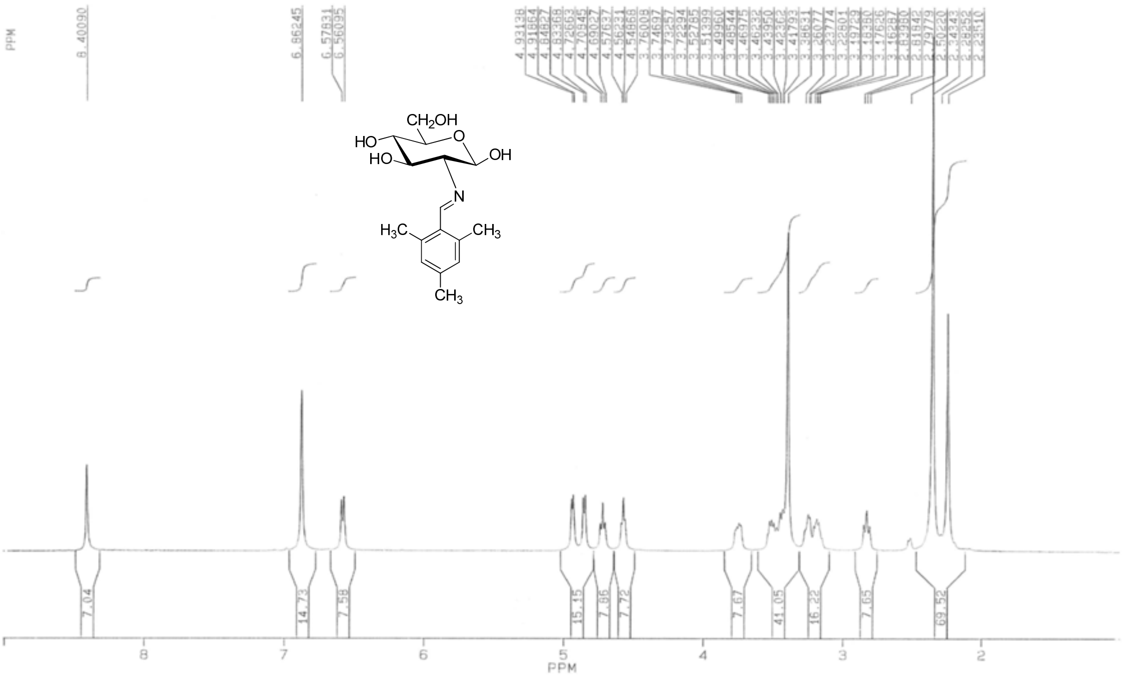

Figure S128. *^{1}H NMR spectrum of **30** (400 MHz, DMSO-d_6)*

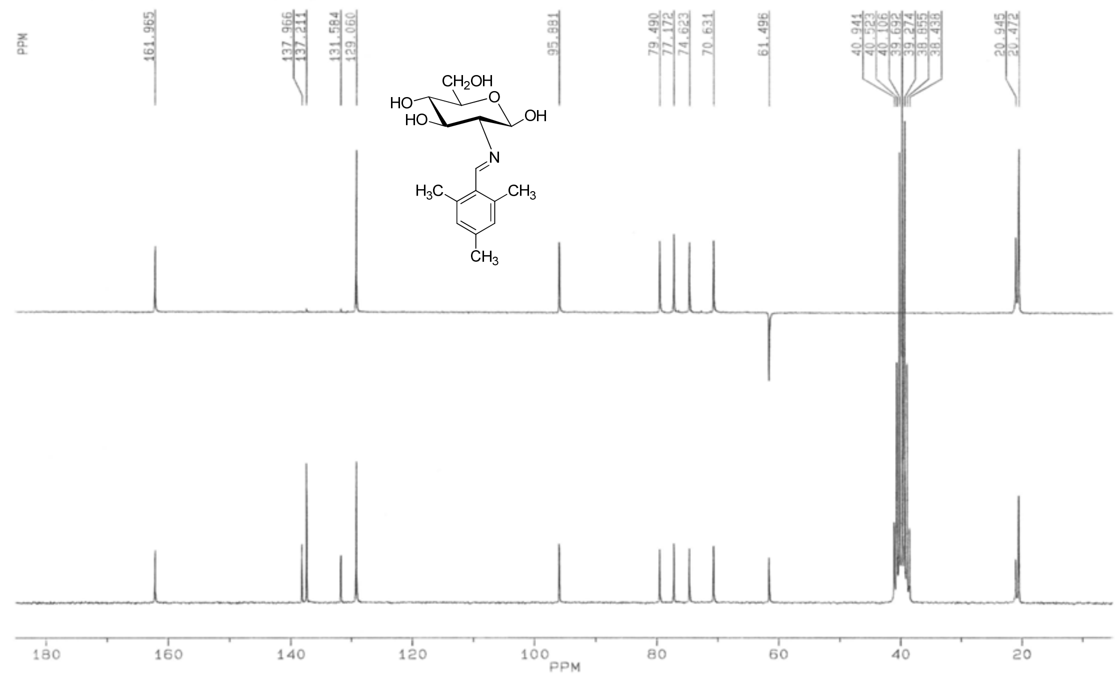

Figure S129. *$^{13}C\{^1H\}$ NMR (top: DEPT) spectra of* ***30*** *(100 MHz, DMSO-d_6)*

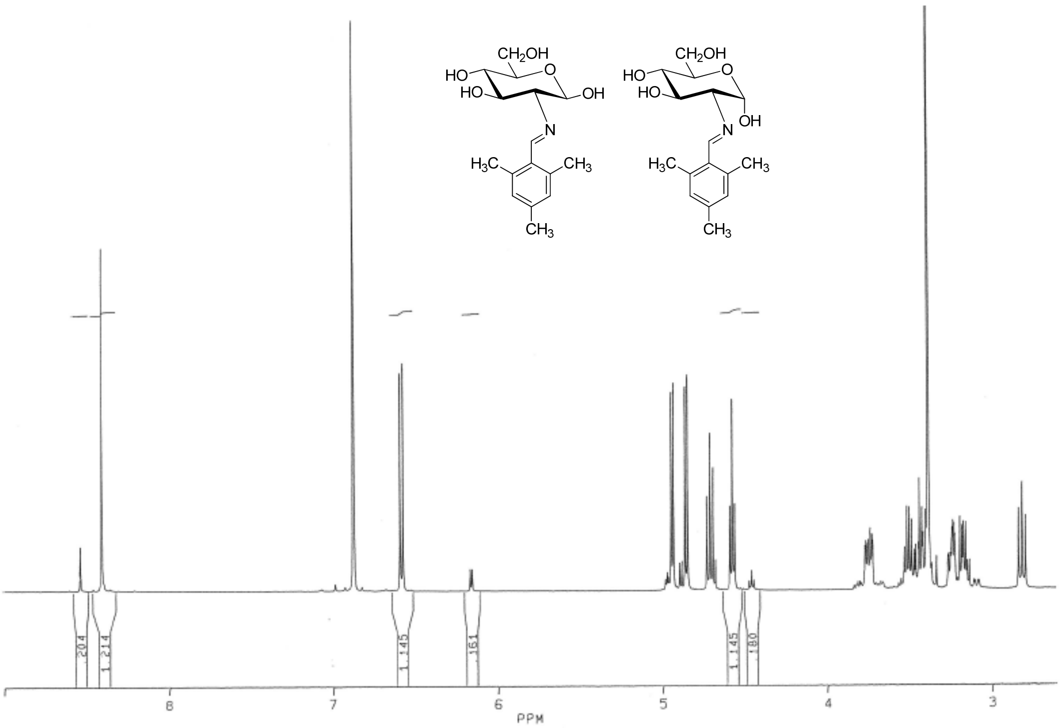

Figure S130. *^1H NMR spectrum of* ***29*** *and* ***30*** *(400 MHz, DMSO-d_6)*

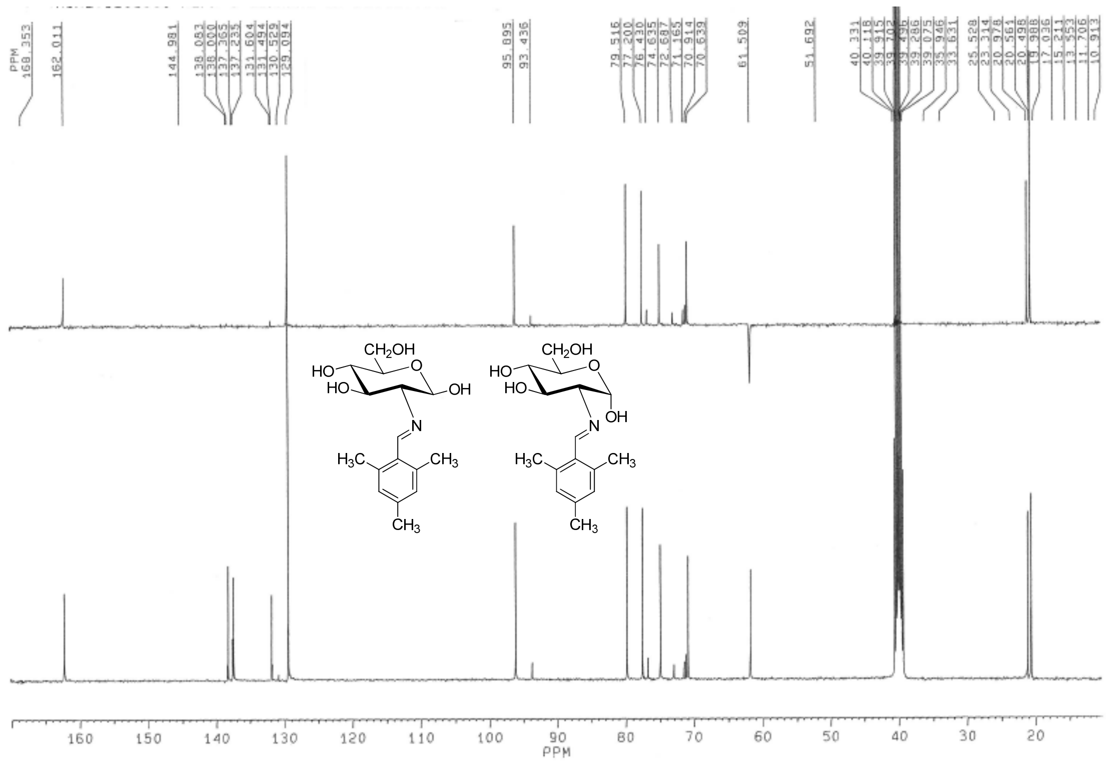

Figure S131. *$^{13}C\{^1H\}$ NMR (top: DEPT) spectra of* **29** *and* **30** *(100 MHz, DMSO-d$_6$)*

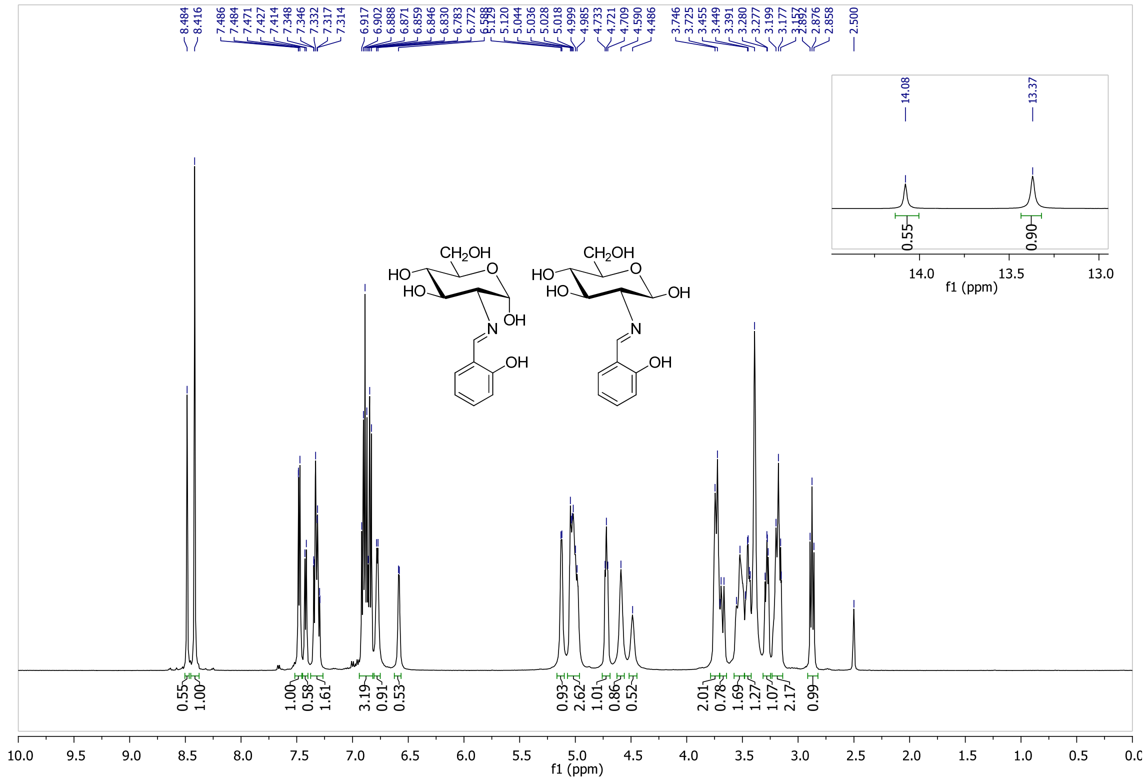

Figure S132. *^1H NMR spectrum of* **32** *and* **33** *(500 MHz, DMSO-d$_6$)*

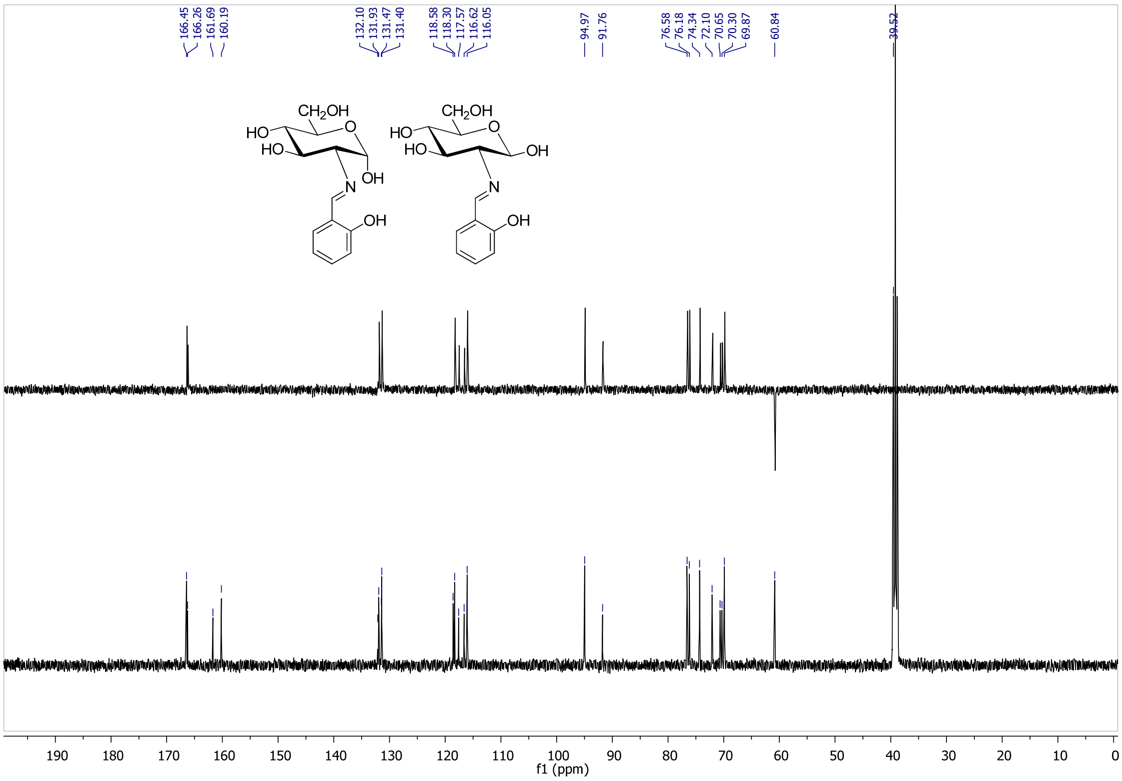

Figure S133. $^{13}C\{^{1}H\}$ *NMR (top: DEPT) spectra of* **32** *and* **33** *(125 MHz, DMSO-d*$_6$*)*

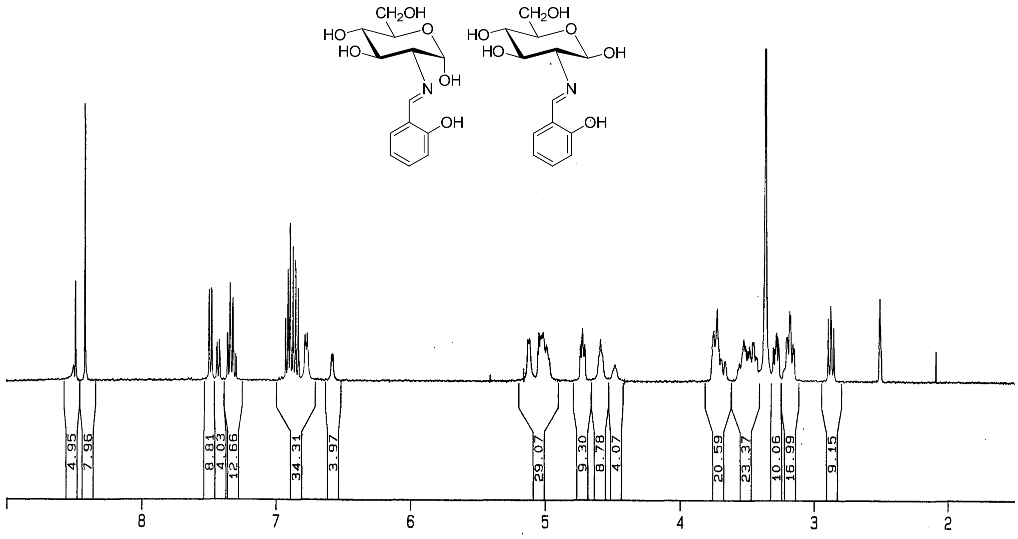

Figure S134. ^{1}H *NMR spectrum of a mixture of* **32** *and* **33** *in equilibrium (400 MHz, DMSO-d*$_6$*)*

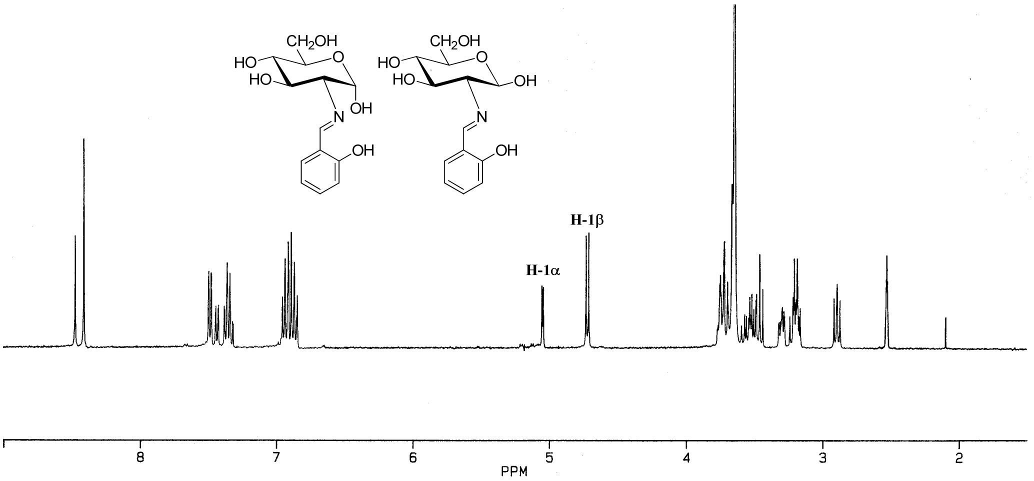

***Figure S135.** ^{1}H NMR spectrum of **32** and **33** in equilibrium after proton exchanging with D_2O*

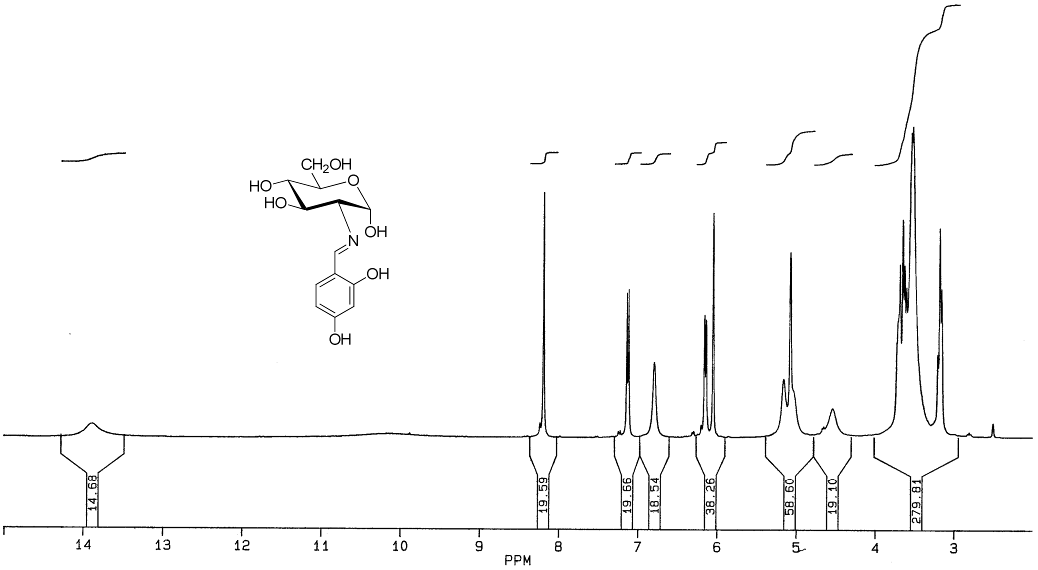

Figure S136. ^{1}H NMR spectrum of ***34*** (400 MHz, DMSO-d_6)

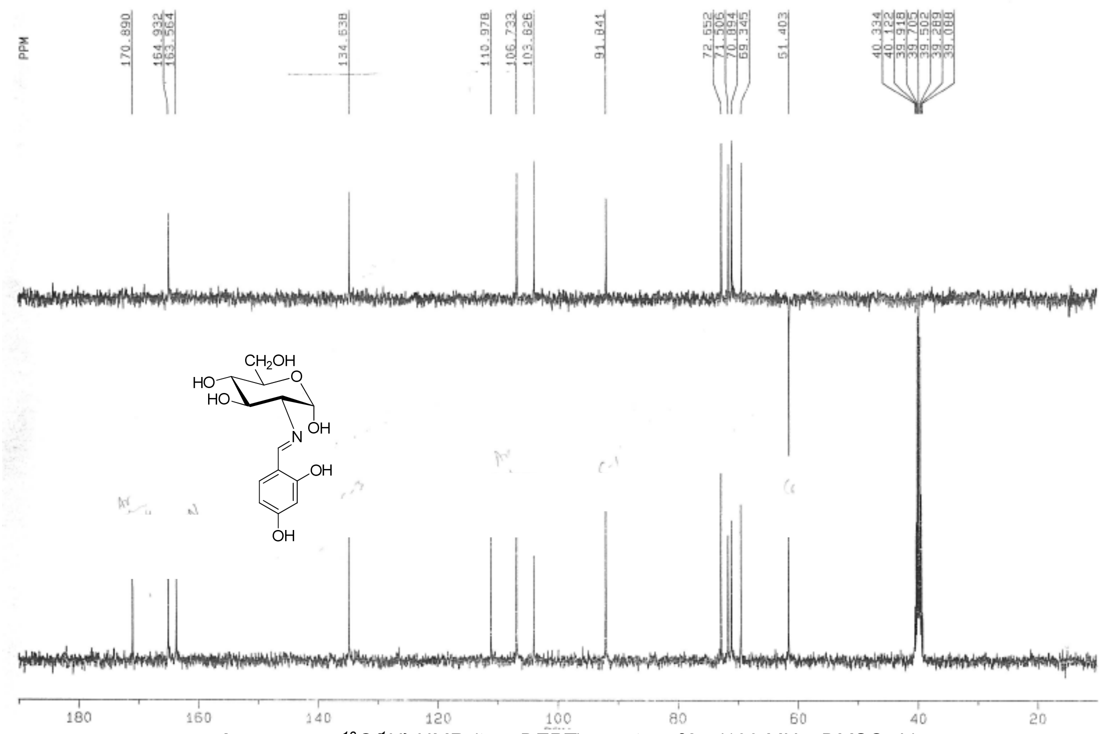

Figure S137. *$^{13}C\{^1H\}$ NMR (top: DEPT) spectra of* ***34*** *(100 MHz, DMSO-d_6)*

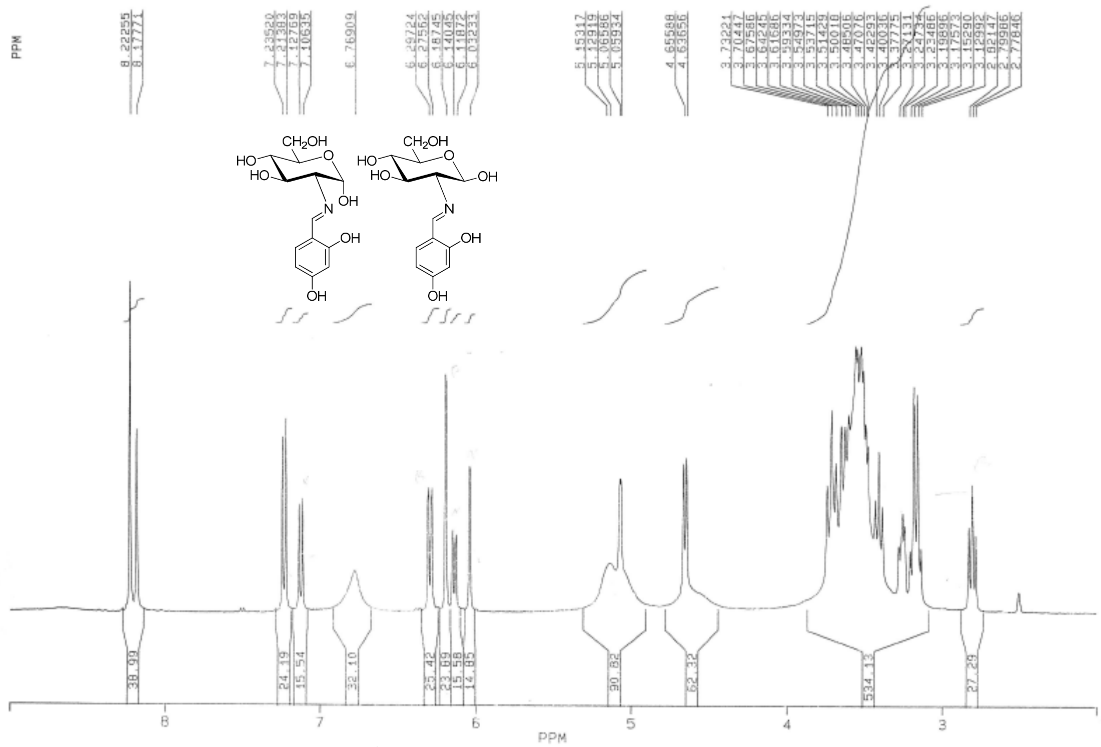

FIgure S138 *^1H NMR spectrum of* ***34*** *and* ***35*** *(400 MHz, DMSO-d_6)*

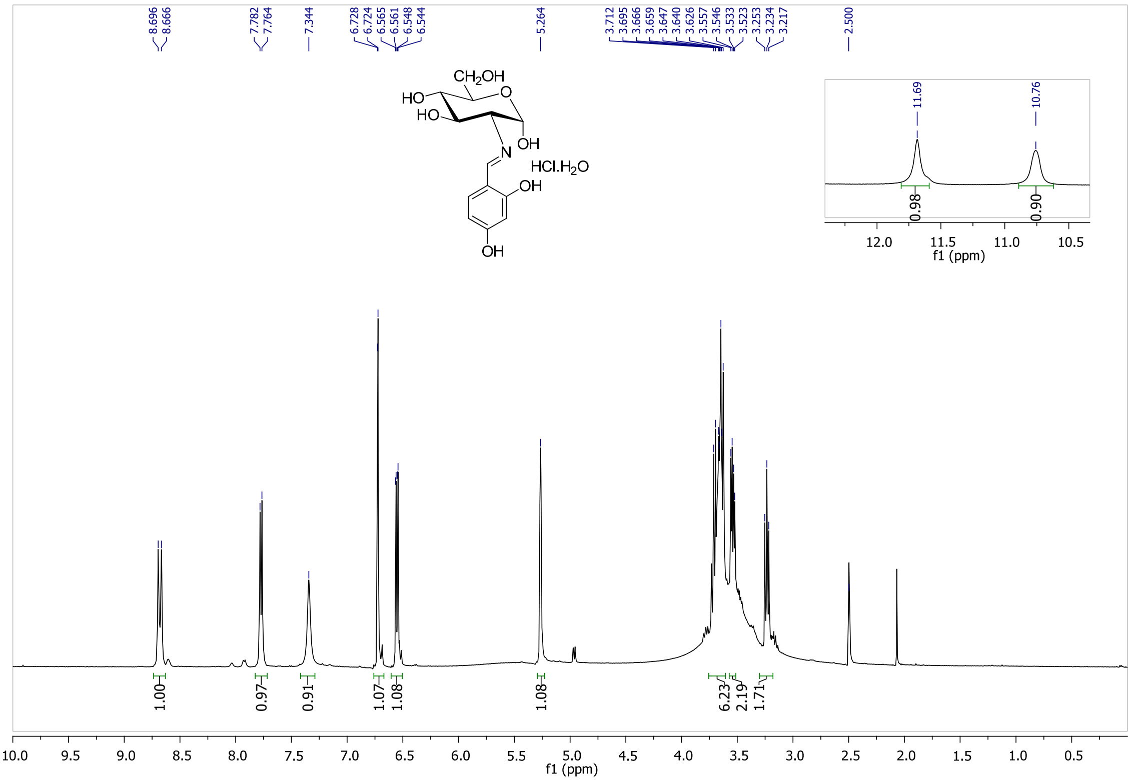

Figure S139. ^{1}H *NMR spectrum of* **36** *(500 MHz, DMSO-d_6)*

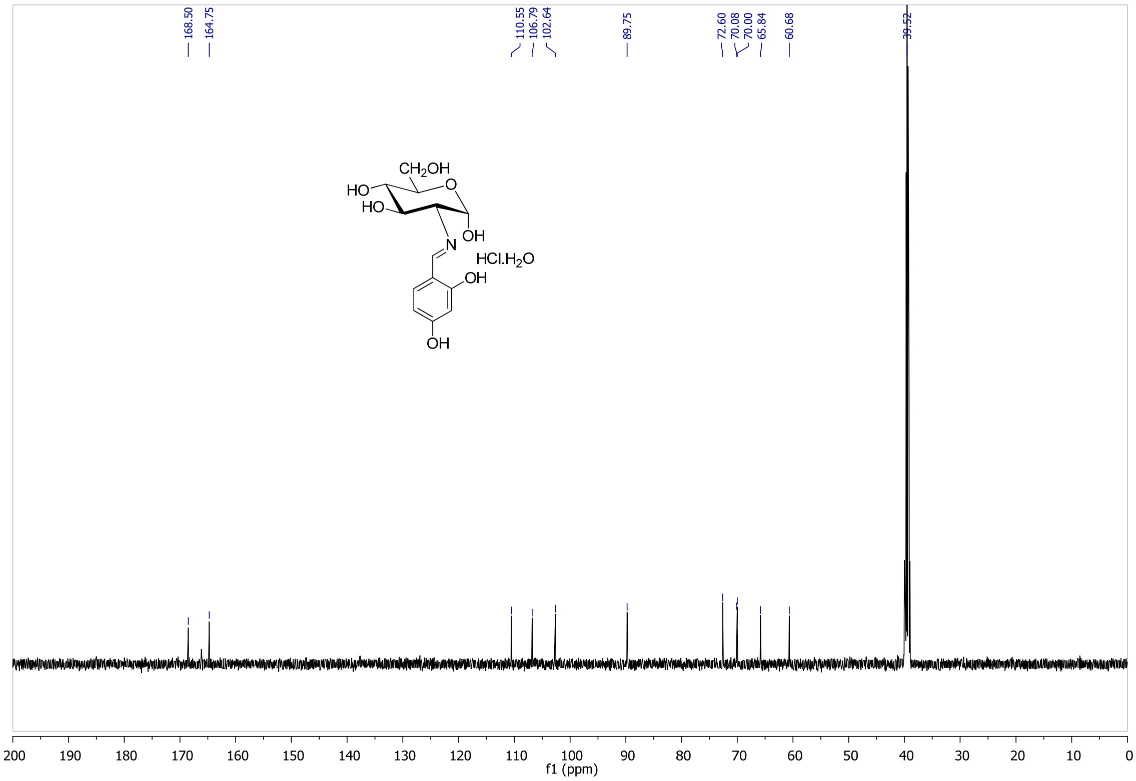

Figure S140. $^{13}C\{^{1}H\}$ *NMR spectrum of* **36** *(125 MHz, DMSO-d_6)*

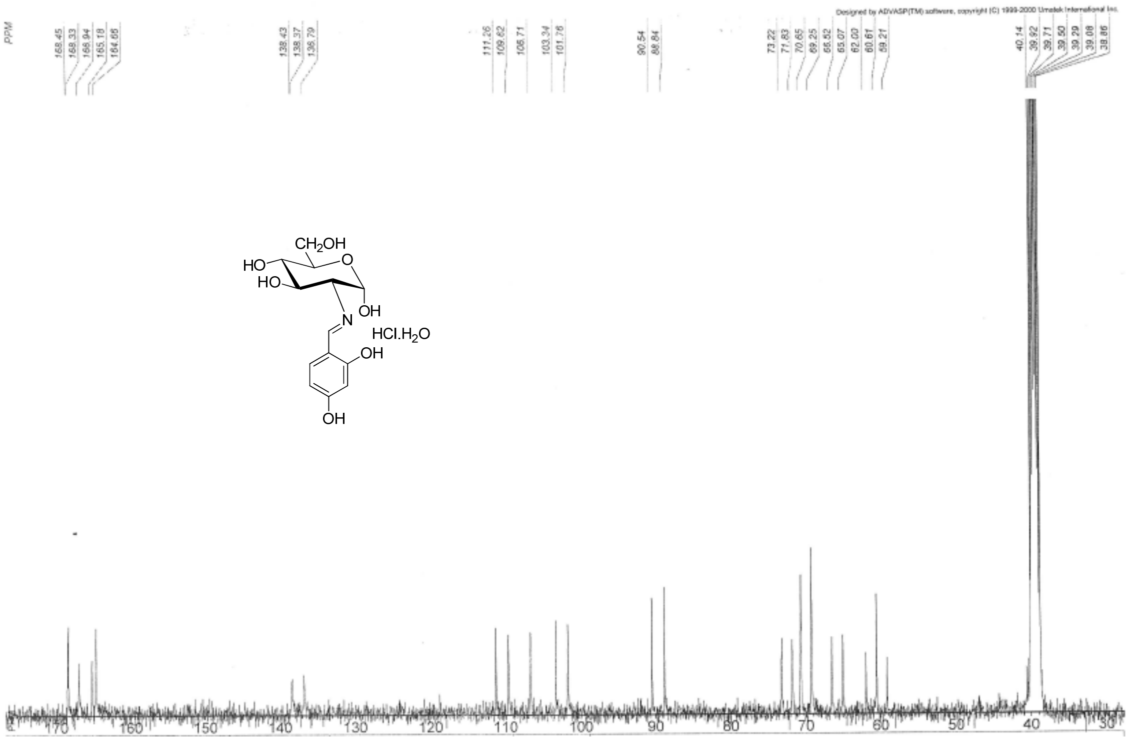

Figure S141. *Proton-coupled* [13]*C NMR spectrum of* ***36*** *(100 MHz, DMSO-d*$_6$*)*

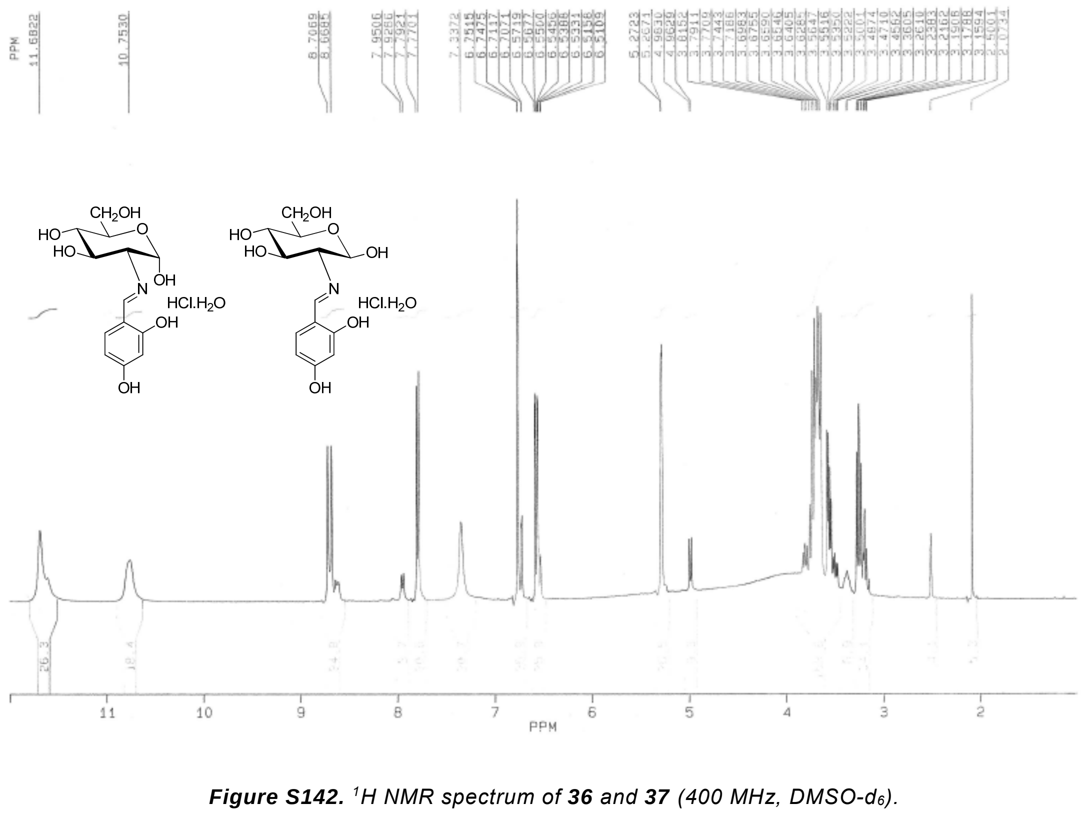

Figure S142. [1]*H NMR spectrum of* ***36*** *and* ***37*** *(400 MHz, DMSO-d*$_6$*).*

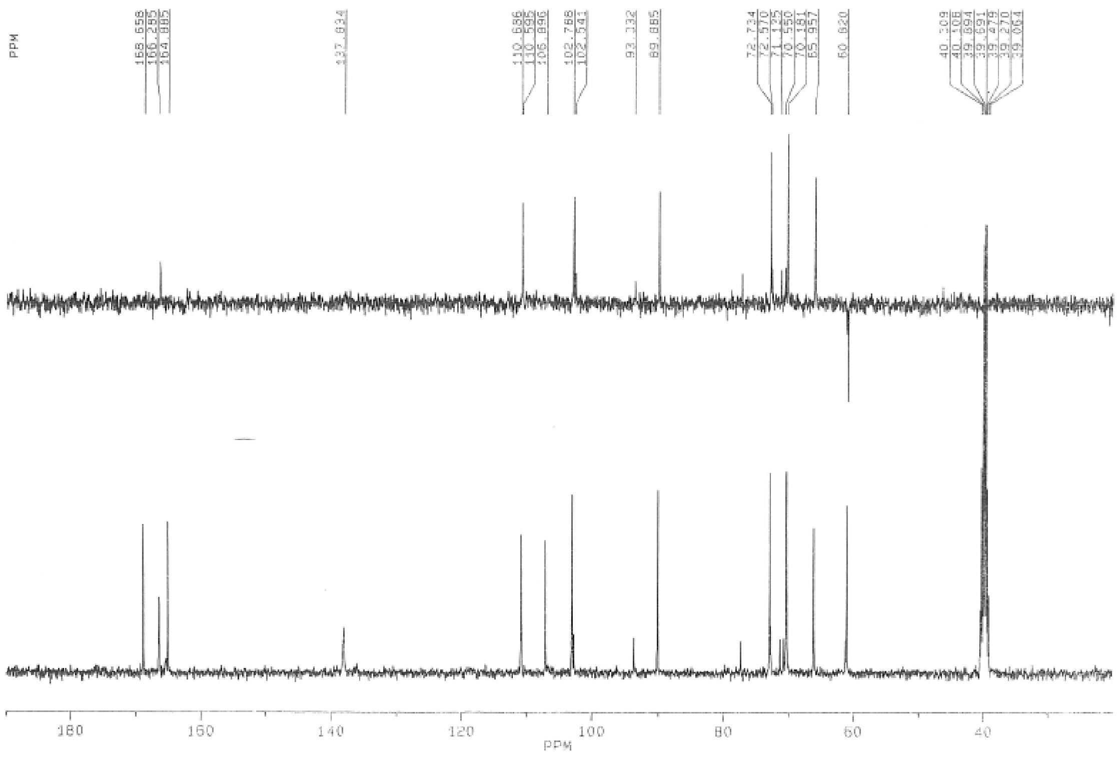

Figure S143. *$^{13}C\{^1H\}$ NMR (top: DEPT) spectra of* **36** and **37** *(100 MHz, DMSO-d_6)*

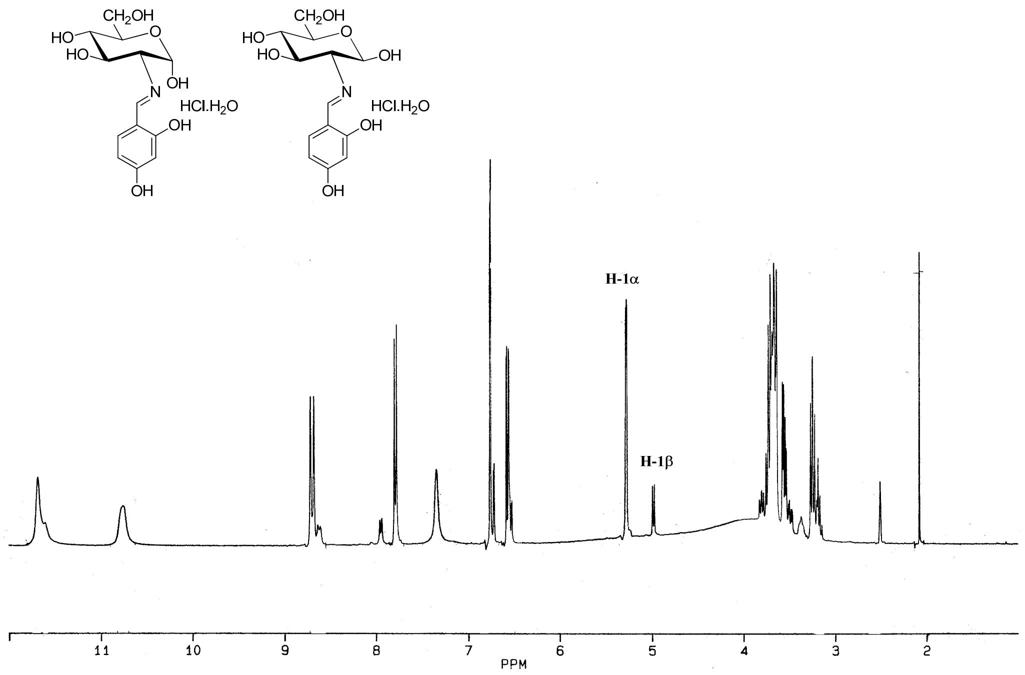

Figure S144. *^1H NMR spectrum of* ***36*** *and* ***37*** *(400 MHz, DMSO-d_6)*

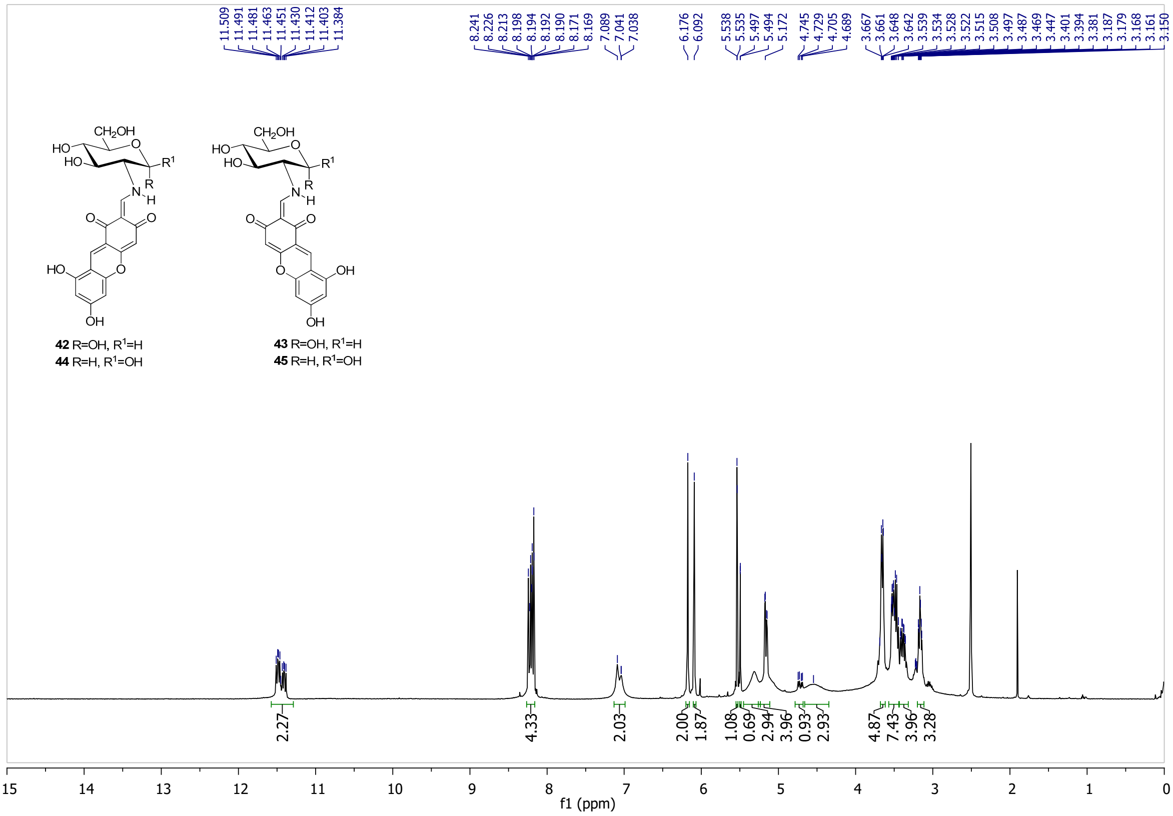

Figure S145. *^{1}H NMR spectrum of **39-42** (500 MHz, DMSO-d6)*

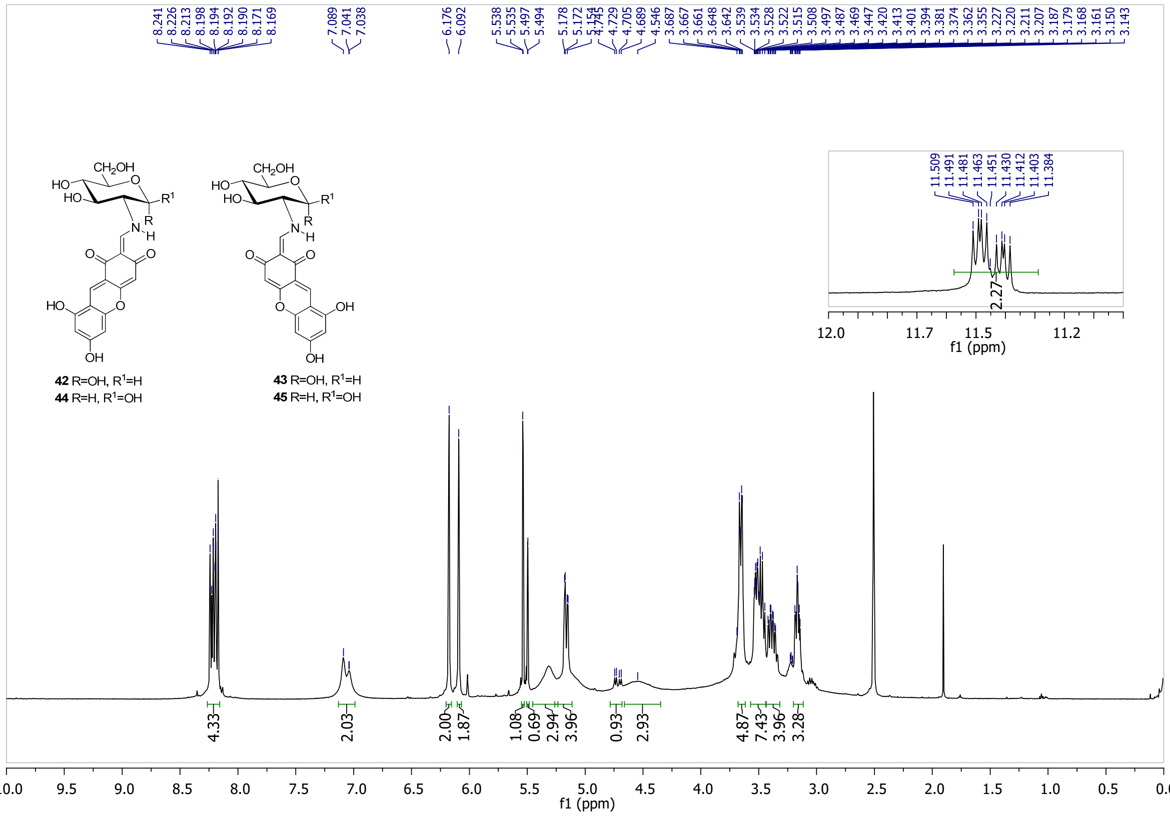

Figure S146. *^{1}H NMR spectrum of **39-42** (500 MHz, DMSO-d6)*

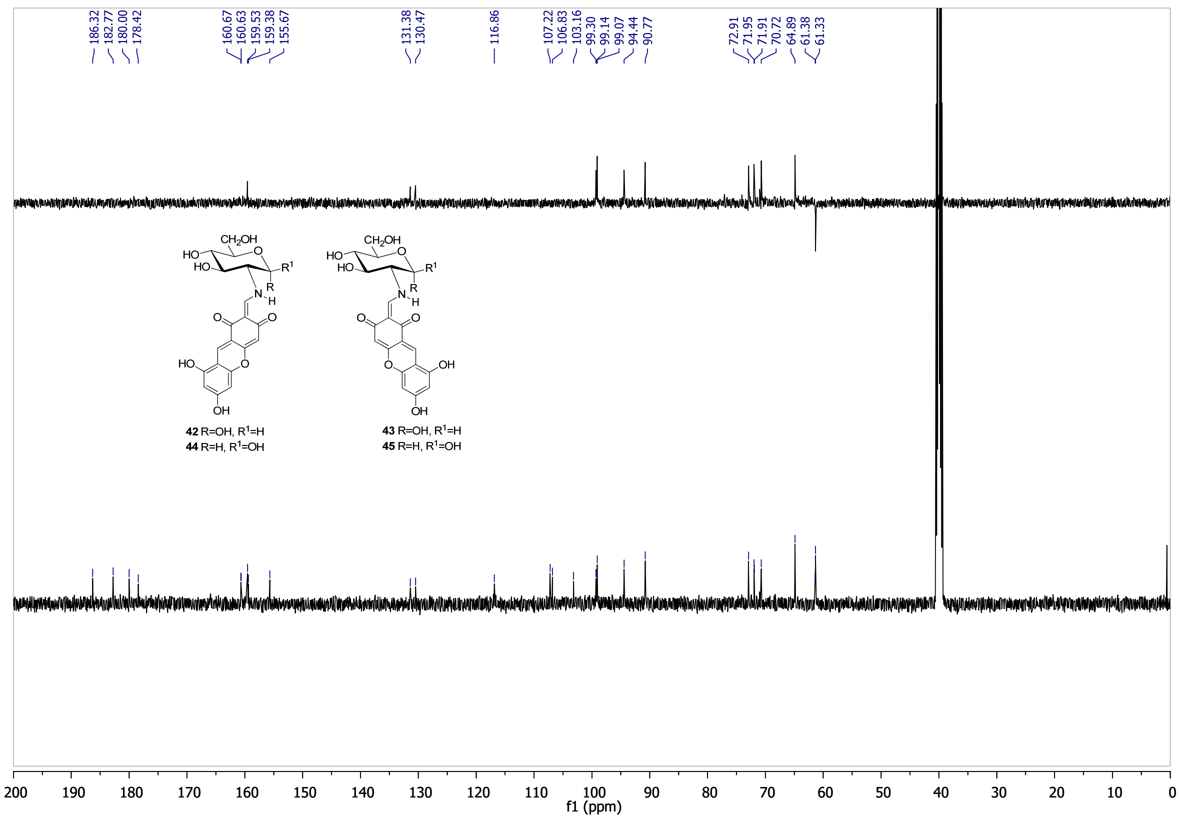

Figure S147. *$^{13}C\{^1H\}$ NMR (top: DEPT) spectra of **39-42** (125 MHz, DMSO-d$_6$)*

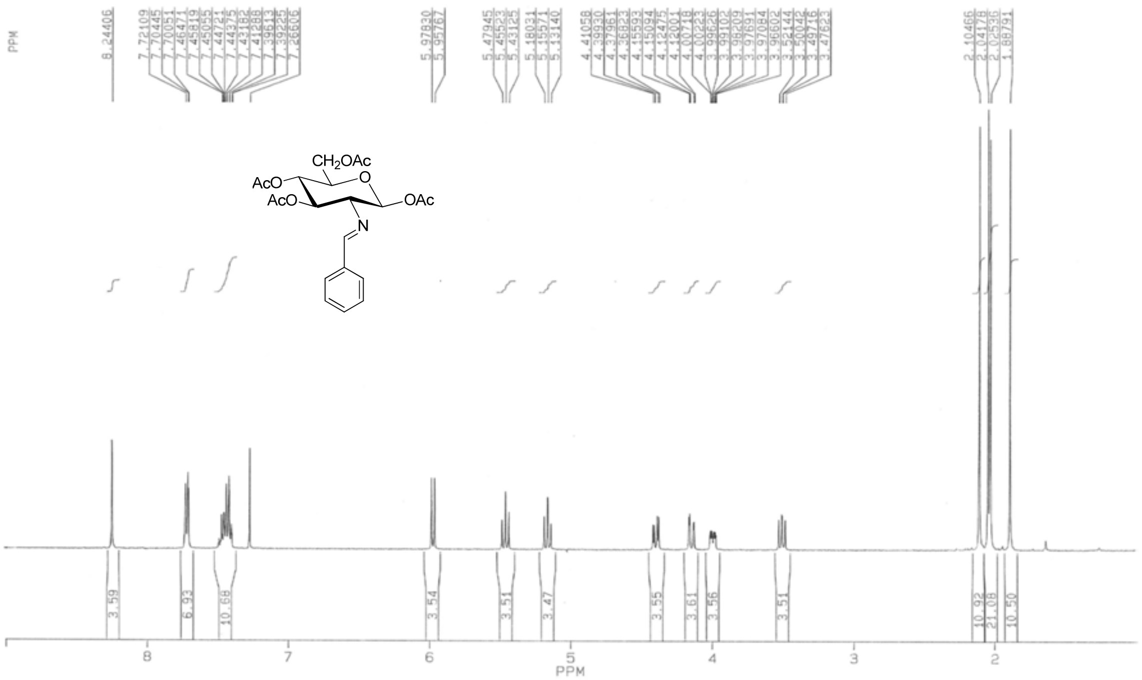

Figure S148. *^1H NMR spectrum of **43** (400 MHz, $CDCl_3$)*

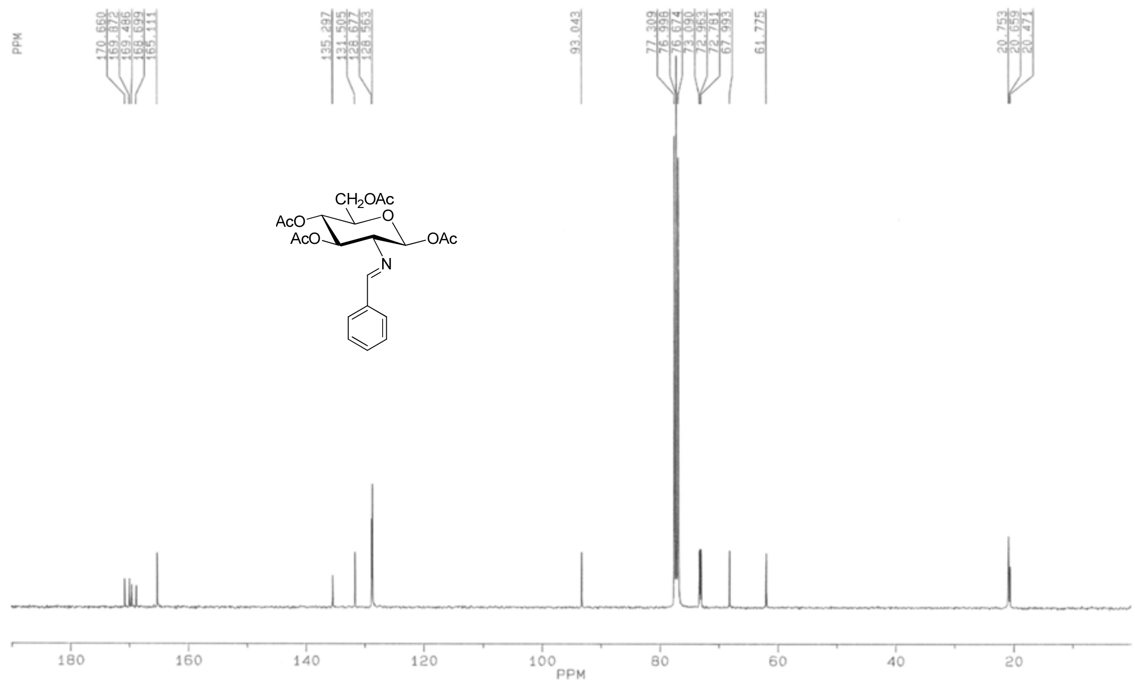

Figure S149. *$^{13}C\{^{1}H\}$ NMR spectrum of **43** (100 MHz, $CDCl_3$)*

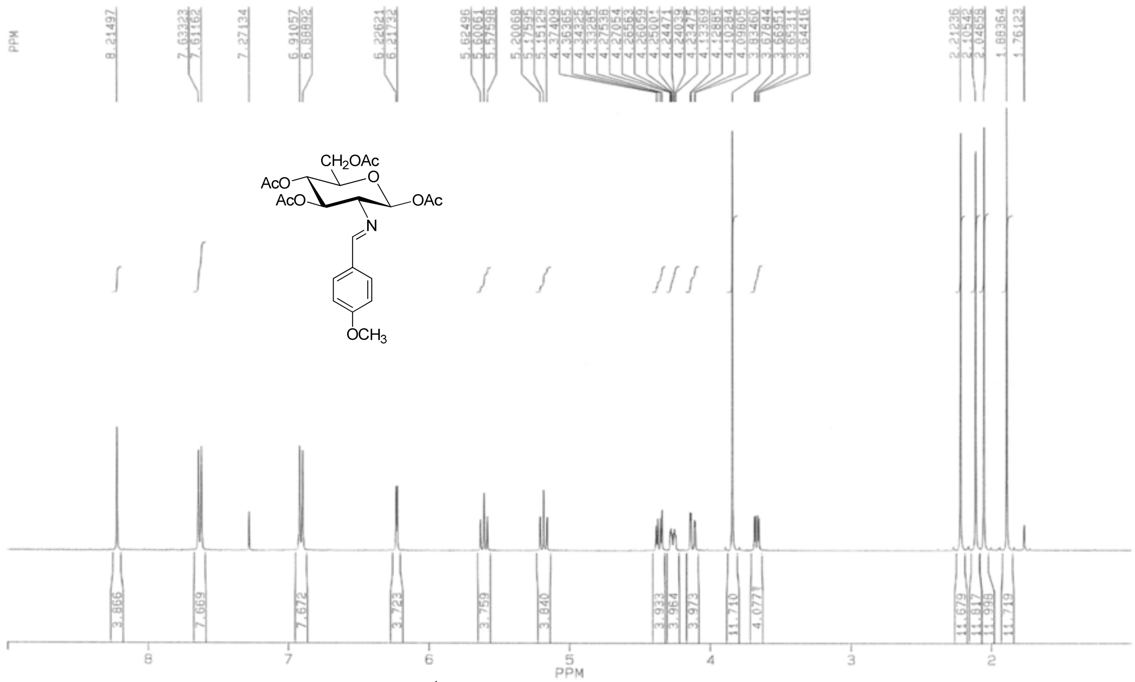

Figure S150. *^{1}H NMR spectrum of **44** (400 MHz, $CDCl_3$)*

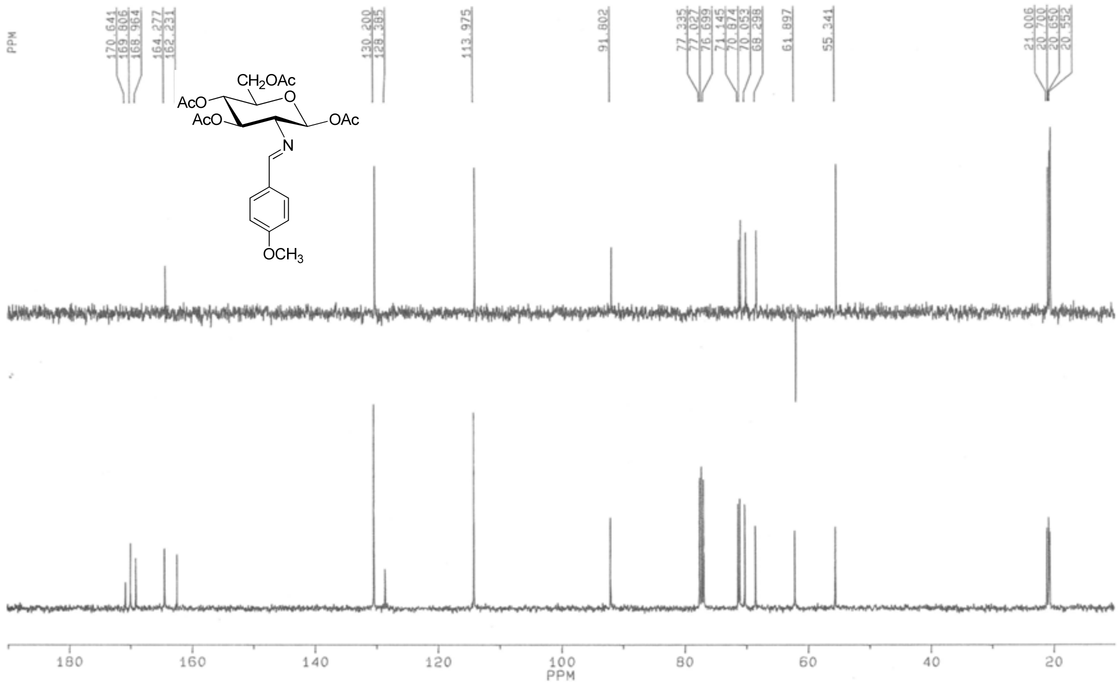

Figure S151. *$^{13}C\{^{1}H\}$ NMR (top: DEPT) spectra of **44** (100 MHz, $CDCl_3$)*

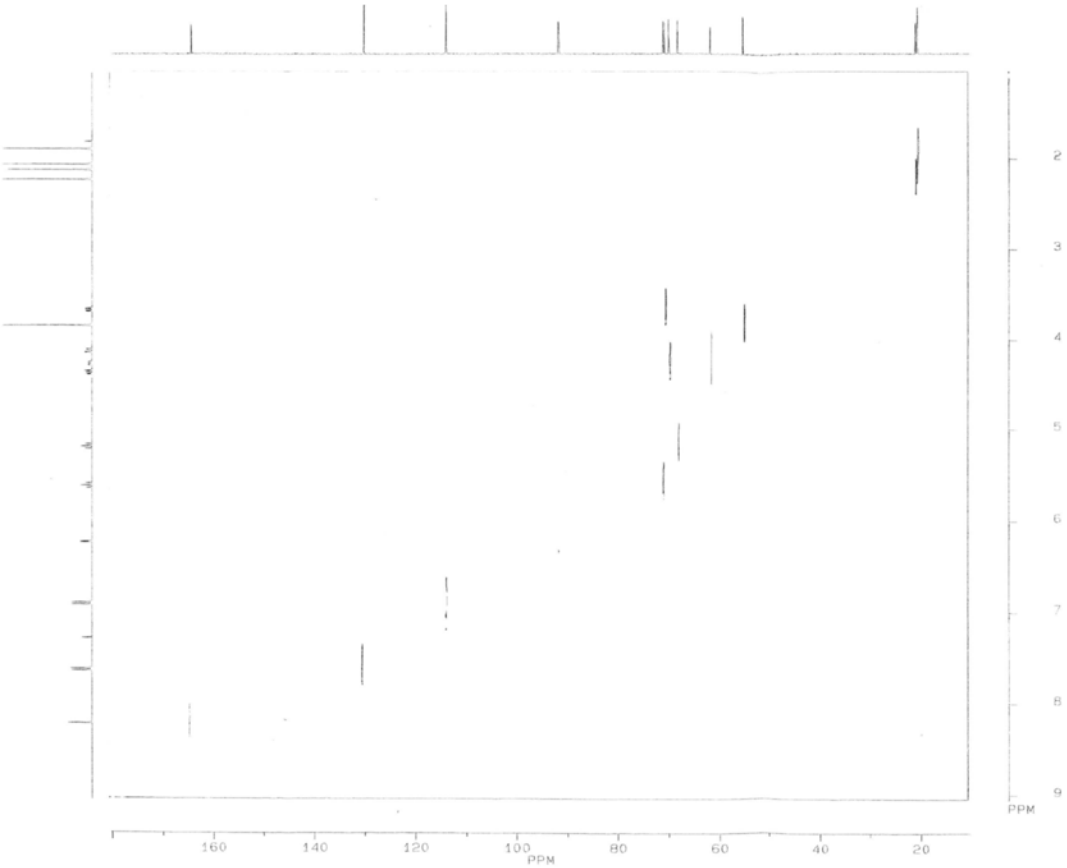

Figure S152. *HMQC spectrum of **44** (CDCl₃)*

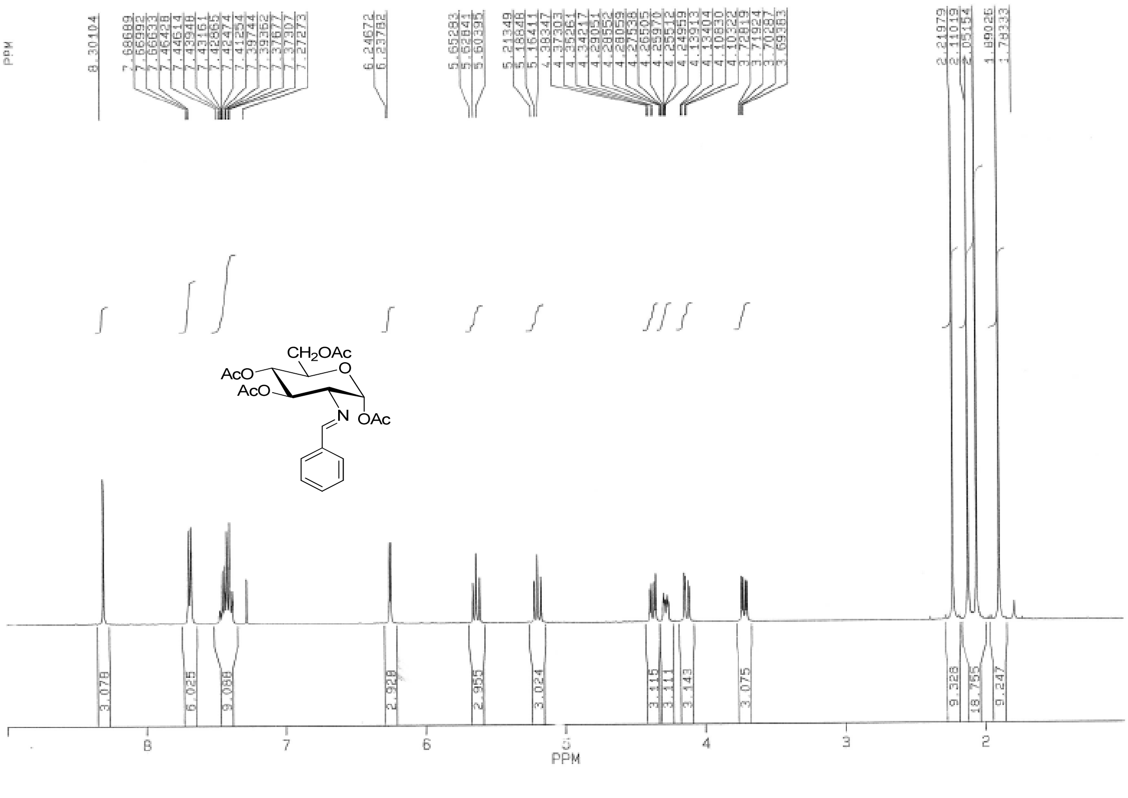

Figure S153. *^{1}H NMR spectrum of **46** (400 MHz, CDCl₃)*

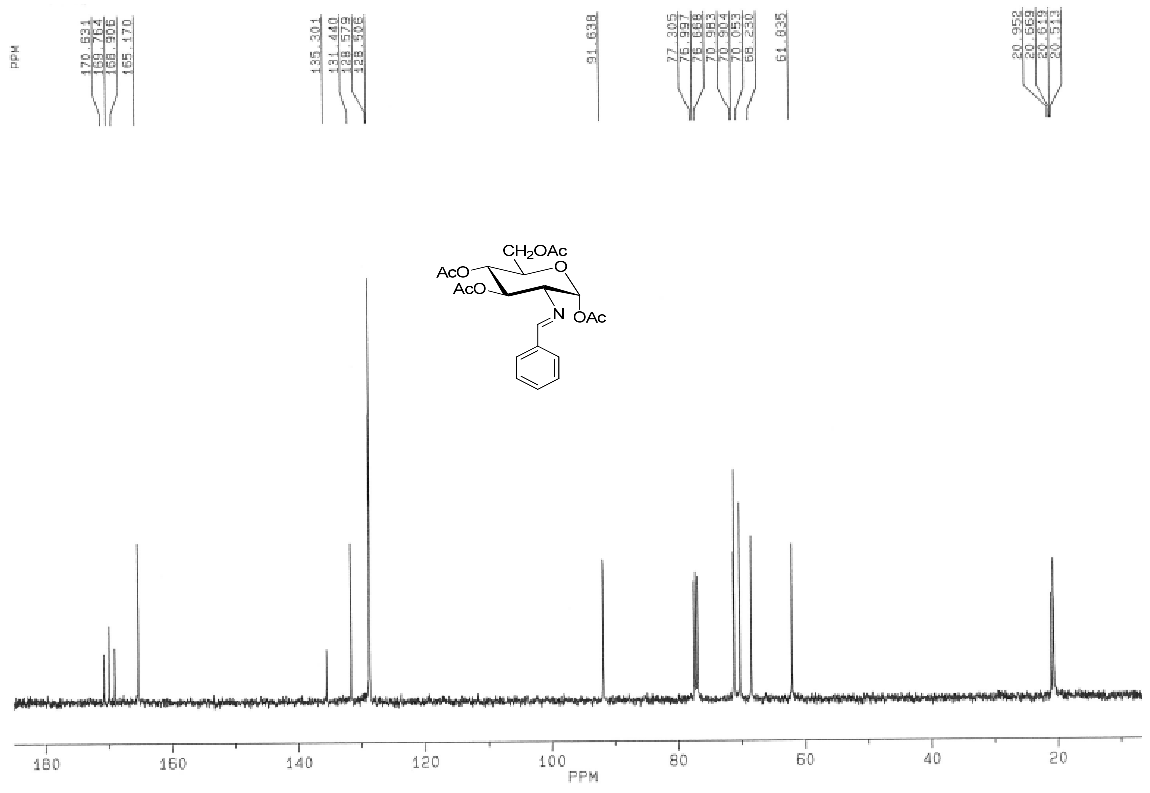

Figure S154. *$^{13}C\{^{1}H\}$ NMR spectrum of **46** (100 MHz, $CDCl_3$)*

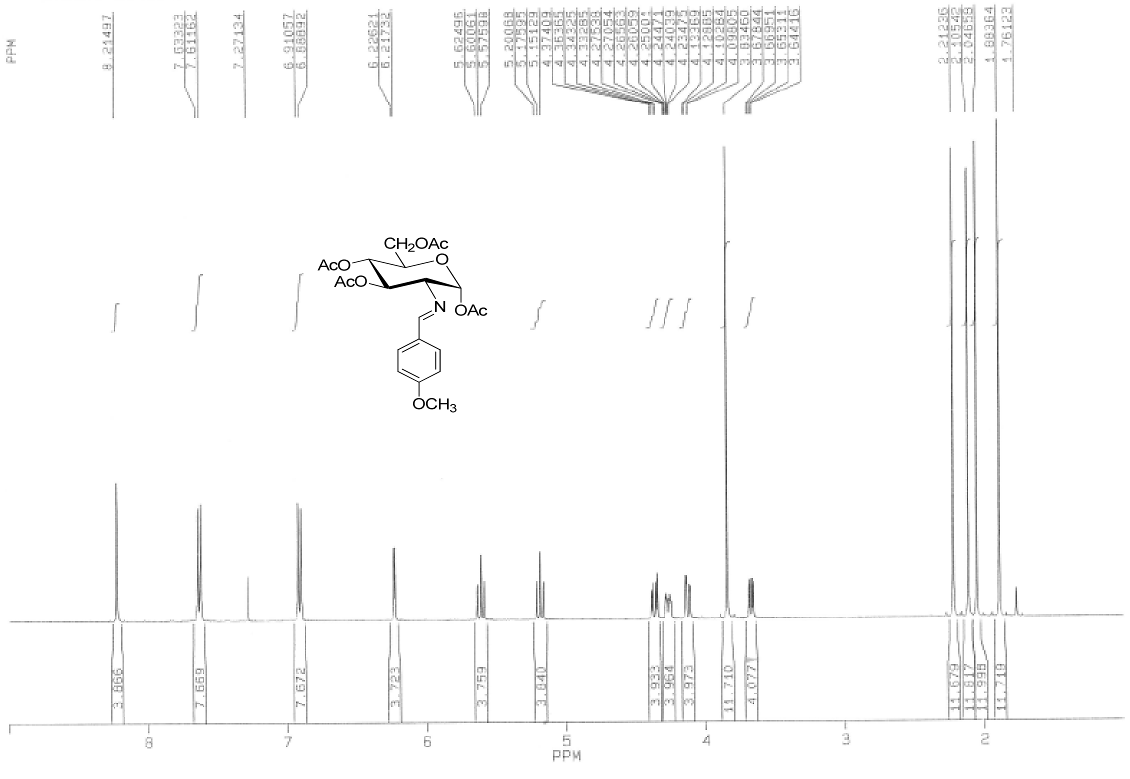

Figure S155. *^{1}H NMR spectrum of **47** (400 MHz, $CDCl_3$)*

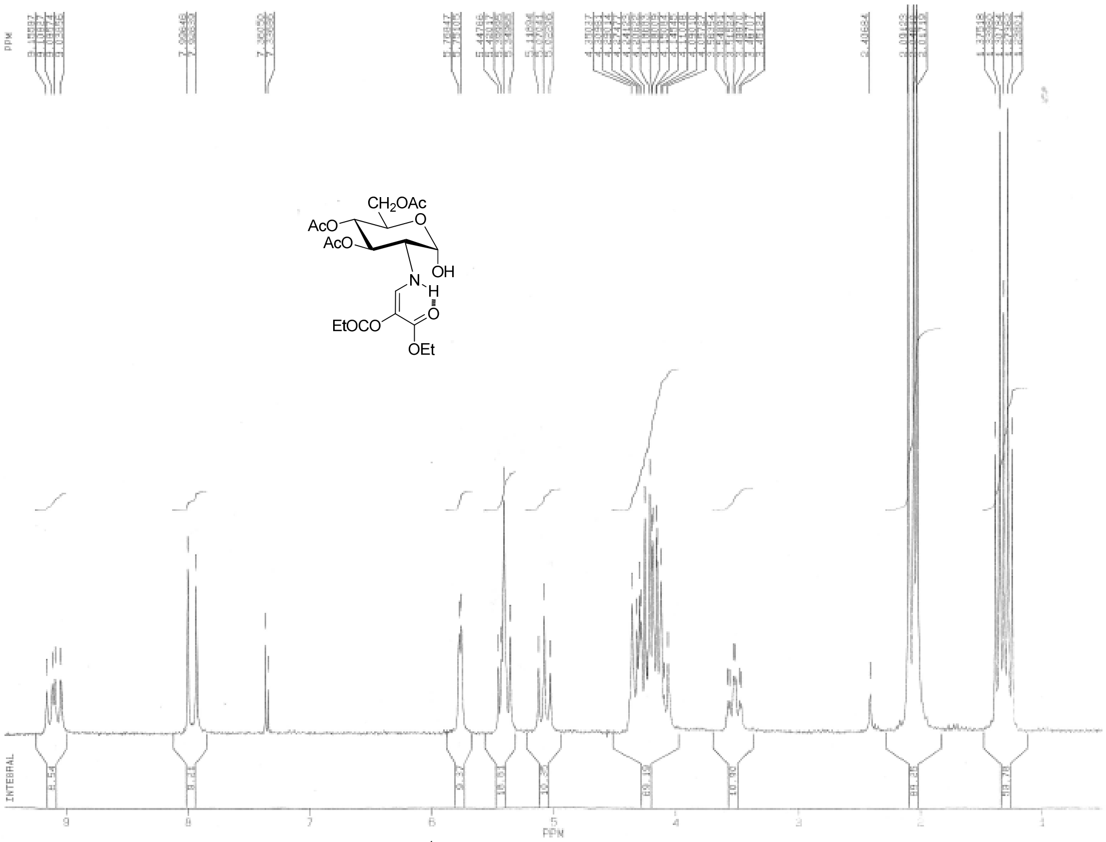

Figure S156. *¹H NMR spectrum of **48** (400 MHz, CDCl₃)*

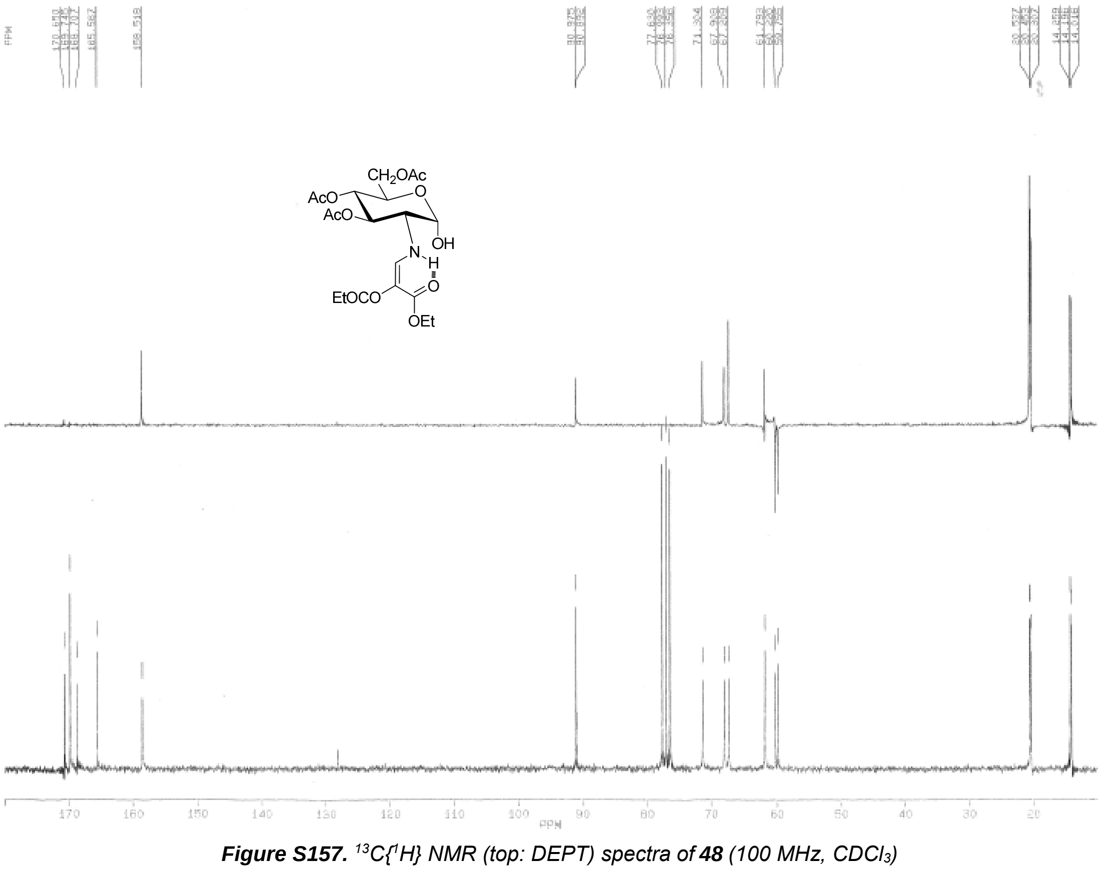

Figure S157. *$^{13}C\{^1H\}$ NMR (top: DEPT) spectra of **48** (100 MHz, $CDCl_3$)*

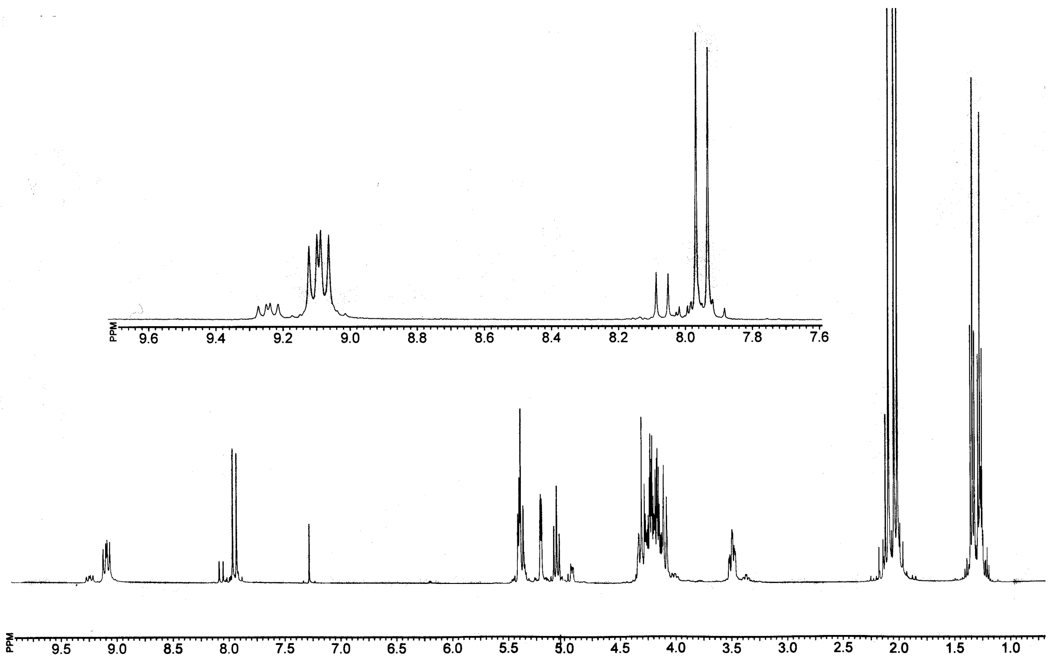

Figure S158. [1]*H NMR spectrum (top: magnified zone) of* ***48*** *(400 MHz, CDCl₃)*

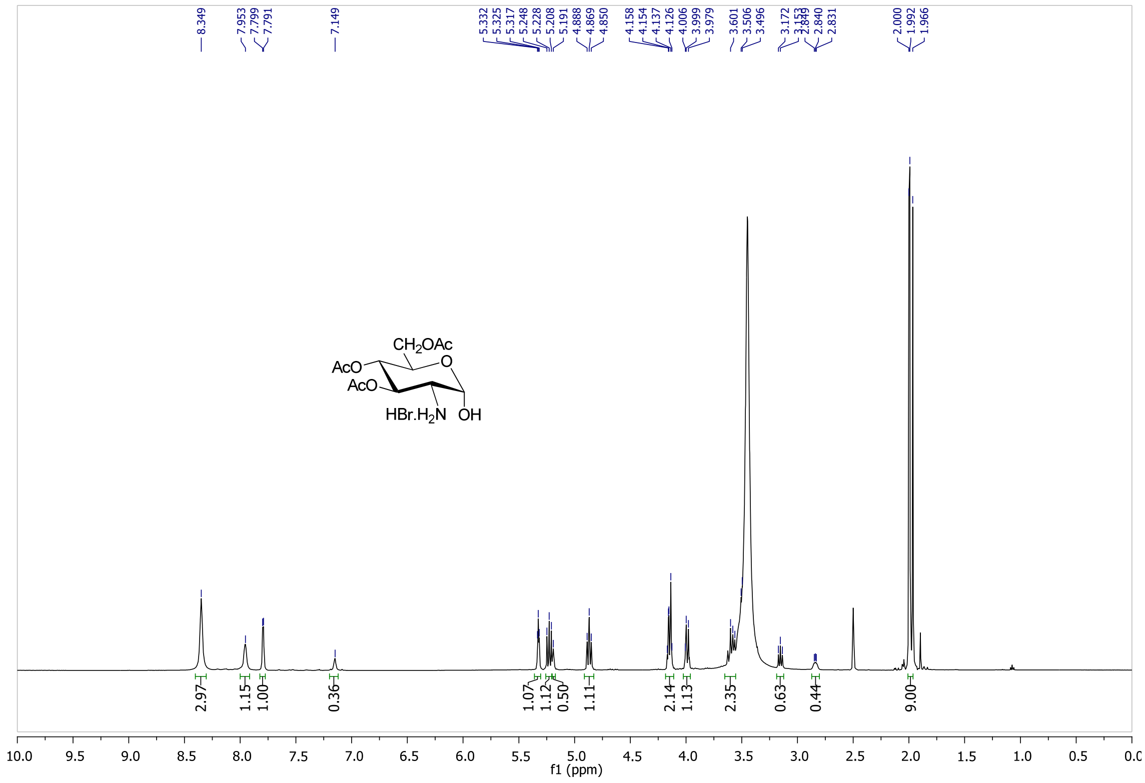

Figure S159. [1]*H NMR spectrum of* ***49*** *(500 MHz, DMSO-d₆)*

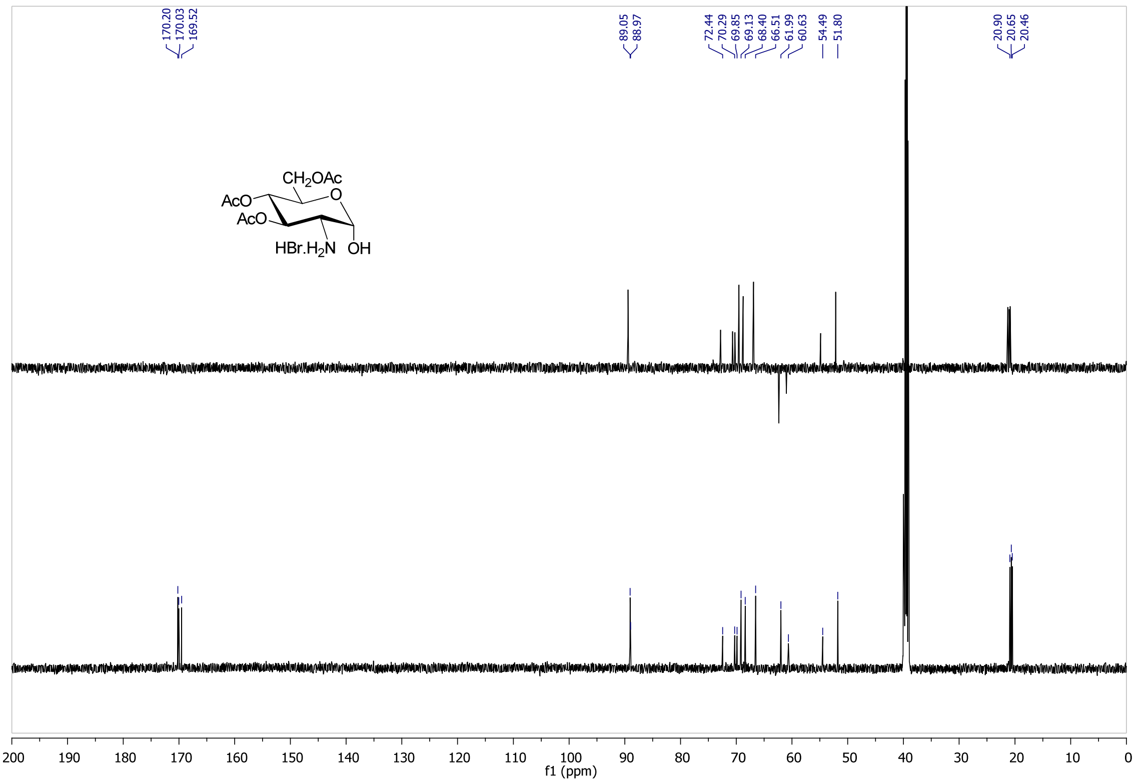

Figure S160. $^{13}C\{^1H\}$ *NMR (top: DEPT) spectra of* ***49*** *(125 MHz, DMSO-d6)*

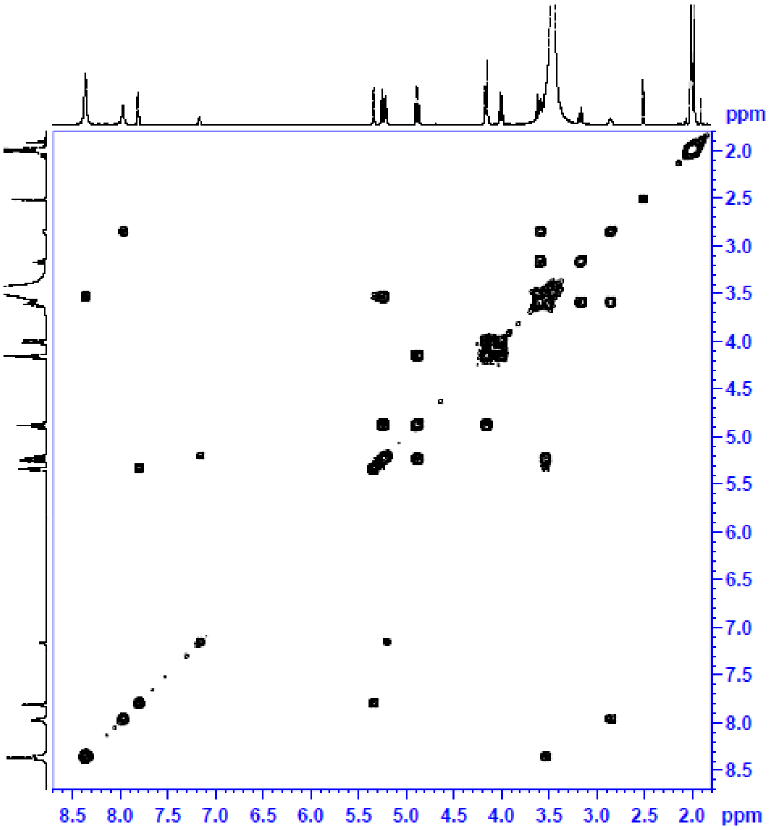

Figure S161.*COSY spectrum of* ***49*** *(DMSO-d6)*

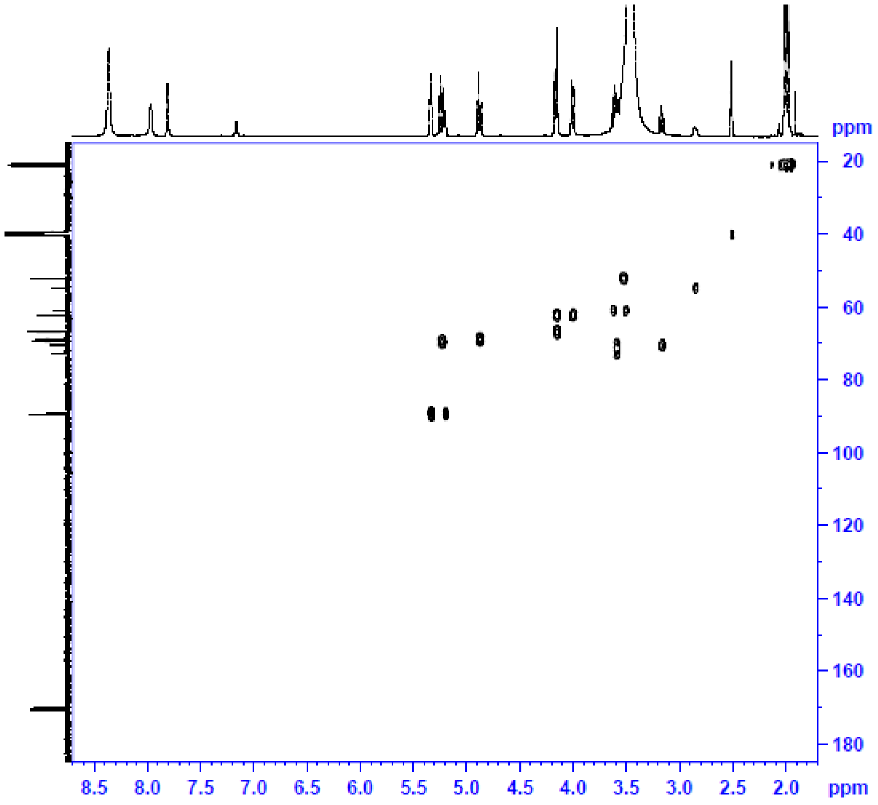

Figure S162. *HMQC spectrum of **49** (DMSO-d₆)*

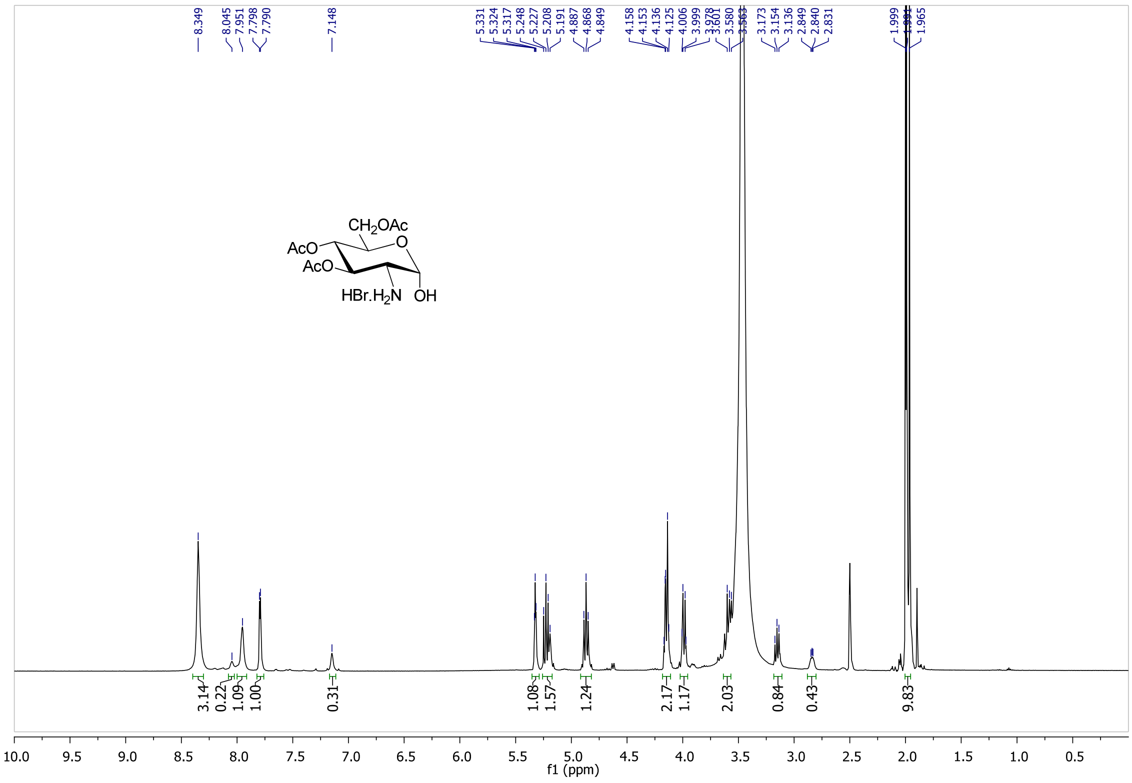

Figure S163. *^{1}H NMR spectrum of **49** in equilibrium (500 MHz, DMSO-d_6)*

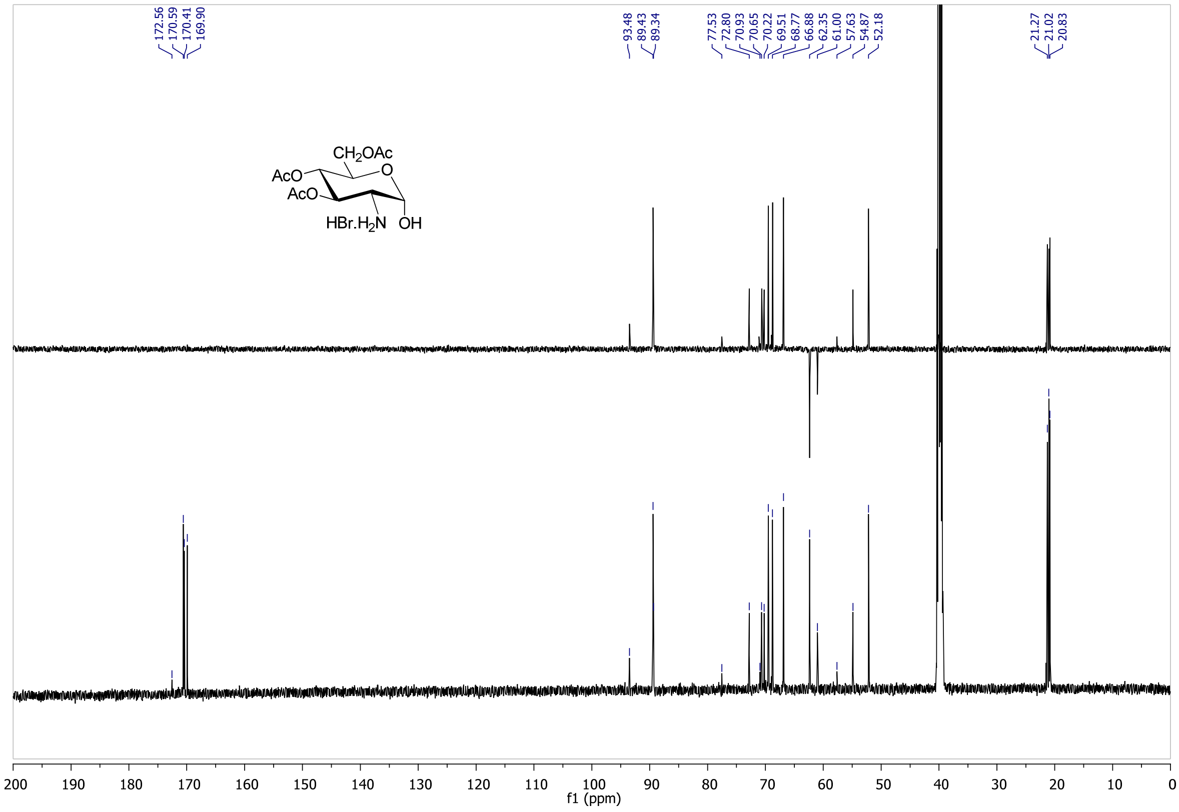

Figure S164. $^{13}C\{^{1}H\}$ *NMR (top: DEPT) spectra of* ***49*** *in equilibrium (125 MHz, DMSO-d$_6$)*

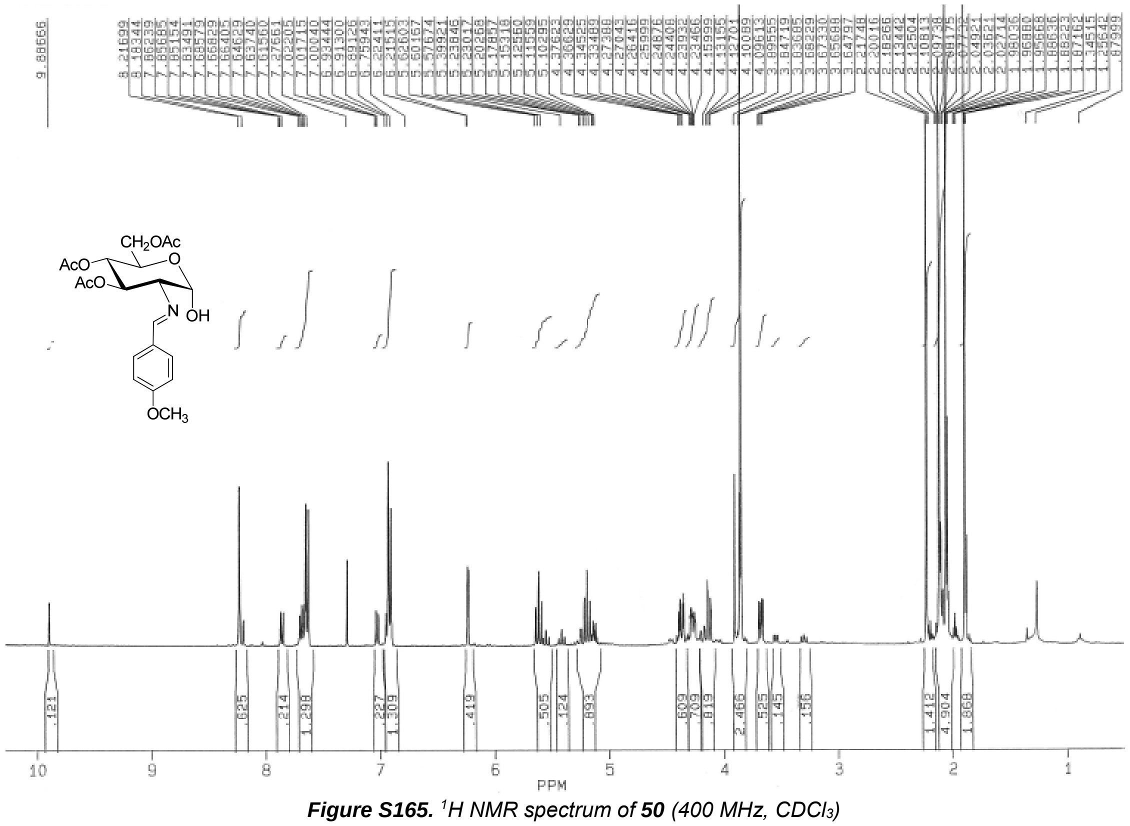

Figure S165. *^{1}H NMR spectrum of* ***50*** *(400 MHz, $CDCl_3$)*

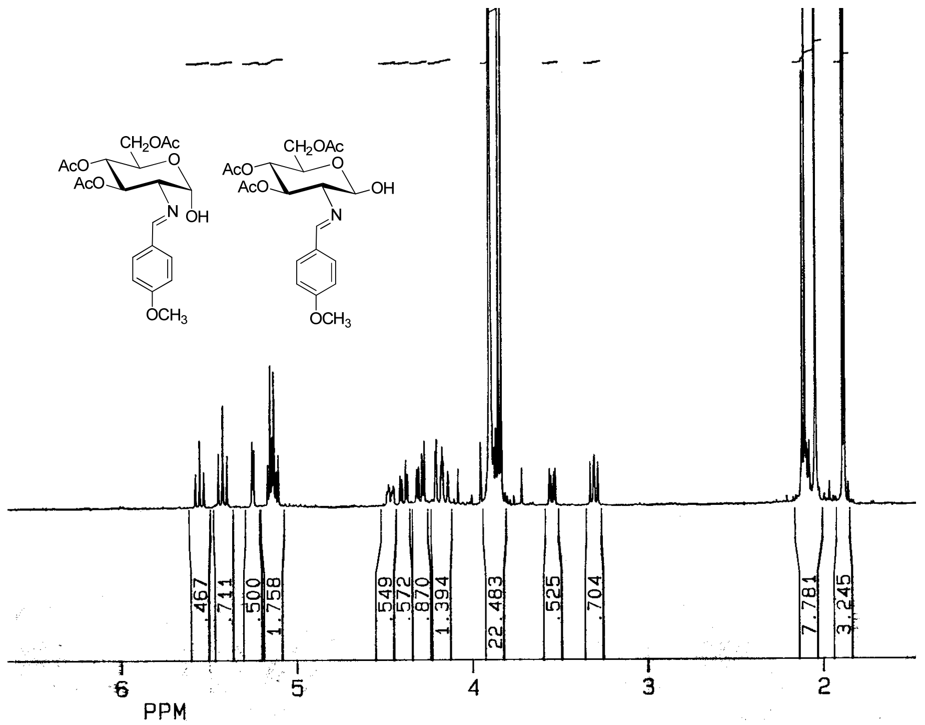

Figure S166. *^{1}H NMR spectrum of **50** and **51*** (α and β mixture) *(400 MHz, CDCl$_3$)*

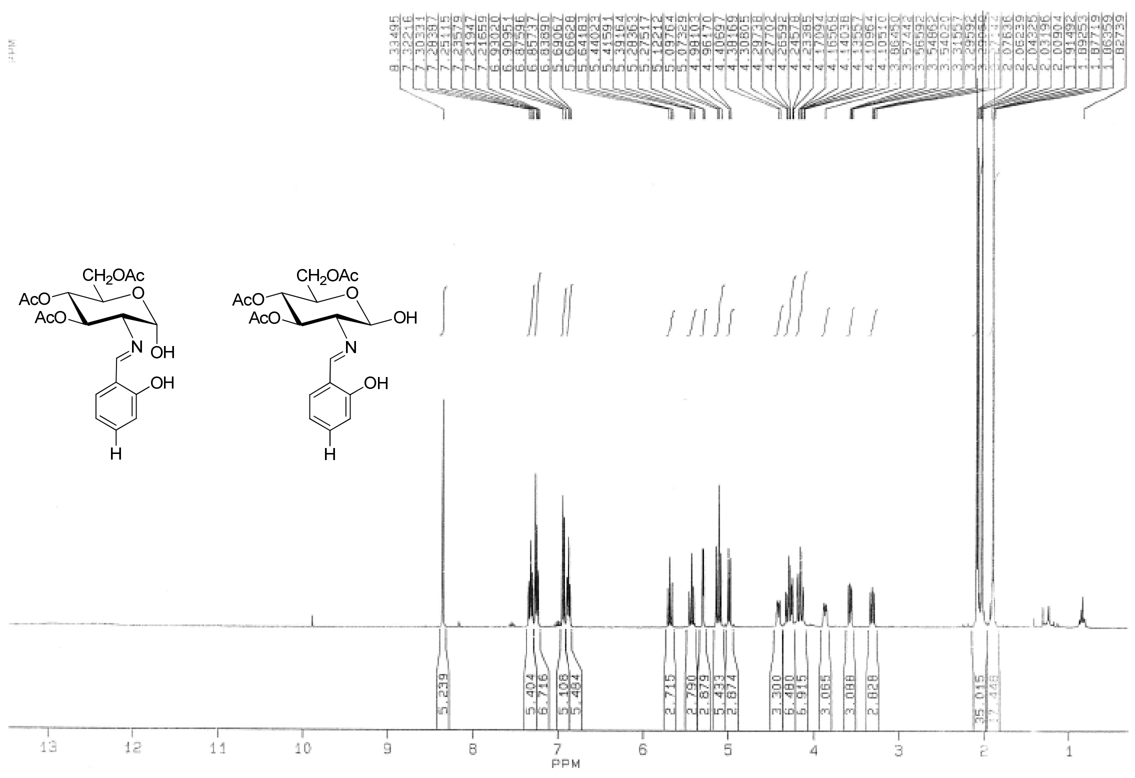

Figure S167. *^{1}H NMR spectrum of **52** and **53** (400 MHz, CDCl$_3$)*

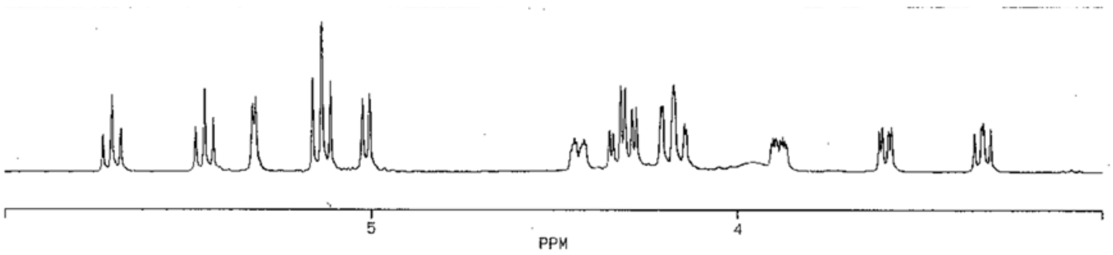

Figure S168. ^{1}H NMR spectrum (magnified zone) of **52/53** (α and β mixture) (400 MHz, $CDCl_3$)

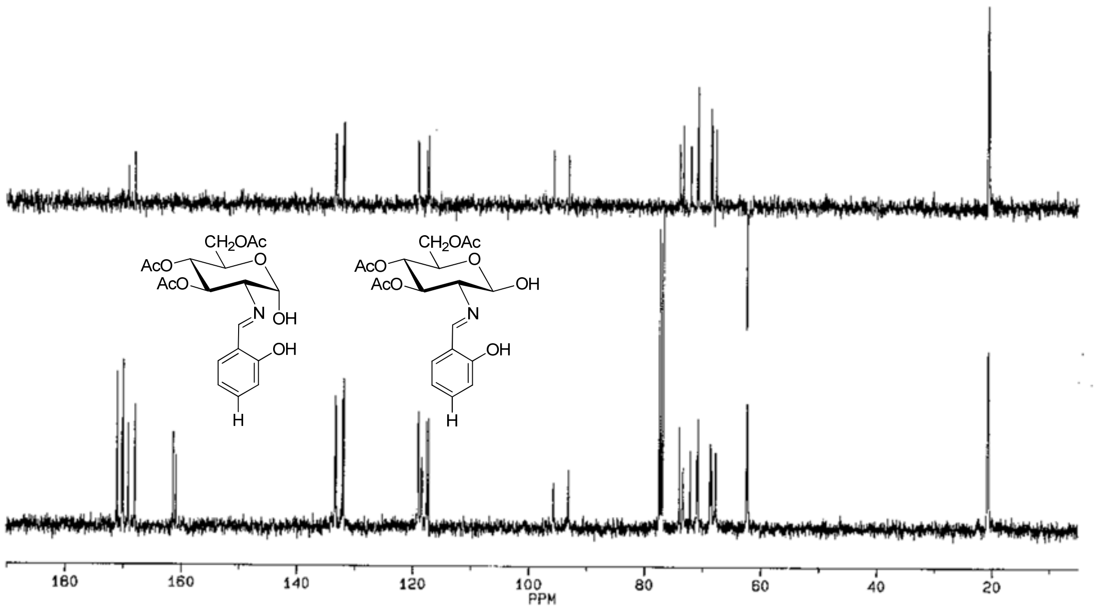

Figure S169. ^{13}C NMR and DEPT spectra of **52/53** (α and β mixture) (100 MHz, $CDCl_3$)

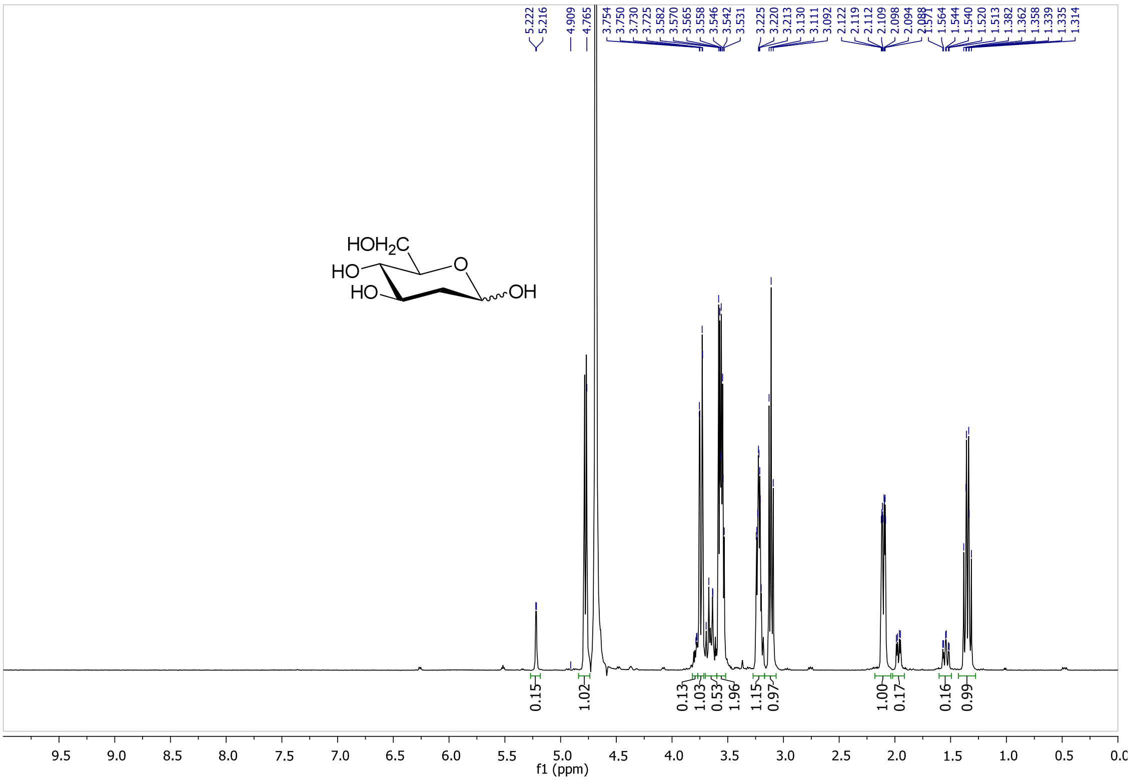

Figure S170. ^1H NMR spectrum of **74** (500 MHz, D_2O)

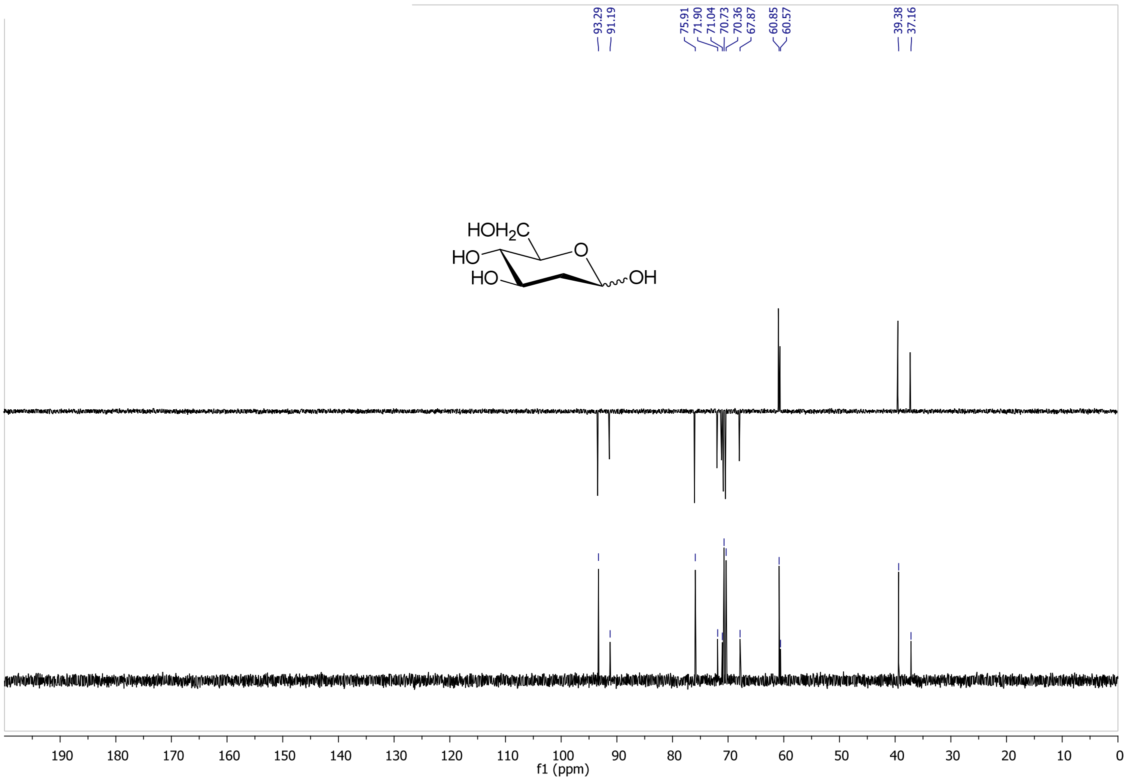

Figure S171. $^{13}C\{^1H\}$ NMR (top: DEPT) spectra of **74** (125 MHz, D_2O)

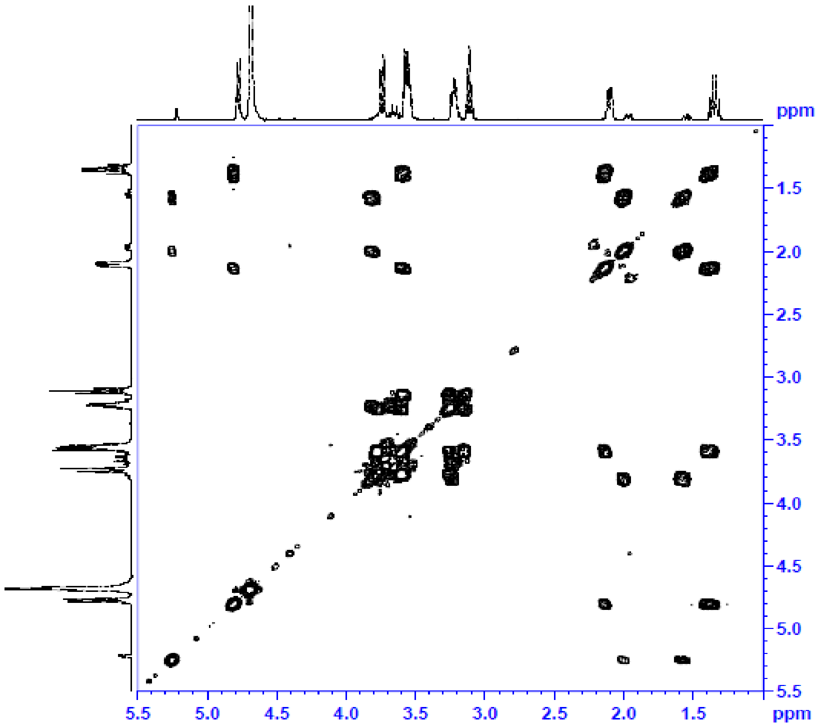

Figure S172. *COSY spectrum of* ***49*** *(D_2O)*

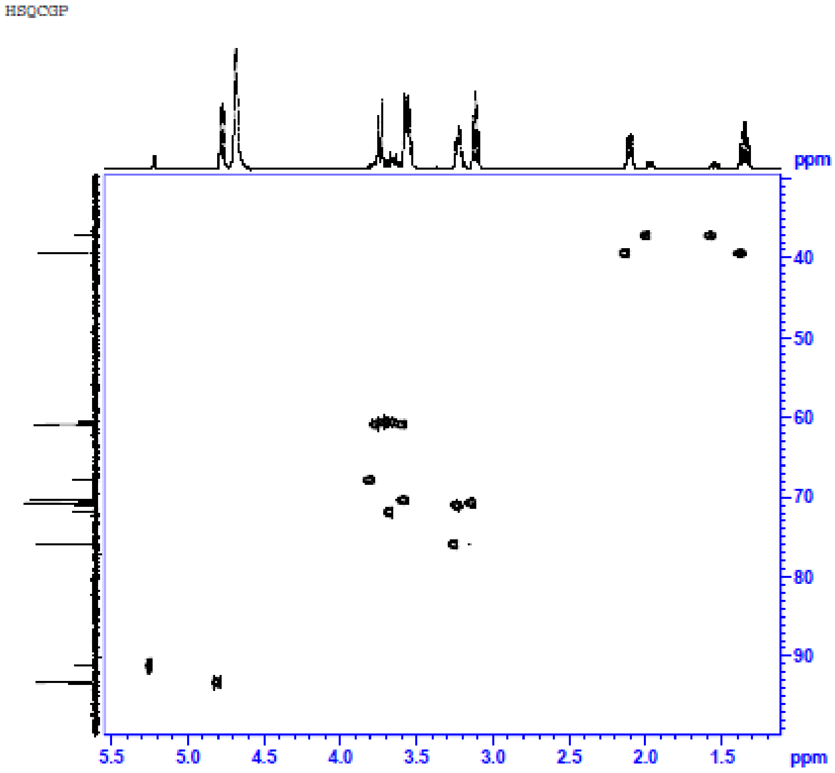

Figure S173. *HSQC spectrum of* ***74*** *(D_2O)*

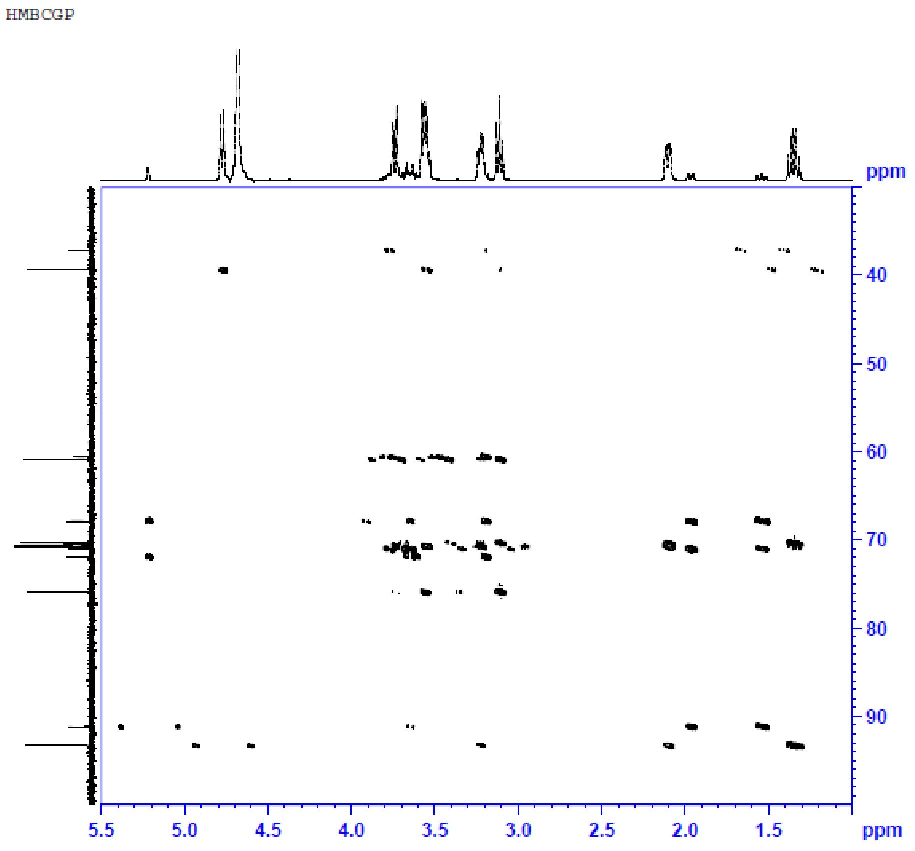

Figure S174. *HMBC spectrum of **74** (D_2O)*

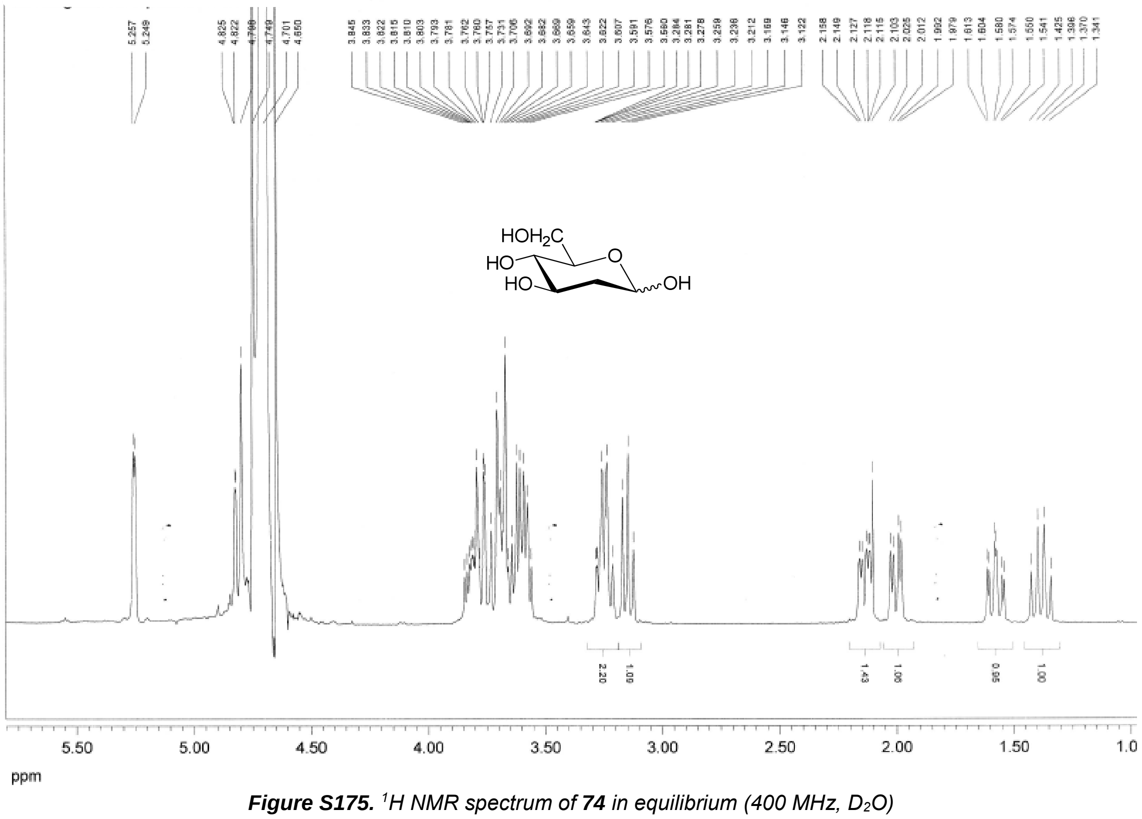

Figure S175. *^{1}H NMR spectrum of **74** in equilibrium (400 MHz, D_2O)*

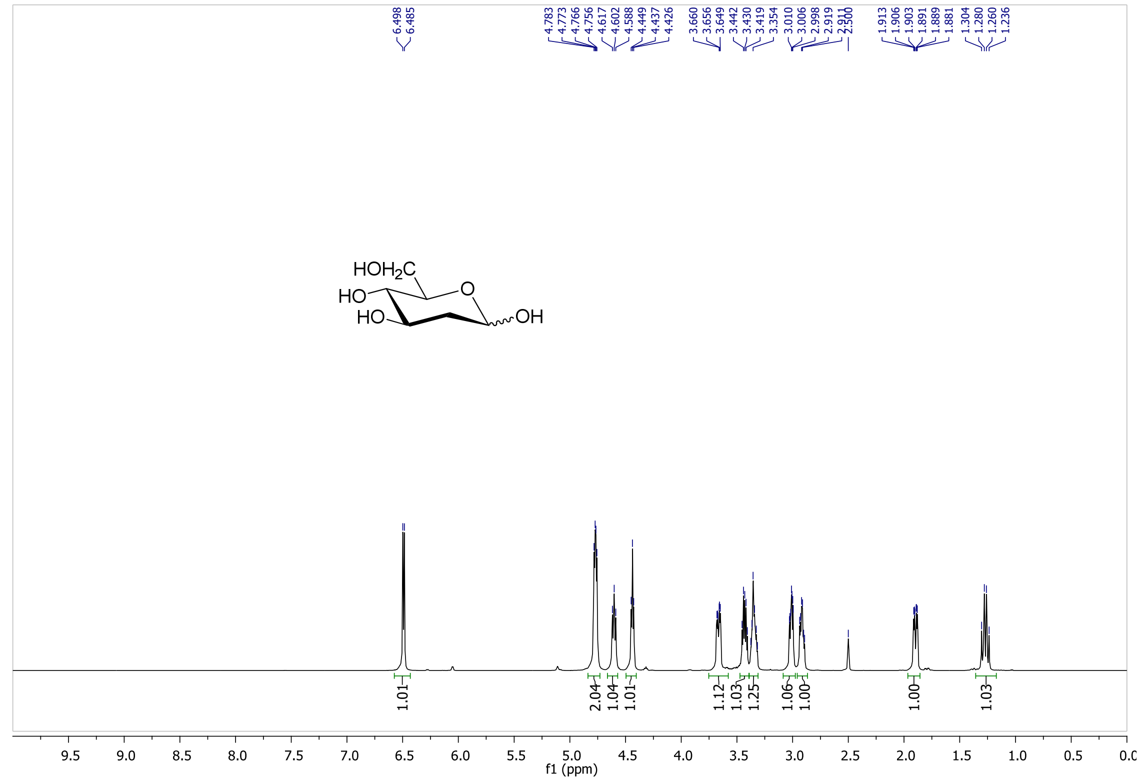

Figure S176. *^{1}H NMR spectrum of **74** (500 MHz, DMSO-d_6)*

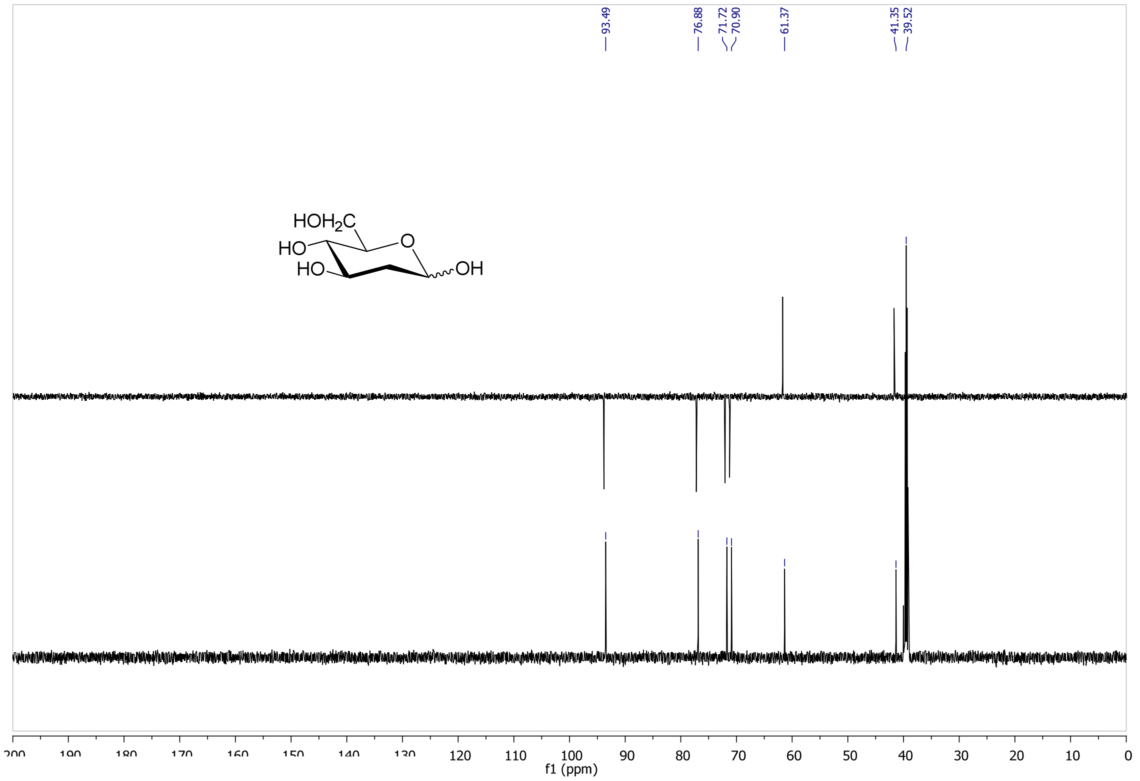

Figure S177. *$^{13}C\{^{1}H\}$ NMR (top: DEPT) spectra of **74** (125 MHz, DMSO-d_6)*

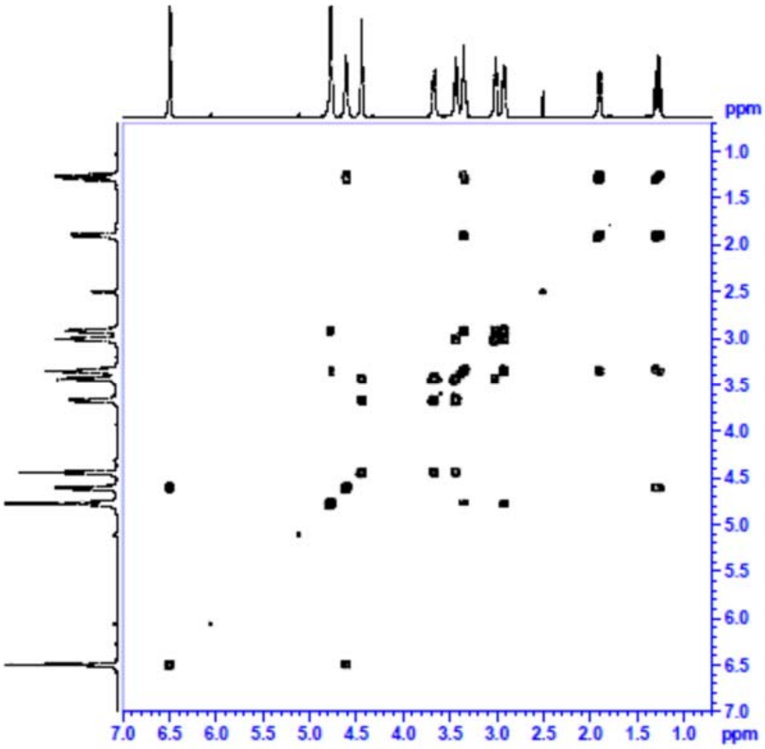

Figure S178. *COSY spectrum of **74** (DMSO-d*$_6$*)*

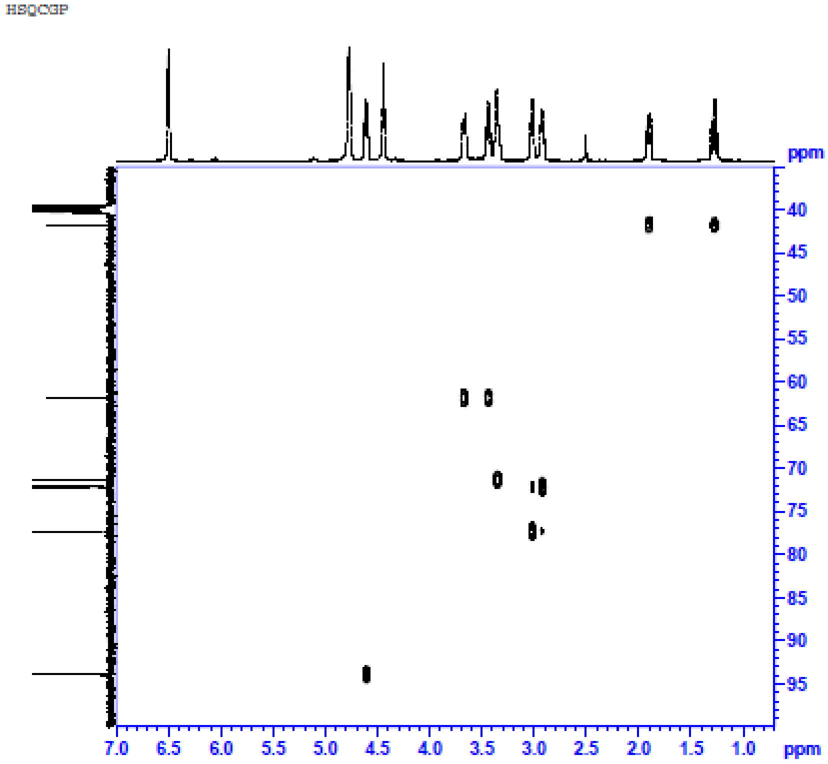

Figure S179. *HSQC spectrum of **74** (DMSO-d*$_6$*)*

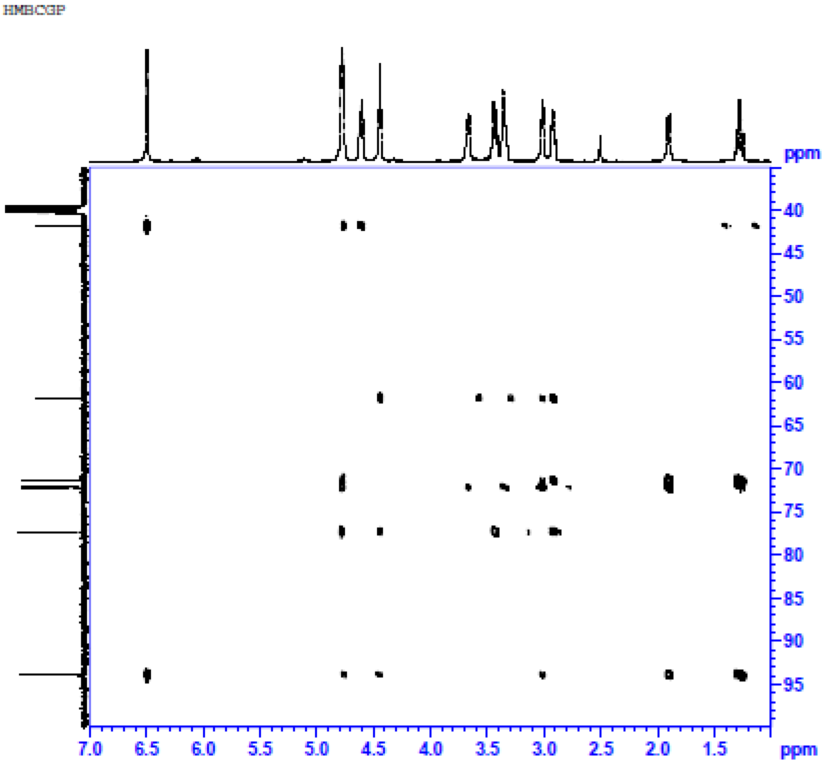

Figure S180. *HMBC spectrum of* ***74*** *(DMSO-d6)*

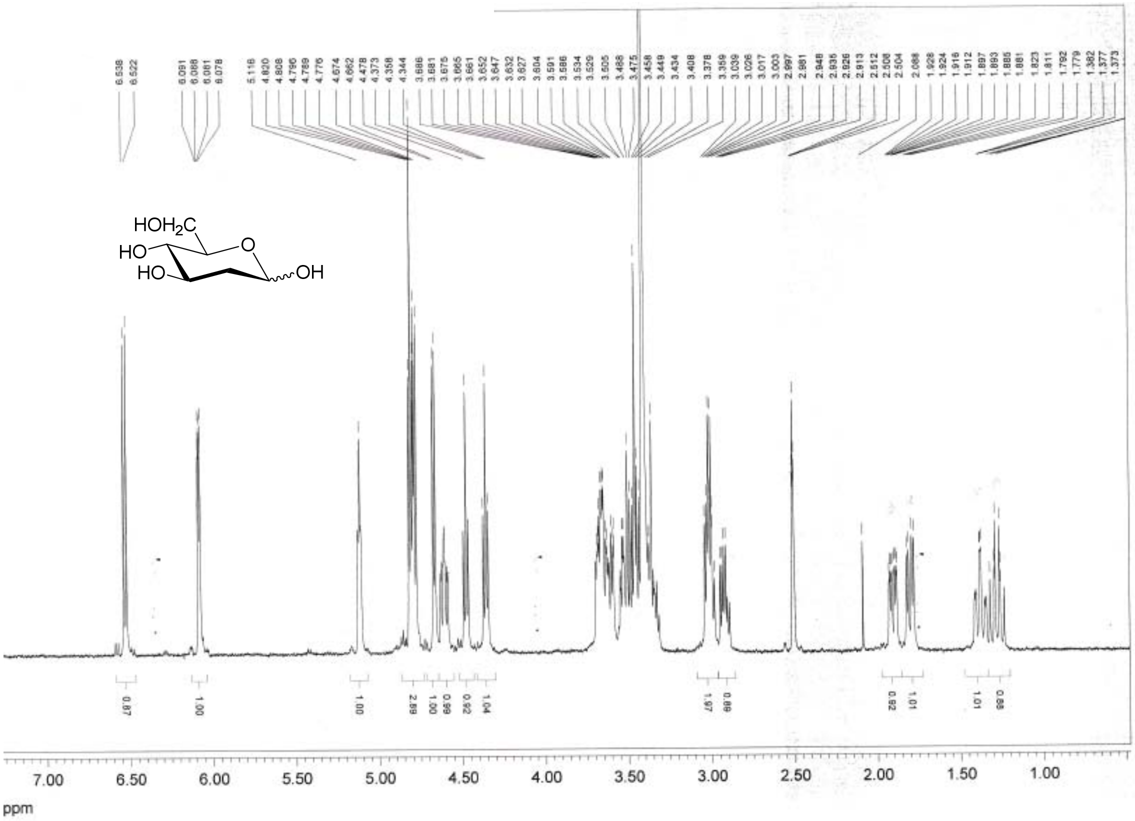

Figure S181. *^{1}H NMR spectrum of* ***74*** *in equilibrium (400 MHz, DMSO-d6)*

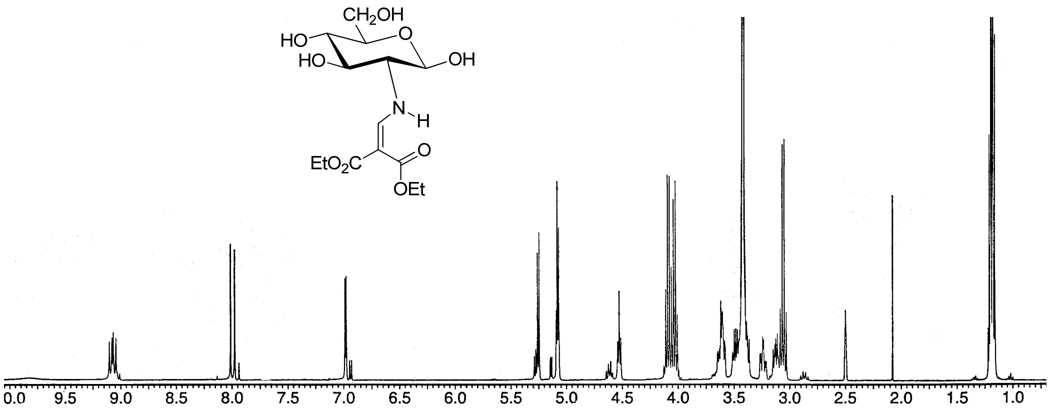

Figure S182. *^{1}H NMR spectrum of* ***5*** *in equilibrium (400 MHz, DMSO-d6)*

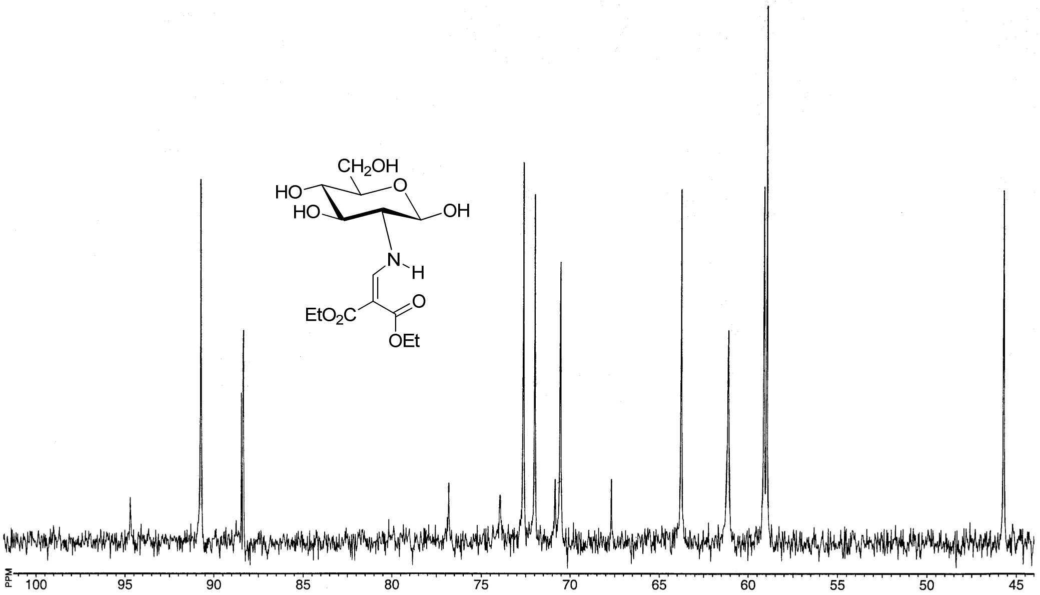

Figure S183. *^{13}C NMR spectrum (magnified zone) of* ***5*** *in equilibrium (400 MHz, DMSO-d6)*

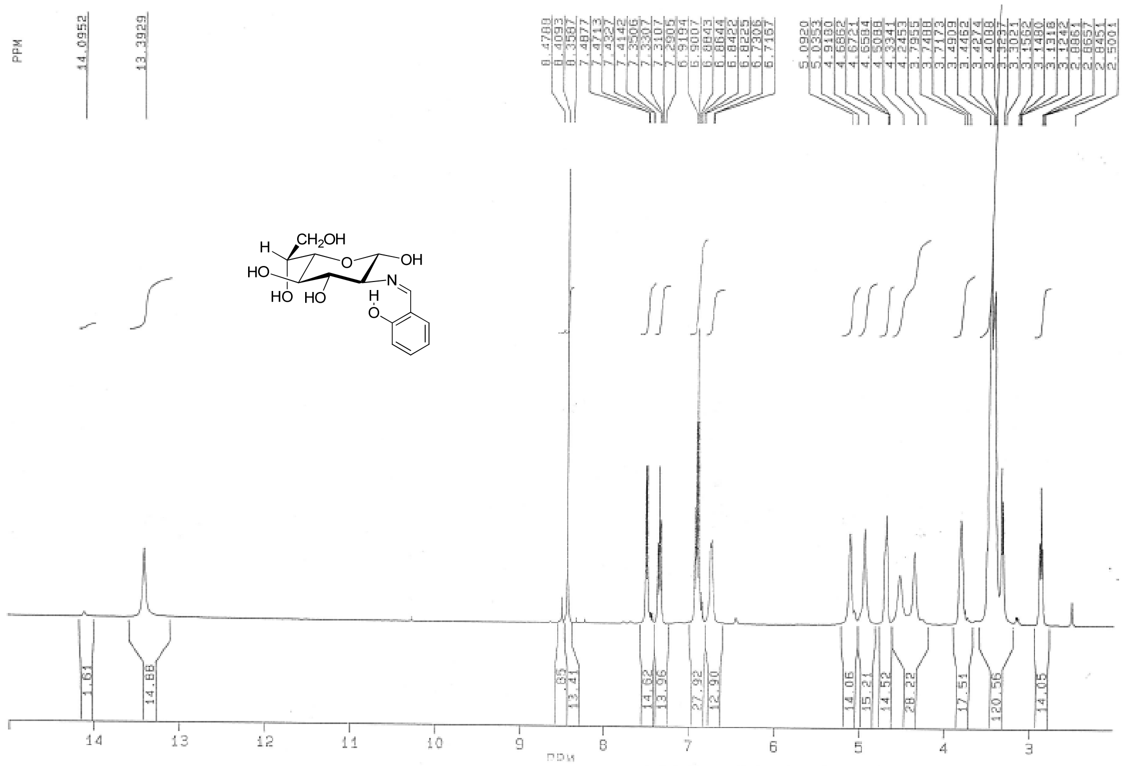

Figure S184. ^{1}H NMR spectrum of **91** (400 MHz, DMSO-d_6)

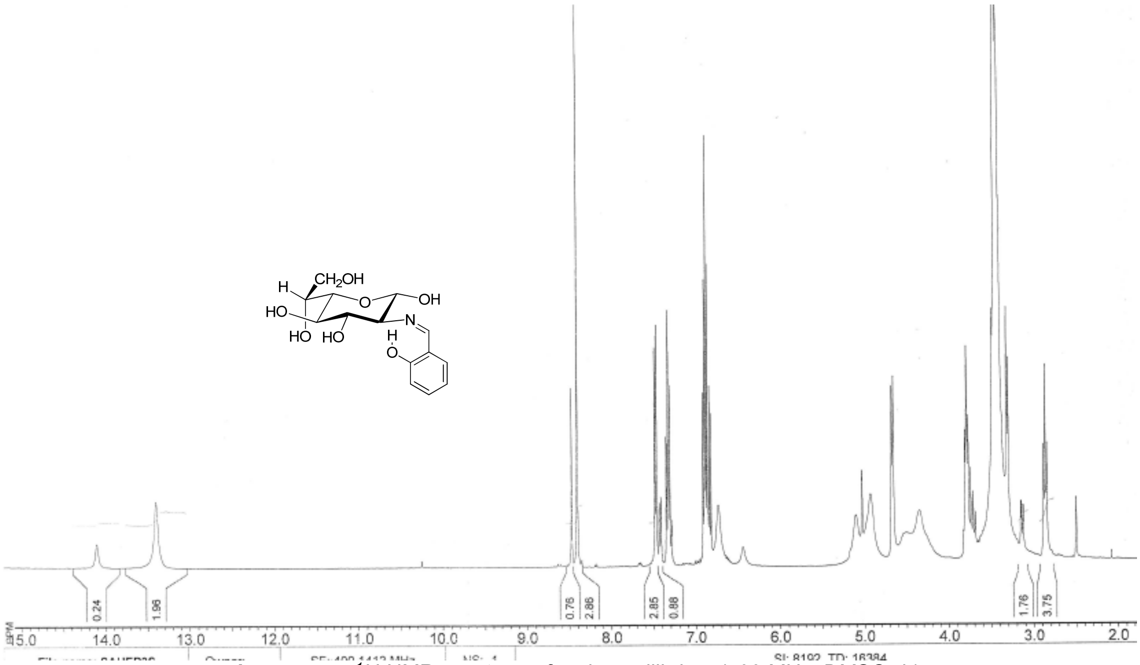

Figure S185. ^{1}H NMR spectrum of **91** in equilibrium (400 MHz, DMSO-d_6)

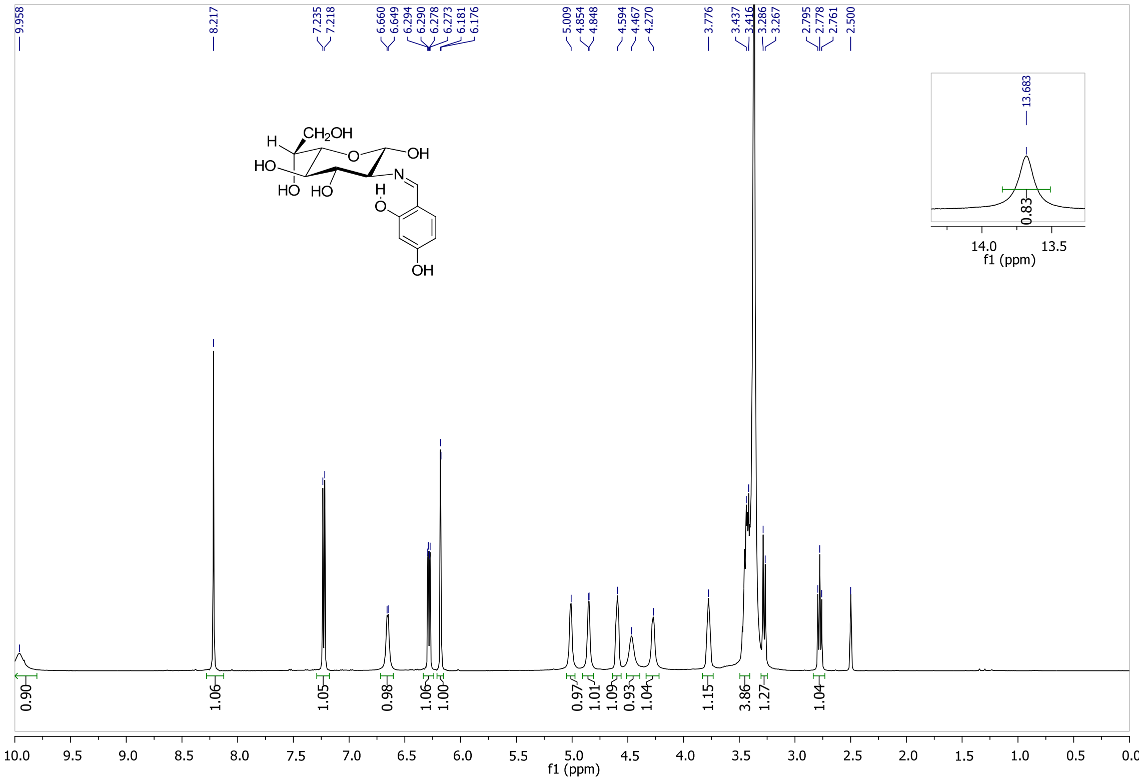

Figure S186. *^{1}H NMR spectrum of **92** (500 MHz, DMSO-d_6)*

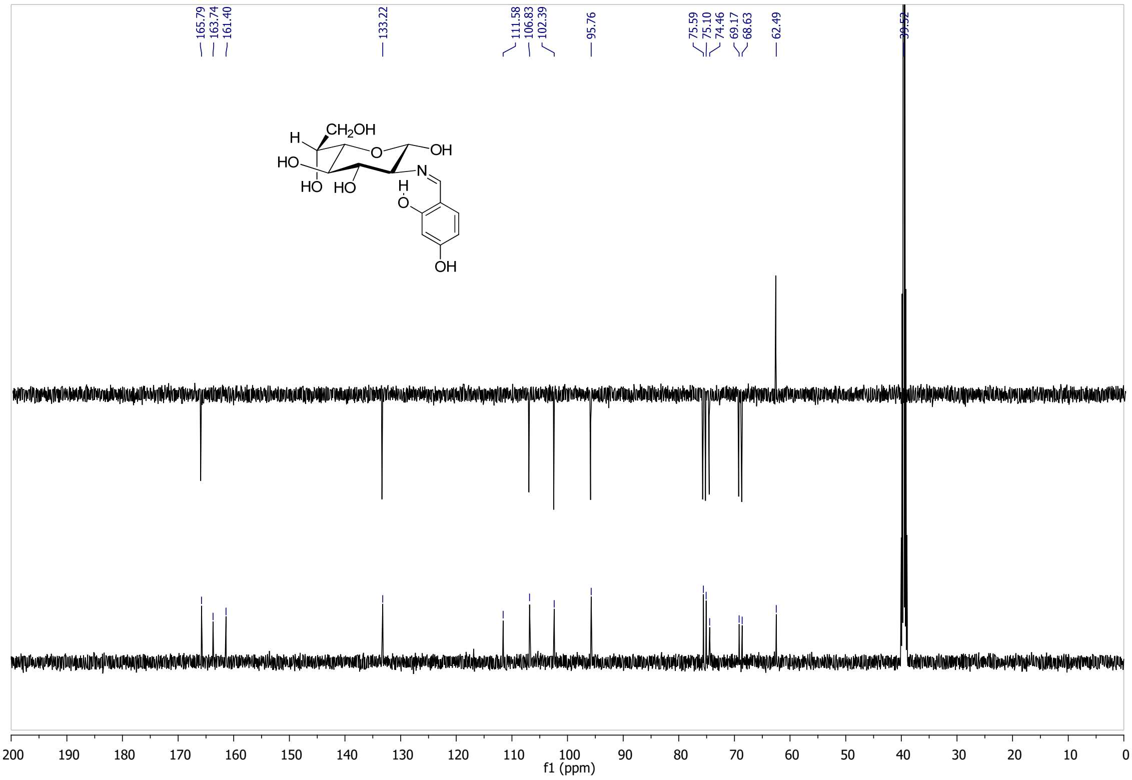

Figure S187. *$^{13}C\{^{1}H\}$ NMR (top: DEPT) spectra of **92** (125 MHz, DMSO-d_6)*

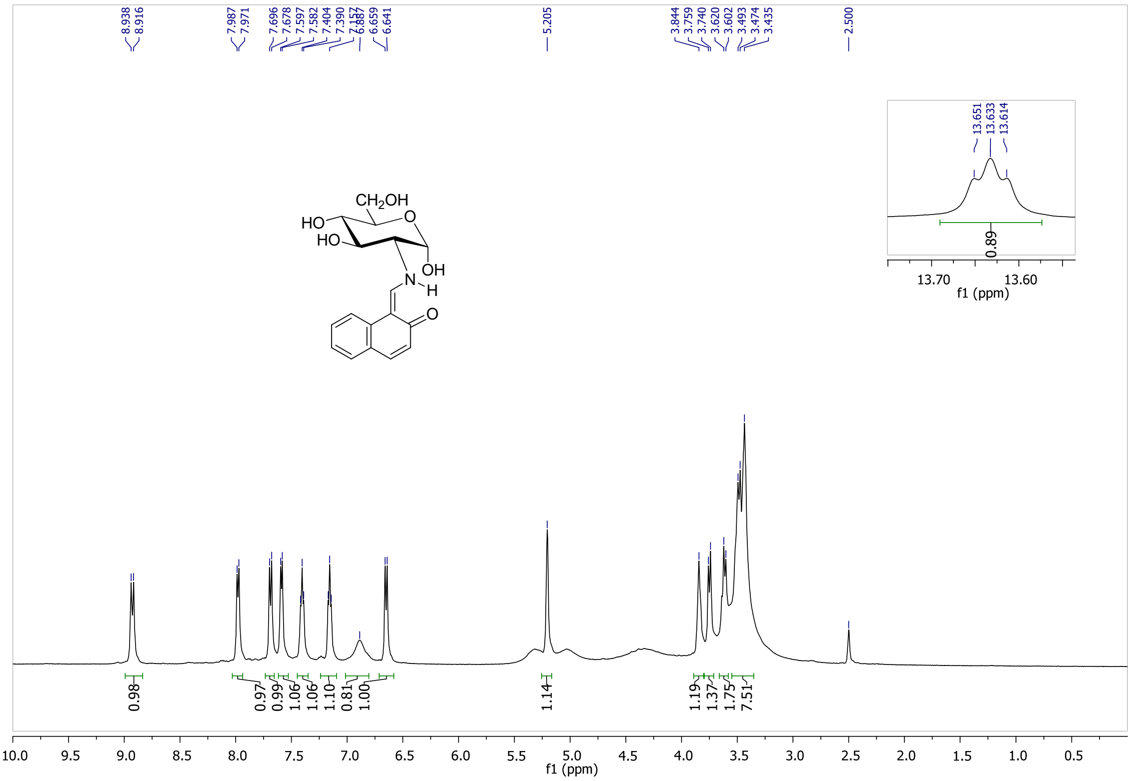

Figure S188. *^{1}H NMR spectrum of **93** (500 MHz, DMSO-d6).*

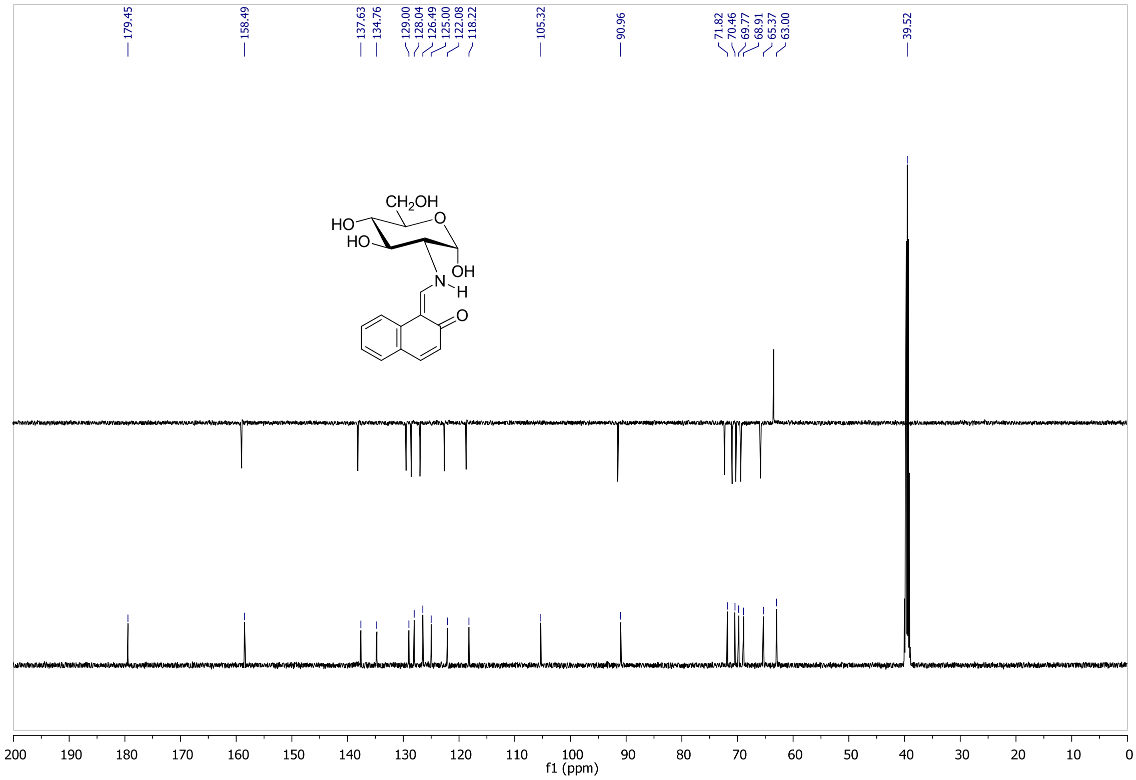

Figure S189. *^{13}C{^{1}H} NMR (top: DEPT) spectra of **93** (125 MHz, DMSO-d6)*

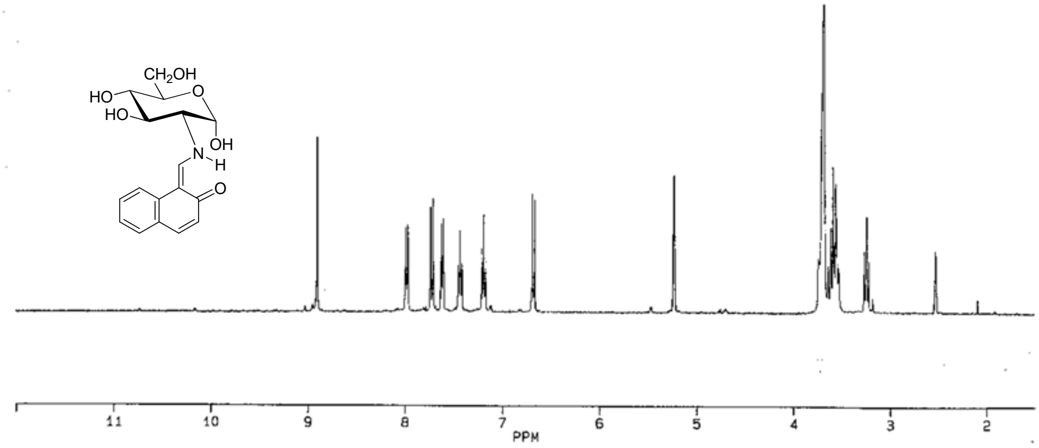

Figure S190. *^{1}H NMR spectrum of **93** exchanged with D2O*

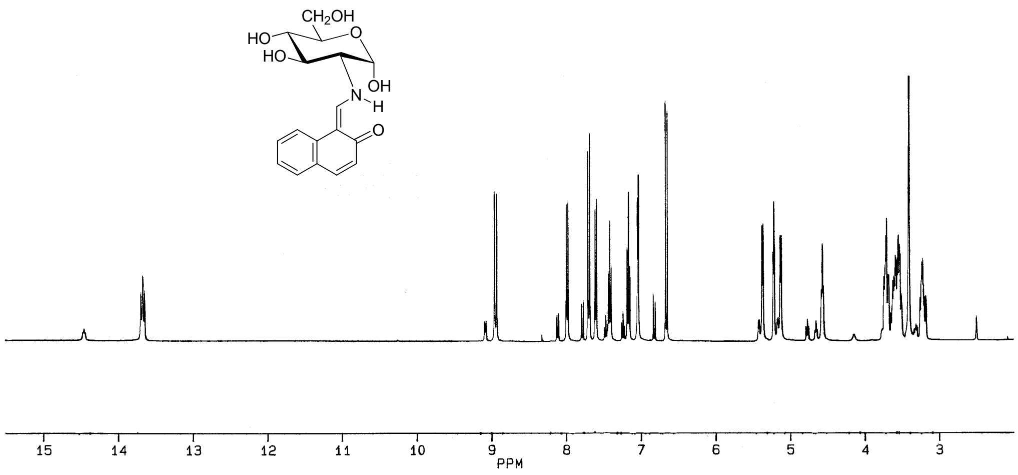

Figure S191. *^{1}H NMR recording of compound **93** in solution after two weeks*

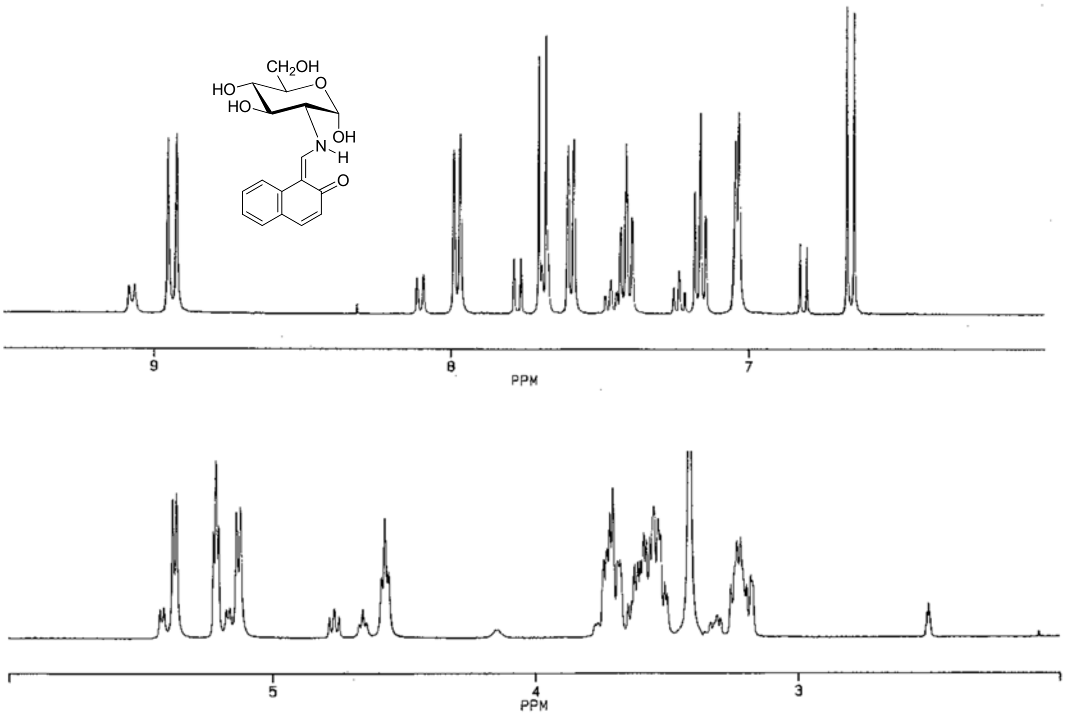

Figure S192. *Magnified* ^{1}*H NMR spectral zones of* ***93*** *after two weeks*

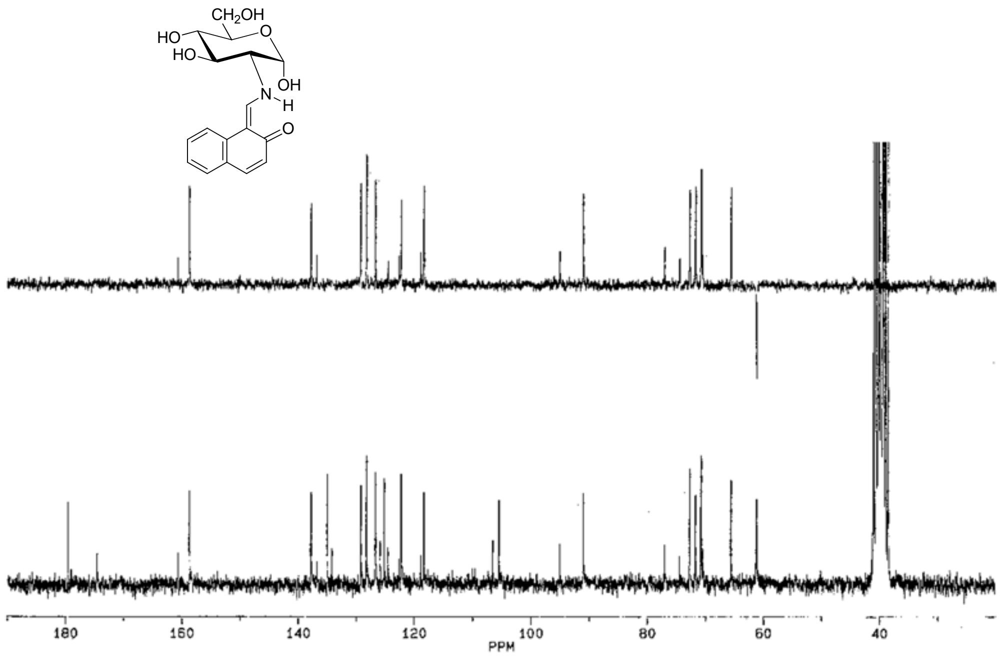

Figure S193. ^{13}*C NMR spectra (top: DEPT) of* ***93*** *after two weeks*

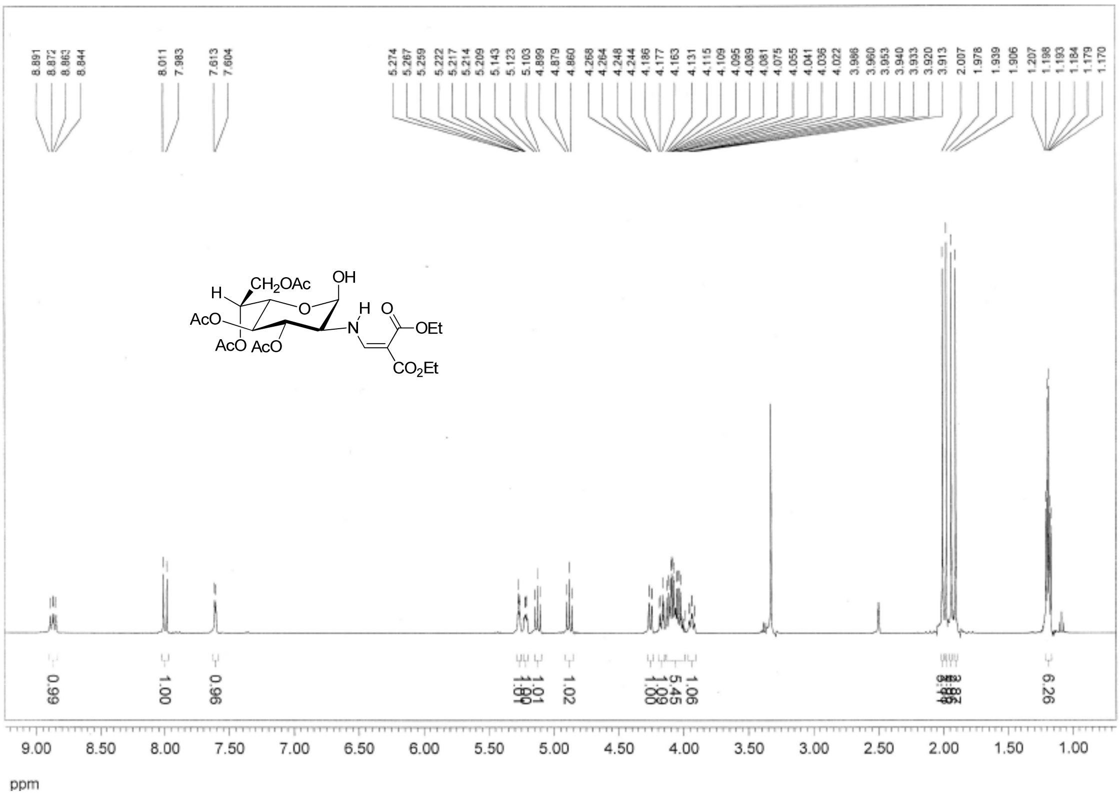

Figure S194. ^1H NMR spectrum of **95** (500 MHz, DMSO-d_6)

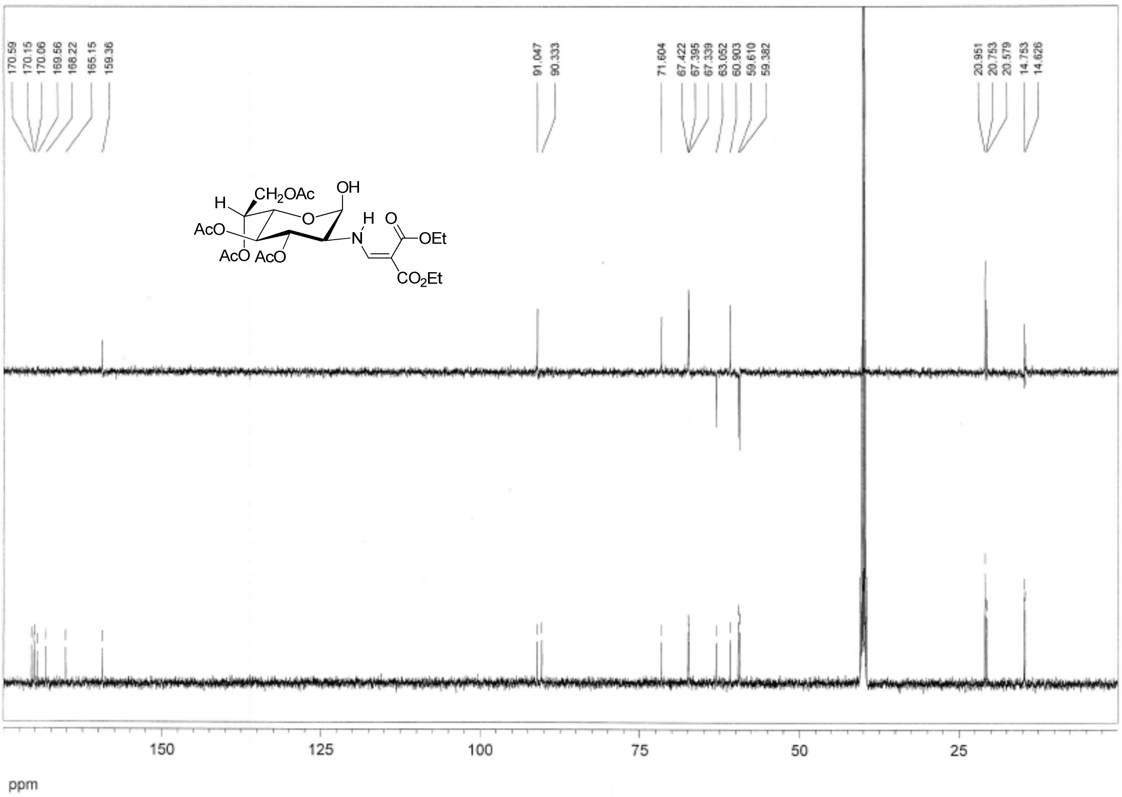

Figure S195. $^{13}C\{^1H\}$ NMR spectrum of **95** (125 MHz, DMSO-d_6)

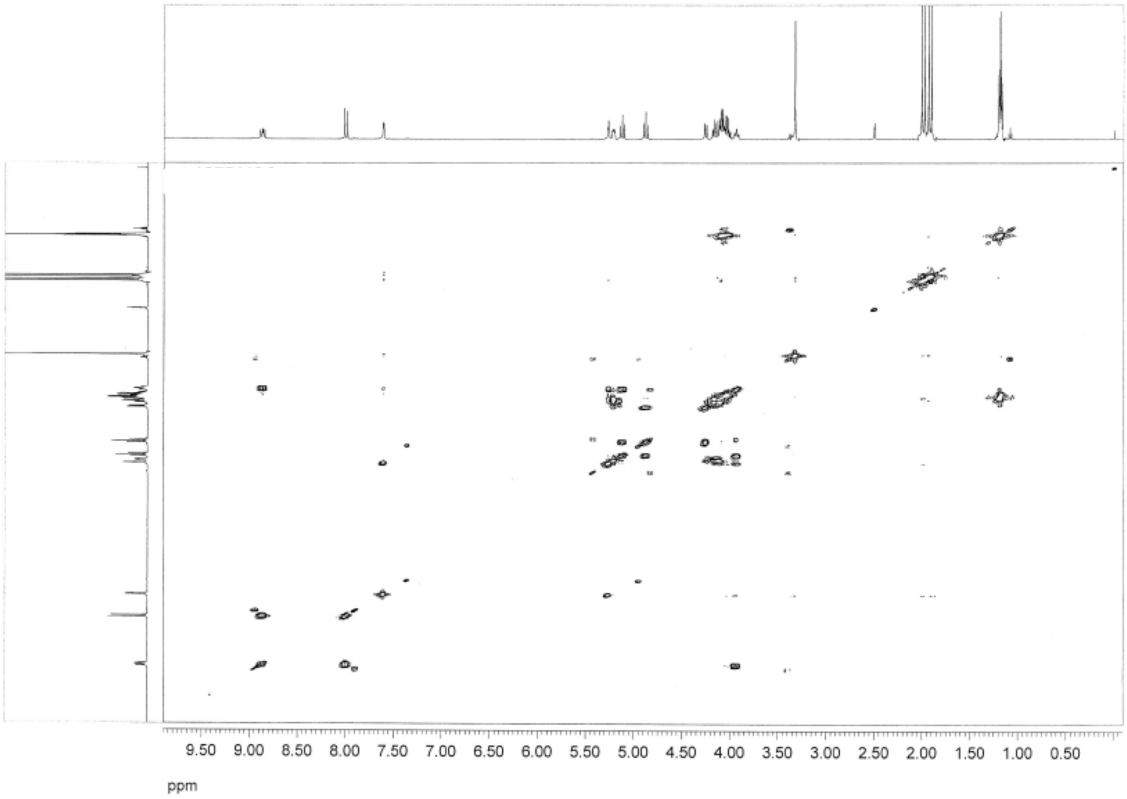

Figure S196. *COSY spectrum of* ***95*** *(DMSO-d$_6$)*

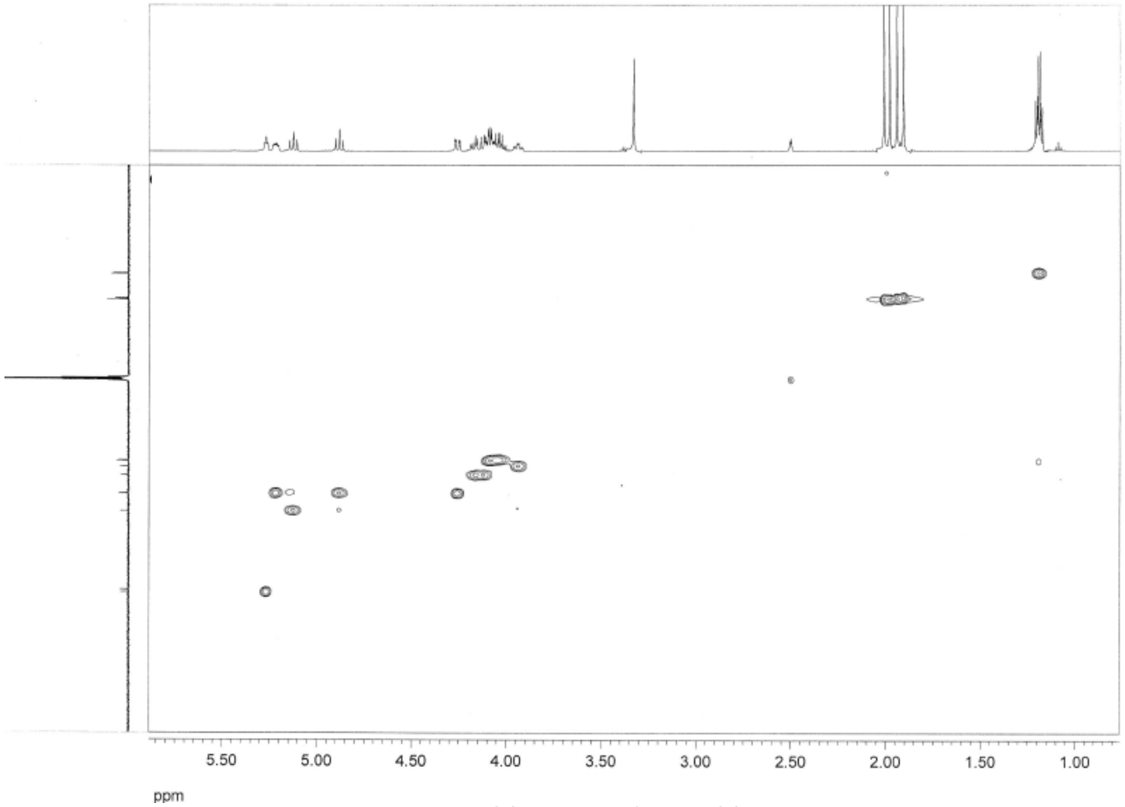

Figure S197. *HMQC spectrum of* ***95*** *(DMSO-d$_6$)*

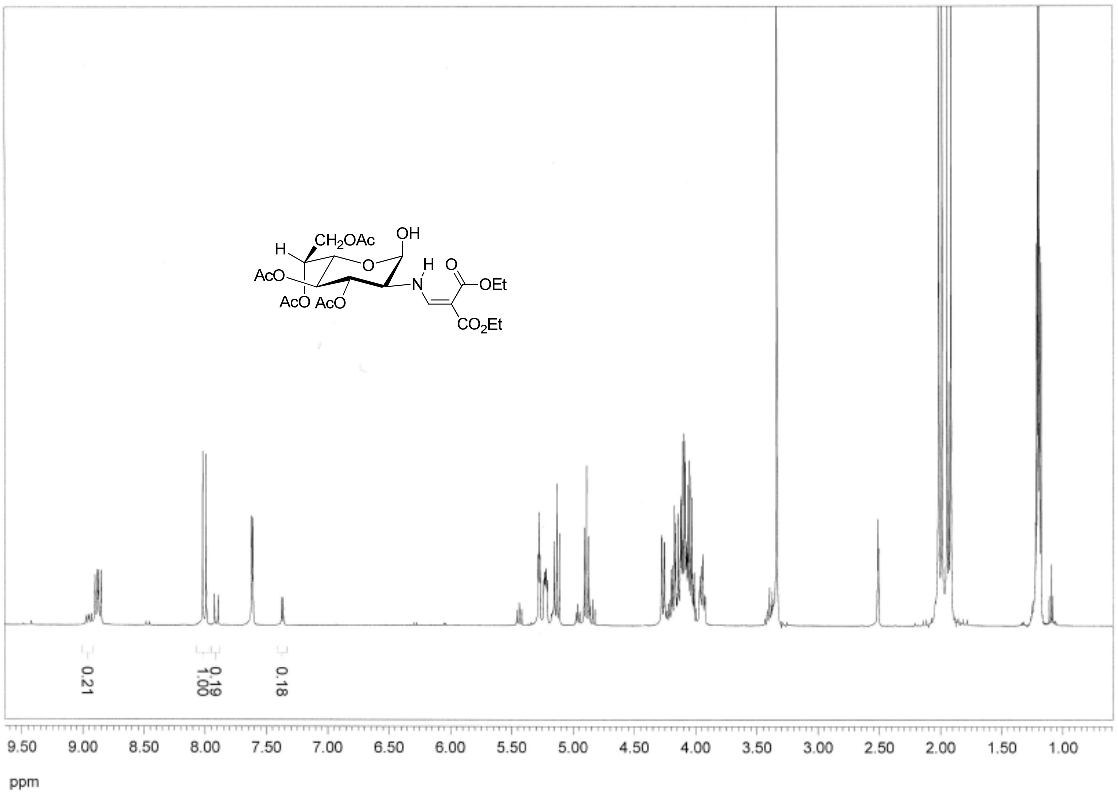

Figure S198. *^{1}H NMR spectrum of **95** in equilibrium (500 MHz, DMSO-d6)*

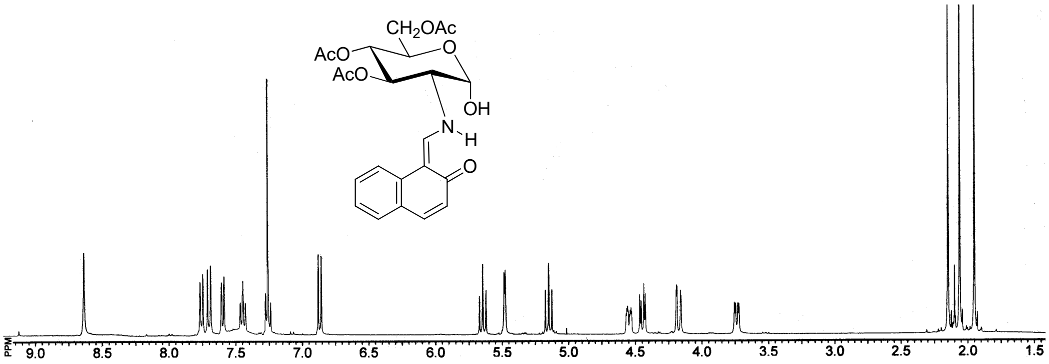

Figure S199. *^{1}H NMR spectrum of **96** (400 MHz, CDCl3)*

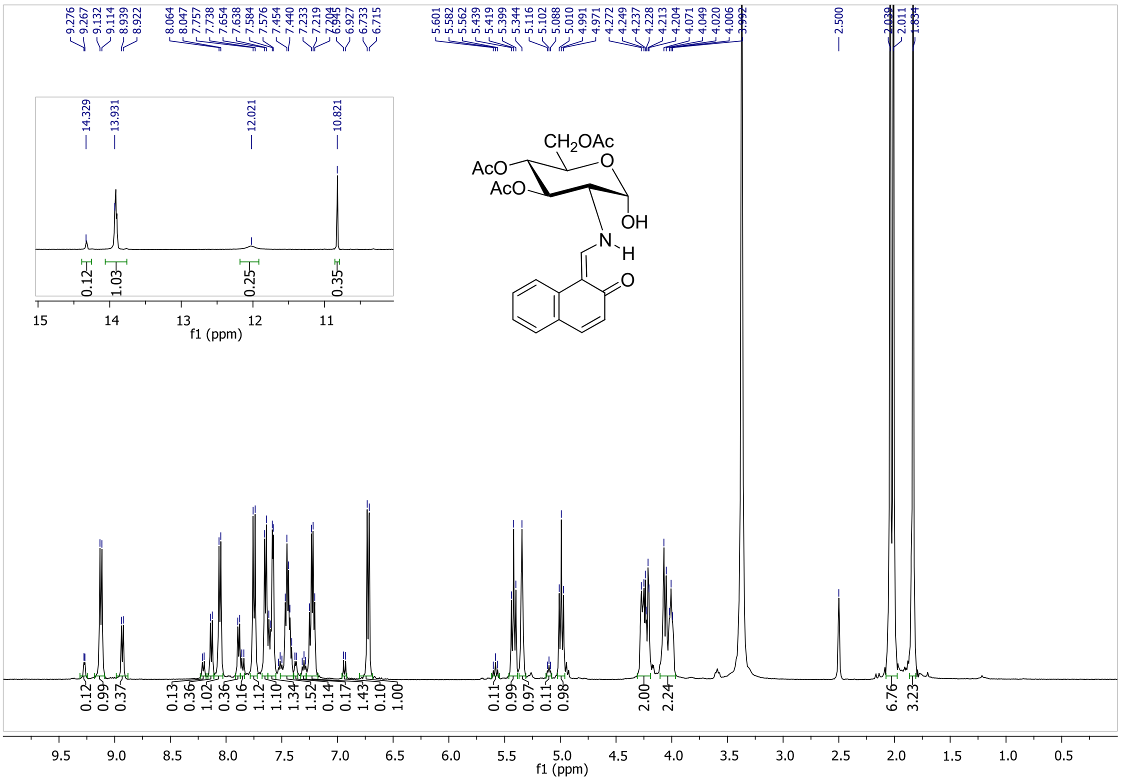

Figure S200. ^1H NMR spectrum of **96** (α and β mixture) (500 MHz, DMSO-d_6)

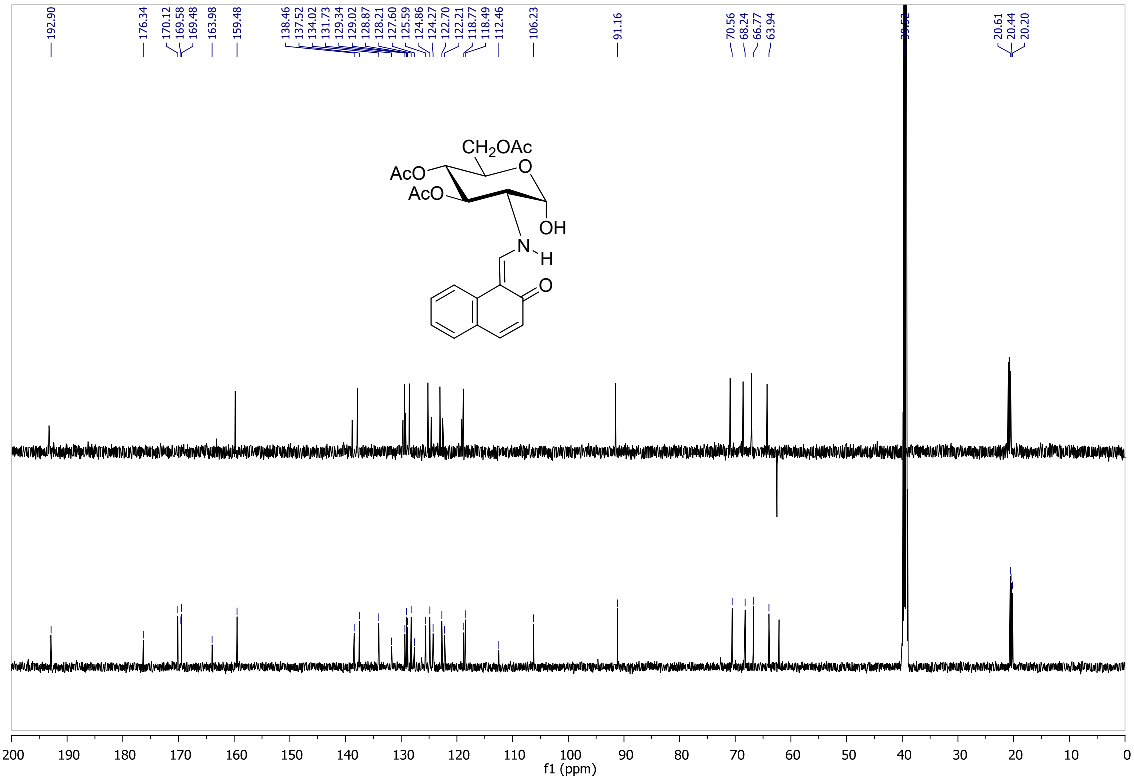

Figure S201. $^{13}C\{^1H\}$ NMR spectrum of **96** (α and β mixture) (125 MHz, DMSO-d_6)

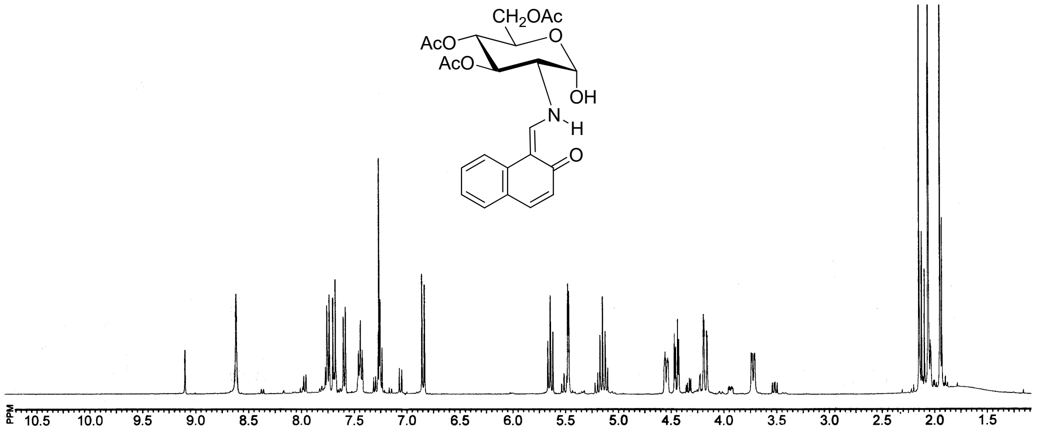

Figure S202. *^{1}H NMR spectrum of **96** in equilibrium (α and β mixture)*

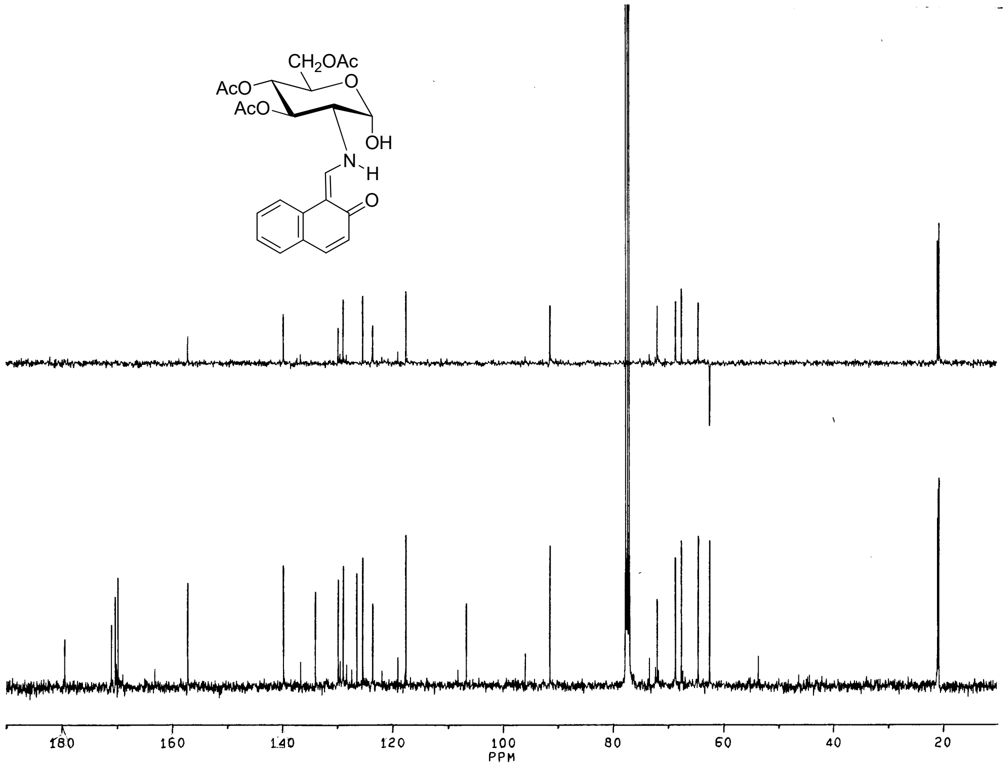

Figure S203. *^{13}C NMR spectra (top: DEPT) of **96** (α and β mixture) in equilibrium*

Structure 11 (B3LYP, Gas Phase)

Energy (Hartrees): = - 936.4725329
No imaginary frequencies

Standard orientation:

Center Number	Atomic Number	Atomic Type	Coordinates (Angstroms) X	Y	Z
1	6	0	1.152454	1.472839	0.243276
2	6	0	0.451362	0.205546	-0.304232
3	6	0	1.250061	-1.024102	0.153227
4	6	0	2.710244	-0.877260	-0.258872
5	6	0	3.283297	0.445082	0.273193
6	1	0	1.134283	1.440479	1.350010
7	1	0	1.203632	-1.089449	1.250961
8	1	0	2.759300	-0.854433	-1.359242
9	1	0	3.272563	0.420588	1.374997
10	1	0	0.485931	0.266522	-1.403913
11	8	0	2.482273	1.522408	-0.212044
12	6	0	4.722742	0.697658	-0.186039
13	1	0	4.764557	0.625429	-1.285800
14	1	0	4.996564	1.721558	0.084941
15	8	0	5.642312	-0.169805	0.446438
16	1	0	5.298276	-1.070423	0.338122
17	8	0	3.486964	-1.958060	0.249969
18	1	0	3.022322	-2.768868	-0.004932
19	8	0	0.781216	-2.236744	-0.430897
20	1	0	-0.088048	-2.427170	-0.053609
21	8	0	0.556327	2.644042	-0.236447
22	1	0	-0.361428	2.632635	0.068924
23	7	0	-0.902077	0.136076	0.208618
24	6	0	-3.275896	-0.026645	-0.231632
25	6	0	-3.658002	-0.011441	1.120991
26	6	0	-4.264926	-0.113526	-1.222475
27	6	0	-5.002536	-0.080931	1.467566
28	1	0	-2.884846	0.055980	1.879088
29	6	0	-5.612778	-0.183324	-0.872819
30	1	0	-3.972892	-0.124867	-2.269704
31	6	0	-5.982963	-0.166925	0.472404
32	1	0	-5.292608	-0.068026	2.514030
33	1	0	-6.371184	-0.249511	-1.646920
34	1	0	-7.032148	-0.220673	0.747637
35	6	0	-1.862618	0.048927	-0.631899
36	1	0	-1.675104	0.027598	-1.717196

Structure 11 (B3LYP, DMSO)

Energy (Hartrees): = - 936.5003158
No imaginary frequencies

Standard orientation:

Center Number	Atomic Number	Atomic Type	Coordinates (Angstroms) X	Y	Z
1	6	0	1.144986	1.486731	0.239315
2	6	0	0.442177	0.238344	-0.320607
3	6	0	1.235135	-1.009644	0.111766
4	6	0	2.705914	-0.875695	-0.263108
5	6	0	3.285177	0.426918	0.304255
6	1	0	1.118923	1.465824	1.342362
7	1	0	1.163370	-1.106243	1.205067
8	1	0	2.787915	-0.842231	-1.360296
9	1	0	3.245143	0.388047	1.404588
10	1	0	0.464864	0.312583	-1.419140
11	8	0	2.508299	1.519375	-0.188061
12	6	0	4.733084	0.670455	-0.128742
13	1	0	4.781135	0.661477	-1.230123
14	1	0	5.038547	1.667802	0.204725
15	8	0	5.633160	-0.264174	0.448002
16	1	0	5.231124	-1.139341	0.316539
17	8	0	3.459045	-1.975876	0.254146
18	1	0	3.016454	-2.780998	-0.057003
19	8	0	0.761148	-2.196449	-0.520425
20	1	0	-0.107226	-2.401053	-0.142413
21	8	0	0.510741	2.614861	-0.268095
22	1	0	0.899264	3.388352	0.169310
23	7	0	-0.907514	0.145197	0.204986
24	6	0	-3.282824	-0.012111	-0.221314
25	6	0	-3.661700	-0.041620	1.132612

26	6	0	-4.275753	-0.077818	-1.211245
27	6	0	-5.005058	-0.134759	1.482532
28	1	0	-2.892843	0.010586	1.896895
29	6	0	-5.622450	-0.170410	-0.858504
30	1	0	-3.986480	-0.054526	-2.258856
31	6	0	-5.989138	-0.198703	0.488373
32	1	0	-5.290609	-0.156453	2.530343
33	1	0	-6.382968	-0.219591	-1.632307
34	1	0	-7.036985	-0.270155	0.765555
35	6	0	-1.872412	0.090693	-0.631635
36	1	0	-1.699017	0.123344	-1.716876

Structure 11 (B3LYP, H_2O)

Energy (Hartrees): = - 936.5086838
No imaginary frequencies

Standard orientation:

Center Number	Atomic Number	Atomic Type	Coordinates (Angstroms) X	Y	Z
1	6	0	1.231705	1.557810	0.234494
2	6	0	0.448039	0.341937	-0.272734
3	6	0	1.170964	-0.947936	0.167871
4	6	0	2.645487	-0.911452	-0.224922
5	6	0	3.310002	0.376754	0.276419
6	1	0	1.239823	1.583438	1.334729
7	1	0	1.108627	-1.035414	1.260891
8	1	0	2.719267	-0.936813	-1.321559
9	1	0	3.297271	0.384655	1.377255
10	1	0	0.461038	0.382123	-1.372547
11	8	0	2.582819	1.496809	-0.235902
12	6	0	4.754205	0.526564	-0.206298
13	1	0	4.772933	0.466381	-1.304968
14	1	0	5.122730	1.515969	0.081044
15	8	0	5.620252	-0.438035	0.385162
16	1	0	5.171591	-1.296245	0.284962
17	8	0	3.348280	-2.023162	0.341952
18	1	0	2.955191	-2.826639	-0.031358
19	8	0	0.605459	-2.102889	-0.451259
20	1	0	-0.239125	-2.289970	-0.016126
21	8	0	0.654586	2.717359	-0.298236
22	1	0	1.043931	3.476449	0.161849
23	7	0	-0.904363	0.337593	0.262838
24	6	0	-3.265796	-0.007131	-0.196171
25	6	0	-3.707455	0.071509	1.136747
26	6	0	-4.210223	-0.205461	-1.216434
27	6	0	-5.062426	-0.039441	1.434205
28	1	0	-2.984015	0.214326	1.931885
29	6	0	-5.567712	-0.317375	-0.916571
30	1	0	-3.872371	-0.271202	-2.247250
31	6	0	-5.996175	-0.234044	0.409525
32	1	0	-5.395218	0.021869	2.466006
33	1	0	-6.287721	-0.470168	-1.714713
34	1	0	-7.052207	-0.322086	0.646703
35	6	0	-1.847245	0.100736	-0.570944
36	1	0	-1.642612	-0.035308	-1.641530

Structure 11 (M06-2X, Gas Phase)

Energy (Hartrees): = - 936.3468146
No imaginary frequencies

Standard orientation:

Center Number	Atomic Number	Atomic Type	Coordinates (Angstroms) X	Y	Z
1	6	0	1.033456	1.340609	0.371620
2	6	0	0.443288	0.127985	-0.350309
3	6	0	1.316650	-1.087389	-0.065326
4	6	0	2.760298	-0.795476	-0.411935
5	6	0	3.223194	0.440192	0.355408
6	1	0	0.983018	1.187589	1.462523
7	1	0	1.258030	-1.316463	1.008427
8	1	0	2.830712	-0.582489	-1.488345
9	1	0	3.155675	0.236745	1.435084
10	1	0	0.472880	0.360351	-1.424455
11	8	0	2.383405	1.525729	-0.004420
12	6	0	4.659690	0.830348	0.034558
13	1	0	4.778294	0.888637	-1.056447
14	1	0	4.845061	1.823251	0.447404
15	8	0	5.582517	-0.053664	0.623418
16	1	0	5.322342	-0.946369	0.369629

17	8	0	3.590413	-1.889664	-0.069073
18	1	0	3.175486	-2.679675	-0.432313
19	8	0	0.925371	-2.214102	-0.825169
20	1	0	0.024369	-2.436849	-0.570767
21	8	0	0.315767	2.454540	-0.026217
22	1	0	0.697017	3.223396	0.407319
23	7	0	-0.897773	-0.148096	0.121821
24	6	0	-3.271552	0.076370	-0.192337
25	6	0	-3.605969	-0.580956	0.994883
26	6	0	-4.283754	0.543031	-1.029638
27	6	0	-4.936694	-0.768719	1.332055
28	1	0	-2.806096	-0.931890	1.635469
29	6	0	-5.618815	0.354416	-0.690410
30	1	0	-4.022553	1.057166	-1.948812
31	6	0	-5.945449	-0.302219	0.489872
32	1	0	-5.194153	-1.278819	2.252511
33	1	0	-6.400879	0.719365	-1.344965
34	1	0	-6.985028	-0.451070	0.756525
35	6	0	-1.863330	0.280254	-0.577871
36	1	0	-1.695634	0.830413	-1.513259

Structure 11 (M06-2X, DMSO)

Energy (Hartrees): = - 936.3730451
No imaginary frequencies

Standard orientation:

Center Number	Atomic Number	Atomic Type	Coordinates (Angstroms) X	Y	Z
1	6	0	1.148351	1.485583	0.194567
2	6	0	0.447767	0.239314	-0.342539
3	6	0	1.222645	-1.000315	0.107543
4	6	0	2.681860	-0.875748	-0.279180
5	6	0	3.255857	0.420855	0.285866
6	1	0	1.121456	1.483468	1.295246
7	1	0	1.157865	-1.082496	1.200772
8	1	0	2.758305	-0.843450	-1.374928
9	1	0	3.190217	0.392616	1.383784
10	1	0	0.464100	0.295226	-1.440304
11	8	0	2.501603	1.506057	-0.229529
12	6	0	4.707759	0.639848	-0.113875
13	1	0	4.797211	0.542200	-1.204514
14	1	0	4.995094	1.656697	0.160441
15	8	0	5.579143	-0.244852	0.562321
16	1	0	5.209012	-1.129157	0.452074
17	8	0	3.431095	-1.966776	0.234596
18	1	0	2.980808	-2.773972	-0.042365
19	8	0	0.736586	-2.180909	-0.505997
20	1	0	-0.128516	-2.380245	-0.130377
21	8	0	0.514678	2.596428	-0.332039
22	1	0	0.888637	3.378202	0.090886
23	7	0	-0.896113	0.165093	0.189732
24	6	0	-3.262312	-0.020837	-0.216187
25	6	0	-3.624813	0.011730	1.134580
26	6	0	-4.253419	-0.134962	-1.192426
27	6	0	-4.961073	-0.068925	1.497300
28	1	0	-2.850908	0.103386	1.887860
29	6	0	-5.593527	-0.216204	-0.827224
30	1	0	-3.969577	-0.158320	-2.239891
31	6	0	-5.948008	-0.182104	0.517213
32	1	0	-5.239072	-0.042294	2.544753
33	1	0	-6.358280	-0.305406	-1.590053
34	1	0	-6.991407	-0.243261	0.804938
35	6	0	-1.851123	0.070700	-0.638841
36	1	0	-1.676777	0.057955	-1.722036

Structure 11 (M06-2X, H_2O)

Energy (Hartrees): = - 936.3823679
No imaginary frequencies

Standard orientation:

Center Number	Atomic Number	Atomic Type	Coordinates (Angstroms) X	Y	Z
1	6	0	1.243691	1.561380	0.205736
2	6	0	0.453768	0.363245	-0.303726
3	6	0	1.146929	-0.929101	0.140359
4	6	0	2.611280	-0.909119	-0.257723
5	6	0	3.278476	0.357188	0.269457
6	1	0	1.245541	1.588161	1.303592
7	1	0	1.088933	-1.015652	1.232016

8	1	0	2.684400	-0.913759	-1.353180
9	1	0	3.231703	0.359119	1.367913
10	1	0	0.466611	0.402823	-1.401614
11	8	0	2.586407	1.482649	-0.253283
12	6	0	4.731030	0.480064	-0.165608
13	1	0	4.793914	0.353927	-1.253379
14	1	0	5.087869	1.479261	0.088323
15	8	0	5.561348	-0.450732	0.510902
16	1	0	5.151455	-1.318785	0.397920
17	8	0	3.295645	-2.028921	0.287266
18	1	0	2.880446	-2.824113	-0.067597
19	8	0	0.561461	-2.065072	-0.475028
20	1	0	-0.268527	-2.263506	-0.026977
21	8	0	0.681004	2.718413	-0.330770
22	1	0	1.055175	3.476371	0.133432
23	7	0	-0.894201	0.375258	0.230531
24	6	0	-3.243087	-0.008862	-0.198588
25	6	0	-3.653324	0.079404	1.135767
26	6	0	-4.196562	-0.216084	-1.197811
27	6	0	-4.997715	-0.032145	1.458925
28	1	0	-2.913742	0.228281	1.912694
29	6	0	-5.544594	-0.326949	-0.872683
30	1	0	-3.874895	-0.289392	-2.231528
31	6	0	-5.945874	-0.234661	0.455666
32	1	0	-5.311677	0.034318	2.493875
33	1	0	-6.278661	-0.485802	-1.653616
34	1	0	-6.995060	-0.322801	0.712266
35	6	0	-1.825922	0.100010	-0.590919
36	1	0	-1.619571	-0.072159	-1.653668

Structure 11 (M06-2X/def2-TZVP, Gas Phase)

Energy (Hartrees): = -936.464045
No imaginary frequencies

Standard orientation:

Center Number	Atomic Number	Atomic Type	Coordinates (Angstroms) X	Y	Z
1	6	0	1.051054	1.354870	0.361005
2	6	0	0.442268	0.139439	-0.336804
3	6	0	1.302431	-1.079148	-0.031002
4	6	0	2.746948	-0.811150	-0.389281
5	6	0	3.229993	0.434886	0.345476
6	1	0	1.004184	1.220634	1.453155
7	1	0	1.244300	-1.285747	1.046144
8	1	0	2.814654	-0.622223	-1.469185
9	1	0	3.175632	0.255261	1.429290
10	1	0	0.468991	0.347806	-1.415248
11	8	0	2.398148	1.519759	-0.023530
12	6	0	4.661579	0.809243	-0.008202
13	1	0	4.763027	0.842192	-1.100511
14	1	0	4.860084	1.809666	0.377369
15	8	0	5.596633	-0.060844	0.579947
16	1	0	5.335563	-0.964682	0.364636
17	8	0	3.567080	-1.906193	-0.030486
18	1	0	3.145770	-2.705891	-0.367167
19	8	0	0.892452	-2.215047	-0.765197
20	1	0	-0.010022	-2.431980	-0.506113
21	8	0	0.344782	2.472797	-0.045880
22	1	0	0.733792	3.250009	0.368434
23	7	0	-0.896434	-0.105396	0.146588
24	6	0	-3.270796	0.057153	-0.199325
25	6	0	-3.614693	-0.490420	1.036625
26	6	0	-4.274666	0.439052	-1.083759
27	6	0	-4.945645	-0.654031	1.374686
28	1	0	-2.823065	-0.777727	1.716301
29	6	0	-5.610079	0.275271	-0.743834
30	1	0	-4.005273	0.867749	-2.042365
31	6	0	-5.945678	-0.272245	0.485046
32	1	0	-5.210891	-1.078701	2.334332
33	1	0	-6.386015	0.575092	-1.435961
34	1	0	-6.986293	-0.401666	0.753475
35	6	0	-1.863596	0.239387	-0.590547
36	1	0	-1.696889	0.700371	-1.572038

Structure 11 (M06-2X/def2-TZVP, DMSO)

Energy (Hartrees): = -936.490491
No imaginary frequencies

Standard orientation:

Center Number	Atomic Number	Atomic Type	Coordinates (Angstroms) X	Y	Z
1	6	0	1.149463	1.481145	0.201544
2	6	0	0.447430	0.238331	-0.338120
3	6	0	1.220943	-0.999975	0.113309
4	6	0	2.679312	-0.879402	-0.273127
5	6	0	3.256139	0.415782	0.288632
6	1	0	1.119130	1.480146	1.300867
7	1	0	1.154702	-1.078890	1.205777
8	1	0	2.756213	-0.847091	-1.367744
9	1	0	3.198614	0.388000	1.386214
10	1	0	0.465143	0.292691	-1.435181
11	8	0	2.500711	1.500826	-0.218225
12	6	0	4.701417	0.640708	-0.126067
13	1	0	4.781598	0.543647	-1.215984
14	1	0	4.987888	1.657824	0.144568
15	8	0	5.590355	-0.235957	0.535419
16	1	0	5.233393	-1.128588	0.438990
17	8	0	3.429373	-1.969389	0.237877
18	1	0	2.988942	-2.783895	-0.037301
19	8	0	0.726843	-2.178850	-0.493803
20	1	0	-0.154512	-2.359693	-0.143303
21	8	0	0.521767	2.597429	-0.321812
22	1	0	0.875556	3.382035	0.115936
23	7	0	-0.895174	0.164573	0.188915
24	6	0	-3.261542	-0.020671	-0.213092
25	6	0	-3.630271	0.006493	1.133164
26	6	0	-4.246673	-0.128473	-1.192050
27	6	0	-4.965798	-0.073640	1.488098
28	1	0	-2.863004	0.093446	1.892294
29	6	0	-5.586315	-0.208661	-0.835150
30	1	0	-3.957527	-0.147440	-2.237019
31	6	0	-5.946454	-0.180353	0.504822
32	1	0	-5.248857	-0.051687	2.533246
33	1	0	-6.346558	-0.292728	-1.601585
34	1	0	-6.990355	-0.240853	0.787051
35	6	0	-1.852285	0.070378	-0.633474
36	1	0	-1.678728	0.058623	-1.715814

Structure 11.1H$_2$O (B3LYP, Gas Phase)

Energy (Hartrees): = - 1012.9079962
No imaginary frequencies

Standard orientation:

Center Number	Atomic Number	Atomic Type	Coordinates (Angstroms) X	Y	Z
1	6	0	4.140314	1.008597	-1.042071
2	6	0	3.195083	0.247154	-0.336750
3	6	0	3.637654	-0.661155	0.642615
4	6	0	4.997989	-0.790363	0.903415
5	6	0	5.932319	-0.023300	0.199515
6	6	0	5.501638	0.877321	-0.775262
7	6	0	1.773651	0.430543	-0.662468
8	7	0	0.815962	-0.151857	-0.050357
9	6	0	-0.547333	0.130042	-0.465024
10	6	0	-1.175542	1.182022	0.461783
11	6	0	-2.666596	1.352110	0.165194
12	6	0	-3.373020	-0.007029	0.202141
13	8	0	-2.736604	-0.880966	-0.739655
14	6	0	-1.377268	-1.173782	-0.418395
15	8	0	-0.486839	2.405904	0.278446
16	8	0	-0.914310	-2.137890	-1.306289
17	6	0	-4.841848	0.029533	-0.204755
18	8	0	-5.412281	-1.261779	-0.191900
19	8	0	-3.158075	2.256977	1.157176
20	1	0	1.569419	1.123463	-1.491048
21	1	0	-0.591025	0.527159	-1.494817
22	1	0	-0.945033	3.049518	0.838405
23	1	0	-1.076999	0.832840	1.502620
24	1	0	-3.964668	2.671537	0.827388
25	1	0	-2.769317	1.783448	-0.841994
26	1	0	-4.786649	-1.827659	-0.668835
27	1	0	-5.405628	0.639568	0.508727
28	1	0	-4.922126	0.501111	-1.199741
29	1	0	-3.294711	-0.430256	1.215846
30	1	0	-0.928394	-1.755440	-2.195959
31	1	0	-1.305983	-1.625760	0.579291
32	1	0	3.800919	1.710168	-1.800130
33	1	0	6.222719	1.474216	-1.325513
34	1	0	6.992432	-0.130210	0.410316

35	1	0	5.333751	-1.493674	1.659584
36	1	0	2.918224	-1.265208	1.186213
37	1	0	0.845575	-1.536979	1.283101
38	8	0	0.784024	-2.350471	1.824934
39	1	0	0.806193	-3.049221	1.157723

Structure 11.1H$_2$O (B3LYP, DMSO)

Energy (Hartrees): = - 1012.9382675
No imaginary frequencies

Standard orientation:

Center Number	Atomic Number	Atomic Type	Coordinates (Angstroms) X	Y	Z
1	6	0	4.150166	0.682822	-1.302171
2	6	0	3.203755	0.167899	-0.400580
3	6	0	3.650191	-0.460179	0.777265
4	6	0	5.013323	-0.563170	1.038525
5	6	0	5.948746	-0.043624	0.136022
6	6	0	5.514982	0.579801	-1.035355
7	6	0	1.779222	0.312812	-0.737209
8	7	0	0.818795	-0.070260	0.015005
9	6	0	-0.543830	0.145255	-0.447911
10	6	0	-1.237012	1.195398	0.431056
11	6	0	-2.719730	1.309199	0.075367
12	6	0	-3.381382	-0.071095	0.144708
13	8	0	-2.686531	-0.965782	-0.738290
14	6	0	-1.324512	-1.188778	-0.376214
15	8	0	-0.582078	2.443419	0.259021
16	8	0	-0.802414	-2.160793	-1.219467
17	6	0	-4.834135	-0.092886	-0.310057
18	8	0	-5.376993	-1.404206	-0.240636
19	8	0	-3.280852	2.229671	1.016654
20	1	0	1.576898	0.790037	-1.704977
21	1	0	-0.570117	0.496832	-1.492513
22	1	0	-1.142440	3.087492	0.720493
23	1	0	-1.169034	0.869178	1.481805
24	1	0	-4.050457	2.649592	0.606265
25	1	0	-2.807493	1.699652	-0.947730
26	1	0	-4.735781	-1.970330	-0.697745
27	1	0	-5.436667	0.542167	0.346504
28	1	0	-4.898694	0.314608	-1.331703
29	1	0	-3.322441	-0.449481	1.177219
30	1	0	-0.764162	-1.786562	-2.115076
31	1	0	-1.268306	-1.599165	0.641055
32	1	0	3.808448	1.167595	-2.213058
33	1	0	6.236742	0.983874	-1.739015
34	1	0	7.011015	-0.126614	0.347232
35	1	0	5.351735	-1.050705	1.948260
36	1	0	2.929452	-0.868997	1.478269
37	1	0	0.822428	-1.089348	1.627587
38	8	0	0.783478	-1.697997	2.398546
39	1	0	0.941724	-2.563220	1.994795

Structure 11.1H$_2$O (B3LYP, H$_2$O)

Energy (Hartrees): = - 1012.9518189
No imaginary frequencies

Standard orientation:

Center Number	Atomic Number	Atomic Type	Coordinates (Angstroms) X	Y	Z
1	6	0	4.171818	-0.019453	-1.463879
2	6	0	3.220284	-0.019002	-0.430521
3	6	0	3.659303	-0.058769	0.905890
4	6	0	5.020552	-0.092908	1.191943
5	6	0	5.961913	-0.088928	0.155708
6	6	0	5.535270	-0.052568	-1.173148
7	6	0	1.799124	0.020623	-0.800765
8	7	0	0.837515	0.085708	0.042039
9	6	0	-0.523838	0.076246	-0.477872
10	6	0	-1.300937	1.286847	0.050690
11	6	0	-2.780822	1.201741	-0.320900
12	6	0	-3.360444	-0.145821	0.128240
13	8	0	-2.588974	-1.206801	-0.461531
14	6	0	-1.228521	-1.224689	-0.032208
15	8	0	-0.716223	2.470281	-0.487474
16	8	0	-0.623606	-2.368683	-0.565532
17	6	0	-4.799312	-0.365423	-0.311435
18	8	0	-5.327891	-1.591425	0.188192
19	8	0	-3.424455	2.301767	0.330370

20	1	0	1.598241	-0.000937	-1.879261
21	1	0	-0.541866	0.097697	-1.579312
22	1	0	-1.304462	3.195135	-0.222590
23	1	0	-1.228406	1.293912	1.149279
24	1	0	-4.203354	2.544267	-0.189805
25	1	0	-2.878952	1.291711	-1.410594
26	1	0	-4.723243	-2.287915	-0.109398
27	1	0	-5.423631	0.438359	0.087517
28	1	0	-4.850810	-0.329777	-1.409530
29	1	0	-3.302781	-0.216111	1.225131
30	1	0	-0.520319	-2.232824	-1.521273
31	1	0	-1.182574	-1.325225	1.059762
32	1	0	3.835484	0.008208	-2.496731
33	1	0	6.261116	-0.050635	-1.980457
34	1	0	7.022784	-0.115408	0.385830
35	1	0	5.353056	-0.123797	2.225086
36	1	0	2.933022	-0.065598	1.711584
37	1	0	0.761748	-0.057784	1.928423
38	8	0	0.633638	-0.183270	2.896930
39	1	0	0.666093	-1.145563	2.993969

Structure 11.1H_2O (M06-2X, Gas Phase)

Energy (Hartrees): = - 1012.7739547
No imaginary frequencies

Standard orientation:

Center Number	Atomic Number	Atomic Type	Coordinates (Angstroms) X	Y	Z
1	6	0	4.081816	1.212324	-0.806000
2	6	0	3.167121	0.289927	-0.295927
3	6	0	3.624315	-0.783089	0.476056
4	6	0	4.979600	-0.913262	0.738702
5	6	0	5.887607	0.018511	0.237975
6	6	0	5.438576	1.081458	-0.536424
7	6	0	1.738603	0.475525	-0.602660
8	7	0	0.815398	-0.190886	-0.045211
9	6	0	-0.549606	0.092898	-0.440880
10	6	0	-1.137972	1.198237	0.431094
11	6	0	-2.616486	1.382730	0.127070
12	6	0	-3.343840	0.046874	0.235756
13	8	0	-2.734499	-0.883681	-0.652110
14	6	0	-1.396151	-1.177583	-0.296413
15	8	0	-0.425327	2.385539	0.176746
16	8	0	-0.937852	-2.212222	-1.095076
17	6	0	-4.803782	0.107462	-0.168186
18	8	0	-5.399232	-1.162357	-0.094645
19	8	0	-3.090327	2.333723	1.067512
20	1	0	1.507678	1.242767	-1.352109
21	1	0	-0.608709	0.427285	-1.489582
22	1	0	-0.835622	3.070741	0.714283
23	1	0	-1.040143	0.904691	1.487029
24	1	0	-3.894532	2.737268	0.734227
25	1	0	-2.709949	1.765429	-0.898659
26	1	0	-4.804817	-1.763868	-0.555818
27	1	0	-5.348815	0.761342	0.516321
28	1	0	-4.868522	0.525446	-1.184022
29	1	0	-3.270547	-0.325727	1.267830
30	1	0	-0.895906	-1.898963	-2.004653
31	1	0	-1.351749	-1.550508	0.733050
32	1	0	3.724943	2.039840	-1.410494
33	1	0	6.142423	1.804539	-0.930609
34	1	0	6.945088	-0.089896	0.448121
35	1	0	5.331206	-1.747301	1.333893
36	1	0	2.921517	-1.518724	0.850237
37	1	0	0.880789	-1.814748	1.088709
38	8	0	0.780147	-2.746891	1.331513
39	1	0	0.505810	-3.138374	0.496223

Structure 11.1H_2O (M06-2X, DMSO)

Energy (Hartrees): = - 1012.8065189
No imaginary frequencies
Standard orientation:

Center Number	Atomic Number	Atomic Type	Coordinates (Angstroms) X	Y	Z
1	6	0	-4.063991	-1.183869	-0.884354
2	6	0	-3.168514	-0.270954	-0.322475
3	6	0	-3.652899	0.756494	0.494215
4	6	0	-5.012777	0.852904	0.753646
5	6	0	-5.900427	-0.072790	0.204828

6	6	0	-5.425372	-1.090185	-0.615983
7	6	0	-1.733219	-0.426473	-0.623574
8	7	0	-0.817092	0.178600	0.012706
9	6	0	0.551710	-0.091520	-0.394550
10	6	0	1.157830	-1.184915	0.480402
11	6	0	2.625508	-1.377783	0.132202
12	6	0	3.362679	-0.044491	0.213391
13	8	0	2.724055	0.901776	-0.642358
14	6	0	1.395607	1.178825	-0.244874
15	8	0	0.434851	-2.379127	0.279261
16	8	0	0.912010	2.233483	-1.005446
17	6	0	4.800355	-0.121551	-0.255756
18	8	0	5.419312	1.147138	-0.191123
19	8	0	3.131976	-2.318754	1.067707
20	1	0	-1.495716	-1.116393	-1.440854
21	1	0	0.602734	-0.417051	-1.444511
22	1	0	0.910815	-3.064322	0.763894
23	1	0	1.094321	-0.867627	1.531905
24	1	0	3.904868	-2.748680	0.686920
25	1	0	2.693909	-1.769791	-0.890554
26	1	0	4.825537	1.752772	-0.649262
27	1	0	5.368068	-0.793098	0.391107
28	1	0	4.818023	-0.521835	-1.278544
29	1	0	3.333292	0.322436	1.249436
30	1	0	0.783147	1.923562	-1.911756
31	1	0	1.386154	1.521439	0.796261
32	1	0	-3.686650	-1.971542	-1.528591
33	1	0	-6.113700	-1.807093	-1.048303
34	1	0	-6.961371	0.005904	0.412814
35	1	0	-5.385258	1.653571	1.382061
36	1	0	-2.967166	1.490148	0.902165
37	1	0	-0.907526	1.808623	1.136391
38	8	0	-0.827081	2.756088	1.332758
39	1	0	-0.463757	3.081792	0.501392

Structure 11.1H_2O (M06-2X, H_2O)

Energy (Hartrees): = - 1012.8204915
No imaginary frequencies

Standard orientation:

Center Number	Atomic Number	Atomic Type	Coordinates (Angstroms) X	Y	Z
1	6	0	-4.057189	-1.147188	-0.934209
2	6	0	-3.171034	-0.260987	-0.317716
3	6	0	-3.666957	0.718857	0.549428
4	6	0	-5.029285	0.796428	0.800506
5	6	0	-5.907838	-0.101329	0.193852
6	6	0	-5.421371	-1.072668	-0.674738
7	6	0	-1.735089	-0.400753	-0.615124
8	7	0	-0.818668	0.203101	0.027482
9	6	0	0.548614	-0.073904	-0.385745
10	6	0	1.152365	-1.168637	0.488733
11	6	0	2.617785	-1.377991	0.139688
12	6	0	3.365767	-0.047358	0.193459
13	8	0	2.725491	0.896648	-0.665720
14	6	0	1.401561	1.188375	-0.253970
15	8	0	0.412390	-2.358482	0.286519
16	8	0	0.920659	2.241454	-1.031026
17	6	0	4.797128	-0.155499	-0.283791
18	8	0	5.471378	1.086866	-0.165196
19	8	0	3.130310	-2.295960	1.094885
20	1	0	-1.492696	-1.079283	-1.439894
21	1	0	0.591830	-0.399946	-1.434918
22	1	0	0.834006	-3.043959	0.818746
23	1	0	1.093732	-0.857496	1.541014
24	1	0	3.896103	-2.739796	0.716558
25	1	0	2.683610	-1.795392	-0.872287
26	1	0	4.949889	1.731270	-0.657598
27	1	0	5.334935	-0.876668	0.333226
28	1	0	4.800309	-0.506742	-1.322697
29	1	0	3.349039	0.332234	1.224709
30	1	0	0.736580	1.908325	-1.919319
31	1	0	1.412484	1.544837	0.781554
32	1	0	-3.669641	-1.898296	-1.614682
33	1	0	-6.102082	-1.768491	-1.150373
34	1	0	-6.970675	-0.037952	0.395699
35	1	0	-5.411417	1.558308	1.469503
36	1	0	-2.987710	1.427321	1.008109
37	1	0	-0.907597	1.831602	1.083933
38	8	0	-0.825830	2.767604	1.342973
39	1	0	-0.451964	3.159302	0.544621

Structure 11.5H_2O (B3LYP, Gas Phase)

Energy (Hartrees): = - 1318.6820983
No imaginary frequencies

Standard orientation:

Center Number	Atomic Number	Atomic Type	Coordinates (Angstroms) X	Y	Z
1	6	0	-4.286837	0.531000	-0.210543
2	6	0	-3.701316	-0.694603	0.153619
3	6	0	-4.510849	-1.837925	0.248250
4	6	0	-5.872212	-1.770649	-0.039900
5	6	0	-6.442724	-0.552463	-0.411105
6	6	0	-5.648335	0.595942	-0.488927
7	6	0	-2.270257	-0.830961	0.457355
8	7	0	-1.383404	0.049297	0.185343
9	6	0	-0.002459	-0.245341	0.552355
10	6	0	0.721387	-0.974334	-0.611573
11	8	0	2.100067	-1.164970	-0.260967
12	6	0	2.879721	0.027970	-0.029797
13	6	0	2.246766	0.789973	1.140969
14	6	0	0.764903	1.047764	0.840216
15	8	0	0.194791	-2.221623	-0.911522
16	6	0	4.316402	-0.426328	0.220256
17	8	0	4.905098	-1.038711	-0.896416
18	8	0	2.887333	2.009436	1.455965
19	8	0	0.171670	1.754671	1.928608
20	8	0	2.644241	3.518031	-0.782372
21	8	0	0.164961	4.132203	-1.622112
22	8	0	-1.615552	2.880149	-0.010773
23	8	0	1.824003	-3.570748	0.977732
24	8	0	3.521562	-3.492856	-1.395953
25	1	0	-1.991215	-1.773700	0.948454
26	1	0	0.718430	1.671084	-0.065339
27	1	0	0.888841	2.331346	2.248673
28	1	0	2.863502	2.586582	0.651214
29	1	0	2.318381	0.164349	2.042363
30	1	0	2.855415	0.648401	-0.937427
31	1	0	4.904656	0.461296	0.473414
32	1	0	4.321679	-1.078883	1.112846
33	1	0	4.487653	-1.914793	-1.034155
34	1	0	0.405363	-2.826758	-0.173926
35	1	0	0.652185	-0.369530	-1.525340
36	1	0	0.047837	-0.907516	1.432116
37	1	0	-3.681361	1.429815	-0.243558
38	1	0	-6.095157	1.546983	-0.762530
39	1	0	-7.504559	-0.494353	-0.631549
40	1	0	-6.485860	-2.663735	0.028008
41	1	0	-4.064029	-2.785309	0.539049
42	1	0	-1.643476	1.922959	-0.234032
43	1	0	-1.199898	2.831025	0.870127
44	1	0	2.176450	-2.665868	0.866502
45	1	0	1.909833	-3.797427	1.911997
46	1	0	-0.527662	3.685707	-1.069240
47	1	0	-0.026402	5.075124	-1.549380
48	1	0	1.734524	3.772392	-1.087844
49	1	0	3.144595	3.318356	-1.581750
50	1	0	2.746769	-3.014102	-1.724545
51	1	0	3.176800	-3.903455	-0.586116

Structure 11.5H_2O (B3LYP, DMSO)

Energy (Hartrees): = - 1318.7188833
No imaginary frequencies

Standard orientation:

Center Number	Atomic Number	Atomic Type	Coordinates (Angstroms) X	Y	Z
1	6	0	-4.258924	0.500133	-0.440571
2	6	0	-3.705933	-0.640286	0.169686
3	6	0	-4.547978	-1.719547	0.487478
4	6	0	-5.909050	-1.670897	0.188974
5	6	0	-6.446487	-0.536950	-0.423027
6	6	0	-5.618834	0.548733	-0.731565
7	6	0	-2.277866	-0.763764	0.495925
8	7	0	-1.375554	0.072113	0.139153
9	6	0	-0.000446	-0.238818	0.520912
10	6	0	0.720823	-0.956312	-0.650673
11	8	0	2.089165	-1.189350	-0.284087
12	6	0	2.888066	-0.017414	-0.026986

13	6	0	2.255758	0.755818	1.139001
14	6	0	0.782171	1.040392	0.826678
15	8	0	0.174384	-2.187014	-0.984441
16	6	0	4.302244	-0.509948	0.264665
17	8	0	4.899900	-1.172434	-0.830209
18	8	0	2.921274	1.969326	1.449005
19	8	0	0.184531	1.744727	1.920740
20	8	0	2.759817	3.598517	-0.722630
21	8	0	0.255525	4.232118	-1.532492
22	8	0	-1.561856	2.922767	0.010276
23	8	0	1.705098	-3.673992	0.874362
24	8	0	3.393830	-3.539504	-1.437934
25	1	0	-2.016925	-1.654774	1.080802
26	1	0	0.751950	1.675490	-0.070579
27	1	0	0.893908	2.330602	2.241153
28	1	0	2.885782	2.556298	0.649978
29	1	0	2.312149	0.132136	2.040945
30	1	0	2.899549	0.609354	-0.931118
31	1	0	4.919115	0.362080	0.503721
32	1	0	4.273844	-1.146322	1.165254
33	1	0	4.424301	-2.017312	-0.972386
34	1	0	0.363306	-2.807338	-0.251385
35	1	0	0.678959	-0.329363	-1.550756
36	1	0	0.031928	-0.912068	1.391122
37	1	0	-3.628809	1.353264	-0.664570
38	1	0	-6.038351	1.435225	-1.198095
39	1	0	-7.506775	-0.494194	-0.654753
40	1	0	-6.547887	-2.513914	0.434850
41	1	0	-4.126444	-2.599978	0.965483
42	1	0	-1.606957	1.972769	-0.240994
43	1	0	-1.119860	2.838084	0.876700
44	1	0	2.068753	-2.773926	0.956337
45	1	0	1.648097	-4.029513	1.774011
46	1	0	-0.432697	3.753281	-1.002681
47	1	0	0.107409	5.167866	-1.333921
48	1	0	1.836245	3.834544	-0.999973
49	1	0	3.163223	3.214291	-1.513571
50	1	0	2.678139	-2.931760	-1.681358
51	1	0	3.042319	-3.917737	-0.611982

Structure 11.5H_2O (B3LYP, H_2O)

Energy (Hartrees): = - 1318.7388955
No imaginary frequencies
Standard orientation:

Center Number	Atomic Number	Atomic Type	Coordinates (Angstroms) X	Y	Z
1	6	0	-4.383479	0.411225	-0.291525
2	6	0	-3.757270	-0.772482	0.139618
3	6	0	-4.536173	-1.925778	0.333410
4	6	0	-5.906941	-1.907685	0.079292
5	6	0	-6.517743	-0.730544	-0.357631
6	6	0	-5.753439	0.428340	-0.535956
7	6	0	-2.316610	-0.862944	0.413060
8	7	0	-1.458004	0.035035	0.096000
9	6	0	-0.065851	-0.242313	0.454589
10	6	0	0.680061	-0.903524	-0.727327
11	8	0	2.049369	-1.119450	-0.367291
12	6	0	2.803213	0.075341	-0.073736
13	6	0	2.151234	0.789562	1.119309
14	6	0	0.671811	1.048045	0.816663
15	8	0	0.147257	-2.143184	-1.094033
16	6	0	4.240721	-0.351548	0.197809
17	8	0	4.876012	-0.917202	-0.938994
18	8	0	2.796523	2.010133	1.458014
19	8	0	0.040829	1.666918	1.944050
20	8	0	2.679915	3.658870	-0.705953
21	8	0	0.121997	4.095283	-1.579745
22	8	0	-1.747283	2.904758	0.048106
23	8	0	2.580776	-3.401929	1.192394
24	8	0	3.918383	-3.528564	-1.250452
25	1	0	-2.003062	-1.781777	0.923591
26	1	0	0.622379	1.729242	-0.044127
27	1	0	0.678966	2.332837	2.252939
28	1	0	2.737550	2.615572	0.672669
29	1	0	2.225820	0.140267	2.000765
30	1	0	2.781156	0.733977	-0.954848
31	1	0	4.804899	0.539509	0.488158
32	1	0	4.251531	-1.047193	1.049498
33	1	0	4.526065	-1.830217	-1.054588
34	1	0	0.074354	-2.690821	-0.294904
35	1	0	0.636140	-0.266964	-1.619247
36	1	0	-0.006149	-0.944324	1.300052
37	1	0	-3.802326	1.319032	-0.405167
38	1	0	-6.230677	1.347617	-0.861646

39	1	0	-7.585946	-0.711160	-0.551641
40	1	0	-6.496808	-2.807163	0.225825
41	1	0	-4.057799	-2.838583	0.677900
42	1	0	-1.759139	1.952533	-0.200485
43	1	0	-1.271266	2.850348	0.898093
44	1	0	2.304030	-2.541626	0.814737
45	1	0	3.246704	-3.160328	1.852912
46	1	0	-0.546479	3.656070	-0.996439
47	1	0	0.037031	5.034699	-1.361514
48	1	0	1.738390	3.786530	-0.988858
49	1	0	3.059565	3.103202	-1.401915
50	1	0	3.153664	-3.285494	-1.793681
51	1	0	3.520005	-3.620268	-0.354297

Structure 11.5H$_2$O (M06-2X, Gas Phase)

Energy (Hartrees): = - 1318.5261666
No imaginary frequencies

Standard orientation:

Center Number	Atomic Number	Atomic Type	Coordinates (Angstroms) X	Y	Z
1	6	0	-4.228748	0.545500	-0.169772
2	6	0	-3.649857	-0.691339	0.129739
3	6	0	-4.448618	-1.835647	0.160215
4	6	0	-5.805375	-1.755236	-0.127277
5	6	0	-6.374233	-0.524348	-0.431416
6	6	0	-5.585148	0.624188	-0.446277
7	6	0	-2.215108	-0.829627	0.431867
8	7	0	-1.350083	0.061674	0.175597
9	6	0	0.030459	-0.231176	0.525647
10	6	0	0.726119	-0.960076	-0.639344
11	8	0	2.090288	-1.161949	-0.300860
12	6	0	2.872064	0.018007	-0.100090
13	6	0	2.272386	0.783394	1.075697
14	6	0	0.795694	1.056286	0.804070
15	8	0	0.184412	-2.203180	-0.911811
16	6	0	4.293281	-0.456723	0.144381
17	8	0	4.821179	-1.137270	-0.957609
18	8	0	2.937461	1.987844	1.368962
19	8	0	0.220408	1.729050	1.912475
20	8	0	2.585019	3.140491	-1.052403
21	8	0	0.221040	4.378220	-1.293027
22	8	0	-1.596751	2.881152	0.050134
23	8	0	1.661465	-3.378957	1.182882
24	8	0	3.289745	-3.560653	-1.226126
25	1	0	-1.924568	-1.777220	0.903372
26	1	0	0.733420	1.695945	-0.089010
27	1	0	0.919084	2.309650	2.243754
28	1	0	2.943221	2.521041	0.549014
29	1	0	2.348296	0.159330	1.976691
30	1	0	2.838152	0.634058	-1.007038
31	1	0	4.918124	0.415883	0.343541
32	1	0	4.295798	-1.079354	1.054487
33	1	0	4.364733	-1.990571	-1.041774
34	1	0	0.336547	-2.769261	-0.138063
35	1	0	0.652168	-0.364061	-1.555902
36	1	0	0.089748	-0.888280	1.406935
37	1	0	-3.623369	1.443697	-0.159289
38	1	0	-6.032197	1.585372	-0.669171
39	1	0	-7.433198	-0.456465	-0.650403
40	1	0	-6.416622	-2.649377	-0.111457
41	1	0	-3.999774	-2.793917	0.400180
42	1	0	-1.625369	1.948764	-0.232413
43	1	0	-1.210145	2.780785	0.932768
44	1	0	2.119781	-2.549520	0.971200
45	1	0	1.749971	-3.529469	2.126080
46	1	0	-0.504838	3.854814	-0.891410
47	1	0	0.085793	5.279821	-0.996552
48	1	0	1.757368	3.652204	-1.185876
49	1	0	3.172645	3.350601	-1.779820
50	1	0	2.510398	-3.081108	-1.529803
51	1	0	2.973790	-3.992284	-0.424782

Structure 11.5H$_2$O (M06-2X, DMSO)

Energy (Hartrees): = - 1318.5662169
No imaginary frequencies

Standard orientation:

Center Number	Atomic Number	Atomic Type	Coordinates (Angstroms) X	Y	Z

1	6	0	-4.048890	0.040348	-0.923643
2	6	0	-3.617768	-0.763499	0.137280
3	6	0	-4.554962	-1.480465	0.883530
4	6	0	-5.910506	-1.382541	0.588205
5	6	0	-6.334179	-0.573836	-0.461292
6	6	0	-5.401014	0.133594	-1.219354
7	6	0	-2.193302	-0.870837	0.499758
8	7	0	-1.307289	-0.095602	0.025751
9	6	0	0.065864	-0.316784	0.436048
10	6	0	0.835979	-0.959488	-0.729438
11	8	0	2.199023	-1.104169	-0.357420
12	6	0	2.900752	0.107194	-0.084417
13	6	0	2.220699	0.803612	1.091497
14	6	0	0.741627	1.005616	0.778443
15	8	0	0.374280	-2.220715	-1.061243
16	6	0	4.330090	-0.304631	0.214072
17	8	0	4.917069	-0.991384	-0.864894
18	8	0	2.816539	2.034914	1.430639
19	8	0	0.089243	1.599943	1.893372
20	8	0	2.470913	3.545079	-0.800724
21	8	0	-0.078274	4.348736	-1.175389
22	8	0	-1.813982	2.713508	0.142127
23	8	0	1.813201	-3.433664	1.040103
24	8	0	3.446597	-3.453356	-1.312580
25	1	0	-1.941130	-1.656427	1.221856
26	1	0	0.663548	1.673663	-0.092257
27	1	0	0.724767	2.231142	2.260187
28	1	0	2.762889	2.612367	0.640155
29	1	0	2.305081	0.155725	1.972968
30	1	0	2.874741	0.747900	-0.976978
31	1	0	4.922456	0.591463	0.407672
32	1	0	4.330765	-0.915250	1.129381
33	1	0	4.446628	-1.834271	-0.973625
34	1	0	0.532827	-2.795949	-0.293729
35	1	0	0.754529	-0.331414	-1.623845
36	1	0	0.127696	-0.993324	1.302006
37	1	0	-3.319493	0.572348	-1.523929
38	1	0	-5.730711	0.753783	-2.044733
39	1	0	-7.389944	-0.498316	-0.695170
40	1	0	-6.633692	-1.936931	1.174711
41	1	0	-4.216865	-2.110212	1.700195
42	1	0	-1.828803	1.818001	-0.236132
43	1	0	-1.344919	2.546054	0.974466
44	1	0	2.184150	-2.544394	1.129936
45	1	0	1.723525	-3.791561	1.930453
46	1	0	-0.748944	3.773554	-0.751199
47	1	0	-0.277577	5.240127	-0.872629
48	1	0	1.550244	3.856408	-0.940308
49	1	0	2.772220	3.227301	-1.657516
50	1	0	2.716835	-2.899553	-1.616443
51	1	0	3.106999	-3.763849	-0.461803

Structure 11.5H_2O (M06-2X, H_2O)

Energy (Hartrees): = - 1318.5879296
No imaginary frequencies
Standard orientation:

Center Number	Atomic Number	Atomic Type	Coordinates (Angstroms) X	Y	Z
1	6	0	-4.154846	0.527052	-0.630594
2	6	0	-3.685824	-0.565917	0.107101
3	6	0	-4.594364	-1.510111	0.591020
4	6	0	-5.956550	-1.362778	0.352741
5	6	0	-6.417144	-0.272035	-0.376693
6	6	0	-5.513854	0.670104	-0.869911
7	6	0	-2.257843	-0.760063	0.409111
8	7	0	-1.361471	0.102127	0.142654
9	6	0	0.007402	-0.249036	0.489820
10	6	0	0.727521	-0.802478	-0.747528
11	8	0	2.084248	-1.067858	-0.412776
12	6	0	2.850150	0.064413	0.003557
13	6	0	2.216897	0.666766	1.257617
14	6	0	0.753376	0.980981	0.981434
15	8	0	0.180570	-2.004142	-1.191804
16	6	0	4.261212	-0.427570	0.255735
17	8	0	4.892779	-0.889298	-0.925104
18	8	0	2.892914	1.839390	1.673253
19	8	0	0.101738	1.466102	2.143516
20	8	0	2.825918	3.467056	-0.554849
21	8	0	0.528828	2.807829	-1.940686
22	8	0	-1.543976	2.938027	-0.068183
23	8	0	1.292124	-3.384191	1.042538
24	8	0	3.484821	-3.330696	-1.277161

25	1	0	-2.003742	-1.703048	0.906093
26	1	0	0.729233	1.744416	0.193856
27	1	0	0.637455	2.197761	2.475398
28	1	0	2.868092	2.467027	0.919801
29	1	0	2.284174	-0.055005	2.079570
30	1	0	2.849322	0.812623	-0.801391
31	1	0	4.851253	0.401553	0.648152
32	1	0	4.224473	-1.218761	1.015955
33	1	0	4.522504	-1.764902	-1.124614
34	1	0	0.286346	-2.648226	-0.466782
35	1	0	0.681821	-0.087019	-1.574825
36	1	0	0.037449	-1.029812	1.262813
37	1	0	-3.456431	1.258006	-1.019500
38	1	0	-5.872692	1.516840	-1.442902
39	1	0	-7.477758	-0.154149	-0.565582
40	1	0	-6.655697	-2.096363	0.736033
41	1	0	-4.227548	-2.358803	1.158940
42	1	0	-1.586043	1.958588	-0.032829
43	1	0	-1.131289	3.170577	0.772295
44	1	0	1.864537	-2.611006	0.930267
45	1	0	0.829016	-3.219272	1.871535
46	1	0	-0.220903	2.956427	-1.332080
47	1	0	0.348308	3.362989	-2.706751
48	1	0	2.013657	3.256282	-1.059854
49	1	0	3.539506	3.093969	-1.084016
50	1	0	2.754925	-2.698180	-1.193727
51	1	0	3.503818	-3.761123	-0.414845

Structure 11·5H_2O (M06-2X/def2-TZVP, Gas Phase)

Energy (Hartrees): = -1318.683000
No imaginary frequencies

Standard orientation:

Center Number	Atomic Number	Atomic Type	Coordinates (Angstroms) X	Y	Z
1	6	0	-4.063549	-0.006630	-0.932881
2	6	0	-3.633216	-0.785347	0.141238
3	6	0	-4.567470	-1.491859	0.893392
4	6	0	-5.918640	-1.410483	0.591542
5	6	0	-6.340611	-0.626165	-0.471633
6	6	0	-5.410768	0.072141	-1.235250
7	6	0	-2.212621	-0.878022	0.506809
8	7	0	-1.317767	-0.132774	0.014842
9	6	0	0.048518	-0.336323	0.436113
10	6	0	0.849879	-0.956618	-0.718124
11	8	0	2.206132	-1.077415	-0.327694
12	6	0	2.881407	0.144326	-0.037953
13	6	0	2.174928	0.810663	1.136289
14	6	0	0.697160	0.988603	0.811421
15	8	0	0.407321	-2.214943	-1.075961
16	6	0	4.320233	-0.228224	0.261879
17	8	0	4.965045	-0.800110	-0.839835
18	8	0	2.737870	2.042595	1.509968
19	8	0	0.022804	1.547811	1.925512
20	8	0	2.388008	3.399249	-0.851181
21	8	0	-0.126692	4.320845	-1.213698
22	8	0	-1.858495	2.709115	0.108988
23	8	0	1.944803	-3.450424	1.022174
24	8	0	3.656980	-3.349780	-1.399107
25	1	0	-1.964999	-1.637987	1.258762
26	1	0	0.617061	1.667901	-0.049709
27	1	0	0.649279	2.164328	2.331620
28	1	0	2.728352	2.630111	0.727737
29	1	0	2.258102	0.153381	2.011362
30	1	0	2.844963	0.798296	-0.918206
31	1	0	4.863562	0.674737	0.541720
32	1	0	4.326844	-0.901454	1.132832
33	1	0	4.589453	-1.679835	-1.013031
34	1	0	0.516305	-2.811403	-0.317795
35	1	0	0.774851	-0.322097	-1.607567
36	1	0	0.113239	-1.024829	1.293445
37	1	0	-3.332220	0.518476	-1.533399
38	1	0	-5.740337	0.675991	-2.070542
39	1	0	-7.394131	-0.561868	-0.711505
40	1	0	-6.639757	-1.958369	1.183625
41	1	0	-4.232126	-2.103021	1.723508
42	1	0	-1.876878	1.814199	-0.269344
43	1	0	-1.448414	2.544978	0.972673
44	1	0	2.307376	-2.564058	0.853315
45	1	0	2.062024	-3.645302	1.955174
46	1	0	-0.807926	3.762677	-0.775048
47	1	0	-0.407317	5.232051	-1.106984
48	1	0	1.510668	3.806757	-1.020662

49	1	0	2.985376	3.695846	-1.540611
50	1	0	2.843847	-2.913277	-1.682728
51	1	0	3.375481	-3.878734	-0.642924

Structure 11·5H_2O (M06-2X/def2-TZVP, DMSO)

Energy (Hartrees): = -1318.723303
No imaginary frequencies

Standard orientation:

Center Number	Atomic Number	Atomic Type	Coordinates (Angstroms) X	Y	Z
1	6	0	-4.059875	-0.048776	-0.961306
2	6	0	-3.628587	-0.770548	0.153329
3	6	0	-4.564111	-1.424328	0.952270
4	6	0	-5.917797	-1.343882	0.656137
5	6	0	-6.341441	-0.616364	-0.447537
6	6	0	-5.410119	0.027483	-1.257839
7	6	0	-2.206272	-0.864110	0.517924
8	7	0	-1.309682	-0.145283	-0.014333
9	6	0	0.058055	-0.343092	0.413025
10	6	0	0.861015	-0.960627	-0.740016
11	8	0	2.216268	-1.089020	-0.346387
12	6	0	2.890477	0.128795	-0.048089
13	6	0	2.176762	0.809517	1.114965
14	6	0	0.703227	0.987873	0.774239
15	8	0	0.422104	-2.221600	-1.099946
16	6	0	4.317704	-0.254599	0.284751
17	8	0	4.965160	-0.890575	-0.791062
18	8	0	2.746672	2.047159	1.467523
19	8	0	0.015195	1.567650	1.872587
20	8	0	2.481994	3.707786	-0.701919
21	8	0	-0.118677	4.285393	-1.252373
22	8	0	-1.891527	2.686786	0.087433
23	8	0	1.918831	-3.521261	0.985189
24	8	0	3.592177	-3.417323	-1.379027
25	1	0	-1.962764	-1.592120	1.299522
26	1	0	0.631397	1.653520	-0.098799
27	1	0	0.626554	2.205174	2.271165
28	1	0	2.700494	2.645525	0.690772
29	1	0	2.255291	0.161200	1.995522
30	1	0	2.874235	0.778602	-0.933989
31	1	0	4.880620	0.647344	0.526516
32	1	0	4.306474	-0.894708	1.177495
33	1	0	4.538997	-1.750415	-0.944893
34	1	0	0.547950	-2.814461	-0.339610
35	1	0	0.789008	-0.323570	-1.628047
36	1	0	0.121619	-1.022771	1.275731
37	1	0	-3.332082	0.433596	-1.602582
38	1	0	-5.740246	0.584762	-2.125625
39	1	0	-7.396725	-0.554916	-0.683131
40	1	0	-6.640086	-1.849457	1.284529
41	1	0	-4.225584	-1.992368	1.811596
42	1	0	-1.901187	1.799197	-0.311254
43	1	0	-1.422814	2.513354	0.920865
44	1	0	2.240206	-2.608513	1.045858
45	1	0	1.862536	-3.862402	1.885920
46	1	0	-0.799401	3.741752	-0.799955
47	1	0	-0.406728	5.201496	-1.177010
48	1	0	1.550684	3.938711	-0.911050
49	1	0	2.892609	3.457156	-1.536607
50	1	0	2.837042	-2.878416	-1.651512
51	1	0	3.279882	-3.804298	-0.548362

Structure 11·5H_2O (M06-2X/def2-TZVP, H_2O)

Energy (Hartrees): = -1318.742159
No imaginary frequencies

Standard orientation:

Center Number	Atomic Number	Atomic Type	Coordinates (Angstroms) X	Y	Z
1	6	0	-4.133722	0.486590	-0.677365
2	6	0	-3.663101	-0.571061	0.103558
3	6	0	-4.567813	-1.496699	0.621511
4	6	0	-5.927403	-1.364188	0.376384
5	6	0	-6.389330	-0.307525	-0.395613

6	6	0	-5.489997	0.615112	-0.924214
7	6	0	-2.237835	-0.754764	0.411136
8	7	0	-1.337282	0.093653	0.125944
9	6	0	0.026866	-0.258659	0.473980
10	6	0	0.754235	-0.807440	-0.758173
11	8	0	2.093979	-1.105386	-0.402731
12	6	0	2.868575	0.019985	0.004705
13	6	0	2.237981	0.639565	1.250275
14	6	0	0.775570	0.961781	0.979075
15	8	0	0.184458	-1.983046	-1.236856
16	6	0	4.273235	-0.477905	0.267412
17	8	0	4.916205	-0.938840	-0.907237
18	8	0	2.924054	1.805770	1.660669
19	8	0	0.129566	1.427587	2.150980
20	8	0	2.865303	3.526904	-0.516239
21	8	0	0.441403	3.262882	-1.849659
22	8	0	-1.612941	2.944097	0.005602
23	8	0	1.109480	-3.518587	1.014492
24	8	0	3.484268	-3.378043	-1.383603
25	1	0	-1.985329	-1.685137	0.930833
26	1	0	0.745157	1.739233	0.203418
27	1	0	0.640618	2.177362	2.484751
28	1	0	2.897016	2.446980	0.917258
29	1	0	2.298161	-0.079009	2.074589
30	1	0	2.877940	0.761058	-0.806810
31	1	0	4.867644	0.343125	0.666997
32	1	0	4.227578	-1.271739	1.022099
33	1	0	4.546375	-1.810099	-1.129899
34	1	0	0.231116	-2.644691	-0.520409
35	1	0	0.743035	-0.077061	-1.573679
36	1	0	0.054828	-1.045315	1.239921
37	1	0	-3.436991	1.200530	-1.096878
38	1	0	-5.850529	1.434722	-1.532545
39	1	0	-7.448933	-0.201583	-0.591359
40	1	0	-6.623964	-2.084592	0.785548
41	1	0	-4.198784	-2.320330	1.221914
42	1	0	-1.602514	1.961453	-0.018010
43	1	0	-1.195858	3.158971	0.850095
44	1	0	1.742433	-2.784986	0.987466
45	1	0	0.583667	-3.358435	1.808068
46	1	0	-0.307729	3.221445	-1.219478
47	1	0	0.278369	4.039941	-2.396887
48	1	0	2.008023	3.428954	-0.982385
49	1	0	3.514684	3.111101	-1.096188
50	1	0	2.766352	-2.770793	-1.139597
51	1	0	3.538839	-4.002115	-0.649803

Structure 11.6H_2O (B3LYP, Gas Phase)

Energy (Hartrees): = - 1395.1280228
No imaginary frequencies

Standard orientation:

Center Number	Atomic Number	Atomic Type	Coordinates (Angstroms) X	Y	Z
1	6	0	-4.491963	0.760276	-0.397874
2	6	0	-4.053254	-0.497814	0.051972
3	6	0	-4.976539	-1.551502	0.143652
4	6	0	-6.306372	-1.365526	-0.227797
5	6	0	-6.730774	-0.116358	-0.682317
6	6	0	-5.822342	0.944216	-0.760274
7	6	0	-2.662425	-0.755089	0.449449
8	7	0	-1.685408	0.035768	0.218539
9	6	0	-0.362802	-0.358287	0.684666
10	6	0	0.424443	-1.038878	-0.465782
11	8	0	1.760963	-1.319516	-0.020334
12	6	0	2.553018	-0.172169	0.358040
13	6	0	1.868770	0.518971	1.542928
14	6	0	0.437656	0.875538	1.119290
15	8	0	-0.119964	-2.234309	-0.912372
16	6	0	3.966554	-0.662521	0.635581
17	8	0	4.643438	-1.070607	-0.542508
18	8	0	2.534596	1.676087	1.994875
19	8	0	-0.227898	1.567766	2.169548
20	8	0	2.971308	3.055082	-0.245198
21	8	0	0.585239	3.582505	-1.574927
22	8	0	-1.613649	2.881038	-0.071790
23	8	0	1.508247	-3.890435	0.777262
24	8	0	3.285538	-3.342050	-1.469560
25	1	0	-2.500289	-1.705129	0.978962
26	1	0	0.520787	1.537484	0.245180
27	1	0	0.482059	2.091909	2.583293
28	1	0	2.715887	2.254669	1.198717
29	1	0	1.827541	-0.179185	2.392068
30	1	0	2.593239	0.520255	-0.490833

31	1	0	4.530457	0.164903	1.073555
32	1	0	3.938047	-1.473696	1.380226
33	1	0	4.224213	-1.897062	-0.879398
34	1	0	0.050431	-2.923617	-0.242654
35	1	0	0.458612	-0.361409	-1.329268
36	1	0	-0.422934	-1.076697	1.519180
37	1	0	-3.798155	1.592788	-0.433579
38	1	0	-6.155751	1.920114	-1.100019
39	1	0	-7.767656	0.034552	-0.967901
40	1	0	-7.009170	-2.190288	-0.159630
41	1	0	-4.643612	-2.522968	0.500417
42	1	0	-1.723438	1.920170	-0.247366
43	1	0	-1.350670	2.853676	0.865077
44	1	0	1.794406	-2.958689	0.849066
45	1	0	1.540022	-4.266502	1.665714
46	1	0	-0.227470	3.382295	-1.049965
47	1	0	0.540439	4.530630	-1.748736
48	1	0	2.150824	3.257533	-0.747718
49	1	0	3.563822	2.571142	-0.856416
50	1	0	2.473336	-2.874438	-1.714605
51	1	0	2.974675	-3.897239	-0.734369
52	8	0	4.554363	1.376206	-1.911549
53	1	0	4.192204	1.226622	-2.793121
54	1	0	4.704726	0.478526	-1.542431

Structure 11.6H$_2$O (B3LYP, DMSO)

Energy (Hartrees): = - 1395.1660804
No imaginary frequencies

Standard orientation:

Center Number	Atomic Number	Atomic Type	Coordinates (Angstroms) X	Y	Z
1	6	0	4.501184	-0.735373	-0.474326
2	6	0	4.067563	0.491816	0.059651
3	6	0	4.998145	1.531249	0.225662
4	6	0	6.328785	1.360391	-0.154055
5	6	0	6.747329	0.141935	-0.692148
6	6	0	5.831791	-0.905506	-0.845147
7	6	0	2.676858	0.737973	0.469320
8	7	0	1.694958	-0.034741	0.191155
9	6	0	0.374409	0.359454	0.672689
10	6	0	-0.420255	1.041917	-0.471924
11	8	0	-1.748378	1.334477	-0.011606
12	6	0	-2.535259	0.192078	0.374866
13	6	0	-1.845816	-0.515000	1.547941
14	6	0	-0.423156	-0.875468	1.107272
15	8	0	0.131966	2.232171	-0.923042
16	6	0	-3.941397	0.684893	0.677246
17	8	0	-4.640909	1.090216	-0.496783
18	8	0	-2.523178	-1.683595	1.977564
19	8	0	0.258795	-1.579318	2.145890
20	8	0	-3.056288	-3.076705	-0.270765
21	8	0	-0.644191	-3.807953	-1.464280
22	8	0	1.591993	-2.875402	-0.118256
23	8	0	-1.547345	3.948386	0.691095
24	8	0	-3.337142	3.369063	-1.454818
25	1	0	2.521735	1.658340	1.046888
26	1	0	-0.518650	-1.533941	0.232346
27	1	0	-0.436140	-2.130589	2.548779
28	1	0	-2.716421	-2.236614	1.170770
29	1	0	-1.800913	0.165584	2.408201
30	1	0	-2.584008	-0.497119	-0.476893
31	1	0	-4.505643	-0.136177	1.127033
32	1	0	-3.899514	1.500901	1.413113
33	1	0	-4.215404	1.913107	-0.838331
34	1	0	-0.025494	2.918711	-0.247605
35	1	0	-0.471953	0.365257	-1.335068
36	1	0	0.442713	1.074273	1.506964
37	1	0	3.802717	-1.559327	-0.570528
38	1	0	6.160431	-1.858305	-1.249793
39	1	0	7.784290	0.003554	-0.984258
40	1	0	7.037156	2.173597	-0.027137
41	1	0	4.669368	2.477108	0.648051
42	1	0	1.702432	-1.913465	-0.298140
43	1	0	1.326690	-2.837097	0.818616
44	1	0	-1.773735	3.022721	0.898921
45	1	0	-1.604795	4.439515	1.524290
46	1	0	0.164881	-3.470045	-1.009561
47	1	0	-0.627134	-4.761673	-1.299307
48	1	0	-2.217774	-3.292630	-0.739280
49	1	0	-3.579999	-2.514194	-0.880570
50	1	0	-2.595896	2.868880	-1.829266
51	1	0	-2.907727	3.806482	-0.693351

52	8	0	-4.545099	-1.313652	-1.940325
53	1	0	-4.033648	-1.137640	-2.743261
54	1	0	-4.645148	-0.429683	-1.519383

Structure 11.6H$_2$O (B3LYP, H$_2$O)

Energy (Hartrees): = - 1395.189459
No imaginary frequencies
Standard orientation:

Center Number	Atomic Number	Atomic Type	Coordinates (Angstroms) X	Y	Z
1	6	0	4.563483	-0.626052	-0.728796
2	6	0	4.204287	0.441495	0.114883
3	6	0	5.210239	1.281496	0.621847
4	6	0	6.548331	1.063213	0.296641
5	6	0	6.894855	0.002265	-0.542013
6	6	0	5.899933	-0.839909	-1.052119
7	6	0	2.816695	0.730432	0.498837
8	7	0	1.796996	0.052205	0.122595
9	6	0	0.488148	0.485666	0.602550
10	6	0	-0.393741	0.894114	-0.595263
11	8	0	-1.702873	1.237365	-0.130577
12	6	0	-2.415363	0.143985	0.481217
13	6	0	-1.649794	-0.321458	1.727921
14	6	0	-0.195276	-0.653683	1.369482
15	8	0	0.106587	1.996699	-1.295534
16	6	0	-3.827910	0.612737	0.801360
17	8	0	-4.675767	0.686303	-0.343701
18	8	0	-2.263619	-1.445329	2.348151
19	8	0	0.556505	-0.917475	2.553203
20	8	0	-2.822344	-3.246917	0.360898
21	8	0	-1.128685	-2.748548	-1.784603
22	8	0	1.548780	-2.407284	-1.280386
23	8	0	-2.506120	3.924435	0.256545
24	8	0	-3.831108	2.780168	-1.900308
25	1	0	2.692008	1.598697	1.157470
26	1	0	-0.203440	-1.545586	0.725573
27	1	0	0.027800	-1.558660	3.054606
28	1	0	-2.417555	-2.130842	1.644827
29	1	0	-1.647739	0.488928	2.467578
30	1	0	-2.471097	-0.682518	-0.237715
31	1	0	-4.277857	-0.110722	1.485272
32	1	0	-3.781889	1.583418	1.312906
33	1	0	-4.358508	1.420185	-0.929078
34	1	0	0.298620	2.700223	-0.654209
35	1	0	-0.469548	0.072069	-1.317172
36	1	0	0.569323	1.361093	1.265573
37	1	0	3.797780	-1.284488	-1.123734
38	1	0	6.170522	-1.665216	-1.703718
39	1	0	7.935785	-0.170896	-0.798172
40	1	0	7.316595	1.718489	0.695300
41	1	0	4.935565	2.106159	1.273866
42	1	0	1.652244	-1.540805	-0.808230
43	1	0	1.734093	-3.064387	-0.593100
44	1	0	-2.082789	3.043999	0.299886
45	1	0	-3.159670	3.895930	0.971121
46	1	0	-0.164303	-2.638972	-1.595665
47	1	0	-1.178882	-3.537325	-2.342975
48	1	0	-2.119132	-3.150677	-0.322980
49	1	0	-3.583410	-2.801581	-0.075601
50	1	0	-3.054029	2.370311	-2.309120
51	1	0	-3.433835	3.310677	-1.169849
52	8	0	-4.707427	-1.923060	-1.263392
53	1	0	-4.044265	-1.958289	-1.968301
54	1	0	-4.716107	-0.971310	-1.004177

Structure 11.6H$_2$O (M06-2X, Gas Phase)

Energy (Hartrees): = - 1394.96972
No imaginary frequencies

Standard orientation:

Center Number	Atomic Number	Atomic Type	Coordinates (Angstroms) X	Y	Z
1	6	0	4.427007	-0.835227	-0.430425
2	6	0	4.016414	0.406896	0.064969
3	6	0	4.952255	1.431102	0.214980
4	6	0	6.281975	1.228676	-0.133093
5	6	0	6.683763	-0.006490	-0.626701
6	6	0	5.755443	-1.035974	-0.771581
7	6	0	2.620856	0.674550	0.449128

8	7	0	1.665943	-0.135822	0.257090
9	6	0	0.337029	0.269207	0.674794
10	6	0	-0.420083	0.890551	-0.511508
11	8	0	-1.733141	1.221770	-0.068736
12	6	0	-2.540469	0.111686	0.322491
13	6	0	-1.888527	-0.560586	1.528825
14	6	0	-0.462335	-0.942756	1.145009
15	8	0	0.149950	2.052010	-0.999154
16	6	0	-3.924295	0.660310	0.596829
17	8	0	-4.514889	1.192224	-0.570118
18	8	0	-2.583777	-1.693836	1.981196
19	8	0	0.203475	-1.568661	2.221009
20	8	0	-2.993626	-2.978320	-0.372631
21	8	0	-0.638858	-2.523003	-1.826481
22	8	0	1.549518	-2.890402	-0.226653
23	8	0	-1.162107	3.649332	0.941506
24	8	0	-2.953994	3.420725	-1.334183
25	1	0	2.444744	1.645184	0.930878
26	1	0	-0.542998	-1.637683	0.300684
27	1	0	-0.471618	-2.106485	2.655461
28	1	0	-2.754297	-2.267023	1.198859
29	1	0	-1.856055	0.150868	2.365443
30	1	0	-2.588177	-0.598384	-0.510770
31	1	0	-4.556855	-0.154010	0.952708
32	1	0	-3.864833	1.419427	1.389489
33	1	0	-4.022519	1.990100	-0.843917
34	1	0	0.097414	2.729428	-0.306487
35	1	0	-0.474133	0.169908	-1.335010
36	1	0	0.381462	1.020702	1.479401
37	1	0	3.711224	-1.641707	-0.534254
38	1	0	6.072419	-2.000383	-1.149531
39	1	0	7.720318	-0.171214	-0.895850
40	1	0	7.001553	2.029830	-0.017183
41	1	0	4.632509	2.392927	0.602487
42	1	0	1.689411	-1.922268	-0.147522
43	1	0	1.336182	-3.139428	0.679591
44	1	0	-1.601201	2.783240	0.933897
45	1	0	-1.138296	3.956760	1.849890
46	1	0	0.162954	-2.812241	-1.344697
47	1	0	-0.505717	-2.764195	-2.745199
48	1	0	-2.215334	-2.949576	-0.954203
49	1	0	-3.694855	-2.508858	-0.855864
50	1	0	-2.176356	2.917749	-1.604936
51	1	0	-2.607893	3.963999	-0.616418
52	8	0	-4.859048	-1.312247	-1.655286
53	1	0	-5.799706	-1.499052	-1.651688
54	1	0	-4.783174	-0.355265	-1.490939

Structure 11.6H$_2$O (M06-2X, DMSO)

Energy (Hartrees): = - 1395.0093753
No imaginary frequencies

Standard orientation:

Center Number	Atomic Number	Atomic Type	Coordinates (Angstroms) X	Y	Z
1	6	0	4.430622	-0.819270	-0.470642
2	6	0	4.018332	0.409852	0.056762
3	6	0	4.955423	1.429901	0.236776
4	6	0	6.285917	1.234705	-0.117526
5	6	0	6.688303	0.012563	-0.645330
6	6	0	5.759622	-1.013996	-0.817297
7	6	0	2.622144	0.674632	0.446205
8	7	0	1.661057	-0.118603	0.203101
9	6	0	0.337501	0.292126	0.641918
10	6	0	-0.427455	0.924808	-0.533785
11	8	0	-1.734776	1.260994	-0.088350
12	6	0	-2.537757	0.157628	0.321279
13	6	0	-1.876973	-0.517615	1.520342
14	6	0	-0.464590	-0.918085	1.109608
15	8	0	0.145360	2.085358	-1.019559
16	6	0	-3.913077	0.716733	0.612082
17	8	0	-4.506570	1.254121	-0.558621
18	8	0	-2.584730	-1.647591	1.976722
19	8	0	0.209907	-1.563779	2.174179
20	8	0	-3.101373	-2.993038	-0.323682
21	8	0	-0.678524	-3.175638	-1.715525
22	8	0	1.549112	-2.929860	-0.082637
23	8	0	-1.144756	3.729297	0.874848
24	8	0	-2.915715	3.470047	-1.361223
25	1	0	2.454017	1.621324	0.972881
26	1	0	-0.562552	-1.610438	0.261653
27	1	0	-0.462191	-2.103685	2.612556
28	1	0	-2.778285	-2.209482	1.192840
29	1	0	-1.825155	0.193630	2.353643

30	1	0	-2.599364	-0.558218	-0.508322
31	1	0	-4.559589	-0.083755	0.974461
32	1	0	-3.837773	1.479404	1.397823
33	1	0	-3.990786	2.036410	-0.832692
34	1	0	0.057189	2.767037	-0.331690
35	1	0	-0.488670	0.213458	-1.366362
36	1	0	0.395523	1.034869	1.451286
37	1	0	3.716656	-1.624870	-0.593429
38	1	0	6.077497	-1.969001	-1.219229
39	1	0	7.725358	-0.145567	-0.918392
40	1	0	7.006464	2.032167	0.020721
41	1	0	4.633877	2.379178	0.652926
42	1	0	1.683994	-1.962179	-0.158828
43	1	0	1.288045	-3.006912	0.844542
44	1	0	-1.535963	2.886746	1.145399
45	1	0	-0.986538	4.236768	1.678758
46	1	0	0.122872	-3.197215	-1.155454
47	1	0	-0.526641	-2.447081	-2.326869
48	1	0	-2.291060	-3.095647	-0.854739
49	1	0	-3.708724	-2.447556	-0.854175
50	1	0	-2.204768	2.884531	-1.650307
51	1	0	-2.522338	3.865150	-0.569506
52	8	0	-4.823031	-1.250536	-1.715770
53	1	0	-5.757349	-1.450797	-1.598026
54	1	0	-4.738443	-0.306940	-1.488580

Structure 11.6H_2O (M06-2X, H_2O)

Energy (Hartrees): = - 1395.0313857
No imaginary frequencies

Standard orientation:

Center Number	Atomic Number	Atomic Type	Coordinates (Angstroms) X	Y	Z
1	6	0	4.401494	-0.898006	-0.539011
2	6	0	4.038633	0.314955	0.057970
3	6	0	5.023251	1.264269	0.340466
4	6	0	6.355988	1.008818	0.035326
5	6	0	6.710466	-0.198747	-0.556339
6	6	0	5.731207	-1.150062	-0.843094
7	6	0	2.646196	0.638211	0.409476
8	7	0	1.670948	-0.160198	0.239137
9	6	0	0.351090	0.330582	0.603000
10	6	0	-0.366625	0.835520	-0.657434
11	8	0	-1.675904	1.263412	-0.297636
12	6	0	-2.519211	0.239949	0.233829
13	6	0	-1.896713	-0.311354	1.516287
14	6	0	-0.476904	-0.781131	1.228728
15	8	0	0.273304	1.926950	-1.237415
16	6	0	-3.880745	0.854317	0.480589
17	8	0	-4.562308	1.159586	-0.727719
18	8	0	-2.657334	-1.378396	2.052474
19	8	0	0.166641	-1.207692	2.417092
20	8	0	-3.056928	-3.055936	-0.081870
21	8	0	-0.942459	-2.557609	-1.811944
22	8	0	1.449683	-2.899164	-0.462637
23	8	0	-0.550671	3.630949	0.848459
24	8	0	-3.043371	3.423701	-1.416941
25	1	0	2.491116	1.631090	0.846379
26	1	0	-0.547587	-1.616842	0.519025
27	1	0	-0.438770	-1.822894	2.849880
28	1	0	-2.773050	-2.041690	1.336134
29	1	0	-1.867509	0.480016	2.274285
30	1	0	-2.607669	-0.567249	-0.504916
31	1	0	-4.492180	0.135527	1.027114
32	1	0	-3.761371	1.754455	1.095322
33	1	0	-4.139708	1.946798	-1.115003
34	1	0	0.261064	2.643428	-0.574648
35	1	0	-0.429114	0.043809	-1.410446
36	1	0	0.414614	1.174422	1.303869
37	1	0	3.645268	-1.642168	-0.757624
38	1	0	6.009073	-2.089859	-1.305235
39	1	0	7.747850	-0.401823	-0.795559
40	1	0	7.114208	1.750173	0.256991
41	1	0	4.738663	2.203689	0.802704
42	1	0	1.597142	-1.954429	-0.235762
43	1	0	1.223293	-3.308786	0.380252
44	1	0	-1.225142	2.936445	0.867463
45	1	0	-0.059669	3.507043	1.668415
46	1	0	-0.081668	-2.748745	-1.388557
47	1	0	-0.938694	-3.053578	-2.637219
48	1	0	-2.296519	-2.925303	-0.681605
49	1	0	-3.778623	-2.543617	-0.493262

50	1	0	-2.322889	2.812494	-1.196772
51	1	0	-3.145845	3.949511	-0.615362
52	8	0	-5.151343	-1.539373	-1.191720
53	1	0	-5.820331	-1.546586	-0.497694
54	1	0	-4.915846	-0.599287	-1.272022

Structure 11 min 2 $\Theta_{H\text{-}O\text{-}C1\text{-}C2}$ = 286.43 (M06-2X, Gas Phase)

Energy (Hartrees): = - 936.3454645
No imaginary frequencies

Standard orientation:

Center Number	Atomic Number	Atomic Type	Coordinates (Angstroms) X	Y	Z
1	6	0	1.036735	1.362985	0.389233
2	6	0	0.446209	0.140075	-0.337113
3	6	0	1.308524	-1.083342	-0.052536
4	6	0	2.751030	-0.801384	-0.408721
5	6	0	3.222983	0.429850	0.359628
6	1	0	0.946316	1.231754	1.474505
7	1	0	1.253959	-1.306712	1.022477
8	1	0	2.815979	-0.586425	-1.485481
9	1	0	3.159647	0.224317	1.438418
10	1	0	0.474797	0.354928	-1.417942
11	8	0	2.388124	1.524808	0.014100
12	6	0	4.659081	0.816992	0.032715
13	1	0	4.770883	0.883138	-1.058951
14	1	0	4.849276	1.805943	0.452270
15	8	0	5.582613	-0.074609	0.608020
16	1	0	5.317249	-0.965040	0.352026
17	8	0	3.577362	-1.900014	-0.074185
18	1	0	3.152641	-2.688168	-0.430144
19	8	0	0.904961	-2.210216	-0.806250
20	1	0	0.010305	-2.437549	-0.534541
21	8	0	0.363796	2.529896	0.067638
22	1	0	0.624393	2.763333	-0.829975
23	7	0	-0.894224	-0.123181	0.134373
24	6	0	-3.270258	0.069608	-0.195773
25	6	0	-3.605255	-0.524722	1.023931
26	6	0	-4.281552	0.480810	-1.062718
27	6	0	-4.936211	-0.705444	1.363826
28	1	0	-2.806148	-0.832047	1.687428
29	6	0	-5.616705	0.299520	-0.720533
30	1	0	-4.020106	0.945914	-2.007679
31	6	0	-5.944091	-0.294680	0.492092
32	1	0	-5.194458	-1.165623	2.309987
33	1	0	-6.398450	0.621753	-1.397493
34	1	0	-6.983800	-0.437245	0.761519
35	6	0	-1.862120	0.263832	-0.584611
36	1	0	-1.697983	0.766478	-1.548134

Structure 11 min 2 $\Theta_{H\text{-}O\text{-}C1\text{-}C2}$ = 305.41 (M06-2X, DMSO)

Energy (Hartrees): = - 936.3730305
No imaginary frequencies

Standard orientation:

Center Number	Atomic Number	Atomic Type	Coordinates (Angstroms) X	Y	Z
1	6	0	1.108554	1.442366	0.335639
2	6	0	0.451080	0.212573	-0.306355
3	6	0	1.258834	-1.034915	0.045759
4	6	0	2.709229	-0.840567	-0.341757
5	6	0	3.248643	0.422510	0.324558
6	1	0	1.060071	1.366802	1.428618
7	1	0	1.207861	-1.194878	1.131219
8	1	0	2.776530	-0.719587	-1.431947
9	1	0	3.196553	0.298986	1.416346
10	1	0	0.469417	0.352212	-1.399183
11	8	0	2.459348	1.529237	-0.083993
12	6	0	4.687442	0.724045	-0.068969
13	1	0	4.759998	0.740589	-1.165167
14	1	0	4.948848	1.716314	0.303576
15	8	0	5.595715	-0.197010	0.500986
16	1	0	5.246253	-1.077081	0.316164
17	8	0	3.492337	-1.947012	0.079801
18	1	0	3.056943	-2.742740	-0.249204
19	8	0	0.797767	-2.182674	-0.644474
20	1	0	-0.071288	-2.410977	-0.295143
21	8	0	0.471256	2.619149	-0.012738
22	1	0	0.440875	2.661284	-0.977540

23	7	0	-0.893741	0.057284	0.204827
24	6	0	-3.264227	0.016013	-0.215489
25	6	0	-3.620679	-0.267599	1.107147
26	6	0	-4.261355	0.168391	-1.180238
27	6	0	-4.957379	-0.394496	1.454501
28	1	0	-2.840958	-0.386956	1.850516
29	6	0	-5.601939	0.041754	-0.830150
30	1	0	-3.982805	0.387792	-2.206082
31	6	0	-5.950681	-0.239588	0.486535
32	1	0	-5.230104	-0.614643	2.480277
33	1	0	-6.371721	0.162504	-1.583534
34	1	0	-6.994509	-0.338768	0.761634
35	6	0	-1.852230	0.159577	-0.619129
36	1	0	-1.678922	0.364267	-1.683312

Structure 11 min 2 $\theta_{H\text{-}O\text{-}C1\text{-}C2}$ = 287.01 (M06-2X/def2-TZVP, Gas Phase)

Energy (Hartrees): = -936.462722
No imaginary frequencies

Standard orientation:

Center Number	Atomic Number	Atomic Type	Coordinates (Angstroms) X	Y	Z
1	6	0	1.056263	1.376564	0.375126
2	6	0	0.445063	0.149948	-0.320458
3	6	0	1.294115	-1.074834	-0.008661
4	6	0	2.737486	-0.820002	-0.377912
5	6	0	3.231907	0.427363	0.346260
6	1	0	0.978358	1.262783	1.462777
7	1	0	1.240135	-1.270039	1.070702
8	1	0	2.799411	-0.638976	-1.459902
9	1	0	3.187192	0.253995	1.430936
10	1	0	0.474289	0.340455	-1.405042
11	8	0	2.403093	1.518338	-0.013576
12	6	0	4.660934	0.796248	-0.023852
13	1	0	4.748874	0.829849	-1.117670
14	1	0	4.867019	1.795373	0.360575
15	8	0	5.599931	-0.078086	0.550834
16	1	0	5.331280	-0.981222	0.342552
17	8	0	3.552901	-1.917390	-0.018054
18	1	0	3.122712	-2.717160	-0.343198
19	8	0	0.871627	-2.214519	-0.730396
20	1	0	-0.025448	-2.432215	-0.454196
21	8	0	0.393206	2.546187	0.047024
22	1	0	0.627472	2.770940	-0.861650
23	7	0	-0.894275	-0.081060	0.159032
24	6	0	-3.269443	0.050957	-0.203217
25	6	0	-3.616699	-0.418146	1.063632
26	6	0	-4.270152	0.363550	-1.117933
27	6	0	-4.948495	-0.574005	1.401875
28	1	0	-2.827460	-0.648905	1.767231
29	6	0	-5.606280	0.206714	-0.778084
30	1	0	-3.998145	0.730971	-2.100934
31	6	0	-5.945403	-0.262708	0.481790
32	1	0	-5.216999	-0.935663	2.385991
33	1	0	-6.380035	0.450365	-1.494273
34	1	0	-6.986636	-0.385362	0.750917
35	6	0	-1.861516	0.223238	-0.594630
36	1	0	-1.694825	0.636663	-1.598315

Structure 11 min 2 $\theta_{H\text{-}O\text{-}C1\text{-}C2}$ = 308.85 (M06-2X/def2-TZVP, DMSO)

Energy (Hartrees): = -936.490485
No imaginary frequencies

Standard orientation:

Center Number	Atomic Number	Atomic Type	Coordinates (Angstroms) X	Y	Z
1	6	0	1.141054	1.480060	0.245397
2	6	0	0.451690	0.228732	-0.307346
3	6	0	1.231945	-1.006612	0.136857
4	6	0	2.684691	-0.875322	-0.265082
5	6	0	3.258996	0.423445	0.291384
6	1	0	1.106365	1.473433	1.340998
7	1	0	1.175671	-1.084991	1.229925
8	1	0	2.748931	-0.842698	-1.360604
9	1	0	3.224324	0.388396	1.389639
10	1	0	0.475631	0.286336	-1.406605
11	8	0	2.487160	1.509605	-0.189000

12	6	0	4.692650	0.665972	-0.153428
13	1	0	4.745222	0.590395	-1.246795
14	1	0	4.978566	1.680050	0.128791
15	8	0	5.604596	-0.215986	0.468179
16	1	0	5.247428	-1.108734	0.373740
17	8	0	3.448274	-1.959809	0.237463
18	1	0	3.009796	-2.777445	-0.031597
19	8	0	0.739466	-2.188450	-0.465852
20	1	0	-0.136920	-2.375127	-0.106038
21	8	0	0.528406	2.648454	-0.167713
22	1	0	0.430323	2.620252	-1.130085
23	7	0	-0.894310	0.143824	0.206809
24	6	0	-3.258829	-0.017977	-0.216534
25	6	0	-3.638569	0.009361	1.126558
26	6	0	-4.235608	-0.127186	-1.203736
27	6	0	-4.976800	-0.072603	1.470535
28	1	0	-2.877828	0.098889	1.891881
29	6	0	-5.577912	-0.210512	-0.857697
30	1	0	-3.937775	-0.145599	-2.246263
31	6	0	-5.949036	-0.182147	0.479240
32	1	0	-5.268743	-0.049358	2.513168
33	1	0	-6.331689	-0.296623	-1.630253
34	1	0	-6.995173	-0.244133	0.752707
35	6	0	-1.846344	0.075283	-0.624362
36	1	0	-1.664752	0.086713	-1.705609

Structure 11 min 3 $\Theta_{H\text{-}O\text{-}C1\text{-}C2}$ = 61.43 (M06-2X, Gas Phase)

Energy (Hartrees): = - 936.3424632
No imaginary frequencies

Standard orientation:

Center Number	Atomic Number	Atomic Type	Coordinates (Angstroms) X	Y	Z
1	6	0	1.173608	1.490954	0.199612
2	6	0	0.458468	0.235353	-0.317140
3	6	0	1.219335	-0.994485	0.169689
4	6	0	2.669234	-0.885229	-0.257214
5	6	0	3.261546	0.427951	0.253350
6	1	0	1.159008	1.484134	1.303540
7	1	0	1.180459	-1.034286	1.266960
8	1	0	2.710016	-0.880713	-1.356029
9	1	0	3.238992	0.422931	1.353761
10	1	0	0.491303	0.268107	-1.415888
11	8	0	2.495735	1.506504	-0.256377
12	6	0	4.703527	0.631956	-0.191940
13	1	0	4.766061	0.480907	-1.278743
14	1	0	4.981946	1.664708	0.021684
15	8	0	5.590724	-0.203819	0.511890
16	1	0	5.252766	-1.103153	0.439502
17	8	0	3.426427	-1.962359	0.261463
18	1	0	2.952678	-2.770422	0.036446
19	8	0	0.716658	-2.196772	-0.383954
20	1	0	-0.142696	-2.374661	0.008535
21	8	0	0.589568	2.649556	-0.302612
22	1	0	-0.320204	2.662257	0.010220
23	7	0	-0.892619	0.205576	0.195615
24	6	0	-3.251127	-0.036541	-0.229914
25	6	0	-3.623324	0.075218	1.112763
26	6	0	-4.232218	-0.209448	-1.205868
27	6	0	-4.961274	0.013563	1.467325
28	1	0	-2.848213	0.209350	1.857663
29	6	0	-5.574275	-0.270720	-0.848715
30	1	0	-3.941283	-0.295156	-2.247791
31	6	0	-5.938655	-0.159044	0.487489
32	1	0	-5.249179	0.100462	2.508066
33	1	0	-6.332525	-0.404341	-1.610623
34	1	0	-6.983923	-0.205890	0.769137
35	6	0	-1.835344	0.027301	-0.634425
36	1	0	-1.636778	-0.089116	-1.709086

Structure 11 min 3 $\Theta_{H\text{-}O\text{-}C1\text{-}C2}$= 57.45 (M06-2X, DMSO)

Energy (Hartrees): = - 936.3701739
No imaginary frequencies

Standard orientation:

Center Number	Atomic Number	Atomic Type	Coordinates (Angstroms) X	Y	Z

1	6	0	1.171275	1.490965	0.201653
2	6	0	0.459060	0.239009	-0.320883
3	6	0	1.217182	-0.993546	0.169965
4	6	0	2.671434	-0.882179	-0.242700
5	6	0	3.262959	0.433311	0.261917
6	1	0	1.156358	1.482790	1.304105
7	1	0	1.164184	-1.037073	1.265818
8	1	0	2.728087	-0.892381	-1.340158
9	1	0	3.236886	0.436311	1.361549
10	1	0	0.487260	0.266806	-1.419366
11	8	0	2.499073	1.514291	-0.251617
12	6	0	4.699754	0.636741	-0.196860
13	1	0	4.749870	0.503035	-1.286096
14	1	0	4.995564	1.661808	0.034165
15	8	0	5.594805	-0.225813	0.476363
16	1	0	5.221457	-1.113050	0.409761
17	8	0	3.432058	-1.951362	0.298867
18	1	0	2.982118	-2.769448	0.055149
19	8	0	0.720701	-2.192566	-0.397781
20	1	0	-0.119916	-2.401187	0.024933
21	8	0	0.592745	2.657037	-0.296641
22	1	0	-0.337269	2.638704	-0.039010
23	7	0	-0.893169	0.217061	0.194765
24	6	0	-3.250386	-0.046205	-0.225403
25	6	0	-3.630460	0.075249	1.115302
26	6	0	-4.227992	-0.221445	-1.206469
27	6	0	-4.971638	0.020469	1.463973
28	1	0	-2.866429	0.210179	1.872068
29	6	0	-5.573173	-0.274367	-0.855243
30	1	0	-3.929228	-0.315057	-2.245669
31	6	0	-5.945256	-0.153650	0.479350
32	1	0	-5.264078	0.113262	2.503500
33	1	0	-6.328033	-0.409068	-1.621176
34	1	0	-6.992666	-0.194689	0.755989
35	6	0	-1.834154	0.011823	-0.632828
36	1	0	-1.638657	-0.130112	-1.703090

Structure 11 min 3 $\theta_{H\text{-}O\text{-}C1\text{-}C2}$ = 64.63 (M06-2X/def2-TZVP, Gas Phase)

Energy (Hartrees): = -936.460350
No imaginary frequencies

Standard orientation:

Center Number	Atomic Number	Atomic Type	Coordinates (Angstroms) X	Y	Z
1	6	0	1.138903	1.451571	0.225614
2	6	0	0.455460	0.188888	-0.312693
3	6	0	1.247379	-1.025320	0.155783
4	6	0	2.693469	-0.874883	-0.266607
5	6	0	3.254137	0.442501	0.264091
6	1	0	1.117629	1.430182	1.327929
7	1	0	1.206408	-1.080637	1.251345
8	1	0	2.736453	-0.850164	-1.363914
9	1	0	3.237643	0.418543	1.363610
10	1	0	0.488587	0.240266	-1.409928
11	8	0	2.460233	1.509002	-0.221025
12	6	0	4.684458	0.695307	-0.189309
13	1	0	4.744910	0.561232	-1.276953
14	1	0	4.934971	1.732063	0.034976
15	8	0	5.608072	-0.118517	0.491133
16	1	0	5.308288	-1.032458	0.417750
17	8	0	3.477652	-1.940642	0.231659
18	1	0	3.027964	-2.762889	0.004149
19	8	0	0.771008	-2.230300	-0.411683
20	1	0	-0.107029	-2.411672	-0.060654
21	8	0	0.532304	2.605552	-0.260718
22	1	0	-0.366840	2.632089	0.083800
23	7	0	-0.891651	0.121785	0.197233
24	6	0	-3.256290	-0.030621	-0.228320
25	6	0	-3.626909	0.002496	1.116457
26	6	0	-4.238531	-0.124375	-1.209759
27	6	0	-4.963391	-0.057485	1.466408
28	1	0	-2.852925	0.077752	1.869287
29	6	0	-5.579363	-0.185458	-0.857536
30	1	0	-3.948525	-0.148226	-2.254172
31	6	0	-5.941589	-0.151307	0.480496
32	1	0	-5.250053	-0.030438	2.509819
33	1	0	-6.338330	-0.259487	-1.625248
34	1	0	-6.986575	-0.197012	0.759240
35	6	0	-1.842987	0.037595	-0.633304
36	1	0	-1.653648	0.014140	-1.714515

Structure 11 min 3 $\theta_{H\text{-}O\text{-}C1\text{-}C2}$ = 58.81 (M06-2X/def2-TZVP, DMSO)

Energy (Hartrees): = -936.488019
No imaginary frequencies

Standard orientation:

Center Number	Atomic Number	Atomic Type	Coordinates (Angstroms) X	Y	Z
1	6	0	1.143662	1.457011	0.223645
2	6	0	0.456876	0.197536	-0.310518
3	6	0	1.241706	-1.018531	0.171881
4	6	0	2.691450	-0.876487	-0.242054
5	6	0	3.259097	0.446233	0.267679
6	1	0	1.127135	1.440160	1.324782
7	1	0	1.189675	-1.067451	1.266764
8	1	0	2.746496	-0.877399	-1.338570
9	1	0	3.245744	0.441193	1.366741
10	1	0	0.486980	0.234534	-1.408008
11	8	0	2.468314	1.513779	-0.226382
12	6	0	4.682141	0.688853	-0.210190
13	1	0	4.721783	0.563383	-1.299361
14	1	0	4.956488	1.718598	0.022211
15	8	0	5.614221	-0.150875	0.439107
16	1	0	5.274338	-1.053263	0.379356
17	8	0	3.476572	-1.932468	0.286984
18	1	0	3.052249	-2.764847	0.041469
19	8	0	0.763689	-2.223071	-0.396779
20	1	0	-0.103927	-2.418407	-0.020933
21	8	0	0.544292	2.617815	-0.261982
22	1	0	-0.383699	2.601380	0.008472
23	7	0	-0.892026	0.146160	0.201383
24	6	0	-3.255120	-0.037907	-0.224548
25	6	0	-3.639257	0.037858	1.115496
26	6	0	-4.228989	-0.169047	-1.212074
27	6	0	-4.979620	-0.017098	1.456135
28	1	0	-2.880458	0.141025	1.880983
29	6	0	-5.573550	-0.223967	-0.869209
30	1	0	-3.926937	-0.226448	-2.251912
31	6	0	-5.949136	-0.147298	0.464532
32	1	0	-5.275446	0.041989	2.496206
33	1	0	-6.325409	-0.326191	-1.641594
34	1	0	-6.996961	-0.188295	0.735561
35	6	0	-1.840742	0.022605	-0.629464
36	1	0	-1.651685	-0.043090	-1.707289

Structure 12 (B3LYP, Gas Phase)

Energy (Hartrees): = - 1051.0036157
No imaginary frequencies

Standard orientation:

Center Number	Atomic Number	Atomic Type	Coordinates (Angstroms) X	Y	Z
1	6	0	-1.892256	1.389342	-0.376844
2	6	0	-1.249700	0.186350	0.337783
3	6	0	-2.088106	-1.064065	0.035385
4	6	0	-3.555301	-0.835383	0.368171
5	6	0	-4.071071	0.403772	-0.375409
6	1	0	-1.835238	1.245414	-1.471590
7	1	0	-2.008924	-1.278920	-1.041953
8	1	0	-3.646134	-0.649937	1.450306
9	1	0	-4.007167	0.222736	-1.460930
10	1	0	-1.295988	0.403894	1.417116
11	8	0	-3.260702	1.518650	-0.000610
12	6	0	-5.523828	0.746079	-0.032215
13	1	0	-5.619519	0.836435	1.062864
14	1	0	-5.761330	1.723931	-0.462535
15	8	0	-6.433147	-0.188689	-0.577373
16	1	0	-6.111902	-1.069605	-0.327043
17	8	0	-4.339365	-1.965251	-0.008299
18	1	0	-3.891555	-2.741177	0.360049
19	8	0	-1.664193	-2.194463	0.790510
20	1	0	-0.750519	-2.379967	0.531922
21	8	0	-1.225743	2.540179	0.028871
22	1	0	-1.656027	3.291732	-0.401874
23	7	0	0.104450	-0.037620	-0.131852
24	6	0	2.477794	0.117281	0.285329
25	6	0	2.868170	-0.387738	-0.971343
26	6	0	3.475537	0.465975	1.202491

27	6	0	4.205255	-0.537471	-1.288331
28	1	0	2.096077	-0.654062	-1.685331
29	6	0	4.829035	0.321445	0.897351
30	1	0	3.191722	0.859838	2.175386
31	6	0	5.198410	-0.184150	-0.355117
32	1	0	4.519646	-0.923922	-2.252164
33	1	0	5.575823	0.601646	1.630129
34	6	0	1.067262	0.280949	0.647196
35	1	0	0.881037	0.703686	1.646810
36	8	0	6.481225	-0.371219	-0.766674
37	6	0	7.534806	-0.033365	0.124967
38	1	0	7.487911	-0.625868	1.047034
39	1	0	8.460380	-0.263604	-0.403825
40	1	0	7.519922	1.033307	0.380710

Structure 12 (B3LYP, DMSO)

Energy (Hartrees): = - 1051.0288449
No imaginary frequencies

Standard orientation:

Center Number	Atomic Number	Atomic Type	Coordinates (Angstroms) X	Y	Z
1	6	0	-1.979032	1.492707	-0.243136
2	6	0	-1.255570	0.262361	0.329346
3	6	0	-2.019166	-1.002985	-0.104596
4	6	0	-3.495718	-0.899925	0.255570
5	6	0	-4.096486	0.387408	-0.324216
6	1	0	-1.941846	1.467487	-1.345871
7	1	0	-1.935289	-1.101696	-1.197068
8	1	0	-3.589311	-0.862989	1.351704
9	1	0	-4.044284	0.343576	-1.423928
10	1	0	-1.293016	0.341378	1.427444
11	8	0	-3.347893	1.498423	0.169933
12	6	0	-5.553551	0.602452	0.092580
13	1	0	-5.613061	0.597287	1.193410
14	1	0	-5.876436	1.591674	-0.248591
15	8	0	-6.427891	-0.353767	-0.488660
16	1	0	-6.007899	-1.219280	-0.349788
17	8	0	-4.220657	-2.018687	-0.263267
18	1	0	-3.762395	-2.812637	0.053644
19	8	0	-1.525326	-2.177451	0.535459
20	1	0	-0.639921	-2.349727	0.181426
21	8	0	-1.375599	2.637450	0.264752
22	1	0	-1.778405	3.399585	-0.179507
23	7	0	0.101600	0.191991	-0.179626
24	6	0	2.470807	0.076606	0.288894
25	6	0	2.884518	0.051659	-1.058855
26	6	0	3.452993	0.021748	1.287138
27	6	0	4.226187	-0.027606	-1.386675
28	1	0	2.134921	0.096024	-1.842554
29	6	0	4.809621	-0.058225	0.973334
30	1	0	3.152289	0.041567	2.331712
31	6	0	5.202990	-0.084727	-0.372153
32	1	0	4.550003	-0.047138	-2.422644
33	1	0	5.542311	-0.099725	1.770146
34	6	0	1.057813	0.158421	0.671302
35	1	0	0.865973	0.193085	1.753873
36	8	0	6.488160	-0.165040	-0.796813
37	6	0	7.524697	-0.234642	0.186426
38	1	0	7.410222	-1.116920	0.825855
39	1	0	8.459449	-0.312247	-0.370694
40	1	0	7.548510	0.667822	0.807827

Structure 12 (M06-2X, Gas Phase)

Energy (Hartrees): = - 1050.8587012
No imaginary frequencies

Standard orientation:

Center Number	Atomic Number	Atomic Type	Coordinates (Angstroms) X	Y	Z
1	6	0	-1.838315	1.336368	-0.384972
2	6	0	-1.250138	0.146487	0.374844
3	6	0	-2.106688	-1.083174	0.100795
4	6	0	-3.559849	-0.799699	0.411022
5	6	0	-4.018686	0.412949	-0.394549
6	1	0	-1.763142	1.160023	-1.470946
7	1	0	-2.023483	-1.335161	-0.966307
8	1	0	-3.655746	-0.562911	1.480489

9	1	0	-3.925190	0.185145	-1.467450
10	1	0	-1.302756	0.402347	1.442961
11	8	0	-3.198540	1.515615	-0.042205
12	6	0	-5.465895	0.794353	-0.114290
13	1	0	-5.608825	0.877689	0.972215
14	1	0	-5.653252	1.774856	-0.554968
15	8	0	-6.366159	-0.114375	-0.700748
16	1	0	-6.098784	-0.997927	-0.423328
17	8	0	-4.371205	-1.910776	0.076337
18	1	0	-3.951848	-2.688581	0.460306
19	8	0	-1.718398	-2.188957	0.892316
20	1	0	-0.804284	-2.394058	0.671128
21	8	0	-1.141950	2.467705	0.002419
22	1	0	-1.518661	3.220708	-0.461663
23	7	0	0.101482	-0.129676	-0.065400
24	6	0	2.463515	0.141346	0.280243
25	6	0	2.833441	-0.551159	-0.882211
26	6	0	3.461321	0.643842	1.107110
27	6	0	4.161861	-0.732913	-1.194940
28	1	0	2.052140	-0.935701	-1.526422
29	6	0	4.809165	0.468431	0.804937
30	1	0	3.186994	1.185779	2.006444
31	6	0	5.161219	-0.225041	-0.351721
32	1	0	4.470474	-1.264152	-2.086597
33	1	0	5.561064	0.871172	1.469102
34	6	0	1.051599	0.336482	0.634045
35	1	0	0.860179	0.916266	1.547000
36	8	0	6.432947	-0.459592	-0.746439
37	6	0	7.478904	0.036263	0.064766
38	1	0	7.442342	-0.402064	1.066990
39	1	0	8.403734	-0.257137	-0.425793
40	1	0	7.436381	1.127267	0.140721

Structure 12 (M06-2X, DMSO)

Energy (Hartrees): = - 1050.8867989
No imaginary frequencies

Standard orientation:

Center Number	Atomic Number	Atomic Type	Coordinates (Angstroms) X	Y	Z
1	6	0	-1.962517	1.485406	-0.191745
2	6	0	-1.255682	0.252693	0.368671
3	6	0	-2.009122	-1.000523	-0.079493
4	6	0	-3.476606	-0.889561	0.278101
5	6	0	-4.052955	0.391806	-0.317979
6	1	0	-1.912795	1.475522	-1.291605
7	1	0	-1.923800	-1.094163	-1.170550
8	1	0	-3.575235	-0.841235	1.371438
9	1	0	-3.958403	0.351149	-1.413430
10	1	0	-1.290407	0.319906	1.465545
11	8	0	-3.325238	1.491956	0.203154
12	6	0	-5.517929	0.595962	0.039047
13	1	0	-5.637398	0.508651	1.127691
14	1	0	-5.811063	1.605710	-0.254671
15	8	0	-6.357448	-0.308163	-0.652076
16	1	0	-5.978275	-1.185926	-0.521538
17	8	0	-4.202411	-1.997884	-0.232756
18	1	0	-3.743911	-2.794832	0.059898
19	8	0	-1.519910	-2.168473	0.555378
20	1	0	-0.638597	-2.350062	0.209429
21	8	0	-1.355027	2.608898	0.339038
22	1	0	-1.728505	3.382079	-0.099643
23	7	0	0.096461	0.188656	-0.142534
24	6	0	2.456561	0.047669	0.302177
25	6	0	2.844875	0.061691	-1.046297
26	6	0	3.442965	-0.031978	1.281692
27	6	0	4.177421	-0.002108	-1.392895
28	1	0	2.084321	0.127067	-1.815810
29	6	0	4.793418	-0.096733	0.948452
30	1	0	3.154796	-0.042497	2.328323
31	6	0	5.164515	-0.080483	-0.396940
32	1	0	4.490787	0.010199	-2.430325
33	1	0	5.534605	-0.158809	1.733938
34	6	0	1.043448	0.123358	0.700542
35	1	0	0.853128	0.126212	1.781529
36	8	0	6.438184	-0.136023	-0.835784
37	6	0	7.472759	-0.197465	0.138511
38	1	0	7.385550	-1.100326	0.749329
39	1	0	8.406202	-0.227597	-0.419432
40	1	0	7.461678	0.687461	0.780931

Structure 12 (M06-2X/def2-TZVP, Gas Phase)

Energy (Hartrees): = -1050.991680
No imaginary frequencies

Standard orientation:

Center Number	Atomic Number	Atomic Type	X	Y	Z
1	6	0	-1.857665	1.353084	-0.374374
2	6	0	-1.248106	0.159271	0.358639
3	6	0	-2.091162	-1.074141	0.065192
4	6	0	-3.544611	-0.815259	0.390797
5	6	0	-4.026266	0.408217	-0.381896
6	1	0	-1.787737	1.197802	-1.462517
7	1	0	-2.011131	-1.303197	-1.006098
8	1	0	-3.635707	-0.602364	1.464504
9	1	0	-3.946551	0.204723	-1.459956
10	1	0	-1.295151	0.390022	1.432034
11	8	0	-3.214639	1.510626	-0.020267
12	6	0	-5.469218	0.773458	-0.067511
13	1	0	-5.593909	0.833355	1.021285
14	1	0	-5.671755	1.761091	-0.482879
15	8	0	-6.381128	-0.123553	-0.651863
16	1	0	-6.110779	-1.018209	-0.411012
17	8	0	-4.345332	-1.928250	0.042054
18	1	0	-3.918158	-2.715208	0.400674
19	8	0	-1.681328	-2.189597	0.830320
20	1	0	-0.766553	-2.389443	0.601606
21	8	0	-1.173073	2.487979	0.023249
22	1	0	-1.558177	3.250609	-0.420564
23	7	0	0.099981	-0.084039	-0.097614
24	6	0	2.463833	0.116575	0.282383
25	6	0	2.840476	-0.466136	-0.933481
26	6	0	3.455316	0.535204	1.157531
27	6	0	4.168278	-0.623640	-1.250959
28	1	0	2.065774	-0.787432	-1.617641
29	6	0	4.802581	0.384536	0.851532
30	1	0	3.174814	0.991861	2.100053
31	6	0	5.160416	-0.199552	-0.358595
32	1	0	4.479372	-1.071032	-2.185707
33	1	0	5.550575	0.722931	1.553210
34	6	0	1.053792	0.291006	0.644767
35	1	0	0.865870	0.775270	1.611503
36	8	0	6.434820	-0.398868	-0.760645
37	6	0	7.473168	0.013773	0.100956
38	1	0	7.423679	-0.512926	1.057708
39	1	0	8.402530	-0.236563	-0.403016
40	1	0	7.433598	1.091906	0.277430

Structure 12 (M06-2X/def2-TZVP, DMSO)

Energy (Hartrees): = -1051.019866
No imaginary frequencies

Standard orientation:

Center Number	Atomic Number	Atomic Type	X	Y	Z
1	6	0	1.993610	1.503398	0.181237
2	6	0	1.256480	0.283234	-0.363219
3	6	0	1.982012	-0.979258	0.101427
4	6	0	3.450115	-0.910031	-0.259832
5	6	0	4.059155	0.365159	0.312518
6	1	0	1.945205	1.510206	1.279932
7	1	0	1.895476	-1.053686	1.192853
8	1	0	3.548151	-0.880267	-1.352774
9	1	0	3.974677	0.343087	1.408497
10	1	0	1.291373	0.332143	-1.460289
11	8	0	3.352747	1.472779	-0.214765
12	6	0	5.521024	0.537324	-0.066937
13	1	0	5.624700	0.436346	-1.154531
14	1	0	5.838242	1.543224	0.211289
15	8	0	6.359712	-0.371951	0.616569
16	1	0	5.968641	-1.249843	0.515332
17	8	0	4.152030	-2.026322	0.263627
18	1	0	3.685500	-2.824407	-0.016889
19	8	0	1.458385	-2.142182	-0.511496
20	1	0	0.565241	-2.291237	-0.176154
21	8	0	1.415196	2.638302	-0.358237
22	1	0	1.787840	3.412424	0.082510
23	7	0	-0.094265	0.252599	0.145465
24	6	0	-2.453915	0.077513	-0.291478
25	6	0	-2.853455	0.150823	1.048679

26	6	0	-3.429661	-0.063621	-1.270959
27	6	0	-4.185198	0.082560	1.387371
28	1	0	-2.103346	0.262917	1.821580
29	6	0	-4.779003	-0.136262	-0.946400
30	1	0	-3.132129	-0.119145	-2.312434
31	6	0	-5.160116	-0.062799	0.390747
32	1	0	-4.503997	0.138311	2.420751
33	1	0	-5.512019	-0.248644	-1.732216
34	6	0	-1.042724	0.145229	-0.688155
35	1	0	-0.851809	0.098969	-1.767123
36	8	0	-6.436696	-0.122579	0.818613
37	6	0	-7.455510	-0.288318	-0.155695
38	1	0	-7.324173	-1.223054	-0.705887
39	1	0	-8.393974	-0.320089	0.392305
40	1	0	-7.471616	0.550467	-0.855303

Structure 12.5H_2O (M06-2X, Gas Phase)

Energy (Hartrees): = - 1433.0382956
No imaginary frequencies

Standard orientation:

Center Number	Atomic Number	Atomic Type	Coordinates (Angstroms) X	Y	Z
1	6	0	-3.551206	0.831732	-0.122965
2	6	0	-3.041692	-0.412706	0.280105
3	6	0	-3.924814	-1.475209	0.445818
4	6	0	-5.286594	-1.334329	0.199153
5	6	0	-5.776401	-0.094934	-0.210353
6	6	0	-4.898457	0.987743	-0.362290
7	6	0	-1.615684	-0.633076	0.540067
8	7	0	-0.698593	0.190936	0.236112
9	6	0	0.667289	-0.190436	0.551487
10	6	0	1.283696	-0.961114	-0.630373
11	8	0	2.642301	-1.250859	-0.330962
12	6	0	3.501504	-0.123326	-0.142902
13	6	0	2.981365	0.673148	1.049429
14	6	0	1.520715	1.043768	0.812346
15	8	0	0.656931	-2.167288	-0.886310
16	6	0	4.894888	-0.689347	0.064714
17	8	0	5.351088	-1.397140	-1.052187
18	8	0	3.732730	1.829310	1.329491
19	8	0	1.014615	1.750067	1.933070
20	8	0	3.410735	3.017035	-1.080583
21	8	0	1.116629	4.384828	-1.283084
22	8	0	-0.768215	3.007390	0.090369
23	8	0	2.115514	-3.440221	1.164964
24	8	0	3.656228	-3.718086	-1.293309
25	1	0	-1.372165	-1.585719	1.028451
26	1	0	1.480731	1.687794	-0.078791
27	1	0	1.755958	2.284621	2.248301
28	1	0	3.756365	2.363348	0.510532
29	1	0	3.036636	0.041505	1.946465
30	1	0	3.484654	0.499210	-1.045849
31	1	0	5.580011	0.139692	0.250668
32	1	0	4.879657	-1.315398	0.972401
33	1	0	4.836835	-2.217481	-1.129644
34	1	0	0.787536	-2.739427	-0.113350
35	1	0	1.222461	-0.361364	-1.545270
36	1	0	0.707363	-0.851436	1.431545
37	1	0	-2.887490	1.682010	-0.220695
38	1	0	-5.312965	1.940438	-0.666693
39	1	0	-5.943353	-2.182869	0.329669
40	1	0	-3.544377	-2.439767	0.765963
41	1	0	-0.852874	2.070105	-0.167189
42	1	0	-0.383162	2.906341	0.973092
43	1	0	2.613296	-2.639134	0.932826
44	1	0	2.224926	-3.588316	2.106334
45	1	0	0.367491	3.902044	-0.872538
46	1	0	1.040614	5.289776	-0.975629
47	1	0	2.609192	3.571910	-1.200999
48	1	0	3.988057	3.180794	-1.827740
49	1	0	2.900956	-3.185536	-1.568694
50	1	0	3.339187	-4.131423	-0.482774
51	8	0	-7.074970	0.164638	-0.476100
52	6	0	-8.002506	-0.894337	-0.345047
53	1	0	-8.971170	-0.478443	-0.611045
54	1	0	-7.760190	-1.717493	-1.024214
55	1	0	-8.035338	-1.265191	0.684065

Structure 12.5H_2O (M06-2X, DMSO)

Energy (Hartrees): = - 1433.0801332
No imaginary frequencies

Standard orientation:

Center Number	Atomic Number	Atomic Type	Coordinates (Angstroms) X	Y	Z
1	6	0	-3.438236	0.391220	-0.766268
2	6	0	-3.011338	-0.505823	0.225674
3	6	0	-3.969032	-1.247259	0.914286
4	6	0	-5.327309	-1.103226	0.649756
5	6	0	-5.737554	-0.199571	-0.332322
6	6	0	-4.780145	0.543485	-1.042024
7	6	0	-1.594714	-0.685066	0.562725
8	7	0	-0.672107	0.057199	0.100178
9	6	0	0.691672	-0.258309	0.480893
10	6	0	1.386755	-0.964166	-0.695535
11	8	0	2.745382	-1.200415	-0.355185
12	6	0	3.534438	-0.036642	-0.119183
13	6	0	2.940265	0.713207	1.070260
14	6	0	1.469089	1.013604	0.798246
15	8	0	0.830634	-2.193429	-1.003417
16	6	0	4.942196	-0.542639	0.131915
17	8	0	5.440855	-1.272430	-0.963619
18	8	0	3.628441	1.903436	1.380054
19	8	0	0.892936	1.656236	1.928611
20	8	0	3.327385	3.411487	-0.859715
21	8	0	0.819411	4.355615	-1.187617
22	8	0	-0.981583	2.877253	0.218647
23	8	0	2.256673	-3.499546	1.046166
24	8	0	3.781693	-3.630792	-1.367046
25	1	0	-1.371473	-1.504023	1.257040
26	1	0	1.413257	1.682943	-0.073144
27	1	0	1.582366	2.240660	2.274130
28	1	0	3.591341	2.477055	0.585946
29	1	0	3.006497	0.067661	1.954961
30	1	0	3.518312	0.596712	-1.017183
31	1	0	5.602240	0.310435	0.298024
32	1	0	4.934069	-1.148420	1.050470
33	1	0	4.901616	-2.074904	-1.058492
34	1	0	0.969725	-2.773152	-0.235530
35	1	0	1.329020	-0.341326	-1.595315
36	1	0	0.725971	-0.932786	1.350383
37	1	0	-2.706696	0.954650	-1.334204
38	1	0	-5.123424	1.228793	-1.807968
39	1	0	-6.044254	-1.692003	1.205694
40	1	0	-3.649091	-1.949519	1.677589
41	1	0	-1.045113	1.969185	-0.128082
42	1	0	-0.505427	2.713227	1.047190
43	1	0	2.688093	-2.634852	1.102054
44	1	0	2.183465	-3.836899	1.945865
45	1	0	0.124736	3.837722	-0.728575
46	1	0	0.689921	5.265677	-0.902994
47	1	0	2.422173	3.770803	-0.984842
48	1	0	3.591642	3.063179	-1.716792
49	1	0	3.072886	-3.038064	-1.645989
50	1	0	3.457178	-3.920546	-0.503151
51	8	0	-7.021826	0.022769	-0.672281
52	6	0	-8.026643	-0.706683	0.022174
53	1	0	-8.002212	-0.488049	1.093320
54	1	0	-8.976455	-0.376670	-0.393011
55	1	0	-7.912276	-1.782156	-0.139004

Structure 12.5H_2O (M06-2X, H_2O)

Energy (Hartrees): = - 1433.1020704
No imaginary frequencies

Standard orientation:

Center Number	Atomic Number	Atomic Type	Coordinates (Angstroms) X	Y	Z
1	6	0	-3.516978	0.888036	-0.241154
2	6	0	-3.034653	-0.317095	0.294175
3	6	0	-3.940782	-1.340718	0.565461
4	6	0	-5.300860	-1.193771	0.311530
5	6	0	-5.762287	0.007757	-0.225095
6	6	0	-4.862337	1.049792	-0.495022
7	6	0	-1.613446	-0.553006	0.563197
8	7	0	-0.695031	0.301057	0.338566
9	6	0	0.668694	-0.138056	0.596158
10	6	0	1.255464	-0.775094	-0.674060
11	8	0	2.597276	-1.166764	-0.409776
12	6	0	3.488445	-0.097201	-0.088545
13	6	0	2.995963	0.605765	1.177144

14	6	0	1.547934	1.037005	0.989529
15	8	0	0.570469	-1.918468	-1.073965
16	6	0	4.864679	-0.703107	0.098846
17	8	0	5.388710	-1.242705	-1.101696
18	8	0	3.796386	1.729890	1.495696
19	8	0	1.029939	1.617457	2.175522
20	8	0	3.670905	3.270671	-0.795431
21	8	0	1.219177	2.802634	-1.975725
22	8	0	-0.673917	3.110950	0.039225
23	8	0	1.538884	-3.402481	1.112613
24	8	0	3.754814	-3.571776	-1.222217
25	1	0	-1.371990	-1.538851	0.977138
26	1	0	1.533031	1.774965	0.177704
27	1	0	1.655515	2.303086	2.441916
28	1	0	3.760159	2.333983	0.724269
29	1	0	3.060659	-0.087864	2.023153
30	1	0	3.502832	0.619346	-0.921773
31	1	0	5.543421	0.080900	0.437023
32	1	0	4.804329	-1.471163	0.880838
33	1	0	4.942333	-2.091709	-1.251339
34	1	0	0.650708	-2.562296	-0.344136
35	1	0	1.233732	-0.063112	-1.505917
36	1	0	0.699912	-0.899052	1.387523
37	1	0	-2.831171	1.698992	-0.455375
38	1	0	-5.250172	1.973225	-0.908127
39	1	0	-5.977217	-2.008651	0.529802
40	1	0	-3.577258	-2.275749	0.979177
41	1	0	-0.774547	2.135839	0.118806
42	1	0	-0.204520	3.350149	0.847426
43	1	0	2.238338	-2.738523	1.029080
44	1	0	1.086831	-3.168785	1.931430
45	1	0	0.532995	3.007454	-1.309877
46	1	0	1.018679	3.368226	-2.729048
47	1	0	2.804269	3.126331	-1.228591
48	1	0	4.300355	2.811862	-1.362604
49	1	0	3.099469	-2.865913	-1.105903
50	1	0	3.836337	-3.947453	-0.338078
51	8	0	-7.061104	0.262281	-0.520249
52	6	0	-8.009285	-0.775779	-0.286026
53	1	0	-8.046463	-1.035905	0.774476
54	1	0	-8.970692	-0.375481	-0.598744
55	1	0	-7.770273	-1.661920	-0.878988

Structure 12·5H_2O (M06-2X/def2-TZVP, Gas Phase)

Energy (Hartrees): = -1433.210776
No imaginary frequencies

Standard orientation:

Center Number	Atomic Number	Atomic Type	Coordinates (Angstroms) X	Y	Z
1	6	0	-3.451402	0.282271	-0.819856
2	6	0	-3.027579	-0.543368	0.228370
3	6	0	-3.984139	-1.223716	0.969230
4	6	0	-5.339908	-1.088140	0.701700
5	6	0	-5.744818	-0.256305	-0.337207
6	6	0	-4.788975	0.425378	-1.099807
7	6	0	-1.612783	-0.706840	0.566357
8	7	0	-0.683892	-0.014097	0.056580
9	6	0	0.674840	-0.295249	0.455835
10	6	0	1.422035	-0.961729	-0.708135
11	8	0	2.775236	-1.159523	-0.337681
12	6	0	3.523922	0.022152	-0.064943
13	6	0	2.876689	0.730209	1.119494
14	6	0	1.405358	0.990374	0.819087
15	8	0	0.903911	-2.193374	-1.058329
16	6	0	4.944538	-0.430289	0.209679
17	8	0	5.535798	-1.039355	-0.902259
18	8	0	3.514220	1.929407	1.480041
19	8	0	0.783414	1.588404	1.943564
20	8	0	3.198972	3.298897	-0.878165
21	8	0	0.733345	4.355763	-1.201181
22	8	0	-1.062575	2.839996	0.144473
23	8	0	2.417135	-3.513866	1.014800
24	8	0	4.078776	-3.512781	-1.435776
25	1	0	-1.392476	-1.477713	1.316006
26	1	0	1.350013	1.672562	-0.041791
27	1	0	1.451088	2.168648	2.337031
28	1	0	3.522181	2.515659	0.696937
29	1	0	2.938585	0.071157	1.994965
30	1	0	3.508143	0.674601	-0.946901
31	1	0	5.542367	0.441262	0.477495
32	1	0	4.929355	-1.101847	1.081785

33	1	0	5.105145	-1.894981	-1.068686
34	1	0	0.985457	-2.794376	-0.300571
35	1	0	1.370251	-0.325596	-1.598038
36	1	0	0.714530	-0.985391	1.313797
37	1	0	-2.713639	0.797216	-1.421271
38	1	0	-5.132994	1.057236	-1.907473
39	1	0	-6.058800	-1.627946	1.299712
40	1	0	-3.668264	-1.870938	1.779536
41	1	0	-1.117539	1.937592	-0.215201
42	1	0	-0.656725	2.675525	1.009733
43	1	0	2.807518	-2.641087	0.835416
44	1	0	2.543930	-3.708617	1.946547
45	1	0	0.029348	3.831719	-0.755860
46	1	0	0.496043	5.279456	-1.097443
47	1	0	2.342920	3.755003	-1.032304
48	1	0	3.803369	3.572196	-1.571000
49	1	0	3.292155	-3.034932	-1.727376
50	1	0	3.769914	-3.998707	-0.661336
51	8	0	-7.031492	-0.048448	-0.687481
52	6	0	-8.035585	-0.714318	0.047772
53	1	0	-8.982665	-0.408730	-0.388266
54	1	0	-7.929293	-1.799174	-0.034488
55	1	0	-8.009761	-0.424653	1.101475

Structure 12·5H_2O (M06-2X/def2-TZVP, DMSO)

Energy (Hartrees): = -1433.252885
No imaginary frequencies

Standard orientation:

Center Number	Atomic Number	Atomic Type	Coordinates (Angstroms) X	Y	Z
1	6	0	-3.432495	0.097183	-0.927174
2	6	0	-3.014561	-0.592068	0.218487
3	6	0	-3.978508	-1.143203	1.054699
4	6	0	-5.334442	-1.003460	0.788629
5	6	0	-5.734867	-0.303611	-0.347295
6	6	0	-4.771682	0.239480	-1.208717
7	6	0	-1.599330	-0.741801	0.566974
8	7	0	-0.670508	-0.111706	-0.023457
9	6	0	0.689820	-0.347245	0.405347
10	6	0	1.479445	-0.984556	-0.744723
11	8	0	2.833376	-1.140217	-0.353041
12	6	0	3.532781	0.064021	-0.058553
13	6	0	2.836518	0.758120	1.107127
14	6	0	1.367073	0.968469	0.767743
15	8	0	1.015403	-2.237430	-1.102644
16	6	0	4.953764	-0.345903	0.268114
17	8	0	5.586496	-0.989854	-0.811764
18	8	0	3.433422	1.982582	1.461205
19	8	0	0.692760	1.562716	1.867139
20	8	0	3.198567	3.661849	-0.696830
21	8	0	0.611514	4.314738	-1.228831
22	8	0	-1.189535	2.736160	0.096692
23	8	0	2.505805	-3.562076	0.979809
24	8	0	4.160856	-3.488485	-1.399798
25	1	0	-1.382726	-1.429918	1.391728
26	1	0	1.309301	1.636685	-0.104349
27	1	0	1.319313	2.184488	2.267113
28	1	0	3.396552	2.584852	0.686891
29	1	0	2.902657	0.106761	1.986439
30	1	0	3.524892	0.713695	-0.944656
31	1	0	5.533148	0.545298	0.510722
32	1	0	4.933824	-0.988658	1.158738
33	1	0	5.145101	-1.841706	-0.966728
34	1	0	1.123441	-2.830448	-0.339983
35	1	0	1.420646	-0.348883	-1.634672
36	1	0	0.735857	-1.026223	1.270107
37	1	0	-2.694841	0.506451	-1.607147
38	1	0	-5.103834	0.766711	-2.094199
39	1	0	-6.058251	-1.437097	1.463453
40	1	0	-3.666042	-1.688780	1.938312
41	1	0	-1.226415	1.856919	-0.317889
42	1	0	-0.718802	2.535066	0.923168
43	1	0	2.835400	-2.650993	1.020206
44	1	0	2.456284	-3.886542	1.886967
45	1	0	-0.078666	3.779145	-0.781164
46	1	0	0.345450	5.235764	-1.135442
47	1	0	2.272549	3.919182	-0.898706
48	1	0	3.597208	3.405648	-1.535624
49	1	0	3.415030	-2.931501	-1.661481
50	1	0	3.848804	-3.872458	-0.567642
51	8	0	-7.019278	-0.104458	-0.699669

52	6	0	-8.030199	-0.618649	0.154608
53	1	0	-7.955268	-0.187697	1.155482
54	1	0	-8.978233	-0.330943	-0.293164
55	1	0	-7.973832	-1.707744	0.217976

Structure 12·5H_2O (M06-2X/def2-TZVP, H_2O)

Energy (Hartrees): = -1433.271362
No imaginary frequencies

Standard orientation:

Center Number	Atomic Number	Atomic Type	Coordinates (Angstroms) X	Y	Z
1	6	0	-3.496352	0.833016	-0.350958
2	6	0	-3.033111	-0.328526	0.280108
3	6	0	-3.953825	-1.308359	0.635411
4	6	0	-5.310246	-1.157349	0.379111
5	6	0	-5.752566	0.004020	-0.246269
6	6	0	-4.837672	1.000222	-0.608015
7	6	0	-1.617690	-0.565971	0.562585
8	7	0	-0.689645	0.268124	0.315804
9	6	0	0.667761	-0.174703	0.582455
10	6	0	1.264308	-0.801346	-0.684995
11	8	0	2.602151	-1.190800	-0.423242
12	6	0	3.483261	-0.118378	-0.099307
13	6	0	2.992704	0.563337	1.176786
14	6	0	1.543140	0.991707	1.002643
15	8	0	0.579370	-1.940248	-1.096671
16	6	0	4.866133	-0.708899	0.070359
17	8	0	5.393295	-1.222036	-1.140134
18	8	0	3.790842	1.679923	1.516578
19	8	0	1.024223	1.533421	2.204870
20	8	0	3.687379	3.399220	-0.664145
21	8	0	1.218321	3.107041	-1.900480
22	8	0	-0.736260	3.110947	0.085868
23	8	0	1.525144	-3.486468	1.151218
24	8	0	3.778914	-3.593976	-1.354627
25	1	0	-1.387880	-1.537685	1.012966
26	1	0	1.518337	1.753076	0.211365
27	1	0	1.624721	2.236392	2.487943
28	1	0	3.749346	2.318327	0.771714
29	1	0	3.060497	-0.147346	2.007300
30	1	0	3.483989	0.607824	-0.924469
31	1	0	5.540643	0.073215	0.417617
32	1	0	4.820965	-1.491462	0.836735
33	1	0	4.964614	-2.076889	-1.314590
34	1	0	0.628491	-2.590314	-0.370243
35	1	0	1.244714	-0.086006	-1.513687
36	1	0	0.688862	-0.944829	1.364677
37	1	0	-2.796397	1.602217	-0.649875
38	1	0	-5.207149	1.892050	-1.097882
39	1	0	-5.999404	-1.938487	0.664599
40	1	0	-3.604605	-2.212792	1.120547
41	1	0	-0.797255	2.129113	0.133999
42	1	0	-0.259356	3.355960	0.889359
43	1	0	2.216769	-2.815611	1.046453
44	1	0	1.037932	-3.212840	1.938583
45	1	0	0.498549	3.181708	-1.240392
46	1	0	1.023775	3.765618	-2.577085
47	1	0	2.817056	3.308637	-1.106740
48	1	0	4.316294	2.974999	-1.260327
49	1	0	3.127558	-2.916575	-1.107820
50	1	0	3.907531	-4.111775	-0.550692
51	8	0	-7.049408	0.255676	-0.548518
52	6	0	-8.003759	-0.761294	-0.270320
53	1	0	-8.050160	-0.969319	0.800156
54	1	0	-8.961187	-0.374423	-0.608607
55	1	0	-7.764012	-1.676111	-0.815532

Structure 12.6H_2O (B3LYP, Gas Phase)

Energy (Hartrees): = - 1509.6551735
No imaginary frequencies

Standard orientation:

Center Number	Atomic Number	Atomic Type	Coordinates (Angstroms) X	Y	Z
1	6	0	3.850192	-0.880963	-0.186361

2	6	0	3.413363	0.380082	0.272136
3	6	0	4.363373	1.399107	0.421900
4	6	0	5.706482	1.199320	0.109036
5	6	0	6.120718	-0.055865	-0.354582
6	6	0	5.181320	-1.094777	-0.492657
7	6	0	2.019107	0.665644	0.609944
8	7	0	1.033378	-0.110338	0.355156
9	6	0	-0.297131	0.319398	0.762999
10	6	0	-1.009258	1.030962	-0.416895
11	8	0	-2.359202	1.344591	-0.036801
12	6	0	-3.198543	0.216555	0.292662
13	6	0	-2.593421	-0.498960	1.505946
14	6	0	-1.153011	-0.892279	1.152154
15	8	0	-0.413482	2.216358	-0.824750
16	6	0	-4.611688	0.741045	0.501454
17	8	0	-5.216880	1.174367	-0.706623
18	8	0	-3.313458	-1.639043	1.916958
19	8	0	-0.560126	-1.605561	2.231674
20	8	0	-3.668571	-2.998576	-0.350782
21	8	0	-1.232228	-3.577482	-1.559889
22	8	0	0.909761	-2.934183	0.041655
23	8	0	-2.081839	3.898824	0.806159
24	8	0	-3.752136	3.419125	-1.537060
25	1	0	1.854550	1.626704	1.118660
26	1	0	-1.210731	-1.549390	0.272342
27	1	0	-1.303222	-2.113777	2.604676
28	1	0	-3.468046	-2.209728	1.109812
29	1	0	-2.576353	0.193962	2.360170
30	1	0	-3.213142	-0.470083	-0.561803
31	1	0	-5.217791	-0.074653	0.903563
32	1	0	-4.601293	1.546434	1.252797
33	1	0	-4.759913	1.992115	-1.014009
34	1	0	-0.594966	2.901766	-0.154232
35	1	0	-1.018185	0.363100	-1.288427
36	1	0	-0.255260	1.030483	1.605118
37	1	0	3.142944	-1.698456	-0.272195
38	1	0	5.530761	-2.062057	-0.837512
39	1	0	6.411234	2.012597	0.230391
40	1	0	4.045794	2.374370	0.782462
41	1	0	1.046150	-1.969699	-0.102701
42	1	0	0.607942	-2.925186	0.966648
43	1	0	-2.390481	2.972189	0.846825
44	1	0	-2.148718	4.258939	1.699237
45	1	0	-0.438863	-3.391100	-1.000293
46	1	0	-1.201778	-4.528179	-1.722436
47	1	0	-2.827028	-3.215828	-0.810877
48	1	0	-4.216004	-2.495704	-0.988057
49	1	0	-2.940750	2.929518	-1.738214
50	1	0	-3.469334	3.957544	-0.778452
51	8	0	-5.117737	-1.266699	-2.088042
52	1	0	-4.691802	-1.118719	-2.940950
53	1	0	-5.264860	-0.368576	-1.718732
54	8	0	7.398724	-0.373982	-0.686977
55	6	0	8.402110	0.625893	-0.565898
56	1	0	9.333488	0.154377	-0.880929
57	1	0	8.194160	1.485618	-1.214552
58	1	0	8.503552	0.970906	0.470371

Structure 12.6H$_2$O (B3LYP, DMSO)

Energy (Hartrees): = - 1509.6950451
No imaginary frequencies

Standard orientation:

Center Number	Atomic Number	Atomic Type	Coordinates (Angstroms) X	Y	Z
1	6	0	3.858387	-0.872533	-0.273387
2	6	0	3.426016	0.351086	0.280793
3	6	0	4.383919	1.350077	0.511268
4	6	0	5.726656	1.166289	0.187461
5	6	0	6.136560	-0.051880	-0.373411
6	6	0	5.189478	-1.072172	-0.593004
7	6	0	2.033020	0.627821	0.632916
8	7	0	1.041063	-0.128128	0.333210
9	6	0	-0.285839	0.308334	0.755403
10	6	0	-1.001200	1.023907	-0.420028
11	8	0	-2.339793	1.359351	-0.022924
12	6	0	-3.181998	0.242588	0.316499
13	6	0	-2.575654	-0.494512	1.517094
14	6	0	-1.144830	-0.899481	1.146863
15	8	0	-0.389496	2.198316	-0.835988
16	6	0	-4.584292	0.781501	0.551905
17	8	0	-5.206832	1.221845	-0.652961
18	8	0	-3.314330	-1.641470	1.902383

19	8	0	-0.537091	-1.624499	2.216776
20	8	0	-3.777997	-2.999620	-0.386407
21	8	0	-1.326109	-3.775469	-1.471106
22	8	0	0.876098	-2.939244	-0.015819
23	8	0	-2.079698	3.956789	0.717107
24	8	0	-3.765060	3.456240	-1.532386
25	1	0	1.875861	1.560691	1.189895
26	1	0	-1.217881	-1.554569	0.267316
27	1	0	-1.264856	-2.162736	2.576379
28	1	0	-3.485158	-2.181676	1.082398
29	1	0	-2.552302	0.178875	2.383855
30	1	0	-3.211500	-0.440352	-0.541120
31	1	0	-5.199010	-0.023400	0.963035
32	1	0	-4.552921	1.589928	1.296644
33	1	0	-4.732239	2.029247	-0.964568
34	1	0	-0.555928	2.884890	-0.162730
35	1	0	-1.033676	0.354671	-1.289886
36	1	0	-0.233068	1.017482	1.595934
37	1	0	3.147886	-1.677236	-0.427886
38	1	0	5.527787	-2.014701	-1.011759
39	1	0	6.436046	1.963153	0.373980
40	1	0	4.070977	2.295277	0.947220
41	1	0	1.015811	-1.971131	-0.149954
42	1	0	0.576087	-2.933972	0.910453
43	1	0	-2.351423	3.038367	0.901509
44	1	0	-2.160789	4.440961	1.552358
45	1	0	-0.527754	-3.468992	-0.975952
46	1	0	-1.344155	-4.731617	-1.321029
47	1	0	-2.921372	-3.229849	-0.813362
48	1	0	-4.253674	-2.415418	-1.014879
49	1	0	-3.023607	2.922320	-1.856640
50	1	0	-3.366139	3.877814	-0.745885
51	8	0	-5.128894	-1.175952	-2.107756
52	1	0	-4.576957	-1.014705	-2.886594
53	1	0	-5.215918	-0.290856	-1.686356
54	8	0	7.410425	-0.345746	-0.728088
55	6	0	8.418639	0.649495	-0.528529
56	1	0	9.348764	0.206666	-0.887389
57	1	0	8.205169	1.557484	-1.103298
58	1	0	8.525556	0.902829	0.532124

Structure 12.6H_2O (B3LYP, H_2O)

Energy (Hartrees): = - 1509.7181989
No imaginary frequencies

Standard orientation:

Center Number	Atomic Number	Atomic Type	Coordinates (Angstroms) X	Y	Z
1	6	0	3.927890	-0.835803	-0.349144
2	6	0	3.519685	0.343062	0.310565
3	6	0	4.497725	1.292842	0.643378
4	6	0	5.841827	1.099789	0.330646
5	6	0	6.226956	-0.075964	-0.326512
6	6	0	5.259896	-1.043449	-0.660031
7	6	0	2.130209	0.631243	0.660835
8	7	0	1.129714	-0.125593	0.387947
9	6	0	-0.194094	0.349377	0.777658
10	6	0	-0.943314	0.869188	-0.468397
11	8	0	-2.268472	1.264620	-0.099729
12	6	0	-3.088300	0.182796	0.385485
13	6	0	-2.463324	-0.385898	1.667177
14	6	0	-1.003225	-0.784332	1.415153
15	8	0	-0.322075	1.974228	-1.058161
16	6	0	-4.499865	0.712081	0.596709
17	8	0	-5.219483	0.902998	-0.620313
18	8	0	-3.188785	-1.502003	2.170850
19	8	0	-0.375932	-1.160683	2.640718
20	8	0	-3.671488	-3.171170	0.049906
21	8	0	-1.653032	-2.732165	-1.799967
22	8	0	0.944039	-2.651054	-0.895884
23	8	0	-2.949958	3.955813	0.459637
24	8	0	-4.116491	3.070491	-1.900383
25	1	0	1.978565	1.575519	1.198842
26	1	0	-1.004181	-1.637463	0.721044
27	1	0	-0.987244	-1.789523	3.056976
28	1	0	-3.319112	-2.139230	1.419145
29	1	0	-2.482719	0.385378	2.447428
30	1	0	-3.117351	-0.602884	-0.379510
31	1	0	-5.053393	-0.023743	1.184468
32	1	0	-4.457748	1.647037	1.171065
33	1	0	-4.806944	1.655128	-1.115141
34	1	0	-0.137568	2.624436	-0.360365
35	1	0	-1.000722	0.090678	-1.238795

36	1	0	-0.130734	1.188540	1.487926
37	1	0	3.193572	-1.586997	-0.618074
38	1	0	5.581291	-1.947038	-1.167964
39	1	0	6.569344	1.856028	0.597655
40	1	0	4.200491	2.204805	1.154000
41	1	0	1.010606	-1.763763	-0.454861
42	1	0	0.953874	-3.274417	-0.154384
43	1	0	-2.578942	3.051047	0.458371
44	1	0	-3.650594	3.910889	1.127213
45	1	0	-0.720455	-2.731527	-1.471701
46	1	0	-1.690397	-3.469231	-2.425996
47	1	0	-2.881065	-3.100705	-0.535196
48	1	0	-4.340930	-2.652531	-0.450739
49	1	0	-3.314508	2.659308	-2.256369
50	1	0	-3.781858	3.513734	-1.085333
51	8	0	-5.299956	-1.651020	-1.687418
52	1	0	-4.587500	-1.685977	-2.342546
53	1	0	-5.283529	-0.713869	-1.380412
54	8	0	7.507932	-0.375279	-0.685832
55	6	0	8.527570	0.600964	-0.438876
56	1	0	9.450606	0.168809	-0.826273
57	1	0	8.313354	1.537424	-0.964087
58	1	0	8.639587	0.795725	0.632729

Structure 12.6H_2O (B3LYP, Gas Phase)

Energy (Hartrees): = - 1509.4818579
No imaginary frequencies

Standard orientation:

Center Number	Atomic Number	Atomic Type	Coordinates (Angstroms) X	Y	Z
1	6	0	3.796038	-0.925230	-0.195775
2	6	0	3.374584	0.326514	0.281724
3	6	0	4.320952	1.332893	0.447734
4	6	0	5.662417	1.128206	0.141362
5	6	0	6.065555	-0.118658	-0.334142
6	6	0	5.121937	-1.143594	-0.496307
7	6	0	1.975917	0.610800	0.615325
8	7	0	1.016853	-0.192636	0.404412
9	6	0	-0.321098	0.237199	0.763375
10	6	0	-1.005218	0.888143	-0.451019
11	8	0	-2.332561	1.242596	-0.072773
12	6	0	-3.181586	0.144569	0.258130
13	6	0	-2.608477	-0.556600	1.487874
14	6	0	-1.170676	-0.961579	1.176661
15	8	0	-0.388780	2.043296	-0.896953
16	6	0	-4.567880	0.715757	0.465685
17	8	0	-5.087392	1.270817	-0.724175
18	8	0	-3.348769	-1.681820	1.885739
19	8	0	-0.579181	-1.614334	2.280359
20	8	0	-3.646363	-2.933050	-0.501116
21	8	0	-1.200455	-2.530464	-1.809450
22	8	0	0.884135	-2.934812	-0.088848
23	8	0	-1.774244	3.654409	0.982877
24	8	0	-3.443801	3.470135	-1.387637
25	1	0	1.793908	1.590768	1.075971
26	1	0	-1.219013	-1.645345	0.320699
27	1	0	-1.288914	-2.142479	2.668896
28	1	0	-3.482621	-2.243533	1.087976
29	1	0	-2.608705	0.142984	2.334941
30	1	0	-3.198258	-0.553063	-0.586779
31	1	0	-5.232067	-0.090918	0.778801
32	1	0	-4.536977	1.465116	1.269190
33	1	0	-4.566586	2.061564	-0.964261
34	1	0	-0.461279	2.713946	-0.199683
35	1	0	-1.031652	0.177885	-1.285011
36	1	0	-0.300561	0.978400	1.578615
37	1	0	3.079300	-1.728632	-0.317473
38	1	0	5.470036	-2.102027	-0.860226
39	1	0	6.370465	1.933509	0.277623
40	1	0	4.006824	2.303184	0.818531
41	1	0	1.033151	-1.968193	0.006121
42	1	0	0.612838	-3.185727	0.801127
43	1	0	-2.222882	2.794009	0.942780
44	1	0	-1.804258	3.956070	1.892964
45	1	0	-0.433055	-2.827946	-1.278212
46	1	0	-1.011982	-2.774616	-2.717645
47	1	0	-2.835086	-2.919774	-1.036577
48	1	0	-4.307507	-2.442182	-1.018022
49	1	0	-2.665386	2.948120	-1.617153
50	1	0	-3.124101	3.999799	-0.647614
51	8	0	-5.414569	-1.216317	-1.856507
52	1	0	-6.357225	-1.389818	-1.888747

53	1	0	-5.331736	-0.262648	-1.677320
54	8	0	7.336692	-0.437900	-0.660620
55	6	0	8.324522	0.566035	-0.537335
56	1	0	9.257359	0.109522	-0.858842
57	1	0	8.097688	1.423202	-1.178827
58	1	0	8.420690	0.900093	0.500367

Structure 12.6H_2O (M06-2X, DMSO)

Energy (Hartrees): = - 1395.0313857
No imaginary frequencies

Standard orientation:

Center Number	Atomic Number	Atomic Type	Coordinates (Angstroms) X	Y	Z
1	6	0	3.799375	-0.912831	-0.270111
2	6	0	3.378917	0.317583	0.260472
3	6	0	4.331861	1.309990	0.482990
4	6	0	5.674664	1.110942	0.178773
5	6	0	6.076783	-0.115276	-0.353743
6	6	0	5.127374	-1.127380	-0.570574
7	6	0	1.979918	0.601866	0.595976
8	7	0	1.013589	-0.181485	0.331572
9	6	0	-0.317863	0.258451	0.712410
10	6	0	-1.012258	0.914687	-0.493128
11	8	0	-2.330895	1.281388	-0.108423
12	6	0	-3.180362	0.198299	0.258095
13	6	0	-2.592007	-0.500423	1.481799
14	6	0	-1.171331	-0.932799	1.136049
15	8	0	-0.390994	2.063991	-0.946139
16	6	0	-4.552418	0.792610	0.490462
17	8	0	-5.083900	1.340741	-0.704882
18	8	0	-3.348519	-1.614705	1.896912
19	8	0	-0.564846	-1.598784	2.229473
20	8	0	-3.763970	-2.944230	-0.434510
21	8	0	-1.276599	-3.171934	-1.701223
22	8	0	0.866406	-2.978371	0.042601
23	8	0	-1.720563	3.721076	0.910353
24	8	0	-3.406523	3.525171	-1.406476
25	1	0	1.806385	1.558172	1.103778
26	1	0	-1.244867	-1.619836	0.281331
27	1	0	-1.269489	-2.126721	2.629481
28	1	0	-3.512077	-2.170629	1.102109
29	1	0	-2.562316	0.204445	2.321529
30	1	0	-3.224153	-0.511504	-0.577574
31	1	0	-5.232984	0.009819	0.828372
32	1	0	-4.489908	1.556336	1.276312
33	1	0	-4.545700	2.116749	-0.952052
34	1	0	-0.487683	2.742061	-0.255923
35	1	0	-1.051218	0.208867	-1.331682
36	1	0	-0.281061	0.996907	1.527140
37	1	0	3.082033	-1.708673	-0.432478
38	1	0	5.466422	-2.074502	-0.973313
39	1	0	6.386679	1.904794	0.359390
40	1	0	4.018970	2.262388	0.898972
41	1	0	1.021799	-2.011112	-0.015561
42	1	0	0.557186	-3.059599	0.954366
43	1	0	-2.146972	2.881719	1.133932
44	1	0	-1.587954	4.198336	1.736963
45	1	0	-0.506491	-3.208040	-1.098788
46	1	0	-1.078324	-2.447237	-2.303798
47	1	0	-2.928298	-3.058664	-0.922105
48	1	0	-4.332898	-2.383613	-0.990961
49	1	0	-2.696169	2.917006	-1.646277
50	1	0	-3.049807	3.919413	-0.597466
51	8	0	-5.410697	-1.161578	-1.871603
52	1	0	-6.348578	-1.344119	-1.752791
53	1	0	-5.309671	-0.219190	-1.646812
54	8	0	7.346329	-0.420731	-0.684308
55	6	0	8.341196	0.576236	-0.482180
56	1	0	9.277866	0.136217	-0.817403
57	1	0	8.127740	1.471047	-1.073228
58	1	0	8.421499	0.841558	0.575599

Structure 12.6H_2O (M06-2X, H_2O)

Energy (Hartrees): = - 1509.5455508
No imaginary frequencies

Standard orientation:

Center Number	Atomic Number	Atomic Type	Coordinates (Angstroms) X	Y	Z

1	6	0	3.772749	-0.916036	-0.355503
2	6	0	3.393126	0.298305	0.238471
3	6	0	4.380461	1.235016	0.538436
4	6	0	5.722274	0.990562	0.262376
5	6	0	6.081433	-0.220042	-0.329095
6	6	0	5.098173	-1.172671	-0.634398
7	6	0	1.999009	0.625219	0.552756
8	7	0	1.023020	-0.170763	0.363037
9	6	0	-0.305432	0.330709	0.675878
10	6	0	-0.972288	0.836702	-0.611634
11	8	0	-2.293153	1.270308	-0.304965
12	6	0	-3.159747	0.248505	0.189314
13	6	0	-2.594043	-0.298214	1.499969
14	6	0	-1.164468	-0.773281	1.274599
15	8	0	-0.307651	1.925498	-1.169376
16	6	0	-4.532273	0.861147	0.370558
17	8	0	-5.154756	1.165821	-0.869497
18	8	0	-3.379518	-1.359956	2.010458
19	8	0	-0.572669	-1.192960	2.492174
20	8	0	-3.693382	-3.040481	-0.139104
21	8	0	-1.505204	-2.557703	-1.775735
22	8	0	0.832899	-2.899514	-0.346303
23	8	0	-1.206098	3.635085	0.885178
24	8	0	-3.597144	3.418317	-1.513066
25	1	0	1.835356	1.622253	0.977667
26	1	0	-1.207421	-1.614902	0.569424
27	1	0	-1.199250	-1.800910	2.904766
28	1	0	-3.469552	-2.025128	1.292478
29	1	0	-2.594288	0.497828	2.253713
30	1	0	-3.213035	-0.560706	-0.550780
31	1	0	-5.168582	0.140714	0.885645
32	1	0	-4.445997	1.761145	0.990941
33	1	0	-4.707485	1.946852	-1.241640
34	1	0	-0.345640	2.644120	-0.510077
35	1	0	-1.008449	0.043966	-1.365366
36	1	0	-0.263089	1.176362	1.376268
37	1	0	3.023693	-1.660805	-0.595851
38	1	0	5.406407	-2.104174	-1.093842
39	1	0	6.464408	1.738258	0.506035
40	1	0	4.097232	2.176318	0.997881
41	1	0	0.965427	-1.954071	-0.107683
42	1	0	0.584968	-3.318974	0.485524
43	1	0	-1.883712	2.943466	0.879079
44	1	0	-0.735640	3.499381	1.715355
45	1	0	-0.658870	-2.750652	-1.324148
46	1	0	-1.472597	-3.047077	-2.604218
47	1	0	-2.902118	-2.919096	-0.699475
48	1	0	-4.388321	-2.526979	-0.592902
49	1	0	-2.887942	2.813184	-1.244082
50	1	0	-3.740924	3.961144	-0.729406
51	8	0	-5.709854	-1.529570	-1.391447
52	1	0	-6.443426	-1.561428	-0.767102
53	1	0	-5.483054	-0.584649	-1.433766
54	8	0	7.354112	-0.564631	-0.646870
55	6	0	8.379067	0.393359	-0.395990
56	1	0	9.304005	-0.065082	-0.737548
57	1	0	8.195754	1.313625	-0.955833
58	1	0	8.452109	0.613982	0.671685

Structure 19 (B3LYP, Gas Phase)

Energy (Hartrees): = - 1140.9750043
No imaginary frequencies

Standard orientation:

Center Number	Atomic Number	Atomic Type	Coordinates (Angstroms) X	Y	Z
1	6	0	-2.133153	1.368934	-0.433324
2	6	0	-1.505787	0.221720	0.383598
3	6	0	-2.311896	-1.061317	0.127690
4	6	0	-3.797132	-0.829713	0.376026
5	6	0	-4.293251	0.354104	-0.464396
6	1	0	-2.030101	1.154846	-1.513073
7	1	0	-2.179407	-1.350374	-0.926209
8	1	0	-3.939357	-0.578394	1.439220
9	1	0	-4.175603	0.111713	-1.533109
10	1	0	-1.596439	0.504952	1.443933
11	8	0	-3.513158	1.500914	-0.118720
12	6	0	-5.764940	0.693480	-0.210421
13	1	0	-5.916993	0.837330	0.872364
14	1	0	-5.991195	1.644900	-0.701492
15	8	0	-6.633607	-0.279672	-0.753027
16	1	0	-6.325564	-1.141603	-0.431078

17	8	0	-4.548747	-1.990368	0.034746
18	1	0	-4.122766	-2.733704	0.486840
19	8	0	-1.917583	-2.129636	0.983041
20	1	0	-1.019504	-2.387193	0.734390
21	8	0	-1.490750	2.548239	-0.074958
22	1	0	-1.916286	3.270601	-0.557645
23	7	0	-0.130229	0.005669	-0.022369
24	6	0	2.223770	0.197452	0.455406
25	6	0	2.634533	-0.322952	-0.784627
26	6	0	3.191019	0.575278	1.399197
27	6	0	3.983727	-0.465340	-1.075634
28	1	0	1.876539	-0.605909	-1.506277
29	6	0	4.547689	0.438345	1.121492
30	1	0	2.877266	0.980663	2.356951
31	6	0	4.921513	-0.081841	-0.114684
32	1	0	4.326467	-0.861825	-2.022902
33	1	0	5.311127	0.723128	1.834049
34	6	0	0.795302	0.354620	0.782777
35	1	0	0.576060	0.797145	1.765586
36	7	0	6.354752	-0.231532	-0.419266
37	8	0	6.658550	-0.693802	-1.518413
38	8	0	7.161012	0.114092	0.443740

Structure 19 (B3LYP, DMSO)

Energy (Hartrees): = - 1141.0021493
No imaginary frequencies

Standard orientation:

Center Number	Atomic Number	Atomic Type	Coordinates (Angstroms) X	Y	Z
1	6	0	-2.201865	1.490845	-0.217271
2	6	0	-1.510261	0.265895	0.406468
3	6	0	-2.249289	-1.007301	-0.051492
4	6	0	-3.744188	-0.893567	0.225686
5	6	0	-4.307427	0.386013	-0.405243
6	1	0	-2.106520	1.452068	-1.315823
7	1	0	-2.105342	-1.122677	-1.135309
8	1	0	-3.897725	-0.838852	1.314203
9	1	0	-4.185368	0.331901	-1.498830
10	1	0	-1.596948	0.356246	1.500013
11	8	0	-3.588264	1.501021	0.123988
12	6	0	-5.788380	0.604707	-0.087744
13	1	0	-5.925851	0.598418	1.005903
14	1	0	-6.083736	1.594712	-0.450682
15	8	0	-6.621103	-0.349210	-0.730774
16	1	0	-6.219260	-1.216322	-0.554101
17	8	0	-4.440713	-2.018755	-0.314788
18	1	0	-4.007148	-2.807961	0.046130
19	8	0	-1.795880	-2.171418	0.634891
20	1	0	-0.922521	-2.399904	0.283412
21	8	0	-1.620281	2.637742	0.309731
22	1	0	-1.993534	3.397431	-0.163781
23	7	0	-0.132141	0.197658	-0.041531
24	6	0	2.214837	0.068575	0.487033
25	6	0	2.638823	0.064502	-0.854026
26	6	0	3.170084	0.002365	1.513531
27	6	0	3.988621	-0.004246	-1.165524
28	1	0	1.898603	0.117876	-1.644428
29	6	0	4.527386	-0.066159	1.217524
30	1	0	2.846280	0.006302	2.549892
31	6	0	4.915482	-0.068667	-0.121277
32	1	0	4.328881	-0.007617	-2.193232
33	1	0	5.271995	-0.115633	2.001752
34	6	0	0.785975	0.147320	0.844121
35	1	0	0.564924	0.166617	1.919798
36	7	0	6.342542	-0.138817	-0.445335
37	8	0	6.668540	-0.153053	-1.635512
38	8	0	7.150411	-0.179602	0.486517

Structure 19 (M06-2X, Gas Phase)

Energy (Hartrees): = - 1140.8290509
No imaginary frequencies

Standard orientation:

Center Number	Atomic Number	Atomic Type	Coordinates (Angstroms) X	Y	Z
1	6	0	-2.063783	1.282230	-0.477245
2	6	0	-1.506102	0.171138	0.416932

3	6	0	-2.343428	-1.086564	0.223431
4	6	0	-3.810992	-0.781902	0.437918
5	6	0	-4.237224	0.351553	-0.490808
6	1	0	-1.946618	1.003907	-1.537855
7	1	0	-2.210099	-1.442764	-0.808002
8	1	0	-3.954500	-0.450831	1.476667
9	1	0	-4.099273	0.031612	-1.534796
10	1	0	-1.604034	0.526600	1.451957
11	8	0	-3.432999	1.485603	-0.203686
12	6	0	-5.695843	0.747711	-0.304118
13	1	0	-5.886118	0.915225	0.765202
14	1	0	-5.865952	1.689536	-0.827917
15	8	0	-6.567035	-0.208888	-0.855764
16	1	0	-6.319800	-1.064729	-0.488150
17	8	0	-4.604676	-1.921249	0.167358
18	1	0	-4.217390	-2.656183	0.655363
19	8	0	-1.995563	-2.105285	1.140346
20	1	0	-1.090058	-2.372350	0.955547
21	8	0	-1.379434	2.445659	-0.171753
22	1	0	-1.750864	3.156554	-0.702238
23	7	0	-0.131441	-0.122062	0.061840
24	6	0	2.209706	0.264245	0.415546
25	6	0	2.608867	-0.556789	-0.643410
26	6	0	3.168890	0.906083	1.198598
27	6	0	3.953202	-0.737891	-0.916211
28	1	0	1.846867	-1.039644	-1.241867
29	6	0	4.521707	0.734336	0.938120
30	1	0	2.855727	1.544522	2.017047
31	6	0	4.883476	-0.087264	-0.115658
32	1	0	4.295322	-1.364097	-1.728466
33	1	0	5.288438	1.217482	1.527452
34	6	0	0.777727	0.462953	0.719470
35	1	0	0.548501	1.151216	1.542313
36	7	0	6.322282	-0.279022	-0.402467
37	8	0	6.612638	-0.987272	-1.339874
38	8	0	7.114163	0.284243	0.319218

Structure 19 (M06-2X, DMSO)

Energy (Hartrees): = - 1140.8584074
No imaginary frequencies

Standard orientation:

Center Number	Atomic Number	Atomic Type	Coordinates (Angstroms) X	Y	Z
1	6	0	-2.206234	1.499073	-0.173642
2	6	0	-1.511180	0.281242	0.432977
3	6	0	-2.221036	-0.987699	-0.045264
4	6	0	-3.706922	-0.893447	0.237066
5	6	0	-4.270244	0.376050	-0.394759
6	1	0	-2.103945	1.478269	-1.269584
7	1	0	-2.079336	-1.086360	-1.129506
8	1	0	-3.861310	-0.840622	1.323729
9	1	0	-4.118275	0.332433	-1.483512
10	1	0	-1.597538	0.353839	1.525960
11	8	0	-3.584667	1.488584	0.157266
12	6	0	-5.754726	0.560219	-0.116229
13	1	0	-5.931196	0.476132	0.964837
14	1	0	-6.046281	1.564125	-0.430773
15	8	0	-6.542178	-0.359533	-0.846882
16	1	0	-6.159245	-1.231339	-0.690291
17	8	0	-4.388529	-2.014929	-0.303305
18	1	0	-3.937337	-2.803178	0.022629
19	8	0	-1.749706	-2.143674	0.623212
20	1	0	-0.876467	-2.359464	0.277222
21	8	0	-1.636808	2.633983	0.373699
22	1	0	-1.997167	3.399154	-0.089523
23	7	0	-0.136188	0.235434	-0.014712
24	6	0	2.199710	0.057489	0.499575
25	6	0	2.605589	0.121157	-0.837346
26	6	0	3.151928	-0.070444	1.511811
27	6	0	3.948805	0.057737	-1.162677
28	1	0	1.859956	0.223776	-1.615646
29	6	0	4.503848	-0.133808	1.202919
30	1	0	2.832930	-0.119570	2.546906
31	6	0	4.872049	-0.067738	-0.131232
32	1	0	4.282916	0.106078	-2.190018
33	1	0	5.254757	-0.233134	1.974829
34	6	0	0.768539	0.129973	0.865080
35	1	0	0.542807	0.093024	1.937298
36	7	0	6.304540	-0.131794	-0.471218
37	8	0	6.608231	-0.144753	-1.645824
38	8	0	7.103094	-0.166862	0.441494

Structure 19 (M06-2X/def2-TZVP, Gas Phase)

Energy (Hartrees): = -1140.978291
No imaginary frequencies

Standard orientation:

Center Number	Atomic Number	Atomic Type	Coordinates (Angstroms) X	Y	Z
1	6	0	-2.095715	1.324402	-0.444875
2	6	0	-1.503735	0.194367	0.398278
3	6	0	-2.316807	-1.071867	0.162958
4	6	0	-3.787026	-0.807003	0.404538
5	6	0	-4.248246	0.353463	-0.470680
6	1	0	-1.983466	1.089667	-1.515140
7	1	0	-2.186739	-1.383697	-0.881825
8	1	0	-3.926434	-0.520760	1.455913
9	1	0	-4.120193	0.078968	-1.528033
10	1	0	-1.594570	0.501238	1.448962
11	8	0	-3.463435	1.489066	-0.151929
12	6	0	-5.707729	0.718203	-0.243145
13	1	0	-5.883611	0.838751	0.833498
14	1	0	-5.900752	1.677725	-0.723500
15	8	0	-6.581421	-0.222003	-0.816325
16	1	0	-6.324136	-1.096904	-0.500898
17	8	0	-4.560596	-1.949778	0.099408
18	1	0	-4.158142	-2.702817	0.548294
19	8	0	-1.934304	-2.117337	1.033969
20	1	0	-1.033925	-2.383351	0.819220
21	8	0	-1.432789	2.490615	-0.106347
22	1	0	-1.816957	3.219198	-0.605286
23	7	0	-0.133867	-0.047402	0.009709
24	6	0	2.210789	0.208089	0.445546
25	6	0	2.610940	-0.405615	-0.742035
26	6	0	3.168516	0.674641	1.341543
27	6	0	3.953315	-0.554820	-1.030322
28	1	0	1.853204	-0.756954	-1.429259
29	6	0	4.519314	0.532643	1.066998
30	1	0	2.854138	1.154547	2.260395
31	6	0	4.881886	-0.081489	-0.116000
32	1	0	4.293871	-1.024139	-1.941641
33	1	0	5.282202	0.886416	1.744690
34	6	0	0.781308	0.376832	0.768385
35	1	0	0.557899	0.900439	1.705448
36	7	0	6.318606	-0.238621	-0.419312
37	8	0	6.612419	-0.782628	-1.458381
38	8	0	7.109322	0.186161	0.390761

Structure 19 (M06-2X/def2-TZVP, DMSO)

Energy (Hartrees): = -1141.008484
No imaginary frequencies

Standard orientation:

Center Number	Atomic Number	Atomic Type	Coordinates (Angstroms) X	Y	Z
1	6	0	-2.206298	1.493053	-0.182409
2	6	0	-1.510137	0.278156	0.425523
3	6	0	-2.220786	-0.988252	-0.053498
4	6	0	-3.704961	-0.897170	0.232130
5	6	0	-4.271549	0.371990	-0.394638
6	1	0	-2.103301	1.472814	-1.277012
7	1	0	-2.080349	-1.082207	-1.137461
8	1	0	-3.857324	-0.845455	1.318043
9	1	0	-4.130622	0.328516	-1.484076
10	1	0	-1.596197	0.349305	1.517829
11	8	0	-3.581674	1.483385	0.147501
12	6	0	-5.749603	0.564091	-0.096262
13	1	0	-5.912639	0.483903	0.985794
14	1	0	-6.041785	1.567526	-0.408802
15	8	0	-6.558588	-0.350158	-0.808243
16	1	0	-6.183129	-1.229695	-0.669309
17	8	0	-4.389175	-2.016913	-0.305236
18	1	0	-3.947546	-2.813259	0.017396
19	8	0	-1.740684	-2.143823	0.606116
20	1	0	-0.851269	-2.337933	0.284897
21	8	0	-1.640104	2.632845	0.359336
22	1	0	-1.978290	3.401032	-0.118186
23	7	0	-0.137521	0.230915	-0.019511
24	6	0	2.200215	0.061010	0.489069
25	6	0	2.611826	0.111985	-0.843646

26	6	0	3.146832	-0.055045	1.504053
27	6	0	3.954060	0.047003	-1.161387
28	1	0	1.872578	0.205522	-1.627781
29	6	0	4.497600	-0.119628	1.202928
30	1	0	2.823192	-0.093649	2.537062
31	6	0	4.871398	-0.066898	-0.127126
32	1	0	4.290108	0.085766	-2.187374
33	1	0	5.242472	-0.210187	1.980160
34	6	0	0.771146	0.134068	0.853495
35	1	0	0.548029	0.106579	1.925545
36	7	0	6.302550	-0.132885	-0.458803
37	8	0	6.615334	-0.131417	-1.630099
38	8	0	7.096444	-0.184041	0.455886

Structure 19.5H_2O (M06-2X, Gas Phase)

Energy (Hartrees): = - 1523.0078903
No imaginary frequencies

Standard orientation:

Center Number	Atomic Number	Atomic Type	Coordinates (Angstroms) X	Y	Z
1	6	0	-3.362538	0.675799	0.118564
2	6	0	-2.807894	-0.587981	0.344118
3	6	0	-3.628578	-1.717499	0.351903
4	6	0	-4.990254	-1.603439	0.112937
5	6	0	-5.507473	-0.339181	-0.113535
6	6	0	-4.722603	0.805296	-0.110469
7	6	0	-1.363926	-0.767382	0.594573
8	7	0	-0.494875	0.099764	0.284419
9	6	0	0.894597	-0.216050	0.575843
10	6	0	1.517044	-0.979588	-0.609608
11	8	0	2.889835	-1.204488	-0.333207
12	6	0	3.704536	-0.037345	-0.184930
13	6	0	3.175925	0.758648	1.004434
14	6	0	1.694938	1.059697	0.796447
15	8	0	0.932801	-2.213395	-0.830043
16	6	0	5.124741	-0.539732	0.004512
17	8	0	5.591379	-1.241787	-1.110837
18	8	0	3.878625	1.952447	1.246555
19	8	0	1.182512	1.758004	1.919771
20	8	0	3.416147	3.063639	-1.174952
21	8	0	1.117259	4.442512	-1.208716
22	8	0	-0.699894	2.941662	0.157671
23	8	0	2.482537	-3.396750	1.193501
24	8	0	4.006493	-3.637601	-1.281619
25	1	0	-1.080388	-1.714884	1.068739
26	1	0	1.603743	1.687484	-0.102563
27	1	0	1.905541	2.329126	2.213700
28	1	0	3.855250	2.472302	0.418228
29	1	0	3.279266	0.146997	1.911212
30	1	0	3.642857	0.565409	-1.099278
31	1	0	5.775368	0.321445	0.167379
32	1	0	5.152595	-1.152559	0.920997
33	1	0	5.118013	-2.088114	-1.164679
34	1	0	1.127342	-2.775478	-0.062129
35	1	0	1.410060	-0.396993	-1.531602
36	1	0	0.980543	-0.860622	1.463791
37	1	0	-2.735256	1.558248	0.146158
38	1	0	-5.183513	1.768695	-0.279331
39	1	0	-5.647435	-2.461672	0.102892
40	1	0	-3.195626	-2.694644	0.534612
41	1	0	-0.746753	2.023781	-0.159019
42	1	0	-0.262835	2.808872	1.012834
43	1	0	2.964029	-2.588036	0.955789
44	1	0	2.621215	-3.557146	2.129097
45	1	0	0.382267	3.939017	-0.802522
46	1	0	1.038398	5.338069	-0.875641
47	1	0	2.615694	3.628449	-1.239493
48	1	0	3.986597	3.290799	-1.911144
49	1	0	3.219854	-3.153845	-1.558188
50	1	0	3.718112	-4.067405	-0.469251
51	7	0	-6.960794	-0.202776	-0.358422
52	8	0	-7.391363	0.913031	-0.541537
53	8	0	-7.622124	-1.216484	-0.359251

Structure 19.5H_2O (M06-2X, DMSO)

Energy (Hartrees): = - 1523.0509238
No imaginary frequencies

Standard orientation:

Center Number	Atomic Number	Atomic Type	Coordinates (Angstroms) X	Y	Z
1	6	0	-3.218004	0.115861	-0.718783
2	6	0	-2.783508	-0.647525	0.369768
3	6	0	-3.714784	-1.282850	1.192543
4	6	0	-5.074600	-1.149185	0.950517
5	6	0	-5.472156	-0.376455	-0.129012
6	6	0	-4.570872	0.258182	-0.974645
7	6	0	-1.347074	-0.798458	0.679024
8	7	0	-0.464501	-0.075243	0.127640
9	6	0	0.921031	-0.318318	0.478144
10	6	0	1.628653	-0.968236	-0.722827
11	8	0	3.000433	-1.141788	-0.402451
12	6	0	3.737145	0.055270	-0.159836
13	6	0	3.117987	0.771421	1.038276
14	6	0	1.629522	0.995084	0.788646
15	8	0	1.127059	-2.216098	-1.044634
16	6	0	5.167391	-0.386455	0.086405
17	8	0	5.701460	-1.081257	-1.014679
18	8	0	3.746154	1.995945	1.339956
19	8	0	1.033394	1.598278	1.929946
20	8	0	3.326626	3.496328	-0.885780
21	8	0	0.797583	4.431181	-1.013317
22	8	0	-0.928925	2.755473	0.276049
23	8	0	2.616727	-3.479573	0.980684
24	8	0	4.164600	-3.507431	-1.424976
25	1	0	-1.092847	-1.561484	1.422973
26	1	0	1.523016	1.664210	-0.077976
27	1	0	1.689889	2.224773	2.266772
28	1	0	3.668165	2.566716	0.546626
29	1	0	3.229996	0.129695	1.921164
30	1	0	3.691588	0.693530	-1.053356
31	1	0	5.784548	0.496715	0.260621
32	1	0	5.187865	-0.999693	0.999824
33	1	0	5.211372	-1.914617	-1.108794
34	1	0	1.307910	-2.802354	-0.290192
35	1	0	1.525248	-0.330667	-1.608228
36	1	0	1.011990	-0.997526	1.339256
37	1	0	-2.493408	0.578953	-1.377572
38	1	0	-4.925021	0.841768	-1.813297
39	1	0	-5.809273	-1.629936	1.581591
40	1	0	-3.373251	-1.881231	2.029685
41	1	0	-0.972830	1.879647	-0.139646
42	1	0	-0.430259	2.548641	1.082506
43	1	0	3.015138	-2.602395	1.072261
44	1	0	2.548838	-3.851083	1.867432
45	1	0	0.128804	3.844412	-0.603915
46	1	0	0.670789	5.290394	-0.598448
47	1	0	2.413638	3.850496	-0.954431
48	1	0	3.549664	3.165441	-1.761132
49	1	0	3.438737	-2.933697	-1.699574
50	1	0	3.844930	-3.820804	-0.567542
51	7	0	-6.914626	-0.228551	-0.398565
52	8	0	-7.247068	0.476346	-1.328085
53	8	0	-7.690010	-0.819076	0.323605

Structure 19.5H_2O (M06-2X, H_2O)

Energy (Hartrees): = - 1523.071957
No imaginary frequencies

Standard orientation:

Center Number	Atomic Number	Atomic Type	Coordinates (Angstroms) X	Y	Z
1	6	0	-3.306661	0.592720	-0.373522
2	6	0	-2.825236	-0.541867	0.288635
3	6	0	-3.716627	-1.516648	0.741361
4	6	0	-5.082282	-1.368348	0.548770
5	6	0	-5.525035	-0.229224	-0.103408
6	6	0	-4.666327	0.757559	-0.571628
7	6	0	-1.385873	-0.750857	0.539013
8	7	0	-0.507281	0.129582	0.282017
9	6	0	0.875540	-0.235226	0.552927
10	6	0	1.527767	-0.775711	-0.729035
11	8	0	2.887543	-1.080822	-0.449065
12	6	0	3.695673	0.032790	-0.064199
13	6	0	3.131542	0.643485	1.219544
14	6	0	1.661031	0.981215	1.015467
15	8	0	0.931719	-1.948768	-1.181883
16	6	0	5.106788	-0.485505	0.125898
17	8	0	5.684393	-0.943475	-1.083634
18	8	0	3.842425	1.805191	1.605541
19	8	0	1.076125	1.471659	2.209759
20	8	0	3.687575	3.438615	-0.615085

21	8	0	1.322371	2.822315	-1.906428
22	8	0	-0.638274	2.991260	0.082866
23	8	0	2.000419	-3.452892	0.939344
24	8	0	4.239701	-3.358918	-1.350713
25	1	0	-1.115315	-1.716426	0.979071
26	1	0	1.611083	1.748147	0.232374
27	1	0	1.644786	2.187727	2.520741
28	1	0	3.788670	2.436278	0.856730
29	1	0	3.227084	-0.081197	2.036145
30	1	0	3.674926	0.784247	-0.865907
31	1	0	5.724652	0.329636	0.504682
32	1	0	5.086759	-1.284509	0.878395
33	1	0	5.299848	-1.815989	-1.270708
34	1	0	1.056505	-2.616444	-0.479646
35	1	0	1.469680	-0.035578	-1.533707
36	1	0	0.937641	-1.027567	1.311055
37	1	0	-2.619476	1.344596	-0.738648
38	1	0	-5.057945	1.628154	-1.079096
39	1	0	-5.785136	-2.111086	0.899472
40	1	0	-3.337329	-2.394683	1.251209
41	1	0	-0.698944	2.015189	0.126596
42	1	0	-0.179880	3.219881	0.900482
43	1	0	2.640034	-2.727139	0.900537
44	1	0	1.527079	-3.308705	1.766652
45	1	0	0.608369	2.981676	-1.259224
46	1	0	1.117219	3.392006	-2.655464
47	1	0	2.851888	3.242830	-1.086659
48	1	0	4.372762	3.056957	-1.174938
49	1	0	3.521325	-2.719378	-1.227521
50	1	0	4.305660	-3.788377	-0.490033
51	7	0	-6.970935	-0.056794	-0.310500
52	8	0	-7.359784	0.990393	-0.786174
53	8	0	-7.707492	-0.969067	0.005203

Structure 19·5H₂O (M06-2X/def2-TZVP, Gas Phase)

Energy (Hartrees): = -1523.196479
No imaginary frequencies

Standard orientation:

Center Number	Atomic Number	Atomic Type	Coordinates (Angstroms) X	Y	Z
1	6	0	-3.233283	0.035150	-0.751600
2	6	0	-2.800760	-0.656490	0.379867
3	6	0	-3.733510	-1.230511	1.239696
4	6	0	-5.090453	-1.105577	0.992561
5	6	0	-5.485239	-0.406296	-0.131620
6	6	0	-4.583334	0.165868	-1.014095
7	6	0	-1.366982	-0.795592	0.690855
8	7	0	-0.473402	-0.130682	0.097062
9	6	0	0.904835	-0.348792	0.468314
10	6	0	1.656180	-0.975962	-0.715037
11	8	0	3.021732	-1.111766	-0.370615
12	6	0	3.720725	0.103322	-0.107114
13	6	0	3.062739	0.779910	1.090094
14	6	0	1.576280	0.972437	0.815910
15	8	0	1.183241	-2.226914	-1.058298
16	6	0	5.164961	-0.283957	0.143658
17	8	0	5.767168	-0.857082	-0.980781
18	8	0	3.648319	2.007020	1.441323
19	8	0	0.943025	1.538450	1.950428
20	8	0	3.222088	3.349954	-0.911710
21	8	0	0.734162	4.399819	-1.073992
22	8	0	-0.991811	2.742270	0.226934
23	8	0	2.774784	-3.500995	0.957605
24	8	0	4.422669	-3.387419	-1.510563
25	1	0	-1.116647	-1.507561	1.486343
26	1	0	1.473186	1.650391	-0.043735
27	1	0	1.587648	2.148620	2.337939
28	1	0	3.619248	2.593029	0.658047
29	1	0	3.169609	0.123317	1.963108
30	1	0	3.661025	0.755879	-0.987120
31	1	0	5.725232	0.613086	0.408749
32	1	0	5.194111	-0.960344	1.011713
33	1	0	5.380751	-1.734111	-1.143301
34	1	0	1.330256	-2.831854	-0.312847
35	1	0	1.556471	-0.337549	-1.599515
36	1	0	0.994197	-1.036006	1.324040
37	1	0	-2.501556	0.452080	-1.430479
38	1	0	-4.946904	0.693709	-1.883448
39	1	0	-5.834484	-1.534927	1.647020
40	1	0	-3.395102	-1.774333	2.113233
41	1	0	-1.033367	1.873464	-0.200899
42	1	0	-0.541121	2.532889	1.060763
43	1	0	3.161913	-2.624608	0.793697

44	1	0	2.928719	-3.726524	1.878357
45	1	0	0.049330	3.843140	-0.643916
46	1	0	0.474276	5.313620	-0.940717
47	1	0	2.361340	3.811617	-1.009090
48	1	0	3.806335	3.675752	-1.599451
49	1	0	3.602305	-2.949573	-1.769454
50	1	0	4.159250	-3.937815	-0.763473
51	7	0	-6.930338	-0.268874	-0.406663
52	8	0	-7.252527	0.343181	-1.397682
53	8	0	-7.696948	-0.777712	0.377690

Structure 19·5H_2O (M06-2X/def2-TZVP, DMSO)

Energy (Hartrees): = -1523.240750
No imaginary frequencies

Standard orientation:

Center Number	Atomic Number	Atomic Type	Coordinates (Angstroms) X	Y	Z
1	6	0	-3.233691	0.003526	-0.775114
2	6	0	-2.794993	-0.649374	0.377707
3	6	0	-3.721513	-1.186970	1.268361
4	6	0	-5.079879	-1.062143	1.030248
5	6	0	-5.481576	-0.401145	-0.116053
6	6	0	-4.585381	0.132509	-1.029950
7	6	0	-1.359749	-0.789911	0.687115
8	7	0	-0.469178	-0.144616	0.063311
9	6	0	0.910606	-0.358876	0.439183
10	6	0	1.664932	-0.978210	-0.744950
11	8	0	3.030832	-1.122037	-0.397510
12	6	0	3.729070	0.086103	-0.113801
13	6	0	3.058978	0.772393	1.072528
14	6	0	1.578026	0.967525	0.776795
15	8	0	1.199707	-2.232022	-1.095597
16	6	0	5.159817	-0.316188	0.178536
17	8	0	5.772529	-0.951203	-0.917771
18	8	0	3.652512	2.002388	1.410352
19	8	0	0.927755	1.553975	1.894223
20	8	0	3.334179	3.684859	-0.731540
21	8	0	0.730550	4.361451	-1.150693
22	8	0	-1.015957	2.731583	0.201851
23	8	0	2.753260	-3.559664	0.924809
24	8	0	4.355848	-3.455241	-1.489158
25	1	0	-1.111617	-1.477021	1.502280
26	1	0	1.485622	1.632623	-0.094579
27	1	0	1.557412	2.182510	2.278890
28	1	0	3.585885	2.605935	0.638850
29	1	0	3.156830	0.121097	1.948947
30	1	0	3.696854	0.739568	-0.996547
31	1	0	5.738415	0.578056	0.411825
32	1	0	5.164183	-0.962540	1.066786
33	1	0	5.336113	-1.807014	-1.064837
34	1	0	1.353376	-2.832499	-0.346213
35	1	0	1.567742	-0.334433	-1.625762
36	1	0	0.999393	-1.039745	1.298220
37	1	0	-2.512887	0.394823	-1.481664
38	1	0	-4.942164	0.631300	-1.919301
39	1	0	-5.808923	-1.468560	1.715983
40	1	0	-3.375221	-1.702380	2.155917
41	1	0	-1.077892	1.876013	-0.252247
42	1	0	-0.510179	2.494575	0.998284
43	1	0	3.089899	-2.652821	0.989470
44	1	0	2.722509	-3.915587	1.821041
45	1	0	0.059750	3.814184	-0.689762
46	1	0	0.467894	5.279524	-1.024259
47	1	0	2.403020	3.951373	-0.892908
48	1	0	3.696286	3.432614	-1.587961
49	1	0	3.604326	-2.899848	-1.737220
50	1	0	4.057592	-3.844634	-0.654417
51	7	0	-6.922616	-0.263710	-0.381000
52	8	0	-7.261626	0.307757	-1.394644
53	8	0	-7.695780	-0.728630	0.428352

Structure 19·5H_2O (M06-2X/def2-TZVP, H_2O)

Energy (Hartrees): = -1523.258211
No imaginary frequencies

Standard orientation:

Center Number	Atomic Number	Atomic Type	Coordinates (Angstroms) X	Y	Z
1	6	0	-3.288585	0.553919	-0.430708
2	6	0	-2.817776	-0.536500	0.302909
3	6	0	-3.716271	-1.472660	0.811772
4	6	0	-5.077513	-1.326365	0.607449
5	6	0	-5.509878	-0.228284	-0.112491
6	6	0	-4.644073	0.717977	-0.639377
7	6	0	-1.382866	-0.746487	0.564483
8	7	0	-0.494580	0.116330	0.293372
9	6	0	0.882229	-0.256877	0.564962
10	6	0	1.536903	-0.779930	-0.720106
11	8	0	2.889172	-1.097702	-0.442812
12	6	0	3.693861	0.015496	-0.062457
13	6	0	3.143496	0.612578	1.231778
14	6	0	1.667870	0.944450	1.060342
15	8	0	0.929470	-1.935978	-1.198814
16	6	0	5.109382	-0.491531	0.105732
17	8	0	5.686019	-0.914411	-1.116759
18	8	0	3.856135	1.769762	1.621403
19	8	0	1.097525	1.375017	2.283271
20	8	0	3.688768	3.521312	-0.530890
21	8	0	1.310765	2.996781	-1.875712
22	8	0	-0.719149	2.997199	0.053654
23	8	0	1.921251	-3.538532	0.954290
24	8	0	4.247679	-3.356125	-1.511430
25	1	0	-1.122096	-1.702979	1.027836
26	1	0	1.594087	1.745153	0.312466
27	1	0	1.628407	2.116365	2.604493
28	1	0	3.794802	2.420854	0.889031
29	1	0	3.254002	-0.121969	2.036473
30	1	0	3.658091	0.772235	-0.858707
31	1	0	5.726367	0.315998	0.498645
32	1	0	5.103603	-1.309145	0.835994
33	1	0	5.310102	-1.783202	-1.339847
34	1	0	1.012959	-2.619810	-0.505996
35	1	0	1.489397	-0.026314	-1.512673
36	1	0	0.938168	-1.063435	1.307584
37	1	0	-2.594544	1.270266	-0.847509
38	1	0	-5.025112	1.556049	-1.204370
39	1	0	-5.784711	-2.041241	1.001326
40	1	0	-3.344867	-2.320047	1.374520
41	1	0	-0.719830	2.019735	0.132867
42	1	0	-0.287862	3.305893	0.860535
43	1	0	2.577005	-2.825218	0.930604
44	1	0	1.412258	-3.375174	1.758199
45	1	0	0.570166	3.069733	-1.240294
46	1	0	1.112073	3.622302	-2.581954
47	1	0	2.859352	3.338607	-1.020591
48	1	0	4.395568	3.199416	-1.103216
49	1	0	3.538705	-2.756196	-1.226406
50	1	0	4.401314	-3.931257	-0.752015
51	7	0	-6.950887	-0.058173	-0.332889
52	8	0	-7.333028	0.968216	-0.854779
53	8	0	-7.694581	-0.950344	0.017815

Structure 19.6H_2O (B3LYP, Gas Phase)

Energy (Hartrees): = - 1599.6253479
No imaginary frequencies
Standard orientation:

Center Number	Atomic Number	Atomic Type	Coordinates (Angstroms) X	Y	Z
1	6	0	3.660638	-0.717560	-0.004737
2	6	0	3.190508	0.557771	0.358244
3	6	0	4.094900	1.629648	0.436212
4	6	0	5.440818	1.451320	0.135592
5	6	0	5.872890	0.179459	-0.230380
6	6	0	5.004237	-0.909913	-0.299997
7	6	0	1.776442	0.812795	0.682865
8	7	0	0.828602	0.003426	0.409056
9	6	0	-0.526431	0.379650	0.790134
10	6	0	-1.244104	1.054494	-0.410615
11	8	0	-2.607896	1.314978	-0.053595
12	6	0	-3.409092	0.155555	0.268382
13	6	0	-2.796633	-0.527732	1.497002
14	6	0	-1.335881	-0.865443	1.168817
15	8	0	-0.681398	2.257217	-0.812515
16	6	0	-4.844743	0.626164	0.451295
17	8	0	-5.449513	1.020252	-0.769390
18	8	0	-3.473289	-1.693069	1.906475

19	8	0	-0.731254	-1.548714	2.261009
20	8	0	-3.735281	-3.079322	-0.348871
21	8	0	-1.259493	-3.596123	-1.507047
22	8	0	0.824525	-2.862631	0.153372
23	8	0	-2.444629	3.900809	0.724501
24	8	0	-4.073689	3.308291	-1.621924
25	1	0	1.577985	1.768494	1.187312
26	1	0	-1.352004	-1.527516	0.290725
27	1	0	-1.460523	-2.079495	2.631022
28	1	0	-3.593828	-2.276330	1.101447
29	1	0	-2.820764	0.171584	2.345805
30	1	0	-3.383025	-0.535205	-0.582519
31	1	0	-5.423167	-0.208607	0.855151
32	1	0	-4.876812	1.439459	1.193526
33	1	0	-5.023057	1.852380	-1.081914
34	1	0	-0.937137	2.950136	-0.173372
35	1	0	-1.209455	0.379213	-1.275999
36	1	0	-0.527539	1.097719	1.626324
37	1	0	2.979293	-1.560355	-0.027920
38	1	0	5.391512	-1.882513	-0.575559
39	1	0	6.152680	2.265371	0.181341
40	1	0	3.737128	2.613366	0.726278
41	1	0	0.917008	-1.906709	-0.044917
42	1	0	0.488674	-2.817041	1.066524
43	1	0	-2.736583	2.972560	0.807842
44	1	0	-2.533305	4.307826	1.595380
45	1	0	-0.489019	-3.403568	-0.921616
46	1	0	-1.197612	-4.539822	-1.698875
47	1	0	-2.882402	-3.281254	-0.793765
48	1	0	-4.292590	-2.609054	-1.002467
49	1	0	-3.241095	2.859383	-1.829322
50	1	0	-3.810660	3.872886	-0.875460
51	8	0	-5.237152	-1.433866	-2.112089
52	1	0	-4.860224	-1.288939	-2.988099
53	1	0	-5.426914	-0.536093	-1.762606
54	7	0	7.298680	-0.023266	-0.544036
55	8	0	7.656072	-1.155756	-0.863960
56	8	0	8.043140	0.953403	-0.466753

Structure 19.6H_2O (B3LYP, DMSO)

Energy (Hartrees): = - 1599.667245
No imaginary frequencies

Standard orientation:

Center Number	Atomic Number	Atomic Type	Coordinates (Angstroms) X	Y	Z
1	6	0	3.663653	-0.701897	-0.104844
2	6	0	3.203973	0.540858	0.367519
3	6	0	4.118747	1.592548	0.543127
4	6	0	5.464624	1.424978	0.237175
5	6	0	5.886738	0.183736	-0.235461
6	6	0	5.005609	-0.886161	-0.407044
7	6	0	1.791030	0.788153	0.706683
8	7	0	0.839749	-0.004355	0.389406
9	6	0	-0.512252	0.378904	0.782899
10	6	0	-1.232403	1.050628	-0.417780
11	8	0	-2.589707	1.330137	-0.049494
12	6	0	-3.391225	0.182419	0.288766
13	6	0	-2.776321	-0.515460	1.508777
14	6	0	-1.323447	-0.862579	1.166813
15	8	0	-0.657993	2.243909	-0.829974
16	6	0	-4.818664	0.664597	0.495468
17	8	0	-5.443906	1.054852	-0.724792
18	8	0	-3.469822	-1.689116	1.894916
19	8	0	-0.702951	-1.553654	2.251963
20	8	0	-3.848116	-3.093976	-0.376211
21	8	0	-1.356846	-3.842327	-1.379748
22	8	0	0.780845	-2.869310	0.105838
23	8	0	-2.463632	3.952133	0.629226
24	8	0	-4.115064	3.343682	-1.617523
25	1	0	1.599837	1.716573	1.257898
26	1	0	-1.352298	-1.525559	0.290502
27	1	0	-1.415064	-2.113730	2.610397
28	1	0	-3.608557	-2.244305	1.078252
29	1	0	-2.796746	0.167509	2.367927
30	1	0	-3.378049	-0.510587	-0.561063
31	1	0	-5.403121	-0.158765	0.914128
32	1	0	-4.831888	1.487026	1.224970
33	1	0	-5.007397	1.881926	-1.041480
34	1	0	-0.894172	2.937077	-0.184190
35	1	0	-1.215125	0.370036	-1.279145
36	1	0	-0.504201	1.098491	1.615143
37	1	0	2.976012	-1.532670	-0.208562

38	1	0	5.372700	-1.839795	-0.764287
39	1	0	6.174677	2.232129	0.363652
40	1	0	3.769955	2.549952	0.917618
41	1	0	0.889070	-1.913915	-0.096473
42	1	0	0.453388	-2.808030	1.021983
43	1	0	-2.704743	3.033140	0.848288
44	1	0	-2.572103	4.467443	1.442595
45	1	0	-0.584435	-3.496369	-0.872182
46	1	0	-1.350620	-4.793737	-1.201044
47	1	0	-2.978077	-3.317545	-0.778685
48	1	0	-4.323405	-2.537102	-1.029363
49	1	0	-3.355432	2.854296	-1.968521
50	1	0	-3.720335	3.782739	-0.838240
51	8	0	-5.210771	-1.343410	-2.161426
52	1	0	-4.643038	-1.160035	-2.923831
53	1	0	-5.351313	-0.461738	-1.747206
54	7	0	7.305418	-0.009422	-0.552467
55	8	0	7.664223	-1.117310	-0.959511
56	8	0	8.070982	0.945343	-0.397045

Structure 19.6H_2O (B3LYP, H_2O)

Energy (Hartrees): = - 1599.6897608
No imaginary frequencies

Standard orientation:

Center Number	Atomic Number	Atomic Type	Coordinates (Angstroms) X	Y	Z
1	6	0	3.737112	-0.584305	-0.388788
2	6	0	3.329595	0.526606	0.372876
3	6	0	4.295739	1.423420	0.859481
4	6	0	5.645886	1.229136	0.593885
5	6	0	6.016083	0.120950	-0.165901
6	6	0	5.081493	-0.791672	-0.661668
7	6	0	1.919144	0.805459	0.691063
8	7	0	0.939294	0.084662	0.296045
9	6	0	-0.401301	0.502922	0.691213
10	6	0	-1.213237	0.885328	-0.564730
11	8	0	-2.547114	1.228012	-0.178337
12	6	0	-3.288577	0.141347	0.413309
13	6	0	-2.586445	-0.308499	1.702977
14	6	0	-1.114151	-0.638983	1.424438
15	8	0	-0.675238	1.978000	-1.251099
16	6	0	-4.714952	0.614041	0.658293
17	8	0	-5.510333	0.663720	-0.524718
18	8	0	-3.227488	-1.428239	2.301694
19	8	0	-0.423072	-0.892213	2.646208
20	8	0	-3.677482	-3.255053	0.310800
21	8	0	-1.846085	-2.736831	-1.711590
22	8	0	0.804884	-2.462377	-1.025581
23	8	0	-3.388655	3.918973	0.094488
24	8	0	-4.602605	2.719090	-2.096590
25	1	0	1.751032	1.697084	1.305652
26	1	0	-1.082934	-1.533888	0.785615
27	1	0	-0.975446	-1.531164	3.124545
28	1	0	-3.342402	-2.122146	1.598752
29	1	0	-2.624369	0.509407	2.433301
30	1	0	-3.308225	-0.694700	-0.296151
31	1	0	-5.194551	-0.097300	1.334600
32	1	0	-4.692921	1.594133	1.153124
33	1	0	-5.163296	1.380685	-1.114225
34	1	0	-0.529735	2.694895	-0.612089
35	1	0	-1.243142	0.050727	-1.275261
36	1	0	-0.372947	1.386141	1.347430
37	1	0	3.002319	-1.285912	-0.764796
38	1	0	5.408064	-1.641227	-1.247399
39	1	0	6.395287	1.916092	0.964549
40	1	0	3.983966	2.279812	1.448793
41	1	0	0.866438	-1.581486	-0.581020
42	1	0	0.917084	-3.092778	-0.298577
43	1	0	-2.962860	3.042915	0.181150
44	1	0	-4.073006	3.906056	0.780226
45	1	0	-0.893054	-2.662690	-1.461669
46	1	0	-1.879638	-3.482954	-2.326981
47	1	0	-2.941754	-3.154474	-0.337318
48	1	0	-4.423067	-2.820855	-0.162807
49	1	0	-3.804794	2.310306	-2.464438
50	1	0	-4.242443	3.269643	-1.361733
51	8	0	-5.535356	-1.971331	-1.371448
52	1	0	-4.889942	-2.029475	-2.091000
53	1	0	-5.537075	-1.012436	-1.140626
54	7	0	7.432443	-0.093727	-0.454122
55	8	0	7.753578	-1.081543	-1.122642
56	8	0	8.251582	0.721610	-0.017911

Structure 19.6H_2O (M06-2X, Gas Phase)

Energy (Hartrees): = - 1599.4513255
No imaginary frequencies

Standard orientation:

Center Number	Atomic Number	Atomic Type	Coordinates (Angstroms) X	Y	Z
1	6	0	3.596321	-0.746701	-0.093212
2	6	0	3.154389	0.503402	0.354212
3	6	0	4.067909	1.546270	0.516474
4	6	0	5.412752	1.361594	0.229552
5	6	0	5.816953	0.114046	-0.213587
6	6	0	4.936571	-0.946195	-0.378859
7	6	0	1.736624	0.761572	0.672714
8	7	0	0.812279	-0.074536	0.455368
9	6	0	-0.547646	0.309289	0.785614
10	6	0	-1.236749	0.901594	-0.457036
11	8	0	-2.579527	1.210553	-0.103751
12	6	0	-3.388900	0.085301	0.239977
13	6	0	-2.808141	-0.560012	1.497173
14	6	0	-1.350193	-0.914169	1.217371
15	8	0	-0.653830	2.066906	-0.919869
16	6	0	-4.802006	0.601312	0.408853
17	8	0	-5.322496	1.102745	-0.803683
18	8	0	-3.505240	-1.703893	1.915656
19	8	0	-0.744262	-1.509678	2.344512
20	8	0	-3.720342	-3.020189	-0.439071
21	8	0	-1.280414	-2.531179	-1.742996
22	8	0	0.795318	-2.851442	0.021201
23	8	0	-2.127571	3.681288	0.868289
24	8	0	-3.766433	3.347989	-1.509574
25	1	0	1.523457	1.740665	1.119216
26	1	0	-1.357530	-1.621597	0.379099
27	1	0	-1.435317	-2.054442	2.744100
28	1	0	-3.607231	-2.290654	1.130976
29	1	0	-2.850040	0.161710	2.324441
30	1	0	-3.360493	-0.632059	-0.587902
31	1	0	-5.436118	-0.226221	0.729875
32	1	0	-4.819564	1.370264	1.193995
33	1	0	-4.833499	1.909691	-1.055936
34	1	0	-0.791662	2.760473	-0.254598
35	1	0	-1.221166	0.169592	-1.272394
36	1	0	-0.567766	1.071839	1.580101
37	1	0	2.896310	-1.565164	-0.205563
38	1	0	5.310126	-1.900816	-0.722548
39	1	0	6.141050	2.152176	0.343457
40	1	0	3.722662	2.512562	0.866645
41	1	0	0.886029	-1.879862	0.106015
42	1	0	0.525608	-3.113198	0.908406
43	1	0	-2.563208	2.814049	0.868593
44	1	0	-2.171142	4.031053	1.760526
45	1	0	-0.511951	-2.814332	-1.208456
46	1	0	-1.092717	-2.790292	-2.647398
47	1	0	-2.907378	-2.984318	-0.970001
48	1	0	-4.398819	-2.576081	-0.976086
49	1	0	-2.958483	2.861970	-1.713065
50	1	0	-3.483394	3.930919	-0.795485
51	8	0	-5.507567	-1.417858	-1.886834
52	1	0	-6.433829	-1.635889	-2.005261
53	1	0	-5.481917	-0.458290	-1.722391
54	7	0	7.250001	-0.098163	-0.517661
55	8	0	7.581271	-1.198833	-0.895320
56	8	0	7.995433	0.843758	-0.368285

Structure 19.6H_2O (M06-2X, DMSO)

Energy (Hartrees): = - 1599.4941437
No imaginary frequencies

Standard orientation:

Center Number	Atomic Number	Atomic Type	Coordinates (Angstroms) X	Y	Z
1	6	0	-3.601936	0.731842	-0.126793
2	6	0	-3.151333	-0.507088	0.341769
3	6	0	-4.058842	-1.551461	0.530212
4	6	0	-5.405493	-1.377567	0.244567
5	6	0	-5.817801	-0.138749	-0.219079
6	6	0	-4.943642	0.923979	-0.408176

7	6	0	-1.731620	-0.756585	0.663696
8	7	0	-0.804471	0.060516	0.380295
9	6	0	0.550023	-0.324730	0.741745
10	6	0	1.261118	-0.923961	-0.485001
11	8	0	2.596386	-1.237781	-0.117330
12	6	0	3.398280	-0.123127	0.264708
13	6	0	2.790165	0.523788	1.506806
14	6	0	1.349365	0.898875	1.177893
15	8	0	0.684966	-2.088552	-0.956006
16	6	0	4.799327	-0.654560	0.476491
17	8	0	5.356817	-1.142734	-0.733249
18	8	0	3.497769	1.663145	1.939000
19	8	0	0.719334	1.519192	2.284194
20	8	0	3.873074	3.051919	-0.369371
21	8	0	1.361339	3.215345	-1.598017
22	8	0	-0.767092	2.891679	0.150741
23	8	0	2.136041	-3.750834	0.803233
24	8	0	3.788663	-3.376760	-1.503656
25	1	0	-1.523699	-1.704955	1.170653
26	1	0	1.388354	1.601031	0.333003
27	1	0	1.403374	2.065390	2.695754
28	1	0	3.648395	2.233984	1.152565
29	1	0	2.798326	-0.197459	2.332842
30	1	0	3.404143	0.601139	-0.558393
31	1	0	5.440444	0.151807	0.836850
32	1	0	4.777008	-1.441215	1.241644
33	1	0	4.857238	-1.938190	-0.999743
34	1	0	0.847768	-2.785213	-0.297118
35	1	0	1.258273	-0.196993	-1.306504
36	1	0	0.552823	-1.078938	1.542116
37	1	0	-2.910023	1.553995	-0.255562
38	1	0	-5.311331	1.876653	-0.763785
39	1	0	-6.119409	-2.177905	0.381480
40	1	0	-3.707312	-2.507384	0.901539
41	1	0	-0.879967	1.925754	0.044723
42	1	0	-0.455315	2.940199	1.064515
43	1	0	2.517007	-2.910430	1.094149
44	1	0	2.031876	-4.299400	1.588810
45	1	0	0.593540	3.216650	-0.992790
46	1	0	1.185740	2.491934	-2.209232
47	1	0	3.019110	3.157862	-0.826626
48	1	0	4.426947	2.522437	-0.969557
49	1	0	3.052283	-2.798771	-1.739094
50	1	0	3.443237	-3.803337	-0.705835
51	8	0	5.314777	1.309321	-2.073488
52	1	0	4.873065	1.201484	-2.922399
53	1	0	5.410407	0.403421	-1.726344
54	7	0	-7.248699	0.063941	-0.514017
55	8	0	-7.601216	1.165564	-0.879447
56	8	0	-7.995329	-0.881842	-0.374682

Structure 19.6H_2O (M06-2X, H_2O)

Energy (Hartrees): = - 1599.5171366
No imaginary frequencies

Standard orientation:

Center Number	Atomic Number	Atomic Type	Coordinates (Angstroms) X	Y	Z
1	6	0	3.640089	-0.551713	-0.431548
2	6	0	3.267085	0.616003	0.243082
3	6	0	4.245685	1.486822	0.726090
4	6	0	5.592038	1.203826	0.546524
5	6	0	5.926388	0.037418	-0.122262
6	6	0	4.978337	-0.848749	-0.619071
7	6	0	1.851802	0.966724	0.472116
8	7	0	0.891365	0.239675	0.073755
9	6	0	-0.456791	0.675545	0.385274
10	6	0	-1.287980	0.688084	-0.899403
11	8	0	-2.630895	1.021356	-0.582590
12	6	0	-3.274906	0.084575	0.286015
13	6	0	-2.545054	0.074397	1.627593
14	6	0	-1.083491	-0.286976	1.389634
15	8	0	-0.833607	1.630182	-1.818965
16	6	0	-4.727449	0.496162	0.410754
17	8	0	-5.404806	0.436696	-0.836166
18	8	0	-3.130320	-0.823092	2.551076
19	8	0	-0.338396	-0.206464	2.590728
20	8	0	-3.639261	-3.107050	1.105858
21	8	0	-2.145625	-2.931244	-1.229334
22	8	0	0.542650	-2.378520	-1.003438
23	8	0	-3.094085	3.613660	0.451363
24	8	0	-4.339033	2.765443	-2.014215
25	1	0	1.674636	1.901775	1.012488

26	1	0	-1.042107	-1.307687	0.978915
27	1	0	-0.820716	-0.722562	3.248617
28	1	0	-3.266953	-1.678318	2.089553
29	1	0	-2.595319	1.076079	2.070500
30	1	0	-3.209496	-0.909897	-0.168341
31	1	0	-5.227862	-0.190615	1.094573
32	1	0	-4.783343	1.508798	0.826142
33	1	0	-5.115587	1.209081	-1.356198
34	1	0	-0.745781	2.479441	-1.364261
35	1	0	-1.260063	-0.288915	-1.394644
36	1	0	-0.474566	1.689917	0.811135
37	1	0	2.884110	-1.227171	-0.811699
38	1	0	5.286279	-1.745401	-1.138538
39	1	0	6.361312	1.867191	0.916765
40	1	0	3.950619	2.389558	1.248340
41	1	0	0.719239	-1.472997	-0.674345
42	1	0	0.827473	-2.949780	-0.280815
43	1	0	-2.788434	2.724912	0.207586
44	1	0	-3.569965	3.477379	1.277753
45	1	0	-1.203135	-2.667709	-1.168288
46	1	0	-2.134972	-3.783801	-1.678950
47	1	0	-2.943434	-3.136530	0.421850
48	1	0	-4.404447	-2.833274	0.574770
49	1	0	-3.514489	2.264513	-2.082507
50	1	0	-4.197332	3.266917	-1.195363
51	8	0	-4.997952	-2.316091	-1.307405
52	1	0	-4.051989	-2.396890	-1.500815
53	1	0	-5.165399	-1.358191	-1.310160
54	7	0	7.349841	-0.277568	-0.317475
55	8	0	7.633633	-1.313324	-0.883899
56	8	0	8.173791	0.511787	0.097387

Structure 29 (B3LYP, Gas Phase)

Energy (Hartrees): = - 1054.4333023
No imaginary frequencies

Standard orientation:

Center Number	Atomic Number	Atomic Type	Coordinates (Angstroms) X	Y	Z
1	6	0	1.906587	-1.156821	1.120400
2	6	0	1.130966	0.066319	0.563049
3	6	0	1.866499	0.611463	-0.675025
4	6	0	3.331244	0.867248	-0.345644
5	6	0	3.983300	-0.417412	0.177536
6	1	0	1.819225	-0.137045	-1.476587
7	1	0	3.383219	1.631350	0.447298
8	1	0	3.951637	-1.187058	-0.602415
9	1	0	1.123413	0.856297	1.330581
10	8	0	3.258508	-0.851531	1.337584
11	6	0	5.437461	-0.213350	0.610865
12	1	0	5.481690	0.613800	1.339979
13	1	0	5.774468	-1.118974	1.124020
14	8	0	6.298965	-0.004211	-0.490858
15	1	0	5.891115	0.685598	-1.037499
16	8	0	4.033084	1.309751	-1.504713
17	1	0	3.509138	2.034690	-1.876310
18	8	0	1.320837	1.853237	-1.121312
19	1	0	0.481999	1.664905	-1.562004
20	7	0	-0.205396	-0.355937	0.181670
21	6	0	-2.611013	0.143393	0.244125
22	6	0	-3.094504	-1.050105	-0.359996
23	6	0	-3.533282	1.168840	0.590845
24	6	0	-4.464674	-1.177664	-0.597720
25	6	0	-4.890814	0.989796	0.323357
26	6	0	-5.381008	-0.174384	-0.272717
27	1	0	-4.827658	-2.096413	-1.052177
28	1	0	-5.586634	1.781312	0.590504
29	6	0	-1.193049	0.378588	0.547474
30	1	0	-0.990286	1.278567	1.140137
31	1	0	1.520542	-1.417568	2.116019
32	8	0	1.791196	-2.248367	0.235879
33	1	0	0.904289	-2.176421	-0.153946
34	6	0	-3.105079	2.469638	1.242008
35	6	0	-6.849487	-0.336118	-0.578108
36	6	0	-2.202104	-2.203569	-0.749176
37	1	0	-2.382156	3.023622	0.633561
38	1	0	-3.971670	3.118310	1.389136
39	1	0	-2.647537	2.311287	2.225058
40	1	0	-7.173484	-1.372851	-0.446283
41	1	0	-7.465095	0.299108	0.064815
42	1	0	-7.066709	-0.058850	-1.617196

43	1	0	-1.613921	-2.562425	0.100636
44	1	0	-2.803599	-3.032928	-1.130692
45	1	0	-1.481205	-1.912964	-1.518595

Structure 29 (B3LYP, DMSO)

Energy (Hartrees): = - 1054.4567198
No imaginary frequencies

Standard orientation:

Center Number	Atomic Number	Atomic Type	Coordinates (Angstroms) X	Y	Z
1	6	0	1.891306	-1.122846	1.153593
2	6	0	1.132423	0.087089	0.555762
3	6	0	1.872951	0.602500	-0.693600
4	6	0	3.340416	0.846328	-0.368623
5	6	0	3.980478	-0.428573	0.191150
6	1	0	1.812848	-0.156062	-1.484862
7	1	0	3.406238	1.635619	0.396310
8	1	0	3.949493	-1.215375	-0.572679
9	1	0	1.120467	0.893212	1.303952
10	8	0	3.254901	-0.839513	1.358857
11	6	0	5.431168	-0.218320	0.631272
12	1	0	5.466380	0.604003	1.364709
13	1	0	5.780391	-1.124278	1.137462
14	8	0	6.299480	0.015515	-0.468011
15	1	0	5.860808	0.687547	-1.016229
16	8	0	4.056759	1.238940	-1.542804
17	1	0	3.566319	1.984358	-1.923457
18	8	0	1.343478	1.843849	-1.157149
19	1	0	0.508027	1.657059	-1.610294
20	7	0	-0.202319	-0.350122	0.179282
21	6	0	-2.608444	0.151974	0.225005
22	6	0	-3.092991	-1.037374	-0.389974
23	6	0	-3.532905	1.169034	0.595647
24	6	0	-4.466313	-1.171695	-0.611099
25	6	0	-4.894423	0.983177	0.345670
26	6	0	-5.385095	-0.178369	-0.257140
27	1	0	-4.830948	-2.084723	-1.075901
28	1	0	-5.591283	1.766957	0.631800
29	6	0	-1.190336	0.397481	0.520132
30	1	0	-0.989849	1.313037	1.085383
31	1	0	1.503863	-1.336998	2.159161
32	8	0	1.754063	-2.254378	0.318241
33	1	0	0.882850	-2.152264	-0.104921
34	6	0	-3.104195	2.465050	1.252494
35	6	0	-6.855050	-0.348711	-0.542187
36	6	0	-2.200031	-2.177301	-0.814805
37	1	0	-2.400947	3.031268	0.631940
38	1	0	-3.974965	3.101885	1.427293
39	1	0	-2.619573	2.298956	2.221258
40	1	0	-7.176054	-1.383175	-0.383472
41	1	0	-7.465137	0.303256	0.089654
42	1	0	-7.082282	-0.099307	-1.586757
43	1	0	-1.613677	-2.569042	0.022382
44	1	0	-2.802930	-2.991972	-1.226050
45	1	0	-1.477025	-1.865058	-1.574502

Structure 29 (M06-2X, Gas Phase)

Energy (Hartrees): = - 1054.2655905
No imaginary frequencies

Standard orientation:

Center Number	Atomic Number	Atomic Type	Coordinates (Angstroms) X	Y	Z
1	6	0	1.904364	-1.260749	1.002759
2	6	0	1.128378	0.005705	0.591905
3	6	0	1.834874	0.666462	-0.592640
4	6	0	3.289772	0.903508	-0.245312
5	6	0	3.944771	-0.423499	0.125406
6	1	0	1.792113	0.001207	-1.462763
7	1	0	3.340492	1.576103	0.624014
8	1	0	3.895885	-1.105149	-0.730260
9	1	0	1.130774	0.714126	1.432158
10	8	0	3.248291	-0.972452	1.239920
11	6	0	5.400539	-0.261999	0.539756
12	1	0	5.471416	0.521599	1.307779
13	1	0	5.733252	-1.200836	0.984566
14	8	0	6.232835	0.004116	-0.564254

15	1	0	5.839948	0.740615	-1.045417
16	8	0	3.979497	1.467157	-1.345137
17	1	0	3.454502	2.215416	-1.648891
18	8	0	1.267228	1.929267	-0.904251
19	1	0	0.437232	1.781482	-1.365531
20	7	0	-0.208425	-0.374759	0.188570
21	6	0	-2.592592	0.133797	0.262212
22	6	0	-3.075664	-1.073920	-0.289861
23	6	0	-3.489425	1.188107	0.531560
24	6	0	-4.436511	-1.188694	-0.552649
25	6	0	-4.840210	1.027036	0.240513
26	6	0	-5.334737	-0.155160	-0.298826
27	1	0	-4.809650	-2.119242	-0.970096
28	1	0	-5.523692	1.845831	0.441525
29	6	0	-1.174488	0.346972	0.594667
30	1	0	-0.963382	1.205842	1.241517
31	1	0	1.521889	-1.642501	1.954623
32	8	0	1.800694	-2.233658	-0.002320
33	1	0	0.905800	-2.162667	-0.357556
34	6	0	-3.033075	2.505195	1.118193
35	6	0	-6.802067	-0.331761	-0.584713
36	6	0	-2.191143	-2.254379	-0.598554
37	1	0	-2.249006	2.972231	0.517983
38	1	0	-3.871041	3.199516	1.169527
39	1	0	-2.643635	2.382703	2.132182
40	1	0	-7.251382	-1.021288	0.134982
41	1	0	-7.335493	0.616990	-0.521672
42	1	0	-6.958014	-0.749580	-1.581366
43	1	0	-1.585233	-2.534712	0.265060
44	1	0	-2.802642	-3.107542	-0.893260
45	1	0	-1.496244	-2.021995	-1.407244

Structure 29 (M06-2X, DMSO)

Energy (Hartrees): = - 1054.2922603
No imaginary frequencies

Standard orientation:

Center Number	Atomic Number	Atomic Type	Coordinates (Angstroms) X	Y	Z
1	6	0	1.865692	-1.361465	0.825180
2	6	0	1.128246	-0.027312	0.612356
3	6	0	1.837482	0.794798	-0.466623
4	6	0	3.306315	0.920043	-0.121031
5	6	0	3.917190	-0.469803	0.024850
6	1	0	1.749086	0.287729	-1.434856
7	1	0	3.405251	1.450034	0.836908
8	1	0	3.822814	-1.008084	-0.925062
9	1	0	1.142946	0.542999	1.549933
10	8	0	3.230744	-1.166717	1.059734
11	6	0	5.384940	-0.421615	0.422664
12	1	0	5.497732	0.229110	1.300866
13	1	0	5.702238	-1.428037	0.702542
14	8	0	6.207400	0.000544	-0.647378
15	1	0	5.807198	0.805487	-0.997121
16	8	0	4.005051	1.615822	-1.142228
17	1	0	3.520575	2.433659	-1.307952
18	8	0	1.316014	2.111187	-0.549188
19	1	0	0.471914	2.075478	-1.013062
20	7	0	-0.215231	-0.327282	0.161700
21	6	0	-2.590790	0.117839	0.308404
22	6	0	-3.080237	-1.122809	-0.149525
23	6	0	-3.466702	1.214327	0.451573
24	6	0	-4.437473	-1.231917	-0.453461
25	6	0	-4.808863	1.061765	0.119982
26	6	0	-5.313858	-0.156265	-0.334681
27	1	0	-4.820320	-2.190759	-0.790678
28	1	0	-5.478045	1.911282	0.219563
29	6	0	-1.177689	0.318884	0.682971
30	1	0	-0.981267	1.074890	1.450762
31	1	0	1.489110	-1.850362	1.728884
32	8	0	1.699167	-2.195841	-0.292891
33	1	0	0.818742	-1.999959	-0.643512
34	6	0	-2.975147	2.556638	0.936835
35	6	0	-6.764278	-0.294274	-0.709730
36	6	0	-2.217493	-2.349602	-0.304823
37	1	0	-2.118538	2.906872	0.355287
38	1	0	-3.768554	3.300118	0.856096
39	1	0	-2.660758	2.512687	1.983816
40	1	0	-7.098048	-1.329647	-0.624485
41	1	0	-7.396040	0.331215	-0.076083
42	1	0	-6.922884	0.021646	-1.745420
43	1	0	-1.537206	-2.478541	0.539030
44	1	0	-2.849622	-3.235219	-0.386758

45	1	0	-1.600714	-2.286973	-1.204641

Structure 29 (M06-2X/def2-TZVP, Gas Phase)

Energy (Hartrees): = -1054.392822
No imaginary frequencies

Standard orientation:

Center Number	Atomic Number	Atomic Type	Coordinates (Angstroms) X	Y	Z
1	6	0	1.877829	-1.272827	0.947711
2	6	0	1.124870	0.011877	0.559347
3	6	0	1.851138	0.695314	-0.596483
4	6	0	3.304050	0.904273	-0.229915
5	6	0	3.939020	-0.438875	0.110502
6	1	0	1.806828	0.057875	-1.486112
7	1	0	3.354525	1.549710	0.658214
8	1	0	3.897584	-1.093418	-0.766090
9	1	0	1.130869	0.694410	1.419514
10	8	0	3.223421	-1.013579	1.195689
11	6	0	5.386370	-0.305649	0.558234
12	1	0	5.449321	0.450518	1.351953
13	1	0	5.702824	-1.260137	0.979041
14	8	0	6.250524	-0.010765	-0.512308
15	1	0	5.881087	0.739756	-0.992747
16	8	0	4.012095	1.492616	-1.303198
17	1	0	3.501737	2.253965	-1.603138
18	8	0	1.299919	1.970446	-0.877915
19	1	0	0.448731	1.850281	-1.311087
20	7	0	-0.212349	-0.333746	0.138044
21	6	0	-2.597610	0.127738	0.274805
22	6	0	-3.082964	-1.081116	-0.263515
23	6	0	-3.486378	1.188026	0.527955
24	6	0	-4.440360	-1.190342	-0.532445
25	6	0	-4.833488	1.033604	0.228525
26	6	0	-5.331486	-0.149345	-0.299225
27	1	0	-4.816669	-2.123588	-0.937804
28	1	0	-5.511815	1.858809	0.414268
29	6	0	-1.181562	0.334021	0.612687
30	1	0	-0.976774	1.138195	1.327343
31	1	0	1.484553	-1.662258	1.891590
32	8	0	1.760350	-2.233797	-0.066373
33	1	0	0.884936	-2.123231	-0.461945
34	6	0	-3.021822	2.504148	1.101007
35	6	0	-6.794977	-0.318561	-0.592918
36	6	0	-2.204589	-2.268795	-0.545698
37	1	0	-2.220870	2.949701	0.508901
38	1	0	-3.848820	3.210903	1.129734
39	1	0	-2.649040	2.390829	2.121108
40	1	0	-7.253465	-0.999018	0.127764
41	1	0	-7.322441	0.632712	-0.540701
42	1	0	-6.947496	-0.744247	-1.585377
43	1	0	-1.572329	-2.510345	0.309267
44	1	0	-2.818931	-3.135022	-0.786459
45	1	0	-1.536614	-2.070490	-1.384167

Structure 29 (M06-2X/def2-TZVP, DMSO)

Energy (Hartrees): = -1054.419399
No imaginary frequencies

Standard orientation:

Center Number	Atomic Number	Atomic Type	Coordinates (Angstroms) X	Y	Z
1	6	0	1.845669	-1.353982	0.799938
2	6	0	1.125244	-0.013651	0.583240
3	6	0	1.850483	0.805314	-0.483387
4	6	0	3.316592	0.915543	-0.127335
5	6	0	3.914221	-0.478497	0.018814
6	1	0	1.762369	0.305895	-1.454893
7	1	0	3.413000	1.439493	0.832928
8	1	0	3.831782	-1.009348	-0.935942
9	1	0	1.143107	0.548881	1.524495
10	8	0	3.209869	-1.177209	1.036695
11	6	0	5.372464	-0.446456	0.447213
12	1	0	5.470937	0.185898	1.338668
13	1	0	5.678761	-1.458643	0.715057
14	8	0	6.226866	-0.010291	-0.590327
15	1	0	5.846535	0.801588	-0.950103

16	8	0	4.030946	1.610900	-1.136404
17	1	0	3.562679	2.437996	-1.309514
18	8	0	1.339521	2.124225	-0.563869
19	1	0	0.465006	2.094858	-0.971953
20	7	0	-0.218787	-0.292804	0.129870
21	6	0	-2.594647	0.116091	0.313355
22	6	0	-3.084090	-1.121855	-0.140194
23	6	0	-3.467146	1.211183	0.456816
24	6	0	-4.438357	-1.229214	-0.446006
25	6	0	-4.805796	1.060905	0.122504
26	6	0	-5.311140	-0.154010	-0.332119
27	1	0	-4.821800	-2.187697	-0.779760
28	1	0	-5.473213	1.910537	0.221227
29	6	0	-1.183023	0.314918	0.685684
30	1	0	-0.988394	1.036629	1.485375
31	1	0	1.459945	-1.833113	1.704590
32	8	0	1.670433	-2.197193	-0.309665
33	1	0	0.798877	-1.990286	-0.679953
34	6	0	-2.973623	2.548313	0.943155
35	6	0	-6.757932	-0.288770	-0.709718
36	6	0	-2.224414	-2.348015	-0.285274
37	1	0	-2.124536	2.902437	0.354796
38	1	0	-3.767383	3.291070	0.875930
39	1	0	-2.646439	2.498831	1.984676
40	1	0	-7.090147	-1.324595	-0.641084
41	1	0	-7.391054	0.326513	-0.069459
42	1	0	-6.915971	0.042445	-1.739553
43	1	0	-1.533338	-2.461346	0.550554
44	1	0	-2.855084	-3.235223	-0.341907
45	1	0	-1.622245	-2.303548	-1.194821

Structure 30 (B3LYP, Gas Phase)

Energy (Hartrees): = - 1054.4318566
No imaginary frequencies

Standard orientation:

Center Number	Atomic Number	Atomic Type	Coordinates (Angstroms) X	Y	Z
1	6	0	-1.782565	0.980481	-1.043538
2	6	0	-1.134563	0.278368	0.164479
3	6	0	-2.002802	-0.928458	0.548531
4	6	0	-3.454049	-0.517858	0.752497
5	6	0	-3.974729	0.196632	-0.501516
6	1	0	-1.766695	0.304701	-1.918710
7	1	0	-1.964112	-1.658255	-0.275460
8	1	0	-3.504269	0.188460	1.596331
9	1	0	-3.949212	-0.505888	-1.350623
10	1	0	-1.141794	1.008974	0.990023
11	8	0	-3.134460	1.323986	-0.753548
12	6	0	-5.409732	0.708636	-0.346648
13	1	0	-5.468269	1.337597	0.557477
14	1	0	-5.645372	1.346713	-1.204053
15	8	0	-6.351301	-0.345359	-0.331684
16	1	0	-6.037207	-0.990947	0.321275
17	8	0	-4.269497	-1.658519	1.011316
18	1	0	-3.824065	-2.157663	1.711798
19	8	0	-1.572494	-1.538975	1.761026
20	1	0	-0.668847	-1.851908	1.614240
21	8	0	-1.083823	2.157713	-1.286897
22	1	0	-1.526295	2.610940	-2.017983
23	7	0	0.200098	-0.189537	-0.160252
24	6	0	2.608271	0.148276	0.135477
25	6	0	3.062921	-0.817157	-0.804040
26	6	0	3.561662	0.880283	0.895319
27	6	0	4.437965	-1.021246	-0.948233
28	6	0	4.921949	0.632598	0.712353
29	6	0	5.385057	-0.316982	-0.202316
30	1	0	4.778513	-1.756326	-1.673694
31	1	0	5.641513	1.199124	1.298777
32	6	0	1.186327	0.447048	0.356350
33	1	0	0.987006	1.294200	1.023565
34	6	0	3.162674	1.933712	1.910161
35	6	0	2.134081	-1.630376	-1.671661
36	6	0	6.860873	-0.588123	-0.360938
37	1	0	2.530981	1.526348	2.706973
38	1	0	4.052774	2.355166	2.383410
39	1	0	2.612592	2.763438	1.452541
40	1	0	7.093228	-0.968014	-1.359921
41	1	0	7.454549	0.315289	-0.192230
42	1	0	7.202412	-1.340191	0.361458
43	1	0	1.459070	-0.992037	-2.247789
44	1	0	2.711849	-2.253391	-2.360383
45	1	0	1.483521	-2.275347	-1.074616

Structure 30 (B3LYP, DMSO)

Energy (Hartrees): = - 1054.4553556
No imaginary frequencies

Standard orientation:

Center Number	Atomic Number	Atomic Type	Coordinates (Angstroms) X	Y	Z
1	6	0	-1.896727	1.425267	0.537673
2	6	0	-1.141527	0.095180	0.384331
3	6	0	-1.896678	-0.784796	-0.630736
4	6	0	-3.365728	-0.917009	-0.249532
5	6	0	-3.997570	0.469585	-0.070546
6	1	0	-1.891566	1.970410	-0.421677
7	1	0	-1.838022	-0.304872	-1.618552
8	1	0	-3.434875	-1.454384	0.708541
9	1	0	-3.969615	1.003429	-1.033890
10	1	0	-1.154385	-0.406419	1.365270
11	8	0	-3.253119	1.181360	0.918655
12	6	0	-5.448641	0.401986	0.410864
13	1	0	-5.488415	-0.182070	1.344988
14	1	0	-5.791068	1.415941	0.642320
15	8	0	-6.319694	-0.124304	-0.579804
16	1	0	-5.888735	-0.928693	-0.914661
17	8	0	-4.086690	-1.622563	-1.263439
18	1	0	-3.607814	-2.452980	-1.412154
19	8	0	-1.372142	-2.109144	-0.692465
20	1	0	-0.503219	-2.058907	-1.118156
21	8	0	-1.292937	2.163836	1.549255
22	1	0	-1.714597	3.036965	1.563755
23	7	0	0.204069	0.331657	-0.106305
24	6	0	2.596413	-0.070116	0.212017
25	6	0	3.109586	0.952207	-0.629938
26	6	0	3.490353	-1.018389	0.782014
27	6	0	4.486155	0.993159	-0.879263
28	6	0	4.852912	-0.939227	0.489884
29	6	0	5.374827	0.058727	-0.340752
30	1	0	4.874498	1.785662	-1.514696
31	1	0	5.525900	-1.674623	0.924501
32	6	0	1.169505	-0.188021	0.558407
33	1	0	0.955102	-0.776096	1.458964
34	6	0	3.013154	-2.133438	1.688264
35	6	0	2.248303	2.020285	-1.259738
36	6	0	6.847476	0.108743	-0.660414
37	1	0	2.248542	-2.755403	1.210434
38	1	0	3.848710	-2.784276	1.958542
39	1	0	2.580034	-1.751101	2.620101
40	1	0	7.150761	1.104153	-0.997805
41	1	0	7.456031	-0.160969	0.208806
42	1	0	7.099102	-0.599201	-1.460630
43	1	0	1.573004	2.482577	-0.534104
44	1	0	2.878814	2.798656	-1.699473
45	1	0	1.609112	1.612272	-2.049108

Structure 30 (M06-2X, Gas Phase)

Energy (Hartrees): = - 1054.2628696
No imaginary frequencies

Standard orientation:

Center Number	Atomic Number	Atomic Type	Coordinates (Angstroms) X	Y	Z
1	6	0	-1.707172	0.941161	-0.982408
2	6	0	-1.134349	0.229695	0.245543
3	6	0	-2.036327	-0.946847	0.591672
4	6	0	-3.468443	-0.487337	0.751870
5	6	0	-3.916311	0.222873	-0.522442
6	1	0	-1.674685	0.266261	-1.854247
7	1	0	-1.994786	-1.673920	-0.232342
8	1	0	-3.520023	0.227914	1.585288
9	1	0	-3.871614	-0.488073	-1.361647
10	1	0	-1.153424	0.962657	1.065279
11	8	0	-3.048305	1.322838	-0.748684
12	6	0	-5.337875	0.760528	-0.427352
13	1	0	-5.431971	1.353434	0.493351
14	1	0	-5.512092	1.425011	-1.275103
15	8	0	-6.291177	-0.272329	-0.493723
16	1	0	-6.040211	-0.933392	0.161335
17	8	0	-4.326245	-1.588208	0.991425
18	1	0	-3.920716	-2.110957	1.691795

19	8	0	-1.658504	-1.567227	1.805124
20	1	0	-0.751986	-1.873440	1.700234
21	8	0	-0.960640	2.087605	-1.192047
22	1	0	-1.333694	2.548453	-1.948921
23	7	0	0.198233	-0.272295	-0.022023
24	6	0	2.592639	0.155725	0.120350
25	6	0	3.028250	-0.877505	-0.736696
26	6	0	3.544375	0.947294	0.799729
27	6	0	4.396819	-1.092796	-0.882647
28	6	0	4.898253	0.688513	0.625054
29	6	0	5.345960	-0.330259	-0.211124
30	1	0	4.730406	-1.882754	-1.548479
31	1	0	5.624377	1.298976	1.152994
32	6	0	1.169373	0.468580	0.333444
33	1	0	0.964707	1.419607	0.835656
34	6	0	3.143322	2.075622	1.723347
35	6	0	2.085884	-1.750454	-1.523104
36	6	0	6.817199	-0.610968	-0.363237
37	1	0	2.495044	1.729755	2.531225
38	1	0	4.030359	2.518806	2.175042
39	1	0	2.612806	2.867152	1.188162
40	1	0	7.028184	-1.108465	-1.310617
41	1	0	7.400109	0.310483	-0.319348
42	1	0	7.166851	-1.263381	0.441891
43	1	0	1.391380	-1.149667	-2.112408
44	1	0	2.654557	-2.400805	-2.188865
45	1	0	1.468178	-2.361984	-0.864886

Structure 30 (M06-2X, DMSO)

Energy (Hartrees): = - 1054.2902771
No imaginary frequencies

Standard orientation:

Center Number	Atomic Number	Atomic Type	Coordinates (Angstroms) X	Y	Z
1	6	0	-1.904943	1.474823	0.405293
2	6	0	-1.140049	0.155172	0.399307
3	6	0	-1.849093	-0.821233	-0.541746
4	6	0	-3.310062	-0.944642	-0.160049
5	6	0	-3.957644	0.437831	-0.139451
6	1	0	-1.887993	1.920814	-0.601414
7	1	0	-1.788277	-0.433854	-1.567378
8	1	0	-3.380169	-1.377320	0.847624
9	1	0	-3.909121	0.870959	-1.149788
10	1	0	-1.164986	-0.250650	1.421123
11	8	0	-3.253798	1.252364	0.784242
12	6	0	-5.411459	0.392366	0.308078
13	1	0	-5.472850	-0.148759	1.262281
14	1	0	-5.757471	1.414425	0.474018
15	8	0	-6.248210	-0.185028	-0.674363
16	1	0	-5.828357	-1.012328	-0.938946
17	8	0	-4.004777	-1.758162	-1.093758
18	1	0	-3.510804	-2.583549	-1.170561
19	8	0	-1.293771	-2.122940	-0.472072
20	1	0	-0.429652	-2.103952	-0.899050
21	8	0	-1.324745	2.309029	1.344381
22	1	0	-1.737898	3.176818	1.263951
23	7	0	0.207443	0.363401	-0.084495
24	6	0	2.577208	-0.047244	0.245400
25	6	0	3.109864	1.039195	-0.477437
26	6	0	3.422219	-1.091354	0.674995
27	6	0	4.476675	1.049132	-0.756179
28	6	0	4.777148	-1.045473	0.360812
29	6	0	5.323453	0.017846	-0.356127
30	1	0	4.892866	1.893161	-1.298654
31	1	0	5.422024	-1.857349	0.684114
32	6	0	1.148213	-0.135925	0.608685
33	1	0	0.923279	-0.679676	1.533367
34	6	0	2.886761	-2.267558	1.454708
35	6	0	2.276086	2.206239	-0.940824
36	6	0	6.786041	0.039341	-0.708939
37	1	0	2.046359	-2.744232	0.943542
38	1	0	3.668410	-3.014973	1.593187
39	1	0	2.534758	-1.963560	2.444836
40	1	0	7.156869	1.062391	-0.793187
41	1	0	7.379925	-0.488008	0.039678
42	1	0	6.954024	-0.452909	-1.671893
43	1	0	1.592713	2.548122	-0.161497
44	1	0	2.925844	3.033227	-1.231371
45	1	0	1.658306	1.934186	-1.799121

Structure 30 (M06-2X/def2-TZVP, Gas Phase)

Energy (Hartrees): = -1054.391478
No imaginary frequencies

Standard orientation:

Center Number	Atomic Number	Atomic Type	Coordinates (Angstroms) X	Y	Z
1	6	0	-1.811131	1.340626	0.528212
2	6	0	-1.129339	-0.011895	0.338587
3	6	0	-1.933051	-0.817817	-0.674268
4	6	0	-3.378590	-0.910687	-0.238729
5	6	0	-3.943882	0.492711	-0.043136
6	1	0	-1.786703	1.905162	-0.417714
7	1	0	-1.893596	-0.305806	-1.644911
8	1	0	-3.423990	-1.437962	0.723783
9	1	0	-3.917114	1.024932	-1.005315
10	1	0	-1.154100	-0.523298	1.311723
11	8	0	-3.154445	1.164268	0.921894
12	6	0	-5.378258	0.482782	0.463880
13	1	0	-5.439191	-0.167724	1.345852
14	1	0	-5.640071	1.494394	0.775179
15	8	0	-6.291641	0.101011	-0.534656
16	1	0	-5.976008	-0.723006	-0.925733
17	8	0	-4.154931	-1.588596	-1.207333
18	1	0	-3.688452	-2.401559	-1.435102
19	8	0	-1.448564	-2.139669	-0.802983
20	1	0	-0.551304	-2.099537	-1.151984
21	8	0	-1.147740	2.014342	1.538686
22	1	0	-1.611414	2.840886	1.709788
23	7	0	0.210097	0.167380	-0.163066
24	6	0	2.590803	-0.073737	0.223807
25	6	0	3.063798	0.953667	-0.609471
26	6	0	3.490843	-1.003050	0.776445
27	6	0	4.427783	1.017295	-0.875342
28	6	0	4.839660	-0.907133	0.470429
29	6	0	5.328715	0.097586	-0.356381
30	1	0	4.796667	1.818556	-1.506431
31	1	0	5.529322	-1.631724	0.889992
32	6	0	1.167844	-0.202328	0.579419
33	1	0	0.957829	-0.654090	1.555750
34	6	0	3.022154	-2.113877	1.682151
35	6	0	2.166158	2.005601	-1.200828
36	6	0	6.791594	0.170990	-0.691783
37	1	0	2.225984	-2.702383	1.223607
38	1	0	3.847099	-2.785529	1.912890
39	1	0	2.636694	-1.725238	2.627399
40	1	0	7.075152	1.175400	-1.003944
41	1	0	7.405816	-0.112032	0.163166
42	1	0	7.031463	-0.512114	-1.509847
43	1	0	1.475149	2.402765	-0.456794
44	1	0	2.766917	2.820475	-1.602994
45	1	0	1.551139	1.592513	-1.999721

Structure 30 (M06-2X/def2-TZVP, DMSO)

Energy (Hartrees): = -1054.418208
No imaginary frequencies

Standard orientation:

Center Number	Atomic Number	Atomic Type	Coordinates (Angstroms) X	Y	Z
1	6	0	-1.901243	1.449785	0.445831
2	6	0	-1.139306	0.129875	0.401696
3	6	0	-1.853672	-0.813907	-0.565477
4	6	0	-3.315247	-0.943947	-0.195048
5	6	0	-3.957544	0.438089	-0.130988
6	1	0	-1.877837	1.928615	-0.544069
7	1	0	-1.786794	-0.395004	-1.577445
8	1	0	-3.392742	-1.407739	0.796923
9	1	0	-3.911200	0.903010	-1.126220
10	1	0	-1.161954	-0.304884	1.410877
11	8	0	-3.249401	1.221327	0.812322
12	6	0	-5.406260	0.386985	0.327185
13	1	0	-5.463872	-0.179212	1.265311
14	1	0	-5.746687	1.404410	0.524350
15	8	0	-6.260781	-0.158468	-0.657111
16	1	0	-5.856748	-0.982093	-0.960013
17	8	0	-4.010527	-1.724901	-1.154385
18	1	0	-3.525310	-2.552910	-1.264952

19	8	0	-1.299724	-2.115737	-0.536958
20	1	0	-0.405785	-2.076774	-0.900319
21	8	0	-1.326018	2.256595	1.411641
22	1	0	-1.716931	3.137392	1.347675
23	7	0	0.205831	0.340935	-0.080836
24	6	0	2.581372	-0.030482	0.253619
25	6	0	3.106489	1.020497	-0.520291
26	6	0	3.431364	-1.048387	0.725843
27	6	0	4.468104	1.019478	-0.809018
28	6	0	4.781465	-1.013728	0.400687
29	6	0	5.319249	0.012159	-0.368878
30	1	0	4.877225	1.837775	-1.392494
31	1	0	5.430245	-1.805858	0.759206
32	6	0	1.156594	-0.115164	0.623860
33	1	0	0.940235	-0.626472	1.567551
34	6	0	2.909325	-2.189484	1.559136
35	6	0	2.273121	2.164925	-1.027371
36	6	0	6.775595	0.021203	-0.734001
37	1	0	2.080716	-2.702945	1.067049
38	1	0	3.700114	-2.916567	1.738369
39	1	0	2.545432	-1.844338	2.529798
40	1	0	7.154608	1.039950	-0.820394
41	1	0	7.371271	-0.513141	0.006290
42	1	0	6.930242	-0.468481	-1.699173
43	1	0	1.596428	2.542070	-0.260276
44	1	0	2.921811	2.976943	-1.355738
45	1	0	1.651880	1.861810	-1.871229

Structure 32 (B3LYP, Gas Phase)

Energy (Hartrees): = - 1011.7102403
No imaginary frequencies

Standard orientation:

Center Number	Atomic Number	Atomic Type	Coordinates (Angstroms) X	Y	Z
1	6	0	-1.245094	-1.434575	-0.576607
2	6	0	-0.570068	-0.033909	-0.576210
3	6	0	-1.373034	0.917761	0.324050
4	6	0	-2.840760	0.908075	-0.087437
5	6	0	-3.395486	-0.520116	-0.054116
6	1	0	-1.301356	0.570640	1.363410
7	1	0	-2.911564	1.283205	-1.121827
8	1	0	-3.357266	-0.905895	0.970860
9	1	0	-0.603873	0.352479	-1.606161
10	8	0	-2.595030	-1.336476	-0.925983
11	6	0	-4.837583	-0.610319	-0.560663
12	1	0	-4.895418	-0.151635	-1.562548
13	1	0	-5.099338	-1.667029	-0.669481
14	8	0	-5.756733	-0.036845	0.347129
15	1	0	-5.415679	0.842318	0.574728
16	8	0	-3.610940	1.725918	0.788199
17	1	0	-3.155890	2.579890	0.831802
18	8	0	-0.930568	2.269735	0.214200
19	1	0	-0.082261	2.344438	0.670497
20	7	0	0.798481	-0.136928	-0.098542
21	6	0	3.162910	0.129989	-0.490577
22	6	0	3.540680	-0.272823	0.820117
23	6	0	4.168702	0.473196	-1.415958
24	6	0	4.899918	-0.325594	1.161488
25	6	0	5.509814	0.417864	-1.072086
26	1	0	3.872109	0.782636	-2.415279
27	6	0	5.866617	0.014482	0.224099
28	1	0	5.165970	-0.636007	2.166106
29	1	0	6.274328	0.682414	-1.794591
30	1	0	6.915279	-0.032451	0.503220
31	6	0	1.767761	0.195847	-0.883365
32	1	0	1.562803	0.551539	-1.902843
33	1	0	-0.790805	-2.056612	-1.361264
34	8	0	-1.136370	-2.056654	0.685601
35	1	0	-0.213923	-1.973419	0.967869
36	8	0	2.627966	-0.600093	1.749572
37	1	0	1.734670	-0.491958	1.329144

Structure 32 (B3LYP, DMSO)

Energy (Hartrees): = - 1011.7360349
No imaginary frequencies

Standard orientation:

Center	Atomic	Atomic	Coordinates (Angstroms)

Number	Number	Type	X	Y	Z
1	6	0	-1.240992	-1.507488	-0.387483
2	6	0	-0.572163	-0.120554	-0.574401
3	6	0	-1.368300	0.950851	0.188148
4	6	0	-2.838163	0.883349	-0.206219
5	6	0	-3.391014	-0.527502	0.023862
6	1	0	-1.285847	0.758314	1.266148
7	1	0	-2.925190	1.123165	-1.277411
8	1	0	-3.345773	-0.764835	1.093452
9	1	0	-0.603517	0.115494	-1.647240
10	8	0	-2.602262	-1.464902	-0.727093
11	6	0	-4.834179	-0.684799	-0.458722
12	1	0	-4.892168	-0.393976	-1.520482
13	1	0	-5.114762	-1.741097	-0.392273
14	8	0	-5.749566	0.049230	0.341188
15	1	0	-5.369238	0.938827	0.434566
16	8	0	-3.611197	1.804263	0.567857
17	1	0	-3.186819	2.670498	0.464577
18	8	0	-0.925475	2.272123	-0.116635
19	1	0	-0.074249	2.406725	0.325845
20	7	0	0.796819	-0.157419	-0.081631
21	6	0	3.160602	0.019844	-0.506897
22	6	0	3.530824	-0.081713	0.862062
23	6	0	4.170771	0.148933	-1.480012
24	6	0	4.887920	-0.055439	1.215114
25	6	0	5.512138	0.168843	-1.124700
26	1	0	3.876760	0.230701	-2.523342
27	6	0	5.862269	0.065440	0.230050
28	1	0	5.155294	-0.132315	2.264270
29	1	0	6.280876	0.264970	-1.884501
30	1	0	6.909259	0.081419	0.519603
31	6	0	1.765766	0.013164	-0.916706
32	1	0	1.569423	0.171060	-1.984052
33	1	0	-0.795010	-2.220285	-1.094454
34	8	0	-1.114700	-1.977814	0.940643
35	1	0	-0.213589	-1.762608	1.229324
36	8	0	2.608920	-0.194208	1.834692
37	1	0	1.718172	-0.191236	1.387589

Structure 32 (M06-2X, Gas Phase)

Energy (Hartrees): = - 1011.5800696
No imaginary frequencies

Standard orientation:

Center Number	Atomic Number	Atomic Type	Coordinates (Angstroms) X	Y	Z
1	6	0	-1.271686	-1.391364	-0.700116
2	6	0	-0.577256	-0.017407	-0.595407
3	6	0	-1.347054	0.861081	0.388669
4	6	0	-2.802974	0.923053	-0.028179
5	6	0	-3.379389	-0.486875	-0.100516
6	1	0	-1.290290	0.419897	1.390775
7	1	0	-2.861038	1.376697	-1.029198
8	1	0	-3.334061	-0.952507	0.889367
9	1	0	-0.609212	0.459781	-1.584664
10	8	0	-2.611627	-1.237437	-1.040046
11	6	0	-4.822079	-0.504560	-0.586227
12	1	0	-4.894258	0.079084	-1.515332
13	1	0	-5.090941	-1.536765	-0.814761
14	8	0	-5.711291	-0.040711	0.401014
15	1	0	-5.374234	0.805990	0.714014
16	8	0	-3.555512	1.680616	0.899425
17	1	0	-3.084573	2.510791	1.029264
18	8	0	-0.865026	2.193767	0.396416
19	1	0	-0.024302	2.213272	0.861269
20	7	0	0.784677	-0.184294	-0.132037
21	6	0	3.141109	0.189521	-0.454021
22	6	0	3.528275	-0.392932	0.771838
23	6	0	4.128001	0.677518	-1.321961
24	6	0	4.885291	-0.472505	1.093852
25	6	0	5.468431	0.593528	-1.000818
26	1	0	3.817405	1.124125	-2.261191
27	6	0	5.837788	0.012364	0.215815
28	1	0	5.158243	-0.923756	2.039293
29	1	0	6.221083	0.971128	-1.680204
30	1	0	6.886266	-0.060186	0.480165
31	6	0	1.739372	0.287878	-0.838585
32	1	0	1.531392	0.798333	-1.787548
33	1	0	-0.825046	-1.971418	-1.514449
34	8	0	-1.189340	-2.086890	0.516350
35	1	0	-0.265656	-2.091326	0.787603
36	8	0	2.637213	-0.869223	1.649877

37	1	0	1.744186	-0.729859	1.269517

Structure 32 (M06-2X, DMSO)

Energy (Hartrees): = - 1011.6085546
No imaginary frequencies

Standard orientation:

Center Number	Atomic Number	Atomic Type	Coordinates (Angstroms) X	Y	Z
1	6	0	1.264668	-1.439227	0.606937
2	6	0	0.578330	-0.061212	0.587045
3	6	0	1.344097	0.879949	-0.341280
4	6	0	2.798704	0.914143	0.080488
5	6	0	3.377243	-0.496491	0.068786
6	1	0	1.281804	0.509676	-1.371592
7	1	0	2.859496	1.310571	1.103980
8	1	0	3.343523	-0.894794	-0.951166
9	1	0	0.608977	0.345729	1.605688
10	8	0	2.612839	-1.318640	0.948261
11	6	0	4.814754	-0.536988	0.566798
12	1	0	4.875605	-0.019431	1.533974
13	1	0	5.100536	-1.579262	0.720940
14	8	0	5.712015	0.014457	-0.376554
15	1	0	5.346890	0.869854	-0.632464
16	8	0	3.561641	1.720337	-0.804391
17	1	0	3.111901	2.571825	-0.867034
18	8	0	0.861906	2.209658	-0.260813
19	1	0	0.013752	2.251080	-0.716868
20	7	0	-0.785364	-0.196972	0.113204
21	6	0	-3.138099	0.137590	0.475457
22	6	0	-3.524784	-0.295473	-0.810871
23	6	0	-4.124340	0.518860	1.394898
24	6	0	-4.881594	-0.339762	-1.141393
25	6	0	-5.467271	0.469080	1.065468
26	1	0	-3.810498	0.854226	2.378397
27	6	0	-5.837135	0.035508	-0.210701
28	1	0	-5.162033	-0.673336	-2.133481
29	1	0	-6.220940	0.763596	1.784833
30	1	0	-6.885890	-0.007172	-0.482840
31	6	0	-1.734139	0.203005	0.870328
32	1	0	-1.528925	0.620125	1.862270
33	1	0	0.816110	-2.059772	1.388757
34	8	0	1.178106	-2.074417	-0.644618
35	1	0	0.265545	-1.989913	-0.946311
36	8	0	-2.627309	-0.658639	-1.737961
37	1	0	-1.734503	-0.570139	-1.332673

Structure 33 (B3LYP, Gas Phase)

Energy (Hartrees): = - 1011.7119234
No imaginary frequencies

Standard orientation:

Center Number	Atomic Number	Atomic Type	Coordinates (Angstroms) X	Y	Z
1	6	0	1.213156	1.391435	0.385780
2	6	0	0.585296	0.228365	-0.406039
3	6	0	1.424491	-1.038288	-0.180011
4	6	0	2.896653	-0.776036	-0.471550
5	6	0	3.393459	0.417192	0.354930
6	1	0	1.145938	1.182848	1.469938
7	1	0	1.332919	-1.334836	0.876953
8	1	0	3.001129	-0.520841	-1.538158
9	1	0	3.311857	0.172518	1.426541
10	1	0	0.634466	0.510527	-1.468452
11	8	0	2.582090	1.548529	0.032229
12	6	0	4.850342	0.785478	0.058657
13	1	0	4.967250	0.934737	-1.027813
14	1	0	5.072247	1.740242	0.545251
15	8	0	5.754047	-0.171395	0.572374
16	1	0	5.450968	-1.038861	0.260597
17	8	0	3.682071	-1.921612	-0.155532
18	1	0	3.257934	-2.673434	-0.595097
19	8	0	1.027183	-2.111283	-1.026850
20	1	0	0.129372	-2.367049	-0.774573
21	8	0	0.538303	2.555686	0.039675
22	1	0	0.948196	3.286944	0.522231
23	7	0	-0.779516	-0.014995	0.022775
24	6	0	-3.143199	0.138750	-0.391799
25	6	0	-3.506046	-0.411468	0.868926

26	6	0	-4.159219	0.509237	-1.293987
27	6	0	-4.863854	-0.573728	1.184021
28	6	0	-5.498284	0.345531	-0.975799
29	1	0	-3.872103	0.931582	-2.254025
30	6	0	-5.841323	-0.199637	0.271117
31	1	0	-5.119823	-0.994057	2.150674
32	1	0	-6.270897	0.635434	-1.679884
33	1	0	-6.887823	-0.331795	0.530937
34	6	0	-1.749860	0.323372	-0.755006
35	1	0	-1.552726	0.774036	-1.736996
36	8	0	-2.584920	-0.782915	1.770597
37	1	0	-1.692170	-0.580325	1.377060

Structure 33 (B3LYP, DMSO)

Energy (Hartrees): = - 1011.7374076
No imaginary frequencies

Standard orientation:

Center Number	Atomic Number	Atomic Type	Coordinates (Angstroms) X	Y	Z
1	6	0	1.264609	1.483827	0.143568
2	6	0	0.588116	0.230992	-0.439253
3	6	0	1.368669	-1.016495	0.016472
4	6	0	2.851127	-0.873109	-0.308305
5	6	0	3.403263	0.429699	0.284553
6	1	0	1.200285	1.464878	1.245148
7	1	0	1.260912	-1.117140	1.106399
8	1	0	2.969198	-0.832720	-1.401923
9	1	0	3.319787	0.389076	1.382257
10	1	0	0.641384	0.306438	-1.534941
11	8	0	2.640720	1.519868	-0.236241
12	6	0	4.865928	0.680636	-0.090274
13	1	0	4.958577	0.673357	-1.188717
14	1	0	5.152522	1.678845	0.256858
15	8	0	5.746556	-0.250319	0.521254
16	1	0	5.356937	-1.127579	0.368558
17	8	0	3.591128	-1.972518	0.227616
18	1	0	3.166674	-2.777730	-0.107829
19	8	0	0.921773	-2.201900	-0.636426
20	1	0	0.054399	-2.430343	-0.270226
21	8	0	0.641999	2.606110	-0.388961
22	1	0	1.004043	3.383454	0.064031
23	7	0	-0.783638	0.136977	0.023723
24	6	0	-3.133889	-0.016185	-0.433399
25	6	0	-3.497117	-0.047893	0.941649
26	6	0	-4.145702	-0.077526	-1.410010
27	6	0	-4.852164	-0.138616	1.294801
28	6	0	-5.484607	-0.166699	-1.053315
29	1	0	-3.856389	-0.051982	-2.457694
30	6	0	-5.829148	-0.196368	0.306321
31	1	0	-5.115714	-0.161153	2.347584
32	1	0	-6.255129	-0.212064	-1.816140
33	1	0	-6.873759	-0.265419	0.597063
34	6	0	-1.739162	0.083147	-0.838099
35	1	0	-1.537899	0.114725	-1.915924
36	8	0	-2.571983	0.008937	1.914450
37	1	0	-1.681447	0.072621	1.457660

Structure 33 (M06-2X, Gas Phase)

Energy (Hartrees): = - 1011.581021
No imaginary frequencies
Standard orientation:

Center Number	Atomic Number	Atomic Type	Coordinates (Angstroms) X	Y	Z
1	6	0	1.162119	1.316758	0.457759
2	6	0	0.594533	0.197650	-0.418658
3	6	0	1.463047	-1.044594	-0.267950
4	6	0	2.915722	-0.707539	-0.531901
5	6	0	3.351962	0.428217	0.389292
6	1	0	1.088376	1.033590	1.521292
7	1	0	1.377709	-1.414692	0.764349
8	1	0	3.013773	-0.366025	-1.572510
9	1	0	3.256854	0.099347	1.435356
10	1	0	0.645577	0.557349	-1.455205
11	8	0	2.517375	1.548087	0.137264
12	6	0	4.795501	0.853493	0.155097
13	1	0	4.945327	1.032071	-0.918942
14	1	0	4.965532	1.794928	0.679642

15	8	0	5.703853	-0.089653	0.668780
16	1	0	5.458540	-0.948013	0.305739
17	8	0	3.741375	-1.832496	-0.299637
18	1	0	3.350594	-2.572186	-0.777660
19	8	0	1.104603	-2.061520	-1.182062
20	1	0	0.204738	-2.337499	-0.982586
21	8	0	0.443878	2.465963	0.179947
22	1	0	0.804919	3.180055	0.713085
23	7	0	-0.763526	-0.124101	-0.028261
24	6	0	-3.121846	0.221258	-0.321453
25	6	0	-3.498255	-0.635379	0.735578
26	6	0	-4.116767	0.867695	-1.067003
27	6	0	-4.855345	-0.820182	1.014880
28	6	0	-5.456621	0.681482	-0.787211
29	1	0	-3.812739	1.524436	-1.875739
30	6	0	-5.816104	-0.170079	0.261258
31	1	0	-5.121096	-1.479377	1.831521
32	1	0	-6.216209	1.186282	-1.369321
33	1	0	-6.863573	-0.325697	0.492114
34	6	0	-1.721082	0.444996	-0.653265
35	1	0	-1.518762	1.140855	-1.475727
36	8	0	-2.599912	-1.282210	1.485239
37	1	0	-1.705432	-1.024749	1.167630

Structure 33 (M06-2X, DMSO)

Energy (Hartrees): = - 1011.6091605
No imaginary frequencies

Standard orientation:

Center Number	Atomic Number	Atomic Type	Coordinates (Angstroms) X	Y	Z
1	6	0	1.266932	1.476238	0.096450
2	6	0	0.595365	0.220569	-0.457200
3	6	0	1.363397	-1.012300	0.021742
4	6	0	2.833012	-0.875081	-0.320468
5	6	0	3.377392	0.427590	0.259888
6	1	0	1.205101	1.478824	1.195866
7	1	0	1.264759	-1.090644	1.112819
8	1	0	2.941684	-0.842441	-1.413484
9	1	0	3.274371	0.403335	1.354897
10	1	0	0.640197	0.272900	-1.553268
11	8	0	2.631507	1.504698	-0.285350
12	6	0	4.840695	0.656650	-0.089337
13	1	0	4.971120	0.550964	-1.175060
14	1	0	5.109289	1.677980	0.187178
15	8	0	5.693698	-0.215205	0.625761
16	1	0	5.338856	-1.104212	0.504312
17	8	0	3.575420	-1.959024	0.216132
18	1	0	3.143955	-2.770431	-0.078102
19	8	0	0.905886	-2.197676	-0.602722
20	1	0	0.038989	-2.414092	-0.241111
21	8	0	0.638843	2.576900	-0.455933
22	1	0	0.986478	3.365117	-0.022649
23	7	0	-0.767068	0.140761	0.021687
24	6	0	-3.114997	-0.032970	-0.417069
25	6	0	-3.479299	-0.014265	0.947391
26	6	0	-4.115395	-0.129363	-1.392551
27	6	0	-4.830786	-0.090747	1.296031
28	6	0	-5.452455	-0.205561	-1.042406
29	1	0	-3.818290	-0.141640	-2.436680
30	6	0	-5.801022	-0.183944	0.310809
31	1	0	-5.095049	-0.073710	2.347001
32	1	0	-6.217231	-0.280503	-1.805294
33	1	0	-6.844485	-0.240881	0.600269
34	6	0	-1.715156	0.055058	-0.827837
35	1	0	-1.517794	0.049432	-1.905455
36	8	0	-2.567773	0.077486	1.923843
37	1	0	-1.679212	0.125984	1.490441

Structure 34 (B3LYP, Gas Phase)

Energy (Hartrees): = - 1086.9329401
No imaginary frequencies

Standard orientation:

Center Number	Atomic Number	Atomic Type	Coordinates (Angstroms) X	Y	Z
1	6	0	1.618493	-1.438737	0.565915
2	6	0	0.946681	-0.037264	0.588391
3	6	0	1.740795	0.922149	-0.310956

4	6	0	3.213567	0.905212	0.080131
5	6	0	3.764024	-0.524047	0.022351
6	1	0	1.654520	0.586615	-1.353193
7	1	0	3.299766	1.268021	1.117734
8	1	0	3.709682	-0.897554	-1.006466
9	1	0	0.994928	0.337798	1.622133
10	8	0	2.974509	-1.348445	0.896186
11	6	0	5.213012	-0.623652	0.506669
12	1	0	5.286070	-0.178408	1.513672
13	1	0	5.474214	-1.682191	0.597889
14	8	0	6.120573	-0.039774	-0.406331
15	1	0	5.776411	0.841829	-0.619541
16	8	0	3.973985	1.731728	-0.796410
17	1	0	3.517389	2.585382	-0.828015
18	8	0	1.302347	2.273818	-0.181686
19	1	0	0.438972	2.347689	-0.609167
20	7	0	-0.428111	-0.130782	0.127112
21	6	0	-2.783715	0.124700	0.564013
22	6	0	-3.188551	-0.249986	-0.752236
23	6	0	-3.780992	0.444655	1.504630
24	6	0	-4.544370	-0.298765	-1.081058
25	6	0	-5.125284	0.396660	1.184177
26	1	0	-3.478736	0.733462	2.508072
27	6	0	-5.502438	0.019846	-0.120178
28	1	0	-4.846790	-0.584117	-2.081397
29	1	0	-5.882268	0.644673	1.923168
30	6	0	-1.388028	0.182616	0.934676
31	1	0	-1.166913	0.514668	1.958900
32	1	0	1.174879	-2.067224	1.351639
33	8	0	1.487813	-2.048844	-0.700068
34	1	0	0.565180	-1.941267	-0.973992
35	8	0	-2.286923	-0.554228	-1.697672
36	1	0	-1.387980	-0.454462	-1.283857
37	8	0	-6.804906	-0.047981	-0.500571
38	1	0	-7.370897	0.190039	0.245912

Structure 34 (B3LYP, DMSO)

Energy (Hartrees): = - 1086.9631134
No imaginary frequencies

Standard orientation:

Center Number	Atomic Number	Atomic Type	Coordinates (Angstroms) X	Y	Z
1	6	0	1.618309	-1.509760	0.381707
2	6	0	0.948478	-0.126240	0.585680
3	6	0	1.733594	0.952128	-0.178124
4	6	0	3.208567	0.885367	0.195683
5	6	0	3.760530	-0.522686	-0.053082
6	1	0	1.638025	0.767322	-1.256621
7	1	0	3.310182	1.116869	1.267439
8	1	0	3.699406	-0.752058	-1.123706
9	1	0	0.993605	0.102550	1.659828
10	8	0	2.984923	-1.466867	0.702558
11	6	0	5.211404	-0.680695	0.405518
12	1	0	5.286325	-0.397265	1.468218
13	1	0	5.492689	-1.736005	0.327117
14	8	0	6.112677	0.060536	-0.403787
15	1	0	5.729133	0.949987	-0.484540
16	8	0	3.969721	1.813764	-0.581687
17	1	0	3.544788	2.678278	-0.467470
18	8	0	1.291454	2.270249	0.141342
19	1	0	0.423022	2.395387	-0.269433
20	7	0	-0.426677	-0.161520	0.110547
21	6	0	-2.781616	0.002706	0.578336
22	6	0	-3.179902	-0.087159	-0.787438
23	6	0	-3.783318	0.122195	1.560689
24	6	0	-4.534557	-0.063086	-1.126022
25	6	0	-5.127886	0.142715	1.232817
26	1	0	-3.482874	0.196891	2.602440
27	6	0	-5.499047	0.047562	-0.122424
28	1	0	-4.833507	-0.130818	-2.166203
29	1	0	-5.889117	0.232258	2.002733
30	6	0	-1.386392	-0.005648	0.963233
31	1	0	-1.172955	0.137639	2.029523
32	1	0	1.184763	-2.228281	1.090733
33	8	0	1.473029	-1.972653	-0.947177
34	1	0	0.572590	-1.740738	-1.225240
35	8	0	-2.270742	-0.186814	-1.771791
36	1	0	-1.373400	-0.183919	-1.334351
37	8	0	-6.798408	0.061425	-0.512546
38	1	0	-7.366165	0.141034	0.270980

Structure 34 (M06-2X, Gas Phase)

Energy (Hartrees): = - 1086.803545
No imaginary frequencies

Standard orientation:

Center Number	Atomic Number	Atomic Type	Coordinates (Angstroms) X	Y	Z
1	6	0	1.641966	-1.404085	0.669078
2	6	0	0.951439	-0.026073	0.602906
3	6	0	1.714806	0.872142	-0.367610
4	6	0	3.175389	0.919242	0.033405
5	6	0	3.746722	-0.494037	0.066647
6	1	0	1.645157	0.455085	-1.379310
7	1	0	3.246795	1.349587	1.043731
8	1	0	3.688273	-0.936690	-0.932971
9	1	0	0.996178	0.427205	1.602913
10	8	0	2.986978	-1.263235	0.997287
11	6	0	5.194946	-0.528208	0.534765
12	1	0	5.280730	0.034216	1.475716
13	1	0	5.462428	-1.566216	0.736980
14	8	0	6.074445	-0.045564	-0.452348
15	1	0	5.737450	0.809518	-0.741289
16	8	0	3.920341	1.695752	-0.885112
17	1	0	3.448493	2.528526	-0.993131
18	8	0	1.237820	2.206464	-0.340201
19	1	0	0.383633	2.233411	-0.779512
20	7	0	-0.416316	-0.175978	0.150721
21	6	0	-2.765616	0.171663	0.532258
22	6	0	-3.178501	-0.346538	-0.720206
23	6	0	-3.744891	0.609363	1.431894
24	6	0	-4.531589	-0.412700	-1.032823
25	6	0	-5.088338	0.542737	1.129879
26	1	0	-3.429610	1.007363	2.390891
27	6	0	-5.475681	0.024973	-0.114880
28	1	0	-4.844795	-0.807192	-1.990042
29	1	0	-5.835589	0.884012	1.837130
30	6	0	-1.363847	0.252915	0.896839
31	1	0	-1.141474	0.713691	1.867933
32	1	0	1.202891	-2.000597	1.475650
33	8	0	1.542198	-2.072710	-0.560911
34	1	0	0.620300	-2.037744	-0.836263
35	8	0	-2.296949	-0.772323	-1.629696
36	1	0	-1.399126	-0.650643	-1.250773
37	8	0	-6.774300	-0.068399	-0.477500
38	1	0	-7.332625	0.260058	0.232226

Structure 34 (M06-2X, DMSO)

Energy (Hartrees): = - 1086.8364919
No imaginary frequencies

Standard orientation:

Center Number	Atomic Number	Atomic Type	Coordinates (Angstroms) X	Y	Z
1	6	0	1.635313	-1.430972	0.620481
2	6	0	0.952293	-0.051638	0.600452
3	6	0	1.709883	0.881698	-0.341830
4	6	0	3.170397	0.913456	0.058336
5	6	0	3.743473	-0.499469	0.046215
6	1	0	1.631897	0.506308	-1.369375
7	1	0	3.247964	1.315823	1.078384
8	1	0	3.692913	-0.903287	-0.970910
9	1	0	0.996797	0.361234	1.616354
10	8	0	2.989141	-1.313467	0.941594
11	6	0	5.187816	-0.543326	0.523547
12	1	0	5.263844	-0.023637	1.488558
13	1	0	5.472189	-1.586158	0.676615
14	8	0	6.073654	0.002495	-0.433749
15	1	0	5.706757	0.857388	-0.688818
16	8	0	3.923057	1.711979	-0.842539
17	1	0	3.472435	2.562829	-0.907317
18	8	0	1.233276	2.213648	-0.262578
19	1	0	0.372041	2.250352	-0.693866
20	7	0	-0.417162	-0.186941	0.144482
21	6	0	-2.761899	0.145295	0.542667
22	6	0	-3.171549	-0.298986	-0.737138
23	6	0	-3.743328	0.531178	1.463996
24	6	0	-4.524758	-0.351668	-1.055981
25	6	0	-5.088434	0.479212	1.157548
26	1	0	-3.427056	0.876299	2.443125

27	6	0	-5.473110	0.031027	-0.114166
28	1	0	-4.835612	-0.692319	-2.035801
29	1	0	-5.839330	0.779346	1.880063
30	6	0	-1.358505	0.210976	0.915694
31	1	0	-1.138889	0.626547	1.905429
32	1	0	1.196243	-2.044802	1.412941
33	8	0	1.527613	-2.074804	-0.625176
34	1	0	0.614558	-1.973971	-0.920203
35	8	0	-2.283685	-0.666029	-1.669232
36	1	0	-1.386221	-0.568437	-1.272034
37	8	0	-6.770137	-0.046647	-0.481200
38	1	0	-7.329299	0.246959	0.249584

Structure 35 (B3LYP, Gas Phase)

Energy (Hartrees): = - 1086.934408
No imaginary frequencies

Standard orientation:

Center Number	Atomic Number	Atomic Type	Coordinates (Angstroms) X	Y	Z
1	6	0	1.582763	1.382381	0.408114
2	6	0	0.960325	0.240100	-0.417200
3	6	0	1.789031	-1.035456	-0.204930
4	6	0	3.266871	-0.777304	-0.468172
5	6	0	3.757566	0.394246	0.392094
6	1	0	1.495763	1.152682	1.486569
7	1	0	1.679055	-1.352993	0.844294
8	1	0	3.389859	-0.499385	-1.527138
9	1	0	3.657026	0.126588	1.456608
10	1	0	1.029068	0.544258	-1.472646
11	8	0	2.959132	1.537847	0.081304
12	6	0	5.221350	0.758969	0.127439
13	1	0	5.356673	0.931108	-0.953513
14	1	0	5.441829	1.701287	0.638384
15	8	0	6.110591	-0.215271	0.634038
16	1	0	5.805843	-1.073582	0.299360
17	8	0	4.040566	-1.934722	-0.165487
18	1	0	3.615204	-2.674780	-0.623428
19	8	0	1.397164	-2.088600	-1.078857
20	1	0	0.488166	-2.330234	-0.853699
21	8	0	0.922662	2.559142	0.074939
22	1	0	1.329041	3.276136	0.581216
23	7	0	-0.412430	-0.005841	-0.017447
24	6	0	-2.764846	0.196958	-0.460281
25	6	0	-3.159171	-0.393356	0.778082
26	6	0	-3.768727	0.610693	-1.354647
27	6	0	-4.514993	-0.550151	1.076622
28	6	0	-5.112449	0.456972	-1.063074
29	1	0	-3.472411	1.063656	-2.297400
30	6	0	-5.479875	-0.128976	0.163983
31	1	0	-4.811031	-0.997639	2.017789
32	1	0	-5.875159	0.782872	-1.765200
33	6	0	-1.369311	0.374545	-0.795880
34	1	0	-1.151531	0.858563	-1.757551
35	8	0	-2.252564	-0.807298	1.673189
36	1	0	-1.352256	-0.602126	1.294176
37	8	0	-6.782352	-0.309244	0.512917
38	1	0	-7.350927	0.021923	-0.194934

Structure 35 (B3LYP, DMSO)

Energy (Hartrees): = - 1086.9642467
No imaginary frequencies

Standard orientation:

Center Number	Atomic Number	Atomic Type	Coordinates (Angstroms) X	Y	Z
1	6	0	1.638147	1.480531	0.162459
2	6	0	0.962893	0.241829	-0.450724
3	6	0	1.730313	-1.016952	-0.004826
4	6	0	3.218252	-0.878143	-0.304400
5	6	0	3.767827	0.410477	0.320574
6	1	0	1.556554	1.444018	1.262495
7	1	0	1.605778	-1.134550	1.081813
8	1	0	3.353907	-0.818607	-1.395110
9	1	0	3.664807	0.351348	1.415876
10	1	0	1.034702	0.335682	-1.544217
11	8	0	3.021097	1.514103	-0.194296
12	6	0	5.238519	0.658644	-0.022501
13	1	0	5.353117	0.666822	-1.118848
14	1	0	5.523413	1.649964	0.345161

15	8	0	6.101870	-0.286422	0.592311
16	1	0	5.710289	-1.158854	0.418565
17	8	0	3.943805	-1.991691	0.222622
18	1	0	3.518839	-2.788264	-0.132091
19	8	0	1.285570	-2.189115	-0.682751
20	1	0	0.401733	-2.403041	-0.348334
21	8	0	1.032237	2.616388	-0.360665
22	1	0	1.391386	3.382742	0.112798
23	7	0	-0.416373	0.147167	-0.012103
24	6	0	-2.757174	0.030841	-0.514864
25	6	0	-3.149522	-0.028700	0.855092
26	6	0	-3.760073	0.002708	-1.501107
27	6	0	-4.503401	-0.113414	1.191502
28	6	0	-5.103424	-0.080964	-1.174008
29	1	0	-3.463395	0.049057	-2.545724
30	6	0	-5.469888	-0.139359	0.184210
31	1	0	-4.799752	-0.158149	2.233747
32	1	0	-5.866484	-0.101607	-1.947180
33	6	0	-1.360462	0.124521	-0.891348
34	1	0	-1.139526	0.180586	-1.964510
35	8	0	-2.237553	-0.003316	1.839814
36	1	0	-1.339175	0.061793	1.393915
37	8	0	-6.767896	-0.223621	0.574038
38	1	0	-7.336669	-0.231846	-0.212566

Structure 35 (M06-2X, Gas Phase)

Energy (Hartrees): = - 1086.8044116
No imaginary frequencies

Standard orientation:

Center Number	Atomic Number	Atomic Type	Coordinates (Angstroms) X	Y	Z
1	6	0	1.537741	1.318126	0.465993
2	6	0	0.967228	0.213999	-0.426819
3	6	0	1.819392	-1.039661	-0.277052
4	6	0	3.278874	-0.718489	-0.520688
5	6	0	3.717868	0.402809	0.416734
6	1	0	1.446201	1.027412	1.526130
7	1	0	1.716899	-1.417815	0.750759
8	1	0	3.393761	-0.368077	-1.556586
9	1	0	3.606013	0.064891	1.458237
10	1	0	1.036503	0.582269	-1.459525
11	8	0	2.900431	1.535440	0.165701
12	6	0	5.169128	0.812392	0.203621
13	1	0	5.332902	1.001161	-0.866611
14	1	0	5.345398	1.745518	0.740819
15	8	0	6.060054	-0.148063	0.716075
16	1	0	5.806853	-0.999224	0.341637
17	8	0	4.088025	-1.856032	-0.290140
18	1	0	3.691823	-2.586475	-0.777809
19	8	0	1.458854	-2.044176	-1.204063
20	1	0	0.549121	-2.300772	-1.023856
21	8	0	0.838792	2.479587	0.188715
22	1	0	1.203445	3.183842	0.732337
23	7	0	-0.398654	-0.096641	-0.057926
24	6	0	-2.745686	0.271877	-0.387992
25	6	0	-3.151371	-0.584052	0.666025
26	6	0	-3.729695	0.920256	-1.142258
27	6	0	-4.505706	-0.764527	0.929683
28	6	0	-5.073636	0.744871	-0.886050
29	1	0	-3.418206	1.576935	-1.947871
30	6	0	-5.454581	-0.107723	0.159823
31	1	0	-4.814149	-1.416887	1.735389
32	1	0	-5.825418	1.254079	-1.478160
33	6	0	-1.343572	0.482943	-0.696114
34	1	0	-1.121464	1.178291	-1.514136
35	8	0	-2.266265	-1.233220	1.424833
36	1	0	-1.365629	-0.980596	1.115121
37	8	0	-6.754249	-0.328052	0.465698
38	1	0	-7.314503	0.171135	-0.134400

Structure 35 (M06-2X, DMSO)

Energy (Hartrees): = - 1086.8368622
No imaginary frequencies

Standard orientation:

Center Number	Atomic Number	Atomic Type	Coordinates (Angstroms) X	Y	Z
1	6	0	-1.616689	1.431601	-0.308753
2	6	0	-0.967384	0.264797	0.435880

3	6	0	-1.743294	-1.015532	0.129315
4	6	0	-3.216460	-0.820480	0.421737
5	6	0	-3.736941	0.387713	-0.352720
6	1	0	-1.536994	1.272535	-1.395263
7	1	0	-1.631009	-1.249375	-0.938409
8	1	0	-3.346963	-0.629142	1.496054
9	1	0	-3.618054	0.200781	-1.430488
10	1	0	-1.030836	0.482426	1.510870
11	8	0	-2.987260	1.526624	0.040459
12	6	0	-5.201014	0.683370	-0.062696
13	1	0	-5.340840	0.760126	1.024300
14	1	0	-5.454638	1.647923	-0.506773
15	8	0	-6.058587	-0.285403	-0.632551
16	1	0	-5.709022	-1.146868	-0.374632
17	8	0	-3.958226	-1.965758	0.031305
18	1	0	-3.534266	-2.730586	0.439343
19	8	0	-1.305538	-2.105267	0.920809
20	1	0	-0.421531	-2.351762	0.625510
21	8	0	-0.986376	2.597132	0.086609
22	1	0	-1.311044	3.313908	-0.470853
23	7	0	0.402335	0.098392	0.001680
24	6	0	2.745431	0.136579	0.482219
25	6	0	3.130843	-0.231185	-0.830117
26	6	0	3.743105	0.353434	1.439503
27	6	0	4.480086	-0.370083	-1.144137
28	6	0	5.083847	0.219575	1.135266
29	1	0	3.445206	0.634699	2.444539
30	6	0	5.445423	-0.145791	-0.168953
31	1	0	4.774020	-0.652039	-2.147647
32	1	0	5.848136	0.390255	1.885473
33	6	0	1.344090	0.284998	0.845047
34	1	0	1.131931	0.565689	1.882689
35	8	0	2.225574	-0.452602	-1.788959
36	1	0	1.331444	-0.304559	-1.385157
37	8	0	6.737094	-0.296153	-0.535514
38	1	0	7.308415	-0.115316	0.221869

Structure 36a (B3LYP, Gas Phase)

Energy (Hartrees): = - 1087.4985373
No imaginary frequencies

Standard orientation:

Center Number	Atomic Number	Atomic Type	Coordinates (Angstroms) X	Y	Z
1	6	0	1.381390	-1.219950	0.419968
2	6	0	0.986694	0.270050	0.441546
3	6	0	2.005370	1.088131	-0.362642
4	6	0	3.412803	0.800805	0.141626
5	6	0	3.705030	-0.702296	0.074233
6	1	0	1.950432	0.794191	-1.422212
7	1	0	3.471888	1.119600	1.195498
8	1	0	3.668865	-1.028264	-0.972836
9	1	0	1.039576	0.605050	1.483420
10	8	0	2.716639	-1.404755	0.849059
11	6	0	5.074121	-1.071339	0.652121
12	1	0	5.157440	-0.658130	1.671665
13	1	0	5.132899	-2.160994	0.737092
14	8	0	6.133420	-0.654211	-0.185499
15	1	0	5.973358	0.280535	-0.391589
16	8	0	4.387312	1.491964	-0.637151
17	1	0	4.101297	2.416126	-0.679287
18	8	0	1.779891	2.483734	-0.220260
19	1	0	0.836681	2.620474	-0.400271
20	7	0	-0.358159	0.536223	-0.046515
21	6	0	-2.787995	0.268708	0.516245
22	6	0	-3.285060	-0.090063	-0.778582
23	6	0	-3.777671	0.385841	1.535211
24	6	0	-4.632590	-0.308759	-1.014545
25	6	0	-5.126883	0.165449	1.305861
26	1	0	-3.450074	0.659593	2.534191
27	6	0	-5.566356	-0.184517	0.023368
28	1	0	-4.984643	-0.579143	-2.006856
29	1	0	-5.841070	0.267106	2.119818
30	6	0	-1.430089	0.492589	0.839432
31	1	0	-1.186140	0.765170	1.861673
32	1	0	0.777764	-1.786147	1.140514
33	8	0	1.167734	-1.680885	-0.896630
34	1	0	1.341071	-2.632056	-0.908915
35	8	0	-2.361428	-0.212114	-1.792991
36	1	0	-2.811702	-0.481186	-2.603848
37	8	0	-6.880574	-0.416841	-0.286038
38	1	0	-7.413137	-0.294614	0.510503
39	1	0	-0.553929	0.116099	-0.949929

Structure 36a (B3LYP, DMSO)

Energy (Hartrees): = - 1087.5281016
No imaginary frequencies

Standard orientation:

Center Number	Atomic Number	Atomic Type	Coordinates (Angstroms) X	Y	Z
1	6	0	-1.401952	-1.291885	-0.241152
2	6	0	-0.994288	0.174976	-0.478509
3	6	0	-1.991241	1.115362	0.212459
4	6	0	-3.409721	0.779227	-0.227186
5	6	0	-3.719370	-0.700324	0.031903
6	1	0	-1.921898	0.979600	1.302383
7	1	0	-3.491006	0.969965	-1.308589
8	1	0	-3.689374	-0.891241	1.112405
9	1	0	-1.049502	0.355865	-1.556898
10	8	0	-2.746612	-1.514272	-0.642553
11	6	0	-5.089881	-1.119473	-0.505233
12	1	0	-5.145174	-0.874775	-1.578731
13	1	0	-5.186673	-2.206247	-0.411806
14	8	0	-6.156934	-0.533286	0.226094
15	1	0	-5.935035	0.409499	0.310137
16	8	0	-4.370478	1.568361	0.483649
17	1	0	-4.138306	2.496988	0.329466
18	8	0	-1.749651	2.475161	-0.132422
19	1	0	-0.807193	2.630030	0.042955
20	7	0	0.359170	0.485178	-0.042292
21	6	0	2.788517	0.144976	-0.551886
22	6	0	3.297489	0.064113	0.787309
23	6	0	3.772512	0.059836	-1.582919
24	6	0	4.651134	-0.083999	1.051899
25	6	0	5.127151	-0.090145	-1.324318
26	1	0	3.434576	0.116154	-2.614222
27	6	0	5.578136	-0.161768	0.001135
28	1	0	5.002512	-0.140816	2.079332
29	1	0	5.837289	-0.150150	-2.145881
30	6	0	1.426468	0.287042	-0.906669
31	1	0	1.174926	0.339657	-1.961381
32	1	0	-0.811104	-1.960154	-0.878419
33	8	0	-1.193348	-1.574763	1.123070
34	1	0	-1.315153	-2.528537	1.248327
35	8	0	2.383292	0.138709	1.809255
36	1	0	2.841991	0.053548	2.659560
37	8	0	6.897172	-0.305801	0.335408
38	1	0	7.420893	-0.344078	-0.479844
39	1	0	0.546776	0.247952	0.926242

Structure 36a (B3LYP, H_2O)

Energy (Hartrees): = - 1087.5387516
No imaginary frequencies

Standard orientation:

Center Number	Atomic Number	Atomic Type	Coordinates (Angstroms) X	Y	Z
1	6	0	-1.336342	-1.249198	-0.174162
2	6	0	-0.994741	0.211778	-0.515727
3	6	0	-2.018357	1.167144	0.112646
4	6	0	-3.432565	0.744169	-0.270265
5	6	0	-3.673242	-0.728289	0.089992
6	1	0	-1.928996	1.127882	1.208331
7	1	0	-3.552273	0.866004	-1.356143
8	1	0	-3.617644	-0.845204	1.179700
9	1	0	-1.068993	0.309188	-1.603736
10	8	0	-2.678407	-1.548430	-0.545645
11	6	0	-5.029485	-1.245375	-0.393910
12	1	0	-5.116832	-1.072356	-1.477228
13	1	0	-5.076143	-2.325135	-0.222616
14	8	0	-6.112280	-0.653052	0.318512
15	1	0	-5.928969	0.303725	0.325787
16	8	0	-4.416772	1.526017	0.421696
17	1	0	-4.380873	2.423913	0.060147
18	8	0	-1.818048	2.502191	-0.348291
19	1	0	-0.877272	2.694227	-0.204118
20	7	0	0.354086	0.601721	-0.123357
21	6	0	2.773166	0.115152	-0.567852
22	6	0	3.273265	0.177003	0.775331
23	6	0	3.757694	-0.118837	-1.575565
24	6	0	4.621542	0.025898	1.065634
25	6	0	5.105841	-0.277021	-1.291392
26	1	0	3.424054	-0.173824	-2.608052

27	6	0	5.545638	-0.203346	0.036817
28	1	0	4.962648	0.081861	2.095834
29	1	0	5.822242	-0.453915	-2.089422
30	6	0	1.418092	0.253164	-0.948868
31	1	0	1.172663	0.188461	-2.004237
32	1	0	-0.726755	-1.933049	-0.773663
33	8	0	-1.107038	-1.448866	1.203016
34	1	0	-1.135208	-2.403088	1.371200
35	8	0	2.354128	0.392821	1.781760
36	1	0	2.806974	0.392759	2.638603
37	8	0	6.871624	-0.346153	0.395208
38	1	0	7.394856	-0.487284	-0.408245
39	1	0	0.539221	0.464101	0.865242

Structure 36a (M06-2X, Gas Phase)

Energy (Hartrees): = - 1087.3669815
No imaginary frequencies

Standard orientation:

Center Number	Atomic Number	Atomic Type	Coordinates (Angstroms) X	Y	Z
1	6	0	-1.340909	-1.162653	-0.463043
2	6	0	-0.984259	0.327955	-0.462446
3	6	0	-2.007726	1.096890	0.364122
4	6	0	-3.398815	0.790221	-0.147376
5	6	0	-3.649567	-0.712545	-0.090830
6	1	0	-1.943846	0.779067	1.414199
7	1	0	-3.462484	1.117712	-1.196119
8	1	0	-3.588701	-1.046314	0.951326
9	1	0	-1.042913	0.692094	-1.492874
10	8	0	-2.664415	-1.373704	-0.884978
11	6	0	-5.013722	-1.100873	-0.643805
12	1	0	-5.148441	-0.634786	-1.630224
13	1	0	-5.033912	-2.183489	-0.778703
14	8	0	-6.050116	-0.764277	0.247015
15	1	0	-5.931005	0.162619	0.483044
16	8	0	-4.381982	1.446261	0.632232
17	1	0	-4.105751	2.365219	0.717337
18	8	0	-1.816826	2.492043	0.254097
19	1	0	-0.885364	2.658838	0.438544
20	7	0	0.348890	0.594718	0.039640
21	6	0	2.766929	0.279231	-0.515348
22	6	0	3.240596	-0.104077	0.771929
23	6	0	3.760153	0.403183	-1.517950
24	6	0	4.578806	-0.336199	1.017496
25	6	0	5.101072	0.168757	-1.278931
26	1	0	3.444924	0.697691	-2.512586
27	6	0	5.520736	-0.203058	-0.003169
28	1	0	4.919728	-0.627287	2.005513
29	1	0	5.824515	0.277925	-2.080178
30	6	0	1.414115	0.522309	-0.842854
31	1	0	1.178820	0.798677	-1.864358
32	1	0	-0.718182	-1.706063	-1.179682
33	8	0	-1.125193	-1.619198	0.846593
34	1	0	-1.248440	-2.572407	0.859985
35	8	0	2.309107	-0.241193	1.765596
36	1	0	2.741707	-0.524599	2.575138
37	8	0	6.822134	-0.449928	0.315485
38	1	0	7.365522	-0.320758	-0.465689
39	1	0	0.525466	0.167959	0.942763

Structure 36a (M06-2X, DMSO)

Energy (Hartrees): = - 1087.3994179
No imaginary frequencies

Standard orientation:

Center Number	Atomic Number	Atomic Type	Coordinates (Angstroms) X	Y	Z
1	6	0	-1.350591	-1.255035	-0.199851
2	6	0	-0.993642	0.194992	-0.544911
3	6	0	-1.998299	1.141407	0.101536
4	6	0	-3.400724	0.737366	-0.299060
5	6	0	-3.654788	-0.717148	0.086674
6	1	0	-1.911681	1.076384	1.194976
7	1	0	-3.494990	0.835406	-1.389972
8	1	0	-3.582626	-0.816926	1.176059
9	1	0	-1.060908	0.309836	-1.630750
10	8	0	-2.685053	-1.545034	-0.552316
11	6	0	-5.022037	-1.210931	-0.362614
12	1	0	-5.143553	-1.005947	-1.435116

13	1	0	-5.065533	-2.292007	-0.216697
14	8	0	-6.066295	-0.637360	0.399055
15	1	0	-5.904528	0.313799	0.410315
16	8	0	-4.368566	1.550001	0.350985
17	1	0	-4.131853	2.468657	0.175162
18	8	0	-1.809326	2.476011	-0.329275
19	1	0	-0.882190	2.692151	-0.170023
20	7	0	0.348393	0.557272	-0.131418
21	6	0	2.764487	0.097805	-0.568444
22	6	0	3.249811	0.170510	0.771208
23	6	0	3.751499	-0.125798	-1.564773
24	6	0	4.592039	0.029610	1.070371
25	6	0	5.095334	-0.269977	-1.270798
26	1	0	3.425833	-0.184350	-2.598008
27	6	0	5.525309	-0.191832	0.054554
28	1	0	4.930478	0.088228	2.100488
29	1	0	5.814766	-0.440016	-2.065655
30	6	0	1.409358	0.223277	-0.951159
31	1	0	1.167479	0.152210	-2.005886
32	1	0	-0.741984	-1.947312	-0.787820
33	8	0	-1.122689	-1.417641	1.172349
34	1	0	-1.180033	-2.357478	1.380182
35	8	0	2.330269	0.383320	1.757235
36	1	0	2.771647	0.395131	2.615679
37	8	0	6.830054	-0.321010	0.419666
38	1	0	7.362680	-0.468018	-0.371167
39	1	0	0.514567	0.427567	0.860151

Structure 36a (M06-2X, H_2O)

Energy (Hartrees): = - 1087.4115838
No imaginary frequencies

Standard orientation:

Center Number	Atomic Number	Atomic Type	Coordinates (Angstroms) X	Y	Z
1	6	0	-1.215954	-1.053116	-0.465236
2	6	0	-0.987603	0.461104	-0.500904
3	6	0	-2.054807	1.178524	0.316916
4	6	0	-3.430769	0.733516	-0.138147
5	6	0	-3.552210	-0.784782	-0.063666
6	1	0	-1.937358	0.929348	1.379800
7	1	0	-3.572627	1.045414	-1.181873
8	1	0	-3.451507	-1.107567	0.978858
9	1	0	-1.075513	0.787836	-1.541005
10	8	0	-2.532458	-1.377464	-0.866544
11	6	0	-4.881164	-1.287449	-0.607826
12	1	0	-5.054003	-0.852360	-1.599930
13	1	0	-4.828825	-2.372333	-0.710720
14	8	0	-5.953104	-0.997436	0.276123
15	1	0	-5.893003	-0.054929	0.481789
16	8	0	-4.447385	1.292624	0.683851
17	1	0	-4.388053	2.252737	0.611044
18	8	0	-1.986439	2.583615	0.133501
19	1	0	-1.100605	2.863816	0.393759
20	7	0	0.335977	0.819286	-0.017763
21	6	0	2.735197	0.314647	-0.523146
22	6	0	3.177461	-0.042333	0.784981
23	6	0	3.741699	0.345249	-1.524426
24	6	0	4.504789	-0.321687	1.054117
25	6	0	5.068702	0.058859	-1.263131
26	1	0	3.445358	0.609700	-2.533731
27	6	0	5.456485	-0.273802	0.034872
28	1	0	4.806434	-0.590624	2.061305
29	1	0	5.809750	0.094244	-2.054637
30	6	0	1.399392	0.608465	-0.881669
31	1	0	1.183391	0.808832	-1.925330
32	1	0	-0.566388	-1.554521	-1.185749
33	8	0	-0.945977	-1.490605	0.842409
34	1	0	-0.888920	-2.453290	0.833762
35	8	0	2.228826	-0.114342	1.772115
36	1	0	2.636778	-0.394527	2.601078
37	8	0	6.755573	-0.565398	0.368380
38	1	0	7.305166	-0.491252	-0.421017
39	1	0	0.498491	0.473343	0.921852

Structure 36b (B3LYP, Gas Phase)

Energy (Hartrees): = - 1087.4945511
No imaginary frequencies

Standard orientation:

Center Number	Atomic Number	Atomic Type	Coordinates (Angstroms) X	Y	Z
1	6	0	-1.191264	0.916876	0.602687
2	6	0	-0.996532	-0.553920	0.183011
3	6	0	-2.138468	-0.994275	-0.739460
4	6	0	-3.479407	-0.689975	-0.085530
5	6	0	-3.572456	0.799275	0.264773
6	1	0	-2.081101	-0.430536	-1.683595
7	1	0	-3.546801	-1.271590	0.848647
8	1	0	-3.523566	1.390509	-0.658378
9	1	0	-1.039442	-1.165754	1.090504
10	8	0	-2.478415	1.142168	1.135334
11	6	0	-4.864134	1.158349	1.005251
12	1	0	-4.974093	0.492936	1.878064
13	1	0	-4.774016	2.180985	1.384547
14	8	0	-5.990574	1.120856	0.153184
15	1	0	-5.969303	0.262906	-0.299644
16	8	0	-4.559073	-1.023236	-0.954014
17	1	0	-4.403898	-1.930945	-1.253415
18	8	0	-2.096105	-2.392148	-0.990512
19	1	0	-1.194664	-2.585211	-1.290017
20	7	0	0.288960	-0.805397	-0.466628
21	6	0	2.681500	-0.400370	0.075436
22	6	0	3.879342	-0.955484	0.632353
23	6	0	2.833293	0.791046	-0.683530
24	6	0	5.109494	-0.339293	0.478870
25	6	0	4.067874	1.404555	-0.849611
26	1	0	1.950689	1.275804	-1.090847
27	6	0	5.209648	0.847435	-0.258956
28	1	0	5.996302	-0.792627	0.906020
29	1	0	4.143703	2.328921	-1.417856
30	6	0	1.428160	-1.025402	0.309279
31	1	0	1.288615	-1.719275	1.132762
32	1	0	-0.503428	1.177026	1.416525
33	8	0	-0.933400	1.701202	-0.547519
34	1	0	-0.995981	2.631259	-0.289926
35	8	0	3.843236	-2.125773	1.341277
36	1	0	3.068575	-2.629257	1.050817
37	8	0	6.452318	1.405235	-0.376674
38	1	0	6.388393	2.200389	-0.921628
39	1	0	0.441110	-0.157095	-1.232228

Structure 36b (B3LYP, DMSO)

Energy (Hartrees): = - 1087.5257457
No imaginary frequencies

Standard orientation:

Center Number	Atomic Number	Atomic Type	Coordinates (Angstroms) X	Y	Z
1	6	0	1.385814	-1.028797	0.738967
2	6	0	1.018632	0.367431	0.197947
3	6	0	2.063569	0.832452	-0.825786
4	6	0	3.458811	0.732805	-0.223709
5	6	0	3.724615	-0.689785	0.283507
6	1	0	2.019671	0.180936	-1.711585
7	1	0	3.518825	1.426357	0.629370
8	1	0	3.705657	-1.384636	-0.565856
9	1	0	1.039025	1.060224	1.045325
10	8	0	2.713917	-1.046697	1.239762
11	6	0	5.071603	-0.822546	0.997236
12	1	0	5.122190	-0.080880	1.811295
13	1	0	5.131320	-1.815710	1.454475
14	8	0	6.167269	-0.700527	0.102069
15	1	0	5.983328	0.088442	-0.435278
16	8	0	4.461359	1.057807	-1.192709
17	1	0	4.254183	1.945674	-1.522489
18	8	0	1.860679	2.190609	-1.200771
19	1	0	0.940276	2.253609	-1.502593
20	7	0	-0.311582	0.426605	-0.393170
21	6	0	-2.738020	0.280078	0.097670
22	6	0	-3.856170	0.860192	0.788383
23	6	0	-3.051750	-0.740556	-0.845984
24	6	0	-5.152084	0.415190	0.584216
25	6	0	-4.353095	-1.182988	-1.056429
26	1	0	-2.251391	-1.225872	-1.396940
27	6	0	-5.409499	-0.616128	-0.332864
28	1	0	-5.970235	0.885127	1.119960
29	1	0	-4.548851	-1.978204	-1.771978
30	6	0	-1.420415	0.715006	0.384918
31	1	0	-1.207283	1.339276	1.245754
32	1	0	0.762183	-1.276606	1.605710

33	8	0	1.190077	-1.951940	-0.307796
34	1	0	1.276982	-2.842941	0.064871
35	8	0	-3.681535	1.875665	1.689905
36	1	0	-2.860944	2.347701	1.475173
37	8	0	-6.710527	-1.008598	-0.485893
38	1	0	-6.746779	-1.711546	-1.153068
39	1	0	-0.449036	-0.261847	-1.124923

Structure 36b (B3LYP, H_2O)

Energy (Hartrees): = - 1087.5380489
No imaginary frequencies

Standard orientation:

Center Number	Atomic Number	Atomic Type	Coordinates (Angstroms) X	Y	Z
1	6	0	-1.973485	1.560932	0.858890
2	6	0	-1.068117	0.325300	0.752125
3	6	0	-1.570035	-0.621639	-0.360010
4	6	0	-3.058500	-0.909754	-0.176977
5	6	0	-3.859349	0.392198	-0.074798
6	1	0	-1.424257	-0.142241	-1.337496
7	1	0	-3.191422	-1.475811	0.756414
8	1	0	-3.779465	0.942430	-1.020542
9	1	0	-1.139162	-0.210090	1.704324
10	8	0	-3.336386	1.184531	1.004094
11	6	0	-5.338840	0.157412	0.236240
12	1	0	-5.425955	-0.463305	1.140697
13	1	0	-5.812852	1.121430	0.444166
14	8	0	-6.027793	-0.426257	-0.866362
15	1	0	-5.482115	-1.181270	-1.149407
16	8	0	-3.579041	-1.657965	-1.282720
17	1	0	-3.131655	-2.517434	-1.281732
18	8	0	-0.900547	-1.883225	-0.325248
19	1	0	0.027593	-1.733093	-0.564848
20	7	0	0.315662	0.750984	0.577644
21	6	0	2.666286	0.019977	0.315721
22	6	0	3.658898	-0.983908	0.564069
23	6	0	3.104662	1.157696	-0.420749
24	6	0	4.973784	-0.833636	0.157128
25	6	0	4.423405	1.310637	-0.835368
26	1	0	2.401163	1.953254	-0.645132
27	6	0	5.366555	0.322381	-0.534526
28	1	0	5.692215	-1.620958	0.359655
29	1	0	4.727992	2.199743	-1.381082
30	6	0	1.344372	-0.150538	0.801904
31	1	0	1.122765	-0.911197	1.541008
32	1	0	-1.746662	2.124132	1.769006
33	8	0	-1.758035	2.355154	-0.288311
34	1	0	-2.169258	3.218947	-0.132635
35	8	0	3.340386	-2.150264	1.234027
36	1	0	2.398259	-2.340789	1.104799
37	8	0	6.692981	0.415360	-0.904350
38	1	0	6.824159	1.242521	-1.392321
39	1	0	0.428681	1.355819	-0.229312

Structure 36b (M06-2X, Gas Phase)

Energy (Hartrees): = - 1087.3646398
No imaginary frequencies

Standard orientation:

Center Number	Atomic Number	Atomic Type	Coordinates (Angstroms) X	Y	Z
1	6	0	1.068937	-0.790738	0.580445
2	6	0	0.984254	0.684877	0.173735
3	6	0	2.156826	1.043391	-0.728140
4	6	0	3.449707	0.652115	-0.044597
5	6	0	3.431994	-0.835784	0.286561
6	1	0	2.080133	0.486163	-1.672587
7	1	0	3.528357	1.219412	0.895095
8	1	0	3.352653	-1.410253	-0.643588
9	1	0	1.052640	1.293008	1.080977
10	8	0	2.316327	-1.105072	1.137191
11	6	0	4.685989	-1.285169	1.024424
12	1	0	4.873682	-0.602023	1.864742
13	1	0	4.505822	-2.280437	1.433746
14	8	0	5.791497	-1.376038	0.158566
15	1	0	5.861458	-0.532066	-0.301432
16	8	0	4.563667	0.918142	-0.875450
17	1	0	4.471952	1.824257	-1.189321

18	8	0	2.215173	2.433066	-0.974063
19	1	0	1.340588	2.700977	-1.276863
20	7	0	-0.270119	1.006203	-0.492438
21	6	0	-2.621086	0.466637	0.058076
22	6	0	-3.840159	0.934787	0.625309
23	6	0	-2.689859	-0.720844	-0.703266
24	6	0	-5.022027	0.241578	0.473185
25	6	0	-3.877710	-1.412924	-0.867771
26	1	0	-1.778610	-1.137483	-1.118608
27	6	0	-5.045193	-0.939082	-0.269819
28	1	0	-5.933511	0.628081	0.909688
29	1	0	-3.896523	-2.333021	-1.442637
30	6	0	-1.408495	1.168481	0.290712
31	1	0	-1.293132	1.837317	1.135712
32	1	0	0.334472	-1.015294	1.359617
33	8	0	0.808976	-1.535569	-0.586385
34	1	0	0.790141	-2.467764	-0.349751
35	8	0	-3.870686	2.090352	1.343367
36	1	0	-3.156209	2.658497	1.036753
37	8	0	-6.241362	-1.574209	-0.384644
38	1	0	-6.131397	-2.359571	-0.926439
39	1	0	-0.419487	0.397453	-1.289812

Structure 36b (M06-2X, DMSO)

Energy (Hartrees): = - 1087.3979626
No imaginary frequencies

Standard orientation:

Center Number	Atomic Number	Atomic Type	Coordinates (Angstroms) X	Y	Z
1	6	0	1.251661	-0.906153	0.686025
2	6	0	1.013153	0.516902	0.167602
3	6	0	2.103551	0.897096	-0.825963
4	6	0	3.458532	0.691252	-0.180741
5	6	0	3.597526	-0.750068	0.299131
6	1	0	2.034115	0.252788	-1.713358
7	1	0	3.535353	1.360952	0.687855
8	1	0	3.543949	-1.424384	-0.563530
9	1	0	1.067528	1.199878	1.020717
10	8	0	2.548031	-1.037111	1.220969
11	6	0	4.904519	-0.997111	1.037778
12	1	0	5.008872	-0.252543	1.838880
13	1	0	4.858796	-1.985964	1.498335
14	8	0	6.017654	-0.985113	0.166510
15	1	0	5.939783	-0.183511	-0.364549
16	8	0	4.505157	0.961584	-1.102427
17	1	0	4.342951	1.840276	-1.466465
18	8	0	2.021516	2.259783	-1.197498
19	1	0	1.122267	2.412672	-1.512671
20	7	0	-0.293612	0.672080	-0.447332
21	6	0	-2.687350	0.357456	0.074435
22	6	0	-3.809675	0.790809	0.845007
23	6	0	-2.957493	-0.591797	-0.942120
24	6	0	-5.073614	0.278547	0.637695
25	6	0	-4.229140	-1.100849	-1.157099
26	1	0	-2.148169	-0.972164	-1.554463
27	6	0	-5.290710	-0.678119	-0.359074
28	1	0	-5.900719	0.633782	1.240812
29	1	0	-4.394962	-1.840890	-1.933704
30	6	0	-1.392350	0.847142	0.368946
31	1	0	-1.184607	1.396018	1.279496
32	1	0	0.574196	-1.125740	1.515453
33	8	0	1.034939	-1.777063	-0.389709
34	1	0	1.011325	-2.678257	-0.047339
35	8	0	-3.666455	1.721888	1.827312
36	1	0	-2.904412	2.280704	1.627144
37	8	0	-6.558650	-1.146298	-0.507629
38	1	0	-6.574764	-1.786360	-1.229370
39	1	0	-0.425411	0.056183	-1.241876

Structure 36b (M06-2X, H_2O)

Energy (Hartrees): = - 1087.4105439
No imaginary frequencies

Standard orientation:

Center Number	Atomic Number	Atomic Type	Coordinates (Angstroms) X	Y	Z
1	6	0	1.242922	-0.910716	0.648587
2	6	0	1.016736	0.530031	0.182521

3	6	0	2.103640	0.942162	-0.802100
4	6	0	3.466428	0.695134	-0.184041
5	6	0	3.594906	-0.757894	0.261997
6	1	0	2.019336	0.344106	-1.719000
7	1	0	3.573929	1.345361	0.694858
8	1	0	3.541276	-1.411781	-0.615821
9	1	0	1.082708	1.178644	1.060641
10	8	0	2.544302	-1.066960	1.177300
11	6	0	4.894716	-1.029874	1.004301
12	1	0	5.003193	-0.303141	1.818755
13	1	0	4.842076	-2.029543	1.438183
14	8	0	6.017899	-1.004831	0.137715
15	1	0	5.970513	-0.173593	-0.352276
16	8	0	4.504158	0.956957	-1.119914
17	1	0	4.428061	1.880690	-1.386967
18	8	0	2.026122	2.324196	-1.109572
19	1	0	1.136333	2.494418	-1.441889
20	7	0	-0.297205	0.713018	-0.414191
21	6	0	-2.681264	0.335456	0.108413
22	6	0	-3.787256	0.653135	0.953195
23	6	0	-2.975958	-0.455034	-1.031223
24	6	0	-5.059690	0.189901	0.694559
25	6	0	-4.255092	-0.918199	-1.296294
26	1	0	-2.181064	-0.737995	-1.711178
27	6	0	-5.298775	-0.606219	-0.428711
28	1	0	-5.873960	0.459047	1.356648
29	1	0	-4.449543	-1.530964	-2.170110
30	6	0	-1.381847	0.780848	0.440570
31	1	0	-1.169202	1.242620	1.396422
32	1	0	0.568245	-1.156895	1.471075
33	8	0	1.020397	-1.754230	-0.452187
34	1	0	0.965430	-2.661772	-0.130155
35	8	0	-3.618522	1.427198	2.075793
36	1	0	-2.877670	2.031568	1.942340
37	8	0	-6.585239	-1.036459	-0.631939
38	1	0	-6.625080	-1.536628	-1.456274
39	1	0	-0.423876	0.115393	-1.224133

Structure 37a (B3LYP, Gas Phase)

Energy (Hartrees): = - 1087.4934857
No imaginary frequencies

Standard orientation:

Center Number	Atomic Number	Atomic Type	Coordinates (Angstroms) X	Y	Z
1	6	0	1.445690	1.242250	0.350661
2	6	0	1.021367	0.037735	-0.511464
3	6	0	1.993595	-1.126395	-0.274126
4	6	0	3.439160	-0.681688	-0.447424
5	6	0	3.727760	0.523634	0.456294
6	1	0	1.326306	0.994312	1.424470
7	1	0	1.877513	-1.476534	0.768317
8	1	0	3.582339	-0.369359	-1.493939
9	1	0	3.598410	0.224409	1.509519
10	1	0	1.104117	0.357745	-1.556629
11	8	0	2.813803	1.566262	0.111434
12	6	0	5.147572	1.073215	0.287982
13	1	0	5.324106	1.284246	-0.779998
14	1	0	5.216914	2.024402	0.824647
15	8	0	6.120464	0.206356	0.834837
16	1	0	5.945637	-0.675146	0.468586
17	8	0	4.337846	-1.738075	-0.116379
18	1	0	4.057095	-2.511533	-0.626821
19	8	0	1.760962	-2.195869	-1.175862
20	1	0	0.799050	-2.324482	-1.193709
21	8	0	0.674943	2.337993	-0.013604
22	1	0	0.960136	3.090379	0.523475
23	7	0	-0.332687	-0.444257	-0.291314
24	6	0	-2.764235	0.102932	-0.492239
25	6	0	-3.238809	-0.535043	0.699883
26	6	0	-3.773823	0.707128	-1.298355
27	6	0	-4.582336	-0.565675	1.034612
28	6	0	-5.118967	0.683479	-0.966528
29	1	0	-3.463246	1.206884	-2.211356
30	6	0	-5.536091	0.042103	0.206371
31	1	0	-4.916333	-1.057304	1.944928
32	1	0	-5.848244	1.160792	-1.617204
33	6	0	-1.415497	0.179534	-0.901058
34	1	0	-1.178958	0.717637	-1.811670
35	8	0	-2.295674	-1.122301	1.520366
36	1	0	-2.739846	-1.501990	2.289314
37	8	0	-6.845996	-0.027768	0.603132
38	1	0	-7.391680	0.430495	-0.048959
39	1	0	-0.527604	-0.777210	0.647186

Structure 37a (B3LYP, DMSO)

Energy (Hartrees): = - 1087.5252724
No imaginary frequencies

Standard orientation:

Center Number	Atomic Number	Atomic Type	Coordinates (Angstroms) X	Y	Z
1	6	0	1.484182	1.274574	0.332485
2	6	0	1.025507	0.048798	-0.479466
3	6	0	1.979663	-1.126221	-0.212245
4	6	0	3.431764	-0.719437	-0.421441
5	6	0	3.754688	0.523758	0.417895
6	1	0	1.390618	1.065071	1.412943
7	1	0	1.864600	-1.432848	0.841204
8	1	0	3.579546	-0.469842	-1.482974
9	1	0	3.635947	0.277460	1.485198
10	1	0	1.083279	0.320704	-1.539414
11	8	0	2.855623	1.567866	0.041693
12	6	0	5.176426	1.044110	0.192035
13	1	0	5.320587	1.238082	-0.883484
14	1	0	5.288148	1.999040	0.716060
15	8	0	6.160748	0.158997	0.706430
16	1	0	5.916392	-0.725259	0.384911
17	8	0	4.318167	-1.774957	-0.034710
18	1	0	4.070029	-2.554703	-0.554731
19	8	0	1.712132	-2.224589	-1.075053
20	1	0	0.757796	-2.390979	-1.004694
21	8	0	0.723120	2.373824	-0.043178
22	1	0	0.922807	3.095282	0.573125
23	7	0	-0.332324	-0.386774	-0.194841
24	6	0	-2.771678	0.072745	-0.489871
25	6	0	-3.254931	-0.425816	0.766770
26	6	0	-3.780661	0.542714	-1.385358
27	6	0	-4.606122	-0.450928	1.078242
28	6	0	-5.133123	0.522753	-1.077538
29	1	0	-3.463907	0.929730	-2.350216
30	6	0	-5.557820	0.021343	0.160813
31	1	0	-4.936736	-0.836745	2.039590
32	1	0	-5.862534	0.892138	-1.794824
33	6	0	-1.415471	0.132164	-0.883259
34	1	0	-1.180706	0.546897	-1.857779
35	8	0	-2.316629	-0.879833	1.662470
36	1	0	-2.761186	-1.181290	2.470035
37	8	0	-6.873867	-0.035532	0.533571
38	1	0	-7.415086	0.322449	-0.186934
39	1	0	-0.512212	-0.637321	0.771718

Structure 37a (B3LYP, H_2O)

Energy (Hartrees): = - 1087.5368032
No imaginary frequencies

Standard orientation:

Center Number	Atomic Number	Atomic Type	Coordinates (Angstroms) X	Y	Z
1	6	0	1.407473	1.219884	0.270478
2	6	0	1.024240	-0.043582	-0.519030
3	6	0	2.023058	-1.170164	-0.207104
4	6	0	3.464936	-0.704293	-0.380889
5	6	0	3.708311	0.580476	0.421965
6	1	0	1.300227	1.040830	1.352012
7	1	0	1.891342	-1.470632	0.844951
8	1	0	3.643444	-0.490738	-1.444142
9	1	0	3.576488	0.367116	1.493945
10	1	0	1.091672	0.203450	-1.584366
11	8	0	2.769747	1.571895	-0.005257
12	6	0	5.103899	1.168319	0.202009
13	1	0	5.259078	1.333485	-0.874905
14	1	0	5.159332	2.140315	0.701222
15	8	0	6.126469	0.346419	0.757971
16	1	0	5.939444	-0.557101	0.446563
17	8	0	4.386184	-1.699498	0.083310
18	1	0	4.317136	-2.457921	-0.515328
19	8	0	1.818077	-2.290379	-1.064198
20	1	0	0.875658	-2.512440	-0.993260
21	8	0	0.606434	2.287056	-0.149843
22	1	0	0.678843	2.991924	0.511566
23	7	0	-0.324163	-0.523483	-0.245842
24	6	0	-2.757989	0.005862	-0.507757
25	6	0	-3.236286	-0.435664	0.771315

26	6	0	-3.764062	0.482983	-1.402829
27	6	0	-4.581932	-0.399326	1.105892
28	6	0	-5.110353	0.524217	-1.073147
29	1	0	-3.448920	0.826789	-2.384090
30	6	0	-5.527735	0.080309	0.188347
31	1	0	-4.906061	-0.743467	2.084431
32	1	0	-5.841490	0.895704	-1.786253
33	6	0	-1.408201	0.013510	-0.925997
34	1	0	-1.180745	0.393635	-1.916383
35	8	0	-2.297365	-0.900344	1.671110
36	1	0	-2.740211	-1.162942	2.492345
37	8	0	-6.850525	0.088451	0.586884
38	1	0	-7.390272	0.439415	-0.137576
39	1	0	-0.500158	-0.737035	0.730832

Structure 37a (M06-2X, Gas Phase)

Energy (Hartrees): = - 1087.3609737
No imaginary frequencies

Standard orientation:

Center Number	Atomic Number	Atomic Type	Coordinates (Angstroms) X	Y	Z
1	6	0	1.454945	1.233345	0.383944
2	6	0	1.024011	0.069125	-0.512011
3	6	0	1.977263	-1.104531	-0.319070
4	6	0	3.413692	-0.658908	-0.486615
5	6	0	3.705668	0.496950	0.464871
6	1	0	1.332032	0.952961	1.446212
7	1	0	1.865189	-1.495079	0.706467
8	1	0	3.549238	-0.302144	-1.517622
9	1	0	3.563116	0.155500	1.501803
10	1	0	1.105296	0.423113	-1.545591
11	8	0	2.813660	1.556388	0.156985
12	6	0	5.127958	1.024686	0.330967
13	1	0	5.336381	1.222474	-0.729683
14	1	0	5.194299	1.971216	0.869556
15	8	0	6.065088	0.144070	0.901781
16	1	0	5.907065	-0.725799	0.517832
17	8	0	4.304845	-1.723479	-0.208638
18	1	0	4.009242	-2.482006	-0.723858
19	8	0	1.738784	-2.131143	-1.255992
20	1	0	0.784059	-2.263942	-1.287975
21	8	0	0.688011	2.333950	0.052640
22	1	0	0.972536	3.070556	0.601746
23	7	0	-0.327372	-0.398228	-0.292747
24	6	0	-2.746325	0.177785	-0.446803
25	6	0	-3.226088	-0.614292	0.635940
26	6	0	-3.741348	0.887189	-1.166922
27	6	0	-4.567735	-0.691735	0.947840
28	6	0	-5.085474	0.816461	-0.856602
29	1	0	-3.422522	1.506661	-1.997424
30	6	0	-5.510722	0.020366	0.205591
31	1	0	-4.910704	-1.303079	1.776222
32	1	0	-5.808275	1.378164	-1.439230
33	6	0	-1.395734	0.299793	-0.829133
34	1	0	-1.145627	0.957582	-1.650836
35	8	0	-2.294544	-1.302774	1.373908
36	1	0	-2.742698	-1.800879	2.062482
37	8	0	-6.817580	-0.104491	0.573431
38	1	0	-7.359552	0.425421	-0.016344
39	1	0	-0.504250	-0.820694	0.610575

Structure 37a (M06-2X, DMSO)

Energy (Hartrees): = - 1087.3959357
No imaginary frequencies

Standard orientation:

Center Number	Atomic Number	Atomic Type	Coordinates (Angstroms) X	Y	Z
1	6	0	1.455927	1.243259	0.322419
2	6	0	1.025481	0.025561	-0.498971
3	6	0	1.985831	-1.129687	-0.232476
4	6	0	3.420027	-0.693378	-0.440904
5	6	0	3.712098	0.532877	0.419291
6	1	0	1.358804	1.019498	1.397350
7	1	0	1.874100	-1.445406	0.816269
8	1	0	3.559010	-0.421246	-1.496410
9	1	0	3.577160	0.268819	1.478879
10	1	0	1.082003	0.303351	-1.556730

11	8	0	2.811636	1.563715	0.045198
12	6	0	5.123710	1.066062	0.222883
13	1	0	5.298565	1.227542	-0.849649
14	1	0	5.203959	2.030233	0.728735
15	8	0	6.095177	0.207937	0.787475
16	1	0	5.895253	-0.679594	0.466401
17	8	0	4.319242	-1.731564	-0.077777
18	1	0	4.049999	-2.523780	-0.558227
19	8	0	1.746873	-2.220452	-1.099308
20	1	0	0.809081	-2.436586	-1.023181
21	8	0	0.673842	2.321070	-0.047030
22	1	0	0.846651	3.040609	0.571282
23	7	0	-0.322650	-0.418189	-0.210989
24	6	0	-2.750056	0.079144	-0.482688
25	6	0	-3.229048	-0.456491	0.751661
26	6	0	-3.749765	0.588579	-1.355211
27	6	0	-4.574436	-0.480277	1.064569
28	6	0	-5.097436	0.570236	-1.044781
29	1	0	-3.430019	1.004597	-2.304858
30	6	0	-5.520276	0.031399	0.171249
31	1	0	-4.907052	-0.894101	2.011803
32	1	0	-5.825586	0.970689	-1.743522
33	6	0	-1.395613	0.136448	-0.873953
34	1	0	-1.157049	0.595426	-1.825880
35	8	0	-2.295916	-0.943118	1.622993
36	1	0	-2.734181	-1.266742	2.419954
37	8	0	-6.828853	-0.023931	0.544935
38	1	0	-7.369570	0.363173	-0.153826
39	1	0	-0.487235	-0.700823	0.748124

Structure 37a (M06-2X, H_2O)

Energy (Hartrees): = - 1087.4088298
No imaginary frequencies

Standard orientation:

Center Number	Atomic Number	Atomic Type	Coordinates (Angstroms) X	Y	Z
1	6	0	1.403683	1.202150	0.274564
2	6	0	1.024334	-0.049098	-0.517647
3	6	0	2.016854	-1.166614	-0.207641
4	6	0	3.443678	-0.693191	-0.402093
5	6	0	3.683728	0.575461	0.411309
6	1	0	1.305177	1.012638	1.353140
7	1	0	1.895907	-1.464886	0.844138
8	1	0	3.602081	-0.463469	-1.464041
9	1	0	3.548570	0.351623	1.479176
10	1	0	1.086765	0.199532	-1.582185
11	8	0	2.750155	1.562680	-0.006564
12	6	0	5.072295	1.159484	0.197148
13	1	0	5.248760	1.282332	-0.878570
14	1	0	5.113021	2.142855	0.667840
15	8	0	6.079278	0.361252	0.798533
16	1	0	5.942924	-0.541367	0.481382
17	8	0	4.368945	-1.681370	0.031968
18	1	0	4.235697	-2.466577	-0.512089
19	8	0	1.815045	-2.282815	-1.056790
20	1	0	0.888683	-2.539751	-0.967490
21	8	0	0.590747	2.257066	-0.131399
22	1	0	0.660223	2.961779	0.523663
23	7	0	-0.317361	-0.519002	-0.230762
24	6	0	-2.740295	0.021257	-0.497024
25	6	0	-3.218771	-0.439465	0.766531
26	6	0	-3.734940	0.509885	-1.387047
27	6	0	-4.561600	-0.413345	1.091910
28	6	0	-5.079141	0.541167	-1.065519
29	1	0	-3.413188	0.868794	-2.358657
30	6	0	-5.499607	0.076079	0.180581
31	1	0	-4.890601	-0.772459	2.061732
32	1	0	-5.807862	0.922358	-1.773060
33	6	0	-1.389321	0.032276	-0.904524
34	1	0	-1.154412	0.436513	-1.882047
35	8	0	-2.286903	-0.906623	1.658882
36	1	0	-2.726207	-1.190344	2.470117
37	8	0	-6.817558	0.073323	0.569081
38	1	0	-7.356543	0.435196	-0.144362
39	1	0	-0.480526	-0.746144	0.743669

Structure 37b (B3LYP, Gas Phase)

Energy (Hartrees): = - 1087.4900643
No imaginary frequencies

Standard orientation:

Center Number	Atomic Number	Atomic Type	Coordinates (Angstroms) X	Y	Z
1	6	0	-1.559547	1.181331	-0.530253
2	6	0	-1.049227	0.022581	0.346965
3	6	0	-1.993416	-1.179368	0.208992
4	6	0	-3.441372	-0.769540	0.445936
5	6	0	-3.816948	0.392575	-0.481829
6	1	0	-1.492716	0.897896	-1.600250
7	1	0	-1.924394	-1.568051	-0.824230
8	1	0	-3.535769	-0.422699	1.486992
9	1	0	-3.734229	0.061471	-1.529901
10	1	0	-1.080261	0.373716	1.384323
11	8	0	-2.917299	1.474145	-0.223144
12	6	0	-5.242574	0.905763	-0.255522
13	1	0	-5.370683	1.146058	0.813120
14	1	0	-5.368983	1.836379	-0.817175
15	8	0	-6.213359	-0.006863	-0.724437
16	1	0	-5.998890	-0.868494	-0.333100
17	8	0	-4.323394	-1.861856	0.202482
18	1	0	-4.002368	-2.604498	0.734448
19	8	0	-1.678910	-2.202464	1.138000
20	1	0	-0.713310	-2.297022	1.120821
21	8	0	-0.802503	2.315963	-0.253862
22	1	0	-1.178813	3.047210	-0.764203
23	7	0	0.309996	-0.424663	0.068056
24	6	0	2.733053	0.021817	0.180537
25	6	0	3.776054	0.960303	0.468906
26	6	0	3.125018	-1.185878	-0.453755
27	6	0	5.103422	0.678563	0.197015
28	6	0	4.455903	-1.466048	-0.740151
29	1	0	2.372247	-1.938458	-0.668209
30	6	0	5.450749	-0.540805	-0.401407
31	1	0	5.867748	1.413851	0.419525
32	1	0	4.725680	-2.411650	-1.205104
33	6	0	1.393889	0.327821	0.527657
34	1	0	1.155406	1.080608	1.267908
35	8	0	3.483994	2.177556	1.021326
36	1	0	2.569234	2.400903	0.792811
37	8	0	6.779072	-0.757999	-0.644876
38	1	0	6.882084	-1.619062	-1.070622
39	1	0	0.427213	-0.758056	-0.886004

Structure 37b (B3LYP, DMSO)

Energy (Hartrees): = - 1086.9642467
No imaginary frequencies

Standard orientation:

Center Number	Atomic Number	Atomic Type	Coordinates (Angstroms) X	Y	Z
1	6	0	1.638147	1.480531	0.162459
2	6	0	0.962893	0.241829	-0.450724
3	6	0	1.730313	-1.016952	-0.004826
4	6	0	3.218252	-0.878143	-0.304400
5	6	0	3.767827	0.410477	0.320574
6	1	0	1.556554	1.444018	1.262495
7	1	0	1.605778	-1.134550	1.081813
8	1	0	3.353907	-0.818607	-1.395110
9	1	0	3.664807	0.351348	1.415876
10	1	0	1.034702	0.335682	-1.544217
11	8	0	3.021097	1.514103	-0.194296
12	6	0	5.238519	0.658644	-0.022501
13	1	0	5.353117	0.666822	-1.118848
14	1	0	5.523413	1.649964	0.345161
15	8	0	6.101870	-0.286422	0.592311
16	1	0	5.710289	-1.158854	0.418565
17	8	0	3.943805	-1.991691	0.222622
18	1	0	3.518839	-2.788264	-0.132091
19	8	0	1.285570	-2.189115	-0.682751
20	1	0	0.401733	-2.403041	-0.348334
21	8	0	1.032237	2.616388	-0.360665
22	1	0	1.391386	3.382742	0.112798
23	7	0	-0.416373	0.147167	-0.012103
24	6	0	-2.757174	0.030841	-0.514864
25	6	0	-3.149522	-0.028700	0.855092
26	6	0	-3.760073	0.002708	-1.501107
27	6	0	-4.503401	-0.113414	1.191502
28	6	0	-5.103424	-0.080964	-1.174008
29	1	0	-3.463395	0.049057	-2.545724
30	6	0	-5.469888	-0.139359	0.184210
31	1	0	-4.799752	-0.158149	2.233747

32	1	0	-5.866484	-0.101607	-1.947180
33	6	0	-1.360462	0.124521	-0.891348
34	1	0	-1.139526	0.180586	-1.964510
35	8	0	-2.237553	-0.003316	1.839814
36	1	0	-1.339175	0.061793	1.393915
37	8	0	-6.767896	-0.223621	0.574038
38	1	0	-7.336669	-0.231846	-0.212566

Structure 37b (B3LYP, H_2O)

Energy (Hartrees): = - 1087.5352492
No imaginary frequencies

Standard orientation:

Center Number	Atomic Number	Atomic Type	Coordinates (Angstroms) X	Y	Z
1	6	0	-1.490951	1.083861	-0.629308
2	6	0	-1.055141	-0.013198	0.357625
3	6	0	-2.046131	-1.186969	0.299737
4	6	0	-3.484831	-0.704564	0.456425
5	6	0	-3.787415	0.413030	-0.550223
6	1	0	-1.428692	0.715169	-1.665329
7	1	0	-1.958127	-1.672861	-0.685118
8	1	0	-3.614562	-0.305478	1.472238
9	1	0	-3.705420	0.009885	-1.571239
10	1	0	-1.084571	0.420960	1.362521
11	8	0	-2.843736	1.471524	-0.358568
12	6	0	-5.178529	1.023937	-0.367645
13	1	0	-5.280950	1.382601	0.667655
14	1	0	-5.274019	1.887919	-1.032086
15	8	0	-6.215727	0.108867	-0.709012
16	1	0	-6.002179	-0.719093	-0.243076
17	8	0	-4.412893	-1.771596	0.224286
18	1	0	-4.299011	-2.413812	0.940587
19	8	0	-1.794654	-2.131856	1.336157
20	1	0	-0.861932	-2.385732	1.255353
21	8	0	-0.685755	2.212564	-0.442607
22	1	0	-0.803656	2.791426	-1.210592
23	7	0	0.295692	-0.513840	0.121666
24	6	0	2.717623	-0.022579	0.199355
25	6	0	3.778436	0.790151	0.721508
26	6	0	3.105869	-1.022763	-0.739896
27	6	0	5.101863	0.581392	0.374056
28	6	0	4.434191	-1.231009	-1.095278
29	1	0	2.350215	-1.671667	-1.171675
30	6	0	5.438641	-0.439683	-0.528178
31	1	0	5.873142	1.223895	0.785711
32	1	0	4.698154	-2.014979	-1.800208
33	6	0	1.384804	0.181445	0.627559
34	1	0	1.162879	0.803552	1.485921
35	8	0	3.519025	1.818854	1.608721
36	1	0	2.623082	2.154988	1.450410
37	8	0	6.776227	-0.599657	-0.828604
38	1	0	6.867202	-1.319779	-1.470872
39	1	0	0.418125	-0.878010	-0.819263

Structure 37b (M06-2X, Gas Phase)

Energy (Hartrees): = - 1087.3591498
No imaginary frequencies

Standard orientation:

Center Number	Atomic Number	Atomic Type	Coordinates (Angstroms) X	Y	Z
1	6	0	-1.531171	1.168945	-0.478090
2	6	0	-1.044802	-0.003617	0.377126
3	6	0	-1.989214	-1.187902	0.207573
4	6	0	-3.422913	-0.762304	0.445384
5	6	0	-3.770817	0.406938	-0.469993
6	1	0	-1.456998	0.906458	-1.549771
7	1	0	-1.918250	-1.562493	-0.827886
8	1	0	-3.515625	-0.425697	1.487831
9	1	0	-3.669340	0.089170	-1.518962
10	1	0	-1.079867	0.326835	1.420406
11	8	0	-2.877201	1.471979	-0.180283
12	6	0	-5.192868	0.912363	-0.264721
13	1	0	-5.362730	1.077446	0.808334
14	1	0	-5.292673	1.872509	-0.773168
15	8	0	-6.138842	0.034849	-0.824364
16	1	0	-5.961566	-0.841395	-0.464343
17	8	0	-4.313444	-1.830826	0.186684

18	1	0	-3.997122	-2.592853	0.684000
19	8	0	-1.697456	-2.222586	1.118943
20	1	0	-0.740012	-2.337322	1.119930
21	8	0	-0.761540	2.280112	-0.173641
22	1	0	-1.129527	3.030192	-0.651352
23	7	0	0.307696	-0.446837	0.091039
24	6	0	2.710302	0.022869	0.184379
25	6	0	3.740637	0.974745	0.427119
26	6	0	3.095683	-1.199219	-0.404697
27	6	0	5.060675	0.693644	0.150272
28	6	0	4.421448	-1.480080	-0.696210
29	1	0	2.344554	-1.961240	-0.577868
30	6	0	5.407692	-0.539956	-0.404685
31	1	0	5.822131	1.439405	0.336779
32	1	0	4.694068	-2.437192	-1.128536
33	6	0	1.373607	0.329241	0.535149
34	1	0	1.127501	1.099416	1.252083
35	8	0	3.440302	2.198828	0.940139
36	1	0	2.537135	2.421927	0.687491
37	8	0	6.727606	-0.755026	-0.653999
38	1	0	6.837566	-1.627864	-1.039613
39	1	0	0.421921	-0.782093	-0.862090

Structure 37b (M06-2X, DMSO)

Energy (Hartrees): = - 1087.395472
No imaginary frequencies

Standard orientation:

Center Number	Atomic Number	Atomic Type	Coordinates (Angstroms) X	Y	Z
1	6	0	-1.574466	1.168066	-0.600117
2	6	0	-1.052432	0.086454	0.348879
3	6	0	-1.970778	-1.131762	0.288467
4	6	0	-3.413040	-0.725743	0.504583
5	6	0	-3.792479	0.361668	-0.495990
6	1	0	-1.519699	0.808384	-1.640472
7	1	0	-1.890507	-1.584532	-0.711703
8	1	0	-3.520468	-0.318043	1.519158
9	1	0	-3.680430	-0.033465	-1.516630
10	1	0	-1.068665	0.497303	1.363124
11	8	0	-2.928096	1.468795	-0.295447
12	6	0	-5.222512	0.849702	-0.317382
13	1	0	-5.383145	1.116858	0.735944
14	1	0	-5.360517	1.748609	-0.921329
15	8	0	-6.161769	-0.109581	-0.760910
16	1	0	-5.919850	-0.942366	-0.338232
17	8	0	-4.281088	-1.835027	0.324189
18	1	0	-3.962885	-2.544420	0.895319
19	8	0	-1.645823	-2.082428	1.282813
20	1	0	-0.708072	-2.286680	1.179359
21	8	0	-0.824201	2.314888	-0.415729
22	1	0	-1.060534	2.939949	-1.111015
23	7	0	0.300559	-0.343418	0.051514
24	6	0	2.711211	0.049437	0.172750
25	6	0	3.787023	0.892871	0.581256
26	6	0	3.060434	-1.088270	-0.594640
27	6	0	5.098317	0.592533	0.276856
28	6	0	4.379075	-1.387850	-0.907869
29	1	0	2.284638	-1.772777	-0.917948
30	6	0	5.403497	-0.555917	-0.461998
31	1	0	5.889394	1.260226	0.596599
32	1	0	4.615829	-2.276856	-1.484266
33	6	0	1.379808	0.357014	0.543987
34	1	0	1.151175	1.061354	1.332834
35	8	0	3.550517	2.027602	1.295157
36	1	0	2.657831	2.341605	1.100585
37	8	0	6.716893	-0.795319	-0.723774
38	1	0	6.791418	-1.606400	-1.241026
39	1	0	0.407147	-0.725499	-0.883987

Structure 37b (M06-2X, H_2O)

Energy (Hartrees): = - 1087.4083957
No imaginary frequencies

Standard orientation:

Center Number	Atomic Number	Atomic Type	Coordinates (Angstroms) X	Y	Z
1	6	0	-1.475032	1.076810	-0.586342
2	6	0	-1.051105	-0.018458	0.394273

3	6	0	-2.035646	-1.182815	0.316271
4	6	0	-3.461395	-0.692131	0.473061
5	6	0	-3.746242	0.415448	-0.535863
6	1	0	-1.411553	0.708658	-1.620298
7	1	0	-1.943733	-1.660876	-0.669656
8	1	0	-3.586939	-0.284590	1.484791
9	1	0	-3.637728	0.013894	-1.553340
10	1	0	-1.083817	0.407855	1.401734
11	8	0	-2.815760	1.468857	-0.322121
12	6	0	-5.139238	1.007763	-0.382935
13	1	0	-5.301591	1.286616	0.665401
14	1	0	-5.202594	1.909060	-0.994375
15	8	0	-6.142270	0.114259	-0.839908
16	1	0	-5.991971	-0.724598	-0.384436
17	8	0	-4.388406	-1.745058	0.246049
18	1	0	-4.215691	-2.433163	0.899672
19	8	0	-1.801873	-2.131141	1.342531
20	1	0	-0.891191	-2.435385	1.250271
21	8	0	-0.662814	2.191679	-0.394903
22	1	0	-0.771741	2.776399	-1.153801
23	7	0	0.293685	-0.512911	0.151006
24	6	0	2.696263	-0.015806	0.202930
25	6	0	3.751885	0.820734	0.672783
26	6	0	3.069905	-1.054844	-0.686214
27	6	0	5.065966	0.601722	0.320690
28	6	0	4.391592	-1.274401	-1.046417
29	1	0	2.313297	-1.726553	-1.074738
30	6	0	5.393953	-0.457141	-0.532222
31	1	0	5.839020	1.262737	0.694029
32	1	0	4.652806	-2.089247	-1.713214
33	6	0	1.369541	0.202041	0.636097
34	1	0	1.146658	0.856741	1.467566
35	8	0	3.491011	1.877804	1.512645
36	1	0	2.611153	2.228997	1.323186
37	8	0	6.721532	-0.627289	-0.836718
38	1	0	6.815963	-1.379830	-1.432891
39	1	0	0.409818	-0.892763	-0.784194

Structure 43 (B3LYP, Gas Phase)

Energy (Hartrees): = - 1547.1382015
No imaginary frequencies

Standard orientation:

Center Number	Atomic Number	Atomic Type	Coordinates (Angstroms) X	Y	Z
1	6	0	-0.456365	-1.276685	0.125797
2	6	0	0.504902	-0.188866	-0.370357
3	6	0	-0.008552	1.193628	0.083542
4	6	0	-1.511199	1.394563	-0.176687
5	6	0	-2.299407	0.167694	0.307280
6	1	0	-0.426620	-1.375902	1.218448
7	1	0	0.190429	1.333723	1.149895
8	1	0	-1.683602	1.580614	-1.240271
9	1	0	-2.198966	0.089998	1.401573
10	1	0	0.487444	-0.221736	-1.471761
11	8	0	-1.764621	-0.994616	-0.313280
12	6	0	-3.781832	0.212679	-0.035674
13	1	0	-4.232475	1.123795	0.362104
14	1	0	-3.934075	0.150566	-1.115675
15	8	0	-4.418622	-0.897693	0.611375
16	8	0	-1.991201	2.515233	0.593721
17	8	0	0.756740	2.159214	-0.663534
18	8	0	-0.074447	-2.498124	-0.476888
19	7	0	1.824059	-0.406331	0.185281
20	6	0	4.185910	-0.749013	-0.181163
21	6	0	4.515396	-0.812451	1.183374
22	6	0	5.200024	-0.893530	-1.138899
23	6	0	5.833827	-1.016764	1.574418
24	1	0	3.722319	-0.697328	1.914393
25	6	0	6.521658	-1.098850	-0.744840
26	1	0	4.948411	-0.844415	-2.195611
27	6	0	6.840008	-1.160447	0.612277
28	1	0	6.083206	-1.063962	2.630389
29	1	0	7.300128	-1.209812	-1.493704
30	6	0	2.800813	-0.531648	-0.625792
31	1	0	2.655263	-0.483088	-1.716519
32	6	0	-4.734659	-1.974401	-0.165973
33	6	0	-2.048479	3.737844	-0.005210
34	6	0	-0.575375	-3.639112	0.091314
35	6	0	1.154245	3.297565	-0.035121
36	8	0	-1.257257	-3.647365	1.088319
37	8	0	-4.722921	-1.964317	-1.374549
38	8	0	-1.789753	3.930273	-1.171877
39	8	0	0.922394	3.547749	1.126988
40	6	0	-0.152895	-4.845057	-0.706097

41	6	0	1.871650	4.202621	-1.002358
42	6	0	-2.433519	4.791981	0.998547
43	6	0	-5.068754	-3.159473	0.702388
44	1	0	-0.463906	-5.750701	-0.186890
45	1	0	-0.618643	-4.807755	-1.695727
46	1	0	0.930457	-4.842868	-0.852592
47	1	0	1.126971	4.644194	-1.672721
48	1	0	2.381421	4.993177	-0.452540
49	1	0	2.580100	3.639309	-1.613582
50	1	0	-2.732467	5.701135	0.477592
51	1	0	-3.233524	4.440252	1.653507
52	1	0	-1.556613	4.998208	1.621122
53	1	0	-5.594287	-3.909759	0.111953
54	1	0	-4.128773	-3.581855	1.074989
55	1	0	-5.666327	-2.858630	1.565695
56	1	0	7.868833	-1.319357	0.922163

Structure 43 (B3LYP, $CHCl_3$)

Energy (Hartrees): = - 1547.1690231
No imaginary frequencies

Standard orientation:

Center Number	Atomic Number	Atomic Type	Coordinates (Angstroms) X	Y	Z
1	6	0	-0.427229	-1.273180	0.164120
2	6	0	0.499459	-0.163805	-0.354981
3	6	0	-0.045503	1.190487	0.135496
4	6	0	-1.538350	1.359055	-0.165573
5	6	0	-2.317138	0.133281	0.339850
6	1	0	-0.373041	-1.361775	1.255600
7	1	0	0.122808	1.291181	1.210735
8	1	0	-1.696504	1.500070	-1.237392
9	1	0	-2.232419	0.077038	1.435584
10	1	0	0.458442	-0.186468	-1.455330
11	8	0	-1.755818	-1.036903	-0.252968
12	6	0	-3.788232	0.170499	-0.042786
13	1	0	-4.254627	1.076951	0.347951
14	1	0	-3.911808	0.122676	-1.126348
15	8	0	-4.455211	-0.943833	0.576354
16	8	0	-2.020984	2.516013	0.546512
17	8	0	0.688169	2.224075	-0.547989
18	8	0	-0.023312	-2.488558	-0.438738
19	7	0	1.837056	-0.357142	0.169285
20	6	0	4.195744	-0.657079	-0.261728
21	6	0	4.573488	-0.690885	1.092094
22	6	0	5.179266	-0.805360	-1.251842
23	6	0	5.907821	-0.869952	1.441602
24	1	0	3.809394	-0.573363	1.853458
25	6	0	6.516609	-0.986336	-0.899121
26	1	0	4.890846	-0.778714	-2.299772
27	6	0	6.882590	-1.018655	0.447634
28	1	0	6.193301	-0.893564	2.489350
29	1	0	7.270558	-1.100354	-1.672466
30	6	0	2.794723	-0.467481	-0.669019
31	1	0	2.619929	-0.426593	-1.754248
32	6	0	-4.804902	-1.991687	-0.214117
33	6	0	-2.272898	3.655956	-0.156160
34	6	0	-0.369575	-3.645579	0.205035
35	6	0	1.243784	3.225304	0.186949
36	8	0	-0.969732	-3.669006	1.255660
37	8	0	-4.729817	-1.985037	-1.424421
38	8	0	-2.170349	3.743165	-1.360591
39	8	0	1.159472	3.308566	1.393475
40	6	0	0.100336	-4.839395	-0.575873
41	6	0	1.954749	4.197625	-0.714363
42	6	0	-2.674677	4.763614	0.778263
43	6	0	-5.282225	-3.145291	0.625469
44	1	0	-0.099885	-5.749646	-0.011000
45	1	0	-0.425618	-4.878428	-1.535479
46	1	0	1.169827	-4.754976	-0.789014
47	1	0	1.244585	4.624168	-1.429672
48	1	0	2.404127	4.991276	-0.117792
49	1	0	2.728765	3.679813	-1.288824
50	1	0	-2.968220	5.642375	0.204242
51	1	0	-3.498024	4.441728	1.422192
52	1	0	-1.829005	5.012021	1.427903
53	1	0	-5.766292	-3.888146	-0.008713
54	1	0	-4.417417	-3.600306	1.121041
55	1	0	-5.969118	-2.803643	1.404036
56	1	0	7.923614	-1.157999	0.724917

Structure 43 (M06-2X, Gas Phase)

Energy (Hartrees): = - 1546.916388
No imaginary frequencies

Standard orientation:

Center Number	Atomic Number	Atomic Type	Coordinates (Angstroms) X	Y	Z
1	6	0	-0.536191	-1.263086	0.166391
2	6	0	0.468150	-0.238715	-0.342506
3	6	0	0.034990	1.160894	0.107518
4	6	0	-1.443068	1.433738	-0.184480
5	6	0	-2.287449	0.263158	0.313104
6	1	0	-0.516972	-1.338987	1.260469
7	1	0	0.216744	1.284185	1.179099
8	1	0	-1.587953	1.598188	-1.255808
9	1	0	-2.198369	0.195600	1.407610
10	1	0	0.443715	-0.277031	-1.441402
11	8	0	-1.817349	-0.926152	-0.282276
12	6	0	-3.755220	0.377509	-0.044184
13	1	0	-4.175301	1.293897	0.366857
14	1	0	-3.884651	0.341750	-1.127312
15	8	0	-4.432753	-0.713859	0.568391
16	8	0	-1.889657	2.576073	0.550544
17	8	0	0.868652	2.064646	-0.619303
18	8	0	-0.214817	-2.502883	-0.408306
19	7	0	1.774300	-0.517760	0.204915
20	6	0	4.121650	-0.879057	-0.176558
21	6	0	4.440667	-1.008671	1.177756
22	6	0	5.125918	-0.999431	-1.136227
23	6	0	5.749414	-1.255995	1.559970
24	1	0	3.647485	-0.909449	1.908739
25	6	0	6.438425	-1.248586	-0.751583
26	1	0	4.876665	-0.898317	-2.187620
27	6	0	6.750293	-1.376909	0.596398
28	1	0	5.995365	-1.355394	2.610359
29	1	0	7.214533	-1.343697	-1.501341
30	6	0	2.737726	-0.616013	-0.610028
31	1	0	2.586987	-0.508203	-1.693121
32	6	0	-4.635149	-1.820825	-0.192989
33	6	0	-1.756177	3.796140	-0.019380
34	6	0	-0.886891	-3.573353	0.093038
35	6	0	1.226350	3.223915	-0.025939
36	8	0	-1.630490	-3.500426	1.027363
37	8	0	-4.497194	-1.845061	-1.380631
38	8	0	-1.379753	3.960481	-1.145944
39	8	0	0.920302	3.516894	1.096784
40	6	0	-0.565602	-4.809366	-0.692838
41	6	0	1.994581	4.086282	-0.980839
42	6	0	-2.093659	4.874889	0.963795
43	6	0	-5.035481	-2.979846	0.671819
44	1	0	-0.988910	-5.675813	-0.192358
45	1	0	-0.997265	-4.706349	-1.690130
46	1	0	0.513947	-4.911010	-0.802855
47	1	0	1.273608	4.494654	-1.693290
48	1	0	2.475384	4.893881	-0.435266
49	1	0	2.724497	3.494492	-1.531274
50	1	0	-2.244284	5.813637	0.437366
51	1	0	-2.971024	4.605208	1.549818
52	1	0	-1.239958	4.960137	1.640771
53	1	0	-5.480577	-3.754653	0.052682
54	1	0	-4.128167	-3.362569	1.146186
55	1	0	-5.723210	-2.656539	1.451825
56	1	0	7.772432	-1.571026	0.899206

Structure 43 (M06-2X, $CHCl_3$)

Energy (Hartrees): = - 1546.9487444
No imaginary frequencies

Standard orientation:

Center Number	Atomic Number	Atomic Type	Coordinates (Angstroms) X	Y	Z
1	6	0	-0.532181	-1.269023	0.181379
2	6	0	0.457781	-0.239821	-0.346894
3	6	0	0.014258	1.152332	0.112890
4	6	0	-1.463112	1.413687	-0.181952
5	6	0	-2.301668	0.238550	0.319798
6	1	0	-0.507929	-1.323211	1.275933
7	1	0	0.189517	1.262375	1.186570
8	1	0	-1.613095	1.570655	-1.253303
9	1	0	-2.215794	0.175003	1.413835

10	1	0	0.425368	-0.280416	-1.445008
11	8	0	-1.821922	-0.954640	-0.268260
12	6	0	-3.763731	0.359050	-0.053731
13	1	0	-4.193574	1.261711	0.377436
14	1	0	-3.884106	0.360456	-1.137703
15	8	0	-4.455055	-0.750842	0.519232
16	8	0	-1.916260	2.556842	0.550189
17	8	0	0.844490	2.072614	-0.599543
18	8	0	-0.191065	-2.513590	-0.372595
19	7	0	1.773232	-0.504659	0.190897
20	6	0	4.122514	-0.839645	-0.208869
21	6	0	4.467064	-0.921180	1.143972
22	6	0	5.112173	-0.982159	-1.182503
23	6	0	5.785697	-1.141003	1.511776
24	1	0	3.691034	-0.806666	1.891605
25	6	0	6.434182	-1.205088	-0.811965
26	1	0	4.842676	-0.918365	-2.232025
27	6	0	6.771449	-1.284193	0.534617
28	1	0	6.050761	-1.200881	2.561022
29	1	0	7.198462	-1.316645	-1.572188
30	6	0	2.730662	-0.601028	-0.633671
31	1	0	2.570824	-0.508561	-1.715585
32	6	0	-4.682777	-1.822664	-0.271726
33	6	0	-1.810034	3.775537	-0.022677
34	6	0	-0.776410	-3.602207	0.192890
35	6	0	1.219314	3.212777	0.015311
36	8	0	-1.521310	-3.527288	1.129080
37	8	0	-4.525766	-1.819443	-1.461393
38	8	0	-1.424420	3.943179	-1.148267
39	8	0	0.895932	3.495946	1.138342
40	6	0	-0.354285	-4.852991	-0.510475
41	6	0	2.047346	4.059731	-0.899158
42	6	0	-2.202699	4.849321	0.941995
43	6	0	-5.147738	-2.990404	0.544363
44	1	0	-0.793558	-5.715734	-0.016129
45	1	0	-0.684105	-4.806612	-1.550146
46	1	0	0.734537	-4.923908	-0.506664
47	1	0	1.393130	4.448512	-1.683235
48	1	0	2.483227	4.883777	-0.339702
49	1	0	2.824903	3.458666	-1.371020
50	1	0	-2.317773	5.793042	0.414282
51	1	0	-3.121450	4.581006	1.463375
52	1	0	-1.403922	4.935178	1.683026
53	1	0	-5.573944	-3.746997	-0.110319
54	1	0	-4.279675	-3.402909	1.065390
55	1	0	-5.873248	-2.670244	1.291886
56	1	0	7.801310	-1.456306	0.825882

Structure 44a (B3LYP, Gas Phase)

Energy (Hartrees): = - 1661.666626
No imaginary frequencies

Standard orientation:

Center Number	Atomic Number	Atomic Type	Coordinates (Angstroms) X	Y	Z
1	6	0	-0.432402	1.872855	-0.406913
2	6	0	0.079207	0.535745	0.150147
3	6	0	-0.790708	-0.600340	-0.420401
4	6	0	-2.279763	-0.307325	-0.233174
5	6	0	-2.619911	1.084796	-0.788531
6	1	0	-0.251135	1.947259	-1.486401
7	1	0	-0.566748	-0.751613	-1.479989
8	1	0	-2.540776	-0.356357	0.825060
9	1	0	-2.409171	1.103977	-1.871398
10	1	0	-0.055338	0.563148	1.244203
11	8	0	-1.806453	2.046533	-0.121931
12	6	0	-4.080530	1.474392	-0.616824
13	1	0	-4.186859	2.542938	-0.820710
14	1	0	-4.708023	0.901753	-1.300569
15	8	0	-4.519283	1.243046	0.734881
16	8	0	-3.057107	-1.276222	-0.959489
17	8	0	-0.454647	-1.800862	0.296586
18	8	0	0.253513	2.910995	0.264048
19	7	0	1.455630	0.336006	-0.247892
20	6	0	3.736906	-0.111338	0.403587
21	6	0	4.249185	-0.136839	-0.909356
22	6	0	4.620172	-0.318484	1.468935
23	6	0	5.594074	-0.360526	-1.137450
24	1	0	3.564970	0.020546	-1.736179
25	6	0	5.979909	-0.544758	1.254726
26	1	0	4.240758	-0.303246	2.487939
27	6	0	6.471823	-0.566521	-0.056233
28	1	0	6.001678	-0.384223	-2.142597
29	1	0	6.636990	-0.701364	2.101379
30	6	0	2.315281	0.120832	0.672173

31	1	0	2.025819	0.098002	1.735342
32	8	0	7.773192	-0.777971	-0.390505
33	6	0	8.713964	-0.994585	0.652093
34	1	0	8.467192	-1.888409	1.238381
35	1	0	9.676865	-1.140721	0.161433
36	1	0	8.777540	-0.129383	1.323593
37	6	0	-5.589783	0.420989	0.909407
38	6	0	-3.771347	-2.190501	-0.242896
39	6	0	0.400941	4.093005	-0.414070
40	6	0	-0.200082	-2.929806	-0.423830
41	8	0	0.017501	4.272111	-1.543917
42	8	0	-6.219410	-0.073944	-0.000970
43	8	0	-3.743746	-2.272154	0.965570
44	8	0	-0.227400	-2.989545	-1.630671
45	6	0	1.105460	5.101895	0.455448
46	6	0	0.101507	-4.075594	0.509856
47	6	0	-4.614349	-3.034541	-1.160131
48	6	0	-5.845467	0.178616	2.373042
49	1	0	1.267911	6.018041	-0.110823
50	1	0	0.498187	5.313286	1.340679
51	1	0	2.060143	4.696958	0.802462
52	1	0	-0.760774	-4.254406	1.159067
53	1	0	0.325546	-4.969049	-0.071600
54	1	0	0.949145	-3.823167	1.153006
55	1	0	-4.908841	-3.948501	-0.644589
56	1	0	-5.513855	-2.461507	-1.408873
57	1	0	-4.081711	-3.263099	-2.085079
58	1	0	-6.898495	-0.061166	2.523002
59	1	0	-5.243821	-0.686052	2.673154
60	1	0	-5.550411	1.036609	2.978901

Structure 44a (B3LYP, $CHCl_3$)

Energy (Hartrees): = - 1661.6980201
No imaginary frequencies

Standard orientation:

Center Number	Atomic Number	Atomic Type	Coordinates (Angstroms) X	Y	Z
1	6	0	-0.451278	1.820777	-0.452774
2	6	0	0.067238	0.497237	0.131451
3	6	0	-0.794912	-0.649236	-0.428246
4	6	0	-2.285842	-0.366162	-0.245457
5	6	0	-2.636095	1.008561	-0.838078
6	1	0	-0.258330	1.878514	-1.530521
7	1	0	-0.569809	-0.804390	-1.486276
8	1	0	-2.548944	-0.390871	0.812995
9	1	0	-2.421174	1.006532	-1.918665
10	1	0	-0.069638	0.540177	1.223703
11	8	0	-1.829759	1.990879	-0.184367
12	6	0	-4.095694	1.396021	-0.681125
13	1	0	-4.223052	2.442442	-0.971258
14	1	0	-4.723803	0.767957	-1.313461
15	8	0	-4.511036	1.268409	0.693946
16	8	0	-3.039472	-1.372674	-0.948378
17	8	0	-0.458056	-1.847260	0.297032
18	8	0	0.219144	2.875766	0.212880
19	7	0	1.448202	0.301584	-0.261009
20	6	0	3.737027	-0.086532	0.406530
21	6	0	4.260524	-0.124005	-0.902455
22	6	0	4.618304	-0.259733	1.481943
23	6	0	5.611421	-0.325698	-1.117653
24	1	0	3.586054	0.006744	-1.742243
25	6	0	5.983095	-0.465055	1.281069
26	1	0	4.231350	-0.234934	2.497653
27	6	0	6.486076	-0.497578	-0.026599
28	1	0	6.023530	-0.355639	-2.121324
29	1	0	6.637176	-0.598241	2.134133
30	6	0	2.311151	0.126388	0.667436
31	1	0	2.021709	0.129079	1.729155
32	8	0	7.791228	-0.688373	-0.346937
33	6	0	8.735030	-0.867814	0.708699
34	1	0	8.505872	-1.758350	1.305406
35	1	0	9.703180	-1.000501	0.223445
36	1	0	8.777269	0.010790	1.363019
37	6	0	-5.622179	0.531105	0.950310
38	6	0	-3.778435	-2.254340	-0.218771
39	6	0	0.344773	4.058773	-0.463576
40	6	0	-0.118367	-2.959719	-0.408776
41	8	0	-0.055274	4.226907	-1.592991
42	8	0	-6.299989	0.008280	0.089092
43	8	0	-3.843074	-2.241394	0.992377
44	8	0	-0.080661	-3.011686	-1.619065
45	6	0	1.039050	5.079490	0.392411

46	6	0	0.197234	-4.093197	0.528922
47	6	0	-4.500387	-3.210434	-1.126654
48	6	0	-5.871826	0.439732	2.430092
49	1	0	1.176509	6.000489	-0.173660
50	1	0	0.440115	5.280157	1.286435
51	1	0	2.007891	4.695135	0.724994
52	1	0	-0.662469	-4.292969	1.175773
53	1	0	0.448043	-4.985581	-0.044198
54	1	0	1.037573	-3.818952	1.174177
55	1	0	-4.951201	-4.007565	-0.535690
56	1	0	-5.287022	-2.669704	-1.663454
57	1	0	-3.814720	-3.626523	-1.869524
58	1	0	-6.892899	0.102528	2.609872
59	1	0	-5.175773	-0.290395	2.856905
60	1	0	-5.694709	1.400665	2.918765

Structure 44a (M06-2X, Gas Phase)

Energy (Hartrees): = - 1661.4315983
No imaginary frequencies

Standard orientation:

Center Number	Atomic Number	Atomic Type	Coordinates (Angstroms) X	Y	Z
1	6	0	-0.422229	2.001138	-0.332056
2	6	0	0.090147	0.660414	0.178425
3	6	0	-0.763912	-0.463703	-0.412566
4	6	0	-2.248699	-0.167505	-0.243730
5	6	0	-2.586466	1.242900	-0.720208
6	1	0	-0.270709	2.095307	-1.414109
7	1	0	-0.527512	-0.612588	-1.469849
8	1	0	-2.511968	-0.272151	0.810588
9	1	0	-2.385778	1.322609	-1.799568
10	1	0	-0.036062	0.653315	1.271125
11	8	0	-1.780573	2.165568	-0.014353
12	6	0	-4.050112	1.581969	-0.494897
13	1	0	-4.170788	2.663608	-0.542741
14	1	0	-4.667032	1.091570	-1.246063
15	8	0	-4.464710	1.164995	0.808305
16	8	0	-3.036469	-1.053883	-1.034226
17	8	0	-0.413960	-1.637214	0.325251
18	8	0	0.284676	3.007948	0.339450
19	7	0	1.460587	0.475318	-0.233725
20	6	0	3.705094	-0.148456	0.359927
21	6	0	4.219220	-0.005810	-0.937241
22	6	0	4.559926	-0.546918	1.380826
23	6	0	5.548597	-0.257562	-1.192657
24	1	0	3.548114	0.303851	-1.729098
25	6	0	5.906610	-0.805489	1.138615
26	1	0	4.172101	-0.660320	2.388222
27	6	0	6.402846	-0.661454	-0.155818
28	1	0	5.967946	-0.155633	-2.185805
29	1	0	6.546044	-1.113966	1.953846
30	6	0	2.289057	0.108734	0.652078
31	1	0	1.981575	-0.039994	1.696900
32	8	0	7.687789	-0.887630	-0.511716
33	6	0	8.588246	-1.302386	0.495337
34	1	0	8.272511	-2.251182	0.940414
35	1	0	9.548833	-1.435036	0.003657
36	1	0	8.681942	-0.543842	1.278843
37	6	0	-5.319093	0.120927	0.890118
38	6	0	-3.537321	-2.158094	-0.438823
39	6	0	0.298915	4.231205	-0.259266
40	6	0	-0.539134	-2.838620	-0.281799
41	8	0	-0.221628	4.453500	-1.310612
42	8	0	-5.832730	-0.399203	-0.062286
43	8	0	-3.350328	-2.419799	0.720122
44	8	0	-0.841563	-2.974731	-1.433494
45	6	0	1.055257	5.212886	0.586676
46	6	0	-0.294546	-3.944993	0.700941
47	6	0	-4.325669	-2.969975	-1.415727
48	6	0	-5.490416	-0.325993	2.311610
49	1	0	1.098071	6.170806	0.076012
50	1	0	0.553606	5.317482	1.549668
51	1	0	2.059502	4.831276	0.774109
52	1	0	-1.171038	-3.995596	1.351330
53	1	0	-0.172009	-4.884187	0.168272
54	1	0	0.577519	-3.724488	1.315175
55	1	0	-4.688125	-3.870000	-0.926881
56	1	0	-5.162290	-2.364976	-1.767765
57	1	0	-3.683569	-3.219483	-2.261403
58	1	0	-6.469047	-0.786255	2.427272
59	1	0	-4.723676	-1.083387	2.498140
60	1	0	-5.354203	0.500343	3.005655

Structure 44a (M06-2X, $CHCl_3$)

Energy (Hartrees): = - 1661.4644253
No imaginary frequencies

Standard orientation:

Center Number	Atomic Number	Atomic Type	Coordinates (Angstroms) X	Y	Z
1	6	0	-0.439079	1.974879	-0.361818
2	6	0	0.076318	0.639574	0.161147
3	6	0	-0.772679	-0.488306	-0.430578
4	6	0	-2.258459	-0.198805	-0.260847
5	6	0	-2.600212	1.202355	-0.764198
6	1	0	-0.275536	2.062818	-1.441745
7	1	0	-0.536692	-0.630335	-1.488307
8	1	0	-2.518938	-0.285468	0.795899
9	1	0	-2.390119	1.270239	-1.841385
10	1	0	-0.050177	0.636194	1.253037
11	8	0	-1.801204	2.136867	-0.058260
12	6	0	-4.062314	1.546033	-0.554279
13	1	0	-4.196372	2.620734	-0.672628
14	1	0	-4.679672	1.009713	-1.272155
15	8	0	-4.468182	1.213940	0.778554
16	8	0	-3.040758	-1.108272	-1.032902
17	8	0	-0.415725	-1.664420	0.302834
18	8	0	0.257329	2.989248	0.314836
19	7	0	1.451394	0.463340	-0.248216
20	6	0	3.700091	-0.143112	0.353280
21	6	0	4.238023	0.056008	-0.927653
22	6	0	4.541802	-0.573940	1.374546
23	6	0	5.575756	-0.170713	-1.167109
24	1	0	3.585948	0.390885	-1.725598
25	6	0	5.894829	-0.810667	1.147981
26	1	0	4.136906	-0.730131	2.369354
27	6	0	6.415864	-0.606822	-0.129983
28	1	0	6.008463	-0.021795	-2.149143
29	1	0	6.521766	-1.147515	1.962074
30	6	0	2.279116	0.092798	0.639302
31	1	0	1.969437	-0.071122	1.679592
32	8	0	7.708806	-0.801265	-0.466414
33	6	0	8.597795	-1.254469	0.543412
34	1	0	8.286722	-2.227494	0.934088
35	1	0	9.569402	-1.352201	0.064220
36	1	0	8.667482	-0.531550	1.361184
37	6	0	-5.309147	0.172072	0.943763
38	6	0	-3.543364	-2.197221	-0.413807
39	6	0	0.261840	4.215644	-0.272303
40	6	0	-0.501985	-2.862171	-0.312110
41	8	0	-0.277552	4.438829	-1.318258
42	8	0	-5.807805	-0.433825	0.031814
43	8	0	-3.349729	-2.438834	0.749206
44	8	0	-0.820914	-2.995081	-1.463033
45	6	0	1.021030	5.196101	0.564524
46	6	0	-0.173703	-3.968753	0.641698
47	6	0	-4.327136	-3.036937	-1.369628
48	6	0	-5.501525	-0.139343	2.395988
49	1	0	1.064513	6.154727	0.054047
50	1	0	0.521340	5.305362	1.528852
51	1	0	2.026640	4.815445	0.749479
52	1	0	-0.960256	-4.010621	1.398513
53	1	0	-0.124256	-4.912080	0.103553
54	1	0	0.771557	-3.763544	1.145326
55	1	0	-4.819614	-3.841113	-0.828648
56	1	0	-5.060186	-2.415476	-1.884144
57	1	0	-3.639135	-3.448201	-2.111748
58	1	0	-6.400968	-0.737373	2.522521
59	1	0	-4.637677	-0.720691	2.729907
60	1	0	-5.551652	0.774491	2.986358

Structure 46 (B3LYP, Gas Phase)

Energy (Hartrees): = - 1547.1380701
No imaginary frequencies

Standard orientation:

Center Number	Atomic Number	Atomic Type	Coordinates (Angstroms) X	Y	Z
1	6	0	-0.229850	-1.576767	-0.519242

2	6	0	0.637595	-0.319668	-0.705104
3	6	0	0.020469	0.859939	0.063200
4	6	0	-1.468326	1.025477	-0.274704
5	6	0	-2.220485	-0.313855	-0.162443
6	1	0	0.145078	0.724899	1.139610
7	1	0	-1.566987	1.453459	-1.276970
8	1	0	-2.267604	-0.601736	0.894485
9	1	0	0.598206	-0.076510	-1.780546
10	8	0	-1.550449	-1.332228	-0.913370
11	6	0	-3.629602	-0.211365	-0.713350
12	1	0	-4.135184	0.670609	-0.312590
13	1	0	-3.612569	-0.137762	-1.804893
14	8	0	-4.337625	-1.398400	-0.316132
15	8	0	-2.092368	1.906461	0.681625
16	8	0	0.747470	2.031657	-0.356217
17	7	0	1.988721	-0.561043	-0.244784
18	6	0	4.360335	-0.479085	-0.701677
19	6	0	4.749849	-0.951558	0.562909
20	6	0	5.344642	-0.174223	-1.652880
21	6	0	6.097598	-1.114458	0.862480
22	1	0	3.978885	-1.182548	1.290006
23	6	0	6.695860	-0.337988	-1.350697
24	1	0	5.046379	0.191446	-2.632497
25	6	0	7.073746	-0.808352	-0.092612
26	1	0	6.393569	-1.480175	1.841313
27	1	0	7.450799	-0.099980	-2.093954
28	6	0	2.943359	-0.295921	-1.049533
29	1	0	2.752927	0.090932	-2.063703
30	6	0	-5.625598	-1.475756	-0.731097
31	6	0	-2.202424	3.229021	0.371493
32	6	0	1.013042	2.994653	0.565760
33	8	0	-6.170279	-0.608470	-1.377286
34	8	0	-1.866989	3.703374	-0.690335
35	8	0	0.687360	2.927802	1.730391
36	6	0	1.720895	4.155448	-0.083624
37	6	0	-2.755492	3.993993	1.544643
38	6	0	-6.255543	-2.771503	-0.281617
39	1	0	0.996912	4.695795	-0.702161
40	1	0	2.112898	4.820049	0.685596
41	1	0	2.524147	3.806422	-0.736366
42	1	0	-3.069482	4.985176	1.218794
43	1	0	-3.587334	3.457977	2.006773
44	1	0	-1.960527	4.087792	2.291735
45	1	0	-7.300451	-2.791488	-0.588735
46	1	0	-5.720943	-3.617284	-0.724003
47	1	0	-6.181115	-2.871171	0.804936
48	8	0	-0.188336	-1.964888	0.861142
49	6	0	0.158365	-3.255026	1.152313
50	8	0	0.434704	-4.084833	0.319877
51	1	0	0.131331	-2.404139	-1.129766
52	6	0	0.150766	-3.468444	2.644208
53	1	0	-0.839652	-3.243116	3.049989
54	1	0	0.419648	-4.500654	2.864800
55	1	0	0.861373	-2.784952	3.117792
56	1	0	8.125535	-0.936905	0.145723

Structure 46 (B3LYP, $CHCl_3$)

Energy (Hartrees): = - 1547.169279
No imaginary frequencies

Standard orientation:

Center Number	Atomic Number	Atomic Type	Coordinates (Angstroms) X	Y	Z
1	6	0	-0.183667	-1.522469	-0.519476
2	6	0	0.665312	-0.249154	-0.691818
3	6	0	0.017731	0.900371	0.093533
4	6	0	-1.456298	1.056731	-0.288815
5	6	0	-2.198870	-0.284214	-0.151039
6	1	0	0.111336	0.729519	1.167433
7	1	0	-1.538969	1.443023	-1.307945
8	1	0	-2.237054	-0.564144	0.908498
9	1	0	0.625140	0.000070	-1.764419
10	8	0	-1.514297	-1.300548	-0.899425
11	6	0	-3.609608	-0.205009	-0.698735
12	1	0	-4.134237	0.656957	-0.279844
13	1	0	-3.596644	-0.114131	-1.788934
14	8	0	-4.290313	-1.417263	-0.318783
15	8	0	-2.073518	1.986402	0.624226
16	8	0	0.715034	2.113564	-0.250706
17	7	0	2.020195	-0.474447	-0.228450
18	6	0	4.386708	-0.467164	-0.722250
19	6	0	4.790669	-0.874347	0.561498
20	6	0	5.359129	-0.250668	-1.710762

21	6	0	6.140057	-1.058627	0.843852
22	1	0	4.034589	-1.040165	1.321883
23	6	0	6.711718	-0.436720	-1.425565
24	1	0	5.049938	0.062918	-2.704784
25	6	0	7.103736	-0.841573	-0.148397
26	1	0	6.446408	-1.372618	1.837527
27	1	0	7.456760	-0.268198	-2.197531
28	6	0	2.968408	-0.273201	-1.061332
29	1	0	2.771441	0.053574	-2.093717
30	6	0	-5.595321	-1.495510	-0.664274
31	6	0	-2.359650	3.243090	0.181521
32	6	0	1.153497	2.917813	0.754717
33	8	0	-6.181863	-0.606519	-1.248310
34	8	0	-2.168350	3.618719	-0.954637
35	8	0	0.982506	2.686259	1.932448
36	6	0	1.866174	4.115874	0.188988
37	6	0	-2.931631	4.069989	1.299717
38	6	0	-6.190156	-2.810726	-0.238114
39	1	0	1.195951	4.665541	-0.478988
40	1	0	2.198810	4.763736	0.999837
41	1	0	2.727007	3.790944	-0.403348
42	1	0	-3.220565	5.050322	0.921403
43	1	0	-3.798248	3.567264	1.738981
44	1	0	-2.182845	4.182590	2.090276
45	1	0	-7.257952	-2.818593	-0.456527
46	1	0	-5.699164	-3.629063	-0.774609
47	1	0	-6.025114	-2.974575	0.830764
48	8	0	-0.119314	-1.929277	0.856281
49	6	0	0.038327	-3.258057	1.127526
50	8	0	0.153056	-4.108080	0.272855
51	1	0	0.185639	-2.330037	-1.151132
52	6	0	0.056117	-3.490321	2.612078
53	1	0	-0.895920	-3.172471	3.048595
54	1	0	0.222778	-4.547545	2.817319
55	1	0	0.845353	-2.889074	3.073168
56	1	0	8.156406	-0.987422	0.076541

Structure 46 (M06-2X, Gas Phase)

Energy (Hartrees): = - 1546.9156523
No imaginary frequencies

Standard orientation:

Center Number	Atomic Number	Atomic Type	Coordinates (Angstroms) X	Y	Z
1	6	0	-0.303719	-1.631018	-0.617929
2	6	0	0.536959	-0.366477	-0.818099
3	6	0	-0.064216	0.783155	-0.010700
4	6	0	-1.554497	0.934536	-0.302313
5	6	0	-2.271747	-0.409886	-0.154233
6	1	0	0.085859	0.617570	1.058328
7	1	0	-1.693068	1.351331	-1.304724
8	1	0	-2.260866	-0.702520	0.900850
9	1	0	0.491314	-0.098499	-1.883775
10	8	0	-1.634086	-1.406589	-0.941101
11	6	0	-3.699092	-0.314689	-0.633335
12	1	0	-4.192019	0.557427	-0.200037
13	1	0	-3.728166	-0.235960	-1.723058
14	8	0	-4.366317	-1.504870	-0.217028
15	8	0	-2.160735	1.794790	0.666058
16	8	0	0.647417	1.951972	-0.422886
17	7	0	1.884028	-0.614345	-0.361731
18	6	0	4.249499	-0.383868	-0.699910
19	6	0	4.598681	-1.255167	0.335183
20	6	0	5.246632	0.297824	-1.396339
21	6	0	5.931735	-1.428345	0.671758
22	1	0	3.810440	-1.796297	0.845223
23	6	0	6.582882	0.128171	-1.052750
24	1	0	4.972759	0.964146	-2.208054
25	6	0	6.925024	-0.733964	-0.017990
26	1	0	6.203479	-2.108560	1.469908
27	1	0	7.354711	0.662957	-1.592898
28	6	0	2.838063	-0.180645	-1.071721
29	1	0	2.659018	0.371954	-2.004554
30	6	0	-5.660934	-1.592543	-0.575802
31	6	0	-2.179667	3.125290	0.419881
32	6	0	0.842579	2.936324	0.480620
33	8	0	-6.230469	-0.730223	-1.183166
34	8	0	-1.800388	3.612041	-0.608450
35	8	0	0.480378	2.870735	1.622572
36	6	0	1.515591	4.111336	-0.162403
37	6	0	-2.686745	3.874225	1.614310
38	6	0	-6.257976	-2.894207	-0.119847
39	1	0	0.777965	4.590785	-0.810281

40	1	0	1.845378	4.805472	0.605909
41	1	0	2.352459	3.782941	-0.777860
42	1	0	-2.957443	4.884994	1.320483
43	1	0	-3.529830	3.356062	2.068569
44	1	0	-1.869203	3.903551	2.338853
45	1	0	-7.317149	-2.908165	-0.361853
46	1	0	-5.745441	-3.717549	-0.619566
47	1	0	-6.107021	-3.012858	0.953386
48	8	0	-0.210864	-2.012242	0.752379
49	6	0	0.700639	-2.965288	1.075384
50	8	0	1.300428	-3.612604	0.269234
51	1	0	0.052050	-2.445724	-1.244998
52	6	0	0.842866	-3.060859	2.566185
53	1	0	-0.138287	-3.098521	3.038711
54	1	0	1.429775	-3.939956	2.818626
55	1	0	1.350810	-2.159228	2.915644
56	1	0	7.966031	-0.872439	0.248704

Structure 46 (M06-2X, $CHCl_3$)

Energy (Hartrees): = - 1546.9485496
No imaginary frequencies

Standard orientation:

Center Number	Atomic Number	Atomic Type	Coordinates (Angstroms) X	Y	Z
1	6	0	-0.302142	-1.625923	-0.606171
2	6	0	0.543415	-0.364377	-0.799654
3	6	0	-0.058612	0.786408	0.006111
4	6	0	-1.543931	0.946251	-0.301108
5	6	0	-2.269463	-0.392718	-0.144940
6	1	0	0.079391	0.614890	1.075621
7	1	0	-1.675087	1.346706	-1.310267
8	1	0	-2.262052	-0.681418	0.911171
9	1	0	0.504745	-0.100202	-1.865520
10	8	0	-1.633445	-1.397235	-0.927353
11	6	0	-3.693491	-0.289673	-0.631755
12	1	0	-4.189661	0.572027	-0.182251
13	1	0	-3.714246	-0.190067	-1.719685
14	8	0	-4.363298	-1.491665	-0.243213
15	8	0	-2.151879	1.822050	0.653470
16	8	0	0.666096	1.954038	-0.391027
17	7	0	1.890491	-0.611524	-0.335531
18	6	0	4.257720	-0.395845	-0.697446
19	6	0	4.622943	-1.137832	0.430373
20	6	0	5.246703	0.182117	-1.494155
21	6	0	5.962409	-1.290704	0.754586
22	1	0	3.846681	-1.594516	1.033249
23	6	0	6.589880	0.031556	-1.164859
24	1	0	4.959479	0.751980	-2.372201
25	6	0	6.947623	-0.704308	-0.040573
26	1	0	6.244373	-1.869611	1.626232
27	1	0	7.354086	0.484875	-1.785006
28	6	0	2.843223	-0.212961	-1.070035
29	1	0	2.663269	0.293409	-2.026889
30	6	0	-5.658377	-1.573125	-0.590062
31	6	0	-2.180755	3.145866	0.386576
32	6	0	0.862969	2.931360	0.516586
33	8	0	-6.231392	-0.692900	-1.176109
34	8	0	-1.777574	3.618819	-0.642042
35	8	0	0.458936	2.874248	1.647632
36	6	0	1.616127	4.073192	-0.090812
37	6	0	-2.743394	3.907349	1.544870
38	6	0	-6.256509	-2.878433	-0.160242
39	1	0	0.976760	4.541495	-0.842534
40	1	0	1.872544	4.794311	0.681256
41	1	0	2.514705	3.706875	-0.588775
42	1	0	-2.941869	4.934040	1.247221
43	1	0	-3.650248	3.427904	1.913373
44	1	0	-1.998868	3.889952	2.344476
45	1	0	-7.317129	-2.889643	-0.398489
46	1	0	-5.747424	-3.693604	-0.677960
47	1	0	-6.105564	-3.018671	0.910980
48	8	0	-0.217441	-2.022469	0.761851
49	6	0	0.645304	-3.016230	1.082809
50	8	0	1.232612	-3.671868	0.268473
51	1	0	0.049099	-2.434337	-1.243131
52	6	0	0.752953	-3.156037	2.569413
53	1	0	-0.240980	-3.207630	3.014707
54	1	0	1.329872	-4.044653	2.813210
55	1	0	1.252758	-2.267407	2.962443
56	1	0	7.993429	-0.825799	0.216977

Structure 54 (B3LYP, Gas Phase)

Energy (Hartrees): = - 936.4783818
No imaginary frequencies

Standard orientation:

Center Number	Atomic Number	Atomic Type	Coordinates (Angstroms) X	Y	Z
1	6	0	1.149262	-1.496922	0.538904
2	6	0	0.428258	-0.121023	0.569008
3	6	0	1.197519	0.874722	-0.318016
4	6	0	2.669136	0.900447	0.076167
5	6	0	3.262757	-0.510262	-0.006941
6	1	0	1.126086	0.549276	-1.363923
7	1	0	2.745227	1.247940	1.119594
8	1	0	3.210018	-0.869732	-1.041124
9	1	0	0.438808	0.259614	1.602200
10	8	0	2.507265	-1.369066	0.859990
11	6	0	4.718626	-0.571296	0.462716
12	1	0	4.787418	-0.138292	1.475360
13	1	0	5.013238	-1.622375	0.536653
14	8	0	5.599942	0.053387	-0.449533
15	1	0	5.227322	0.927590	-0.644327
16	8	0	3.400766	1.766442	-0.787408
17	1	0	2.915631	2.604647	-0.803153
18	8	0	0.710465	2.209217	-0.176734
19	1	0	-0.127983	2.270967	-0.652578
20	7	0	-0.917187	-0.288718	0.051495
21	6	0	-3.299864	0.084202	0.288192
22	6	0	-3.658279	-0.530330	-0.923955
23	6	0	-4.305347	0.616060	1.109413
24	6	0	-4.994430	-0.609686	-1.299380
25	1	0	-2.876087	-0.938258	-1.555041
26	6	0	-5.644755	0.535343	0.731621
27	1	0	-4.032455	1.092630	2.047733
28	6	0	-5.990833	-0.077796	-0.473073
29	1	0	-5.265047	-1.086480	-2.236730
30	1	0	-6.415467	0.948959	1.374782
31	1	0	-7.033459	-0.142264	-0.769889
32	6	0	-1.898466	0.185086	0.720235
33	1	0	-1.735366	0.702340	1.678161
34	1	0	0.738565	-2.144917	1.326224
35	8	0	1.004818	-2.093079	-0.729716
36	1	0	0.116311	-1.847097	-1.036555

Structure 54 (B3LYP, DMSO)

Energy (Hartrees): = - 936.5017019
No imaginary frequencies

Standard orientation:

Center Number	Atomic Number	Atomic Type	Coordinates (Angstroms) X	Y	Z
1	6	0	1.147561	-1.485311	0.595253
2	6	0	0.432599	-0.109909	0.597534
3	6	0	1.191838	0.878503	-0.309406
4	6	0	2.669351	0.900087	0.060746
5	6	0	3.255661	-0.513296	-0.013091
6	1	0	1.099837	0.552004	-1.353428
7	1	0	2.769901	1.266338	1.094217
8	1	0	3.182631	-0.883026	-1.043335
9	1	0	0.440518	0.287526	1.622455
10	8	0	2.520543	-1.365598	0.876385
11	6	0	4.719119	-0.571924	0.430301
12	1	0	4.808149	-0.140017	1.440672
13	1	0	5.025320	-1.621267	0.494152
14	8	0	5.585433	0.066650	-0.496323
15	1	0	5.181092	0.929380	-0.687766
16	8	0	3.398951	1.743603	-0.833911
17	1	0	2.939405	2.597946	-0.838337
18	8	0	0.712907	2.214699	-0.164966
19	1	0	-0.119468	2.285732	-0.655142
20	7	0	-0.912310	-0.302144	0.082210
21	6	0	-3.292291	0.097925	0.276778
22	6	0	-3.651562	-0.613928	-0.881773
23	6	0	-4.295983	0.710765	1.043530
24	6	0	-4.987336	-0.709105	-1.258574
25	1	0	-2.875720	-1.086205	-1.475682
26	6	0	-5.634584	0.614724	0.663343
27	1	0	-4.020558	1.262736	1.938597
28	6	0	-5.982148	-0.095361	-0.487530
29	1	0	-5.258383	-1.261476	-2.153672

30	1	0	-6.403734	1.092308	1.263037
31	1	0	-7.023890	-0.171855	-0.785414
32	6	0	-1.893819	0.220976	0.714919
33	1	0	-1.736892	0.795457	1.637702
34	1	0	0.753076	-2.101994	1.414474
35	8	0	0.965101	-2.133585	-0.646208
36	1	0	0.090972	-1.839610	-0.959993

Structure 54 (B3LYP, H_2O)

Energy (Hartrees): = - 936.5080437
No imaginary frequencies

Standard orientation:

Center Number	Atomic Number	Atomic Type	Coordinates (Angstroms) X	Y	Z
1	6	0	1.183060	-1.528448	0.557701
2	6	0	0.433788	-0.180297	0.552939
3	6	0	1.160901	0.830139	-0.357545
4	6	0	2.636690	0.906387	0.021063
5	6	0	3.279719	-0.484393	-0.003399
6	1	0	1.083331	0.501341	-1.401030
7	1	0	2.717676	1.313924	1.039315
8	1	0	3.268193	-0.870251	-1.030155
9	1	0	0.445794	0.218055	1.576779
10	8	0	2.550844	-1.365504	0.867242
11	6	0	4.721273	-0.478162	0.510414
12	1	0	4.743220	-0.033510	1.516797
13	1	0	5.071405	-1.511419	0.594203
14	8	0	5.604047	0.198584	-0.379941
15	1	0	5.174461	1.047095	-0.588242
16	8	0	3.357846	1.733691	-0.898941
17	1	0	2.977332	2.623087	-0.838672
18	8	0	0.621724	2.143449	-0.211551
19	1	0	-0.206934	2.181373	-0.710455
20	7	0	-0.916702	-0.402420	0.060989
21	6	0	-3.284448	0.092867	0.266825
22	6	0	-3.704554	-0.679817	-0.830765
23	6	0	-4.243322	0.792618	1.017207
24	6	0	-5.053762	-0.747882	-1.163597
25	1	0	-2.968562	-1.221720	-1.414767
26	6	0	-5.595076	0.723900	0.681099
27	1	0	-3.921678	1.390732	1.865683
28	6	0	-6.002446	-0.046673	-0.409534
29	1	0	-5.370605	-1.346888	-2.012079
30	1	0	-6.327670	1.269231	1.268115
31	1	0	-7.054264	-0.102355	-0.673549
32	6	0	-1.873806	0.196620	0.666151
33	1	0	-1.682101	0.830646	1.541255
34	1	0	0.796029	-2.166069	1.361998
35	8	0	1.056906	-2.182424	-0.692659
36	1	0	0.192243	-1.909856	-1.046677

Structure 54 (M06-2X, Gas Phase)

Energy (Hartrees): = - 936.3487594
No imaginary frequencies

Standard orientation:

Center Number	Atomic Number	Atomic Type	Coordinates (Angstroms) X	Y	Z
1	6	0	1.199145	-1.515244	0.586850
2	6	0	0.436237	-0.176718	0.597702
3	6	0	1.143742	0.809017	-0.335712
4	6	0	2.603524	0.913273	0.058016
5	6	0	3.247672	-0.468522	0.008532
6	1	0	1.088461	0.441080	-1.366571
7	1	0	2.663068	1.291745	1.089492
8	1	0	3.198108	-0.857132	-1.014016
9	1	0	0.456928	0.239268	1.614305
10	8	0	2.543636	-1.325604	0.902158
11	6	0	4.702041	-0.451963	0.458164
12	1	0	4.771469	0.062033	1.427780
13	1	0	5.025515	-1.484013	0.600052
14	8	0	5.543532	0.129051	-0.509401
15	1	0	5.156104	0.977194	-0.750722
16	8	0	3.296453	1.776436	-0.823756
17	1	0	2.783479	2.589900	-0.878115
18	8	0	0.593586	2.112486	-0.239857

19	1	0	-0.236941	2.130249	-0.722686
20	7	0	-0.912295	-0.395869	0.118458
21	6	0	-3.267283	0.097595	0.283775
22	6	0	-3.639178	-0.703406	-0.799675
23	6	0	-4.243683	0.805836	0.983990
24	6	0	-4.970789	-0.791942	-1.171471
25	1	0	-2.870066	-1.247670	-1.333740
26	6	0	-5.579250	0.717685	0.609060
27	1	0	-3.954550	1.425931	1.826394
28	6	0	-5.942871	-0.081396	-0.468074
29	1	0	-5.257190	-1.414123	-2.010799
30	1	0	-6.333856	1.269070	1.156471
31	1	0	-6.983269	-0.152915	-0.761914
32	6	0	-1.859806	0.209151	0.705169
33	1	0	-1.669039	0.857768	1.571244
34	1	0	0.810605	-2.176094	1.368175
35	8	0	1.094318	-2.116407	-0.675442
36	1	0	0.188033	-1.969102	-0.972128

Structure 54 (M06-2X, DMSO)

Energy (Hartrees): = - 936.3746193
No imaginary frequencies

Standard orientation:

Center Number	Atomic Number	Atomic Type	Coordinates (Angstroms) X	Y	Z
1	6	0	1.174856	-1.497436	0.601255
2	6	0	0.436880	-0.147494	0.602498
3	6	0	1.156257	0.831607	-0.329996
4	6	0	2.619003	0.906415	0.057694
5	6	0	3.239478	-0.485117	0.004517
6	1	0	1.085173	0.476490	-1.364787
7	1	0	2.694848	1.287216	1.086007
8	1	0	3.180061	-0.868632	-1.020193
9	1	0	0.452646	0.266348	1.618644
10	8	0	2.532444	-1.340526	0.897053
11	6	0	4.694730	-0.489053	0.449383
12	1	0	4.775340	0.015096	1.422300
13	1	0	5.017049	-1.524379	0.576508
14	8	0	5.541317	0.103346	-0.515963
15	1	0	5.139433	0.948989	-0.747919
16	8	0	3.331706	1.751274	-0.832987
17	1	0	2.849405	2.586067	-0.875251
18	8	0	0.632682	2.143925	-0.220443
19	1	0	-0.197129	2.183148	-0.708952
20	7	0	-0.910190	-0.363790	0.114123
21	6	0	-3.267147	0.107586	0.275521
22	6	0	-3.632192	-0.691551	-0.813344
23	6	0	-4.250827	0.800774	0.983003
24	6	0	-4.964884	-0.794249	-1.182931
25	1	0	-2.863456	-1.226279	-1.359119
26	6	0	-5.587091	0.698055	0.609819
27	1	0	-3.964189	1.419342	1.827597
28	6	0	-5.944559	-0.099806	-0.471954
29	1	0	-5.245449	-1.414328	-2.026530
30	1	0	-6.347381	1.237740	1.162262
31	1	0	-6.985211	-0.182119	-0.764098
32	6	0	-1.861651	0.234422	0.703603
33	1	0	-1.682880	0.877155	1.574039
34	1	0	0.780391	-2.135916	1.397556
35	8	0	1.042757	-2.125873	-0.648006
36	1	0	0.158305	-1.901502	-0.968565

Structure 54 (M06-2X, H_2O)

Energy (Hartrees): = - 936.3820882
No imaginary frequencies

Standard orientation:

Center Number	Atomic Number	Atomic Type	Coordinates (Angstroms) X	Y	Z
1	6	0	1.212488	-1.536936	0.586949
2	6	0	0.438256	-0.214824	0.589966
3	6	0	1.122377	0.791327	-0.342194
4	6	0	2.584685	0.915777	0.041155
5	6	0	3.255884	-0.452375	0.005966
6	1	0	1.058793	0.443186	-1.378624
7	1	0	2.651878	1.317860	1.061107
8	1	0	3.228852	-0.841216	-1.017992

9	1	0	0.457408	0.195003	1.606759
10	8	0	2.570477	-1.337393	0.890972
11	6	0	4.701600	-0.404980	0.478200
12	1	0	4.752403	0.117294	1.441436
13	1	0	5.056041	-1.427002	0.619921
14	8	0	5.547522	0.205033	-0.484034
15	1	0	5.149049	1.057757	-0.702147
16	8	0	3.276509	1.760404	-0.867954
17	1	0	2.840264	2.620787	-0.852745
18	8	0	0.542823	2.080125	-0.218579
19	1	0	-0.242344	2.120516	-0.775519
20	7	0	-0.912297	-0.463614	0.123589
21	6	0	-3.256776	0.100915	0.263347
22	6	0	-3.668129	-0.749417	-0.768543
23	6	0	-4.204837	0.869224	0.941728
24	6	0	-5.009966	-0.827228	-1.110563
25	1	0	-2.930898	-1.344174	-1.294040
26	6	0	-5.550203	0.790616	0.597137
27	1	0	-3.882146	1.527315	1.741875
28	6	0	-5.953185	-0.057685	-0.428670
29	1	0	-5.325746	-1.486418	-1.910431
30	1	0	-6.281167	1.388595	1.127974
31	1	0	-7.000624	-0.121020	-0.699514
32	6	0	-1.843857	0.211174	0.666651
33	1	0	-1.638404	0.913348	1.482432
34	1	0	0.839888	-2.196432	1.374236
35	8	0	1.117786	-2.170077	-0.669780
36	1	0	0.241885	-1.958367	-1.021625

Structure 54 (M06-2X/def2-TZVP, Gas Phase)

Energy (Hartrees): = -936.464964
No imaginary frequencies

Standard orientation:

Center Number	Atomic Number	Atomic Type	Coordinates (Angstroms) X	Y	Z
1	6	0	1.176840	-1.499022	0.571540
2	6	0	0.433815	-0.151316	0.575400
3	6	0	1.161132	0.828998	-0.344065
4	6	0	2.618954	0.910681	0.057250
5	6	0	3.243374	-0.478167	0.004262
6	1	0	1.104582	0.471795	-1.377874
7	1	0	2.679040	1.280377	1.090582
8	1	0	3.199072	-0.858484	-1.021362
9	1	0	0.452668	0.257502	1.594015
10	8	0	2.522358	-1.330322	0.883981
11	6	0	4.690497	-0.487833	0.473033
12	1	0	4.755229	0.013827	1.447759
13	1	0	4.997137	-1.525009	0.609289
14	8	0	5.561225	0.088440	-0.469613
15	1	0	5.199246	0.946060	-0.721959
16	8	0	3.329304	1.770723	-0.811561
17	1	0	2.830646	2.593711	-0.875029
18	8	0	0.625498	2.136608	-0.242854
19	1	0	-0.220902	2.164018	-0.699704
20	7	0	-0.911704	-0.349574	0.089207
21	6	0	-3.272276	0.093837	0.284515
22	6	0	-3.639369	-0.656081	-0.833356
23	6	0	-4.252436	0.751644	1.021969
24	6	0	-4.969157	-0.743588	-1.201816
25	1	0	-2.868745	-1.163811	-1.398233
26	6	0	-5.586589	0.664567	0.650883
27	1	0	-3.966532	1.333509	1.890869
28	6	0	-5.944889	-0.083255	-0.460659
29	1	0	-5.251819	-1.327625	-2.068037
30	1	0	-6.344289	1.178108	1.227857
31	1	0	-6.984855	-0.154136	-0.752332
32	6	0	-1.867159	0.204155	0.706587
33	1	0	-1.685000	0.804758	1.607149
34	1	0	0.778327	-2.145504	1.359288
35	8	0	1.059339	-2.118083	-0.680310
36	1	0	0.169920	-1.928730	-1.007123

Structure 54 (M06-2X/def2-TZVP, DMSO)

Energy (Hartrees): = -936.490747
No imaginary frequencies

Standard orientation:

Center Number	Atomic Number	Atomic Type	Coordinates (Angstroms) X	Y	Z

1	6	0	1.154699	-1.482129	0.588092
2	6	0	0.435357	-0.124362	0.580667
3	6	0	1.173011	0.849145	-0.338869
4	6	0	2.632886	0.905147	0.056912
5	6	0	3.235446	-0.492424	-0.001845
6	1	0	1.102864	0.502330	-1.375902
7	1	0	2.708199	1.276063	1.087642
8	1	0	3.180433	-0.867321	-1.029693
9	1	0	0.449416	0.283314	1.598426
10	8	0	2.513035	-1.344200	0.877315
11	6	0	4.683493	-0.522134	0.460804
12	1	0	4.759918	-0.031905	1.439475
13	1	0	4.990344	-1.562249	0.579520
14	8	0	5.557580	0.068280	-0.479628
15	1	0	5.181041	0.925405	-0.717572
16	8	0	3.362287	1.748689	-0.819653
17	1	0	2.897642	2.594602	-0.863908
18	8	0	0.659283	2.163874	-0.227590
19	1	0	-0.194688	2.203052	-0.675816
20	7	0	-0.908710	-0.321008	0.085685
21	6	0	-3.270966	0.100623	0.278113
22	6	0	-3.634276	-0.658051	-0.836440
23	6	0	-4.255048	0.757693	1.012988
24	6	0	-4.964853	-0.755453	-1.204459
25	1	0	-2.866396	-1.168032	-1.404642
26	6	0	-5.589502	0.660913	0.641580
27	1	0	-3.969526	1.345433	1.878292
28	6	0	-5.944765	-0.095941	-0.466315
29	1	0	-5.244245	-1.345872	-2.068169
30	1	0	-6.350326	1.174255	1.215886
31	1	0	-6.984898	-0.174156	-0.757795
32	6	0	-1.868114	0.224210	0.707120
33	1	0	-1.696683	0.817349	1.612371
34	1	0	0.753113	-2.104000	1.393239
35	8	0	1.005508	-2.130728	-0.648593
36	1	0	0.139016	-1.871955	-0.996035

Structure 54.1H_2O (B3LYP, Gas Phase)

Energy (Hartrees): = - 1012.9158178
No imaginary frequencies

Standard orientation:

Center Number	Atomic Number	Atomic Type	Coordinates (Angstroms) X	Y	Z
1	6	0	-4.069055	-1.317998	-0.813868
2	6	0	-3.204409	-0.297022	-0.386218
3	6	0	-3.750624	0.855565	0.207716
4	6	0	-5.127511	0.967152	0.373485
5	6	0	-5.978522	-0.060559	-0.045511
6	6	0	-5.446755	-1.204936	-0.640637
7	6	0	-1.762773	-0.490733	-0.598048
8	7	0	-0.839908	0.270500	-0.142965
9	6	0	0.539703	-0.093774	-0.454926
10	6	0	1.155079	-0.951154	0.659365
11	6	0	2.607266	-1.285424	0.308009
12	6	0	3.405034	-0.014074	0.002086
13	8	0	2.727399	0.723564	-1.033655
14	6	0	1.432313	1.162158	-0.672433
15	8	0	0.395269	-2.141392	0.795945
16	8	0	1.541659	1.971976	0.459269
17	6	0	4.802290	-0.269253	-0.550861
18	8	0	5.472679	0.940375	-0.838172
19	8	0	3.122199	-2.002915	1.435194
20	1	0	-1.500724	-1.375469	-1.192518
21	1	0	0.577836	-0.680251	-1.385206
22	1	0	0.894618	-2.693511	1.415625
23	1	0	1.151152	-0.373346	1.594648
24	1	0	3.889052	-2.515133	1.150848
25	1	0	2.594744	-1.930030	-0.584707
26	1	0	4.827287	1.485809	-1.312523
27	1	0	5.402410	-0.801342	0.195829
28	1	0	4.719973	-0.911062	-1.445559
29	1	0	3.478932	0.601101	0.905423
30	1	0	0.686753	2.418138	0.650077
31	1	0	1.063868	1.721826	-1.544023
32	1	0	-3.652183	-2.208042	-1.278152
33	1	0	-6.102489	-2.004776	-0.970968
34	1	0	-7.052168	0.034501	0.087889
35	1	0	-5.541688	1.862140	0.828102
36	1	0	-3.100501	1.663731	0.524545
37	1	0	-1.078442	1.768589	0.827379
38	8	0	-1.009946	2.691038	1.188484
39	1	0	-0.985364	2.582766	2.148576

Structure 54.1H$_2$O (B3LYP, DMSO)

Energy (Hartrees): = - 1012.9423242
No imaginary frequencies

Standard orientation:

Center Number	Atomic Number	Atomic Type	Coordinates (Angstroms) X	Y	Z
1	6	0	-4.145713	-0.931476	-1.287224
2	6	0	-3.242727	-0.223852	-0.475742
3	6	0	-3.745941	0.639803	0.515103
4	6	0	-5.119571	0.780500	0.686962
5	6	0	-6.010592	0.066570	-0.122799
6	6	0	-5.521382	-0.789401	-1.111119
7	6	0	-1.806766	-0.426855	-0.712218
8	7	0	-0.871048	0.097187	-0.013035
9	6	0	0.505186	-0.200952	-0.387918
10	6	0	1.239254	-0.939007	0.737535
11	6	0	2.684900	-1.213291	0.315934
12	6	0	3.378781	0.083225	-0.115509
13	8	0	2.602708	0.716336	-1.152002
14	6	0	1.293134	1.091304	-0.752636
15	8	0	0.562700	-2.156686	1.016292
16	8	0	1.394323	2.043311	0.264989
17	6	0	4.759023	-0.126169	-0.722635
18	8	0	5.339997	1.107440	-1.124588
19	8	0	3.320846	-1.814430	1.449942
20	1	0	-1.564932	-1.085333	-1.556083
21	1	0	0.532667	-0.833688	-1.286722
22	1	0	1.163473	-2.649466	1.597735
23	1	0	1.260192	-0.300585	1.634301
24	1	0	4.037675	-2.379803	1.128563
25	1	0	2.667937	-1.914869	-0.529920
26	1	0	4.649958	1.556401	-1.637199
27	1	0	5.426974	-0.570957	0.021701
28	1	0	4.679785	-0.826881	-1.569607
29	1	0	3.458682	0.755799	0.746493
30	1	0	0.629257	2.026255	0.882809
31	1	0	0.847344	1.527869	-1.657769
32	1	0	-3.760668	-1.596414	-2.055845
33	1	0	-6.208491	-1.343929	-1.743214
34	1	0	-7.081624	0.180815	0.017080
35	1	0	-5.500308	1.449954	1.452698
36	1	0	-3.063843	1.203524	1.142383
37	1	0	-0.970169	1.170607	1.417985
38	8	0	-0.748737	1.926183	2.023042
39	1	0	-0.486798	1.510754	2.858055

Structure 54.1H$_2$O (B3LYP, H$_2$O)

Energy (Hartrees): = - 1012.9531439
No imaginary frequencies

Standard orientation:

Center Number	Atomic Number	Atomic Type	Coordinates (Angstroms) X	Y	Z
1	6	0	-4.073744	-1.235315	-1.006316
2	6	0	-3.218521	-0.298197	-0.400836
3	6	0	-3.779121	0.744990	0.359966
4	6	0	-5.159719	0.838219	0.507141
5	6	0	-6.001700	-0.103005	-0.096230
6	6	0	-5.456097	-1.140781	-0.853910
7	6	0	-1.774507	-0.474262	-0.603447
8	7	0	-0.857729	0.262160	-0.091314
9	6	0	0.523852	-0.107546	-0.395788
10	6	0	1.173692	-0.865790	0.768819
11	6	0	2.608620	-1.242178	0.392373
12	6	0	3.403774	-0.010102	-0.060539
13	8	0	2.689736	0.680568	-1.109773
14	6	0	1.396413	1.125587	-0.746165
15	8	0	0.410328	-2.038006	1.040898
16	8	0	1.542315	2.057823	0.301733
17	6	0	4.763094	-0.360195	-0.646252
18	8	0	5.528884	0.802089	-0.955879
19	8	0	3.195066	-1.841519	1.553122
20	1	0	-1.505947	-1.317679	-1.250554
21	1	0	0.554454	-0.762073	-1.278179
22	1	0	0.935879	-2.550546	1.675222
23	1	0	1.202684	-0.217139	1.655326
24	1	0	3.872271	-2.466321	1.257768
25	1	0	2.565465	-1.969938	-0.428740

26	1	0	4.990958	1.326623	-1.567641
27	1	0	5.335276	-0.931018	0.090225
28	1	0	4.621049	-0.990567	-1.536214
29	1	0	3.543399	0.661243	0.794482
30	1	0	0.660739	2.380715	0.602300
31	1	0	0.994815	1.605468	-1.647853
32	1	0	-3.645398	-2.041531	-1.595528
33	1	0	-6.104383	-1.872934	-1.325235
34	1	0	-7.077922	-0.024624	0.024809
35	1	0	-5.584026	1.647158	1.093994
36	1	0	-3.138431	1.484336	0.826904
37	1	0	-1.073980	1.771902	0.879454
38	8	0	-0.961413	2.676925	1.274791
39	1	0	-0.818351	2.500209	2.216620

Structure 54.1H_2O (M06-2X, Gas Phase)

Energy (Hartrees): = - 1012.7805207
No imaginary frequencies

Standard orientation:

Center Number	Atomic Number	Atomic Type	Coordinates (Angstroms) X	Y	Z
1	6	0	-4.015103	-1.425418	-0.489021
2	6	0	-3.165008	-0.325215	-0.368373
3	6	0	-3.701869	0.929141	-0.061776
4	6	0	-5.067614	1.066479	0.137646
5	6	0	-5.908457	-0.039706	0.035550
6	6	0	-5.381432	-1.286731	-0.278470
7	6	0	-1.721957	-0.529149	-0.590540
8	7	0	-0.829682	0.288504	-0.206789
9	6	0	0.552718	-0.057254	-0.513674
10	6	0	1.127608	-1.024422	0.516588
11	6	0	2.582675	-1.311206	0.176566
12	6	0	3.372956	-0.013399	0.057430
13	8	0	2.740710	0.828943	-0.908825
14	6	0	1.435472	1.206584	-0.548370
15	8	0	0.375184	-2.219038	0.488576
16	8	0	1.486011	1.845210	0.687781
17	6	0	4.785748	-0.209626	-0.455072
18	8	0	5.453976	1.021112	-0.575407
19	8	0	3.066628	-2.145773	1.219887
20	1	0	-1.446350	-1.454621	-1.107214
21	1	0	0.616240	-0.537089	-1.499086
22	1	0	0.825238	-2.830535	1.080741
23	1	0	1.086020	-0.557183	1.508216
24	1	0	3.859667	-2.595438	0.921079
25	1	0	2.604776	-1.843200	-0.785543
26	1	0	4.837828	1.612260	-1.021094
27	1	0	5.355382	-0.818335	0.251658
28	1	0	4.739197	-0.737225	-1.419776
29	1	0	3.400504	0.494103	1.026659
30	1	0	0.696948	2.402059	0.805315
31	1	0	1.087263	1.880320	-1.337591
32	1	0	-3.597851	-2.395491	-0.737955
33	1	0	-6.033253	-2.147675	-0.363205
34	1	0	-6.974396	0.074412	0.193200
35	1	0	-5.481305	2.041498	0.365508
36	1	0	-3.052841	1.794636	-0.000151
37	1	0	-1.144628	1.860584	0.712903
38	8	0	-1.068600	2.797687	0.993153
39	1	0	-1.376353	2.831795	1.901627

Structure 54.1H_2O (M06-2X, DMSO)

Energy (Hartrees): = - 1012.8098004
No imaginary frequencies

Standard orientation:

Center Number	Atomic Number	Atomic Type	Coordinates (Angstroms) X	Y	Z
1	6	0	-4.175465	-0.823962	-1.303626
2	6	0	-3.230975	-0.157759	-0.520185
3	6	0	-3.660869	0.691931	0.505405
4	6	0	-5.016903	0.859188	0.744643
5	6	0	-5.955746	0.183251	-0.035037
6	6	0	-5.534669	-0.658136	-1.059130
7	6	0	-1.802614	-0.371092	-0.812914
8	7	0	-0.870214	0.070183	-0.073974
9	6	0	0.499203	-0.192123	-0.478666
10	6	0	1.223598	-1.007699	0.586927

11	6	0	2.671460	-1.212322	0.172637
12	6	0	3.329668	0.134859	-0.104528
13	8	0	2.580587	0.830053	-1.102673
14	6	0	1.256045	1.129884	-0.718915
15	8	0	0.560791	-2.245508	0.729651
16	8	0	1.281908	1.991238	0.374898
17	6	0	4.733564	0.014642	-0.658979
18	8	0	5.282059	1.291141	-0.921868
19	8	0	3.296856	-1.898406	1.248847
20	1	0	-1.582272	-0.946819	-1.719093
21	1	0	0.533527	-0.751185	-1.422719
22	1	0	1.123927	-2.783484	1.299235
23	1	0	1.218893	-0.459827	1.540194
24	1	0	4.065559	-2.368354	0.909496
25	1	0	2.688738	-1.824557	-0.738434
26	1	0	4.614984	1.759877	-1.436319
27	1	0	5.381530	-0.471838	0.072926
28	1	0	4.708863	-0.601318	-1.568455
29	1	0	3.351320	0.727982	0.816117
30	1	0	0.630541	1.762924	1.059639
31	1	0	0.822979	1.635773	-1.586821
32	1	0	-3.839199	-1.475883	-2.103466
33	1	0	-6.262292	-1.181648	-1.667994
34	1	0	-7.014254	0.317971	0.156027
35	1	0	-5.346441	1.520153	1.537751
36	1	0	-2.933864	1.225249	1.107132
37	1	0	-0.914755	0.731036	1.660218
38	8	0	-0.602411	1.329211	2.368776
39	1	0	-0.372383	0.766884	3.115536

Structure 54.1H_2O (M06-2X, H_2O)

Energy (Hartrees): = - 1012.8208475
No imaginary frequencies

Standard orientation:

Center Number	Atomic Number	Atomic Type	Coordinates (Angstroms) X	Y	Z
1	6	0	-4.015310	-1.359060	-0.757167
2	6	0	-3.178848	-0.306653	-0.375265
3	6	0	-3.738815	0.843027	0.193390
4	6	0	-5.110877	0.925466	0.382510
5	6	0	-5.938059	-0.134709	0.012390
6	6	0	-5.389173	-1.277321	-0.559517
7	6	0	-1.732626	-0.474828	-0.598933
8	7	0	-0.834846	0.311725	-0.155256
9	6	0	0.541633	-0.065643	-0.462582
10	6	0	1.130823	-0.951019	0.631550
11	6	0	2.567952	-1.298645	0.272440
12	6	0	3.378893	-0.032637	0.015823
13	8	0	2.733137	0.767666	-0.982673
14	6	0	1.431748	1.175723	-0.633839
15	8	0	0.350616	-2.129485	0.733447
16	8	0	1.521377	1.948895	0.537802
17	6	0	4.762919	-0.327147	-0.518828
18	8	0	5.519233	0.862823	-0.678929
19	8	0	3.098779	-2.031731	1.368215
20	1	0	-1.459028	-1.357892	-1.185099
21	1	0	0.581550	-0.623638	-1.405893
22	1	0	0.788464	-2.694558	1.381717
23	1	0	1.127355	-0.409177	1.585024
24	1	0	3.805624	-2.599928	1.044643
25	1	0	2.556106	-1.917949	-0.632805
26	1	0	5.018880	1.432559	-1.274423
27	1	0	5.300795	-0.956128	0.191823
28	1	0	4.672586	-0.865332	-1.470265
29	1	0	3.465166	0.536728	0.947190
30	1	0	0.694329	2.451912	0.655133
31	1	0	1.071892	1.779193	-1.470739
32	1	0	-3.580795	-2.246004	-1.206212
33	1	0	-6.027599	-2.101885	-0.853017
34	1	0	-7.008511	-0.064815	0.166156
35	1	0	-5.539594	1.820142	0.817921
36	1	0	-3.109428	1.678927	0.471769
37	1	0	-1.120453	1.982771	0.660919
38	8	0	-1.030917	2.924562	0.919810
39	1	0	-1.192801	2.931830	1.869736

Structure 54.5H_2O (B3LYP, Gas Phase)

Energy (Hartrees): = - 1318.6810625
No imaginary frequencies

Standard orientation:

Center Number	Atomic Number	Atomic Type	Coordinates (Angstroms) X	Y	Z
1	6	0	4.709858	-0.165588	-1.675941
2	6	0	3.739785	-0.342573	-0.677032
3	6	0	4.152564	-0.498387	0.658885
4	6	0	5.505960	-0.471169	0.977357
5	6	0	6.465016	-0.290152	-0.025219
6	6	0	6.065279	-0.137677	-1.353138
7	6	0	2.324685	-0.359450	-1.072043
8	7	0	1.353154	-0.457555	-0.248341
9	6	0	-0.005504	-0.480666	-0.767119
10	6	0	-0.839081	0.672700	-0.190470
11	6	0	-2.287274	0.615745	-0.701199
12	6	0	-2.862548	-0.767666	-0.371663
13	8	0	-2.024481	-1.775079	-0.963201
14	6	0	-0.710509	-1.829325	-0.452723
15	8	0	-0.181265	1.901047	-0.519280
16	8	0	-0.718756	-2.204117	0.900595
17	6	0	-4.277065	-1.027371	-0.877014
18	8	0	-4.891645	-2.112196	-0.221351
19	8	0	-3.071087	1.613339	-0.044344
20	1	0	2.135592	-0.276623	-2.152284
21	1	0	-0.011008	-0.385911	-1.863946
22	1	0	-0.776709	2.625968	-0.242416
23	1	0	-0.883153	0.564087	0.901768
24	1	0	-3.193749	2.384881	-0.640532
25	1	0	-2.297154	0.774720	-1.788219
26	1	0	-4.231013	-2.822333	-0.087352
27	1	0	-4.884499	-0.137607	-0.681845
28	1	0	-4.225939	-1.171978	-1.970345
29	1	0	-2.871663	-0.886045	0.720286
30	1	0	-0.297485	-1.558921	1.514267
31	1	0	-0.226648	-2.626488	-1.028661
32	1	0	4.395340	-0.048022	-2.709858
33	1	0	6.806140	0.000507	-2.134577
34	1	0	7.520345	-0.271927	0.230265
35	1	0	5.817985	-0.596198	2.009837
36	1	0	3.412013	-0.646153	1.437819
37	1	0	-3.521581	-3.690937	1.851052
38	8	0	-3.089073	-3.907294	1.015359
39	1	0	-2.248150	-3.414049	1.043883
40	1	0	-2.479039	4.352098	-0.406284
41	8	0	-3.005759	4.101353	-1.188910
42	1	0	-3.822203	4.612632	-1.123804
43	8	0	-1.661523	3.681637	1.191583
44	1	0	-0.886001	3.477669	1.748505
45	1	0	-2.230424	2.889707	1.227697
46	1	0	0.739211	2.222663	1.075695
47	8	0	0.726172	2.416120	2.039910
48	1	0	1.570638	2.839364	2.240191
49	1	0	1.274632	-0.472333	1.590209
50	8	0	0.840995	-0.522180	2.472676
51	1	0	0.666829	0.412539	2.676654

Structure 54.5H_2O (B3LYP, DMSO)

Energy (Hartrees): = - 1318.7170818
No imaginary frequencies

Standard orientation:

Center Number	Atomic Number	Atomic Type	Coordinates (Angstroms) X	Y	Z
1	6	0	4.712553	-0.245297	-1.679539
2	6	0	3.742330	-0.332958	-0.667328
3	6	0	4.157512	-0.386187	0.676223
4	6	0	5.512210	-0.352223	0.990903
5	6	0	6.471446	-0.266578	-0.025124
6	6	0	6.069663	-0.212695	-1.360921
7	6	0	2.328537	-0.363733	-1.066162
8	7	0	1.352262	-0.438182	-0.242018
9	6	0	-0.001740	-0.471730	-0.781730
10	6	0	-0.854232	0.673415	-0.213943
11	6	0	-2.297546	0.592479	-0.735388
12	6	0	-2.856357	-0.793409	-0.389702
13	8	0	-2.011124	-1.807941	-0.962707
14	6	0	-0.683478	-1.829996	-0.475792
15	8	0	-0.216270	1.910464	-0.546224
16	8	0	-0.653192	-2.225957	0.878519
17	6	0	-4.263015	-1.065687	-0.901669
18	8	0	-4.877550	-2.170939	-0.261225

19	8	0	-3.109555	1.581057	-0.097017
20	1	0	2.146057	-0.316531	-2.147443
21	1	0	0.006560	-0.379835	-1.877309
22	1	0	-0.799096	2.623497	-0.217610
23	1	0	-0.907062	0.573667	0.878586
24	1	0	-3.182157	2.369210	-0.682281
25	1	0	-2.303828	0.736305	-1.823741
26	1	0	-4.182020	-2.794068	0.035796
27	1	0	-4.880511	-0.180825	-0.713311
28	1	0	-4.209950	-1.208002	-1.994343
29	1	0	-2.868081	-0.894688	0.704820
30	1	0	-0.321375	-1.529567	1.492031
31	1	0	-0.191319	-2.613818	-1.060492
32	1	0	4.395606	-0.203607	-2.718261
33	1	0	6.810423	-0.145972	-2.152128
34	1	0	7.527682	-0.241754	0.226790
35	1	0	5.825120	-0.393669	2.030023
36	1	0	3.420298	-0.453924	1.469321
37	1	0	-3.406551	-3.505510	2.043546
38	8	0	-3.045612	-3.779542	1.188098
39	1	0	-2.202635	-3.287392	1.117662
40	1	0	-2.477240	4.319880	-0.441044
41	8	0	-2.966610	4.063895	-1.245435
42	1	0	-3.799367	4.557133	-1.198839
43	8	0	-1.706274	3.650930	1.214204
44	1	0	-0.923884	3.418692	1.754080
45	1	0	-2.276344	2.860118	1.232727
46	1	0	0.750782	2.232588	1.120714
47	8	0	0.659826	2.399392	2.082257
48	1	0	1.464616	2.871759	2.345400
49	1	0	1.251344	-0.467667	1.596599
50	8	0	0.802851	-0.496262	2.476008
51	1	0	0.627860	0.448076	2.642037

Structure 54.5H_2O (B3LYP, H_2O)

Energy (Hartrees): = - 1318.7372715
No imaginary frequencies

Standard orientation:

Center Number	Atomic Number	Atomic Type	Coordinates (Angstroms) X	Y	Z
1	6	0	4.698039	-0.300059	-1.670993
2	6	0	3.726449	-0.351955	-0.657701
3	6	0	4.139119	-0.373694	0.687433
4	6	0	5.494197	-0.350876	1.001962
5	6	0	6.455437	-0.308666	-0.014970
6	6	0	6.055380	-0.281952	-1.352249
7	6	0	2.313781	-0.382473	-1.058358
8	7	0	1.337848	-0.526767	-0.241668
9	6	0	-0.012533	-0.522661	-0.793318
10	6	0	-0.830383	0.662251	-0.250799
11	6	0	-2.274211	0.609584	-0.776813
12	6	0	-2.871449	-0.746106	-0.383711
13	8	0	-2.067258	-1.798559	-0.953736
14	6	0	-0.730141	-1.858428	-0.488009
15	8	0	-0.149903	1.863564	-0.626950
16	8	0	-0.677330	-2.271579	0.863484
17	6	0	-4.293420	-0.986901	-0.865389
18	8	0	-4.930017	-2.059024	-0.175417
19	8	0	-3.069295	1.640183	-0.188992
20	1	0	2.131596	-0.280329	-2.135493
21	1	0	0.008927	-0.437553	-1.889196
22	1	0	-0.675329	2.609256	-0.287965
23	1	0	-0.883109	0.601852	0.843162
24	1	0	-3.002744	2.448494	-0.749902
25	1	0	-2.271539	0.709986	-1.870093
26	1	0	-4.250528	-2.717418	0.087085
27	1	0	-4.886289	-0.084346	-0.692254
28	1	0	-4.267549	-1.169376	-1.950963
29	1	0	-2.861393	-0.827549	0.712096
30	1	0	-0.429025	-1.546336	1.476012
31	1	0	-0.273696	-2.655137	-1.081364
32	1	0	4.381511	-0.278532	-2.710147
33	1	0	6.797134	-0.246617	-2.144112
34	1	0	7.511466	-0.293459	0.237220
35	1	0	5.806286	-0.366526	2.041714
36	1	0	3.398601	-0.404931	1.479437
37	1	0	-3.527783	-3.550166	1.967497
38	8	0	-3.125712	-3.801080	1.123462
39	1	0	-2.297224	-3.280759	1.098023
40	1	0	-2.241083	4.295029	-0.485860
41	8	0	-2.674968	4.114935	-1.343720
42	1	0	-3.539251	4.544492	-1.261090
43	8	0	-1.703540	3.745953	1.299059

44	1	0	-0.891858	3.415112	1.738261
45	1	0	-2.246767	2.945319	1.185360
46	1	0	0.752269	2.206484	1.261377
47	8	0	0.588806	2.368944	2.208619
48	1	0	1.367068	2.861598	2.509422
49	1	0	1.207165	-0.532395	1.619699
50	8	0	0.804654	-0.501208	2.520156
51	1	0	0.633122	0.455751	2.623528

Structure 54.5H_2O (M06-2X, Gas Phase)

Energy (Hartrees): = - 1318.532368
No imaginary frequencies

Standard orientation:

Center Number	Atomic Number	Atomic Type	Coordinates (Angstroms) X	Y	Z
1	6	0	4.494874	-0.506045	-1.604609
2	6	0	3.485024	-0.672477	-0.656189
3	6	0	3.825948	-0.816972	0.693488
4	6	0	5.158745	-0.803186	1.075843
5	6	0	6.162640	-0.644081	0.121495
6	6	0	5.830066	-0.496438	-1.219502
7	6	0	2.082586	-0.649650	-1.108153
8	7	0	1.102734	-0.906714	-0.346710
9	6	0	-0.232206	-0.683010	-0.869165
10	6	0	-0.727604	0.669627	-0.342771
11	6	0	-2.176341	0.936665	-0.740986
12	6	0	-3.029541	-0.274730	-0.377211
13	8	0	-2.485232	-1.443369	-0.980533
14	6	0	-1.213771	-1.807957	-0.505267
15	8	0	0.183401	1.645771	-0.831184
16	8	0	-1.265013	-2.146127	0.848531
17	6	0	-4.465397	-0.169415	-0.854796
18	8	0	-5.296721	-1.111513	-0.233422
19	8	0	-2.685147	2.048918	-0.023150
20	1	0	1.923623	-0.362731	-2.155329
21	1	0	-0.227912	-0.618289	-1.965872
22	1	0	-0.249355	2.515308	-0.911152
23	1	0	-0.696734	0.657638	0.755557
24	1	0	-2.454826	2.862783	-0.492702
25	1	0	-2.230935	1.108991	-1.825093
26	1	0	-4.814377	-1.949992	-0.149491
27	1	0	-4.843212	0.824644	-0.609158
28	1	0	-4.458857	-0.285029	-1.949021
29	1	0	-3.036928	-0.385421	0.715336
30	1	0	-0.793429	-1.535004	1.443301
31	1	0	-0.957272	-2.710638	-1.062389
32	1	0	4.230310	-0.385567	-2.649912
33	1	0	6.607504	-0.371764	-1.963324
34	1	0	7.202280	-0.635354	0.426730
35	1	0	5.418718	-0.920091	2.120970
36	1	0	3.048073	-0.941514	1.437164
37	1	0	-4.296766	-2.999678	1.829002
38	8	0	-3.924887	-3.242445	0.978219
39	1	0	-2.995456	-2.975091	1.027634
40	1	0	-0.874834	4.153604	0.216388
41	8	0	-1.053937	4.118574	-0.750488
42	1	0	-0.871433	4.975780	-1.137863
43	8	0	-0.777095	3.388219	1.762086
44	1	0	0.031738	2.877923	1.922831
45	1	0	-1.492741	2.746074	1.664977
46	1	0	1.313534	1.705445	0.594604
47	8	0	1.517151	1.748921	1.549534
48	1	0	2.461770	1.901671	1.628243
49	1	0	1.010298	-1.031112	1.555338
50	8	0	0.583901	-0.818390	2.404806
51	1	0	0.786575	0.121939	2.491942

Structure 54.5H_2O (M06-2X, DMSO)

Energy (Hartrees): = - 1318.5710306
No imaginary frequencies

Standard orientation:

Center Number	Atomic Number	Atomic Type	Coordinates (Angstroms) X	Y	Z
1	6	0	4.589041	-0.538787	-1.607994
2	6	0	3.598575	-0.579148	-0.624306
3	6	0	3.968483	-0.594881	0.725438
4	6	0	5.309946	-0.578295	1.077397

5	6	0	6.294516	-0.545900	0.089720
6	6	0	5.933713	-0.525243	-1.253164
7	6	0	2.189819	-0.585554	-1.054861
8	7	0	1.214317	-0.709238	-0.251746
9	6	0	-0.125202	-0.624358	-0.807988
10	6	0	-0.786145	0.680689	-0.354867
11	6	0	-2.227128	0.753594	-0.853955
12	6	0	-2.965195	-0.515410	-0.437012
13	8	0	-2.278918	-1.669091	-0.917805
14	6	0	-0.978389	-1.836243	-0.407796
15	8	0	0.032693	1.739126	-0.830495
16	8	0	-1.025760	-2.101078	0.968815
17	6	0	-4.371945	-0.596010	-0.993156
18	8	0	-5.149567	-1.580913	-0.349344
19	8	0	-2.912306	1.845587	-0.255762
20	1	0	2.024177	-0.467698	-2.131705
21	1	0	-0.100336	-0.613052	-1.905367
22	1	0	-0.490361	2.553733	-0.948883
23	1	0	-0.826593	0.712445	0.743589
24	1	0	-2.705970	2.653079	-0.750296
25	1	0	-2.233348	0.844503	-1.947949
26	1	0	-4.558995	-2.270937	-0.004250
27	1	0	-4.863051	0.369430	-0.855676
28	1	0	-4.292099	-0.792985	-2.071654
29	1	0	-3.015481	-0.542093	0.660384
30	1	0	-0.619269	-1.407473	1.522708
31	1	0	-0.593950	-2.727752	-0.906178
32	1	0	4.299712	-0.519157	-2.653822
33	1	0	6.696449	-0.495730	-2.022476
34	1	0	7.341547	-0.533074	0.370424
35	1	0	5.592344	-0.588579	2.123760
36	1	0	3.208653	-0.614391	1.497716
37	1	0	-3.920743	-3.020088	2.124158
38	8	0	-3.593962	-3.291706	1.260391
39	1	0	-2.691536	-2.936017	1.215949
40	1	0	-1.403023	4.197911	0.030461
41	8	0	-1.476628	4.052110	-0.934826
42	1	0	-1.323589	4.885507	-1.391182
43	8	0	-1.419346	3.450265	1.666126
44	1	0	-0.554719	3.046389	1.849382
45	1	0	-2.000027	2.714604	1.425802
46	1	0	1.022298	2.032458	0.710961
47	8	0	1.077950	2.158528	1.677375
48	1	0	1.916548	2.599224	1.854461
49	1	0	1.109414	-0.681548	1.628316
50	8	0	0.647850	-0.523281	2.475390
51	1	0	0.685154	0.442857	2.529917

Structure 54.5H_2O (M06-2X, H_2O)

Energy (Hartrees): = - 1318.5910981
No imaginary frequencies

Standard orientation:

Center Number	Atomic Number	Atomic Type	Coordinates (Angstroms) X	Y	Z
1	6	0	4.544706	-0.642639	-1.550509
2	6	0	3.524996	-0.646297	-0.596322
3	6	0	3.851960	-0.633790	0.764700
4	6	0	5.182885	-0.633770	1.156685
5	6	0	6.196771	-0.644328	0.198745
6	6	0	5.877726	-0.648284	-1.155167
7	6	0	2.131066	-0.647251	-1.070815
8	7	0	1.132714	-0.851749	-0.310159
9	6	0	-0.187152	-0.702479	-0.901670
10	6	0	-0.753549	0.661666	-0.494580
11	6	0	-2.205312	0.816693	-0.933938
12	6	0	-3.005525	-0.384026	-0.443500
13	8	0	-2.422442	-1.590623	-0.942116
14	6	0	-1.113944	-1.849106	-0.481086
15	8	0	0.099361	1.639219	-1.071377
16	8	0	-1.109619	-2.135719	0.896787
17	6	0	-4.441393	-0.373658	-0.921739
18	8	0	-5.250495	-1.300266	-0.214760
19	8	0	-2.787707	1.970720	-0.340909
20	1	0	1.996888	-0.462291	-2.142251
21	1	0	-0.142481	-0.724883	-1.997716
22	1	0	-0.276732	2.532373	-0.932583
23	1	0	-0.736223	0.747719	0.599780
24	1	0	-2.429316	2.763462	-0.767503
25	1	0	-2.257424	0.876444	-2.028081
26	1	0	-4.687042	-2.016118	0.127585
27	1	0	-4.863060	0.621325	-0.774195
28	1	0	-4.441183	-0.595232	-1.996421

29	1	0	-2.991584	-0.394639	0.655435
30	1	0	-0.863622	-1.368503	1.441035
31	1	0	-0.814267	-2.765350	-0.988671
32	1	0	4.286599	-0.640345	-2.604407
33	1	0	6.664385	-0.652797	-1.900024
34	1	0	7.234441	-0.644994	0.511422
35	1	0	5.433818	-0.621915	2.210716
36	1	0	3.065387	-0.616903	1.509449
37	1	0	-4.053143	-2.609030	2.147665
38	8	0	-3.761255	-3.079186	1.358766
39	1	0	-2.841551	-2.788689	1.236686
40	1	0	-1.010966	4.108255	0.295784
41	8	0	-1.040339	4.115105	-0.677990
42	1	0	-0.587132	4.912593	-0.969796
43	8	0	-1.180634	3.117248	1.896370
44	1	0	-0.311222	2.689051	1.796188
45	1	0	-1.795187	2.550807	1.406833
46	1	0	1.232959	1.896687	0.452812
47	8	0	1.402864	2.036560	1.400684
48	1	0	2.039976	2.757825	1.446153
49	1	0	0.949571	-0.701952	1.586958
50	8	0	0.547309	-0.452400	2.442759
51	1	0	0.673377	0.507699	2.437727

Structure 54·5H_2O (M06-2X/def2-TZVP, Gas Phase)

Energy (Hartrees): = -1318.686836
No imaginary frequencies

Standard orientation:

Center Number	Atomic Number	Atomic Type	Coordinates (Angstroms) X	Y	Z
1	6	0	4.875034	-0.194168	-1.037055
2	6	0	3.741764	-0.720844	-0.424879
3	6	0	3.869332	-1.406460	0.784328
4	6	0	5.114412	-1.556863	1.368221
5	6	0	6.243480	-1.025596	0.751719
6	6	0	6.123600	-0.345153	-0.450418
7	6	0	2.427306	-0.514209	-1.054343
8	7	0	1.347117	-0.888955	-0.514529
9	6	0	0.093649	-0.540175	-1.150925
10	6	0	-0.401654	0.769968	-0.536692
11	6	0	-1.849915	1.067993	-0.905559
12	6	0	-2.704649	-0.151929	-0.587517
13	8	0	-2.195664	-1.288876	-1.278104
14	6	0	-0.911635	-1.672781	-0.918064
15	8	0	0.501873	1.770127	-0.971074
16	8	0	-0.905254	-2.069201	0.432729
17	6	0	-4.142345	0.003637	-1.032029
18	8	0	-4.976226	-0.997957	-0.489129
19	8	0	-2.351521	2.138383	-0.130277
20	1	0	2.429740	0.016985	-2.012792
21	1	0	0.202378	-0.384873	-2.230988
22	1	0	0.142750	2.664502	-0.818068
23	1	0	-0.360989	0.673573	0.557047
24	1	0	-2.009592	2.983421	-0.457119
25	1	0	-1.915236	1.290301	-1.979149
26	1	0	-4.484296	-1.839173	-0.428421
27	1	0	-4.517250	0.968026	-0.690361
28	1	0	-4.172153	-0.020938	-2.127666
29	1	0	-2.681658	-0.327088	0.495116
30	1	0	0.004302	-1.985114	0.751571
31	1	0	-0.670951	-2.524865	-1.559315
32	1	0	4.774303	0.340162	-1.974687
33	1	0	7.000405	0.069585	-0.929866
34	1	0	7.216060	-1.144334	1.211677
35	1	0	5.210787	-2.090484	2.304822
36	1	0	2.983161	-1.820398	1.248586
37	1	0	-3.942274	-2.603254	1.597252
38	8	0	-3.633518	-3.091500	0.818859
39	1	0	-2.680262	-2.937167	0.780581
40	1	0	-0.441780	4.082154	0.649799
41	8	0	-0.562841	4.261577	-0.314014
42	1	0	-0.273285	5.155058	-0.508295
43	8	0	-0.409805	3.114953	2.063548
44	1	0	0.400350	2.573543	2.082892
45	1	0	-1.145531	2.514138	1.888621
46	1	0	1.717092	1.722947	0.490837
47	8	0	1.965729	1.768772	1.430197
48	1	0	2.448712	0.957786	1.616211
49	8	0	-4.793904	-0.950316	2.267062
50	1	0	-5.080155	-0.822844	1.343341
51	1	0	-5.565671	-0.817187	2.820963

Structure 54·5H_2O (M06-2X/def2-TZVP, DMSO)

Energy (Hartrees): = -1318.723120
No imaginary frequencies

Standard orientation:

Center Number	Atomic Number	Atomic Type	Coordinates (Angstroms) X	Y	Z
1	6	0	4.947151	-0.401813	-1.166506
2	6	0	3.813430	-0.689417	-0.410098
3	6	0	3.957313	-1.131569	0.906792
4	6	0	5.220166	-1.279382	1.453876
5	6	0	6.350147	-0.988693	0.693314
6	6	0	6.213410	-0.550693	-0.616544
7	6	0	2.489942	-0.497905	-1.023978
8	7	0	1.408084	-0.703572	-0.399050
9	6	0	0.156401	-0.433502	-1.076377
10	6	0	-0.434491	0.848963	-0.496869
11	6	0	-1.877877	1.063915	-0.929020
12	6	0	-2.676030	-0.193869	-0.613480
13	8	0	-2.083993	-1.323903	-1.248977
14	6	0	-0.784962	-1.618976	-0.845173
15	8	0	0.425274	1.903188	-0.894619
16	8	0	-0.803202	-1.992551	0.515559
17	6	0	-4.099466	-0.134244	-1.114081
18	8	0	-4.863712	-1.231769	-0.636531
19	8	0	-2.463269	2.122369	-0.187211
20	1	0	2.490732	-0.152523	-2.062789
21	1	0	0.284003	-0.302569	-2.156970
22	1	0	0.003177	2.771207	-0.755796
23	1	0	-0.438778	0.756806	0.598296
24	1	0	-2.165316	2.975401	-0.536860
25	1	0	-1.920051	1.268626	-2.005557
26	1	0	-4.282424	-2.007743	-0.545996
27	1	0	-4.574314	0.779255	-0.757226
28	1	0	-4.093070	-0.121664	-2.209232
29	1	0	-2.682461	-0.338687	0.474367
30	1	0	0.073423	-1.801620	0.883494
31	1	0	-0.471257	-2.466282	-1.458995
32	1	0	4.831511	-0.056769	-2.187805
33	1	0	7.090616	-0.322207	-1.208599
34	1	0	7.336272	-1.105036	1.125721
35	1	0	5.329195	-1.623618	2.474675
36	1	0	3.074947	-1.362090	1.492183
37	1	0	-3.871643	-2.696503	1.537655
38	8	0	-3.416432	-3.220561	0.859234
39	1	0	-2.506875	-2.887842	0.839031
40	1	0	-0.687402	4.251859	0.623171
41	8	0	-0.811405	4.362255	-0.341398
42	1	0	-0.538390	5.251981	-0.590883
43	8	0	-0.666698	3.138006	2.069655
44	1	0	0.162852	2.624549	2.057195
45	1	0	-1.350336	2.545453	1.725607
46	1	0	1.596005	1.840907	0.614388
47	8	0	1.798108	1.822036	1.566781
48	1	0	1.957854	0.888655	1.757229
49	8	0	-5.004052	-1.243482	2.139762
50	1	0	-5.177317	-1.092318	1.192375
51	1	0	-5.863285	-1.333824	2.565493

Structure 54·5H_2O (M06-2X/def2-TZVP, H_2O)

Energy (Hartrees): = -1318.739212
No imaginary frequencies

Standard orientation:

Center Number	Atomic Number	Atomic Type	Coordinates (Angstroms) X	Y	Z
1	6	0	4.925048	-0.476376	-1.219968
2	6	0	3.826442	-0.683734	-0.388330
3	6	0	4.031848	-0.980544	0.960428
4	6	0	5.319234	-1.079418	1.459977
5	6	0	6.413109	-0.881248	0.621655
6	6	0	6.215743	-0.578347	-0.718430
7	6	0	2.479937	-0.566151	-0.968538
8	7	0	1.419947	-0.862011	-0.337770
9	6	0	0.156712	-0.612572	-1.002446

10	6	0	-0.417717	0.705758	-0.489575
11	6	0	-1.842062	0.914610	-0.982257
12	6	0	-2.693014	-0.305886	-0.655004
13	8	0	-2.090716	-1.492574	-1.168894
14	6	0	-0.798482	-1.766082	-0.701329
15	8	0	0.456667	1.731090	-0.931645
16	8	0	-0.855101	-2.032529	0.682909
17	6	0	-4.058322	-0.218192	-1.299173
18	8	0	-4.903604	-1.288719	-0.897938
19	8	0	-2.444345	2.019494	-0.323065
20	1	0	2.442764	-0.202980	-2.000533
21	1	0	0.267328	-0.539203	-2.090081
22	1	0	0.072326	2.612996	-0.752782
23	1	0	-0.446540	0.681388	0.608023
24	1	0	-2.092779	2.845848	-0.687527
25	1	0	-1.831349	1.073894	-2.066763
26	1	0	-4.427814	-2.119861	-1.030499
27	1	0	-4.550439	0.703039	-0.992514
28	1	0	-3.941033	-0.214033	-2.387092
29	1	0	-2.800979	-0.386623	0.433188
30	1	0	-0.017872	-1.750664	1.081609
31	1	0	-0.484718	-2.663015	-1.238075
32	1	0	4.760717	-0.237094	-2.264454
33	1	0	7.063694	-0.419194	-1.372080
34	1	0	7.417865	-0.959914	1.017362
35	1	0	5.475849	-1.307600	2.506579
36	1	0	3.181900	-1.125796	1.613947
37	1	0	-3.929982	-2.108938	1.848050
38	8	0	-3.359668	-2.889023	1.744331
39	1	0	-2.518789	-2.548848	1.399668
40	1	0	-0.660794	4.155942	0.505032
41	8	0	-0.669268	4.255317	-0.465257
42	1	0	-0.144688	5.036691	-0.673778
43	8	0	-0.849526	3.044648	2.037234
44	1	0	0.024922	2.620006	1.949107
45	1	0	-1.448319	2.506696	1.495877
46	1	0	1.568303	1.866190	0.629405
47	8	0	1.767093	1.966402	1.577604
48	1	0	2.423157	2.671836	1.626214
49	8	0	-5.221942	-0.697357	1.802218
50	1	0	-5.279725	-0.920746	0.853484
51	1	0	-6.050204	-1.000835	2.190989

Structure 54.5H_2O* (M06-2X, Gas Phase)

Energy (Hartrees): = - 1318.5322242
No imaginary frequencies

Standard orientation:

Center Number	Atomic Number	Atomic Type	Coordinates (Angstroms) X	Y	Z
1	6	0	4.881886	-0.057688	-1.034695
2	6	0	3.765601	-0.636011	-0.431692
3	6	0	3.918883	-1.350282	0.760737
4	6	0	5.171774	-1.475038	1.339741
5	6	0	6.283218	-0.889865	0.734527
6	6	0	6.138106	-0.182839	-0.452692
7	6	0	2.441210	-0.458313	-1.053722
8	7	0	1.372234	-0.858373	-0.502103
9	6	0	0.108304	-0.548590	-1.147697
10	6	0	-0.427344	0.752667	-0.545300
11	6	0	-1.882518	1.004073	-0.929609
12	6	0	-2.703606	-0.238678	-0.601902
13	8	0	-2.157036	-1.373354	-1.276243
14	6	0	-0.863468	-1.708867	-0.898314
15	8	0	0.456940	1.772805	-0.978133
16	8	0	-0.854832	-2.064733	0.465802
17	6	0	-4.144961	-0.128663	-1.056596
18	8	0	-4.973068	-1.106383	-0.457847
19	8	0	-2.427625	2.061792	-0.161939
20	1	0	2.423854	0.072051	-2.013405
21	1	0	0.215798	-0.400457	-2.229888
22	1	0	0.067271	2.654925	-0.831931
23	1	0	-0.393881	0.663677	0.548703
24	1	0	-2.092853	2.911263	-0.483930
25	1	0	-1.946025	1.219033	-2.005998
26	1	0	-4.465800	-1.932919	-0.343321
27	1	0	-4.527996	0.851066	-0.767793
28	1	0	-4.168743	-0.215918	-2.149990
29	1	0	-2.680493	-0.396795	0.482792
30	1	0	0.055787	-1.968839	0.773445
31	1	0	-0.590997	-2.569676	-1.515471
32	1	0	4.762477	0.495816	-1.960167
33	1	0	7.000767	0.271782	-0.924019

34	1	0	7.261221	-0.988819	1.190188
35	1	0	5.287827	-2.029882	2.262827
36	1	0	3.047787	-1.810032	1.212737
37	1	0	-3.770304	-2.443646	1.667274
38	8	0	-3.545435	-3.025570	0.926417
39	1	0	-2.591378	-2.921805	0.824825
40	1	0	-0.603058	3.990378	0.646040
41	8	0	-0.702382	4.180074	-0.318065
42	1	0	-0.444079	5.086553	-0.489790
43	8	0	-0.611225	2.985908	2.012596
44	1	0	0.195530	2.444627	2.071691
45	1	0	-1.332589	2.386318	1.784285
46	1	0	1.627985	1.730149	0.535127
47	8	0	1.784623	1.659642	1.490206
48	1	0	2.141467	0.775742	1.612758
49	8	0	-4.486145	-0.731782	2.212241
50	1	0	-4.927092	-0.738210	1.343831
51	1	0	-5.151163	-0.486388	2.856917

Structure 54.5H_2O* (M06-2X, DMSO)

Energy (Hartrees): = - 1318.5686685
No imaginary frequencies

Standard orientation:

Center Number	Atomic Number	Atomic Type	Coordinates (Angstroms) X	Y	Z
1	6	0	4.951932	-0.326589	-1.179784
2	6	0	3.822667	-0.627098	-0.416417
3	6	0	3.976565	-1.054734	0.907237
4	6	0	5.245264	-1.174755	1.454569
5	6	0	6.370667	-0.870816	0.687624
6	6	0	6.223734	-0.447878	-0.629127
7	6	0	2.491476	-0.467916	-1.028702
8	7	0	1.416659	-0.677600	-0.387020
9	6	0	0.155045	-0.447304	-1.068894
10	6	0	-0.467737	0.826795	-0.500265
11	6	0	-1.922490	0.994914	-0.923520
12	6	0	-2.685662	-0.285842	-0.604920
13	8	0	-2.059418	-1.408038	-1.230086
14	6	0	-0.752905	-1.655110	-0.813094
15	8	0	0.363309	1.897898	-0.918379
16	8	0	-0.769453	-1.985186	0.560804
17	6	0	-4.108473	-0.264124	-1.117376
18	8	0	-4.872302	-1.346307	-0.599984
19	8	0	-2.542076	2.029154	-0.170992
20	1	0	2.480369	-0.145086	-2.075716
21	1	0	0.277209	-0.325718	-2.152136
22	1	0	-0.092800	2.751087	-0.799247
23	1	0	-0.461966	0.746654	0.595565
24	1	0	-2.260510	2.890386	-0.514531
25	1	0	-1.976495	1.204369	-1.999866
26	1	0	-4.277720	-2.103109	-0.447968
27	1	0	-4.589627	0.662747	-0.801925
28	1	0	-4.089335	-0.297449	-2.212914
29	1	0	-2.695475	-0.423906	0.483533
30	1	0	0.112237	-1.794441	0.912526
31	1	0	-0.411105	-2.510215	-1.401614
32	1	0	4.828837	0.005610	-2.205548
33	1	0	7.096854	-0.210929	-1.225782
34	1	0	7.360454	-0.966629	1.119268
35	1	0	5.362019	-1.508351	2.479080
36	1	0	3.099105	-1.300497	1.495295
37	1	0	-3.729096	-2.553885	1.601411
38	8	0	-3.365230	-3.157560	0.934702
39	1	0	-2.436202	-2.897695	0.864031
40	1	0	-0.823403	4.164736	0.572553
41	8	0	-0.977618	4.259145	-0.389685
42	1	0	-0.756139	5.156883	-0.656356
43	8	0	-0.749996	3.069279	1.987442
44	1	0	0.080162	2.559386	2.001595
45	1	0	-1.428009	2.457743	1.669982
46	1	0	1.545899	1.869286	0.578646
47	8	0	1.714561	1.781021	1.531502
48	1	0	1.838683	0.830923	1.644686
49	8	0	-4.659021	-0.987741	2.125670
50	1	0	-5.013936	-0.993849	1.219812
51	1	0	-5.419157	-0.935428	2.712826

Structure 54.5H_2O* (M06-2X, H_2O)

Energy (Hartrees): = - 1318.5860591
No imaginary frequencies

Standard orientation:

Center Number	Atomic Number	Atomic Type	Coordinates (Angstroms) X	Y	Z
1	6	0	4.933697	-0.531088	-1.200685
2	6	0	3.820347	-0.664406	-0.368947
3	6	0	4.002816	-0.871138	1.002539
4	6	0	5.284555	-0.960232	1.525721
5	6	0	6.393856	-0.840463	0.688215
6	6	0	6.218193	-0.623531	-0.674586
7	6	0	2.476874	-0.572976	-0.966430
8	7	0	1.417908	-0.864823	-0.327278
9	6	0	0.153005	-0.640738	-1.003930
10	6	0	-0.422705	0.692791	-0.526453
11	6	0	-1.856988	0.886109	-1.007737
12	6	0	-2.693039	-0.331268	-0.629738
13	8	0	-2.092629	-1.529857	-1.131027
14	6	0	-0.797609	-1.787643	-0.659568
15	8	0	0.449249	1.705268	-1.007502
16	8	0	-0.858294	-1.996735	0.735814
17	6	0	-4.084018	-0.288809	-1.223497
18	8	0	-4.941018	-1.265353	-0.635642
19	8	0	-2.458000	2.000835	-0.359735
20	1	0	2.441673	-0.237603	-2.008837
21	1	0	0.262452	-0.600383	-2.094301
22	1	0	0.058468	2.587876	-0.849373
23	1	0	-0.439398	0.697333	0.571893
24	1	0	-2.098228	2.819388	-0.733760
25	1	0	-1.863978	1.021738	-2.096380
26	1	0	-4.401545	-2.025685	-0.359505
27	1	0	-4.530800	0.689018	-1.042636
28	1	0	-4.008417	-0.450989	-2.304177
29	1	0	-2.765683	-0.382954	0.463391
30	1	0	0.004037	-1.762155	1.107820
31	1	0	-0.483987	-2.703928	-1.164070
32	1	0	4.786800	-0.358804	-2.261935
33	1	0	7.078039	-0.526257	-1.326521
34	1	0	7.393352	-0.912381	1.100979
35	1	0	5.424492	-1.119535	2.588396
36	1	0	3.138294	-0.948002	1.649992
37	1	0	-3.900120	-2.177297	1.717939
38	8	0	-3.526003	-2.930189	1.231292
39	1	0	-2.591242	-2.696548	1.130819
40	1	0	-0.683191	4.106534	0.390235
41	8	0	-0.727235	4.171327	-0.582226
42	1	0	-0.259384	4.973440	-0.835425
43	8	0	-0.833603	3.067920	1.923880
44	1	0	0.028128	2.617225	1.850159
45	1	0	-1.451094	2.505839	1.433436
46	1	0	1.548009	1.866311	0.534791
47	8	0	1.730673	1.938227	1.487223
48	1	0	2.361303	2.662705	1.561873
49	8	0	-4.842818	-0.537837	2.065258
50	1	0	-5.109663	-0.673868	1.139501
51	1	0	-5.640162	-0.705742	2.578537

Structure 54.6H_2O (B3LYP, Gas Phase)

Energy (Hartrees): = - 1395.1250056
No imaginary frequencies

Standard orientation:

Center Number	Atomic Number	Atomic Type	Coordinates (Angstroms) X	Y	Z
1	6	0	5.050805	-0.263700	-1.512644
2	6	0	4.009993	-0.490825	-0.598678
3	6	0	4.326050	-0.844660	0.725807
4	6	0	5.655415	-0.960827	1.117341
5	6	0	6.685821	-0.727724	0.199952
6	6	0	6.381890	-0.378826	-1.116258
7	6	0	2.624457	-0.352108	-1.067638
8	7	0	1.599864	-0.460544	-0.312364
9	6	0	0.278189	-0.319002	-0.901672
10	6	0	-0.492970	0.845468	-0.261506
11	6	0	-1.902922	0.964577	-0.862259
12	6	0	-2.608412	-0.388927	-0.705333
13	8	0	-1.821386	-1.405132	-1.343506
14	6	0	-0.553636	-1.624416	-0.755938
15	8	0	0.283246	2.035977	-0.429479
16	8	0	-0.691937	-2.110800	0.549478
17	6	0	-3.996393	-0.462071	-1.327104
18	8	0	-4.744933	-1.570764	-0.850757

19	8	0	-2.651760	1.959470	-0.163665
20	1	0	2.507882	-0.138971	-2.140398
21	1	0	0.347774	-0.123563	-1.982892
22	1	0	-0.261591	2.779608	-0.101910
23	1	0	-0.617842	0.638589	0.810068
24	1	0	-2.650588	2.798182	-0.676491
25	1	0	-1.822192	1.222228	-1.927416
26	1	0	-4.139618	-2.339691	-0.702537
27	1	0	-4.549253	0.445236	-1.068258
28	1	0	-3.887550	-0.503691	-2.421670
29	1	0	-2.699982	-0.605535	0.367347
30	1	0	-0.239062	-1.578525	1.243674
31	1	0	-0.100833	-2.406062	-1.377158
32	1	0	4.810933	0.006306	-2.537986
33	1	0	7.178303	-0.199363	-1.831926
34	1	0	7.721772	-0.821991	0.511797
35	1	0	5.892308	-1.238945	2.139846
36	1	0	3.529417	-1.033218	1.437721
37	1	0	-3.806866	-3.126543	1.194863
38	8	0	-3.312503	-3.508009	0.442560
39	1	0	-2.403618	-3.178823	0.552894
40	1	0	-1.790950	4.652134	-0.188591
41	8	0	-2.282944	4.534018	-1.023487
42	1	0	-3.056586	5.106817	-0.948247
43	8	0	-1.149544	3.752432	1.371922
44	1	0	-0.432548	3.429313	1.951004
45	1	0	-1.788430	3.018544	1.303052
46	1	0	1.135162	2.116419	1.242024
47	8	0	1.069014	2.219422	2.217416
48	1	0	1.928808	2.545136	2.512403
49	1	0	1.413250	-0.643905	1.509325
50	8	0	0.925467	-0.750926	2.357188
51	1	0	0.805978	0.167254	2.653140
52	8	0	-5.108987	-1.877134	1.883214
53	1	0	-5.162861	-1.591829	0.944732
54	1	0	-5.919838	-2.381486	2.026649

Structure 54.6H₂O (B3LYP, DMSO)

Energy (Hartrees): = - 1395.1624748
No imaginary frequencies

Standard orientation:

Center Number	Atomic Number	Atomic Type	Coordinates (Angstroms) X	Y	Z
1	6	0	5.031127	-0.405616	-1.537015
2	6	0	4.001608	-0.493573	-0.585213
3	6	0	4.334260	-0.701235	0.766173
4	6	0	5.666999	-0.817267	1.148136
5	6	0	6.685644	-0.730261	0.191750
6	6	0	6.365830	-0.523873	-1.151354
7	6	0	2.615165	-0.360724	-1.053234
8	7	0	1.589724	-0.425676	-0.290065
9	6	0	0.273625	-0.286641	-0.901714
10	6	0	-0.511872	0.878475	-0.277346
11	6	0	-1.920006	0.977480	-0.888233
12	6	0	-2.611590	-0.377941	-0.701445
13	8	0	-1.826951	-1.405585	-1.327722
14	6	0	-0.541022	-1.599326	-0.765299
15	8	0	0.251606	2.074688	-0.455877
16	8	0	-0.640470	-2.115587	0.540752
17	6	0	-4.002092	-0.477785	-1.306550
18	8	0	-4.708630	-1.629870	-0.841508
19	8	0	-2.688750	1.975668	-0.213763
20	1	0	2.499166	-0.195865	-2.132324
21	1	0	0.359580	-0.096447	-1.980853
22	1	0	-0.280023	2.805553	-0.082043
23	1	0	-0.643850	0.687434	0.796466
24	1	0	-2.641124	2.819333	-0.719105
25	1	0	-1.841175	1.212158	-1.957936
26	1	0	-4.066822	-2.364618	-0.699201
27	1	0	-4.585606	0.401672	-1.023299
28	1	0	-3.915853	-0.495730	-2.402674
29	1	0	-2.689498	-0.573377	0.377311
30	1	0	-0.279673	-1.517077	1.235023
31	1	0	-0.083327	-2.367976	-1.396497
32	1	0	4.777903	-0.243962	-2.581459
33	1	0	7.152986	-0.455403	-1.896265
34	1	0	7.724294	-0.822534	0.495725
35	1	0	5.916021	-0.977474	2.192996
36	1	0	3.549688	-0.770705	1.512000
37	1	0	-3.734026	-3.163480	1.194122
38	8	0	-3.197313	-3.558074	0.477180
39	1	0	-2.326039	-3.127150	0.558525

40	1	0	-1.777350	4.657915	-0.239786
41	8	0	-2.232917	4.529681	-1.093009
42	1	0	-3.019121	5.093988	-1.043470
43	8	0	-1.187032	3.762013	1.382243
44	1	0	-0.467831	3.406477	1.942610
45	1	0	-1.829910	3.034436	1.292321
46	1	0	1.143462	2.139006	1.283986
47	8	0	1.004254	2.223499	2.250374
48	1	0	1.827149	2.594542	2.604300
49	1	0	1.374685	-0.630381	1.530635
50	8	0	0.871123	-0.696228	2.377302
51	1	0	0.770546	0.241712	2.622350
52	8	0	-5.067655	-1.984968	1.895801
53	1	0	-5.120814	-1.684919	0.961855
54	1	0	-5.865672	-2.519555	2.019010

Structure 54.6H_2O (B3LYP, H_2O)

Energy (Hartrees): = - 1395.1841372
No imaginary frequencies

Standard orientation:

Center Number	Atomic Number	Atomic Type	Coordinates (Angstroms) X	Y	Z
1	6	0	5.015451	-0.308669	-1.518499
2	6	0	3.980794	-0.494190	-0.586675
3	6	0	4.307259	-0.790767	0.749849
4	6	0	5.639532	-0.899242	1.135601
5	6	0	6.663648	-0.713824	0.199390
6	6	0	6.349711	-0.417896	-1.128335
7	6	0	2.596088	-0.365622	-1.059629
8	7	0	1.565116	-0.527995	-0.317054
9	6	0	0.254775	-0.352481	-0.932352
10	6	0	-0.497525	0.840848	-0.315503
11	6	0	-1.902817	0.961744	-0.927759
12	6	0	-2.631187	-0.370727	-0.719701
13	8	0	-1.877606	-1.427067	-1.345605
14	6	0	-0.583969	-1.646462	-0.806333
15	8	0	0.297206	2.010503	-0.529158
16	8	0	-0.659572	-2.191991	0.494852
17	6	0	-4.023478	-0.426898	-1.324997
18	8	0	-4.777923	-1.549319	-0.851695
19	8	0	-2.657214	1.989947	-0.285133
20	1	0	2.485748	-0.118286	-2.123123
21	1	0	0.349562	-0.161938	-2.010789
22	1	0	-0.164907	2.753519	-0.100379
23	1	0	-0.624096	0.678052	0.761740
24	1	0	-2.480082	2.844772	-0.743169
25	1	0	-1.816210	1.169582	-2.002444
26	1	0	-4.163127	-2.297901	-0.687707
27	1	0	-4.576002	0.473950	-1.050467
28	1	0	-3.936365	-0.459549	-2.419907
29	1	0	-2.709315	-0.561561	0.359206
30	1	0	-0.381678	-1.561812	1.194173
31	1	0	-0.151548	-2.416517	-1.451043
32	1	0	4.766232	-0.078392	-2.550699
33	1	0	7.141007	-0.272621	-1.857210
34	1	0	7.701486	-0.799264	0.507015
35	1	0	5.883909	-1.127622	2.168528
36	1	0	3.518017	-0.930575	1.480593
37	1	0	-3.778111	-3.031347	1.214536
38	8	0	-3.267924	-3.519561	0.536409
39	1	0	-2.390546	-3.091139	0.559797
40	1	0	-1.567746	4.570798	-0.223483
41	8	0	-1.984694	4.546277	-1.107935
42	1	0	-2.814600	5.031325	-0.987168
43	8	0	-1.155334	3.781066	1.481214
44	1	0	-0.418081	3.328508	1.944309
45	1	0	-1.785020	3.065023	1.283611
46	1	0	1.169239	2.054069	1.443607
47	8	0	0.936682	2.138029	2.386245
48	1	0	1.720846	2.531551	2.797303
49	1	0	1.320937	-0.722209	1.514785
50	8	0	0.868762	-0.754231	2.391719
51	1	0	0.785613	0.196737	2.601688
52	8	0	-4.997694	-1.730426	1.941409
53	1	0	-5.091420	-1.506425	0.990161
54	1	0	-5.756753	-2.305458	2.116998

Structure 54.6H_2O (M06-2X, Gas Phase)

Energy (Hartrees): = - 1394.9700388
No imaginary frequencies

Standard orientation:

Center Number	Atomic Number	Atomic Type	Coordinates (Angstroms) X	Y	Z
1	6	0	4.834148	-0.357932	-1.416427
2	6	0	3.756375	-0.672652	-0.587924
3	6	0	3.993510	-1.021696	0.746692
4	6	0	5.291938	-1.060565	1.231640
5	6	0	6.364146	-0.750134	0.396437
6	6	0	6.135004	-0.398998	-0.928179
7	6	0	2.391174	-0.587145	-1.137998
8	7	0	1.358359	-0.902364	-0.474959
9	6	0	0.063397	-0.610246	-1.060340
10	6	0	-0.432407	0.717464	-0.472361
11	6	0	-1.848653	1.044553	-0.935300
12	6	0	-2.745173	-0.165138	-0.687692
13	8	0	-2.200507	-1.306036	-1.339442
14	6	0	-0.959695	-1.734621	-0.829104
15	8	0	0.525898	1.699861	-0.844556
16	8	0	-1.076266	-2.175792	0.486767
17	6	0	-4.142956	0.025710	-1.245388
18	8	0	-5.060974	-0.916698	-0.732181
19	8	0	-2.377767	2.126379	-0.188044
20	1	0	2.310737	-0.196062	-2.159899
21	1	0	0.136398	-0.470598	-2.147689
22	1	0	0.121645	2.585886	-0.877886
23	1	0	-0.466247	0.634013	0.622555
24	1	0	-2.078815	2.960039	-0.577778
25	1	0	-1.835342	1.276130	-2.009922
26	1	0	-4.605859	-1.772120	-0.597460
27	1	0	-4.498571	1.019442	-0.968398
28	1	0	-4.084304	-0.042749	-2.339195
29	1	0	-2.816601	-0.337113	0.394984
30	1	0	-0.672274	-1.592172	1.153839
31	1	0	-0.695385	-2.598399	-1.441009
32	1	0	4.649384	-0.081385	-2.449316
33	1	0	6.966126	-0.156809	-1.579111
34	1	0	7.376475	-0.783269	0.780971
35	1	0	5.471910	-1.336295	2.263738
36	1	0	3.163580	-1.267103	1.397847
37	1	0	-4.110509	-2.426102	1.419101
38	8	0	-3.856053	-2.957972	0.651287
39	1	0	-2.891862	-2.890893	0.626807
40	1	0	-0.537652	4.144907	0.341020
41	8	0	-0.649562	4.194532	-0.636040
42	1	0	-0.424150	5.075302	-0.938438
43	8	0	-0.553916	3.274721	1.823837
44	1	0	0.237876	2.740457	1.992439
45	1	0	-1.276350	2.654846	1.658501
46	1	0	1.576324	1.642486	0.644314
47	8	0	1.724659	1.625647	1.609701
48	1	0	2.671577	1.698612	1.750473
49	1	0	1.134528	-1.126364	1.400573
50	8	0	0.671997	-0.962935	2.241846
51	1	0	0.893890	-0.038361	2.409226
52	8	0	-4.754574	-0.671298	1.982426
53	1	0	-5.124204	-0.618777	1.083069
54	1	0	-5.449166	-0.395877	2.582219

Structure 54.6H_2O (M06-2X, DMSO)

Energy (Hartrees): = - 1395.0100641
No imaginary frequencies

Standard orientation:

Center Number	Atomic Number	Atomic Type	Coordinates (Angstroms) X	Y	Z
1	6	0	4.922415	-0.682166	-1.467684
2	6	0	3.895300	-0.626427	-0.523118
3	6	0	4.212105	-0.609358	0.839950
4	6	0	5.538127	-0.656803	1.244238
5	6	0	6.559084	-0.720709	0.295636
6	6	0	6.250923	-0.733453	-1.060572
7	6	0	2.506936	-0.578141	-1.012536
8	7	0	1.491586	-0.660569	-0.254577
9	6	0	0.186548	-0.542605	-0.882387
10	6	0	-0.484062	0.764462	-0.451518
11	6	0	-1.879176	0.867489	-1.063532
12	6	0	-2.666927	-0.395657	-0.727174
13	8	0	-1.964084	-1.555364	-1.163140
14	6	0	-0.708527	-1.745881	-0.553144
15	8	0	0.381624	1.819236	-0.844046
16	8	0	-0.868438	-2.011098	0.812674

17	6	0	-4.014059	-0.443591	-1.416045
18	8	0	-4.830954	-1.487608	-0.902892
19	8	0	-2.598645	1.960331	-0.511025
20	1	0	2.393595	-0.464107	-2.096694
21	1	0	0.277116	-0.517288	-1.975747
22	1	0	-0.119777	2.645871	-0.974384
23	1	0	-0.607891	0.775771	0.640857
24	1	0	-2.333726	2.773329	-0.967400
25	1	0	-1.792853	0.970378	-2.153206
26	1	0	-4.255511	-2.214490	-0.601747
27	1	0	-4.538788	0.499232	-1.255895
28	1	0	-3.850126	-0.579115	-2.491616
29	1	0	-2.816662	-0.432648	0.360852
30	1	0	-0.455225	-1.348816	1.399646
31	1	0	-0.300562	-2.639764	-1.028370
32	1	0	4.674046	-0.687200	-2.524173
33	1	0	7.042558	-0.781268	-1.799112
34	1	0	7.593591	-0.757162	0.617444
35	1	0	5.780040	-0.640921	2.300585
36	1	0	3.422084	-0.550274	1.579300
37	1	0	-3.968693	-2.466184	1.526496
38	8	0	-3.518137	-3.115535	0.964504
39	1	0	-2.592299	-2.829768	0.965850
40	1	0	-1.094566	4.274195	-0.020904
41	8	0	-1.078654	4.160019	-0.993529
42	1	0	-0.873157	5.004565	-1.406506
43	8	0	-1.262158	3.494887	1.581596
44	1	0	-0.419068	3.075159	1.820886
45	1	0	-1.833860	2.773488	1.284199
46	1	0	1.245097	2.087664	0.774022
47	8	0	1.225253	2.197421	1.743898
48	1	0	2.045648	2.637824	1.992448
49	1	0	1.280409	-0.648355	1.622619
50	8	0	0.759764	-0.505099	2.437176
51	1	0	0.792404	0.459760	2.514721
52	8	0	-4.994939	-0.880506	1.781247
53	1	0	-5.221759	-0.978528	0.840162
54	1	0	-5.830358	-0.813787	2.253316

Structure 54.6H_2O (M06-2X, H_2O)

Energy (Hartrees): = - 1395.0320644
No imaginary frequencies

Standard orientation:

Center Number	Atomic Number	Atomic Type	Coordinates (Angstroms) X	Y	Z
1	6	0	4.877573	-0.625029	-1.422667
2	6	0	3.807329	-0.654757	-0.526128
3	6	0	4.058599	-0.711293	0.849489
4	6	0	5.365640	-0.752481	1.313502
5	6	0	6.430499	-0.737205	0.412641
6	6	0	6.186352	-0.673237	-0.955366
7	6	0	2.440104	-0.606642	-1.070718
8	7	0	1.404145	-0.837092	-0.370641
9	6	0	0.116713	-0.634784	-1.014035
10	6	0	-0.452472	0.716691	-0.565781
11	6	0	-1.868935	0.919334	-1.094331
12	6	0	-2.721474	-0.292934	-0.737068
13	8	0	-2.118593	-1.486968	-1.238049
14	6	0	-0.848243	-1.783383	-0.698666
15	8	0	0.450984	1.707161	-1.033592
16	8	0	-0.939085	-2.114097	0.665204
17	6	0	-4.101550	-0.231376	-1.356176
18	8	0	-4.981355	-1.203446	-0.797586
19	8	0	-2.482191	2.043785	-0.477048
20	1	0	2.361108	-0.360358	-2.135277
21	1	0	0.215150	-0.601080	-2.106152
22	1	0	0.058177	2.596322	-0.923125
23	1	0	-0.504388	0.747780	0.531463
24	1	0	-2.108379	2.855397	-0.853635
25	1	0	-1.839524	1.044622	-2.183878
26	1	0	-4.460339	-1.981172	-0.534110
27	1	0	-4.543273	0.748570	-1.175953
28	1	0	-4.003132	-0.383514	-2.436658
29	1	0	-2.812480	-0.352726	0.356307
30	1	0	-0.711017	-1.370004	1.247170
31	1	0	-0.528822	-2.686018	-1.218275
32	1	0	4.677445	-0.569329	-2.487556
33	1	0	7.012892	-0.656896	-1.655492
34	1	0	7.449440	-0.769819	0.780092
35	1	0	5.558107	-0.792251	2.378971
36	1	0	3.231981	-0.714105	1.549653
37	1	0	-4.015277	-2.205262	1.540490

38	8	0	-3.651982	-2.950021	1.034379
39	1	0	-2.715486	-2.719240	0.925911
40	1	0	-0.732292	4.172427	0.238098
41	8	0	-0.742685	4.183000	-0.736596
42	1	0	-0.301244	4.990189	-1.019152
43	8	0	-0.948483	3.172999	1.812595
44	1	0	-0.082727	2.727316	1.785184
45	1	0	-1.540927	2.594454	1.310330
46	1	0	1.493808	1.872809	0.556037
47	8	0	1.583044	1.951658	1.522252
48	1	0	2.373842	2.480131	1.673843
49	1	0	1.130551	-0.857426	1.532260
50	8	0	0.713281	-0.591666	2.374886
51	1	0	0.889275	0.362466	2.368838
52	8	0	-4.912393	-0.534818	1.917632
53	1	0	-5.158113	-0.644929	0.982575
54	1	0	-5.728530	-0.686830	2.405748

Structure 54 min 2 $\Theta_{H\text{-}O\text{-}C1\text{-}C2}$ = 182.65 (M06-2X, Gas Phase)

Energy (Hartrees): = - 936.3467917
No imaginary frequencies

Standard orientation:

Center Number	Atomic Number	Atomic Type	Coordinates (Angstroms) X	Y	Z
1	6	0	1.166946	-1.540238	0.394688
2	6	0	0.414383	-0.205251	0.450782
3	6	0	1.162734	0.808371	-0.415540
4	6	0	2.593448	0.911966	0.072619
5	6	0	3.259316	-0.459451	0.041994
6	1	0	1.158520	0.466016	-1.456167
7	1	0	2.577601	1.272636	1.112180
8	1	0	3.310531	-0.804428	-0.996961
9	1	0	0.452561	0.145533	1.493360
10	8	0	2.494593	-1.366010	0.834384
11	6	0	4.669459	-0.436576	0.615244
12	1	0	4.648528	0.055220	1.598329
13	1	0	4.997335	-1.466998	0.761944
14	8	0	5.580344	0.178297	-0.264282
15	1	0	5.198825	1.024874	-0.521762
16	8	0	3.341817	1.796598	-0.740769
17	1	0	2.818960	2.600824	-0.829594
18	8	0	0.605979	2.106822	-0.308004
19	1	0	-0.249552	2.101634	-0.746524
20	7	0	-0.941240	-0.332872	-0.027845
21	6	0	-3.288433	0.054522	0.322256
22	6	0	-3.670692	-0.479120	-0.911760
23	6	0	-4.259386	0.553834	1.189242
24	6	0	-5.009617	-0.507780	-1.267364
25	1	0	-2.901047	-0.867140	-1.568237
26	6	0	-5.602493	0.524064	0.830996
27	1	0	-3.959671	0.966162	2.147432
28	6	0	-5.977415	-0.006325	-0.397400
29	1	0	-5.306277	-0.921988	-2.223521
30	1	0	-6.352741	0.913440	1.508457
31	1	0	-7.023427	-0.030918	-0.679556
32	6	0	-1.869210	0.094871	0.720873
33	1	0	-1.661395	0.522062	1.713286
34	1	0	0.729327	-2.252545	1.100119
35	8	0	1.110851	-2.015658	-0.916498
36	1	0	1.547696	-2.871994	-0.936306

Structure 54 min 2 $\Theta_{H\text{-}O\text{-}C1\text{-}C2}$= 186.21 (M06-2X, DMSO)

Energy (Hartrees): = - 936.3729984
No imaginary frequencies

Standard orientation:

Center Number	Atomic Number	Atomic Type	Coordinates (Angstroms) X	Y	Z
1	6	0	1.163883	-1.547491	0.377934
2	6	0	0.417774	-0.210096	0.439064
3	6	0	1.166294	0.812973	-0.417071
4	6	0	2.601093	0.904630	0.062298
5	6	0	3.261283	-0.470237	0.034748
6	1	0	1.155934	0.490722	-1.464317
7	1	0	2.598377	1.273324	1.097947
8	1	0	3.317984	-0.818222	-1.003114
9	1	0	0.448284	0.130075	1.483571
10	8	0	2.494786	-1.376702	0.822649

11	6	0	4.663099	-0.451907	0.626034
12	1	0	4.622177	-0.000213	1.626873
13	1	0	5.010200	-1.481665	0.732496
14	8	0	5.581645	0.221269	-0.212510
15	1	0	5.166378	1.056561	-0.458768
16	8	0	3.354514	1.778485	-0.766526
17	1	0	2.865650	2.608998	-0.815861
18	8	0	0.615869	2.113064	-0.288873
19	1	0	-0.234007	2.124160	-0.743278
20	7	0	-0.940188	-0.337655	-0.045750
21	6	0	-3.287889	0.057356	0.311232
22	6	0	-3.688386	-0.455671	-0.927065
23	6	0	-4.249217	0.540301	1.200609
24	6	0	-5.033238	-0.482398	-1.265314
25	1	0	-2.936346	-0.831472	-1.611298
26	6	0	-5.598062	0.512594	0.860125
27	1	0	-3.934476	0.936883	2.160757
28	6	0	-5.990438	0.001680	-0.372594
29	1	0	-5.341970	-0.879484	-2.225494
30	1	0	-6.339357	0.889898	1.554992
31	1	0	-7.040637	-0.020409	-0.640838
32	6	0	-1.865891	0.097442	0.704552
33	1	0	-1.657658	0.527341	1.693082
34	1	0	0.723245	-2.259497	1.080558
35	8	0	1.115059	-2.027047	-0.932273
36	1	0	1.500724	-2.911007	-0.934110

Structure 54 min 2 $\theta_{H\text{-}O\text{-}C1\text{-}C2}$ = 184.55 (M06-2X, Gas Phase)

Energy (Hartrees): = -936.463049
No imaginary frequencies

Standard orientation:

Center Number	Atomic Number	Atomic Type	Coordinates (Angstroms) X	Y	Z
1	6	0	1.143900	-1.525610	0.360234
2	6	0	0.413509	-0.179731	0.425075
3	6	0	1.182661	0.834720	-0.416721
4	6	0	2.609811	0.908864	0.082982
5	6	0	3.253973	-0.470457	0.032079
6	1	0	1.180969	0.511194	-1.462543
7	1	0	2.592920	1.248172	1.128379
8	1	0	3.309220	-0.797596	-1.011862
9	1	0	0.450570	0.152008	1.472835
10	8	0	2.471088	-1.377675	0.800258
11	6	0	4.655018	-0.482999	0.624412
12	1	0	4.628521	-0.014394	1.617093
13	1	0	4.964992	-1.520545	0.753123
14	8	0	5.595123	0.134632	-0.219983
15	1	0	5.241212	0.994323	-0.478531
16	8	0	3.377685	1.798514	-0.704219
17	1	0	2.870083	2.613602	-0.794945
18	8	0	0.639984	2.136487	-0.294999
19	1	0	-0.228501	2.145375	-0.710725
20	7	0	-0.939945	-0.280677	-0.058033
21	6	0	-3.291568	0.045282	0.319792
22	6	0	-3.679087	-0.427780	-0.934193
23	6	0	-4.258536	0.490050	1.216025
24	6	0	-5.017947	-0.449767	-1.280093
25	1	0	-2.914582	-0.776658	-1.616506
26	6	0	-5.601734	0.467869	0.867665
27	1	0	-3.954589	0.855189	2.190561
28	6	0	-5.981292	-0.001725	-0.380705
29	1	0	-5.318538	-0.817882	-2.252498
30	1	0	-6.348866	0.816860	1.568448
31	1	0	-7.028139	-0.020845	-0.655862
32	6	0	-1.873481	0.079516	0.714239
33	1	0	-1.669902	0.440545	1.732314
34	1	0	0.692625	-2.231782	1.062286
35	8	0	1.079244	-2.002304	-0.949558
36	1	0	1.475261	-2.879456	-0.972077

Structure 54 min 2 $\theta_{H\text{-}O\text{-}C1\text{-}C2}$ = 187.92 (M06-2X, DMSO)

Energy (Hartrees): = -936.489243
No imaginary frequencies

Standard orientation:

Center Number	Atomic Number	Atomic Type	Coordinates (Angstroms) X	Y	Z

1	6	0	1.153262	-1.539178	0.361925
2	6	0	0.418076	-0.196847	0.416583
3	6	0	1.177658	0.825416	-0.426327
4	6	0	2.608353	0.905602	0.062893
5	6	0	3.258400	-0.472292	0.028518
6	1	0	1.172190	0.512877	-1.475770
7	1	0	2.601949	1.263462	1.101099
8	1	0	3.320157	-0.813611	-1.010859
9	1	0	0.446951	0.136318	1.462366
10	8	0	2.482727	-1.377700	0.803244
11	6	0	4.652539	-0.472122	0.634601
12	1	0	4.605542	-0.032958	1.639207
13	1	0	4.990990	-1.504681	0.732889
14	8	0	5.591929	0.201944	-0.178093
15	1	0	5.196065	1.046113	-0.431308
16	8	0	3.374056	1.782711	-0.748994
17	1	0	2.899628	2.622863	-0.798027
18	8	0	0.628500	2.124575	-0.295944
19	1	0	-0.237582	2.135440	-0.722241
20	7	0	-0.937723	-0.312225	-0.068495
21	6	0	-3.288673	0.048861	0.306590
22	6	0	-3.700852	-0.441718	-0.933899
23	6	0	-4.240043	0.517003	1.210058
24	6	0	-5.046021	-0.459773	-1.259980
25	1	0	-2.959126	-0.808601	-1.632485
26	6	0	-5.589328	0.498809	0.881984
27	1	0	-3.916255	0.896253	2.172932
28	6	0	-5.992701	0.010846	-0.353104
29	1	0	-5.363987	-0.840609	-2.222588
30	1	0	-6.322791	0.866322	1.588664
31	1	0	-7.044153	-0.004498	-0.612559
32	6	0	-1.867525	0.084953	0.693455
33	1	0	-1.660181	0.480494	1.695255
34	1	0	0.705549	-2.240231	1.070122
35	8	0	1.099761	-2.037872	-0.940664
36	1	0	1.454461	-2.936000	-0.933417

Structure 55 (B3LYP, Gas Phase)

Energy (Hartrees): = - 1051.0055976
No imaginary frequencies

Standard orientation:

Center Number	Atomic Number	Atomic Type	Coordinates (Angstroms) X	Y	Z
1	6	0	1.971762	-1.486503	0.568844
2	6	0	1.244877	-0.114114	0.591506
3	6	0	1.994741	0.872218	-0.321570
4	6	0	3.472717	0.910588	0.044994
5	6	0	4.070427	-0.498803	-0.031468
6	1	0	1.906018	0.531619	-1.361498
7	1	0	3.566724	1.271577	1.082374
8	1	0	3.998528	-0.871490	-1.059882
9	1	0	1.271587	0.280781	1.619317
10	8	0	3.336166	-1.349027	0.861113
11	6	0	5.535624	-0.548851	0.409795
12	1	0	5.622569	-0.104110	1.415947
13	1	0	5.835685	-1.597972	0.489860
14	8	0	6.397121	0.068850	-0.526194
15	1	0	6.015485	0.938039	-0.726094
16	8	0	4.184694	1.769115	-0.842760
17	1	0	3.690923	2.602059	-0.866117
18	8	0	1.503682	2.206594	-0.191842
19	1	0	0.643106	2.247013	-0.629203
20	7	0	-0.108197	-0.295195	0.098983
21	6	0	-2.485922	0.064325	0.384814
22	6	0	-2.875554	-0.567766	-0.813775
23	6	0	-3.482675	0.597906	1.210880
24	6	0	-4.209381	-0.659778	-1.162890
25	1	0	-2.108806	-0.982256	-1.459331
26	6	0	-4.832583	0.513251	0.872463
27	1	0	-3.200253	1.089255	2.138787
28	6	0	-5.201225	-0.119902	-0.321479
29	1	0	-4.522561	-1.144320	-2.081572
30	1	0	-5.578032	0.936024	1.534596
31	6	0	-1.082000	0.175667	0.784185
32	1	0	-0.902191	0.700784	1.735273
33	1	0	1.579947	-2.124119	1.374134
34	8	0	1.804018	-2.103010	-0.687138
35	1	0	0.910807	-1.857215	-0.981153
36	8	0	-6.479112	-0.264357	-0.757751
37	6	0	-7.534130	0.256984	0.040650
38	1	0	-7.447262	1.343651	0.161727

39	1	0	-8.455699	0.026833	-0.494736
40	1	0	-7.561816	-0.216448	1.029577

Structure 55 (B3LYP, DMSO)

Energy (Hartrees): = - 1051.03046
No imaginary frequencies

Standard orientation:

Center Number	Atomic Number	Atomic Type	Coordinates (Angstroms) X	Y	Z
1	6	0	1.951295	-1.465284	0.612183
2	6	0	1.246354	-0.084607	0.606387
3	6	0	2.005510	0.888959	-0.314951
4	6	0	3.486936	0.901901	0.037916
5	6	0	4.059753	-0.517293	-0.031508
6	1	0	1.899622	0.553883	-1.355188
7	1	0	3.603094	1.275331	1.067224
8	1	0	3.968992	-0.895243	-1.057343
9	1	0	1.266219	0.321485	1.628140
10	8	0	3.329183	-1.354805	0.875301
11	6	0	5.528582	-0.586100	0.391622
12	1	0	5.636210	-0.147011	1.397134
13	1	0	5.825786	-1.637761	0.459644
14	8	0	6.387752	0.036915	-0.552337
15	1	0	5.988832	0.901970	-0.744759
16	8	0	4.213616	1.732238	-0.871783
17	1	0	3.757802	2.588461	-0.882318
18	8	0	1.539661	2.230961	-0.178563
19	1	0	0.688311	2.295241	-0.636085
20	7	0	-0.104437	-0.273801	0.105008
21	6	0	-2.482093	0.100421	0.360726
22	6	0	-2.870513	-0.575831	-0.814560
23	6	0	-3.481837	0.660064	1.168598
24	6	0	-4.205739	-0.688813	-1.156644
25	1	0	-2.108478	-1.011339	-1.452946
26	6	0	-4.831924	0.554455	0.837509
27	1	0	-3.199323	1.188040	2.075976
28	6	0	-5.201144	-0.127420	-0.331020
29	1	0	-4.511246	-1.210001	-2.058531
30	1	0	-5.577730	0.998223	1.485490
31	6	0	-1.081506	0.227726	0.766301
32	1	0	-0.909328	0.784886	1.697479
33	1	0	1.561946	-2.068896	1.443612
34	8	0	1.747443	-2.126604	-0.619201
35	1	0	0.873895	-1.822623	-0.926028
36	8	0	-6.477726	-0.300573	-0.750745
37	6	0	-7.537153	0.217371	0.059088
38	1	0	-7.487459	1.309578	0.131940
39	1	0	-8.462117	-0.067867	-0.444038
40	1	0	-7.522513	-0.218096	1.064266

Structure 55 (M06-2X, Gas Phase)

Energy (Hartrees): = - 1050.8609313
No imaginary frequencies

Standard orientation:

Center Number	Atomic Number	Atomic Type	Coordinates (Angstroms) X	Y	Z
1	6	0	2.027981	-1.513140	0.603908
2	6	0	1.248555	-0.184812	0.620084
3	6	0	1.925605	0.802337	-0.333730
4	6	0	3.390751	0.929715	0.029916
5	6	0	4.051633	-0.444218	-0.023602
6	1	0	1.855285	0.425034	-1.360392
7	1	0	3.466018	1.316480	1.057287
8	1	0	3.985993	-0.839594	-1.042718
9	1	0	1.285561	0.238538	1.633443
10	8	0	3.377090	-1.304696	0.889098
11	6	0	5.514369	-0.406200	0.396254
12	1	0	5.595991	0.112079	1.362654
13	1	0	5.855130	-1.433107	0.535011
14	8	0	6.328343	0.183187	-0.589804
15	1	0	5.921865	1.022986	-0.829249
16	8	0	4.054775	1.795965	-0.871383
17	1	0	3.526230	2.599391	-0.925931
18	8	0	1.359136	2.099121	-0.238954
19	1	0	0.511223	2.093559	-0.690951
20	7	0	-0.106549	-0.422642	0.169634

21	6	0	-2.459876	0.050371	0.391564
22	6	0	-2.859144	-0.746976	-0.691498
23	6	0	-3.431013	0.737879	1.111462
24	6	0	-4.188450	-0.845876	-1.034442
25	1	0	-2.102183	-1.283003	-1.250743
26	6	0	-4.778873	0.649435	0.776720
27	1	0	-3.135178	1.356057	1.953071
28	6	0	-5.160361	-0.146340	-0.302778
29	1	0	-4.518679	-1.456047	-1.865648
30	1	0	-5.509216	1.195918	1.356597
31	6	0	-1.049230	0.172335	0.777319
32	1	0	-0.844102	0.822469	1.639369
33	1	0	1.664955	-2.172135	1.399033
34	8	0	1.903307	-2.126648	-0.650578
35	1	0	0.990803	-1.985289	-0.931207
36	8	0	-6.433712	-0.307295	-0.721725
37	6	0	-7.453561	0.377496	-0.020952
38	1	0	-7.305503	1.460660	-0.069455
39	1	0	-8.384764	0.116468	-0.517315
40	1	0	-7.495426	0.058918	1.025118

Structure 55 (M06-2X, DMSO)

Energy (Hartrees): = - 1050.8885343
No imaginary frequencies

Standard orientation:

Center Number	Atomic Number	Atomic Type	Coordinates (Angstroms) X	Y	Z
1	6	0	2.009179	-1.495295	0.633719
2	6	0	1.251756	-0.156171	0.644137
3	6	0	1.933300	0.826700	-0.312555
4	6	0	3.405466	0.924183	0.029756
5	6	0	4.040692	-0.460070	-0.037397
6	1	0	1.837177	0.463290	-1.342603
7	1	0	3.509157	1.311673	1.053113
8	1	0	3.950182	-0.849412	-1.057686
9	1	0	1.286376	0.265144	1.657003
10	8	0	3.374157	-1.318228	0.882926
11	6	0	5.509961	-0.446090	0.357629
12	1	0	5.616648	0.055698	1.329269
13	1	0	5.849960	-1.477399	0.470496
14	8	0	6.316117	0.160153	-0.633508
15	1	0	5.893788	0.999324	-0.852597
16	8	0	4.078364	1.773665	-0.887484
17	1	0	3.580154	2.599330	-0.923612
18	8	0	1.394483	2.132709	-0.197879
19	1	0	0.544364	2.151050	-0.651293
20	7	0	-0.102599	-0.394737	0.187552
21	6	0	-2.458071	0.058829	0.393469
22	6	0	-2.846825	-0.744838	-0.689565
23	6	0	-3.439349	0.743581	1.106244
24	6	0	-4.175369	-0.853500	-1.039485
25	1	0	-2.090483	-1.281396	-1.250783
26	6	0	-4.784984	0.645900	0.764357
27	1	0	-3.150079	1.366922	1.946569
28	6	0	-5.157073	-0.156466	-0.315918
29	1	0	-4.488981	-1.470197	-1.873563
30	1	0	-5.522188	1.191018	1.338024
31	6	0	-1.051530	0.196147	0.792242
32	1	0	-0.861048	0.845678	1.655531
33	1	0	1.651382	-2.130169	1.449942
34	8	0	1.843083	-2.139652	-0.603284
35	1	0	0.949374	-1.918440	-0.900330
36	8	0	-6.425544	-0.322562	-0.739129
37	6	0	-7.453210	0.372216	-0.042744
38	1	0	-7.301622	1.453854	-0.096160
39	1	0	-8.382647	0.110777	-0.543839
40	1	0	-7.502268	0.056986	1.003183

Structure 55 (M06-2X/def2-TZVP, Gas Phase)

Energy (Hartrees): = -1050.992904
No imaginary frequencies

Standard orientation:

Center Number	Atomic Number	Atomic Type	Coordinates (Angstroms) X	Y	Z
1	6	0	1.995894	-1.491874	0.590300
2	6	0	1.243838	-0.149638	0.595787
3	6	0	1.949449	0.828014	-0.342636

4	6	0	3.413247	0.923090	0.031310
5	6	0	4.045387	-0.462276	-0.023654
6	1	0	1.876926	0.462060	-1.372549
7	1	0	3.489896	1.300569	1.060723
8	1	0	3.983745	-0.850003	-1.045667
9	1	0	1.277729	0.266731	1.611224
10	8	0	3.346709	-1.312491	0.875431
11	6	0	5.500969	-0.460085	0.417936
12	1	0	5.580017	0.045987	1.389336
13	1	0	5.817600	-1.494458	0.552688
14	8	0	6.350279	0.118533	-0.542886
15	1	0	5.975054	0.970593	-0.794839
16	8	0	4.102096	1.782096	-0.856203
17	1	0	3.592324	2.598046	-0.922150
18	8	0	1.406549	2.132987	-0.243616
19	1	0	0.541614	2.142074	-0.665405
20	7	0	-0.108085	-0.360293	0.133657
21	6	0	-2.466092	0.059916	0.383325
22	6	0	-2.857640	-0.673964	-0.743259
23	6	0	-3.443046	0.684757	1.145570
24	6	0	-4.184232	-0.773285	-1.086108
25	1	0	-2.097406	-1.162421	-1.338595
26	6	0	-4.788781	0.594803	0.812761
27	1	0	-3.152675	1.255634	2.020435
28	6	0	-5.161561	-0.138058	-0.309470
29	1	0	-4.504665	-1.337367	-1.951879
30	1	0	-5.525052	1.091679	1.426561
31	6	0	-1.058902	0.179115	0.773837
32	1	0	-0.863183	0.774771	1.675216
33	1	0	1.617129	-2.133367	1.391841
34	8	0	1.857906	-2.123798	-0.652955
35	1	0	0.962775	-1.936669	-0.965799
36	8	0	-6.434539	-0.293634	-0.728300
37	6	0	-7.460929	0.324355	0.018623
38	1	0	-7.333960	1.409815	0.038041
39	1	0	-8.391436	0.077625	-0.484724
40	1	0	-7.487455	-0.058006	1.042241

Structure 55 (M06-2X/def2-TZVP, DMSO)

Energy (Hartrees): = -1051.020287
No imaginary frequencies

Standard orientation:

Center Number	Atomic Number	Atomic Type	Coordinates (Angstroms) X	Y	Z
1	6	0	1.994092	-1.483176	0.626549
2	6	0	1.249649	-0.138995	0.628640
3	6	0	1.944295	0.841375	-0.317240
4	6	0	3.415107	0.924500	0.028399
5	6	0	4.036469	-0.464005	-0.045386
6	1	0	1.846414	0.486334	-1.349455
7	1	0	3.520731	1.302421	1.053909
8	1	0	3.947742	-0.845438	-1.068507
9	1	0	1.282363	0.276748	1.642929
10	8	0	3.360103	-1.320144	0.865042
11	6	0	5.500994	-0.470821	0.362384
12	1	0	5.605507	0.017367	1.339526
13	1	0	5.829565	-1.505896	0.466210
14	8	0	6.329811	0.136795	-0.607645
15	1	0	5.927496	0.985619	-0.832958
16	8	0	4.099406	1.774996	-0.877541
17	1	0	3.615747	2.610505	-0.915218
18	8	0	1.412291	2.148453	-0.198782
19	1	0	0.537535	2.165154	-0.606655
20	7	0	-0.101962	-0.362793	0.166973
21	6	0	-2.461101	0.048125	0.395273
22	6	0	-2.847973	-0.725858	-0.706371
23	6	0	-3.442322	0.707809	1.125625
24	6	0	-4.173642	-0.828744	-1.058116
25	1	0	-2.092543	-1.245478	-1.282730
26	6	0	-4.785443	0.616809	0.782420
27	1	0	-3.154186	1.307983	1.981686
28	6	0	-5.154321	-0.154652	-0.317011
29	1	0	-4.482530	-1.424118	-1.908179
30	1	0	-5.524019	1.143060	1.369506
31	6	0	-1.057835	0.184056	0.796326
32	1	0	-0.873881	0.793936	1.688235
33	1	0	1.634604	-2.102354	1.453344
34	8	0	1.810438	-2.149149	-0.596070
35	1	0	0.931629	-1.897770	-0.917753
36	8	0	-6.423053	-0.309551	-0.741745
37	6	0	-7.448115	0.375556	-0.037556

38	1	0	-7.288320	1.455832	-0.066536
39	1	0	-8.377586	0.132586	-0.546384
40	1	0	-7.503638	0.040010	1.000524

Structure 55.5H_2O (M06-2X, Gas Phase)

Energy (Hartrees): = - 1433.0446219
No imaginary frequencies

Standard orientation:

Center Number	Atomic Number	Atomic Type	Coordinates (Angstroms) X	Y	Z
1	6	0	3.882888	-0.280594	-1.600939
2	6	0	2.879244	-0.537292	-0.672021
3	6	0	3.240091	-0.742192	0.670474
4	6	0	4.562640	-0.696204	1.052282
5	6	0	5.566425	-0.444754	0.104428
6	6	0	5.223307	-0.235620	-1.230713
7	6	0	1.481340	-0.550001	-1.117862
8	7	0	0.508520	-0.845953	-0.358792
9	6	0	-0.833376	-0.658860	-0.876153
10	6	0	-1.373641	0.672165	-0.340495
11	6	0	-2.828827	0.894678	-0.741443
12	6	0	-3.646290	-0.345664	-0.392453
13	8	0	-3.061014	-1.494290	-0.996085
14	6	0	-1.779337	-1.815447	-0.516406
15	8	0	-0.494334	1.682248	-0.818337
16	8	0	-1.832258	-2.148696	0.838965
17	6	0	-5.080257	-0.280132	-0.883330
18	8	0	-5.894222	-1.241821	-0.268697
19	8	0	-3.376828	1.982777	-0.014729
20	1	0	1.310666	-0.254703	-2.160787
21	1	0	-0.834029	-0.588461	-1.972516
22	1	0	-0.953049	2.539099	-0.886407
23	1	0	-1.345403	0.650575	0.757947
24	1	0	-3.168852	2.808603	-0.473706
25	1	0	-2.884400	1.076534	-1.823821
26	1	0	-5.387240	-2.063531	-0.165610
27	1	0	-5.486081	0.704341	-0.644096
28	1	0	-5.059613	-0.398078	-1.977021
29	1	0	-3.658340	-0.461538	0.699114
30	1	0	-1.334152	-1.555654	1.431661
31	1	0	-1.490547	-2.709495	-1.071960
32	1	0	3.616609	-0.113741	-2.639517
33	1	0	5.977491	-0.039029	-1.979763
34	1	0	4.862325	-0.856441	2.080270
35	1	0	2.475774	-0.942191	1.411731
36	1	0	-4.831709	-3.019363	1.834741
37	8	0	-4.460338	-3.297685	0.994536
38	1	0	-3.534076	-3.016241	1.027566
39	1	0	-1.639073	4.140095	0.266946
40	8	0	-1.810842	4.116270	-0.701762
41	1	0	-1.667294	4.988715	-1.071012
42	8	0	-1.522436	3.352867	1.798713
43	1	0	-0.692594	2.874489	1.951414
44	1	0	-2.208391	2.682588	1.678413
45	1	0	0.637999	1.760287	0.609814
46	8	0	0.834745	1.809510	1.565658
47	1	0	1.778038	1.967215	1.649962
48	1	0	0.435366	-0.962706	1.512435
49	8	0	0.016902	-0.810624	2.380757
50	1	0	0.164546	0.135795	2.502438
51	8	0	6.827975	-0.422343	0.582532
52	6	0	7.880475	-0.189512	-0.333603
53	1	0	7.782527	0.793116	-0.804809
54	1	0	8.797110	-0.222340	0.249450
55	1	0	7.909858	-0.965386	-1.104335

Structure 55.5H_2O (M06-2X, DMSO)

Energy (Hartrees): = - 1433.0851376
No imaginary frequencies

Standard orientation:

Center Number	Atomic Number	Atomic Type	Coordinates (Angstroms) X	Y	Z
1	6	0	3.961106	-0.358499	-1.583530
2	6	0	2.967292	-0.490108	-0.615121
3	6	0	3.347889	-0.559562	0.734816
4	6	0	4.678007	-0.502883	1.090694
5	6	0	5.670969	-0.374003	0.105399

6	6	0	5.307932	-0.301387	-1.240619
7	6	0	1.566254	-0.525852	-1.045603
8	7	0	0.588266	-0.693644	-0.250192
9	6	0	-0.749404	-0.617208	-0.810795
10	6	0	-1.425040	0.677586	-0.349269
11	6	0	-2.864535	0.743532	-0.853308
12	6	0	-3.593280	-0.536191	-0.453696
13	8	0	-2.894280	-1.678935	-0.942005
14	6	0	-1.594689	-1.839377	-0.426440
15	8	0	-0.614323	1.748192	-0.811739
16	8	0	-1.649660	-2.115824	0.947911
17	6	0	-4.996215	-0.623287	-1.018613
18	8	0	-5.770694	-1.618549	-0.386683
19	8	0	-3.561564	1.823153	-0.245840
20	1	0	1.399582	-0.384161	-2.119621
21	1	0	-0.720402	-0.595220	-1.908112
22	1	0	-1.143932	2.559662	-0.920913
23	1	0	-1.469939	0.698487	0.749374
24	1	0	-3.359255	2.637934	-0.729934
25	1	0	-2.867227	0.846789	-1.946228
26	1	0	-5.176765	-2.305165	-0.040532
27	1	0	-5.495232	0.337547	-0.877370
28	1	0	-4.908393	-0.812107	-2.097940
29	1	0	-3.649188	-0.574095	0.643040
30	1	0	-1.238569	-1.430685	1.509294
31	1	0	-1.200355	-2.723516	-0.930395
32	1	0	3.678434	-0.298534	-2.629751
33	1	0	6.052564	-0.198039	-2.018256
34	1	0	4.984132	-0.552794	2.128929
35	1	0	2.595042	-0.653655	1.508723
36	1	0	-4.540309	-3.059843	2.084920
37	8	0	-4.209178	-3.326207	1.221186
38	1	0	-3.309063	-2.963923	1.180802
39	1	0	-2.070905	4.184652	0.074172
40	8	0	-2.143111	4.051494	-0.893072
41	1	0	-1.993803	4.891718	-1.337975
42	8	0	-2.080761	3.416112	1.698980
43	1	0	-1.212735	3.017378	1.877885
44	1	0	-2.655307	2.678947	1.448769
45	1	0	0.374017	2.027035	0.733685
46	8	0	0.428275	2.147196	1.700956
47	1	0	1.264097	2.591705	1.881247
48	1	0	0.477963	-0.682341	1.612528
49	8	0	0.021007	-0.548413	2.467802
50	1	0	0.045913	0.416542	2.542313
51	8	0	6.939678	-0.325378	0.553782
52	6	0	7.978349	-0.182984	-0.408421
53	1	0	7.872064	0.751923	-0.965654
54	1	0	8.907461	-0.163930	0.156972
55	1	0	7.991324	-1.028194	-1.101821

Structure 55.5H_2O (M06-2X, H_2O)

Energy (Hartrees): = - 1433.105282
No imaginary frequencies

Standard orientation:

Center Number	Atomic Number	Atomic Type	Coordinates (Angstroms) X	Y	Z
1	6	0	3.922713	-0.444018	-1.571095
2	6	0	2.903073	-0.516186	-0.623806
3	6	0	3.243894	-0.518003	0.738431
4	6	0	4.565982	-0.464376	1.126193
5	6	0	5.583128	-0.408989	0.161628
6	6	0	5.261066	-0.396156	-1.195339
7	6	0	1.514160	-0.566777	-1.090759
8	7	0	0.522311	-0.802577	-0.327878
9	6	0	-0.802835	-0.683425	-0.913320
10	6	0	-1.400795	0.664310	-0.496132
11	6	0	-2.859157	0.788247	-0.923521
12	6	0	-3.627403	-0.436433	-0.440629
13	8	0	-3.017216	-1.624859	-0.950098
14	6	0	-1.700348	-1.854171	-0.496418
15	8	0	-0.574349	1.664441	-1.073024
16	8	0	-1.683830	-2.149479	0.879785
17	6	0	-5.064845	-0.457791	-0.913663
18	8	0	-5.848592	-1.407295	-0.208381
19	8	0	-3.466126	1.920637	-0.312263
20	1	0	1.369909	-0.384885	-2.161739
21	1	0	-0.763178	-0.698440	-2.009890
22	1	0	-0.968613	2.548441	-0.926544
23	1	0	-1.377675	0.743580	0.598716
24	1	0	-3.139337	2.727303	-0.737582
25	1	0	-2.921373	0.859525	-2.016406

26	1	0	-5.266289	-2.111461	0.126153
27	1	0	-5.509561	0.526159	-0.759641
28	1	0	-5.063349	-0.674066	-1.989421
29	1	0	-3.609438	-0.455151	0.658146
30	1	0	-1.454469	-1.379979	1.427862
31	1	0	-1.381672	-2.760242	-1.010608
32	1	0	3.667485	-0.429817	-2.625624
33	1	0	6.029666	-0.345690	-1.954245
34	1	0	4.845860	-0.462091	2.172899
35	1	0	2.466178	-0.555415	1.491955
36	1	0	-4.605974	-2.684262	2.143523
37	8	0	-4.307934	-3.154050	1.356727
38	1	0	-3.395161	-2.844698	1.228330
39	1	0	-1.726837	4.086909	0.325714
40	8	0	-1.763291	4.110157	-0.647853
41	1	0	-1.317754	4.916063	-0.928116
42	8	0	-1.856932	3.088130	1.915947
43	1	0	-0.978357	2.682153	1.803461
44	1	0	-2.463454	2.509597	1.430355
45	1	0	0.567152	1.930062	0.440012
46	8	0	0.747182	2.070776	1.386040
47	1	0	1.373322	2.801933	1.425100
48	1	0	0.343764	-0.664222	1.557477
49	8	0	-0.048770	-0.436614	2.425062
50	1	0	0.054314	0.526118	2.431121
51	8	0	6.849515	-0.363327	0.644647
52	6	0	7.916978	-0.312623	-0.298295
53	1	0	7.853731	0.591054	-0.909372
54	1	0	8.831255	-0.292401	0.289979
55	1	0	7.912763	-1.197810	-0.938827

Structure 55·5H_2O (M06-2X/def2-TZVP, Gas Phase)

Energy (Hartrees): = -1433.206839
No imaginary frequencies

Standard orientation:

Center Number	Atomic Number	Atomic Type	Coordinates (Angstroms) X	Y	Z
1	6	0	4.211872	-0.093245	-1.449885
2	6	0	3.182422	-0.567335	-0.648783
3	6	0	3.501842	-1.125689	0.595590
4	6	0	4.808631	-1.201220	1.014643
5	6	0	5.838871	-0.720155	0.196413
6	6	0	5.537790	-0.163454	-1.042371
7	6	0	1.795508	-0.447773	-1.107573
8	7	0	0.805650	-0.814109	-0.408279
9	6	0	-0.529622	-0.552813	-0.904704
10	6	0	-1.006995	0.775295	-0.317880
11	6	0	-2.503411	0.992893	-0.504775
12	6	0	-3.253617	-0.238127	-0.012792
13	8	0	-2.779773	-1.397677	-0.692379
14	6	0	-1.444955	-1.704477	-0.482068
15	8	0	-0.217518	1.778324	-0.931250
16	8	0	-1.251313	-2.015486	0.881660
17	6	0	-4.741296	-0.173838	-0.271296
18	8	0	-5.442026	-1.167245	0.446712
19	8	0	-2.940660	2.086813	0.279063
20	1	0	1.653153	0.008961	-2.093428
21	1	0	-0.561997	-0.467369	-1.997494
22	1	0	-0.615569	2.661894	-0.819996
23	1	0	-0.819098	0.757554	0.764849
24	1	0	-2.731225	2.922433	-0.163104
25	1	0	-2.722118	1.154612	-1.568220
26	1	0	-4.893523	-1.966570	0.544334
27	1	0	-5.118154	0.796212	0.051757
28	1	0	-4.913597	-0.276223	-1.349325
29	1	0	-3.083662	-0.342052	1.065519
30	1	0	-0.312090	-1.878505	1.072843
31	1	0	-1.245173	-2.587179	-1.095099
32	1	0	3.977559	0.344990	-2.413324
33	1	0	6.314826	0.216033	-1.688950
34	1	0	5.073781	-1.631676	1.971126
35	1	0	2.703441	-1.503096	1.222100
36	1	0	-4.000162	-3.402519	2.322650
37	8	0	-3.797750	-3.277910	1.392324
38	1	0	-2.880238	-2.969065	1.350357
39	1	0	-1.067161	4.207824	0.557047
40	8	0	-1.353764	4.273260	-0.384294
41	1	0	-1.170780	5.156605	-0.710249
42	8	0	-0.769847	3.349155	2.030703
43	1	0	0.068842	2.855273	1.984448
44	1	0	-1.480984	2.697910	1.969309
45	1	0	1.184072	1.898781	0.322626
46	8	0	1.570556	2.042026	1.204285

47	1	0	2.134885	1.281839	1.374229
48	8	0	-7.888712	-0.878429	-0.938186
49	1	0	-7.110126	-1.148266	-0.425599
50	1	0	-8.460797	-0.437078	-0.307350
51	8	0	7.085643	-0.840429	0.694562
52	6	0	8.163265	-0.366664	-0.086111
53	1	0	8.073544	0.707212	-0.268542
54	1	0	9.062866	-0.563393	0.490072
55	1	0	8.219422	-0.895976	-1.040668

Structure 55·5H_2O (M06-2X/def2-TZVP, DMSO)

Energy (Hartrees): = -1433.249021
No imaginary frequencies

Standard orientation:

Center Number	Atomic Number	Atomic Type	Coordinates (Angstroms) X	Y	Z
1	6	0	4.156637	-0.533886	-1.493657
2	6	0	3.141398	-0.612334	-0.547566
3	6	0	3.488427	-0.754636	0.802247
4	6	0	4.809126	-0.814451	1.181954
5	6	0	5.824600	-0.735452	0.218186
6	6	0	5.494879	-0.595313	-1.127708
7	6	0	1.748262	-0.530845	-0.991892
8	7	0	0.761868	-0.591003	-0.196935
9	6	0	-0.572961	-0.456865	-0.743301
10	6	0	-1.122332	0.911610	-0.351661
11	6	0	-2.616948	1.032111	-0.613499
12	6	0	-3.327413	-0.147629	0.039123
13	8	0	-2.792542	-1.376091	-0.441244
14	6	0	-1.443545	-1.579365	-0.172991
15	8	0	-0.356393	1.869051	-1.063651
16	8	0	-1.266531	-1.694421	1.225279
17	6	0	-4.810381	-0.166617	-0.251652
18	8	0	-5.472099	-1.243662	0.393424
19	8	0	-3.128515	2.209845	-0.008351
20	1	0	1.599245	-0.405457	-2.069874
21	1	0	-0.588985	-0.537366	-1.836285
22	1	0	-0.788291	2.742834	-1.050592
23	1	0	-0.976827	1.039695	0.730543
24	1	0	-2.906182	2.982156	-0.549741
25	1	0	-2.810990	1.029951	-1.693113
26	1	0	-4.904438	-1.609390	1.098735
27	1	0	-5.239636	0.784473	0.070484
28	1	0	-4.965586	-0.267883	-1.330187
29	1	0	-3.175771	-0.073718	1.123198
30	1	0	-0.361146	-1.412885	1.430017
31	1	0	-1.187742	-2.525961	-0.654478
32	1	0	3.898784	-0.421758	-2.540943
33	1	0	6.259687	-0.531249	-1.888209
34	1	0	5.087652	-0.924897	2.222409
35	1	0	2.707890	-0.825388	1.551035
36	1	0	-3.852567	-2.058624	3.257176
37	8	0	-3.799329	-2.362631	2.343950
38	1	0	-2.884313	-2.197920	2.059137
39	1	0	-1.321071	4.473510	0.081780
40	8	0	-1.599133	4.379845	-0.851830
41	1	0	-1.415782	5.204137	-1.315824
42	8	0	-1.029042	3.685133	1.702778
43	1	0	-0.192236	3.184001	1.670316
44	1	0	-1.732882	3.027846	1.607001
45	1	0	1.018298	2.102609	0.228924
46	8	0	1.364143	2.277091	1.122722
47	1	0	1.562047	1.399419	1.473802
48	8	0	-6.243732	-3.002750	-1.640898
49	1	0	-5.942865	-2.451141	-0.896035
50	1	0	-7.118939	-2.667955	-1.858005
51	8	0	7.085545	-0.804529	0.684972
52	6	0	8.147687	-0.729817	-0.254443
53	1	0	8.131112	0.221671	-0.790830
54	1	0	9.066525	-0.803935	0.321862
55	1	0	8.098360	-1.553879	-0.969811

Structure 55·5H_2O (M06-2X/def2-TZVP, H_2O)

Energy (Hartrees): = -1433.266174
No imaginary frequencies

Standard orientation:

Center Number	Atomic Number	Atomic Type	Coordinates (Angstroms) X	Y	Z
1	6	0	4.101499	-0.400462	-1.397809
2	6	0	3.107399	-0.620760	-0.450740
3	6	0	3.486506	-0.912753	0.865597
4	6	0	4.816968	-0.983390	1.210588
5	6	0	5.806631	-0.761046	0.245307
6	6	0	5.448408	-0.468177	-1.066651
7	6	0	1.708630	-0.537177	-0.877096
8	7	0	0.716351	-0.746181	-0.111308
9	6	0	-0.604245	-0.562466	-0.680499
10	6	0	-1.112305	0.840706	-0.353615
11	6	0	-2.579921	0.990753	-0.732611
12	6	0	-3.375680	-0.109833	-0.048487
13	8	0	-2.874397	-1.380240	-0.455745
14	6	0	-1.540710	-1.628851	-0.110852
15	8	0	-0.285689	1.755108	-1.053245
16	8	0	-1.444024	-1.706605	1.295862
17	6	0	-4.846828	-0.086532	-0.395619
18	8	0	-5.548058	-1.155237	0.228401
19	8	0	-3.090139	2.237032	-0.281539
20	1	0	1.559759	-0.286171	-1.932539
21	1	0	-0.600915	-0.672432	-1.770764
22	1	0	-0.634311	2.666353	-0.968574
23	1	0	-1.029993	1.008302	0.728904
24	1	0	-2.756501	2.947217	-0.850904
25	1	0	-2.691782	0.902028	-1.819088
26	1	0	-5.085502	-1.415549	1.050764
27	1	0	-5.268282	0.873025	-0.090789
28	1	0	-4.972669	-0.196446	-1.475891
29	1	0	-3.264038	0.003700	1.036458
30	1	0	-0.561024	-1.400909	1.554089
31	1	0	-1.307634	-2.600950	-0.549123
32	1	0	3.819097	-0.171629	-2.419147
33	1	0	6.195867	-0.296257	-1.827105
34	1	0	5.120901	-1.210179	2.224590
35	1	0	2.728328	-1.084463	1.618526
36	1	0	-4.191594	-1.579180	3.214633
37	8	0	-4.088094	-2.115123	2.419472
38	1	0	-3.163413	-1.987709	2.146539
39	1	0	-1.224232	4.452115	0.055295
40	8	0	-1.275539	4.371166	-0.914138
41	1	0	-0.709204	5.058893	-1.282331
42	8	0	-1.363533	3.576809	1.772506
43	1	0	-0.496241	3.144630	1.656818
44	1	0	-1.999345	2.995521	1.326025
45	1	0	0.977654	2.176310	0.350551
46	8	0	1.238055	2.485456	1.236153
47	1	0	1.832421	3.228660	1.079952
48	8	0	-5.363266	-3.255026	-1.601627
49	1	0	-5.428643	-2.548345	-0.930408
50	1	0	-6.235237	-3.290866	-2.008741
51	8	0	7.088507	-0.851844	0.678547
52	6	0	8.122367	-0.653932	-0.277601
53	1	0	8.069880	0.349747	-0.703566
54	1	0	9.057945	-0.772412	0.262244
55	1	0	8.061333	-1.398204	-1.073908

Structure 55.5H_2O* (M06-2X, Gas Phase)

Energy (Hartrees): = - 1433.0335323
No imaginary frequencies

Standard orientation:

Center Number	Atomic Number	Atomic Type	Coordinates (Angstroms) X	Y	Z
1	6	0	4.193480	-0.029597	-1.469493
2	6	0	3.176972	-0.535437	-0.666574
3	6	0	3.517616	-1.131142	0.557873
4	6	0	4.832672	-1.209231	0.957467
5	6	0	5.850021	-0.692970	0.140278
6	6	0	5.527402	-0.100933	-1.080154
7	6	0	1.780183	-0.419236	-1.102636
8	7	0	0.804633	-0.792700	-0.381535
9	6	0	-0.541699	-0.548496	-0.868501
10	6	0	-1.029154	0.780491	-0.287917
11	6	0	-2.532327	0.974364	-0.465734
12	6	0	-3.269522	-0.265289	0.029853

13	8	0	-2.777707	-1.430457	-0.632927
14	6	0	-1.435807	-1.706482	-0.414228
15	8	0	-0.253701	1.786104	-0.919271
16	8	0	-1.235330	-1.962563	0.962007
17	6	0	-4.756275	-0.217774	-0.246301
18	8	0	-5.461201	-1.212949	0.469342
19	8	0	-2.989358	2.057168	0.326324
20	1	0	1.617074	0.039119	-2.085610
21	1	0	-0.587702	-0.475589	-1.962626
22	1	0	-0.665791	2.662667	-0.808874
23	1	0	-0.829696	0.774820	0.792290
24	1	0	-2.769531	2.894979	-0.106629
25	1	0	-2.758096	1.136225	-1.528925
26	1	0	-4.882456	-1.981448	0.616829
27	1	0	-5.138125	0.755088	0.068395
28	1	0	-4.918179	-0.329660	-1.325515
29	1	0	-3.106281	-0.358046	1.110442
30	1	0	-0.291791	-1.836944	1.131164
31	1	0	-1.219083	-2.604985	-0.998474
32	1	0	3.943412	0.434945	-2.417639
33	1	0	6.292981	0.304103	-1.726883
34	1	0	5.118367	-1.667908	1.895685
35	1	0	2.729686	-1.539532	1.179827
36	1	0	-3.955999	-2.784245	2.577988
37	8	0	-3.771486	-3.073150	1.681569
38	1	0	-2.844344	-2.843604	1.526799
39	1	0	-1.147310	4.138812	0.603273
40	8	0	-1.439479	4.209356	-0.336029
41	1	0	-1.298188	5.104976	-0.645761
42	8	0	-0.861985	3.252577	2.037015
43	1	0	-0.032720	2.743408	2.009828
44	1	0	-1.581362	2.612875	1.967236
45	1	0	1.141997	1.893728	0.355864
46	8	0	1.461351	1.935984	1.271566
47	1	0	1.926902	1.107279	1.411340
48	8	0	-7.500108	-1.002890	-1.449828
49	1	0	-6.933732	-1.340267	-0.741206
50	1	0	-7.900384	-0.226160	-1.054637
51	8	0	7.103289	-0.819177	0.622422
52	6	0	8.169372	-0.305654	-0.153513
53	1	0	8.064274	0.773508	-0.300829
54	1	0	9.075082	-0.508686	0.412151
55	1	0	8.226671	-0.806285	-1.124794

Structure 55.5H_2O* (M06-2X, DMSO)

Energy (Hartrees): = - 1433.076106
No imaginary frequencies

Standard orientation:

Center Number	Atomic Number	Atomic Type	Coordinates (Angstroms) X	Y	Z
1	6	0	4.148046	-0.529886	-1.497182
2	6	0	3.138818	-0.595486	-0.539360
3	6	0	3.497801	-0.694076	0.814479
4	6	0	4.824450	-0.722778	1.186149
5	6	0	5.834345	-0.656132	0.211645
6	6	0	5.492001	-0.560918	-1.138369
7	6	0	1.738740	-0.544623	-0.974088
8	7	0	0.760951	-0.582771	-0.162410
9	6	0	-0.581849	-0.483098	-0.707316
10	6	0	-1.147584	0.889105	-0.345973
11	6	0	-2.648374	0.980559	-0.598910
12	6	0	-3.338191	-0.200586	0.074915
13	8	0	-2.783644	-1.435666	-0.374619
14	6	0	-1.430302	-1.601440	-0.094134
15	8	0	-0.394645	1.837348	-1.086065
16	8	0	-1.260052	-1.644790	1.310764
17	6	0	-4.823323	-0.255292	-0.215388
18	8	0	-5.447719	-1.372450	0.399291
19	8	0	-3.183158	2.151626	0.005047
20	1	0	1.577859	-0.460901	-2.055334
21	1	0	-0.604173	-0.592783	-1.798570
22	1	0	-0.849223	2.698977	-1.100537
23	1	0	-0.997705	1.043867	0.731407
24	1	0	-2.957313	2.924045	-0.535214
25	1	0	-2.849763	0.966824	-1.678082
26	1	0	-4.904538	-1.669709	1.152776
27	1	0	-5.276936	0.679415	0.125756
28	1	0	-4.975979	-0.346927	-1.295649
29	1	0	-3.185169	-0.103120	1.156553
30	1	0	-0.349234	-1.374844	1.499435
31	1	0	-1.152550	-2.563171	-0.532459
32	1	0	3.881553	-0.452977	-2.546535

33	1	0	6.250768	-0.508317	-1.907411
34	1	0	5.115530	-0.799918	2.227080
35	1	0	2.723798	-0.760249	1.571469
36	1	0	-3.813367	-1.751708	3.288749
37	8	0	-3.788079	-2.241190	2.459799
38	1	0	-2.877730	-2.141756	2.137824
39	1	0	-1.407218	4.380543	0.037123
40	8	0	-1.702478	4.267182	-0.889055
41	1	0	-1.566852	5.092623	-1.364752
42	8	0	-1.108731	3.605188	1.630120
43	1	0	-0.275550	3.099572	1.629991
44	1	0	-1.812551	2.947662	1.549779
45	1	0	0.983807	2.101767	0.202470
46	8	0	1.283577	2.204701	1.121146
47	1	0	1.409104	1.292579	1.409286
48	8	0	-5.896124	-2.810938	-1.944367
49	1	0	-5.643607	-2.486479	-1.065103
50	1	0	-6.789544	-2.471004	-2.046655
51	8	0	7.098588	-0.692467	0.674501
52	6	0	8.155857	-0.644308	-0.276695
53	1	0	8.131078	0.289142	-0.845736
54	1	0	9.077632	-0.692424	0.298983
55	1	0	8.108314	-1.495925	-0.960901

Structure 55.5H_2O* (M06-2X, H_2O)

Energy (Hartrees): = - 1433.0959027
No imaginary frequencies

Standard orientation:

Center Number	Atomic Number	Atomic Type	Coordinates (Angstroms) X	Y	Z
1	6	0	4.085684	-0.641360	-1.432023
2	6	0	3.080584	-0.603555	-0.467806
3	6	0	3.444339	-0.560387	0.886874
4	6	0	4.773589	-0.575249	1.253932
5	6	0	5.775001	-0.632434	0.273557
6	6	0	5.431101	-0.662828	-1.077592
7	6	0	1.679432	-0.599332	-0.904430
8	7	0	0.697798	-0.776359	-0.113759
9	6	0	-0.632691	-0.630372	-0.678059
10	6	0	-1.124639	0.790987	-0.392182
11	6	0	-2.607328	0.947804	-0.718004
12	6	0	-3.385233	-0.143985	0.003230
13	8	0	-2.898006	-1.430161	-0.381460
14	6	0	-1.559005	-1.670353	-0.045009
15	8	0	-0.303454	1.661185	-1.157240
16	8	0	-1.441107	-1.675426	1.363442
17	6	0	-4.867447	-0.129341	-0.308558
18	8	0	-5.544010	-1.204654	0.335682
19	8	0	-3.107026	2.188617	-0.234769
20	1	0	1.521743	-0.436201	-1.976741
21	1	0	-0.646463	-0.783521	-1.764035
22	1	0	-0.655715	2.573398	-1.124248
23	1	0	-1.000495	0.997033	0.679788
24	1	0	-2.748606	2.911879	-0.772971
25	1	0	-2.759801	0.863102	-1.801145
26	1	0	-5.082384	-1.411136	1.172011
27	1	0	-5.285691	0.831511	0.000226
28	1	0	-5.016366	-0.252024	-1.385178
29	1	0	-3.245276	-0.006064	1.081656
30	1	0	-0.546674	-1.378694	1.585674
31	1	0	-1.337189	-2.663611	-0.440328
32	1	0	3.813886	-0.661045	-2.482441
33	1	0	6.188120	-0.701437	-1.848904
34	1	0	5.071581	-0.538390	2.295023
35	1	0	2.674667	-0.501632	1.646458
36	1	0	-4.059323	-1.176044	3.194860
37	8	0	-4.041348	-1.917780	2.579661
38	1	0	-3.132970	-1.915512	2.235922
39	1	0	-1.210423	4.311731	-0.081338
40	8	0	-1.376208	4.194061	-1.035640
41	1	0	-0.979333	4.943348	-1.490887
42	8	0	-1.200645	3.575880	1.623372
43	1	0	-0.365550	3.080746	1.528823
44	1	0	-1.888650	2.964405	1.323159
45	1	0	0.984954	2.053532	0.197570
46	8	0	1.260301	2.276310	1.103251
47	1	0	1.912436	2.977579	0.998439
48	8	0	-5.167277	-3.030792	-1.746095
49	1	0	-5.263542	-2.519567	-0.924239
50	1	0	-5.996972	-2.869134	-2.206068
51	8	0	7.050940	-0.648734	0.735630
52	6	0	8.101787	-0.717042	-0.224216

53	1	0	8.087181	0.155653	-0.881563
54	1	0	9.025825	-0.726680	0.348869
55	1	0	8.028108	-1.632076	-0.817036

Structure 55.6H_2O (B3LYP, Gas Phase)

Energy (Hartrees): = - 1395.0320644
No imaginary frequencies

Standard orientation:

Center Number	Atomic Number	Atomic Type	Coordinates (Angstroms) X	Y	Z
1	6	0	4.307724	-0.452979	-1.697591
2	6	0	3.369781	-0.484005	-0.657116
3	6	0	3.844346	-0.544296	0.670995
4	6	0	5.201106	-0.566163	0.933359
5	6	0	6.131626	-0.530157	-0.123021
6	6	0	5.678527	-0.473660	-1.447292
7	6	0	1.945645	-0.453617	-0.988942
8	7	0	1.004631	-0.428065	-0.121883
9	6	0	-0.373394	-0.410001	-0.584792
10	6	0	-1.104565	0.852619	-0.105147
11	6	0	-2.572558	0.847554	-0.559723
12	6	0	-3.226398	-0.452876	-0.073609
13	8	0	-2.480762	-1.573897	-0.573817
14	6	0	-1.153867	-1.662133	-0.101274
15	8	0	-0.380355	1.991584	-0.581195
16	8	0	-1.145419	-1.877462	1.287938
17	6	0	-4.665534	-0.650956	-0.522703
18	8	0	-5.339257	-1.636641	0.248842
19	8	0	-3.263284	1.956194	0.018801
20	1	0	1.711722	-0.449965	-2.063740
21	1	0	-0.421565	-0.431067	-1.684592
22	1	0	-0.910545	2.782289	-0.357204
23	1	0	-1.112132	0.857502	0.993519
24	1	0	-3.353593	2.674449	-0.646314
25	1	0	-2.615067	0.898818	-1.656175
26	1	0	-4.698581	-2.319865	0.551440
27	1	0	-5.203975	0.292893	-0.391258
28	1	0	-4.678711	-0.909191	-1.591174
29	1	0	-3.207775	-0.454592	1.024669
30	1	0	-0.616679	-1.227732	1.808694
31	1	0	-0.746229	-2.551032	-0.596156
32	1	0	3.960079	-0.409685	-2.726746
33	1	0	6.374485	-0.447318	-2.276555
34	1	0	5.578093	-0.617045	1.949232
35	1	0	3.138606	-0.579730	1.494302
36	1	0	-3.935279	-3.048925	2.572049
37	8	0	-3.623611	-3.286906	1.689956
38	1	0	-2.731559	-2.894174	1.627174
39	1	0	-2.494612	4.597594	-0.634345
40	8	0	-3.065277	4.305415	-1.370343
41	1	0	-3.838625	4.882727	-1.342467
42	8	0	-1.662336	4.029525	0.996098
43	1	0	-0.878387	3.823664	1.540602
44	1	0	-2.280332	3.286169	1.128052
45	1	0	0.630404	2.392993	0.940347
46	8	0	0.667523	2.681309	1.879947
47	1	0	1.546318	3.059884	2.009705
48	1	0	1.018083	-0.266247	1.694391
49	8	0	0.630985	-0.204313	2.599425
50	1	0	0.518758	0.754495	2.716447
51	8	0	-7.077362	-2.320853	-1.864219
52	1	0	-6.529981	-2.276526	-1.056637
53	1	0	-7.860270	-1.810373	-1.624812
54	8	0	7.436853	-0.555473	0.247459
55	6	0	8.434298	-0.534586	-0.766716
56	1	0	8.378953	0.382230	-1.366111
57	1	0	9.391017	-0.566529	-0.244811
58	1	0	8.352934	-1.405566	-1.428056

Structure 55.6H_2O (B3LYP, DMSO)

Energy (Hartrees): = - 1509.6843979
No imaginary frequencies

Standard orientation:

Center Number	Atomic Number	Atomic Type	Coordinates (Angstroms) X	Y	Z
1	6	0	4.275279	-0.607321	-1.660576

2	6	0	3.341351	-0.508407	-0.618042
3	6	0	3.823963	-0.431586	0.706182
4	6	0	5.182102	-0.449927	0.965155
5	6	0	6.108151	-0.547990	-0.093279
6	6	0	5.646567	-0.629188	-1.414929
7	6	0	1.918832	-0.487520	-0.953955
8	7	0	0.973058	-0.404467	-0.091627
9	6	0	-0.400471	-0.393842	-0.577477
10	6	0	-1.130104	0.890475	-0.152836
11	6	0	-2.594059	0.869973	-0.621364
12	6	0	-3.253973	-0.399798	-0.069132
13	8	0	-2.523628	-1.555803	-0.512448
14	6	0	-1.183473	-1.628163	-0.062413
15	8	0	-0.401401	2.007145	-0.670409
16	8	0	-1.143251	-1.826037	1.335063
17	6	0	-4.693260	-0.601734	-0.509001
18	8	0	-5.368869	-1.602006	0.255249
19	8	0	-3.296094	2.005037	-0.110174
20	1	0	1.690921	-0.547244	-2.026341
21	1	0	-0.432052	-0.454812	-1.674951
22	1	0	-0.900830	2.809727	-0.420766
23	1	0	-1.149376	0.944454	0.944341
24	1	0	-3.310627	2.712750	-0.794407
25	1	0	-2.629459	0.859647	-1.718793
26	1	0	-4.712332	-2.147791	0.749167
27	1	0	-5.232364	0.343502	-0.387838
28	1	0	-4.709585	-0.869335	-1.574881
29	1	0	-3.232464	-0.344187	1.028464
30	1	0	-0.689451	-1.103811	1.831506
31	1	0	-0.784365	-2.528438	-0.540834
32	1	0	3.921876	-0.667945	-2.686649
33	1	0	6.338274	-0.706827	-2.244669
34	1	0	5.558032	-0.390833	1.981618
35	1	0	3.126576	-0.357251	1.533817
36	1	0	-3.908178	-2.607846	2.820140
37	8	0	-3.666212	-2.980571	1.959846
38	1	0	-2.766408	-2.632200	1.787891
39	1	0	-2.423309	4.600968	-0.831790
40	8	0	-2.962605	4.290641	-1.583429
41	1	0	-3.748278	4.857878	-1.577649
42	8	0	-1.662608	4.087957	0.883968
43	1	0	-0.883174	3.855246	1.428025
44	1	0	-2.299998	3.363194	1.022276
45	1	0	0.661363	2.446394	0.913718
46	8	0	0.622767	2.748485	1.845204
47	1	0	1.472619	3.182584	2.016685
48	1	0	0.962499	-0.199591	1.719249
49	8	0	0.563533	-0.080784	2.616994
50	1	0	0.468393	0.887761	2.666945
51	8	0	-6.610567	-2.988059	-1.835962
52	1	0	-6.210776	-2.597243	-1.031286
53	1	0	-7.381800	-2.426214	-1.990077
54	8	0	7.412720	-0.555477	0.267724
55	6	0	8.403060	-0.689379	-0.756460
56	1	0	8.363248	0.145315	-1.464982
57	1	0	9.364822	-0.679612	-0.242134
58	1	0	8.290730	-1.634756	-1.298649

Structure 55.6H$_2$O (B3LYP, H$_2$O)

Energy (Hartrees): = - 1509.7110188
No imaginary frequencies

Standard orientation:

Center Number	Atomic Number	Atomic Type	Coordinates (Angstroms) X	Y	Z
1	6	0	4.224695	-0.828696	-1.471564
2	6	0	3.257605	-0.587861	-0.484009
3	6	0	3.695213	-0.308349	0.828353
4	6	0	5.045061	-0.277615	1.129584
5	6	0	6.001824	-0.526991	0.127057
6	6	0	5.587531	-0.802819	-1.182388
7	6	0	1.847052	-0.636914	-0.865294
8	7	0	0.869701	-0.481170	-0.048813
9	6	0	-0.481508	-0.537074	-0.592859
10	6	0	-1.191784	0.825364	-0.461355
11	6	0	-2.634428	0.734031	-0.985073
12	6	0	-3.340359	-0.400072	-0.234823
13	8	0	-2.628381	-1.632209	-0.431630
14	6	0	-1.312815	-1.642046	0.096877
15	8	0	-0.388861	1.791674	-1.148437
16	8	0	-1.337407	-1.579835	1.511104
17	6	0	-4.777420	-0.645688	-0.667011
18	8	0	-5.390832	-1.702377	0.079953

19	8	0	-3.366363	1.932677	-0.720289
20	1	0	1.658272	-0.828113	-1.929452
21	1	0	-0.465977	-0.799232	-1.660295
22	1	0	-0.935897	2.563784	-1.408368
23	1	0	-1.261075	1.099811	0.597865
24	1	0	-2.961813	2.661068	-1.233258
25	1	0	-2.625321	0.520777	-2.063297
26	1	0	-4.942681	-1.784537	0.956848
27	1	0	-5.343381	0.285167	-0.548262
28	1	0	-4.806463	-0.933068	-1.723911
29	1	0	-3.340045	-0.146628	0.833262
30	1	0	-0.946859	-0.749282	1.863328
31	1	0	-0.917565	-2.622884	-0.181624
32	1	0	3.904207	-1.042292	-2.487595
33	1	0	6.307086	-0.993566	-1.968655
34	1	0	5.388980	-0.062049	2.135974
35	1	0	2.969851	-0.109624	1.609927
36	1	0	-4.200918	-1.327192	3.006108
37	8	0	-4.017588	-2.108650	2.464394
38	1	0	-3.089647	-1.978068	2.176960
39	1	0	-2.013811	4.406127	-0.729137
40	8	0	-1.810415	4.126240	-1.656053
41	1	0	-2.475932	4.543915	-2.220855
42	8	0	-2.312804	4.262027	1.017549
43	1	0	-1.414626	3.923394	1.236193
44	1	0	-2.820501	3.446576	0.867760
45	1	0	0.213985	2.708970	0.444348
46	8	0	0.183703	3.060575	1.359724
47	1	0	0.958430	3.636833	1.434568
48	1	0	0.769034	0.083412	1.697558
49	8	0	0.376556	0.431577	2.535808
50	1	0	0.261534	1.378370	2.316022
51	8	0	-4.705769	-3.976781	-1.371572
52	1	0	-4.946669	-3.216511	-0.798027
53	1	0	-5.514312	-4.134344	-1.877725
54	8	0	7.303896	-0.474157	0.528567
55	6	0	8.323648	-0.693657	-0.453998
56	1	0	8.271378	0.054101	-1.252015
57	1	0	9.271107	-0.594116	0.076296
58	1	0	8.249945	-1.698381	-0.882775

Structure 55.6H_2O (M06-2X, Gas Phase)

Energy (Hartrees): = - 1509.4725227
No imaginary frequencies

Standard orientation:

Center Number	Atomic Number	Atomic Type	Coordinates (Angstroms) X	Y	Z
1	6	0	4.148527	-0.421236	-1.708018
2	6	0	3.194807	-0.595909	-0.709613
3	6	0	3.628443	-0.735734	0.619258
4	6	0	4.971563	-0.704665	0.922154
5	6	0	5.924031	-0.536129	-0.094392
6	6	0	5.508964	-0.393212	-1.417583
7	6	0	1.772944	-0.596567	-1.072086
8	7	0	0.840614	-0.813813	-0.238835
9	6	0	-0.526820	-0.638300	-0.688110
10	6	0	-1.027755	0.724961	-0.199448
11	6	0	-2.498670	0.941063	-0.542140
12	6	0	-3.312392	-0.271126	-0.094666
13	8	0	-2.760855	-1.462342	-0.644375
14	6	0	-1.459196	-1.759853	-0.206025
15	8	0	-0.166537	1.698786	-0.774847
16	8	0	-1.455424	-1.990516	1.172858
17	6	0	-4.752940	-0.206044	-0.555865
18	8	0	-5.573777	-1.138208	0.116540
19	8	0	-3.003843	2.069788	0.152990
20	1	0	1.547360	-0.368487	-2.121482
21	1	0	-0.591766	-0.633132	-1.784901
22	1	0	-0.625933	2.551735	-0.877743
23	1	0	-0.945578	0.763672	0.895989
24	1	0	-2.821261	2.867661	-0.363048
25	1	0	-2.603511	1.069193	-1.628656
26	1	0	-5.040744	-1.901573	0.405417
27	1	0	-5.141391	0.793493	-0.349337
28	1	0	-4.779439	-0.376187	-1.639504
29	1	0	-3.291002	-0.319432	1.001867
30	1	0	-0.882428	-1.396029	1.694134
31	1	0	-1.201781	-2.693047	-0.710926
32	1	0	3.826103	-0.306446	-2.737721
33	1	0	6.223324	-0.260721	-2.217989
34	1	0	5.327320	-0.815704	1.938663
35	1	0	2.905012	-0.875180	1.413480

36	1	0	-4.351973	-2.848540	2.462389
37	8	0	-4.084576	-2.986131	1.551507
38	1	0	-3.142930	-2.756612	1.519747
39	1	0	-1.241584	4.234131	0.196981
40	8	0	-1.472417	4.142111	-0.754826
41	1	0	-1.348053	4.983998	-1.195316
42	8	0	-1.044772	3.541656	1.770458
43	1	0	-0.212944	3.064570	1.916005
44	1	0	-1.740848	2.872599	1.728804
45	1	0	1.028849	1.849227	0.591353
46	8	0	1.274173	1.949390	1.531988
47	1	0	2.219435	2.114911	1.559390
48	1	0	0.882836	-0.809074	1.626746
49	8	0	0.512947	-0.625842	2.511604
50	1	0	0.659615	0.325336	2.588866
51	8	0	-7.429922	-0.866358	-1.958594
52	1	0	-6.929479	-1.234313	-1.216008
53	1	0	-7.840976	-0.089776	-1.574324
54	8	0	7.211631	-0.523955	0.308283
55	6	0	8.214991	-0.390962	-0.680509
56	1	0	8.125455	0.564403	-1.206534
57	1	0	9.163291	-0.425240	-0.150397
58	1	0	8.168720	-1.213495	-1.400529

Structure 55.6H$_2$O (M06-2X, DMSO)

Energy (Hartrees): = - 1509.5173896
No imaginary frequencies

Standard orientation:

Center Number	Atomic Number	Atomic Type	Coordinates (Angstroms) X	Y	Z
1	6	0	4.212574	-0.705802	-1.641888
2	6	0	3.265000	-0.585067	-0.626958
3	6	0	3.710990	-0.445574	0.697285
4	6	0	5.058649	-0.433841	0.984790
5	6	0	6.004172	-0.562992	-0.045742
6	6	0	5.576498	-0.700828	-1.367348
7	6	0	1.842635	-0.601390	-0.983536
8	7	0	0.904511	-0.596320	-0.124845
9	6	0	-0.460790	-0.552359	-0.618116
10	6	0	-1.084252	0.805959	-0.286446
11	6	0	-2.548114	0.845528	-0.717342
12	6	0	-3.273484	-0.353260	-0.111783
13	8	0	-2.631344	-1.567712	-0.489617
14	6	0	-1.304036	-1.696043	-0.036458
15	8	0	-0.280191	1.789089	-0.921311
16	8	0	-1.281915	-1.800721	1.362401
17	6	0	-4.708142	-0.464729	-0.577408
18	8	0	-5.458671	-1.378536	0.208160
19	8	0	-3.191052	2.009982	-0.217951
20	1	0	1.623881	-0.616221	-2.057497
21	1	0	-0.494845	-0.670495	-1.708981
22	1	0	-0.792263	2.603849	-1.079977
23	1	0	-1.067629	0.962176	0.802385
24	1	0	-2.989976	2.756213	-0.803068
25	1	0	-2.613427	0.803999	-1.812532
26	1	0	-4.854618	-1.980460	0.683223
27	1	0	-5.184025	0.515328	-0.506701
28	1	0	-4.705390	-0.779649	-1.628129
29	1	0	-3.258204	-0.245806	0.981674
30	1	0	-0.813360	-1.070626	1.811936
31	1	0	-0.957647	-2.644652	-0.449785
32	1	0	3.879715	-0.808520	-2.669781
33	1	0	6.285664	-0.801966	-2.177753
34	1	0	5.415613	-0.324190	2.001880
35	1	0	2.996515	-0.340292	1.504988
36	1	0	-4.084140	-2.477580	2.827571
37	8	0	-3.857340	-2.811425	1.953509
38	1	0	-2.940420	-2.525003	1.805842
39	1	0	-1.641146	4.352354	-0.216370
40	8	0	-1.753319	4.117576	-1.160128
41	1	0	-1.613287	4.903050	-1.698140
42	8	0	-1.593760	3.754621	1.479735
43	1	0	-0.720904	3.361835	1.648480
44	1	0	-2.187732	3.006111	1.328420
45	1	0	0.799317	2.223201	0.529919
46	8	0	0.902498	2.463833	1.470071
47	1	0	1.748831	2.917645	1.550909
48	1	0	0.906779	-0.349277	1.718744
49	8	0	0.505359	-0.109993	2.579371
50	1	0	0.545554	0.856511	2.540839
51	8	0	-7.224877	-1.882052	-1.882793
52	1	0	-6.699635	-1.882619	-1.067066

53	1	0	-7.500385	-0.964333	-1.962054
54	8	0	7.295242	-0.538638	0.336315
55	6	0	8.287604	-0.685022	-0.672862
56	1	0	8.226979	0.123993	-1.406028
57	1	0	9.245088	-0.636321	-0.159121
58	1	0	8.192659	-1.649669	-1.179139

Structure 55.6H_2O (M06-2X, H_2O)

Energy (Hartrees): = - 1509.540473
No imaginary frequencies

Standard orientation:

Center Number	Atomic Number	Atomic Type	Coordinates (Angstroms) X	Y	Z
1	6	0	4.138387	-0.634553	-1.651892
2	6	0	3.162432	-0.659803	-0.657036
3	6	0	3.568251	-0.651608	0.687227
4	6	0	4.908070	-0.634853	1.012106
5	6	0	5.879538	-0.623545	0.000657
6	6	0	5.494404	-0.622681	-1.339710
7	6	0	1.749930	-0.660540	-1.052555
8	7	0	0.791420	-0.847988	-0.235245
9	6	0	-0.556295	-0.658223	-0.746847
10	6	0	-1.020418	0.758098	-0.383618
11	6	0	-2.496144	0.974004	-0.710867
12	6	0	-3.315502	-0.161605	-0.109420
13	8	0	-2.833517	-1.416297	-0.591114
14	6	0	-1.512443	-1.724648	-0.201111
15	8	0	-0.170562	1.651923	-1.087360
16	8	0	-1.432205	-1.932427	1.188662
17	6	0	-4.778437	-0.093180	-0.486429
18	8	0	-5.582522	-0.938687	0.329302
19	8	0	-2.969303	2.177070	-0.119439
20	1	0	1.558459	-0.479232	-2.116024
21	1	0	-0.590061	-0.741147	-1.840474
22	1	0	-0.509991	2.566545	-1.008167
23	1	0	-0.906735	0.908602	0.698428
24	1	0	-2.604439	2.935703	-0.601699
25	1	0	-2.635680	0.995721	-1.798905
26	1	0	-5.039465	-1.674656	0.673949
27	1	0	-5.140534	0.927739	-0.363634
28	1	0	-4.875894	-0.377456	-1.540870
29	1	0	-3.223244	-0.123085	0.984822
30	1	0	-1.135274	-1.140978	1.666724
31	1	0	-1.298940	-2.685099	-0.668576
32	1	0	3.834488	-0.627298	-2.693535
33	1	0	6.227545	-0.608323	-2.134715
34	1	0	5.237029	-0.624241	2.044321
35	1	0	2.829570	-0.650578	1.479392
36	1	0	-4.312619	-2.456682	2.651390
37	8	0	-4.103370	-2.789783	1.771956
38	1	0	-3.178902	-2.526796	1.627791
39	1	0	-1.052905	4.276443	0.151917
40	8	0	-1.209268	4.187036	-0.805632
41	1	0	-0.827565	4.960309	-1.232826
42	8	0	-1.082731	3.413588	1.835170
43	1	0	-0.241362	2.941504	1.700498
44	1	0	-1.758443	2.828713	1.462437
45	1	0	1.107234	1.914717	0.310471
46	8	0	1.335625	2.086674	1.240883
47	1	0	2.181726	2.546334	1.228356
48	1	0	0.735062	-0.648490	1.674413
49	8	0	0.446347	-0.267460	2.527200
50	1	0	0.670059	0.666238	2.388433
51	8	0	-7.327664	-1.524267	-1.776116
52	1	0	-6.817961	-1.439228	-0.953177
53	1	0	-7.549360	-0.612774	-1.991405
54	8	0	7.168054	-0.606552	0.423319
55	6	0	8.191242	-0.568282	-0.567746
56	1	0	8.103463	0.331003	-1.182189
57	1	0	9.130866	-0.547339	-0.021068
58	1	0	8.154759	-1.458296	-1.200548

Structure 62 (B3LYP, Gas Phase)

Energy (Hartrees): = - 1140.9752323
No imaginary frequencies

Standard orientation:

Center	Atomic	Atomic	Coordinates (Angstroms)

Number	Number	Type	X	Y	Z
1	6	0	2.207303	-1.407457	0.746959
2	6	0	1.502000	-0.026790	0.630045
3	6	0	2.232645	0.828458	-0.422771
4	6	0	3.722961	0.882927	-0.103859
5	6	0	4.301243	-0.533879	-0.018435
6	1	0	2.106791	0.366711	-1.410006
7	1	0	3.850855	1.369533	0.877122
8	1	0	4.200221	-1.030946	-0.989949
9	1	0	1.574370	0.487864	1.600338
10	8	0	3.576823	-1.255962	0.989666
11	6	0	5.776017	-0.550805	0.393085
12	1	0	5.894482	0.021607	1.328898
13	1	0	6.062413	-1.585311	0.604249
14	8	0	6.621343	-0.078544	-0.636558
15	1	0	6.256095	0.769616	-0.933145
16	8	0	4.419451	1.609911	-1.110886
17	1	0	3.954539	2.454316	-1.205982
18	8	0	1.764723	2.177006	-0.435893
19	1	0	0.939341	2.204914	-0.936428
20	7	0	0.127505	-0.236998	0.216577
21	6	0	-2.229565	0.212849	0.514240
22	6	0	-2.649753	-0.573080	-0.573491
23	6	0	-3.187990	0.879884	1.293335
24	6	0	-3.998833	-0.692052	-0.876195
25	1	0	-1.903264	-1.084068	-1.170649
26	6	0	-4.544041	0.769394	1.002111
27	1	0	-2.867936	1.487999	2.134666
28	6	0	-4.926613	-0.016993	-0.080963
29	1	0	-4.348144	-1.290892	-1.707695
30	1	0	-5.300579	1.273348	1.589748
31	6	0	-0.804594	0.353539	0.859585
32	1	0	-0.585356	1.004264	1.718693
33	1	0	1.824849	-1.941665	1.628205
34	8	0	2.004494	-2.160249	-0.426880
35	1	0	1.098511	-1.967331	-0.717252
36	7	0	-6.360617	-0.139940	-0.399005
37	8	0	-7.156570	0.463626	0.318923
38	8	0	-6.671525	-0.837956	-1.362832

Structure 62 (B3LYP, DMSO)

Energy (Hartrees): = - 1141.0031387
No imaginary frequencies

Standard orientation:

Center Number	Atomic Number	Atomic Type	Coordinates (Angstroms) X	Y	Z
1	6	0	2.207446	-1.406692	0.776498
2	6	0	1.507955	-0.027393	0.665197
3	6	0	2.220474	0.835745	-0.395779
4	6	0	3.716577	0.878512	-0.111017
5	6	0	4.290598	-0.540283	-0.040598
6	1	0	2.065402	0.388698	-1.385721
7	1	0	3.875106	1.368642	0.862171
8	1	0	4.170269	-1.031188	-1.014116
9	1	0	1.582845	0.482766	1.635684
10	8	0	3.592171	-1.274017	0.975994
11	6	0	5.771013	-0.558570	0.345708
12	1	0	5.901950	-0.010316	1.293078
13	1	0	6.076200	-1.595201	0.521831
14	8	0	6.600847	-0.040809	-0.684048
15	1	0	6.190710	0.795183	-0.962219
16	8	0	4.401143	1.596280	-1.140360
17	1	0	3.954420	2.454143	-1.216616
18	8	0	1.761638	2.186124	-0.383364
19	1	0	0.921314	2.219865	-0.863745
20	7	0	0.133025	-0.248356	0.254017
21	6	0	-2.221561	0.214192	0.520101
22	6	0	-2.634589	-0.578290	-0.565974
23	6	0	-3.183085	0.889243	1.288808
24	6	0	-3.981141	-0.697771	-0.877234
25	1	0	-1.891288	-1.095909	-1.161784
26	6	0	-4.536583	0.778977	0.989439
27	1	0	-2.866438	1.502779	2.126862
28	6	0	-4.914299	-0.015540	-0.091653
29	1	0	-4.312621	-1.303891	-1.710774
30	1	0	-5.286281	1.293713	1.576404
31	6	0	-0.800107	0.360449	0.879719
32	1	0	-0.590129	1.025293	1.726832
33	1	0	1.848527	-1.925632	1.675557
34	8	0	1.960281	-2.182918	-0.377596
35	1	0	1.072764	-1.922550	-0.681033

36	7	0	-6.338663	-0.138790	-0.415979
37	8	0	-7.149474	0.477070	0.280801
38	8	0	-6.657790	-0.853210	-1.369925

Structure 62 (M06-2X, Gas Phase)

Energy (Hartrees): = - 1140.8296339
No imaginary frequencies

Standard orientation:

Center Number	Atomic Number	Atomic Type	Coordinates (Angstroms) X	Y	Z
1	6	0	2.245944	-1.425244	0.781547
2	6	0	1.503651	-0.078616	0.669637
3	6	0	2.174169	0.776860	-0.409470
4	6	0	3.654256	0.895996	-0.102199
5	6	0	4.275041	-0.494755	-0.019007
6	1	0	2.061188	0.292139	-1.385425
7	1	0	3.771588	1.390901	0.873538
8	1	0	4.166108	-1.001473	-0.983510
9	1	0	1.582729	0.454752	1.626429
10	8	0	3.605281	-1.223957	1.005927
11	6	0	5.751649	-0.455256	0.349671
12	1	0	5.882883	0.176691	1.239943
13	1	0	6.063901	-1.468331	0.606312
14	8	0	6.548257	-0.023821	-0.727374
15	1	0	6.170356	0.801638	-1.049093
16	8	0	4.313100	1.634432	-1.112183
17	1	0	3.820906	2.454401	-1.225789
18	8	0	1.646279	2.092621	-0.436741
19	1	0	0.821782	2.085174	-0.929348
20	7	0	0.128018	-0.322934	0.289885
21	6	0	-2.206179	0.220445	0.510741
22	6	0	-2.625219	-0.672954	-0.479666
23	6	0	-3.145798	0.992400	1.194490
24	6	0	-3.969524	-0.795789	-0.783689
25	1	0	-1.882844	-1.263888	-1.000317
26	6	0	-4.497979	0.880094	0.901785
27	1	0	-2.817429	1.684076	1.962325
28	6	0	-4.879204	-0.013903	-0.083805
29	1	0	-4.327833	-1.476992	-1.543067
30	1	0	-5.249982	1.462526	1.415565
31	6	0	-0.776318	0.363319	0.851529
32	1	0	-0.537792	1.103021	1.626903
33	1	0	1.888133	-1.979884	1.654834
34	8	0	2.071445	-2.170718	-0.392720
35	1	0	1.151021	-2.059177	-0.657512
36	7	0	-6.318610	-0.141729	-0.404857
37	8	0	-7.091752	0.538710	0.230275
38	8	0	-6.624798	-0.917081	-1.281731

Structure 62 (M06-2X, DMSO)

Energy (Hartrees): = - 1140.8596948
No imaginary frequencies

Standard orientation:

Center Number	Atomic Number	Atomic Type	Coordinates (Angstroms) X	Y	Z
1	6	0	2.217871	-1.402125	0.800375
2	6	0	1.505303	-0.042626	0.682673
3	6	0	2.185896	0.802614	-0.398840
4	6	0	3.670423	0.884966	-0.107091
5	6	0	4.261423	-0.518184	-0.027272
6	1	0	2.046586	0.330415	-1.377969
7	1	0	3.814668	1.383744	0.861734
8	1	0	4.134650	-1.020703	-0.992694
9	1	0	1.585013	0.486006	1.640675
10	8	0	3.591878	-1.242485	1.000554
11	6	0	5.739380	-0.505780	0.334216
12	1	0	5.883724	0.107648	1.234313
13	1	0	6.048403	-1.526851	0.566035
14	8	0	6.540549	-0.052837	-0.739312
15	1	0	6.141805	0.769016	-1.049256
16	8	0	4.341909	1.600017	-1.132786
17	1	0	3.872183	2.435330	-1.246411
18	8	0	1.694345	2.131369	-0.414556
19	1	0	0.847516	2.139594	-0.874264
20	7	0	0.130256	-0.279051	0.292398
21	6	0	-2.206094	0.232229	0.516400

22	6	0	-2.609468	-0.620087	-0.516519
23	6	0	-3.157342	0.958424	1.234312
24	6	0	-3.951158	-0.749557	-0.828991
25	1	0	-1.864797	-1.177546	-1.070848
26	6	0	-4.507198	0.838078	0.935291
27	1	0	-2.838897	1.619217	2.032753
28	6	0	-4.873776	-0.016212	-0.092156
29	1	0	-4.283506	-1.402168	-1.624645
30	1	0	-5.258336	1.390297	1.482970
31	6	0	-0.780220	0.383953	0.873608
32	1	0	-0.559085	1.099044	1.673745
33	1	0	1.858810	-1.932017	1.687621
34	8	0	2.006186	-2.170694	-0.355992
35	1	0	1.111324	-1.966466	-0.659703
36	7	0	-6.305203	-0.153382	-0.418431
37	8	0	-7.101869	0.470282	0.250952
38	8	0	-6.608706	-0.883361	-1.338593

Structure 62 (M06-2X/def2-TZVP, Gas Phase)

Energy (Hartrees): = -1140.977994
No imaginary frequencies

Standard orientation:

Center Number	Atomic Number	Atomic Type	Coordinates (Angstroms) X	Y	Z
1	6	0	2.220317	-1.408931	0.762808
2	6	0	1.499852	-0.053815	0.637912
3	6	0	2.193670	0.793633	-0.428684
4	6	0	3.670982	0.890945	-0.107067
5	6	0	4.271663	-0.506529	-0.017320
6	1	0	2.080963	0.317162	-1.407806
7	1	0	3.785369	1.381747	0.869733
8	1	0	4.173094	-1.008498	-0.985091
9	1	0	1.577305	0.474313	1.596759
10	8	0	3.579374	-1.229746	0.992512
11	6	0	5.739236	-0.487857	0.382794
12	1	0	5.858593	0.133163	1.280620
13	1	0	6.034638	-1.505649	0.637727
14	8	0	6.572141	-0.057473	-0.665282
15	1	0	6.217924	0.772555	-1.006047
16	8	0	4.352443	1.622539	-1.105209
17	1	0	3.875387	2.450284	-1.236134
18	8	0	1.683098	2.114827	-0.456289
19	1	0	0.844939	2.121623	-0.927967
20	7	0	0.127001	-0.279015	0.249346
21	6	0	-2.211834	0.217885	0.507403
22	6	0	-2.630833	-0.644391	-0.506423
23	6	0	-3.151453	0.958797	1.219154
24	6	0	-3.973499	-0.766401	-0.805868
25	1	0	-1.890177	-1.213616	-1.051200
26	6	0	-4.502117	0.848018	0.930428
27	1	0	-2.823550	1.626544	2.006577
28	6	0	-4.882998	-0.014800	-0.078510
29	1	0	-4.328307	-1.426577	-1.583640
30	1	0	-5.252125	1.409934	1.467172
31	6	0	-0.783895	0.360817	0.847209
32	1	0	-0.551801	1.060192	1.659626
33	1	0	1.848332	-1.947184	1.639940
34	8	0	2.038408	-2.170975	-0.399021
35	1	0	1.131881	-2.026893	-0.698978
36	7	0	-6.320714	-0.141787	-0.395339
37	8	0	-7.094235	0.522607	0.253863
38	8	0	-6.628859	-0.901491	-1.283371

Structure 62 (M06-2X/def2-TZVP, DMSO)

Energy (Hartrees): = -1141.008530
No imaginary frequencies

Standard orientation:

Center Number	Atomic Number	Atomic Type	Coordinates (Angstroms) X	Y	Z
1	6	0	2.199416	-1.391079	0.783168
2	6	0	1.502668	-0.026862	0.655225
3	6	0	2.200512	0.815760	-0.413482
4	6	0	3.681914	0.883535	-0.109904
5	6	0	4.258670	-0.524068	-0.029722
6	1	0	2.063367	0.352447	-1.396392

7	1	0	3.822763	1.375602	0.861587
8	1	0	4.140997	-1.020160	-0.999235
9	1	0	1.580295	0.495602	1.615863
10	8	0	3.572467	-1.247699	0.983499
11	6	0	5.728565	-0.529148	0.358146
12	1	0	5.862493	0.072152	1.266266
13	1	0	6.024457	-1.554308	0.585036
14	8	0	6.561088	-0.073530	-0.688617
15	1	0	6.184314	0.755094	-1.012244
16	8	0	4.371465	1.597317	-1.122755
17	1	0	3.919135	2.442501	-1.244044
18	8	0	1.716624	2.146175	-0.427038
19	1	0	0.852469	2.161125	-0.856517
20	7	0	0.130645	-0.248548	0.257787
21	6	0	-2.209439	0.224323	0.511852
22	6	0	-2.617069	-0.614414	-0.526641
23	6	0	-3.156288	0.939256	1.241026
24	6	0	-3.957469	-0.740256	-0.833761
25	1	0	-1.877245	-1.166605	-1.090836
26	6	0	-4.504736	0.824218	0.946042
27	1	0	-2.834634	1.588587	2.046176
28	6	0	-4.875453	-0.016137	-0.087271
29	1	0	-4.290495	-1.385021	-1.634052
30	1	0	-5.250768	1.371234	1.503603
31	6	0	-0.785302	0.375025	0.867049
32	1	0	-0.567842	1.056343	1.695877
33	1	0	1.832874	-1.906304	1.675396
34	8	0	1.976090	-2.175711	-0.359817
35	1	0	1.098153	-1.943284	-0.695710
36	7	0	-6.305269	-0.148762	-0.408543
37	8	0	-7.097855	0.486347	0.253136
38	8	0	-6.616618	-0.887246	-1.317888

Structure 62.5H_2O (M06-2X, Gas Phase)

Energy (Hartrees): = - 1523.0128651
No imaginary frequencies

Standard orientation:

Center Number	Atomic Number	Atomic Type	Coordinates (Angstroms) X	Y	Z
1	6	0	3.675683	-0.279575	-1.802038
2	6	0	2.703238	-0.453741	-0.815796
3	6	0	3.086301	-0.574207	0.524806
4	6	0	4.425806	-0.527034	0.872398
5	6	0	5.366872	-0.357107	-0.134162
6	6	0	5.022093	-0.233441	-1.468905
7	6	0	1.283433	-0.478415	-1.220756
8	7	0	0.346691	-0.745327	-0.412820
9	6	0	-1.017423	-0.623073	-0.891233
10	6	0	-1.606384	0.684594	-0.349890
11	6	0	-3.079265	0.826320	-0.725966
12	6	0	-3.826519	-0.447572	-0.339437
13	8	0	-3.198530	-1.575187	-0.941880
14	6	0	-1.891435	-1.824993	-0.495451
15	8	0	-0.784465	1.731158	-0.851757
16	8	0	-1.878148	-2.154514	0.861166
17	6	0	-5.273887	-0.459701	-0.795231
18	8	0	-6.033301	-1.427442	-0.124772
19	8	0	-3.668839	1.896851	-0.008326
20	1	0	1.079242	-0.230811	-2.269381
21	1	0	-1.052349	-0.561646	-1.987461
22	1	0	-1.288748	2.561292	-0.937903
23	1	0	-1.555754	0.675299	0.747625
24	1	0	-3.509536	2.724068	-0.484159
25	1	0	-3.164659	0.985152	-1.810235
26	1	0	-5.495248	-2.227251	-0.006109
27	1	0	-5.714617	0.516236	-0.583448
28	1	0	-5.272863	-0.618146	-1.884114
29	1	0	-3.808577	-0.548584	0.753862
30	1	0	-1.453189	-1.497463	1.441680
31	1	0	-1.571504	-2.707166	-1.052874
32	1	0	3.376054	-0.181165	-2.839327
33	1	0	5.795754	-0.103401	-2.212847
34	1	0	4.753868	-0.619091	1.898528
35	1	0	2.336081	-0.704135	1.294435
36	1	0	-4.789769	-3.288788	1.956153
37	8	0	-4.458134	-3.421390	1.065472
38	1	0	-3.546978	-3.094905	1.084946
39	1	0	-2.055729	4.139115	0.214587
40	8	0	-2.217766	4.086424	-0.754854
41	1	0	-2.154206	4.963067	-1.136804
42	8	0	-1.918215	3.389977	1.764362
43	1	0	-1.075860	2.936179	1.921166

44	1	0	-2.585249	2.698041	1.659969
45	1	0	0.325637	1.928190	0.573106
46	8	0	0.527059	1.980245	1.528417
47	1	0	1.402568	2.364103	1.614939
48	1	0	0.310251	-0.845691	1.504120
49	8	0	-0.109908	-0.668025	2.362811
50	1	0	0.022183	0.284589	2.454352
51	7	0	6.799587	-0.300326	0.235088
52	8	0	7.598929	-0.166248	-0.663236
53	8	0	7.073590	-0.389328	1.410010

Structure 62.5H_2O (M06-2X, DMSO)

Energy (Hartrees): = - 1523.0553803
No imaginary frequencies

Standard orientation:

Center Number	Atomic Number	Atomic Type	Coordinates (Angstroms) X	Y	Z
1	6	0	3.718777	-0.309572	-1.793097
2	6	0	2.752521	-0.423211	-0.791463
3	6	0	3.143531	-0.477676	0.550672
4	6	0	4.484357	-0.424568	0.888992
5	6	0	5.419795	-0.318145	-0.132535
6	6	0	5.067678	-0.259379	-1.470980
7	6	0	1.332475	-0.463794	-1.195087
8	7	0	0.390317	-0.646729	-0.366727
9	6	0	-0.970085	-0.592052	-0.875091
10	6	0	-1.645974	0.687025	-0.370618
11	6	0	-3.108682	0.729846	-0.806814
12	6	0	-3.792236	-0.565877	-0.380209
13	8	0	-3.098461	-1.690724	-0.915693
14	6	0	-1.773511	-1.836502	-0.467661
15	8	0	-0.874219	1.774074	-0.859321
16	8	0	-1.747365	-2.139491	0.901164
17	6	0	-5.222359	-0.676402	-0.868221
18	8	0	-5.943405	-1.684461	-0.195471
19	8	0	-3.793473	1.791982	-0.158088
20	1	0	1.138358	-0.316718	-2.262608
21	1	0	-0.984660	-0.558116	-1.971795
22	1	0	-1.420307	2.578824	-0.938635
23	1	0	-1.641399	0.697824	0.728946
24	1	0	-3.624286	2.614711	-0.641673
25	1	0	-3.165868	0.839184	-1.897696
26	1	0	-5.322016	-2.369986	0.100629
27	1	0	-5.729506	0.275229	-0.697824
28	1	0	-5.191011	-0.862710	-1.951005
29	1	0	-3.788979	-0.613578	0.717847
30	1	0	-1.379006	-1.428676	1.458781
31	1	0	-1.389269	-2.704882	-1.004944
32	1	0	3.411752	-0.260612	-2.831688
33	1	0	5.827124	-0.172443	-2.236011
34	1	0	4.805138	-0.462687	1.920915
35	1	0	2.403317	-0.557718	1.336337
36	1	0	-4.563395	-3.123009	2.171443
37	8	0	-4.275144	-3.395849	1.294313
38	1	0	-3.381925	-3.026029	1.204169
39	1	0	-2.329814	4.175711	0.119818
40	8	0	-2.438443	4.046615	-0.844560
41	1	0	-2.339060	4.894388	-1.289277
42	8	0	-2.262410	3.391948	1.736815
43	1	0	-1.381361	3.005238	1.872551
44	1	0	-2.837094	2.648344	1.506695
45	1	0	0.174860	2.068035	0.642093
46	8	0	0.270172	2.171270	1.608019
47	1	0	1.100971	2.635265	1.760590
48	1	0	0.335773	-0.663052	1.534811
49	8	0	-0.104957	-0.517349	2.393209
50	1	0	-0.074521	0.448564	2.456924
51	7	0	6.849482	-0.260269	0.224172
52	8	0	7.657813	-0.235638	-0.679768
53	8	0	7.138625	-0.241162	1.402025

Structure 62.5H_2O (M06-2X, H_2O)

Energy (Hartrees): = - 1523.0752303
No imaginary frequencies

Standard orientation:

Center Number	Atomic Number	Atomic Type	Coordinates (Angstroms) X	Y	Z

1	6	0	3.671596	-0.390472	-1.788549
2	6	0	2.688935	-0.481020	-0.800335
3	6	0	3.054562	-0.507165	0.549996
4	6	0	4.390134	-0.455379	0.909107
5	6	0	5.341702	-0.374802	-0.099697
6	6	0	5.014593	-0.340746	-1.445706
7	6	0	1.276895	-0.520410	-1.228175
8	7	0	0.324698	-0.791038	-0.432856
9	6	0	-1.025184	-0.669611	-0.960441
10	6	0	-1.605888	0.672219	-0.499415
11	6	0	-3.079594	0.795774	-0.874982
12	6	0	-3.828151	-0.438247	-0.383492
13	8	0	-3.232292	-1.618961	-0.926908
14	6	0	-1.900853	-1.849043	-0.521162
15	8	0	-0.800916	1.680846	-1.089576
16	8	0	-1.832710	-2.158915	0.849501
17	6	0	-5.280159	-0.457703	-0.810243
18	8	0	-6.039105	-1.416199	-0.090446
19	8	0	-3.668158	1.915967	-0.225619
20	1	0	1.096418	-0.299082	-2.284823
21	1	0	-1.030707	-0.669571	-2.057392
22	1	0	-1.190767	2.563518	-0.919858
23	1	0	-1.545488	0.735162	0.594994
24	1	0	-3.363741	2.730802	-0.651728
25	1	0	-3.179195	0.883363	-1.963862
26	1	0	-5.445061	-2.122250	0.218435
27	1	0	-5.721174	0.523752	-0.631537
28	1	0	-5.312503	-0.662352	-1.887773
29	1	0	-3.774866	-0.470317	0.713805
30	1	0	-1.616758	-1.388134	1.400562
31	1	0	-1.595940	-2.748335	-1.054827
32	1	0	3.380898	-0.361502	-2.832266
33	1	0	5.787298	-0.274040	-2.199050
34	1	0	4.693493	-0.471315	1.946686
35	1	0	2.297054	-0.561477	1.321169
36	1	0	-4.708507	-2.730410	2.208582
37	8	0	-4.444487	-3.181153	1.398883
38	1	0	-3.536827	-2.871007	1.241514
39	1	0	-1.916901	4.066027	0.393373
40	8	0	-1.969041	4.109649	-0.579160
41	1	0	-1.530317	4.922786	-0.849098
42	8	0	-2.013450	3.057532	1.970744
43	1	0	-1.135094	2.659092	1.833315
44	1	0	-2.627145	2.478897	1.494208
45	1	0	0.375703	1.954282	0.404555
46	8	0	0.576904	2.059353	1.350572
47	1	0	1.208509	2.784935	1.404021
48	1	0	0.200501	-0.704630	1.502206
49	8	0	-0.164250	-0.460262	2.373635
50	1	0	-0.042067	0.500861	2.364361
51	7	0	6.761309	-0.310296	0.279262
52	8	0	7.582687	-0.157478	-0.601728
53	8	0	7.044771	-0.412890	1.455621

Structure 62·5H_2O (M06-2X/def2-TZVP, Gas Phase)

Energy (Hartrees): = -1523.199068
No imaginary frequencies

Standard orientation:

Center Number	Atomic Number	Atomic Type	Coordinates (Angstroms) X	Y	Z
1	6	0	4.014871	-0.027819	-1.653343
2	6	0	2.935930	-0.341831	-0.831227
3	6	0	3.156446	-0.718188	0.494586
4	6	0	4.443983	-0.792670	0.990637
5	6	0	5.496836	-0.484872	0.143192
6	6	0	5.311951	-0.102071	-1.171332
7	6	0	1.564827	-0.244752	-1.365721
8	7	0	0.569559	-0.691297	-0.730994
9	6	0	-0.756696	-0.445771	-1.250960
10	6	0	-1.310993	0.793960	-0.547202
11	6	0	-2.795471	0.981790	-0.835482
12	6	0	-3.531343	-0.311931	-0.504390
13	8	0	-2.970064	-1.392289	-1.244420
14	6	0	-1.637661	-1.667416	-0.971833
15	8	0	-0.507973	1.880157	-0.976812
16	8	0	-1.509482	-2.075401	0.368525
17	6	0	-4.998209	-0.264240	-0.872266
18	8	0	-5.725249	-1.331563	-0.302499
19	8	0	-3.342542	1.991114	-0.011240
20	1	0	1.452837	0.256237	-2.333560
21	1	0	-0.755978	-0.250815	-2.330346
22	1	0	-0.948711	2.735581	-0.817867
23	1	0	-1.205023	0.653933	0.537299

24	1	0	-3.109339	2.866793	-0.352342
25	1	0	-2.934835	1.220653	-1.898454
26	1	0	-5.164199	-2.130091	-0.262459
27	1	0	-5.426615	0.665573	-0.498681
28	1	0	-5.082097	-0.279655	-1.965281
29	1	0	-3.438261	-0.503817	0.571399
30	1	0	-0.594206	-1.916782	0.638522
31	1	0	-1.365513	-2.487455	-1.641865
32	1	0	3.838217	0.274105	-2.678595
33	1	0	6.167520	0.131974	-1.787502
34	1	0	4.647193	-1.078370	2.012380
35	1	0	2.304068	-0.932394	1.125229
36	1	0	-4.425349	-2.823025	1.721462
37	8	0	-4.132394	-3.287611	0.922704
38	1	0	-3.195398	-3.072016	0.827250
39	1	0	-1.628487	4.113009	0.666350
40	8	0	-1.783984	4.269590	-0.296396
41	1	0	-1.660991	5.199953	-0.494282
42	8	0	-1.476188	3.153819	2.078721
43	1	0	-0.626675	2.677872	2.071032
44	1	0	-2.165164	2.493780	1.924099
45	1	0	0.732416	1.870937	0.466086
46	8	0	0.932242	1.841788	1.418291
47	1	0	1.846773	2.113205	1.527854
48	8	0	-5.382667	-1.248208	2.438143
49	1	0	-5.746301	-1.157073	1.537884
50	1	0	-6.120710	-1.187739	3.048119
51	7	0	6.875203	-0.558171	0.670233
52	8	0	7.776918	-0.289090	-0.088932
53	8	0	7.011965	-0.881460	1.826869

Structure 62·5H_2O (M06-2X/def2-TZVP, DMSO)

Energy (Hartrees): = -1523.238960
No imaginary frequencies

Standard orientation:

Center Number	Atomic Number	Atomic Type	Coordinates (Angstroms) X	Y	Z
1	6	0	4.050281	0.056545	-1.618862
2	6	0	2.987508	-0.275835	-0.782713
3	6	0	3.234007	-0.719094	0.517661
4	6	0	4.530694	-0.834261	0.978802
5	6	0	5.567209	-0.500719	0.119385
6	6	0	5.356944	-0.056736	-1.173006
7	6	0	1.611748	-0.140838	-1.297999
8	7	0	0.607781	-0.508354	-0.624497
9	6	0	-0.712792	-0.302467	-1.178020
10	6	0	-1.360227	0.885524	-0.467705
11	6	0	-2.844545	0.987846	-0.798245
12	6	0	-3.506580	-0.350812	-0.502045
13	8	0	-2.872461	-1.386470	-1.247617
14	6	0	-1.525573	-1.582568	-0.960352
15	8	0	-0.624944	2.034815	-0.852478
16	8	0	-1.404062	-2.041157	0.368563
17	6	0	-4.964288	-0.393887	-0.894735
18	8	0	-5.583848	-1.589182	-0.442480
19	8	0	-3.473033	1.957250	0.024765
20	1	0	1.511097	0.296134	-2.296021
21	1	0	-0.687734	-0.088939	-2.252537
22	1	0	-1.147677	2.847329	-0.720609
23	1	0	-1.278915	0.732751	0.617984
24	1	0	-3.320636	2.841539	-0.341869
25	1	0	-2.973010	1.235256	-1.859186
26	1	0	-4.930070	-2.310602	-0.460085
27	1	0	-5.492114	0.443889	-0.440405
28	1	0	-5.043334	-0.310874	-1.983938
29	1	0	-3.417096	-0.556214	0.572199
30	1	0	-0.516853	-1.805522	0.680818
31	1	0	-1.185880	-2.355813	-1.653546
32	1	0	3.852468	0.403436	-2.625764
33	1	0	6.192196	0.194644	-1.810043
34	1	0	4.740049	-1.173326	1.982911
35	1	0	2.402545	-0.966280	1.164360
36	1	0	-4.304441	-3.116859	1.538075
37	8	0	-3.861872	-3.544813	0.787711
38	1	0	-2.988821	-3.129122	0.729528
39	1	0	-1.971463	4.305404	0.636449
40	8	0	-2.131478	4.351963	-0.329202
41	1	0	-2.016427	5.262073	-0.623302
42	8	0	-1.769437	3.297746	2.128189
43	1	0	-0.859522	2.946957	2.080347
44	1	0	-2.344618	2.577768	1.831920

45	1	0	0.581031	2.172173	0.609731
46	8	0	0.773280	2.277392	1.558489
47	1	0	1.608074	2.754859	1.619651
48	8	0	-5.497845	-1.806502	2.329333
49	1	0	-5.753333	-1.608898	1.409455
50	1	0	-6.313483	-1.990173	2.807377
51	7	0	6.951092	-0.622811	0.604656
52	8	0	7.848758	-0.351459	-0.163470
53	8	0	7.121883	-0.989536	1.747245

Structure 62·5H_2O (M06-2X/def2-TZVP, H_2O)

Energy (Hartrees): = -1523.255604
No imaginary frequencies

Standard orientation:

Center Number	Atomic Number	Atomic Type	Coordinates (Angstroms) X	Y	Z
1	6	0	4.002464	0.001484	-1.626472
2	6	0	2.962377	-0.313879	-0.754786
3	6	0	3.243998	-0.700285	0.556612
4	6	0	4.552122	-0.782952	0.991346
5	6	0	5.563542	-0.479359	0.092491
6	6	0	5.320246	-0.087136	-1.210757
7	6	0	1.577812	-0.217769	-1.253001
8	7	0	0.590300	-0.674381	-0.604285
9	6	0	-0.733139	-0.448294	-1.148190
10	6	0	-1.328408	0.789798	-0.482111
11	6	0	-2.798301	0.950478	-0.842646
12	6	0	-3.557094	-0.340546	-0.559822
13	8	0	-2.933709	-1.445919	-1.212109
14	6	0	-1.596838	-1.675735	-0.865892
15	8	0	-0.549737	1.894738	-0.908749
16	8	0	-1.530705	-2.041221	0.494731
17	6	0	-4.968137	-0.281763	-1.100636
18	8	0	-5.724766	-1.427458	-0.731591
19	8	0	-3.400894	1.961217	-0.047343
20	1	0	1.454345	0.262490	-2.227771
21	1	0	-0.714503	-0.283593	-2.230748
22	1	0	-0.967575	2.737615	-0.638589
23	1	0	-1.259061	0.675570	0.608218
24	1	0	-3.166024	2.832807	-0.398211
25	1	0	-2.883583	1.198166	-1.907005
26	1	0	-5.217033	-2.216225	-0.966599
27	1	0	-5.483878	0.583173	-0.687356
28	1	0	-4.930572	-0.186127	-2.190001
29	1	0	-3.581825	-0.515336	0.522473
30	1	0	-0.657918	-1.790981	0.833712
31	1	0	-1.277749	-2.511385	-1.491152
32	1	0	3.776533	0.311799	-2.639018
33	1	0	6.137314	0.145777	-1.877488
34	1	0	4.788405	-1.076155	2.003669
35	1	0	2.435107	-0.927530	1.236798
36	1	0	-4.501874	-2.551321	1.807712
37	8	0	-3.892185	-3.262656	1.548760
38	1	0	-3.094971	-2.813589	1.227016
39	1	0	-1.679191	4.159415	0.742965
40	8	0	-1.771480	4.316938	-0.214896
41	1	0	-1.292954	5.130215	-0.411910
42	8	0	-1.666052	2.938931	2.212790
43	1	0	-0.777897	2.579323	2.028322
44	1	0	-2.273124	2.397690	1.683988
45	1	0	0.693262	1.986612	0.557742
46	8	0	0.957814	2.036029	1.493761
47	1	0	1.588926	2.763884	1.541940
48	8	0	-5.836851	-1.205286	2.041006
49	1	0	-5.941058	-1.282925	1.073001
50	1	0	-6.637371	-1.587426	2.418409
51	7	0	6.955200	-0.576963	0.546976
52	8	0	7.838641	-0.389657	-0.263507
53	8	0	7.160166	-0.844006	1.712676

Structure 62.5H_2O* (M06-2X, Gas Phase)

Energy (Hartrees): = - 1523.0126908
No imaginary frequencies

Standard orientation:

Center Number	Atomic Number	Atomic Type	Coordinates (Angstroms) X	Y	Z

1	6	0	4.007220	-0.090709	-1.691262
2	6	0	2.916098	-0.332036	-0.856319
3	6	0	3.119381	-0.610966	0.499290
4	6	0	4.404415	-0.666225	1.012260
5	6	0	5.470113	-0.437125	0.151776
6	6	0	5.301323	-0.148118	-1.191962
7	6	0	1.545119	-0.265088	-1.402395
8	7	0	0.557653	-0.721417	-0.756834
9	6	0	-0.775191	-0.490514	-1.275365
10	6	0	-1.323700	0.758803	-0.579186
11	6	0	-2.813862	0.939798	-0.852752
12	6	0	-3.540708	-0.353111	-0.492560
13	8	0	-2.986506	-1.446458	-1.226532
14	6	0	-1.648967	-1.711013	-0.960102
15	8	0	-0.520345	1.839036	-1.028351
16	8	0	-1.506108	-2.077503	0.392110
17	6	0	-5.015728	-0.314362	-0.839019
18	8	0	-5.746377	-1.336114	-0.190158
19	8	0	-3.358185	1.952201	-0.026410
20	1	0	1.425869	0.221988	-2.377421
21	1	0	-0.784734	-0.310369	-2.358227
22	1	0	-0.965273	2.693001	-0.875479
23	1	0	-1.206665	0.627771	0.505215
24	1	0	-3.115594	2.823284	-0.371799
25	1	0	-2.968109	1.170142	-1.916717
26	1	0	-5.189912	-2.135767	-0.120229
27	1	0	-5.426143	0.642602	-0.513509
28	1	0	-5.115127	-0.396405	-1.928618
29	1	0	-3.429734	-0.526024	0.584318
30	1	0	-0.584642	-1.921311	0.637319
31	1	0	-1.380270	-2.547952	-1.611004
32	1	0	3.843404	0.137315	-2.738592
33	1	0	6.167709	0.026969	-1.814520
34	1	0	4.598140	-0.877781	2.054885
35	1	0	2.255791	-0.759222	1.135048
36	1	0	-4.302076	-2.622359	1.823005
37	8	0	-4.107601	-3.179013	1.054653
38	1	0	-3.169511	-3.029697	0.884607
39	1	0	-1.630444	4.012489	0.621797
40	8	0	-1.800962	4.176234	-0.336738
41	1	0	-1.678553	5.106625	-0.529749
42	8	0	-1.481659	3.021103	1.991812
43	1	0	-0.644070	2.527286	2.001007
44	1	0	-2.180760	2.375821	1.825482
45	1	0	0.721919	1.797330	0.401713
46	8	0	0.906245	1.711065	1.352099
47	1	0	1.816568	1.983910	1.482742
48	8	0	-5.060624	-0.947975	2.436714
49	1	0	-5.572339	-0.975119	1.608568
50	1	0	-5.680155	-0.738111	3.136988
51	7	0	6.845681	-0.491798	0.695447
52	8	0	7.757130	-0.300740	-0.077564
53	8	0	6.967278	-0.723078	1.876923

Structure 62.5H_2O* (M06-2X, DMSO)

Energy (Hartrees): = - 1523.0512248
No imaginary frequencies

Standard orientation:

Center Number	Atomic Number	Atomic Type	Coordinates (Angstroms) X	Y	Z
1	6	0	4.026749	-0.059407	-1.682677
2	6	0	2.945825	-0.289616	-0.830953
3	6	0	3.164892	-0.585971	0.518464
4	6	0	4.455604	-0.670867	1.011404
5	6	0	5.511333	-0.451668	0.134467
6	6	0	5.326933	-0.143992	-1.204309
7	6	0	1.572229	-0.198837	-1.367523
8	7	0	0.578218	-0.606571	-0.696924
9	6	0	-0.750739	-0.395524	-1.236226
10	6	0	-1.349469	0.822576	-0.529021
11	6	0	-2.841586	0.958232	-0.816696
12	6	0	-3.531270	-0.362295	-0.491256
13	8	0	-2.934847	-1.431537	-1.228025
14	6	0	-1.585790	-1.649061	-0.957263
15	8	0	-0.589069	1.941266	-0.955873
16	8	0	-1.450561	-2.046879	0.391352
17	6	0	-4.996949	-0.371814	-0.866595
18	8	0	-5.670246	-1.504280	-0.331205
19	8	0	-3.426854	1.938734	0.028147
20	1	0	1.464044	0.243712	-2.363127
21	1	0	-0.745749	-0.204708	-2.316036
22	1	0	-1.071836	2.771006	-0.785889

23	1	0	-1.238915	0.681580	0.555792
24	1	0	-3.222140	2.820443	-0.318512
25	1	0	-2.995963	1.209604	-1.874341
26	1	0	-5.036824	-2.241908	-0.263392
27	1	0	-5.478373	0.521454	-0.466307
28	1	0	-5.078785	-0.358642	-1.959666
29	1	0	-3.432854	-0.549750	0.585469
30	1	0	-0.540723	-1.849976	0.658588
31	1	0	-1.277346	-2.460044	-1.622012
32	1	0	3.848872	0.182679	-2.724478
33	1	0	6.177388	0.026179	-1.849959
34	1	0	4.647627	-0.897358	2.051336
35	1	0	2.316355	-0.736421	1.173689
36	1	0	-4.278087	-2.756703	1.695327
37	8	0	-3.963048	-3.315094	0.967194
38	1	0	-3.053469	-3.021605	0.818390
39	1	0	-1.788531	4.153675	0.637319
40	8	0	-1.998804	4.233397	-0.316847
41	1	0	-1.873810	5.146413	-0.593344
42	8	0	-1.574963	3.097642	2.046660
43	1	0	-0.701620	2.668271	1.992307
44	1	0	-2.213971	2.425412	1.774465
45	1	0	0.689643	1.911038	0.466585
46	8	0	0.848724	1.889255	1.424333
47	1	0	1.759298	2.171114	1.555617
48	8	0	-5.230816	-1.262837	2.377132
49	1	0	-5.663059	-1.241145	1.505627
50	1	0	-5.938387	-1.284887	3.028258
51	7	0	6.888877	-0.541467	0.652769
52	8	0	7.800119	-0.316366	-0.115922
53	8	0	7.036737	-0.836262	1.820113

Structure 62.5H_2O* (M06-2X, H_2O)

Energy (Hartrees): = - 1523.0704055
No imaginary frequencies

Standard orientation:

Center Number	Atomic Number	Atomic Type	Coordinates (Angstroms) X	Y	Z
1	6	0	3.995382	-0.058850	-1.646251
2	6	0	2.935434	-0.336604	-0.781527
3	6	0	3.187005	-0.656676	0.556699
4	6	0	4.487782	-0.716782	1.025149
5	6	0	5.520489	-0.452522	0.133698
6	6	0	5.305824	-0.122427	-1.194989
7	6	0	1.556115	-0.267552	-1.304413
8	7	0	0.570266	-0.735641	-0.656124
9	6	0	-0.755858	-0.509290	-1.202201
10	6	0	-1.324863	0.753251	-0.552025
11	6	0	-2.806254	0.923736	-0.874331
12	6	0	-3.556132	-0.355907	-0.521665
13	8	0	-2.972985	-1.475929	-1.196033
14	6	0	-1.629645	-1.722242	-0.880858
15	8	0	-0.539111	1.837214	-1.022373
16	8	0	-1.541745	-2.060785	0.487249
17	6	0	-5.004203	-0.318600	-0.957748
18	8	0	-5.758003	-1.389108	-0.393979
19	8	0	-3.375283	1.958881	-0.081609
20	1	0	1.435896	0.206366	-2.283577
21	1	0	-0.743580	-0.364997	-2.288572
22	1	0	-0.943639	2.688945	-0.759230
23	1	0	-1.230024	0.659516	0.538767
24	1	0	-3.111786	2.818574	-0.441944
25	1	0	-2.926741	1.144560	-1.942073
26	1	0	-5.166562	-2.148321	-0.256715
27	1	0	-5.461076	0.613187	-0.623891
28	1	0	-5.042103	-0.361564	-2.051725
29	1	0	-3.504260	-0.509645	0.562819
30	1	0	-0.640300	-1.865241	0.781789
31	1	0	-1.338100	-2.573677	-1.498964
32	1	0	3.791896	0.202178	-2.678439
33	1	0	6.138947	0.081570	-1.852973
34	1	0	4.703708	-0.959023	2.056578
35	1	0	2.360085	-0.846233	1.227854
36	1	0	-4.447077	-2.463085	1.733951
37	8	0	-4.105187	-3.160195	1.149927
38	1	0	-3.191960	-2.886489	0.978354
39	1	0	-1.603807	4.071874	0.635056
40	8	0	-1.740033	4.214616	-0.320248
41	1	0	-1.295151	5.036998	-0.548680
42	8	0	-1.590157	2.916530	2.090961
43	1	0	-0.722025	2.507919	1.918538
44	1	0	-2.223455	2.370010	1.602054

45	1	0	0.698700	1.914272	0.423865
46	8	0	0.963797	1.910935	1.359542
47	1	0	1.593291	2.635901	1.439128
48	8	0	-5.366180	-0.896101	2.340315
49	1	0	-5.756312	-0.969239	1.452401
50	1	0	-6.075750	-1.137189	2.944920
51	7	0	6.904902	-0.523976	0.624307
52	8	0	7.805239	-0.350088	-0.171928
53	8	0	7.083218	-0.756851	1.802700

Structure 62.6H_2O (B3LYP, Gas Phase)

Energy (Hartrees): = - 1599.6215521
No imaginary frequencies

Standard orientation:

Center Number	Atomic Number	Atomic Type	Coordinates (Angstroms) X	Y	Z
1	6	0	4.172414	0.086239	-1.743183
2	6	0	3.170689	-0.209212	-0.804359
3	6	0	3.537618	-0.579993	0.502967
4	6	0	4.876939	-0.644379	0.863242
5	6	0	5.847055	-0.337455	-0.091883
6	6	0	5.517673	0.026702	-1.394239
7	6	0	1.763434	-0.124468	-1.230555
8	7	0	0.778318	-0.279628	-0.434990
9	6	0	-0.572697	-0.208033	-0.967900
10	6	0	-1.391339	0.885649	-0.264496
11	6	0	-2.830509	0.920607	-0.808040
12	6	0	-3.435430	-0.481534	-0.658938
13	8	0	-2.607534	-1.425798	-1.354286
14	6	0	-1.306821	-1.573513	-0.824547
15	8	0	-0.702466	2.128331	-0.429077
16	8	0	-1.348852	-2.103278	0.469734
17	6	0	-4.839069	-0.634481	-1.229487
18	8	0	-5.492008	-1.802262	-0.755741
19	8	0	-3.616559	1.844224	-0.056694
20	1	0	1.601279	0.087530	-2.296873
21	1	0	-0.559090	0.017791	-2.044992
22	1	0	-1.282690	2.826842	-0.062277
23	1	0	-1.458340	0.644022	0.804743
24	1	0	-3.699846	2.689922	-0.551757
25	1	0	-2.809250	1.207799	-1.868515
26	1	0	-4.832922	-2.533854	-0.657080
27	1	0	-5.440639	0.226431	-0.925074
28	1	0	-4.770565	-0.640891	-2.328073
29	1	0	-3.469480	-0.730215	0.409907
30	1	0	-0.931350	-1.545333	1.164900
31	1	0	-0.827755	-2.304624	-1.486098
32	1	0	3.893711	0.367694	-2.754579
33	1	0	6.305186	0.252732	-2.101588
34	1	0	5.184708	-0.929649	1.861164
35	1	0	2.771951	-0.823012	1.231318
36	1	0	-4.371391	-3.351800	1.200962
37	8	0	-3.884948	-3.677871	0.417757
38	1	0	-2.994092	-3.298474	0.506919
39	1	0	-2.957156	4.599803	-0.075834
40	8	0	-3.468747	4.443046	-0.891734
41	1	0	-4.272867	4.969083	-0.797153
42	8	0	-2.177704	3.707618	1.440154
43	1	0	-1.434796	3.428989	2.008948
44	1	0	-2.775786	2.938806	1.386633
45	1	0	0.198010	2.246343	1.226730
46	8	0	0.158771	2.313876	2.206087
47	1	0	0.992756	2.714030	2.483328
48	1	0	0.662972	-0.484796	1.404481
49	8	0	0.215297	-0.653113	2.261897
50	1	0	0.049604	0.243339	2.599689
51	8	0	-5.695345	-2.194633	1.984570
52	1	0	-5.824182	-1.896419	1.057731
53	1	0	-6.472524	-2.735237	2.174554
54	7	0	7.269516	-0.402075	0.290329
55	8	0	8.103457	-0.138107	-0.574562
56	8	0	7.532884	-0.713499	1.450522

Structure 62.6H_2O (B3LYP, DMSO)

Energy (Hartrees): = - 1599.663127
No imaginary frequencies

Standard orientation:

Center Number	Atomic Number	Atomic Type	Coordinates (Angstroms) X	Y	Z

1	6	0	4.151914	0.003607	-1.750560
2	6	0	3.158086	-0.214390	-0.782013
3	6	0	3.535517	-0.499906	0.543029
4	6	0	4.876586	-0.564488	0.892964
5	6	0	5.840013	-0.339855	-0.093139
6	6	0	5.499778	-0.055517	-1.413993
7	6	0	1.750493	-0.133428	-1.208586
8	7	0	0.761453	-0.293050	-0.415305
9	6	0	-0.582868	-0.201380	-0.973447
10	6	0	-1.397543	0.904346	-0.281148
11	6	0	-2.834218	0.947965	-0.831176
12	6	0	-3.446455	-0.447376	-0.662841
13	8	0	-2.637620	-1.412432	-1.354683
14	6	0	-1.323011	-1.560227	-0.850133
15	8	0	-0.701404	2.140823	-0.453627
16	8	0	-1.337242	-2.126520	0.438358
17	6	0	-4.854144	-0.601027	-1.213991
18	8	0	-5.480610	-1.802262	-0.759076
19	8	0	-3.622511	1.880468	-0.089786
20	1	0	1.593994	0.078397	-2.273222
21	1	0	-0.547641	0.026363	-2.047792
22	1	0	-1.249661	2.832955	-0.031582
23	1	0	-1.475927	0.676964	0.790502
24	1	0	-3.646421	2.740330	-0.569246
25	1	0	-2.813168	1.221652	-1.894232
26	1	0	-4.796953	-2.506311	-0.666375
27	1	0	-5.469253	0.237599	-0.878649
28	1	0	-4.812837	-0.580865	-2.312671
29	1	0	-3.468799	-0.680794	0.410699
30	1	0	-1.007539	-1.522772	1.142887
31	1	0	-0.852928	-2.281834	-1.525688
32	1	0	3.864478	0.222256	-2.774335
33	1	0	6.271931	0.112716	-2.153646
34	1	0	5.179810	-0.783742	1.908655
35	1	0	2.780437	-0.672264	1.300942
36	1	0	-4.348038	-3.339348	1.192408
37	8	0	-3.825749	-3.691005	0.443097
38	1	0	-2.971604	-3.223763	0.499812
39	1	0	-2.864868	4.614540	-0.081507
40	8	0	-3.359513	4.478651	-0.911162
41	1	0	-4.174237	4.991929	-0.802235
42	8	0	-2.140404	3.698658	1.481984
43	1	0	-1.388935	3.369907	2.015311
44	1	0	-2.752774	2.943928	1.403332
45	1	0	0.261716	2.206521	1.264509
46	8	0	0.149899	2.249777	2.236595
47	1	0	0.966057	2.645545	2.579246
48	1	0	0.614187	-0.559209	1.423231
49	8	0	0.135949	-0.671863	2.277112
50	1	0	0.005192	0.252811	2.556498
51	8	0	-5.696785	-2.238754	1.981071
52	1	0	-5.809512	-1.923504	1.057513
53	1	0	-6.463180	-2.811087	2.132259
54	7	0	7.258513	-0.404888	0.275314
55	8	0	8.097004	-0.169667	-0.598087
56	8	0	7.542963	-0.690035	1.441144

Structure 62.6H_2O (B3LYP, H_2O)

Energy (Hartrees): = - 1599.6843406
No imaginary frequencies

Standard orientation:

Center Number	Atomic Number	Atomic Type	Coordinates (Angstroms) X	Y	Z
1	6	0	4.135232	0.069267	-1.755665
2	6	0	3.134082	-0.198143	-0.807180
3	6	0	3.498959	-0.532334	0.510193
4	6	0	4.836104	-0.597397	0.872281
5	6	0	5.807454	-0.327082	-0.095237
6	6	0	5.479692	0.005791	-1.408460
7	6	0	1.729967	-0.116570	-1.243894
8	7	0	0.738499	-0.346227	-0.470093
9	6	0	-0.601875	-0.225492	-1.032469
10	6	0	-1.388598	0.900321	-0.336154
11	6	0	-2.831407	0.953053	-0.871938
12	6	0	-3.466753	-0.427102	-0.679937
13	8	0	-2.687878	-1.405057	-1.395891
14	6	0	-1.359801	-1.573559	-0.928993
15	8	0	-0.686774	2.123550	-0.549673
16	8	0	-1.337063	-2.183504	0.343928
17	6	0	-4.887577	-0.548905	-1.202952
18	8	0	-5.540767	-1.733561	-0.730299
19	8	0	-3.611388	1.913357	-0.158532

20	1	0	1.579270	0.154149	-2.295477
21	1	0	-0.558566	0.011574	-2.104772
22	1	0	-1.095761	2.787324	0.039179
23	1	0	-1.444809	0.692624	0.739406
24	1	0	-3.491964	2.790954	-0.591774
25	1	0	-2.812044	1.202275	-1.941294
26	1	0	-4.875376	-2.451764	-0.651714
27	1	0	-5.475987	0.303992	-0.858887
28	1	0	-4.868245	-0.535927	-2.301540
29	1	0	-3.467131	-0.667268	0.391800
30	1	0	-1.092651	-1.564951	1.067308
31	1	0	-0.916769	-2.285969	-1.630019
32	1	0	3.855609	0.328144	-2.771860
33	1	0	6.257166	0.208329	-2.133318
34	1	0	5.129361	-0.852990	1.882106
35	1	0	2.734947	-0.737902	1.250356
36	1	0	-4.330980	-3.262154	1.190530
37	8	0	-3.834074	-3.681546	0.458644
38	1	0	-2.984860	-3.198273	0.457018
39	1	0	-2.586046	4.547195	-0.123923
40	8	0	-3.069100	4.501433	-0.970517
41	1	0	-3.892293	4.980877	-0.794022
42	8	0	-2.058206	3.668295	1.565751
43	1	0	-1.358645	3.219175	2.087139
44	1	0	-2.702164	2.962875	1.373927
45	1	0	0.375073	2.107402	1.691299
46	8	0	-0.007982	2.068891	2.583122
47	1	0	0.675579	2.448371	3.156105
48	1	0	0.561674	-0.646691	1.390556
49	8	0	0.128612	-0.782135	2.264345
50	1	0	-0.022331	0.137469	2.560697
51	8	0	-5.540905	-2.044593	2.058740
52	1	0	-5.706804	-1.785421	1.126271
53	1	0	-6.269437	-2.649572	2.260723
54	7	0	7.216911	-0.399557	0.283138
55	8	0	8.070892	-0.199627	-0.586611
56	8	0	7.497563	-0.659809	1.457657

Structure 62.6H_2O (M06-2X, Gas Phase)

Energy (Hartrees): = - 1599.4505711
No imaginary frequencies

Standard orientation:

Center Number	Atomic Number	Atomic Type	Coordinates (Angstroms) X	Y	Z
1	6	0	4.018157	-0.238358	-1.756859
2	6	0	3.006046	-0.415785	-0.812207
3	6	0	3.335393	-0.578832	0.538197
4	6	0	4.661767	-0.571345	0.935828
5	6	0	5.643487	-0.397700	-0.030700
6	6	0	5.351714	-0.232076	-1.373594
7	6	0	1.603602	-0.401942	-1.273773
8	7	0	0.629197	-0.675173	-0.514334
9	6	0	-0.710151	-0.524276	-1.050279
10	6	0	-1.324091	0.758575	-0.479045
11	6	0	-2.768136	0.924652	-0.945365
12	6	0	-3.540081	-0.359044	-0.648935
13	8	0	-2.886921	-1.466154	-1.260206
14	6	0	-1.603849	-1.741845	-0.754654
15	8	0	-0.470381	1.822879	-0.879342
16	8	0	-1.658737	-2.134684	0.579726
17	6	0	-4.950482	-0.329034	-1.206898
18	8	0	-5.773658	-1.330574	-0.646629
19	8	0	-3.406208	1.972050	-0.236824
20	1	0	1.447456	-0.122417	-2.322915
21	1	0	-0.690119	-0.411158	-2.142884
22	1	0	-0.960812	2.663912	-0.932214
23	1	0	-1.342696	0.694475	0.617853
24	1	0	-3.186881	2.818660	-0.650688
25	1	0	-2.778798	1.118196	-2.027602
26	1	0	-5.239714	-2.132093	-0.476199
27	1	0	-5.400779	0.637299	-0.973987
28	1	0	-4.886156	-0.441514	-2.296743
29	1	0	-3.591126	-0.495378	0.439391
30	1	0	-1.259438	-1.512069	1.214218
31	1	0	-1.257429	-2.596066	-1.339539
32	1	0	3.760300	-0.106342	-2.801607
33	1	0	6.155113	-0.101455	-2.085026
34	1	0	4.949050	-0.697604	1.970574
35	1	0	2.553350	-0.711495	1.275246
36	1	0	-4.654477	-2.612016	1.567124
37	8	0	-4.358894	-3.163467	0.828479

38	1	0	-3.405026	-3.013718	0.788788
39	1	0	-1.804173	4.166488	0.276222
40	8	0	-1.879604	4.188777	-0.705667
41	1	0	-1.764496	5.088682	-1.014104
42	8	0	-1.794611	3.320092	1.772936
43	1	0	-0.961215	2.858060	1.952367
44	1	0	-2.450614	2.636545	1.580375
45	1	0	0.552587	1.952470	0.624158
46	8	0	0.684938	1.964083	1.592359
47	1	0	1.542774	2.360701	1.759607
48	1	0	0.507185	-0.853450	1.389979
49	8	0	0.045649	-0.734166	2.237008
50	1	0	0.158095	0.211586	2.396091
51	8	0	-5.458318	-0.896716	2.041903
52	1	0	-5.853210	-0.938010	1.152878
53	1	0	-6.163774	-0.663955	2.647186
54	7	0	7.062132	-0.385198	0.392693
55	8	0	7.897762	-0.245239	-0.471112
56	8	0	7.289667	-0.513589	1.573949

Structure 62.6H_2O (M06-2X, DMSO)

Energy (Hartrees): = - 1599.4944982
No imaginary frequencies

Standard orientation:

Center Number	Atomic Number	Atomic Type	Coordinates (Angstroms) X	Y	Z
1	6	0	4.048587	-0.321060	-1.745161
2	6	0	3.050049	-0.405450	-0.772787
3	6	0	3.398674	-0.475266	0.580130
4	6	0	4.730166	-0.468749	0.957709
5	6	0	5.698321	-0.391752	-0.035610
6	6	0	5.388114	-0.317350	-1.383782
7	6	0	1.643737	-0.397867	-1.222554
8	7	0	0.666163	-0.572361	-0.434286
9	6	0	-0.668814	-0.471259	-1.000829
10	6	0	-1.349560	0.797687	-0.478180
11	6	0	-2.784646	0.884740	-0.993800
12	6	0	-3.510705	-0.416932	-0.668714
13	8	0	-2.804888	-1.529298	-1.210276
14	6	0	-1.511255	-1.716664	-0.686133
15	8	0	-0.537245	1.890243	-0.880442
16	8	0	-1.573924	-2.066242	0.668008
17	6	0	-4.901172	-0.474728	-1.264788
18	8	0	-5.657746	-1.555681	-0.736180
19	8	0	-3.494670	1.925612	-0.338634
20	1	0	1.491170	-0.223311	-2.292756
21	1	0	-0.629459	-0.393137	-2.094692
22	1	0	-1.064901	2.708704	-0.942700
23	1	0	-1.405473	0.763210	0.619269
24	1	0	-3.281666	2.768433	-0.767385
25	1	0	-2.775812	1.043524	-2.079996
26	1	0	-5.050436	-2.286917	-0.519526
27	1	0	-5.435777	0.447461	-1.033946
28	1	0	-4.808252	-0.567085	-2.353391
29	1	0	-3.583495	-0.511818	0.423661
30	1	0	-1.202350	-1.394431	1.270508
31	1	0	-1.109909	-2.570961	-1.233639
32	1	0	3.774118	-0.259340	-2.792138
33	1	0	6.172036	-0.254758	-2.126093
34	1	0	5.018828	-0.519637	1.998575
35	1	0	2.631588	-0.529547	1.341905
36	1	0	-4.598788	-2.653893	1.558232
37	8	0	-4.179379	-3.255380	0.923387
38	1	0	-3.262297	-2.946699	0.875681
39	1	0	-2.015600	4.258347	0.158270
40	8	0	-2.063268	4.186349	-0.817126
41	1	0	-1.920822	5.055980	-1.204341
42	8	0	-2.053176	3.396030	1.732077
43	1	0	-1.184369	2.991339	1.892205
44	1	0	-2.623711	2.673507	1.435095
45	1	0	0.432782	2.104989	0.688181
46	8	0	0.474777	2.162708	1.661593
47	1	0	1.299853	2.609843	1.880937
48	1	0	0.526488	-0.666756	1.467976
49	8	0	0.037795	-0.552936	2.304577
50	1	0	0.072560	0.408948	2.412003
51	8	0	-5.604306	-1.094400	1.984969
52	1	0	-5.907717	-1.141862	1.061733
53	1	0	-6.397793	-1.043844	2.526403
54	7	0	7.118188	-0.386610	0.363801
55	8	0	7.953393	-0.353730	-0.515061
56	8	0	7.372607	-0.416976	1.549402

Structure 62.6H_2O (M06-2X, H_2O)

Energy (Hartrees): = - 1599.5162114
No imaginary frequencies

Standard orientation:

Center Number	Atomic Number	Atomic Type	Coordinates (Angstroms) X	Y	Z
1	6	0	3.999972	-0.457065	-1.749840
2	6	0	2.976331	-0.467555	-0.800103
3	6	0	3.284981	-0.430960	0.564122
4	6	0	4.606339	-0.405848	0.976412
5	6	0	5.599519	-0.409725	0.005585
6	6	0	5.328583	-0.433375	-1.353488
7	6	0	1.581007	-0.484359	-1.281737
8	7	0	0.598971	-0.773541	-0.530638
9	6	0	-0.727783	-0.608743	-1.102700
10	6	0	-1.304523	0.723823	-0.609888
11	6	0	-2.752415	0.894924	-1.058214
12	6	0	-3.553003	-0.343200	-0.668755
13	8	0	-2.946796	-1.516630	-1.213467
14	6	0	-1.642980	-1.784119	-0.743663
15	8	0	-0.452817	1.740366	-1.114795
16	8	0	-1.649276	-2.127507	0.619690
17	6	0	-4.966645	-0.308724	-1.209328
18	8	0	-5.798792	-1.295886	-0.606418
19	8	0	-3.356962	1.995491	-0.391225
20	1	0	1.440901	-0.229293	-2.337067
21	1	0	-0.690629	-0.563858	-2.198061
22	1	0	-0.849468	2.620632	-0.952864
23	1	0	-1.297756	0.741662	0.488685
24	1	0	-3.026824	2.821892	-0.774883
25	1	0	-2.786131	1.034830	-2.145687
26	1	0	-5.251196	-2.062079	-0.361270
27	1	0	-5.413525	0.663906	-1.003084
28	1	0	-4.925374	-0.456813	-2.294104
29	1	0	-3.581637	-0.414816	0.427048
30	1	0	-1.441596	-1.374714	1.197348
31	1	0	-1.328080	-2.675024	-1.285402
32	1	0	3.752961	-0.470894	-2.805117
33	1	0	6.133608	-0.431488	-2.075192
34	1	0	4.866451	-0.372857	2.025330
35	1	0	2.492447	-0.405849	1.301028
36	1	0	-4.672396	-2.283664	1.655003
37	8	0	-4.329654	-3.015524	1.115907
38	1	0	-3.400964	-2.775364	0.968552
39	1	0	-1.613227	4.129823	0.317644
40	8	0	-1.646183	4.179983	-0.655515
41	1	0	-1.205311	4.995852	-0.913093
42	8	0	-1.763241	3.095321	1.871609
43	1	0	-0.885044	2.682934	1.787142
44	1	0	-2.358473	2.517771	1.371136
45	1	0	0.669493	1.924812	0.426253
46	8	0	0.794917	1.993045	1.388904
47	1	0	1.615214	2.481045	1.517695
48	1	0	0.415479	-0.859815	1.420980
49	8	0	0.043255	-0.572284	2.274664
50	1	0	0.210811	0.383252	2.231381
51	8	0	-5.553126	-0.636430	2.108570
52	1	0	-5.892499	-0.746269	1.203755
53	1	0	-6.310450	-0.798541	2.680529
54	7	0	7.003956	-0.371351	0.440609
55	8	0	7.863056	-0.246422	-0.408229
56	8	0	7.237671	-0.465181	1.628566

Structure 97α (B3LYP, Gas Phase)

Energy (Hartrees): = - 1222.635508
No imaginary frequencies

Standard orientation:

Center Number	Atomic Number	Atomic Type	Coordinates (Angstroms) X	Y	Z
1	6	0	0.761580	1.941173	-0.992425
2	6	0	-0.743744	1.933459	-1.224573
3	6	0	-1.450698	0.923584	-0.320857
4	6	0	-0.767490	-0.448117	-0.398242
5	6	0	0.754530	-0.309259	-0.190829
6	1	0	-1.467134	1.253219	0.720781
7	1	0	-0.997415	-0.917801	-1.359613

8	1	0	0.936163	0.003943	0.844937
9	1	0	-0.920351	1.658395	-2.269755
10	8	0	1.307183	0.654310	-1.092997
11	6	0	1.477259	-1.617845	-0.447244
12	1	0	0.986469	-2.438545	0.081244
13	1	0	1.488215	-1.847970	-1.516836
14	8	0	2.824729	-1.468485	0.032496
15	8	0	-1.229891	-1.295112	0.673109
16	8	0	-2.807578	0.833324	-0.805720
17	6	0	3.623120	-2.552489	-0.125776
18	6	0	-2.263824	-2.148240	0.428392
19	6	0	-3.809621	0.660448	0.095888
20	8	0	3.243081	-3.593374	-0.614110
21	8	0	-2.783847	-2.284258	-0.656079
22	8	0	-3.644515	0.623943	1.295830
23	6	0	-5.126313	0.483738	-0.614694
24	6	0	-2.673542	-2.846895	1.697894
25	6	0	5.016708	-2.269125	0.379369
26	1	0	-5.147853	-0.523351	-1.043979
27	1	0	-5.943711	0.593731	0.097407
28	1	0	-5.228868	1.198015	-1.434503
29	1	0	-3.325423	-3.686422	1.458345
30	1	0	-1.800110	-3.184984	2.259802
31	1	0	-3.215036	-2.127492	2.320915
32	1	0	5.625746	-3.167128	0.283501
33	1	0	5.461871	-1.453680	-0.198352
34	1	0	4.982495	-1.947748	1.424158
35	8	0	0.998571	2.489549	0.322166
36	6	0	2.126662	3.247588	0.489601
37	8	0	2.869878	3.555209	-0.410673
38	1	0	1.296107	2.548191	-1.722979
39	6	0	2.284092	3.637910	1.937243
40	1	0	2.465949	2.743521	2.541116
41	1	0	3.122272	4.326220	2.038352
42	1	0	1.365424	4.100464	2.307947
43	1	0	-1.158014	2.932986	-1.067170

Structure 97α (B3LYP, $CHCl_3$)

Energy (Hartrees): = - 1222.6581481
No imaginary frequencies

Standard orientation:

Center Number	Atomic Number	Atomic Type	Coordinates (Angstroms) X	Y	Z
1	6	0	0.635666	1.947957	-1.044994
2	6	0	-0.868582	1.862049	-1.274215
3	6	0	-1.510662	0.850266	-0.327271
4	6	0	-0.773018	-0.487746	-0.393114
5	6	0	0.734860	-0.278550	-0.157762
6	1	0	-1.514739	1.213686	0.702216
7	1	0	-0.950953	-0.965478	-1.360024
8	1	0	0.889869	0.072123	0.870415
9	1	0	-1.029757	1.543337	-2.309299
10	8	0	1.250033	0.686457	-1.085867
11	6	0	1.517909	-1.559024	-0.373025
12	1	0	1.080646	-2.376772	0.205075
13	1	0	1.519012	-1.836771	-1.431148
14	8	0	2.868597	-1.321667	0.069870
15	8	0	-1.262845	-1.341897	0.660099
16	8	0	-2.876133	0.645256	-0.751501
17	6	0	3.718390	-2.367473	-0.040389
18	6	0	-2.070319	-2.388357	0.329419
19	6	0	-3.864623	0.713582	0.178288
20	8	0	3.380839	-3.455181	-0.462910
21	8	0	-2.368560	-2.678093	-0.808953
22	8	0	-3.679760	0.972928	1.348980
23	6	0	-5.197928	0.422241	-0.455527
24	6	0	-2.528000	-3.104536	1.570411
25	6	0	5.101365	-1.991209	0.419167
26	1	0	-5.190131	-0.586203	-0.880858
27	1	0	-5.987169	0.504105	0.291700
28	1	0	-5.385555	1.121904	-1.275553
29	1	0	-3.127261	-3.972139	1.294916
30	1	0	-1.666318	-3.418708	2.166698
31	1	0	-3.122225	-2.422975	2.187560
32	1	0	5.757189	-2.859788	0.361295
33	1	0	5.495464	-1.188063	-0.211600
34	1	0	5.069836	-1.615440	1.446312
35	8	0	0.840974	2.572354	0.241972
36	6	0	2.011151	3.250702	0.428208
37	8	0	2.839107	3.406474	-0.441650
38	1	0	1.134912	2.543718	-1.808975
39	6	0	2.103636	3.768883	1.836590

40	1	0	2.102559	2.930875	2.541110
41	1	0	3.018978	4.348326	1.955758
42	1	0	1.233644	4.391023	2.067193
43	1	0	-1.325084	2.847466	-1.147808

Structure 97α (M06-2X, Gas Phase)

Energy (Hartrees): = - 1222.4741199
No imaginary frequencies

Standard orientation:

Center Number	Atomic Number	Atomic Type	Coordinates (Angstroms) X	Y	Z
1	6	0	0.767163	1.941009	-1.020317
2	6	0	-0.728480	1.941421	-1.270854
3	6	0	-1.437428	0.966424	-0.342132
4	6	0	-0.774749	-0.407716	-0.413670
5	6	0	0.736399	-0.276297	-0.196693
6	1	0	-1.421981	1.311550	0.694081
7	1	0	-0.999770	-0.874093	-1.377766
8	1	0	0.910177	0.049877	0.835140
9	1	0	-0.901360	1.634080	-2.305124
10	8	0	1.301074	0.655355	-1.106115
11	6	0	1.442370	-1.590450	-0.425025
12	1	0	0.940446	-2.399080	0.108759
13	1	0	1.465906	-1.825831	-1.491460
14	8	0	2.773426	-1.438299	0.066400
15	8	0	-1.233284	-1.245148	0.651004
16	8	0	-2.791156	0.899847	-0.803919
17	6	0	3.591599	-2.485043	-0.151682
18	6	0	-2.336509	-1.999149	0.443804
19	6	0	-3.760429	0.618263	0.091042
20	8	0	3.232502	-3.493381	-0.690983
21	8	0	-2.901441	-2.065288	-0.612280
22	8	0	-3.561745	0.494484	1.268824
23	6	0	-5.079401	0.439246	-0.598400
24	6	0	-2.756958	-2.677490	1.711915
25	6	0	4.973856	-2.201947	0.365964
26	1	0	-5.057179	-0.543783	-1.075000
27	1	0	-5.880899	0.477313	0.134628
28	1	0	-5.215661	1.192738	-1.372445
29	1	0	-3.468186	-3.466521	1.482534
30	1	0	-1.892770	-3.069003	2.246344
31	1	0	-3.232222	-1.915985	2.334957
32	1	0	5.610947	-3.061864	0.179045
33	1	0	5.370968	-1.316451	-0.131583
34	1	0	4.926736	-1.988615	1.434447
35	8	0	0.977190	2.477367	0.287290
36	6	0	2.200803	3.023843	0.530598
37	8	0	3.036311	3.164684	-0.310507
38	1	0	1.316729	2.538683	-1.744083
39	6	0	2.322617	3.412752	1.975875
40	1	0	2.311717	2.509719	2.588716
41	1	0	3.253461	3.952690	2.125609
42	1	0	1.468899	4.023638	2.269157
43	1	0	-1.134096	2.944767	-1.136214

Structure 97α (M06-2X, $CHCl_3$)

Energy (Hartrees): = - 1222.4741199
No imaginary frequencies

Standard orientation:

Center Number	Atomic Number	Atomic Type	Coordinates (Angstroms) X	Y	Z
1	6	0	0.767163	1.941009	-1.020317
2	6	0	-0.728480	1.941421	-1.270854
3	6	0	-1.437428	0.966424	-0.342132
4	6	0	-0.774749	-0.407716	-0.413670
5	6	0	0.736399	-0.276297	-0.196693
6	1	0	-1.421981	1.311550	0.694081
7	1	0	-0.999770	-0.874093	-1.377766
8	1	0	0.910177	0.049877	0.835140
9	1	0	-0.901360	1.634080	-2.305124
10	8	0	1.301074	0.655355	-1.106115
11	6	0	1.442370	-1.590450	-0.425025
12	1	0	0.940446	-2.399080	0.108759
13	1	0	1.465906	-1.825831	-1.491460
14	8	0	2.773426	-1.438299	0.066400
15	8	0	-1.233284	-1.245148	0.651004
16	8	0	-2.791156	0.899847	-0.803919

17	6	0	3.591599	-2.485043	-0.151682
18	6	0	-2.336509	-1.999149	0.443804
19	6	0	-3.760429	0.618263	0.091042
20	8	0	3.232502	-3.493381	-0.690983
21	8	0	-2.901441	-2.065288	-0.612280
22	8	0	-3.561745	0.494484	1.268824
23	6	0	-5.079401	0.439246	-0.598400
24	6	0	-2.756958	-2.677490	1.711915
25	6	0	4.973856	-2.201947	0.365964
26	1	0	-5.057179	-0.543783	-1.075000
27	1	0	-5.880899	0.477313	0.134628
28	1	0	-5.215661	1.192738	-1.372445
29	1	0	-3.468186	-3.466521	1.482534
30	1	0	-1.892770	-3.069003	2.246344
31	1	0	-3.232222	-1.915985	2.334957
32	1	0	5.610947	-3.061864	0.179045
33	1	0	5.370968	-1.316451	-0.131583
34	1	0	4.926736	-1.988615	1.434447
35	8	0	0.977190	2.477367	0.287290
36	6	0	2.200803	3.023843	0.530598
37	8	0	3.036311	3.164684	-0.310507
38	1	0	1.316729	2.538683	-1.744083
39	6	0	2.322617	3.412752	1.975875
40	1	0	2.311717	2.509719	2.588716
41	1	0	3.253461	3.952690	2.125609
42	1	0	1.468899	4.023638	2.269157
43	1	0	-1.134096	2.944767	-1.136214

Structure 97β (B3LYP, Gas Phase)

Energy (Hartrees): = - 1222.633159
No imaginary frequencies

Standard orientation:

Center Number	Atomic Number	Atomic Type	Coordinates (Angstroms) X	Y	Z
1	6	0	0.880757	-1.471963	-0.195017
2	6	0	-0.511667	-1.872643	0.274553
3	6	0	-1.533711	-0.835509	-0.204832
4	6	0	-1.103147	0.590023	0.140485
5	6	0	0.343159	0.820701	-0.334351
6	1	0	0.988772	-1.520660	-1.287588
7	1	0	-1.672866	-0.917063	-1.287341
8	1	0	-1.187074	0.768555	1.215184
9	1	0	0.374942	0.736145	-1.433061
10	1	0	-0.505523	-1.906768	1.368601
11	8	0	1.183160	-0.165984	0.253654
12	6	0	0.918241	2.175039	0.057174
13	1	0	0.279953	2.980421	-0.311505
14	1	0	1.037646	2.250245	1.140113
15	8	0	2.192048	2.314253	-0.587677
16	8	0	-1.974026	1.498625	-0.562535
17	8	0	-2.809663	-1.057264	0.431215
18	8	0	1.822600	-2.339499	0.410255
19	6	0	3.303844	2.123325	0.181732
20	6	0	-2.736168	2.355690	0.178019
21	6	0	3.083274	-2.337418	-0.126720
22	6	0	-3.657177	-1.928107	-0.179212
23	8	0	3.387949	-1.717919	-1.117980
24	8	0	3.292154	2.074102	1.389111
25	8	0	-2.676057	2.449924	1.380978
26	8	0	-3.389530	-2.522340	-1.199558
27	6	0	4.000565	-3.203103	0.696347
28	6	0	-4.944627	-2.045829	0.597880
29	6	0	-3.643059	3.154277	-0.724758
30	6	0	4.518416	1.960821	-0.694928
31	1	0	4.954951	-3.312234	0.182699
32	1	0	4.159554	-2.733545	1.672197
33	1	0	3.547119	-4.181769	0.873229
34	1	0	-5.381824	-1.057221	0.761230
35	1	0	-5.639901	-2.682700	0.052438
36	1	0	-4.742466	-2.477591	1.582820
37	1	0	-4.191365	3.885597	-0.132370
38	1	0	-3.058833	3.657501	-1.500045
39	1	0	-4.344553	2.483910	-1.230399
40	1	0	5.420642	2.113942	-0.103033
41	1	0	4.513652	0.939653	-1.092442
42	1	0	4.489205	2.650763	-1.541034
43	1	0	-0.783305	-2.860197	-0.107622

Structure 97β (B3LYP, $CHCl_3$)

Energy (Hartrees): = - 1222.6559716
No imaginary frequencies

Standard orientation:

Center Number	Atomic Number	Atomic Type	Coordinates (Angstroms) X	Y	Z
1	6	0	0.886618	-1.501995	-0.187601
2	6	0	-0.501138	-1.880966	0.310434
3	6	0	-1.520567	-0.852741	-0.188124
4	6	0	-1.078850	0.574772	0.129195
5	6	0	0.367142	0.798871	-0.354856
6	1	0	0.974908	-1.579297	-1.278572
7	1	0	-1.669290	-0.953678	-1.266692
8	1	0	-1.154704	0.768680	1.201531
9	1	0	0.393857	0.715368	-1.452612
10	1	0	-0.486732	-1.886256	1.405197
11	8	0	1.214810	-0.187764	0.229388
12	6	0	0.921747	2.156383	0.050238
13	1	0	0.283432	2.956369	-0.329960
14	1	0	1.013450	2.233408	1.135129
15	8	0	2.211793	2.325713	-0.564958
16	8	0	-1.948318	1.477534	-0.584792
17	8	0	-2.790618	-1.056270	0.469665
18	8	0	1.828161	-2.366438	0.427378
19	6	0	3.311092	2.219690	0.225337
20	6	0	-2.714612	2.345130	0.132902
21	6	0	3.051740	-2.481235	-0.173414
22	6	0	-3.700545	-1.851295	-0.148801
23	8	0	3.335640	-1.940049	-1.218494
24	8	0	3.275024	2.147406	1.435282
25	8	0	-2.668520	2.448900	1.339286
26	8	0	-3.505131	-2.390413	-1.218600
27	6	0	3.959957	-3.349847	0.649530
28	6	0	-4.948928	-1.976234	0.681571
29	6	0	-3.608130	3.134538	-0.784143
30	6	0	4.559137	2.193950	-0.614630
31	1	0	4.860534	-3.583288	0.081655
32	1	0	4.235737	-2.813790	1.564169
33	1	0	3.448729	-4.268972	0.947702
34	1	0	-5.303023	-0.990623	0.995511
35	1	0	-5.720685	-2.490870	0.109236
36	1	0	-4.726116	-2.548982	1.588255
37	1	0	-4.131737	3.902739	-0.215436
38	1	0	-3.022165	3.592895	-1.585718
39	1	0	-4.336264	2.464051	-1.252209
40	1	0	5.433856	2.344354	0.018327
41	1	0	4.631208	1.217035	-1.105575
42	1	0	4.519265	2.956908	-1.396314
43	1	0	-0.774974	-2.879113	-0.042716

Structure 97β (M06-2X, Gas Phase)

Energy (Hartrees): = - 1222.4701114
No imaginary frequencies

Standard orientation:

Center Number	Atomic Number	Atomic Type	Coordinates (Angstroms) X	Y	Z
1	6	0	0.797152	-1.470560	-0.235479
2	6	0	-0.577773	-1.877565	0.253636
3	6	0	-1.595288	-0.838775	-0.209000
4	6	0	-1.164195	0.572134	0.154551
5	6	0	0.268254	0.807823	-0.331304
6	1	0	0.880315	-1.494944	-1.329981
7	1	0	-1.734131	-0.901043	-1.292633
8	1	0	-1.227089	0.725884	1.234317
9	1	0	0.287568	0.738166	-1.430051
10	1	0	-0.550489	-1.904929	1.345936
11	8	0	1.104370	-0.182874	0.228185
12	6	0	0.866441	2.141657	0.074128
13	1	0	0.270207	2.971961	-0.300312
14	1	0	0.960351	2.205636	1.158697
15	8	0	2.144713	2.232760	-0.548556
16	8	0	-2.064832	1.456845	-0.516543
17	8	0	-2.860642	-1.053491	0.423654
18	8	0	1.749782	-2.335355	0.331899
19	6	0	3.222318	1.857114	0.186886
20	6	0	-2.439151	2.579749	0.137676
21	6	0	3.017462	-2.201917	-0.142564
22	6	0	-3.650895	-2.002614	-0.121624
23	8	0	3.297436	-1.509673	-1.077583
24	8	0	3.192503	1.672438	1.368003

25	8	0	-1.986691	2.909466	1.195971
26	8	0	-3.327636	-2.664256	-1.067335
27	6	0	3.973757	-3.019853	0.672617
28	6	0	-4.955474	-2.098811	0.616991
29	6	0	-3.477133	3.327606	-0.649102
30	6	0	4.424647	1.699643	-0.697584
31	1	0	4.930145	-3.075614	0.159862
32	1	0	4.100692	-2.531124	1.640721
33	1	0	3.564659	-4.014169	0.848523
34	1	0	-5.470706	-1.138710	0.569658
35	1	0	-5.565064	-2.878650	0.169272
36	1	0	-4.763386	-2.319786	1.667501
37	1	0	-3.691278	4.271322	-0.155267
38	1	0	-3.119356	3.495950	-1.665083
39	1	0	-4.381983	2.721183	-0.711883
40	1	0	5.324014	1.703747	-0.086721
41	1	0	4.330332	0.737346	-1.207034
42	1	0	4.456002	2.488326	-1.447872
43	1	0	-0.853561	-2.861208	-0.128181

Structure 97β (M06-2X, $CHCl_3$)

Energy (Hartrees): = - 1222.494061
No imaginary frequencies

Standard orientation:

Center Number	Atomic Number	Atomic Type	Coordinates (Angstroms) X	Y	Z
1	6	0	0.816371	-1.468659	-0.225735
2	6	0	-0.554818	-1.876338	0.273004
3	6	0	-1.580704	-0.854846	-0.207387
4	6	0	-1.157798	0.561187	0.143294
5	6	0	0.268097	0.803120	-0.359698
6	1	0	0.894263	-1.514894	-1.318641
7	1	0	-1.717632	-0.929413	-1.289854
8	1	0	-1.208851	0.717258	1.223070
9	1	0	0.280713	0.709971	-1.455819
10	1	0	-0.531195	-1.885880	1.366064
11	8	0	1.121579	-0.168367	0.214526
12	6	0	0.841304	2.154689	0.018582
13	1	0	0.251021	2.964584	-0.407824
14	1	0	0.904105	2.263538	1.101259
15	8	0	2.140344	2.240982	-0.571150
16	8	0	-2.070644	1.438949	-0.521465
17	8	0	-2.844551	-1.069049	0.431408
18	8	0	1.773223	-2.321327	0.357829
19	6	0	3.202247	1.919763	0.200126
20	6	0	-2.454813	2.558206	0.129895
21	6	0	3.032275	-2.239285	-0.144001
22	6	0	-3.651319	-2.003350	-0.109011
23	8	0	3.313540	-1.562843	-1.093940
24	8	0	3.147527	1.788123	1.391615
25	8	0	-2.005064	2.886154	1.193873
26	8	0	-3.339742	-2.666156	-1.062376
27	6	0	3.977166	-3.084042	0.650492
28	6	0	-4.952055	-2.082874	0.630281
29	6	0	-3.493747	3.300418	-0.652761
30	6	0	4.431003	1.752007	-0.641167
31	1	0	4.931749	-3.148780	0.134141
32	1	0	4.116511	-2.619390	1.629390
33	1	0	3.553607	-4.076564	0.806450
34	1	0	-5.462037	-1.119922	0.564474
35	1	0	-5.570338	-2.864926	0.196942
36	1	0	-4.761923	-2.289224	1.684660
37	1	0	-3.714006	4.244889	-0.161593
38	1	0	-3.138265	3.471459	-1.669779
39	1	0	-4.397483	2.690262	-0.712142
40	1	0	5.313535	1.748079	-0.005496
41	1	0	4.348614	0.794176	-1.161708
42	1	0	4.495159	2.543001	-1.388289
43	1	0	-0.816499	-2.869932	-0.093337

Structure 98α (B3LYP, Gas Phase)

Energy (Hartrees): = - 1321.8601674
No imaginary frequencies

Standard orientation:

Center Number	Atomic Number	Atomic Type	Coordinates (Angstroms) X	Y	Z
1	6	0	0.632615	1.879409	-0.884701

2	6	0	-0.861138	1.683194	-1.143718
3	6	0	-1.443335	0.577181	-0.273022
4	6	0	-0.587196	-0.697241	-0.354419
5	6	0	0.907441	-0.377698	-0.163339
6	1	0	-1.509221	0.910111	0.765580
7	1	0	-0.767100	-1.193691	-1.313119
8	1	0	1.071511	-0.064227	0.874221
9	1	0	-0.998878	1.430048	-2.201237
10	8	0	1.313309	0.669599	-1.055745
11	6	0	1.779708	-1.580190	-0.466331
12	1	0	1.398690	-2.467794	0.044784
13	1	0	1.800240	-1.780083	-1.541963
14	8	0	3.106097	-1.282398	0.000368
15	8	0	-0.942105	-1.589720	0.718264
16	8	0	-2.765599	0.321120	-0.777629
17	6	0	4.022234	-2.265427	-0.186063
18	6	0	-1.862050	-2.567740	0.471190
19	6	0	-3.754191	0.047157	0.116825
20	8	0	3.756731	-3.333328	-0.690799
21	8	0	-2.351131	-2.770917	-0.616400
22	8	0	-3.590180	0.019753	1.316287
23	6	0	-5.040568	-0.251158	-0.606486
24	6	0	-2.190396	-3.304463	1.742028
25	6	0	5.379655	-1.833653	0.311467
26	1	0	-4.943696	-1.228636	-1.090044
27	1	0	-5.863322	-0.275143	0.107256
28	1	0	-5.228426	0.490035	-1.386563
29	1	0	-2.732812	-4.218822	1.503734
30	1	0	-1.285388	-3.529619	2.310512
31	1	0	-2.819839	-2.653652	2.357789
32	1	0	6.084778	-2.656080	0.197841
33	1	0	5.724967	-0.966184	-0.258778
34	1	0	5.319800	-1.531533	1.360796
35	8	0	0.783353	2.371416	0.452241
36	6	0	1.572338	3.478839	0.637644
37	8	0	2.128086	4.062896	-0.259554
38	1	0	1.073871	2.595470	-1.577975
39	6	0	1.620530	3.841587	2.098432
40	1	0	2.019798	3.004496	2.678390
41	1	0	2.247420	4.722067	2.232539
42	1	0	0.609278	4.040582	2.464651
43	9	0	-1.529340	2.871833	-0.891303

Structure 98α (B3LYP, $CHCl_3$)

Energy (Hartrees): = - 1321.8829363
No imaginary frequencies

Standard orientation:

Center Number	Atomic Number	Atomic Type	Coordinates (Angstroms) X	Y	Z
1	6	0	0.302694	1.889090	-0.936159
2	6	0	-1.153229	1.468075	-1.148935
3	6	0	-1.535633	0.321861	-0.223902
4	6	0	-0.528749	-0.821992	-0.357446
5	6	0	0.902615	-0.294954	-0.148300
6	1	0	-1.575153	0.674061	0.809019
7	1	0	-0.624313	-1.296037	-1.337450
8	1	0	1.009208	0.059561	0.883782
9	1	0	-1.279341	1.169133	-2.194835
10	8	0	1.155018	0.785850	-1.064611
11	6	0	1.943584	-1.361145	-0.423537
12	1	0	1.711329	-2.272583	0.132546
13	1	0	1.980622	-1.600386	-1.490807
14	8	0	3.217307	-0.842824	0.005620
15	8	0	-0.809884	-1.790892	0.668082
16	8	0	-2.834709	-0.159239	-0.607733
17	6	0	4.256728	-1.707243	-0.051075
18	6	0	-1.276195	-3.017056	0.287893
19	6	0	-3.853907	-0.044840	0.290714
20	8	0	4.143047	-2.857129	-0.424702
21	8	0	-1.443320	-3.344152	-0.865910
22	8	0	-3.721041	0.406677	1.406917
23	6	0	-5.136289	-0.550401	-0.309252
24	6	0	-1.527978	-3.875892	1.495512
25	6	0	5.533304	-1.048874	0.398123
26	1	0	-5.001908	-1.567662	-0.688196
27	1	0	-5.925305	-0.530792	0.442286
28	1	0	-5.419447	0.081248	-1.157380
29	1	0	-1.925388	-4.841186	1.182715
30	1	0	-0.594544	-4.021489	2.048328
31	1	0	-2.233324	-3.379723	2.168721
32	1	0	6.341301	-1.780299	0.399516
33	1	0	5.783140	-0.224843	-0.277831

34	1	0	5.410776	-0.625770	1.399542
35	8	0	0.397646	2.468651	0.370886
36	6	0	1.157083	3.599918	0.512484
37	8	0	1.723409	4.140262	-0.409918
38	1	0	0.621452	2.619933	-1.678798
39	6	0	1.166145	4.048703	1.945277
40	1	0	1.620071	3.274600	2.572119
41	1	0	1.731318	4.976039	2.036194
42	1	0	0.140752	4.196616	2.296785
43	9	0	-1.988007	2.555604	-0.907077

Structure 98α (M06-2X, Gas Phase)

Energy (Hartrees): = - 1321.7066166
No imaginary frequencies

Standard orientation:

Center Number	Atomic Number	Atomic Type	Coordinates (Angstroms) X	Y	Z
1	6	0	0.701977	1.864726	-0.896206
2	6	0	-0.792096	1.748557	-1.157040
3	6	0	-1.424884	0.693160	-0.272043
4	6	0	-0.646430	-0.620991	-0.366530
5	6	0	0.851818	-0.378781	-0.172383
6	1	0	-1.445517	1.035010	0.765594
7	1	0	-0.847390	-1.094906	-1.332600
8	1	0	1.028005	-0.067755	0.862803
9	1	0	-0.948251	1.491168	-2.208410
10	8	0	1.312081	0.625242	-1.069191
11	6	0	1.649933	-1.625953	-0.461039
12	1	0	1.217445	-2.487163	0.051913
13	1	0	1.668560	-1.823679	-1.535515
14	8	0	2.974751	-1.393312	0.012797
15	8	0	-1.034059	-1.497795	0.691757
16	8	0	-2.756169	0.528464	-0.757205
17	6	0	3.853571	-2.386603	-0.226250
18	6	0	-2.078921	-2.334230	0.479426
19	6	0	-3.719200	0.186707	0.126733
20	8	0	3.549821	-3.406660	-0.776425
21	8	0	-2.631457	-2.440803	-0.578986
22	8	0	-3.522181	0.063701	1.303921
23	6	0	-5.015610	-0.061443	-0.581646
24	6	0	-2.452041	-3.042372	1.745321
25	6	0	5.220228	-2.028678	0.285184
26	1	0	-4.925328	-1.030215	-1.079463
27	1	0	-5.826071	-0.090440	0.141732
28	1	0	-5.189650	0.700041	-1.340281
29	1	0	-3.078645	-3.898612	1.509645
30	1	0	-1.563900	-3.343087	2.298698
31	1	0	-3.012197	-2.327514	2.353345
32	1	0	5.907278	-2.843614	0.075128
33	1	0	5.557894	-1.110347	-0.196489
34	1	0	5.170400	-1.841283	1.358428
35	8	0	0.858877	2.332758	0.433116
36	6	0	1.920045	3.155868	0.679579
37	8	0	2.677206	3.518575	-0.167770
38	1	0	1.183515	2.554922	-1.585884
39	6	0	1.972772	3.513323	2.135295
40	1	0	2.194865	2.614970	2.714042
41	1	0	2.747272	4.258761	2.293411
42	1	0	1.001532	3.887363	2.458726
43	9	0	-1.390860	2.963920	-0.916815

Structure 98α (M06-2X, $CHCl_3$)

Energy (Hartrees): = - 1321.7306998
No imaginary frequencies

Standard orientation:

Center Number	Atomic Number	Atomic Type	Coordinates (Angstroms) X	Y	Z
1	6	0	0.670760	1.889531	-0.908028
2	6	0	-0.824549	1.763797	-1.155843
3	6	0	-1.445954	0.711031	-0.259985
4	6	0	-0.669854	-0.601336	-0.375610
5	6	0	0.826650	-0.355380	-0.173130
6	1	0	-1.446644	1.051068	0.778461
7	1	0	-0.859904	-1.057442	-1.351466
8	1	0	1.000143	-0.042270	0.862390
9	1	0	-0.986647	1.508284	-2.205898
10	8	0	1.284822	0.650527	-1.072464

11	6	0	1.630971	-1.598935	-0.459965
12	1	0	1.221902	-2.454431	0.080969
13	1	0	1.627963	-1.814617	-1.531213
14	8	0	2.965132	-1.338069	-0.019566
15	8	0	-1.059757	-1.497703	0.665853
16	8	0	-2.788628	0.552767	-0.720265
17	6	0	3.851928	-2.327143	-0.221774
18	6	0	-2.078345	-2.356103	0.430744
19	6	0	-3.740732	0.221668	0.177817
20	8	0	3.550031	-3.378246	-0.721405
21	8	0	-2.642342	-2.431939	-0.626915
22	8	0	-3.519452	0.078775	1.350444
23	6	0	-5.061444	0.032907	-0.498126
24	6	0	-2.395727	-3.147128	1.659604
25	6	0	5.219038	-1.933918	0.249416
26	1	0	-5.011818	-0.894063	-1.075003
27	1	0	-5.847721	-0.040448	0.249339
28	1	0	-5.256350	0.853417	-1.188456
29	1	0	-3.095736	-3.941359	1.412430
30	1	0	-1.482917	-3.557087	2.092443
31	1	0	-2.843785	-2.468505	2.389490
32	1	0	5.915518	-2.749572	0.072451
33	1	0	5.540387	-1.038570	-0.285562
34	1	0	5.183546	-1.691921	1.313053
35	8	0	0.832439	2.378142	0.414191
36	6	0	2.001464	3.021022	0.690598
37	8	0	2.852146	3.198556	-0.132662
38	1	0	1.137750	2.572101	-1.615204
39	6	0	2.041536	3.447437	2.123317
40	1	0	2.016016	2.561051	2.760552
41	1	0	2.949699	4.014796	2.310307
42	1	0	1.160058	4.048436	2.351403
43	9	0	-1.430116	2.981551	-0.910265

Structure 98β (B3LYP, Gas Phase)

Energy (Hartrees): = - 1321.8588973
No imaginary frequencies

Standard orientation:

Center Number	Atomic Number	Atomic Type	Coordinates (Angstroms) X	Y	Z
1	6	0	-1.110129	-1.326058	0.232902
2	6	0	0.249603	-1.855723	-0.210366
3	6	0	1.366263	-0.879999	0.165557
4	6	0	1.030264	0.580060	-0.191018
5	6	0	-0.400668	0.916656	0.257635
6	1	0	-1.199938	-1.305696	1.327419
7	1	0	1.561715	-0.945497	1.240538
8	1	0	1.153088	0.735336	-1.266545
9	1	0	-0.451343	0.870372	1.356930
10	1	0	0.231503	-2.014167	-1.293850
11	8	0	-1.291500	-0.039013	-0.310106
12	6	0	-0.880611	2.290956	-0.186194
13	1	0	-0.187045	3.059667	0.159613
14	1	0	-0.989008	2.334051	-1.272453
15	8	0	-2.144895	2.541510	0.441587
16	8	0	1.906432	1.467127	0.528877
17	8	0	2.533792	-1.317348	-0.550202
18	8	0	-2.105475	-2.160688	-0.316228
19	6	0	-3.263094	2.395050	-0.329673
20	6	0	3.039452	1.904914	-0.093565
21	6	0	-3.354959	-2.062198	0.243101
22	6	0	3.735688	-1.268140	0.087822
23	8	0	-3.605876	-1.356036	1.189791
24	8	0	-3.244927	2.286152	-1.532961
25	8	0	3.317208	1.646636	-1.242337
26	8	0	3.877627	-0.891354	1.229795
27	6	0	-4.325279	-2.943756	-0.495995
28	6	0	4.836424	-1.702516	-0.842617
29	6	0	3.884217	2.701927	0.863825
30	6	0	-4.492081	2.365345	0.541029
31	1	0	-5.279942	-2.952028	0.028358
32	1	0	-4.464614	-2.557998	-1.510704
33	1	0	-3.927522	-3.958219	-0.582411
34	1	0	4.998229	-0.906704	-1.577065
35	1	0	5.751116	-1.865914	-0.273756
36	1	0	4.552956	-2.606438	-1.386191
37	1	0	4.636998	3.260824	0.308702
38	1	0	3.269865	3.373626	1.467154
39	1	0	4.377702	1.998506	1.542673
40	1	0	-5.377153	2.545766	-0.068678
41	1	0	-4.561110	1.371207	0.996801
42	1	0	-4.421281	3.099229	1.346743

43	9	0	0.499573	-3.072325	0.410705

Structure 98β (B3LYP, $CHCl_3$)

Energy (Hartrees): = - 1321.8813263
No imaginary frequencies

Standard orientation:

Center Number	Atomic Number	Atomic Type	Coordinates (Angstroms) X	Y	Z
1	6	0	0.988372	-1.398707	-0.150923
2	6	0	-0.393913	-1.801341	0.356049
3	6	0	-1.439129	-0.802547	-0.130015
4	6	0	-1.026565	0.640611	0.169949
5	6	0	0.405965	0.889051	-0.334901
6	1	0	1.053777	-1.485027	-1.242595
7	1	0	-1.586171	-0.928977	-1.206138
8	1	0	-1.089023	0.840236	1.242244
9	1	0	0.431374	0.780733	-1.429542
10	1	0	-0.380757	-1.854731	1.449394
11	8	0	1.266893	-0.079415	0.270482
12	6	0	0.940398	2.261400	0.042125
13	1	0	0.278684	3.040448	-0.342025
14	1	0	1.044385	2.358537	1.124360
15	8	0	2.217025	2.447472	-0.593920
16	8	0	-1.936416	1.506289	-0.532353
17	8	0	-2.677758	-1.079154	0.544013
18	8	0	1.939169	-2.242798	0.461946
19	6	0	3.328828	2.398422	0.185949
20	6	0	-2.717570	2.355791	0.196854
21	6	0	3.154220	-2.362936	-0.163975
22	6	0	-3.750544	-1.455909	-0.208847
23	8	0	3.415825	-1.815336	-1.210103
24	8	0	3.306978	2.345659	1.397029
25	8	0	-2.650721	2.463887	1.401245
26	8	0	-3.727807	-1.547050	-1.416455
27	6	0	4.065191	-3.250947	0.632320
28	6	0	-4.932126	-1.740348	0.675674
29	6	0	-3.641778	3.119048	-0.710230
30	6	0	4.567477	2.409355	-0.667626
31	1	0	5.012559	-3.370859	0.107309
32	1	0	4.239874	-2.808197	1.618042
33	1	0	3.595687	-4.226763	0.788737
34	1	0	-5.125273	-0.890794	1.336855
35	1	0	-5.808747	-1.947082	0.062083
36	1	0	-4.714006	-2.606849	1.308427
37	1	0	-4.275127	3.779356	-0.118249
38	1	0	-3.060165	3.707012	-1.427061
39	1	0	-4.259572	2.422063	-1.284433
40	1	0	5.444405	2.579620	-0.042942
41	1	0	4.660550	1.438515	-1.166822
42	1	0	4.499251	3.176271	-1.443691
43	9	0	-0.720019	-3.062842	-0.140382

Structure 98β (M06-2X, Gas Phase)

Energy (Hartrees): = - 1321.7065553
No imaginary frequencies

Standard orientation:

Center Number	Atomic Number	Atomic Type	Coordinates (Angstroms) X	Y	Z
1	6	0	1.123065	-1.285588	-0.279190
2	6	0	-0.213769	-1.842735	0.170878
3	6	0	-1.340285	-0.884508	-0.189831
4	6	0	-1.027254	0.564142	0.202128
5	6	0	0.383185	0.925166	-0.256128
6	1	0	1.191579	-1.238441	-1.373710
7	1	0	-1.522672	-0.926607	-1.268400
8	1	0	-1.134342	0.688223	1.283438
9	1	0	0.426188	0.898662	-1.354972
10	1	0	-0.183798	-2.015573	1.249654
11	8	0	1.286634	-0.017139	0.284059
12	6	0	0.847138	2.289621	0.209584
13	1	0	0.166743	3.061266	-0.146973
14	1	0	0.925752	2.312965	1.297920
15	8	0	2.117089	2.535691	-0.382433
16	8	0	-1.906274	1.456908	-0.482541
17	8	0	-2.489697	-1.363762	0.504031
18	8	0	2.136481	-2.104268	0.233447

19	6	0	3.214055	2.223540	0.357508
20	6	0	-3.087846	1.766999	0.104629
21	6	0	3.386707	-1.853279	-0.246627
22	6	0	-3.697678	-1.205308	-0.081682
23	8	0	3.601836	-1.061421	-1.116698
24	8	0	3.177924	1.981141	1.527739
25	8	0	-3.387769	1.406887	1.207752
26	8	0	-3.844087	-0.733888	-1.175399
27	6	0	4.401419	-2.688552	0.472697
28	6	0	-4.790559	-1.645261	0.842720
29	6	0	-3.947094	2.560778	-0.830403
30	6	0	4.439636	2.221118	-0.507778
31	1	0	5.367503	-2.580631	-0.012509
32	1	0	4.461418	-2.344708	1.506894
33	1	0	4.084215	-3.731206	0.483835
34	1	0	-4.899318	-0.866258	1.601447
35	1	0	-5.717581	-1.750469	0.285500
36	1	0	-4.521100	-2.574996	1.341139
37	1	0	-4.750999	3.033742	-0.272560
38	1	0	-3.355059	3.297221	-1.371422
39	1	0	-4.362011	1.853584	-1.553052
40	1	0	5.325235	2.246601	0.122112
41	1	0	4.428424	1.297412	-1.091657
42	1	0	4.423100	3.065245	-1.195775
43	9	0	-0.448300	-3.044890	-0.460176

Structure 98β (M06-2X, $CHCl_3$)

Energy (Hartrees): = - 1321.7295638
No imaginary frequencies

Standard orientation:

Center Number	Atomic Number	Atomic Type	Coordinates (Angstroms) X	Y	Z
1	6	0	1.123986	-1.290634	-0.272019
2	6	0	-0.210523	-1.835893	0.200151
3	6	0	-1.337896	-0.891220	-0.186211
4	6	0	-1.026073	0.560358	0.188907
5	6	0	0.381957	0.920426	-0.281358
6	1	0	1.181019	-1.263710	-1.366919
7	1	0	-1.507480	-0.952027	-1.265285
8	1	0	-1.121668	0.697219	1.269282
9	1	0	0.423753	0.876986	-1.378755
10	1	0	-0.179400	-1.987224	1.281776
11	8	0	1.293616	-0.011792	0.270338
12	6	0	0.827318	2.293339	0.173947
13	1	0	0.153891	3.057080	-0.211770
14	1	0	0.874776	2.340962	1.262679
15	8	0	2.113342	2.543100	-0.392178
16	8	0	-1.911832	1.446386	-0.497614
17	8	0	-2.493462	-1.362901	0.507107
18	8	0	2.136432	-2.106806	0.249825
19	6	0	3.197562	2.266196	0.367587
20	6	0	-3.074031	1.790201	0.102294
21	6	0	3.384217	-1.899652	-0.255720
22	6	0	-3.697705	-1.226449	-0.088696
23	8	0	3.601201	-1.120189	-1.139626
24	8	0	3.145179	2.032471	1.543080
25	8	0	-3.377963	1.420907	1.204069
26	8	0	-3.838982	-0.763511	-1.188994
27	6	0	4.386819	-2.756898	0.446595
28	6	0	-4.791818	-1.693759	0.817084
29	6	0	-3.903364	2.646428	-0.800992
30	6	0	4.442672	2.292822	-0.465112
31	1	0	5.359428	-2.642447	-0.025172
32	1	0	4.438448	-2.448839	1.493058
33	1	0	4.065663	-3.799030	0.419462
34	1	0	-4.885900	-0.968023	1.628721
35	1	0	-5.725773	-1.751158	0.263384
36	1	0	-4.537925	-2.660305	1.252261
37	1	0	-4.725354	3.082302	-0.238447
38	1	0	-3.291592	3.422945	-1.260052
39	1	0	-4.296992	2.008486	-1.596198
40	1	0	5.316350	2.311197	0.182285
41	1	0	4.456526	1.385523	-1.074787
42	1	0	4.437367	3.153478	-1.133796
43	9	0	-0.443791	-3.059593	-0.403171

Structure 99α (B3LYP, Gas Phase)

Energy (Hartrees): = - 1297.846108
No imaginary frequencies

Standard orientation:

Center Number	Atomic Number	Atomic Type	X	Y	Z
1	6	0	0.653373	1.892270	-0.878617
2	6	0	-0.838759	1.713180	-1.154759
3	6	0	-1.425742	0.621469	-0.255234
4	6	0	-0.609904	-0.673048	-0.361731
5	6	0	0.891665	-0.376950	-0.167800
6	1	0	-1.459222	0.958577	0.783190
7	1	0	-0.800741	-1.148565	-1.328800
8	1	0	1.055484	-0.080362	0.875232
9	1	0	-0.917430	1.373362	-2.199185
10	8	0	1.321729	0.671175	-1.043859
11	6	0	1.744436	-1.591433	-0.480728
12	1	0	1.347493	-2.478865	0.018105
13	1	0	1.766593	-1.777863	-1.558745
14	8	0	3.074204	-1.322289	-0.005496
15	8	0	-0.965908	-1.586215	0.695654
16	8	0	-2.777464	0.428216	-0.734181
17	6	0	3.973054	-2.319298	-0.196489
18	6	0	-1.914991	-2.530685	0.443839
19	6	0	-3.743480	0.085531	0.165331
20	8	0	3.691675	-3.378515	-0.711452
21	8	0	-2.448434	-2.678231	-0.633050
22	8	0	-3.553559	-0.004337	1.356913
23	6	0	-5.040401	-0.201253	-0.544453
24	6	0	-2.217722	-3.313081	1.693789
25	6	0	5.335788	-1.916028	0.310769
26	1	0	-4.957008	-1.190421	-1.006748
27	1	0	-5.858448	-0.204501	0.175322
28	1	0	-5.227645	0.523897	-1.339302
29	1	0	-2.783062	-4.208106	1.435924
30	1	0	-1.300386	-3.575788	2.225056
31	1	0	-2.817681	-2.677984	2.353759
32	1	0	6.027682	-2.748626	0.189885
33	1	0	5.697223	-1.047435	-0.247584
34	1	0	5.276978	-1.625517	1.363453
35	8	0	0.807880	2.382911	0.458619
36	6	0	1.684801	3.416670	0.657973
37	8	0	2.321755	3.938448	-0.224018
38	1	0	1.110174	2.598485	-1.571274
39	6	0	1.715037	3.795470	2.115998
40	1	0	2.006174	2.932465	2.721709
41	1	0	2.420937	4.611648	2.264088
42	1	0	0.715371	4.099978	2.439039
43	8	0	-1.487469	2.953148	-0.971346
44	1	0	-2.420692	2.814248	-1.182740

Structure 99α (B3LYP, $CHCl_3$)

Energy (Hartrees): = - 1297.8699547
No imaginary frequencies

Standard orientation:

Center Number	Atomic Number	Atomic Type	X	Y	Z
1	6	0	0.556190	1.895090	-0.913213
2	6	0	-0.933942	1.671305	-1.176435
3	6	0	-1.473146	0.579361	-0.248355
4	6	0	-0.627013	-0.690363	-0.360957
5	6	0	0.860948	-0.356117	-0.141712
6	1	0	-1.491032	0.932611	0.784566
7	1	0	-0.781222	-1.156073	-1.337865
8	1	0	1.005059	-0.035992	0.897276
9	1	0	-1.011927	1.316125	-2.214186
10	8	0	1.266976	0.692581	-1.035716
11	6	0	1.753483	-1.547509	-0.427856
12	1	0	1.395533	-2.430944	0.106679
13	1	0	1.771948	-1.767358	-1.499430
14	8	0	3.079397	-1.211620	0.024701
15	8	0	-1.014203	-1.616458	0.673238
16	8	0	-2.825778	0.305065	-0.674372
17	6	0	4.018804	-2.171698	-0.131036
18	6	0	-1.762005	-2.702392	0.328734
19	6	0	-3.789924	0.152943	0.276542
20	8	0	3.778707	-3.264439	-0.603799
21	8	0	-2.114975	-2.944963	-0.805214
22	8	0	-3.587512	0.261754	1.466374
23	6	0	-5.108680	-0.181052	-0.365360
24	6	0	-2.075155	-3.526758	1.546450
25	6	0	5.363821	-1.693234	0.345423
26	1	0	-5.040328	-1.167733	-0.835161

27	1	0	-5.894339	-0.187574	0.390002
28	1	0	-5.346981	0.540508	-1.151780
29	1	0	-2.626568	-4.420235	1.254169
30	1	0	-1.151524	-3.807657	2.061014
31	1	0	-2.673029	-2.933282	2.245364
32	1	0	6.088561	-2.504416	0.276579
33	1	0	5.694722	-0.850561	-0.270151
34	1	0	5.297107	-1.338048	1.378022
35	8	0	0.696858	2.440912	0.406492
36	6	0	1.664023	3.385907	0.604582
37	8	0	2.384863	3.799260	-0.275712
38	1	0	0.988768	2.586171	-1.635950
39	6	0	1.687143	3.818942	2.043366
40	1	0	1.943081	2.966579	2.681055
41	1	0	2.421900	4.612620	2.177358
42	1	0	0.695858	4.169090	2.346097
43	8	0	-1.610705	2.900145	-1.004460
44	1	0	-2.521900	2.771728	-1.306945

Structure 99α (M06-2X, Gas Phase)

Energy (Hartrees): = - 1297.6884821
No imaginary frequencies

Standard orientation:

Center Number	Atomic Number	Atomic Type	Coordinates (Angstroms) X	Y	Z
1	6	0	0.692740	1.870899	-0.892920
2	6	0	-0.790591	1.723634	-1.188609
3	6	0	-1.402443	0.675285	-0.269630
4	6	0	-0.629199	-0.635940	-0.375834
5	6	0	0.867785	-0.375134	-0.167760
6	1	0	-1.401202	1.029072	0.763683
7	1	0	-0.821100	-1.099764	-1.348236
8	1	0	1.025900	-0.075775	0.873947
9	1	0	-0.872919	1.366652	-2.224000
10	8	0	1.334041	0.641485	-1.043953
11	6	0	1.680934	-1.612058	-0.460403
12	1	0	1.255486	-2.482649	0.041726
13	1	0	1.709266	-1.799038	-1.536475
14	8	0	3.000724	-1.370430	0.023488
15	8	0	-1.002880	-1.536224	0.669974
16	8	0	-2.754937	0.533075	-0.725746
17	6	0	3.889661	-2.353136	-0.216637
18	6	0	-2.047283	-2.366957	0.452042
19	6	0	-3.692638	0.135779	0.166662
20	8	0	3.598493	-3.374784	-0.771605
21	8	0	-2.626495	-2.435006	-0.596468
22	8	0	-3.467695	-0.019024	1.334002
23	6	0	-4.999024	-0.123567	-0.519963
24	6	0	-2.387698	-3.125261	1.698199
25	6	0	5.251820	-1.982318	0.298374
26	1	0	-4.902444	-1.087558	-1.025521
27	1	0	-5.794736	-0.171392	0.218690
28	1	0	-5.203388	0.637798	-1.271064
29	1	0	-3.042982	-3.955823	1.449768
30	1	0	-1.484930	-3.471554	2.198997
31	1	0	-2.904413	-2.428068	2.362146
32	1	0	5.944435	-2.795694	0.100923
33	1	0	5.586552	-1.068154	-0.193205
34	1	0	5.195436	-1.781230	1.368710
35	8	0	0.819422	2.346364	0.438282
36	6	0	1.839363	3.210849	0.697393
37	8	0	2.612772	3.584800	-0.131214
38	1	0	1.174748	2.565688	-1.576612
39	6	0	1.823984	3.610043	2.144103
40	1	0	1.942307	2.722388	2.766949
41	1	0	2.629783	4.314080	2.331697
42	1	0	0.858914	4.058075	2.382882
43	8	0	-1.405606	2.974128	-1.029521
44	1	0	-2.341912	2.855152	-1.213005

Structure 99α (M06-2X, $CHCl_3$)

Energy (Hartrees): = - 1297.7137443
No imaginary frequencies

Standard orientation:

Center Number	Atomic Number	Atomic Type	Coordinates (Angstroms) X	Y	Z
1	6	0	0.667302	1.894424	-0.897582

2	6	0	-0.825281	1.770151	-1.155815
3	6	0	-1.433103	0.709226	-0.246395
4	6	0	-0.668709	-0.604775	-0.377708
5	6	0	0.828299	-0.355997	-0.171203
6	1	0	-1.422376	1.048018	0.792138
7	1	0	-0.858687	-1.050846	-1.358096
8	1	0	0.995636	-0.054104	0.869021
9	1	0	-0.935348	1.439916	-2.196697
10	8	0	1.292626	0.657582	-1.055930
11	6	0	1.635693	-1.596193	-0.465624
12	1	0	1.226806	-2.458042	0.065060
13	1	0	1.638120	-1.800976	-1.539020
14	8	0	2.968305	-1.338302	-0.017199
15	8	0	-1.049543	-1.519941	0.653546
16	8	0	-2.789405	0.573259	-0.692137
17	6	0	3.858392	-2.321256	-0.229635
18	6	0	-2.073314	-2.367562	0.415107
19	6	0	-3.731488	0.206874	0.203984
20	8	0	3.562866	-3.366318	-0.746015
21	8	0	-2.654012	-2.418833	-0.635838
22	8	0	-3.500161	0.032214	1.369983
23	6	0	-5.057374	0.024708	-0.464793
24	6	0	-2.378766	-3.184205	1.630548
25	6	0	5.222713	-1.930757	0.252602
26	1	0	-5.011579	-0.900556	-1.044878
27	1	0	-5.839027	-0.052388	0.287210
28	1	0	-5.258460	0.846633	-1.151570
29	1	0	-3.074007	-3.979107	1.372172
30	1	0	-1.461463	-3.594279	2.053398
31	1	0	-2.830053	-2.521866	2.373389
32	1	0	5.920827	-2.744473	0.073007
33	1	0	5.546239	-1.031260	-0.274126
34	1	0	5.181409	-1.696493	1.317701
35	8	0	0.835047	2.382390	0.426469
36	6	0	1.997847	3.029978	0.698658
37	8	0	2.848832	3.215330	-0.124106
38	1	0	1.136091	2.576968	-1.603386
39	6	0	2.039835	3.457847	2.132006
40	1	0	2.015438	2.572475	2.770624
41	1	0	2.947570	4.026419	2.317682
42	1	0	1.158112	4.058576	2.359983
43	8	0	-1.418274	3.027183	-0.953097
44	1	0	-2.337778	2.964097	-1.233921

Structure 99β (B3LYP, Gas Phase)

Energy (Hartrees): = - 1297.8445462
No imaginary frequencies

Standard orientation:

Center Number	Atomic Number	Atomic Type	Coordinates (Angstroms) X	Y	Z
1	6	0	-1.123482	-1.330416	0.232771
2	6	0	0.229811	-1.868794	-0.222518
3	6	0	1.343789	-0.889601	0.187792
4	6	0	1.034210	0.564415	-0.200916
5	6	0	-0.391865	0.912752	0.259289
6	1	0	-1.213560	-1.323115	1.327132
7	1	0	1.505292	-0.944566	1.268751
8	1	0	1.146509	0.698008	-1.280413
9	1	0	-0.428282	0.871167	1.359578
10	1	0	0.199620	-1.928725	-1.320275
11	8	0	-1.298077	-0.033154	-0.293917
12	6	0	-0.865621	2.290322	-0.182818
13	1	0	-0.166633	3.055977	0.158480
14	1	0	-0.980520	2.333281	-1.268427
15	8	0	-2.125399	2.547048	0.451865
16	8	0	1.915211	1.470685	0.492569
17	8	0	2.528129	-1.375485	-0.484281
18	8	0	-2.134453	-2.140915	-0.326822
19	6	0	-3.248264	2.405059	-0.312809
20	6	0	3.053675	1.875348	-0.136045
21	6	0	-3.378524	-2.034045	0.235251
22	6	0	3.732266	-1.246281	0.143475
23	8	0	-3.623441	-1.336716	1.191021
24	8	0	-3.238458	2.306377	-1.517226
25	8	0	3.348090	1.556010	-1.266301
26	8	0	3.864316	-0.814217	1.265960
27	6	0	-4.360991	-2.897079	-0.511294
28	6	0	4.847931	-1.671972	-0.773760
29	6	0	3.886798	2.724603	0.786495
30	6	0	-4.471376	2.365899	0.565725
31	1	0	-5.318218	-2.890484	0.008466
32	1	0	-4.488526	-2.507895	-1.526175

33	1	0	-3.979745	-3.918003	-0.596830
34	1	0	5.017561	-0.862230	-1.491293
35	1	0	5.755197	-1.838060	-0.193569
36	1	0	4.579499	-2.567445	-1.338199
37	1	0	4.652194	3.245330	0.211841
38	1	0	3.265870	3.434961	1.336517
39	1	0	4.364231	2.061864	1.515723
40	1	0	-5.360576	2.554411	-0.035545
41	1	0	-4.537356	1.365897	1.009072
42	1	0	-4.394448	3.089520	1.380122
43	8	0	0.435643	-3.137275	0.364788
44	1	0	1.279264	-3.470030	0.029975

Structure 99β (B3LYP, $CHCl_3$)

Energy (Hartrees): = - 1297.8683097
No imaginary frequencies

Standard orientation:

Center Number	Atomic Number	Atomic Type	Coordinates (Angstroms) X	Y	Z
1	6	0	1.080325	-1.374757	-0.171728
2	6	0	-0.282661	-1.855002	0.325031
3	6	0	-1.363554	-0.874469	-0.154935
4	6	0	-1.019759	0.578804	0.182177
5	6	0	0.403084	0.892091	-0.314594
6	1	0	1.157018	-1.442217	-1.263956
7	1	0	-1.498797	-0.977548	-1.234997
8	1	0	-1.099035	0.749363	1.258439
9	1	0	0.429361	0.810338	-1.411810
10	1	0	-0.255431	-1.851769	1.423756
11	8	0	1.304855	-0.049315	0.266694
12	6	0	0.879484	2.277784	0.090918
13	1	0	0.184321	3.035383	-0.276165
14	1	0	0.980433	2.354675	1.175174
15	8	0	2.147277	2.532374	-0.539646
16	8	0	-1.932509	1.454084	-0.509418
17	8	0	-2.589459	-1.254235	0.505930
18	8	0	2.075931	-2.176754	0.431945
19	6	0	3.260916	2.503100	0.237706
20	6	0	-2.921910	2.060389	0.204800
21	6	0	3.284961	-2.249330	-0.204570
22	6	0	-3.733982	-1.328397	-0.230498
23	8	0	3.521835	-1.688095	-1.250460
24	8	0	3.243178	2.423140	1.447521
25	8	0	-3.070411	1.924590	1.400117
26	8	0	-3.782344	-1.130635	-1.425011
27	6	0	4.235001	-3.111598	0.576046
28	6	0	-4.901050	-1.669229	0.654662
29	6	0	-3.777632	2.902372	-0.700837
30	6	0	4.498024	2.572573	-0.615599
31	1	0	5.180367	-3.194926	0.040345
32	1	0	4.405201	-2.671414	1.563677
33	1	0	3.800838	-4.104316	0.728612
34	1	0	-5.082295	-0.840050	1.346500
35	1	0	-5.788277	-1.839075	0.044945
36	1	0	-4.681160	-2.555617	1.256272
37	1	0	-4.562099	3.386281	-0.119433
38	1	0	-3.164232	3.658206	-1.200684
39	1	0	-4.221845	2.273268	-1.478285
40	1	0	5.368499	2.766034	0.011464
41	1	0	4.625849	1.611781	-1.126680
42	1	0	4.402307	3.345811	-1.382327
43	8	0	-0.509422	-3.154552	-0.187067
44	1	0	-1.299968	-3.505012	0.248833

Structure 99β (M06-2X, Gas Phase)

Energy (Hartrees): = - 1297.6880758
No imaginary frequencies

Standard orientation:

Center Number	Atomic Number	Atomic Type	Coordinates (Angstroms) X	Y	Z
1	6	0	1.128256	-1.291157	-0.273426
2	6	0	-0.201454	-1.845707	0.199425
3	6	0	-1.320988	-0.883543	-0.201249
4	6	0	-1.027376	0.558920	0.212488
5	6	0	0.378954	0.924605	-0.260570
6	1	0	1.189284	-1.265566	-1.368910

7	1	0	-1.469899	-0.922594	-1.284798
8	1	0	-1.123095	0.669108	1.296251
9	1	0	0.404713	0.901031	-1.360255
10	1	0	-0.156336	-1.909323	1.294215
11	8	0	1.297187	-0.008947	0.263557
12	6	0	0.840321	2.291576	0.202375
13	1	0	0.157866	3.061929	-0.152739
14	1	0	0.922260	2.315797	1.290484
15	8	0	2.108553	2.539319	-0.392625
16	8	0	-1.908894	1.467855	-0.451367
17	8	0	-2.489301	-1.398600	0.449633
18	8	0	2.155243	-2.088710	0.248196
19	6	0	3.207006	2.229732	0.345620
20	6	0	-3.091206	1.755414	0.138441
21	6	0	3.395463	-1.843611	-0.250516
22	6	0	-3.693529	-1.190573	-0.135100
23	8	0	3.602290	-1.070512	-1.140420
24	8	0	3.175634	2.000848	1.518843
25	8	0	-3.401573	1.349031	1.223653
26	8	0	-3.823389	-0.692446	-1.217849
27	6	0	4.424048	-2.655581	0.477656
28	6	0	-4.803003	-1.617392	0.776367
29	6	0	-3.945030	2.588982	-0.767163
30	6	0	4.428593	2.211054	-0.525064
31	1	0	5.368833	-2.604266	-0.056541
32	1	0	4.542065	-2.239973	1.480344
33	1	0	4.084867	-3.685947	0.577957
34	1	0	-4.913090	-0.828670	1.525097
35	1	0	-5.723786	-1.717230	0.207691
36	1	0	-4.552837	-2.544210	1.290019
37	1	0	-4.748535	3.042987	-0.193185
38	1	0	-3.349101	3.343487	-1.278013
39	1	0	-4.361308	1.912264	-1.517667
40	1	0	5.317345	2.241412	0.100223
41	1	0	4.410222	1.279329	-1.096112
42	1	0	4.412020	3.045752	-1.224477
43	8	0	-0.401884	-3.104231	-0.389030
44	1	0	-1.242757	-3.440499	-0.066000

Structure 99β (M06-2X, $CHCl_3$)

Energy (Hartrees): = - 1297.7124874
No imaginary frequencies

Standard orientation:

Center Number	Atomic Number	Atomic Type	Coordinates (Angstroms) X	Y	Z
1	6	0	1.123102	-1.295008	-0.266799
2	6	0	-0.207225	-1.842347	0.216154
3	6	0	-1.324917	-0.882442	-0.194808
4	6	0	-1.024892	0.562231	0.201260
5	6	0	0.379458	0.922684	-0.282512
6	1	0	1.180486	-1.283893	-1.362034
7	1	0	-1.470747	-0.936095	-1.277481
8	1	0	-1.109541	0.686891	1.283951
9	1	0	0.405270	0.882080	-1.380877
10	1	0	-0.162913	-1.898881	1.310831
11	8	0	1.300985	-0.005593	0.253942
12	6	0	0.829114	2.295847	0.169120
13	1	0	0.156029	3.061185	-0.213733
14	1	0	0.882644	2.344231	1.257556
15	8	0	2.113392	2.542258	-0.403282
16	8	0	-1.911608	1.465134	-0.465806
17	8	0	-2.496876	-1.378472	0.463944
18	8	0	2.148076	-2.093134	0.264229
19	6	0	3.199982	2.261785	0.350858
20	6	0	-3.074606	1.788768	0.138810
21	6	0	3.386909	-1.892774	-0.255184
22	6	0	-3.695488	-1.209634	-0.136684
23	8	0	3.598694	-1.134320	-1.159787
24	8	0	3.154698	2.038061	1.528768
25	8	0	-3.381812	1.389198	1.229851
26	8	0	-3.824034	-0.711895	-1.222845
27	6	0	4.402716	-2.723450	0.462204
28	6	0	-4.803510	-1.691878	0.745022
29	6	0	-3.906241	2.666174	-0.742432
30	6	0	4.439730	2.270670	-0.490331
31	1	0	5.346476	-2.690996	-0.076377
32	1	0	4.535621	-2.314737	1.466666
33	1	0	4.046639	-3.749177	0.560038
34	1	0	-4.909709	-0.976720	1.564632
35	1	0	-5.729228	-1.739928	0.176685
36	1	0	-4.558999	-2.664190	1.172548
37	1	0	-4.726993	3.088872	-0.168025
38	1	0	-3.296108	3.452963	-1.185762

39	1	0	-4.302017	2.046219	-1.550690
40	1	0	5.317959	2.293563	0.150833
41	1	0	4.444732	1.354471	-1.086796
42	1	0	4.434435	3.121418	-1.171518
43	8	0	-0.405637	-3.107406	-0.362859
44	1	0	-1.195846	-3.491690	0.032231

Structure 100α (B3LYP, Gas Phase)

Energy (Hartrees): = - 1450.513823
No imaginary frequencies

Standard orientation:

Center Number	Atomic Number	Atomic Type	Coordinates (Angstroms) X	Y	Z
1	6	0	0.462325	-1.654456	-0.501201
2	6	0	1.384763	-0.449740	-0.727491
3	6	0	0.877782	0.790260	-0.000168
4	6	0	-0.612172	1.027281	-0.294062
5	6	0	-1.432357	-0.262824	-0.106978
6	1	0	1.026050	0.698630	1.078383
7	1	0	-0.722550	1.424874	-1.307574
8	1	0	-1.452420	-0.518185	0.958668
9	1	0	1.432107	-0.264354	-1.803570
10	8	0	-0.848825	-1.341946	-0.851195
11	6	0	-2.854026	-0.098281	-0.607643
12	1	0	-3.293888	0.821972	-0.216016
13	1	0	-2.874519	-0.056481	-1.701100
14	8	0	-3.609636	-1.231825	-0.148855
15	8	0	-1.146245	1.973248	0.652009
16	8	0	1.657623	1.894196	-0.494159
17	6	0	-4.932171	-1.209987	-0.449428
18	6	0	-1.187367	3.291549	0.300481
19	6	0	2.030573	2.865457	0.381779
20	8	0	-5.463259	-0.300688	-1.046754
21	8	0	-0.862649	3.707975	-0.787819
22	8	0	1.762911	2.850599	1.563091
23	6	0	2.772666	3.959202	-0.340115
24	6	0	-1.649887	4.124896	1.465792
25	6	0	-5.616340	-2.456385	0.055953
26	1	0	2.047782	4.537690	-0.922177
27	1	0	3.260416	4.609736	0.385214
28	1	0	3.502169	3.541759	-1.037543
29	1	0	-1.903772	5.126377	1.119954
30	1	0	-2.503270	3.661548	1.965791
31	1	0	-0.828885	4.182428	2.187968
32	1	0	-6.682871	-2.394993	-0.156711
33	1	0	-5.189880	-3.337809	-0.431998
34	1	0	-5.453019	-2.567763	1.131563
35	8	0	0.500333	-2.033434	0.891300
36	6	0	1.280318	-3.092624	1.245736
37	8	0	1.971589	-3.711612	0.469047
38	1	0	0.776228	-2.501773	-1.110553
39	6	0	1.156025	-3.364753	2.723049
40	1	0	0.108785	-3.535762	2.987219
41	1	0	1.756161	-4.235880	2.982685
42	1	0	1.498880	-2.493729	3.289449
43	6	0	3.559619	-1.333327	-1.121603
44	8	0	3.286894	-1.532766	-2.281701
45	6	0	4.841110	-1.716105	-0.432310
46	1	0	4.655484	-2.628052	0.144680
47	1	0	5.610008	-1.914218	-1.178456
48	1	0	5.164251	-0.936228	0.260521
49	8	0	2.698963	-0.741342	-0.237108

Structure 100α (B3LYP, $CHCl_3$)

Energy (Hartrees): = - 1450.5382843
No imaginary frequencies

Standard orientation:

Center Number	Atomic Number	Atomic Type	Coordinates (Angstroms) X	Y	Z
1	6	0	0.476962	-1.595657	-0.541950
2	6	0	1.367377	-0.359567	-0.748302
3	6	0	0.830628	0.833057	0.034860
4	6	0	-0.650649	1.052668	-0.281635
5	6	0	-1.444568	-0.246603	-0.060114
6	1	0	0.964798	0.679339	1.107580
7	1	0	-0.767627	1.404432	-1.309864

8	1	0	-1.423652	-0.508953	1.004034
9	1	0	1.392202	-0.135457	-1.817499
10	8	0	-0.854841	-1.306644	-0.834634
11	6	0	-2.883289	-0.113681	-0.517057
12	1	0	-3.343109	0.772921	-0.073791
13	1	0	-2.937201	-0.030884	-1.606784
14	8	0	-3.585932	-1.294078	-0.082725
15	8	0	-1.158389	2.049250	0.623809
16	8	0	1.563927	2.003397	-0.366246
17	6	0	-4.918757	-1.307289	-0.315328
18	6	0	-1.499998	3.271239	0.118605
19	6	0	2.232977	2.706442	0.589693
20	8	0	-5.509522	-0.384962	-0.839815
21	8	0	-1.439197	3.553876	-1.057274
22	8	0	2.236603	2.413975	1.765686
23	6	0	2.950885	3.873267	-0.030627
24	6	0	-1.956617	4.181123	1.224673
25	6	0	-5.536560	-2.597912	0.151003
26	1	0	2.242200	4.501382	-0.578699
27	1	0	3.445356	4.456170	0.746256
28	1	0	3.691627	3.511346	-0.750653
29	1	0	-2.226474	5.153010	0.812261
30	1	0	-2.818353	3.742513	1.737168
31	1	0	-1.159037	4.296284	1.965014
32	1	0	-6.615062	-2.564846	-0.002728
33	1	0	-5.110027	-3.436082	-0.409143
34	1	0	-5.314774	-2.764416	1.209348
35	8	0	0.572589	-2.027013	0.831008
36	6	0	1.374946	-3.089495	1.118374
37	8	0	2.035086	-3.674128	0.285936
38	1	0	0.783425	-2.410761	-1.197281
39	6	0	1.315984	-3.414344	2.584391
40	1	0	0.288849	-3.662318	2.869191
41	1	0	1.974531	-4.255149	2.801058
42	1	0	1.618992	-2.541788	3.171339
43	6	0	3.580585	-1.147482	-1.181022
44	8	0	3.306482	-1.338985	-2.345514
45	6	0	4.890667	-1.462125	-0.517386
46	1	0	4.769612	-2.379242	0.069388
47	1	0	5.657473	-1.622719	-1.275362
48	1	0	5.187168	-0.662976	0.166006
49	8	0	2.697648	-0.625143	-0.280969

Structure 100α (M06-2X, Gas Phase)

Energy (Hartrees): = - 1450.3347314
No imaginary frequencies

Standard orientation:

Center Number	Atomic Number	Atomic Type	Coordinates (Angstroms) X	Y	Z
1	6	0	0.492647	-1.639855	-0.486295
2	6	0	1.369807	-0.420467	-0.765556
3	6	0	0.849468	0.800050	-0.032638
4	6	0	-0.639895	0.994263	-0.313909
5	6	0	-1.414481	-0.306614	-0.085553
6	1	0	1.006566	0.701621	1.044034
7	1	0	-0.773571	1.362019	-1.336063
8	1	0	-1.410006	-0.538791	0.984191
9	1	0	1.371056	-0.242481	-1.843557
10	8	0	-0.826150	-1.377423	-0.815403
11	6	0	-2.838511	-0.176857	-0.565865
12	1	0	-3.288166	0.742952	-0.187480
13	1	0	-2.869457	-0.165519	-1.658265
14	8	0	-3.556224	-1.306414	-0.072720
15	8	0	-1.192789	1.930686	0.613033
16	8	0	1.599282	1.906497	-0.531874
17	6	0	-4.856215	-1.357118	-0.422742
18	6	0	-1.171814	3.245729	0.287858
19	6	0	1.844151	2.939540	0.301438
20	8	0	-5.388029	-0.508803	-1.080950
21	8	0	-0.798455	3.655811	-0.774893
22	8	0	1.511340	2.954356	1.454893
23	6	0	2.524086	4.055610	-0.431592
24	6	0	-1.627092	4.079759	1.445918
25	6	0	-5.509019	-2.599720	0.113970
26	1	0	1.759367	4.551719	-1.034637
27	1	0	2.944266	4.757770	0.283769
28	1	0	3.289226	3.666516	-1.101369
29	1	0	-1.871316	5.080238	1.098852
30	1	0	-2.476234	3.616898	1.946227
31	1	0	-0.792946	4.123200	2.150560
32	1	0	-6.567971	-2.582802	-0.128742
33	1	0	-5.033730	-3.474989	-0.331070

34	1	0	-5.363148	-2.655536	1.192982
35	8	0	0.560571	-1.949784	0.911340
36	6	0	1.459394	-2.874251	1.316027
37	8	0	2.219928	-3.429397	0.574553
38	1	0	0.821602	-2.498226	-1.068438
39	6	0	1.363879	-3.088278	2.798643
40	1	0	0.334920	-3.324582	3.070348
41	1	0	2.032476	-3.893608	3.089707
42	1	0	1.641172	-2.165336	3.310576
43	6	0	3.468465	-1.422086	-1.126251
44	8	0	3.099685	-1.797250	-2.200467
45	6	0	4.784589	-1.727420	-0.479787
46	1	0	4.611636	-2.528891	0.241776
47	1	0	5.486589	-2.066140	-1.237243
48	1	0	5.165126	-0.855529	0.050032
49	8	0	2.694064	-0.656306	-0.309783

Structure 100α (M06-2X, $CHCl_3$)

Energy (Hartrees): = - 1450.3600094
No imaginary frequencies

Standard orientation:

Center Number	Atomic Number	Atomic Type	Coordinates (Angstroms) X	Y	Z
1	6	0	0.485977	-1.635424	-0.481261
2	6	0	1.358645	-0.413767	-0.766797
3	6	0	0.842436	0.804258	-0.025298
4	6	0	-0.645349	1.001629	-0.304700
5	6	0	-1.420959	-0.295455	-0.058484
6	1	0	1.001883	0.695938	1.050025
7	1	0	-0.786172	1.350838	-1.331810
8	1	0	-1.410152	-0.522886	1.012411
9	1	0	1.351933	-0.231533	-1.843977
10	8	0	-0.838938	-1.372624	-0.788506
11	6	0	-2.845899	-0.163648	-0.535491
12	1	0	-3.299309	0.745365	-0.136025
13	1	0	-2.876653	-0.132032	-1.627369
14	8	0	-3.557075	-1.309878	-0.063679
15	8	0	-1.191561	1.952473	0.612508
16	8	0	1.597722	1.912630	-0.516648
17	6	0	-4.853153	-1.373880	-0.413333
18	6	0	-1.199145	3.258776	0.262191
19	6	0	1.862334	2.933813	0.324002
20	8	0	-5.394585	-0.517016	-1.060188
21	8	0	-0.812078	3.656192	-0.803246
22	8	0	1.513723	2.949884	1.474665
23	6	0	2.605568	4.021696	-0.384711
24	6	0	-1.723184	4.101734	1.381006
25	6	0	-5.493175	-2.630018	0.095045
26	1	0	1.915717	4.498264	-1.085451
27	1	0	2.964310	4.751422	0.337040
28	1	0	3.433383	3.602356	-0.956584
29	1	0	-1.863931	5.122100	1.033200
30	1	0	-2.658692	3.690124	1.760950
31	1	0	-0.989171	4.081580	2.190000
32	1	0	-6.556854	-2.614348	-0.129518
33	1	0	-5.022556	-3.489588	-0.386448
34	1	0	-5.331192	-2.719663	1.170041
35	8	0	0.576440	-1.957719	0.910675
36	6	0	1.470688	-2.897082	1.292343
37	8	0	2.212111	-3.448438	0.524619
38	1	0	0.800222	-2.489969	-1.076659
39	6	0	1.398519	-3.132306	2.768457
40	1	0	0.374670	-3.382302	3.050311
41	1	0	2.075773	-3.937383	3.041750
42	1	0	1.676154	-2.214442	3.290732
43	6	0	3.472282	-1.403919	-1.120549
44	8	0	3.105712	-1.802440	-2.190980
45	6	0	4.797967	-1.665382	-0.480733
46	1	0	4.645630	-2.399489	0.314154
47	1	0	5.486969	-2.067121	-1.219892
48	1	0	5.193832	-0.752367	-0.036328
49	8	0	2.687639	-0.644441	-0.315555

Structure 100β (B3LYP, Gas Phase)

Energy (Hartrees): = - 1450.5113429
No imaginary frequencies

Standard orientation:

Center Number	Atomic Number	Atomic Type	Coordinates (Angstroms) X	Y	Z
1	6	0	-1.040418	0.997647	-0.297847
2	6	0	0.391880	1.306561	0.135735
3	6	0	1.329514	0.145930	-0.219237
4	6	0	0.753061	-1.226928	0.175384
5	6	0	-0.714347	-1.330362	-0.267468
6	1	0	-1.142373	0.964615	-1.391012
7	1	0	1.534664	0.143228	-1.294248
8	1	0	0.851504	-1.374440	1.254452
9	1	0	-0.757628	-1.303030	-1.367805
10	1	0	0.405119	1.495650	1.211413
11	8	0	-1.434025	-0.227852	0.273985
12	6	0	-1.413890	-2.595661	0.208395
13	1	0	-0.857557	-3.476666	-0.117125
14	1	0	-1.525276	-2.593605	1.295175
15	8	0	-2.703982	-2.648980	-0.414964
16	8	0	1.464154	-2.266892	-0.523814
17	8	0	2.555589	0.397260	0.488709
18	8	0	-1.881870	1.999408	0.232065
19	6	0	-3.782574	-2.318523	0.355317
20	6	0	2.517186	-2.866694	0.103022
21	6	0	-3.139358	2.085639	-0.309777
22	6	0	3.733609	0.133022	-0.139390
23	8	0	-3.509644	1.415432	-1.243627
24	8	0	-3.744670	-2.199764	1.557217
25	8	0	2.847782	-2.625677	1.241511
26	8	0	3.816380	-0.272634	-1.277719
27	6	0	-3.954208	3.111343	0.431439
28	6	0	4.886503	0.377443	0.797461
29	6	0	3.208841	-3.818565	-0.836025
30	6	0	-4.993747	-2.104437	-0.514631
31	1	0	-4.895461	3.275106	-0.091791
32	1	0	-4.153861	2.748268	1.444510
33	1	0	-3.398254	4.047721	0.524333
34	1	0	4.913235	-0.441549	1.523741
35	1	0	5.818621	0.395137	0.233495
36	1	0	4.749180	1.308659	1.351302
37	1	0	3.864026	-4.479252	-0.269016
38	1	0	2.486087	-4.395321	-1.417021
39	1	0	3.806039	-3.225671	-1.536746
40	1	0	-5.894621	-2.135623	0.097834
41	1	0	-4.905320	-1.117134	-0.981684
42	1	0	-5.042292	-2.849202	-1.312008
43	6	0	1.194094	3.567667	0.181257
44	8	0	1.144343	3.611153	1.386335
45	6	0	1.637178	4.687218	-0.726655
46	1	0	0.826863	4.956156	-1.410449
47	1	0	1.919626	5.550377	-0.125382
48	1	0	2.483725	4.361437	-1.337792
49	8	0	0.850192	2.473213	-0.562954

Structure 100β (B3LYP, $CHCl_3$)

Energy (Hartrees): = - 1450.5364819
No imaginary frequencies

Standard orientation:

Center Number	Atomic Number	Atomic Type	Coordinates (Angstroms) X	Y	Z
1	6	0	-0.902856	1.119730	-0.282222
2	6	0	0.549084	1.237824	0.183910
3	6	0	1.334763	-0.003732	-0.242284
4	6	0	0.612992	-1.296071	0.157539
5	6	0	-0.848281	-1.245958	-0.319314
6	1	0	-0.981703	1.152077	-1.375733
7	1	0	1.489621	0.004259	-1.324735
8	1	0	0.659543	-1.437472	1.239985
9	1	0	-0.869745	-1.205181	-1.418786
10	1	0	0.572285	1.359351	1.268732
11	8	0	-1.460425	-0.074505	0.224298
12	6	0	-1.666757	-2.438307	0.148025
13	1	0	-1.195680	-3.367581	-0.179059
14	1	0	-1.777846	-2.436445	1.234004
15	8	0	-2.959781	-2.377322	-0.479466
16	8	0	1.263901	-2.398169	-0.501926
17	8	0	2.614647	0.043031	0.411566
18	8	0	-1.626070	2.191176	0.287540
19	6	0	-4.028500	-2.049792	0.293066
20	6	0	2.041672	-3.236201	0.243220
21	6	0	-2.797242	2.548416	-0.329954
22	6	0	3.738904	-0.079238	-0.348229
23	8	0	-3.197013	2.024900	-1.344527

24	8	0	-3.989849	-1.953824	1.501069
25	8	0	2.184395	-3.134483	1.441624
26	8	0	3.728908	-0.212886	-1.552856
27	6	0	-3.475616	3.648437	0.433549
28	6	0	4.962044	-0.027944	0.524361
29	6	0	2.675503	-4.275953	-0.638505
30	6	0	-5.241608	-1.814656	-0.564988
31	1	0	-4.348684	3.994492	-0.119258
32	1	0	-3.786227	3.271841	1.413756
33	1	0	-2.782240	4.476544	0.605869
34	1	0	4.919396	-0.831057	1.266703
35	1	0	5.856632	-0.133559	-0.088971
36	1	0	4.993152	0.919649	1.070693
37	1	0	3.279827	-4.951758	-0.033874
38	1	0	1.903304	-4.840050	-1.170338
39	1	0	3.301315	-3.786973	-1.391525
40	1	0	-6.135618	-1.783903	0.058052
41	1	0	-5.125370	-0.853697	-1.078719
42	1	0	-5.336101	-2.589912	-1.329532
43	6	0	1.602212	3.391377	0.349187
44	8	0	1.542823	3.375997	1.558250
45	6	0	2.180914	4.494340	-0.492771
46	1	0	1.421122	4.878210	-1.180500
47	1	0	2.539504	5.297556	0.150510
48	1	0	3.005404	4.106664	-1.098955
49	8	0	1.145307	2.381501	-0.446306

Structure 100β (M06-2X, Gas Phase)

Energy (Hartrees): = - 1450.3300927
No imaginary frequencies

Standard orientation:

Center Number	Atomic Number	Atomic Type	Coordinates (Angstroms) X	Y	Z
1	6	0	-1.081947	0.950850	-0.334040
2	6	0	0.328231	1.303689	0.101385
3	6	0	1.294939	0.177321	-0.249916
4	6	0	0.767965	-1.190080	0.196877
5	6	0	-0.679487	-1.345932	-0.262526
6	1	0	-1.174757	0.900244	-1.426519
7	1	0	1.468436	0.153038	-1.330277
8	1	0	0.852180	-1.286659	1.282944
9	1	0	-0.710166	-1.339432	-1.362296
10	1	0	0.332134	1.485015	1.177909
11	8	0	-1.438924	-0.269437	0.247018
12	6	0	-1.345467	-2.615186	0.227285
13	1	0	-0.782184	-3.488677	-0.096965
14	1	0	-1.443397	-2.596300	1.314094
15	8	0	-2.629543	-2.684850	-0.381952
16	8	0	1.499062	-2.234344	-0.448233
17	8	0	2.511971	0.506397	0.416429
18	8	0	-1.948671	1.925451	0.179447
19	6	0	-3.682575	-2.217834	0.339462
20	6	0	2.625748	-2.689331	0.150860
21	6	0	-3.234406	1.857657	-0.264714
22	6	0	3.678316	0.141449	-0.159769
23	8	0	-3.584955	1.113131	-1.132659
24	8	0	-3.630779	-1.974493	1.508964
25	8	0	2.984973	-2.329042	1.236126
26	8	0	3.748379	-0.383257	-1.236948
27	6	0	-4.103996	2.812425	0.495260
28	6	0	4.826906	0.439887	0.754040
29	6	0	3.350983	-3.642878	-0.748076
30	6	0	-4.882428	-2.043931	-0.544221
31	1	0	-5.060223	2.912734	-0.010990
32	1	0	-4.257173	2.405651	1.497039
33	1	0	-3.609086	3.777385	0.597822
34	1	0	4.822709	-0.331105	1.528782
35	1	0	5.758075	0.398172	0.195175
36	1	0	4.693420	1.407765	1.235081
37	1	0	4.100190	-4.182913	-0.175337
38	1	0	2.654328	-4.327261	-1.230017
39	1	0	3.835272	-3.041846	-1.521575
40	1	0	-5.778370	-1.997359	0.069902
41	1	0	-4.756381	-1.100457	-1.081689
42	1	0	-4.945898	-2.851638	-1.271626
43	6	0	1.158681	3.535248	0.145140
44	8	0	1.195951	3.543680	1.338220
45	6	0	1.555277	4.664917	-0.761548
46	1	0	0.709498	4.937092	-1.393852
47	1	0	1.870013	5.513765	-0.160902
48	1	0	2.365888	4.338301	-1.414149
49	8	0	0.741917	2.472374	-0.593353

Structure 100β (M06-2X, $CHCl_3$)

Energy (Hartrees): = - 1450.3559706
No imaginary frequencies

Standard orientation:

Center Number	Atomic Number	Atomic Type	Coordinates (Angstroms) X	Y	Z
1	6	0	-1.078952	0.946341	-0.342907
2	6	0	0.326879	1.294090	0.112411
3	6	0	1.296850	0.178012	-0.255902
4	6	0	0.770639	-1.193063	0.174910
5	6	0	-0.677291	-1.351920	-0.286078
6	1	0	-1.155892	0.903949	-1.436039
7	1	0	1.468056	0.171549	-1.336117
8	1	0	0.849295	-1.302076	1.259852
9	1	0	-0.710725	-1.340567	-1.384865
10	1	0	0.320687	1.450522	1.192562
11	8	0	-1.444314	-0.278564	0.228155
12	6	0	-1.323325	-2.627924	0.209877
13	1	0	-0.770040	-3.497153	-0.142278
14	1	0	-1.384745	-2.629921	1.298787
15	8	0	-2.629243	-2.699304	-0.362035
16	8	0	1.506888	-2.229138	-0.479894
17	8	0	2.514700	0.499970	0.417631
18	8	0	-1.943960	1.925030	0.168185
19	6	0	-3.666621	-2.265784	0.388995
20	6	0	2.611919	-2.716659	0.128090
21	6	0	-3.214972	1.911825	-0.318986
22	6	0	3.684816	0.166809	-0.166726
23	8	0	-3.567306	1.163851	-1.186543
24	8	0	-3.588244	-2.034602	1.563660
25	8	0	2.973004	-2.361590	1.217195
26	8	0	3.757638	-0.354791	-1.247422
27	6	0	-4.060846	2.923853	0.384323
28	6	0	4.835096	0.505190	0.727304
29	6	0	3.303738	-3.710468	-0.749702
30	6	0	-4.897600	-2.110362	-0.450812
31	1	0	-5.016443	3.019022	-0.125170
32	1	0	-4.219829	2.585793	1.411030
33	1	0	-3.544009	3.882994	0.423918
34	1	0	4.846132	-0.222534	1.542798
35	1	0	5.765327	0.443291	0.167635
36	1	0	4.704100	1.496614	1.160866
37	1	0	4.079794	-4.220718	-0.184491
38	1	0	2.587716	-4.424974	-1.156480
39	1	0	3.751403	-3.164998	-1.584106
40	1	0	-5.774931	-2.062098	0.190278
41	1	0	-4.801214	-1.174582	-1.008324
42	1	0	-4.983103	-2.927461	-1.166450
43	6	0	1.131378	3.538463	0.194852
44	8	0	1.151605	3.529674	1.392494
45	6	0	1.521070	4.683872	-0.687717
46	1	0	0.676677	4.954953	-1.323671
47	1	0	1.819239	5.531300	-0.075557
48	1	0	2.343739	4.378132	-1.336544
49	8	0	0.740744	2.478877	-0.556330

Structure 101α (B3LYP, Gas Phase)

Energy (Hartrees): = - 1277.9830263
No imaginary frequencies

Standard orientation:

Center Number	Atomic Number	Atomic Type	Coordinates (Angstroms) X	Y	Z
1	6	0	0.425971	1.935139	-0.858047
2	6	0	-1.028481	1.523867	-1.130774
3	6	0	-1.414847	0.327523	-0.255233
4	6	0	-0.381467	-0.801480	-0.360076
5	6	0	1.041047	-0.253899	-0.135787
6	1	0	-1.521913	0.638516	0.786223
7	1	0	-0.469791	-1.294122	-1.333055
8	1	0	1.134836	0.064592	0.909191
9	1	0	-1.035244	1.178151	-2.179500
10	8	0	1.296217	0.856294	-1.004559
11	6	0	2.096969	-1.299564	-0.437655
12	1	0	1.859623	-2.240579	0.064330
13	1	0	2.157938	-1.484526	-1.514466
14	8	0	3.356089	-0.797578	0.042268

15	8	0	-0.591980	-1.771825	0.685766
16	8	0	-2.699903	-0.117497	-0.748870
17	6	0	4.420082	-1.615217	-0.148920
18	6	0	-1.345505	-2.872102	0.408262
19	6	0	-3.602536	-0.614104	0.140380
20	8	0	4.337194	-2.705047	-0.670243
21	8	0	-1.809278	-3.113814	-0.683634
22	8	0	-3.416762	-0.671262	1.335356
23	6	0	-4.826036	-1.115388	-0.582426
24	6	0	-1.539375	-3.697961	1.652193
25	6	0	5.685804	-0.975946	0.367787
26	1	0	-4.574085	-2.073240	-1.049468
27	1	0	-5.636992	-1.261451	0.130605
28	1	0	-5.127738	-0.426897	-1.374698
29	1	0	-1.928605	-4.679081	1.381854
30	1	0	-0.605148	-3.793893	2.209824
31	1	0	-2.260635	-3.180073	2.292760
32	1	0	6.517880	-1.667592	0.242076
33	1	0	5.885117	-0.050283	-0.180236
34	1	0	5.572282	-0.711990	1.422994
35	8	0	0.549907	2.462254	0.477983
36	6	0	0.520629	3.815388	0.617579
37	8	0	0.434055	4.587259	-0.311924
38	1	0	0.752238	2.701582	-1.562926
39	6	0	0.599272	4.199124	2.072111
40	1	0	1.413192	3.664619	2.568133
41	1	0	0.741376	5.275898	2.156401
42	1	0	-0.334620	3.913640	2.566491
43	7	0	-1.937922	2.632560	-0.864803
44	1	0	-1.654859	3.458148	-1.386554
45	1	0	-2.872864	2.378110	-1.169848

Structure 101α (B3LYP, $CHCl_3$)

Energy (Hartrees): = - 1278.0061703
No imaginary frequencies

Standard orientation:

Center Number	Atomic Number	Atomic Type	Coordinates (Angstroms) X	Y	Z
1	6	0	0.389090	1.903409	-0.869728
2	6	0	-1.058009	1.471722	-1.153889
3	6	0	-1.435058	0.299982	-0.243557
4	6	0	-0.400307	-0.823930	-0.344476
5	6	0	1.013622	-0.271609	-0.086580
6	1	0	-1.528794	0.635095	0.791356
7	1	0	-0.459169	-1.298011	-1.327591
8	1	0	1.083445	0.060968	0.956005
9	1	0	-1.047365	1.096765	-2.190821
10	8	0	1.277663	0.830470	-0.968195
11	6	0	2.082227	-1.312790	-0.356023
12	1	0	1.857917	-2.237965	0.180923
13	1	0	2.147073	-1.532524	-1.425619
14	8	0	3.335947	-0.775910	0.109560
15	8	0	-0.660271	-1.809107	0.675766
16	8	0	-2.718766	-0.187574	-0.697144
17	6	0	4.427299	-1.542770	-0.111019
18	6	0	-1.256221	-2.979219	0.314512
19	6	0	-3.661588	-0.516032	0.227478
20	8	0	4.377662	-2.629013	-0.652398
21	8	0	-1.563713	-3.255864	-0.825071
22	8	0	-3.502177	-0.413457	1.424905
23	6	0	-4.903204	-1.024435	-0.454016
24	6	0	-1.477471	-3.846383	1.522839
25	6	0	5.673012	-0.868588	0.398459
26	1	0	-4.666269	-1.937169	-1.010023
27	1	0	-5.672059	-1.233727	0.289543
28	1	0	-5.270667	-0.287797	-1.174519
29	1	0	-1.877883	-4.811886	1.214216
30	1	0	-0.540087	-3.985111	2.069032
31	1	0	-2.181945	-3.351816	2.199357
32	1	0	6.537369	-1.505987	0.212791
33	1	0	5.808227	0.095203	-0.101964
34	1	0	5.581437	-0.670684	1.470981
35	8	0	0.482244	2.466745	0.454248
36	6	0	0.558990	3.819204	0.567667
37	8	0	0.587353	4.573345	-0.382597
38	1	0	0.721152	2.650430	-1.591819
39	6	0	0.596732	4.228442	2.013539
40	1	0	1.395058	3.696886	2.539271
41	1	0	0.749846	5.304996	2.087696
42	1	0	-0.350622	3.957564	2.490760
43	7	0	-1.979559	2.584812	-0.939203
44	1	0	-1.736366	3.353379	-1.561269

45	1	0	-2.915587	2.288609	-1.207719

Structure 101α (M06-2X, Gas Phase)

Energy (Hartrees): = - 1277.8206948
No imaginary frequencies

Standard orientation:

Center Number	Atomic Number	Atomic Type	Coordinates (Angstroms) X	Y	Z
1	6	0	0.441724	1.924172	-0.860680
2	6	0	-0.990390	1.490090	-1.169473
3	6	0	-1.368545	0.310689	-0.282857
4	6	0	-0.330105	-0.802809	-0.378695
5	6	0	1.068527	-0.233424	-0.123652
6	1	0	-1.465127	0.638100	0.754778
7	1	0	-0.392973	-1.288542	-1.357329
8	1	0	1.131962	0.089044	0.921079
9	1	0	-0.982522	1.139416	-2.212773
10	8	0	1.328545	0.865988	-0.985760
11	6	0	2.134297	-1.264886	-0.399585
12	1	0	1.895648	-2.209020	0.093567
13	1	0	2.223391	-1.436743	-1.475017
14	8	0	3.363269	-0.749973	0.110773
15	8	0	-0.531728	-1.770509	0.655128
16	8	0	-2.643844	-0.134146	-0.764426
17	6	0	4.450062	-1.504256	-0.138212
18	6	0	-1.359615	-2.811086	0.410189
19	6	0	-3.497580	-0.707100	0.112534
20	8	0	4.401038	-2.546717	-0.727593
21	8	0	-1.872312	-3.010687	-0.655854
22	8	0	-3.280084	-0.797158	1.288713
23	6	0	-4.700013	-1.249974	-0.599590
24	6	0	-1.565014	-3.620649	1.654119
25	6	0	5.689265	-0.860826	0.417881
26	1	0	-4.388676	-2.182595	-1.076984
27	1	0	-5.489226	-1.448584	0.120910
28	1	0	-5.037367	-0.564061	-1.375043
29	1	0	-2.048181	-4.560351	1.400447
30	1	0	-0.616999	-3.790631	2.162811
31	1	0	-2.208472	-3.033163	2.313242
32	1	0	6.550341	-1.482882	0.189515
33	1	0	5.809189	0.131900	-0.017370
34	1	0	5.583333	-0.739119	1.496674
35	8	0	0.502791	2.424634	0.476038
36	6	0	0.345495	3.758389	0.640404
37	8	0	0.263823	4.532779	-0.270616
38	1	0	0.776948	2.701179	-1.546164
39	6	0	0.277935	4.105662	2.097755
40	1	0	1.076711	3.603741	2.642920
41	1	0	0.341587	5.183937	2.215854
42	1	0	-0.676594	3.744972	2.486532
43	7	0	-1.916394	2.580881	-0.912913
44	1	0	-1.649597	3.411619	-1.431563
45	1	0	-2.846855	2.307989	-1.207764

Structure 101α (M06-2X, $CHCl_3$)

Energy (Hartrees): = - 1277.8446797
No imaginary frequencies

Standard orientation:

Center Number	Atomic Number	Atomic Type	Coordinates (Angstroms) X	Y	Z
1	6	0	0.438709	1.915597	-0.859513
2	6	0	-0.988647	1.477007	-1.179178
3	6	0	-1.371276	0.307324	-0.282548
4	6	0	-0.335749	-0.807652	-0.371334
5	6	0	1.062860	-0.243315	-0.104775
6	1	0	-1.466685	0.642722	0.752560
7	1	0	-0.386070	-1.290018	-1.351626
8	1	0	1.122162	0.077428	0.940883
9	1	0	-0.967986	1.117693	-2.218212
10	8	0	1.331751	0.857768	-0.965933
11	6	0	2.124502	-1.279454	-0.379225
12	1	0	1.893484	-2.213267	0.137084
13	1	0	2.196672	-1.469612	-1.452853
14	8	0	3.362740	-0.752901	0.104312
15	8	0	-0.554657	-1.776029	0.659832
16	8	0	-2.647411	-0.144280	-0.758122
17	6	0	4.450611	-1.501533	-0.140077

18	6	0	-1.358415	-2.828802	0.398958
19	6	0	-3.517727	-0.676683	0.124345
20	8	0	4.398713	-2.558618	-0.711264
21	8	0	-1.858641	-3.026471	-0.676044
22	8	0	-3.296773	-0.763325	1.302770
23	6	0	-4.751216	-1.159920	-0.572513
24	6	0	-1.548559	-3.664423	1.625088
25	6	0	5.691221	-0.845723	0.386794
26	1	0	-4.486316	-2.055520	-1.140093
27	1	0	-5.515394	-1.401968	0.162238
28	1	0	-5.113866	-0.408998	-1.274605
29	1	0	-2.055287	-4.590009	1.363257
30	1	0	-0.588604	-3.868009	2.100015
31	1	0	-2.159555	-3.090886	2.326354
32	1	0	6.554275	-1.470231	0.169508
33	1	0	5.808868	0.135530	-0.076093
34	1	0	5.595034	-0.696114	1.463756
35	8	0	0.491489	2.422776	0.476155
36	6	0	0.372851	3.757682	0.646167
37	8	0	0.319759	4.537491	-0.265955
38	1	0	0.778339	2.685742	-1.550235
39	6	0	0.312308	4.103767	2.101075
40	1	0	1.120836	3.608497	2.639770
41	1	0	0.371102	5.182456	2.223836
42	1	0	-0.635813	3.736511	2.500910
43	7	0	-1.917407	2.573534	-0.948583
44	1	0	-1.661655	3.373187	-1.521209
45	1	0	-2.846284	2.285621	-1.240756

Structure 101β (B3LYP, Gas Phase)

Energy (Hartrees): = - 1277.9828222
No imaginary frequencies

Standard orientation:

Center Number	Atomic Number	Atomic Type	Coordinates (Angstroms) X	Y	Z
1	6	0	-1.105345	-1.329414	0.241555
2	6	0	0.237909	-1.877235	-0.238717
3	6	0	1.349682	-0.905797	0.185801
4	6	0	1.029897	0.551320	-0.190918
5	6	0	-0.397973	0.907008	0.259585
6	1	0	-1.172940	-1.320440	1.337348
7	1	0	1.518177	-0.973182	1.264771
8	1	0	1.152156	0.692208	-1.268367
9	1	0	-0.437518	0.876615	1.360119
10	1	0	0.194256	-1.874816	-1.340843
11	8	0	-1.309328	-0.039342	-0.284381
12	6	0	-0.862274	2.282692	-0.198038
13	1	0	-0.159615	3.047700	0.136931
14	1	0	-0.974378	2.314825	-1.284254
15	8	0	-2.122027	2.556141	0.430915
16	8	0	1.906722	1.451848	0.515638
17	8	0	2.540562	-1.357224	-0.499284
18	8	0	-2.123909	-2.161073	-0.291256
19	6	0	-3.244439	2.408955	-0.332002
20	6	0	3.034943	1.885941	-0.111277
21	6	0	-3.372178	-2.024888	0.260712
22	6	0	3.742719	-1.225171	0.126754
23	8	0	-3.612482	-1.306466	1.200541
24	8	0	-3.235406	2.288120	-1.534566
25	8	0	3.322408	1.604574	-1.253385
26	8	0	3.877017	-0.807935	1.255189
27	6	0	-4.365725	-2.881353	-0.479794
28	6	0	4.860369	-1.625775	-0.800467
29	6	0	3.869115	2.713999	0.829571
30	6	0	-4.469008	2.395742	0.545750
31	1	0	-5.313983	-2.882466	0.056272
32	1	0	-4.512939	-2.474807	-1.485372
33	1	0	-3.988485	-3.901292	-0.589809
34	1	0	5.016954	-0.806556	-1.510132
35	1	0	5.772101	-1.786631	-0.225759
36	1	0	4.600752	-2.518753	-1.372920
37	1	0	4.617009	3.268630	0.263543
38	1	0	3.246175	3.392269	1.416493
39	1	0	4.368442	2.031052	1.524728
40	1	0	-5.355973	2.580753	-0.059930
41	1	0	-4.543886	1.404614	1.007097
42	1	0	-4.387063	3.133294	1.347057
43	7	0	0.484196	-3.188036	0.352817
44	1	0	-0.212562	-3.843135	0.007526
45	1	0	1.389353	-3.528921	0.039407

Structure 101β (B3LYP, $CHCl_3$)

Energy (Hartrees): = - 1278.0060672
No imaginary frequencies

Standard orientation:

Center Number	Atomic Number	Atomic Type	Coordinates (Angstroms) X	Y	Z
1	6	0	-1.070819	1.372466	-0.180096
2	6	0	0.282590	1.863947	0.337471
3	6	0	1.362299	0.891692	-0.153967
4	6	0	1.015562	-0.563714	0.178202
5	6	0	-0.408750	-0.887022	-0.310093
6	1	0	-1.128875	1.434573	-1.273667
7	1	0	1.501742	1.000519	-1.232884
8	1	0	1.103896	-0.736793	1.253317
9	1	0	-0.436565	-0.816316	-1.408197
10	1	0	0.241085	1.801800	1.437095
11	8	0	-1.316791	0.053222	0.261166
12	6	0	-0.874149	-2.272690	0.108483
13	1	0	-0.174500	-3.028754	-0.253091
14	1	0	-0.972970	-2.340619	1.193507
15	8	0	-2.141279	-2.542811	-0.517591
16	8	0	1.923840	-1.436798	-0.522855
17	8	0	2.593096	1.254017	0.511369
18	8	0	-2.073626	2.191425	0.400416
19	6	0	-3.253968	-2.513242	0.260437
20	6	0	2.915645	-2.049998	0.180584
21	6	0	-3.287700	2.234897	-0.232690
22	6	0	3.743242	1.297577	-0.216011
23	8	0	-3.521990	1.646432	-1.263260
24	8	0	-3.236045	-2.420767	1.469450
25	8	0	3.072366	-1.923964	1.376169
26	8	0	3.799532	1.085636	-1.408190
27	6	0	-4.245673	3.102142	0.533089
28	6	0	4.910024	1.624849	0.675428
29	6	0	3.765069	-2.885834	-0.736847
30	6	0	-4.491733	-2.599974	-0.590563
31	1	0	-5.188306	3.175242	-0.008853
32	1	0	-4.420245	2.669943	1.523589
33	1	0	-3.818658	4.098851	0.679518
34	1	0	5.070207	0.798481	1.375807
35	1	0	5.805139	1.772299	0.071374
36	1	0	4.702195	2.520676	1.267239
37	1	0	4.548685	-3.380292	-0.163205
38	1	0	3.147180	-3.632485	-1.244790
39	1	0	4.210523	-2.248917	-1.507238
40	1	0	-5.359718	-2.795821	0.039249
41	1	0	-4.628302	-1.644202	-1.108754
42	1	0	-4.390774	-3.378073	-1.351639
43	7	0	0.554109	3.204103	-0.177758
44	1	0	-0.119663	3.852514	0.224581
45	1	0	1.466513	3.506947	0.157385

Structure 101β (M06-2X, Gas Phase)

Energy (Hartrees): = - 1277.8205255
No imaginary frequencies

Standard orientation:

Center Number	Atomic Number	Atomic Type	Coordinates (Angstroms) X	Y	Z
1	6	0	-1.112815	1.284018	-0.275413
2	6	0	0.204923	1.848931	0.222924
3	6	0	1.323894	0.898388	-0.196297
4	6	0	1.024237	-0.548128	0.207314
5	6	0	-0.383410	-0.923377	-0.256106
6	1	0	-1.151157	1.257357	-1.371803
7	1	0	1.474301	0.949799	-1.278999
8	1	0	1.130472	-0.664351	1.289537
9	1	0	-0.411651	-0.909179	-1.355881
10	1	0	0.149705	1.849706	1.321777
11	8	0	-1.309446	0.007743	0.259471
12	6	0	-0.833829	-2.289804	0.218727
13	1	0	-0.146204	-3.058047	-0.130837
14	1	0	-0.915177	-2.306014	1.306988
15	8	0	-2.100867	-2.552962	-0.373640
16	8	0	1.901901	-1.452362	-0.468766
17	8	0	2.500290	1.382790	0.463443
18	8	0	-2.147481	2.101155	0.218441

19	6	0	-3.201251	-2.242207	0.359717
20	6	0	3.080031	-1.761572	0.117764
21	6	0	-3.389347	1.837157	-0.274106
22	6	0	3.699129	1.177054	-0.127337
23	8	0	-3.590074	1.051665	-1.152838
24	8	0	-3.174069	-2.001850	1.530908
25	8	0	3.391836	-1.382759	1.212526
26	8	0	3.826721	0.694041	-1.217904
27	6	0	-4.424366	2.648928	0.446098
28	6	0	4.813889	1.583099	0.788390
29	6	0	3.928318	-2.583031	-0.804353
30	6	0	-4.421624	-2.237773	-0.513384
31	1	0	-5.382449	2.537815	-0.054112
32	1	0	-4.494731	2.284756	1.472666
33	1	0	-4.123669	3.695980	0.481008
34	1	0	4.916475	0.786628	1.529865
35	1	0	5.735288	1.679126	0.220007
36	1	0	4.573156	2.507861	1.310274
37	1	0	4.729914	-3.052484	-0.240182
38	1	0	3.327038	-3.324442	-1.328060
39	1	0	4.346786	-1.894817	-1.543041
40	1	0	-5.311554	-2.262631	0.110480
41	1	0	-4.404472	-1.313817	-1.096867
42	1	0	-4.401613	-3.081371	-1.202012
43	7	0	0.437553	3.154005	-0.371080
44	1	0	-0.273508	3.800550	-0.046221
45	1	0	1.333012	3.509753	-0.053788

Structure 101β (M06-2X, $CHCl_3$)

Energy (Hartrees): = - 1277.8442683
No imaginary frequencies

Standard orientation:

Center Number	Atomic Number	Atomic Type	Coordinates (Angstroms) X	Y	Z
1	6	0	-1.113386	1.286808	-0.259024
2	6	0	0.205305	1.842343	0.250827
3	6	0	1.321620	0.897241	-0.187236
4	6	0	1.020967	-0.551977	0.197017
5	6	0	-0.383897	-0.922924	-0.279146
6	1	0	-1.148828	1.278832	-1.355170
7	1	0	1.463948	0.964431	-1.269672
8	1	0	1.115059	-0.683286	1.278117
9	1	0	-0.411721	-0.887203	-1.377744
10	1	0	0.152111	1.826537	1.349095
11	8	0	-1.314551	-0.001682	0.252820
12	6	0	-0.818477	-2.299043	0.178404
13	1	0	-0.138751	-3.059293	-0.202851
14	1	0	-0.869954	-2.344036	1.267057
15	8	0	-2.101544	-2.560360	-0.390778
16	8	0	1.906575	-1.447358	-0.482318
17	8	0	2.504673	1.366916	0.473253
18	8	0	-2.146456	2.097507	0.249087
19	6	0	-3.189094	-2.288860	0.364649
20	6	0	3.065180	-1.790148	0.119061
21	6	0	-3.382611	1.894187	-0.280546
22	6	0	3.696991	1.199707	-0.136628
23	8	0	-3.586739	1.125533	-1.177184
24	8	0	-3.144882	-2.067527	1.543115
25	8	0	3.373060	-1.415033	1.218688
26	8	0	3.818519	0.721917	-1.233033
27	6	0	-4.401399	2.741866	0.412843
28	6	0	4.813968	1.656205	0.748574
29	6	0	3.892879	-2.656099	-0.777453
30	6	0	-4.429161	-2.303287	-0.476102
31	1	0	-5.366770	2.622679	-0.072523
32	1	0	-4.465316	2.429666	1.457277
33	1	0	-4.089470	3.787104	0.393460
34	1	0	4.911622	0.932137	1.561260
35	1	0	5.739089	1.696903	0.178620
36	1	0	4.584827	2.628010	1.185933
37	1	0	4.710489	-3.094368	-0.210269
38	1	0	3.278313	-3.431065	-1.235340
39	1	0	4.292730	-2.023802	-1.574017
40	1	0	-5.307295	-2.331700	0.164955
41	1	0	-4.438419	-1.386297	-1.071362
42	1	0	-4.419427	-3.152754	-1.158827
43	7	0	0.438758	3.160169	-0.319206
44	1	0	-0.261808	3.803615	0.037380
45	1	0	1.335718	3.507290	0.008422

Structure 102α (B3LYP, Gas Phase)

Energy (Hartrees): = - 1430.6535068
No imaginary frequencies

Standard orientation:

Center Number	Atomic Number	Atomic Type	Coordinates (Angstroms) X	Y	Z
1	6	0	0.379566	-1.579657	-0.488521
2	6	0	1.414129	-0.453858	-0.673729
3	6	0	0.971502	0.781938	0.116949
4	6	0	-0.460551	1.168802	-0.250006
5	6	0	-1.404376	-0.038637	-0.096845
6	1	0	1.038527	0.589902	1.191336
7	1	0	-0.490138	1.562745	-1.269532
8	1	0	-1.470520	-0.302592	0.966120
9	1	0	1.420251	-0.222816	-1.744203
10	8	0	-0.905103	-1.151247	-0.845754
11	6	0	-2.795738	0.262470	-0.619218
12	1	0	-3.164384	1.210115	-0.219493
13	1	0	-2.790355	0.324975	-1.711813
14	8	0	-3.656975	-0.808410	-0.197303
15	8	0	-0.899942	2.192109	0.663762
16	8	0	1.803821	1.919735	-0.197247
17	7	0	2.752312	-0.867211	-0.305731
18	6	0	-4.977707	-0.629201	-0.449285
19	6	0	-1.084572	3.454418	0.169055
20	6	0	2.866656	2.167466	0.604895
21	8	0	-5.426993	0.369420	-0.965843
22	8	0	-0.937255	3.754313	-0.990992
23	8	0	3.202269	1.451512	1.527484
24	6	0	3.570312	3.429975	0.179708
25	6	0	-1.509163	4.386100	1.276214
26	6	0	-5.774236	-1.832309	-0.008040
27	1	0	2.854362	4.248334	0.071182
28	1	0	4.336773	3.683563	0.910942
29	1	0	4.034119	3.273233	-0.799309
30	1	0	-1.605953	5.396457	0.881090
31	1	0	-2.466867	4.057629	1.690909
32	1	0	-0.778073	4.365438	2.089102
33	1	0	-6.836597	-1.631342	-0.140577
34	1	0	-5.485239	-2.703799	-0.603240
35	1	0	-5.561209	-2.067282	1.038332
36	8	0	0.415829	-1.979454	0.893742
37	6	0	0.278305	-3.315310	1.169625
38	8	0	0.193492	-4.171374	0.324611
39	1	0	0.610962	-2.435445	-1.120844
40	6	0	0.256297	-3.538341	2.660730
41	1	0	-0.631028	-3.064104	3.091296
42	1	0	0.237960	-4.607753	2.866854
43	1	0	1.131565	-3.077274	3.126414
44	6	0	3.566136	-1.498491	-1.210161
45	8	0	3.182838	-1.788498	-2.337072
46	6	0	4.959720	-1.836942	-0.708685
47	1	0	5.017382	-2.913629	-0.520729
48	1	0	5.678351	-1.603251	-1.496819
49	1	0	5.234805	-1.305563	0.206310
50	1	0	3.105589	-0.582437	0.597563

Structure 102α (B3LYP, $CHCl_3$)

Energy (Hartrees): = - 1430.6816507
No imaginary frequencies

Standard orientation:

Center Number	Atomic Number	Atomic Type	Coordinates (Angstroms) X	Y	Z
1	6	0	0.162893	-1.664314	-0.523407
2	6	0	1.338998	-0.687075	-0.673633
3	6	0	1.064856	0.592416	0.132280
4	6	0	-0.305320	1.171558	-0.217771
5	6	0	-1.396520	0.097758	-0.060433
6	1	0	1.115979	0.389298	1.204759
7	1	0	-0.298757	1.568742	-1.235778
8	1	0	-1.477707	-0.178102	0.998261
9	1	0	1.411895	-0.433348	-1.734421
10	8	0	-1.057337	-1.058874	-0.841445
11	6	0	-2.745957	0.579956	-0.555474
12	1	0	-2.996757	1.545727	-0.109386
13	1	0	-2.744778	0.685010	-1.644088
14	8	0	-3.721720	-0.403369	-0.158706
15	8	0	-0.587428	2.236955	0.710734
16	8	0	2.053355	1.584593	-0.198109

17	7	0	2.598629	-1.296388	-0.308571
18	6	0	-4.997495	-0.157255	-0.533949
19	6	0	-0.662443	3.512798	0.232007
20	6	0	3.048154	1.821242	0.697559
21	8	0	-5.328162	0.836677	-1.148478
22	8	0	-0.553237	3.801159	-0.938940
23	8	0	3.148793	1.248396	1.762703
24	6	0	3.983310	2.876540	0.175596
25	6	0	-0.902955	4.477497	1.360063
26	6	0	-5.912336	-1.270747	-0.099798
27	1	0	3.425851	3.784889	-0.071301
28	1	0	4.746004	3.095025	0.922785
29	1	0	4.457114	2.524364	-0.746087
30	1	0	-0.982377	5.490507	0.966109
31	1	0	-1.821603	4.212113	1.892114
32	1	0	-0.080320	4.421508	2.079940
33	1	0	-6.944746	-1.009433	-0.331805
34	1	0	-5.641119	-2.193703	-0.622677
35	1	0	-5.804170	-1.457412	0.972564
36	8	0	0.168890	-2.127610	0.842500
37	6	0	-0.323798	-3.381937	1.085653
38	8	0	-0.693303	-4.131572	0.211334
39	1	0	0.267252	-2.517929	-1.192670
40	6	0	-0.333064	-3.668069	2.561022
41	1	0	-1.032996	-2.989844	3.060096
42	1	0	-0.638284	-4.699976	2.733279
43	1	0	0.657378	-3.492064	2.990682
44	6	0	3.591819	-1.547582	-1.215934
45	8	0	3.480063	-1.285194	-2.411675
46	6	0	4.837369	-2.196179	-0.645738
47	1	0	4.904801	-3.222390	-1.021387
48	1	0	5.715143	-1.656021	-1.009741
49	1	0	4.855214	-2.218867	0.446840
50	1	0	2.752224	-1.522311	0.664303

Structure 102α (M06-2X, Gas Phase)

Energy (Hartrees): = - 1430.4649252
No imaginary frequencies

Standard orientation:

Center Number	Atomic Number	Atomic Type	Coordinates (Angstroms) X	Y	Z
1	6	0	0.263664	-1.577938	-0.482706
2	6	0	1.367050	-0.537072	-0.697502
3	6	0	1.029874	0.720292	0.092258
4	6	0	-0.366308	1.204171	-0.261966
5	6	0	-1.381516	0.076103	-0.072920
6	1	0	1.085850	0.523221	1.166389
7	1	0	-0.378976	1.576669	-1.289661
8	1	0	-1.441872	-0.171515	0.993069
9	1	0	1.376704	-0.306409	-1.767001
10	8	0	-0.987095	-1.064207	-0.821349
11	6	0	-2.748695	0.479695	-0.566667
12	1	0	-3.028753	1.459561	-0.174313
13	1	0	-2.760481	0.517349	-1.658388
14	8	0	-3.667973	-0.507634	-0.104656
15	8	0	-0.719793	2.256223	0.635515
16	8	0	1.924984	1.785730	-0.235922
17	7	0	2.667896	-1.041037	-0.329790
18	6	0	-4.945197	-0.331596	-0.495678
19	6	0	-0.638633	3.534975	0.179652
20	6	0	3.017450	1.957058	0.531317
21	8	0	-5.302097	0.604995	-1.152569
22	8	0	-0.336435	3.824561	-0.938908
23	8	0	3.318378	1.224692	1.436189
24	6	0	3.789800	3.163852	0.085884
25	6	0	-0.988263	4.496931	1.279338
26	6	0	-5.817092	-1.450416	0.000051
27	1	0	3.115049	4.009604	-0.045042
28	1	0	4.565982	3.386176	0.812860
29	1	0	4.240108	2.949405	-0.885132
30	1	0	-0.943767	5.512168	0.895264
31	1	0	-1.987915	4.275673	1.654835
32	1	0	-0.288677	4.371228	2.106781
33	1	0	-6.841181	-1.275895	-0.317841
34	1	0	-5.449757	-2.396725	-0.398704
35	1	0	-5.759557	-1.504676	1.087699
36	8	0	0.301196	-1.938827	0.895616
37	6	0	-0.138797	-3.188583	1.216439
38	8	0	-0.488917	-3.990279	0.405864
39	1	0	0.424476	-2.455601	-1.104377
40	6	0	-0.107813	-3.384967	2.705206
41	1	0	-0.806688	-2.689891	3.173322

42	1	0	-0.386630	-4.408857	2.938071
43	1	0	0.888990	-3.161237	3.085890
44	6	0	3.343647	-1.879549	-1.168638
45	8	0	2.880737	-2.227323	-2.234924
46	6	0	4.690067	-2.352128	-0.666978
47	1	0	4.584222	-3.373941	-0.297854
48	1	0	5.380944	-2.369711	-1.508125
49	1	0	5.088632	-1.724192	0.130109
50	1	0	3.048722	-0.783564	0.569291

Structure 102α (M06-2X, $CHCl_3$)

Energy (Hartrees): = - 1430.4952475
No imaginary frequencies
Standard orientation:

Center Number	Atomic Number	Atomic Type	Coordinates (Angstroms) X	Y	Z
1	6	0	0.278791	-1.591989	-0.482942
2	6	0	1.384183	-0.553572	-0.697185
3	6	0	1.052846	0.704005	0.094860
4	6	0	-0.342940	1.190399	-0.252192
5	6	0	-1.363921	0.068789	-0.059116
6	1	0	1.114339	0.509854	1.168945
7	1	0	-0.365853	1.558326	-1.280751
8	1	0	-1.426367	-0.181586	1.006306
9	1	0	1.392366	-0.318410	-1.765266
10	8	0	-0.974320	-1.075888	-0.810786
11	6	0	-2.724081	0.486492	-0.558976
12	1	0	-3.007154	1.454039	-0.138289
13	1	0	-2.720744	0.556580	-1.649392
14	8	0	-3.653111	-0.514507	-0.137930
15	8	0	-0.674663	2.244897	0.650804
16	8	0	1.948496	1.767012	-0.242261
17	7	0	2.683718	-1.061634	-0.328452
18	6	0	-4.931763	-0.310935	-0.497725
19	6	0	-0.687783	3.516631	0.177263
20	6	0	3.034374	1.954199	0.533665
21	8	0	-5.286723	0.658333	-1.114705
22	8	0	-0.507683	3.795900	-0.973856
23	8	0	3.322919	1.230355	1.451352
24	6	0	3.805666	3.156952	0.089397
25	6	0	-0.953591	4.483663	1.289145
26	6	0	-5.810385	-1.435936	-0.041840
27	1	0	3.142435	4.021543	0.034917
28	1	0	4.623830	3.341871	0.780725
29	1	0	4.196882	2.975301	-0.913897
30	1	0	-1.044805	5.488417	0.884211
31	1	0	-1.865597	4.200752	1.816581
32	1	0	-0.128492	4.440164	2.003460
33	1	0	-6.849365	-1.197167	-0.255443
34	1	0	-5.521382	-2.347380	-0.569279
35	1	0	-5.668730	-1.609622	1.025537
36	8	0	0.327286	-1.967100	0.890930
37	6	0	-0.221571	-3.167351	1.219173
38	8	0	-0.683150	-3.915399	0.406357
39	1	0	0.434004	-2.464436	-1.114589
40	6	0	-0.150227	-3.395635	2.696947
41	1	0	-0.687995	-2.598735	3.214129
42	1	0	-0.587451	-4.361450	2.937218
43	1	0	0.891593	-3.360652	3.020434
44	6	0	3.386445	-1.871024	-1.164109
45	8	0	2.953766	-2.203813	-2.254823
46	6	0	4.725722	-2.334104	-0.646766
47	1	0	4.662596	-3.401771	-0.426055
48	1	0	5.466826	-2.199910	-1.435029
49	1	0	5.041238	-1.800116	0.249768
50	1	0	3.048681	-0.835995	0.586662

Structure 102β (B3LYP, Gas Phase)

Energy (Hartrees): = - 1430.6496435
No imaginary frequencies

Standard orientation:

Center Number	Atomic Number	Atomic Type	Coordinates (Angstroms) X	Y	Z
1	6	0	-0.707199	1.121458	-0.294739
2	6	0	0.760055	1.074196	0.167225
3	6	0	1.359449	-0.280046	-0.256185
4	6	0	0.484743	-1.465613	0.156840
5	6	0	-0.958443	-1.224651	-0.315205

6	1	0	-0.793464	1.142610	-1.390767
7	1	0	1.487772	-0.304033	-1.344213
8	1	0	0.519617	-1.613065	1.238805
9	1	0	-0.972240	-1.181531	-1.416571
10	1	0	0.758768	1.165755	1.257845
11	8	0	-1.405917	0.011113	0.226495
12	6	0	-1.950161	-2.289377	0.132710
13	1	0	-1.618965	-3.277888	-0.192214
14	1	0	-2.082072	-2.270173	1.216758
15	8	0	-3.199275	-2.023152	-0.519122
16	8	0	1.001354	-2.638192	-0.502310
17	8	0	2.652862	-0.468276	0.351079
18	8	0	-1.303489	2.274362	0.252600
19	7	0	1.548927	2.168469	-0.362618
20	6	0	-4.184422	-1.447305	0.233014
21	6	0	1.471347	-3.657124	0.279033
22	6	0	-2.475894	2.688890	-0.320801
23	6	0	3.748226	-0.123653	-0.370959
24	8	0	-2.963293	2.169377	-1.297255
25	8	0	-4.154302	-1.364613	1.437801
26	8	0	1.433817	-3.656408	1.485934
27	8	0	3.702387	0.381676	-1.474289
28	6	0	-3.041983	3.853377	0.446570
29	6	0	5.007183	-0.440329	0.393301
30	6	0	2.020239	-4.762566	-0.587614
31	6	0	-5.276576	-0.921164	-0.661372
32	1	0	-3.865309	4.294346	-0.114254
33	1	0	-3.406992	3.498445	1.415714
34	1	0	-2.264575	4.595252	0.644148
35	1	0	4.997192	-1.480034	0.730159
36	1	0	5.873820	-0.251588	-0.238917
37	1	0	5.057233	0.189340	1.287062
38	1	0	2.387512	-5.569573	0.044947
39	1	0	1.239818	-5.137319	-1.256219
40	1	0	2.829298	-4.379464	-1.216094
41	1	0	-6.174137	-0.734039	-0.072326
42	1	0	-4.928552	0.019256	-1.103333
43	1	0	-5.487106	-1.616993	-1.476481
44	6	0	1.921599	3.239724	0.420144
45	8	0	1.525116	3.392218	1.565555
46	6	0	2.833978	4.236758	-0.277453
47	1	0	2.229892	4.946495	-0.852849
48	1	0	3.386004	4.793226	0.480374
49	1	0	3.534365	3.752837	-0.965024
50	1	0	2.062694	1.998576	-1.217164

Structure 102β (B3LYP, $CHCl_3$)

Energy (Hartrees): = - 1430.6780821
No imaginary frequencies

Standard orientation:

Center Number	Atomic Number	Atomic Type	Coordinates (Angstroms) X	Y	Z
1	6	0	-0.622482	1.200437	-0.324941
2	6	0	0.827460	1.034399	0.160114
3	6	0	1.332580	-0.351762	-0.281312
4	6	0	0.369256	-1.467164	0.123117
5	6	0	-1.054202	-1.128528	-0.353080
6	1	0	-0.683841	1.241456	-1.420798
7	1	0	1.468237	-0.371166	-1.367185
8	1	0	0.384449	-1.612574	1.205504
9	1	0	-1.068415	-1.100096	-1.453694
10	1	0	0.822193	1.099760	1.251442
11	8	0	-1.420512	0.145865	0.173161
12	6	0	-2.097113	-2.124923	0.130376
13	1	0	-1.841079	-3.133663	-0.199922
14	1	0	-2.188218	-2.100044	1.217732
15	8	0	-3.358215	-1.793654	-0.477829
16	8	0	0.807826	-2.674703	-0.530396
17	8	0	2.599862	-0.627461	0.342955
18	8	0	-1.133807	2.394737	0.230191
19	7	0	1.698262	2.083331	-0.324136
20	6	0	-4.310301	-1.222265	0.305600
21	6	0	1.131065	-3.748189	0.246252
22	6	0	-2.213906	2.959317	-0.392186
23	6	0	3.728425	-0.433869	-0.388865
24	8	0	-2.706371	2.513733	-1.404409
25	8	0	-4.228727	-1.123237	1.511101
26	8	0	1.017843	-3.764623	1.452103
27	8	0	3.728271	-0.042109	-1.538243
28	6	0	-2.668657	4.178632	0.356812
29	6	0	4.947590	-0.762521	0.426479
30	6	0	1.630097	-4.881241	-0.607604

31	6	0	-5.454083	-0.727054	-0.537212
32	1	0	-3.477076	4.665791	-0.187858
33	1	0	-3.016886	3.886147	1.352647
34	1	0	-1.833108	4.871273	0.493803
35	1	0	4.871570	-1.775903	0.830911
36	1	0	5.840085	-0.670629	-0.192121
37	1	0	5.015045	-0.075469	1.276131
38	1	0	1.858239	-5.741134	0.021796
39	1	0	0.874393	-5.152939	-1.350803
40	1	0	2.527251	-4.570231	-1.151698
41	1	0	-6.308050	-0.493279	0.098852
42	1	0	-5.133457	0.180879	-1.060291
43	1	0	-5.732202	-1.465768	-1.293168
44	6	0	2.229946	3.043331	0.496205
45	8	0	1.975090	3.107309	1.695831
46	6	0	3.129386	4.044854	-0.201970
47	1	0	2.526578	4.890944	-0.550966
48	1	0	3.861036	4.424394	0.513292
49	1	0	3.647332	3.616355	-1.064605
50	1	0	2.017642	2.035001	-1.282490

Structure 102β (M06-2X, Gas Phase)

Energy (Hartrees): = - 1430.4623786
No imaginary frequencies

Standard orientation:

Center Number	Atomic Number	Atomic Type	Coordinates (Angstroms) X	Y	Z
1	6	0	-0.556158	1.139009	-0.324425
2	6	0	0.886325	0.999918	0.156278
3	6	0	1.399981	-0.384786	-0.245403
4	6	0	0.448765	-1.494360	0.174911
5	6	0	-0.959846	-1.165798	-0.322814
6	1	0	-0.623014	1.149984	-1.421446
7	1	0	1.536155	-0.435400	-1.331312
8	1	0	0.448864	-1.611685	1.260950
9	1	0	-0.952012	-1.131144	-1.423299
10	1	0	0.875999	1.098706	1.245802
11	8	0	-1.328060	0.093802	0.196304
12	6	0	-2.038798	-2.137448	0.117186
13	1	0	-1.823462	-3.147790	-0.226015
14	1	0	-2.148080	-2.124302	1.202032
15	8	0	-3.246492	-1.729441	-0.517261
16	8	0	0.935232	-2.686609	-0.445095
17	8	0	2.656155	-0.645159	0.384359
18	8	0	-1.073207	2.328612	0.199246
19	7	0	1.743622	2.029645	-0.378568
20	6	0	-4.088209	-0.939297	0.200373
21	6	0	0.809842	-3.842936	0.249212
22	6	0	-2.276912	2.717652	-0.296418
23	6	0	3.778443	-0.344639	-0.298148
24	8	0	-2.815845	2.166918	-1.213119
25	8	0	-3.990792	-0.761683	1.378382
26	8	0	0.245287	-3.919997	1.301501
27	8	0	3.782407	0.166866	-1.385447
28	6	0	-2.805370	3.888555	0.474854
29	6	0	4.996208	-0.727756	0.490527
30	6	0	1.460801	-4.975852	-0.490487
31	6	0	-5.121415	-0.328390	-0.700409
32	1	0	-3.622510	4.347634	-0.075182
33	1	0	-3.169114	3.521249	1.436907
34	1	0	-2.006402	4.602384	0.671199
35	1	0	4.952911	-1.788153	0.740432
36	1	0	5.886820	-0.506363	-0.090866
37	1	0	5.004389	-0.167409	1.426615
38	1	0	1.309362	-5.899251	0.061332
39	1	0	1.032844	-5.053434	-1.490586
40	1	0	2.526162	-4.770160	-0.601864
41	1	0	-5.946253	0.045799	-0.099182
42	1	0	-4.645036	0.501436	-1.229162
43	1	0	-5.468828	-1.053977	-1.434589
44	6	0	2.139228	3.103055	0.382733
45	8	0	1.718606	3.304395	1.497765
46	6	0	3.129226	4.013955	-0.312502
47	1	0	2.616133	4.584827	-1.089337
48	1	0	3.544860	4.700596	0.420531
49	1	0	3.928292	3.438972	-0.785492
50	1	0	2.257575	1.823038	-1.223392

Structure 102β (M06-2X, $CHCl_3$)

Energy (Hartrees): = - 1430.4930116

No imaginary frequencies

Standard orientation:

Center Number	Atomic Number	Atomic Type	Coordinates (Angstroms) X	Y	Z
1	6	0	0.582426	-1.260583	-0.277742
2	6	0	-0.857568	-1.024912	0.163577
3	6	0	-1.296414	0.384579	-0.264688
4	6	0	-0.279261	1.445679	0.122151
5	6	0	1.102513	1.024213	-0.374756
6	1	0	0.665784	-1.331406	-1.370628
7	1	0	-1.457644	0.414307	-1.346139
8	1	0	-0.262731	1.580980	1.205866
9	1	0	1.084004	0.939460	-1.471368
10	1	0	-0.901315	-1.094114	1.252921
11	8	0	1.405558	-0.234287	0.200829
12	6	0	2.228085	1.958065	0.024165
13	1	0	2.081154	2.950522	-0.398979
14	1	0	2.316017	2.022142	1.108507
15	8	0	3.430434	1.440453	-0.549464
16	8	0	-0.698695	2.654278	-0.516023
17	8	0	-2.513394	0.716634	0.403880
18	8	0	1.034621	-2.449319	0.315769
19	7	0	-1.722446	-2.045455	-0.384239
20	6	0	4.215851	0.666406	0.232192
21	6	0	-0.496881	3.815621	0.146803
22	6	0	2.202468	-2.947790	-0.170126
23	6	0	-3.683711	0.451980	-0.211509
24	8	0	2.759530	-2.480698	-1.124082
25	8	0	4.077311	0.557615	1.418879
26	8	0	0.083886	3.877325	1.195595
27	8	0	-3.762527	0.032527	-1.336404
28	6	0	2.666389	-4.107850	0.650051
29	6	0	-4.834735	0.720465	0.705766
30	6	0	-1.085860	4.968116	-0.605430
31	6	0	5.260620	-0.023252	-0.591647
32	1	0	3.475074	-4.620710	0.135004
33	1	0	3.024583	-3.725638	1.609198
34	1	0	1.837232	-4.786880	0.848553
35	1	0	-4.702932	1.674192	1.216568
36	1	0	-5.764451	0.706867	0.141956
37	1	0	-4.842777	-0.071711	1.459078
38	1	0	-0.858555	5.895822	-0.086555
39	1	0	-0.683800	4.989545	-1.619343
40	1	0	-2.166716	4.832526	-0.677813
41	1	0	6.030691	-0.429235	0.060238
42	1	0	4.774228	-0.837659	-1.135209
43	1	0	5.690898	0.665300	-1.318751
44	6	0	-2.849051	-2.466424	0.260452
45	8	0	-3.114692	-2.131126	1.401378
46	6	0	-3.753590	-3.347437	-0.563852
47	1	0	-3.190148	-3.998705	-1.233262
48	1	0	-4.375542	-3.943524	0.101001
49	1	0	-4.396571	-2.700761	-1.167902
50	1	0	-1.663304	-2.221616	-1.378576

Structure 103α (B3LYP, Gas Phase)

Energy (Hartrees): = - 1622.3574371
No imaginary frequencies

Standard orientation:

Center Number	Atomic Number	Atomic Type	Coordinates (Angstroms) X	Y	Z
1	6	0	-0.020977	1.217835	-1.230058
2	6	0	-0.649777	-0.156370	-0.904103
3	6	0	0.293196	-0.933078	0.037620
4	6	0	1.702244	-0.992079	-0.573578
5	6	0	2.205114	0.444962	-0.820479
6	1	0	0.338741	-0.418034	0.996742
7	1	0	1.650487	-1.502889	-1.536929
8	1	0	2.250528	0.992297	0.124591
9	1	0	-0.734093	-0.700302	-1.852558
10	8	0	1.286945	1.090392	-1.717219
11	6	0	3.558581	0.521454	-1.531254
12	1	0	3.570504	-0.140163	-2.401474
13	1	0	3.711099	1.545330	-1.880992
14	8	0	4.621402	0.072623	-0.683990
15	8	0	2.609038	-1.813056	0.186188
16	8	0	-0.211056	-2.238464	0.397779
17	7	0	-1.939032	0.041935	-0.276507
18	6	0	-4.317081	-0.211887	-0.244860

19	6	0	-4.468684	0.570492	0.935600
20	6	0	-5.461580	-0.757141	-0.857488
21	6	0	-5.753907	0.779584	1.459144
22	6	0	-6.726666	-0.548233	-0.331104
23	1	0	-5.335010	-1.350011	-1.760382
24	6	0	-6.862219	0.225761	0.831836
25	1	0	-5.850506	1.378520	2.358349
26	1	0	-7.600532	-0.974334	-0.812249
27	1	0	-7.849401	0.396802	1.251790
28	6	0	-3.004137	-0.429992	-0.824903
29	1	0	-2.957650	-1.010051	-1.757575
30	1	0	-0.584938	1.736586	-2.005265
31	8	0	-0.043796	2.008343	-0.034061
32	8	0	-3.415964	1.115165	1.564025
33	1	0	-2.599215	0.833182	1.056656
34	6	0	-0.408300	-3.190045	-0.544877
35	6	0	-0.871363	-4.474194	0.098299
36	1	0	-1.748294	-4.289626	0.724548
37	1	0	-1.105049	-5.203562	-0.676332
38	1	0	-0.082555	-4.864134	0.748529
39	6	0	3.004950	-1.420919	1.426971
40	6	0	4.096506	-2.314893	1.947702
41	1	0	4.078877	-2.311869	3.037926
42	1	0	4.008758	-3.329713	1.557150
43	1	0	5.048481	-1.887698	1.611536
44	6	0	5.335468	0.907905	0.125304
45	6	0	5.147234	2.403398	-0.021592
46	1	0	5.798966	2.889877	0.702375
47	1	0	5.414146	2.739496	-1.028661
48	1	0	4.112314	2.702995	0.168056
49	6	0	-0.565347	3.275176	-0.118541
50	6	0	-0.693919	3.877145	1.255100
51	1	0	-1.569838	3.434332	1.742545
52	1	0	0.179915	3.648763	1.868996
53	1	0	-0.837981	4.953804	1.169455
54	8	0	-0.904709	3.790519	-1.155973
55	8	0	2.564007	-0.447187	2.001015
56	8	0	6.108212	0.408167	0.907567
57	8	0	-0.235397	-3.028055	-1.735294

Structure 103α (B3LYP, $CHCl_3$)

Energy (Hartrees): = - 1622.3875334
No imaginary frequencies

Standard orientation:

Center Number	Atomic Number	Atomic Type	Coordinates (Angstroms) X	Y	Z
1	6	0	-0.010716	-1.281048	-1.199947
2	6	0	0.687815	0.063535	-0.906350
3	6	0	-0.211874	0.901509	0.025694
4	6	0	-1.614406	1.024187	-0.589716
5	6	0	-2.198786	-0.384742	-0.814929
6	1	0	-0.280669	0.403154	0.992157
7	1	0	-1.537184	1.513743	-1.561567
8	1	0	-2.281848	-0.919368	0.134685
9	1	0	0.785080	0.578407	-1.868804
10	8	0	-1.310083	-1.093870	-1.696997
11	6	0	-3.552218	-0.394323	-1.528154
12	1	0	-3.539813	0.274474	-2.392312
13	1	0	-3.760488	-1.406309	-1.880978
14	8	0	-4.591554	0.099122	-0.668431
15	8	0	-2.476003	1.911432	0.153964
16	8	0	0.360742	2.182836	0.371893
17	7	0	1.977923	-0.168547	-0.288960
18	6	0	4.355901	0.098075	-0.263237
19	6	0	4.514725	-0.657664	0.932467
20	6	0	5.495038	0.647286	-0.882602
21	6	0	5.799846	-0.840335	1.465687
22	6	0	6.762004	0.463808	-0.347716
23	1	0	5.362495	1.222041	-1.796054
24	6	0	6.904541	-0.284121	0.831154
25	1	0	5.904306	-1.418525	2.378297
26	1	0	7.631940	0.893127	-0.833847
27	1	0	7.892155	-0.433362	1.258764
28	6	0	3.039675	0.305188	-0.843777
29	1	0	2.990071	0.883175	-1.775701
30	1	0	0.526420	-1.842625	-1.964094
31	8	0	-0.034678	-2.040710	0.016954
32	8	0	3.464565	-1.203068	1.567857
33	1	0	2.645841	-0.950882	1.044620
34	6	0	0.602278	3.123694	-0.571207
35	6	0	1.145842	4.375096	0.064481
36	1	0	2.030952	4.141818	0.663615

37	1	0	1.397443	5.099875	-0.709633
38	1	0	0.396232	4.800753	0.739153
39	6	0	-2.876030	1.592161	1.409635
40	6	0	-3.881908	2.585117	1.919579
41	1	0	-3.877080	2.583832	3.010358
42	1	0	-3.691582	3.588071	1.533034
43	1	0	-4.870257	2.261038	1.573365
44	6	0	-5.325511	-0.712173	0.141637
45	6	0	-5.178545	-2.207931	0.007717
46	1	0	-5.825554	-2.677348	0.748016
47	1	0	-5.478125	-2.540501	-0.991787
48	1	0	-4.146734	-2.531503	0.174201
49	6	0	0.099478	-3.399052	-0.078002
50	6	0	0.104634	-4.021172	1.289314
51	1	0	1.000596	-3.697183	1.829563
52	1	0	-0.763657	-3.687241	1.864501
53	1	0	0.103748	-5.107311	1.200008
54	8	0	0.217167	-3.983977	-1.131076
55	8	0	-2.496019	0.608409	2.013676
56	8	0	-6.089609	-0.179625	0.917794
57	8	0	0.401666	2.970185	-1.760284

Structure 103α (M06-2X, Gas Phase)

Energy (Hartrees): = - 1622.1383002
No imaginary frequencies

Standard orientation:

Center Number	Atomic Number	Atomic Type	Coordinates (Angstroms) X	Y	Z
1	6	0	0.023801	-1.141512	-1.377114
2	6	0	0.573422	0.234021	-0.950545
3	6	0	-0.414885	0.893873	0.018556
4	6	0	-1.793832	0.930488	-0.638144
5	6	0	-2.206484	-0.522585	-0.908452
6	1	0	-0.464460	0.297251	0.929306
7	1	0	-1.727199	1.443517	-1.598130
8	1	0	-2.166969	-1.102534	0.013813
9	1	0	0.667976	0.843400	-1.855682
10	8	0	-1.281644	-1.060157	-1.850718
11	6	0	-3.576151	-0.653559	-1.556446
12	1	0	-3.657524	0.028404	-2.403619
13	1	0	-3.698403	-1.674887	-1.922065
14	8	0	-4.611740	-0.277630	-0.656700
15	8	0	-2.741320	1.702870	0.095024
16	8	0	0.026016	2.171375	0.484684
17	7	0	1.839664	0.040586	-0.286578
18	6	0	4.211464	0.294788	-0.196015
19	6	0	4.349033	-0.509959	0.956849
20	6	0	5.354262	0.849121	-0.786086
21	6	0	5.623523	-0.732900	1.484436
22	6	0	6.611512	0.627609	-0.258036
23	1	0	5.234672	1.459381	-1.675751
24	6	0	6.735025	-0.171026	0.882142
25	1	0	5.706013	-1.352902	2.368187
26	1	0	7.487473	1.061363	-0.721879
27	1	0	7.715580	-0.354294	1.306100
28	6	0	2.904177	0.520950	-0.797870
29	1	0	2.874803	1.116031	-1.719852
30	1	0	0.629730	-1.561736	-2.176866
31	8	0	0.059301	-2.018312	-0.253621
32	8	0	3.295895	-1.061487	1.571817
33	1	0	2.487423	-0.765135	1.085102
34	6	0	0.196825	3.182166	-0.386789
35	6	0	0.617977	4.431335	0.333277
36	1	0	1.529411	4.235301	0.899118
37	1	0	0.778463	5.225814	-0.390205
38	1	0	-0.159249	4.714999	1.043937
39	6	0	-3.201112	1.230813	1.274506
40	6	0	-4.329306	2.066946	1.790064
41	1	0	-4.401760	1.945791	2.868114
42	1	0	-4.207165	3.111000	1.509521
43	1	0	-5.239585	1.671249	1.330579
44	6	0	-5.121941	-1.141688	0.259286
45	6	0	-4.698091	-2.588904	0.195967
46	1	0	-5.161253	-3.097333	1.036503
47	1	0	-5.042854	-3.042673	-0.735802
48	1	0	-3.614743	-2.701018	0.250246
49	6	0	1.092388	-2.906072	-0.194200
50	6	0	1.092175	-3.647008	1.109625
51	1	0	1.723449	-3.086061	1.803679
52	1	0	0.089003	-3.725626	1.522787
53	1	0	1.537269	-4.627895	0.958094
54	8	0	1.896841	-3.034610	-1.069098

55	8	0	-2.775215	0.234722	1.795077
56	8	0	-5.910792	-0.717848	1.050329
57	8	0	0.035103	3.082717	-1.572951

Structure 103α (M06-2X, $CHCl_3$)

Energy (Hartrees): = - 1622.1707125
No imaginary frequencies

Standard orientation:

Center Number	Atomic Number	Atomic Type	Coordinates (Angstroms) X	Y	Z
1	6	0	0.015101	1.282165	-1.259584
2	6	0	-0.695740	-0.042223	-0.948869
3	6	0	0.188640	-0.862022	0.001946
4	6	0	1.559521	-1.031530	-0.653979
5	6	0	2.158326	0.367680	-0.849678
6	1	0	0.294418	-0.320349	0.941621
7	1	0	1.440848	-1.479628	-1.640486
8	1	0	2.201743	0.896890	0.103753
9	1	0	-0.806785	-0.575712	-1.898031
10	8	0	1.307198	1.072924	-1.751327
11	6	0	3.534432	0.353482	-1.495739
12	1	0	3.544833	-0.308845	-2.361926
13	1	0	3.789064	1.361455	-1.826527
14	8	0	4.506935	-0.172657	-0.592747
15	8	0	2.402567	-1.954449	0.035205
16	8	0	-0.408546	-2.097297	0.405474
17	7	0	-1.970737	0.225730	-0.327367
18	6	0	-4.328979	-0.114430	-0.189255
19	6	0	-4.488600	0.774065	0.897879
20	6	0	-5.453894	-0.758104	-0.721274
21	6	0	-5.766680	0.985948	1.423357
22	6	0	-6.715910	-0.543473	-0.197504
23	1	0	-5.314700	-1.434805	-1.558674
24	6	0	-6.861570	0.335413	0.879624
25	1	0	-5.871088	1.671046	2.256192
26	1	0	-7.578348	-1.045440	-0.617080
27	1	0	-7.844872	0.513239	1.300622
28	6	0	-3.015505	-0.349640	-0.780251
29	1	0	-2.969269	-1.037079	-1.633939
30	1	0	-0.517330	1.842177	-2.025907
31	8	0	0.052258	2.032508	-0.053288
32	8	0	-3.456328	1.426809	1.446143
33	1	0	-2.637861	1.155498	0.957801
34	6	0	-0.684944	-3.053053	-0.500620
35	6	0	-1.274043	-4.257840	0.167164
36	1	0	-2.177223	-3.969173	0.707970
37	1	0	-1.503684	-5.012852	-0.580537
38	1	0	-0.561787	-4.651576	0.894547
39	6	0	2.899448	-1.625208	1.244933
40	6	0	3.908808	-2.628687	1.700537
41	1	0	4.115015	-2.483106	2.758191
42	1	0	3.564079	-3.643175	1.502024
43	1	0	4.821437	-2.448717	1.126561
44	6	0	5.117590	0.599695	0.337377
45	6	0	4.916677	2.089858	0.284474
46	1	0	5.458874	2.527612	1.118410
47	1	0	5.310692	2.489118	-0.653004
48	1	0	3.862223	2.363580	0.353275
49	6	0	0.171936	3.381654	-0.170265
50	6	0	0.159501	4.031461	1.177431
51	1	0	-0.815685	3.865551	1.641263
52	1	0	0.915277	3.574226	1.818138
53	1	0	0.344430	5.097330	1.069370
54	8	0	0.257812	3.936164	-1.228700
55	8	0	2.581464	-0.628094	1.839319
56	8	0	5.822845	0.050250	1.137329
57	8	0	-0.482935	-2.933487	-1.680943

Structure 103β (B3LYP, Gas Phase)

Energy (Hartrees): = - 1622.3665917
No imaginary frequencies

Standard orientation:

Center Number	Atomic Number	Atomic Type	Coordinates (Angstroms) X	Y	Z
1	6	0	0.159070	1.324978	-0.327523
2	6	0	-0.592288	0.004664	-0.578659
3	6	0	0.289553	-1.139902	-0.037901
4	6	0	1.710221	-1.070029	-0.620640

5	6	0	2.309576	0.338901	-0.417200
6	1	0	0.278945	1.527748	0.743996
7	1	0	0.348448	-1.044160	1.046143
8	1	0	1.660865	-1.277217	-1.691035
9	1	0	2.448510	0.524124	0.654330
10	1	0	-0.715833	-0.097313	-1.663134
11	8	0	1.413420	1.300814	-0.975363
12	6	0	3.642796	0.478466	-1.146636
13	1	0	4.185018	-0.467431	-1.123883
14	1	0	3.456220	0.787257	-2.176931
15	8	0	4.462301	1.512100	-0.571351
16	8	0	2.584244	-2.103502	-0.116397
17	8	0	-0.309361	-2.445184	-0.206527
18	8	0	-0.587267	2.362438	-0.930617
19	7	0	-1.863379	0.012680	0.119164
20	6	0	-4.266082	0.011433	0.031060
21	6	0	-4.422570	0.078025	1.443512
22	6	0	-5.414221	-0.024387	-0.783703
23	6	0	-5.715142	0.108295	1.990896
24	6	0	-6.686982	0.005390	-0.235458
25	1	0	-5.283691	-0.075293	-1.862229
26	6	0	-6.826690	0.072459	1.159558
27	1	0	-5.813460	0.159803	3.069923
28	1	0	-7.562940	-0.022321	-0.874825
29	1	0	-7.819391	0.096754	1.600240
30	6	0	-2.947087	-0.016977	-0.575610
31	1	0	-2.912261	-0.065831	-1.672879
32	8	0	-3.371354	0.113252	2.273258
33	1	0	-2.548960	0.083027	1.712297
34	6	0	-0.569771	-2.933467	-1.442292
35	6	0	-1.178474	-4.310852	-1.340464
36	1	0	-1.396886	-4.684888	-2.339948
37	1	0	-0.485900	-4.986673	-0.830602
38	1	0	-2.094029	-4.272208	-0.743480
39	8	0	-0.346012	-2.343675	-2.479031
40	6	0	2.886847	-2.160936	1.212531
41	6	0	3.959805	-3.186438	1.463685
42	1	0	3.923546	-3.499205	2.507198
43	1	0	3.859165	-4.043750	0.795786
44	1	0	4.924753	-2.705818	1.271266
45	8	0	2.379785	-1.457222	2.057810
46	6	0	5.291224	1.126627	0.430638
47	6	0	6.065935	2.304096	0.965159
48	1	0	6.806413	1.955842	1.684151
49	1	0	6.551358	2.843272	0.147584
50	1	0	5.378051	3.001386	1.453324
51	8	0	5.378223	-0.014683	0.833198
52	6	0	-0.459270	3.615708	-0.384807
53	6	0	-1.272893	4.606916	-1.175104
54	1	0	-0.913169	4.641058	-2.207637
55	1	0	-2.320371	4.293507	-1.203357
56	1	0	-1.188228	5.591712	-0.717636
57	8	0	0.208038	3.856916	0.590137

Structure 103β (B3LYP, $CHCl_3$)

Energy (Hartrees): = - 1622.3954554
No imaginary frequencies

Standard orientation:

Center Number	Atomic Number	Atomic Type	Coordinates (Angstroms) X	Y	Z
1	6	0	0.215943	1.299161	-0.336737
2	6	0	-0.571882	0.003607	-0.603771
3	6	0	0.269546	-1.169612	-0.061137
4	6	0	1.701471	-1.138986	-0.618406
5	6	0	2.340754	0.244508	-0.372704
6	1	0	0.308902	1.499453	0.737151
7	1	0	0.312122	-1.078933	1.024130
8	1	0	1.671906	-1.318129	-1.694090
9	1	0	2.450515	0.419185	0.703604
10	1	0	-0.696848	-0.088850	-1.688459
11	8	0	1.486925	1.237842	-0.949694
12	6	0	3.693944	0.355577	-1.062217
13	1	0	4.272701	-0.556093	-0.910034
14	1	0	3.547476	0.529961	-2.130245
15	8	0	4.438593	1.490194	-0.580343
16	8	0	2.517524	-2.226760	-0.124236
17	8	0	-0.366731	-2.455500	-0.243571
18	8	0	-0.483005	2.356343	-0.966927
19	7	0	-1.843747	0.047012	0.092957
20	6	0	-4.241984	0.095163	0.036105
21	6	0	-4.372547	0.163872	1.450939
22	6	0	-5.405088	0.081386	-0.757778

23	6	0	-5.652388	0.215184	2.023996
24	6	0	-6.667531	0.132265	-0.184281
25	1	0	-5.294574	0.029387	-1.838223
26	6	0	-6.781108	0.199590	1.212798
27	1	0	-5.733624	0.266649	3.105041
28	1	0	-7.555917	0.120737	-0.807249
29	1	0	-7.764739	0.239608	1.672390
30	6	0	-2.932999	0.040468	-0.594221
31	1	0	-2.914823	-0.007344	-1.690432
32	8	0	-3.300392	0.180564	2.259758
33	1	0	-2.491257	0.133743	1.673694
34	6	0	-0.615875	-2.944521	-1.481851
35	6	0	-1.272340	-4.295704	-1.388900
36	1	0	-1.446378	-4.688256	-2.390561
37	1	0	-0.635593	-4.982733	-0.823531
38	1	0	-2.221984	-4.211743	-0.851456
39	8	0	-0.346800	-2.363500	-2.515002
40	6	0	2.771603	-2.365428	1.204608
41	6	0	3.695160	-3.528189	1.443894
42	1	0	3.696299	-3.779861	2.504531
43	1	0	3.400189	-4.393428	0.845396
44	1	0	4.709432	-3.242367	1.144069
45	8	0	2.324322	-1.635860	2.064930
46	6	0	5.231781	1.277208	0.498948
47	6	0	5.918545	2.547770	0.918908
48	1	0	6.603225	2.340879	1.741196
49	1	0	6.463224	2.982149	0.075577
50	1	0	5.170298	3.280396	1.238180
51	8	0	5.351165	0.195805	1.039762
52	6	0	-0.330830	3.611898	-0.440024
53	6	0	-1.110329	4.611873	-1.244989
54	1	0	-0.760022	4.608318	-2.281727
55	1	0	-2.169768	4.337696	-1.254684
56	1	0	-0.987571	5.605408	-0.814552
57	8	0	0.337144	3.846190	0.540386

Structure 103β (M06-2X, Gas Phase)

Energy (Hartrees): = - 1622.1443049
No imaginary frequencies

Standard orientation:

Center Number	Atomic Number	Atomic Type	Coordinates (Angstroms) X	Y	Z
1	6	0	0.258639	1.433979	-0.292043
2	6	0	-0.567515	0.183005	-0.574724
3	6	0	0.233417	-1.003660	-0.021874
4	6	0	1.627219	-1.032874	-0.651780
5	6	0	2.322821	0.323109	-0.452936
6	1	0	0.419083	1.578654	0.783438
7	1	0	0.325236	-0.877079	1.055732
8	1	0	1.525391	-1.218353	-1.722710
9	1	0	2.509832	0.486548	0.614120
10	1	0	-0.680938	0.103095	-1.660173
11	8	0	1.487327	1.349349	-0.965273
12	6	0	3.631064	0.372578	-1.227661
13	1	0	4.045216	-0.630240	-1.334875
14	1	0	3.445065	0.818957	-2.203087
15	8	0	4.589832	1.218001	-0.589401
16	8	0	2.436492	-2.115124	-0.187646
17	8	0	-0.449299	-2.255725	-0.140468
18	8	0	-0.430366	2.529913	-0.827933
19	7	0	-1.839520	0.251249	0.106591
20	6	0	-4.223030	-0.033852	0.021538
21	6	0	-4.428269	0.289359	1.380362
22	6	0	-5.325272	-0.360586	-0.779907
23	6	0	-5.728581	0.277073	1.894851
24	6	0	-6.607690	-0.371610	-0.265781
25	1	0	-5.151528	-0.605245	-1.823228
26	6	0	-6.798054	-0.048774	1.080827
27	1	0	-5.863128	0.528140	2.939304
28	1	0	-7.451020	-0.625680	-0.894409
29	1	0	-7.798369	-0.052932	1.497967
30	6	0	-2.887097	-0.034633	-0.562641
31	1	0	-2.818978	-0.300971	-1.625858
32	8	0	-3.425880	0.610895	2.201506
33	1	0	-2.587387	0.568182	1.690795
34	6	0	-0.823473	-2.712427	-1.348622
35	6	0	-1.570023	-4.008844	-1.210399
36	1	0	-1.755052	-4.424898	-2.197007
37	1	0	-0.995224	-4.702754	-0.597533
38	1	0	-2.515347	-3.818182	-0.698461
39	8	0	-0.603862	-2.142657	-2.383547
40	6	0	2.758290	-2.197977	1.128232

41	6	0	3.734691	-3.310278	1.362423
42	1	0	3.746534	-3.555737	2.421241
43	1	0	3.482316	-4.180135	0.758028
44	1	0	4.717870	-2.941992	1.063276
45	8	0	2.320577	-1.463251	1.967816
46	6	0	5.250574	0.662084	0.440661
47	6	0	6.200172	1.640214	1.068220
48	1	0	6.748841	1.148113	1.866467
49	1	0	6.883369	2.026170	0.311254
50	1	0	5.632101	2.483758	1.463174
51	8	0	5.078989	-0.474327	0.794774
52	6	0	-0.096401	3.753059	-0.325353
53	6	0	-0.866588	4.829799	-1.030941
54	1	0	-0.579250	4.841007	-2.083484
55	1	0	-1.933246	4.609906	-0.979258
56	1	0	-0.648901	5.789236	-0.570178
57	8	0	0.696788	3.905578	0.553251

Structure 103β (M06-2X, $CHCl_3$)

Energy (Hartrees): = - 1622.175583
No imaginary frequencies

Standard orientation:

Center Number	Atomic Number	Atomic Type	Coordinates (Angstroms) X	Y	Z
1	6	0	0.260076	1.422509	-0.324262
2	6	0	-0.570505	0.174485	-0.608045
3	6	0	0.222412	-1.017993	-0.056658
4	6	0	1.626669	-1.048746	-0.661916
5	6	0	2.323674	0.302131	-0.444889
6	1	0	0.401083	1.572775	0.752763
7	1	0	0.296919	-0.896083	1.022689
8	1	0	1.552934	-1.229579	-1.735346
9	1	0	2.483547	0.470455	0.625608
10	1	0	-0.694069	0.094055	-1.692116
11	8	0	1.499002	1.330530	-0.977888
12	6	0	3.648191	0.347956	-1.189269
13	1	0	4.079595	-0.649861	-1.268703
14	1	0	3.485674	0.764999	-2.182152
15	8	0	4.578648	1.222661	-0.545365
16	8	0	2.421660	-2.138698	-0.184069
17	8	0	-0.465550	-2.266110	-0.194054
18	8	0	-0.419873	2.516268	-0.882429
19	7	0	-1.837314	0.254164	0.083788
20	6	0	-4.217911	-0.026120	0.053744
21	6	0	-4.388220	0.326162	1.410211
22	6	0	-5.341469	-0.364475	-0.712338
23	6	0	-5.672948	0.330491	1.960398
24	6	0	-6.610932	-0.359846	-0.162933
25	1	0	-5.195069	-0.632029	-1.754127
26	6	0	-6.766296	-0.008460	1.180838
27	1	0	-5.783734	0.603574	3.002979
28	1	0	-7.471637	-0.623618	-0.764437
29	1	0	-7.755421	0.000138	1.624588
30	6	0	-2.895432	-0.042813	-0.564334
31	1	0	-2.852351	-0.326498	-1.623061
32	8	0	-3.357398	0.660230	2.196730
33	1	0	-2.534267	0.600094	1.656163
34	6	0	-0.807696	-2.728681	-1.411019
35	6	0	-1.561911	-4.017959	-1.292709
36	1	0	-1.732539	-4.432992	-2.283009
37	1	0	-1.001088	-4.719670	-0.674242
38	1	0	-2.516233	-3.825184	-0.797076
39	8	0	-0.549663	-2.160158	-2.439959
40	6	0	2.725222	-2.238052	1.131150
41	6	0	3.684307	-3.362129	1.366931
42	1	0	3.739061	-3.572576	2.432332
43	1	0	3.381370	-4.249070	0.810647
44	1	0	4.664789	-3.043150	1.006477
45	8	0	2.279078	-1.509279	1.975221
46	6	0	5.210239	0.708781	0.524782
47	6	0	6.119317	1.715380	1.158957
48	1	0	6.666161	1.249667	1.975047
49	1	0	6.809224	2.114085	0.413936
50	1	0	5.518735	2.545637	1.536276
51	8	0	5.036171	-0.421073	0.905271
52	6	0	-0.073016	3.748724	-0.420193
53	6	0	-0.858687	4.811330	-1.121192
54	1	0	-0.678652	4.744024	-2.195490
55	1	0	-1.923559	4.645468	-0.948320
56	1	0	-0.562920	5.788914	-0.749381
57	8	0	0.747067	3.914532	0.436282

Structure 105 (B3LYP, Gas Phase)

Energy (Hartrees): = - 1011.7052402
No imaginary frequencies

Standard orientation:

Center Number	Atomic Number	Atomic Type	Coordinates (Angstroms) X	Y	Z
1	6	0	1.292480	-1.377904	0.772467
2	6	0	0.573394	-0.025554	0.602863
3	6	0	1.314265	0.824422	-0.447595
4	6	0	2.785582	0.936036	-0.059082
5	6	0	3.407018	-0.456129	0.101102
6	1	0	1.243947	0.335518	-1.427397
7	1	0	2.844327	1.454164	0.912925
8	1	0	3.376294	-0.978054	-0.863075
9	1	0	0.628750	0.503173	1.561024
10	8	0	2.655292	-1.185690	1.087471
11	6	0	4.859254	-0.408773	0.584657
12	1	0	4.914396	0.204894	1.499687
13	1	0	5.167448	-1.423681	0.853929
14	8	0	5.738278	0.046427	-0.423141
15	1	0	5.367588	0.874639	-0.766337
16	8	0	3.515119	1.655142	-1.046623
17	1	0	3.026542	2.477320	-1.200833
18	8	0	0.805198	2.154640	-0.507211
19	1	0	-0.005521	2.145768	-1.032678
20	7	0	-0.822167	-0.210392	0.263898
21	6	0	-3.159885	0.294440	0.437264
22	6	0	-3.483984	-0.636979	-0.651166
23	6	0	-4.192665	1.031407	1.094720
24	6	0	-4.884743	-0.741072	-0.993098
25	6	0	-5.502375	0.888602	0.726769
26	1	0	-3.915402	1.711453	1.898253
27	6	0	-5.835963	-0.012081	-0.330982
28	1	0	-5.143714	-1.424238	-1.795526
29	1	0	-6.285155	1.448357	1.227462
30	1	0	-6.880490	-0.117991	-0.614897
31	6	0	-1.826628	0.454628	0.824879
32	1	0	-1.571016	1.151074	1.624187
33	1	0	0.881572	-1.922557	1.631319
34	8	0	1.098321	-2.085156	-0.427201
35	1	0	1.417053	-2.989876	-0.306556
36	8	0	-2.592296	-1.303060	-1.250395
37	1	0	-1.145254	-0.861901	-0.488757

Structure 105 (B3LYP, DMSO)

Energy (Hartrees): = - 1011.7357133
No imaginary frequencies

Standard orientation:

Center Number	Atomic Number	Atomic Type	Coordinates (Angstroms) X	Y	Z
1	6	0	1.271088	-1.367769	0.773794
2	6	0	0.579303	-0.000978	0.607658
3	6	0	1.327742	0.852235	-0.435289
4	6	0	2.805211	0.924312	-0.061171
5	6	0	3.399170	-0.479874	0.094523
6	1	0	1.236125	0.387530	-1.424665
7	1	0	2.891912	1.447111	0.904219
8	1	0	3.356781	-1.000826	-0.870097
9	1	0	0.621982	0.515843	1.570613
10	8	0	2.642660	-1.200276	1.081650
11	6	0	4.849858	-0.457236	0.581065
12	1	0	4.906162	0.120951	1.517849
13	1	0	5.160958	-1.482211	0.807685
14	8	0	5.737312	0.046770	-0.406178
15	1	0	5.335482	0.869140	-0.732905
16	8	0	3.543032	1.620206	-1.066989
17	1	0	3.102525	2.476634	-1.185171
18	8	0	0.847428	2.194350	-0.465814
19	1	0	0.060343	2.219298	-1.029663
20	7	0	-0.817082	-0.173279	0.249710
21	6	0	-3.164407	0.278169	0.432427
22	6	0	-3.487713	-0.612891	-0.682896
23	6	0	-4.192440	0.983675	1.122348
24	6	0	-4.888941	-0.711145	-1.016342
25	6	0	-5.510813	0.850093	0.761356
26	1	0	-3.907206	1.635129	1.945995
27	6	0	-5.845867	-0.010790	-0.322037
28	1	0	-5.158441	-1.364922	-1.841260

29	1	0	-6.290957	1.389172	1.288963
30	1	0	-6.891048	-0.114358	-0.605739
31	6	0	-1.825371	0.445783	0.834367
32	1	0	-1.587514	1.108692	1.665058
33	1	0	0.854737	-1.899540	1.636391
34	8	0	1.062617	-2.086234	-0.418276
35	1	0	1.342701	-3.002311	-0.266367
36	8	0	-2.595684	-1.257362	-1.316571
37	1	0	-1.114800	-0.793506	-0.530275

Structure 105 (M06-2X, Gas Phase)

Energy (Hartrees): = - 1011.5703323
No imaginary frequencies

Standard orientation:

Center Number	Atomic Number	Atomic Type	Coordinates (Angstroms) X	Y	Z
1	6	0	1.361212	-1.413455	0.795605
2	6	0	0.592807	-0.097302	0.665983
3	6	0	1.256102	0.777529	-0.401556
4	6	0	2.717590	0.958529	-0.041650
5	6	0	3.393691	-0.404605	0.069967
6	1	0	1.187657	0.281274	-1.376231
7	1	0	2.775207	1.459106	0.937349
8	1	0	3.346465	-0.907672	-0.902402
9	1	0	0.650033	0.431853	1.622000
10	8	0	2.716250	-1.174637	1.063687
11	6	0	4.852439	-0.301567	0.494299
12	1	0	4.924046	0.346451	1.379402
13	1	0	5.196792	-1.297128	0.778002
14	8	0	5.670105	0.146233	-0.558786
15	1	0	5.278127	0.957538	-0.899746
16	8	0	3.390655	1.719376	-1.023719
17	1	0	2.869362	2.516439	-1.168116
18	8	0	0.672842	2.067500	-0.444991
19	1	0	-0.135503	2.026864	-0.963724
20	7	0	-0.795784	-0.343210	0.354862
21	6	0	-3.111871	0.289629	0.406361
22	6	0	-3.477956	-0.732613	-0.575379
23	6	0	-4.101187	1.159256	0.962756
24	6	0	-4.884474	-0.777801	-0.929063
25	6	0	-5.403898	1.063327	0.593794
26	1	0	-3.784711	1.902136	1.689636
27	6	0	-5.783117	0.072728	-0.368962
28	1	0	-5.176864	-1.525697	-1.655912
29	1	0	-6.152706	1.721706	1.013550
30	1	0	-6.827066	0.004589	-0.657483
31	6	0	-1.784830	0.419212	0.795137
32	1	0	-1.506823	1.195759	1.507040
33	1	0	0.994668	-1.991021	1.648246
34	8	0	1.160897	-2.093866	-0.410596
35	1	0	1.486320	-2.994208	-0.323735
36	8	0	-2.641029	-1.507393	-1.073249
37	1	0	-1.071339	-1.060429	-0.334418

Structure 105 (M06-2X, DMSO)

Energy (Hartrees): = - 1011.6040116
No imaginary frequencies

Standard orientation:

Center Number	Atomic Number	Atomic Type	Coordinates (Angstroms) X	Y	Z
1	6	0	-1.294686	1.358278	0.806444
2	6	0	-0.589639	0.009416	0.639248
3	6	0	-1.306817	-0.830249	-0.420997
4	6	0	-2.772607	-0.937781	-0.047150
5	6	0	-3.379684	0.454406	0.093780
6	1	0	-1.223922	-0.342045	-1.398742
7	1	0	-2.851439	-1.454795	0.920099
8	1	0	-3.320071	0.973302	-0.869806
9	1	0	-0.622176	-0.524915	1.591578
10	8	0	-2.657655	1.175086	1.091363
11	6	0	-4.832749	0.414720	0.543457
12	1	0	-4.913201	-0.211639	1.442415
13	1	0	-5.142093	1.428107	0.806321
14	8	0	-5.689390	-0.036381	-0.486506
15	1	0	-5.302768	-0.851728	-0.827406
16	8	0	-3.492622	-1.643991	-1.043710
17	1	0	-3.028605	-2.477415	-1.190422

18	8	0	-0.793363	-2.148789	-0.472032
19	1	0	-0.002313	-2.151779	-1.022625
20	7	0	0.797138	0.214699	0.276509
21	6	0	3.140075	-0.270058	0.411740
22	6	0	3.472772	0.649072	-0.667465
23	6	0	4.150458	-1.017892	1.078137
24	6	0	4.878465	0.725990	-0.997914
25	6	0	5.464572	-0.901138	0.725783
26	1	0	3.847499	-1.687268	1.878524
27	6	0	5.813767	-0.011854	-0.330618
28	1	0	5.159328	1.398128	-1.801287
29	1	0	6.233296	-1.469271	1.233850
30	1	0	6.858422	0.079413	-0.612288
31	6	0	1.803652	-0.431851	0.812699
32	1	0	1.564862	-1.127240	1.615403
33	1	0	-0.887706	1.896638	1.665216
34	8	0	-1.088516	2.065759	-0.384079
35	1	0	-1.360950	2.980822	-0.246141
36	8	0	2.606368	1.326552	-1.270253
37	1	0	1.047822	0.866542	-0.481397

Structure 106 (B3LYP, Gas Phase)

Energy (Hartrees): = - 1011.7028997
No imaginary frequencies

Standard orientation:

Center Number	Atomic Number	Atomic Type	Coordinates (Angstroms) X	Y	Z
1	6	0	1.211799	1.414862	0.430135
2	6	0	0.586344	0.307295	-0.440039
3	6	0	1.382849	-0.993917	-0.261406
4	6	0	2.869273	-0.744960	-0.494964
5	6	0	3.367283	0.393810	0.404948
6	1	0	1.107252	1.154930	1.500269
7	1	0	1.258543	-1.345212	0.776239
8	1	0	3.006586	-0.437042	-1.544013
9	1	0	3.250785	0.098206	1.460107
10	1	0	0.669079	0.642579	-1.480640
11	8	0	2.589069	1.559888	0.119216
12	6	0	4.839636	0.743088	0.166988
13	1	0	4.992629	0.935134	-0.908100
14	1	0	5.065503	1.671371	0.700483
15	8	0	5.706581	-0.253442	0.666812
16	1	0	5.404913	-1.099247	0.299605
17	8	0	3.620772	-1.920064	-0.217453
18	1	0	3.198085	-2.638587	-0.711320
19	8	0	0.990972	-2.010136	-1.174625
20	1	0	0.111417	-2.327567	-0.925794
21	8	0	0.562980	2.606081	0.122991
22	1	0	0.934667	3.302877	0.681434
23	7	0	-0.804910	0.053702	-0.116368
24	6	0	-3.157080	0.381863	-0.227687
25	6	0	-3.381177	-0.843433	0.543206
26	6	0	-4.259790	1.165205	-0.679356
27	6	0	-4.755553	-1.185214	0.801718
28	6	0	-5.548528	0.794787	-0.398732
29	1	0	-4.055624	2.068258	-1.251231
30	6	0	-5.782152	-0.394939	0.350664
31	1	0	-4.939652	-2.090303	1.371339
32	1	0	-6.386177	1.393020	-0.740949
33	1	0	-6.808135	-0.682012	0.568807
34	6	0	-1.842630	0.757658	-0.543406
35	1	0	-1.642861	1.638210	-1.151818
36	8	0	-2.417314	-1.567248	0.955562
37	1	0	-1.138765	-0.757792	0.477722

Structure 106 (B3LYP, DMSO)

Energy (Hartrees): = - 1011.73331
No imaginary frequencies

Standard orientation:

Center Number	Atomic Number	Atomic Type	Coordinates (Angstroms) X	Y	Z
1	6	0	-1.206416	1.425107	-0.279663
2	6	0	-0.595715	0.212218	0.450741
3	6	0	-1.414089	-1.052707	0.133896
4	6	0	-2.896015	-0.806901	0.400056
5	6	0	-3.379714	0.440948	-0.350411
6	1	0	-1.126357	1.284379	-1.371275

7	1	0	-1.291351	-1.295358	-0.932078
8	1	0	-3.032602	-0.633523	1.478550
9	1	0	-3.272304	0.277323	-1.434214
10	1	0	-0.638317	0.415393	1.525626
11	8	0	-2.582922	1.551848	0.069740
12	6	0	-4.838991	0.788093	-0.046349
13	1	0	-4.960101	0.901026	1.043330
14	1	0	-5.073301	1.754091	-0.505178
15	8	0	-5.741308	-0.166965	-0.584106
16	1	0	-5.399634	-1.037452	-0.319484
17	8	0	-3.671182	-1.929371	-0.022681
18	1	0	-3.300868	-2.705112	0.427108
19	8	0	-1.034200	-2.160575	0.943814
20	1	0	-0.188777	-2.498649	0.612533
21	8	0	-0.538291	2.565991	0.143101
22	1	0	-0.822662	3.301722	-0.421152
23	7	0	0.793454	0.020644	0.073546
24	6	0	3.160403	0.069880	0.427238
25	6	0	3.435573	-0.297691	-0.961954
26	6	0	4.225952	0.287692	1.347496
27	6	0	4.830240	-0.413495	-1.312521
28	6	0	5.537026	0.161519	0.957695
29	1	0	3.975902	0.558759	2.371175
30	6	0	5.825265	-0.193373	-0.390276
31	1	0	5.063989	-0.683633	-2.338600
32	1	0	6.346315	0.328922	1.660950
33	1	0	6.864623	-0.291945	-0.696126
34	6	0	1.829704	0.217090	0.864984
35	1	0	1.623110	0.500438	1.896099
36	8	0	2.507734	-0.501090	-1.807359
37	1	0	1.080169	-0.229883	-0.897747

Structure 106 (M06-2X, Gas Phase)

Energy (Hartrees): = - 1011.5671285
No imaginary frequencies

Standard orientation:

Center Number	Atomic Number	Atomic Type	Coordinates (Angstroms) X	Y	Z
1	6	0	-1.182072	1.383490	-0.405020
2	6	0	-0.592637	0.268505	0.461400
3	6	0	-1.400316	-1.010756	0.270545
4	6	0	-2.871976	-0.728357	0.501906
5	6	0	-3.332443	0.413830	-0.400181
6	1	0	-1.077121	1.123835	-1.472335
7	1	0	-1.279287	-1.362840	-0.765487
8	1	0	-3.004270	-0.416956	1.548392
9	1	0	-3.203222	0.117043	-1.452001
10	1	0	-0.673853	0.598243	1.502681
11	8	0	-2.546468	1.559232	-0.102880
12	6	0	-4.796315	0.776307	-0.186259
13	1	0	-4.977535	0.913389	0.888928
14	1	0	-4.988959	1.727522	-0.684510
15	8	0	-5.654938	-0.181798	-0.753236
16	1	0	-5.395411	-1.041234	-0.403062
17	8	0	-3.648391	-1.874802	0.222322
18	1	0	-3.256269	-2.608254	0.709089
19	8	0	-1.031056	-2.025698	1.179808
20	1	0	-0.149438	-2.342826	0.959105
21	8	0	-0.502581	2.547801	-0.091257
22	1	0	-0.853037	3.259896	-0.634446
23	7	0	0.790553	-0.001289	0.138070
24	6	0	3.131457	0.365534	0.223847
25	6	0	3.367840	-0.853545	-0.538310
26	6	0	4.217778	1.175073	0.663807
27	6	0	4.749746	-1.163688	-0.807902
28	6	0	5.503896	0.835588	0.376783
29	1	0	3.994350	2.073451	1.231754
30	6	0	5.753942	-0.353572	-0.370541
31	1	0	4.946328	-2.066082	-1.373441
32	1	0	6.330635	1.450339	0.706727
33	1	0	6.781774	-0.618591	-0.596400
34	6	0	1.817437	0.717045	0.544862
35	1	0	1.602043	1.600783	1.142282
36	8	0	2.428703	-1.592234	-0.933485
37	1	0	1.109224	-0.811421	-0.453550

Structure 106 (M06-2X, DMSO)

Energy (Hartrees): = - 1011.6007392
No imaginary frequencies

Standard orientation:

Center Number	Atomic Number	Atomic Type	Coordinates (Angstroms) X	Y	Z
1	6	0	-1.213243	1.379056	-0.415534
2	6	0	-0.605793	0.244298	0.413761
3	6	0	-1.417907	-1.033199	0.212527
4	6	0	-2.884713	-0.758663	0.482414
5	6	0	-3.367661	0.414530	-0.366689
6	1	0	-1.154349	1.133396	-1.487248
7	1	0	-1.314028	-1.362485	-0.830791
8	1	0	-3.002871	-0.492388	1.542144
9	1	0	-3.267990	0.155612	-1.430863
10	1	0	-0.636567	0.542012	1.465748
11	8	0	-2.571088	1.548480	-0.055366
12	6	0	-4.817047	0.781721	-0.082543
13	1	0	-4.942577	0.930432	0.998689
14	1	0	-5.040704	1.725607	-0.583154
15	8	0	-5.713926	-0.188058	-0.585123
16	1	0	-5.399284	-1.043709	-0.269316
17	8	0	-3.666978	-1.899367	0.169846
18	1	0	-3.289817	-2.644675	0.653195
19	8	0	-1.026655	-2.062098	1.100626
20	1	0	-0.173418	-2.407411	0.813766
21	8	0	-0.525891	2.538197	-0.114520
22	1	0	-0.796825	3.220065	-0.740517
23	7	0	0.774789	0.009659	0.041609
24	6	0	3.141559	0.173062	0.369566
25	6	0	3.439664	-0.502998	-0.884650
26	6	0	4.183327	0.614342	1.232142
27	6	0	4.845585	-0.679059	-1.170077
28	6	0	5.496275	0.421362	0.910019
29	1	0	3.906127	1.112098	2.157208
30	6	0	5.812141	-0.237372	-0.312587
31	1	0	5.101055	-1.181441	-2.096489
32	1	0	6.288419	0.758092	1.566392
33	1	0	6.856093	-0.393028	-0.567190
34	6	0	1.807952	0.385388	0.755174
35	1	0	1.593943	0.880696	1.700386
36	8	0	2.543566	-0.909354	-1.663959
37	1	0	1.016198	-0.437697	-0.856400

Structure 107 (B3LYP, Gas Phase)

Energy (Hartrees): = - 1086.9283733
No imaginary frequencies

Standard orientation:

Center Number	Atomic Number	Atomic Type	Coordinates (Angstroms) X	Y	Z
1	6	0	1.668135	-1.373838	0.781225
2	6	0	0.949941	-0.020415	0.615919
3	6	0	1.677491	0.821104	-0.450282
4	6	0	3.154573	0.931714	-0.084795
5	6	0	3.775122	-0.460952	0.074849
6	1	0	1.591204	0.326015	-1.425763
7	1	0	3.229339	1.455967	0.882853
8	1	0	3.729444	-0.988150	-0.885862
9	1	0	1.022896	0.512712	1.570627
10	8	0	3.036190	-1.183373	1.075892
11	6	0	5.234025	-0.413838	0.537845
12	1	0	5.302572	0.204125	1.449125
13	1	0	5.544291	-1.427950	0.807802
14	8	0	6.100047	0.035268	-0.483903
15	1	0	5.724259	0.860796	-0.828085
16	8	0	3.871220	1.642860	-1.087694
17	1	0	3.379921	2.463216	-1.242920
18	8	0	1.170929	2.152280	-0.511751
19	1	0	0.346055	2.138218	-1.014708
20	7	0	-0.451099	-0.201006	0.300654
21	6	0	-2.783034	0.303295	0.521732
22	6	0	-3.136906	-0.616818	-0.574880
23	6	0	-3.802617	1.030872	1.203472
24	6	0	-4.533280	-0.717963	-0.894169
25	6	0	-5.118130	0.896973	0.861294
26	1	0	-3.517251	1.702685	2.010428
27	6	0	-5.475161	0.006465	-0.204406
28	1	0	-4.832146	-1.384136	-1.694910
29	1	0	-5.893244	1.452335	1.383175
30	6	0	-1.445972	0.458757	0.886945
31	1	0	-1.175506	1.146290	1.689206
32	1	0	1.268252	-1.913328	1.648536
33	8	0	1.455803	-2.086549	-0.411891

34	1	0	1.772058	-2.992018	-0.290522
35	8	0	-2.251092	-1.273118	-1.193635
36	1	0	-0.791879	-0.843922	-0.452410
37	8	0	-6.782895	-0.137746	-0.557770
38	1	0	-7.329310	0.425070	0.006359

Structure 107 (B3LYP, DMSO)

Energy (Hartrees): = - 1086.9638356
No imaginary frequencies

Standard orientation:

Center Number	Atomic Number	Atomic Type	Coordinates (Angstroms) X	Y	Z
1	6	0	1.653775	-1.364967	0.787863
2	6	0	0.957832	-0.000013	0.624735
3	6	0	1.689312	0.847349	-0.434872
4	6	0	3.172715	0.922128	-0.086683
5	6	0	3.769460	-0.481098	0.065742
6	1	0	1.580815	0.377049	-1.420043
7	1	0	3.275531	1.449316	0.874760
8	1	0	3.709278	-1.007468	-0.894993
9	1	0	1.017575	0.522498	1.583902
10	8	0	3.031039	-1.195744	1.070385
11	6	0	5.228510	-0.456039	0.526263
12	1	0	5.300825	0.126290	1.459382
13	1	0	5.543785	-1.479922	0.752053
14	8	0	6.098829	0.043750	-0.478337
15	1	0	5.690786	0.864279	-0.802016
16	8	0	3.893296	1.613982	-1.107927
17	1	0	3.448612	2.468515	-1.224076
18	8	0	1.208936	2.189611	-0.465626
19	1	0	0.406093	2.208796	-1.006990
20	7	0	-0.444058	-0.174597	0.292022
21	6	0	-2.786514	0.286868	0.511185
22	6	0	-3.143032	-0.605867	-0.599475
23	6	0	-3.800762	0.996935	1.213635
24	6	0	-4.541051	-0.698050	-0.910182
25	6	0	-5.124677	0.875694	0.881178
26	1	0	-3.505178	1.649527	2.032067
27	6	0	-5.485701	0.012529	-0.198503
28	1	0	-4.848759	-1.345640	-1.725013
29	1	0	-5.895272	1.420176	1.420118
30	6	0	-1.444876	0.450324	0.889204
31	1	0	-1.191845	1.115970	1.713197
32	1	0	1.253296	-1.891081	1.661394
33	8	0	1.425241	-2.092259	-0.395319
34	1	0	1.710008	-3.006602	-0.241846
35	8	0	-2.262303	-1.254736	-1.244216
36	1	0	-0.753784	-0.796165	-0.480166
37	8	0	-6.790572	-0.128822	-0.552854
38	1	0	-7.339039	0.425615	0.024731

Structure 107 (M06-2X, Gas Phase)

Energy (Hartrees): = - 1086.7947323
No imaginary frequencies

Standard orientation:

Center Number	Atomic Number	Atomic Type	Coordinates (Angstroms) X	Y	Z
1	6	0	1.759010	-1.428729	0.797971
2	6	0	0.971458	-0.121535	0.697801
3	6	0	1.595626	0.766659	-0.382215
4	6	0	3.063423	0.965579	-0.060407
5	6	0	3.759484	-0.389409	0.025189
6	1	0	1.507790	0.273642	-1.356885
7	1	0	3.140776	1.461676	0.919502
8	1	0	3.693013	-0.886667	-0.949059
9	1	0	1.049491	0.402162	1.655516
10	8	0	3.118243	-1.174152	1.031199
11	6	0	5.227195	-0.270077	0.412531
12	1	0	5.312474	0.370654	1.301808
13	1	0	5.592543	-1.263294	0.677654
14	8	0	6.011139	0.199103	-0.656771
15	1	0	5.598044	1.006772	-0.980978
16	8	0	3.700065	1.740520	-1.055756
17	1	0	3.161678	2.528643	-1.185515
18	8	0	0.993806	2.048985	-0.405307
19	1	0	0.174706	1.998847	-0.905976

20	7	0	-0.422079	-0.380078	0.422994
21	6	0	-2.733268	0.263856	0.506680
22	6	0	-3.127262	-0.746679	-0.486261
23	6	0	-3.707944	1.133164	1.080100
24	6	0	-4.525487	-0.777330	-0.828229
25	6	0	-5.014137	1.056091	0.725234
26	1	0	-3.384336	1.867296	1.812028
27	6	0	-5.414140	0.077736	-0.248018
28	1	0	-4.861635	-1.503024	-1.556749
29	1	0	-5.756089	1.717718	1.158245
30	6	0	-1.403600	0.384609	0.879039
31	1	0	-1.112516	1.156854	1.590414
32	1	0	1.423445	-2.016312	1.656385
33	8	0	1.535014	-2.103796	-0.406726
34	1	0	1.872225	-3.001070	-0.334446
35	8	0	-2.294117	-1.518805	-0.994825
36	1	0	-0.710757	-1.089687	-0.268992
37	8	0	-6.716763	-0.003649	-0.610308
38	1	0	-7.227551	0.647082	-0.122452

Structure 107 (M06-2X, DMSO)

Energy (Hartrees): = - 1086.8336196
No imaginary frequencies

Standard orientation:

Center Number	Atomic Number	Atomic Type	Coordinates (Angstroms) X	Y	Z
1	6	0	-1.681520	1.358602	0.820014
2	6	0	-0.967586	0.014063	0.658243
3	6	0	-1.662956	-0.823323	-0.418511
4	6	0	-3.134203	-0.939559	-0.070624
5	6	0	-3.748971	0.449842	0.062942
6	1	0	-1.565449	-0.329507	-1.392119
7	1	0	-3.227492	-1.458567	0.894278
8	1	0	-3.671874	0.972363	-0.897476
9	1	0	-1.016319	-0.525279	1.607198
10	8	0	-3.049533	1.169332	1.077109
11	6	0	-5.210571	0.402801	0.483145
12	1	0	-5.307141	-0.231356	1.375051
13	1	0	-5.528146	1.413098	0.748045
14	8	0	-6.045284	-0.042298	-0.567298
15	1	0	-5.650248	-0.854651	-0.905561
16	8	0	-3.834479	-1.646948	-1.080413
17	1	0	-3.362678	-2.476653	-1.223133
18	8	0	-1.142425	-2.139344	-0.468353
19	1	0	-0.335455	-2.132545	-0.995296
20	7	0	0.424626	0.227422	0.323491
21	6	0	2.762075	-0.279527	0.482573
22	6	0	3.132662	0.665486	-0.569814
23	6	0	3.754568	-1.052219	1.143245
24	6	0	4.534874	0.740097	-0.872601
25	6	0	5.074629	-0.944909	0.822070
26	1	0	3.437737	-1.741224	1.920674
27	6	0	5.453811	-0.028381	-0.205816
28	1	0	4.861166	1.424320	-1.646937
29	1	0	5.833131	-1.533756	1.325776
30	6	0	1.423023	-0.439532	0.856688
31	1	0	1.167850	-1.153292	1.638013
32	1	0	-1.293997	1.893494	1.689856
33	8	0	-1.455363	2.074714	-0.361925
34	1	0	-1.733545	2.987854	-0.222890
35	8	0	2.279263	1.363186	-1.167565
36	1	0	0.686225	0.894963	-0.414645
37	8	0	6.756178	0.098820	-0.544939
38	1	0	7.287993	-0.501559	-0.007526

Structure 108 (B3LYP, Gas Phase)

Energy (Hartrees): = - 1086.9262752
No imaginary frequencies

Standard orientation:

Center Number	Atomic Number	Atomic Type	Coordinates (Angstroms) X	Y	Z
1	6	0	-1.627430	1.438054	-0.439179
2	6	0	-0.961396	0.354432	0.429362
3	6	0	-1.707317	-0.975835	0.242768
4	6	0	-3.201800	-0.786074	0.478275
5	6	0	-3.743474	0.336431	-0.417026
6	1	0	-1.512006	1.184026	-1.509579

7	1	0	-1.569587	-1.313915	-0.797541
8	1	0	-3.350940	-0.488772	1.528844
9	1	0	-3.617098	0.048347	-1.473147
10	1	0	-1.062240	0.682161	1.471006
11	8	0	-3.009732	1.530524	-0.129056
12	6	0	-5.227381	0.629498	-0.175762
13	1	0	-5.385056	0.813553	0.900063
14	1	0	-5.489278	1.549615	-0.706968
15	8	0	-6.057442	-0.398085	-0.675567
16	1	0	-5.721773	-1.232993	-0.312812
17	8	0	-3.907384	-1.988117	0.195143
18	1	0	-3.452173	-2.692710	0.680223
19	8	0	-1.274408	-1.982812	1.147582
20	1	0	-0.385875	-2.269640	0.891975
21	8	0	-1.026147	2.654583	-0.131023
22	1	0	-1.431812	3.336755	-0.683940
23	7	0	0.437248	0.146253	0.108174
24	6	0	2.777691	0.515327	0.248559
25	6	0	3.044190	-0.768063	-0.418917
26	6	0	3.859905	1.342632	0.660618
27	6	0	4.417578	-1.112958	-0.616644
28	6	0	5.158997	0.974069	0.442325
29	1	0	3.638354	2.285902	1.154911
30	6	0	5.427832	-0.272380	-0.205665
31	1	0	4.651585	-2.051807	-1.104498
32	1	0	5.983110	1.609480	0.756150
33	6	0	1.455371	0.892733	0.512638
34	1	0	1.230257	1.806612	1.059889
35	8	0	2.094811	-1.532240	-0.792919
36	1	0	0.802047	-0.699784	-0.415420
37	8	0	6.712865	-0.660882	-0.432179
38	1	0	7.314652	0.018435	-0.100333

Structure 108 (B3LYP, DMSO)

Energy (Hartrees): = - 1086.9615166
No imaginary frequencies

Standard orientation:

Center Number	Atomic Number	Atomic Type	Coordinates (Angstroms) X	Y	Z
1	6	0	-1.577319	1.396841	-0.384739
2	6	0	-0.972673	0.247114	0.445594
3	6	0	-1.780763	-1.041117	0.210937
4	6	0	-3.268291	-0.786314	0.432384
5	6	0	-3.746462	0.403316	-0.410042
6	1	0	-1.484130	1.172927	-1.461285
7	1	0	-1.639192	-1.361511	-0.832124
8	1	0	-3.425033	-0.541209	1.494045
9	1	0	-3.625918	0.162927	-1.478059
10	1	0	-1.038404	0.531662	1.500772
11	8	0	-2.959081	1.544793	-0.061799
12	6	0	-5.211118	0.762866	-0.149029
13	1	0	-5.348724	0.944558	0.929383
14	1	0	-5.443192	1.696459	-0.671772
15	8	0	-6.101750	-0.229085	-0.637420
16	1	0	-5.761804	-1.078722	-0.310156
17	8	0	-4.030896	-1.939528	0.073396
18	1	0	-3.668012	-2.680019	0.584346
19	8	0	-1.410771	-2.084823	1.106515
20	1	0	-0.548882	-2.427467	0.826311
21	8	0	-0.918533	2.570290	-0.044414
22	1	0	-1.197697	3.257963	-0.668626
23	7	0	0.423909	0.029604	0.114089
24	6	0	2.783184	0.184245	0.489300
25	6	0	3.101334	-0.395658	-0.821751
26	6	0	3.828569	0.567537	1.375996
27	6	0	4.495385	-0.537599	-1.127307
28	6	0	5.148082	0.412271	1.040332
29	1	0	3.560953	0.993521	2.340266
30	6	0	5.471880	-0.149911	-0.232294
31	1	0	4.775000	-0.961578	-2.086379
32	1	0	5.942993	0.707154	1.719871
33	6	0	1.447396	0.367931	0.878559
34	1	0	1.218902	0.801117	1.851262
35	8	0	2.190691	-0.751465	-1.634619
36	1	0	0.728088	-0.356302	-0.804286
37	8	0	6.771319	-0.316639	-0.594658
38	1	0	7.344645	-0.006178	0.124171

Structure 108 (M06-2X, Gas Phase)

Energy (Hartrees): = - 1086.7917769
No imaginary frequencies

Standard orientation:

Center Number	Atomic Number	Atomic Type	Coordinates (Angstroms) X	Y	Z
1	6	0	-1.560076	1.379023	-0.418998
2	6	0	-0.967160	0.286769	0.473493
3	6	0	-1.752012	-1.007626	0.288995
4	6	0	-3.231416	-0.745168	0.491852
5	6	0	-3.694758	0.375156	-0.435719
6	1	0	-1.432273	1.105991	-1.480450
7	1	0	-1.608075	-1.373401	-0.739322
8	1	0	-3.385854	-0.419497	1.530931
9	1	0	-3.542809	0.064151	-1.480366
10	1	0	-1.072171	0.630459	1.508226
11	8	0	-2.932362	1.537717	-0.143315
12	6	0	-5.167512	0.717098	-0.251598
13	1	0	-5.368090	0.868805	0.818174
14	1	0	-5.367699	1.656595	-0.768738
15	8	0	-6.001536	-0.264229	-0.815870
16	1	0	-5.733246	-1.113527	-0.447972
17	8	0	-3.985130	-1.908313	0.218397
18	1	0	-3.587261	-2.627888	0.720932
19	8	0	-1.382449	-2.002871	1.219685
20	1	0	-0.490438	-2.305462	1.021128
21	8	0	-0.905549	2.559545	-0.111073
22	1	0	-1.261674	3.257747	-0.668457
23	7	0	0.424621	0.034585	0.177858
24	6	0	2.755686	0.446364	0.300604
25	6	0	3.039521	-0.776694	-0.452670
26	6	0	3.817268	1.276697	0.751999
27	6	0	4.420326	-1.060045	-0.700519
28	6	0	5.114008	0.966184	0.489762
29	1	0	3.573599	2.174163	1.312195
30	6	0	5.403832	-0.224281	-0.250380
31	1	0	4.674037	-1.952453	-1.256746
32	1	0	5.925527	1.599281	0.830547
33	6	0	1.433776	0.775161	0.594872
34	1	0	1.191966	1.660918	1.178973
35	8	0	2.117349	-1.532564	-0.853324
36	1	0	0.767202	-0.774023	-0.400641
37	8	0	6.690023	-0.547612	-0.522002
38	1	0	7.273249	0.120833	-0.153562

Structure 108 (M06-2X, DMSO)

Energy (Hartrees): = - 1086.8303944
No imaginary frequencies

Standard orientation:

Center Number	Atomic Number	Atomic Type	Coordinates (Angstroms) X	Y	Z
1	6	0	-1.582606	1.373973	-0.420687
2	6	0	-0.981006	0.251491	0.429261
3	6	0	-1.783590	-1.032821	0.233303
4	6	0	-3.256026	-0.763555	0.474760
5	6	0	-3.730008	0.393010	-0.401430
6	1	0	-1.501101	1.119766	-1.488985
7	1	0	-1.660447	-1.376904	-0.803196
8	1	0	-3.393647	-0.480487	1.527781
9	1	0	-3.605094	0.119734	-1.459454
10	1	0	-1.028653	0.560796	1.477419
11	8	0	-2.948746	1.537160	-0.089104
12	6	0	-5.187856	0.752628	-0.153557
13	1	0	-5.337277	0.915838	0.922539
14	1	0	-5.408267	1.687473	-0.672246
15	8	0	-6.066770	-0.231270	-0.660548
16	1	0	-5.752486	-1.079653	-0.325259
17	8	0	-4.027013	-1.913718	0.168317
18	1	0	-3.653155	-2.648924	0.669266
19	8	0	-1.400889	-2.046710	1.142229
20	1	0	-0.533850	-2.378802	0.882689
21	8	0	-0.910179	2.541451	-0.116889
22	1	0	-1.171620	3.214289	-0.756459
23	7	0	0.405168	0.019619	0.078996
24	6	0	2.765679	0.221108	0.436090
25	6	0	3.104541	-0.462717	-0.810875
26	6	0	3.787991	0.682964	1.308113
27	6	0	4.507490	-0.617932	-1.074208
28	6	0	5.107866	0.511805	1.015020
29	1	0	3.495407	1.184187	2.226012
30	6	0	5.456206	-0.152197	-0.200809

31	1	0	4.809929	-1.120208	-1.985407
32	1	0	5.888087	0.864161	1.680558
33	6	0	1.428920	0.418230	0.799040
34	1	0	1.197241	0.923133	1.734933
35	8	0	2.223894	-0.887669	-1.597159
36	1	0	0.658925	-0.433144	-0.810818
37	8	0	6.757410	-0.336975	-0.516296
38	1	0	7.312579	0.037347	0.179126

Structure 109 (B3LYP, Gas Phase)

Energy (Hartrees): = - 1162.1537563
No imaginary frequencies

Standard orientation:

Center Number	Atomic Number	Atomic Type	Coordinates (Angstroms) X	Y	Z
1	6	0	1.779878	-0.544624	1.438159
2	6	0	1.096832	0.346371	0.363602
3	6	0	1.912066	0.289987	-0.936728
4	6	0	3.371691	0.622571	-0.653795
5	6	0	3.934433	-0.318575	0.417174
6	1	0	1.861408	-0.728766	-1.344289
7	1	0	3.423087	1.654844	-0.269847
8	1	0	3.913629	-1.349906	0.047111
9	1	0	1.107847	1.381919	0.736005
10	8	0	3.125153	-0.195090	1.598555
11	6	0	5.369009	0.030899	0.821710
12	1	0	5.409381	1.092641	1.119662
13	1	0	5.635101	-0.565062	1.699867
14	8	0	6.299216	-0.276540	-0.197598
15	1	0	5.953968	0.109755	-1.017798
16	8	0	4.153044	0.497997	-1.839067
17	1	0	3.687802	1.000925	-2.523790
18	8	0	1.460833	1.238342	-1.902543
19	1	0	0.599437	0.943535	-2.226393
20	7	0	-0.261240	-0.110522	0.120485
21	6	0	-2.627284	0.301676	0.182231
22	6	0	-2.976191	-0.953668	-0.404012
23	6	0	-3.680478	1.177652	0.538947
24	6	0	-4.310615	-1.302967	-0.615352
25	6	0	-5.010956	0.834716	0.332814
26	6	0	-5.313671	-0.410803	-0.245366
27	1	0	-4.558985	-2.257628	-1.061149
28	1	0	-5.806895	1.519771	0.616653
29	6	0	-1.250969	0.677285	0.400226
30	1	0	-1.071129	1.673869	0.815918
31	1	0	1.317360	-0.352187	2.417031
32	8	0	1.692046	-1.912574	1.100745
33	1	0	0.779243	-2.077304	0.821821
34	8	0	-2.023953	-1.820125	-0.771322
35	1	0	-1.144977	-1.398315	-0.553834
36	8	0	-6.596743	-0.796791	-0.469083
37	1	0	-7.201660	-0.103057	-0.174987
38	8	0	-3.324554	2.370099	1.095721
39	1	0	-4.118729	2.882942	1.293705

Structure 109 (B3LYP, DMSO)

Energy (Hartrees): = - 1162.1872299
No imaginary frequencies

Standard orientation:

Center Number	Atomic Number	Atomic Type	Coordinates (Angstroms) X	Y	Z
1	6	0	-1.779367	0.133941	-1.538786
2	6	0	-1.098782	0.471190	-0.187635
3	6	0	-1.906154	-0.137973	0.969299
4	6	0	-3.368203	0.273035	0.860185
5	6	0	-3.933227	-0.110536	-0.512134
6	1	0	-1.844587	-1.233136	0.908471
7	1	0	-3.433709	1.366651	0.971044
8	1	0	-3.913110	-1.201569	-0.621525
9	1	0	-1.105900	1.565306	-0.083482
10	8	0	-3.134095	0.505889	-1.534630
11	6	0	-5.366806	0.381450	-0.719974
12	1	0	-5.402748	1.470046	-0.549996
13	1	0	-5.653460	0.201706	-1.761332
14	8	0	-6.294697	-0.312435	0.101393
15	1	0	-5.913428	-0.310009	0.995442

16	8	0	-4.152336	-0.370877	1.868436
17	1	0	-3.720139	-0.177713	2.715117
18	8	0	-1.449984	0.314312	2.243038
19	1	0	-0.585986	-0.093267	2.404753
20	7	0	0.258622	-0.051594	-0.166322
21	6	0	2.624924	0.348392	-0.064185
22	6	0	2.969251	-1.034300	0.026677
23	6	0	3.683802	1.292010	-0.071452
24	6	0	4.301271	-1.441986	0.101227
25	6	0	5.013965	0.893329	0.001333
26	6	0	5.309843	-0.476579	0.086127
27	1	0	4.546639	-2.495139	0.171385
28	1	0	5.813287	1.630529	-0.007685
29	6	0	1.249155	0.782464	-0.119355
30	1	0	1.067608	1.860821	-0.110180
31	1	0	-1.323468	0.739132	-2.334230
32	8	0	-1.682664	-1.243879	-1.846107
33	1	0	-0.793193	-1.526333	-1.579635
34	8	0	2.010449	-1.972393	0.052732
35	1	0	1.132791	-1.492887	-0.007405
36	8	0	6.588970	-0.920022	0.157158
37	1	0	7.195186	-0.161914	0.139686
38	8	0	3.337596	2.602396	-0.158130
39	1	0	4.139929	3.148376	-0.145256

Structure 109 (M06-2X, Gas Phase)

Energy (Hartrees): = - 1162.0263935
No imaginary frequencies

Standard orientation:

Center Number	Atomic Number	Atomic Type	Coordinates (Angstroms) X	Y	Z
1	6	0	-1.817944	-0.829886	-1.325869
2	6	0	-1.100360	0.237395	-0.474974
3	6	0	-1.868992	0.440441	0.828804
4	6	0	-3.317279	0.757018	0.516312
5	6	0	-3.917839	-0.363572	-0.326229
6	1	0	-1.835561	-0.483443	1.418682
7	1	0	-3.353883	1.691356	-0.063848
8	1	0	-3.893896	-1.299723	0.241091
9	1	0	-1.108498	1.183314	-1.033557
10	8	0	-3.152342	-0.486068	-1.523288
11	6	0	-5.354547	-0.078421	-0.740811
12	1	0	-5.405400	0.921589	-1.195294
13	1	0	-5.641243	-0.808134	-1.499470
14	8	0	-6.246681	-0.214051	0.339527
15	1	0	-5.891514	0.304945	1.069320
16	8	0	-4.067694	0.889019	1.708778
17	1	0	-3.581520	1.494272	2.278962
18	8	0	-1.363001	1.532786	1.576383
19	1	0	-0.507985	1.283754	1.937802
20	7	0	0.251749	-0.188325	-0.177233
21	6	0	2.607393	0.253329	-0.220190
22	6	0	2.969539	-0.923656	0.483194
23	6	0	3.637291	1.110527	-0.647692
24	6	0	4.303354	-1.219850	0.740672
25	6	0	4.968393	0.822966	-0.397320
26	6	0	5.287812	-0.349892	0.298444
27	1	0	4.565416	-2.119336	1.279315
28	1	0	5.750344	1.493880	-0.738226
29	6	0	1.224901	0.581420	-0.501890
30	1	0	1.040951	1.532361	-1.009548
31	1	0	-1.373313	-0.868843	-2.325748
32	8	0	-1.761430	-2.086820	-0.702740
33	1	0	-0.844818	-2.244970	-0.454483
34	8	0	2.039884	-1.770680	0.925102
35	1	0	1.160451	-1.408392	0.664703
36	8	0	6.569014	-0.681341	0.570077
37	1	0	7.161816	-0.010361	0.221615
38	8	0	3.260734	2.228627	-1.316855
39	1	0	4.038194	2.737319	-1.561259

Structure 109 (M06-2X, DMSO)

Energy (Hartrees): = - 1162.0623455
No imaginary frequencies

Standard orientation:

Center Number	Atomic Number	Atomic Type	Coordinates (Angstroms) X	Y	Z

1	6	0	-1.818438	-0.744054	-1.382535
2	6	0	-1.101904	0.258140	-0.460126
3	6	0	-1.861887	0.372938	0.860353
4	6	0	-3.309477	0.713232	0.573502
5	6	0	-3.918563	-0.339247	-0.346776
6	1	0	-1.821882	-0.585956	1.391169
7	1	0	-3.349216	1.689055	0.068575
8	1	0	-3.907267	-1.311533	0.158404
9	1	0	-1.108576	1.237202	-0.955957
10	8	0	-3.160303	-0.396935	-1.552851
11	6	0	-5.349269	-0.005231	-0.743547
12	1	0	-5.383555	1.020113	-1.137137
13	1	0	-5.656472	-0.684893	-1.540714
14	8	0	-6.247401	-0.180365	0.334188
15	1	0	-5.863944	0.284321	1.087820
16	8	0	-4.065804	0.750746	1.774425
17	1	0	-3.594816	1.330225	2.385608
18	8	0	-1.350401	1.412811	1.675947
19	1	0	-0.502260	1.125722	2.033057
20	7	0	0.251097	-0.190666	-0.197360
21	6	0	2.604820	0.256750	-0.224355
22	6	0	2.964140	-0.932071	0.458344
23	6	0	3.638767	1.125402	-0.627013
24	6	0	4.296459	-1.233290	0.718145
25	6	0	4.970467	0.833737	-0.375872
26	6	0	5.285137	-0.350944	0.297249
27	1	0	4.556678	-2.142664	1.243609
28	1	0	5.753545	1.512900	-0.698475
29	6	0	1.220850	0.595078	-0.492532
30	1	0	1.029434	1.564836	-0.958184
31	1	0	-1.375671	-0.703837	-2.382337
32	8	0	-1.760242	-2.050545	-0.864488
33	1	0	-0.853095	-2.202015	-0.573314
34	8	0	2.028467	-1.786927	0.880793
35	1	0	1.149223	-1.417546	0.615129
36	8	0	6.562993	-0.685803	0.568716
37	1	0	7.156823	-0.000792	0.235322
38	8	0	3.271411	2.255040	-1.270544
39	1	0	4.057012	2.772712	-1.489555

Structure 110 (B3LYP, Gas Phase)

Energy (Hartrees): = - 1162.15471
No imaginary frequencies

Standard orientation:

Center Number	Atomic Number	Atomic Type	Coordinates (Angstroms) X	Y	Z
1	6	0	-1.758600	1.171419	-0.852270
2	6	0	-1.112996	0.306320	0.246473
3	6	0	-1.953418	-0.965789	0.433706
4	6	0	-3.420131	-0.624548	0.659799
5	6	0	-3.935962	0.255252	-0.485763
6	1	0	-1.706868	0.640628	-1.821368
7	1	0	-1.881371	-1.570094	-0.484787
8	1	0	-3.505566	-0.052705	1.597577
9	1	0	-3.871546	-0.309794	-1.430040
10	1	0	-1.144642	0.900026	1.172412
11	8	0	-3.124772	1.429913	-0.543535
12	6	0	-5.388630	0.700400	-0.291564
13	1	0	-5.483764	1.184583	0.694856
14	1	0	-5.624875	1.453913	-1.049261
15	8	0	-6.298415	-0.367856	-0.458334
16	1	0	-5.980367	-1.095241	0.099749
17	8	0	-4.207146	-1.810818	0.727959
18	1	0	-3.766028	-2.394561	1.362705
19	8	0	-1.534746	-1.731728	1.557979
20	1	0	-0.628956	-2.025104	1.387787
21	8	0	-1.087309	2.387405	-0.891994
22	1	0	-1.512794	2.933672	-1.567337
23	7	0	0.243974	-0.065101	-0.106571
24	6	0	2.604927	0.170318	0.225216
25	6	0	2.948101	-0.717449	-0.840609
26	6	0	3.660800	0.752660	0.964434
27	6	0	4.283765	-0.996244	-1.140483
28	6	0	4.991434	0.478529	0.670421
29	6	0	5.289620	-0.398896	-0.386688
30	1	0	4.529057	-1.669278	-1.952097
31	1	0	5.789839	0.934189	1.252244
32	6	0	1.227600	0.473431	0.537844
33	1	0	1.046908	1.186581	1.347647
34	8	0	1.995256	-1.301105	-1.574433
35	1	0	1.112917	-0.976793	-1.225050
36	8	0	6.573884	-0.701948	-0.716825

37	1	0	7.178638	-0.228837	-0.130377
38	8	0	3.310065	1.592268	1.980650
39	1	0	4.107990	1.943063	2.396515

Structure 110 (B3LYP, DMSO)

Energy (Hartrees): = - 1162.1883199
No imaginary frequencies

Standard orientation:

Center Number	Atomic Number	Atomic Type	Coordinates (Angstroms) X	Y	Z
1	6	0	-1.799122	1.373187	-0.565485
2	6	0	-1.115434	0.293901	0.292618
3	6	0	-1.913992	-1.016812	0.173766
4	6	0	-3.388937	-0.784543	0.477085
5	6	0	-3.946016	0.343415	-0.400902
6	1	0	-1.765792	1.082066	-1.629491
7	1	0	-1.828218	-1.385736	-0.859042
8	1	0	-3.487921	-0.480454	1.530262
9	1	0	-3.887228	0.040005	-1.458192
10	1	0	-1.146371	0.637865	1.336855
11	8	0	-3.165886	1.518375	-0.173527
12	6	0	-5.400634	0.685576	-0.071370
13	1	0	-5.479624	0.920787	1.002537
14	1	0	-5.687588	1.584194	-0.627302
15	8	0	-6.292410	-0.352218	-0.452436
16	1	0	-5.910303	-1.174250	-0.101723
17	8	0	-4.145814	-1.973466	0.234438
18	1	0	-3.717856	-2.679170	0.744093
19	8	0	-1.461515	-2.008965	1.092073
20	1	0	-0.590224	-2.308634	0.792347
21	8	0	-1.155820	2.584621	-0.343785
22	1	0	-1.522477	3.228927	-0.968976
23	7	0	0.246647	0.072524	-0.153621
24	6	0	2.600913	0.095132	0.276611
25	6	0	2.942381	-0.336919	-1.041061
26	6	0	3.658597	0.332185	1.188743
27	6	0	4.275690	-0.518642	-1.414291
28	6	0	4.989178	0.155165	0.823170
29	6	0	5.284295	-0.270133	-0.481945
30	1	0	4.520767	-0.845812	-2.417892
31	1	0	5.787687	0.344286	1.536602
32	6	0	1.222134	0.290203	0.666286
33	1	0	1.031608	0.634843	1.686753
34	8	0	1.983257	-0.574651	-1.946484
35	1	0	1.102437	-0.386726	-1.491039
36	8	0	6.564214	-0.457817	-0.891536
37	1	0	7.168935	-0.257236	-0.159193
38	8	0	3.313236	0.740475	2.438137
39	1	0	4.117217	0.876247	2.964353

Structure 110 (M06-2X, Gas Phase)

Energy (Hartrees): = - 1162.0265094
No imaginary frequencies

Standard orientation:

Center Number	Atomic Number	Atomic Type	Coordinates (Angstroms) X	Y	Z
1	6	0	-1.728862	1.123867	-0.859183
2	6	0	-1.116456	0.274098	0.255433
3	6	0	-1.969791	-0.972656	0.455255
4	6	0	-3.416326	-0.588595	0.681437
5	6	0	-3.903177	0.265397	-0.485441
6	1	0	-1.681568	0.575478	-1.815311
7	1	0	-1.916282	-1.590203	-0.453398
8	1	0	-3.482036	0.009385	1.601870
9	1	0	-3.838711	-0.325220	-1.412040
10	1	0	-1.143665	0.886149	1.167438
11	8	0	-3.079951	1.418456	-0.566460
12	6	0	-5.344142	0.728253	-0.316699
13	1	0	-5.457633	1.186660	0.675591
14	1	0	-5.547323	1.493938	-1.066994
15	8	0	-6.255555	-0.323507	-0.523790
16	1	0	-5.977982	-1.054851	0.039305
17	8	0	-4.231082	-1.741679	0.781324
18	1	0	-3.804137	-2.332124	1.411653
19	8	0	-1.561384	-1.724234	1.580845
20	1	0	-0.658992	-2.020536	1.427107

21	8	0	-1.025470	2.313153	-0.912273
22	1	0	-1.420140	2.860660	-1.597076
23	7	0	0.232022	-0.125819	-0.082566
24	6	0	2.588540	0.171279	0.195896
25	6	0	2.946567	-0.804513	-0.768881
26	6	0	3.621976	0.841338	0.873825
27	6	0	4.283449	-1.084467	-1.036637
28	6	0	4.954945	0.567979	0.614331
29	6	0	5.271060	-0.399879	-0.346746
30	1	0	4.543673	-1.828663	-1.775859
31	1	0	5.739925	1.093958	1.148592
32	6	0	1.203963	0.483613	0.487574
33	1	0	1.020947	1.272034	1.221700
34	8	0	2.015841	-1.476810	-1.442477
35	1	0	1.131561	-1.150899	-1.142532
36	8	0	6.554712	-0.707963	-0.641456
37	1	0	7.149251	-0.174180	-0.108081
38	8	0	3.250027	1.764652	1.795928
39	1	0	4.031369	2.168331	2.181888

Structure 110 (M06-2X, DMSO)

Energy (Hartrees): = - 1162.062626
No imaginary frequencies

Standard orientation:

Center Number	Atomic Number	Atomic Type	Coordinates (Angstroms) X	Y	Z
1	6	0	-1.799344	1.313943	-0.673723
2	6	0	-1.118870	0.328545	0.275930
3	6	0	-1.901377	-0.984520	0.277763
4	6	0	-3.364253	-0.725455	0.572712
5	6	0	-3.916404	0.297424	-0.416749
6	1	0	-1.763291	0.926845	-1.704001
7	1	0	-1.822561	-1.443031	-0.717347
8	1	0	-3.455748	-0.314141	1.587479
9	1	0	-3.836794	-0.110182	-1.435418
10	1	0	-1.143010	0.768248	1.282390
11	8	0	-3.155732	1.489270	-0.298284
12	6	0	-5.371704	0.647549	-0.142795
13	1	0	-5.481103	0.928813	0.913516
14	1	0	-5.643905	1.510353	-0.753631
15	8	0	-6.240270	-0.412232	-0.492456
16	1	0	-5.882104	-1.206455	-0.077974
17	8	0	-4.118779	-1.922769	0.456797
18	1	0	-3.682675	-2.586368	1.005002
19	8	0	-1.434653	-1.878819	1.271357
20	1	0	-0.560465	-2.188479	1.008179
21	8	0	-1.156917	2.532939	-0.560912
22	1	0	-1.503791	3.115498	-1.246584
23	7	0	0.234124	0.065468	-0.160727
24	6	0	2.585628	0.126704	0.247465
25	6	0	2.926012	-0.454741	-1.000041
26	6	0	3.631458	0.469228	1.125508
27	6	0	4.255854	-0.679159	-1.343106
28	6	0	4.959678	0.251234	0.792651
29	6	0	5.257030	-0.325208	-0.446152
30	1	0	4.503195	-1.125138	-2.297453
31	1	0	5.753185	0.521978	1.482216
32	6	0	1.203247	0.369014	0.618360
33	1	0	1.018910	0.825849	1.593664
34	8	0	1.977705	-0.799537	-1.873318
35	1	0	1.100252	-0.564742	-1.464233
36	8	0	6.531441	-0.562092	-0.821907
37	1	0	7.134078	-0.278449	-0.122582
38	8	0	3.280884	1.020394	2.309157
39	1	0	4.075792	1.216547	2.821440

Structure 111 (B3LYP, Gas Phase)

Energy (Hartrees): = - 1162.1509546
No imaginary frequencies

Standard orientation:

Center Number	Atomic Number	Atomic Type	Coordinates (Angstroms) X	Y	Z
1	6	0	1.874584	-1.153656	1.126950
2	6	0	1.108611	0.061150	0.569455
3	6	0	1.820358	0.583818	-0.693357
4	6	0	3.285815	0.856652	-0.371018
5	6	0	3.955921	-0.403349	0.187214

6	1	0	1.767182	-0.177181	-1.482216
7	1	0	3.325334	1.641311	0.403166
8	1	0	3.941831	-1.188370	-0.578683
9	1	0	1.144567	0.853051	1.325994
10	8	0	3.231638	-0.832492	1.354268
11	6	0	5.405681	-0.166351	0.619600
12	1	0	5.437708	0.688096	1.316746
13	1	0	5.751563	-1.047525	1.168737
14	8	0	6.267315	0.007888	-0.486376
15	1	0	5.862096	0.682061	-1.054314
16	8	0	3.989143	1.276396	-1.535147
17	1	0	3.465394	1.992135	-1.924834
18	8	0	1.265532	1.817698	-1.142867
19	1	0	0.443589	1.625972	-1.613205
20	7	0	-0.278967	-0.262369	0.312706
21	6	0	-2.620095	0.228773	0.291749
22	6	0	-2.932496	-0.998958	-0.467953
23	6	0	-3.679295	1.113616	0.673762
24	6	0	-4.310118	-1.230743	-0.788974
25	6	0	-4.983800	0.844855	0.343664
26	6	0	-5.281313	-0.343122	-0.394574
27	1	0	-4.572164	-2.121480	-1.346297
28	1	0	-5.784113	1.520888	0.637606
29	6	0	-1.301421	0.527847	0.627391
30	1	0	-1.074783	1.442791	1.170138
31	1	0	1.484559	-1.429713	2.114290
32	8	0	1.704810	-2.194191	0.196444
33	1	0	2.056581	-3.009286	0.579175
34	8	0	-2.012788	-1.793633	-0.812415
35	1	0	-0.578707	-1.118450	-0.206625
36	8	0	-6.573737	-0.617907	-0.727451
37	1	0	-7.148523	0.081816	-0.391326
38	8	0	-3.312391	2.227186	1.375348
39	1	0	-4.100101	2.749722	1.574800

Structure 111 (B3LYP, DMSO)

Energy (Hartrees): = - 1162.1892184
No imaginary frequencies

Standard orientation:

Center Number	Atomic Number	Atomic Type	Coordinates (Angstroms) X	Y	Z
1	6	0	1.851873	-1.146439	1.118841
2	6	0	1.115335	0.081133	0.551480
3	6	0	1.841840	0.605290	-0.703308
4	6	0	3.311409	0.844055	-0.371501
5	6	0	3.953555	-0.427308	0.194932
6	1	0	1.776257	-0.141502	-1.504199
7	1	0	3.369136	1.633796	0.393965
8	1	0	3.935677	-1.213953	-0.569673
9	1	0	1.138458	0.866315	1.313036
10	8	0	3.215875	-0.847187	1.354993
11	6	0	5.398464	-0.209175	0.650034
12	1	0	5.422464	0.615280	1.381264
13	1	0	5.745986	-1.112513	1.161869
14	8	0	6.275952	0.024307	-0.441450
15	1	0	5.843139	0.696879	-0.993561
16	8	0	4.034256	1.236742	-1.539775
17	1	0	3.563472	2.000814	-1.908305
18	8	0	1.310082	1.854543	-1.139352
19	1	0	0.520644	1.674359	-1.670617
20	7	0	-0.272919	-0.235810	0.270890
21	6	0	-2.624483	0.225887	0.273541
22	6	0	-2.945694	-0.990983	-0.490901
23	6	0	-3.680204	1.106441	0.677951
24	6	0	-4.326506	-1.220258	-0.791030
25	6	0	-4.995073	0.842040	0.366731
26	6	0	-5.299276	-0.334383	-0.373917
27	1	0	-4.599262	-2.105900	-1.354345
28	1	0	-5.788980	1.516858	0.677707
29	6	0	-1.298874	0.530939	0.604853
30	1	0	-1.073268	1.433874	1.166052
31	1	0	1.449928	-1.407340	2.104146
32	8	0	1.675475	-2.197615	0.198808
33	1	0	1.990352	-3.014679	0.615738
34	8	0	-2.031710	-1.790232	-0.861283
35	1	0	-0.547971	-1.081207	-0.263517
36	8	0	-6.590159	-0.613512	-0.693658
37	1	0	-7.166121	0.084733	-0.343840
38	8	0	-3.312354	2.211592	1.379096
39	1	0	-4.103015	2.732328	1.592789

Structure 111 (M06-2X, Gas Phase)

Energy (Hartrees): = - 1162.0199547
No imaginary frequencies

Standard orientation:

Center Number	Atomic Number	Atomic Type	Coordinates (Angstroms) X	Y	Z
1	6	0	1.897982	-1.178325	1.117350
2	6	0	1.115848	0.038433	0.615601
3	6	0	1.789258	0.592221	-0.641634
4	6	0	3.244172	0.882267	-0.331040
5	6	0	3.930207	-0.387639	0.160009
6	1	0	1.737876	-0.154070	-1.442894
7	1	0	3.285081	1.634963	0.471341
8	1	0	3.897064	-1.142360	-0.633628
9	1	0	1.144325	0.813278	1.387623
10	8	0	3.247692	-0.857210	1.323327
11	6	0	5.383402	-0.155394	0.550833
12	1	0	5.439356	0.707581	1.229530
13	1	0	5.735650	-1.033620	1.094104
14	8	0	6.206604	0.004074	-0.578546
15	1	0	5.806012	0.685580	-1.129509
16	8	0	3.921738	1.350354	-1.479760
17	1	0	3.386710	2.060598	-1.850189
18	8	0	1.200635	1.814475	-1.047934
19	1	0	0.378039	1.625358	-1.508064
20	7	0	-0.260779	-0.313840	0.360656
21	6	0	-2.596147	0.199863	0.285319
22	6	0	-2.925514	-1.032094	-0.450836
23	6	0	-3.633416	1.115436	0.647190
24	6	0	-4.310021	-1.232935	-0.775451
25	6	0	-4.933648	0.874698	0.317728
26	6	0	-5.252155	-0.321426	-0.402685
27	1	0	-4.591940	-2.124705	-1.317458
28	1	0	-5.719184	1.569692	0.596444
29	6	0	-1.283875	0.482115	0.629478
30	1	0	-1.054828	1.408089	1.151083
31	1	0	1.524156	-1.500427	2.092611
32	8	0	1.723029	-2.170606	0.146531
33	1	0	2.055107	-3.006670	0.485235
34	8	0	-2.035453	-1.842285	-0.767867
35	1	0	-0.505207	-1.181366	-0.143174
36	8	0	-6.544028	-0.564263	-0.729973
37	1	0	-7.099654	0.149558	-0.408470
38	8	0	-3.238419	2.221752	1.328638
39	1	0	-4.004031	2.771749	1.514314

Structure 111 (M06-2X, DMSO)

Energy (Hartrees): = - 1162.0613854
No imaginary frequencies

Standard orientation:

Center Number	Atomic Number	Atomic Type	Coordinates (Angstroms) X	Y	Z
1	6	0	1.865179	-1.152650	1.119450
2	6	0	1.121162	0.077610	0.593140
3	6	0	1.819355	0.619131	-0.656950
4	6	0	3.278147	0.870374	-0.330081
5	6	0	3.925220	-0.414300	0.175196
6	1	0	1.756522	-0.119567	-1.464176
7	1	0	3.335722	1.628650	0.464186
8	1	0	3.881373	-1.174820	-0.612810
9	1	0	1.133010	0.851580	1.364787
10	8	0	3.222804	-0.860819	1.334264
11	6	0	5.376310	-0.214459	0.587449
12	1	0	5.437791	0.628728	1.288858
13	1	0	5.716719	-1.113158	1.105388
14	8	0	6.218577	-0.024631	-0.531940
15	1	0	5.805519	0.659841	-1.072090
16	8	0	3.982383	1.307631	-1.480650
17	1	0	3.496660	2.060219	-1.839773
18	8	0	1.264292	1.856361	-1.065512
19	1	0	0.469148	1.681863	-1.581423
20	7	0	-0.255983	-0.263895	0.304571
21	6	0	-2.604315	0.204634	0.262058
22	6	0	-2.933270	-1.016927	-0.478684
23	6	0	-3.643254	1.106711	0.647980
24	6	0	-4.318953	-1.229422	-0.775471
25	6	0	-4.954710	0.859928	0.341248
26	6	0	-5.271407	-0.326746	-0.377178

27	1	0	-4.605109	-2.119053	-1.322243
28	1	0	-5.738617	1.549350	0.638107
29	6	0	-1.282857	0.502044	0.600819
30	1	0	-1.058562	1.414792	1.146184
31	1	0	1.473489	-1.446622	2.095664
32	8	0	1.681569	-2.164754	0.168949
33	1	0	1.970276	-3.001228	0.552961
34	8	0	-2.041436	-1.824651	-0.828175
35	1	0	-0.478210	-1.120915	-0.217445
36	8	0	-6.560015	-0.588332	-0.686810
37	1	0	-7.123954	0.121037	-0.354218
38	8	0	-3.256696	2.209497	1.326106
39	1	0	-4.031373	2.748585	1.532330

Structure 112 (B3LYP, Gas Phase)

Energy (Hartrees): = - 1162.1483594
No imaginary frequencies

Standard orientation:

Center Number	Atomic Number	Atomic Type	Coordinates (Angstroms) X	Y	Z
1	6	0	-1.733834	1.257737	-0.695899
2	6	0	-1.122061	0.306082	0.350865
3	6	0	-1.944279	-0.990201	0.406006
4	6	0	-3.424865	-0.678371	0.595396
5	6	0	-3.907014	0.293716	-0.489472
6	1	0	-1.639745	0.815275	-1.705520
7	1	0	-1.832216	-1.520909	-0.554014
8	1	0	-3.551242	-0.189248	1.574643
9	1	0	-3.799318	-0.184493	-1.476542
10	1	0	-1.189912	0.819307	1.317167
11	8	0	-3.108761	1.477609	-0.412421
12	6	0	-5.372122	0.705650	-0.314677
13	1	0	-5.514527	1.091810	0.708477
14	1	0	-5.585598	1.526097	-1.006555
15	8	0	-6.259434	-0.349262	-0.622412
16	1	0	-5.964494	-1.121462	-0.114387
17	8	0	-4.198301	-1.870747	0.529602
18	1	0	-3.783914	-2.500309	1.138382
19	8	0	-1.565934	-1.836813	1.483077
20	1	0	-0.678780	-2.179665	1.305508
21	8	0	-1.065345	2.472389	-0.599770
22	1	0	-1.422900	3.066013	-1.274333
23	7	0	0.261373	-0.024744	0.071954
24	6	0	2.616220	0.271715	0.158903
25	6	0	2.842470	-1.012094	-0.528802
26	6	0	3.737231	1.064378	0.559213
27	6	0	4.197909	-1.396776	-0.764410
28	6	0	5.024408	0.654694	0.307499
29	6	0	5.235635	-0.588541	-0.360331
30	1	0	4.395640	-2.334665	-1.268252
31	1	0	5.873013	1.263876	0.611638
32	6	0	1.314737	0.692527	0.442475
33	1	0	1.136828	1.620264	0.979089
34	8	0	1.861458	-1.738441	-0.885025
35	1	0	0.571729	-0.880852	-0.463140
36	8	0	6.505821	-1.008092	-0.615870
37	1	0	7.134313	-0.352847	-0.286039
38	8	0	3.450454	2.236676	1.196082
39	1	0	4.275220	2.685370	1.423492

Structure 112 (B3LYP, DMSO)

Energy (Hartrees): = - 1162.186756
No imaginary frequencies

Standard orientation:

Center Number	Atomic Number	Atomic Type	Coordinates (Angstroms) X	Y	Z
1	6	0	-1.719282	1.161958	-0.824324
2	6	0	-1.129116	0.299112	0.309475
3	6	0	-1.980291	-0.968726	0.497938
4	6	0	-3.456279	-0.610765	0.639601
5	6	0	-3.912305	0.273266	-0.528145
6	1	0	-1.650564	0.620120	-1.783323
7	1	0	-1.867315	-1.604027	-0.393472
8	1	0	-3.590212	-0.043482	1.573395
9	1	0	-3.809470	-0.289079	-1.469568
10	1	0	-1.154766	0.896765	1.226567

11	8	0	-3.091129	1.443124	-0.551014
12	6	0	-5.363441	0.739071	-0.388994
13	1	0	-5.483440	1.250786	0.579850
14	1	0	-5.574643	1.470575	-1.175759
15	8	0	-6.287040	-0.327955	-0.543545
16	1	0	-5.963988	-1.045845	0.026432
17	8	0	-4.257228	-1.793740	0.658992
18	1	0	-3.907200	-2.353506	1.369702
19	8	0	-1.623440	-1.691851	1.672003
20	1	0	-0.773328	-2.126786	1.507840
21	8	0	-1.023183	2.362539	-0.858863
22	1	0	-1.283180	2.834673	-1.664902
23	7	0	0.249276	-0.062396	0.031555
24	6	0	2.620629	0.152537	0.254589
25	6	0	2.883634	-0.781901	-0.852151
26	6	0	3.720770	0.751539	0.949681
27	6	0	4.253869	-1.033149	-1.176217
28	6	0	5.024869	0.474487	0.604989
29	6	0	5.272145	-0.425009	-0.469262
30	1	0	4.483021	-1.714348	-1.988313
31	1	0	5.852351	0.933022	1.140885
32	6	0	1.306796	0.469031	0.622152
33	1	0	1.119112	1.174799	1.426737
34	8	0	1.928575	-1.333391	-1.483967
35	1	0	0.511701	-0.707128	-0.742685
36	8	0	6.550975	-0.710936	-0.828749
37	1	0	7.162991	-0.221500	-0.256421
38	8	0	3.406078	1.602211	1.962035
39	1	0	4.223149	1.953008	2.351031

Structure 112 (M06-2X, Gas Phase)

Energy (Hartrees): = - 1162.0163121
No imaginary frequencies

Standard orientation:

Center Number	Atomic Number	Atomic Type	Coordinates (Angstroms) X	Y	Z
1	6	0	-1.713668	1.267626	-0.617488
2	6	0	-1.124337	0.272105	0.383890
3	6	0	-1.945858	-1.012730	0.369932
4	6	0	-3.412639	-0.690058	0.575935
5	6	0	-3.872209	0.326376	-0.466374
6	1	0	-1.621282	0.866746	-1.641479
7	1	0	-1.839022	-1.496177	-0.613487
8	1	0	-3.532287	-0.242052	1.572940
9	1	0	-3.754627	-0.109980	-1.469774
10	1	0	-1.192046	0.737972	1.372783
11	8	0	-3.075086	1.493617	-0.330037
12	6	0	-5.331001	0.727203	-0.290582
13	1	0	-5.500400	1.008226	0.758276
14	1	0	-5.522292	1.604880	-0.909825
15	8	0	-6.203456	-0.290350	-0.715929
16	1	0	-5.941553	-1.099466	-0.262838
17	8	0	-4.200142	-1.857033	0.456469
18	1	0	-3.800792	-2.526702	1.022737
19	8	0	-1.574871	-1.906823	1.397696
20	1	0	-0.690333	-2.242506	1.219307
21	8	0	-1.023081	2.456737	-0.467026
22	1	0	-1.361187	3.088403	-1.108688
23	7	0	0.250816	-0.058167	0.086543
24	6	0	2.594889	0.264912	0.130453
25	6	0	2.832933	-1.053535	-0.468324
26	6	0	3.698276	1.100157	0.476155
27	6	0	4.195845	-1.426451	-0.683454
28	6	0	4.984489	0.701739	0.248552
29	6	0	5.211442	-0.579317	-0.338885
30	1	0	4.409748	-2.389116	-1.126003
31	1	0	5.822355	1.339939	0.510303
32	6	0	1.296081	0.683313	0.402612
33	1	0	1.109316	1.638293	0.885389
34	8	0	1.874662	-1.808025	-0.767429
35	1	0	0.540467	-0.939316	-0.398102
36	8	0	6.482567	-0.983965	-0.570047
37	1	0	7.096578	-0.300022	-0.292193
38	8	0	3.387018	2.296353	1.034571
39	1	0	4.195298	2.775917	1.234163

Structure 112 (M06-2X, DMSO)

Energy (Hartrees): = - 1162.0578682
No imaginary frequencies

Standard orientation:

Center Number	Atomic Number	Atomic Type	Coordinates (Angstroms) X	Y	Z
1	6	0	-1.706173	1.129651	-0.826929
2	6	0	-1.134311	0.268781	0.303083
3	6	0	-1.993070	-0.979315	0.491987
4	6	0	-3.448154	-0.585650	0.655410
5	6	0	-3.889228	0.288022	-0.515718
6	1	0	-1.652614	0.580681	-1.780165
7	1	0	-1.906010	-1.612037	-0.402599
8	1	0	-3.553730	-0.004367	1.582338
9	1	0	-3.791874	-0.284411	-1.449965
10	1	0	-1.144744	0.867930	1.218216
11	8	0	-3.059028	1.439631	-0.549566
12	6	0	-5.327800	0.764935	-0.379550
13	1	0	-5.459740	1.228194	0.607661
14	1	0	-5.514040	1.525789	-1.139733
15	8	0	-6.251427	-0.283498	-0.592459
16	1	0	-5.969697	-1.014530	-0.029409
17	8	0	-4.272532	-1.739126	0.698174
18	1	0	-3.921825	-2.314061	1.389287
19	8	0	-1.632983	-1.705407	1.651140
20	1	0	-0.788760	-2.141383	1.487991
21	8	0	-0.981464	2.304975	-0.868304
22	1	0	-1.212883	2.774501	-1.678174
23	7	0	0.232383	-0.114619	0.016130
24	6	0	2.604304	0.132245	0.230359
25	6	0	2.898384	-0.835263	-0.830903
26	6	0	3.674246	0.785005	0.917485
27	6	0	4.282102	-1.058647	-1.125824
28	6	0	4.983201	0.533549	0.604297
29	6	0	5.265028	-0.398804	-0.432830
30	1	0	4.542979	-1.760775	-1.907509
31	1	0	5.790193	1.032743	1.131698
32	6	0	1.286803	0.438898	0.573845
33	1	0	1.089253	1.173644	1.348998
34	8	0	1.978065	-1.427431	-1.441799
35	1	0	0.444886	-0.788511	-0.732220
36	8	0	6.549872	-0.656181	-0.760395
37	1	0	7.139130	-0.135061	-0.200581
38	8	0	3.319287	1.657913	1.885292
39	1	0	4.112492	2.046531	2.276310
